# Genome-wide DNA polymorphisms in low Phosphate tolerant and sensitive rice genotypes

**DOI:** 10.1038/srep13090

**Published:** 2015-08-17

**Authors:** Poonam Mehra, Bipin K. Pandey, Jitender Giri

**Affiliations:** 1 National Institute of Plant Genome Research, Aruna Asaf Ali Marg, New Delhi-110067, India

## Abstract

Soil Phosphorus (P) deficiency is one of the major challenges to rice crop world-wide. Modern rice genotypes are highly P-responsive and rely on high input of P fertilizers. However, low P tolerant traditional cultivars and landraces have genetic potential to sustain well under low P. Identification of high resolution DNA polymorphisms (SNPs and InDels) in such contrasting genotypes is largely missing for low P response at gene levels. Here, we report high quality DNA polymorphisms in low P sensitive genotype, PB1 and tolerant traditional genotype, Dular. We performed whole genome resequencing using Illumina NGS platform and identified a total of 5,157,939 sequence variants in PB1 and Dular with reference to Nipponbare genome. We have identified approximately 2.3 million and 2.9 million high quality polymorphisms in PB1 and Dular, respectively, with an average read depth of ≥24X. We further mapped several DNA polymorphisms (non-synonymous and regulatory variants) having potential functional significance to key Phosphate Starvation Responsive (PSR) and root architecture genes in Dular and Kasalath using a compiled list of low P responsive genes. These identified variants can serve as a useful source of genetic variability for improving low P tolerance and root architecture of high yielding modern genotypes.

Phosphorous (P) is the second most essential element after Nitrogen for plant growth and development. Given the slow rate of diffusion and high rate of P fixation in soil, it is one of the least bio-available elements to plants. Rice, a staple food crop for more than half of the world’s population, is grown in areas with soil P deficiency^1^. To compensate this deficiency, modern agriculture relies heavily on high input of P fertilizers. Farmers across the world use 170 million tons of P fertilizers annually and at the current rate of P mining, depletion of all the existing reserves of rock P has been predicted variably in next 25–150 years^2^. Latest reports by International Fertiliser Development Centre; however, claims sufficient availability of rock P for next 300–400 years (www.ifdc.org). Nevertheless, P rocks are finite resource and would end up sooner or later. Approximately 80% of the soil P exists in the form of bound organic and inorganic compounds. To trap this unavailable P, plants have evolved a number of architectural, physiological and molecular adaptations. Root System Architecture (RSA) is altered for increasing the foraging capacity of plants to acquire more P from soil. These alterations include inhibition of primary root length^3^, proliferation of lateral roots^4^, increase in the density and length of root hairs^5^ and formation of cluster roots^6^. Besides these responses, plant roots also secrete phosphatases and organic acids for solubilisation of the soil bound P^7^. Few of the molecular regulators controlling these adaptations have been identified in plants including rice^8^. Recently, a major QTL for P deficiency tolerance, *Pup1*, was cloned from a traditional low P tolerant rice genotype, Kasalath. The candidate gene *PSTOL1* encodes a kinase which enhances the early root growth resulting in more P uptake^9^. In this context, it is noteworthy that traditional genotypes have immense potential to resist the various abiotic and biotic stresses naturally^10^. Since traditional genotypes have been long growing on low P ecosystems, they have genetic competence to sustain in such environment. On the other hand modern genotypes, as a result of artificial selection for few desirable traits, are highly P responsive but have low tolerance to P deficiency. Therefore, identifying the genetic basis of low P tolerance mechanisms in traditional genotypes can pave the way for improving the elite rice cultivars for low P tolerance.

Rich genetic diversity exists among the traditional and modern cultivars for important agronomical traits including low P tolerance^11^,^12^,^13^. However, comprehensive phenotypic and genotypic screening of such diverse collections for elucidating the mechanisms of low P tolerance is required. Few studies have led to the identification of major and minor QTLs for low P tolerance by conventional genetic mapping approaches based on RFLP, AFLP and SSR markers in population generated from low P tolerant and susceptible genotypes^14^,^15^,^16^. However, utilising conventional markers is a time consuming and laborious process. With the availability of high quality rice genome sequence and advent of next generation DNA sequencing (NGS) technologies, it has now become easier to explore high level of genetic variability for low P tolerance at genome-wide scale by resequencing diverse rice genotypes. Whole genome re-sequencing studies have also been utilized in genes/QTLs identification, genetic mapping, genome diversification, evolutionary and phylogenetic analysis^17^,^18^,^19^. Apart from bridging the gap of genotype to phenotype, NGS has immense potential to unravel the functional complexity of rice genome and can enhance the pace of molecular breeding program to improve the beneficial traits.

Genetic diversity for low P tolerance in rice genotypes can be therefore, investigated using NGS-based whole genome resequencing of genotypes with contrasting low P tolerance/sensitive traits. Therefore, in the present study, we have carried out the whole genome re-sequencing of low P tolerant traditional genotype “Dular”^13^,^20^ and sensitive modern genotype “PB1” using Illumina HiSeq® NGS platform followed by their comparison with another low P tolerant (Kasalath) and sensitive (Nipponbare) genotypes. Further, we have identified numerous SNP and InDel markers in these genotypes at a genome-wide scale and evaluated functional significance of these SNPs by correlating their presence (structural and functional annotations) in PSR (Phosphate Starvation Response) genes. The genome-wide high resolution SNP and InDel markers discovered from contrasting rice genotypes for low P tolerance would accelerate the identification and functional validation of novel PSR genes for genomics-assisted crop improvement.

## Results and Discussion

### Growth behaviour of PB1 and Dular under different P regimes

**Figure 1:**
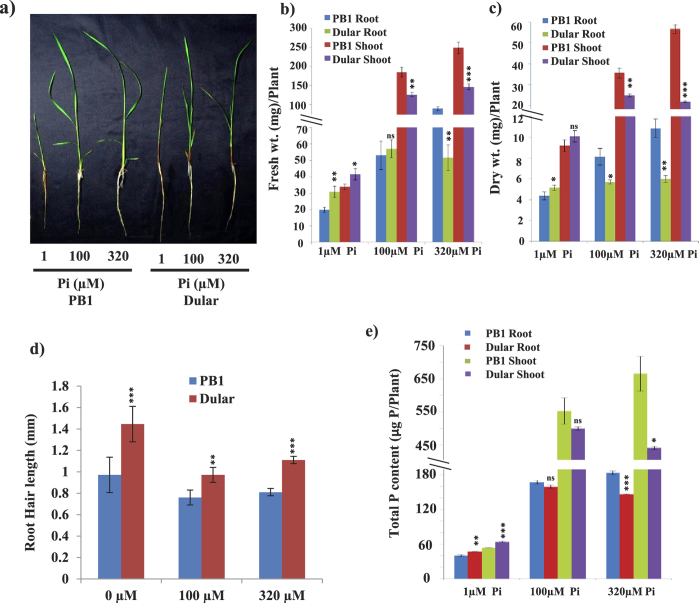
Growth behaviour of Dular and PB1 under P deficiency. (**a**) Plant phenotype of 15-days-old hydroponically grown seedlings under 1, 100 and 320 μM concentrations of Pi (NaH_2_PO_4_). Scale bar = 8.5 cm. (**b**) average fresh weight, (**c**) average dry weight of Dular and PB1 under different P regimes. (**d**) Root hair length of 5-days-old MS grown Dular and PB1 with 0, 100 and 320 μM concentrations of Pi (KH_2_PO_4_). (**e**) Total P content of hydroponically grown seedlings under 1, 100 and 320 μM concentrations of Pi (NaH_2_PO_4_). *p values* determined by student t-test using PB1 as control for each condition have been represented as ‘***’=*p* ≤ *0.001*, ‘**’ =*p* ≤ *0.01,* ‘*’=*p* ≤ *0.05* and ‘ns’ = not significant.

Dular has been proven to be one of the most tolerant genotype under low P in field conditions^13^. We found similar trend in our experiments under low P media in hydroponic system (Fig. 1a). While PB1 showed a drastic reduction in shoot and root biomass; Dular still maintains higher biomass accumulation than PB1 under low P (1 μM) (Figure 1a-c). Our analysis further revealed that PB1 is highly P responsive as it fosters its growth under increasing concentrations of Pi in media (Fig. 1b,c). However, Dular being a traditional genotype is able to sustain its growth under low P environment. Root hairs (RH) are one of the most important root traits for P uptake^5^. Increase in RH length in Dular (lesser in PB1) also reflects its better adaptation resources under low P (Fig. 1d). Similarly, Dular showed better performance in terms of growth parameters like tiller number, leaf width, root number, shoot biomass and root biomass in pot system under low P (Fig.S1). Analysis of total P content revealed significantly higher P demand of PB1 under P sufficient conditions (100 and 320 μM Pi) as compared to Dular (Fig. 1e). But under P deficient condition (1 μM Pi) Dular has relatively higher P content both in root and shoot tissues. All this information collectively showed that Dular is a ‘P-efficient’ genotype^21^ whereas PB1 is a ‘P-responsive’ genotype^22^,^23^. This contrasting behaviour of PB1 and Dular towards P homeostasis prompted us to investigate nucleotide polymorphism in these genotypes at genome levels.

### Whole genome resequencing of rice genotypes

**Figure 2:**
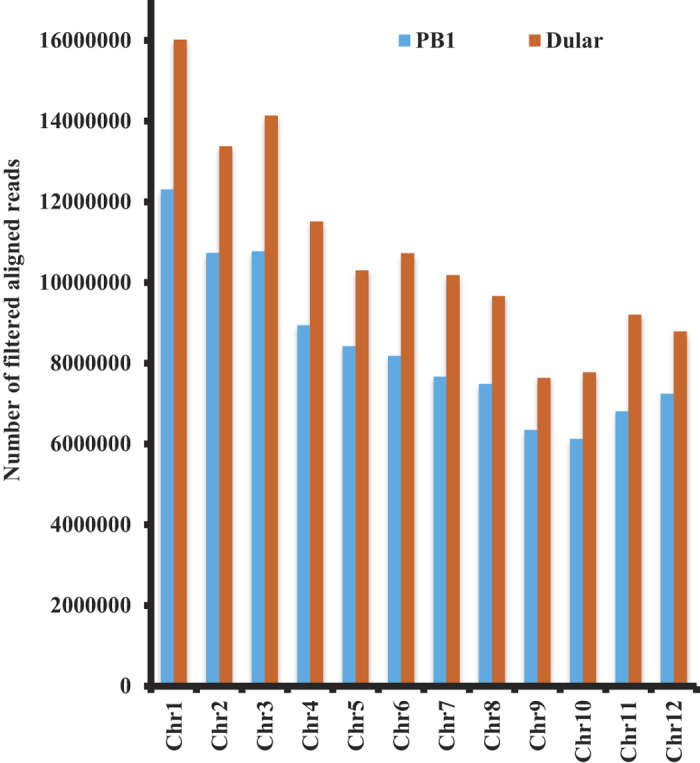
Chromosome-wise read count distribution of all filtered reads in PB1 and Dular. Reads were obtained throughout the rice genome and distributed on different chromosomes using Nipponbare sequence as reference (see methods for details). Raw reads were filtered using the criteria of MAPQ>20 and insert size 100–1000 bp.

Whole genome resequencing of Dular and PB1 revealed the occurrence of DNA polymorphisms at a genome-wide scale and their probable effect on differential low P tolerance in these genotypes. We obtained ~173 and 224 million sequence reads (average 101 bp reads) each in PB1 and Dular, respectively, with ~85% high quality sequences (Q30 passed quality score) (Table 1). Overall ~87% of the total pre-processed sequence reads from PB1 and Dular mapped to the reference Nipponbare genome with an average mapping quality (MAPQ) of 32.0 (Figure S2a) and average insert size of ~275–280 bp (Figure S2b). The average read depth and coverage obtained for each chromosome was 35.31X and 94.53% for PB1 and 46.14X and 94.04% for Dular, respectively (Table S1). Overall 58% of the total reads passed the alignment filter’s criteria (see methods). Total filtered reads were uniformly distributed across all the 12 rice chromosomes (Fig. 2). Approximately, 82% (306.1 Mb) of the reference genome of Nipponbare was covered by filtered reads of both genotypes with an average depth of 24X and 30.6X in PB1 and Dular, respectively (Table S2). Such a high coverage and read depth indicates the high quality assembly and sequencing data in accordance with earlier reports^24^.

### Discovery and validation of genome-wide sequence variants

We identified a total of 5,157,939 variants in low P sensitive PB1 and tolerant Dular with reference to low P sensitive Nipponbare reference genome (Table S3). Further, approximately 2.3 million high quality variants were mined in PB1, while 2.9 million variants were discovered in traditional low P tolerant genotype Dular. Out of which, 2,526,661 and 345,504 variants were high quality SNPs and InDels, respectively (Table S3). *In silico* validation of identified polymorphisms was performed with earlier SNP data (dbSNPs), which revealed ~26 to 28% SNPs are common between previous and present study. The remaining ~72% and 74% SNPs along with ~96% InDels discovered in each of PB1 and Dular as compared to Nipponbare are novel. Further, we validated the identified variants by Sanger-based amplicon sequencing of 50 randomly selected SNPs and InDels from both genotypes (Table S4). All the variants were successfully validated giving 100% experimental validation success rate. Majority of the variants (~87% of SNPs and ~92% of InDels) identified were of homozygous type. Majority of InDels identified in both genotypes had single base insertion or deletion. However, we also detected 7,779 and 10,524 insertions and 15,282 and 20,591 deletions of up to ≥9 nucleotides in PB1 and Dular, respectively (Figure S3b). Thus, *aus* genotype Dular showed higher DNA polymorphisms with reference to *japonica* genotype, Nipponbare as compared to *indica* genotype PB1. This is in concordance with previous study^18^ where highest numbers of SNPs were detected in *aus* rice genotype due to its higher evolutionary divergence from *japonica* than *indica* rice^18^.

The percentage of homozygous and heterozygous variants was almost similar in both the genotypes. More than 96% of all the identified variants have quality score ≥100 (Table S5a). About 62% of the reads in Dular and 40% of the reads in PB1 had a read depth ≥30, which ensures high quality and reliable variant calling (Table S5b). Therefore, heterozygous SNPs (~13%) in both of the rice genotypes are valid and non-erroneous. Similar level of heterozygous SNPs has also been estimated in previous genome-wide SNP discovery studies in diverse rice genotypes^18^,^24^. The total number of transitional changes (Ts) observed were more than twice as compared to transversion changes (Tv) in both PB1 and Dular (Figure S3a). A higher Ts/Tv ratio of 2.4 indicates ‘transition bias’. Transitions are usually favoured over transversions because transitions provide easy tolerance from selection pressure as they result into synonymous substitutions, which do not alter the conformational structures of protein unlike transversions^25^. Moreover, transitions affect the RNA secondary structure less severely than transversions. Therefore, transition bias has been frequently reported in rice and many other plant species^26^,^27^,^28^. However, in our study, the detected Ts/Tv ratio is higher than the previously reported Ts/Tv ratios in rice^24^,^28^. A higher Ts/Tv ratio is also suggestive of low level of genetic divergence. These ratios are expected to decline with increasing genetic distance between the comparative genotypes as in due course of time; transversions erase the record of frequent transitions^29^. Collectively, this suggests that resequencing of low P tolerant (Dular) and sensitive (PB1 and Nipponbare) rice genotypes are reliable and robust for utilization in large-scale genotyping applications.

### Genomic distribution of variants

**Figure 3:**
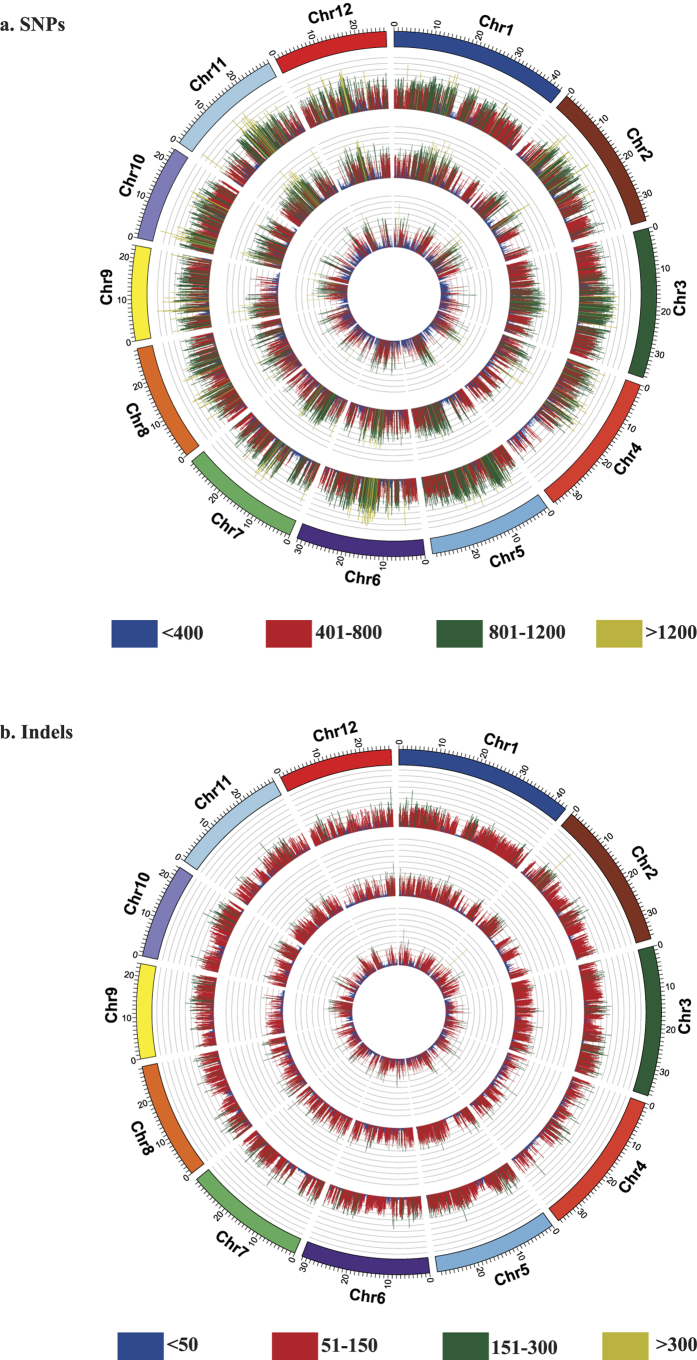
Distribution of variant density across rice chromosomes. Distribution of SNP (**a**) and InDel (**b**) density on all 12 rice chromosomes. Variant densities were computed across 100 kb window size. Outer circle of Circos diagram represents variants present in Dular, inner circle represents variant density in PB1 and innermost circle represents variant density of Dular-specific variants. Color scale of range representing variant densities has been depicted at bottom.

Variant distribution was not uniform across the chromosomes (Table S6, Fig. 3). We detected an average of 578 SNPs and 74 InDels in PB1 and 677 SNPs and 92 InDels in Dular per 100 kb (Table S6). In PB1, highest SNP density (666 SNPs/100 kb) was observed in chromosome 11, whereas it was maximum in Dular chromosome 10 (780 SNPs/100 kb). We found lowest SNP density on chromosome 2 (403 SNPs/100 kb) of PB1 and on chromosome 4 (516 SNPs/100kb) of Dular. Interestingly, Dular had a higher density of SNPs than PB1 across all 12 rice chromosomes which could be due to its higher phylogenomic divergence from *japonica* rice^18^.

Further, we identified the high and low resolution SNP regions with a stringent criterion of ≥1000 SNPs/100 kb and ≤20 SNPs/100 kb, respectively^24^. Based on this, 281 SNP high and 79 low resolution genomic regions were identified in PB1. Similarly, 514 SNP high and 27 low resolution genomic regions were detected in Dular. Highest numbers of high resolution genomic regions (59) were identified in chromosome 6 of Dular. However in PB1, chromosome 11 possessed a maximum number of SNP high resolution regions (47). Such variation in chromosomal distribution of polymorphisms has been attributed to the selective sweep-based natural selection^30^ and has been frequently reported in rice^27^,^31^.

### Structural and functional annotation of variants

Of the total variants, ~18% of the SNPs and ~21% of the InDels fell in the genic regions. In terms of genotypes, Dular had more number of variants in genic region (Table 2). Approximately, 27% of the genic SNPs and 10% of the genic InDels lied in coding region in both genotypes. This frequency of variants in coding is higher than earlier reports^24^,^28^,^31^. A large number of polymorphisms were also detected in up and down stream regulatory regions (Table 2). These polymorphisms in regulatory regions might be functionally relevant and can regulate the functions of associated genes^32^. It is noteworthy that many UTR- based regulatory polymorphisms have also been successfully employed to generate potential trait-associated markers in plants^33^,^34^. Interestingly, majority of the genic variants were intronic. Approximately, 52% of genic SNPs were identified in intronic regions. SNPs in intronic regions may reside at splice junctions^35^ or in intron splice enhancer or silencer elements^36^. Thus, such intronic SNPs may potentially change the fate of splicing and can alter protein structure and function^37^,^38^. Intronic SNPs may also shift the expression dynamics of crucial genes or regulate the transcript level of these genes by changing the binding sites of miRNAs which sometime reside in the intronic regions^39^.

All coding variants were classified as silent, missense, nonsense, startloss, stoploss, inframe and frameshift polymorphisms to dissect their functional relevance (Table S7). The number of identified non-synonymous polymorphisms (~50%) was higher than the synonymous polymorphisms (44%) in both genotypes. Nonsynonymous or missense variants can lead to drastic phenotypic consequences which could be either deleterious or beneficial^40^,^41^. Deleterious polymorphisms are mostly eliminated from the population through negative selection whereas beneficial polymorphisms could be fixed in the population leading to differential responses of cultivars towards biotic and abiotic stresses^42^.

### Structural and functional annotation of variants between PB1 and Dular

To extract the potential polymorphisms responsible for low P tolerance in Dular, we identified a total of 2,442,979 polymorphisms (SNPs and InDels) directly between Dular and PB1 (Table S8). Among these, 958,296 variants (SNPs and InDels) were specifically present in PB1 with respect to Nipponbare. Remaining 1,484,683 variants were absent in low P sensitive genotype PB1. It is noteworthy that the reference genome, Nipponbare is also a low P sensitive genotype^13^,^16^,^43^,^44^. Of the total polymorphisms between Dular and PB1, 86% were SNPs and remaining ~14% were InDels.

**Figure 4:**
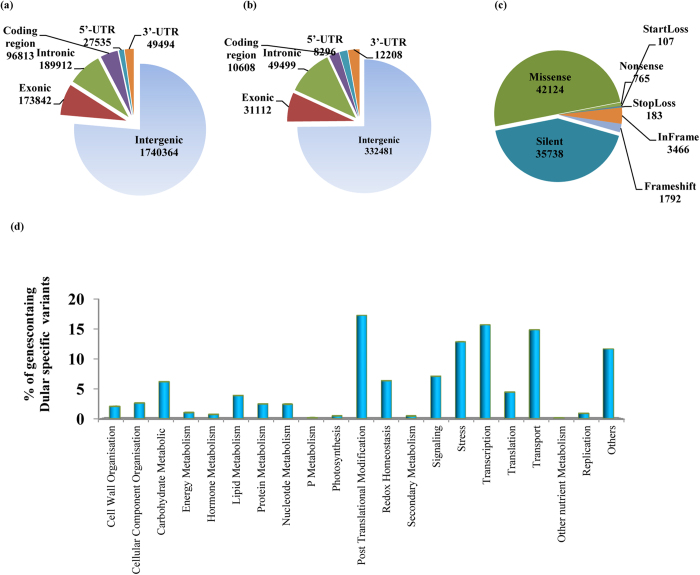
Structural and functional annotation of variants between Dular and PB1. Graphical illustrations of variant annotations of Dular vs PB1. (**a**) SNPs and (**b**) InDels. (**c**) Different class predictions of variants (SNPs and InDels) between Dular and PB1. (**d**) GO functional class prediction of genes associated with Dular vs PB1 polymorphisms (SNPs and InDels). Gene ontology classes were identified from Rice Oligonucleotide Array Database.

Structural annotation of polymorphisms between Dular and PB1 revealed that approximately 85% and 15% of total were intergenic and genic, respectively (Fig. 4a,b). Further, among the genic polymorphisms, we detected 1,73,842 SNPs and 31,112 InDels in exonic regions. A relatively higher percentage of genic variants comprising 52% SNPs and 62% InDels were intronic, whereas 27% SNPs and 13% InDels were detected in coding regions. Many InDels were also identified in regulatory regions. Functional annotation of these variants revealed 42,124 missense and 35,738 silent variants (Fig. 4c).

We further mapped these variants to 23,218 rice genes and assigned them GO classes (Fig. 4d). Variants were falling in various functional classes, including cell wall organisation, primary and secondary metabolisms, signalling, stress pathways, regulatory pathway and other miscellaneous classes. Importantly, the energy metabolism pathways remain largely unaffected due to their vital roles for sustaining life. These genotypes differ in their response to environmental stresses as also reflected by high number of variants in “stress” genes. Whether these variants also determine their behaviour under low P would be a subject of future research.

### Variants in low P responsive genes

**Figure 5:**
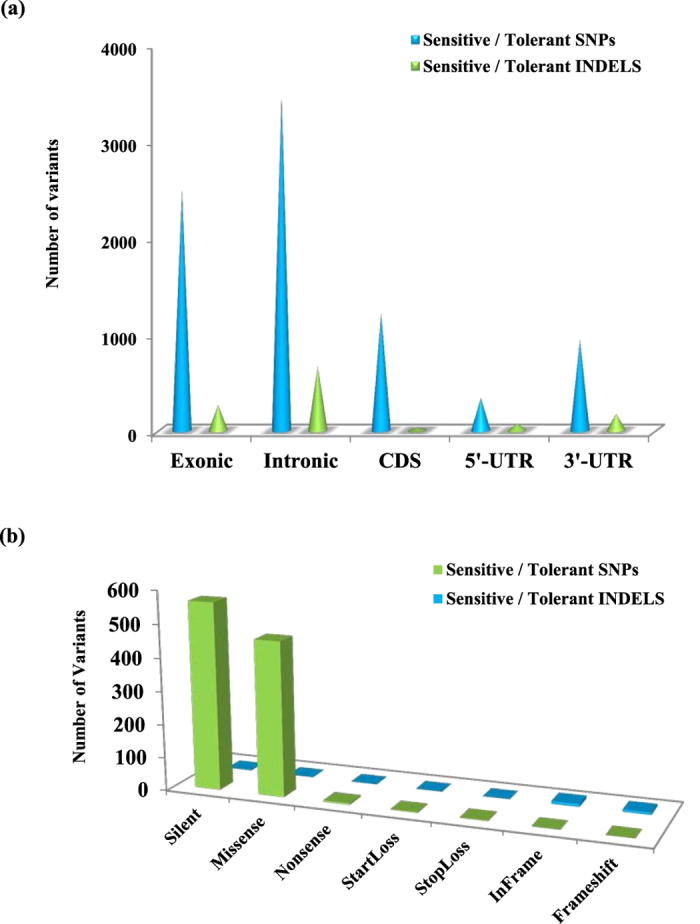
Annotation and class prediction of variants present in Phosphate Starvation Responsive (PSR) genes. (**a**) Variant (SNPs/InDels) annotation underlying PSR genes between sensitive (PB1 and Nipponbare) and tolerant (Dular and Kasalath) group. (**b**) Functional class distribution of SNPs/InDels underlying PSR genes between sensitive (PB1 and Nipponbare) and tolerant (Dular and Kasalath) group.

Mechanisms for low P tolerance are orchestrated by a set of PSR genes. We mapped all the variants between Dular and PB1 on these selected PSR (2152) genes (see methods). We found 1,731 PSR genes containing 18,872 SNPs and 4,271 InDels between Dular and PB1 (Table S9). To deduce more promising SNP/InDel variants potentially associated with low P tolerance, we compared all variants between Dular and PB1, underlying PSR genes with another low P tolerant *aus* genotype, Kasalath variants^45^. Interestingly, we found 30% variants identical in Dular and Kasalath (Table S10). This could be due to the same ‘*aus*’ group of both Dular and Kasalath as well as low P tolerance of both genotypes. These common variants in Dular and Kasalath can be further validated in natural and mapping population to establish their potential association with low P tolerance. Moreover, to address the question of differential genotypic behaviour of low P sensitive and tolerant cultivars, we also identified variants of PSR genes between two low P sensitive genotypes (Nipponbare and PB1) and two low P tolerant genotypes (Dular and Kasalath). From this analysis, we found 930 PSR genes containing 5,948 SNPs and 923 InDels in sensitive vs tolerant group (Table S10). However, to discern the differences between contrasting genotypes for low P tolerance, we annotated all such PSR gene variants. We found 2,481 exonic SNPs underlying PSR genes (Fig. 5a). We detected 1211 and 1270 PSR SNPs in CDS and regulatory regions (UTR), respectively. Thus, variation in PSR gene polymorphisms between sensitive and tolerant genotypes further strengthens the role of these genes in their differential low P response.

**Figure 6:**
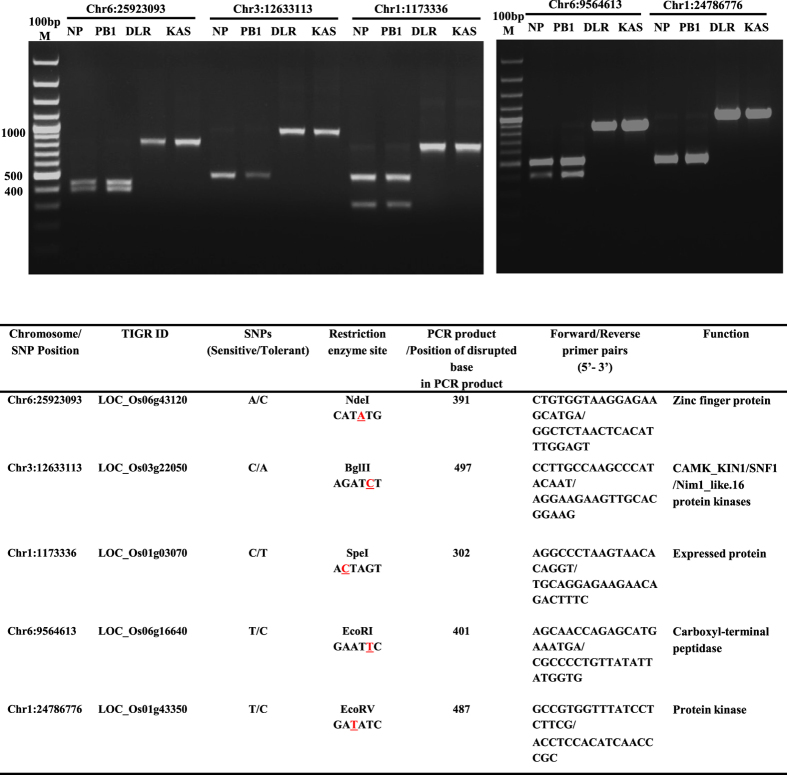
Validation of predicted CAPS (Cleaved Amplified Polymorphic Sequences) polymorphisms caused by disruption of restriction endonuclease site by Dular-specific exonic SNPs underlying PSR genes. Position of SNP underlying restriction site has been indicated on the top with details mentioned in table below. NP, PB1, DLR and KAS indicate Nipponbare, PB1, Dular and Kasalath rice genotypes, respectively. Altered base underlying restriction site has been highlighted in red.

In order to explore the potential use of these SNP variants (sensitive vs tolerant genotypes) in breeding programmes, we also identified 149 restriction sites disrupted by these SNPs in exonic regions of PSR genes (Table S10). These 149 SNPs falling in 116 PSR genes can be converted into allele-specific PCR based marker system for their validation and genotyping in a larger set of germplasm lines and bi-parental mapping population. We have successfully verified and converted five of these randomly selected SNP loci into CAPS (Cleavage Amplified Polymorphic Sequences) markers by use of a common set of restriction enzymes in Dular, PB1, Nipponbare and Kasalath genotypes (Fig. 6). Conversion of SNPs into CAPS markers can be an efficient and cost effective system for large scale genotyping of SNPs in natural and mapping population to expedite marker-assisted selection in developing high yielding low P tolerant cultivars^46^. The validation of SNPs with CAPS markers further suggests the reliability and robustness of our SNPs discovery through whole genome resequencing.

### Functional relevance of sequence variants in PSR genes between tolerant and sensitive genotypes

To further, dissect the significance of identified PSR variants between sensitive (PB1 and Nipponbare) vs tolerant (Dular and Kasalath) group, we assigned them functional classes. We identified 564 synonymous and 465 nonsynonymous substitutions in PSR genes containing variants. There were 259 PSR genes associated with nonsynonymous substitutions (Fig. 5b). Some genes like peptidyl-prolyl isomerase, protein kinases, zinc-finger family protein, glucan endo-1,3-beta-glucosidase possessed 8 to 16 missense variants (Table S10). Interestingly, kinase domain containing proteins were overrepresented in our analysis of missense variants. About 34 kinases contained nonsynonymous substitutions between sensitive vs tolerant group. Besides this, we detected 25 large effect variants in 21 PSR genes. These large effect variants include loss of stop codon, introduction of premature stop codon, loss of start codon and frameshifts in coding frame leading to generation of non-functional protein products^18^,^47^. We further explored the identified PSR gene variants for their presence in SNP high resolution genomic region. Our analysis revealed about 28 PSR genes (Table S11) containing SNPs encompassed SNP high resolution genomic regions. These genes include ABC transporter, Ser/Thr phosphatase, glycosyl hydrolase, WD domain containing protein and phosphatidylinositol 3-kinase. Interestingly, these families of genes are significantly known to be involved in PSR.

### Variants in phosphatases and genes involved in lipid metabolism

Phosphatases play vital roles in signaling and internal P utilization under P deficiency. We found 7 missense SNPs between sensitive vs tolerant genotypes in 5 different phosphatases (Table 3, Table S10). It is noteworthy that protein phosphatase 2C (Os02g0471500) contained 3 missense SNPs and one InDel. Besides phosphatases, variants were also identified in lipid metabolism genes. We found a missense SNP in glycerophosphoryl diester phosphodiesterase gene (Os08g0535700). This PSR gene is involved in release of sn-glycerol 3-phosphate under P starvation. SNP in CDS region of this biomarker gene can change the fate of this GDPD under P deficiency. Moreover, a MGD2 (monogalacatosyl diacyl glycerol synthase) gene showed one InDel. This PSR gene is involved in galactolipids synthesis under P deficiency 48,49,50. Further investigation of these genes can shed light on differential role of this variant in galactolipids accumulation under P deficiency.

### Variants in genes involved in carbohydrate metabolism

Carbohydrate metabolism genes alter the rate of P consumption under its deficiency through switching the alternate glycolytic pathway which utilises lesser amount of P^51^. We found five missense SNPs in some key carbohydrate metabolic genes. Interestingly, we identified SNPs in pyrophosphate-fructose 6-phosphate 1-phosphotransferase gene (Os02g0714200), which use a diphosphate and convert this diphosphate to monophosphate. Moreover, we detected higher number of variants associated with another carbohydrate metabolic PSR gene, phosphoenolpyruvate carboxylase (Os01g0208700) which showed 33 intronic SNPs. This gene plays crucial role in releasing phosphate during carbon fixation.

### Variants in genes encoding membrane transporters

Here, we identified 20 missense SNPs between low P sensitive and tolerant genotypes in various membrane transporters. It is noteworthy that only low P sensitive genotype PB1 showed accumulation of 28 SNPs in 5′UTR and one missense SNP in coding region of inorganic P transporter gene (Os04g0186400) with respect to all other 3 genotypes (Table S9). Recently same gene (*OsPht1;4*) has been found to be highly expressing in root tissues and essential for phosphate acquisition under P deficiency^52^. Since PB1 is a high yielding variety which being P responsive demands higher accumulation of P as compared to low P tolerant traditional genotypes Dular and Kasalath, investigating this transporter in diverse genotypes would help understand the role of these variants affecting its transcriptional regulation under low P.

### Variants in genes involved in RSA

RSA modulation is a key strategy to alleviate the P deficiency by increasing top soil foraging^5^. Traditional and modern genotypes differ significantly in their RSA under P deficiency. Higher density of root hairs, increased lateral root length and density contribute significantly to improve the P deficiency by enhancing P uptake from soil solution to plant cells^53^. We identified 7 missense variants in PSR genes involved in RSA modulation like lateral root development (Table S10). It is noteworthy here that Dular responds to low P by altering its root architecture while sensitive genotype failed to do so. *PSTOL1* gene is responsible for RSA alteration in low P tolerant traditional genotype Kasalath^9^. However, we found *PSTOL1* locus between Dular and PB1 without any nucleotide change; therefore, variants identified here become more relevant to investigate the novel PSR candidates.

In conclusion, we have identified many DNA polymorphisms in key PSR genes in low P tolerant and sensitive genotypes. Dular and Kasalath are highly tolerant to low P due to efficient root system architecture. Given this, variants identified here can serve as an important resource to improve root system architecture of elite rice cultivars for low P tolerance. Large number of sequence variants were detected between tolerant (Dular and Kasalath) and sensitive (PB1 and Nipponbare) genotypes; and also in PSR genes, probably due to their divergence from *japonica* and *indica* group. However, partly this could also be due to their unique adaptation to marginal soil. A larger population screen is needed to investigate this notion.

## Methods

### Plant material and phenotyping under low P environment

Seeds of PB1 and Dular genotypes were surface-sterilized with 0.1% mercuric chloride containing few drops of Triton X-100 for 15 minutes. Seeds were then washed 5–7 times with sterile H_2_O to wash off detergent. Sterilized seeds were germinated on wet filter paper for two days in dark. Uniformly germinated seedlings were transferred to different concentrations of Pi (1, 100 and 320 μM of NaH_2_PO_4_) in Yoshida medium with iron supplemented as FeNaEDTA. Seedlings of both genotypes were raised in separate containers in growth chamber with 16 h day (30 °C)/8 h night (28 °C) photoperiod, 250–300 μM photons/m^2^/sec photon density and ∼70% relative humidity. 30 seedlings per genotype were raised in 15 litres containers filled with nutrient solution. Nutrient medium was changed every day and pH was maintained strictly around 5.5. Root and shoot biomass was recorded after 15 days of growth in hydroponics. For measurement of total P content, root and shoot tissue of both genotypes was oven dried at 80 °C till constant weight. Dried plant material was weighed and subjected to ashing at 550 °C for 5 hrs in a muffle furnace. Total P content was measured colorimetrically by Ammonium Vanadate-Ammonium Molybdate yellow method as described^54^.

For analysis of root hair, excised embryos of PB1 and Dular seeds were surface sterilized and germinated in dark on MS^55^ medium containing 0.2% phytagel (Sigma P8169). Only embryo part was used to minimize the intrinsic variability of different seed vigour. After two days, uniformly germinated seedlings were transferred to MS medium containing 0, 100, 320 μM of KH_2_PO_4_ with 0.2% (w/v) phytagel. In -P medium, KH_2_PO_4_ was replaced with equimolar concentration of K_2_SO_4_. After 5 days of growth, root images were analysed in ImageJ 1.46r (http://imagej.nih.gov/ij) for root hair length measurement. Seeds were also sown in pots filled with a mixture of sand and perlite in order to study the effect of Pi deficiency in soil-grown plants. Plants were kept in a field under natural conditions and irrigated with Yoshida medium carrying low and high Pi for six weeks.

### Extraction and sequencing of genomic DNA

Genomic DNA (gDNA) was extracted from leaf tissue using Qiagen DNeasy kit (Qiagen USA) as per manufacturer’s instructions. Integrity of gDNA was assessed by Bioanalyzer 2100 (Agilent Technologies, Singapore). gDNA sample preparation for sequencing was done using Illumina TruSeq DNA sample preparation kit (Illumina, USA). Briefly, one μg high quality gDNA was subjected to fragmentation by Covaris shearing to obtain a final library of 300 to 400 bp average insert size. Sheared DNA was then subjected to end repairing, adenylation of 3′ ends followed by ligation of indexed paired end adapters. Ligated products falling in size range of 400–500 bp were purified using Qiagen MinElute gel extraction kit and selectively enriched by PCR. The libraries so generated were validated for right fragment size, concentration and pooled as per manufactures’ instructions. Finally, libraries were sequenced by Illumina HiSeq 2000 NGS platform.

### Read alignment and filtering

Read quality check and alignment were performed according to standard Illumina analysis pipeline. Low quality sequence reads were excluded from further analysis and only high quality sequences with mapping quality (MAPQ) ≥20 were retained for read alignment. Before performing the alignment, raw reads were first trimmed based on base quality, base composition, and adapter sequence. After trimming, reads <30 bp was removed from further analysis. The trimmed reads were aligned to the reference genome, Nipponbare (IRGSP-1.0 pseudomolecule/MSU7). Alignment was performed using BWA program with -q20 parameter to trim the low quality portion of the read. The aligned reads were first sorted using Picard tool and SortSam subsequently. Duplicate reads were removed using Picard Mark Duplicates command. After removing duplicate reads the reads were realigned to reference genome.

### Variant identification and annotation

After performing realignment, Genome Analysis TKLite-2.3-9 toolkit Unified Genotyper^56^ was used to identify single nucleotide polymorphisms (SNPs) and short InDels. After calling, variants were further filtered for read depth and quality score parameters in order to retain the good quality variants. Variants with stringent criteria of read depth ≥10 and quality score  ≥ 50 at corresponding regions in both the genotypes were considered for further analysis. Genomic distribution of variants (SNPs/InDels) was analysed in 100 kb sliding window size across all rice chromosomes in PB1 and Dular. Genomic distribution of SNPs/InDels/100 kb was represented pictorially using Circos program^57^. The identified variants were annotated using VariMAT program. The gene models used for annotation were downloaded from plant Ensembl database. The VariMAT (SciGenome, India) programme was used for genic and intergenic, exon, intron, 5′UTR, 3′UTR, coding-region, splice-site annotation of SNPs and InDels. The same programme was also used for variant class prediction (silent, missense, non-sense, stop-loss, start-loss, inframe, frameshift) and mapping of variants to all transcript form of the gene.

### Validation of SNPs and InDels

A total of 50 SNPs/InDels in Dular were randomly selected from all 12 rice chromosomes. Primers were designed from ~150 bp flanking region of each variant. Approximately, 300 bp amplicons for each variant were amplified by PCR from both PB1 and Dular genomic DNA. The amplified PCR products were sequenced using automated AB1 3730xl DNA Analyzer.

### Development and validation of CAPS marker

About 500 bp flanking region of each Dular-specific SNP variant underlying PSR genes (identical to Kasalath) were retrieved from Rice Genome Browser (http://rice.plantbiology.msu.edu/cgi-bin/gbrowse/rice). These sequences were searched and annotated for the presence of restriction sites altered by the introduction of SNP at specific genomic position employing NEB cutter (http://nc2.neb.com/NEBcutter2/). The primer pairs (Fig. 6) amplifying 800–1000 bp fragments possessing the restriction sites alleles with SNPs were designed and amplified using genomic DNA of Dular, PB1, Nipponbare and Kasalath genotypes. PCR amplicons were digested with specific predicted restriction endonuclease as per manufacturer’s protocol (New England BioLabs, Inc). Polymorphisms were analysed through agarose gel electrophoresis.

### Identification and annotation of variants within PSR genes

For elucidating the functional relevance of variants in context of low P tolerance, a selected set of PSR genes, previously reported in five independent transcriptome studies 43,44,58,59,60 under P deficiency in different rice genotypes, were downloaded. A total of 2,152 differentially expressing unique PSR genes present in at least ≥2 transcriptomic studies were identified and selected for further analysis. All Dular vs PB1 variants present on these selected PSR genes were structurally and functionally annotated. Variants were also compared between low P sensitive genotypes (PB1 and Nipponbare) and low P tolerant genotypes (Dular and Kasalath) by utilizing publically accessible Kasalath genome sequence^45^.

## Additional Information

**How to cite this article**: Mehra, P. *et al.* Genome-wide DNA polymorphisms in low Phosphate tolerant and sensitive rice genotypes. *Sci. Rep.* **5**, 13090; doi: 10.1038/srep13090 (2015).

**Accession codes**: Sequencing data has been submitted in NCBISRA database with accession IDs (SRR2056129, SRR2056130, SRR2056128, SRR2056248).

## Supplementary Material

Supplementary Information

## Figures and Tables

**Table 1:** Raw read and alignment summary of whole genome resequencing data.

	**PB1**	**Dular**
Total raw reads	17,33,50,122	22,43,21,256
Total data (Gb)	17.5	22.7
Total data >= Q30 (%)	84.6	85.7
GC Content (%)	41.76	42.1
Trimming from front of read (R1, R2)	(5,5)	(5,5)
Trimming from end of read (R1, R2)	(3,3)	(3,3)
Total # of reads after pre-processing	17,33,21,104	22,42,73,890
Total read aligned (%)	150,300,232 (86.72%)	195,391.637 (87.12%)
Average mapping quality (MAPQ)	32.76	32.4
Average insert size (bp)	~276	~280
Total reads passed alignment filter	101,037,532 (58.29%)	129,313,224 (57.66%)

**Table 2:** Brief statistics of SNPs and InDels annotation in PB1 and Dular.

**Class**	**SNPs**		**InDels**	
	**PB1**	**Dular**	**PB1**	**Dular**
Intergenic	1654408	2085048	218014	272935
Genic	355583	441613	57869	72469
Exonic	1,68,648	2,11,921	20,692	26,915
Coding region	94920	118036	5356	7266
Pt Coding Gene ncRNA	203	250	51	59
UTR	73525	93635	15285	19590
5′-UTR	25650	33484	6049	7934
3′-UTR	47875	60151	9236	11656
Intronic	186935	229692	37177	45554
5-SpliceSite	157	184	82	107
3-SpliceSite	162	187	64	79
Others	186616	229321	37031	45368
db SNP variant region	554009	660414	11583	16058
Repeat region	847171	1068887	88522	111877

**Table 3:** Summary of PB1 vs Dular SNPs and InDels underlying Phosphate Starvation Responsive (PSR) and Root System Architecture (RSA) genes.

	**Phosphatases**	**Lipid metabolism**	**Carbohydrate metabolism**	**Transporters**	**Root System Architecture**
Total SNPs	177	20	97	259	80
a. Exonic	41	8	28	90	32
Non-synonymous	7	3	5	20	7
Synonymous	13	4	7	34	14
5′UTR	3	—	—	3	1
3′UTR	17	1	8	30	10
b. Intronic	136	12	69	169	48
Total InDels	26	6	10	37	5
a. Exonic	3	—	3	7	3
CDS	—	—	—	—	—
5′UTR	—	—	1	2	—
3′UTR	3	—	2	5	3
b. Intronic	23	6	7	30	2
Total number of PSR genes	28	7	8	25	17

PSR genes belonging to different functional classes were identified from published genome-wide transcriptome studies.
