# Supplementary material for: Genome-wide DNA polymorphisms in low Phosphate tolerant and sensitive rice genotypes
Source: Sci Rep. 2015 Aug 17;5:13090. doi: 10.1038/srep13090 (PMC4538390; doi:10.1038/srep13090)
Supplement: Supplementary Information [file srep13090-s1.pdf]

Genome-wide DNA polymorphism in low Phosphate tolerant and sensitive rice genotypes

Poonam Mehra[1], Bipin K. Pandey[1], Jitender Giri*

National Institute of Plant Genome Research, Aruna Asaf Ali Marg, New Delhi-110067, India

[1]contributed equally

*Author for correspondence

Jitender Giri

National Institute of Plant Genome Research, Aruna Asaf Ali Marg, New Delhi-110067, India

jitender@nipgr.ac.in

91-11-26735227

Fax No. : 91-11-26742658

Keywords: Phosphate starvation response (PSR); Root; SNPs; InDels; Whole genome resequencing; QTL

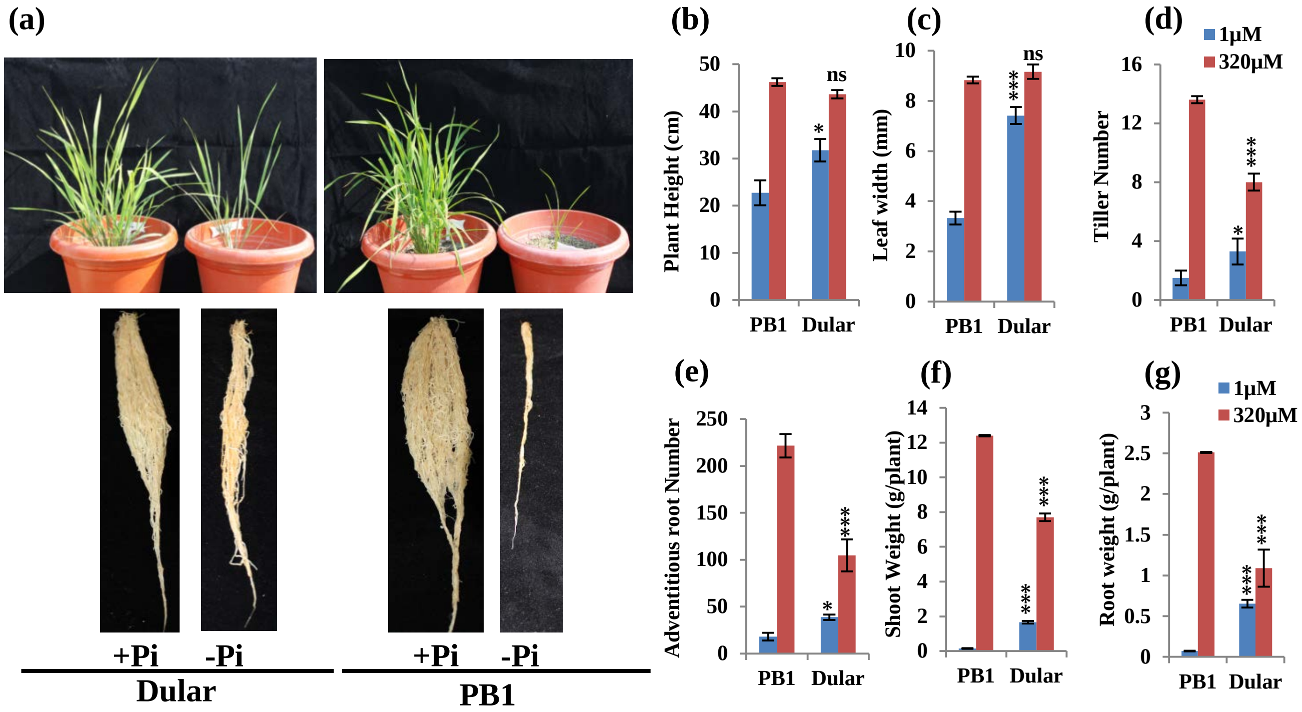

Figure S1 Phenotype of six week-old Dular and PB1 raised under Pi sufficient (320µM) and Pi deficient (1µM) conditions in soil. (a) root and shoot growth (b) height of above ground part (c) leaf width of Dular and PB1 measured at the centre of each leaf (d) tiller number per plant (e) adventitious root number (f) shoot dry weight per plant and (g) root dry weight per plant. All quantitative analysis was carried out with 5 replicates per genotype per treatment. *p values* determined by student t-test using PB1 as control for each treatment have been represented as '***'= $p \leq 0.001$, '**' = $p \leq 0.01$, '*' = $p \leq 0.05$ and 'ns'= not significant.

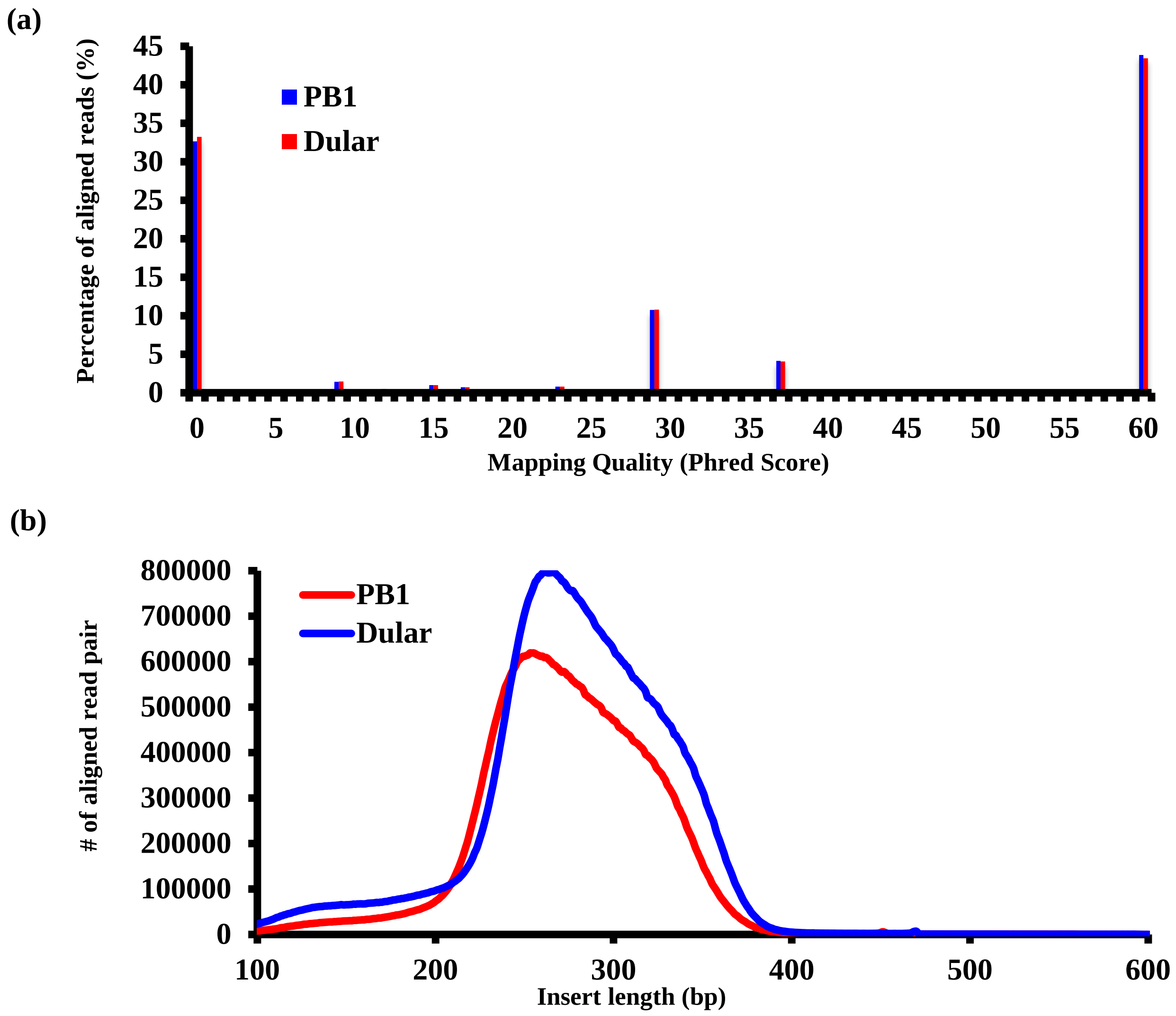

Figure S2 (a) Distribution of mapping quality of all aligned reads. (b) Insert-size distribution of all aligned reads in Dular and PB1.

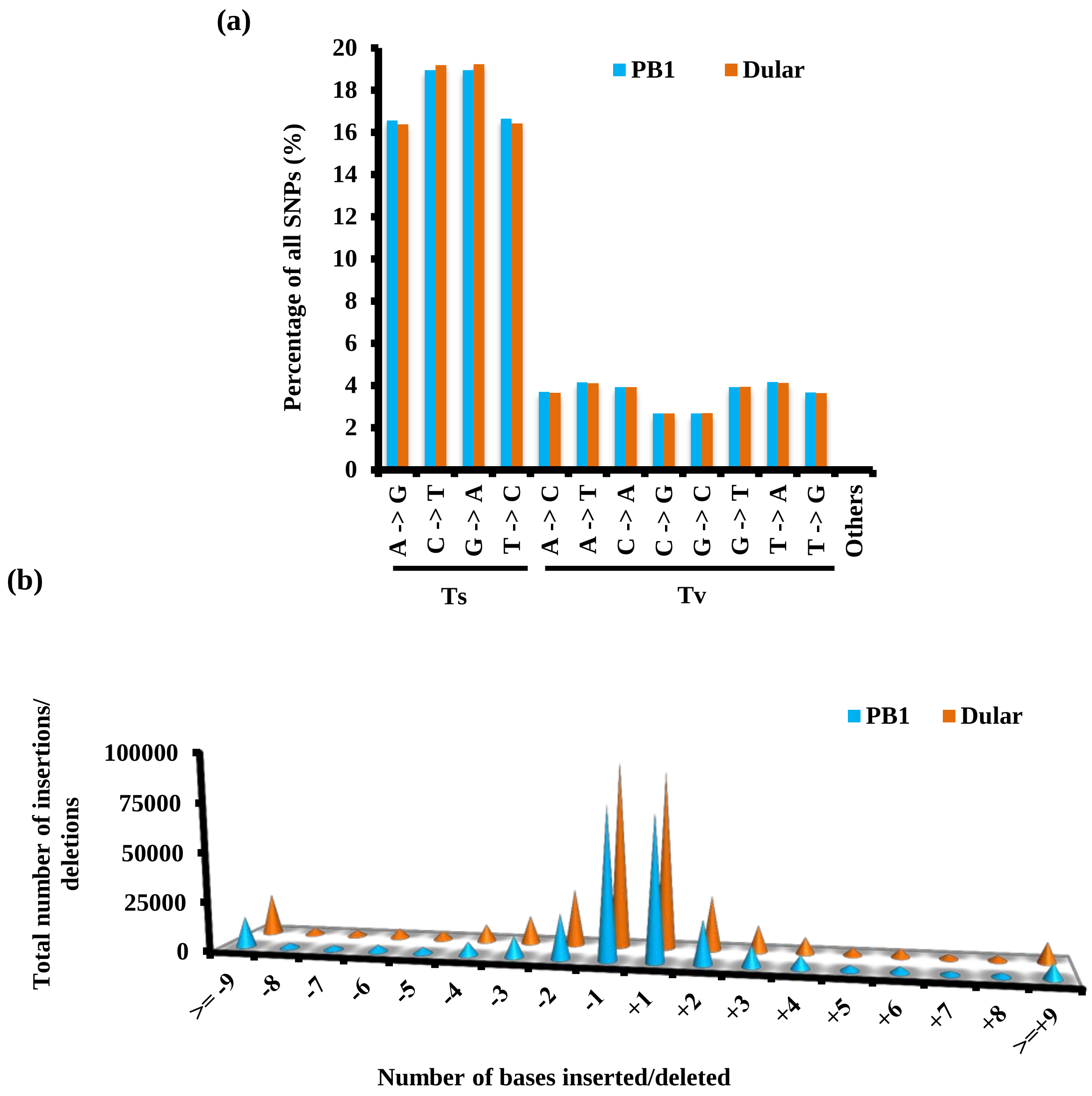

Figure S3 Frequency of transitions/transversions and insertions/deletions in Dular and PB1. (**a)** Frequency of transitions (Ts) and transversions (Tv) for SNPs in PB1 and Dular. Both genotypes showed more than twice the number of transitional changes (Ts) as compared to transversion changes (Tv). (**b)** Frequency of insertions and deletions observed for different nucleotide bases in PB1 and Dular.

Table S1: Read alignment summary for raw reads in PB1 and Dular with chromosome coverage and average read depth of sequencing

		Chromosome coverage (%)		Average Read Depth (X)	
**Chromosome**	**Chromosome length (bp)**	**PB1**	**Dular**	**PB1**	**Dular**
Chr1	4,32,70,922	95.69	95.49	33.93	44.6
Chr2	3,59,37,249	96.96	96	34.67	44.27
Chr3	3,64,13,818	97.28	97.19	32.16	42.65
Chr4	3,55,02,693	93.65	92.73	40.58	53.69
Chr5	2,99,58,433	96.04	96.04	35.27	42.83
Chr6	3,12,48,786	94.94	94.71	32.68	43.19
Chr7	2,96,97,620	93.76	93.68	31.9	43.23
Chr8	2,84,43,021	94.57	94.26	35.37	47.13
Chr9	2,30,12,719	94.66	92.93	36.79	46.53
Chr10	2,32,07,286	93.21	92.52	50.89	68.31
Chr11	2,90,21,105	90.08	91	28.12	37.91
Chr12	2,75,31,855	91.44	89.34	35.56	44.56
**Total/Average**	**37,32,45,507**	**94.53**	**94.04**	**35.31**	**46.14**

Table S2 Read alignment summary for filtered reads in PB1 and Dular with chromosome coverage and average read depth of sequencing

		**Chromosome coverage (%)**		**Average Read Depth (X)**	
**Chromosome**	**Chromosome length (bp)**	**PB1**	**Dular**	**PB1**	**Dular**
Chr1	4,32,70,922	86.61	86.39	25.03	32.67
Chr2	3,59,37,249	90.23	87.75	26.34	32.89
Chr3	3,64,13,818	90.31	90.31	26.04	34.28
Chr4	3,55,02,693	78.77	77.95	22.18	28.68
Chr5	2,99,58,433	87.25	83.44	24.78	30.37
Chr6	3,12,48,786	82.26	82.89	23.05	30.35
Chr7	2,96,97,620	81.69	82.44	22.72	30.32
Chr8	2,84,43,021	82.41	80.88	23.19	30.05
Chr9	2,30,12,719	83.78	80.85	24.34	29.32
Chr10	2,32,07,286	80.54	79.51	24.29	29.63
Chr11	2,90,21,105	76.18	78.41	20.66	28.07
Chr12	2,75,31,855	77.58	74.14	23.24	28.25
**Total/Average**	**37,32,45,507**	**83.52**	**82.54**	**23.91**	**30.62**

Table S3 Details of variants calling of single nucleotide polymorphism (SNPs) and insertions/deletions (InDels) in PB1 and Dular

	**PB1**	**Dular**
Total Variants	22,85,874	28,72,065
Total SNPs	2,009,991 (87.93%)	2,526,661 (87.97%)
Total Homozygous SNPs	1,744,273 (86.78%)	2,204,499 (87.25%)
Total Heterozygous SNPs	265,718 (13.22%)	322,162 (12.75%)
Total Transition SNPs (Ts)	1,428,764 (71.08%)	1,799,367 (71.21%)
Total Transversion SNPs (Tv)	581,227 (298.92%)	727,294 (28.78%)
Ts/Tv	2.46	2.47
Total InDels	275,883 (12.07%)	3,45,404
Total Homozygous InDels	249,446 (93.88%)	317,422 (91.90%)
Total Heterozygous InDels	26,437 (6.12%)	27,982 (8.10%)

Table S4 List of randomly selected variants (SNPs/Indels) for PCR validation

	5'-3'		5'-3'
**SNP_F27**	GTGATGGATCTCCGTATG	**SNP_R27**	TACATTCCTTCAACTTACCTC
**SNP_F89**	AGTTCTTGATCAGCTACCAG	**SNP_R89**	ATACACCTCCGAGGAGA
**SNP_F31**	ATGCATAATCTTGTTAATCTCTAA	**SNP_R31**	CTGACACGAACAGGTTAATT
**SNP_F78**	ATGCTCTCTACTGGTAGTAGAAAT	**SNP_R78**	TTGTGACTACACCCGC
**SNP_F82**	AACCATTGAAATTCTAACCA	**SNP_R82**	TGACAATATTGTAATTTTACTAAACC
**SNP_F84**	AGAGGATTCATTGTGCTG	**SNP_R84**	GATCCAAGCTGCCTTTA
**SNP_F93**	CAGGCAACTGAACTAACTG	**SNP_R93**	CAATAAACAGGATGGATGTT
**SNP_F22**	AAAGAGGAATAGATTGCTAACTG	**SNP_R22**	CCGACATTAGTTGCTTGG
**SNP_F26**	CTAAATAAACACTTGTCAAGAAAT	**SNP_R26**	ATGTTTGTACTTTGTGGTAGTT
**SNP_F61**	GCAAACCTATGCTAATTTCTG	**SNP_R61**	TCAGTCACAATTCACACAAC
**SNP_F74**	AACATGTTGCAATCTTTAGC	**SNP_R74**	TTGAGTTGAACAGATGCAT
**SNP_F75**	GTCATTTTACTAGTTAGTGGCTTC	**SNP_R75**	ACTGCAGCACTTCATCACTT
**SNP_F14**	GTGAGTATTTCTTTTTCCTTCAG	**SNP_R14**	AGAAAGGACTTATCCTCGAG
**SNP_F19**	AAAGGTGTTGTTTTCACCTC	**SNP_R19**	TCAGCCCTTTATTATTGCA
**SNP_F34**	TGCTCATTCATGTATTCTTCA	**SNP_R34**	CAGTGTATGTGACACTGCG
**SNP_F43**	GCATAAAGTATCATCATTCACC	**SNP_R43**	GCCATTCAGGGATCTCTAT
**SNP_F48**	CGTTCGAATTCTCCAAAC	**SNP_R48**	TGTATATACGTACTAACATGCTCG
**SNP_F54**	AAGAATTGCACCCATGTC	**SNP_R54**	CTCTCTTTCTCAGAATATATGCAC
**SNP_F64**	GGGATCAATCAGAAGATGTC	**SNP_R64**	CATGAACAAACTTGAAGTACTCA
**SNP_F77**	AGAGATGACTCGACGAATCT	**SNP_R77**	CCAAGAAGCTCTTTGGAG
**SNP_F88**	AAACTTTATTAAACCCACCAAC	**SNP_R88**	GAGGAGATCATGCGGA
**SNP_F16**	TTCCCTGCAATCACATCA	**SNP_R16**	GAGACTCAAAACAGTACTTGACCTA
**SNP_F25**	GTGTTTTGTGTTCTAGGTGAG	**SNP_R25**	TCTCTATCACCAAATTTCTCA
**SNP_F59**	ACATCTCTTGCAGAAGGAC	**SNP_R59**	ATGCATTGGAATGTAACTTC
**SNP_F79**	AGCTACGAAGATAACAACTCA	**SNP_R79**	TATATATGCTATTGTTGTCGTGA
**SNP_F5**	GCCATGGATTCCTTCATC	**SNP_R5**	AGAAGGAAGAATCGAGAAATTAAC
**SNP_F13**	CCACTCTGACATAAGTATGATACTT	**SNP_R13**	CAAAACTGATGCACTAAGAGA
**SNP_F18**	CATGAACCTCCAGAAGATCC	**SNP_R18**	ACATCTCAGCTTTTTACACACAC
**SNP_F49**	GAGAACATACCACGTTTTACA	**SNP_R49**	ATCTAACCTTTGTCTGTGGAT
**SNP_F33**	AAATTTAGCAGGTGATAGCCAC	**SNP_R33**	AGCTAGTGAGAGGGTCGCT
**SNP_F66**	CACAAAATCATCCATCCATTCT	**SNP_R66**	CGTCTCTCTCGTCGGC
**SNP_F86**	TTCTGGATTTGTGCATCAAT	**SNP_R86**	TTTACAAAGGCAAAGTGAAGC
**SNP_F2**	GATCACACACACACCTTCCAT	**SNP_R2**	AAGTTGTTTCCACAGAGTGGTTAG
**SNP_F40**	GTGCTGAGCTATAGCTTTCTGC	**SNP_R40**	CTCGTGATCGACGGAGAC
**SNP_F56**	TGTTCCTTCATCATCCCG	**SNP_R56**	AATGCGTCAGTACCTCAAGG
**SNP_F57**	GCGGATCAAGATGACGAG	**SNP_R57**	CCAAGTGAGAATCTGGATGAAG
**SNP_F4**	AAGGATCTGAACAAGCGGC	**SNP_R4**	CTACGATCTCATTCTCCTCCG
**SNP_F12**	CGTCAACACATCTATCAGCAAA	**SNP_R12**	CTTGAAGCATTTTCATCTTCCAT
**SNP_F36**	CTTCTCCCTCCGCATCAC	**SNP_R36**	CCGACCATTGATTGATTCATTA
**SNP_F37**	AACGTTCGACGTGATGTGC	**SNP_R37**	GATCGGCAAGATCGACATC
**SNP_F45**	AGGGTACCCATGCTGTGG	**SNP_R45**	TCAAGAATTCAGTTTGCTCATACC
**SNP_F80**	AGCAGTGCCCTTCAAAAGTT	**SNP_R80**	CATGACCACCGGCGA
**SNP_F83**	AACAGCAATGCAGCAGGC	**SNP_R83**	GTTGTTCTTGGCTTTCAGTTTCTT
**SNP_F95**	AGAGGGCGACGTCGC	**SNP_R95**	GGAAATGGGCTTGGCAG
**SNP_F24**	CCCTAACATTCTCCCTCTTCTCTTC	**SNP_R24**	ACCACCAGCAACAAGAAACG
**SNP_F44**	GCGGTGCGAGGAAGAGG	**SNP_R44**	CACCTAGCCGGTAGGCACTAG
**SNP_F76**	GCGAAGCTGCACAGATCAAT	**SNP_R76**	AGATCATGACGGACGAGGC
**SNP_F81**	TCGTCGTCGGCTCGTC	**SNP_R81**	CACGACACCATGCGCC
**SNP_F87**	GTCGTCGGCGGCCTC	**SNP_R87**	GGGTTGAGTTTGGTCACCATT
**SNP_F7**	TGGCATTGGAAGGTGATGG	**SNP_R7**	CCGCCACCTTAGAAGAAGAAGA
**SNP_F15**	TTCATGCTGAAGCTGACTAGAAG	**SNP_R15**	TACTCCCAATCATCTTTTTCCG

Table S5a. Quality score distribution of all identified variants (SNPs/InDels) in PB1 and Dular

Quality Score	PB1		Dular	
	SNPs	InDels	SNPs	InDels
50-60	7863	44	7301	34
60-70	9680	44	9095	27
70-80	8788	59	9150	67
80-90	7710	73	8476	74
90-100	7692	111	8823	105
>=100	1968254	275550	2483796	345095

Table S5b. Read depth distribution of all identified variants (SNPs/InDels) in PB1 and Dular

Read depth	PB1		Dular	
	SNPs	InDel	SNPs	InDels
10-15	227110	32919	201896	27623
15-20	264387	41155	209767	33447
20-25	327526	49605	234467	39561
25-30	378043	53627	283078	46310
>=30	737617	93317	1285511	171482
>=50	75304	5258	311922	26979

Table S6 Total variants (SNPs and InDels) and average variant density across all rice chromosomes in PB1 and Dular

	PB1				Dular			
**Chromosome**	**Total SNPs**	**SNPs/100Kb**	**Total Indels**	**InDels/100Kb**	**Total SNPs**	**SNPs/100kb**	**Total Indels**	**InDels/100kb**
Chr1	227320	525.34	34810	80.45	299607	692.4	45290	104.67
Chr2	144890	403.17	22379	62.27	234935	653.74	33984	94.56
Chr3	240389	660.16	35140	96.5	266569	732.05	39157	107.53
Chr4	165077	464.97	21973	61.89	183365	516.48	23325	65.7
Chr5	168644	562.93	23887	79.73	232320	775.47	30983	103.42
Chr6	175598	561.94	24001	76.81	214411	686.14	28678	91.77
Chr7	173067	582.76	22725	76.52	175563	591.17	24827	83.6
Chr8	154943	544.75	20864	73.35	197703	695.08	26103	91.77
Chr9	98190	426.68	12231	53.15	166329	722.77	21913	95.22
Chr10	139969	603.13	17373	74.86	181173	780.67	22924	98.78
Chr11	193399	666.41	23966	82.58	195577	673.91	24639	84.9
Chr12	128505	466.75	16534	60.05	179109	650.55	23581	85.65
Total	2009991	538.52	275883	73.91	2526661	676.94	345404	92.54

Table S7 Functional class prediction for coding variants (SNPs and InDels) in PB1 and Dular

**Variant Class**	**PB1**	**Dular**
Silent	36339	45337
Missense	41570	51589
Nonsense	609	748
StartLoss	143	165
StopLoss	261	302
InFrame	2642	3694
Frameshift	1504	1955

Table S8 Summary of SNPs and InDels present between PB1 and Dular

	**PB1-specific SNPs/InDels**			**Dular-specific SNPs/InDels**			**Dular vs PB1 SNPs/InDels**		
	**SNPs**	**InDels**	**Total**	**SNPs**	**InDels**	**Total**	**SNPs**	**InDels**	**Total**
**Chr. 1**	81831	15831	97662	147631	26167	173798	229462	41998	271460
**Chr. 2**	57734	11173	68907	144446	22803	167249	202180	33976	236156
**Chr. 3**	80966	15578	96544	101056	19452	120508	182022	35030	217052
**Chr. 4**	96347	13996	110343	110269	15352	125621	206616	29348	235964
**Chr. 5**	54952	9755	64707	114242	16745	130987	169194	26500	195694
**Chr. 6**	92559	14607	107166	126530	19309	145839	219089	33916	253005
**Chr. 7**	87301	12694	99995	85111	14769	99880	172412	27463	199875
**Chr. 8**	67251	10119	77370	105272	15212	120484	172523	25331	197854
**Chr. 9**	42451	6813	49264	108041	16622	124663	150492	23435	173927
**Chr. 10**	39862	6400	46262	75906	11700	87606	115768	18100	133868
**Chr. 11**	87146	12709	99855	83130	13163	96293	170276	25872	196148
**Chr. 12**	34644	5577	40221	79413	12342	91755	114057	17919	131976
**Total**	**823044**	**135252**	**958296**	**1281047**	**203636**	**1484683**	**2104091**	**338888**	**2442979**

Table S9 List of Phosphate Starvation Response (PSR) genes containing variants (SNPs/InDels) between Dular and PB1

Chr	Variant Start	Variant End	Reference Base (Nipponbare)	Observed Alt Base (Dular)	Observed Base (PB1)	Variant	Gene	Variant Class	Inside Exon Info	Variant Class	TIGR ID	Function
Chr.6	1416121	1416129	AAGAGAGAG	AAGAG	A	INDEL	OS06G0126500	Exonic	3UTR	NA	LOC_Os06g03640	BAG domain containing protein, expressed
Chr.2	5251459	5251465	AAGAGAG	AAGAGAG	A	INDEL	OS02G0194200	Intronic	NA	NA	LOC_Os02g10080	zinc finger C-x8-C-x5-C-x3-H type family protein, expressed
Chr.4	7308911	7308915	AATCT	AATCTATCT	A	INDEL	OS04G0209200	Intronic	NA	NA	LOC_Os04g13210	multidrug resistance-associated protein, putative, expressed
Chr.1	39085646	39085647	AC	ACC	A	INDEL	OS01G0898900	Intronic	NA	NA	LOC_Os01g67330	nucleotide-sugar transporter family protein, putative, expressed
Chr.1	4338342	4338345	ACGG	ACGGCGGCGGCGG	A	INDEL	OS01G0182600	Exonic	5UTR	NA	LOC_Os01g08700	GIGANTEA, putative, expressed
Chr.11	28766354	28766357	ACGT	ACGTCGT	A	INDEL	OS11G0702400	Exonic	CDS	InFrame	LOC_Os11g47630	ZOS11-10 - C2H2 zinc finger protein, expressed
Chr.1	20584611	20584612	AG	AGG	A	INDEL	OS01G0550100	Intronic	NA	NA	LOC_Os01g36930	ubiquitin carboxyl-terminal hydrolase 6, putative, expressed
Chr7	24659684	24659685	AG	AGGG	A	INDEL	OS07G0603100	Intronic	NA	NA	LOC_Os07g41180	RNA-binding protein-like, putative, expressed
Chr.2	23527463	23527467	ATTTT	AT	A	INDEL	OS02G0601300	Intronic	NA	NA	LOC_Os02g38920	glyceraldehyde-3-phosphate dehydrogenase, putative, expressed
Chr.6	13480792	13480794	ATC	ATCTCTCTC	A	INDEL	OS06G0338200	Intronic	NA	NA	LOC_Os06g23114	copper methylamine oxidase precursor, putative, expressed
Chr.11	18831847	18831848	AT	ATT	A	INDEL	OS11G0522900	Exonic	5UTR	NA	LOC_Os11g31980	OsSCP63 - Putative Serine Carboxypeptidase homologue, expressed
Chr.10	20844687	20844688	AT	ATT	A	INDEL	OS10G0535800	Intronic	NA	NA	LOC_Os10g39100	uncharacterized Cys-rich domain containing protein, putative, expressed
Chr.6	27283078	27283079	AT	ATT	A	INDEL	OS06G0661900	Intronic	NA	NA	LOC_Os06g45110	DNA binding protein, putative, expressed
Chr.4	13648861	13648862	AT	ATTTT	A	INDEL	OS04G0304200	Intronic	NA	NA	LOC_Os04g23890	AGC_PVPK_like_kin82y.10 - ACG kinases include homologs to PKA, PKG and PKC, expressed
Chr.1	744428	744428	T	C	A	SNP	OS01G0113700	Intronic	NA	NA	LOC_Os01g02350	receptor-like kinase ARK1AS, putative, expressed
Chr.11	6639357	6639357	G	C	A	SNP	OS11G0226933	Exonic	CDS	NA	LOC_Os11g11960	disease resistance protein RPM1, putative, expressed
Chr.10	12023270	12023270	G	C	A	SNP	OS10G0377000	Intronic	NA	NA	LOC_Os10g23060	expressed protein
Chr.10	20551354	20551354	T	C	A	SNP	OS10G0528100	Exonic	CDS	Missense	LOC_Os10g38470	glutathione S-transferase, putative, expressed
Chr.5	26928314	26928314	G	C	A	SNP	OS05G0542500	Exonic	CDS	Silent	LOC_Os05g46480	late embryogenesis abundant protein, group 3, putative, expressed
Chr.10	20894551	20894551	C	CA	A	INDEL	OS10G0536500	Intronic	NA	NA	LOC_Os10g39150	expressed protein
Chr.2	17160424	17160424	C	CA	A	INDEL	OS02G0491400	Intronic	NA	NA	LOC_Os02g28980	peptidyl-prolyl isomerase, putative, expressed
Chr.1	39222425	39222425	C	CAAA	A	INDEL	OS01G0901000	Exonic	3UTR	NA	LOC_Os01g67500	armadillo/beta-catenin repeat family protein, putative, expressed
Chr.10	15403921	15403921	C	CCCCAAAA	A	INDEL	OS10G0431900	Intronic	NA	NA	LOC_Os10g29620	tyrosine protein kinase domain containing protein, putative, expressed
Chr.1	37048171	37048172	AT	CT	A	INDEL	OS01G0857400	Intronic	NA	NA	LOC_Os01g63854	transmembrane amino acid transporter protein, putative, expressed
Chr7	21414536	21414536	T	G	A	SNP	OS07G0541900	Exonic	3UTR	NA	LOC_Os07g35750	TKL_IRAK_DUF26-ld.3 - DUF26 kinases have homology to DUF26 containing loci, expressed
Chr.6	13480518	13480518	G	GAA	A	INDEL	OS06G0338200	Intronic	NA	NA	LOC_Os06g23114	copper methylamine oxidase precursor, putative, expressed
Chr.1	27107248	27107248	G	GAC	A	INDEL	OS01G0663800	Intronic	NA	NA	LOC_Os01g47430	protein of unknown function DUF1296 domain containing protein, expressed
Chr.5	742937	742937	G	GAGAGAA	A	INDEL	OS05G0114000	Exonic	5UTR	NA	LOC_Os05g02310	soluble inorganic pyrophosphatase, putative, expressed
Chr.10	2360663	2360663	G	T	A	SNP	OS10G0138100	Intronic	NA	NA	LOC_Os10g04860	aldehyde oxidase, putative, expressed
Chr.10	2914030	2914030	C	T	A	SNP	OS10G0148400	Exonic	CDS	Missense	LOC_Os10g05780	POT domain containing peptide transporter, putative, expressed
Chr.2	7221585	7221585	G	T	A	SNP	OS02G0228300	Exonic	CDS	Missense	LOC_Os02g13510	receptor-like protein kinase 5 precursor, putative, expressed
Chr.4	31240150	31240150	G	T	A	SNP	OS04G0615700	Exonic	CDS	Missense	LOC_Os04g52540	retrotransposon protein, putative, unclassified, expressed
Chr.3	33215727	33215727	T	TA	A	INDEL	OS03G0797400	Intronic	NA	NA	LOC_Os03g58300	indole-3-glycerol phosphate lyase, chloroplast precursor, putative, expressed
Chr.2	34612222	34612222	T	TA	A	INDEL	OS02G0809800	Intronic	NA	NA	LOC_Os02g56510	phosphate transporter 1, putative, expressed
Chr.10	2361112	2361112	T	TA	A	INDEL	OS10G0138100	Intronic	NA	NA	LOC_Os10g04860	aldehyde oxidase, putative, expressed
Chr.1	35499746	35499747	AT	TT	A	INDEL	OS01G0829800	Intronic	NA	NA	LOC_Os01g61380	lactate/malate dehydrogenase, putative, expressed
Chr.4	5847288	5847288	T	T	A	SNP	OS04G0186400	Exonic	5UTR	NA	LOC_Os04g10750	inorganic phosphate transporter, putative, expressed
Chr.4	5846450	5846450	T	T	A	SNP	OS04G0186400	Exonic	5UTR	NA	LOC_Os04g10750	inorganic phosphate transporter, putative, expressed
Chr.4	5847289	5847289	G	G	A	SNP	OS04G0186400	Exonic	5UTR	NA	LOC_Os04g10750	inorganic phosphate transporter, putative, expressed
Chr.4	5847035	5847035	G	G	A	SNP	OS04G0186400	Exonic	5UTR	NA	LOC_Os04g10750	inorganic phosphate transporter, putative, expressed
Chr.4	5846996	5846996	G	G	A	SNP	OS04G0186400	Exonic	5UTR	NA	LOC_Os04g10750	inorganic phosphate transporter, putative, expressed
Chr.4	5846868	5846868	G	G	A	SNP	OS04G0186400	Exonic	5UTR	NA	LOC_Os04g10750	inorganic phosphate transporter, putative, expressed
Chr.4	5846460	5846460	G	G	A	SNP	OS04G0186400	Exonic	5UTR	NA	LOC_Os04g10750	inorganic phosphate transporter, putative, expressed
Chr.4	5846443	5846443	G	G	A	SNP	OS04G0186400	Exonic	5UTR	NA	LOC_Os04g10750	inorganic phosphate transporter, putative, expressed
Chr.4	5849209	5849209	C	C	A	SNP	OS04G0186400	Exonic	3UTR	NA	LOC_Os04g10750	inorganic phosphate transporter, putative, expressed
Chr.4	5846973	5846973	C	C	A	SNP	OS04G0186400	Exonic	5UTR	NA	LOC_Os04g10750	inorganic phosphate transporter, putative, expressed
Chr.4	5848554	5848554	G	G	A	SNP	OS04G0186400	Exonic	CDS	Silent	LOC_Os04g10750	inorganic phosphate transporter, putative, expressed
Chr.4	5846785	5846788	ATAG	ATAG	A	INDEL	OS04G0186400	Exonic	5UTR	NA	LOC_Os04g10750	inorganic phosphate transporter, putative, expressed
Chr7	1567704	1567704	T	T	A	SNP	OS07G0129700	Intronic	NA	NA	LOC_Os07g03770	Homeobox domain containing protein, expressed
Chr7	4264370	4264370	T	T	A	SNP	OS07G0180900	Intronic	NA	NA	LOC_Os07g08330	ribosomal protein L4, putative, expressed
Chr7	4265068	4265068	T	T	A	SNP	OS07G0180900	Intronic	NA	NA	LOC_Os07g08330	ribosomal protein L4, putative, expressed
Chr7	4484627	4484627	T	T	A	SNP	OS07G0184800	Exonic	3UTR	NA	LOC_Os07g08710	AT hook-containing DNA-binding protein, putative, expressed
Chr7	4683422	4683422	T	T	A	SNP	OS07G0187700	Intronic	NA	NA	LOC_Os07g09000	WD domain, G-beta repeat domain containing protein, expressed
Chr7	4684203	4684203	T	T	A	SNP	OS07G0187700	Intronic	NA	NA	LOC_Os07g09000	WD domain, G-beta repeat domain containing protein, expressed
Chr7	18337073	18337073	T	T	A	SNP	OS07G0492000	Exonic	CDS	Missense	LOC_Os07g30970	nucleoside diphosphate kinase, putative, expressed
Chr7	19656189	19656189	T	T	A	SNP	OS07G0513000	Exonic	3UTR	NA	LOC_Os07g32880	ATP synthase gamma chain, putative, expressed
Chr7	23059034	23059034	T	T	A	SNP	OS07G0571300	Exonic	3UTR	NA	LOC_Os07g38370	expressed protein
Chr7	23679760	23679760	T	T	A	SNP	OS07G0584100	Exonic	3UTR	NA	LOC_Os07g39520	STE_PAK_Ste20_Slob_Wnk.5 - STE kinases include homologs to sterile 7, sterile 11 and sterile 20 from yeast, expressed
Chr7	24701046	24701046	T	T	A	SNP	OS07G0603600	Intronic	NA	NA	LOC_Os07g41230	esterase, putative, expressed
Chr7	27723520	27723520	T	T	A	SNP	OS07G0658400	Exonic	3UTR	NA	LOC_Os07g46460	ferredoxin-dependent glutamate synthase, chloroplast precursor, putative, expressed
Chr7	27725514	27725514	T	T	A	SNP	OS07G0658400	Exonic	CDS	NA	LOC_Os07g46460	ferredoxin-dependent glutamate synthase, chloroplast precursor, putative, expressed
Chr7	27726076	27726076	T	T	A	SNP	OS07G0658400	Exonic	CDS	NA	LOC_Os07g46460	ferredoxin-dependent glutamate synthase, chloroplast precursor, putative, expressed
Chr7	27727058	27727058	T	T	A	SNP	OS07G0658400	Intronic	NA	NA	LOC_Os07g46460	ferredoxin-dependent glutamate synthase, chloroplast precursor, putative, expressed
Chr7	27727132	27727132	T	T	A	SNP	OS07G0658400	Intronic	NA	NA	LOC_Os07g46460	ferredoxin-dependent glutamate synthase, chloroplast precursor, putative, expressed
Chr7	27728737	27728737	T	T	A	SNP	OS07G0658400	Intronic	NA	NA	LOC_Os07g46460	ferredoxin-dependent glutamate synthase, chloroplast precursor, putative, expressed
Chr7	27729162	27729162	T	T	A	SNP	OS07G0658400	Intronic	NA	NA	LOC_Os07g46460	ferredoxin-dependent glutamate synthase, chloroplast precursor, putative, expressed
Chr7	27729623	27729623	T	T	A	SNP	OS07G0658400	Intronic	NA	NA	LOC_Os07g46460	ferredoxin-dependent glutamate synthase, chloroplast precursor, putative, expressed
Chr7	27732135	27732135	T	T	A	SNP	OS07G0658400	Intronic	NA	NA	LOC_Os07g46460	ferredoxin-dependent glutamate synthase, chloroplast precursor, putative, expressed
Chr7	27732259	27732259	T	T	A	SNP	OS07G0658400	Intronic	NA	NA	LOC_Os07g46460	ferredoxin-dependent glutamate synthase, chloroplast precursor, putative, expressed
Chr7	27732535	27732535	T	T	A	SNP	OS07G0658400	Intronic	NA	NA	LOC_Os07g46460	ferredoxin-dependent glutamate synthase, chloroplast precursor, putative, expressed
Chr7	27735470	27735470	T	T	A	SNP	OS07G0658400	Intronic	NA	NA	LOC_Os07g46460	ferredoxin-dependent glutamate synthase, chloroplast precursor, putative, expressed
Chr7	925609	925609	G	G	A	SNP	OS07G0116900	Exonic	3UTR	NA	LOC_Os07g02570	NB-ARC domain containing protein, expressed
Chr7	925937	925937	G	G	A	SNP	OS07G0116900	Exonic	3UTR	NA	LOC_Os07g02570	NB-ARC domain containing protein, expressed
Chr7	1591176	1591176	G	G	A	SNP	OS07G0130100	Exonic	3UTR	NA	LOC_Os07g03810	lectin-like receptor kinase 7, putative, expressed
Chr7	2195307	2195307	G	G	A	SNP	OS07G0142900	Intronic	NA	NA	LOC_Os07g04990	oxidoreductase, aldo/keto reductase family protein, putative, expressed
Chr7	3605398	3605398	G	G	A	SNP	OS07G0167100	Exonic	CDS	NA	LOC_Os07g07260	glutamine-dependent NAD, putative, expressed
Chr7	4264373	4264373	G	G	A	SNP	OS07G0180900	Intronic	NA	NA	LOC_Os07g08330	ribosomal protein L4, putative, expressed
Chr7	4264789	4264789	G	G	A	SNP	OS07G0180900	Intronic	NA	NA	LOC_Os07g08330	ribosomal protein L4, putative, expressed
Chr7	4265151	4265151	G	G	A	SNP	OS07G0180900	Intronic	NA	NA	LOC_Os07g08330	ribosomal protein L4, putative, expressed
Chr7	4265181	4265181	G	G	A	SNP	OS07G0180900	Intronic	NA	NA	LOC_Os07g08330	ribosomal protein L4, putative, expressed
Chr7	4504751	4504751	G	G	A	SNP	OS07G0185200	Intronic	NA	NA	LOC_Os07g08760	START domain containing protein, putative, expressed
Chr7	4506537	4506537	G	G	A	SNP	OS07G0185200	Exonic	5UTR	NA	LOC_Os07g08760	START domain containing protein, putative, expressed

Chr7	4665261	4665261	G	G	A	SNP	OS07G0187400	Exonic	CDS	Missense	LOC_Os07g08970	uncharacterized protein ycf45, putative, expressed
Chr7	4665654	4665654	G	G	A	SNP	OS07G0187400	Intronic	NA	NA	LOC_Os07g08970	uncharacterized protein ycf45, putative, expressed
Chr7	4684059	4684059	G	G	A	SNP	OS07G0187700	Intronic	NA	NA	LOC_Os07g09000	WD domain, G-beta repeat domain containing protein, expressed
Chr7	7108856	7108856	G	G	A	SNP	OS07G0227200	Exonic	3UTR	NA	LOC_Os07g12470	expressed protein
Chr7	7109175	7109175	G	G	A	SNP	OS07G0227200	Exonic	3UTR	NA	LOC_Os07g12470	expressed protein
Chr7	12576919	12576919	G	G	A	SNP	OS07G0406800	Intronic	NA	NA	LOC_Os07g22400	POLA3 - Putative DNA polymerase alpha complex subunit, expressed
Chr7	13100730	13100730	G	G	A	SNP	OS07G0415200	Intronic	NA	NA	LOC_Os07g23244	ribosomal protein L23 family protein, putative, expressed
Chr7	13354537	13354537	G	G	A	SNP	OS07G0418600	Exonic	CDS	NA	LOC_Os07g23640	retrotransposon protein, putative, Ty3-gypsy subclass, expressed
Chr7	13354590	13354590	G	G	A	SNP	OS07G0418600	Exonic	CDS	NA	LOC_Os07g23640	retrotransposon protein, putative, Ty3-gypsy subclass, expressed
Chr7	13354844	13354844	G	G	A	SNP	OS07G0418600	Exonic	CDS	NA	LOC_Os07g23640	retrotransposon protein, putative, Ty3-gypsy subclass, expressed
Chr7	18335915	18335915	G	G	A	SNP	OS07G0492000	Exonic	CDS	Silent	LOC_Os07g30970	nucleoside diphosphate kinase, putative, expressed
Chr7	18763189	18763189	G	G	A	SNP	OS07G0499300	Intronic	NA	NA	LOC_Os07g31599	expressed protein
Chr7	21245347	21245347	G	G	A	SNP	OS07G0539400	Intronic	NA	NA	LOC_Os07g35520	glucan endo-1,3-beta-glucosidase precursor, putative, expressed
Chr7	21246318	21246318	G	G	A	SNP	OS07G0539400	Exonic	CDS	Silent	LOC_Os07g35520	glucan endo-1,3-beta-glucosidase precursor, putative, expressed
Chr7	21246886	21246886	G	G	A	SNP	OS07G0539400	Exonic	CDS	Missense	LOC_Os07g35520	glucan endo-1,3-beta-glucosidase precursor, putative, expressed
Chr7	21280927	21280927	G	G	A	SNP	OS07G0539900	Exonic	CDS	Silent	LOC_Os07g35560	glucan endo-1,3-beta-glucosidase precursor, putative, expressed
Chr7	21280977	21280977	G	G	A	SNP	OS07G0539900	Intronic	NA	NA	LOC_Os07g35560	glucan endo-1,3-beta-glucosidase precursor, putative, expressed
Chr7	21280997	21280997	G	G	A	SNP	OS07G0539900	Intronic	NA	NA	LOC_Os07g35560	glucan endo-1,3-beta-glucosidase precursor, putative, expressed
Chr7	21415398	21415398	G	G	A	SNP	OS07G0541900	Exonic	CDS	Silent	LOC_Os07g35750	TKL_IRAK_DUF26-ld.3 - DUF26 kinases have homology to DUF26 containing loci, expressed
Chr7	21415436	21415436	G	G	A	SNP	OS07G0541900	Exonic	CDS	Missense	LOC_Os07g35750	TKL_IRAK_DUF26-ld.3 - DUF26 kinases have homology to DUF26 containing loci, expressed
Chr7	21415564	21415564	G	G	A	SNP	OS07G0541900	Exonic	CDS	Silent	LOC_Os07g35750	TKL_IRAK_DUF26-ld.3 - DUF26 kinases have homology to DUF26 containing loci, expressed
Chr7	21415597	21415597	G	G	A	SNP	OS07G0541900	Exonic	CDS	Silent	LOC_Os07g35750	TKL_IRAK_DUF26-ld.3 - DUF26 kinases have homology to DUF26 containing loci, expressed
Chr7	21415734	21415734	G	G	A	SNP	OS07G0541900	Exonic	CDS	Silent	LOC_Os07g35750	TKL_IRAK_DUF26-ld.3 - DUF26 kinases have homology to DUF26 containing loci, expressed
Chr7	21590257	21590257	G	G	A	SNP	OS07G0545300	Intronic	NA	NA	LOC_Os07g36130	core histone H2A/H2B/H3/H4, putative, expressed
Chr7	22428514	22428514	G	G	A	SNP	OS07G0561800	Exonic	CDS	NA	LOC_Os07g37454	urate anion exchanger, putative, expressed
Chr7	22429315	22429315	G	G	A	SNP	OS07G0561800	Exonic	3UTR	NA	LOC_Os07g37454	urate anion exchanger, putative, expressed
Chr7	23679795	23679795	G	G	A	SNP	OS07G0584100	Exonic	3UTR	NA	LOC_Os07g39520	STE_PAK_Ste20_Slob_Wnk.5 - STE kinases include homologs to sterile 7, sterile 11 and sterile 20 from yeast, expressed
Chr7	24397731	24397731	G	G	A	SNP	OS07G0598200	Intronic	NA	NA	LOC_Os07g40710	circadian clock coupling factor-related, putative, expressed
Chr7	24657417	24657417	G	G	A	SNP	OS07G0603100	Exonic	CDS	Missense	LOC_Os07g41180	RNA-binding protein-like, putative, expressed
Chr7	24701496	24701496	G	G	A	SNP	OS07G0603600	Intronic	NA	NA	LOC_Os07g41230	esterase, putative, expressed
Chr7	26407686	26407686	G	G	A	SNP	OS07G0636000	Exonic	CDS	Silent	LOC_Os07g44190	h/ACA ribonucleoprotein complex subunit 4, putative, expressed
Chr7	26482539	26482539	G	G	A	SNP	OS07G0637300	Intronic	NA	NA	LOC_Os07g44330	kinase, putative, expressed
Chr7	26631808	26631808	G	G	A	SNP	OS07G0639900	Intronic	NA	NA	LOC_Os07g44630	thymidylate kinase, putative, expressed
Chr7	26773259	26773259	G	G	A	SNP	OS07G0642800	Exonic	CDS	Missense	LOC_Os07g44830	proline-rich family protein, putative, expressed
Chr7	27723987	27723987	G	G	A	SNP	OS07G0658400	Intronic	NA	NA	LOC_Os07g46460	ferredoxin-dependent glutamate synthase, chloroplast precursor, putative, expressed
Chr7	27724064	27724064	G	G	A	SNP	OS07G0658400	Intronic	NA	NA	LOC_Os07g46460	ferredoxin-dependent glutamate synthase, chloroplast precursor, putative, expressed
Chr7	27724457	27724457	G	G	A	SNP	OS07G0658400	Exonic	CDS	NA	LOC_Os07g46460	ferredoxin-dependent glutamate synthase, chloroplast precursor, putative, expressed
Chr7	27724718	27724718	G	G	A	SNP	OS07G0658400	Intronic	NA	NA	LOC_Os07g46460	ferredoxin-dependent glutamate synthase, chloroplast precursor, putative, expressed
Chr7	27724803	27724803	G	G	A	SNP	OS07G0658400	Exonic	CDS	NA	LOC_Os07g46460	ferredoxin-dependent glutamate synthase, chloroplast precursor, putative, expressed
Chr7	27725486	27725486	G	G	A	SNP	OS07G0658400	Exonic	CDS	NA	LOC_Os07g46460	ferredoxin-dependent glutamate synthase, chloroplast precursor, putative, expressed
Chr7	27725789	27725789	G	G	A	SNP	OS07G0658400	Intronic	NA	NA	LOC_Os07g46460	ferredoxin-dependent glutamate synthase, chloroplast precursor, putative, expressed
Chr7	27727787	27727787	G	G	A	SNP	OS07G0658400	Intronic	NA	NA	LOC_Os07g46460	ferredoxin-dependent glutamate synthase, chloroplast precursor, putative, expressed
Chr7	27727970	27727970	G	G	A	SNP	OS07G0658400	Intronic	NA	NA	LOC_Os07g46460	ferredoxin-dependent glutamate synthase, chloroplast precursor, putative, expressed
Chr7	27728896	27728896	G	G	A	SNP	OS07G0658400	Intronic	NA	NA	LOC_Os07g46460	ferredoxin-dependent glutamate synthase, chloroplast precursor, putative, expressed
Chr7	27729708	27729708	G	G	A	SNP	OS07G0658400	Intronic	NA	NA	LOC_Os07g46460	ferredoxin-dependent glutamate synthase, chloroplast precursor, putative, expressed
Chr7	27729727	27729727	G	G	A	SNP	OS07G0658400	Intronic	NA	NA	LOC_Os07g46460	ferredoxin-dependent glutamate synthase, chloroplast precursor, putative, expressed
Chr7	27729763	27729763	G	G	A	SNP	OS07G0658400	Intronic	NA	NA	LOC_Os07g46460	ferredoxin-dependent glutamate synthase, chloroplast precursor, putative, expressed
Chr7	27729889	27729889	G	G	A	SNP	OS07G0658400	Exonic	CDS	NA	LOC_Os07g46460	ferredoxin-dependent glutamate synthase, chloroplast precursor, putative, expressed
Chr7	27730208	27730208	G	G	A	SNP	OS07G0658400	Intronic	NA	NA	LOC_Os07g46460	ferredoxin-dependent glutamate synthase, chloroplast precursor, putative, expressed
Chr7	27730508	27730508	G	G	A	SNP	OS07G0658400	Intronic	NA	NA	LOC_Os07g46460	ferredoxin-dependent glutamate synthase, chloroplast precursor, putative, expressed
Chr7	27730859	27730859	G	G	A	SNP	OS07G0658400	Intronic	NA	NA	LOC_Os07g46460	ferredoxin-dependent glutamate synthase, chloroplast precursor, putative, expressed
Chr7	27731705	27731705	G	G	A	SNP	OS07G0658400	Intronic	NA	NA	LOC_Os07g46460	ferredoxin-dependent glutamate synthase, chloroplast precursor, putative, expressed
Chr7	27732229	27732229	G	G	A	SNP	OS07G0658400	Intronic	NA	NA	LOC_Os07g46460	ferredoxin-dependent glutamate synthase, chloroplast precursor, putative, expressed
Chr7	27732427	27732427	G	G	A	SNP	OS07G0658400	Intronic	NA	NA	LOC_Os07g46460	ferredoxin-dependent glutamate synthase, chloroplast precursor, putative, expressed
Chr7	27733159	27733159	G	G	A	SNP	OS07G0658400	Intronic	NA	NA	LOC_Os07g46460	ferredoxin-dependent glutamate synthase, chloroplast precursor, putative, expressed
Chr7	27733521	27733521	G	G	A	SNP	OS07G0658400	Intronic	NA	NA	LOC_Os07g46460	ferredoxin-dependent glutamate synthase, chloroplast precursor, putative, expressed
Chr7	27733571	27733571	G	G	A	SNP	OS07G0658400	Intronic	NA	NA	LOC_Os07g46460	ferredoxin-dependent glutamate synthase, chloroplast precursor, putative, expressed
Chr7	27733928	27733928	G	G	A	SNP	OS07G0658400	Intronic	NA	NA	LOC_Os07g46460	ferredoxin-dependent glutamate synthase, chloroplast precursor, putative, expressed
Chr7	27736511	27736511	G	G	A	SNP	OS07G0658400	Intronic	NA	NA	LOC_Os07g46460	ferredoxin-dependent glutamate synthase, chloroplast precursor, putative, expressed
Chr7	27736874	27736874	G	G	A	SNP	OS07G0658400	Exonic	CDS	NA	LOC_Os07g46460	ferredoxin-dependent glutamate synthase, chloroplast precursor, putative, expressed
Chr7	28782084	28782084	G	G	A	SNP	OS07G0679500	Intronic	NA	NA	LOC_Os07g48180	bZIP transcription factor domain containing protein, expressed
Chr7	29635325	29635325	G	G	A	SNP	OS07G0695400	Exonic	CDS	Missense	LOC_Os07g49480	KIP1, putative, expressed
Chr7	19883	19883	C	C	A	SNP	OS07G0100300	Exonic	CDS	Silent	LOC_Os07g01030	glycosyl transferase, group 1 domain containing protein, expressed
Chr7	559381	559381	C	C	A	SNP	OS07G0110200	Exonic	3UTR	NA	LOC_Os07g01920	nucleolar GTP-binding protein 1, putative, expressed
Chr7	926112	926112	C	C	A	SNP	OS07G0116900	Exonic	CDS	Missense	LOC_Os07g02570	NB-ARC domain containing protein, expressed
Chr7	926356	926356	C	C	A	SNP	OS07G0116900	Exonic	CDS	Missense	LOC_Os07g02570	NB-ARC domain containing protein, expressed
Chr7	3607760	3607760	C	C	A	SNP	OS07G0167100	Intronic	NA	NA	LOC_Os07g07260	glutamine-dependent NAD, putative, expressed
Chr7	4331253	4331253	C	C	A	SNP	OS07G0182100	Intronic	NA	NA	LOC_Os07g08430	indole-3-glycerol phosphate lyase, chloroplast precursor, putative, expressed
Chr7	4506650	4506650	C	C	A	SNP	OS07G0185200	Exonic	5UTR	NA	LOC_Os07g08760	START domain containing protein, putative, expressed
Chr7	12575300	12575300	C	C	A	SNP	OS07G0406800	Intronic	NA	NA	LOC_Os07g22400	POLA3 - Putative DNA polymerase alpha complex subunit, expressed
Chr7	13102911	13102911	C	C	A	SNP	OS07G0415200	Intronic	NA	NA	LOC_Os07g23244	ribosomal protein L23 family protein, putative, expressed
Chr7	16503744	16503744	C	C	A	SNP	OS07G0466000	Exonic	CDS	Missense	LOC_Os07g28250	lipase class 3 family protein, putative, expressed
Chr7	16504360	16504360	C	C	A	SNP	OS07G0466000	Exonic	CDS	Silent	LOC_Os07g28250	lipase class 3 family protein, putative, expressed
Chr7	16504465	16504465	C	C	A	SNP	OS07G0466000	Exonic	CDS	Missense	LOC_Os07g28250	lipase class 3 family protein, putative, expressed
Chr7	18337216	18337216	C	C	A	SNP	OS07G0492000	Intronic	NA	NA	LOC_Os07g30970	nucleoside diphosphate kinase, putative, expressed
Chr7	20725949	20725949	C	C	A	SNP	OS07G0529600	Exonic	3UTR	NA	LOC_Os07g34570	FAD dependent oxidoreductase domain containing protein, expressed
Chr7	21281894	21281894	C	C	A	SNP	OS07G0539900	Exonic	CDS	Missense	LOC_Os07g35560	glucan endo-1,3-beta-glucosidase precursor, putative, expressed
Chr7	22412420	22412420	C	C	A	SNP	OS07G0561300	Intronic	NA	NA	LOC_Os07g37400	OsFBX257 - F-box domain containing protein, expressed
Chr7	26384241	26384241	C	C	A	SNP	OS07G0635500	Exonic	CDS	Missense	LOC_Os07g44140	cytochrome P450 72A1, putative, expressed
Chr7	26482628	26482628	C	C	A	SNP	OS07G0637300	Intronic	NA	NA	LOC_Os07g44330	kinase, putative, expressed
Chr7	26772339	26772339	C	C	A	SNP	OS07G0642800	Intronic	NA	NA	LOC_Os07g44830	proline-rich family protein, putative, expressed
Chr7	27724038	27724038	C	C	A	SNP	OS07G0658400	Intronic	NA	NA	LOC_Os07g46460	ferredoxin-dependent glutamate synthase, chloroplast precursor, putative, expressed
Chr7	27728522	27728522	C	C	A	SNP	OS07G0658400	Intronic	NA	NA	LOC_Os07g46460	ferredoxin-dependent glutamate synthase, chloroplast precursor, putative, expressed
Chr7	27728773	27728773	C	C	A	SNP	OS07G0658400	Intronic	NA	NA	LOC_Os07g46460	ferredoxin-dependent glutamate synthase, chloroplast precursor, putative, expressed
Chr7	27729268	27729268	C	C	A	SNP	OS07G0658400	Intronic	NA	NA	LOC_Os07g46460	ferredoxin-dependent glutamate synthase, chloroplast precursor, putative, expressed

Chr7	27729650	27729650	C	C	A	SNP	OS07G0658400	Intronic	NA	NA	LOC_Os07g46460	ferredoxin-dependent glutamate synthase, chloroplast precursor, putative, expressed
Chr7	27730232	27730232	C	C	A	SNP	OS07G0658400	Intronic	NA	NA	LOC_Os07g46460	ferredoxin-dependent glutamate synthase, chloroplast precursor, putative, expressed
Chr7	27730607	27730607	C	C	A	SNP	OS07G0658400	Intronic	NA	NA	LOC_Os07g46460	ferredoxin-dependent glutamate synthase, chloroplast precursor, putative, expressed
Chr7	27732495	27732495	C	C	A	SNP	OS07G0658400	Intronic	NA	NA	LOC_Os07g46460	ferredoxin-dependent glutamate synthase, chloroplast precursor, putative, expressed
Chr7	27737006	27737006	C	C	A	SNP	OS07G0658400	Exonic	CDS	NA	LOC_Os07g46460	ferredoxin-dependent glutamate synthase, chloroplast precursor, putative, expressed
Chr7	27737488	27737488	C	C	A	SNP	OS07G0658400	Intronic	NA	NA	LOC_Os07g46460	ferredoxin-dependent glutamate synthase, chloroplast precursor, putative, expressed
Chr7	7108104	7108104	T	T	A	SNP	OS07G0227200	Exonic	3UTR	NA	LOC_Os07g12470	expressed protein
Chr7	8970518	8970518	T	T	A	SNP	OS07G0258400	Exonic	3UTR	NA	LOC_Os07g15460	metal transporter Nramp6, putative, expressed
Chr7	10232334	10232334	T	T	A	SNP	OS07G0274700	Intronic	NA	NA	LOC_Os07g17330	B12D protein, putative, expressed
Chr7	15409260	15409260	T	T	A	SNP	OS07G0448800	Intronic	NA	NA	LOC_Os07g26690	aquaporin protein, putative, expressed
Chr7	17413081	17413081	T	T	A	SNP	OS07G0479300	Intronic	NA	NA	LOC_Os07g29620	OsSCP39 - Putative Serine Carboxypeptidase homologue, expressed
Chr7	29172078	29172078	T	T	A	SNP	OS07G0686600	Exonic	3UTR	NA	LOC_Os07g48710	VQ domain containing protein, putative, expressed
Chr7	8967253	8967253	G	G	A	SNP	OS07G0258400	Exonic	CDS	Missense	LOC_Os07g15460	metal transporter Nramp6, putative, expressed
Chr7	15602468	15602468	G	G	A	SNP	OS07G0452500	Exonic	CDS	Silent	LOC_Os07g26940	ORM1, putative, expressed
Chr7	17410763	17410763	G	G	A	SNP	OS07G0479300	Exonic	CDS	Silent	LOC_Os07g29620	OsSCP39 - Putative Serine Carboxypeptidase homologue, expressed
Chr7	17411013	17411013	G	G	A	SNP	OS07G0479300	Intronic	NA	NA	LOC_Os07g29620	OsSCP39 - Putative Serine Carboxypeptidase homologue, expressed
Chr7	17411781	17411781	G	G	A	SNP	OS07G0479300	Exonic	CDS	Missense	LOC_Os07g29620	OsSCP39 - Putative Serine Carboxypeptidase homologue, expressed
Chr7	18841778	18841778	G	G	A	SNP	OS07G0500300	Intronic	NA	NA	LOC_Os07g31720	GTPase activating protein, putative, expressed
Chr7	21380128	21380128	G	G	A	SNP	OS07G0541400	Exonic	CDS	Missense	LOC_Os07g35690	TKL_IRAK_DUF26-lc.3 - DUF26 kinases have homology to DUF26 containing loci, expressed
Chr7	23651286	23651286	G	G	A	SNP	OS07G0583600	Intronic	NA	NA	LOC_Os07g39470	gibberellin response modulator protein, putative, expressed
Chr7	27172651	27172651	G	G	A	SNP	OS07G0650100	Exonic	CDS	Silent	LOC_Os07g45550	expressed protein
Chr7	27172735	27172735	G	G	A	SNP	OS07G0650100	Exonic	CDS	Silent	LOC_Os07g45550	expressed protein
Chr7	27172879	27172879	G	G	A	SNP	OS07G0650100	Exonic	CDS	Silent	LOC_Os07g45550	expressed protein
Chr7	27173499	27173499	G	G	A	SNP	OS07G0650100	Exonic	CDS	Missense	LOC_Os07g45550	expressed protein
Chr7	27174473	27174473	G	G	A	SNP	OS07G0650100	Exonic	CDS	Missense	LOC_Os07g45550	expressed protein
Chr7	27175491	27175491	G	G	A	SNP	OS07G0650100	Exonic	CDS	Missense	LOC_Os07g45550	expressed protein
Chr7	27175502	27175502	G	G	A	SNP	OS07G0650100	Exonic	CDS	Silent	LOC_Os07g45550	expressed protein
Chr7	9037924	9037924	C	C	A	SNP	OS07G0259700	Intronic	NA	NA	LOC_Os07g15600	H-BTB6 - Bric-a-Brac, Tramtrack, Broad Complex BTB domain with H family conserved sequence, expressed
Chr7	17411334	17411334	C	C	A	SNP	OS07G0479300	Intronic	NA	NA	LOC_Os07g29620	OsSCP39 - Putative Serine Carboxypeptidase homologue, expressed
Chr7	15407879	15407880	AT	AT	A	INDEL	OS07G0448800	Intronic	NA	NA	LOC_Os07g26690	aquaporin protein, putative, expressed
Chr7	17541	17541	T	T	A	SNP	OS07G0100300	Exonic	5UTR	NA	LOC_Os07g01030	glycosyl transferase, group 1 domain containing protein, expressed
Chr7	925375	925375	C	C	A	SNP	OS07G0116900	Exonic	3UTR	NA	LOC_Os07g02570	NB-ARC domain containing protein, expressed
Chr7	926217	926217	G	G	A	SNP	OS07G0116900	Intronic	NA	NA	LOC_Os07g02570	NB-ARC domain containing protein, expressed
Chr7	926724	926724	G	G	A	SNP	OS07G0116900	Exonic	CDS	Silent	LOC_Os07g02570	NB-ARC domain containing protein, expressed
Chr7	1512701	1512701	T	T	A	SNP	OS07G0129200	Exonic	3UTR	NA	LOC_Os07g03710	SCP-like extracellular protein, expressed
Chr7	1566731	1566731	G	G	A	SNP	OS07G0129700	Exonic	CDS	Silent	LOC_Os07g03770	Homeobox domain containing protein, expressed
Chr7	1567088	1567088	G	G	A	SNP	OS07G0129700	Exonic	CDS	Missense	LOC_Os07g03770	Homeobox domain containing protein, expressed
Chr7	1567227	1567227	C	C	A	SNP	OS07G0129700	Intronic	NA	NA	LOC_Os07g03770	Homeobox domain containing protein, expressed
Chr7	1567908	1567908	T	T	A	SNP	OS07G0129700	Intronic	NA	NA	LOC_Os07g03770	Homeobox domain containing protein, expressed
Chr7	1592539	1592539	G	G	A	SNP	OS07G0130100	Exonic	CDS	Silent	LOC_Os07g03810	lectin-like receptor kinase 7, putative, expressed
Chr7	3663140	3663140	C	C	A	SNP	OS07G0168300	Exonic	3UTR	NA	LOC_Os07g07320	glutathione S-transferase, putative, expressed
Chr7	3663369	3663369	G	G	A	SNP	OS07G0168300	Exonic	3UTR	NA	LOC_Os07g07320	glutathione S-transferase, putative, expressed
Chr7	4263205	4263205	C	C	A	SNP	OS07G0180900	Exonic	5UTR	NA	LOC_Os07g08330	ribosomal protein L4, putative, expressed
Chr7	4265481	4265481	G	G	A	SNP	OS07G0180900	Exonic	3UTR	NA	LOC_Os07g08330	ribosomal protein L4, putative, expressed
Chr7	4484499	4484499	G	G	A	SNP	OS07G0184800	Exonic	CDS	Silent	LOC_Os07g08710	AT hook-containing DNA-binding protein, putative, expressed
Chr7	4665149	4665149	G	G	A	SNP	OS07G0187400	Exonic	CDS	Silent	LOC_Os07g08970	uncharacterized protein ycf45, putative, expressed
Chr7	4686195	4686195	G	G	A	SNP	OS07G0187700	Intronic	NA	NA	LOC_Os07g09000	WD domain, G-beta repeat domain containing protein, expressed
Chr7	7107135	7107135	G	G	A	SNP	OS07G0227200	Exonic	5UTR	NA	LOC_Os07g12470	expressed protein
Chr7	7108693	7108693	C	C	A	SNP	OS07G0227200	Exonic	3UTR	NA	LOC_Os07g12470	expressed protein
Chr7	8970719	8970719	T	T	A	SNP	OS07G0258400	Exonic	3UTR	NA	LOC_Os07g15460	metal transporter Nramp6, putative, expressed
Chr7	8970824	8970824	T	T	A	SNP	OS07G0258400	Exonic	3UTR	NA	LOC_Os07g15460	metal transporter Nramp6, putative, expressed
Chr7	9038691	9038691	G	G	A	SNP	OS07G0259700	Exonic	CDS	Missense	LOC_Os07g15600	H-BTB6 - Bric-a-Brac, Tramtrack, Broad Complex BTB domain with H family conserved sequence, expressed
Chr7	9039191	9039191	G	G	A	SNP	OS07G0259700	Exonic	5UTR	NA	LOC_Os07g15600	H-BTB6 - Bric-a-Brac, Tramtrack, Broad Complex BTB domain with H family conserved sequence, expressed
Chr7	11090629	11090629	C	C	A	SNP	OS07G0287400	Exonic	5UTR	NA	LOC_Os07g18750	LTPL42 - Protease inhibitor/seed storage/LTP family protein precursor, expressed
Chr7	13366791	13366791	G	G	A	SNP	OS07G0418700	Exonic	CDS	NA	LOC_Os07g23660	retrotransposon protein, putative, unclassified, expressed
Chr7	16503367	16503367	G	G	A	SNP	OS07G0466000	Intronic	NA	NA	LOC_Os07g28250	lipase class 3 family protein, putative, expressed
Chr7	16503451	16503451	G	G	A	SNP	OS07G0466000	Intronic	NA	NA	LOC_Os07g28250	lipase class 3 family protein, putative, expressed
Chr7	16503487	16503487	G	G	A	SNP	OS07G0466000	Intronic	NA	NA	LOC_Os07g28250	lipase class 3 family protein, putative, expressed
Chr7	17413657	17413657	G	G	A	SNP	OS07G0479300	Exonic	CDS	Silent	LOC_Os07g29620	OsSCP39 - Putative Serine Carboxypeptidase homologue, expressed
Chr7	18761455	18761455	C	C	A	SNP	OS07G0499300	Exonic	CDS	NA	LOC_Os07g31599	expressed protein
Chr7	18840631	18840631	G	G	A	SNP	OS07G0500300	Intronic	NA	NA	LOC_Os07g31720	GTPase activating protein, putative, expressed
Chr7	18841144	18841144	C	C	A	SNP	OS07G0500300	Intronic	NA	NA	LOC_Os07g31720	GTPase activating protein, putative, expressed
Chr7	18841509	18841509	G	G	A	SNP	OS07G0500300	Intronic	NA	NA	LOC_Os07g31720	GTPase activating protein, putative, expressed
Chr7	19473623	19473623	C	C	A	SNP	OS07G0510800	Exonic	CDS	Silent	LOC_Os07g32650	retrotransposon protein, putative, unclassified, expressed
Chr7	19656254	19656254	G	G	A	SNP	OS07G0513000	Exonic	3UTR	NA	LOC_Os07g32880	ATP synthase gamma chain, putative, expressed
Chr7	20085389	20085389	G	G	A	SNP	OS07G0520300	Exonic	CDS	Silent	LOC_Os07g33620	cytochrome P450 domain containing protein, expressed
Chr7	20724747	20724747	C	C	A	SNP	OS07G0529600	Exonic	CDS	Silent	LOC_Os07g34570	FAD dependent oxidoreductase domain containing protein, expressed
Chr7	21146963	21146963	G	G	A	SNP	OS07G0537900	Exonic	CDS	Missense	LOC_Os07g35340	TKL_IRAK_DUF26-lc.14 - DUF26 kinases have homology to DUF26 containing loci, expressed
Chr7	21280771	21280771	G	G	A	SNP	OS07G0539900	Exonic	CDS	Silent	LOC_Os07g35560	glucan endo-1,3-beta-glucosidase precursor, putative, expressed
Chr7	21280788	21280788	T	T	A	SNP	OS07G0539900	Exonic	CDS	Missense	LOC_Os07g35560	glucan endo-1,3-beta-glucosidase precursor, putative, expressed
Chr7	21281153	21281153	C	C	A	SNP	OS07G0539900	Exonic	CDS	Missense	LOC_Os07g35560	glucan endo-1,3-beta-glucosidase precursor, putative, expressed
Chr7	21281253	21281253	G	G	A	SNP	OS07G0539900	Exonic	CDS	Silent	LOC_Os07g35560	glucan endo-1,3-beta-glucosidase precursor, putative, expressed
Chr7	21378989	21378989	T	T	A	SNP	OS07G0541400	Exonic	CDS	Missense	LOC_Os07g35690	TKL_IRAK_DUF26-lc.3 - DUF26 kinases have homology to DUF26 containing loci, expressed
Chr7	21379041	21379041	G	G	A	SNP	OS07G0541400	Intronic	NA	NA	LOC_Os07g35690	TKL_IRAK_DUF26-lc.3 - DUF26 kinases have homology to DUF26 containing loci, expressed
Chr7	21419028	21419028	G	G	A	SNP	OS07G0541900	Exonic	CDS	Missense	LOC_Os07g35750	TKL_IRAK_DUF26-ld.3 - DUF26 kinases have homology to DUF26 containing loci, expressed
Chr7	22372417	22372417	C	C	A	SNP	OS07G0560300	Exonic	5UTR	NA	LOC_Os07g37350	armadillo/beta-catenin repeat family protein, putative, expressed
Chr7	22428857	22428857	G	G	A	SNP	OS07G0561800	Exonic	3UTR	NA	LOC_Os07g37454	urate anion exchanger, putative, expressed
Chr7	22428901	22428901	G	G	A	SNP	OS07G0561800	Exonic	3UTR	NA	LOC_Os07g37454	urate anion exchanger, putative, expressed
Chr7	22696369	22696369	G	G	A	SNP	OS07G0565800	Exonic	CDS	Silent	LOC_Os07g37850	expressed protein
Chr7	22696579	22696579	C	C	A	SNP	OS07G0565800	Exonic	CDS	Silent	LOC_Os07g37850	expressed protein
Chr7	22875693	22875693	T	T	A	SNP	OS07G0568700	Exonic	5UTR	NA	LOC_Os07g38130	polygalacturonase inhibitor 1 precursor, putative, expressed
Chr7	24398736	24398736	G	G	A	SNP	OS07G0598200	Intronic	NA	NA	LOC_Os07g40710	circadian clock coupling factor-related, putative, expressed
Chr7	24399166	24399166	G	G	A	SNP	OS07G0598200	Intronic	NA	NA	LOC_Os07g40710	circadian clock coupling factor-related, putative, expressed
Chr7	24658766	24658766	T	T	A	SNP	OS07G0603100	Intronic	NA	NA	LOC_Os07g41180	RNA-binding protein-like, putative, expressed
Chr7	24702938	24702938	G	G	A	SNP	OS07G0603600	Exonic	CDS	NA	LOC_Os07g41230	esterase, putative, expressed

Chr7	25037095	25037095	T	T	A	SNP	OS07G0608700	Intronic	NA	NA	LOC_Os07g41790	LSM domain containing protein, expressed
Chr7	26484502	26484502	T	T	A	SNP	OS07G0637300	Intronic	NA	NA	LOC_Os07g44330	kinase, putative, expressed
Chr7	26543152	26543152	G	G	A	SNP	OS07G0638100	Exonic	CDS	Missense	LOC_Os07g44410	WD40-like Beta Propeller Repeat family protein, expressed
Chr7	26604072	26604072	G	G	A	SNP	OS07G0639000	Exonic	CDS	NA	LOC_Os07g44550	peroxidase precursor, putative, expressed
Chr7	26616059	26616059	G	G	A	SNP	OS07G0639400	Exonic	CDS	Silent	LOC_Os07g44590	peroxidase precursor, putative, expressed
Chr7	26768522	26768522	C	C	A	SNP	OS07G0642800	Exonic	5UTR	NA	LOC_Os07g44830	proline-rich family protein, putative, expressed
Chr7	27172223	27172223	G	G	A	SNP	OS07G0650100	Exonic	CDS	Missense	LOC_Os07g45550	expressed protein
Chr7	27173977	27173977	G	G	A	SNP	OS07G0650100	Exonic	CDS	Silent	LOC_Os07g45550	expressed protein
Chr7	27174235	27174235	G	G	A	SNP	OS07G0650100	Exonic	CDS	Silent	LOC_Os07g45550	expressed protein
Chr7	27174238	27174238	G	G	A	SNP	OS07G0650100	Exonic	CDS	Silent	LOC_Os07g45550	expressed protein
Chr7	27552356	27552356	G	G	A	SNP	OS07G0655400	Intronic	NA	NA	LOC_Os07g46170	OsRhmbd13 - Putative Rhomboid homologue, expressed
Chr7	27725989	27725989	G	G	A	SNP	OS07G0658400	Exonic	CDS	NA	LOC_Os07g46460	ferredoxin-dependent glutamate synthase, chloroplast precursor, putative, expressed
Chr7	27726523	27726523	G	G	A	SNP	OS07G0658400	Intronic	NA	NA	LOC_Os07g46460	ferredoxin-dependent glutamate synthase, chloroplast precursor, putative, expressed
Chr7	27726534	27726534	G	G	A	SNP	OS07G0658400	Intronic	NA	NA	LOC_Os07g46460	ferredoxin-dependent glutamate synthase, chloroplast precursor, putative, expressed
Chr7	27727566	27727566	G	G	A	SNP	OS07G0658400	Intronic	NA	NA	LOC_Os07g46460	ferredoxin-dependent glutamate synthase, chloroplast precursor, putative, expressed
Chr7	27732202	27732202	G	G	A	SNP	OS07G0658400	Intronic	NA	NA	LOC_Os07g46460	ferredoxin-dependent glutamate synthase, chloroplast precursor, putative, expressed
Chr7	27734011	27734011	G	G	A	SNP	OS07G0658400	Intronic	NA	NA	LOC_Os07g46460	ferredoxin-dependent glutamate synthase, chloroplast precursor, putative, expressed
Chr7	28120858	28120858	G	G	A	SNP	OS07G0665900	Intronic	NA	NA	LOC_Os07g47040	expressed protein
Chr7	28859402	28859402	G	G	A	SNP	OS07G0680900	Exonic	3UTR	NA	LOC_Os07g48290	AGC_AGC_other_RS6K_like.2 - ACG kinases include homologs to PKA, PKG and PKC, expressed
Chr7	1569183	1569184	AT	AT	A	INDEL	OS07G0129700	Intronic	NA	NA	LOC_Os07g03770	Homeobox domain containing protein, expressed
Chr7	4483701	4483704	AAGG	AAGG	A	INDEL	OS07G0184800	Exonic	CDS	InFrame	LOC_Os07g08710	AT hook-containing DNA-binding protein, putative, expressed
Chr7	18840549	18840551	ACG	ACG	A	INDEL	OS07G0500300	Intronic	NA	NA	LOC_Os07g31720	GTPase activating protein, putative, expressed
Chr7	18841734	18841743	ACTTGAGTTG	ACTTGAGTTG	A	INDEL	OS07G0500300	Intronic	NA	NA	LOC_Os07g31720	GTPase activating protein, putative, expressed
Chr7	19474024	19474031	AGTTTTGT	AGTTTTGT	A	INDEL	OS07G0510800	Exonic	5UTR	NA	LOC_Os07g32650	retrotransposon protein, putative, unclassified, expressed
Chr7	26883303	26883304	AT	AT	A	INDEL	OS07G0645000	Intronic	NA	NA	LOC_Os07g45060	uncharacterized GPI-anchored protein At5g19240 precursor, putative, expressed
Chr7	27552499	27552500	AT	AT	A	INDEL	OS07G0655400	Intronic	NA	NA	LOC_Os07g46170	OsRhmbd13 - Putative Rhomboid homologue, expressed
Chr7	27723304	27723305	AG	AG	A	INDEL	OS07G0658400	Exonic	3UTR	NA	LOC_Os07g46460	ferredoxin-dependent glutamate synthase, chloroplast precursor, putative, expressed
Chr7	27726987	27726988	AT	AT	A	INDEL	OS07G0658400	Intronic	NA	NA	LOC_Os07g46460	ferredoxin-dependent glutamate synthase, chloroplast precursor, putative, expressed
Chr7	27729258	27729263	AAAAAT	AAAAAT	A	INDEL	OS07G0658400	Intronic	NA	NA	LOC_Os07g46460	ferredoxin-dependent glutamate synthase, chloroplast precursor, putative, expressed
Chr7	27732740	27732742	AAT	AAT	A	INDEL	OS07G0658400	Intronic	NA	NA	LOC_Os07g46460	ferredoxin-dependent glutamate synthase, chloroplast precursor, putative, expressed
Chr7	27733898	27733900	ACT	ACT	A	INDEL	OS07G0658400	Intronic	NA	NA	LOC_Os07g46460	ferredoxin-dependent glutamate synthase, chloroplast precursor, putative, expressed
Chr7	27736445	27736446	AT	AT	A	INDEL	OS07G0658400	Intronic	NA	NA	LOC_Os07g46460	ferredoxin-dependent glutamate synthase, chloroplast precursor, putative, expressed
Chr7	28120729	28120749	AGGGCGATGCCTTCGGTAATG	AGGGCGATGCCTTCGGTAATG	A	INDEL	OS07G0665900	Exonic	CDS	FrameShift	LOC_Os07g47040	expressed protein
Chr.9	3655825	3655825	T	T	A	SNP	OS09G0248100	Intronic	NA	NA	LOC_Os09g07350	fasciclin-like arabinogalactan protein 8 precursor, putative, expressed
Chr.9	6996979	6996979	T	T	A	SNP	OS09G0294000	Intronic	NA	NA	LOC_Os09g12290	bifunctional aspartokinase/homoserine dehydrogenase, chloroplast precursor, putative, expressed
Chr.9	9653906	9653906	T	T	A	SNP	OS09G0327300	Exonic	CDS	Missense	LOC_Os09g15810	bifunctional protein folD, putative, expressed
Chr.9	9978321	9978321	T	T	A	SNP	OS09G0332700	Intronic	NA	NA	LOC_Os09g16330	pleiotropic drug resistance protein, putative, expressed
Chr.9	10523921	10523921	T	T	A	SNP	OS09G0341600	Exonic	CDS	Missense	LOC_Os09g17152	OsFBX319 - F-box domain containing protein, expressed
Chr.9	3051802	3051802	G	G	A	SNP	OS09G0240200	Intronic	NA	NA	LOC_Os09g06464	CCT/B-box zinc finger protein, putative, expressed
Chr.9	7002025	7002025	G	G	A	SNP	OS09G0294000	Exonic	CDS	Missense	LOC_Os09g12290	bifunctional aspartokinase/homoserine dehydrogenase, chloroplast precursor, putative, expressed
Chr.9	7006878	7006878	G	G	A	SNP	OS09G0294000	Exonic	CDS	Missense	LOC_Os09g12290	bifunctional aspartokinase/homoserine dehydrogenase, chloroplast precursor, putative, expressed
Chr.9	6995915	6995915	G	G	A	SNP	OS09G0294000	Intronic	NA	NA	LOC_Os09g12290	bifunctional aspartokinase/homoserine dehydrogenase, chloroplast precursor, putative, expressed
Chr.9	7006713	7006713	G	G	A	SNP	OS09G0294000	Intronic	NA	NA	LOC_Os09g12290	bifunctional aspartokinase/homoserine dehydrogenase, chloroplast precursor, putative, expressed
Chr.9	7203174	7203174	G	G	A	SNP	OS09G0297000	Exonic	CDS	NA	LOC_Os09g12560	ferrochelatase-2, chloroplast precursor, putative, expressed
Chr.9	7279095	7279095	G	G	A	SNP	OS09G0298500	Intronic	NA	NA	LOC_Os09g12720	zinc finger, C3HC4 type domain containing protein, expressed
Chr.9	7280291	7280291	G	G	A	SNP	OS09G0298500	Intronic	NA	NA	LOC_Os09g12720	zinc finger, C3HC4 type domain containing protein, expressed
Chr.9	9360471	9360471	G	G	A	SNP	OS09G0321900	Intronic	NA	NA	LOC_Os09g15320	ubiquitin-conjugating enzyme, putative, expressed
Chr.9	9360540	9360540	G	G	A	SNP	OS09G0321900	Intronic	NA	NA	LOC_Os09g15320	ubiquitin-conjugating enzyme, putative, expressed
Chr.9	9361614	9361614	G	G	A	SNP	OS09G0321900	Intronic	NA	NA	LOC_Os09g15320	ubiquitin-conjugating enzyme, putative, expressed
Chr.9	9653183	9653183	G	G	A	SNP	OS09G0327300	Exonic	CDS	Missense	LOC_Os09g15810	bifunctional protein folD, putative, expressed
Chr.9	13573188	13573188	G	G	A	SNP	OS09G0392400	Exonic	5UTR	NA	LOC_Os09g22500	expressed protein
Chr.9	13567710	13567710	G	G	A	SNP	OS09G0392400	Intronic	NA	NA	LOC_Os09g22500	expressed protein
Chr.9	14533005	14533005	G	G	A	SNP	OS09G0409950	Intronic	NA	NA	LOC_Os09g24440	expressed protein
Chr.9	14976218	14976218	G	G	A	SNP	OS09G0417600	Exonic	3UTR	NA	LOC_Os09g25060	WRKY76, expressed
Chr.9	16934077	16934077	G	G	A	SNP	OS09G0451500	Intronic	NA	NA	LOC_Os09g27830	OsPDIL2-3 protein disulfide isomerase PDIL2-3, expressed
Chr.9	16962091	16962091	G	G	A	SNP	OS09G0452200	Exonic	3UTR	NA	LOC_Os09g27890	lysM domain-containing GPI-anchored protein precursor, putative, expressed
Chr.9	17132469	17132469	G	G	A	SNP	OS09G0455300	Exonic	CDS	Missense	LOC_Os09g28210	bHelix-loop-helix transcription factor, putative, expressed
Chr.9	18982405	18982405	G	G	A	SNP	OS09G0491740	Intronic	NA	NA	LOC_Os09g31478	auxin efflux carrier component, putative, expressed
Chr.9	19583150	19583150	G	G	A	SNP	OS09G0506000	Exonic	CDS	Silent	LOC_Os09g32840	nucleotide pyrophosphatase/phosphodiesterase, putative, expressed
Chr.9	21345902	21345902	G	G	A	SNP	OS09G0542000	Intronic	NA	NA	LOC_Os09g37006	protein of unknown function domain containing protein, expressed
Chr.9	21368504	21368504	G	G	A	SNP	OS09G0542500	Intronic	NA	NA	LOC_Os09g37040	DUF630/DUF632 domains containing protein, putative, expressed
Chr.9	7003209	7003209	C	C	A	SNP	OS09G0294000	Exonic	CDS	Missense	LOC_Os09g12290	bifunctional aspartokinase/homoserine dehydrogenase, chloroplast precursor, putative, expressed
Chr.9	7000937	7000937	C	C	A	SNP	OS09G0294000	Intronic	NA	NA	LOC_Os09g12290	bifunctional aspartokinase/homoserine dehydrogenase, chloroplast precursor, putative, expressed
Chr.9	7203371	7203371	C	C	A	SNP	OS09G0297000	Intronic	NA	NA	LOC_Os09g12560	ferrochelatase-2, chloroplast precursor, putative, expressed
Chr.9	10523254	10523254	C	C	A	SNP	OS09G0341600	Exonic	CDS	Silent	LOC_Os09g17152	OsFBX319 - F-box domain containing protein, expressed
Chr.9	16960746	16960746	C	C	A	SNP	OS09G0452200	Intronic	NA	NA	LOC_Os09g27890	lysM domain-containing GPI-anchored protein precursor, putative, expressed
Chr.9	9369596	9369596	C	C	A	SNP	OS09G0322000	Intronic	NA	NA	LOC_Os09g15330	transporter family protein, putative, expressed
Chr.9	18017761	18017761	G	G	A	SNP	OS09G0471900	Exonic	CDS	Missense	LOC_Os09g29630	NOL1/NOP2/sun family protein, putative, expressed
Chr.9	18067526	18067526	C	C	A	SNP	OS09G0472900	Exonic	5UTR	NA	LOC_Os09g29710	beta-expansin precursor, putative, expressed
Chr.9	18068067	18068067	G	G	A	SNP	OS09G0472900	Intronic	NA	NA	LOC_Os09g29710	beta-expansin precursor, putative, expressed
Chr.9	18670814	18670814	G	G	A	SNP	OS09G0483400	Intronic	NA	NA	LOC_Os09g31031	ubiquitin family protein, putative, expressed
Chr.9	19013026	19013026	T	T	A	SNP	OS09G0491820	Exonic	3UTR	NA	LOC_Os09g31502	dehydrogenase, putative, expressed
Chr.9	9368650	9368654	ATTTT	ATTTT	A	INDEL	OS09G0322000	Intronic	NA	NA	LOC_Os09g15330	transporter family protein, putative, expressed
Chr.9	1538139	1538139	G	G	A	SNP	OS09G0120033	Exonic	5UTR	NA	LOC_Os09g03190	expressed protein
Chr.9	1537384	1537384	G	G	A	SNP	OS09G0120033	Intronic	NA	NA	LOC_Os09g03190	expressed protein
Chr.9	1537470	1537470	G	G	A	SNP	OS09G0120033	Intronic	NA	NA	LOC_Os09g03190	expressed protein
Chr.9	3051599	3051599	G	G	A	SNP	OS09G0240200	Intronic	NA	NA	LOC_Os09g06464	CCT/B-box zinc finger protein, putative, expressed
Chr.9	3054769	3054769	C	C	A	SNP	OS09G0240200	Intronic	NA	NA	LOC_Os09g06464	CCT/B-box zinc finger protein, putative, expressed
Chr.9	3055103	3055103	G	G	A	SNP	OS09G0240200	Intronic	NA	NA	LOC_Os09g06464	CCT/B-box zinc finger protein, putative, expressed
Chr.9	3057762	3057762	G	G	A	SNP	OS09G0240200	Intronic	NA	NA	LOC_Os09g06464	CCT/B-box zinc finger protein, putative, expressed
Chr.9	3582890	3582890	C	C	A	SNP	OS09G0247600	Exonic	CDS	Silent	LOC_Os09g07290	GDSL-like lipase/acylhydrolase, putative, expressed
Chr.9	3583634	3583634	G	G	A	SNP	OS09G0247600	Intronic	NA	NA	LOC_Os09g07290	GDSL-like lipase/acylhydrolase, putative, expressed
Chr.9	6387991	6387991	C	C	A	SNP	OS09G0286600	Exonic	3UTR	NA	LOC_Os09g11460	AP2 domain containing protein, expressed
Chr.9	6997033	6997033	T	T	A	SNP	OS09G0294000	Intronic	NA	NA	LOC_Os09g12290	bifunctional aspartokinase/homoserine dehydrogenase, chloroplast precursor, putative, expressed
Chr.9	6997399	6997399	G	G	A	SNP	OS09G0294000	Intronic	NA	NA	LOC_Os09g12290	bifunctional aspartokinase/homoserine dehydrogenase, chloroplast precursor, putative, expressed

Chr.9	7000484	7000484	C	C	A	SNP	OS09G0294000	Intronic	NA	NA	LOC_Os09g12290	bifunctional aspartokinase/homoserine dehydrogenase, chloroplast precursor, putative, expressed
Chr.9	7000525	7000525	G	G	A	SNP	OS09G0294000	Intronic	NA	NA	LOC_Os09g12290	bifunctional aspartokinase/homoserine dehydrogenase, chloroplast precursor, putative, expressed
Chr.9	7282109	7282109	G	G	A	SNP	OS09G0298500	Exonic	CDS	Silent	LOC_Os09g12720	zinc finger, C3HC4 type domain containing protein, expressed
Chr.9	13782270	13782270	C	C	A	SNP	OS09G0395600	Exonic	CDS	Missense	LOC_Os09g23220	glycosyl hydrolases family 16, putative, expressed
Chr.9	14531999	14531999	T	T	A	SNP	OS09G0409950	Intronic	NA	NA	LOC_Os09g24440	expressed protein
Chr.9	14976197	14976197	G	G	A	SNP	OS09G0417600	Exonic	3UTR	NA	LOC_Os09g25060	WRKY76, expressed
Chr.9	16447626	16447626	G	G	A	SNP	OS09G0442400	Exonic	CDS	Missense	LOC_Os09g27040	GEX1, putative, expressed
Chr.9	18013275	18013275	C	C	A	SNP	OS09G0471900	Exonic	5UTR	NA	LOC_Os09g29630	NOL1/NOP2/sun family protein, putative, expressed
Chr.9	19585947	19585947	G	G	A	SNP	OS09G0506000	Exonic	5UTR	NA	LOC_Os09g32840	nucleotide pyrophosphatase/phosphodiesterase, putative, expressed
Chr.9	3052498	3052499	AT	AT	A	INDEL	OS09G0240200	Intronic	NA	NA	LOC_Os09g06464	CCT/B-box zinc finger protein, putative, expressed
Chr.9	3584787	3584788	AT	AT	A	INDEL	OS09G0247600	Intronic	NA	NA	LOC_Os09g07290	GDSL-like lipase/acylhydrolase, putative, expressed
Chr.9	7202805	7202806	AT	AT	A	INDEL	OS09G0297000	Intronic	NA	NA	LOC_Os09g12560	ferrochelatase-2, chloroplast precursor, putative, expressed
Chr.9	7249330	7249331	AC	AC	A	INDEL	OS09G0298200	Intronic	NA	NA	LOC_Os09g12660	glucose-1-phosphate adenylyltransferase large subunit, chloroplast precursor, putative, expressed
Chr.9	9568737	9568738	AT	AT	A	INDEL	OS09G0325700	Exonic	3UTR	NA	LOC_Os09g15670	protein phosphatase 2C, putative, expressed
Chr.9	13569072	13569080	ATCTATACT	ATCTATACT	A	INDEL	OS09G0392400	Exonic	3UTR	NA	LOC_Os09g22500	expressed protein
Chr.9	13569089	13569091	ATT	ATT	A	INDEL	OS09G0392400	Exonic	3UTR	NA	LOC_Os09g22500	expressed protein
Chr.9	18442995	18442997	ACT	ACT	A	INDEL	OS09G0480400	Intronic	NA	NA	LOC_Os09g30280	glycosyl transferase 8 domain containing protein, putative, expressed
Chr.8	26466154	26466154	T	T	A	SNP	OS08G0531300	Exonic	5UTR	NA	LOC_Os08g41910	Sua5/YciO/YrdC/YwlC family protein, putative, expressed
Chr.8	26420488	26420488	T	T	A	SNP	OS08G0530400	Intronic	NA	NA	LOC_Os08g41830	sulfite oxidase, putative, expressed
Chr.8	24277202	24277202	T	T	A	SNP	OS08G0491200	Exonic	3UTR	NA	LOC_Os08g38320	AGC_PVPK_like_kin82y.14 - ACG kinases include homologs to PKA, PKG and PKC, expressed
Chr.8	12267505	12267505	T	T	A	SNP	OS08G0299400	Intronic	NA	NA	LOC_Os08g20420	MGD2, putative, expressed
Chr.8	10811032	10811032	T	T	A	SNP	OS08G0278900	Intronic	NA	NA	LOC_Os08g17680	stromal cell-derived factor 2-like protein precursor, putative, expressed
Chr.8	8763219	8763219	T	T	A	SNP	OS08G0243500	Exonic	CDS	NA	LOC_Os08g14570	NADPH reductase, putative, expressed
Chr.8	3424535	3424535	T	T	A	SNP	OS08G0158500	Exonic	3UTR	NA	LOC_Os08g06200	stomatin-like protein 2, putative, expressed
Chr.8	3424386	3424386	T	T	A	SNP	OS08G0158500	Exonic	3UTR	NA	LOC_Os08g06200	stomatin-like protein 2, putative, expressed
Chr.8	3423682	3423682	T	T	A	SNP	OS08G0158500	Intronic	NA	NA	LOC_Os08g06200	stomatin-like protein 2, putative, expressed
Chr.8	3423493	3423493	T	T	A	SNP	OS08G0158500	Intronic	NA	NA	LOC_Os08g06200	stomatin-like protein 2, putative, expressed
Chr.8	3423356	3423356	T	T	A	SNP	OS08G0158500	Intronic	NA	NA	LOC_Os08g06200	stomatin-like protein 2, putative, expressed
Chr.8	3423334	3423334	T	T	A	SNP	OS08G0158500	Intronic	NA	NA	LOC_Os08g06200	stomatin-like protein 2, putative, expressed
Chr.8	3423022	3423022	T	T	A	SNP	OS08G0158500	Intronic	NA	NA	LOC_Os08g06200	stomatin-like protein 2, putative, expressed
Chr.8	3422708	3422708	T	T	A	SNP	OS08G0158500	Intronic	NA	NA	LOC_Os08g06200	stomatin-like protein 2, putative, expressed
Chr.8	3389785	3389785	T	T	A	SNP	OS08G0157900	Intronic	NA	NA	LOC_Os08g06140	no apical meristem protein, putative, expressed
Chr.8	3365468	3365468	T	T	A	SNP	OS08G0157600	Intronic	NA	NA	LOC_Os08g06110	MYB family transcription factor, putative, expressed
Chr.8	3281601	3281601	T	T	A	SNP	OS08G0156600	Intronic	NA	NA	LOC_Os08g06010	transporter, major facilitator family, putative, expressed
Chr.8	2176241	2176241	T	T	A	SNP	OS08G0139000	Intronic	NA	NA	LOC_Os08g04440	RNA recognition motif containing protein, putative, expressed
Chr.8	2174828	2174828	T	T	A	SNP	OS08G0139000	Exonic	3UTR	NA	LOC_Os08g04440	RNA recognition motif containing protein, putative, expressed
Chr.8	1621381	1621381	T	T	A	SNP	OS08G0128500	Exonic	3UTR	NA	LOC_Os08g03450	ribosomal protein L37, putative, expressed
Chr.8	1620057	1620057	T	T	A	SNP	OS08G0128500	Intronic	NA	NA	LOC_Os08g03450	ribosomal protein L37, putative, expressed
Chr.8	1619962	1619962	T	T	A	SNP	OS08G0128500	Intronic	NA	NA	LOC_Os08g03450	ribosomal protein L37, putative, expressed
Chr.8	1561842	1561842	T	T	A	SNP	OS08G0127100	Exonic	5UTR	NA	LOC_Os08g03350	amino acid transporter, putative, expressed
Chr.8	687647	687647	T	T	A	SNP	OS08G0112900	Intronic	NA	NA	LOC_Os08g02094	GDSL-like lipase/acylhydrolase, putative, expressed
Chr.8	687008	687008	T	T	A	SNP	OS08G0112900	Intronic	NA	NA	LOC_Os08g02094	GDSL-like lipase/acylhydrolase, putative, expressed
Chr.8	26842531	26842531	G	G	A	SNP	OS08G0536800	Intronic	NA	NA	LOC_Os08g42470	BEE 1, putative, expressed
Chr.8	26466693	26466693	G	G	A	SNP	OS08G0531300	Exonic	5UTR	NA	LOC_Os08g41910	Sua5/YciO/YrdC/YwlC family protein, putative, expressed
Chr.8	26466357	26466357	G	G	A	SNP	OS08G0531300	Exonic	5UTR	NA	LOC_Os08g41910	Sua5/YciO/YrdC/YwlC family protein, putative, expressed
Chr.8	26421611	26421611	G	G	A	SNP	OS08G0530400	Exonic	CDS	Missense	LOC_Os08g41830	sulfite oxidase, putative, expressed
Chr.8	26418625	26418625	G	G	A	SNP	OS08G0530400	Intronic	NA	NA	LOC_Os08g41830	sulfite oxidase, putative, expressed
Chr.8	26391102	26391102	G	G	A	SNP	OS08G0530000	Intronic	NA	NA	LOC_Os08g41790	phosphoribulokinase/Uridine kinase family protein, putative, expressed
Chr.8	26391094	26391094	G	G	A	SNP	OS08G0530000	Intronic	NA	NA	LOC_Os08g41790	phosphoribulokinase/Uridine kinase family protein, putative, expressed
Chr.8	26390816	26390816	G	G	A	SNP	OS08G0530000	Intronic	NA	NA	LOC_Os08g41790	phosphoribulokinase/Uridine kinase family protein, putative, expressed
Chr.8	23842214	23842214	G	G	A	SNP	OS08G0482100	Intronic	NA	NA	LOC_Os08g37610	expressed protein
Chr.8	18328191	18328191	G	G	A	SNP	OS08G0387700	Intronic	NA	NA	LOC_Os08g29809	resistance protein LR10, putative, expressed
Chr.8	15663707	15663707	G	G	A	SNP	OS08G0345700	Exonic	3UTR	NA	LOC_Os08g25720	pyrophosphate--fructose 6-phosphate 1-phosphotransferase subunit alpha, putative, expressed
Chr.8	15662654	15662654	G	G	A	SNP	OS08G0345700	Intronic	NA	NA	LOC_Os08g25720	pyrophosphate--fructose 6-phosphate 1-phosphotransferase subunit alpha, putative, expressed
Chr.8	15659977	15659977	G	G	A	SNP	OS08G0345700	Intronic	NA	NA	LOC_Os08g25720	pyrophosphate--fructose 6-phosphate 1-phosphotransferase subunit alpha, putative, expressed
Chr.8	12268480	12268480	G	G	A	SNP	OS08G0299400	Intronic	NA	NA	LOC_Os08g20420	MGD2, putative, expressed
Chr.8	12268072	12268072	G	G	A	SNP	OS08G0299400	Intronic	NA	NA	LOC_Os08g20420	MGD2, putative, expressed
Chr.8	7366022	7366022	G	G	A	SNP	OS08G0221300	Intronic	NA	NA	LOC_Os08g12449	expressed protein
Chr.8	7365288	7365288	G	G	A	SNP	OS08G0221300	Intronic	NA	NA	LOC_Os08g12449	expressed protein
Chr.8	7363988	7363988	G	G	A	SNP	OS08G0221300	Exonic	3UTR	NA	LOC_Os08g12449	expressed protein
Chr.8	7363964	7363964	G	G	A	SNP	OS08G0221300	Exonic	3UTR	NA	LOC_Os08g12449	expressed protein
Chr.8	6211244	6211244	G	G	A	SNP	OS08G0206400	Exonic	3UTR	NA	LOC_Os08g10550	potassium transporter, putative, expressed
Chr.8	6206941	6206941	G	G	A	SNP	OS08G0206400	Intronic	NA	NA	LOC_Os08g10550	potassium transporter, putative, expressed
Chr.8	6206544	6206544	G	G	A	SNP	OS08G0206400	Exonic	5UTR	NA	LOC_Os08g10550	potassium transporter, putative, expressed
Chr.8	5243642	5243642	G	G	A	SNP	OS08G0189600	Exonic	3UTR	NA	LOC_Os08g09010	Cupin domain containing protein, expressed
Chr.8	5234606	5234606	G	G	A	SNP	OS08G0189400	Exonic	CDS	Missense	LOC_Os08g08990	Cupin domain containing protein, expressed
Chr.8	5234549	5234549	G	G	A	SNP	OS08G0189400	Exonic	CDS	Missense	LOC_Os08g08990	Cupin domain containing protein, expressed
Chr.8	3461515	3461515	G	G	A	SNP	OS08G0159500	Exonic	3UTR	NA	LOC_Os08g06280	LSD1 zinc finger domain containing protein, expressed
Chr.8	3424541	3424541	G	G	A	SNP	OS08G0158500	Exonic	3UTR	NA	LOC_Os08g06200	stomatin-like protein 2, putative, expressed
Chr.8	3424074	3424074	G	G	A	SNP	OS08G0158500	Exonic	CDS	Missense	LOC_Os08g06200	stomatin-like protein 2, putative, expressed
Chr.8	3422611	3422611	G	G	A	SNP	OS08G0158500	Intronic	NA	NA	LOC_Os08g06200	stomatin-like protein 2, putative, expressed
Chr.8	3422589	3422589	G	G	A	SNP	OS08G0158500	Intronic	NA	NA	LOC_Os08g06200	stomatin-like protein 2, putative, expressed
Chr.8	3422436	3422436	G	G	A	SNP	OS08G0158500	Intronic	NA	NA	LOC_Os08g06200	stomatin-like protein 2, putative, expressed
Chr.8	3421965	3421965	G	G	A	SNP	OS08G0158500	Intronic	NA	NA	LOC_Os08g06200	stomatin-like protein 2, putative, expressed
Chr.8	3373115	3373115	G	G	A	SNP	OS08G0157600	Exonic	CDS	Silent	LOC_Os08g06110	MYB family transcription factor, putative, expressed
Chr.8	3369008	3369008	G	G	A	SNP	OS08G0157600	Intronic	NA	NA	LOC_Os08g06110	MYB family transcription factor, putative, expressed
Chr.8	3364458	3364458	G	G	A	SNP	OS08G0157600	Intronic	NA	NA	LOC_Os08g06110	MYB family transcription factor, putative, expressed
Chr.8	3362484	3362484	G	G	A	SNP	OS08G0157600	Intronic	NA	NA	LOC_Os08g06110	MYB family transcription factor, putative, expressed
Chr.8	3340020	3340020	G	G	A	SNP	OS08G0157500	Intronic	NA	NA	LOC_Os08g06100	O-methyltransferase, putative, expressed
Chr.8	3339495	3339495	G	G	A	SNP	OS08G0157500	Intronic	NA	NA	LOC_Os08g06100	O-methyltransferase, putative, expressed
Chr.8	3338682	3338682	G	G	A	SNP	OS08G0157500	Exonic	CDS	Silent	LOC_Os08g06100	O-methyltransferase, putative, expressed
Chr.8	3294821	3294821	G	G	A	SNP	OS08G0156800	Intronic	NA	NA	LOC_Os08g06040	60S ribosomal protein L34, putative, expressed
Chr.8	3282345	3282345	G	G	A	SNP	OS08G0156600	Exonic	5UTR	NA	LOC_Os08g06010	transporter, major facilitator family, putative, expressed
Chr.8	2175554	2175554	G	G	A	SNP	OS08G0139000	Exonic	3UTR	NA	LOC_Os08g04440	RNA recognition motif containing protein, putative, expressed
Chr.8	2175226	2175226	G	G	A	SNP	OS08G0139000	Exonic	3UTR	NA	LOC_Os08g04440	RNA recognition motif containing protein, putative, expressed

Chr.8	2173807	2173807	G	G	A	SNP	OS08G0139000	Exonic	3UTR	NA	LOC_Os08g04440	RNA recognition motif containing protein, putative, expressed
Chr.8	2129695	2129695	G	G	A	SNP	OS08G0137800	Exonic	5UTR	NA	LOC_Os08g04340	plastocyanin-like domain containing protein, putative, expressed
Chr.8	1621044	1621044	G	G	A	SNP	OS08G0128500	Exonic	CDS	Missense	LOC_Os08g03450	ribosomal protein L37, putative, expressed
Chr.8	1619949	1619949	G	G	A	SNP	OS08G0128500	Intronic	NA	NA	LOC_Os08g03450	ribosomal protein L37, putative, expressed
Chr.8	1563760	1563760	G	G	A	SNP	OS08G0127100	Intronic	NA	NA	LOC_Os08g03350	amino acid transporter, putative, expressed
Chr.8	1562154	1562154	G	G	A	SNP	OS08G0127100	Intronic	NA	NA	LOC_Os08g03350	amino acid transporter, putative, expressed
Chr.8	689193	689193	G	G	A	SNP	OS08G0112900	Intronic	NA	NA	LOC_Os08g02094	GDSL-like lipase/acylhydrolase, putative, expressed
Chr.8	688330	688330	G	G	A	SNP	OS08G0112900	Intronic	NA	NA	LOC_Os08g02094	GDSL-like lipase/acylhydrolase, putative, expressed
Chr.8	687097	687097	G	G	A	SNP	OS08G0112900	Intronic	NA	NA	LOC_Os08g02094	GDSL-like lipase/acylhydrolase, putative, expressed
Chr.8	687056	687056	G	G	A	SNP	OS08G0112900	Intronic	NA	NA	LOC_Os08g02094	GDSL-like lipase/acylhydrolase, putative, expressed
Chr.8	581691	581691	G	G	A	SNP	OS08G0111200	Intronic	NA	NA	LOC_Os08g01940	non-lysosomal glucosylceramidase, putative, expressed
Chr.8	363565	363565	G	G	A	SNP	OS08G0107400	Exonic	3UTR	NA	LOC_Os08g01610	DUF250 domain containing protein, putative, expressed
Chr.8	26420128	26420128	C	C	A	SNP	OS08G0530400	Intronic	NA	NA	LOC_Os08g41830	sulfite oxidase, putative, expressed
Chr.8	11607650	11607650	C	C	A	SNP	OS08G0290700	Exonic	CDS	Missense	LOC_Os08g19420	O-methyltransferase, putative, expressed
Chr.8	9785725	9785725	C	C	A	SNP	OS08G0260600	Intronic	NA	NA	LOC_Os08g16050	tetraspanin family protein, putative, expressed
Chr.8	7941413	7941413	C	C	A	SNP	OS08G0230200	Intronic	NA	NA	LOC_Os08g13350	expressed protein
Chr.8	7367441	7367441	C	C	A	SNP	OS08G0221300	Intronic	NA	NA	LOC_Os08g12449	expressed protein
Chr.8	7366908	7366908	C	C	A	SNP	OS08G0221300	Intronic	NA	NA	LOC_Os08g12449	expressed protein
Chr.8	7366045	7366045	C	C	A	SNP	OS08G0221300	Intronic	NA	NA	LOC_Os08g12449	expressed protein
Chr.8	3463747	3463747	C	C	A	SNP	OS08G0159500	Exonic	5UTR	NA	LOC_Os08g06280	LSD1 zinc finger domain containing protein, expressed
Chr.8	3424350	3424350	C	C	A	SNP	OS08G0158500	Exonic	3UTR	NA	LOC_Os08g06200	stomatin-like protein 2, putative, expressed
Chr.8	3423168	3423168	C	C	A	SNP	OS08G0158500	Intronic	NA	NA	LOC_Os08g06200	stomatin-like protein 2, putative, expressed
Chr.8	3423045	3423045	C	C	A	SNP	OS08G0158500	Intronic	NA	NA	LOC_Os08g06200	stomatin-like protein 2, putative, expressed
Chr.8	3422523	3422523	C	C	A	SNP	OS08G0158500	Intronic	NA	NA	LOC_Os08g06200	stomatin-like protein 2, putative, expressed
Chr.8	3364326	3364326	C	C	A	SNP	OS08G0157600	Intronic	NA	NA	LOC_Os08g06110	MYB family transcription factor, putative, expressed
Chr.8	3243203	3243203	C	C	A	SNP	OS08G0155900	Exonic	CDS	Missense	LOC_Os08g05960	expressed protein
Chr.8	2174829	2174829	C	C	A	SNP	OS08G0139000	Exonic	3UTR	NA	LOC_Os08g04440	RNA recognition motif containing protein, putative, expressed
Chr.8	1620535	1620535	C	C	A	SNP	OS08G0128500	Intronic	NA	NA	LOC_Os08g03450	ribosomal protein L37, putative, expressed
Chr.8	687027	687027	C	C	A	SNP	OS08G0112900	Intronic	NA	NA	LOC_Os08g02094	GDSL-like lipase/acylhydrolase, putative, expressed
Chr.8	21172493	21172493	G	G	A	SNP	OS08G0435900	Exonic	CDS	Silent	LOC_Os08g33820	chlorophyll A-B binding protein, putative, expressed
Chr.8	21172365	21172365	G	G	A	SNP	OS08G0435900	Intronic	NA	NA	LOC_Os08g33820	chlorophyll A-B binding protein, putative, expressed
Chr.8	14416150	14416150	G	G	A	SNP	OS08G0327400	Intronic	NA	NA	LOC_Os08g23810	enoyl-acyl-carrier-protein reductase NADH, chloroplast precursor, expressed
Chr.8	14415979	14415980	AT	AT	A	INDEL	OS08G0327400	Intronic	NA	NA	LOC_Os08g23810	enoyl-acyl-carrier-protein reductase NADH, chloroplast precursor, expressed
Chr.8	693422	693422	G	G	A	SNP	OS08G0112900	Exonic	CDS	Silent	LOC_Os08g02094	GDSL-like lipase/acylhydrolase, putative, expressed
Chr.8	689431	689431	G	G	A	SNP	OS08G0112900	Intronic	NA	NA	LOC_Os08g02094	GDSL-like lipase/acylhydrolase, putative, expressed
Chr.8	692360	692360	G	G	A	SNP	OS08G0112900	Intronic	NA	NA	LOC_Os08g02094	GDSL-like lipase/acylhydrolase, putative, expressed
Chr.8	1834086	1834086	T	T	A	SNP	OS08G0132600	Intronic	NA	NA	LOC_Os08g03840	expressed protein
Chr.8	2130258	2130258	T	T	A	SNP	OS08G0137800	Exonic	CDS	Silent	LOC_Os08g04340	plastocyanin-like domain containing protein, putative, expressed
Chr.8	2130349	2130349	G	G	A	SNP	OS08G0137800	Exonic	CDS	Missense	LOC_Os08g04340	plastocyanin-like domain containing protein, putative, expressed
Chr.8	2173949	2173949	G	G	A	SNP	OS08G0139000	Exonic	3UTR	NA	LOC_Os08g04440	RNA recognition motif containing protein, putative, expressed
Chr.8	3243086	3243086	G	G	A	SNP	OS08G0155900	Exonic	CDS	Missense	LOC_Os08g05960	expressed protein
Chr.8	3338754	3338754	C	C	A	SNP	OS08G0157500	Intronic	NA	NA	LOC_Os08g06100	O-methyltransferase, putative, expressed
Chr.8	3372913	3372913	G	G	A	SNP	OS08G0157600	Exonic	CDS	Missense	LOC_Os08g06110	MYB family transcription factor, putative, expressed
Chr.8	3369344	3369344	T	T	A	SNP	OS08G0157600	Intronic	NA	NA	LOC_Os08g06110	MYB family transcription factor, putative, expressed
Chr.8	3423555	3423555	G	G	A	SNP	OS08G0158500	Intronic	NA	NA	LOC_Os08g06200	stomatin-like protein 2, putative, expressed
Chr.8	3423556	3423556	G	G	A	SNP	OS08G0158500	Intronic	NA	NA	LOC_Os08g06200	stomatin-like protein 2, putative, expressed
Chr.8	3462972	3462972	T	T	A	SNP	OS08G0159500	Exonic	CDS	Silent	LOC_Os08g06280	LSD1 zinc finger domain containing protein, expressed
Chr.8	3547773	3547773	G	G	A	SNP	OS08G0160500	Exonic	CDS	Silent	LOC_Os08g06380	CSLF6 - cellulose synthase-like family F; beta1,3;1,4 glucan synthase, expressed
Chr.8	3547848	3547848	G	G	A	SNP	OS08G0160500	Exonic	CDS	Silent	LOC_Os08g06380	CSLF6 - cellulose synthase-like family F; beta1,3;1,4 glucan synthase, expressed
Chr.8	3724776	3724776	T	T	A	SNP	OS08G0163100	Intronic	NA	NA	LOC_Os08g06600	nuf2 family protein, expressed
Chr.8	7366969	7366969	T	T	A	SNP	OS08G0221300	Intronic	NA	NA	LOC_Os08g12449	expressed protein
Chr.8	7367097	7367097	G	G	A	SNP	OS08G0221300	Intronic	NA	NA	LOC_Os08g12449	expressed protein
Chr.8	9387017	9387017	C	C	A	SNP	OS08G0253800	Exonic	CDS	Missense	LOC_Os08g15420	CSLC3 - cellulose synthase-like family C, expressed
Chr.8	10812866	10812866	T	T	A	SNP	OS08G0278900	Intronic	NA	NA	LOC_Os08g17680	stromal cell-derived factor 2-like protein precursor, putative, expressed
Chr.8	12266930	12266930	G	G	A	SNP	OS08G0299400	Intronic	NA	NA	LOC_Os08g20420	MGD2, putative, expressed
Chr.8	12268887	12268887	G	G	A	SNP	OS08G0299400	Intronic	NA	NA	LOC_Os08g20420	MGD2, putative, expressed
Chr.8	13954658	13954658	G	G	A	SNP	OS08G0320400	Exonic	CDS	Missense	LOC_Os08g23150	indole-3-glycerol phosphate synthase, chloroplast precursor, putative, expressed
Chr.8	15659445	15659445	G	G	A	SNP	OS08G0345700	Exonic	CDS	Missense	LOC_Os08g25720	pyrophosphate--fructose 6-phosphate 1-phosphotransferase subunit alpha, putative, expressed
Chr.8	21172544	21172544	G	G	A	SNP	OS08G0435900	Exonic	CDS	Silent	LOC_Os08g33820	chlorophyll A-B binding protein, putative, expressed
Chr.8	26418102	26418102	C	C	A	SNP	OS08G0530400	Intronic	NA	NA	LOC_Os08g41830	sulfite oxidase, putative, expressed
Chr.8	26466712	26466712	T	T	A	SNP	OS08G0531300	Exonic	5UTR	NA	LOC_Os08g41910	Sua5/YciO/YrdC/YwlC family protein, putative, expressed
Chr.8	27480823	27480823	G	G	A	SNP	OS08G0547900	Exonic	5UTR	NA	LOC_Os08g43440	cytochrome P450, putative, expressed
Chr.8	28009670	28009670	G	G	A	SNP	OS08G0559700	Exonic	5UTR	NA	LOC_Os08g44540	expressed protein
Chr.8	581776	581784	AAACTTTCT	AAACTTTCT	A	INDEL	OS08G0111200	Intronic	NA	NA	LOC_Os08g01940	non-lysosomal glucosylceramidase, putative, expressed
Chr.8	3281608	3281609	AC	AC	A	INDEL	OS08G0156600	Intronic	NA	NA	LOC_Os08g06010	transporter, major facilitator family, putative, expressed
Chr.8	3338734	3338738	AGATC	AGATC	A	INDEL	OS08G0157500	Intronic	NA	NA	LOC_Os08g06100	O-methyltransferase, putative, expressed
Chr.8	3361986	3361987	AT	AT	A	INDEL	OS08G0157600	Intronic	NA	NA	LOC_Os08g06110	MYB family transcription factor, putative, expressed
Chr.8	6168481	6168484	ACTC	ACTC	A	INDEL	OS08G0205800	Exonic	5UTR	NA	LOC_Os08g10500	expressed protein
Chr.8	7366938	7366939	AC	AC	A	INDEL	OS08G0221300	Intronic	NA	NA	LOC_Os08g12449	expressed protein
Chr.8	9099585	9099586	AG	AG	A	INDEL	OS08G0249000	Exonic	3UTR	NA	LOC_Os08g15050	CCT/B-box zinc finger protein, putative, expressed
Chr.8	26391547	26391555	AAAGGATAT	AAAGGATAT	A	INDEL	OS08G0530000	Intronic	NA	NA	LOC_Os08g41790	phosphoribulokinase/Uridine kinase family protein, expressed
Chr.8	26421751	26421753	AAT	AAT	A	INDEL	OS08G0530400	Intronic	NA	NA	LOC_Os08g41830	sulfite oxidase, putative, expressed
Chr.8	26464727	26464729	ACT	ACT	A	INDEL	OS08G0531300	Intronic	NA	NA	LOC_Os08g41910	Sua5/YciO/YrdC/YwlC family protein, putative, expressed
Chr.8	26843248	26843251	AGAG	AGAG	A	INDEL	OS08G0536800	Exonic	CDS	NA	LOC_Os08g42470	BEE 1, putative, expressed
Chr.8	26918370	26918371	AG	AG	A	INDEL	OS08G0538600	Intronic	NA	NA	LOC_Os08g42590	mtN19, putative, expressed
Chr.6	415340	415340	T	T	A	SNP	OS06G0106100	Intronic	NA	NA	LOC_Os06g01700	CWC15 homolog A, putative, expressed
Chr.6	1560424	1560424	T	T	A	SNP	OS06G0129400	Intronic	NA	NA	LOC_Os06g03860	uncharacterized membrane protein, putative, expressed
Chr.6	4654637	4654637	T	T	A	SNP	OS06G0192400	Intronic	NA	NA	LOC_Os06g09270	hypro1, putative, expressed
Chr.6	8761012	8761012	T	T	A	SNP	OS06G0265000	Intronic	NA	NA	LOC_Os06g15420	asparagine synthetase, putative, expressed
Chr.6	9095272	9095272	T	T	A	SNP	OS06G0270900	Intronic	NA	NA	LOC_Os06g15990	aldehyde dehydrogenase, putative, expressed
Chr.6	13408704	13408704	T	T	A	SNP	OS06G0336200	Exonic	3UTR	NA	LOC_Os06g22960	aquaporin protein, putative, expressed
Chr.6	13482165	13482165	T	T	A	SNP	OS06G0338200	Intronic	NA	NA	LOC_Os06g23114	copper methylamine oxidase precursor, putative, expressed
Chr.6	16267008	16267008	T	T	A	SNP	OS06G0480000	Intronic	NA	NA	LOC_Os06g28590	bg55, putative, expressed
Chr.6	16269395	16269395	T	T	A	SNP	OS06G0480000	Intronic	NA	NA	LOC_Os06g28590	bg55, putative, expressed
Chr.6	19617395	19617395	T	T	A	SNP	OS06G0528600	Intronic	NA	NA	LOC_Os06g33710	spermidine synthase, putative, expressed

Chr.6	21896475	21896475	T	T	A	SNP	OS06G0567200	Intronic	NA	NA	LOC_Os06g37080	L-ascorbate oxidase precursor, putative, expressed
Chr.6	23112365	23112365	T	T	A	SNP	OS06G0589300	Intronic	NA	NA	LOC_Os06g38950	ABC transporter, ATP-binding protein, putative, expressed
Chr.6	29385213	29385213	T	T	A	SNP	OS06G0699200	Intronic	NA	NA	LOC_Os06g48570	Ser/Thr protein phosphatase family protein, putative, expressed
Chr.6	30303949	30303949	T	T	A	SNP	OS06G0714200	Intronic	NA	NA	LOC_Os06g50030	CAMK_CAMK_like.30 - CAMK includes calcium/calmodulin depedent protein kinases, expressed
Chr.6	30307010	30307010	T	T	A	SNP	OS06G0714200	Intronic	NA	NA	LOC_Os06g50030	CAMK_CAMK_like.30 - CAMK includes calcium/calmodulin depedent protein kinases, expressed
Chr.6	30446499	30446499	T	T	A	SNP	OS06G0716700	Exonic	CDS	Missense	LOC_Os06g50300	heat shock protein, putative, expressed
Chr.6	2200015	2200015	T	T	A	SNP	OS06G0142200	Intronic	NA	NA	LOC_Os06g04990	early nodulin 93 ENOD93 protein, putative, expressed
Chr.6	307959	307959	G	G	A	SNP	OS06G0104400	Exonic	CDS	Silent	LOC_Os06g01500	IQ calmodulin-binding motif family protein, putative, expressed
Chr.6	679106	679106	G	G	A	SNP	OS06G0112100	Exonic	3UTR	NA	LOC_Os06g02210	bark storage protein A precursor, putative, expressed
Chr.6	1362267	1362267	G	G	A	SNP	OS06G0125200	Exonic	3UTR	NA	LOC_Os06g03520	DUF581 domain containing protein, expressed
Chr.6	1555976	1555976	G	G	A	SNP	OS06G0129400	Intronic	NA	NA	LOC_Os06g03860	uncharacterized membrane protein, putative, expressed
Chr.6	1559666	1559666	G	G	A	SNP	OS06G0129400	Intronic	NA	NA	LOC_Os06g03860	uncharacterized membrane protein, putative, expressed
Chr.6	1560203	1560203	G	G	A	SNP	OS06G0129400	Intronic	NA	NA	LOC_Os06g03860	uncharacterized membrane protein, putative, expressed
Chr.6	3198256	3198256	G	G	A	SNP	OS06G0163300	Intronic	NA	NA	LOC_Os06g06780	harpin-induced protein, putative, expressed
Chr.6	4323917	4323917	G	G	A	SNP	OS06G0185900	Intronic	NA	NA	LOC_Os06g08670	glutathione peroxidase, putative, expressed
Chr.6	4324224	4324224	G	G	A	SNP	OS06G0185900	Intronic	NA	NA	LOC_Os06g08670	glutathione peroxidase, putative, expressed
Chr.6	4654755	4654755	G	G	A	SNP	OS06G0192400	Intronic	NA	NA	LOC_Os06g09270	hypro1, putative, expressed
Chr.6	5281882	5281882	G	G	A	SNP	OS06G0204400	Intronic	NA	NA	LOC_Os06g10280	CDP-alcohol phosphatidyltransferase, putative, expressed
Chr.6	5409845	5409845	G	G	A	SNP	OS06G0206900	Exonic	3UTR	NA	LOC_Os06g10510	oxidoreductase/ transition metal ion binding protein, putative, expressed
Chr.6	5681254	5681254	G	G	A	SNP	OS06G0211200	Intronic	NA	NA	LOC_Os06g10880	bZIP transcription factor, putative, expressed
Chr.6	10686813	10686813	G	G	A	SNP	OS06G0291500	Exonic	3UTR	NA	LOC_Os06g18820	serine threonine kinase, putative, expressed
Chr.6	10687278	10687278	G	G	A	SNP	OS06G0291500	Exonic	3UTR	NA	LOC_Os06g18820	serine threonine kinase, putative, expressed
Chr.6	13478910	13478910	G	G	A	SNP	OS06G0338200	Intronic	NA	NA	LOC_Os06g23114	copper methylamine oxidase precursor, putative, expressed
Chr.6	13478918	13478918	G	G	A	SNP	OS06G0338200	Intronic	NA	NA	LOC_Os06g23114	copper methylamine oxidase precursor, putative, expressed
Chr.6	13479052	13479052	G	G	A	SNP	OS06G0338200	Intronic	NA	NA	LOC_Os06g23114	copper methylamine oxidase precursor, putative, expressed
Chr.6	13479067	13479067	G	G	A	SNP	OS06G0338200	Intronic	NA	NA	LOC_Os06g23114	copper methylamine oxidase precursor, putative, expressed
Chr.6	13479264	13479264	G	G	A	SNP	OS06G0338200	Intronic	NA	NA	LOC_Os06g23114	copper methylamine oxidase precursor, putative, expressed
Chr.6	13479453	13479453	G	G	A	SNP	OS06G0338200	Intronic	NA	NA	LOC_Os06g23114	copper methylamine oxidase precursor, putative, expressed
Chr.6	13479457	13479457	G	G	A	SNP	OS06G0338200	Intronic	NA	NA	LOC_Os06g23114	copper methylamine oxidase precursor, putative, expressed
Chr.6	13481707	13481707	G	G	A	SNP	OS06G0338200	Intronic	NA	NA	LOC_Os06g23114	copper methylamine oxidase precursor, putative, expressed
Chr.6	13484004	13484004	G	G	A	SNP	OS06G0338200	Intronic	NA	NA	LOC_Os06g23114	copper methylamine oxidase precursor, putative, expressed
Chr.6	13484438	13484438	G	G	A	SNP	OS06G0338200	Exonic	CDS	Missense	LOC_Os06g23114	copper methylamine oxidase precursor, putative, expressed
Chr.6	13484816	13484816	G	G	A	SNP	OS06G0338200	Exonic	CDS	Missense	LOC_Os06g23114	copper methylamine oxidase precursor, putative, expressed
Chr.6	16270803	16270803	G	G	A	SNP	OS06G0480000	Exonic	5UTR	NA	LOC_Os06g28590	bg55, putative, expressed
Chr.6	19517930	19517930	G	G	A	SNP	OS06G0526600	Intronic	NA	NA	LOC_Os06g33520	DEAD/DEAH box helicase, putative, expressed
Chr.6	19518131	19518131	G	G	A	SNP	OS06G0526600	Intronic	NA	NA	LOC_Os06g33520	DEAD/DEAH box helicase, putative, expressed
Chr.6	19614681	19614681	G	G	A	SNP	OS06G0528600	Intronic	NA	NA	LOC_Os06g33710	spermidine synthase, putative, expressed
Chr.6	19618172	19618172	G	G	A	SNP	OS06G0528600	Intronic	NA	NA	LOC_Os06g33710	spermidine synthase, putative, expressed
Chr.6	20832786	20832786	G	G	A	SNP	OS06G0549900	Exonic	3UTR	NA	LOC_Os06g35700	reticuline oxidase-like protein precursor, putative, expressed
Chr.6	21598996	21598996	G	G	A	SNP	OS06G0562600	Intronic	NA	NA	LOC_Os06g36700	T-complex protein, putative, expressed
Chr.6	21600245	21600245	G	G	A	SNP	OS06G0562600	Intronic	NA	NA	LOC_Os06g36700	T-complex protein, putative, expressed
Chr.6	21600886	21600886	G	G	A	SNP	OS06G0562600	Exonic	CDS	Silent	LOC_Os06g36700	T-complex protein, putative, expressed
Chr.6	21601991	21601991	G	G	A	SNP	OS06G0562600	Intronic	NA	NA	LOC_Os06g36700	T-complex protein, putative, expressed
Chr.6	21894716	21894716	G	G	A	SNP	OS06G0567200	Intronic	NA	NA	LOC_Os06g37080	L-ascorbate oxidase precursor, putative, expressed
Chr.6	21896372	21896372	G	G	A	SNP	OS06G0567200	Intronic	NA	NA	LOC_Os06g37080	L-ascorbate oxidase precursor, putative, expressed
Chr.6	22352788	22352788	G	G	A	SNP	OS06G0575400	Exonic	CDS	Missense	LOC_Os06g37750	S-locus-like receptor protein kinase, putative, expressed
Chr.6	22353601	22353601	G	G	A	SNP	OS06G0575400	Exonic	CDS	Missense	LOC_Os06g37750	S-locus-like receptor protein kinase, putative, expressed
Chr.6	22353842	22353842	G	G	A	SNP	OS06G0575400	Exonic	CDS	Missense	LOC_Os06g37750	S-locus-like receptor protein kinase, putative, expressed
Chr.6	23111314	23111314	G	G	A	SNP	OS06G0589300	Intronic	NA	NA	LOC_Os06g38950	ABC transporter, ATP-binding protein, putative, expressed
Chr.6	23112463	23112463	G	G	A	SNP	OS06G0589300	Intronic	NA	NA	LOC_Os06g38950	ABC transporter, ATP-binding protein, putative, expressed
Chr.6	23112507	23112507	G	G	A	SNP	OS06G0589300	Intronic	NA	NA	LOC_Os06g38950	ABC transporter, ATP-binding protein, putative, expressed
Chr.6	23468598	23468598	G	G	A	SNP	OS06G0596300	Intronic	NA	NA	LOC_Os06g39520	myristoyl-acyl carrier protein thioesterase, chloroplast precursor, putative, expressed
Chr.6	23470425	23470425	G	G	A	SNP	OS06G0596300	Exonic	3UTR	NA	LOC_Os06g39520	myristoyl-acyl carrier protein thioesterase, chloroplast precursor, putative, expressed
Chr.6	23855468	23855468	G	G	A	SNP	OS06G0603000	Intronic	NA	NA	LOC_Os06g40080	heme oxygenase 1, putative, expressed
Chr.6	24402783	24402783	G	G	A	SNP	OS06G0611900	Intronic	NA	NA	LOC_Os06g40940	glycine dehydrogenase, putative, expressed
Chr.6	24402784	24402784	G	G	A	SNP	OS06G0611900	Intronic	NA	NA	LOC_Os06g40940	glycine dehydrogenase, putative, expressed
Chr.6	25833335	25833335	G	G	A	SNP	OS06G0636100	Intronic	NA	NA	LOC_Os06g43000	nitrate-induced NOI protein, expressed
Chr.6	30446308	30446308	G	G	A	SNP	OS06G0716700	Exonic	CDS	Silent	LOC_Os06g50300	heat shock protein, putative, expressed
Chr.6	30449756	30449756	G	G	A	SNP	OS06G0716700	Intronic	NA	NA	LOC_Os06g50300	heat shock protein, putative, expressed
Chr.6	297616	297616	C	C	A	SNP	OS06G0104300	Exonic	3UTR	NA	LOC_Os06g01490	monocopper oxidase, putative, expressed
Chr.6	307687	307687	C	C	A	SNP	OS06G0104400	Intronic	NA	NA	LOC_Os06g01500	IQ calmodulin-binding motif family protein, putative, expressed
Chr.6	797803	797803	C	C	A	SNP	OS06G0113900	Exonic	CDS	Silent	LOC_Os06g02370	expressed protein
Chr.6	1556278	1556278	C	C	A	SNP	OS06G0129400	Intronic	NA	NA	LOC_Os06g03860	uncharacterized membrane protein, putative, expressed
Chr.6	1560735	1560735	C	C	A	SNP	OS06G0129400	Exonic	3UTR	NA	LOC_Os06g03860	uncharacterized membrane protein, putative, expressed
Chr.6	7230628	7230628	C	C	A	SNP	OS06G0239200	Exonic	3UTR	NA	LOC_Os06g13190	expressed protein
Chr.6	9092916	9092916	C	C	A	SNP	OS06G0270900	Intronic	NA	NA	LOC_Os06g15990	aldehyde dehydrogenase, putative, expressed
Chr.6	13481462	13481462	C	C	A	SNP	OS06G0338200	Intronic	NA	NA	LOC_Os06g23114	copper methylamine oxidase precursor, putative, expressed
Chr.6	15782239	15782239	C	C	A	SNP	OS06G0473000	Intronic	NA	NA	LOC_Os06g27860	poor homologous synapsis 1 protein, putative, expressed
Chr.6	17067869	17067869	C	C	A	SNP	OS06G0493100	Intronic	NA	NA	LOC_Os06g29730	RALFL28 - Rapid ALkalinization Factor RALF family protein precursor, expressed
Chr.6	19518367	19518367	C	C	A	SNP	OS06G0526600	Intronic	NA	NA	LOC_Os06g33520	DEAD/DEAH box helicase, putative, expressed
Chr.6	20901156	20901156	C	C	A	SNP	OS06G0551400	Intronic	NA	NA	LOC_Os06g35814	ras-related protein, putative, expressed
Chr.6	21599847	21599847	C	C	A	SNP	OS06G0562600	Intronic	NA	NA	LOC_Os06g36700	T-complex protein, putative, expressed
Chr.6	21600986	21600986	C	C	A	SNP	OS06G0562600	Exonic	CDS	Missense	LOC_Os06g36700	T-complex protein, putative, expressed
Chr.6	21601108	21601108	C	C	A	SNP	OS06G0562600	Intronic	NA	NA	LOC_Os06g36700	T-complex protein, putative, expressed
Chr.6	22354379	22354379	C	C	A	SNP	OS06G0575400	Exonic	CDS	Missense	LOC_Os06g37750	S-locus-like receptor protein kinase, putative, expressed
Chr.6	23109896	23109896	C	C	A	SNP	OS06G0589300	Intronic	NA	NA	LOC_Os06g38950	ABC transporter, ATP-binding protein, putative, expressed
Chr.6	24402761	24402761	C	C	A	SNP	OS06G0611900	Intronic	NA	NA	LOC_Os06g40940	glycine dehydrogenase, putative, expressed
Chr.6	25836247	25836247	C	C	A	SNP	OS06G0636100	Intronic	NA	NA	LOC_Os06g43000	nitrate-induced NOI protein, expressed
Chr.6	27284662	27284662	C	C	A	SNP	OS06G0661900	Exonic	5UTR	NA	LOC_Os06g45110	DNA binding protein, putative, expressed
Chr.6	28617950	28617950	C	C	A	SNP	OS06G0686400	Exonic	3UTR	NA	LOC_Os06g47200	LTPL85 - Protease inhibitor/seed storage/LTP family protein precursor, expressed
Chr.6	3363474	3363474	G	G	A	SNP	OS06G0166900	Exonic	3UTR	NA	LOC_Os06g07070	BRASSINOSTEROID INSENSITIVE 1-associated receptor kinase 1 precursor, putative, expressed
Chr.6	3450059	3450059	C	C	A	SNP	OS06G0168600	Exonic	3UTR	NA	LOC_Os06g07210	ribonucleoside-diphosphate reductase large subunit, putative, expressed
Chr.6	3451040	3451040	G	G	A	SNP	OS06G0168600	Exonic	CDS	Silent	LOC_Os06g07210	ribonucleoside-diphosphate reductase large subunit, putative, expressed
Chr.6	3453786	3453786	C	C	A	SNP	OS06G0168600	Intronic	NA	NA	LOC_Os06g07210	ribonucleoside-diphosphate reductase large subunit, putative, expressed
Chr.6	3455913	3455913	G	G	A	SNP	OS06G0168700	Exonic	3UTR	NA	LOC_Os06g07220	LTPL128 - Protease inhibitor/seed storage/LTP family protein precursor, expressed

Chr.6	6103425	6103425	T	T	A	SNP	OS06G0218900	Intronic	NA	NA	LOC_Os06g11520	LMBR1 integral membrane protein, putative, expressed
Chr.6	27135677	27135677	C	C	A	SNP	OS06G0659300	Exonic	3UTR	NA	LOC_Os06g44890	villin protein, putative, expressed
Chr.6	27136019	27136019	C	C	A	SNP	OS06G0659300	Exonic	3UTR	NA	LOC_Os06g44890	villin protein, putative, expressed
Chr.6	27136831	27136831	T	T	A	SNP	OS06G0659300	Exonic	CDS	Missense	LOC_Os06g44890	villin protein, putative, expressed
Chr.6	27138371	27138371	G	G	A	SNP	OS06G0659300	Intronic	NA	NA	LOC_Os06g44890	villin protein, putative, expressed
Chr.6	27281626	27281626	C	C	A	SNP	OS06G0661900	Intronic	NA	NA	LOC_Os06g45110	DNA binding protein, putative, expressed
Chr.6	28194337	28194337	G	G	A	SNP	OS06G0678200	Intronic	NA	NA	LOC_Os06g46450	polyprenyl synthetase, putative, expressed
Chr.6	28194744	28194744	G	G	A	SNP	OS06G0678200	Intronic	NA	NA	LOC_Os06g46450	polyprenyl synthetase, putative, expressed
Chr.6	28195860	28195860	G	G	A	SNP	OS06G0678200	Intronic	NA	NA	LOC_Os06g46450	polyprenyl synthetase, putative, expressed
Chr.6	29145292	29145292	G	G	A	SNP	OS06G0696600	Intronic	NA	NA	LOC_Os06g48180	glycosyl hydrolases family 16, putative, expressed
Chr.6	29145637	29145637	C	C	A	SNP	OS06G0696600	Intronic	NA	NA	LOC_Os06g48180	glycosyl hydrolases family 16, putative, expressed
Chr.6	29383517	29383517	G	G	A	SNP	OS06G0699200	Exonic	3UTR	NA	LOC_Os06g48570	Ser/Thr protein phosphatase family protein, putative, expressed
Chr.6	30918653	30918653	T	T	A	SNP	OS06G0726800	Intronic	NA	NA	LOC_Os06g51110	cyclin, putative, expressed
Chr.6	31069353	31069353	T	T	A	SNP	OS06G0729300	Intronic	NA	NA	LOC_Os06g51310	PAZ domain containing protein, putative, expressed
Chr.6	31070155	31070155	C	C	A	SNP	OS06G0729300	Intronic	NA	NA	LOC_Os06g51310	PAZ domain containing protein, putative, expressed
Chr.6	31070257	31070257	G	G	A	SNP	OS06G0729300	Intronic	NA	NA	LOC_Os06g51310	PAZ domain containing protein, putative, expressed
Chr.6	31071811	31071811	G	G	A	SNP	OS06G0729300	Exonic	CDS	Silent	LOC_Os06g51310	PAZ domain containing protein, putative, expressed
Chr.6	31077166	31077166	G	G	A	SNP	OS06G0729300	Exonic	3UTR	NA	LOC_Os06g51310	PAZ domain containing protein, putative, expressed
Chr.6	31129678	31129678	G	G	A	SNP	OS06G0730300	Exonic	CDS	Missense	LOC_Os06g51390	expressed protein
Chr.6	31143547	31143547	G	G	A	SNP	OS06G0730600	Exonic	CDS	Missense	LOC_Os06g51410	OsPOP15 - Putative Prolyl Oligopeptidase homologue, expressed
Chr.6	1471478	1471478	C	C	A	SNP	OS06G0127900	Exonic	CDS	Silent	LOC_Os06g03720	ribonucleoside-diphosphate reductase small chain, putative, expressed
Chr.6	2321564	2321564	T	T	A	SNP	OS06G0143900	Intronic	NA	NA	LOC_Os06g05180	coatomer subunit beta, putative, expressed
Chr.6	3640917	3640917	T	T	A	SNP	OS06G0172000	Exonic	CDS	Missense	LOC_Os06g07550	PPR repeat domain containing protein, putative, expressed
Chr.6	4322399	4322399	G	G	A	SNP	OS06G0185900	Intronic	NA	NA	LOC_Os06g08670	glutathione peroxidase, putative, expressed
Chr.6	4332793	4332793	C	C	A	SNP	OS06G0186100	Exonic	CDS	Missense	LOC_Os06g08690	leucine-rich repeat receptor protein kinase EXS precursor, putative, expressed
Chr.6	4334110	4334110	G	G	A	SNP	OS06G0186100	Exonic	CDS	Missense	LOC_Os06g08690	leucine-rich repeat receptor protein kinase EXS precursor, putative, expressed
Chr.6	4653812	4653812	G	G	A	SNP	OS06G0192400	Intronic	NA	NA	LOC_Os06g09270	hypro1, putative, expressed
Chr.6	4679485	4679485	C	C	A	SNP	OS06G0192800	Exonic	CDS	Missense	LOC_Os06g09310	zinc finger, C3HC4 type domain containing protein, expressed
Chr.6	4869598	4869598	G	G	A	SNP	OS06G0195800	Intronic	NA	NA	LOC_Os06g09560	heat shock protein DnaJ, putative, expressed
Chr.6	5677345	5677345	G	G	A	SNP	OS06G0211200	Exonic	5UTR	NA	LOC_Os06g10880	bZIP transcription factor, putative, expressed
Chr.6	5677380	5677380	G	G	A	SNP	OS06G0211200	Exonic	CDS	Silent	LOC_Os06g10880	bZIP transcription factor, putative, expressed
Chr.6	7709724	7709724	G	G	A	SNP	OS06G0248500	Exonic	CDS	Missense	LOC_Os06g13870	U-box protein CMPG1, putative, expressed
Chr.6	9092782	9092782	G	G	A	SNP	OS06G0270900	Intronic	NA	NA	LOC_Os06g15990	aldehyde dehydrogenase, putative, expressed
Chr.6	10686971	10686971	T	T	A	SNP	OS06G0291500	Exonic	3UTR	NA	LOC_Os06g18820	serine threonine kinase, putative, expressed
Chr.6	10685697	10685697	G	G	A	SNP	OS06G0291500	Exonic	CDS	Missense	LOC_Os06g18820	serine threonine kinase, putative, expressed
Chr.6	11701025	11701025	C	C	A	SNP	OS06G0308300	Intronic	NA	NA	LOC_Os06g20370	microtubule associated protein, putative, expressed
Chr.6	11701026	11701026	T	T	A	SNP	OS06G0308300	Intronic	NA	NA	LOC_Os06g20370	microtubule associated protein, putative, expressed
Chr.6	11701027	11701027	C	C	A	SNP	OS06G0308300	Intronic	NA	NA	LOC_Os06g20370	microtubule associated protein, putative, expressed
Chr.6	13043109	13043109	G	G	A	SNP	OS06G0329900	Intronic	NA	NA	LOC_Os06g22440	SAM dependent carboxyl methyltransferase, putative, expressed
Chr.6	13407563	13407563	G	G	A	SNP	OS06G0336200	Intronic	NA	NA	LOC_Os06g22960	aquaporin protein, putative, expressed
Chr.6	13407953	13407953	G	G	A	SNP	OS06G0336200	Intronic	NA	NA	LOC_Os06g22960	aquaporin protein, putative, expressed
Chr.6	13478121	13478121	G	G	A	SNP	OS06G0338200	Intronic	NA	NA	LOC_Os06g23114	copper methylamine oxidase precursor, putative, expressed
Chr.6	13479688	13479688	G	G	A	SNP	OS06G0338200	Intronic	NA	NA	LOC_Os06g23114	copper methylamine oxidase precursor, putative, expressed
Chr.6	13480721	13480721	G	G	A	SNP	OS06G0338200	Intronic	NA	NA	LOC_Os06g23114	copper methylamine oxidase precursor, putative, expressed
Chr.6	13481105	13481105	G	G	A	SNP	OS06G0338200	Intronic	NA	NA	LOC_Os06g23114	copper methylamine oxidase precursor, putative, expressed
Chr.6	13481148	13481148	G	G	A	SNP	OS06G0338200	Intronic	NA	NA	LOC_Os06g23114	copper methylamine oxidase precursor, putative, expressed
Chr.6	13481341	13481341	G	G	A	SNP	OS06G0338200	Intronic	NA	NA	LOC_Os06g23114	copper methylamine oxidase precursor, putative, expressed
Chr.6	13481505	13481505	T	T	A	SNP	OS06G0338200	Intronic	NA	NA	LOC_Os06g23114	copper methylamine oxidase precursor, putative, expressed
Chr.6	13482385	13482385	G	G	A	SNP	OS06G0338200	Intronic	NA	NA	LOC_Os06g23114	copper methylamine oxidase precursor, putative, expressed
Chr.6	16270346	16270346	T	T	A	SNP	OS06G0480000	Intronic	NA	NA	LOC_Os06g28590	bg55, putative, expressed
Chr.6	17067508	17067508	G	G	A	SNP	OS06G0493100	Intronic	NA	NA	LOC_Os06g29730	RALFL28 - Rapid ALkalinization Factor RALF family protein precursor, expressed
Chr.6	20676610	20676610	C	C	A	SNP	OS06G0546500	Exonic	CDS	Silent	LOC_Os06g35480	peroxidase precursor, putative, expressed
Chr.6	21602059	21602059	G	G	A	SNP	OS06G0562600	Intronic	NA	NA	LOC_Os06g36700	T-complex protein, putative, expressed
Chr.6	21895459	21895459	G	G	A	SNP	OS06G0567200	Intronic	NA	NA	LOC_Os06g37080	L-ascorbate oxidase precursor, putative, expressed
Chr.6	23112944	23112944	C	C	A	SNP	OS06G0589300	Exonic	CDS	Silent	LOC_Os06g38950	ABC transporter, ATP-binding protein, putative, expressed
Chr.6	23304632	23304632	G	G	A	SNP	OS06G0593100	Intronic	NA	NA	LOC_Os06g39260	solute carrier family 35 member B1, putative, expressed
Chr.6	23467935	23467935	G	G	A	SNP	OS06G0596300	Intronic	NA	NA	LOC_Os06g39520	myristoyl-acyl carrier protein thioesterase, chloroplast precursor, putative, expressed
Chr.6	28389149	28389149	G	G	A	SNP	OS06G0681200	Exonic	CDS	Silent	LOC_Os06g46740	early nodulin 20 precursor, putative, expressed
Chr.6	28389217	28389217	C	C	A	SNP	OS06G0681200	Intronic	NA	NA	LOC_Os06g46740	early nodulin 20 precursor, putative, expressed
Chr.6	28389218	28389218	C	C	A	SNP	OS06G0681200	Intronic	NA	NA	LOC_Os06g46740	early nodulin 20 precursor, putative, expressed
Chr.6	28389281	28389281	G	G	A	SNP	OS06G0681200	Intronic	NA	NA	LOC_Os06g46740	early nodulin 20 precursor, putative, expressed
Chr.6	31069647	31069647	C	C	A	SNP	OS06G0729300	Intronic	NA	NA	LOC_Os06g51310	PAZ domain containing protein, putative, expressed
Chr.6	31069981	31069981	G	G	A	SNP	OS06G0729300	Intronic	NA	NA	LOC_Os06g51310	PAZ domain containing protein, putative, expressed
Chr.6	1361368	1361370	ATC	ATC	A	INDEL	OS06G0125200	Exonic	5UTR	NA	LOC_Os06g03520	DUF581 domain containing protein, expressed
Chr.6	1361789	1361801	ATTGATTGGTTCT	ATTGATTGGTTCT	A	INDEL	OS06G0125200	Intronic	NA	NA	LOC_Os06g03520	DUF581 domain containing protein, expressed
Chr.6	2321546	2321548	ATT	ATT	A	INDEL	OS06G0143900	Intronic	NA	NA	LOC_Os06g05180	coatomer subunit beta, putative, expressed
Chr.6	4869720	4869732	AATCCTGAAGCTG	AATCCTGAAGCTG	A	INDEL	OS06G0195800	Intronic	NA	NA	LOC_Os06g09560	heat shock protein DnaJ, putative, expressed
Chr.6	13042238	13042239	AT	AT	A	INDEL	OS06G0329900	Intronic	NA	NA	LOC_Os06g22440	SAM dependent carboxyl methyltransferase, putative, expressed
Chr.6	13042302	13042303	AT	AT	A	INDEL	OS06G0329900	Intronic	NA	NA	LOC_Os06g22440	SAM dependent carboxyl methyltransferase, putative, expressed
Chr.6	13481784	13481812	AGCCTATTCAGATTCGTTCGACTGAAAGC	AGCCTATTCAGATTCGTTCGACTGAAAGC	A	INDEL	OS06G0338200	Intronic	NA	NA	LOC_Os06g23114	copper methylamine oxidase precursor, putative, expressed
Chr.6	13482114	13482116	AAT	AAT	A	INDEL	OS06G0338200	Intronic	NA	NA	LOC_Os06g23114	copper methylamine oxidase precursor, putative, expressed
Chr.6	13483905	13483907	AAT	AAT	A	INDEL	OS06G0338200	Intronic	NA	NA	LOC_Os06g23114	copper methylamine oxidase precursor, putative, expressed
Chr.6	19615917	19615919	AAT	AAT	A	INDEL	OS06G0528600	Intronic	NA	NA	LOC_Os06g33710	spermidine synthase, putative, expressed
Chr.6	19618453	19618467	AGCAGCTGCAGTGCT	AGCAGCTGCAGTGCT	A	INDEL	OS06G0528600	Intronic	NA	NA	LOC_Os06g33710	spermidine synthase, putative, expressed
Chr.6	20900123	20900125	ACT	ACT	A	INDEL	OS06G0551400	Intronic	NA	NA	LOC_Os06g35814	ras-related protein, putative, expressed
Chr.6	21895570	21895572	ATT	ATT	A	INDEL	OS06G0567200	Intronic	NA	NA	LOC_Os06g37080	L-ascorbate oxidase precursor, putative, expressed
Chr.6	23468990	23468996	AGTTTTC	AGTTTTC	A	INDEL	OS06G0596300	Intronic	NA	NA	LOC_Os06g39520	myristoyl-acyl carrier protein thioesterase, chloroplast precursor, putative, expressed
Chr.6	27137287	27137295	AGGTGATCC	AGGTGATCC	A	INDEL	OS06G0659300	Intronic	NA	NA	LOC_Os06g44890	villin protein, putative, expressed
Chr.5	28855100	28855100	T	T	A	SNP	OS05G0579800	Intronic	NA	NA	LOC_Os05g50360	anaphase-promoting complex subunit 10, putative, expressed
Chr.5	24777694	24777694	T	T	A	SNP	OS05G0503300	Exonic	3UTR	NA	LOC_Os05g42350	ferredoxin--nitrite reductase, putative, expressed
Chr.5	23423317	23423317	G	G	A	SNP	OS05G0476200	Exonic	CDS	Missense	LOC_Os05g39850	MCM3 - Putative minichromosome maintenance MCM complex subunit 3, expressed
Chr.5	22072123	22072123	T	T	A	SNP	OS05G0449600	Exonic	3UTR	NA	LOC_Os05g37700	periplasmic beta-glucosidase precursor, putative, expressed
Chr.5	22071846	22071846	G	G	A	SNP	OS05G0449600	Exonic	CDS	Missense	LOC_Os05g37700	periplasmic beta-glucosidase precursor, putative, expressed
Chr.5	19770128	19770128	G	G	A	SNP	OS05G0405900	Intronic	NA	NA	LOC_Os05g33610	expressed protein
Chr.5	5653616	5653616	T	T	A	SNP	OS05G0192100	Intronic	NA	NA	LOC_Os05g10370	acid phosphatase, putative, expressed

Chr.5	741526	741526	G	G	A	SNP	OS05G0114000	Intronic	NA	NA	LOC_Os05g02310	soluble inorganic pyrophosphatase, putative, expressed
Chr.5	742338	742338	T	T	A	SNP	OS05G0114000	Intronic	NA	NA	LOC_Os05g02310	soluble inorganic pyrophosphatase, putative, expressed
Chr.5	27960675	27960675	C	C	A	SNP	OS05G0562000	Intronic	NA	NA	LOC_Os05g48790	expressed protein
Chr.5	27701364	27701364	G	G	A	SNP	OS05G0557000	Intronic	NA	NA	LOC_Os05g48320	60S ribosomal protein L37a, putative, expressed
Chr.5	27700579	27700579	G	G	A	SNP	OS05G0557000	Intronic	NA	NA	LOC_Os05g48320	60S ribosomal protein L37a, putative, expressed
Chr.5	27633043	27633043	G	G	A	SNP	OS05G0555600	Intronic	NA	NA	LOC_Os05g48200	glutamate synthase, chloroplast precursor, putative, expressed
Chr.5	26369002	26369002	G	G	A	SNP	OS05G0531100	Exonic	3UTR	NA	LOC_Os05g45450	DUF584 domain containing protein, putative, expressed
Chr.5	25546070	25546070	G	G	A	SNP	OS05G0515200	Exonic	3UTR	NA	LOC_Os05g43910	cytochrome P450, putative, expressed
Chr.5	25545778	25545778	G	G	A	SNP	OS05G0515200	Exonic	3UTR	NA	LOC_Os05g43910	cytochrome P450, putative, expressed
Chr.5	25545748	25545748	G	G	A	SNP	OS05G0515200	Exonic	3UTR	NA	LOC_Os05g43910	cytochrome P450, putative, expressed
Chr.5	25545430	25545430	G	G	A	SNP	OS05G0515200	Intronic	NA	NA	LOC_Os05g43910	cytochrome P450, putative, expressed
Chr.5	25545336	25545336	G	G	A	SNP	OS05G0515200	Intronic	NA	NA	LOC_Os05g43910	cytochrome P450, putative, expressed
Chr.5	25545045	25545045	C	C	A	SNP	OS05G0515200	Intronic	NA	NA	LOC_Os05g43910	cytochrome P450, putative, expressed
Chr.5	23869048	23869048	C	C	A	SNP	OS05G0485300	Intronic	NA	NA	LOC_Os05g40700	transmembrane protein 56, putative, expressed
Chr.5	23865817	23865817	C	C	A	SNP	OS05G0485300	Exonic	5UTR	NA	LOC_Os05g40700	transmembrane protein 56, putative, expressed
Chr.5	22070908	22070908	G	G	A	SNP	OS05G0449600	Intronic	NA	NA	LOC_Os05g37700	periplasmic beta-glucosidase precursor, putative, expressed
Chr.5	22070773	22070773	T	T	A	SNP	OS05G0449600	Intronic	NA	NA	LOC_Os05g37700	periplasmic beta-glucosidase precursor, putative, expressed
Chr.5	20039287	20039287	G	G	A	SNP	OS05G0410200	Exonic	3UTR	NA	LOC_Os05g33940	alpha/beta hydrolase fold, putative, expressed
Chr.5	19960768	19960768	G	G	A	SNP	OS05G0408900	Intronic	NA	NA	LOC_Os05g33840	transketolase, putative, expressed
Chr.5	19364896	19364896	G	G	A	SNP	OS05G0398000	Exonic	5UTR	NA	LOC_Os05g33040	cyclin, putative, expressed
Chr.5	14297401	14297401	C	C	A	SNP	OS05G0312000	Intronic	NA	NA	LOC_Os05g24684	structural constituent of ribosome, putative, expressed
Chr.5	14166941	14166941	G	G	A	SNP	OS05G0310500	Exonic	3UTR	NA	LOC_Os05g24550	Papain family cysteine protease domain containing protein, expressed
Chr.5	14163496	14163496	G	G	A	SNP	OS05G0310500	Intronic	NA	NA	LOC_Os05g24550	Papain family cysteine protease domain containing protein, expressed
Chr.5	14161731	14161731	G	G	A	SNP	OS05G0310500	Exonic	CDS	NA	LOC_Os05g24550	Papain family cysteine protease domain containing protein, expressed
Chr.5	8002886	8002886	C	C	A	SNP	OS05G0231700	Exonic	3UTR	NA	LOC_Os05g14240	aquaporin protein, putative, expressed
Chr.5	7164053	7164053	C	C	A	SNP	OS05G0215600	Exonic	3UTR	NA	LOC_Os05g12474	expressed protein
Chr.5	5654000	5654000	G	G	A	SNP	OS05G0192100	Intronic	NA	NA	LOC_Os05g10370	acid phosphatase, putative, expressed
Chr.5	5653818	5653818	T	T	A	SNP	OS05G0192100	Intronic	NA	NA	LOC_Os05g10370	acid phosphatase, putative, expressed
Chr.5	3759627	3759627	G	G	A	SNP	OS05G0163900	Exonic	CDS	Missense	LOC_Os05g07120	basic helix-loop-helix, putative, expressed
Chr.5	3758871	3758871	G	G	A	SNP	OS05G0163900	Intronic	NA	NA	LOC_Os05g07120	basic helix-loop-helix, putative, expressed
Chr.5	3758769	3758769	G	G	A	SNP	OS05G0163900	Intronic	NA	NA	LOC_Os05g07120	basic helix-loop-helix, putative, expressed
Chr.5	3758434	3758434	G	G	A	SNP	OS05G0163900	Intronic	NA	NA	LOC_Os05g07120	basic helix-loop-helix, putative, expressed
Chr.5	3758206	3758206	G	G	A	SNP	OS05G0163900	Intronic	NA	NA	LOC_Os05g07120	basic helix-loop-helix, putative, expressed
Chr.5	3731514	3731514	C	C	A	SNP	OS05G0163700	Intronic	NA	NA	LOC_Os05g07090	acyl-coenzyme A dehydrogenase, mitochondrial precursor, putative, expressed
Chr.5	3610248	3610248	G	G	A	SNP	OS05G0161200	Intronic	NA	NA	LOC_Os05g06890	relA-SpoT like protein RSH4, putative, expressed
Chr.5	2789835	2789835	G	G	A	SNP	OS05G0148700	Intronic	NA	NA	LOC_Os05g05600	ATA15 protein, putative, expressed
Chr.5	2788366	2788366	G	G	A	SNP	OS05G0148700	Exonic	CDS	Silent	LOC_Os05g05600	ATA15 protein, putative, expressed
Chr.5	2718868	2718868	G	G	A	SNP	OS05G0147400	Exonic	3UTR	NA	LOC_Os05g05470	T-complex protein, putative, expressed
Chr.5	2178154	2178154	C	C	A	SNP	OS05G0137400	Intronic	NA	NA	LOC_Os05g04630	retrotransposon protein, putative, SINE subclass, expressed
Chr.5	19768835	19768835	G	G	A	SNP	OS05G0405900	Exonic	5UTR	NA	LOC_Os05g33610	expressed protein
Chr.5	27714905	27714905	T	T	A	SNP	OS05G0557200	Intronic	NA	NA	LOC_Os05g48340	proteasome-related, putative, expressed
Chr.5	25544972	25544972	G	G	A	SNP	OS05G0515200	Intronic	NA	NA	LOC_Os05g43910	cytochrome P450, putative, expressed
Chr.5	23868017	23868017	G	G	A	SNP	OS05G0485300	Intronic	NA	NA	LOC_Os05g40700	transmembrane protein 56, putative, expressed
Chr.5	23423850	23423850	C	C	A	SNP	OS05G0476200	Intronic	NA	NA	LOC_Os05g39850	MCM3 - Putative minichromosome maintenance MCM complex subunit 3, expressed
Chr.5	23423576	23423576	T	T	A	SNP	OS05G0476200	Intronic	NA	NA	LOC_Os05g39850	MCM3 - Putative minichromosome maintenance MCM complex subunit 3, expressed
Chr.5	15487116	15487116	G	G	A	SNP	OS05G0331200	Intronic	NA	NA	LOC_Os05g26660	NADH-ubiquinone oxidoreductase, mitochondrial precursor, putative, expressed
Chr.5	3723091	3723091	T	T	A	SNP	OS05G0163400	Exonic	5UTR	NA	LOC_Os05g07070	zinc finger, C3HC4 type domain containing protein, expressed
Chr.5	1002430	1002430	C	C	A	SNP	OS05G0119300	Exonic	5UTR	NA	LOC_Os05g02780	glycine-rich protein A3, putative, expressed
Chr.5	26928313	26928313	C	C	A	SNP	OS05G0542500	Exonic	CDS	Missense	LOC_Os05g46480	late embryogenesis abundant protein, group 3, putative, expressed
Chr.5	24108948	24108948	G	G	A	SNP	OS05G0490500	Intronic	NA	NA	LOC_Os05g41160	expressed protein
Chr.5	24015731	24015731	G	G	A	SNP	OS05G0488600	Exonic	CDS	Silent	LOC_Os05g40960	Myb transcription factor, putative, expressed
Chr.5	23750699	23750699	G	G	A	SNP	OS05G0482700	Exonic	CDS	Missense	LOC_Os05g40420	2,3-bisphosphoglycerate-independent phosphoglycerate mutase, putative, expressed
Chr.5	21189934	21189934	G	G	A	SNP	OS05G0431700	Intronic	NA	NA	LOC_Os05g35650	peptide transporter PTR2, putative, expressed
Chr.5	20630374	20630374	G	G	A	SNP	OS05G0420600	Intronic	NA	NA	LOC_Os05g34770	cytochrome c, putative, expressed
Chr.5	20320335	20320335	G	G	A	SNP	OS05G0415700	Exonic	CDS	Silent	LOC_Os05g34320	beta-hexosaminidase precursor, putative, expressed
Chr.5	19771144	19771144	T	T	A	SNP	OS05G0405900	Exonic	CDS	Silent	LOC_Os05g33610	expressed protein
Chr.5	19768892	19768892	G	G	A	SNP	OS05G0405900	Exonic	5UTR	NA	LOC_Os05g33610	expressed protein
Chr.5	15077802	15077802	G	G	A	SNP	OS05G0324700	Exonic	3UTR	NA	LOC_Os05g25920	expressed protein
Chr.5	6789086	6789086	G	G	A	SNP	OS05G0209600	Exonic	CDS	Silent	LOC_Os05g11910	GDSL-like lipase/acylhydrolase, putative, expressed
Chr.5	2786172	2786172	C	C	A	SNP	OS05G0148700	Exonic	5UTR	NA	LOC_Os05g05600	ATA15 protein, putative, expressed
Chr.5	873768	873768	T	T	A	SNP	OS05G0116000	Intronic	NA	NA	LOC_Os05g02520	cupin domain containing protein, expressed
Chr.5	740720	740720	T	T	A	SNP	OS05G0114000	Intronic	NA	NA	LOC_Os05g02310	soluble inorganic pyrophosphatase, putative, expressed
Chr.5	874155	874155	G	G	A	SNP	OS05G0116000	Exonic	CDS	Silent	LOC_Os05g02520	cupin domain containing protein, expressed
Chr.5	880267	880267	G	G	A	SNP	OS05G0116100	Intronic	NA	NA	LOC_Os05g02530	glutathione S-transferase, N-terminal domain containing protein, expressed
Chr.5	1001753	1001753	G	G	A	SNP	OS05G0119300	Exonic	CDS	Missense	LOC_Os05g02780	glycine-rich protein A3, putative, expressed
Chr.5	1994412	1994412	G	G	A	SNP	OS05G0134200	Exonic	5UTR	NA	LOC_Os05g04360	protein phosphatase 2C, putative, expressed
Chr.5	2104201	2104201	C	C	A	SNP	OS05G0135900	Intronic	NA	NA	LOC_Os05g04530	IF, putative, expressed
Chr.5	2176721	2176721	G	G	A	SNP	OS05G0137400	Exonic	CDS	Silent	LOC_Os05g04630	retrotransposon protein, putative, SINE subclass, expressed
Chr.5	3607877	3607877	G	G	A	SNP	OS05G0161200	Exonic	CDS	Missense	LOC_Os05g06890	relA-SpoT like protein RSH4, putative, expressed
Chr.5	3609276	3609276	G	G	A	SNP	OS05G0161200	Exonic	CDS	Missense	LOC_Os05g06890	relA-SpoT like protein RSH4, putative, expressed
Chr.5	3757952	3757952	C	C	A	SNP	OS05G0163900	Intronic	NA	NA	LOC_Os05g07120	basic helix-loop-helix, putative, expressed
Chr.5	3758641	3758641	G	G	A	SNP	OS05G0163900	Intronic	NA	NA	LOC_Os05g07120	basic helix-loop-helix, putative, expressed
Chr.5	3758911	3758911	G	G	A	SNP	OS05G0163900	Intronic	NA	NA	LOC_Os05g07120	basic helix-loop-helix, putative, expressed
Chr.5	3759033	3759033	C	C	A	SNP	OS05G0163900	Intronic	NA	NA	LOC_Os05g07120	basic helix-loop-helix, putative, expressed
Chr.5	4139179	4139179	C	C	A	SNP	OS05G0169000	Exonic	5UTR	NA	LOC_Os05g07690	thioredoxin, putative, expressed
Chr.5	4137899	4137899	T	T	A	SNP	OS05G0169000	Exonic	CDS	NA	LOC_Os05g07690	thioredoxin, putative, expressed
Chr.5	5653090	5653090	G	G	A	SNP	OS05G0192100	Exonic	3UTR	NA	LOC_Os05g10370	acid phosphatase, putative, expressed
Chr.5	5653275	5653275	G	G	A	SNP	OS05G0192100	Exonic	3UTR	NA	LOC_Os05g10370	acid phosphatase, putative, expressed
Chr.5	5653339	5653339	C	C	A	SNP	OS05G0192100	Intronic	NA	NA	LOC_Os05g10370	acid phosphatase, putative, expressed
Chr.5	7164376	7164376	G	G	A	SNP	OS05G0215600	Intronic	NA	NA	LOC_Os05g12474	expressed protein
Chr.5	7164841	7164841	G	G	A	SNP	OS05G0215600	Intronic	NA	NA	LOC_Os05g12474	expressed protein
Chr.5	8902788	8902788	T	T	A	SNP	OS05G0247100	Exonic	3UTR	NA	LOC_Os05g15770	glycosyl hydrolase, putative, expressed
Chr.5	8903298	8903298	G	G	A	SNP	OS05G0247100	Exonic	CDS	Silent	LOC_Os05g15770	glycosyl hydrolase, putative, expressed
Chr.5	9328044	9328044	G	G	A	SNP	OS05G0253200	Exonic	3UTR	NA	LOC_Os05g16420	SHR5-receptor-like kinase, putative, expressed
Chr.5	14162933	14162933	T	T	A	SNP	OS05G0310500	Intronic	NA	NA	LOC_Os05g24550	Papain family cysteine protease domain containing protein, expressed

Chr.5	14992834	14992834	C	C	A	SNP	OS05G0322900	Exonic	CDS	Missense	LOC_Os05g25770	WRKY45, expressed
Chr.5	18487429	18487429	G	G	A	SNP	OS05G0382500	Exonic	CDS	Missense	LOC_Os05g31740	cytochrome P450 86A2, putative, expressed
Chr.5	18759125	18759125	G	G	A	SNP	OS05G0387700	Exonic	CDS	Missense	LOC_Os05g32170	TKL_IRAK_CR4L.5 - The CR4L subfamily has homology with Crinkly4, expressed
Chr.5	19828971	19828971	T	T	A	SNP	OS05G0406800	Exonic	5UTR	NA	LOC_Os05g33690	receptor-like protein kinase precursor, putative, expressed
Chr.5	19829821	19829821	G	G	A	SNP	OS05G0406800	Exonic	CDS	Silent	LOC_Os05g33690	receptor-like protein kinase precursor, putative, expressed
Chr.5	19959878	19959878	G	G	A	SNP	OS05G0408900	Intronic	NA	NA	LOC_Os05g33840	transketolase, putative, expressed
Chr.5	19960930	19960930	G	G	A	SNP	OS05G0408900	Intronic	NA	NA	LOC_Os05g33840	transketolase, putative, expressed
Chr.5	21188845	21188845	G	G	A	SNP	OS05G0431700	Intronic	NA	NA	LOC_Os05g35650	peptide transporter PTR2, putative, expressed
Chr.5	21189877	21189877	T	T	A	SNP	OS05G0431700	Intronic	NA	NA	LOC_Os05g35650	peptide transporter PTR2, putative, expressed
Chr.5	23497442	23497442	C	C	A	SNP	OS05G0477600	Exonic	CDS	Silent	LOC_Os05g39990	expansin precursor, putative, expressed
Chr.5	24281133	24281133	G	G	A	SNP	OS05G0494200	Exonic	3UTR	NA	LOC_Os05g41460	cysteine proteinase inhibitor precursor protein, putative, expressed
Chr.5	24326478	24326478	T	T	A	SNP	OS05G0495300	Exonic	3UTR	NA	LOC_Os05g41550	expressed protein
Chr.5	24583425	24583425	G	G	A	SNP	OS05G0499300	Exonic	CDS	Silent	LOC_Os05g41990	peroxidase precursor, putative, expressed
Chr.5	24584050	24584050	G	G	A	SNP	OS05G0499300	Exonic	CDS	Silent	LOC_Os05g41990	peroxidase precursor, putative, expressed
Chr.5	24584441	24584441	T	T	A	SNP	OS05G0499300	Exonic	CDS	Missense	LOC_Os05g41990	peroxidase precursor, putative, expressed
Chr.5	24632568	24632568	G	G	A	SNP	OS05G0500600	Exonic	3UTR	NA	LOC_Os05g42130	MONOCULM 1, putative, expressed
Chr.5	24632073	24632073	G	G	A	SNP	OS05G0500600	Exonic	CDS	Silent	LOC_Os05g42130	MONOCULM 1, putative, expressed
Chr.5	24633390	24633390	G	G	A	SNP	OS05G0500700	Exonic	CDS	NA	LOC_Os05g42140	methyltransferase domain containing protein, expressed
Chr.5	24633676	24633676	T	T	A	SNP	OS05G0500700	Exonic	CDS	NA	LOC_Os05g42140	methyltransferase domain containing protein, expressed
Chr.5	25545464	25545464	G	G	A	SNP	OS05G0515200	Intronic	NA	NA	LOC_Os05g43910	cytochrome P450, putative, expressed
Chr.5	27122184	27122184	C	C	A	SNP	OS05G0546400	Exonic	CDS	Silent	LOC_Os05g46840	proline-rich protein, putative, expressed
Chr.5	27700048	27700048	G	G	A	SNP	OS05G0557000	Intronic	NA	NA	LOC_Os05g48320	60S ribosomal protein L37a, putative, expressed
Chr.5	27959567	27959567	G	G	A	SNP	OS05G0562000	Intronic	NA	NA	LOC_Os05g48790	expressed protein
Chr.5	28472597	28472597	G	G	A	SNP	OS05G0571200	Exonic	CDS	Missense	LOC_Os05g49620	WRKY19, expressed
Chr.5	1994375	1994376	AT	AT	A	INDEL	OS05G0134200	Exonic	5UTR	NA	LOC_Os05g04360	protein phosphatase 2C, putative, expressed
Chr.5	3758981	3758982	AT	AT	A	INDEL	OS05G0163900	Intronic	NA	NA	LOC_Os05g07120	basic helix-loop-helix, putative, expressed
Chr.5	4303247	4303248	AT	AT	A	INDEL	OS05G0171900	Intronic	NA	NA	LOC_Os05g07940	glyoxalase family protein, putative, expressed
Chr.5	7161889	7161892	AGAG	AGAG	A	INDEL	OS05G0215600	Exonic	CDS	InFrame	LOC_Os05g12474	expressed protein
Chr.5	8001475	8001476	AT	AT	A	INDEL	OS05G0231700	Intronic	NA	NA	LOC_Os05g14240	aquaporin protein, putative, expressed
Chr.5	14166898	14166899	AT	AT	A	INDEL	OS05G0310500	Exonic	3UTR	NA	LOC_Os05g24550	Papain family cysteine protease domain containing protein, expressed
Chr.5	19633371	19633374	AAAG	AAAG	A	INDEL	OS05G0402900	Exonic	3UTR	NA	LOC_Os05g33400	basic 7S globulin precursor, putative, expressed
Chr.5	23752595	23752597	AAG	AAG	A	INDEL	OS05G0482700	Intronic	NA	NA	LOC_Os05g40420	2,3-bisphosphoglycerate-independent phosphoglycerate mutase, putative, expressed
Chr.5	23867397	23867398	AT	AT	A	INDEL	OS05G0485300	Intronic	NA	NA	LOC_Os05g40700	transmembrane protein 56, putative, expressed
Chr.5	25546126	25546127	AG	AG	A	INDEL	OS05G0515200	Exonic	3UTR	NA	LOC_Os05g43910	cytochrome P450, putative, expressed
Chr.5	27960073	27960085	ATGTGTGTGTGTG	ATGTGTGTGTGTG	A	INDEL	OS05G0562000	Intronic	NA	NA	LOC_Os05g48790	expressed protein
Chr.4	31441342	31441342	T	T	A	SNP	OS04G0619000	Intronic	NA	NA	LOC_Os04g52810	no apical meristem protein, putative, expressed
Chr.4	31439755	31439755	T	T	A	SNP	OS04G0619000	Exonic	3UTR	NA	LOC_Os04g52810	no apical meristem protein, putative, expressed
Chr.4	31220854	31220854	T	T	A	SNP	OS04G0615200	Exonic	5UTR	NA	LOC_Os04g52504	adhesive/proline-rich protein, putative, expressed
Chr.4	31181514	31181514	C	C	A	SNP	OS04G0614600	Intronic	NA	NA	LOC_Os04g52450	aminotransferase, putative, expressed
Chr.4	30288651	30288651	T	T	A	SNP	OS04G0600200	Intronic	NA	NA	LOC_Os04g51150	transposon protein, putative, unclassified, expressed
Chr.4	30288505	30288505	T	T	A	SNP	OS04G0600200	Intronic	NA	NA	LOC_Os04g51150	transposon protein, putative, unclassified, expressed
Chr.4	29562892	29562892	T	T	A	SNP	OS04G0585100	Intronic	NA	NA	LOC_Os04g49560	zinc finger family protein, putative, expressed
Chr.4	29557288	29557288	T	T	A	SNP	OS04G0585100	Exonic	CDS	Missense	LOC_Os04g49560	zinc finger family protein, putative, expressed
Chr.4	29044918	29044918	T	T	A	SNP	OS04G0576500	Intronic	NA	NA	LOC_Os04g48710	expressed protein
Chr.4	27505701	27505701	T	T	A	SNP	OS04G0549600	Intronic	NA	NA	LOC_Os04g46390	chaperone protein dnaJ, putative, expressed
Chr.4	22624227	22624227	T	T	A	SNP	OS04G0453233	Intronic	NA	NA	LOC_Os04g38026	transporter family protein, putative, expressed
Chr.4	22185485	22185485	T	T	A	SNP	OS04G0444800	Intronic	NA	NA	LOC_Os04g36720	ferric-chelate reductase, putative, expressed
Chr.4	16005049	16005049	T	T	A	SNP	OS04G0339400	Exonic	3UTR	NA	LOC_Os04g27060	oxidoreductase, aldo/keto reductase family protein, putative, expressed
Chr.4	16004173	16004173	T	T	A	SNP	OS04G0339400	Intronic	NA	NA	LOC_Os04g27060	oxidoreductase, aldo/keto reductase family protein, putative, expressed
Chr.4	16003862	16003862	T	T	A	SNP	OS04G0339400	Intronic	NA	NA	LOC_Os04g27060	oxidoreductase, aldo/keto reductase family protein, putative, expressed
Chr.4	16003297	16003297	T	T	A	SNP	OS04G0339400	Intronic	NA	NA	LOC_Os04g27060	oxidoreductase, aldo/keto reductase family protein, putative, expressed
Chr.4	7307880	7307880	T	T	A	SNP	OS04G0209200	Intronic	NA	NA	LOC_Os04g13210	multidrug resistance-associated protein, putative, expressed
Chr.4	6481133	6481133	T	T	A	SNP	OS04G0194500	Intronic	NA	NA	LOC_Os04g11820	white-brown complex homolog protein, putative, expressed
Chr.4	6480536	6480536	T	T	A	SNP	OS04G0194500	Intronic	NA	NA	LOC_Os04g11820	white-brown complex homolog protein, putative, expressed
Chr.4	6479798	6479798	T	T	A	SNP	OS04G0194500	Intronic	NA	NA	LOC_Os04g11820	white-brown complex homolog protein, putative, expressed
Chr.4	6226145	6226145	T	T	A	SNP	OS04G0191400	Intronic	NA	NA	LOC_Os04g11390	expressed protein
Chr.4	33532001	33532001	G	G	A	SNP	OS04G0657500	Intronic	NA	NA	LOC_Os04g56240	lipase, putative, expressed
Chr.4	31441271	31441271	G	G	A	SNP	OS04G0619000	Intronic	NA	NA	LOC_Os04g52810	no apical meristem protein, putative, expressed
Chr.4	31180040	31180040	G	G	A	SNP	OS04G0614600	Intronic	NA	NA	LOC_Os04g52450	aminotransferase, putative, expressed
Chr.4	31178615	31178615	G	G	A	SNP	OS04G0614600	Intronic	NA	NA	LOC_Os04g52450	aminotransferase, putative, expressed
Chr.4	30911426	30911426	G	G	A	SNP	OS04G0609900	Exonic	CDS	Silent	LOC_Os04g52040	TNP1, putative, expressed
Chr.4	30601123	30601123	G	G	A	SNP	OS04G0605900	Exonic	CDS	Silent	LOC_Os04g51630	60S ribosomal protein L7, putative, expressed
Chr.4	29976583	29976583	G	G	A	SNP	OS04G0592600	Intronic	NA	NA	LOC_Os04g50216	SNARE associated Golgi protein, putative, expressed
Chr.4	29561075	29561075	G	G	A	SNP	OS04G0585100	Intronic	NA	NA	LOC_Os04g49560	zinc finger family protein, putative, expressed
Chr.4	29559523	29559523	G	G	A	SNP	OS04G0585100	Intronic	NA	NA	LOC_Os04g49560	zinc finger family protein, putative, expressed
Chr.4	29559220	29559220	G	G	A	SNP	OS04G0585100	Exonic	CDS	Silent	LOC_Os04g49560	zinc finger family protein, putative, expressed
Chr.4	29558768	29558768	G	G	A	SNP	OS04G0585100	Intronic	NA	NA	LOC_Os04g49560	zinc finger family protein, putative, expressed
Chr.4	29523770	29523770	G	G	A	SNP	OS04G0584500	Exonic	CDS	Missense	LOC_Os04g49500	U-box domain-containing protein, putative, expressed
Chr.4	29317573	29317573	G	G	A	SNP	OS04G0580800	Exonic	3UTR	NA	LOC_Os04g49160	zinc finger, C3HC4 type domain containing protein, expressed
Chr.4	29317372	29317372	G	G	A	SNP	OS04G0580800	Exonic	3UTR	NA	LOC_Os04g49160	zinc finger, C3HC4 type domain containing protein, expressed
Chr.4	28302795	28302795	G	G	A	SNP	OS04G0564700	Exonic	CDS	Missense	LOC_Os04g47700	expressed protein
Chr.4	28302549	28302549	G	G	A	SNP	OS04G0564700	Intronic	NA	NA	LOC_Os04g47700	expressed protein
Chr.4	27848630	27848630	G	G	A	SNP	OS04G0556500	Exonic	3UTR	NA	LOC_Os04g46980	cis-zeatin O-glucosyltransferase, putative, expressed
Chr.4	27236453	27236453	G	G	A	SNP	OS04G0543900	Exonic	3UTR	NA	LOC_Os04g45970	glutamate dehydrogenase protein, putative, expressed
Chr.4	24852443	24852443	G	G	A	SNP	OS04G0496800	Exonic	CDS	Silent	LOC_Os04g41950	calcium-binding mitochondrial protein anon-60Da, putative, expressed
Chr.4	24056995	24056995	G	G	A	SNP	OS04G0480900	Exonic	CDS	Silent	LOC_Os04g40490	glycosyl hydrolase family 5 protein, putative, expressed
Chr.4	23846763	23846763	G	G	A	SNP	OS04G0476400	Intronic	NA	NA	LOC_Os04g40050	ribonuclease H2 subunit B, putative, expressed
Chr.4	23845427	23845427	G	G	A	SNP	OS04G0476400	Exonic	3UTR	NA	LOC_Os04g40050	ribonuclease H2 subunit B, putative, expressed
Chr.4	23830082	23830082	G	G	A	SNP	OS04G0476100	Exonic	CDS	NA	LOC_Os04g40040	copper methylamine oxidase precursor, putative, expressed
Chr.4	23409979	23409979	G	G	A	SNP	OS04G0468600	Exonic	5UTR	NA	LOC_Os04g39320	expressed protein
Chr.4	23316060	23316060	G	G	A	SNP	OS04G0466600	Intronic	NA	NA	LOC_Os04g39210	agmatine deiminase, putative, expressed
Chr.4	22626282	22626282	G	G	A	SNP	OS04G0453233	Intronic	NA	NA	LOC_Os04g38026	transporter family protein, putative, expressed
Chr.4	22625884	22625884	G	G	A	SNP	OS04G0453233	Intronic	NA	NA	LOC_Os04g38026	transporter family protein, putative, expressed
Chr.4	22625276	22625276	G	G	A	SNP	OS04G0453233	Intronic	NA	NA	LOC_Os04g38026	transporter family protein, putative, expressed
Chr.4	22624520	22624520	G	G	A	SNP	OS04G0453233	Intronic	NA	NA	LOC_Os04g38026	transporter family protein, putative, expressed

Chr.4	22209389	22209389	G	G	A	SNP	OS04G0445400	Exonic	3UTR	NA	LOC_Os04g36780	expressed protein
Chr.4	22185377	22185377	G	G	A	SNP	OS04G0444800	Exonic	CDS	Missense	LOC_Os04g36720	ferric-chelate reductase, putative, expressed
Chr.4	22023704	22023704	G	G	A	SNP	OS04G0442200	Intronic	NA	NA	LOC_Os04g36062	expressed protein
Chr.4	22021000	22021000	G	G	A	SNP	OS04G0442200	Intronic	NA	NA	LOC_Os04g36062	expressed protein
Chr.4	20426320	20426320	G	G	A	SNP	OS04G0413500	Intronic	NA	NA	LOC_Os04g33740	glycosyl hydrolases, putative, expressed
Chr.4	20426241	20426241	G	G	A	SNP	OS04G0413500	Intronic	NA	NA	LOC_Os04g33740	glycosyl hydrolases, putative, expressed
Chr.4	20425708	20425708	G	G	A	SNP	OS04G0413500	Intronic	NA	NA	LOC_Os04g33740	glycosyl hydrolases, putative, expressed
Chr.4	20425616	20425616	G	G	A	SNP	OS04G0413500	Intronic	NA	NA	LOC_Os04g33740	glycosyl hydrolases, putative, expressed
Chr.4	20423199	20423199	G	G	A	SNP	OS04G0413500	Intronic	NA	NA	LOC_Os04g33740	glycosyl hydrolases, putative, expressed
Chr.4	20422552	20422552	G	G	A	SNP	OS04G0413500	Intronic	NA	NA	LOC_Os04g33740	glycosyl hydrolases, putative, expressed
Chr.4	20422523	20422523	G	G	A	SNP	OS04G0413500	Intronic	NA	NA	LOC_Os04g33740	glycosyl hydrolases, putative, expressed
Chr.4	20389377	20389377	G	G	A	SNP	OS04G0412500	Exonic	CDS	Silent	LOC_Os04g33660	bifunctional monodehydroascorbate reductase and carbonic anhydrasenectarin-3 precursor, putative, expressed
Chr.4	16005016	16005016	G	G	A	SNP	OS04G0339400	Exonic	CDS	Silent	LOC_Os04g27060	oxidoreductase, aldo/keto reductase family protein, putative, expressed
Chr.4	16004524	16004524	G	G	A	SNP	OS04G0339400	Intronic	NA	NA	LOC_Os04g27060	oxidoreductase, aldo/keto reductase family protein, putative, expressed
Chr.4	16004403	16004403	G	G	A	SNP	OS04G0339400	Intronic	NA	NA	LOC_Os04g27060	oxidoreductase, aldo/keto reductase family protein, putative, expressed
Chr.4	16004159	16004159	G	G	A	SNP	OS04G0339400	Intronic	NA	NA	LOC_Os04g27060	oxidoreductase, aldo/keto reductase family protein, putative, expressed
Chr.4	16004030	16004030	G	G	A	SNP	OS04G0339400	Exonic	CDS	Missense	LOC_Os04g27060	oxidoreductase, aldo/keto reductase family protein, putative, expressed
Chr.4	16003215	16003215	G	G	A	SNP	OS04G0339400	Intronic	NA	NA	LOC_Os04g27060	oxidoreductase, aldo/keto reductase family protein, putative, expressed
Chr.4	13651269	13651269	G	G	A	SNP	OS04G0304200	Intronic	NA	NA	LOC_Os04g23890	AGC_PVPK_like_kin82y.10 - ACG kinases include homologs to PKA, PKG and PKC, expressed
Chr.4	13647561	13647561	G	G	A	SNP	OS04G0304200	Intronic	NA	NA	LOC_Os04g23890	AGC_PVPK_like_kin82y.10 - ACG kinases include homologs to PKA, PKG and PKC, expressed
Chr.4	13645111	13645111	G	G	A	SNP	OS04G0304200	Intronic	NA	NA	LOC_Os04g23890	AGC_PVPK_like_kin82y.10 - ACG kinases include homologs to PKA, PKG and PKC, expressed
Chr.4	11285678	11285678	G	G	A	SNP	OS04G0270100	Intronic	NA	NA	LOC_Os04g20220	elongation factor Tu, putative, expressed
Chr.4	11236581	11236581	G	G	A	SNP	OS04G0269600	Exonic	CDS	Missense	LOC_Os04g20164	amine oxidase precursor, putative, expressed
Chr.4	8587147	8587147	G	G	A	SNP	OS04G0228100	Intronic	NA	NA	LOC_Os04g15800	expressed protein
Chr.4	7309030	7309030	G	G	A	SNP	OS04G0209200	Intronic	NA	NA	LOC_Os04g13210	multidrug resistance-associated protein, putative, expressed
Chr.4	7308586	7308586	G	G	A	SNP	OS04G0209200	Intronic	NA	NA	LOC_Os04g13210	multidrug resistance-associated protein, putative, expressed
Chr.4	7308411	7308411	G	G	A	SNP	OS04G0209200	Intronic	NA	NA	LOC_Os04g13210	multidrug resistance-associated protein, putative, expressed
Chr.4	6481336	6481336	G	G	A	SNP	OS04G0194500	Intronic	NA	NA	LOC_Os04g11820	white-brown complex homolog protein, putative, expressed
Chr.4	6480772	6480772	G	G	A	SNP	OS04G0194500	Intronic	NA	NA	LOC_Os04g11820	white-brown complex homolog protein, putative, expressed
Chr.4	6480727	6480727	G	G	A	SNP	OS04G0194500	Intronic	NA	NA	LOC_Os04g11820	white-brown complex homolog protein, putative, expressed
Chr.4	6480223	6480223	G	G	A	SNP	OS04G0194500	Intronic	NA	NA	LOC_Os04g11820	white-brown complex homolog protein, putative, expressed
Chr.4	6480047	6480047	G	G	A	SNP	OS04G0194500	Intronic	NA	NA	LOC_Os04g11820	white-brown complex homolog protein, putative, expressed
Chr.4	6479506	6479506	G	G	A	SNP	OS04G0194500	Intronic	NA	NA	LOC_Os04g11820	white-brown complex homolog protein, putative, expressed
Chr.4	6479501	6479501	G	G	A	SNP	OS04G0194500	Intronic	NA	NA	LOC_Os04g11820	white-brown complex homolog protein, putative, expressed
Chr.4	6479499	6479499	G	G	A	SNP	OS04G0194500	Intronic	NA	NA	LOC_Os04g11820	white-brown complex homolog protein, putative, expressed
Chr.4	6479444	6479444	G	G	A	SNP	OS04G0194500	Intronic	NA	NA	LOC_Os04g11820	white-brown complex homolog protein, putative, expressed
Chr.4	6479264	6479264	G	G	A	SNP	OS04G0194500	Intronic	NA	NA	LOC_Os04g11820	white-brown complex homolog protein, putative, expressed
Chr.4	6478972	6478972	G	G	A	SNP	OS04G0194500	Exonic	CDS	Silent	LOC_Os04g11820	white-brown complex homolog protein, putative, expressed
Chr.4	6478964	6478964	G	G	A	SNP	OS04G0194500	Exonic	CDS	Silent	LOC_Os04g11820	white-brown complex homolog protein, putative, expressed
Chr.4	6478580	6478580	G	G	A	SNP	OS04G0194500	Exonic	CDS	Silent	LOC_Os04g11820	white-brown complex homolog protein, putative, expressed
Chr.4	6478283	6478283	G	G	A	SNP	OS04G0194500	Exonic	CDS	Silent	LOC_Os04g11820	white-brown complex homolog protein, putative, expressed
Chr.4	6478042	6478042	G	G	A	SNP	OS04G0194500	Exonic	CDS	Silent	LOC_Os04g11820	white-brown complex homolog protein, putative, expressed
Chr.4	6477959	6477959	G	G	A	SNP	OS04G0194500	Exonic	CDS	Silent	LOC_Os04g11820	white-brown complex homolog protein, putative, expressed
Chr.4	1149764	1149764	G	G	A	SNP	OS04G0119500	Intronic	NA	NA	LOC_Os04g02910	membrane related protein CP5, putative, expressed
Chr.4	621145	621145	G	G	A	SNP	OS04G0110600	Intronic	NA	NA	LOC_Os04g02000	zinc finger family protein, putative, expressed
Chr.4	617956	617956	G	G	A	SNP	OS04G0110600	Exonic	3UTR	NA	LOC_Os04g02000	zinc finger family protein, putative, expressed
Chr.4	617637	617637	G	G	A	SNP	OS04G0110600	Exonic	3UTR	NA	LOC_Os04g02000	zinc finger family protein, putative, expressed
Chr.4	31178842	31178842	C	C	A	SNP	OS04G0614600	Intronic	NA	NA	LOC_Os04g52450	aminotransferase, putative, expressed
Chr.4	31177764	31177764	C	C	A	SNP	OS04G0614600	Intronic	NA	NA	LOC_Os04g52450	aminotransferase, putative, expressed
Chr.4	31177157	31177157	C	C	A	SNP	OS04G0614600	Intronic	NA	NA	LOC_Os04g52450	aminotransferase, putative, expressed
Chr.4	30288933	30288933	C	C	A	SNP	OS04G0600200	Intronic	NA	NA	LOC_Os04g51150	transposon protein, putative, unclassified, expressed
Chr.4	29977189	29977189	C	C	A	SNP	OS04G0592600	Intronic	NA	NA	LOC_Os04g50216	SNARE associated Golgi protein, putative, expressed
Chr.4	29661993	29661993	C	C	A	SNP	OS04G0587400	Exonic	CDS	Silent	LOC_Os04g49748	purine permease, putative, expressed
Chr.4	29562908	29562908	C	C	A	SNP	OS04G0585100	Intronic	NA	NA	LOC_Os04g49560	zinc finger family protein, putative, expressed
Chr.4	29559107	29559107	C	C	A	SNP	OS04G0585100	Exonic	CDS	Missense	LOC_Os04g49560	zinc finger family protein, putative, expressed
Chr.4	29525446	29525446	C	C	A	SNP	OS04G0584500	Intronic	NA	NA	LOC_Os04g49500	U-box domain-containing protein, putative, expressed
Chr.4	29466861	29466861	C	C	A	SNP	OS04G0583200	Exonic	5UTR	NA	LOC_Os04g49370	expressed protein
Chr.4	29466069	29466069	C	C	A	SNP	OS04G0583200	Exonic	3UTR	NA	LOC_Os04g49370	expressed protein
Chr.4	29317553	29317553	C	C	A	SNP	OS04G0580800	Exonic	3UTR	NA	LOC_Os04g49160	zinc finger, C3HC4 type domain containing protein, expressed
Chr.4	29045569	29045569	C	C	A	SNP	OS04G0576500	Intronic	NA	NA	LOC_Os04g48710	expressed protein
Chr.4	27848813	27848813	C	C	A	SNP	OS04G0556500	Exonic	3UTR	NA	LOC_Os04g46980	cis-zeatin O-glucosyltransferase, putative, expressed
Chr.4	27235372	27235372	C	C	A	SNP	OS04G0543900	Exonic	3UTR	NA	LOC_Os04g45970	glutamate dehydrogenase protein, putative, expressed
Chr.4	24847672	24847672	C	C	A	SNP	OS04G0496800	Intronic	NA	NA	LOC_Os04g41950	calcium-binding mitochondrial protein anon-60Da, putative, expressed
Chr.4	24847561	24847561	C	C	A	SNP	OS04G0496800	Intronic	NA	NA	LOC_Os04g41950	calcium-binding mitochondrial protein anon-60Da, putative, expressed
Chr.4	24301736	24301736	C	C	A	SNP	OS04G0486500	Exonic	3UTR	NA	LOC_Os04g40940	mitotic spindle checkpoint protein MAD2, putative, expressed
Chr.4	22259883	22259883	C	C	A	SNP	OS04G0446500	Intronic	NA	NA	LOC_Os04g36890	peptidyl-prolyl cis-trans isomerase, FKBP-type, putative, expressed
Chr.4	22021281	22021281	C	C	A	SNP	OS04G0442200	Intronic	NA	NA	LOC_Os04g36062	expressed protein
Chr.4	20425640	20425640	C	C	A	SNP	OS04G0413500	Intronic	NA	NA	LOC_Os04g33740	glycosyl hydrolases, putative, expressed
Chr.4	16005028	16005028	C	C	A	SNP	OS04G0339400	Exonic	CDS	Silent	LOC_Os04g27060	oxidoreductase, aldo/keto reductase family protein, putative, expressed
Chr.4	16004288	16004288	C	C	A	SNP	OS04G0339400	Intronic	NA	NA	LOC_Os04g27060	oxidoreductase, aldo/keto reductase family protein, putative, expressed
Chr.4	16003755	16003755	C	C	A	SNP	OS04G0339400	Intronic	NA	NA	LOC_Os04g27060	oxidoreductase, aldo/keto reductase family protein, putative, expressed
Chr.4	6481279	6481279	C	C	A	SNP	OS04G0194500	Exonic	CDS	Missense	LOC_Os04g11820	white-brown complex homolog protein, putative, expressed
Chr.4	6481233	6481233	C	C	A	SNP	OS04G0194500	Intronic	NA	NA	LOC_Os04g11820	white-brown complex homolog protein, putative, expressed
Chr.4	6479462	6479462	C	C	A	SNP	OS04G0194500	Intronic	NA	NA	LOC_Os04g11820	white-brown complex homolog protein, putative, expressed
Chr.4	6478279	6478279	C	C	A	SNP	OS04G0194500	Exonic	CDS	Missense	LOC_Os04g11820	white-brown complex homolog protein, putative, expressed
Chr.4	318684	318684	C	C	A	SNP	OS04G0104900	Intronic	NA	NA	LOC_Os04g01470	O-methyltransferase, putative, expressed
Chr.4	33419501	33419501	G	G	A	SNP	OS04G0655300	Exonic	5UTR	NA	LOC_Os04g56110	protein kinase, putative, expressed
Chr.4	33181144	33181144	C	C	A	SNP	OS04G0651000	Exonic	CDS	Silent	LOC_Os04g55740	peroxidase precursor, putative, expressed
Chr.4	33181143	33181143	G	G	A	SNP	OS04G0651000	Exonic	CDS	Silent	LOC_Os04g55740	peroxidase precursor, putative, expressed
Chr.4	33180655	33180655	G	G	A	SNP	OS04G0651000	Exonic	CDS	Silent	LOC_Os04g55740	peroxidase precursor, putative, expressed
Chr.4	33169435	33169435	G	G	A	SNP	OS04G0650800	Exonic	CDS	Silent	LOC_Os04g55720	D-3-phosphoglycerate dehydrogenase, chloroplast precursor, putative, expressed
Chr.4	33169387	33169387	G	G	A	SNP	OS04G0650800	Exonic	CDS	Silent	LOC_Os04g55720	D-3-phosphoglycerate dehydrogenase, chloroplast precursor, putative, expressed
Chr.4	33168268	33168268	G	G	A	SNP	OS04G0650800	Exonic	CDS	Silent	LOC_Os04g55720	D-3-phosphoglycerate dehydrogenase, chloroplast precursor, putative, expressed
Chr.4	33126220	33126220	C	C	A	SNP	OS04G0650000	Intronic	NA	NA	LOC_Os04g55650	oryzain alpha chain precursor, putative, expressed
Chr.4	33124268	33124268	G	G	A	SNP	OS04G0650000	Exonic	CDS	NA	LOC_Os04g55650	oryzain alpha chain precursor, putative, expressed

Chr.4	31465586	31465586	T	T	A	SNP	OS04G0619400	Intronic	NA	NA	LOC_Os04g52840	tyrosine protein kinase domain containing protein, putative, expressed
Chr.4	31465324	31465324	C	C	A	SNP	OS04G0619400	Intronic	NA	NA	LOC_Os04g52840	tyrosine protein kinase domain containing protein, putative, expressed
Chr.4	31239824	31239824	T	T	A	SNP	OS04G0615700	Intronic	NA	NA	LOC_Os04g52540	retrotransposon protein, putative, unclassified, expressed
Chr.4	31179957	31179957	G	G	A	SNP	OS04G0614600	Intronic	NA	NA	LOC_Os04g52450	aminotransferase, putative, expressed
Chr.4	31109761	31109761	C	C	A	SNP	OS04G0613700	Intronic	NA	NA	LOC_Os04g52370	UTP--glucose-1-phosphate uridylyltransferase, putative, expressed
Chr.4	29525738	29525738	T	T	A	SNP	OS04G0584500	Intronic	NA	NA	LOC_Os04g49500	U-box domain-containing protein, putative, expressed
Chr.4	28304778	28304778	T	T	A	SNP	OS04G0564700	Intronic	NA	NA	LOC_Os04g47700	expressed protein
Chr.4	28304569	28304569	G	G	A	SNP	OS04G0564700	Intronic	NA	NA	LOC_Os04g47700	expressed protein
Chr.4	28301325	28301325	G	G	A	SNP	OS04G0564700	Intronic	NA	NA	LOC_Os04g47700	expressed protein
Chr.4	28299539	28299539	G	G	A	SNP	OS04G0564700	Intronic	NA	NA	LOC_Os04g47700	expressed protein
Chr.4	28296715	28296715	G	G	A	SNP	OS04G0564700	Exonic	CDS	Silent	LOC_Os04g47700	expressed protein
Chr.4	28084543	28084543	G	G	A	SNP	OS04G0560700	Exonic	5UTR	NA	LOC_Os04g47310	expressed protein
Chr.4	27848769	27848769	G	G	A	SNP	OS04G0556500	Exonic	3UTR	NA	LOC_Os04g46980	cis-zeatin O-glucosyltransferase, putative, expressed
Chr.4	27848731	27848731	G	G	A	SNP	OS04G0556500	Exonic	3UTR	NA	LOC_Os04g46980	cis-zeatin O-glucosyltransferase, putative, expressed
Chr.4	27786662	27786662	C	C	A	SNP	OS04G0555300	Intronic	NA	NA	LOC_Os04g46880	transporter, major facilitator family, putative, expressed
Chr.4	27786392	27786392	G	G	A	SNP	OS04G0555300	Intronic	NA	NA	LOC_Os04g46880	transporter, major facilitator family, putative, expressed
Chr.4	27784668	27784668	C	C	A	SNP	OS04G0555300	Intronic	NA	NA	LOC_Os04g46880	transporter, major facilitator family, putative, expressed
Chr.4	27784558	27784558	G	G	A	SNP	OS04G0555300	Intronic	NA	NA	LOC_Os04g46880	transporter, major facilitator family, putative, expressed
Chr.4	27767235	27767235	G	G	A	SNP	OS04G0555000	Exonic	3UTR	NA	LOC_Os04g46860	scarecrow, putative, expressed
Chr.4	27638912	27638912	C	C	A	SNP	OS04G0551800	Exonic	CDS	Silent	LOC_Os04g46620	T-complex protein, putative, expressed
Chr.4	27638650	27638650	G	G	A	SNP	OS04G0551800	Intronic	NA	NA	LOC_Os04g46620	T-complex protein, putative, expressed
Chr.4	27638604	27638604	G	G	A	SNP	OS04G0551800	Intronic	NA	NA	LOC_Os04g46620	T-complex protein, putative, expressed
Chr.4	27570368	27570368	G	G	A	SNP	OS04G0550600	Exonic	3UTR	NA	LOC_Os04g46470	carotenoid cleavage dioxygenase 7, chloroplast precursor, putative, expressed
Chr.4	27568838	27568838	G	G	A	SNP	OS04G0550600	Exonic	CDS	Silent	LOC_Os04g46470	carotenoid cleavage dioxygenase 7, chloroplast precursor, putative, expressed
Chr.4	27507843	27507843	T	T	A	SNP	OS04G0549600	Intronic	NA	NA	LOC_Os04g46390	chaperone protein dnaJ, putative, expressed
Chr.4	27237544	27237544	C	C	A	SNP	OS04G0543900	Intronic	NA	NA	LOC_Os04g45970	glutamate dehydrogenase protein, putative, expressed
Chr.4	27236507	27236507	G	G	A	SNP	OS04G0543900	Exonic	3UTR	NA	LOC_Os04g45970	glutamate dehydrogenase protein, putative, expressed
Chr.4	25194265	25194265	G	G	A	SNP	OS04G0504800	Exonic	3UTR	NA	LOC_Os04g42600	polyadenylate-binding protein, putative, expressed
Chr.4	25133907	25133907	T	T	A	SNP	OS04G0503500	Intronic	NA	NA	LOC_Os04g42470	regulatory subunit, putative, expressed
Chr.4	25133906	25133906	G	G	A	SNP	OS04G0503500	Intronic	NA	NA	LOC_Os04g42470	regulatory subunit, putative, expressed
Chr.4	24886210	24886210	G	G	A	SNP	OS04G0497600	Exonic	CDS	Silent	LOC_Os04g42010	RNA binding protein, putative, expressed
Chr.4	24886126	24886126	C	C	A	SNP	OS04G0497600	Intronic	NA	NA	LOC_Os04g42010	RNA binding protein, putative, expressed
Chr.4	24886064	24886064	T	T	A	SNP	OS04G0497600	Intronic	NA	NA	LOC_Os04g42010	RNA binding protein, putative, expressed
Chr.4	24885965	24885965	G	G	A	SNP	OS04G0497600	Exonic	CDS	Silent	LOC_Os04g42010	RNA binding protein, putative, expressed
Chr.4	24885588	24885588	G	G	A	SNP	OS04G0497600	Exonic	CDS	Silent	LOC_Os04g42010	RNA binding protein, putative, expressed
Chr.4	24884548	24884548	G	G	A	SNP	OS04G0497600	Intronic	NA	NA	LOC_Os04g42010	RNA binding protein, putative, expressed
Chr.4	24883919	24883919	G	G	A	SNP	OS04G0497600	Exonic	CDS	Silent	LOC_Os04g42010	RNA binding protein, putative, expressed
Chr.4	24853021	24853021	C	C	A	SNP	OS04G0496800	Intronic	NA	NA	LOC_Os04g41950	calcium-binding mitochondrial protein anon-60Da, putative, expressed
Chr.4	24708822	24708822	G	G	A	SNP	OS04G0494100	Exonic	3UTR	NA	LOC_Os04g41680	CHIT3 - Chitinase family protein precursor, expressed
Chr.4	24530000	24530000	G	G	A	SNP	OS04G0490800	Intronic	NA	NA	LOC_Os04g41340	4-nitrophenylphosphatase, putative, expressed
Chr.4	24529999	24529999	G	G	A	SNP	OS04G0490800	Intronic	NA	NA	LOC_Os04g41340	4-nitrophenylphosphatase, putative, expressed
Chr.4	24324364	24324364	G	G	A	SNP	OS04G0486900	Intronic	NA	NA	LOC_Os04g40980	Divergent PAP2 family domain containing protein, expressed
Chr.4	24323403	24323403	G	G	A	SNP	OS04G0486900	Exonic	CDS	Silent	LOC_Os04g40980	Divergent PAP2 family domain containing protein, expressed
Chr.4	24321782	24321782	T	T	A	SNP	OS04G0486900	Intronic	NA	NA	LOC_Os04g40980	Divergent PAP2 family domain containing protein, expressed
Chr.4	24058738	24058738	T	T	A	SNP	OS04G0480900	Exonic	3UTR	NA	LOC_Os04g40490	glycosyl hydrolase family 5 protein, putative, expressed
Chr.4	24058086	24058086	G	G	A	SNP	OS04G0480900	Intronic	NA	NA	LOC_Os04g40490	glycosyl hydrolase family 5 protein, putative, expressed
Chr.4	24056539	24056539	G	G	A	SNP	OS04G0480900	Intronic	NA	NA	LOC_Os04g40490	glycosyl hydrolase family 5 protein, putative, expressed
Chr.4	24055938	24055938	G	G	A	SNP	OS04G0480900	Intronic	NA	NA	LOC_Os04g40490	glycosyl hydrolase family 5 protein, putative, expressed
Chr.4	24055581	24055581	G	G	A	SNP	OS04G0480900	Intronic	NA	NA	LOC_Os04g40490	glycosyl hydrolase family 5 protein, putative, expressed
Chr.4	24055516	24055516	C	C	A	SNP	OS04G0480900	Intronic	NA	NA	LOC_Os04g40490	glycosyl hydrolase family 5 protein, putative, expressed
Chr.4	23847905	23847905	C	C	A	SNP	OS04G0476400	Exonic	3UTR	NA	LOC_Os04g40050	ribonuclease H2 subunit B, putative, expressed
Chr.4	23847876	23847876	G	G	A	SNP	OS04G0476400	Exonic	3UTR	NA	LOC_Os04g40050	ribonuclease H2 subunit B, putative, expressed
Chr.4	23831700	23831700	G	G	A	SNP	OS04G0476100	Intronic	NA	NA	LOC_Os04g40040	copper methylamine oxidase precursor, putative, expressed
Chr.4	23315537	23315537	G	G	A	SNP	OS04G0466600	Intronic	NA	NA	LOC_Os04g39210	agmatine deiminase, putative, expressed
Chr.4	22261594	22261594	G	G	A	SNP	OS04G0446500	Intronic	NA	NA	LOC_Os04g36890	peptidyl-prolyl cis-trans isomerase, FKBP-type, putative, expressed
Chr.4	22260035	22260035	G	G	A	SNP	OS04G0446500	Intronic	NA	NA	LOC_Os04g36890	peptidyl-prolyl cis-trans isomerase, FKBP-type, putative, expressed
Chr.4	22259856	22259856	T	T	A	SNP	OS04G0446500	Intronic	NA	NA	LOC_Os04g36890	peptidyl-prolyl cis-trans isomerase, FKBP-type, putative, expressed
Chr.4	22259814	22259814	C	C	A	SNP	OS04G0446500	Intronic	NA	NA	LOC_Os04g36890	peptidyl-prolyl cis-trans isomerase, FKBP-type, putative, expressed
Chr.4	22209458	22209458	G	G	A	SNP	OS04G0445400	Exonic	3UTR	NA	LOC_Os04g36780	expressed protein
Chr.4	22209319	22209319	C	C	A	SNP	OS04G0445400	Exonic	3UTR	NA	LOC_Os04g36780	expressed protein
Chr.4	22208428	22208428	G	G	A	SNP	OS04G0445400	Intronic	NA	NA	LOC_Os04g36780	expressed protein
Chr.4	22183880	22183880	G	G	A	SNP	OS04G0444800	Intronic	NA	NA	LOC_Os04g36720	ferric-chelate reductase, putative, expressed
Chr.4	20144862	20144862	T	T	A	SNP	OS04G0405900	Exonic	5UTR	NA	LOC_Os04g33310	expressed protein
Chr.4	20144445	20144445	G	G	A	SNP	OS04G0405900	Exonic	5UTR	NA	LOC_Os04g33310	expressed protein
Chr.4	5325832	5325832	G	G	A	SNP	OS04G0178300	Exonic	CDS	Missense	LOC_Os04g09900	ent-kaurene synthase, chloroplast precursor, putative, expressed
Chr.4	5325731	5325731	G	G	A	SNP	OS04G0178300	Intronic	NA	NA	LOC_Os04g09900	ent-kaurene synthase, chloroplast precursor, putative, expressed
Chr.4	5325448	5325448	T	T	A	SNP	OS04G0178300	Intronic	NA	NA	LOC_Os04g09900	ent-kaurene synthase, chloroplast precursor, putative, expressed
Chr.4	5325413	5325413	C	C	A	SNP	OS04G0178300	Exonic	CDS	Missense	LOC_Os04g09900	ent-kaurene synthase, chloroplast precursor, putative, expressed
Chr.4	5325212	5325212	G	G	A	SNP	OS04G0178300	Intronic	NA	NA	LOC_Os04g09900	ent-kaurene synthase, chloroplast precursor, putative, expressed
Chr.4	5325091	5325091	T	T	A	SNP	OS04G0178300	Exonic	CDS	Silent	LOC_Os04g09900	ent-kaurene synthase, chloroplast precursor, putative, expressed
Chr.4	5324996	5324996	G	G	A	SNP	OS04G0178300	Intronic	NA	NA	LOC_Os04g09900	ent-kaurene synthase, chloroplast precursor, putative, expressed
Chr.4	5324677	5324677	G	G	A	SNP	OS04G0178300	Intronic	NA	NA	LOC_Os04g09900	ent-kaurene synthase, chloroplast precursor, putative, expressed
Chr.4	5324655	5324655	G	G	A	SNP	OS04G0178300	Intronic	NA	NA	LOC_Os04g09900	ent-kaurene synthase, chloroplast precursor, putative, expressed
Chr.4	5324082	5324082	T	T	A	SNP	OS04G0178300	Intronic	NA	NA	LOC_Os04g09900	ent-kaurene synthase, chloroplast precursor, putative, expressed
Chr.4	5323874	5323874	G	G	A	SNP	OS04G0178300	Intronic	NA	NA	LOC_Os04g09900	ent-kaurene synthase, chloroplast precursor, putative, expressed
Chr.4	5323362	5323362	G	G	A	SNP	OS04G0178300	Intronic	NA	NA	LOC_Os04g09900	ent-kaurene synthase, chloroplast precursor, putative, expressed
Chr.4	5322134	5322134	G	G	A	SNP	OS04G0178300	Exonic	CDS	Silent	LOC_Os04g09900	ent-kaurene synthase, chloroplast precursor, putative, expressed
Chr.4	5322014	5322014	C	C	A	SNP	OS04G0178300	Intronic	NA	NA	LOC_Os04g09900	ent-kaurene synthase, chloroplast precursor, putative, expressed
Chr.4	5321775	5321775	T	T	A	SNP	OS04G0178300	Intronic	NA	NA	LOC_Os04g09900	ent-kaurene synthase, chloroplast precursor, putative, expressed
Chr.4	5321717	5321717	T	T	A	SNP	OS04G0178300	Intronic	NA	NA	LOC_Os04g09900	ent-kaurene synthase, chloroplast precursor, putative, expressed
Chr.4	5318856	5318856	T	T	A	SNP	OS04G0178300	Exonic	CDS	NA	LOC_Os04g09900	ent-kaurene synthase, chloroplast precursor, putative, expressed
Chr.4	318650	318650	G	G	A	SNP	OS04G0104900	Intronic	NA	NA	LOC_Os04g01470	O-methyltransferase, putative, expressed
Chr.4	318623	318623	G	G	A	SNP	OS04G0104900	Intronic	NA	NA	LOC_Os04g01470	O-methyltransferase, putative, expressed
Chr.4	5324084	5324085	AT	AT	A	INDEL	OS04G0178300	Intronic	NA	NA	LOC_Os04g09900	ent-kaurene synthase, chloroplast precursor, putative, expressed
Chr.4	20144864	20144865	AT	AT	A	INDEL	OS04G0405900	Exonic	5UTR	NA	LOC_Os04g33310	expressed protein

Chr.4	24849377	24849378	AT	AT	A	INDEL	OS04G0496800	Intronic	NA	NA	LOC_Os04g41950	calcium-binding mitochondrial protein anon-60Da, putative, expressed
Chr.4	28304406	28304407	AT	AT	A	INDEL	OS04G0564700	Intronic	NA	NA	LOC_Os04g47700	expressed protein
Chr.4	24056234	24056235	AC	AC	A	INDEL	OS04G0480900	Intronic	NA	NA	LOC_Os04g40490	glycosyl hydrolase family 5 protein, putative, expressed
Chr.4	33180955	33180958	AATC	AATC	A	INDEL	OS04G0651000	Intronic	NA	NA	LOC_Os04g55740	peroxidase precursor, putative, expressed
Chr.4	316791	316791	C	C	A	SNP	OS04G0104900	Exonic	CDS	Silent	LOC_Os04g01470	O-methyltransferase, putative, expressed
Chr.4	360319	360319	C	C	A	SNP	OS04G0105400	Exonic	CDS	Silent	LOC_Os04g01520	expressed protein
Chr.4	485684	485684	G	G	A	SNP	OS04G0107900	Exonic	CDS	NA	LOC_Os04g01740	heat shock protein, putative, expressed
Chr.4	619420	619420	G	G	A	SNP	OS04G0110600	Intronic	NA	NA	LOC_Os04g02000	zinc finger family protein, putative, expressed
Chr.4	5318418	5318418	G	G	A	SNP	OS04G0178300	Exonic	5UTR	NA	LOC_Os04g09900	ent-kaurene synthase, chloroplast precursor, putative, expressed
Chr.4	5319890	5319890	G	G	A	SNP	OS04G0178300	Intronic	NA	NA	LOC_Os04g09900	ent-kaurene synthase, chloroplast precursor, putative, expressed
Chr.4	5321449	5321449	G	G	A	SNP	OS04G0178300	Intronic	NA	NA	LOC_Os04g09900	ent-kaurene synthase, chloroplast precursor, putative, expressed
Chr.4	5321454	5321454	G	G	A	SNP	OS04G0178300	Intronic	NA	NA	LOC_Os04g09900	ent-kaurene synthase, chloroplast precursor, putative, expressed
Chr.4	5321460	5321460	G	G	A	SNP	OS04G0178300	Intronic	NA	NA	LOC_Os04g09900	ent-kaurene synthase, chloroplast precursor, putative, expressed
Chr.4	5321478	5321478	G	G	A	SNP	OS04G0178300	Intronic	NA	NA	LOC_Os04g09900	ent-kaurene synthase, chloroplast precursor, putative, expressed
Chr.4	5321510	5321510	G	G	A	SNP	OS04G0178300	Intronic	NA	NA	LOC_Os04g09900	ent-kaurene synthase, chloroplast precursor, putative, expressed
Chr.4	5321563	5321563	G	G	A	SNP	OS04G0178300	Intronic	NA	NA	LOC_Os04g09900	ent-kaurene synthase, chloroplast precursor, putative, expressed
Chr.4	5321570	5321570	G	G	A	SNP	OS04G0178300	Intronic	NA	NA	LOC_Os04g09900	ent-kaurene synthase, chloroplast precursor, putative, expressed
Chr.4	5323451	5323451	G	G	A	SNP	OS04G0178300	Intronic	NA	NA	LOC_Os04g09900	ent-kaurene synthase, chloroplast precursor, putative, expressed
Chr.4	5324347	5324347	T	T	A	SNP	OS04G0178300	Intronic	NA	NA	LOC_Os04g09900	ent-kaurene synthase, chloroplast precursor, putative, expressed
Chr.4	5324484	5324484	T	T	A	SNP	OS04G0178300	Intronic	NA	NA	LOC_Os04g09900	ent-kaurene synthase, chloroplast precursor, putative, expressed
Chr.4	5324515	5324515	G	G	A	SNP	OS04G0178300	Intronic	NA	NA	LOC_Os04g09900	ent-kaurene synthase, chloroplast precursor, putative, expressed
Chr.4	5324975	5324975	G	G	A	SNP	OS04G0178300	Intronic	NA	NA	LOC_Os04g09900	ent-kaurene synthase, chloroplast precursor, putative, expressed
Chr.4	5325777	5325777	T	T	A	SNP	OS04G0178300	Intronic	NA	NA	LOC_Os04g09900	ent-kaurene synthase, chloroplast precursor, putative, expressed
Chr.4	5522589	5522589	G	G	A	SNP	OS04G0180900	Exonic	3UTR	NA	LOC_Os04g10214	expressed protein
Chr.4	6479738	6479738	G	G	A	SNP	OS04G0194500	Exonic	CDS	Silent	LOC_Os04g11820	white-brown complex homolog protein, putative, expressed
Chr.4	6480282	6480282	G	G	A	SNP	OS04G0194500	Intronic	NA	NA	LOC_Os04g11820	white-brown complex homolog protein, putative, expressed
Chr.4	7308175	7308175	G	G	A	SNP	OS04G0209200	Intronic	NA	NA	LOC_Os04g13210	multidrug resistance-associated protein, putative, expressed
Chr.4	7308649	7308649	C	C	A	SNP	OS04G0209200	Intronic	NA	NA	LOC_Os04g13210	multidrug resistance-associated protein, putative, expressed
Chr.4	7309663	7309663	G	G	A	SNP	OS04G0209200	Intronic	NA	NA	LOC_Os04g13210	multidrug resistance-associated protein, putative, expressed
Chr.4	11278711	11278711	G	G	A	SNP	OS04G0270100	Intronic	NA	NA	LOC_Os04g20220	elongation factor Tu, putative, expressed
Chr.4	13650884	13650884	G	G	A	SNP	OS04G0304200	Intronic	NA	NA	LOC_Os04g23890	AGC_PVPK_like_kin82y.10 - ACG kinases include homologs to PKA, PKG and PKC, expressed
Chr.4	13651130	13651130	G	G	A	SNP	OS04G0304200	Intronic	NA	NA	LOC_Os04g23890	AGC_PVPK_like_kin82y.10 - ACG kinases include homologs to PKA, PKG and PKC, expressed
Chr.4	16004156	16004156	T	T	A	SNP	OS04G0339400	Intronic	NA	NA	LOC_Os04g27060	oxidoreductase, aldo/keto reductase family protein, putative, expressed
Chr.4	20319692	20319692	G	G	A	SNP	OS04G0411400	Exonic	3UTR	NA	LOC_Os04g33570	RCN4 Centroradialis-like1 homologous to TFL1 gene; contains Pfam profile PF01161: Phosphatidylethanolamine-binding protein,
Chr.4	20319813	20319813	G	G	A	SNP	OS04G0411400	Exonic	CDS	Silent	LOC_Os04g33570	RCN4 Centroradialis-like1 homologous to TFL1 gene; contains Pfam profile PF01161: Phosphatidylethanolamine-binding protein,
Chr.4	20389893	20389893	T	T	A	SNP	OS04G0412500	Exonic	3UTR	NA	LOC_Os04g33660	bifunctional monodehydroascorbate reductase and carbonic anhydrasenectarin-3 precursor, putative, expressed
Chr.4	20423672	20423672	C	C	A	SNP	OS04G0413500	Exonic	CDS	Silent	LOC_Os04g33740	glycosyl hydrolases, putative, expressed
Chr.4	20424590	20424590	G	G	A	SNP	OS04G0413500	Intronic	NA	NA	LOC_Os04g33740	glycosyl hydrolases, putative, expressed
Chr.4	20424675	20424675	G	G	A	SNP	OS04G0413500	Intronic	NA	NA	LOC_Os04g33740	glycosyl hydrolases, putative, expressed
Chr.4	20424705	20424705	G	G	A	SNP	OS04G0413500	Intronic	NA	NA	LOC_Os04g33740	glycosyl hydrolases, putative, expressed
Chr.4	20424721	20424721	G	G	A	SNP	OS04G0413500	Intronic	NA	NA	LOC_Os04g33740	glycosyl hydrolases, putative, expressed
Chr.4	20519400	20519400	C	C	A	SNP	OS04G0415100	Intronic	NA	NA	LOC_Os04g33870	DUF623 domain containing protein, expressed
Chr.4	22017994	22017994	G	G	A	SNP	OS04G0442200	Exonic	5UTR	NA	LOC_Os04g36062	expressed protein
Chr.4	22258354	22258354	C	C	A	SNP	OS04G0446500	Exonic	5UTR	NA	LOC_Os04g36890	peptidyl-prolyl cis-trans isomerase, FKBP-type, putative, expressed
Chr.4	22624039	22624039	G	G	A	SNP	OS04G0453233	Intronic	NA	NA	LOC_Os04g38026	transporter family protein, putative, expressed
Chr.4	23142426	23142426	T	T	A	SNP	OS04G0463500	Exonic	CDS	Missense	LOC_Os04g38950	class I glutamine amidotransferase, putative, expressed
Chr.4	23142625	23142625	G	G	A	SNP	OS04G0463500	Exonic	CDS	Missense	LOC_Os04g38950	class I glutamine amidotransferase, putative, expressed
Chr.4	23422885	23422885	G	G	A	SNP	OS04G0469000	Exonic	CDS	Silent	LOC_Os04g39350	heavy metal associated domain containing protein, expressed
Chr.4	24055036	24055036	G	G	A	SNP	OS04G0480900	Exonic	CDS	Missense	LOC_Os04g40490	glycosyl hydrolase family 5 protein, putative, expressed
Chr.4	24302396	24302396	G	G	A	SNP	OS04G0486500	Intronic	NA	NA	LOC_Os04g40940	mitotic spindle checkpoint protein MAD2, putative, expressed
Chr.4	24355171	24355171	G	G	A	SNP	OS04G0487300	Exonic	CDS	Missense	LOC_Os04g41040	DNA-directed RNA polymerases I, II, and III subunit RPABC1, putative, expressed
Chr.4	24355056	24355056	C	C	A	SNP	OS04G0487300	Intronic	NA	NA	LOC_Os04g41040	DNA-directed RNA polymerases I, II, and III subunit RPABC1, putative, expressed
Chr.4	24858554	24858554	G	G	A	SNP	OS04G0497000	Exonic	CDS	Silent	LOC_Os04g41960	NADP-dependent oxidoreductase, putative, expressed
Chr.4	24858572	24858572	G	G	A	SNP	OS04G0497000	Exonic	CDS	Silent	LOC_Os04g41960	NADP-dependent oxidoreductase, putative, expressed
Chr.4	24859029	24859029	G	G	A	SNP	OS04G0497000	Exonic	CDS	Silent	LOC_Os04g41960	NADP-dependent oxidoreductase, putative, expressed
Chr.4	25194106	25194106	C	C	A	SNP	OS04G0504800	Exonic	3UTR	NA	LOC_Os04g42600	polyadenylate-binding protein, putative, expressed
Chr.4	25194154	25194154	G	G	A	SNP	OS04G0504800	Exonic	3UTR	NA	LOC_Os04g42600	polyadenylate-binding protein, putative, expressed
Chr.4	25196314	25196314	G	G	A	SNP	OS04G0504800	Intronic	NA	NA	LOC_Os04g42600	polyadenylate-binding protein, putative, expressed
Chr.4	26696910	26696910	G	G	A	SNP	OS04G0534100	Intronic	NA	NA	LOC_Os04g45160	DTA2, putative, expressed
Chr.4	27765440	27765440	C	C	A	SNP	OS04G0555000	Exonic	CDS	Missense	LOC_Os04g46860	scarecrow, putative, expressed
Chr.4	27765467	27765467	G	G	A	SNP	OS04G0555000	Exonic	CDS	Missense	LOC_Os04g46860	scarecrow, putative, expressed
Chr.4	27765836	27765836	G	G	A	SNP	OS04G0555000	Exonic	CDS	Missense	LOC_Os04g46860	scarecrow, putative, expressed
Chr.4	27784150	27784150	G	G	A	SNP	OS04G0555300	Exonic	CDS	NA	LOC_Os04g46880	transporter, major facilitator family, putative, expressed
Chr.4	27784425	27784425	G	G	A	SNP	OS04G0555300	Exonic	CDS	NA	LOC_Os04g46880	transporter, major facilitator family, putative, expressed
Chr.4	27849125	27849125	G	G	A	SNP	OS04G0556500	Exonic	CDS	Silent	LOC_Os04g46980	cis-zeatin O-glucosyltransferase, putative, expressed
Chr.4	27849844	27849844	C	C	A	SNP	OS04G0556500	Exonic	CDS	Missense	LOC_Os04g46980	cis-zeatin O-glucosyltransferase, putative, expressed
Chr.4	29042996	29042996	G	G	A	SNP	OS04G0576500	Exonic	5UTR	NA	LOC_Os04g48710	expressed protein
Chr.4	29045925	29045925	C	C	A	SNP	OS04G0576500	Exonic	CDS	Missense	LOC_Os04g48710	expressed protein
Chr.4	29132957	29132957	G	G	A	SNP	OS04G0578000	Exonic	CDS	Silent	LOC_Os04g48850	aminotransferase, classes I and II, domain containing protein, expressed
Chr.4	29522809	29522809	G	G	A	SNP	OS04G0584500	Exonic	CDS	Silent	LOC_Os04g49500	U-box domain-containing protein, putative, expressed
Chr.4	29523064	29523064	G	G	A	SNP	OS04G0584500	Exonic	CDS	Silent	LOC_Os04g49500	U-box domain-containing protein, putative, expressed
Chr.4	29522465	29522465	G	G	A	SNP	OS04G0584500	Exonic	CDS	Missense	LOC_Os04g49500	U-box domain-containing protein, putative, expressed
Chr.4	29562992	29562992	G	G	A	SNP	OS04G0585100	Intronic	NA	NA	LOC_Os04g49560	zinc finger family protein, putative, expressed
Chr.4	30080184	30080184	G	G	A	SNP	OS04G0596300	Exonic	CDS	Silent	LOC_Os04g50860	expressed protein
Chr.4	30554119	30554119	C	C	A	SNP	OS04G0605300	Intronic	NA	NA	LOC_Os04g51580	leucine rich repeat containing protein, expressed
Chr.4	30555455	30555455	T	T	A	SNP	OS04G0605300	Intronic	NA	NA	LOC_Os04g51580	leucine rich repeat containing protein, expressed
Chr.4	30939192	30939192	G	G	A	SNP	OS04G0610400	Exonic	5UTR	NA	LOC_Os04g52090	AP2 domain containing protein, expressed
Chr.4	30939351	30939351	C	C	A	SNP	OS04G0610400	Exonic	CDS	Missense	LOC_Os04g52090	AP2 domain containing protein, expressed
Chr.4	31239297	31239297	G	G	A	SNP	OS04G0615700	Exonic	CDS	Missense	LOC_Os04g52540	retrotransposon protein, putative, unclassified, expressed
Chr.4	31240160	31240160	G	G	A	SNP	OS04G0615700	Exonic	CDS	Missense	LOC_Os04g52540	retrotransposon protein, putative, unclassified, expressed
Chr.4	31440061	31440061	G	G	A	SNP	OS04G0619000	Exonic	3UTR	NA	LOC_Os04g52810	no apical meristem protein, putative, expressed
Chr.4	31440150	31440150	G	G	A	SNP	OS04G0619000	Exonic	3UTR	NA	LOC_Os04g52810	no apical meristem protein, putative, expressed
Chr.4	31440223	31440223	G	G	A	SNP	OS04G0619000	Exonic	3UTR	NA	LOC_Os04g52810	no apical meristem protein, putative, expressed
Chr.4	31440977	31440977	C	C	A	SNP	OS04G0619000	Intronic	NA	NA	LOC_Os04g52810	no apical meristem protein, putative, expressed
Chr.4	32585638	32585638	G	G	A	SNP	OS04G0640600	Exonic	5UTR	NA	LOC_Os04g54800	shikimate kinase, putative, expressed

Chr.4	33164016	33164016	G	G	A	SNP	OS04G0650700	Exonic	CDS	Silent	LOC_Os04g55710	transposon protein, putative, unclassified, expressed
Chr.4	33164088	33164088	C	C	A	SNP	OS04G0650700	Exonic	CDS	Silent	LOC_Os04g55710	transposon protein, putative, unclassified, expressed
Chr.4	33180589	33180589	G	G	A	SNP	OS04G0651000	Exonic	CDS	Silent	LOC_Os04g55740	peroxidase precursor, putative, expressed
Chr.4	35450887	35450887	G	G	A	SNP	OS04G0692600	Exonic	3UTR	NA	LOC_Os04g59610	expressed protein
Chr.4	318593	318601	AATGAATCT	AATGAATCT	A	INDEL	OS04G0104900	Intronic	NA	NA	LOC_Os04g01470	O-methyltransferase, putative, expressed
Chr.4	5324341	5324346	ATACAG	ATACAG	A	INDEL	OS04G0178300	Intronic	NA	NA	LOC_Os04g09900	ent-kaurene synthase, chloroplast precursor, putative, expressed
Chr.4	5324385	5324386	AC	AC	A	INDEL	OS04G0178300	Intronic	NA	NA	LOC_Os04g09900	ent-kaurene synthase, chloroplast precursor, putative, expressed
Chr.4	6475428	6475447	AGTTGGTGCAATTGCCCTCT	AGTTGGTGCAATTGCCCTCT	A	INDEL	OS04G0194500	Exonic	3UTR	NA	LOC_Os04g11820	white-brown complex homolog protein, putative, expressed
Chr.4	6477559	6477566	ATATGACT	ATATGACT	A	INDEL	OS04G0194500	Intronic	NA	NA	LOC_Os04g11820	white-brown complex homolog protein, putative, expressed
Chr.4	6480737	6480738	AG	AG	A	INDEL	OS04G0194500	Intronic	NA	NA	LOC_Os04g11820	white-brown complex homolog protein, putative, expressed
Chr.4	6481401	6481402	AG	AG	A	INDEL	OS04G0194500	Intronic	NA	NA	LOC_Os04g11820	white-brown complex homolog protein, putative, expressed
Chr.4	13651930	13651931	AT	AT	A	INDEL	OS04G0304200	Intronic	NA	NA	LOC_Os04g23890	AGC_PVPK_like_kin82y.10 - ACG kinases include homologs to PKA, PKG and PKC, expressed
Chr.4	20935991	20935994	AAAG	AAAG	A	INDEL	OS04G0423400	Exonic	5UTR	NA	LOC_Os04g34600	abscisic stress-ripening, putative, expressed
Chr.4	22262471	22262478	ATACTCCG	ATACTCCG	A	INDEL	OS04G0446500	Exonic	3UTR	NA	LOC_Os04g36890	peptidyl-prolyl cis-trans isomerase, FKBP-type, putative, expressed
Chr.4	24302021	24302037	AAATCAATCAATCAATC	AAATCAATCAATCAATC	A	INDEL	OS04G0486500	Intronic	NA	NA	LOC_Os04g40940	mitotic spindle checkpoint protein MAD2, putative, expressed
Chr.4	24327168	24327169	AC	AC	A	INDEL	OS04G0486950	Intronic	NA	NA	LOC_Os04g40990	malate synthase, glyoxysomal, putative, expressed
Chr.4	27191819	27191820	AC	AC	A	INDEL	OS04G0542900	Intronic	NA	NA	LOC_Os04g45910	placental protein 11 precursor, putative, expressed
Chr.4	27642900	27642903	ATTT	ATTT	A	INDEL	OS04G0551800	Intronic	NA	NA	LOC_Os04g46620	T-complex protein, putative, expressed
Chr.4	28343203	28343231	AGCGCCGCCGCCGCGGCCGCGCACAGCCG	AGCGCCGCCGCCGCGGCCGCGCACAGCCG	A	INDEL	OS04G0565500	Exonic	5UTR	NA	LOC_Os04g47780	transmembrane amino acid transporter protein, putative, expressed
Chr.4	29317539	29317541	AGC	AGC	A	INDEL	OS04G0580800	Exonic	3UTR	NA	LOC_Os04g49160	zinc finger, C3HC4 type domain containing protein, expressed
Chr.4	29524993	29524995	ACC	ACC	A	INDEL	OS04G0584500	Intronic	NA	NA	LOC_Os04g49500	U-box domain-containing protein, putative, expressed
Chr.4	30940035	30940036	AG	AG	A	INDEL	OS04G0610400	Exonic	3UTR	NA	LOC_Os04g52090	AP2 domain containing protein, expressed
Chr.4	31176728	31176730	ATC	ATC	A	INDEL	OS04G0614600	Exonic	5UTR	NA	LOC_Os04g52450	aminotransferase, putative, expressed
Chr.4	31181943	31181949	AAAAAAC	AAAAAAC	A	INDEL	OS04G0614600	Intronic	NA	NA	LOC_Os04g52450	aminotransferase, putative, expressed
Chr.4	31439610	31439613	ATCG	ATCG	A	INDEL	OS04G0619000	Exonic	3UTR	NA	LOC_Os04g52810	no apical meristem protein, putative, expressed
Chr.4	31439907	31439913	ATAGCTC	ATAGCTC	A	INDEL	OS04G0619000	Exonic	3UTR	NA	LOC_Os04g52810	no apical meristem protein, putative, expressed
Chr.4	31443392	31443404	AAGACACAAAGAG	AAGACACAAAGAG	A	INDEL	OS04G0619000	Exonic	5UTR	NA	LOC_Os04g52810	no apical meristem protein, putative, expressed
Chr.4	31441382	31441383	AT	AT	A	INDEL	OS04G0619000	Intronic	NA	NA	LOC_Os04g52810	no apical meristem protein, putative, expressed
Chr.4	31671834	31671836	AAG	AAG	A	INDEL	OS04G0623300	Exonic	5UTR	NA	LOC_Os04g53190	CPuORF12 - conserved peptide uORF-containing transcript, expressed
Chr.4	33126953	33126956	AGAG	AGAG	A	INDEL	OS04G0650000	Exonic	5UTR	NA	LOC_Os04g55650	oryzain alpha chain precursor, putative, expressed
Chr.4	33532016	33532026	ATGAAAAAATT	ATGAAAAAATT	A	INDEL	OS04G0657500	Intronic	NA	NA	LOC_Os04g56240	lipase, putative, expressed
Chr.4	33973027	33973030	AATT	AATT	A	INDEL	OS04G0665600	Exonic	3UTR	NA	LOC_Os04g56990	Myb-like DNA-binding domain containing protein, putative, expressed
Chr.3	3525695	3525695	T	T	A	SNP	OS03G0165400	Exonic	CDS	Missense	LOC_Os03g06940	beta-galactosidase, putative, expressed
Chr.3	3562476	3562476	T	T	A	SNP	OS03G0166000	Intronic	NA	NA	LOC_Os03g06980	nucleic acid binding protein, putative, expressed
Chr.3	8789688	8789688	T	T	A	SNP	OS03G0265900	Exonic	CDS	Missense	LOC_Os03g15920	expressed protein
Chr.3	10041790	10041790	T	T	A	SNP	OS03G0289800	Intronic	NA	NA	LOC_Os03g18030	leucoanthocyanidin dioxygenase, putative, expressed
Chr.3	23371868	23371868	T	T	A	SNP	OS03G0616700	Intronic	NA	NA	LOC_Os03g42050	exportin-7-A, putative, expressed
Chr.3	24599052	24599052	T	T	A	SNP	OS03G0639800	Intronic	NA	NA	LOC_Os03g43860	SNF7 domain containing protein, putative, expressed
Chr.3	24705012	24705012	T	T	A	SNP	OS03G0641300	Exonic	3UTR	NA	LOC_Os03g43990	BTBN8 - Bric-a-Brac, Tramtrack, Broad Complex BTB domain with non-phototropic hypocotyl 3 NPH3 and coiled-coil domains, ex
Chr.3	24705313	24705313	T	T	A	SNP	OS03G0641300	Exonic	3UTR	NA	LOC_Os03g43990	BTBN8 - Bric-a-Brac, Tramtrack, Broad Complex BTB domain with non-phototropic hypocotyl 3 NPH3 and coiled-coil domains, ex
Chr.3	24705420	24705420	T	T	A	SNP	OS03G0641300	Exonic	3UTR	NA	LOC_Os03g43990	BTBN8 - Bric-a-Brac, Tramtrack, Broad Complex BTB domain with non-phototropic hypocotyl 3 NPH3 and coiled-coil domains, ex
Chr.3	24705542	24705542	T	T	A	SNP	OS03G0641300	Exonic	3UTR	NA	LOC_Os03g43990	BTBN8 - Bric-a-Brac, Tramtrack, Broad Complex BTB domain with non-phototropic hypocotyl 3 NPH3 and coiled-coil domains, ex
Chr.3	24705583	24705583	T	T	A	SNP	OS03G0641300	Exonic	3UTR	NA	LOC_Os03g43990	BTBN8 - Bric-a-Brac, Tramtrack, Broad Complex BTB domain with non-phototropic hypocotyl 3 NPH3 and coiled-coil domains, ex
Chr.3	24816622	24816622	T	T	A	SNP	OS03G0643300	Exonic	CDS	Silent	LOC_Os03g44150	aminotransferase, putative, expressed
Chr.3	24814815	24814815	T	T	A	SNP	OS03G0643300	Intronic	NA	NA	LOC_Os03g44150	aminotransferase, putative, expressed
Chr.3	24815645	24815645	T	T	A	SNP	OS03G0643300	Intronic	NA	NA	LOC_Os03g44150	aminotransferase, putative, expressed
Chr.3	24817278	24817278	T	T	A	SNP	OS03G0643300	Intronic	NA	NA	LOC_Os03g44150	aminotransferase, putative, expressed
Chr.3	24817412	24817412	T	T	A	SNP	OS03G0643300	Intronic	NA	NA	LOC_Os03g44150	aminotransferase, putative, expressed
Chr.3	26384499	26384499	T	T	A	SNP	OS03G0669000	Intronic	NA	NA	LOC_Os03g46610	DEAD-box ATP-dependent RNA helicase, putative, expressed
Chr.3	27497603	27497603	T	T	A	SNP	OS03G0689100	Intronic	NA	NA	LOC_Os03g48300	histidine acid phosphatase, putative, expressed
Chr.3	27497604	27497604	T	T	A	SNP	OS03G0689100	Intronic	NA	NA	LOC_Os03g48300	histidine acid phosphatase, putative, expressed
Chr.3	27739800	27739800	T	T	A	SNP	OS03G0693000	Intronic	NA	NA	LOC_Os03g48660	DUF1336 domain containing protein, expressed
Chr.3	28241673	28241673	T	T	A	SNP	OS03G0703000	Intronic	NA	NA	LOC_Os03g49600	Os3bglu7 - beta-glucosidase, exo-beta-glucanse, expressed
Chr.3	28244142	28244142	T	T	A	SNP	OS03G0703000	Intronic	NA	NA	LOC_Os03g49600	Os3bglu7 - beta-glucosidase, exo-beta-glucanse, expressed
Chr.3	29143281	29143281	T	T	A	SNP	OS03G0719300	Intronic	NA	NA	LOC_Os03g51000	3,4-dihydroxy-2-butanone kinase, putative, expressed
Chr.3	29562637	29562637	T	T	A	SNP	OS03G0726500	Intronic	NA	NA	LOC_Os03g51650	membrane protein, putative, expressed
Chr.3	30063219	30063219	T	T	A	SNP	OS03G0733800	Intronic	NA	NA	LOC_Os03g52340	endoplasmic oxidoreductin-1 precursor, putative, expressed
Chr.3	32625921	32625921	T	T	A	SNP	OS03G0786000	Intronic	NA	NA	LOC_Os03g57210	glycine-rich protein, putative, expressed
Chr.3	34908238	34908238	T	T	A	SNP	OS03G0831100	Intronic	NA	NA	LOC_Os03g61560	expressed protein
Chr.3	32181878	32181878	G	G	A	SNP	OS03G0776000	Exonic	3UTR	NA	LOC_Os03g56460	glucose-6-phosphate isomerase, putative, expressed
Chr.3	541237	541237	G	G	A	SNP	OS03G0109300	Intronic	NA	NA	LOC_Os03g01880	possible lysine decarboxylase domain containing protein, expressed
Chr.3	1236846	1236846	G	G	A	SNP	OS03G0122300	Intronic	NA	NA	LOC_Os03g03034	flavonol synthase/flavanone 3-hydroxylase, putative, expressed
Chr.3	1236865	1236865	G	G	A	SNP	OS03G0122300	Intronic	NA	NA	LOC_Os03g03034	flavonol synthase/flavanone 3-hydroxylase, putative, expressed
Chr.3	1238608	1238608	G	G	A	SNP	OS03G0122300	Intronic	NA	NA	LOC_Os03g03034	flavonol synthase/flavanone 3-hydroxylase, putative, expressed
Chr.3	1243405	1243405	G	G	A	SNP	OS03G0122300	Intronic	NA	NA	LOC_Os03g03034	flavonol synthase/flavanone 3-hydroxylase, putative, expressed
Chr.3	1243406	1243406	G	G	A	SNP	OS03G0122300	Intronic	NA	NA	LOC_Os03g03034	flavonol synthase/flavanone 3-hydroxylase, putative, expressed
Chr.3	1243924	1243924	G	G	A	SNP	OS03G0122300	Intronic	NA	NA	LOC_Os03g03034	flavonol synthase/flavanone 3-hydroxylase, putative, expressed
Chr.3	1243937	1243937	G	G	A	SNP	OS03G0122300	Intronic	NA	NA	LOC_Os03g03034	flavonol synthase/flavanone 3-hydroxylase, putative, expressed
Chr.3	1244072	1244072	G	G	A	SNP	OS03G0122300	Intronic	NA	NA	LOC_Os03g03034	flavonol synthase/flavanone 3-hydroxylase, putative, expressed
Chr.3	1716982	1716982	G	G	A	SNP	OS03G0130400	Intronic	NA	NA	LOC_Os03g03820	adenylate kinase, putative, expressed
Chr.3	1789333	1789333	G	G	A	SNP	OS03G0131200	Exonic	CDS	Silent	LOC_Os03g03910	catalase domain containing protein, expressed
Chr.3	1876389	1876389	G	G	A	SNP	OS03G0133300	Intronic	NA	NA	LOC_Os03g04100	expressed protein
Chr.3	2751957	2751957	G	G	A	SNP	OS03G0149300	Intronic	NA	NA	LOC_Os03g05530	nodulin, putative, expressed
Chr.3	2752114	2752114	G	G	A	SNP	OS03G0149300	Intronic	NA	NA	LOC_Os03g05530	nodulin, putative, expressed
Chr.3	4418460	4418460	G	G	A	SNP	OS03G0184100	Exonic	CDS	Silent	LOC_Os03g08580	expressed protein
Chr.3	4827182	4827182	G	G	A	SNP	OS03G0192700	Exonic	CDS	Silent	LOC_Os03g09250	inositol-3-phosphate synthase, putative, expressed
Chr.3	6459910	6459910	G	G	A	SNP	OS03G0223400	Intronic	NA	NA	LOC_Os03g12290	glutamine synthetase, catalytic domain containing protein, expressed
Chr.3	7332504	7332504	G	G	A	SNP	OS03G0238800	Intronic	NA	NA	LOC_Os03g13560	hydroxyproline-rich glycoprotein family protein, putative, expressed
Chr.3	8706231	8706231	G	G	A	SNP	OS03G0264400	Intronic	NA	NA	LOC_Os03g15780	anthranilate synthase component I-1, chloroplast precursor, putative, expressed
Chr.3	9014282	9014282	G	G	A	SNP	OS03G0269900	Intronic	NA	NA	LOC_Os03g16334	fringe-related protein, putative, expressed
Chr.3	10997003	10997003	G	G	A	SNP	OS03G0308800	Intronic	NA	NA	LOC_Os03g19560	tetratricopeptide repeat domain containing protein, expressed
Chr.3	12351015	12351015	G	G	A	SNP	OS03G0334100	Intronic	NA	NA	LOC_Os03g21630	PRC-barrel domain protein, putative, expressed
Chr.3	12588441	12588441	G	G	A	SNP	OS03G0338400	Intronic	NA	NA	LOC_Os03g21970	basic helix-loop-helix domain containing protein, expressed
Chr.3	15669582	15669582	G	G	A	SNP	OS03G0391100	Intronic	NA	NA	LOC_Os03g27360	RING-H2 finger protein ATL5H precursor, putative, expressed
Chr.3	16283199	16283199	G	G	A	SNP	OS03G0401100	Exonic	5UTR	NA	LOC_Os03g28300	protein kinase domain containing protein, expressed

Chr.3	16288139	16288139	G	G	A	SNP	OS03G0401100	Intronic	NA	NA	LOC_Os03g28300	protein kinase domain containing protein, expressed
Chr.3	16288485	16288485	G	G	A	SNP	OS03G0401100	Intronic	NA	NA	LOC_Os03g28300	protein kinase domain containing protein, expressed
Chr.3	16288628	16288628	G	G	A	SNP	OS03G0401100	Intronic	NA	NA	LOC_Os03g28300	protein kinase domain containing protein, expressed
Chr.3	16595731	16595731	G	G	A	SNP	OS03G0405500	Exonic	3UTR	NA	LOC_Os03g29190	PDI, putative, expressed
Chr.3	16591535	16591535	G	G	A	SNP	OS03G0405500	Intronic	NA	NA	LOC_Os03g29190	PDI, putative, expressed
Chr.3	16592238	16592238	G	G	A	SNP	OS03G0405500	Intronic	NA	NA	LOC_Os03g29190	PDI, putative, expressed
Chr.3	16593454	16593454	G	G	A	SNP	OS03G0405500	Intronic	NA	NA	LOC_Os03g29190	PDI, putative, expressed
Chr.3	21088019	21088019	G	G	A	SNP	OS03G0576700	Intronic	NA	NA	LOC_Os03g37970	ribosomal protein L13, putative, expressed
Chr.3	22085860	22085860	G	G	A	SNP	OS03G0594100	Exonic	3UTR	NA	LOC_Os03g39690	cytochrome P450, putative, expressed
Chr.3	22085920	22085920	G	G	A	SNP	OS03G0594100	Exonic	3UTR	NA	LOC_Os03g39690	cytochrome P450, putative, expressed
Chr.3	22086158	22086158	G	G	A	SNP	OS03G0594100	Exonic	3UTR	NA	LOC_Os03g39690	cytochrome P450, putative, expressed
Chr.3	22337473	22337473	G	G	A	SNP	OS03G0598800	Intronic	NA	NA	LOC_Os03g40180	60S ribosomal protein L15, putative, expressed
Chr.3	22616252	22616252	G	G	A	SNP	OS03G0603600	Intronic	NA	NA	LOC_Os03g40670	glycerophosphoryl diester phosphodiesterase family protein, putative, expressed
Chr.3	24816989	24816989	G	G	A	SNP	OS03G0643300	Exonic	CDS	Silent	LOC_Os03g44150	aminotransferase, putative, expressed
Chr.3	24815959	24815959	G	G	A	SNP	OS03G0643300	Exonic	CDS	Missense	LOC_Os03g44150	aminotransferase, putative, expressed
Chr.3	24814539	24814539	G	G	A	SNP	OS03G0643300	Intronic	NA	NA	LOC_Os03g44150	aminotransferase, putative, expressed
Chr.3	24814860	24814860	G	G	A	SNP	OS03G0643300	Intronic	NA	NA	LOC_Os03g44150	aminotransferase, putative, expressed
Chr.3	24815448	24815448	G	G	A	SNP	OS03G0643300	Intronic	NA	NA	LOC_Os03g44150	aminotransferase, putative, expressed
Chr.3	24815772	24815772	G	G	A	SNP	OS03G0643300	Intronic	NA	NA	LOC_Os03g44150	aminotransferase, putative, expressed
Chr.3	24816235	24816235	G	G	A	SNP	OS03G0643300	Intronic	NA	NA	LOC_Os03g44150	aminotransferase, putative, expressed
Chr.3	24817014	24817014	G	G	A	SNP	OS03G0643300	Intronic	NA	NA	LOC_Os03g44150	aminotransferase, putative, expressed
Chr.3	24817093	24817093	G	G	A	SNP	OS03G0643300	Intronic	NA	NA	LOC_Os03g44150	aminotransferase, putative, expressed
Chr.3	25065270	25065270	G	G	A	SNP	OS03G0647500	Intronic	NA	NA	LOC_Os03g44530	GTPase of unknown function domain containing protein, putative, expressed
Chr.3	25103166	25103166	G	G	A	SNP	OS03G0648100	Exonic	CDS	Silent	LOC_Os03g44580	DNA binding protein, putative, expressed
Chr.3	26380854	26380854	G	G	A	SNP	OS03G0669000	Exonic	3UTR	NA	LOC_Os03g46610	DEAD-box ATP-dependent RNA helicase, putative, expressed
Chr.3	26383683	26383683	G	G	A	SNP	OS03G0669000	Exonic	3UTR	NA	LOC_Os03g46610	DEAD-box ATP-dependent RNA helicase, putative, expressed
Chr.3	26381895	26381895	G	G	A	SNP	OS03G0669000	Intronic	NA	NA	LOC_Os03g46610	DEAD-box ATP-dependent RNA helicase, putative, expressed
Chr.3	26396807	26396807	G	G	A	SNP	OS03G0669100	Exonic	CDS	Missense	LOC_Os03g46640	deoxyuridine 5-triphosphate nucleotidohydrolase, putative, expressed
Chr.3	26952594	26952594	G	G	A	SNP	OS03G0679700	Intronic	NA	NA	LOC_Os03g47610	thiamine biosynthesis protein thiC, putative, expressed
Chr.3	27290941	27290941	G	G	A	SNP	OS03G0684400	Intronic	NA	NA	LOC_Os03g48000	CorA-like magnesium transporter protein, putative, expressed
Chr.3	27336417	27336417	G	G	A	SNP	OS03G0685300	Intronic	NA	NA	LOC_Os03g48060	class I glutamine amidotransferase, putative, expressed
Chr.3	27497072	27497072	G	G	A	SNP	OS03G0689100	Intronic	NA	NA	LOC_Os03g48300	histidine acid phosphatase, putative, expressed
Chr.3	27501848	27501848	G	G	A	SNP	OS03G0689100	Intronic	NA	NA	LOC_Os03g48300	histidine acid phosphatase, putative, expressed
Chr.3	27740291	27740291	G	G	A	SNP	OS03G0693000	Exonic	CDS	Missense	LOC_Os03g48660	DUF1336 domain containing protein, expressed
Chr.3	28241327	28241327	G	G	A	SNP	OS03G0703000	Intronic	NA	NA	LOC_Os03g49600	Os3bglu7 - beta-glucosidase, exo-beta-glucanse, expressed
Chr.3	28243027	28243027	G	G	A	SNP	OS03G0703000	Intronic	NA	NA	LOC_Os03g49600	Os3bglu7 - beta-glucosidase, exo-beta-glucanse, expressed
Chr.3	28594822	28594822	G	G	A	SNP	OS03G0709000	Intronic	NA	NA	LOC_Os03g50130	microsomal glutathione S-transferase 3, putative, expressed
Chr.3	28595606	28595606	G	G	A	SNP	OS03G0709000	Intronic	NA	NA	LOC_Os03g50130	microsomal glutathione S-transferase 3, putative, expressed
Chr.3	29017168	29017168	G	G	A	SNP	OS03G0717000	Intronic	NA	NA	LOC_Os03g50810	receptor protein kinase TMK1 precursor, putative, expressed
Chr.3	29116042	29116042	G	G	A	SNP	OS03G0719000	Exonic	CDS	NA	LOC_Os03g50970	retrotransposon protein, putative, Ty1-copia subclass, expressed
Chr.3	29139750	29139750	G	G	A	SNP	OS03G0719300	Exonic	CDS	Missense	LOC_Os03g51000	3,4-dihydroxy-2-butanone kinase, putative, expressed
Chr.3	29140027	29140027	G	G	A	SNP	OS03G0719300	Intronic	NA	NA	LOC_Os03g51000	3,4-dihydroxy-2-butanone kinase, putative, expressed
Chr.3	30064722	30064722	G	G	A	SNP	OS03G0733800	Exonic	CDS	Silent	LOC_Os03g52340	endoplasmic oxidoreductin-1 precursor, putative, expressed
Chr.3	30064054	30064054	G	G	A	SNP	OS03G0733800	Exonic	CDS	Missense	LOC_Os03g52340	endoplasmic oxidoreductin-1 precursor, putative, expressed
Chr.3	30075143	30075143	G	G	A	SNP	OS03G0734100	Intronic	NA	NA	LOC_Os03g52370	PIII4 - Proteinase inhibitor II family protein precursor, expressed
Chr.3	30534078	30534078	G	G	A	SNP	OS03G0743900	Intronic	NA	NA	LOC_Os03g53230	bifunctional 3-phosphoadenosine 5-phosphosulfate synthetase, putative, expressed
Chr.3	32177559	32177559	G	G	A	SNP	OS03G0776000	Exonic	CDS	Silent	LOC_Os03g56460	glucose-6-phosphate isomerase, putative, expressed
Chr.3	32378424	32378424	G	G	A	SNP	OS03G0780800	Exonic	3UTR	NA	LOC_Os03g56820	fatty acid hydroxylase, putative, expressed
Chr.3	32667448	32667448	G	G	A	SNP	OS03G0786700	Intronic	NA	NA	LOC_Os03g57280	N-dimethylguanosine tRNA methyltransferase, putative, expressed
Chr.3	32931252	32931252	G	G	A	SNP	OS03G0791800	Intronic	NA	NA	LOC_Os03g57790	ubiquitin-conjugating enzyme, putative, expressed
Chr.3	33215655	33215655	G	G	A	SNP	OS03G0797400	Intronic	NA	NA	LOC_Os03g58300	indole-3-glycerol phosphate lyase, chloroplast precursor, putative, expressed
Chr.3	33216114	33216114	G	G	A	SNP	OS03G0797400	Intronic	NA	NA	LOC_Os03g58300	indole-3-glycerol phosphate lyase, chloroplast precursor, putative, expressed
Chr.3	33349289	33349289	G	G	A	SNP	OS03G0799900	Exonic	3UTR	NA	LOC_Os03g58570	WD domain, G-beta repeat domain containing protein, expressed
Chr.3	33349586	33349586	G	G	A	SNP	OS03G0799900	Exonic	3UTR	NA	LOC_Os03g58570	WD domain, G-beta repeat domain containing protein, expressed
Chr.3	33347784	33347784	G	G	A	SNP	OS03G0799900	Exonic	CDS	Missense	LOC_Os03g58570	WD domain, G-beta repeat domain containing protein, expressed
Chr.3	33347516	33347516	G	G	A	SNP	OS03G0799900	Intronic	NA	NA	LOC_Os03g58570	WD domain, G-beta repeat domain containing protein, expressed
Chr.3	33502177	33502177	G	G	A	SNP	OS03G0802900	Intronic	NA	NA	LOC_Os03g58830	BEE 1, putative, expressed
Chr.3	33503113	33503113	G	G	A	SNP	OS03G0802900	Intronic	NA	NA	LOC_Os03g58830	BEE 1, putative, expressed
Chr.3	33504450	33504450	G	G	A	SNP	OS03G0802900	Intronic	NA	NA	LOC_Os03g58830	BEE 1, putative, expressed
Chr.3	33697975	33697975	G	G	A	SNP	OS03G0806600	Intronic	NA	NA	LOC_Os03g59180	expressed protein
Chr.3	35662955	35662955	G	G	A	SNP	OS03G0848100	Exonic	3UTR	NA	LOC_Os03g63074	Ser/Thr protein phosphatase family protein, expressed
Chr.3	16289691	16289691	C	C	A	SNP	OS03G0401100	Intronic	NA	NA	LOC_Os03g28300	protein kinase domain containing protein, expressed
Chr.3	28262213	28262213	C	C	A	SNP	OS03G0703300	Intronic	NA	NA	LOC_Os03g49630	expressed protein
Chr.3	1236300	1236300	C	C	A	SNP	OS03G0122300	Intronic	NA	NA	LOC_Os03g03034	flavonol synthase/flavanone 3-hydroxylase, putative, expressed
Chr.3	1236522	1236522	C	C	A	SNP	OS03G0122300	Intronic	NA	NA	LOC_Os03g03034	flavonol synthase/flavanone 3-hydroxylase, putative, expressed
Chr.3	1238257	1238257	C	C	A	SNP	OS03G0122300	Intronic	NA	NA	LOC_Os03g03034	flavonol synthase/flavanone 3-hydroxylase, putative, expressed
Chr.3	3562175	3562175	C	C	A	SNP	OS03G0166000	Intronic	NA	NA	LOC_Os03g06980	nucleic acid binding protein, putative, expressed
Chr.3	3562479	3562479	C	C	A	SNP	OS03G0166000	Intronic	NA	NA	LOC_Os03g06980	nucleic acid binding protein, putative, expressed
Chr.3	4827107	4827107	C	C	A	SNP	OS03G0192700	Intronic	NA	NA	LOC_Os03g09250	inositol-3-phosphate synthase, putative, expressed
Chr.3	9014105	9014105	C	C	A	SNP	OS03G0269900	Intronic	NA	NA	LOC_Os03g16334	fringe-related protein, putative, expressed
Chr.3	15669684	15669684	C	C	A	SNP	OS03G0391100	Intronic	NA	NA	LOC_Os03g27360	RING-H2 finger protein ATL5H precursor, putative, expressed
Chr.3	16285870	16285870	C	C	A	SNP	OS03G0401100	Exonic	3UTR	NA	LOC_Os03g28300	protein kinase domain containing protein, expressed
Chr.3	16592248	16592248	C	C	A	SNP	OS03G0405500	Intronic	NA	NA	LOC_Os03g29190	PDI, putative, expressed
Chr.3	20795235	20795235	C	C	A	SNP	OS03G0571900	Intronic	NA	NA	LOC_Os03g37490	MATE efflux family protein, putative, expressed
Chr.3	21104398	21104398	C	C	A	SNP	OS03G0577100	Intronic	NA	NA	LOC_Os03g38010	nuf2 family protein, expressed
Chr.3	24451257	24451257	C	C	A	SNP	OS03G0638200	Exonic	CDS	Missense	LOC_Os03g43720	transporter family protein, putative, expressed
Chr.3	24705149	24705149	C	C	A	SNP	OS03G0641300	Exonic	3UTR	NA	LOC_Os03g43990	BTBN8 - Bric-a-Brac, Tramtrack, Broad Complex BTB domain with non-phototropic hypocotyl 3 NPH3 and coiled-coil domains, ex
Chr.3	24705270	24705270	C	C	A	SNP	OS03G0641300	Exonic	3UTR	NA	LOC_Os03g43990	BTBN8 - Bric-a-Brac, Tramtrack, Broad Complex BTB domain with non-phototropic hypocotyl 3 NPH3 and coiled-coil domains, ex
Chr.3	24705504	24705504	C	C	A	SNP	OS03G0641300	Exonic	3UTR	NA	LOC_Os03g43990	BTBN8 - Bric-a-Brac, Tramtrack, Broad Complex BTB domain with non-phototropic hypocotyl 3 NPH3 and coiled-coil domains, ex
Chr.3	24816697	24816697	C	C	A	SNP	OS03G0643300	Exonic	CDS	Missense	LOC_Os03g44150	aminotransferase, putative, expressed
Chr.3	24815115	24815115	C	C	A	SNP	OS03G0643300	Intronic	NA	NA	LOC_Os03g44150	aminotransferase, putative, expressed
Chr.3	24815611	24815611	C	C	A	SNP	OS03G0643300	Intronic	NA	NA	LOC_Os03g44150	aminotransferase, putative, expressed
Chr.3	24816100	24816100	C	C	A	SNP	OS03G0643300	Intronic	NA	NA	LOC_Os03g44150	aminotransferase, putative, expressed
Chr.3	24817017	24817017	C	C	A	SNP	OS03G0643300	Intronic	NA	NA	LOC_Os03g44150	aminotransferase, putative, expressed
Chr.3	28264918	28264918	C	C	A	SNP	OS03G0703300	Intronic	NA	NA	LOC_Os03g49630	expressed protein

Chr.3	29564255	29564255	C	C	A	SNP	OS03G0726500	Intronic	NA	NA	LOC_Os03g51650	membrane protein, putative, expressed
Chr.3	32666409	32666409	C	C	A	SNP	OS03G0786700	Intronic	NA	NA	LOC_Os03g57280	N-dimethylguanosine tRNA methyltransferase, putative, expressed
Chr.3	33347518	33347518	C	C	A	SNP	OS03G0799900	Intronic	NA	NA	LOC_Os03g58570	WD domain, G-beta repeat domain containing protein, expressed
Chr.3	33501523	33501523	C	C	A	SNP	OS03G0802900	Intronic	NA	NA	LOC_Os03g58830	BEE 1, putative, expressed
Chr.3	34910782	34910782	C	C	A	SNP	OS03G0831100	Intronic	NA	NA	LOC_Os03g61560	expressed protein
Chr.3	538350	538350	G	G	A	SNP	OS03G0109300	Intronic	NA	NA	LOC_Os03g01880	possible lysine decarboxylase domain containing protein, expressed
Chr.3	538473	538473	G	G	A	SNP	OS03G0109300	Intronic	NA	NA	LOC_Os03g01880	possible lysine decarboxylase domain containing protein, expressed
Chr.3	538798	538798	G	G	A	SNP	OS03G0109300	Intronic	NA	NA	LOC_Os03g01880	possible lysine decarboxylase domain containing protein, expressed
Chr.3	538935	538935	G	G	A	SNP	OS03G0109300	Intronic	NA	NA	LOC_Os03g01880	possible lysine decarboxylase domain containing protein, expressed
Chr.3	541137	541137	G	G	A	SNP	OS03G0109300	Intronic	NA	NA	LOC_Os03g01880	possible lysine decarboxylase domain containing protein, expressed
Chr.3	541948	541948	C	C	A	SNP	OS03G0109300	Intronic	NA	NA	LOC_Os03g01880	possible lysine decarboxylase domain containing protein, expressed
Chr.3	907081	907081	G	G	A	SNP	OS03G0116000	Intronic	NA	NA	LOC_Os03g02480	inner membrane protein, putative, expressed
Chr.3	909440	909440	G	G	A	SNP	OS03G0116000	Intronic	NA	NA	LOC_Os03g02480	inner membrane protein, putative, expressed
Chr.3	1614780	1614780	G	G	A	SNP	OS03G0128500	Intronic	NA	NA	LOC_Os03g03650	POLD2 - Putative DNA polymerase delta complex subunit, expressed
Chr.3	1616210	1616210	C	C	A	SNP	OS03G0128500	Intronic	NA	NA	LOC_Os03g03650	POLD2 - Putative DNA polymerase delta complex subunit, expressed
Chr.3	1628447	1628447	G	G	A	SNP	OS03G0128700	Exonic	CDS	Missense	LOC_Os03g03660	CAMK_CAMK_like.17 - CAMK includes calcium/calmodulin depedent protein kinases, expressed
Chr.3	1627813	1627813	C	C	A	SNP	OS03G0128700	Intronic	NA	NA	LOC_Os03g03660	CAMK_CAMK_like.17 - CAMK includes calcium/calmodulin depedent protein kinases, expressed
Chr.3	1627957	1627957	G	G	A	SNP	OS03G0128700	Intronic	NA	NA	LOC_Os03g03660	CAMK_CAMK_like.17 - CAMK includes calcium/calmodulin depedent protein kinases, expressed
Chr.3	1628717	1628717	T	T	A	SNP	OS03G0128700	Intronic	NA	NA	LOC_Os03g03660	CAMK_CAMK_like.17 - CAMK includes calcium/calmodulin depedent protein kinases, expressed
Chr.3	1717781	1717781	G	G	A	SNP	OS03G0130400	Intronic	NA	NA	LOC_Os03g03820	adenylate kinase, putative, expressed
Chr.3	1739638	1739638	G	G	A	SNP	OS03G0130700	Exonic	CDS	NA	LOC_Os03g03860	expressed protein
Chr.3	1790627	1790627	G	G	A	SNP	OS03G0131200	Exonic	5UTR	NA	LOC_Os03g03910	catalase domain containing protein, expressed
Chr.3	1790693	1790693	G	G	A	SNP	OS03G0131200	Exonic	5UTR	NA	LOC_Os03g03910	catalase domain containing protein, expressed
Chr.3	1790713	1790713	G	G	A	SNP	OS03G0131200	Exonic	5UTR	NA	LOC_Os03g03910	catalase domain containing protein, expressed
Chr.3	5979946	5979946	G	G	A	SNP	OS03G0214100	Intronic	NA	NA	LOC_Os03g11540	RPA1B - Putative single-stranded DNA binding complex subunit 1, expressed
Chr.3	7061824	7061824	G	G	A	SNP	OS03G0233000	Exonic	5UTR	NA	LOC_Os03g13050	E2F-related protein, putative, expressed
Chr.3	7330860	7330860	G	G	A	SNP	OS03G0238800	Intronic	NA	NA	LOC_Os03g13560	hydroxyproline-rich glycoprotein family protein, putative, expressed
Chr.3	7848726	7848726	C	C	A	SNP	OS03G0248600	Intronic	NA	NA	LOC_Os03g14450	enolase, putative, expressed
Chr.3	7851983	7851983	T	T	A	SNP	OS03G0248600	Intronic	NA	NA	LOC_Os03g14450	enolase, putative, expressed
Chr.3	7852212	7852212	G	G	A	SNP	OS03G0248600	Intronic	NA	NA	LOC_Os03g14450	enolase, putative, expressed
Chr.3	10622016	10622016	G	G	A	SNP	OS03G0301700	Exonic	3UTR	NA	LOC_Os03g18970	protein phosphotase protein, putative, expressed
Chr.3	10693074	10693074	G	G	A	SNP	OS03G0303100	Exonic	CDS	Missense	LOC_Os03g19090	expressed protein
Chr.3	12007388	12007388	C	C	A	SNP	OS03G0328000	Exonic	CDS	NA	LOC_Os03g21080	guanine nucleotide exchange factor, putative, expressed
Chr.3	14480868	14480868	C	C	A	SNP	OS03G0369000	Exonic	CDS	Silent	LOC_Os03g25340	peroxidase precursor, putative, expressed
Chr.3	14482088	14482088	G	G	A	SNP	OS03G0369000	Exonic	CDS	Missense	LOC_Os03g25340	peroxidase precursor, putative, expressed
Chr.3	14481206	14481206	G	G	A	SNP	OS03G0369000	Intronic	NA	NA	LOC_Os03g25340	peroxidase precursor, putative, expressed
Chr.3	14851202	14851202	C	C	A	SNP	OS03G0376100	Exonic	CDS	NA	LOC_Os03g25940	cystathionine gamma-synthase, putative, expressed
Chr.3	29114675	29114675	C	C	A	SNP	OS03G0719000	Exonic	3UTR	NA	LOC_Os03g50970	retrotransposon protein, putative, Ty1-copia subclass, expressed
Chr.3	29116485	29116485	G	G	A	SNP	OS03G0719000	Intronic	NA	NA	LOC_Os03g50970	retrotransposon protein, putative, Ty1-copia subclass, expressed
Chr.3	29138042	29138042	T	T	A	SNP	OS03G0719300	Exonic	CDS	Silent	LOC_Os03g51000	3,4-dihydroxy-2-butanone kinase, putative, expressed
Chr.3	29142822	29142822	G	G	A	SNP	OS03G0719300	Intronic	NA	NA	LOC_Os03g51000	3,4-dihydroxy-2-butanone kinase, putative, expressed
Chr.3	35871240	35871240	G	G	A	SNP	OS03G0851900	Exonic	CDS	Silent	LOC_Os03g63500	ATPase, AFG1 family domain containing protein, expressed
Chr.3	35870708	35870708	T	T	A	SNP	OS03G0851900	Exonic	CDS	Missense	LOC_Os03g63500	ATPase, AFG1 family domain containing protein, expressed
Chr.3	35869406	35869406	G	G	A	SNP	OS03G0851900	Intronic	NA	NA	LOC_Os03g63500	ATPase, AFG1 family domain containing protein, expressed
Chr.3	35871576	35871576	C	C	A	SNP	OS03G0851900	Intronic	NA	NA	LOC_Os03g63500	ATPase, AFG1 family domain containing protein, expressed
Chr.3	35873975	35873975	G	G	A	SNP	OS03G0851900	Intronic	NA	NA	LOC_Os03g63500	ATPase, AFG1 family domain containing protein, expressed
Chr.3	36006352	36006352	G	G	A	SNP	OS03G0854800	Exonic	5UTR	NA	LOC_Os03g63770	RCD1, putative, expressed
Chr.3	36005331	36005332	AT	AT	A	INDEL	OS03G0854800	Exonic	CDS	FrameShift	LOC_Os03g63770	RCD1, putative, expressed
Chr.3	10393719	10393721	ATC	ATC	A	INDEL	OS03G0297100	Intronic	NA	NA	LOC_Os03g18570	40S ribosomal protein S7, putative, expressed
Chr.3	3381622	3381624	AAC	AAC	A	INDEL	OS03G0162500	Exonic	3UTR	NA	LOC_Os03g06680	plant-specific domain TIGR01615 family protein, expressed
Chr.3	1788568	1788576	AGATTTAAT	AGATTTAAT	A	INDEL	OS03G0131200	Intronic	NA	NA	LOC_Os03g03910	catalase domain containing protein, expressed
Chr.3	1741854	1741855	AG	AG	A	INDEL	OS03G0130700	Intronic	NA	NA	LOC_Os03g03860	expressed protein
Chr.3	1737338	1737339	AT	AT	A	INDEL	OS03G0130700	Exonic	3UTR	NA	LOC_Os03g03860	expressed protein
Chr.3	1646634	1646636	AAG	AAG	A	INDEL	OS03G0129100	Intronic	NA	NA	LOC_Os03g03700	MLO domain containing protein, putative, expressed
Chr.3	1627835	1627836	AT	AT	A	INDEL	OS03G0128700	Intronic	NA	NA	LOC_Os03g03660	CAMK_CAMK_like.17 - CAMK includes calcium/calmodulin depedent protein kinases, expressed
Chr.3	471546	471546	G	G	A	SNP	OS03G0107800	Exonic	3UTR	NA	LOC_Os03g01750	dual specificity protein phosphatase, putative, expressed
Chr.3	538649	538649	T	T	A	SNP	OS03G0109300	Intronic	NA	NA	LOC_Os03g01880	possible lysine decarboxylase domain containing protein, expressed
Chr.3	538650	538650	T	T	A	SNP	OS03G0109300	Intronic	NA	NA	LOC_Os03g01880	possible lysine decarboxylase domain containing protein, expressed
Chr.3	538651	538651	G	G	A	SNP	OS03G0109300	Intronic	NA	NA	LOC_Os03g01880	possible lysine decarboxylase domain containing protein, expressed
Chr.3	1739386	1739386	G	G	A	SNP	OS03G0130700	Exonic	CDS	NA	LOC_Os03g03860	expressed protein
Chr.3	1788472	1788472	G	G	A	SNP	OS03G0131200	Intronic	NA	NA	LOC_Os03g03910	catalase domain containing protein, expressed
Chr.3	1788515	1788515	G	G	A	SNP	OS03G0131200	Intronic	NA	NA	LOC_Os03g03910	catalase domain containing protein, expressed
Chr.3	1790503	1790503	C	C	A	SNP	OS03G0131200	Intronic	NA	NA	LOC_Os03g03910	catalase domain containing protein, expressed
Chr.3	2337559	2337559	G	G	A	SNP	OS03G0142500	Intronic	NA	NA	LOC_Os03g04890	zinc finger family protein, putative, expressed
Chr.3	2749591	2749591	G	G	A	SNP	OS03G0149300	Intronic	NA	NA	LOC_Os03g05530	nodulin, putative, expressed
Chr.3	3371450	3371450	G	G	A	SNP	OS03G0162200	Exonic	5UTR	NA	LOC_Os03g06670	Core histone H2A/H2B/H3/H4 domain containing protein, putative, expressed
Chr.3	3561791	3561791	G	G	A	SNP	OS03G0166000	Exonic	3UTR	NA	LOC_Os03g06980	nucleic acid binding protein, putative, expressed
Chr.3	3561819	3561819	C	C	A	SNP	OS03G0166000	Exonic	3UTR	NA	LOC_Os03g06980	nucleic acid binding protein, putative, expressed
Chr.3	3565053	3565053	G	G	A	SNP	OS03G0166000	Intronic	NA	NA	LOC_Os03g06980	nucleic acid binding protein, putative, expressed
Chr.3	4387372	4387372	G	G	A	SNP	OS03G0183600	Intronic	NA	NA	LOC_Os03g08530	aminotransferase, classes I and II, domain containing protein, expressed
Chr.3	4546769	4546769	G	G	A	SNP	OS03G0187000	Intronic	NA	NA	LOC_Os03g08810	expressed protein
Chr.3	5114006	5114006	G	G	A	SNP	OS03G0197200	Exonic	3UTR	NA	LOC_Os03g10100	transporter family protein, putative, expressed
Chr.3	5402953	5402953	G	G	A	SNP	OS03G0203100	Exonic	CDS	NA	LOC_Os03g10600	expressed protein
Chr.3	6373455	6373455	G	G	A	SNP	OS03G0221500	Exonic	3UTR	NA	LOC_Os03g12140	glucan endo-1,3-beta-glucosidase precursor, putative, expressed
Chr.3	6371164	6371164	G	G	A	SNP	OS03G0221500	Intronic	NA	NA	LOC_Os03g12140	glucan endo-1,3-beta-glucosidase precursor, putative, expressed
Chr.3	7062193	7062193	G	G	A	SNP	OS03G0233000	Exonic	CDS	Silent	LOC_Os03g13050	E2F-related protein, putative, expressed
Chr.3	8200964	8200964	G	G	A	SNP	OS03G0255100	Intronic	NA	NA	LOC_Os03g15020	beta-galactosidase precursor, putative, expressed
Chr.3	8200966	8200966	G	G	A	SNP	OS03G0255100	Intronic	NA	NA	LOC_Os03g15020	beta-galactosidase precursor, putative, expressed
Chr.3	8200989	8200989	G	G	A	SNP	OS03G0255100	Intronic	NA	NA	LOC_Os03g15020	beta-galactosidase precursor, putative, expressed
Chr.3	8223604	8223604	G	G	A	SNP	OS03G0255500	Intronic	NA	NA	LOC_Os03g15050	phosphoenolpyruvate carboxykinase, putative, expressed
Chr.3	8842399	8842399	G	G	A	SNP	OS03G0267300	Exonic	CDS	Silent	LOC_Os03g16050	fructose-1,6-bisphosphatase, putative, expressed
Chr.3	9846211	9846211	T	T	A	SNP	OS03G0285700	Intronic	NA	NA	LOC_Os03g17690	OsAPx1 - Cytosolic Ascorbate Peroxidase encoding gene 1-8, expressed
Chr.3	10773011	10773011	G	G	A	SNP	OS03G0304500	Exonic	CDS	Silent	LOC_Os03g19200	DNAJ heat shock N-terminal domain-containing protein, putative, expressed
Chr.3	10906347	10906347	G	G	A	SNP	OS03G0306700	Exonic	CDS	NA	LOC_Os03g19370	CPuORF4 - conserved peptide uORF-containing transcript, expressed
Chr.3	11668110	11668110	C	C	A	SNP	OS03G0322200	Exonic	5UTR	NA	LOC_Os03g20600	expressed protein

Chr.3	13130000	13130000	C	C	A	SNP	OS03G0350100	Exonic	5UTR	NA	LOC_Os03g22730	nucleolar protein NOP5-1, putative, expressed
Chr.3	15668970	15668970	T	T	A	SNP	OS03G0391100	Exonic	CDS	Nonsense	LOC_Os03g27360	RING-H2 finger protein ATL5H precursor, putative, expressed
Chr.3	15668882	15668882	G	G	A	SNP	OS03G0391100	Exonic	CDS	Missense	LOC_Os03g27360	RING-H2 finger protein ATL5H precursor, putative, expressed
Chr.3	15669086	15669086	G	G	A	SNP	OS03G0391100	Exonic	CDS	Missense	LOC_Os03g27360	RING-H2 finger protein ATL5H precursor, putative, expressed
Chr.3	15669154	15669154	T	T	A	SNP	OS03G0391100	Intronic	NA	NA	LOC_Os03g27360	RING-H2 finger protein ATL5H precursor, putative, expressed
Chr.3	15669326	15669326	G	G	A	SNP	OS03G0391100	Intronic	NA	NA	LOC_Os03g27360	RING-H2 finger protein ATL5H precursor, putative, expressed
Chr.3	16286429	16286429	G	G	A	SNP	OS03G0401100	Exonic	3UTR	NA	LOC_Os03g28300	protein kinase domain containing protein, expressed
Chr.3	17291204	17291204	T	T	A	SNP	OS03G0416500	Exonic	5UTR	NA	LOC_Os03g30300	6-phosphogluconolactonase, putative, expressed
Chr.3	17289288	17289288	T	T	A	SNP	OS03G0416500	Intronic	NA	NA	LOC_Os03g30300	6-phosphogluconolactonase, putative, expressed
Chr.3	21635511	21635511	T	T	A	SNP	OS03G0586500	Intronic	NA	NA	LOC_Os03g38950	chloroplast post-illumination chlorophyll fluorescence increase protein, putative, expressed
Chr.3	22948606	22948606	T	T	A	SNP	OS03G0609000	Exonic	5UTR	NA	LOC_Os03g41280	expressed protein
Chr.3	22949242	22949242	G	G	A	SNP	OS03G0609000	Exonic	CDS	Missense	LOC_Os03g41280	expressed protein
Chr.3	24416839	24416839	G	G	A	SNP	OS03G0637800	Intronic	NA	NA	LOC_Os03g43670	TKL_IRAK_CR4L.1 - The CR4L subfamily has homology with Crinkly4, expressed
Chr.3	24449862	24449862	C	C	A	SNP	OS03G0638200	Intronic	NA	NA	LOC_Os03g43720	transporter family protein, putative, expressed
Chr.3	24473134	24473134	G	G	A	SNP	OS03G0638400	Exonic	3UTR	NA	LOC_Os03g43750	retrotransposon, putative, centromere-specific, expressed
Chr.3	24471653	24471653	G	G	A	SNP	OS03G0638400	Exonic	5UTR	NA	LOC_Os03g43750	retrotransposon, putative, centromere-specific, expressed
Chr.3	24467427	24467427	C	C	A	SNP	OS03G0638400	Intronic	NA	NA	LOC_Os03g43750	retrotransposon, putative, centromere-specific, expressed
Chr.3	24468620	24468620	G	G	A	SNP	OS03G0638400	Intronic	NA	NA	LOC_Os03g43750	retrotransposon, putative, centromere-specific, expressed
Chr.3	24469184	24469184	G	G	A	SNP	OS03G0638400	Intronic	NA	NA	LOC_Os03g43750	retrotransposon, putative, centromere-specific, expressed
Chr.3	24470639	24470639	G	G	A	SNP	OS03G0638400	Intronic	NA	NA	LOC_Os03g43750	retrotransposon, putative, centromere-specific, expressed
Chr.3	24705009	24705009	G	G	A	SNP	OS03G0641300	Exonic	3UTR	NA	LOC_Os03g43990	BTBN8 - Bric-a-Brac, Tramtrack, Broad Complex BTB domain with non-phototropic hypocotyl 3 NPH3 and coiled-coil domains, ex
Chr.3	24810115	24810115	G	G	A	SNP	OS03G0643300	Exonic	3UTR	NA	LOC_Os03g44150	aminotransferase, putative, expressed
Chr.3	24814722	24814722	G	G	A	SNP	OS03G0643300	Intronic	NA	NA	LOC_Os03g44150	aminotransferase, putative, expressed
Chr.3	24814730	24814730	G	G	A	SNP	OS03G0643300	Intronic	NA	NA	LOC_Os03g44150	aminotransferase, putative, expressed
Chr.3	25972930	25972930	G	G	A	SNP	OS03G0661600	Exonic	CDS	NA	LOC_Os03g45960	thaumatin, putative, expressed
Chr.3	26385302	26385302	T	T	A	SNP	OS03G0669000	Exonic	CDS	Missense	LOC_Os03g46610	DEAD-box ATP-dependent RNA helicase, putative, expressed
Chr.3	26385048	26385048	G	G	A	SNP	OS03G0669000	Intronic	NA	NA	LOC_Os03g46610	DEAD-box ATP-dependent RNA helicase, putative, expressed
Chr.3	26752107	26752107	C	C	A	SNP	OS03G0676400	Exonic	3UTR	NA	LOC_Os03g47280	VQ domain containing protein, putative, expressed
Chr.3	26752586	26752586	G	G	A	SNP	OS03G0676400	Exonic	CDS	Silent	LOC_Os03g47280	VQ domain containing protein, putative, expressed
Chr.3	26957174	26957174	G	G	A	SNP	OS03G0679700	Intronic	NA	NA	LOC_Os03g47610	thiamine biosynthesis protein thiC, putative, expressed
Chr.3	27287894	27287894	G	G	A	SNP	OS03G0684400	Exonic	5UTR	NA	LOC_Os03g48000	CorA-like magnesium transporter protein, putative, expressed
Chr.3	27288151	27288151	G	G	A	SNP	OS03G0684400	Exonic	CDS	Silent	LOC_Os03g48000	CorA-like magnesium transporter protein, putative, expressed
Chr.3	28053316	28053316	G	G	A	SNP	OS03G0699700	Exonic	CDS	Missense	LOC_Os03g49260	lipoxygenase, putative, expressed
Chr.3	28244532	28244532	G	G	A	SNP	OS03G0703000	Intronic	NA	NA	LOC_Os03g49600	Os3bglu7 - beta-glucosidase, exo-beta-glucanse, expressed
Chr.3	28330623	28330623	G	G	A	SNP	OS03G0704700	Intronic	NA	NA	LOC_Os03g49770	oxysterol-binding protein, putative, expressed
Chr.3	28595705	28595705	C	C	A	SNP	OS03G0709000	Exonic	CDS	Missense	LOC_Os03g50130	microsomal glutathione S-transferase 3, putative, expressed
Chr.3	29018521	29018521	G	G	A	SNP	OS03G0717000	Exonic	CDS	NA	LOC_Os03g50810	receptor protein kinase TMK1 precursor, putative, expressed
Chr.3	29115020	29115020	C	C	A	SNP	OS03G0719000	Exonic	CDS	NA	LOC_Os03g50970	retrotransposon protein, putative, Ty1-copia subclass, expressed
Chr.3	29141757	29141757	T	T	A	SNP	OS03G0719300	Intronic	NA	NA	LOC_Os03g51000	3,4-dihydroxy-2-butanone kinase, putative, expressed
Chr.3	29141758	29141758	T	T	A	SNP	OS03G0719300	Intronic	NA	NA	LOC_Os03g51000	3,4-dihydroxy-2-butanone kinase, putative, expressed
Chr.3	29141863	29141863	G	G	A	SNP	OS03G0719300	Intronic	NA	NA	LOC_Os03g51000	3,4-dihydroxy-2-butanone kinase, putative, expressed
Chr.3	30226164	30226164	G	G	A	SNP	OS03G0737300	Exonic	CDS	Silent	LOC_Os03g52720	magnesium-dependent phosphatase 1, putative, expressed
Chr.3	30517963	30517963	C	C	A	SNP	OS03G0743500	Exonic	5UTR	NA	LOC_Os03g53200	OsCML4 - Calmodulin-related calcium sensor protein, expressed
Chr.3	30534835	30534835	G	G	A	SNP	OS03G0743900	Exonic	CDS	Silent	LOC_Os03g53230	bifunctional 3-phosphoadenosine 5-phosphosulfate synthetase, putative, expressed
Chr.3	31360126	31360126	G	G	A	SNP	OS03G0758500	Exonic	CDS	Missense	LOC_Os03g55120	plastocyanin-like domain containing protein, putative, expressed
Chr.3	31556558	31556558	G	G	A	SNP	OS03G0762900	Intronic	NA	NA	LOC_Os03g55470	expressed protein
Chr.3	32008709	32008709	G	G	A	SNP	OS03G0772600	Exonic	CDS	NA	LOC_Os03g56160	lectin-like receptor kinase 7, putative, expressed
Chr.3	32377250	32377250	T	T	A	SNP	OS03G0780800	Intronic	NA	NA	LOC_Os03g56820	fatty acid hydroxylase, putative, expressed
Chr.3	32710091	32710091	G	G	A	SNP	OS03G0787300	Exonic	CDS	Silent	LOC_Os03g57340	chaperone protein dnaJ, putative, expressed
Chr.3	33217141	33217141	G	G	A	SNP	OS03G0797400	Exonic	CDS	Missense	LOC_Os03g58300	indole-3-glycerol phosphate lyase, chloroplast precursor, putative, expressed
Chr.3	33550772	33550772	G	G	A	SNP	OS03G0803900	Exonic	CDS	Silent	LOC_Os03g58920	galactosyltransferase family protein, putative, expressed
Chr.3	34193117	34193117	G	G	A	SNP	OS03G0815800	Exonic	CDS	Silent	LOC_Os03g60120	AP2 domain containing protein, expressed
Chr.3	34193019	34193019	G	G	A	SNP	OS03G0815800	Exonic	CDS	Missense	LOC_Os03g60120	AP2 domain containing protein, expressed
Chr.3	34908115	34908115	T	T	A	SNP	OS03G0831100	Intronic	NA	NA	LOC_Os03g61560	expressed protein
Chr.3	35162224	35162224	G	G	A	SNP	OS03G0836800	Intronic	NA	NA	LOC_Os03g62060	hydrolase, putative, expressed
Chr.3	35162272	35162272	G	G	A	SNP	OS03G0836800	Intronic	NA	NA	LOC_Os03g62060	hydrolase, putative, expressed
Chr.3	36283198	36283198	G	G	A	SNP	OS03G0859600	Exonic	CDS	NA	LOC_Os03g64210	T-complex protein, putative, expressed
Chr.3	538219	538230	ATAATACTAAAT	ATAATACTAAAT	A	INDEL	OS03G0109300	Intronic	NA	NA	LOC_Os03g01880	possible lysine decarboxylase domain containing protein, expressed
Chr.3	1244126	1244127	AC	AC	A	INDEL	OS03G0122300	Intronic	NA	NA	LOC_Os03g03034	flavonol synthase/flavanone 3-hydroxylase, putative, expressed
Chr.3	1788446	1788447	AC	AC	A	INDEL	OS03G0131200	Intronic	NA	NA	LOC_Os03g03910	catalase domain containing protein, expressed
Chr.3	6636689	6636691	AAG	AAG	A	INDEL	OS03G0226400	Exonic	5UTR	NA	LOC_Os03g12530	cation efflux family protein, putative, expressed
Chr.3	8223600	8223602	AAG	AAG	A	INDEL	OS03G0255500	Intronic	NA	NA	LOC_Os03g15050	phosphoenolpyruvate carboxykinase, putative, expressed
Chr.3	9844709	9844729	ATGGAAGAAGCCTTGGGGACC	ATGGAAGAAGCCTTGGGGACC	A	INDEL	OS03G0285700	Intronic	NA	NA	LOC_Os03g17690	OsAPx1 - Cytosolic Ascorbate Peroxidase encoding gene 1-8, expressed
Chr.3	10042529	10042530	AT	AT	A	INDEL	OS03G0289800	Intronic	NA	NA	LOC_Os03g18030	leucoanthocyanidin dioxygenase, putative, expressed
Chr.3	10623349	10623351	ACC	ACC	A	INDEL	OS03G0301700	Intronic	NA	NA	LOC_Os03g18970	protein phosphotase protein, putative, expressed
Chr.3	13189714	13189729	ATGCGGCGGCGGCGGC	ATGCGGCGGCGGCGGC	A	INDEL	OS03G0351700	Exonic	CDS	InFrame	LOC_Os03g22820	expressed protein
Chr.3	13189776	13189785	AGCGGCGGCG	AGCGGCGGCG	A	INDEL	OS03G0351700	Exonic	CDS	InFrame	LOC_Os03g22820	expressed protein
Chr.3	16289683	16289684	AT	AT	A	INDEL	OS03G0401100	Intronic	NA	NA	LOC_Os03g28300	protein kinase domain containing protein, expressed
Chr.3	17288316	17288321	ATCAAC	ATCAAC	A	INDEL	OS03G0416500	Exonic	3UTR	NA	LOC_Os03g30300	6-phosphogluconolactonase, putative, expressed
Chr.3	21088432	21088433	AG	AG	A	INDEL	OS03G0576700	Intronic	NA	NA	LOC_Os03g37970	ribosomal protein L13, putative, expressed
Chr.3	23375269	23375270	AT	AT	A	INDEL	OS03G0616700	Intronic	NA	NA	LOC_Os03g42050	exportin-7-A, putative, expressed
Chr.3	24469337	24469339	ATC	ATC	A	INDEL	OS03G0638400	Intronic	NA	NA	LOC_Os03g43750	retrotransposon, putative, centromere-specific, expressed
Chr.3	24597921	24597937	ATCTGAGAACAGTACAG	ATCTGAGAACAGTACAG	A	INDEL	OS03G0639800	Intronic	NA	NA	LOC_Os03g43860	SNF7 domain containing protein, putative, expressed
Chr.3	24814514	24814517	AAAG	AAAG	A	INDEL	OS03G0643300	Intronic	NA	NA	LOC_Os03g44150	aminotransferase, putative, expressed
Chr.3	24814715	24814720	AGAAAG	AGAAAG	A	INDEL	OS03G0643300	Intronic	NA	NA	LOC_Os03g44150	aminotransferase, putative, expressed
Chr.3	24814781	24814783	AAG	AAG	A	INDEL	OS03G0643300	Intronic	NA	NA	LOC_Os03g44150	aminotransferase, putative, expressed
Chr.3	24815459	24815461	AGT	AGT	A	INDEL	OS03G0643300	Intronic	NA	NA	LOC_Os03g44150	aminotransferase, putative, expressed
Chr.3	24815643	24815644	AT	AT	A	INDEL	OS03G0643300	Intronic	NA	NA	LOC_Os03g44150	aminotransferase, putative, expressed
Chr.3	24817250	24817252	AAC	AAC	A	INDEL	OS03G0643300	Intronic	NA	NA	LOC_Os03g44150	aminotransferase, putative, expressed
Chr.3	25103850	25103851	AC	AC	A	INDEL	OS03G0648100	Exonic	5UTR	NA	LOC_Os03g44580	DNA binding protein, putative, expressed
Chr.3	25104023	25104025	AAG	AAG	A	INDEL	OS03G0648100	Exonic	5UTR	NA	LOC_Os03g44580	DNA binding protein, putative, expressed
Chr.3	27493948	27493953	ACTCTG	ACTCTG	A	INDEL	OS03G0689100	Intronic	NA	NA	LOC_Os03g48300	histidine acid phosphatase, putative, expressed
Chr.3	28052291	28052293	ACT	ACT	A	INDEL	OS03G0699700	Intronic	NA	NA	LOC_Os03g49260	lipoxygenase, putative, expressed
Chr.3	29563711	29563712	AG	AG	A	INDEL	OS03G0726500	Intronic	NA	NA	LOC_Os03g51650	membrane protein, putative, expressed
Chr.3	30316685	30316686	AC	AC	A	INDEL	OS03G0738600	Intronic	NA	NA	LOC_Os03g52860	lipoxygenase, putative, expressed

Chr.3	30482610	30482613	ATGC	ATGC	A	INDEL	OS03G0742900	Intronic	NA	NA	LOC_Os03g53150	OsIAA13 - Auxin-responsive Aux/IAA gene family member, expressed
Chr.3	31785465	31785471	AATCATC	AATCATC	A	INDEL	OS03G0767600	Exonic	CDS	InFrame	LOC_Os03g55830	BTBN10 - Bric-a-Brac, Tramtrack, Broad Complex BTB domain with non-phototropic hypocotyl 3 NPH3 and coiled-coil domains, e
Chr.3	32181960	32181961	AC	AC	A	INDEL	OS03G0776000	Exonic	3UTR	NA	LOC_Os03g56460	glucose-6-phosphate isomerase, putative, expressed
Chr.3	32178482	32178483	AT	AT	A	INDEL	OS03G0776000	Intronic	NA	NA	LOC_Os03g56460	glucose-6-phosphate isomerase, putative, expressed
Chr.3	32709287	32709288	AT	AT	A	INDEL	OS03G0787300	Intronic	NA	NA	LOC_Os03g57340	chaperone protein dnaJ, putative, expressed
Chr.3	35793713	35793715	ACT	ACT	A	INDEL	OS03G0850400	Intronic	NA	NA	LOC_Os03g63330	aspartokinase, chloroplast precursor, putative, expressed
Chr.2	4294119	4294119	T	T	A	SNP	OS02G0177800	Exonic	CDS	Silent	LOC_Os02g08120	calmodulin binding protein, putative, expressed
Chr.2	5155055	5155055	T	T	A	SNP	OS02G0192500	Intronic	NA	NA	LOC_Os02g09930	CSLA1 - cellulose synthase-like family A; mannan synthase, expressed
Chr.2	5156679	5156679	T	T	A	SNP	OS02G0192500	Intronic	NA	NA	LOC_Os02g09930	CSLA1 - cellulose synthase-like family A; mannan synthase, expressed
Chr.2	5157542	5157542	T	T	A	SNP	OS02G0192500	Intronic	NA	NA	LOC_Os02g09930	CSLA1 - cellulose synthase-like family A; mannan synthase, expressed
Chr.2	5252650	5252650	T	T	A	SNP	OS02G0194200	Intronic	NA	NA	LOC_Os02g10080	zinc finger C-x8-C-x5-C-x3-H type family protein, expressed
Chr.2	6813214	6813214	T	T	A	SNP	OS02G0222100	Intronic	NA	NA	LOC_Os02g12900	cysteine synthase, putative, expressed
Chr.2	9973665	9973665	T	T	A	SNP	OS02G0273700	Intronic	NA	NA	LOC_Os02g17350	VHS and GAT domain containing protein, expressed
Chr.2	9973683	9973683	T	T	A	SNP	OS02G0273700	Intronic	NA	NA	LOC_Os02g17350	VHS and GAT domain containing protein, expressed
Chr.2	9973705	9973705	T	T	A	SNP	OS02G0273700	Intronic	NA	NA	LOC_Os02g17350	VHS and GAT domain containing protein, expressed
Chr.2	9974909	9974909	T	T	A	SNP	OS02G0273700	Intronic	NA	NA	LOC_Os02g17350	VHS and GAT domain containing protein, expressed
Chr.2	9976013	9976013	T	T	A	SNP	OS02G0273700	Intronic	NA	NA	LOC_Os02g17350	VHS and GAT domain containing protein, expressed
Chr.2	9989319	9989319	T	T	A	SNP	OS02G0273900	Intronic	NA	NA	LOC_Os02g17370	expressed protein
Chr.2	9989755	9989755	T	T	A	SNP	OS02G0273900	Intronic	NA	NA	LOC_Os02g17370	expressed protein
Chr.2	9990197	9990197	T	T	A	SNP	OS02G0273900	Intronic	NA	NA	LOC_Os02g17370	expressed protein
Chr.2	11486670	11486670	T	T	A	SNP	OS02G0299300	Intronic	NA	NA	LOC_Os02g19650	hydrolase, alpha/beta fold family domain containing protein, expressed
Chr.2	17528150	17528150	T	T	A	SNP	OS02G0498300	Intronic	NA	NA	LOC_Os02g29510	DUF803 domain containing, putative, expressed
Chr.2	21810431	21810431	T	T	A	SNP	OS02G0570700	Exonic	3UTR	NA	LOC_Os02g36190	cytochrome P450, putative, expressed
Chr.2	21810981	21810981	T	T	A	SNP	OS02G0570700	Exonic	CDS	Missense	LOC_Os02g36190	cytochrome P450, putative, expressed
Chr.2	22509701	22509701	T	T	A	SNP	OS02G0583700	Exonic	3UTR	NA	LOC_Os02g37250	hypothetical protein
Chr.2	24566917	24566917	T	T	A	SNP	OS02G0618100	Intronic	NA	NA	LOC_Os02g40500	OsGrx_C2.1 - glutaredoxin subgroup I, expressed
Chr.2	24737501	24737501	T	T	A	SNP	OS02G0621600	Exonic	3UTR	NA	LOC_Os02g40820	expressed protein
Chr.2	35098095	35098095	T	T	A	SNP	OS02G0817900	Exonic	CDS	Missense	LOC_Os02g57290	cytochrome P450, putative, expressed
Chr.2	35095260	35095260	T	T	A	SNP	OS02G0817900	Intronic	NA	NA	LOC_Os02g57290	cytochrome P450, putative, expressed
Chr.2	35098210	35098210	T	T	A	SNP	OS02G0817900	Intronic	NA	NA	LOC_Os02g57290	cytochrome P450, putative, expressed
Chr.2	121573	121573	G	G	A	SNP	OS02G0102200	Exonic	3UTR	NA	LOC_Os02g01210	amino acid transporter, putative, expressed
Chr.2	317350	317350	G	G	A	SNP	OS02G0105900	Exonic	CDS	Silent	LOC_Os02g01560	40S ribosomal protein S4, putative, expressed
Chr.2	317698	317698	G	G	A	SNP	OS02G0105900	Exonic	CDS	Silent	LOC_Os02g01560	40S ribosomal protein S4, putative, expressed
Chr.2	317679	317679	G	G	A	SNP	OS02G0105900	Exonic	CDS	Missense	LOC_Os02g01560	40S ribosomal protein S4, putative, expressed
Chr.2	639349	639349	G	G	A	SNP	OS02G0111800	Exonic	CDS	Silent	LOC_Os02g02140	receptor protein kinase CLAVATA1 precursor, putative, expressed
Chr.2	3829400	3829400	G	G	A	SNP	OS02G0170100	Intronic	NA	NA	LOC_Os02g07410	glycine cleavage system H protein, putative, expressed
Chr.2	4294177	4294177	G	G	A	SNP	OS02G0177800	Intronic	NA	NA	LOC_Os02g08120	calmodulin binding protein, putative, expressed
Chr.2	4295202	4295202	G	G	A	SNP	OS02G0177800	Intronic	NA	NA	LOC_Os02g08120	calmodulin binding protein, putative, expressed
Chr.2	4453460	4453460	G	G	A	SNP	OS02G0180000	Exonic	CDS	Missense	LOC_Os02g08364	protein phosphatase 2C, putative, expressed
Chr.2	5156123	5156123	G	G	A	SNP	OS02G0192500	Exonic	CDS	Silent	LOC_Os02g09930	CSLA1 - cellulose synthase-like family A; mannan synthase, expressed
Chr.2	5155505	5155505	G	G	A	SNP	OS02G0192500	Intronic	NA	NA	LOC_Os02g09930	CSLA1 - cellulose synthase-like family A; mannan synthase, expressed
Chr.2	5155594	5155594	G	G	A	SNP	OS02G0192500	Intronic	NA	NA	LOC_Os02g09930	CSLA1 - cellulose synthase-like family A; mannan synthase, expressed
Chr.2	5157497	5157497	G	G	A	SNP	OS02G0192500	Intronic	NA	NA	LOC_Os02g09930	CSLA1 - cellulose synthase-like family A; mannan synthase, expressed
Chr.2	5157499	5157499	G	G	A	SNP	OS02G0192500	Intronic	NA	NA	LOC_Os02g09930	CSLA1 - cellulose synthase-like family A; mannan synthase, expressed
Chr.2	5157502	5157502	G	G	A	SNP	OS02G0192500	Intronic	NA	NA	LOC_Os02g09930	CSLA1 - cellulose synthase-like family A; mannan synthase, expressed
Chr.2	5551445	5551445	G	G	A	SNP	OS02G0198900	Intronic	NA	NA	LOC_Os02g10540	ribosomal protein L7/L12 C-terminal domain containing protein, expressed
Chr.2	5707974	5707974	G	G	A	SNP	OS02G0202200	Intronic	NA	NA	LOC_Os02g10780	SPX domain containing protein, putative, expressed
Chr.2	5760289	5760289	G	G	A	SNP	OS02G0202950	Exonic	CDS	NA	LOC_Os02g10860	bZIP transcription factor domain containing protein, expressed
Chr.2	6078998	6078998	G	G	A	SNP	OS02G0208500	Exonic	CDS	Missense	LOC_Os02g11770	expressed protein
Chr.2	6833339	6833339	G	G	A	SNP	OS02G0222500	Exonic	3UTR	NA	LOC_Os02g12939	expressed protein
Chr.2	6834605	6834605	G	G	A	SNP	OS02G0222500	Exonic	CDS	NA	LOC_Os02g12939	expressed protein
Chr.2	6834653	6834653	G	G	A	SNP	OS02G0222500	Exonic	CDS	NA	LOC_Os02g12939	expressed protein
Chr.2	6834710	6834710	G	G	A	SNP	OS02G0222500	Exonic	CDS	NA	LOC_Os02g12939	expressed protein
Chr.2	6835140	6835140	G	G	A	SNP	OS02G0222500	Intronic	NA	NA	LOC_Os02g12939	expressed protein
Chr.2	6835325	6835325	G	G	A	SNP	OS02G0222500	Intronic	NA	NA	LOC_Os02g12939	expressed protein
Chr.2	9651021	9651021	G	G	A	SNP	OS02G0269600	Exonic	CDS	Silent	LOC_Os02g16940	OsSub13 - Putative Subtilisin homologue, expressed
Chr.2	9651039	9651039	G	G	A	SNP	OS02G0269600	Exonic	CDS	Silent	LOC_Os02g16940	OsSub13 - Putative Subtilisin homologue, expressed
Chr.2	9651057	9651057	G	G	A	SNP	OS02G0269600	Exonic	CDS	Silent	LOC_Os02g16940	OsSub13 - Putative Subtilisin homologue, expressed
Chr.2	9651189	9651189	G	G	A	SNP	OS02G0269600	Exonic	CDS	Silent	LOC_Os02g16940	OsSub13 - Putative Subtilisin homologue, expressed
Chr.2	9651294	9651294	G	G	A	SNP	OS02G0269600	Exonic	CDS	Silent	LOC_Os02g16940	OsSub13 - Putative Subtilisin homologue, expressed
Chr.2	9654851	9654851	G	G	A	SNP	OS02G0269600	Exonic	CDS	Silent	LOC_Os02g16940	OsSub13 - Putative Subtilisin homologue, expressed
Chr.2	9925989	9925989	G	G	A	SNP	OS02G0272900	Intronic	NA	NA	LOC_Os02g17304	resistance protein, putative, expressed
Chr.2	9927153	9927153	G	G	A	SNP	OS02G0272900	Intronic	NA	NA	LOC_Os02g17304	resistance protein, putative, expressed
Chr.2	9978042	9978042	G	G	A	SNP	OS02G0273700	Exonic	3UTR	NA	LOC_Os02g17350	VHS and GAT domain containing protein, expressed
Chr.2	9973848	9973848	G	G	A	SNP	OS02G0273700	Exonic	CDS	Silent	LOC_Os02g17350	VHS and GAT domain containing protein, expressed
Chr.2	9973881	9973881	G	G	A	SNP	OS02G0273700	Exonic	CDS	Silent	LOC_Os02g17350	VHS and GAT domain containing protein, expressed
Chr.2	9975660	9975660	G	G	A	SNP	OS02G0273700	Exonic	CDS	Silent	LOC_Os02g17350	VHS and GAT domain containing protein, expressed
Chr.2	9973874	9973874	G	G	A	SNP	OS02G0273700	Exonic	CDS	Missense	LOC_Os02g17350	VHS and GAT domain containing protein, expressed
Chr.2	9973262	9973262	G	G	A	SNP	OS02G0273700	Intronic	NA	NA	LOC_Os02g17350	VHS and GAT domain containing protein, expressed
Chr.2	9973312	9973312	G	G	A	SNP	OS02G0273700	Intronic	NA	NA	LOC_Os02g17350	VHS and GAT domain containing protein, expressed
Chr.2	9973600	9973600	G	G	A	SNP	OS02G0273700	Intronic	NA	NA	LOC_Os02g17350	VHS and GAT domain containing protein, expressed
Chr.2	9973711	9973711	G	G	A	SNP	OS02G0273700	Intronic	NA	NA	LOC_Os02g17350	VHS and GAT domain containing protein, expressed
Chr.2	9973942	9973942	G	G	A	SNP	OS02G0273700	Intronic	NA	NA	LOC_Os02g17350	VHS and GAT domain containing protein, expressed
Chr.2	9974662	9974662	G	G	A	SNP	OS02G0273700	Intronic	NA	NA	LOC_Os02g17350	VHS and GAT domain containing protein, expressed
Chr.2	9989030	9989030	G	G	A	SNP	OS02G0273900	Exonic	3UTR	NA	LOC_Os02g17370	expressed protein
Chr.2	9989249	9989249	G	G	A	SNP	OS02G0273900	Intronic	NA	NA	LOC_Os02g17370	expressed protein
Chr.2	9989551	9989551	G	G	A	SNP	OS02G0273900	Intronic	NA	NA	LOC_Os02g17370	expressed protein
Chr.2	9989750	9989750	G	G	A	SNP	OS02G0273900	Intronic	NA	NA	LOC_Os02g17370	expressed protein
Chr.2	9989954	9989954	G	G	A	SNP	OS02G0273900	Intronic	NA	NA	LOC_Os02g17370	expressed protein
Chr.2	9990226	9990226	G	G	A	SNP	OS02G0273900	Intronic	NA	NA	LOC_Os02g17370	expressed protein
Chr.2	11487173	11487173	G	G	A	SNP	OS02G0299300	Intronic	NA	NA	LOC_Os02g19650	hydrolase, alpha/beta fold family domain containing protein, expressed
Chr.2	16012498	16012498	G	G	A	SNP	OS02G0471500	Intronic	NA	NA	LOC_Os02g27220	protein phosphatase 2C, putative, expressed
Chr.2	16115646	16115646	G	G	A	SNP	OS02G0473200	Exonic	CDS	Silent	LOC_Os02g27360	aspartic proteinase-like protein 2 precursor, putative, expressed
Chr.2	16115649	16115649	G	G	A	SNP	OS02G0473200	Exonic	CDS	Silent	LOC_Os02g27360	aspartic proteinase-like protein 2 precursor, putative, expressed
Chr.2	16115647	16115647	G	G	A	SNP	OS02G0473200	Exonic	CDS	Missense	LOC_Os02g27360	aspartic proteinase-like protein 2 precursor, putative, expressed

Chr.2	16117021	16117021	G	G	A	SNP	OS02G0473200	Intronic	NA	NA	LOC_Os02g27360	aspartic proteinase-like protein 2 precursor, putative, expressed
Chr.2	17156697	17156697	G	G	A	SNP	OS02G0491400	Exonic	3UTR	NA	LOC_Os02g28980	peptidyl-prolyl isomerase, putative, expressed
Chr.2	17157660	17157660	G	G	A	SNP	OS02G0491400	Exonic	CDS	Silent	LOC_Os02g28980	peptidyl-prolyl isomerase, putative, expressed
Chr.2	17528706	17528706	G	G	A	SNP	OS02G0498300	Intronic	NA	NA	LOC_Os02g29510	DUF803 domain containing, putative, expressed
Chr.2	17537604	17537604	G	G	A	SNP	OS02G0498300	Intronic	NA	NA	LOC_Os02g29510	DUF803 domain containing, putative, expressed
Chr.2	21810469	21810469	G	G	A	SNP	OS02G0570700	Exonic	3UTR	NA	LOC_Os02g36190	cytochrome P450, putative, expressed
Chr.2	22297672	22297672	G	G	A	SNP	OS02G0579600	Intronic	NA	NA	LOC_Os02g36924	OsMADS27 - MADS-box family gene with MIKCc type-box, expressed
Chr.2	22297699	22297699	G	G	A	SNP	OS02G0579600	Intronic	NA	NA	LOC_Os02g36924	OsMADS27 - MADS-box family gene with MIKCc type-box, expressed
Chr.2	22298257	22298257	G	G	A	SNP	OS02G0579600	Intronic	NA	NA	LOC_Os02g36924	OsMADS27 - MADS-box family gene with MIKCc type-box, expressed
Chr.2	22509703	22509703	G	G	A	SNP	OS02G0583700	Exonic	3UTR	NA	LOC_Os02g37250	hypothetical protein
Chr.2	24903349	24903349	G	G	A	SNP	OS02G0624400	Exonic	CDS	Silent	LOC_Os02g41520	glycosyl transferase 8 domain containing protein, putative, expressed
Chr.2	30520106	30520106	G	G	A	SNP	OS02G0732200	Intronic	NA	NA	LOC_Os02g49950	spotted leaf 11, putative, expressed
Chr.2	31665353	31665353	G	G	A	SNP	OS02G0753000	Exonic	CDS	Silent	LOC_Os02g51680	uncharacterized glycosyl hydrolase Rv2006/MT2062, putative, expressed
Chr.2	34027866	34027866	G	G	A	SNP	OS02G0799000	Intronic	NA	NA	LOC_Os02g55560	protein phosphatase 2C, putative, expressed
Chr.2	34675326	34675326	G	G	A	SNP	OS02G0810900	Intronic	NA	NA	LOC_Os02g56600	no apical meristem protein, putative, expressed
Chr.2	35081965	35081965	G	G	A	SNP	OS02G0817700	Exonic	CDS	Silent	LOC_Os02g57260	3-ketoacyl-CoA thiolase, peroxisomal precursor, putative, expressed
Chr.2	35094261	35094261	G	G	A	SNP	OS02G0817900	Exonic	CDS	Silent	LOC_Os02g57290	cytochrome P450, putative, expressed
Chr.2	35098234	35098234	G	G	A	SNP	OS02G0817900	Intronic	NA	NA	LOC_Os02g57290	cytochrome P450, putative, expressed
Chr.2	35560723	35560723	G	G	A	SNP	OS02G0827400	Exonic	CDS	NA	LOC_Os02g58100	expressed protein
Chr.2	35674879	35674879	G	G	A	SNP	OS02G0829800	Exonic	3UTR	NA	LOC_Os02g58300	expressed protein
Chr.2	317725	317725	C	C	A	SNP	OS02G0105900	Exonic	CDS	Missense	LOC_Os02g01560	40S ribosomal protein S4, putative, expressed
Chr.2	5551700	5551700	C	C	A	SNP	OS02G0198900	Intronic	NA	NA	LOC_Os02g10540	ribosomal protein L7/L12 C-terminal domain containing protein, expressed
Chr.2	6078917	6078917	C	C	A	SNP	OS02G0208500	Exonic	CDS	Missense	LOC_Os02g11770	expressed protein
Chr.2	6835344	6835344	C	C	A	SNP	OS02G0222500	Intronic	NA	NA	LOC_Os02g12939	expressed protein
Chr.2	9651756	9651756	C	C	A	SNP	OS02G0269600	Exonic	CDS	Silent	LOC_Os02g16940	OsSub13 - Putative Subtilisin homologue, expressed
Chr.2	9651416	9651416	C	C	A	SNP	OS02G0269600	Exonic	CDS	Missense	LOC_Os02g16940	OsSub13 - Putative Subtilisin homologue, expressed
Chr.2	9927567	9927567	C	C	A	SNP	OS02G0272900	Exonic	CDS	Missense	LOC_Os02g17304	resistance protein, putative, expressed
Chr.2	9925595	9925595	C	C	A	SNP	OS02G0272900	Intronic	NA	NA	LOC_Os02g17304	resistance protein, putative, expressed
Chr.2	9927240	9927240	C	C	A	SNP	OS02G0272900	Intronic	NA	NA	LOC_Os02g17304	resistance protein, putative, expressed
Chr.2	9977799	9977799	C	C	A	SNP	OS02G0273700	Exonic	3UTR	NA	LOC_Os02g17350	VHS and GAT domain containing protein, expressed
Chr.2	9973778	9973778	C	C	A	SNP	OS02G0273700	Intronic	NA	NA	LOC_Os02g17350	VHS and GAT domain containing protein, expressed
Chr.2	9989387	9989387	C	C	A	SNP	OS02G0273900	Intronic	NA	NA	LOC_Os02g17370	expressed protein
Chr.2	9989447	9989447	C	C	A	SNP	OS02G0273900	Intronic	NA	NA	LOC_Os02g17370	expressed protein
Chr.2	9989485	9989485	C	C	A	SNP	OS02G0273900	Intronic	NA	NA	LOC_Os02g17370	expressed protein
Chr.2	9989486	9989486	C	C	A	SNP	OS02G0273900	Intronic	NA	NA	LOC_Os02g17370	expressed protein
Chr.2	9989778	9989778	C	C	A	SNP	OS02G0273900	Intronic	NA	NA	LOC_Os02g17370	expressed protein
Chr.2	9989807	9989807	C	C	A	SNP	OS02G0273900	Intronic	NA	NA	LOC_Os02g17370	expressed protein
Chr.2	9989840	9989840	C	C	A	SNP	OS02G0273900	Intronic	NA	NA	LOC_Os02g17370	expressed protein
Chr.2	16115652	16115652	C	C	A	SNP	OS02G0473200	Exonic	CDS	Missense	LOC_Os02g27360	aspartic proteinase-like protein 2 precursor, putative, expressed
Chr.2	21810951	21810951	C	C	A	SNP	OS02G0570700	Exonic	CDS	Missense	LOC_Os02g36190	cytochrome P450, putative, expressed
Chr.2	22295008	22295008	C	C	A	SNP	OS02G0579600	Exonic	CDS	Silent	LOC_Os02g36924	OsMADS27 - MADS-box family gene with MIKCc type-box, expressed
Chr.2	22298105	22298105	C	C	A	SNP	OS02G0579600	Intronic	NA	NA	LOC_Os02g36924	OsMADS27 - MADS-box family gene with MIKCc type-box, expressed
Chr.2	23993561	23993561	C	C	A	SNP	OS02G0610600	Intronic	NA	NA	LOC_Os02g39720	splicing factor, arginine/serine-rich 7, putative, expressed
Chr.2	28750887	28750887	C	C	A	SNP	OS02G0699000	Intronic	NA	NA	LOC_Os02g47090	peptide transporter PTR2, putative, expressed
Chr.2	33515629	33515629	C	C	A	SNP	OS02G0788800	Intronic	NA	NA	LOC_Os02g54730	transmembrane amino acid transporter protein, putative, expressed
Chr.2	35098232	35098232	C	C	A	SNP	OS02G0817900	Intronic	NA	NA	LOC_Os02g57290	cytochrome P450, putative, expressed
Chr.2	35098566	35098566	C	C	A	SNP	OS02G0817900	Intronic	NA	NA	LOC_Os02g57290	cytochrome P450, putative, expressed
Chr.2	35675126	35675126	C	C	A	SNP	OS02G0829800	Exonic	CDS	Missense	LOC_Os02g58300	expressed protein
Chr.2	3039185	3039185	T	T	A	SNP	OS02G0155400	Exonic	CDS	Silent	LOC_Os02g06090	phytosulfokine receptor precursor, putative, expressed
Chr.2	3038974	3038974	T	T	A	SNP	OS02G0155400	Exonic	CDS	Missense	LOC_Os02g06090	phytosulfokine receptor precursor, putative, expressed
Chr.2	18580360	18580360	T	T	A	SNP	OS02G0515200	Intronic	NA	NA	LOC_Os02g31080	hydroxyproline-rich glycoprotein family protein, putative, expressed
Chr.2	21933817	21933817	T	T	A	SNP	OS02G0572400	Intronic	NA	NA	LOC_Os02g36340	riboflavin biosynthesis protein ribAB, chloroplast precursor, putative, expressed
Chr.2	768154	768154	G	G	A	SNP	OS02G0114200	Exonic	CDS	Silent	LOC_Os02g02320	OsSCP6 - Putative Serine Carboxypeptidase homologue, expressed
Chr.2	768774	768774	G	G	A	SNP	OS02G0114200	Intronic	NA	NA	LOC_Os02g02320	OsSCP6 - Putative Serine Carboxypeptidase homologue, expressed
Chr.2	2744956	2744956	G	G	A	SNP	OS02G0149800	Intronic	NA	NA	LOC_Os02g05630	protein phosphatase 2C, putative, expressed
Chr.2	3038780	3038780	G	G	A	SNP	OS02G0155400	Exonic	CDS	Silent	LOC_Os02g06090	phytosulfokine receptor precursor, putative, expressed
Chr.2	3039221	3039221	G	G	A	SNP	OS02G0155400	Exonic	CDS	Silent	LOC_Os02g06090	phytosulfokine receptor precursor, putative, expressed
Chr.2	3039239	3039239	G	G	A	SNP	OS02G0155400	Exonic	CDS	Silent	LOC_Os02g06090	phytosulfokine receptor precursor, putative, expressed
Chr.2	3037825	3037825	G	G	A	SNP	OS02G0155400	Exonic	CDS	Missense	LOC_Os02g06090	phytosulfokine receptor precursor, putative, expressed
Chr.2	3038379	3038379	G	G	A	SNP	OS02G0155400	Exonic	CDS	Missense	LOC_Os02g06090	phytosulfokine receptor precursor, putative, expressed
Chr.2	3039351	3039351	G	G	A	SNP	OS02G0155400	Exonic	CDS	Missense	LOC_Os02g06090	phytosulfokine receptor precursor, putative, expressed
Chr.2	3039789	3039789	G	G	A	SNP	OS02G0155400	Exonic	CDS	Missense	LOC_Os02g06090	phytosulfokine receptor precursor, putative, expressed
Chr.2	22510248	22510248	G	G	A	SNP	OS02G0583700	Intronic	NA	NA	LOC_Os02g37250	hypothetical protein
Chr.2	22510357	22510357	G	G	A	SNP	OS02G0583700	Intronic	NA	NA	LOC_Os02g37250	hypothetical protein
Chr.2	769333	769333	C	C	A	SNP	OS02G0114200	Exonic	CDS	Missense	LOC_Os02g02320	OsSCP6 - Putative Serine Carboxypeptidase homologue, expressed
Chr.2	3038204	3038204	C	C	A	SNP	OS02G0155400	Exonic	CDS	Silent	LOC_Os02g06090	phytosulfokine receptor precursor, putative, expressed
Chr.2	3038291	3038291	C	C	A	SNP	OS02G0155400	Exonic	CDS	Silent	LOC_Os02g06090	phytosulfokine receptor precursor, putative, expressed
Chr.2	3037699	3037699	C	C	A	SNP	OS02G0155400	Exonic	CDS	Missense	LOC_Os02g06090	phytosulfokine receptor precursor, putative, expressed
Chr.2	3038433	3038433	C	C	A	SNP	OS02G0155400	Exonic	CDS	Missense	LOC_Os02g06090	phytosulfokine receptor precursor, putative, expressed
Chr.2	3038809	3038809	C	C	A	SNP	OS02G0155400	Exonic	CDS	Missense	LOC_Os02g06090	phytosulfokine receptor precursor, putative, expressed
Chr.2	3039224	3039224	C	C	A	SNP	OS02G0155400	Exonic	CDS	Missense	LOC_Os02g06090	phytosulfokine receptor precursor, putative, expressed
Chr.2	81587	81587	G	G	A	SNP	OS02G0101500	Intronic	NA	NA	LOC_Os02g01150	erythronate-4-phosphate dehydrogenase domain containing protein, expressed
Chr.2	640087	640087	C	C	A	SNP	OS02G0111800	Exonic	CDS	Silent	LOC_Os02g02140	receptor protein kinase CLAVATA1 precursor, putative, expressed
Chr.2	3038159	3038159	T	T	A	SNP	OS02G0155400	Exonic	CDS	Silent	LOC_Os02g06090	phytosulfokine receptor precursor, putative, expressed
Chr.2	3541034	3541034	G	G	A	SNP	OS02G0165500	Exonic	5UTR	NA	LOC_Os02g06960	expressed protein
Chr.2	4297187	4297187	T	T	A	SNP	OS02G0177800	Intronic	NA	NA	LOC_Os02g08120	calmodulin binding protein, putative, expressed
Chr.2	4754878	4754878	G	G	A	SNP	OS02G0185200	Exonic	CDS	Missense	LOC_Os02g09240	cytochrome P450 71D8, putative, expressed
Chr.2	5155383	5155383	T	T	A	SNP	OS02G0192500	Intronic	NA	NA	LOC_Os02g09930	CSLA1 - cellulose synthase-like family A; mannan synthase, expressed
Chr.2	5156019	5156019	G	G	A	SNP	OS02G0192500	Intronic	NA	NA	LOC_Os02g09930	CSLA1 - cellulose synthase-like family A; mannan synthase, expressed
Chr.2	5194970	5194970	C	C	A	SNP	OS02G0193300	Exonic	CDS	Missense	LOC_Os02g09990	expressed protein
Chr.2	5760155	5760155	G	G	A	SNP	OS02G0202950	Exonic	CDS	NA	LOC_Os02g10860	bZIP transcription factor domain containing protein, expressed
Chr.2	5760190	5760190	G	G	A	SNP	OS02G0202950	Exonic	CDS	NA	LOC_Os02g10860	bZIP transcription factor domain containing protein, expressed
Chr.2	5760391	5760391	G	G	A	SNP	OS02G0202950	Exonic	CDS	NA	LOC_Os02g10860	bZIP transcription factor domain containing protein, expressed
Chr.2	5760433	5760433	G	G	A	SNP	OS02G0202950	Exonic	CDS	NA	LOC_Os02g10860	bZIP transcription factor domain containing protein, expressed
Chr.2	5760468	5760468	G	G	A	SNP	OS02G0202950	Exonic	CDS	NA	LOC_Os02g10860	bZIP transcription factor domain containing protein, expressed

Chr.2	6718054	6718054	C	C	A	SNP	OS02G0220500	Intronic	NA	NA	LOC_Os02g12794	elongation factor 1-gamma, putative, expressed
Chr.2	6834635	6834635	G	G	A	SNP	OS02G0222500	Exonic	CDS	NA	LOC_Os02g12939	expressed protein
Chr.2	7220694	7220694	C	C	A	SNP	OS02G0228300	Exonic	3UTR	NA	LOC_Os02g13510	receptor-like protein kinase 5 precursor, putative, expressed
Chr.2	7220757	7220757	G	G	A	SNP	OS02G0228300	Exonic	3UTR	NA	LOC_Os02g13510	receptor-like protein kinase 5 precursor, putative, expressed
Chr.2	7224453	7224453	G	G	A	SNP	OS02G0228300	Exonic	5UTR	NA	LOC_Os02g13510	receptor-like protein kinase 5 precursor, putative, expressed
Chr.2	7222248	7222248	G	G	A	SNP	OS02G0228300	Exonic	CDS	Silent	LOC_Os02g13510	receptor-like protein kinase 5 precursor, putative, expressed
Chr.2	7222491	7222491	G	G	A	SNP	OS02G0228300	Exonic	CDS	Silent	LOC_Os02g13510	receptor-like protein kinase 5 precursor, putative, expressed
Chr.2	7222509	7222509	G	G	A	SNP	OS02G0228300	Exonic	CDS	Silent	LOC_Os02g13510	receptor-like protein kinase 5 precursor, putative, expressed
Chr.2	7222551	7222551	C	C	A	SNP	OS02G0228300	Exonic	CDS	Silent	LOC_Os02g13510	receptor-like protein kinase 5 precursor, putative, expressed
Chr.2	7222620	7222620	G	G	A	SNP	OS02G0228300	Exonic	CDS	Silent	LOC_Os02g13510	receptor-like protein kinase 5 precursor, putative, expressed
Chr.2	7222623	7222623	G	G	A	SNP	OS02G0228300	Exonic	CDS	Silent	LOC_Os02g13510	receptor-like protein kinase 5 precursor, putative, expressed
Chr.2	7222695	7222695	G	G	A	SNP	OS02G0228300	Exonic	CDS	Silent	LOC_Os02g13510	receptor-like protein kinase 5 precursor, putative, expressed
Chr.2	7222859	7222859	G	G	A	SNP	OS02G0228300	Exonic	CDS	Silent	LOC_Os02g13510	receptor-like protein kinase 5 precursor, putative, expressed
Chr.2	7222968	7222968	G	G	A	SNP	OS02G0228300	Exonic	CDS	Silent	LOC_Os02g13510	receptor-like protein kinase 5 precursor, putative, expressed
Chr.2	7223085	7223085	G	G	A	SNP	OS02G0228300	Exonic	CDS	Silent	LOC_Os02g13510	receptor-like protein kinase 5 precursor, putative, expressed
Chr.2	7223502	7223502	G	G	A	SNP	OS02G0228300	Exonic	CDS	Silent	LOC_Os02g13510	receptor-like protein kinase 5 precursor, putative, expressed
Chr.2	7223550	7223550	G	G	A	SNP	OS02G0228300	Exonic	CDS	Silent	LOC_Os02g13510	receptor-like protein kinase 5 precursor, putative, expressed
Chr.2	7224039	7224039	G	G	A	SNP	OS02G0228300	Exonic	CDS	Silent	LOC_Os02g13510	receptor-like protein kinase 5 precursor, putative, expressed
Chr.2	7222460	7222460	G	G	A	SNP	OS02G0228300	Exonic	CDS	Nonsense	LOC_Os02g13510	receptor-like protein kinase 5 precursor, putative, expressed
Chr.2	7221994	7221994	G	G	A	SNP	OS02G0228300	Exonic	CDS	Missense	LOC_Os02g13510	receptor-like protein kinase 5 precursor, putative, expressed
Chr.2	7222016	7222016	T	T	A	SNP	OS02G0228300	Exonic	CDS	Missense	LOC_Os02g13510	receptor-like protein kinase 5 precursor, putative, expressed
Chr.2	7222430	7222430	G	G	A	SNP	OS02G0228300	Exonic	CDS	Missense	LOC_Os02g13510	receptor-like protein kinase 5 precursor, putative, expressed
Chr.2	7222444	7222444	C	C	A	SNP	OS02G0228300	Exonic	CDS	Missense	LOC_Os02g13510	receptor-like protein kinase 5 precursor, putative, expressed
Chr.2	7222558	7222558	G	G	A	SNP	OS02G0228300	Exonic	CDS	Missense	LOC_Os02g13510	receptor-like protein kinase 5 precursor, putative, expressed
Chr.2	7222577	7222577	G	G	A	SNP	OS02G0228300	Exonic	CDS	Missense	LOC_Os02g13510	receptor-like protein kinase 5 precursor, putative, expressed
Chr.2	7222603	7222603	G	G	A	SNP	OS02G0228300	Exonic	CDS	Missense	LOC_Os02g13510	receptor-like protein kinase 5 precursor, putative, expressed
Chr.2	7222649	7222649	C	C	A	SNP	OS02G0228300	Exonic	CDS	Missense	LOC_Os02g13510	receptor-like protein kinase 5 precursor, putative, expressed
Chr.2	7223937	7223937	C	C	A	SNP	OS02G0228300	Exonic	CDS	Missense	LOC_Os02g13510	receptor-like protein kinase 5 precursor, putative, expressed
Chr.2	7223950	7223950	G	G	A	SNP	OS02G0228300	Exonic	CDS	Missense	LOC_Os02g13510	receptor-like protein kinase 5 precursor, putative, expressed
Chr.2	7224034	7224034	C	C	A	SNP	OS02G0228300	Exonic	CDS	Missense	LOC_Os02g13510	receptor-like protein kinase 5 precursor, putative, expressed
Chr.2	7465214	7465214	C	C	A	SNP	OS02G0232000	Exonic	3UTR	NA	LOC_Os02g13800	HSF-type DNA-binding domain containing protein, expressed
Chr.2	8584037	8584037	G	G	A	SNP	OS02G0252200	Exonic	CDS	Silent	LOC_Os02g15340	no apical meristem protein, putative, expressed
Chr.2	8935417	8935417	G	G	A	SNP	OS02G0258200	Exonic	CDS	Silent	LOC_Os02g15810	HMG1/2, putative, expressed
Chr.2	9925522	9925522	G	G	A	SNP	OS02G0272900	Intronic	NA	NA	LOC_Os02g17304	resistance protein, putative, expressed
Chr.2	9976318	9976318	G	G	A	SNP	OS02G0273700	Exonic	CDS	Silent	LOC_Os02g17350	VHS and GAT domain containing protein, expressed
Chr.2	9973499	9973499	T	T	A	SNP	OS02G0273700	Intronic	NA	NA	LOC_Os02g17350	VHS and GAT domain containing protein, expressed
Chr.2	9976706	9976706	G	G	A	SNP	OS02G0273700	Intronic	NA	NA	LOC_Os02g17350	VHS and GAT domain containing protein, expressed
Chr.2	9976886	9976886	G	G	A	SNP	OS02G0273700	Intronic	NA	NA	LOC_Os02g17350	VHS and GAT domain containing protein, expressed
Chr.2	9990307	9990307	G	G	A	SNP	OS02G0273900	Exonic	CDS	Silent	LOC_Os02g17370	expressed protein
Chr.2	9990379	9990379	G	G	A	SNP	OS02G0273900	Exonic	CDS	Silent	LOC_Os02g17370	expressed protein
Chr.2	9989458	9989458	C	C	A	SNP	OS02G0273900	Intronic	NA	NA	LOC_Os02g17370	expressed protein
Chr.2	16120195	16120195	G	G	A	SNP	OS02G0473200	Exonic	CDS	Silent	LOC_Os02g27360	aspartic proteinase-like protein 2 precursor, putative, expressed
Chr.2	17160746	17160746	G	G	A	SNP	OS02G0491400	Exonic	CDS	Silent	LOC_Os02g28980	peptidyl-prolyl isomerase, putative, expressed
Chr.2	20750249	20750249	T	T	A	SNP	OS02G0551100	Intronic	NA	NA	LOC_Os02g34600	CAMK_CAMK_like.13 - CAMK includes calcium/calmodulin depedent protein kinases, expressed
Chr.2	22298160	22298160	G	G	A	SNP	OS02G0579600	Intronic	NA	NA	LOC_Os02g36924	OsMADS27 - MADS-box family gene with MIKCc type-box, expressed
Chr.2	23527550	23527550	G	G	A	SNP	OS02G0601300	Intronic	NA	NA	LOC_Os02g38920	glyceraldehyde-3-phosphate dehydrogenase, putative, expressed
Chr.2	23996146	23996146	C	C	A	SNP	OS02G0610600	Intronic	NA	NA	LOC_Os02g39720	splicing factor, arginine/serine-rich 7, putative, expressed
Chr.2	24738116	24738116	G	G	A	SNP	OS02G0621600	Intronic	NA	NA	LOC_Os02g40820	expressed protein
Chr.2	24899780	24899780	C	C	A	SNP	OS02G0624400	Exonic	CDS	Missense	LOC_Os02g41520	glycosyl transferase 8 domain containing protein, putative, expressed
Chr.2	25879570	25879570	G	G	A	SNP	OS02G0643800	Exonic	3UTR	NA	LOC_Os02g42990	OsSAUR11 - Auxin-responsive SAUR gene family member, expressed
Chr.2	32569014	32569014	C	C	A	SNP	OS02G0771700	Exonic	CDS	Missense	LOC_Os02g53200	glucan endo-1,3-beta-glucosidase precursor, putative, expressed
Chr.2	33953750	33953750	G	G	A	SNP	OS02G0797600	Exonic	CDS	Silent	LOC_Os02g55430	alginate regulatory protein AlgP, putative, expressed
Chr.2	33953897	33953897	G	G	A	SNP	OS02G0797600	Exonic	CDS	Silent	LOC_Os02g55430	alginate regulatory protein AlgP, putative, expressed
Chr.2	34351432	34351432	C	C	A	SNP	OS02G0805200	Exonic	5UTR	NA	LOC_Os02g56130	PCNA - Putative DNA replicative polymerase clamp, expressed
Chr.2	34469962	34469962	G	G	A	SNP	OS02G0807000	Exonic	CDS	Missense	LOC_Os02g56310	calcium-dependent protein kinase isoform AK1, putative, expressed
Chr.2	34612241	34612241	G	G	A	SNP	OS02G0809800	Intronic	NA	NA	LOC_Os02g56510	phosphate transporter 1, putative, expressed
Chr.2	34673834	34673834	C	C	A	SNP	OS02G0810900	Intronic	NA	NA	LOC_Os02g56600	no apical meristem protein, putative, expressed
Chr.2	35099287	35099287	G	G	A	SNP	OS02G0817900	Exonic	5UTR	NA	LOC_Os02g57290	cytochrome P450, putative, expressed
Chr.2	35098744	35098744	G	G	A	SNP	OS02G0817900	Exonic	CDS	Silent	LOC_Os02g57290	cytochrome P450, putative, expressed
Chr.2	35096605	35096605	G	G	A	SNP	OS02G0817900	Intronic	NA	NA	LOC_Os02g57290	cytochrome P450, putative, expressed
Chr.2	35097920	35097920	G	G	A	SNP	OS02G0817900	Intronic	NA	NA	LOC_Os02g57290	cytochrome P450, putative, expressed
Chr.2	35098550	35098550	G	G	A	SNP	OS02G0817900	Intronic	NA	NA	LOC_Os02g57290	cytochrome P450, putative, expressed
Chr.2	80939	80955	ATACATGCTCTGTTAAT	ATACATGCTCTGTTAAT	A	INDEL	OS02G0101500	Intronic	NA	NA	LOC_Os02g01150	erythronate-4-phosphate dehydrogenase domain containing protein, expressed
Chr.2	81362	81368	ATCTATC	ATCTATC	A	INDEL	OS02G0101500	Intronic	NA	NA	LOC_Os02g01150	erythronate-4-phosphate dehydrogenase domain containing protein, expressed
Chr.2	185213	185228	AAGGAGAGGAGAGGAG	AAGGAGAGGAGAGGAG	A	INDEL	OS02G0103800	Exonic	5UTR	NA	LOC_Os02g01340	ferredoxin--NADP reductase, chloroplast precursor, putative, expressed
Chr.2	4295881	4295883	ACT	ACT	A	INDEL	OS02G0177800	Intronic	NA	NA	LOC_Os02g08120	calmodulin binding protein, putative, expressed
Chr.2	5155942	5155955	AATATTCCGACTAT	AATATTCCGACTAT	A	INDEL	OS02G0192500	Intronic	NA	NA	LOC_Os02g09930	CSLA1 - cellulose synthase-like family A; mannan synthase, expressed
Chr.2	5156033	5156050	AACACGAGATATAGGGGG	AACACGAGATATAGGGGG	A	INDEL	OS02G0192500	Intronic	NA	NA	LOC_Os02g09930	CSLA1 - cellulose synthase-like family A; mannan synthase, expressed
Chr.2	5156468	5156481	AACATGAGAATGAT	AACATGAGAATGAT	A	INDEL	OS02G0192500	Intronic	NA	NA	LOC_Os02g09930	CSLA1 - cellulose synthase-like family A; mannan synthase, expressed
Chr.2	5250595	5250598	ACTG	ACTG	A	INDEL	OS02G0194200	Exonic	5UTR	NA	LOC_Os02g10080	zinc finger C-x8-C-x5-C-x3-H type family protein, expressed
Chr.2	5707231	5707233	ACT	ACT	A	INDEL	OS02G0202200	Intronic	NA	NA	LOC_Os02g10780	SPX domain containing protein, putative, expressed
Chr.2	6610942	6610945	AAAG	AAAG	A	INDEL	OS02G0218400	Intronic	NA	NA	LOC_Os02g12660	protein kinase domain containing protein, putative, expressed
Chr.2	7088276	7088278	ATT	ATT	A	INDEL	OS02G0226300	Intronic	NA	NA	LOC_Os02g13300	mitochondrial carrier protein, putative, expressed
Chr.2	9973519	9973527	ACCTTGAGT	ACCTTGAGT	A	INDEL	OS02G0273700	Intronic	NA	NA	LOC_Os02g17350	VHS and GAT domain containing protein, expressed
Chr.2	9976059	9976062	AAAT	AAAT	A	INDEL	OS02G0273700	Intronic	NA	NA	LOC_Os02g17350	VHS and GAT domain containing protein, expressed
Chr.2	9989759	9989760	AG	AG	A	INDEL	OS02G0273900	Intronic	NA	NA	LOC_Os02g17370	expressed protein
Chr.2	9990098	9990099	AT	AT	A	INDEL	OS02G0273900	Intronic	NA	NA	LOC_Os02g17370	expressed protein
Chr.2	14961651	14961660	ACAGCAGCAT	ACAGCAGCAT	A	INDEL	OS02G0452500	Intronic	NA	NA	LOC_Os02g25580	Sec1 family transport protein, putative, expressed
Chr.2	28780266	28780268	ACT	ACT	A	INDEL	OS02G0699700	Intronic	NA	NA	LOC_Os02g47150	DNA topoisomerase 2, putative, expressed
Chr.2	30458930	30458931	AT	AT	A	INDEL	OS02G0731200	Intronic	NA	NA	LOC_Os02g49840	OsMADS57 - MADS-box family gene with MIKCc type-box, expressed
Chr.2	31269093	31269095	AGG	AGG	A	INDEL	OS02G0745100	Intronic	NA	NA	LOC_Os02g51110	aquaporin protein, putative, expressed
Chr.2	34612686	34612689	ACTT	ACTT	A	INDEL	OS02G0809800	Intronic	NA	NA	LOC_Os02g56510	phosphate transporter 1, putative, expressed
Chr.2	35098839	35098840	AG	AG	A	INDEL	OS02G0817900	Intronic	NA	NA	LOC_Os02g57290	cytochrome P450, putative, expressed
Chr.12	9918317	9918317	T	T	A	SNP	OS12G0271700	Intronic	NA	NA	LOC_Os12g17320	polyprenyl synthetase, putative, expressed
Chr.12	9919656	9919656	T	T	A	SNP	OS12G0271700	Intronic	NA	NA	LOC_Os12g17320	polyprenyl synthetase, putative, expressed

Chr.12	9919672	9919672	T	T	A	SNP	OS12G0271700	Intronic	NA	NA	LOC_Os12g17320	polyprenyl synthetase, putative, expressed
Chr.12	12987881	12987881	T	T	A	SNP	OS12G0418600	Intronic	NA	NA	LOC_Os12g22970	expressed protein
Chr.12	12987892	12987892	T	T	A	SNP	OS12G0418600	Intronic	NA	NA	LOC_Os12g22970	expressed protein
Chr.12	12987893	12987893	T	T	A	SNP	OS12G0418600	Intronic	NA	NA	LOC_Os12g22970	expressed protein
Chr.12	23052069	23052069	T	T	A	SNP	OS12G0562400	Intronic	NA	NA	LOC_Os12g37560	phospholipase C, putative, expressed
Chr.12	25651828	25651828	T	T	A	SNP	OS12G0607100	Exonic	3UTR	NA	LOC_Os12g41400	eukaryotic translation initiation factor 2 subunit gamma, putative, expressed
Chr.12	25651520	25651520	T	T	A	SNP	OS12G0607100	Exonic	CDS	Silent	LOC_Os12g41400	eukaryotic translation initiation factor 2 subunit gamma, putative, expressed
Chr.12	25651090	25651090	T	T	A	SNP	OS12G0607100	Intronic	NA	NA	LOC_Os12g41400	eukaryotic translation initiation factor 2 subunit gamma, putative, expressed
Chr.12	3754100	3754100	G	G	A	SNP	OS12G0174100	Intronic	NA	NA	LOC_Os12g07540	growth regulator related protein, putative, expressed
Chr.12	9352912	9352912	G	G	A	SNP	OS12G0264500	Intronic	NA	NA	LOC_Os12g16350	enoyl-CoA hydratase/isomerase family protein, putative, expressed
Chr.12	9353003	9353003	G	G	A	SNP	OS12G0264500	Intronic	NA	NA	LOC_Os12g16350	enoyl-CoA hydratase/isomerase family protein, putative, expressed
Chr.12	9353231	9353231	G	G	A	SNP	OS12G0264500	Intronic	NA	NA	LOC_Os12g16350	enoyl-CoA hydratase/isomerase family protein, putative, expressed
Chr.12	9919663	9919663	G	G	A	SNP	OS12G0271700	Intronic	NA	NA	LOC_Os12g17320	polyprenyl synthetase, putative, expressed
Chr.12	12987883	12987883	G	G	A	SNP	OS12G0418600	Intronic	NA	NA	LOC_Os12g22970	expressed protein
Chr.12	12987891	12987891	G	G	A	SNP	OS12G0418600	Intronic	NA	NA	LOC_Os12g22970	expressed protein
Chr.12	12987908	12987908	G	G	A	SNP	OS12G0418600	Intronic	NA	NA	LOC_Os12g22970	expressed protein
Chr.12	22136540	22136540	G	G	A	SNP	OS12G0547600	Intronic	NA	NA	LOC_Os12g36110	calmodulin binding protein, putative, expressed
Chr.12	22592206	22592206	G	G	A	SNP	OS12G0555200	Intronic	NA	NA	LOC_Os12g36850	pathogenesis-related Bet v I family protein, putative, expressed
Chr.12	22592221	22592221	G	G	A	SNP	OS12G0555200	Intronic	NA	NA	LOC_Os12g36850	pathogenesis-related Bet v I family protein, putative, expressed
Chr.12	22592433	22592433	G	G	A	SNP	OS12G0555200	Intronic	NA	NA	LOC_Os12g36850	pathogenesis-related Bet v I family protein, putative, expressed
Chr.12	22592442	22592442	G	G	A	SNP	OS12G0555200	Intronic	NA	NA	LOC_Os12g36850	pathogenesis-related Bet v I family protein, putative, expressed
Chr.12	23501941	23501941	G	G	A	SNP	OS12G0570700	Intronic	NA	NA	LOC_Os12g38270	metallothionein, putative, expressed
Chr.12	24872311	24872311	G	G	A	SNP	OS12G0592900	Exonic	3UTR	NA	LOC_Os12g40180	expressed protein
Chr.12	25651559	25651559	G	G	A	SNP	OS12G0607100	Exonic	CDS	Silent	LOC_Os12g41400	eukaryotic translation initiation factor 2 subunit gamma, putative, expressed
Chr.12	25648708	25648708	G	G	A	SNP	OS12G0607100	Intronic	NA	NA	LOC_Os12g41400	eukaryotic translation initiation factor 2 subunit gamma, putative, expressed
Chr.12	25650816	25650816	G	G	A	SNP	OS12G0607100	Intronic	NA	NA	LOC_Os12g41400	eukaryotic translation initiation factor 2 subunit gamma, putative, expressed
Chr.12	25651475	25651475	G	G	A	SNP	OS12G0607100	Intronic	NA	NA	LOC_Os12g41400	eukaryotic translation initiation factor 2 subunit gamma, putative, expressed
Chr.12	25651833	25651833	C	C	A	SNP	OS12G0607100	Exonic	3UTR	NA	LOC_Os12g41400	eukaryotic translation initiation factor 2 subunit gamma, putative, expressed
Chr.12	26007790	26007790	C	C	A	SNP	OS12G0613700	Exonic	5UTR	NA	LOC_Os12g41950	auxin response factor, putative, expressed
Chr.12	25326502	25326502	T	T	A	SNP	OS12G0601400	Intronic	NA	NA	LOC_Os12g40900	OsIAA31 - Auxin-responsive Aux/IAA gene family member, expressed
Chr.12	9919089	9919089	G	G	A	SNP	OS12G0271700	Intronic	NA	NA	LOC_Os12g17320	polyprenyl synthetase, putative, expressed
Chr.12	23806322	23806322	G	G	A	SNP	OS12G0576600	Intronic	NA	NA	LOC_Os12g38750	nucleotide pyrophosphatase/phosphodiesterase, putative, expressed
Chr.12	24184998	24184998	G	G	A	SNP	OS12G0582700	Intronic	NA	NA	LOC_Os12g39310	cytochrome P450, putative, expressed
Chr.12	22592101	22592102	AG	AG	A	INDEL	OS12G0555200	Intronic	NA	NA	LOC_Os12g36850	pathogenesis-related Bet v I family protein, putative, expressed
Chr.12	25326306	25326307	AT	AT	A	INDEL	OS12G0601400	Intronic	NA	NA	LOC_Os12g40900	OsIAA31 - Auxin-responsive Aux/IAA gene family member, expressed
Chr.12	728089	728089	C	C	A	SNP	OS12G0114800	Exonic	CDS	Silent	LOC_Os12g02300	LTPL26 - Protease inhibitor/seed storage/LTP family protein precursor, expressed
Chr.12	5675425	5675425	G	G	A	SNP	OS12G0208900	Exonic	5UTR	NA	LOC_Os12g10630	ZF-HD protein dimerisation region containing protein, expressed
Chr.12	9353462	9353462	G	G	A	SNP	OS12G0264500	Intronic	NA	NA	LOC_Os12g16350	enoyl-CoA hydratase/isomerase family protein, putative, expressed
Chr.12	9918748	9918748	T	T	A	SNP	OS12G0271700	Intronic	NA	NA	LOC_Os12g17320	polyprenyl synthetase, putative, expressed
Chr.12	18339793	18339793	G	G	A	SNP	OS12G0489100	Intronic	NA	NA	LOC_Os12g30540	ubiquitin carboxyl-terminal hydrolase, putative, expressed
Chr.12	23420342	23420342	G	G	A	SNP	OS12G0569200	Exonic	CDS	Missense	LOC_Os12g38140	expressed protein
Chr.12	25445177	25445177	G	G	A	SNP	OS12G0603700	Exonic	CDS	Silent	LOC_Os12g41090	CAMK_KIN1/SNF1/Nim1_like.38 - CAMK includes calcium/calmodulin depedent protein kinases, expressed
Chr.12	25445844	25445844	G	G	A	SNP	OS12G0603700	Exonic	CDS	Missense	LOC_Os12g41090	CAMK_KIN1/SNF1/Nim1_like.38 - CAMK includes calcium/calmodulin depedent protein kinases, expressed
Chr.12	25446216	25446216	G	G	A	SNP	OS12G0603700	Exonic	CDS	Missense	LOC_Os12g41090	CAMK_KIN1/SNF1/Nim1_like.38 - CAMK includes calcium/calmodulin depedent protein kinases, expressed
Chr.12	25648115	25648115	G	G	A	SNP	OS12G0607100	Intronic	NA	NA	LOC_Os12g41400	eukaryotic translation initiation factor 2 subunit gamma, putative, expressed
Chr.12	25648143	25648143	G	G	A	SNP	OS12G0607100	Intronic	NA	NA	LOC_Os12g41400	eukaryotic translation initiation factor 2 subunit gamma, putative, expressed
Chr.12	25648226	25648226	T	T	A	SNP	OS12G0607100	Intronic	NA	NA	LOC_Os12g41400	eukaryotic translation initiation factor 2 subunit gamma, putative, expressed
Chr.12	27048446	27048446	G	G	A	SNP	OS12G0631600	Intronic	NA	NA	LOC_Os12g43580	expressed protein
Chr.12	5676270	5676271	AC	AC	A	INDEL	OS12G0208900	Exonic	CDS	FrameShift	LOC_Os12g10630	ZF-HD protein dimerisation region containing protein, expressed
Chr.12	22198204	22198208	ACCGG	ACCGG	A	INDEL	OS12G0548501	Exonic	CDS	FrameShift	LOC_Os12g36220	inhibitor I family protein, putative, expressed
Chr.12	25651855	25651856	AG	AG	A	INDEL	OS12G0607100	Exonic	3UTR	NA	LOC_Os12g41400	eukaryotic translation initiation factor 2 subunit gamma, putative, expressed
Chr.12	27061032	27061035	AACT	AACT	A	INDEL	OS12G0632000	Exonic	3UTR	NA	LOC_Os12g43600	RNA recognition motif containing protein, expressed
Chr.11	2762784	2762784	T	T	A	SNP	OS11G0157400	Intronic	NA	NA	LOC_Os11g05880	exo70 exocyst complex subunit, putative, expressed
Chr.11	4251282	4251282	T	T	A	SNP	OS11G0183900	Exonic	3UTR	NA	LOC_Os11g08100	eukaryotic aspartyl protease domain containing protein, expressed
Chr.11	4249052	4249052	T	T	A	SNP	OS11G0183900	Intronic	NA	NA	LOC_Os11g08100	eukaryotic aspartyl protease domain containing protein, expressed
Chr.11	4249490	4249490	T	T	A	SNP	OS11G0183900	Intronic	NA	NA	LOC_Os11g08100	eukaryotic aspartyl protease domain containing protein, expressed
Chr.11	4251091	4251091	T	T	A	SNP	OS11G0183900	Intronic	NA	NA	LOC_Os11g08100	eukaryotic aspartyl protease domain containing protein, expressed
Chr.11	6639528	6639528	T	T	A	SNP	OS11G0226933	Exonic	CDS	NA	LOC_Os11g11960	disease resistance protein RPM1, putative, expressed
Chr.11	14048989	14048989	T	T	A	SNP	OS11G0434000	Intronic	NA	NA	LOC_Os11g24630	magnesium-dependent phosphatase 1, putative, expressed
Chr.11	14049005	14049005	T	T	A	SNP	OS11G0434000	Intronic	NA	NA	LOC_Os11g24630	magnesium-dependent phosphatase 1, putative, expressed
Chr.11	14049011	14049011	T	T	A	SNP	OS11G0434000	Intronic	NA	NA	LOC_Os11g24630	magnesium-dependent phosphatase 1, putative, expressed
Chr.11	14050942	14050942	T	T	A	SNP	OS11G0434000	Intronic	NA	NA	LOC_Os11g24630	magnesium-dependent phosphatase 1, putative, expressed
Chr.11	15421908	15421908	T	T	A	SNP	OS11G0455500	Intronic	NA	NA	LOC_Os11g26850	erythronate-4-phosphate dehydrogenase, putative, expressed
Chr.11	17353544	17353544	T	T	A	SNP	OS11G0490900	Intronic	NA	NA	LOC_Os11g29870	WRKY72, expressed
Chr.11	17353605	17353605	T	T	A	SNP	OS11G0490900	Intronic	NA	NA	LOC_Os11g29870	WRKY72, expressed
Chr.11	17353637	17353637	T	T	A	SNP	OS11G0490900	Intronic	NA	NA	LOC_Os11g29870	WRKY72, expressed
Chr.11	17353690	17353690	T	T	A	SNP	OS11G0490900	Intronic	NA	NA	LOC_Os11g29870	WRKY72, expressed
Chr.11	17354209	17354209	T	T	A	SNP	OS11G0490900	Intronic	NA	NA	LOC_Os11g29870	WRKY72, expressed
Chr.11	17354546	17354546	T	T	A	SNP	OS11G0490900	Intronic	NA	NA	LOC_Os11g29870	WRKY72, expressed
Chr.11	17355094	17355094	T	T	A	SNP	OS11G0490900	Intronic	NA	NA	LOC_Os11g29870	WRKY72, expressed
Chr.11	18832311	18832311	T	T	A	SNP	OS11G0522900	Intronic	NA	NA	LOC_Os11g31980	OsSCP63 - Putative Serine Carboxypeptidase homologue, expressed
Chr.11	18834251	18834251	T	T	A	SNP	OS11G0522900	Intronic	NA	NA	LOC_Os11g31980	OsSCP63 - Putative Serine Carboxypeptidase homologue, expressed
Chr.11	18937366	18937366	T	T	A	SNP	OS11G0523800	Exonic	3UTR	NA	LOC_Os11g32110	auxin response factor, putative, expressed
Chr.11	18936229	18936229	T	T	A	SNP	OS11G0523800	Intronic	NA	NA	LOC_Os11g32110	auxin response factor, putative, expressed
Chr.11	18939367	18939367	T	T	A	SNP	OS11G0523800	Intronic	NA	NA	LOC_Os11g32110	auxin response factor, putative, expressed
Chr.11	19675678	19675678	T	T	A	SNP	OS11G0539200	Intronic	NA	NA	LOC_Os11g33270	glycosyl hydrolases family 16, putative, expressed
Chr.11	21298221	21298221	T	T	A	SNP	OS11G0570000	Exonic	CDS	Missense	LOC_Os11g36200	receptor-like protein kinase 2 precursor, putative, expressed
Chr.11	23233954	23233954	T	T	A	SNP	OS11G0603200	Exonic	3UTR	NA	LOC_Os11g39020	ABC transporter, ATP-binding protein, putative, expressed
Chr.11	23908039	23908039	T	T	A	SNP	OS11G0615000	Intronic	NA	NA	LOC_Os11g40080	lipin, N-terminal conserved region family protein, expressed
Chr.11	26313550	26313550	T	T	A	SNP	OS11G0657300	Intronic	NA	NA	LOC_Os11g43620	LSM domain containing protein, expressed
Chr.11	27488238	27488238	T	T	A	SNP	OS11G0679700	Intronic	NA	NA	LOC_Os11g45400	glycerol-3-phosphate acyltransferase, putative, expressed
Chr.11	28854126	28854126	T	T	A	SNP	OS11G0704800	Intronic	NA	NA	LOC_Os11g47840	OsRhmbd18 - Putative Rhomboid homologue, expressed
Chr.11	1226401	1226401	G	G	A	SNP	OS11G0126800	Exonic	CDS	NA	LOC_Os11g03290	nucleoside-triphosphatase, putative, expressed
Chr.11	1226460	1226460	G	G	A	SNP	OS11G0126800	Exonic	CDS	NA	LOC_Os11g03290	nucleoside-triphosphatase, putative, expressed
Chr.11	2762557	2762557	G	G	A	SNP	OS11G0157400	Exonic	CDS	Silent	LOC_Os11g05880	exo70 exocyst complex subunit, putative, expressed

Chr.11	2760469	2760469	G	G	A	SNP	OS11G0157400	Intronic	NA	NA	LOC_Os11g05880	exo70 exocyst complex subunit, putative, expressed
Chr.11	4251338	4251338	G	G	A	SNP	OS11G0183900	Exonic	3UTR	NA	LOC_Os11g08100	eukaryotic aspartyl protease domain containing protein, expressed
Chr.11	4251348	4251348	G	G	A	SNP	OS11G0183900	Exonic	3UTR	NA	LOC_Os11g08100	eukaryotic aspartyl protease domain containing protein, expressed
Chr.11	4251607	4251607	G	G	A	SNP	OS11G0183900	Exonic	3UTR	NA	LOC_Os11g08100	eukaryotic aspartyl protease domain containing protein, expressed
Chr.11	4249662	4249662	G	G	A	SNP	OS11G0183900	Exonic	CDS	Silent	LOC_Os11g08100	eukaryotic aspartyl protease domain containing protein, expressed
Chr.11	4249185	4249185	G	G	A	SNP	OS11G0183900	Intronic	NA	NA	LOC_Os11g08100	eukaryotic aspartyl protease domain containing protein, expressed
Chr.11	4251088	4251088	G	G	A	SNP	OS11G0183900	Intronic	NA	NA	LOC_Os11g08100	eukaryotic aspartyl protease domain containing protein, expressed
Chr.11	6639391	6639391	G	G	A	SNP	OS11G0226933	Exonic	CDS	NA	LOC_Os11g11960	disease resistance protein RPM1, putative, expressed
Chr.11	6639427	6639427	G	G	A	SNP	OS11G0226933	Exonic	CDS	NA	LOC_Os11g11960	disease resistance protein RPM1, putative, expressed
Chr.11	6639664	6639664	G	G	A	SNP	OS11G0226933	Exonic	CDS	NA	LOC_Os11g11960	disease resistance protein RPM1, putative, expressed
Chr.11	6640264	6640264	G	G	A	SNP	OS11G0226933	Exonic	CDS	NA	LOC_Os11g11960	disease resistance protein RPM1, putative, expressed
Chr.11	6650127	6650127	G	G	A	SNP	OS11G0227000	Exonic	3UTR	NA	LOC_Os11g11980	expressed protein
Chr.11	6649868	6649868	G	G	A	SNP	OS11G0227000	Exonic	CDS	Missense	LOC_Os11g11980	expressed protein
Chr.11	9058748	9058748	G	G	A	SNP	OS11G0265200	Intronic	NA	NA	LOC_Os11g16390	expressed protein
Chr.11	9187762	9187762	G	G	A	SNP	OS11G0267300	Exonic	5UTR	NA	LOC_Os11g16580	endonuclease III-like protein 1, putative, expressed
Chr.11	9189010	9189010	G	G	A	SNP	OS11G0267300	Intronic	NA	NA	LOC_Os11g16580	endonuclease III-like protein 1, putative, expressed
Chr.11	14049104	14049104	G	G	A	SNP	OS11G0434000	Exonic	CDS	Missense	LOC_Os11g24630	magnesium-dependent phosphatase 1, putative, expressed
Chr.11	14048844	14048844	G	G	A	SNP	OS11G0434000	Intronic	NA	NA	LOC_Os11g24630	magnesium-dependent phosphatase 1, putative, expressed
Chr.11	14048972	14048972	G	G	A	SNP	OS11G0434000	Intronic	NA	NA	LOC_Os11g24630	magnesium-dependent phosphatase 1, putative, expressed
Chr.11	14049279	14049279	G	G	A	SNP	OS11G0434000	Intronic	NA	NA	LOC_Os11g24630	magnesium-dependent phosphatase 1, putative, expressed
Chr.11	14049648	14049648	G	G	A	SNP	OS11G0434000	Intronic	NA	NA	LOC_Os11g24630	magnesium-dependent phosphatase 1, putative, expressed
Chr.11	14050832	14050832	G	G	A	SNP	OS11G0434000	Intronic	NA	NA	LOC_Os11g24630	magnesium-dependent phosphatase 1, putative, expressed
Chr.11	14050842	14050842	G	G	A	SNP	OS11G0434000	Intronic	NA	NA	LOC_Os11g24630	magnesium-dependent phosphatase 1, putative, expressed
Chr.11	14051022	14051022	G	G	A	SNP	OS11G0434000	Intronic	NA	NA	LOC_Os11g24630	magnesium-dependent phosphatase 1, putative, expressed
Chr.11	14051035	14051035	G	G	A	SNP	OS11G0434000	Intronic	NA	NA	LOC_Os11g24630	magnesium-dependent phosphatase 1, putative, expressed
Chr.11	14052029	14052029	G	G	A	SNP	OS11G0434000	Intronic	NA	NA	LOC_Os11g24630	magnesium-dependent phosphatase 1, putative, expressed
Chr.11	17236708	17236708	G	G	A	SNP	OS11G0489250	Intronic	NA	NA	LOC_Os11g29720	cytochrome P450, putative, expressed
Chr.11	17236740	17236740	G	G	A	SNP	OS11G0489250	Intronic	NA	NA	LOC_Os11g29720	cytochrome P450, putative, expressed
Chr.11	17353169	17353169	G	G	A	SNP	OS11G0490900	Intronic	NA	NA	LOC_Os11g29870	WRKY72, expressed
Chr.11	17353575	17353575	G	G	A	SNP	OS11G0490900	Intronic	NA	NA	LOC_Os11g29870	WRKY72, expressed
Chr.11	17353680	17353680	G	G	A	SNP	OS11G0490900	Intronic	NA	NA	LOC_Os11g29870	WRKY72, expressed
Chr.11	17354147	17354147	G	G	A	SNP	OS11G0490900	Intronic	NA	NA	LOC_Os11g29870	WRKY72, expressed
Chr.11	17354791	17354791	G	G	A	SNP	OS11G0490900	Intronic	NA	NA	LOC_Os11g29870	WRKY72, expressed
Chr.11	17355034	17355034	G	G	A	SNP	OS11G0490900	Intronic	NA	NA	LOC_Os11g29870	WRKY72, expressed
Chr.11	18834914	18834914	G	G	A	SNP	OS11G0522900	Exonic	CDS	Silent	LOC_Os11g31980	OsSCP63 - Putative Serine Carboxypeptidase homologue, expressed
Chr.11	18936070	18936070	G	G	A	SNP	OS11G0523800	Exonic	3UTR	NA	LOC_Os11g32110	auxin response factor, putative, expressed
Chr.11	18940078	18940078	G	G	A	SNP	OS11G0523800	Exonic	CDS	Missense	LOC_Os11g32110	auxin response factor, putative, expressed
Chr.11	19677053	19677053	G	G	A	SNP	OS11G0539200	Intronic	NA	NA	LOC_Os11g33270	glycosyl hydrolases family 16, putative, expressed
Chr.11	19677270	19677270	G	G	A	SNP	OS11G0539200	Intronic	NA	NA	LOC_Os11g33270	glycosyl hydrolases family 16, putative, expressed
Chr.11	21299709	21299709	G	G	A	SNP	OS11G0570000	Exonic	3UTR	NA	LOC_Os11g36200	receptor-like protein kinase 2 precursor, putative, expressed
Chr.11	21300219	21300219	G	G	A	SNP	OS11G0570000	Exonic	3UTR	NA	LOC_Os11g36200	receptor-like protein kinase 2 precursor, putative, expressed
Chr.11	22273258	22273258	G	G	A	SNP	OS11G0587600	Exonic	3UTR	NA	LOC_Os11g37700	pleiotropic drug resistance protein, putative, expressed
Chr.11	22275750	22275750	G	G	A	SNP	OS11G0587600	Exonic	3UTR	NA	LOC_Os11g37700	pleiotropic drug resistance protein, putative, expressed
Chr.11	22281325	22281325	G	G	A	SNP	OS11G0587600	Intronic	NA	NA	LOC_Os11g37700	pleiotropic drug resistance protein, putative, expressed
Chr.11	22502444	22502444	G	G	A	SNP	OS11G0592000	Intronic	NA	NA	LOC_Os11g37950	WIP3 - Wound-induced protein precursor, expressed
Chr.11	23079651	23079651	G	G	A	SNP	OS11G0600900	Exonic	CDS	NA	LOC_Os11g38810	mannose-6-phosphate isomerase, putative, expressed
Chr.11	23079783	23079783	G	G	A	SNP	OS11G0600900	Intronic	NA	NA	LOC_Os11g38810	mannose-6-phosphate isomerase, putative, expressed
Chr.11	23907156	23907156	G	G	A	SNP	OS11G0615000	Intronic	NA	NA	LOC_Os11g40080	lipin, N-terminal conserved region family protein, expressed
Chr.11	25425210	25425210	G	G	A	SNP	OS11G0641500	Exonic	CDS	Silent	LOC_Os11g42200	laccase precursor protein, putative, expressed
Chr.11	25424294	25424294	G	G	A	SNP	OS11G0641500	Intronic	NA	NA	LOC_Os11g42200	laccase precursor protein, putative, expressed
Chr.11	26313471	26313471	G	G	A	SNP	OS11G0657300	Intronic	NA	NA	LOC_Os11g43620	LSM domain containing protein, expressed
Chr.11	26511063	26511063	G	G	A	SNP	OS11G0660500	Exonic	3UTR	NA	LOC_Os11g43900	translationally-controlled tumor protein, putative, expressed
Chr.11	26879392	26879392	G	G	A	SNP	OS11G0666200	Intronic	NA	NA	LOC_Os11g44430	protein kinase, putative, expressed
Chr.11	27489991	27489991	G	G	A	SNP	OS11G0679700	Exonic	CDS	NA	LOC_Os11g45400	glycerol-3-phosphate acyltransferase, putative, expressed
Chr.11	28120929	28120929	G	G	A	SNP	OS11G0691100	Exonic	CDS	Silent	LOC_Os11g46860	wall-associated receptor kinase-like 4 precursor, putative, expressed
Chr.11	28955700	28955700	G	G	A	SNP	OS11G0707600	Intronic	NA	NA	LOC_Os11g48020	fatty acid hydroxylase, putative, expressed
Chr.11	2761007	2761007	C	C	A	SNP	OS11G0157400	Intronic	NA	NA	LOC_Os11g05880	exo70 exocyst complex subunit, putative, expressed
Chr.11	4974033	4974033	C	C	A	SNP	OS11G0199200	Intronic	NA	NA	LOC_Os11g09280	OsPDIL1-1 protein disulfide isomerase PDIL1-1, expressed
Chr.11	4974267	4974267	C	C	A	SNP	OS11G0199200	Intronic	NA	NA	LOC_Os11g09280	OsPDIL1-1 protein disulfide isomerase PDIL1-1, expressed
Chr.11	5565964	5565964	C	C	A	SNP	OS11G0208600	Exonic	3UTR	NA	LOC_Os11g10250	expressed protein
Chr.11	6639556	6639556	C	C	A	SNP	OS11G0226933	Exonic	CDS	NA	LOC_Os11g11960	disease resistance protein RPM1, putative, expressed
Chr.11	9058141	9058141	C	C	A	SNP	OS11G0265200	Intronic	NA	NA	LOC_Os11g16390	expressed protein
Chr.11	10134484	10134484	C	C	A	SNP	OS11G0283500	Intronic	NA	NA	LOC_Os11g18044	peptide transporter PTR2, putative, expressed
Chr.11	14051017	14051017	C	C	A	SNP	OS11G0434000	Intronic	NA	NA	LOC_Os11g24630	magnesium-dependent phosphatase 1, putative, expressed
Chr.11	16938044	16938044	C	C	A	SNP	OS11G0482200	Intronic	NA	NA	LOC_Os11g29210	resistance-gene-interacting protein, putative, expressed
Chr.11	17352325	17352325	C	C	A	SNP	OS11G0490900	Exonic	5UTR	NA	LOC_Os11g29870	WRKY72, expressed
Chr.11	17352882	17352882	C	C	A	SNP	OS11G0490900	Intronic	NA	NA	LOC_Os11g29870	WRKY72, expressed
Chr.11	17353530	17353530	C	C	A	SNP	OS11G0490900	Intronic	NA	NA	LOC_Os11g29870	WRKY72, expressed
Chr.11	17354847	17354847	C	C	A	SNP	OS11G0490900	Intronic	NA	NA	LOC_Os11g29870	WRKY72, expressed
Chr.11	19676855	19676855	C	C	A	SNP	OS11G0539200	Intronic	NA	NA	LOC_Os11g33270	glycosyl hydrolases family 16, putative, expressed
Chr.11	21301210	21301210	C	C	A	SNP	OS11G0570000	Exonic	3UTR	NA	LOC_Os11g36200	receptor-like protein kinase 2 precursor, putative, expressed
Chr.11	27488266	27488266	C	C	A	SNP	OS11G0679700	Intronic	NA	NA	LOC_Os11g45400	glycerol-3-phosphate acyltransferase, putative, expressed
Chr.11	28854165	28854165	C	C	A	SNP	OS11G0704800	Exonic	CDS	Silent	LOC_Os11g47840	OsRhmbd18 - Putative Rhomboid homologue, expressed
Chr.11	759649	759649	T	T	A	SNP	OS11G0116900	Exonic	3UTR	NA	LOC_Os11g02480	WRKY46, expressed
Chr.11	2424380	2424380	T	T	A	SNP	OS11G0151700	Intronic	NA	NA	LOC_Os11g05400	Ser/Thr protein phosphatase family protein, putative, expressed
Chr.11	2612417	2612417	T	T	A	SNP	OS11G0155600	Intronic	NA	NA	LOC_Os11g05700	ABC transporter family protein, putative, expressed
Chr.11	22176828	22176828	T	T	A	SNP	OS11G0586300	Intronic	NA	NA	LOC_Os11g37560	expressed protein
Chr.11	26511767	26511767	T	T	A	SNP	OS11G0660500	Intronic	NA	NA	LOC_Os11g43900	translationally-controlled tumor protein, putative, expressed
Chr.11	1225591	1225591	G	G	A	SNP	OS11G0126800	Intronic	NA	NA	LOC_Os11g03290	nucleoside-triphosphatase, putative, expressed
Chr.11	2423636	2423636	G	G	A	SNP	OS11G0151700	Exonic	3UTR	NA	LOC_Os11g05400	Ser/Thr protein phosphatase family protein, putative, expressed
Chr.11	2423747	2423747	G	G	A	SNP	OS11G0151700	Exonic	3UTR	NA	LOC_Os11g05400	Ser/Thr protein phosphatase family protein, putative, expressed
Chr.11	2424219	2424219	G	G	A	SNP	OS11G0151700	Intronic	NA	NA	LOC_Os11g05400	Ser/Thr protein phosphatase family protein, putative, expressed
Chr.11	2424344	2424344	G	G	A	SNP	OS11G0151700	Intronic	NA	NA	LOC_Os11g05400	Ser/Thr protein phosphatase family protein, putative, expressed
Chr.11	2606828	2606828	G	G	A	SNP	OS11G0155600	Exonic	3UTR	NA	LOC_Os11g05700	ABC transporter family protein, putative, expressed
Chr.11	2607951	2607951	G	G	A	SNP	OS11G0155600	Exonic	3UTR	NA	LOC_Os11g05700	ABC transporter family protein, putative, expressed

Chr.11	2612881	2612881	G	G	A	SNP	OS11G0155600	Exonic	CDS	Silent	LOC_Os11g05700	ABC transporter family protein, putative, expressed
Chr.11	2613803	2613803	G	G	A	SNP	OS11G0155600	Intronic	NA	NA	LOC_Os11g05700	ABC transporter family protein, putative, expressed
Chr.11	6649597	6649597	G	G	A	SNP	OS11G0227000	Intronic	NA	NA	LOC_Os11g11980	expressed protein
Chr.11	8389504	8389504	G	G	A	SNP	OS11G0255500	Exonic	CDS	Silent	LOC_Os11g14910	NADP-dependent oxidoreductase, putative, expressed
Chr.11	10867760	10867760	G	G	A	SNP	OS11G0295900	Exonic	CDS	Silent	LOC_Os11g19060	BABY BOOM 1, putative, expressed
Chr.11	22175740	22175740	G	G	A	SNP	OS11G0586300	Exonic	CDS	NA	LOC_Os11g37560	expressed protein
Chr.11	22177450	22177450	G	G	A	SNP	OS11G0586300	Exonic	CDS	NA	LOC_Os11g37560	expressed protein
Chr.11	22176653	22176653	G	G	A	SNP	OS11G0586300	Intronic	NA	NA	LOC_Os11g37560	expressed protein
Chr.11	26512562	26512562	G	G	A	SNP	OS11G0660500	Intronic	NA	NA	LOC_Os11g43900	translationally-controlled tumor protein, putative, expressed
Chr.11	27405120	27405120	G	G	A	SNP	OS11G0678000	Exonic	3UTR	NA	LOC_Os11g45280	protein kinase family protein, putative, expressed
Chr.11	762434	762434	C	C	A	SNP	OS11G0116900	Intronic	NA	NA	LOC_Os11g02480	WRKY46, expressed
Chr.11	2424451	2424451	C	C	A	SNP	OS11G0151700	Intronic	NA	NA	LOC_Os11g05400	Ser/Thr protein phosphatase family protein, putative, expressed
Chr.11	7963277	7963277	C	C	A	SNP	OS11G0247300	Exonic	3UTR	NA	LOC_Os11g14220	tubulin/FtsZ domain containing protein, putative, expressed
Chr.11	7963348	7963348	C	C	A	SNP	OS11G0247300	Exonic	3UTR	NA	LOC_Os11g14220	tubulin/FtsZ domain containing protein, putative, expressed
Chr.11	8388930	8388930	C	C	A	SNP	OS11G0255500	Intronic	NA	NA	LOC_Os11g14910	NADP-dependent oxidoreductase, putative, expressed
Chr.11	8445445	8445445	C	C	A	SNP	OS11G0256200	Intronic	NA	NA	LOC_Os11g14990	expressed protein
Chr.11	22176845	22176845	C	C	A	SNP	OS11G0586300	Intronic	NA	NA	LOC_Os11g37560	expressed protein
Chr.11	10865570	10865572	AAC	AAC	A	INDEL	OS11G0295900	Intronic	NA	NA	LOC_Os11g19060	BABY BOOM 1, putative, expressed
Chr.11	2424683	2424683	G	G	A	SNP	OS11G0151700	Exonic	CDS	NA	LOC_Os11g05400	Ser/Thr protein phosphatase family protein, putative, expressed
Chr.11	2424646	2424646	G	G	A	SNP	OS11G0151700	Intronic	NA	NA	LOC_Os11g05400	Ser/Thr protein phosphatase family protein, putative, expressed
Chr.11	2762008	2762008	T	T	A	SNP	OS11G0157400	Intronic	NA	NA	LOC_Os11g05880	exo70 exocyst complex subunit, putative, expressed
Chr.11	3672790	3672790	C	C	A	SNP	OS11G0174000	Exonic	CDS	Silent	LOC_Os11g07280	coatomer subunit beta-1, putative, expressed
Chr.11	4248995	4248995	G	G	A	SNP	OS11G0183900	Intronic	NA	NA	LOC_Os11g08100	eukaryotic aspartyl protease domain containing protein, expressed
Chr.11	4971713	4971713	G	G	A	SNP	OS11G0199200	Exonic	CDS	Silent	LOC_Os11g09280	OsPDIL1-1 protein disulfide isomerase PDIL1-1, expressed
Chr.11	6649509	6649509	G	G	A	SNP	OS11G0227000	Exonic	CDS	Missense	LOC_Os11g11980	expressed protein
Chr.11	6649794	6649794	C	C	A	SNP	OS11G0227000	Exonic	CDS	Missense	LOC_Os11g11980	expressed protein
Chr.11	6649608	6649608	G	G	A	SNP	OS11G0227000	Intronic	NA	NA	LOC_Os11g11980	expressed protein
Chr.11	7552411	7552411	G	G	A	SNP	OS11G0241200	Exonic	CDS	Silent	LOC_Os11g13720	expressed protein
Chr.11	9059315	9059315	G	G	A	SNP	OS11G0265200	Exonic	5UTR	NA	LOC_Os11g16390	expressed protein
Chr.11	9057515	9057515	T	T	A	SNP	OS11G0265200	Exonic	CDS	Missense	LOC_Os11g16390	expressed protein
Chr.11	10141679	10141679	G	G	A	SNP	OS11G0283500	Exonic	CDS	Missense	LOC_Os11g18044	peptide transporter PTR2, putative, expressed
Chr.11	10141158	10141158	G	G	A	SNP	OS11G0283500	Intronic	NA	NA	LOC_Os11g18044	peptide transporter PTR2, putative, expressed
Chr.11	10865068	10865068	G	G	A	SNP	OS11G0295900	Intronic	NA	NA	LOC_Os11g19060	BABY BOOM 1, putative, expressed
Chr.11	11431891	11431891	G	G	A	SNP	OS11G0303600	Intronic	NA	NA	LOC_Os11g19840	O-methyltransferase, putative, expressed
Chr.11	14740125	14740125	G	G	A	SNP	OS11G0445300	Exonic	CDS	Silent	LOC_Os11g25860	protein Kinase, putative, expressed
Chr.11	14738068	14738068	G	G	A	SNP	OS11G0445300	Exonic	CDS	Missense	LOC_Os11g25860	protein Kinase, putative, expressed
Chr.11	15422788	15422788	G	G	A	SNP	OS11G0455500	Exonic	CDS	NA	LOC_Os11g26850	erythronate-4-phosphate dehydrogenase, putative, expressed
Chr.11	17049412	17049412	C	C	A	SNP	OS11G0484300	Exonic	CDS	Missense	LOC_Os11g29380	MCM2 - Putative minichromosome maintenance MCM complex subunit 2, expressed
Chr.11	17237643	17237643	C	C	A	SNP	OS11G0489250	Exonic	CDS	Missense	LOC_Os11g29720	cytochrome P450, putative, expressed
Chr.11	17284239	17284239	G	G	A	SNP	OS11G0490100	Exonic	CDS	Silent	LOC_Os11g29780	plant-specific domain TIGR01627 family protein, expressed
Chr.11	17284398	17284398	G	G	A	SNP	OS11G0490100	Exonic	CDS	Silent	LOC_Os11g29780	plant-specific domain TIGR01627 family protein, expressed
Chr.11	17284410	17284410	G	G	A	SNP	OS11G0490100	Exonic	CDS	Silent	LOC_Os11g29780	plant-specific domain TIGR01627 family protein, expressed
Chr.11	17352564	17352564	G	G	A	SNP	OS11G0490900	Exonic	CDS	Missense	LOC_Os11g29870	WRKY72, expressed
Chr.11	17355149	17355149	T	T	A	SNP	OS11G0490900	Intronic	NA	NA	LOC_Os11g29870	WRKY72, expressed
Chr.11	18936354	18936354	G	G	A	SNP	OS11G0523800	Exonic	3UTR	NA	LOC_Os11g32110	auxin response factor, putative, expressed
Chr.11	18940993	18940993	G	G	A	SNP	OS11G0523800	Intronic	NA	NA	LOC_Os11g32110	auxin response factor, putative, expressed
Chr.11	19675909	19675909	G	G	A	SNP	OS11G0539200	Intronic	NA	NA	LOC_Os11g33270	glycosyl hydrolases family 16, putative, expressed
Chr.11	21299452	21299452	G	G	A	SNP	OS11G0570000	Exonic	3UTR	NA	LOC_Os11g36200	receptor-like protein kinase 2 precursor, putative, expressed
Chr.11	21993337	21993337	G	G	A	SNP	OS11G0582100	Intronic	NA	NA	LOC_Os11g37230	zinc finger, C3HC4 type domain containing protein, expressed
Chr.11	22175972	22175972	T	T	A	SNP	OS11G0586300	Exonic	CDS	NA	LOC_Os11g37560	expressed protein
Chr.11	22177645	22177645	T	T	A	SNP	OS11G0586300	Intronic	NA	NA	LOC_Os11g37560	expressed protein
Chr.11	22177655	22177655	G	G	A	SNP	OS11G0586300	Intronic	NA	NA	LOC_Os11g37560	expressed protein
Chr.11	22177670	22177670	T	T	A	SNP	OS11G0586300	Intronic	NA	NA	LOC_Os11g37560	expressed protein
Chr.11	22177713	22177713	G	G	A	SNP	OS11G0586300	Intronic	NA	NA	LOC_Os11g37560	expressed protein
Chr.11	22281706	22281706	G	G	A	SNP	OS11G0587600	Exonic	CDS	Silent	LOC_Os11g37700	pleiotropic drug resistance protein, putative, expressed
Chr.11	22281824	22281824	G	G	A	SNP	OS11G0587600	Intronic	NA	NA	LOC_Os11g37700	pleiotropic drug resistance protein, putative, expressed
Chr.11	22502116	22502116	G	G	A	SNP	OS11G0592000	Exonic	CDS	Silent	LOC_Os11g37950	WIP3 - Wound-induced protein precursor, expressed
Chr.11	25424115	25424115	C	C	A	SNP	OS11G0641500	Exonic	CDS	Silent	LOC_Os11g42200	laccase precursor protein, putative, expressed
Chr.11	27399662	27399662	T	T	A	SNP	OS11G0678000	Exonic	5UTR	NA	LOC_Os11g45280	protein kinase family protein, putative, expressed
Chr.11	27489200	27489200	G	G	A	SNP	OS11G0679700	Exonic	CDS	NA	LOC_Os11g45400	glycerol-3-phosphate acyltransferase, putative, expressed
Chr.11	27489854	27489854	G	G	A	SNP	OS11G0679700	Exonic	CDS	NA	LOC_Os11g45400	glycerol-3-phosphate acyltransferase, putative, expressed
Chr.11	27489919	27489919	G	G	A	SNP	OS11G0679700	Exonic	CDS	NA	LOC_Os11g45400	glycerol-3-phosphate acyltransferase, putative, expressed
Chr.11	27488652	27488652	G	G	A	SNP	OS11G0679700	Intronic	NA	NA	LOC_Os11g45400	glycerol-3-phosphate acyltransferase, putative, expressed
Chr.11	27489035	27489035	C	C	A	SNP	OS11G0679700	Intronic	NA	NA	LOC_Os11g45400	glycerol-3-phosphate acyltransferase, putative, expressed
Chr.11	28933388	28933388	T	T	A	SNP	OS11G0707000	Intronic	NA	NA	LOC_Os11g47970	AAA-type ATPase family protein, putative, expressed
Chr.11	7126767	7126768	AG	AG	A	INDEL	OS11G0234200	Intronic	NA	NA	LOC_Os11g12650	PHD-finger domain containing protein, putative, expressed
Chr.11	9059362	9059366	AGAGG	AGAGG	A	INDEL	OS11G0265200	Exonic	5UTR	NA	LOC_Os11g16390	expressed protein
Chr.11	17285335	17285342	ATTTTGTT	ATTTTGTT	A	INDEL	OS11G0490100	Exonic	3UTR	NA	LOC_Os11g29780	plant-specific domain TIGR01627 family protein, expressed
Chr.11	17352262	17352264	ATC	ATC	A	INDEL	OS11G0490900	Exonic	5UTR	NA	LOC_Os11g29870	WRKY72, expressed
Chr.11	17352293	17352302	AGCTATTCTC	AGCTATTCTC	A	INDEL	OS11G0490900	Exonic	5UTR	NA	LOC_Os11g29870	WRKY72, expressed
Chr.11	17353708	17353709	AT	AT	A	INDEL	OS11G0490900	Intronic	NA	NA	LOC_Os11g29870	WRKY72, expressed
Chr.11	18940060	18940061	AT	AT	A	INDEL	OS11G0523800	Intronic	NA	NA	LOC_Os11g32110	auxin response factor, putative, expressed
Chr.11	27488242	27488250	AAACTCCCC	AAACTCCCC	A	INDEL	OS11G0679700	Intronic	NA	NA	LOC_Os11g45400	glycerol-3-phosphate acyltransferase, putative, expressed
Chr.11	27488903	27488904	AT	AT	A	INDEL	OS11G0679700	Intronic	NA	NA	LOC_Os11g45400	glycerol-3-phosphate acyltransferase, putative, expressed
Chr.10	15640016	15640016	T	T	A	SNP	OS10G0437200	Exonic	3UTR	NA	LOC_Os10g30100	3-5 exonuclease family protein, putative, expressed
Chr.10	7560245	7560245	T	T	A	SNP	OS10G0206500	Intronic	NA	NA	LOC_Os10g13914	POEI27 - Pollen Ole e I allergen and extensin family protein precursor, expressed
Chr.10	3756149	3756149	T	T	A	SNP	OS10G0159300	Exonic	5UTR	NA	LOC_Os10g07160	retrotransposon, putative, centromere-specific, expressed
Chr.10	3755378	3755378	T	T	A	SNP	OS10G0159300	Exonic	5UTR	NA	LOC_Os10g07160	retrotransposon, putative, centromere-specific, expressed
Chr.10	380844	380844	T	T	A	SNP	OS10G0104900	Intronic	NA	NA	LOC_Os10g01570	C-5 cytosine-specific DNA methylase, putative, expressed
Chr.10	22133119	22133119	G	G	A	SNP	OS10G0561400	Intronic	NA	NA	LOC_Os10g41200	MYB family transcription factor, putative, expressed
Chr.10	21562968	21562968	G	G	A	SNP	OS10G0550000	Exonic	3UTR	NA	LOC_Os10g40250	expressed protein
Chr.10	21179724	21179724	G	G	A	SNP	OS10G0542400	Exonic	5UTR	NA	LOC_Os10g39640	expansin precursor, putative, expressed
Chr.10	20844282	20844282	G	G	A	SNP	OS10G0535800	Intronic	NA	NA	LOC_Os10g39100	uncharacterized Cys-rich domain containing protein, putative, expressed
Chr.10	19607538	19607538	G	G	A	SNP	OS10G0510000	Intronic	NA	NA	LOC_Os10g36650	actin, putative, expressed
Chr.10	19607507	19607507	G	G	A	SNP	OS10G0510000	Intronic	NA	NA	LOC_Os10g36650	actin, putative, expressed

Chr.10	19435344	19435344	G	G	A	SNP	OS10G0507800	Exonic	CDS	Missense	LOC_Os10g36370	heat shock protein DnaJ, putative, expressed
Chr.10	19095496	19095496	G	G	A	SNP	OS10G0500600	Exonic	3UTR	NA	LOC_Os10g35710	expressed protein
Chr.10	19095096	19095096	G	G	A	SNP	OS10G0500600	Exonic	3UTR	NA	LOC_Os10g35710	expressed protein
Chr.10	18637672	18637672	G	G	A	SNP	OS10G0490900	Exonic	3UTR	NA	LOC_Os10g34920	secretory protein, putative, expressed
Chr.10	15640601	15640601	G	G	A	SNP	OS10G0437200	Intronic	NA	NA	LOC_Os10g30100	3-5 exonuclease family protein, putative, expressed
Chr.10	15308401	15308401	G	G	A	SNP	OS10G0430200	Intronic	NA	NA	LOC_Os10g29470	dehydrogenase, putative, expressed
Chr.10	15308389	15308389	G	G	A	SNP	OS10G0430200	Intronic	NA	NA	LOC_Os10g29470	dehydrogenase, putative, expressed
Chr.10	15308223	15308223	G	G	A	SNP	OS10G0430200	Intronic	NA	NA	LOC_Os10g29470	dehydrogenase, putative, expressed
Chr.10	15307490	15307490	G	G	A	SNP	OS10G0430200	Intronic	NA	NA	LOC_Os10g29470	dehydrogenase, putative, expressed
Chr.10	15307477	15307477	G	G	A	SNP	OS10G0430200	Intronic	NA	NA	LOC_Os10g29470	dehydrogenase, putative, expressed
Chr.10	15307469	15307469	G	G	A	SNP	OS10G0430200	Intronic	NA	NA	LOC_Os10g29470	dehydrogenase, putative, expressed
Chr.10	15305378	15305378	G	G	A	SNP	OS10G0430200	Intronic	NA	NA	LOC_Os10g29470	dehydrogenase, putative, expressed
Chr.10	12970806	12970806	G	G	A	SNP	OS10G0390500	Intronic	NA	NA	LOC_Os10g25130	aminotransferase, classes I and II, domain containing protein, expressed
Chr.10	12048828	12048828	G	G	A	SNP	OS10G0377400	Exonic	CDS	Silent	LOC_Os10g23100	ras-related protein, putative, expressed
Chr.10	7686620	7686620	G	G	A	SNP	OS10G0208500	Intronic	NA	NA	LOC_Os10g14180	expressed protein
Chr.10	6301395	6301395	G	G	A	SNP	OS10G0190900	Intronic	NA	NA	LOC_Os10g11354	MATE efflux family protein, putative, expressed
Chr.10	6298746	6298746	G	G	A	SNP	OS10G0190900	Intronic	NA	NA	LOC_Os10g11354	MATE efflux family protein, putative, expressed
Chr.10	6298582	6298582	G	G	A	SNP	OS10G0190900	Intronic	NA	NA	LOC_Os10g11354	MATE efflux family protein, putative, expressed
Chr.10	6297306	6297306	G	G	A	SNP	OS10G0190900	Intronic	NA	NA	LOC_Os10g11354	MATE efflux family protein, putative, expressed
Chr.10	3755814	3755814	G	G	A	SNP	OS10G0159300	Exonic	5UTR	NA	LOC_Os10g07160	retrotransposon, putative, centromere-specific, expressed
Chr.10	3755361	3755361	G	G	A	SNP	OS10G0159300	Exonic	5UTR	NA	LOC_Os10g07160	retrotransposon, putative, centromere-specific, expressed
Chr.10	3755184	3755184	G	G	A	SNP	OS10G0159300	Exonic	CDS	Silent	LOC_Os10g07160	retrotransposon, putative, centromere-specific, expressed
Chr.10	3754087	3754087	G	G	A	SNP	OS10G0159300	Exonic	3UTR	NA	LOC_Os10g07160	retrotransposon, putative, centromere-specific, expressed
Chr.10	3753519	3753519	G	G	A	SNP	OS10G0159300	Exonic	3UTR	NA	LOC_Os10g07160	retrotransposon, putative, centromere-specific, expressed
Chr.10	2365922	2365922	G	G	A	SNP	OS10G0138100	Exonic	CDS	Silent	LOC_Os10g04860	aldehyde oxidase, putative, expressed
Chr.10	2362667	2362667	G	G	A	SNP	OS10G0138100	Exonic	CDS	Silent	LOC_Os10g04860	aldehyde oxidase, putative, expressed
Chr.10	2361607	2361607	G	G	A	SNP	OS10G0138100	Intronic	NA	NA	LOC_Os10g04860	aldehyde oxidase, putative, expressed
Chr.10	2361594	2361594	G	G	A	SNP	OS10G0138100	Intronic	NA	NA	LOC_Os10g04860	aldehyde oxidase, putative, expressed
Chr.10	2361380	2361380	G	G	A	SNP	OS10G0138100	Intronic	NA	NA	LOC_Os10g04860	aldehyde oxidase, putative, expressed
Chr.10	2360100	2360100	G	G	A	SNP	OS10G0138100	Intronic	NA	NA	LOC_Os10g04860	aldehyde oxidase, putative, expressed
Chr.10	2359796	2359796	G	G	A	SNP	OS10G0138100	Intronic	NA	NA	LOC_Os10g04860	aldehyde oxidase, putative, expressed
Chr.10	2359676	2359676	G	G	A	SNP	OS10G0138100	Intronic	NA	NA	LOC_Os10g04860	aldehyde oxidase, putative, expressed
Chr.10	2359630	2359630	G	G	A	SNP	OS10G0138100	Intronic	NA	NA	LOC_Os10g04860	aldehyde oxidase, putative, expressed
Chr.10	381010	381010	G	G	A	SNP	OS10G0104900	Intronic	NA	NA	LOC_Os10g01570	C-5 cytosine-specific DNA methylase, putative, expressed
Chr.10	377522	377522	G	G	A	SNP	OS10G0104900	Exonic	3UTR	NA	LOC_Os10g01570	C-5 cytosine-specific DNA methylase, putative, expressed
Chr.10	20843761	20843761	C	C	A	SNP	OS10G0535800	Intronic	NA	NA	LOC_Os10g39100	uncharacterized Cys-rich domain containing protein, putative, expressed
Chr.10	19607714	19607714	C	C	A	SNP	OS10G0510000	Intronic	NA	NA	LOC_Os10g36650	actin, putative, expressed
Chr.10	19607653	19607653	C	C	A	SNP	OS10G0510000	Intronic	NA	NA	LOC_Os10g36650	actin, putative, expressed
Chr.10	19607561	19607561	C	C	A	SNP	OS10G0510000	Intronic	NA	NA	LOC_Os10g36650	actin, putative, expressed
Chr.10	19360153	19360153	C	C	A	SNP	OS10G0506200	Exonic	CDS	Silent	LOC_Os10g36210	valyl-tRNA synthetase, putative, expressed
Chr.10	15680316	15680316	C	C	A	SNP	OS10G0437600	Exonic	CDS	Missense	LOC_Os10g30156	starch synthase, putative, expressed
Chr.10	15643442	15643442	C	C	A	SNP	OS10G0437200	Intronic	NA	NA	LOC_Os10g30100	3-5 exonuclease family protein, putative, expressed
Chr.10	15641855	15641855	C	C	A	SNP	OS10G0437200	Intronic	NA	NA	LOC_Os10g30100	3-5 exonuclease family protein, putative, expressed
Chr.10	6298749	6298749	C	C	A	SNP	OS10G0190900	Intronic	NA	NA	LOC_Os10g11354	MATE efflux family protein, putative, expressed
Chr.10	6298529	6298529	C	C	A	SNP	OS10G0190900	Intronic	NA	NA	LOC_Os10g11354	MATE efflux family protein, putative, expressed
Chr.10	6274748	6274748	C	C	A	SNP	OS10G0190500	Exonic	CDS	Missense	LOC_Os10g11310	expressed protein
Chr.10	6274516	6274516	C	C	A	SNP	OS10G0190500	Exonic	CDS	Silent	LOC_Os10g11310	expressed protein
Chr.10	3755885	3755885	C	C	A	SNP	OS10G0159300	Exonic	5UTR	NA	LOC_Os10g07160	retrotransposon, putative, centromere-specific, expressed
Chr.10	3755799	3755799	C	C	A	SNP	OS10G0159300	Exonic	5UTR	NA	LOC_Os10g07160	retrotransposon, putative, centromere-specific, expressed
Chr.10	1084867	1084867	C	C	A	SNP	OS10G0116800	Exonic	3UTR	NA	LOC_Os10g02750	Ser/Thr protein phosphatase family protein, putative, expressed
Chr.10	6299741	6299741	C	C	A	SNP	OS10G0190900	Intronic	NA	NA	LOC_Os10g11354	MATE efflux family protein, putative, expressed
Chr.10	20894657	20894657	G	G	A	SNP	OS10G0536500	Intronic	NA	NA	LOC_Os10g39150	expressed protein
Chr.10	18641874	18641874	T	T	A	SNP	OS10G0491000	Exonic	3UTR	NA	LOC_Os10g34930	secretory protein, putative, expressed
Chr.10	18637592	18637592	G	G	A	SNP	OS10G0490900	Exonic	3UTR	NA	LOC_Os10g34920	secretory protein, putative, expressed
Chr.10	18637529	18637529	G	G	A	SNP	OS10G0490900	Exonic	3UTR	NA	LOC_Os10g34920	secretory protein, putative, expressed
Chr.10	18537990	18537990	T	T	A	SNP	OS10G0488800	Intronic	NA	NA	LOC_Os10g34710	myosin-2 heavy chain, non muscle, putative, expressed
Chr.10	18535274	18535274	G	G	A	SNP	OS10G0488800	Intronic	NA	NA	LOC_Os10g34710	myosin-2 heavy chain, non muscle, putative, expressed
Chr.10	18534998	18534998	G	G	A	SNP	OS10G0488800	Intronic	NA	NA	LOC_Os10g34710	myosin-2 heavy chain, non muscle, putative, expressed
Chr.10	18533550	18533550	C	C	A	SNP	OS10G0488800	Intronic	NA	NA	LOC_Os10g34710	myosin-2 heavy chain, non muscle, putative, expressed
Chr.10	10303139	10303139	G	G	A	SNP	OS10G0345100	Intronic	NA	NA	LOC_Os10g20470	MATE efflux family protein, putative, expressed
Chr.10	68118	68118	T	T	A	SNP	OS10G0100500	Intronic	NA	NA	LOC_Os10g01060	protein kinase family protein, putative, expressed
Chr.10	68203	68203	G	G	A	SNP	OS10G0100500	Intronic	NA	NA	LOC_Os10g01060	protein kinase family protein, putative, expressed
Chr.10	378878	378878	T	T	A	SNP	OS10G0104900	Intronic	NA	NA	LOC_Os10g01570	C-5 cytosine-specific DNA methylase, putative, expressed
Chr.10	379831	379831	G	G	A	SNP	OS10G0104900	Intronic	NA	NA	LOC_Os10g01570	C-5 cytosine-specific DNA methylase, putative, expressed
Chr.10	2365600	2365600	G	G	A	SNP	OS10G0138100	Exonic	CDS	Missense	LOC_Os10g04860	aldehyde oxidase, putative, expressed
Chr.10	2367650	2367650	G	G	A	SNP	OS10G0138100	Exonic	CDS	Missense	LOC_Os10g04860	aldehyde oxidase, putative, expressed
Chr.10	2359083	2359083	C	C	A	SNP	OS10G0138100	Intronic	NA	NA	LOC_Os10g04860	aldehyde oxidase, putative, expressed
Chr.10	2360583	2360583	G	G	A	SNP	OS10G0138100	Intronic	NA	NA	LOC_Os10g04860	aldehyde oxidase, putative, expressed
Chr.10	2507862	2507862	G	G	A	SNP	OS10G0140700	Exonic	CDS	Silent	LOC_Os10g05130	expressed protein
Chr.10	2913240	2913240	C	C	A	SNP	OS10G0148400	Exonic	CDS	Silent	LOC_Os10g05780	POT domain containing peptide transporter, putative, expressed
Chr.10	2913061	2913061	G	G	A	SNP	OS10G0148400	Exonic	CDS	Missense	LOC_Os10g05780	POT domain containing peptide transporter, putative, expressed
Chr.10	2913790	2913790	G	G	A	SNP	OS10G0148400	Exonic	CDS	Missense	LOC_Os10g05780	POT domain containing peptide transporter, putative, expressed
Chr.10	3753605	3753605	G	G	A	SNP	OS10G0159300	Exonic	3UTR	NA	LOC_Os10g07160	retrotransposon, putative, centromere-specific, expressed
Chr.10	3753624	3753624	G	G	A	SNP	OS10G0159300	Exonic	3UTR	NA	LOC_Os10g07160	retrotransposon, putative, centromere-specific, expressed
Chr.10	3753807	3753807	T	T	A	SNP	OS10G0159300	Exonic	3UTR	NA	LOC_Os10g07160	retrotransposon, putative, centromere-specific, expressed
Chr.10	3756842	3756842	G	G	A	SNP	OS10G0159300	Intronic	NA	NA	LOC_Os10g07160	retrotransposon, putative, centromere-specific, expressed
Chr.10	3756875	3756875	T	T	A	SNP	OS10G0159300	Intronic	NA	NA	LOC_Os10g07160	retrotransposon, putative, centromere-specific, expressed
Chr.10	3756933	3756933	G	G	A	SNP	OS10G0159300	Intronic	NA	NA	LOC_Os10g07160	retrotransposon, putative, centromere-specific, expressed
Chr.10	7689380	7689380	G	G	A	SNP	OS10G0208500	Exonic	5UTR	NA	LOC_Os10g14180	expressed protein
Chr.10	7691989	7691989	G	G	A	SNP	OS10G0208500	Exonic	5UTR	NA	LOC_Os10g14180	expressed protein
Chr.10	10293882	10293882	G	G	A	SNP	OS10G0344900	Exonic	CDS	Silent	LOC_Os10g20450	MATE efflux family protein, putative, expressed
Chr.10	12869842	12869842	G	G	A	SNP	OS10G0388900	Exonic	CDS	Missense	LOC_Os10g25000	expressed protein
Chr.10	12871878	12871878	G	G	A	SNP	OS10G0389000	Intronic	NA	NA	LOC_Os10g25010	OsCML8 - Calmodulin-related calcium sensor protein, expressed
Chr.10	15307488	15307488	G	G	A	SNP	OS10G0430200	Intronic	NA	NA	LOC_Os10g29470	dehydrogenase, putative, expressed
Chr.10	15308007	15308007	T	T	A	SNP	OS10G0430200	Intronic	NA	NA	LOC_Os10g29470	dehydrogenase, putative, expressed

Chr.10	15308702	15308702	T	T	A	SNP	OS10G0430200	Intronic	NA	NA	LOC_Os10g29470	dehydrogenase, putative, expressed
Chr.10	15308743	15308743	C	C	A	SNP	OS10G0430200	Intronic	NA	NA	LOC_Os10g29470	dehydrogenase, putative, expressed
Chr.10	15404348	15404348	G	G	A	SNP	OS10G0431900	Intronic	NA	NA	LOC_Os10g29620	tyrosine protein kinase domain containing protein, putative, expressed
Chr.10	15404355	15404355	C	C	A	SNP	OS10G0431900	Intronic	NA	NA	LOC_Os10g29620	tyrosine protein kinase domain containing protein, putative, expressed
Chr.10	15404379	15404379	G	G	A	SNP	OS10G0431900	Intronic	NA	NA	LOC_Os10g29620	tyrosine protein kinase domain containing protein, putative, expressed
Chr.10	15642876	15642876	G	G	A	SNP	OS10G0437200	Exonic	CDS	Missense	LOC_Os10g30100	3-5 exonuclease family protein, putative, expressed
Chr.10	19607219	19607219	G	G	A	SNP	OS10G0510000	Intronic	NA	NA	LOC_Os10g36650	actin, putative, expressed
Chr.10	6299786	6299787	AG	AG	A	INDEL	OS10G0190900	Intronic	NA	NA	LOC_Os10g11354	MATE efflux family protein, putative, expressed
Chr.10	12023674	12023675	AT	AT	A	INDEL	OS10G0377000	Intronic	NA	NA	LOC_Os10g23060	expressed protein
Chr.10	12968625	12968626	AC	AC	A	INDEL	OS10G0390500	Intronic	NA	NA	LOC_Os10g25130	aminotransferase, classes I and II, domain containing protein, expressed
Chr.10	15308005	15308006	AG	AG	A	INDEL	OS10G0430200	Intronic	NA	NA	LOC_Os10g29470	dehydrogenase, putative, expressed
Chr.10	15308105	15308106	AG	AG	A	INDEL	OS10G0430200	Intronic	NA	NA	LOC_Os10g29470	dehydrogenase, putative, expressed
Chr.10	15643336	15643345	ATTATCCTAG	ATTATCCTAG	A	INDEL	OS10G0437200	Intronic	NA	NA	LOC_Os10g30100	3-5 exonuclease family protein, putative, expressed
Chr.10	19607712	19607713	AC	AC	A	INDEL	OS10G0510000	Intronic	NA	NA	LOC_Os10g36650	actin, putative, expressed
Chr.10	23079694	23079695	AC	AC	A	INDEL	OS10G0578900	Exonic	3UTR	NA	LOC_Os10g42790	cytidylyltransferase domain containing protein, expressed
Chr.1	267818	267818	T	T	A	SNP	OS01G0104800	Intronic	NA	NA	LOC_Os01g01510	sas10/Utp3 family protein, expressed
Chr.1	7029583	7029583	T	T	A	SNP	OS01G0227100	Intronic	NA	NA	LOC_Os01g12710	oxidoreductase, short chain dehydrogenase/reductase family domain containing protein, expressed
Chr.1	7365148	7365148	T	T	A	SNP	OS01G0233000	Intronic	NA	NA	LOC_Os01g13210	salt stress root protein RS1, putative, expressed
Chr.1	15171118	15171118	T	T	A	SNP	OS01G0369700	Intronic	NA	NA	LOC_Os01g27210	glutathione S-transferase, putative, expressed
Chr.1	15293055	15293055	T	T	A	SNP	OS01G0371500	Intronic	NA	NA	LOC_Os01g27390	glutathione S-transferase, putative, expressed
Chr.1	15572842	15572842	T	T	A	SNP	OS01G0376700	Intronic	NA	NA	LOC_Os01g27880	sucrose-phosphatase, putative, expressed
Chr.1	20739202	20739202	T	T	A	SNP	OS01G0552300	Intronic	NA	NA	LOC_Os01g37130	protein phosphatase 2C, putative, expressed
Chr.1	26224677	26224677	T	T	A	SNP	OS01G0649900	Intronic	NA	NA	LOC_Os01g46120	GDSL-like lipase/acylhydrolase, putative, expressed
Chr.1	27083356	27083356	T	T	A	SNP	OS01G0663300	Intronic	NA	NA	LOC_Os01g47400	OsMan01 - Endo-Beta-Mannanase, expressed
Chr.1	27177083	27177083	T	T	A	SNP	OS01G0665200	Exonic	3UTR	NA	LOC_Os01g47530	CGMC_MAPKCMGC_2.6 - CGMC includes CDA, MAPK, GSK3, and CLKC kinases, expressed
Chr.1	31838762	31838762	T	T	A	SNP	OS01G0757900	Intronic	NA	NA	LOC_Os01g55310	haloacid dehalogenase-like hydrolase domain-containing protein 1A, putative, expressed
Chr.1	32131821	32131821	T	T	A	SNP	OS01G0763600	Intronic	NA	NA	LOC_Os01g55780	glycerophosphoryl diester phosphodiesterase family protein, putative, expressed
Chr.1	33074682	33074682	T	T	A	SNP	OS01G0780800	Intronic	NA	NA	LOC_Os01g57240	expressed protein
Chr.1	33075675	33075675	T	T	A	SNP	OS01G0780800	Intronic	NA	NA	LOC_Os01g57240	expressed protein
Chr.1	41097399	41097399	T	T	A	SNP	OS01G0936100	Exonic	CDS	Silent	LOC_Os01g71000	protein kinase APK1B, chloroplast precursor, putative, expressed
Chr.1	43073687	43073687	T	T	A	SNP	OS01G0974800	Exonic	CDS	Silent	LOC_Os01g74350	soluble inorganic pyrophosphatase, putative, expressed
Chr.1	264831	264831	G	G	A	SNP	OS01G0104800	Intronic	NA	NA	LOC_Os01g01510	sas10/Utp3 family protein, expressed
Chr.1	604175	604175	G	G	A	SNP	OS01G0111500	Intronic	NA	NA	LOC_Os01g02110	helix-loop-helix DNA-binding domain containing protein, expressed
Chr.1	604431	604431	G	G	A	SNP	OS01G0111500	Intronic	NA	NA	LOC_Os01g02110	helix-loop-helix DNA-binding domain containing protein, expressed
Chr.1	604439	604439	G	G	A	SNP	OS01G0111500	Intronic	NA	NA	LOC_Os01g02110	helix-loop-helix DNA-binding domain containing protein, expressed
Chr.1	4215468	4215468	G	G	A	SNP	OS01G0180400	Exonic	3UTR	NA	LOC_Os01g08520	DUF581 domain containing protein, expressed
Chr.1	7026339	7026339	G	G	A	SNP	OS01G0227100	Exonic	CDS	Silent	LOC_Os01g12710	oxidoreductase, short chain dehydrogenase/reductase family domain containing protein, expressed
Chr.1	7028802	7028802	G	G	A	SNP	OS01G0227100	Intronic	NA	NA	LOC_Os01g12710	oxidoreductase, short chain dehydrogenase/reductase family domain containing protein, expressed
Chr.1	7029663	7029663	G	G	A	SNP	OS01G0227100	Intronic	NA	NA	LOC_Os01g12710	oxidoreductase, short chain dehydrogenase/reductase family domain containing protein, expressed
Chr.1	7279385	7279385	G	G	A	SNP	OS01G0231700	Intronic	NA	NA	LOC_Os01g13080	60S acidic ribosomal protein, putative, expressed
Chr.1	7366036	7366036	G	G	A	SNP	OS01G0233000	Exonic	5UTR	NA	LOC_Os01g13210	salt stress root protein RS1, putative, expressed
Chr.1	7459800	7459800	G	G	A	SNP	OS01G0235100	Exonic	3UTR	NA	LOC_Os01g13390	expressed protein
Chr.1	7619652	7619652	G	G	A	SNP	OS01G0237500	Intronic	NA	NA	LOC_Os01g13610	isoflavone reductase homolog IRL, putative, expressed
Chr.1	7865500	7865500	G	G	A	SNP	OS01G0242600	Intronic	NA	NA	LOC_Os01g14050	C2 domain containing protein, putative, expressed
Chr.1	9660124	9660124	G	G	A	SNP	OS01G0276000	Intronic	NA	NA	LOC_Os01g16890	ribosomal protein L7Ae, putative, expressed
Chr.1	9671934	9671934	G	G	A	SNP	OS01G0276200	Exonic	CDS	NA	LOC_Os01g16910	outer mitochondrial membrane porin, putative, expressed
Chr.1	9671433	9671433	G	G	A	SNP	OS01G0276200	Intronic	NA	NA	LOC_Os01g16910	outer mitochondrial membrane porin, putative, expressed
Chr.1	9671867	9671867	G	G	A	SNP	OS01G0276200	Intronic	NA	NA	LOC_Os01g16910	outer mitochondrial membrane porin, putative, expressed
Chr.1	15293219	15293219	G	G	A	SNP	OS01G0371500	Intronic	NA	NA	LOC_Os01g27390	glutathione S-transferase, putative, expressed
Chr.1	20739200	20739200	G	G	A	SNP	OS01G0552300	Intronic	NA	NA	LOC_Os01g37130	protein phosphatase 2C, putative, expressed
Chr.1	20739810	20739810	G	G	A	SNP	OS01G0552300	Intronic	NA	NA	LOC_Os01g37130	protein phosphatase 2C, putative, expressed
Chr.1	20739859	20739859	G	G	A	SNP	OS01G0552300	Intronic	NA	NA	LOC_Os01g37130	protein phosphatase 2C, putative, expressed
Chr.1	20740109	20740109	G	G	A	SNP	OS01G0552300	Intronic	NA	NA	LOC_Os01g37130	protein phosphatase 2C, putative, expressed
Chr.1	20740891	20740891	G	G	A	SNP	OS01G0552300	Intronic	NA	NA	LOC_Os01g37130	protein phosphatase 2C, putative, expressed
Chr.1	21633062	21633062	G	G	A	SNP	OS01G0565900	Exonic	3UTR	NA	LOC_Os01g38510	protein transport protein Sec61 subunit beta, putative, expressed
Chr.1	21632644	21632644	G	G	A	SNP	OS01G0565900	Exonic	CDS	Missense	LOC_Os01g38510	protein transport protein Sec61 subunit beta, putative, expressed
Chr.1	23344654	23344654	G	G	A	SNP	OS01G0595600	Exonic	CDS	Silent	LOC_Os01g41240	hydrolase, alpha/beta fold family domain containing protein, expressed
Chr.1	23343777	23343777	G	G	A	SNP	OS01G0595600	Exonic	CDS	Missense	LOC_Os01g41240	hydrolase, alpha/beta fold family domain containing protein, expressed
Chr.1	23673850	23673850	G	G	A	SNP	OS01G0602400	Exonic	CDS	Silent	LOC_Os01g41810	cytochrome P450 72A1, putative, expressed
Chr.1	24113928	24113928	G	G	A	SNP	OS01G0609900	Intronic	NA	NA	LOC_Os01g42410	pleiotropic drug resistance protein, putative, expressed
Chr.1	25235729	25235729	G	G	A	SNP	OS01G0631200	Intronic	NA	NA	LOC_Os01g44050	siroheme synthase, putative, expressed
Chr.1	25237051	25237051	G	G	A	SNP	OS01G0631200	Intronic	NA	NA	LOC_Os01g44050	siroheme synthase, putative, expressed
Chr.1	26803378	26803378	G	G	A	SNP	OS01G0658900	Intronic	NA	NA	LOC_Os01g46970	transcription factor, putative, expressed
Chr.1	27008928	27008928	G	G	A	SNP	OS01G0661500	Exonic	5UTR	NA	LOC_Os01g47262	AMSH-like protease, putative, expressed
Chr.1	27084290	27084290	G	G	A	SNP	OS01G0663300	Intronic	NA	NA	LOC_Os01g47400	OsMan01 - Endo-Beta-Mannanase, expressed
Chr.1	27177273	27177273	G	G	A	SNP	OS01G0665200	Exonic	CDS	Silent	LOC_Os01g47530	CGMC_MAPKCMGC_2.6 - CGMC includes CDA, MAPK, GSK3, and CLKC kinases, expressed
Chr.1	27180110	27180110	G	G	A	SNP	OS01G0665300	Exonic	CDS	Silent	LOC_Os01g47540	NLI interacting factor-like phosphatase, putative, expressed
Chr.1	28619643	28619643	G	G	A	SNP	OS01G0693400	Exonic	3UTR	NA	LOC_Os01g49830	B3 DNA binding domain containing protein, expressed
Chr.1	29897298	29897298	G	G	A	SNP	OS01G0717700	Exonic	CDS	Silent	LOC_Os01g52010	alliin lyase precursor, putative, expressed
Chr.1	29896240	29896240	G	G	A	SNP	OS01G0717700	Intronic	NA	NA	LOC_Os01g52010	alliin lyase precursor, putative, expressed
Chr.1	30025439	30025439	G	G	A	SNP	OS01G0720400	Exonic	3UTR	NA	LOC_Os01g52230	phosphoethanolamine/phosphocholine phosphatase, putative, expressed
Chr.1	30157961	30157961	G	G	A	SNP	OS01G0723200	Exonic	3UTR	NA	LOC_Os01g52490	40S ribosomal protein S24, putative, expressed
Chr.1	30158509	30158509	G	G	A	SNP	OS01G0723200	Intronic	NA	NA	LOC_Os01g52490	40S ribosomal protein S24, putative, expressed
Chr.1	30158601	30158601	G	G	A	SNP	OS01G0723200	Intronic	NA	NA	LOC_Os01g52490	40S ribosomal protein S24, putative, expressed
Chr.1	30158637	30158637	G	G	A	SNP	OS01G0723200	Intronic	NA	NA	LOC_Os01g52490	40S ribosomal protein S24, putative, expressed
Chr.1	30183413	30183413	G	G	A	SNP	OS01G0723600	Intronic	NA	NA	LOC_Os01g52530	phosphoribosyl transferase, putative, expressed
Chr.1	30261079	30261079	G	G	A	SNP	OS01G0725800	Exonic	CDS	Silent	LOC_Os01g52640	suppressor of phythchrome A, putative, expressed
Chr.1	30261650	30261650	G	G	A	SNP	OS01G0725800	Intronic	NA	NA	LOC_Os01g52640	suppressor of phythchrome A, putative, expressed
Chr.1	32001723	32001723	G	G	A	SNP	OS01G0760600	Intronic	NA	NA	LOC_Os01g55540	aminotransferase, classes I and II, domain containing protein, expressed
Chr.1	32131896	32131896	G	G	A	SNP	OS01G0763600	Intronic	NA	NA	LOC_Os01g55780	glycerophosphoryl diester phosphodiesterase family protein, putative, expressed
Chr.1	32135176	32135176	G	G	A	SNP	OS01G0763600	Intronic	NA	NA	LOC_Os01g55780	glycerophosphoryl diester phosphodiesterase family protein, putative, expressed
Chr.1	33074934	33074934	G	G	A	SNP	OS01G0780800	Intronic	NA	NA	LOC_Os01g57240	expressed protein
Chr.1	33075080	33075080	G	G	A	SNP	OS01G0780800	Intronic	NA	NA	LOC_Os01g57240	expressed protein
Chr.1	34550589	34550589	G	G	A	SNP	OS01G0812800	Exonic	3UTR	NA	LOC_Os01g59730	ribosomal protein L7Ae, putative, expressed
Chr.1	34550862	34550862	G	G	A	SNP	OS01G0812800	Exonic	CDS	Silent	LOC_Os01g59730	ribosomal protein L7Ae, putative, expressed

Chr.1	34550681	34550681	G	G	A	SNP	OS01G0812800	Exonic	CDS	Missense	LOC_Os01g59730	ribosomal protein L7Ae, putative, expressed
Chr.1	34550927	34550927	G	G	A	SNP	OS01G0812800	Intronic	NA	NA	LOC_Os01g59730	ribosomal protein L7Ae, putative, expressed
Chr.1	34551170	34551170	G	G	A	SNP	OS01G0812800	Intronic	NA	NA	LOC_Os01g59730	ribosomal protein L7Ae, putative, expressed
Chr.1	35231804	35231804	G	G	A	SNP	OS01G0824600	Intronic	NA	NA	LOC_Os01g60910	CAMK_KIN1/SNF1/Nim1_like.12 - CAMK includes calcium/calmodulin depedent protein kinases, expressed
Chr.1	36083567	36083567	G	G	A	SNP	OS01G0840700	Intronic	NA	NA	LOC_Os01g62350	60S ribosomal protein L36-2, putative, expressed
Chr.1	37046841	37046841	G	G	A	SNP	OS01G0857400	Intronic	NA	NA	LOC_Os01g63854	transmembrane amino acid transporter protein, putative, expressed
Chr.1	37443343	37443343	G	G	A	SNP	OS01G0865100	Intronic	NA	NA	LOC_Os01g64520	uricase, putative, expressed
Chr.1	38483658	38483658	G	G	A	SNP	OS01G0885700	Exonic	CDS	Silent	LOC_Os01g66250	S-locus-like receptor protein kinase, putative, expressed
Chr.1	39222436	39222436	G	G	A	SNP	OS01G0901000	Exonic	3UTR	NA	LOC_Os01g67500	armadillo/beta-catenin repeat family protein, putative, expressed
Chr.1	39549527	39549527	G	G	A	SNP	OS01G0908400	Intronic	NA	NA	LOC_Os01g68030	expressed protein
Chr.1	39549584	39549584	G	G	A	SNP	OS01G0908400	Intronic	NA	NA	LOC_Os01g68030	expressed protein
Chr.1	39566409	39566409	G	G	A	SNP	OS01G0908700	Intronic	NA	NA	LOC_Os01g68060	copine, putative, expressed
Chr.1	39831112	39831112	G	G	A	SNP	OS01G0914000	Intronic	NA	NA	LOC_Os01g68570	expressed protein
Chr.1	41004984	41004984	G	G	A	SNP	OS01G0934800	Exonic	CDS	NA	LOC_Os01g70850	esterase, putative, expressed
Chr.1	41502121	41502121	C	C	A	SNP	OS01G0944100	Intronic	NA	NA	LOC_Os01g71624	expressed protein
Chr.1	605634	605634	C	C	A	SNP	OS01G0111500	Exonic	5UTR	NA	LOC_Os01g02110	helix-loop-helix DNA-binding domain containing protein, expressed
Chr.1	6969594	6969594	C	C	A	SNP	OS01G0226300	Intronic	NA	NA	LOC_Os01g12650	reticulon domain containing protein, putative, expressed
Chr.1	7030524	7030524	C	C	A	SNP	OS01G0227100	Exonic	3UTR	NA	LOC_Os01g12710	oxidoreductase, short chain dehydrogenase/reductase family domain containing protein, expressed
Chr.1	9116212	9116212	C	C	A	SNP	OS01G0266500	Intronic	NA	NA	LOC_Os01g16146	phenazine biosynthesis protein, putative, expressed
Chr.1	9671868	9671868	C	C	A	SNP	OS01G0276200	Intronic	NA	NA	LOC_Os01g16910	outer mitochondrial membrane porin, putative, expressed
Chr.1	9673797	9673797	C	C	A	SNP	OS01G0276200	Intronic	NA	NA	LOC_Os01g16910	outer mitochondrial membrane porin, putative, expressed
Chr.1	15292843	15292843	C	C	A	SNP	OS01G0371500	Intronic	NA	NA	LOC_Os01g27390	glutathione S-transferase, putative, expressed
Chr.1	20740257	20740257	C	C	A	SNP	OS01G0552300	Intronic	NA	NA	LOC_Os01g37130	protein phosphatase 2C, putative, expressed
Chr.1	20741700	20741700	C	C	A	SNP	OS01G0552300	Intronic	NA	NA	LOC_Os01g37130	protein phosphatase 2C, putative, expressed
Chr.1	26120064	26120064	C	C	A	SNP	OS01G0648000	Exonic	CDS	Missense	LOC_Os01g45990	potassium channel AKT1, putative, expressed
Chr.1	29833822	29833822	C	C	A	SNP	OS01G0716800	Intronic	NA	NA	LOC_Os01g51890	endonuclease/exonuclease/phosphatase family domain containing protein, expressed
Chr.1	30159155	30159155	C	C	A	SNP	OS01G0723200	Intronic	NA	NA	LOC_Os01g52490	40S ribosomal protein S24, putative, expressed
Chr.1	31581756	31581756	C	C	A	SNP	OS01G0752700	Intronic	NA	NA	LOC_Os01g54910	GTP-binding protein typA/bipA, putative, expressed
Chr.1	35083380	35083380	C	C	A	SNP	OS01G0821900	Exonic	CDS	NA	LOC_Os01g60670	receptor-like protein kinase precursor, putative, expressed
Chr.1	36083162	36083162	C	C	A	SNP	OS01G0840700	Exonic	3UTR	NA	LOC_Os01g62350	60S ribosomal protein L36-2, putative, expressed
Chr.1	39955529	39955529	C	C	A	SNP	OS01G0916600	Intronic	NA	NA	LOC_Os01g68790	RNA recognition motif containing protein, putative, expressed
Chr.1	43073853	43073853	C	C	A	SNP	OS01G0974800	Intronic	NA	NA	LOC_Os01g74350	soluble inorganic pyrophosphatase, putative, expressed
Chr.1	604869	604869	T	T	A	SNP	OS01G0111500	Intronic	NA	NA	LOC_Os01g02110	helix-loop-helix DNA-binding domain containing protein, expressed
Chr.1	648321	648321	G	G	A	SNP	OS01G0112500	Exonic	3UTR	NA	LOC_Os01g02200	armadillo/beta-catenin repeat family protein, putative, expressed
Chr.1	649072	649072	G	G	A	SNP	OS01G0112500	Exonic	CDS	Missense	LOC_Os01g02200	armadillo/beta-catenin repeat family protein, putative, expressed
Chr.1	649610	649610	G	G	A	SNP	OS01G0112500	Intronic	NA	NA	LOC_Os01g02200	armadillo/beta-catenin repeat family protein, putative, expressed
Chr.1	650444	650444	G	G	A	SNP	OS01G0112500	Intronic	NA	NA	LOC_Os01g02200	armadillo/beta-catenin repeat family protein, putative, expressed
Chr.1	650637	650637	G	G	A	SNP	OS01G0112500	Intronic	NA	NA	LOC_Os01g02200	armadillo/beta-catenin repeat family protein, putative, expressed
Chr.1	650738	650738	C	C	A	SNP	OS01G0112500	Intronic	NA	NA	LOC_Os01g02200	armadillo/beta-catenin repeat family protein, putative, expressed
Chr.1	650887	650887	G	G	A	SNP	OS01G0112500	Intronic	NA	NA	LOC_Os01g02200	armadillo/beta-catenin repeat family protein, putative, expressed
Chr.1	650898	650898	T	T	A	SNP	OS01G0112500	Intronic	NA	NA	LOC_Os01g02200	armadillo/beta-catenin repeat family protein, putative, expressed
Chr.1	651001	651001	T	T	A	SNP	OS01G0112500	Intronic	NA	NA	LOC_Os01g02200	armadillo/beta-catenin repeat family protein, putative, expressed
Chr.1	651284	651284	G	G	A	SNP	OS01G0112500	Intronic	NA	NA	LOC_Os01g02200	armadillo/beta-catenin repeat family protein, putative, expressed
Chr.1	744695	744695	G	G	A	SNP	OS01G0113700	Exonic	CDS	Silent	LOC_Os01g02350	receptor-like kinase ARK1AS, putative, expressed
Chr.1	744764	744764	C	C	A	SNP	OS01G0113700	Exonic	CDS	Missense	LOC_Os01g02350	receptor-like kinase ARK1AS, putative, expressed
Chr.1	744780	744780	C	C	A	SNP	OS01G0113700	Exonic	CDS	Missense	LOC_Os01g02350	receptor-like kinase ARK1AS, putative, expressed
Chr.1	4215622	4215622	G	G	A	SNP	OS01G0180400	Exonic	CDS	NA	LOC_Os01g08520	DUF581 domain containing protein, expressed
Chr.1	9228045	9228045	G	G	A	SNP	OS01G0268300	Intronic	NA	NA	LOC_Os01g16290	DNA gyrase subunit B, chloroplast/mitochondrial precursor, putative, expressed
Chr.1	9228343	9228343	C	C	A	SNP	OS01G0268300	Intronic	NA	NA	LOC_Os01g16290	DNA gyrase subunit B, chloroplast/mitochondrial precursor, putative, expressed
Chr.1	9231064	9231064	G	G	A	SNP	OS01G0268300	Intronic	NA	NA	LOC_Os01g16290	DNA gyrase subunit B, chloroplast/mitochondrial precursor, putative, expressed
Chr.1	9231109	9231109	T	T	A	SNP	OS01G0268300	Intronic	NA	NA	LOC_Os01g16290	DNA gyrase subunit B, chloroplast/mitochondrial precursor, putative, expressed
Chr.1	9232321	9232321	T	T	A	SNP	OS01G0268300	Intronic	NA	NA	LOC_Os01g16290	DNA gyrase subunit B, chloroplast/mitochondrial precursor, putative, expressed
Chr.1	9233057	9233057	G	G	A	SNP	OS01G0268300	Intronic	NA	NA	LOC_Os01g16290	DNA gyrase subunit B, chloroplast/mitochondrial precursor, putative, expressed
Chr.1	9233563	9233563	G	G	A	SNP	OS01G0268300	Intronic	NA	NA	LOC_Os01g16290	DNA gyrase subunit B, chloroplast/mitochondrial precursor, putative, expressed
Chr.1	9233582	9233582	T	T	A	SNP	OS01G0268300	Intronic	NA	NA	LOC_Os01g16290	DNA gyrase subunit B, chloroplast/mitochondrial precursor, putative, expressed
Chr.1	9235370	9235370	T	T	A	SNP	OS01G0268300	Intronic	NA	NA	LOC_Os01g16290	DNA gyrase subunit B, chloroplast/mitochondrial precursor, putative, expressed
Chr.1	9235993	9235993	G	G	A	SNP	OS01G0268300	Intronic	NA	NA	LOC_Os01g16290	DNA gyrase subunit B, chloroplast/mitochondrial precursor, putative, expressed
Chr.1	10009800	10009800	G	G	A	SNP	OS01G0281100	Intronic	NA	NA	LOC_Os01g17396	expressed protein
Chr.1	10010393	10010393	G	G	A	SNP	OS01G0281100	Intronic	NA	NA	LOC_Os01g17396	expressed protein
Chr.1	10102294	10102294	G	G	A	SNP	OS01G0282800	Intronic	NA	NA	LOC_Os01g18050	tubulin/FtsZ domain containing protein, putative, expressed
Chr.1	10102771	10102771	C	C	A	SNP	OS01G0282800	Intronic	NA	NA	LOC_Os01g18050	tubulin/FtsZ domain containing protein, putative, expressed
Chr.1	10200532	10200532	C	C	A	SNP	OS01G0284900	Exonic	3UTR	NA	LOC_Os01g18220	expressed protein
Chr.1	10202227	10202227	G	G	A	SNP	OS01G0284900	Intronic	NA	NA	LOC_Os01g18220	expressed protein
Chr.1	10342615	10342615	G	G	A	SNP	OS01G0287600	Intronic	NA	NA	LOC_Os01g18400	CHIT10 - Chitinase family protein precursor, expressed
Chr.1	10342854	10342854	G	G	A	SNP	OS01G0287600	Intronic	NA	NA	LOC_Os01g18400	CHIT10 - Chitinase family protein precursor, expressed
Chr.1	10342968	10342968	T	T	A	SNP	OS01G0287600	Intronic	NA	NA	LOC_Os01g18400	CHIT10 - Chitinase family protein precursor, expressed
Chr.1	10342969	10342969	C	C	A	SNP	OS01G0287600	Intronic	NA	NA	LOC_Os01g18400	CHIT10 - Chitinase family protein precursor, expressed
Chr.1	10343023	10343023	T	T	A	SNP	OS01G0287600	Intronic	NA	NA	LOC_Os01g18400	CHIT10 - Chitinase family protein precursor, expressed
Chr.1	10494001	10494001	G	G	A	SNP	OS01G0290000	Exonic	CDS	Silent	LOC_Os01g18630	aspartic proteinase oryzasin-1 precursor, putative, expressed
Chr.1	10655208	10655208	C	C	A	SNP	OS01G0292900	Exonic	CDS	Missense	LOC_Os01g18850	OsSPL1 - SBP-box gene family member, expressed
Chr.1	10655893	10655893	G	G	A	SNP	OS01G0292900	Intronic	NA	NA	LOC_Os01g18850	OsSPL1 - SBP-box gene family member, expressed
Chr.1	15291968	15291968	G	G	A	SNP	OS01G0371500	Intronic	NA	NA	LOC_Os01g27390	glutathione S-transferase, putative, expressed
Chr.1	15292081	15292081	T	T	A	SNP	OS01G0371500	Intronic	NA	NA	LOC_Os01g27390	glutathione S-transferase, putative, expressed
Chr.1	15292509	15292509	G	G	A	SNP	OS01G0371500	Intronic	NA	NA	LOC_Os01g27390	glutathione S-transferase, putative, expressed
Chr.1	15292848	15292848	T	T	A	SNP	OS01G0371500	Intronic	NA	NA	LOC_Os01g27390	glutathione S-transferase, putative, expressed
Chr.1	15292944	15292944	G	G	A	SNP	OS01G0371500	Intronic	NA	NA	LOC_Os01g27390	glutathione S-transferase, putative, expressed
Chr.1	20575886	20575886	G	G	A	SNP	OS01G0550000	Exonic	3UTR	NA	LOC_Os01g36920	DEAD-box ATP-dependent RNA helicase, putative, expressed
Chr.1	20574320	20574320	G	G	A	SNP	OS01G0550000	Intronic	NA	NA	LOC_Os01g36920	DEAD-box ATP-dependent RNA helicase, putative, expressed
Chr.1	20575040	20575040	G	G	A	SNP	OS01G0550000	Intronic	NA	NA	LOC_Os01g36920	DEAD-box ATP-dependent RNA helicase, putative, expressed
Chr.1	20575063	20575063	C	C	A	SNP	OS01G0550000	Intronic	NA	NA	LOC_Os01g36920	DEAD-box ATP-dependent RNA helicase, putative, expressed
Chr.1	20587600	20587600	G	G	A	SNP	OS01G0550100	Exonic	CDS	Silent	LOC_Os01g36930	ubiquitin carboxyl-terminal hydrolase 6, putative, expressed
Chr.1	20582591	20582591	T	T	A	SNP	OS01G0550100	Intronic	NA	NA	LOC_Os01g36930	ubiquitin carboxyl-terminal hydrolase 6, putative, expressed
Chr.1	20582592	20582592	C	C	A	SNP	OS01G0550100	Intronic	NA	NA	LOC_Os01g36930	ubiquitin carboxyl-terminal hydrolase 6, putative, expressed
Chr.1	20586299	20586299	G	G	A	SNP	OS01G0550100	Intronic	NA	NA	LOC_Os01g36930	ubiquitin carboxyl-terminal hydrolase 6, putative, expressed
Chr.1	20587384	20587384	G	G	A	SNP	OS01G0550100	Intronic	NA	NA	LOC_Os01g36930	ubiquitin carboxyl-terminal hydrolase 6, putative, expressed

Chr.1	20587729	20587729	G	G	A	SNP	OS01G0550100	Intronic	NA	NA	LOC_Os01g36930	ubiquitin carboxyl-terminal hydrolase 6, putative, expressed
Chr.1	20588131	20588131	C	C	A	SNP	OS01G0550100	Intronic	NA	NA	LOC_Os01g36930	ubiquitin carboxyl-terminal hydrolase 6, putative, expressed
Chr.1	20588346	20588346	G	G	A	SNP	OS01G0550100	Intronic	NA	NA	LOC_Os01g36930	ubiquitin carboxyl-terminal hydrolase 6, putative, expressed
Chr.1	20588480	20588480	G	G	A	SNP	OS01G0550100	Intronic	NA	NA	LOC_Os01g36930	ubiquitin carboxyl-terminal hydrolase 6, putative, expressed
Chr.1	20600978	20600978	G	G	A	SNP	OS01G0550300	Intronic	NA	NA	LOC_Os01g36950	N-rich protein, putative, expressed
Chr.1	20601214	20601214	G	G	A	SNP	OS01G0550300	Intronic	NA	NA	LOC_Os01g36950	N-rich protein, putative, expressed
Chr.1	20601422	20601422	T	T	A	SNP	OS01G0550300	Intronic	NA	NA	LOC_Os01g36950	N-rich protein, putative, expressed
Chr.1	21126455	21126455	T	T	A	SNP	OS01G0558300	Exonic	CDS	Missense	LOC_Os01g37770	RWD domain containing protein, expressed
Chr.1	21125790	21125790	T	T	A	SNP	OS01G0558300	Intronic	NA	NA	LOC_Os01g37770	RWD domain containing protein, expressed
Chr.1	21125897	21125897	G	G	A	SNP	OS01G0558300	Intronic	NA	NA	LOC_Os01g37770	RWD domain containing protein, expressed
Chr.1	21126055	21126055	C	C	A	SNP	OS01G0558300	Intronic	NA	NA	LOC_Os01g37770	RWD domain containing protein, expressed
Chr.1	21126090	21126090	G	G	A	SNP	OS01G0558300	Intronic	NA	NA	LOC_Os01g37770	RWD domain containing protein, expressed
Chr.1	21126826	21126826	G	G	A	SNP	OS01G0558300	Intronic	NA	NA	LOC_Os01g37770	RWD domain containing protein, expressed
Chr.1	21127512	21127512	T	T	A	SNP	OS01G0558300	Intronic	NA	NA	LOC_Os01g37770	RWD domain containing protein, expressed
Chr.1	21127868	21127868	C	C	A	SNP	OS01G0558300	Intronic	NA	NA	LOC_Os01g37770	RWD domain containing protein, expressed
Chr.1	22498976	22498976	G	G	A	SNP	OS01G0580800	Exonic	CDS	Missense	LOC_Os01g39890	transposon protein, putative, unclassified, expressed
Chr.1	22496923	22496923	T	T	A	SNP	OS01G0580800	Intronic	NA	NA	LOC_Os01g39890	transposon protein, putative, unclassified, expressed
Chr.1	22497162	22497162	C	C	A	SNP	OS01G0580800	Intronic	NA	NA	LOC_Os01g39890	transposon protein, putative, unclassified, expressed
Chr.1	22498177	22498177	G	G	A	SNP	OS01G0580800	Intronic	NA	NA	LOC_Os01g39890	transposon protein, putative, unclassified, expressed
Chr.1	22498512	22498512	C	C	A	SNP	OS01G0580800	Intronic	NA	NA	LOC_Os01g39890	transposon protein, putative, unclassified, expressed
Chr.1	22627302	22627302	G	G	A	SNP	OS01G0583200	Intronic	NA	NA	LOC_Os01g40110	ZOS1-11 - C2H2 zinc finger protein, expressed
Chr.1	29461928	29461928	C	C	A	SNP	OS01G0708900	Intronic	NA	NA	LOC_Os01g51250	mitochondrial carrier protein, putative, expressed
Chr.1	29491102	29491102	G	G	A	SNP	OS01G0709500	Exonic	5UTR	NA	LOC_Os01g51290	protein kinase family protein, putative, expressed
Chr.1	29483935	29483935	T	T	A	SNP	OS01G0709500	Exonic	CDS	Silent	LOC_Os01g51290	protein kinase family protein, putative, expressed
Chr.1	29490895	29490895	G	G	A	SNP	OS01G0709500	Exonic	CDS	Silent	LOC_Os01g51290	protein kinase family protein, putative, expressed
Chr.1	29489725	29489725	G	G	A	SNP	OS01G0709500	Intronic	NA	NA	LOC_Os01g51290	protein kinase family protein, putative, expressed
Chr.1	29490062	29490062	C	C	A	SNP	OS01G0709500	Intronic	NA	NA	LOC_Os01g51290	protein kinase family protein, putative, expressed
Chr.1	31837519	31837519	C	C	A	SNP	OS01G0757900	Exonic	3UTR	NA	LOC_Os01g55310	haloacid dehalogenase-like hydrolase domain-containing protein 1A, putative, expressed
Chr.1	41502465	41502465	G	G	A	SNP	OS01G0944100	Exonic	3UTR	NA	LOC_Os01g71624	expressed protein
Chr.1	41495772	41495772	G	G	A	SNP	OS01G0944100	Exonic	5UTR	NA	LOC_Os01g71624	expressed protein
Chr.1	41496013	41496013	G	G	A	SNP	OS01G0944100	Exonic	CDS	Missense	LOC_Os01g71624	expressed protein
Chr.1	41497248	41497248	G	G	A	SNP	OS01G0944100	Intronic	NA	NA	LOC_Os01g71624	expressed protein
Chr.1	41499163	41499163	C	C	A	SNP	OS01G0944100	Intronic	NA	NA	LOC_Os01g71624	expressed protein
Chr.1	41499182	41499182	G	G	A	SNP	OS01G0944100	Intronic	NA	NA	LOC_Os01g71624	expressed protein
Chr.1	41500191	41500191	G	G	A	SNP	OS01G0944100	Intronic	NA	NA	LOC_Os01g71624	expressed protein
Chr.1	42979115	42979115	T	T	A	SNP	OS01G0973400	Intronic	NA	NA	LOC_Os01g74190	radical SAM enzyme, putative, expressed
Chr.1	650539	650540	AT	AT	A	INDEL	OS01G0112500	Intronic	NA	NA	LOC_Os01g02200	armadillo/beta-catenin repeat family protein, putative, expressed
Chr.1	744345	744357	AAAAGAATGGCTT	AAAAGAATGGCTT	A	INDEL	OS01G0113700	Intronic	NA	NA	LOC_Os01g02350	receptor-like kinase ARK1AS, putative, expressed
Chr.1	10011296	10011297	AT	AT	A	INDEL	OS01G0281100	Intronic	NA	NA	LOC_Os01g17396	expressed protein
Chr.1	10342650	10342651	AT	AT	A	INDEL	OS01G0287600	Intronic	NA	NA	LOC_Os01g18400	CHIT10 - Chitinase family protein precursor, expressed
Chr.1	21075977	21075979	AAG	AAG	A	INDEL	OS01G0557500	Intronic	NA	NA	LOC_Os01g37690	sodium/calcium exchanger protein, putative, expressed
Chr.1	21125547	21125551	AATAC	AATAC	A	INDEL	OS01G0558300	Intronic	NA	NA	LOC_Os01g37770	RWD domain containing protein, expressed
Chr.1	22497006	22497007	AT	AT	A	INDEL	OS01G0580800	Intronic	NA	NA	LOC_Os01g39890	transposon protein, putative, unclassified, expressed
Chr.1	29844308	29844311	AGTT	AGTT	A	INDEL	OS01G0717000	Intronic	NA	NA	LOC_Os01g51920	phosphotransferase, putative, expressed
Chr.1	41503172	41503174	AAC	AAC	A	INDEL	OS01G0944100	Exonic	3UTR	NA	LOC_Os01g71624	expressed protein
Chr.1	605157	605157	G	G	A	SNP	OS01G0111500	Exonic	CDS	Silent	LOC_Os01g02110	helix-loop-helix DNA-binding domain containing protein, expressed
Chr.1	651096	651096	G	G	A	SNP	OS01G0112500	Intronic	NA	NA	LOC_Os01g02200	armadillo/beta-catenin repeat family protein, putative, expressed
Chr.1	744666	744666	C	C	A	SNP	OS01G0113700	Exonic	CDS	Missense	LOC_Os01g02350	receptor-like kinase ARK1AS, putative, expressed
Chr.1	2277402	2277402	T	T	A	SNP	OS01G0142300	Intronic	NA	NA	LOC_Os01g04920	glycosyl transferase, group 1 domain containing protein, expressed
Chr.1	2358530	2358530	C	C	A	SNP	OS01G0143300	Exonic	CDS	Missense	LOC_Os01g05010	mitochondrial glycoprotein, putative, expressed
Chr.1	3621981	3621981	C	C	A	SNP	OS01G0170300	Exonic	CDS	Missense	LOC_Os01g07560	receptor-like protein kinase 2 precursor, putative, expressed
Chr.1	5508351	5508351	T	T	A	SNP	OS01G0201100	Intronic	NA	NA	LOC_Os01g10440	xylosyltransferase, putative, expressed
Chr.1	6565911	6565911	G	G	A	SNP	OS01G0220100	Exonic	CDS	Silent	LOC_Os01g12070	endoglucanase precursor, putative, expressed
Chr.1	6566409	6566409	C	C	A	SNP	OS01G0220100	Exonic	CDS	Silent	LOC_Os01g12070	endoglucanase precursor, putative, expressed
Chr.1	6566650	6566650	G	G	A	SNP	OS01G0220100	Exonic	CDS	Silent	LOC_Os01g12070	endoglucanase precursor, putative, expressed
Chr.1	6789448	6789448	C	C	A	SNP	OS01G0223900	Exonic	CDS	Missense	LOC_Os01g12420	receptor-like protein kinase, putative, expressed
Chr.1	7550991	7550991	G	G	A	SNP	OS01G0236300	Intronic	NA	NA	LOC_Os01g13520	auxin response factor 1, putative, expressed
Chr.1	7619749	7619749	G	G	A	SNP	OS01G0237500	Intronic	NA	NA	LOC_Os01g13610	isoflavone reductase homolog IRL, putative, expressed
Chr.1	7619805	7619805	G	G	A	SNP	OS01G0237500	Intronic	NA	NA	LOC_Os01g13610	isoflavone reductase homolog IRL, putative, expressed
Chr.1	9659670	9659670	G	G	A	SNP	OS01G0276000	Intronic	NA	NA	LOC_Os01g16890	ribosomal protein L7Ae, putative, expressed
Chr.1	9659716	9659716	C	C	A	SNP	OS01G0276000	Intronic	NA	NA	LOC_Os01g16890	ribosomal protein L7Ae, putative, expressed
Chr.1	9671870	9671870	C	C	A	SNP	OS01G0276200	Intronic	NA	NA	LOC_Os01g16910	outer mitochondrial membrane porin, putative, expressed
Chr.1	13904103	13904103	C	C	A	SNP	OS01G0348900	Intronic	NA	NA	LOC_Os01g24710	jacalin-like lectin domain containing protein, expressed
Chr.1	13904125	13904125	T	T	A	SNP	OS01G0348900	Intronic	NA	NA	LOC_Os01g24710	jacalin-like lectin domain containing protein, expressed
Chr.1	14451764	14451764	G	G	A	SNP	OS01G0357100	Exonic	CDS	Missense	LOC_Os01g25484	ferredoxin--nitrite reductase, putative, expressed
Chr.1	15294164	15294164	G	G	A	SNP	OS01G0371500	Exonic	CDS	Silent	LOC_Os01g27390	glutathione S-transferase, putative, expressed
Chr.1	15294179	15294179	G	G	A	SNP	OS01G0371500	Exonic	CDS	Silent	LOC_Os01g27390	glutathione S-transferase, putative, expressed
Chr.1	15294068	15294068	C	C	A	SNP	OS01G0371500	Exonic	CDS	Missense	LOC_Os01g27390	glutathione S-transferase, putative, expressed
Chr.1	15294189	15294189	C	C	A	SNP	OS01G0371500	Exonic	CDS	Missense	LOC_Os01g27390	glutathione S-transferase, putative, expressed
Chr.1	15293662	15293662	C	C	A	SNP	OS01G0371500	Intronic	NA	NA	LOC_Os01g27390	glutathione S-transferase, putative, expressed
Chr.1	15293668	15293668	T	T	A	SNP	OS01G0371500	Intronic	NA	NA	LOC_Os01g27390	glutathione S-transferase, putative, expressed
Chr.1	20572957	20572957	G	G	A	SNP	OS01G0550000	Exonic	CDS	Missense	LOC_Os01g36920	DEAD-box ATP-dependent RNA helicase, putative, expressed
Chr.1	20589495	20589495	G	G	A	SNP	OS01G0550100	Exonic	5UTR	NA	LOC_Os01g36930	ubiquitin carboxyl-terminal hydrolase 6, putative, expressed
Chr.1	20601196	20601196	G	G	A	SNP	OS01G0550300	Intronic	NA	NA	LOC_Os01g36950	N-rich protein, putative, expressed
Chr.1	21126751	21126751	T	T	A	SNP	OS01G0558300	Intronic	NA	NA	LOC_Os01g37770	RWD domain containing protein, expressed
Chr.1	22499364	22499364	C	C	A	SNP	OS01G0580800	Exonic	CDS	Missense	LOC_Os01g39890	transposon protein, putative, unclassified, expressed
Chr.1	22495912	22495912	G	G	A	SNP	OS01G0580800	Intronic	NA	NA	LOC_Os01g39890	transposon protein, putative, unclassified, expressed
Chr.1	22495990	22495990	G	G	A	SNP	OS01G0580800	Intronic	NA	NA	LOC_Os01g39890	transposon protein, putative, unclassified, expressed
Chr.1	22497397	22497397	G	G	A	SNP	OS01G0580800	Intronic	NA	NA	LOC_Os01g39890	transposon protein, putative, unclassified, expressed
Chr.1	23343647	23343647	G	G	A	SNP	OS01G0595600	Exonic	CDS	Missense	LOC_Os01g41240	hydrolase, alpha/beta fold family domain containing protein, expressed
Chr.1	23344997	23344997	T	T	A	SNP	OS01G0595600	Exonic	CDS	Missense	LOC_Os01g41240	hydrolase, alpha/beta fold family domain containing protein, expressed
Chr.1	23673264	23673264	G	G	A	SNP	OS01G0602400	Exonic	3UTR	NA	LOC_Os01g41810	cytochrome P450 72A1, putative, expressed
Chr.1	23675756	23675756	G	G	A	SNP	OS01G0602400	Exonic	CDS	Silent	LOC_Os01g41810	cytochrome P450 72A1, putative, expressed
Chr.1	23675518	23675518	G	G	A	SNP	OS01G0602400	Exonic	CDS	Missense	LOC_Os01g41810	cytochrome P450 72A1, putative, expressed
Chr.1	23675532	23675532	G	G	A	SNP	OS01G0602400	Exonic	CDS	Missense	LOC_Os01g41810	cytochrome P450 72A1, putative, expressed

Chr.1	24109574	24109574	G	G	A	SNP	OS01G0609900	Exonic	CDS	NA	LOC_Os01g42410	pleiotropic drug resistance protein, putative, expressed
Chr.1	24114446	24114446	G	G	A	SNP	OS01G0609900	Intronic	NA	NA	LOC_Os01g42410	pleiotropic drug resistance protein, putative, expressed
Chr.1	24114465	24114465	G	G	A	SNP	OS01G0609900	Intronic	NA	NA	LOC_Os01g42410	pleiotropic drug resistance protein, putative, expressed
Chr.1	24786440	24786440	G	G	A	SNP	OS01G0621600	Exonic	CDS	Silent	LOC_Os01g43350	protein kinase family protein, putative, expressed
Chr.1	25235253	25235253	G	G	A	SNP	OS01G0631200	Intronic	NA	NA	LOC_Os01g44050	siroheme synthase, putative, expressed
Chr.1	25681906	25681906	C	C	A	SNP	OS01G0639600	Exonic	CDS	Missense	LOC_Os01g45250	DUF1645 domain containing protein, putative, expressed
Chr.1	26126100	26126100	G	G	A	SNP	OS01G0648000	Exonic	5UTR	NA	LOC_Os01g45990	potassium channel AKT1, putative, expressed
Chr.1	26126146	26126146	T	T	A	SNP	OS01G0648000	Exonic	5UTR	NA	LOC_Os01g45990	potassium channel AKT1, putative, expressed
Chr.1	26893577	26893577	G	G	A	SNP	OS01G0660300	Intronic	NA	NA	LOC_Os01g47080	pyruvate kinase, putative, expressed
Chr.1	27082415	27082415	G	G	A	SNP	OS01G0663300	Intronic	NA	NA	LOC_Os01g47400	OsMan01 - Endo-Beta-Mannanase, expressed
Chr.1	27084864	27084864	G	G	A	SNP	OS01G0663300	Intronic	NA	NA	LOC_Os01g47400	OsMan01 - Endo-Beta-Mannanase, expressed
Chr.1	28848886	28848886	G	G	A	SNP	OS01G0697100	Exonic	CDS	Silent	LOC_Os01g50200	UDP-glucoronosyl and UDP-glucosyl transferase domain containing protein, expressed
Chr.1	28848928	28848928	G	G	A	SNP	OS01G0697100	Exonic	CDS	Silent	LOC_Os01g50200	UDP-glucoronosyl and UDP-glucosyl transferase domain containing protein, expressed
Chr.1	29896341	29896341	G	G	A	SNP	OS01G0717700	Intronic	NA	NA	LOC_Os01g52010	alliin lyase precursor, putative, expressed
Chr.1	29970706	29970706	G	G	A	SNP	OS01G0719100	Exonic	5UTR	NA	LOC_Os01g52110	RING finger and CHY zinc finger domain-containing protein 1, putative, expressed
Chr.1	30159451	30159451	G	G	A	SNP	OS01G0723200	Intronic	NA	NA	LOC_Os01g52490	40S ribosomal protein S24, putative, expressed
Chr.1	30159575	30159575	T	T	A	SNP	OS01G0723200	Intronic	NA	NA	LOC_Os01g52490	40S ribosomal protein S24, putative, expressed
Chr.1	30181056	30181056	G	G	A	SNP	OS01G0723600	Exonic	CDS	Missense	LOC_Os01g52530	phosphoribosyl transferase, putative, expressed
Chr.1	30257958	30257958	G	G	A	SNP	OS01G0725800	Exonic	5UTR	NA	LOC_Os01g52640	suppressor of phythchrome A, putative, expressed
Chr.1	30258596	30258596	G	G	A	SNP	OS01G0725800	Exonic	CDS	Missense	LOC_Os01g52640	suppressor of phythchrome A, putative, expressed
Chr.1	31839754	31839754	G	G	A	SNP	OS01G0757900	Exonic	5UTR	NA	LOC_Os01g55310	haloacid dehalogenase-like hydrolase domain-containing protein 1A, putative, expressed
Chr.1	32152014	32152014	C	C	A	SNP	OS01G0764000	Exonic	5UTR	NA	LOC_Os01g55830	glutathione S-transferase, putative, expressed
Chr.1	32462555	32462555	G	G	A	SNP	OS01G0769700	Exonic	CDS	Silent	LOC_Os01g56330	TKL_IRAK_CrRLK1L-1.5 - The CrRLK1L-1 subfamily has homology to the CrRLK1L homolog, expressed
Chr.1	32462996	32462996	G	G	A	SNP	OS01G0769700	Exonic	CDS	Silent	LOC_Os01g56330	TKL_IRAK_CrRLK1L-1.5 - The CrRLK1L-1 subfamily has homology to the CrRLK1L homolog, expressed
Chr.1	32462892	32462892	G	G	A	SNP	OS01G0769700	Exonic	CDS	Missense	LOC_Os01g56330	TKL_IRAK_CrRLK1L-1.5 - The CrRLK1L-1 subfamily has homology to the CrRLK1L homolog, expressed
Chr.1	32770280	32770280	C	C	A	SNP	OS01G0775200	Exonic	3UTR	NA	LOC_Os01g56790	expressed protein
Chr.1	33078597	33078597	C	C	A	SNP	OS01G0780800	Exonic	5UTR	NA	LOC_Os01g57240	expressed protein
Chr.1	39569739	39569739	G	G	A	SNP	OS01G0908700	Exonic	CDS	Silent	LOC_Os01g68060	copine, putative, expressed
Chr.1	39569609	39569609	G	G	A	SNP	OS01G0908700	Intronic	NA	NA	LOC_Os01g68060	copine, putative, expressed
Chr.1	41188313	41188313	G	G	A	SNP	OS01G0938100	Exonic	CDS	Silent	LOC_Os01g71190	photosystem II reaction center PSB28 protein, chloroplast precursor, putative, expressed
Chr.1	41386286	41386286	G	G	A	SNP	OS01G0941800	Exonic	5UTR	NA	LOC_Os01g71420	Ser/Thr protein phosphatase family protein, putative, expressed
Chr.1	41582540	41582540	G	G	A	SNP	OS01G0946100	Exonic	CDS	Missense	LOC_Os01g71780	WD domain, G-beta repeat domain containing protein, expressed
Chr.1	42067060	42067060	G	G	A	SNP	OS01G0955000	Exonic	3UTR	NA	LOC_Os01g72520	phosphoesterase family protein, putative, expressed
Chr.1	42065820	42065820	G	G	A	SNP	OS01G0955000	Intronic	NA	NA	LOC_Os01g72520	phosphoesterase family protein, putative, expressed
Chr.1	42977477	42977477	C	C	A	SNP	OS01G0973400	Exonic	CDS	Missense	LOC_Os01g74190	radical SAM enzyme, putative, expressed
Chr.1	603895	603901	ACAATTC	ACAATTC	A	INDEL	OS01G0111500	Exonic	3UTR	NA	LOC_Os01g02110	helix-loop-helix DNA-binding domain containing protein, expressed
Chr.1	1170986	1170988	AGG	AGG	A	INDEL	OS01G0120700	Intronic	NA	NA	LOC_Os01g03060	splicing factor, putative, expressed
Chr.1	5902726	5902727	AC	AC	A	INDEL	OS01G0208700	Intronic	NA	NA	LOC_Os01g11054	phosphoenolpyruvate carboxylase, putative, expressed
Chr.1	7365097	7365098	AT	AT	A	INDEL	OS01G0233000	Intronic	NA	NA	LOC_Os01g13210	salt stress root protein RS1, putative, expressed
Chr.1	20740600	20740602	ACT	ACT	A	INDEL	OS01G0552300	Intronic	NA	NA	LOC_Os01g37130	protein phosphatase 2C, putative, expressed
Chr.1	20741491	20741492	AT	AT	A	INDEL	OS01G0552300	Intronic	NA	NA	LOC_Os01g37130	protein phosphatase 2C, putative, expressed
Chr.1	21128223	21128226	AGGC	AGGC	A	INDEL	OS01G0558300	Exonic	5UTR	NA	LOC_Os01g37770	RWD domain containing protein, expressed
Chr.1	25236232	25236233	AG	AG	A	INDEL	OS01G0631200	Intronic	NA	NA	LOC_Os01g44050	siroheme synthase, putative, expressed
Chr.1	25681974	25681977	ACGT	ACGT	A	INDEL	OS01G0639600	Exonic	CDS	InFrame	LOC_Os01g45250	DUF1645 domain containing protein, putative, expressed
Chr.1	27084224	27084225	AT	AT	A	INDEL	OS01G0663300	Intronic	NA	NA	LOC_Os01g47400	OsMan01 - Endo-Beta-Mannanase, expressed
Chr.1	30159731	30159737	AGAGGAG	AGAGGAG	A	INDEL	OS01G0723200	Exonic	5UTR	NA	LOC_Os01g52490	40S ribosomal protein S24, putative, expressed
Chr.1	30158349	30158352	AACC	AACC	A	INDEL	OS01G0723200	Intronic	NA	NA	LOC_Os01g52490	40S ribosomal protein S24, putative, expressed
Chr.1	30158838	30158839	AT	AT	A	INDEL	OS01G0723200	Intronic	NA	NA	LOC_Os01g52490	40S ribosomal protein S24, putative, expressed
Chr.1	30159142	30159143	AT	AT	A	INDEL	OS01G0723200	Intronic	NA	NA	LOC_Os01g52490	40S ribosomal protein S24, putative, expressed
Chr.1	30159405	30159406	AC	AC	A	INDEL	OS01G0723200	Intronic	NA	NA	LOC_Os01g52490	40S ribosomal protein S24, putative, expressed
Chr.1	33077285	33077286	AG	AG	A	INDEL	OS01G0780800	Intronic	NA	NA	LOC_Os01g57240	expressed protein
Chr.1	34552468	34552469	AC	AC	A	INDEL	OS01G0812800	Intronic	NA	NA	LOC_Os01g59730	ribosomal protein L7Ae, putative, expressed
Chr.1	39438150	39438153	AAAG	AAAG	A	INDEL	OS01G0906000	Exonic	3UTR	NA	LOC_Os01g67870	expressed protein
Chr.1	39829756	39829757	AT	AT	A	INDEL	OS01G0914000	Exonic	3UTR	NA	LOC_Os01g68570	expressed protein
Chr.1	39922500	39922502	AAT	AAT	A	INDEL	OS01G0916000	Exonic	3UTR	NA	LOC_Os01g68730	RNA-binding protein FUS, putative, expressed
Chr.3	27490029	27490029	T	T	A	SNP	OS03G0689100	Intronic	NA	NA	LOC_Os03g48300	histidine acid phosphatase, putative, expressed
Chr3	27287880	27287881	CA	C	AA	INDEL	OS03G0684400	Exonic	5UTR	NA	LOC_Os03g48000	CorA-like magnesium transporter protein, putative, expressed
Chr9	19585434	19585435	TA	T	AA	INDEL	OS09G0506000	Intronic	NA	NA	LOC_Os09g32840	nucleotide pyrophosphatase/phosphodiesterase, putative, expressed
Chr.2	11486964	11486964	A	A	AAAAAAG	INDEL	OS02G0299300	Intronic	NA	NA	LOC_Os02g19650	hydrolase, alpha/beta fold family domain containing protein, expressed
Chr.3	8703678	8703678	A	AAAAAAGG	AAAAAGG	INDEL	OS03G0264400	Exonic	3UTR	NA	LOC_Os03g15780	anthranilate synthase component I-1, chloroplast precursor, putative, expressed
Chr.11	28956347	28956347	A	AGC	AAAACACACAC	INDEL	OS11G0707600	Intronic	NA	NA	LOC_Os11g48020	fatty acid hydroxylase, putative, expressed
Chr.3	31360370	31360370	A	A	AAAAG	INDEL	OS03G0758500	Exonic	5UTR	NA	LOC_Os03g55120	plastocyanin-like domain containing protein, putative, expressed
Chr.11	2423699	2423699	A	A	AAAAGGCAGCATAC	INDEL	OS11G0151700	Exonic	3UTR	NA	LOC_Os11g05400	Ser/Thr protein phosphatase family protein, putative, expressed
Chr.11	27488301	27488301	A	A	AAAC	INDEL	OS11G0679700	Intronic	NA	NA	LOC_Os11g45400	glycerol-3-phosphate acyltransferase, putative, expressed
Chr.3	25103991	25103991	A	AAG	AAAG	INDEL	OS03G0648100	Exonic	5UTR	NA	LOC_Os03g44580	DNA binding protein, putative, expressed
Chr.3	15669680	15669680	A	A	AAAG	INDEL	OS03G0391100	Intronic	NA	NA	LOC_Os03g27360	RING-H2 finger protein ATL5H precursor, putative, expressed
Chr.6	413811	413811	A	AATAT	AAAT	INDEL	OS06G0106100	Intronic	NA	NA	LOC_Os06g01700	CWC15 homolog A, putative, expressed
Chr7	27728927	27728927	A	A	AAC	INDEL	OS07G0658400	Intronic	NA	NA	LOC_Os07g46460	ferredoxin-dependent glutamate synthase, chloroplast precursor, putative, expressed
Chr.8	3422530	3422530	A	A	AAC	INDEL	OS08G0158500	Intronic	NA	NA	LOC_Os08g06200	stomatin-like protein 2, putative, expressed
Chr.6	27142534	27142534	A	AAC	AACACAC	INDEL	OS06G0659300	Intronic	NA	NA	LOC_Os06g44890	villin protein, putative, expressed
Chr.2	24567173	24567173	A	A	AACAT	INDEL	OS02G0618100	Intronic	NA	NA	LOC_Os02g40500	OsGrx_C2.1 - glutaredoxin subgroup I, expressed
Chr6	21602304	21602308	AAGAG	A	AAG	INDEL	OS06G0562600	Intronic	NA	NA	LOC_Os06g36700	T-complex protein, putative, expressed
Chr.6	30993390	30993390	A	AAGAGAGAGAGAG	AAG	INDEL	OS06G0728000	Exonic	5UTR	NA	LOC_Os06g51220	HMG1/2, putative, expressed
Chr.4	23830308	23830308	A	A	AAG	INDEL	OS04G0476100	Intronic	NA	NA	LOC_Os04g40040	copper methylamine oxidase precursor, putative, expressed
Chr.3	1630511	1630511	A	A	AAG	INDEL	OS03G0128800	Intronic	NA	NA	LOC_Os03g03670	expressed protein
Chr.12	9363804	9363804	A	A	AAG	INDEL	OS12G0264500	Intronic	NA	NA	LOC_Os12g16350	enoyl-CoA hydratase/isomerase family protein, putative, expressed
Chr.1	10102916	10102916	A	A	AAG	INDEL	OS01G0282800	Exonic	5UTR	NA	LOC_Os01g18050	tubulin/FtsZ domain containing protein, putative, expressed
Chr.2	33777426	33777426	A	AAGAGAG	AAGAG	INDEL	OS02G0794600	Intronic	NA	NA	LOC_Os02g55134	cytochrome c oxidase copper chaperone, putative, expressed
Chr.2	3827917	3827917	A	AAGAGAGAGAGAG	AAGAGAGAG	INDEL	OS02G0170100	Exonic	5UTR	NA	LOC_Os02g07410	glycine cleavage system H protein, putative, expressed
Chr.2	9973438	9973438	A	A	AAGT	INDEL	OS02G0273700	Intronic	NA	NA	LOC_Os02g17350	VHS and GAT domain containing protein, expressed
Chr.3	24817454	24817454	A	A	AAT	INDEL	OS03G0643300	Intronic	NA	NA	LOC_Os03g44150	aminotransferase, putative, expressed
Chr.3	16285385	16285385	A	A	AAT	INDEL	OS03G0401100	Intronic	NA	NA	LOC_Os03g28300	protein kinase domain containing protein, expressed
Chr.2	23993110	23993110	A	A	AAT	INDEL	OS02G0610600	Intronic	NA	NA	LOC_Os02g39720	splicing factor, arginine/serine-rich 7, putative, expressed
Chr.11	2424611	2424611	A	A	AAT	INDEL	OS11G0151700	Intronic	NA	NA	LOC_Os11g05400	Ser/Thr protein phosphatase family protein, putative, expressed
Chr.1	29894705	29894705	A	A	AAT	INDEL	OS01G0717700	Intronic	NA	NA	LOC_Os01g52010	alliin lyase precursor, putative, expressed

Chr.4	5325180	5325180	A	A	AATAT	INDEL	OS04G0178300	Intronic	NA	NA	LOC_Os04g09900	ent-kaurene synthase, chloroplast precursor, putative, expressed
Chr.3	1235950	1235950	A	A	AATATATC	INDEL	OS03G0122300	Intronic	NA	NA	LOC_Os03g03034	flavonol synthase/flavanone 3-hydroxylase, putative, expressed
Chr.2	5250485	5250485	A	ATGTGGACCCAGAT	AATGTGAACCCAGAT	INDEL	OS02G0194200	Exonic	5UTR	NA	LOC_Os02g10080	zinc finger C-x8-C-x5-C-x3-H type family protein, expressed
Chr7	21146577	21146577	A	A	AATTTCTCCCC	INDEL	OS07G0537900	Exonic	5UTR	NA	LOC_Os07g35340	TKL_IRAK_DUF26-lc.14 - DUF26 kinases have homology to DUF26 containing loci, expressed
Chr.11	22281549	22281549	A	G	AC	INDEL	OS11G0587600	Intronic	NA	NA	LOC_Os11g37700	pleiotropic drug resistance protein, putative, expressed
Chr7	4265150	4265150	A	A	AC	INDEL	OS07G0180900	Intronic	NA	NA	LOC_Os07g08330	ribosomal protein L4, putative, expressed
Chr7	21379710	21379710	A	A	AC	INDEL	OS07G0541400	Intronic	NA	NA	LOC_Os07g35690	TKL_IRAK_DUF26-lc.3 - DUF26 kinases have homology to DUF26 containing loci, expressed
Chr.8	23038348	23038348	A	A	AC	INDEL	OS08G0468100	Exonic	3UTR	NA	LOC_Os08g36480	nitrate reductase, putative, expressed
Chr.6	23468569	23468569	A	A	AC	INDEL	OS06G0596300	Intronic	NA	NA	LOC_Os06g39520	myristoyl-acyl carrier protein thioesterase, chloroplast precursor, putative, expressed
Chr.6	643742	643742	A	A	AC	INDEL	OS06G0111500	Exonic	5UTR	NA	LOC_Os06g02144	6-phosphogluconate dehydrogenase, decarboxylating, putative, expressed
Chr.4	5321574	5321574	A	A	AC	INDEL	OS04G0178300	Intronic	NA	NA	LOC_Os04g09900	ent-kaurene synthase, chloroplast precursor, putative, expressed
Chr.4	33162034	33162034	A	A	AC	INDEL	OS04G0650700	Exonic	CDS	FrameShift	LOC_Os04g55710	transposon protein, putative, unclassified, expressed
Chr.12	25650523	25650523	A	A	AC	INDEL	OS12G0607100	Intronic	NA	NA	LOC_Os12g41400	eukaryotic translation initiation factor 2 subunit gamma, putative, expressed
Chr.12	25651487	25651487	A	A	AC	INDEL	OS12G0607100	Intronic	NA	NA	LOC_Os12g41400	eukaryotic translation initiation factor 2 subunit gamma, putative, expressed
Chr.1	15292112	15292112	A	A	AC	INDEL	OS01G0371500	Intronic	NA	NA	LOC_Os01g27390	glutathione S-transferase, putative, expressed
Chr.1	10011541	10011541	A	A	AC	INDEL	OS01G0281100	Exonic	5UTR	NA	LOC_Os01g17396	expressed protein
Chr1	3665198	3665218	AACACACACACACACACACAC	A	ACACACACACAC	INDEL	OS01G0170900	Exonic	3UTR	NA	LOC_Os01g07620	expressed protein
Chr.2	35096479	35096479	A	A	ACATAAACCACTG	INDEL	OS02G0817900	Intronic	NA	NA	LOC_Os02g57290	cytochrome P450, putative, expressed
Chr.5	8902648	8902648	A	A	ACATG	INDEL	OS05G0247100	Exonic	3UTR	NA	LOC_Os05g15770	glycosyl hydrolase, putative, expressed
Chr.3	8221949	8221949	A	A	ACC	INDEL	OS03G0255500	Intronic	NA	NA	LOC_Os03g15050	phosphoenolpyruvate carboxykinase, putative, expressed
Chr.6	21895479	21895479	A	ACC	ACCC	INDEL	OS06G0567200	Intronic	NA	NA	LOC_Os06g37080	L-ascorbate oxidase precursor, putative, expressed
Chr.1	744641	744641	A	A	ACCC	INDEL	OS01G0113700	Exonic	CDS	InFrame	LOC_Os01g02350	receptor-like kinase ARK1AS, putative, expressed
Chr11	8388937	8388939	ACC	A	ACCCC	INDEL	OS11G0255500	Intronic	NA	NA	LOC_Os11g14910	NADP-dependent oxidoreductase, putative, expressed
Chr.5	27534706	27534706	A	A	ACCTCCT	INDEL	OS05G0553800	Intronic	NA	NA	LOC_Os05g48030	anti-silencing protein, ASF1-like domain containing protein, expressed
Chr.4	5847261	5847261	A	A	ACG	INDEL	OS04G0186400	Exonic	5UTR	NA	LOC_Os04g10750	inorganic phosphate transporter, putative, expressed
Chr.1	27107249	27107249	A	A	ACG	INDEL	OS01G0663800	Intronic	NA	NA	LOC_Os01g47430	protein of unknown function DUF1296 domain containing protein, expressed
Chr.8	657353	657353	A	A	ACGC	INDEL	OS08G0112300	Exonic	CDS	NA	LOC_Os08g02030	transferase family protein, putative, expressed
Chr.3	21088517	21088517	A	ACGG	ACGGCGG	INDEL	OS03G0576700	Exonic	5UTR	NA	LOC_Os03g37970	ribosomal protein L13, putative, expressed
Chr8	9387094	9387097	ACGG	A	ACGGCGGCGGCGG	INDEL	OS08G0253800	Exonic	CDS	InFrame	LOC_Os08g15420	CSLC3 - cellulose synthase-like family C, expressed
Chr7	3662432	3662432	A	ACGGCGGCGGCGG	ACGGCGGCGGCGGCGGCGG	INDEL	OS07G0168300	Exonic	CDS	InFrame	LOC_Os07g07320	glutathione S-transferase, putative, expressed
Chr.5	14165379	14165395	ATTCTCTCTCTCTCTCT	A	ACTCTCTCTCTCTCT	INDEL	OS05G0310500	Exonic	3UTR	NA	LOC_Os05g24550	Papain family cysteine protease domain containing protein, expressed
Chr.2	7220760	7220760	A	A	ACTTGTGTC	INDEL	OS02G0228300	Exonic	3UTR	NA	LOC_Os02g13510	receptor-like protein kinase 5 precursor, putative, expressed
Chr1	42025949	42025954	AGGGGG	A	AG	INDEL	OS01G0954400	Intronic	NA	NA	LOC_Os01g72480	zinc finger, C3HC4 type domain containing protein, expressed
Chr7	27732273	27732273	A	A	AG	INDEL	OS07G0658400	Intronic	NA	NA	LOC_Os07g46460	ferredoxin-dependent glutamate synthase, chloroplast precursor, putative, expressed
Chr.6	23879908	23879908	A	A	AG	INDEL	OS06G0603600	Exonic	5UTR	NA	LOC_Os06g40120	SPX domain containing protein, putative, expressed
Chr.4	31239212	31239212	A	A	AG	INDEL	OS04G0615700	Exonic	5UTR	NA	LOC_Os04g52540	retrotransposon protein, putative, unclassified, expressed
Chr.11	22177623	22177623	A	A	AG	INDEL	OS11G0586300	Intronic	NA	NA	LOC_Os11g37560	expressed protein
Chr.10	15404702	15404702	A	A	AG	INDEL	OS10G0431900	Intronic	NA	NA	LOC_Os10g29620	tyrosine protein kinase domain containing protein, putative, expressed
Chr.1	15293028	15293028	A	A	AG	INDEL	OS01G0371500	Intronic	NA	NA	LOC_Os01g27390	glutathione S-transferase, putative, expressed
Chr.1	41499064	41499064	A	A	AG	INDEL	OS01G0944100	Intronic	NA	NA	LOC_Os01g71624	expressed protein
Chr10	21758110	21758113	CGAA	C	AGAA	INDEL	OS10G0554200	Exonic	3UTR	NA	LOC_Os10g40600	peptide transporter PTR2, putative, expressed
Chr7	27728819	27728819	A	A	AGAG	INDEL	OS07G0658400	Intronic	NA	NA	LOC_Os07g46460	ferredoxin-dependent glutamate synthase, chloroplast precursor, putative, expressed
Chr.3	8201854	8201854	A	A	AGAG	INDEL	OS03G0255100	Exonic	CDS	InFrame	LOC_Os03g15020	beta-galactosidase precursor, putative, expressed
Chr.1	6565696	6565696	A	A	AGCAATAT	INDEL	OS01G0220100	Intronic	NA	NA	LOC_Os01g12070	endoglucanase precursor, putative, expressed
Chr7	29171798	29171798	A	A	AGCT	INDEL	OS07G0686600	Exonic	3UTR	NA	LOC_Os07g48710	VQ domain containing protein, putative, expressed
Chr.12	25445279	25445279	A	A	AGCT	INDEL	OS12G0603700	Exonic	CDS	InFrame	LOC_Os12g41090	CAMK_KIN1/SNF1/Nim1_like.38 - CAMK includes calcium/calmodulin depedent protein kinases, expressed
Chr.1	39831119	39831119	A	A	AGCT	INDEL	OS01G0914000	Intronic	NA	NA	LOC_Os01g68570	expressed protein
Chr7	27172521	27172521	A	A	AGCTTTGGTG	INDEL	OS07G0650100	Exonic	CDS	InFrame	LOC_Os07g45550	expressed protein
Chr.1	43073851	43073851	A	A	AGG	INDEL	OS01G0974800	Intronic	NA	NA	LOC_Os01g74350	soluble inorganic pyrophosphatase, putative, expressed
Chr7	26633613	26633619	AGGAGGC	A	AGGAGGCGGAGGC	INDEL	OS07G0639900	Exonic	5UTR	NA	LOC_Os07g44630	thymidylate kinase, putative, expressed
Chr7	20725709	20725709	A	A	AGGC	INDEL	OS07G0529600	Exonic	CDS	InFrame	LOC_Os07g34570	FAD dependent oxidoreductase domain containing protein, expressed
Chr.2	8981051	8981051	A	A	AGGC	INDEL	OS02G0258900	Exonic	5UTR	NA	LOC_Os02g15870	molybdenum cofactor biosynthesis protein 1, putative, expressed
Chr.5	17583581	17583581	A	C	AGGTGTACATGTTTTC	INDEL	OS05G0366300	Intronic	NA	NA	LOC_Os05g30340	expressed protein
Chr8	752650	752652	ATT	A	AT	INDEL	OS08G0114100	Intronic	NA	NA	LOC_Os08g02210	expressed protein
Chr11	19676704	19676706	ATT	A	AT	INDEL	OS11G0539200	Intronic	NA	NA	LOC_Os11g33270	glycosyl hydrolases family 16, putative, expressed
Chr.4	23315848	23315850	ATT	A	AT	INDEL	OS04G0466600	Intronic	NA	NA	LOC_Os04g39210	agmatine deiminase, putative, expressed
Chr3	27487899	27487901	ATT	A	AT	INDEL	OS03G0689100	Intronic	NA	NA	LOC_Os03g48300	histidine acid phosphatase, putative, expressed
Chr1	29895661	29895664	ATTT	A	AT	INDEL	OS01G0717700	Intronic	NA	NA	LOC_Os01g52010	alliin lyase precursor, putative, expressed
Chr.9	7280701	7280701	A	ATT	AT	INDEL	OS09G0298500	Intronic	NA	NA	LOC_Os09g12720	zinc finger, C3HC4 type domain containing protein, expressed
Chr.4	31276226	31276226	A	ATT	AT	INDEL	OS04G0616300	Intronic	NA	NA	LOC_Os04g52600	SHR5-receptor-like kinase, putative, expressed
Chr.2	5709030	5709030	A	ATTT	AT	INDEL	OS02G0202200	Exonic	3UTR	NA	LOC_Os02g10780	SPX domain containing protein, putative, expressed
Chr.4	22021124	22021124	A	ATTTT	AT	INDEL	OS04G0442200	Intronic	NA	NA	LOC_Os04g36062	expressed protein
Chr.4	336862	336862	A	G	AT	INDEL	OS04G0105100	Exonic	3UTR	NA	LOC_Os04g01490	zinc finger, C3HC4 type, domain containing protein, expressed
Chr.4	5320028	5320028	A	T	AT	INDEL	OS04G0178300	Intronic	NA	NA	LOC_Os04g09900	ent-kaurene synthase, chloroplast precursor, putative, expressed
Chr.3	17451682	17451682	A	T	AT	INDEL	OS03G0419100	Intronic	NA	NA	LOC_Os03g30570	protein of unknown function DUF1675 domain containing protein, expressed
Chr.3	27502043	27502043	A	T	AT	INDEL	OS03G0689100	Intronic	NA	NA	LOC_Os03g48300	histidine acid phosphatase, putative, expressed
Chr7	2645599	2645599	A	A	AT	INDEL	OS07G0150700	Intronic	NA	NA	LOC_Os07g05620	CAMK_KIN1/SNF1/Nim1_like.28 - CAMK includes calcium/calmodulin depedent protein kinases, expressed
Chr7	18763612	18763612	A	A	AT	INDEL	OS07G0499300	Exonic	3UTR	NA	LOC_Os07g31599	expressed protein
Chr7	4265070	4265070	A	A	AT	INDEL	OS07G0180900	Intronic	NA	NA	LOC_Os07g08330	ribosomal protein L4, putative, expressed
Chr7	27726387	27726387	A	A	AT	INDEL	OS07G0658400	Intronic	NA	NA	LOC_Os07g46460	ferredoxin-dependent glutamate synthase, chloroplast precursor, putative, expressed
Chr7	27734448	27734448	A	A	AT	INDEL	OS07G0658400	Intronic	NA	NA	LOC_Os07g46460	ferredoxin-dependent glutamate synthase, chloroplast precursor, putative, expressed
Chr7	13103641	13103641	A	A	AT	INDEL	OS07G0415200	Exonic	3UTR	NA	LOC_Os07g23244	ribosomal protein L23 family protein, putative, expressed
Chr.9	18850615	18850615	A	A	AT	INDEL	OS09G0488700	Intronic	NA	NA	LOC_Os09g31360	expressed protein
Chr.9	16960038	16960038	A	A	AT	INDEL	OS09G0452200	Intronic	NA	NA	LOC_Os09g27890	lysM domain-containing GPI-anchored protein precursor, putative, expressed
Chr.9	14030897	14030897	A	A	AT	INDEL	OS09G0401000	Exonic	3UTR	NA	LOC_Os09g23620	MYB family transcription factor, putative, expressed
Chr.9	6388596	6388596	A	A	AT	INDEL	OS09G0286600	Exonic	3UTR	NA	LOC_Os09g11460	AP2 domain containing protein, expressed
Chr.9	9654113	9654113	A	A	AT	INDEL	OS09G0327300	Intronic	NA	NA	LOC_Os09g15810	bifunctional protein folD, putative, expressed
Chr.8	692238	692238	A	A	AT	INDEL	OS08G0112900	Intronic	NA	NA	LOC_Os08g02094	GDSL-like lipase/acylhydrolase, putative, expressed
Chr.8	15660526	15660526	A	A	AT	INDEL	OS08G0345700	Intronic	NA	NA	LOC_Os08g25720	pyrophosphate--fructose 6-phosphate 1-phosphotransferase subunit alpha, putative, expressed
Chr.8	3424516	3424516	A	A	AT	INDEL	OS08G0158500	Exonic	3UTR	NA	LOC_Os08g06200	stomatin-like protein 2, putative, expressed
Chr.8	3421781	3421781	A	A	AT	INDEL	OS08G0158500	Intronic	NA	NA	LOC_Os08g06200	stomatin-like protein 2, putative, expressed
Chr.8	6211304	6211304	A	A	AT	INDEL	OS08G0206400	Exonic	3UTR	NA	LOC_Os08g10550	potassium transporter, putative, expressed
Chr.8	12268992	12268992	A	A	AT	INDEL	OS08G0299400	Intronic	NA	NA	LOC_Os08g20420	MGD2, putative, expressed
Chr.8	13956050	13956050	A	A	AT	INDEL	OS08G0320400	Intronic	NA	NA	LOC_Os08g23150	indole-3-glycerol phosphate synthase, chloroplast precursor, putative, expressed
Chr.8	3422000	3422000	A	A	AT	INDEL	OS08G0158500	Intronic	NA	NA	LOC_Os08g06200	stomatin-like protein 2, putative, expressed

Chr.8	23988421	23988421	A	A	AT	INDEL	OS08G0485400	Intronic	NA	NA	LOC_Os08g37874	2-nitropropane dioxygenase family, putative, expressed
Chr.6	19518342	19518342	A	A	AT	INDEL	OS06G0526600	Intronic	NA	NA	LOC_Os06g33520	DEAD/DEAH box helicase, putative, expressed
Chr.6	1560399	1560399	A	A	AT	INDEL	OS06G0129400	Intronic	NA	NA	LOC_Os06g03860	uncharacterized membrane protein, putative, expressed
Chr.6	23305428	23305428	A	A	AT	INDEL	OS06G0593100	Intronic	NA	NA	LOC_Os06g39260	solute carrier family 35 member B1, putative, expressed
Chr.6	30302890	30302890	A	A	AT	INDEL	OS06G0714200	Intronic	NA	NA	LOC_Os06g50030	CAMK_CAMK_like.30 - CAMK includes calcium/calmodulin depedent protein kinases, expressed
Chr.5	22070801	22070801	A	A	AT	INDEL	OS05G0449600	Intronic	NA	NA	LOC_Os05g37700	periplasmic beta-glucosidase precursor, putative, expressed
Chr.4	27192334	27192334	A	A	AT	INDEL	OS04G0542900	Intronic	NA	NA	LOC_Os04g45910	placental protein 11 precursor, putative, expressed
Chr.4	31177552	31177552	A	A	AT	INDEL	OS04G0614600	Intronic	NA	NA	LOC_Os04g52450	aminotransferase, putative, expressed
Chr.4	20426088	20426088	A	A	AT	INDEL	OS04G0413500	Intronic	NA	NA	LOC_Os04g33740	glycosyl hydrolases, putative, expressed
Chr.4	27505877	27505877	A	A	AT	INDEL	OS04G0549600	Intronic	NA	NA	LOC_Os04g46390	chaperone protein dnaJ, putative, expressed
Chr.3	1646269	1646269	A	A	AT	INDEL	OS03G0129100	Intronic	NA	NA	LOC_Os03g03700	MLO domain containing protein, putative, expressed
Chr.3	31785736	31785736	A	A	AT	INDEL	OS03G0767600	Intronic	NA	NA	LOC_Os03g55830	BTBN10 - Bric-a-Brac, Tramtrack, Broad Complex BTB domain with non-phototropic hypocotyl 3 NPH3 and coiled-coil domains, e
Chr.3	27498546	27498546	A	A	AT	INDEL	OS03G0689100	Intronic	NA	NA	LOC_Os03g48300	histidine acid phosphatase, putative, expressed
Chr.3	8223383	8223383	A	A	AT	INDEL	OS03G0255500	Intronic	NA	NA	LOC_Os03g15050	phosphoenolpyruvate carboxykinase, putative, expressed
Chr.3	6140764	6140764	A	A	AT	INDEL	OS03G0216700	Intronic	NA	NA	LOC_Os03g11734	MATE efflux protein, putative, expressed
Chr.3	24810121	24810121	A	A	AT	INDEL	OS03G0643300	Exonic	3UTR	NA	LOC_Os03g44150	aminotransferase, putative, expressed
Chr.3	35162007	35162007	A	A	AT	INDEL	OS03G0836800	Intronic	NA	NA	LOC_Os03g62060	hydrolase, putative, expressed
Chr.2	5153256	5153256	A	A	AT	INDEL	OS02G0192500	Intronic	NA	NA	LOC_Os02g09930	CSLA1 - cellulose synthase-like family A; mannan synthase, expressed
Chr.2	9926559	9926559	A	A	AT	INDEL	OS02G0272900	Intronic	NA	NA	LOC_Os02g17304	resistance protein, putative, expressed
Chr.2	34042364	34042364	A	A	AT	INDEL	OS02G0799300	Intronic	NA	NA	LOC_Os02g55590	expressed protein
Chr.11	2146110	2146110	A	A	AT	INDEL	OS11G0147100	Intronic	NA	NA	LOC_Os11g04990	expressed protein
Chr.11	17354201	17354201	A	A	AT	INDEL	OS11G0490900	Intronic	NA	NA	LOC_Os11g29870	WRKY72, expressed
Chr.11	17354755	17354755	A	A	AT	INDEL	OS11G0490900	Intronic	NA	NA	LOC_Os11g29870	WRKY72, expressed
Chr.11	27488404	27488404	A	A	AT	INDEL	OS11G0679700	Intronic	NA	NA	LOC_Os11g45400	glycerol-3-phosphate acyltransferase, putative, expressed
Chr.10	21866403	21866403	A	A	AT	INDEL	OS10G0556200	Intronic	NA	NA	LOC_Os10g40740	helix-loop-helix DNA-binding domain containing protein, expressed
Chr.10	19607492	19607492	A	A	AT	INDEL	OS10G0510000	Intronic	NA	NA	LOC_Os10g36650	actin, putative, expressed
Chr.10	13070017	13070017	A	A	AT	INDEL	OS10G0392400	Exonic	3UTR	NA	LOC_Os10g25290	ZIM domain containing protein, putative, expressed
Chr.1	649558	649558	A	A	AT	INDEL	OS01G0112500	Intronic	NA	NA	LOC_Os01g02200	armadillo/beta-catenin repeat family protein, putative, expressed
Chr.1	650437	650437	A	A	AT	INDEL	OS01G0112500	Intronic	NA	NA	LOC_Os01g02200	armadillo/beta-catenin repeat family protein, putative, expressed
Chr.1	15293303	15293303	A	A	AT	INDEL	OS01G0371500	Intronic	NA	NA	LOC_Os01g27390	glutathione S-transferase, putative, expressed
Chr.1	41496176	41496176	A	A	AT	INDEL	OS01G0944100	Intronic	NA	NA	LOC_Os01g71624	expressed protein
Chr.1	10625231	10625231	A	A	AT	INDEL	OS01G0292200	Intronic	NA	NA	LOC_Os01g18800	CAMK_KIN1/SNF1/Nim1_like.9 - CAMK includes calcium/calmodulin depedent protein kinases, expressed
Chr.1	11319600	11319600	A	A	AT	INDEL	OS01G0305200	Intronic	NA	NA	LOC_Os01g19940	expressed protein
Chr.1	20739834	20739834	A	A	AT	INDEL	OS01G0552300	Intronic	NA	NA	LOC_Os01g37130	protein phosphatase 2C, putative, expressed
Chr.1	27083357	27083357	A	A	AT	INDEL	OS01G0663300	Intronic	NA	NA	LOC_Os01g47400	OsMan01 - Endo-Beta-Mannanase, expressed
Chr.1	34550437	34550437	A	A	AT	INDEL	OS01G0812800	Exonic	3UTR	NA	LOC_Os01g59730	ribosomal protein L7Ae, putative, expressed
Chr.1	39569153	39569153	A	A	AT	INDEL	OS01G0908700	Intronic	NA	NA	LOC_Os01g68060	copine, putative, expressed
Chr.1	7619639	7619639	A	A	AT	INDEL	OS01G0237500	Intronic	NA	NA	LOC_Os01g13610	isoflavone reductase homolog IRL, putative, expressed
Chr.1	7619736	7619736	A	A	AT	INDEL	OS01G0237500	Intronic	NA	NA	LOC_Os01g13610	isoflavone reductase homolog IRL, putative, expressed
Chr.1	9673331	9673331	A	A	AT	INDEL	OS01G0276200	Intronic	NA	NA	LOC_Os01g16910	outer mitochondrial membrane porin, putative, expressed
Chr.1	29458686	29458686	A	A	AT	INDEL	OS01G0708900	Exonic	5UTR	NA	LOC_Os01g51250	mitochondrial carrier protein, putative, expressed
Chr.1	42065995	42065995	A	A	AT	INDEL	OS01G0955000	Intronic	NA	NA	LOC_Os01g72520	phosphoesterase family protein, putative, expressed
Chr1	7666136	7666137	AT	A	ATA	INDEL	OS01G0238500	Intronic	NA	NA	LOC_Os01g13690	ligA, putative, expressed
Chr.6	7230521	7230521	A	A	ATAC	INDEL	OS06G0239200	Exonic	3UTR	NA	LOC_Os06g13190	expressed protein
Chr.2	9989675	9989675	A	A	ATAC	INDEL	OS02G0273900	Intronic	NA	NA	LOC_Os02g17370	expressed protein
Chr7	24701985	24701985	A	A	ATAG	INDEL	OS07G0603600	Intronic	NA	NA	LOC_Os07g41230	esterase, putative, expressed
Chr.2	81409	81409	A	A	ATAG	INDEL	OS02G0101500	Intronic	NA	NA	LOC_Os02g01150	erythronate-4-phosphate dehydrogenase domain containing protein, expressed
Chr.2	4296362	4296362	A	A	ATAG	INDEL	OS02G0177800	Intronic	NA	NA	LOC_Os02g08120	calmodulin binding protein, putative, expressed
Chr.11	7127892	7127892	A	A	ATAG	INDEL	OS11G0234200	Intronic	NA	NA	LOC_Os11g12650	PHD-finger domain containing protein, putative, expressed
Chr.1	34552535	34552535	A	A	ATAG	INDEL	OS01G0812800	Intronic	NA	NA	LOC_Os01g59730	ribosomal protein L7Ae, putative, expressed
Chr.4	5324650	5324650	A	A	ATAT	INDEL	OS04G0178300	Intronic	NA	NA	LOC_Os04g09900	ent-kaurene synthase, chloroplast precursor, putative, expressed
Chr.11	22280670	22280670	A	A	ATATAATT	INDEL	OS11G0587600	Intronic	NA	NA	LOC_Os11g37700	pleiotropic drug resistance protein, putative, expressed
Chr.3	1626920	1626920	A	A	ATATGCCAGCT	INDEL	OS03G0128700	Intronic	NA	NA	LOC_Os03g03660	CAMK_CAMK_like.17 - CAMK includes calcium/calmodulin depedent protein kinases, expressed
Chr.1	22498552	22498552	A	A	ATATGGTT	INDEL	OS01G0580800	Intronic	NA	NA	LOC_Os01g39890	transposon protein, putative, unclassified, expressed
Chr.5	20320510	20320510	A	A	ATATTAT	INDEL	OS05G0415700	Exonic	3UTR	NA	LOC_Os05g34320	beta-hexosaminidase precursor, putative, expressed
Chr.8	21171492	21171492	A	A	ATC	INDEL	OS08G0435900	Exonic	3UTR	NA	LOC_Os08g33820	chlorophyll A-B binding protein, putative, expressed
Chr.2	3828122	3828122	A	A	ATC	INDEL	OS02G0170100	Intronic	NA	NA	LOC_Os02g07410	glycine cleavage system H protein, putative, expressed
Chr.1	41502283	41502283	A	A	ATC	INDEL	OS01G0944100	Intronic	NA	NA	LOC_Os01g71624	expressed protein
Chr.1	21637283	21637283	A	A	ATCAG	INDEL	OS01G0566050	Exonic	3UTR	NA	LOC_Os01g38530	ELF3 protein, putative, expressed
Chr9	21189619	21189623	ATCTC	A	ATCTCTC	INDEL	OS09G0538400	Exonic	3UTR	NA	LOC_Os09g36730	MYB family transcription factor, putative, expressed
Chr.1	7547541	7547541	A	ATC	ATCTCTCTCTC	INDEL	OS01G0236300	Intronic	NA	NA	LOC_Os01g13520	auxin response factor 1, putative, expressed
Chr.6	2206395	2206395	A	ATCTTCTTCT	ATCTTCTTCTTCT	INDEL	OS06G0142300	Exonic	CDS	InFrame	LOC_Os06g05000	early nodulin 93 ENOD93 protein, putative, expressed
Chr7	27731031	27731031	A	A	ATGAAAAT	INDEL	OS07G0658400	Intronic	NA	NA	LOC_Os07g46460	ferredoxin-dependent glutamate synthase, chloroplast precursor, putative, expressed
Chr.11	15420413	15420413	A	A	ATGGCGAACT	INDEL	OS11G0455500	Exonic	3UTR	NA	LOC_Os11g26850	erythronate-4-phosphate dehydrogenase, putative, expressed
Chr3	31944884	31944890	ATGTGTG	A	ATGTG	INDEL	OS03G0770900	Intronic	NA	NA	LOC_Os03g56070	expressed protein
Chr11	27488919	27488920	AT	A	ATT	INDEL	OS11G0679700	Intronic	NA	NA	LOC_Os11g45400	glycerol-3-phosphate acyltransferase, putative, expressed
Chr.10	15403401	15403401	A	AT	ATT	INDEL	OS10G0431900	Intronic	NA	NA	LOC_Os10g29620	tyrosine protein kinase domain containing protein, putative, expressed
Chr.5	28307973	28307973	A	AT	ATT	INDEL	OS05G0568600	Exonic	3UTR	NA	LOC_Os05g49350	DUF1645 domain containing protein, putative, expressed
Chr.3	943727	943727	A	ATTT	ATT	INDEL	OS03G0116700	Intronic	NA	NA	LOC_Os03g02550	OsFBX76 - F-box domain containing protein, expressed
Chr.8	15659716	15659716	A	A	ATT	INDEL	OS08G0345700	Intronic	NA	NA	LOC_Os08g25720	pyrophosphate--fructose 6-phosphate 1-phosphotransferase subunit alpha, putative, expressed
Chr.6	3198140	3198140	A	A	ATT	INDEL	OS06G0163300	Intronic	NA	NA	LOC_Os06g06780	harpin-induced protein, putative, expressed
Chr.2	9928109	9928109	A	A	ATT	INDEL	OS02G0272900	Exonic	CDS	FrameShift	LOC_Os02g17304	resistance protein, putative, expressed
Chr.2	6452287	6452287	A	A	ATT	INDEL	OS02G0215200	Intronic	NA	NA	LOC_Os02g12380	histone deacetylase, putative, expressed
Chr.11	4250441	4250441	A	A	ATT	INDEL	OS11G0183900	Intronic	NA	NA	LOC_Os11g08100	eukaryotic aspartyl protease domain containing protein, expressed
Chr.11	22273837	22273837	A	A	ATT	INDEL	OS11G0587600	Intronic	NA	NA	LOC_Os11g37700	pleiotropic drug resistance protein, putative, expressed
Chr.10	19172402	19172402	A	A	ATT	INDEL	OS10G0502600	Exonic	3UTR	NA	LOC_Os10g35870	cytochrome b5-like Heme/Steroid binding domain containing protein, expressed
Chr.10	2361525	2361525	A	ATTACTC	ATTACTG	INDEL	OS10G0138100	Intronic	NA	NA	LOC_Os10g04860	aldehyde oxidase, putative, expressed
Chr.6	301490	301490	A	A	ATTGATTTGAT	INDEL	OS06G0104300	Intronic	NA	NA	LOC_Os06g01490	monocopper oxidase, putative, expressed
Chr.5	6192684	6192686	ATT	A	ATTT	INDEL	OS05G0199500	Intronic	NA	NA	LOC_Os05g11060	galactosyltransferase family protein, putative, expressed
Chr8	13956286	13956287	AT	A	ATTT	INDEL	OS08G0320400	Intronic	NA	NA	LOC_Os08g23150	indole-3-glycerol phosphate synthase, chloroplast precursor, putative, expressed
Chr.5	23873792	23873792	A	A	ATTT	INDEL	OS05G0485500	Exonic	3UTR	NA	LOC_Os05g40720	glycosyl transferase, family 8, putative, expressed
Chr.1	25055933	25055933	A	A	ATTT	INDEL	OS01G0627600	Exonic	3UTR	NA	LOC_Os01g43720	cytochrome P450 72A1, putative, expressed
Chr1	30259734	30259735	AT	A	ATTTT	INDEL	OS01G0725800	Intronic	NA	NA	LOC_Os01g52640	suppressor of phythchrome A, putative, expressed
Chr.4	317663	317663	T	A	C	SNP	OS04G0104900	Intronic	NA	NA	LOC_Os04g01470	O-methyltransferase, putative, expressed
Chr7	15409540	15409540	T	A	C	SNP	OS07G0448800	Exonic	3UTR	NA	LOC_Os07g26690	aquaporin protein, putative, expressed

Chr.11	16938120	16938120	A	AAC	C	INDEL	OS11G0482200	Intronic	NA	NA	LOC_Os11g29210	resistance-gene-interacting protein, putative, expressed
Chr8	1619297	1619297	A	AGCCGCC	C	INDEL	OS08G0128500	Intronic	NA	NA	LOC_Os08g03450	ribosomal protein L37, putative, expressed
Chr.2	34043779	34043789	CTTTTTTTTTT	C	C	INDEL	OS02G0799300	Intronic	NA	NA	LOC_Os02g55590	expressed protein
Chr.5	268586	268588	CAA	CA	C	INDEL	OS05G0105000	Intronic	NA	NA	LOC_Os05g01470	methionine S-methyltransferase, putative, expressed
Chr.4	33974094	33974097	CAAA	CA	C	INDEL	OS04G0665600	Intronic	NA	NA	LOC_Os04g56990	Myb-like DNA-binding domain containing protein, putative, expressed
Chr.4	30173848	30173859	CAAAAAAAAAAA	CAA	C	INDEL	OS04G0598000	Intronic	NA	NA	LOC_Os04g50970	seed specific protein Bn15D1B, putative, expressed
Chr.6	297799	297800	CA	CAA	C	INDEL	OS06G0104300	Intronic	NA	NA	LOC_Os06g01490	monocopper oxidase, putative, expressed
Chr.8	3294973	3294976	CAAA	CAA	C	INDEL	OS08G0156800	Intronic	NA	NA	LOC_Os08g06040	60S ribosomal protein L34, putative, expressed
Chr.3	22618368	22618369	CA	CAA	C	INDEL	OS03G0603600	Intronic	NA	NA	LOC_Os03g40670	glycerophosphoryl diester phosphodiesterase family protein, putative, expressed
Chr.4	31239226	31239229	CAAA	CAA	C	INDEL	OS04G0615700	Exonic	5UTR	NA	LOC_Os04g52540	retrotransposon protein, putative, unclassified, expressed
Chr.2	34674556	34674557	CA	CAA	C	INDEL	OS02G0810900	Intronic	NA	NA	LOC_Os02g56600	no apical meristem protein, putative, expressed
Chr.4	24323147	24323149	CAA	CAAAA	C	INDEL	OS04G0486900	Intronic	NA	NA	LOC_Os04g40980	Divergent PAP2 family domain containing protein, expressed
Chr7	27731510	27731513	CAAA	CAAAA	C	INDEL	OS07G0658400	Intronic	NA	NA	LOC_Os07g46460	ferredoxin-dependent glutamate synthase, chloroplast precursor, putative, expressed
Chr.8	9385738	9385742	CAATT	CAATTAATT	C	INDEL	OS08G0253800	Intronic	NA	NA	LOC_Os08g15420	CSLC3 - cellulose synthase-like family C, expressed
Chr.1	27081888	27081892	CAGAG	CAGAGAG	C	INDEL	OS01G0663300	Exonic	5UTR	NA	LOC_Os01g47400	OsMan01 - Endo-Beta-Mannanase, expressed
Chr.2	21935462	21935465	CCGG	CCGGCGG	C	INDEL	OS02G0572400	Exonic	5UTR	NA	LOC_Os02g36340	riboflavin biosynthesis protein ribAB, chloroplast precursor, putative, expressed
Chr.4	333974	333978	CCTTT	CCTTTCTTT	C	INDEL	OS04G0105100	Intronic	NA	NA	LOC_Os04g01490	zinc finger, C3HC4 type, domain containing protein, expressed
Chr.8	5364555	5364558	CTTT	CT	C	INDEL	OS08G0191600	Intronic	NA	NA	LOC_Os08g09240	autophagy-related protein, putative, expressed
Chr.6	13481367	13481370	CTTT	CT	C	INDEL	OS06G0338200	Intronic	NA	NA	LOC_Os06g23114	copper methylamine oxidase precursor, putative, expressed
Chr.11	9057857	9057859	CTT	CT	C	INDEL	OS11G0265200	Intronic	NA	NA	LOC_Os11g16390	expressed protein
Chr.3	20793762	20793768	CTAATTT	CT	C	INDEL	OS03G0571900	Intronic	NA	NA	LOC_Os03g37490	MATE efflux family protein, putative, expressed
Chr.5	26928280	26928282	CTT	CT	C	INDEL	OS05G0542500	Exonic	CDS	FrameShift	LOC_Os05g46480	late embryogenesis abundant protein, group 3, putative, expressed
Chr.2	35561507	35561509	CTT	CT	C	INDEL	OS02G0827400	Exonic	3UTR	NA	LOC_Os02g58100	expressed protein
Chr.10	15405102	15405103	CT	CTTT	C	INDEL	OS10G0431900	Intronic	NA	NA	LOC_Os10g29620	tyrosine protein kinase domain containing protein, putative, expressed
Chr.1	13903423	13903423	A	T	C	SNP	OS01G0348900	Exonic	3UTR	NA	LOC_Os01g24710	jacalin-like lectin domain containing protein, expressed
Chr.11	27487277	27487277	A	T	C	SNP	OS11G0679700	Exonic	CDS	NA	LOC_Os11g45400	glycerol-3-phosphate acyltransferase, putative, expressed
Chr7	21146581	21146581	T	TCCCCTTCC	C	INDEL	OS07G0537900	Exonic	5UTR	NA	LOC_Os07g35340	TKL_IRAK_DUF26-lc.14 - DUF26 kinases have homology to DUF26 containing loci, expressed
Chr.6	13480784	13480785	CT	TT	C	INDEL	OS06G0338200	Intronic	NA	NA	LOC_Os06g23114	copper methylamine oxidase precursor, putative, expressed
Chr.4	5846837	5846837	T	T	C	SNP	OS04G0186400	Exonic	5UTR	NA	LOC_Os04g10750	inorganic phosphate transporter, putative, expressed
Chr.4	5846635	5846635	T	T	C	SNP	OS04G0186400	Exonic	5UTR	NA	LOC_Os04g10750	inorganic phosphate transporter, putative, expressed
Chr.4	5846602	5846602	T	T	C	SNP	OS04G0186400	Exonic	5UTR	NA	LOC_Os04g10750	inorganic phosphate transporter, putative, expressed
Chr.4	5846322	5846322	T	T	C	SNP	OS04G0186400	Exonic	5UTR	NA	LOC_Os04g10750	inorganic phosphate transporter, putative, expressed
Chr.4	5846478	5846478	G	G	C	SNP	OS04G0186400	Exonic	5UTR	NA	LOC_Os04g10750	inorganic phosphate transporter, putative, expressed
Chr.4	5846455	5846455	G	G	C	SNP	OS04G0186400	Exonic	5UTR	NA	LOC_Os04g10750	inorganic phosphate transporter, putative, expressed
Chr.4	5847316	5847316	A	A	C	SNP	OS04G0186400	Exonic	5UTR	NA	LOC_Os04g10750	inorganic phosphate transporter, putative, expressed
Chr.4	5847004	5847004	A	A	C	SNP	OS04G0186400	Exonic	5UTR	NA	LOC_Os04g10750	inorganic phosphate transporter, putative, expressed
Chr.4	5849088	5849088	T	T	C	SNP	OS04G0186400	Exonic	3UTR	NA	LOC_Os04g10750	inorganic phosphate transporter, putative, expressed
Chr.4	5848914	5848914	G	G	C	SNP	OS04G0186400	Exonic	CDS	Silent	LOC_Os04g10750	inorganic phosphate transporter, putative, expressed
Chr7	925590	925590	T	T	C	SNP	OS07G0116900	Exonic	3UTR	NA	LOC_Os07g02570	NB-ARC domain containing protein, expressed
Chr7	3605895	3605895	T	T	C	SNP	OS07G0167100	Intronic	NA	NA	LOC_Os07g07260	glutamine-dependent NAD, putative, expressed
Chr7	4263980	4263980	T	T	C	SNP	OS07G0180900	Exonic	CDS	Silent	LOC_Os07g08330	ribosomal protein L4, putative, expressed
Chr7	4265012	4265012	T	T	C	SNP	OS07G0180900	Intronic	NA	NA	LOC_Os07g08330	ribosomal protein L4, putative, expressed
Chr7	4265090	4265090	T	T	C	SNP	OS07G0180900	Intronic	NA	NA	LOC_Os07g08330	ribosomal protein L4, putative, expressed
Chr7	4333130	4333130	T	T	C	SNP	OS07G0182100	Intronic	NA	NA	LOC_Os07g08430	indole-3-glycerol phosphate lyase, chloroplast precursor, putative, expressed
Chr7	4506027	4506027	T	T	C	SNP	OS07G0185200	Intronic	NA	NA	LOC_Os07g08760	START domain containing protein, putative, expressed
Chr7	4666271	4666271	T	T	C	SNP	OS07G0187400	Intronic	NA	NA	LOC_Os07g08970	uncharacterized protein ycf45, putative, expressed
Chr7	8078190	8078190	T	T	C	SNP	OS07G0245100	Exonic	3UTR	NA	LOC_Os07g14150	cytidine deaminase, putative, expressed
Chr7	9036350	9036350	T	T	C	SNP	OS07G0259700	Exonic	CDS	Silent	LOC_Os07g15600	H-BTB6 - Bric-a-Brac, Tramtrack, Broad Complex BTB domain with H family conserved sequence, expressed
Chr7	16897534	16897534	T	T	C	SNP	OS07G0471300	Intronic	NA	NA	LOC_Os07g28850	retrotransposon protein, putative, unclassified, expressed
Chr7	18336846	18336846	T	T	C	SNP	OS07G0492000	Intronic	NA	NA	LOC_Os07g30970	nucleoside diphosphate kinase, putative, expressed
Chr7	20217029	20217029	T	T	C	SNP	OS07G0522500	Intronic	NA	NA	LOC_Os07g33780	pleiotropic drug resistance protein 5, putative, expressed
Chr7	21280703	21280703	T	T	C	SNP	OS07G0539900	Exonic	CDS	Missense	LOC_Os07g35560	glucan endo-1,3-beta-glucosidase precursor, putative, expressed
Chr7	21280867	21280867	T	T	C	SNP	OS07G0539900	Exonic	CDS	Silent	LOC_Os07g35560	glucan endo-1,3-beta-glucosidase precursor, putative, expressed
Chr7	21281118	21281118	T	T	C	SNP	OS07G0539900	Exonic	CDS	Silent	LOC_Os07g35560	glucan endo-1,3-beta-glucosidase precursor, putative, expressed
Chr7	21415362	21415362	T	T	C	SNP	OS07G0541900	Exonic	CDS	Missense	LOC_Os07g35750	TKL_IRAK_DUF26-ld.3 - DUF26 kinases have homology to DUF26 containing loci, expressed
Chr7	21415393	21415393	T	T	C	SNP	OS07G0541900	Exonic	CDS	Silent	LOC_Os07g35750	TKL_IRAK_DUF26-ld.3 - DUF26 kinases have homology to DUF26 containing loci, expressed
Chr7	21415455	21415455	T	T	C	SNP	OS07G0541900	Exonic	CDS	Missense	LOC_Os07g35750	TKL_IRAK_DUF26-ld.3 - DUF26 kinases have homology to DUF26 containing loci, expressed
Chr7	21417700	21417700	T	T	C	SNP	OS07G0541900	Exonic	CDS	Silent	LOC_Os07g35750	TKL_IRAK_DUF26-ld.3 - DUF26 kinases have homology to DUF26 containing loci, expressed
Chr7	22370588	22370588	T	T	C	SNP	OS07G0560300	Exonic	CDS	Missense	LOC_Os07g37350	armadillo/beta-catenin repeat family protein, putative, expressed
Chr7	24398172	24398172	T	T	C	SNP	OS07G0598200	Intronic	NA	NA	LOC_Os07g40710	circadian clock coupling factor-related, putative, expressed
Chr7	24700561	24700561	T	T	C	SNP	OS07G0603600	Intronic	NA	NA	LOC_Os07g41230	esterase, putative, expressed
Chr7	24700860	24700860	T	T	C	SNP	OS07G0603600	Intronic	NA	NA	LOC_Os07g41230	esterase, putative, expressed
Chr7	25442185	25442185	T	T	C	SNP	OS07G0617000	Intronic	NA	NA	LOC_Os07g42510	AP2 domain containing protein, expressed
Chr7	26404720	26404720	T	T	C	SNP	OS07G0635900	Exonic	3UTR	NA	LOC_Os07g44180	OsRCI2-10 - Hydrophobic protein LTI6A, expressed
Chr7	26769514	26769514	T	T	C	SNP	OS07G0642800	Intronic	NA	NA	LOC_Os07g44830	proline-rich family protein, putative, expressed
Chr7	27723157	27723157	T	T	C	SNP	OS07G0658400	Exonic	3UTR	NA	LOC_Os07g46460	ferredoxin-dependent glutamate synthase, chloroplast precursor, putative, expressed
Chr7	27723315	27723315	T	T	C	SNP	OS07G0658400	Exonic	3UTR	NA	LOC_Os07g46460	ferredoxin-dependent glutamate synthase, chloroplast precursor, putative, expressed
Chr7	27724886	27724886	T	T	C	SNP	OS07G0658400	Intronic	NA	NA	LOC_Os07g46460	ferredoxin-dependent glutamate synthase, chloroplast precursor, putative, expressed
Chr7	27725788	27725788	T	T	C	SNP	OS07G0658400	Intronic	NA	NA	LOC_Os07g46460	ferredoxin-dependent glutamate synthase, chloroplast precursor, putative, expressed
Chr7	27727077	27727077	T	T	C	SNP	OS07G0658400	Intronic	NA	NA	LOC_Os07g46460	ferredoxin-dependent glutamate synthase, chloroplast precursor, putative, expressed
Chr7	27727141	27727141	T	T	C	SNP	OS07G0658400	Intronic	NA	NA	LOC_Os07g46460	ferredoxin-dependent glutamate synthase, chloroplast precursor, putative, expressed
Chr7	27727407	27727407	T	T	C	SNP	OS07G0658400	Exonic	CDS	NA	LOC_Os07g46460	ferredoxin-dependent glutamate synthase, chloroplast precursor, putative, expressed
Chr7	27727743	27727743	T	T	C	SNP	OS07G0658400	Intronic	NA	NA	LOC_Os07g46460	ferredoxin-dependent glutamate synthase, chloroplast precursor, putative, expressed
Chr7	27727786	27727786	T	T	C	SNP	OS07G0658400	Intronic	NA	NA	LOC_Os07g46460	ferredoxin-dependent glutamate synthase, chloroplast precursor, putative, expressed
Chr7	27727996	27727996	T	T	C	SNP	OS07G0658400	Intronic	NA	NA	LOC_Os07g46460	ferredoxin-dependent glutamate synthase, chloroplast precursor, putative, expressed
Chr7	27729790	27729790	T	T	C	SNP	OS07G0658400	Intronic	NA	NA	LOC_Os07g46460	ferredoxin-dependent glutamate synthase, chloroplast precursor, putative, expressed
Chr7	27729937	27729937	T	T	C	SNP	OS07G0658400	Exonic	CDS	NA	LOC_Os07g46460	ferredoxin-dependent glutamate synthase, chloroplast precursor, putative, expressed
Chr7	27729963	27729963	T	T	C	SNP	OS07G0658400	Exonic	CDS	NA	LOC_Os07g46460	ferredoxin-dependent glutamate synthase, chloroplast precursor, putative, expressed
Chr7	27730476	27730476	T	T	C	SNP	OS07G0658400	Intronic	NA	NA	LOC_Os07g46460	ferredoxin-dependent glutamate synthase, chloroplast precursor, putative, expressed
Chr7	27730579	27730579	T	T	C	SNP	OS07G0658400	Intronic	NA	NA	LOC_Os07g46460	ferredoxin-dependent glutamate synthase, chloroplast precursor, putative, expressed
Chr7	27733982	27733982	T	T	C	SNP	OS07G0658400	Intronic	NA	NA	LOC_Os07g46460	ferredoxin-dependent glutamate synthase, chloroplast precursor, putative, expressed
Chr7	27734559	27734559	T	T	C	SNP	OS07G0658400	Intronic	NA	NA	LOC_Os07g46460	ferredoxin-dependent glutamate synthase, chloroplast precursor, putative, expressed
Chr7	27735431	27735431	T	T	C	SNP	OS07G0658400	Intronic	NA	NA	LOC_Os07g46460	ferredoxin-dependent glutamate synthase, chloroplast precursor, putative, expressed
Chr7	27736513	27736513	T	T	C	SNP	OS07G0658400	Intronic	NA	NA	LOC_Os07g46460	ferredoxin-dependent glutamate synthase, chloroplast precursor, putative, expressed
Chr7	27736788	27736788	T	T	C	SNP	OS07G0658400	Intronic	NA	NA	LOC_Os07g46460	ferredoxin-dependent glutamate synthase, chloroplast precursor, putative, expressed

Chr7	27737166	27737166	T	T	C	SNP	OS07G0658400	Intronic	NA	NA	LOC_Os07g46460	ferredoxin-dependent glutamate synthase, chloroplast precursor, putative, expressed
Chr7	28124072	28124072	T	T	C	SNP	OS07G0665900	Intronic	NA	NA	LOC_Os07g47040	expressed protein
Chr7	1568609	1568609	G	G	C	SNP	OS07G0129700	Intronic	NA	NA	LOC_Os07g03770	Homeobox domain containing protein, expressed
Chr7	4264052	4264052	G	G	C	SNP	OS07G0180900	Exonic	CDS	Missense	LOC_Os07g08330	ribosomal protein L4, putative, expressed
Chr7	4264223	4264223	G	G	C	SNP	OS07G0180900	Exonic	CDS	Silent	LOC_Os07g08330	ribosomal protein L4, putative, expressed
Chr7	10232312	10232312	G	G	C	SNP	OS07G0274700	Intronic	NA	NA	LOC_Os07g17330	B12D protein, putative, expressed
Chr7	21246585	21246585	G	G	C	SNP	OS07G0539400	Exonic	CDS	Missense	LOC_Os07g35520	glucan endo-1,3-beta-glucosidase precursor, putative, expressed
Chr7	26483389	26483389	G	G	C	SNP	OS07G0637300	Intronic	NA	NA	LOC_Os07g44330	kinase, putative, expressed
Chr7	27723323	27723323	G	G	C	SNP	OS07G0658400	Exonic	3UTR	NA	LOC_Os07g46460	ferredoxin-dependent glutamate synthase, chloroplast precursor, putative, expressed
Chr7	27726978	27726978	G	G	C	SNP	OS07G0658400	Intronic	NA	NA	LOC_Os07g46460	ferredoxin-dependent glutamate synthase, chloroplast precursor, putative, expressed
Chr7	27727236	27727236	G	G	C	SNP	OS07G0658400	Exonic	CDS	NA	LOC_Os07g46460	ferredoxin-dependent glutamate synthase, chloroplast precursor, putative, expressed
Chr7	27727598	27727598	G	G	C	SNP	OS07G0658400	Intronic	NA	NA	LOC_Os07g46460	ferredoxin-dependent glutamate synthase, chloroplast precursor, putative, expressed
Chr7	27736510	27736510	G	G	C	SNP	OS07G0658400	Intronic	NA	NA	LOC_Os07g46460	ferredoxin-dependent glutamate synthase, chloroplast precursor, putative, expressed
Chr7	28382634	28382634	G	G	C	SNP	OS07G0671400	Exonic	5UTR	NA	LOC_Os07g47480	heavy metal-associated domain containing protein, expressed
Chr7	2646632	2646632	T	T	C	SNP	OS07G0150700	Intronic	NA	NA	LOC_Os07g05620	CAMK_KIN1/SNF1/Nim1_like.28 - CAMK includes calcium/calmodulin depedent protein kinases, expressed
Chr7	7109296	7109296	T	T	C	SNP	OS07G0227200	Exonic	3UTR	NA	LOC_Os07g12470	expressed protein
Chr7	8076367	8076367	T	T	C	SNP	OS07G0245100	Exonic	5UTR	NA	LOC_Os07g14150	cytidine deaminase, putative, expressed
Chr7	8077243	8077243	T	T	C	SNP	OS07G0245100	Exonic	CDS	NA	LOC_Os07g14150	cytidine deaminase, putative, expressed
Chr7	8077683	8077683	T	T	C	SNP	OS07G0245100	Intronic	NA	NA	LOC_Os07g14150	cytidine deaminase, putative, expressed
Chr7	8968913	8968913	T	T	C	SNP	OS07G0258400	Exonic	CDS	Missense	LOC_Os07g15460	metal transporter Nramp6, putative, expressed
Chr7	10232039	10232039	T	T	C	SNP	OS07G0274700	Intronic	NA	NA	LOC_Os07g17330	B12D protein, putative, expressed
Chr7	15407933	15407933	T	T	C	SNP	OS07G0448800	Intronic	NA	NA	LOC_Os07g26690	aquaporin protein, putative, expressed
Chr7	17411392	17411392	T	T	C	SNP	OS07G0479300	Intronic	NA	NA	LOC_Os07g29620	OsSCP39 - Putative Serine Carboxypeptidase homologue, expressed
Chr7	17412636	17412636	T	T	C	SNP	OS07G0479300	Intronic	NA	NA	LOC_Os07g29620	OsSCP39 - Putative Serine Carboxypeptidase homologue, expressed
Chr7	18840841	18840841	T	T	C	SNP	OS07G0500300	Intronic	NA	NA	LOC_Os07g31720	GTPase activating protein, putative, expressed
Chr7	18841058	18841058	T	T	C	SNP	OS07G0500300	Intronic	NA	NA	LOC_Os07g31720	GTPase activating protein, putative, expressed
Chr7	18841866	18841866	T	T	C	SNP	OS07G0500300	Intronic	NA	NA	LOC_Os07g31720	GTPase activating protein, putative, expressed
Chr7	21381029	21381029	T	T	C	SNP	OS07G0541400	Exonic	3UTR	NA	LOC_Os07g35690	TKL_IRAK_DUF26-lc.3 - DUF26 kinases have homology to DUF26 containing loci, expressed
Chr7	21381120	21381120	T	T	C	SNP	OS07G0541400	Exonic	3UTR	NA	LOC_Os07g35690	TKL_IRAK_DUF26-lc.3 - DUF26 kinases have homology to DUF26 containing loci, expressed
Chr7	21415594	21415594	T	T	C	SNP	OS07G0541900	Exonic	CDS	Missense	LOC_Os07g35750	TKL_IRAK_DUF26-ld.3 - DUF26 kinases have homology to DUF26 containing loci, expressed
Chr7	21415602	21415602	T	T	C	SNP	OS07G0541900	Exonic	CDS	Missense	LOC_Os07g35750	TKL_IRAK_DUF26-ld.3 - DUF26 kinases have homology to DUF26 containing loci, expressed
Chr7	23651829	23651829	T	T	C	SNP	OS07G0583600	Intronic	NA	NA	LOC_Os07g39470	gibberellin response modulator protein, putative, expressed
Chr7	23653844	23653844	T	T	C	SNP	OS07G0583600	Exonic	3UTR	NA	LOC_Os07g39470	gibberellin response modulator protein, putative, expressed
Chr7	27172813	27172813	T	T	C	SNP	OS07G0650100	Exonic	CDS	Silent	LOC_Os07g45550	expressed protein
Chr7	27173056	27173056	T	T	C	SNP	OS07G0650100	Exonic	CDS	Silent	LOC_Os07g45550	expressed protein
Chr7	27174518	27174518	T	T	C	SNP	OS07G0650100	Exonic	CDS	Missense	LOC_Os07g45550	expressed protein
Chr7	27175349	27175349	T	T	C	SNP	OS07G0650100	Exonic	CDS	Silent	LOC_Os07g45550	expressed protein
Chr7	848970	848970	G	G	C	SNP	OS07G0115500	Exonic	CDS	NA	LOC_Os07g02460	expressed protein
Chr7	18840746	18840746	G	G	C	SNP	OS07G0500300	Intronic	NA	NA	LOC_Os07g31720	GTPase activating protein, putative, expressed
Chr7	27172888	27172888	G	G	C	SNP	OS07G0650100	Exonic	CDS	Silent	LOC_Os07g45550	expressed protein
Chr7	27174447	27174447	G	G	C	SNP	OS07G0650100	Exonic	CDS	Missense	LOC_Os07g45550	expressed protein
Chr7	8076848	8076848	A	A	C	SNP	OS07G0245100	Exonic	5UTR	NA	LOC_Os07g14150	cytidine deaminase, putative, expressed
Chr7	9038122	9038122	A	A	C	SNP	OS07G0259700	Intronic	NA	NA	LOC_Os07g15600	H-BTB6 - Bric-a-Brac, Tramtrack, Broad Complex BTB domain with H family conserved sequence, expressed
Chr7	18842041	18842041	A	A	C	SNP	OS07G0500300	Exonic	CDS	Silent	LOC_Os07g31720	GTPase activating protein, putative, expressed
Chr7	27174479	27174479	A	A	C	SNP	OS07G0650100	Exonic	CDS	Missense	LOC_Os07g45550	expressed protein
Chr7	22862572	22862574	CCT	CCT	C	INDEL	OS07G0568600	Exonic	5UTR	NA	LOC_Os07g38120	CAMK_CAMK_like.34 - CAMK includes calcium/calmodulin depedent protein kinases, expressed
Chr7	26484744	26484746	CCA	CCA	C	INDEL	OS07G0637300	Intronic	NA	NA	LOC_Os07g44330	kinase, putative, expressed
Chr7	7110000	7110001	CA	CA	C	INDEL	OS07G0227200	Exonic	3UTR	NA	LOC_Os07g12470	expressed protein
Chr7	1848630	1848630	A	A	C	SNP	OS07G0134500	Exonic	3UTR	NA	LOC_Os07g04210	Ser/Thr protein phosphatase family protein, putative, expressed
Chr7	4264347	4264347	A	A	C	SNP	OS07G0180900	Intronic	NA	NA	LOC_Os07g08330	ribosomal protein L4, putative, expressed
Chr7	4264986	4264986	A	A	C	SNP	OS07G0180900	Intronic	NA	NA	LOC_Os07g08330	ribosomal protein L4, putative, expressed
Chr7	4506573	4506573	A	A	C	SNP	OS07G0185200	Exonic	5UTR	NA	LOC_Os07g08760	START domain containing protein, putative, expressed
Chr7	16897462	16897462	A	A	C	SNP	OS07G0471300	Intronic	NA	NA	LOC_Os07g28850	retrotransposon protein, putative, unclassified, expressed
Chr7	20214803	20214803	A	A	C	SNP	OS07G0522500	Intronic	NA	NA	LOC_Os07g33780	pleiotropic drug resistance protein 5, putative, expressed
Chr7	21414562	21414562	A	A	C	SNP	OS07G0541900	Exonic	3UTR	NA	LOC_Os07g35750	TKL_IRAK_DUF26-ld.3 - DUF26 kinases have homology to DUF26 containing loci, expressed
Chr7	21417747	21417747	A	A	C	SNP	OS07G0541900	Exonic	CDS	Missense	LOC_Os07g35750	TKL_IRAK_DUF26-ld.3 - DUF26 kinases have homology to DUF26 containing loci, expressed
Chr7	22889422	22889422	A	A	C	SNP	OS07G0569100	Exonic	3UTR	NA	LOC_Os07g38170	remorin, putative, expressed
Chr7	26481987	26481987	A	A	C	SNP	OS07G0637300	Intronic	NA	NA	LOC_Os07g44330	kinase, putative, expressed
Chr7	26771672	26771672	A	A	C	SNP	OS07G0642800	Intronic	NA	NA	LOC_Os07g44830	proline-rich family protein, putative, expressed
Chr7	27725806	27725806	A	A	C	SNP	OS07G0658400	Intronic	NA	NA	LOC_Os07g46460	ferredoxin-dependent glutamate synthase, chloroplast precursor, putative, expressed
Chr7	27726984	27726984	A	A	C	SNP	OS07G0658400	Intronic	NA	NA	LOC_Os07g46460	ferredoxin-dependent glutamate synthase, chloroplast precursor, putative, expressed
Chr7	27727905	27727905	A	A	C	SNP	OS07G0658400	Intronic	NA	NA	LOC_Os07g46460	ferredoxin-dependent glutamate synthase, chloroplast precursor, putative, expressed
Chr7	27730181	27730181	A	A	C	SNP	OS07G0658400	Intronic	NA	NA	LOC_Os07g46460	ferredoxin-dependent glutamate synthase, chloroplast precursor, putative, expressed
Chr7	27730727	27730727	A	A	C	SNP	OS07G0658400	Intronic	NA	NA	LOC_Os07g46460	ferredoxin-dependent glutamate synthase, chloroplast precursor, putative, expressed
Chr7	27732631	27732631	A	A	C	SNP	OS07G0658400	Intronic	NA	NA	LOC_Os07g46460	ferredoxin-dependent glutamate synthase, chloroplast precursor, putative, expressed
Chr7	27734939	27734939	A	A	C	SNP	OS07G0658400	Intronic	NA	NA	LOC_Os07g46460	ferredoxin-dependent glutamate synthase, chloroplast precursor, putative, expressed
Chr7	27734967	27734967	A	A	C	SNP	OS07G0658400	Intronic	NA	NA	LOC_Os07g46460	ferredoxin-dependent glutamate synthase, chloroplast precursor, putative, expressed
Chr7	27737686	27737686	A	A	C	SNP	OS07G0658400	Intronic	NA	NA	LOC_Os07g46460	ferredoxin-dependent glutamate synthase, chloroplast precursor, putative, expressed
Chr7	849497	849497	T	T	C	SNP	OS07G0115500	Exonic	3UTR	NA	LOC_Os07g02460	expressed protein
Chr7	925382	925382	A	A	C	SNP	OS07G0116900	Exonic	3UTR	NA	LOC_Os07g02570	NB-ARC domain containing protein, expressed
Chr7	926725	926725	T	T	C	SNP	OS07G0116900	Exonic	CDS	Silent	LOC_Os07g02570	NB-ARC domain containing protein, expressed
Chr7	3606221	3606221	G	G	C	SNP	OS07G0167100	Intronic	NA	NA	LOC_Os07g07260	glutamine-dependent NAD, putative, expressed
Chr7	3663224	3663224	T	T	C	SNP	OS07G0168300	Exonic	3UTR	NA	LOC_Os07g07320	glutathione S-transferase, putative, expressed
Chr7	4263452	4263452	G	G	C	SNP	OS07G0180900	Exonic	CDS	Silent	LOC_Os07g08330	ribosomal protein L4, putative, expressed
Chr7	4484119	4484119	T	T	C	SNP	OS07G0184800	Exonic	CDS	Missense	LOC_Os07g08710	AT hook-containing DNA-binding protein, putative, expressed
Chr7	4506151	4506151	T	T	C	SNP	OS07G0185200	Exonic	CDS	Silent	LOC_Os07g08760	START domain containing protein, putative, expressed
Chr7	4507633	4507633	T	T	C	SNP	OS07G0185200	Exonic	5UTR	NA	LOC_Os07g08760	START domain containing protein, putative, expressed
Chr7	6968342	6968342	G	G	C	SNP	OS07G0225300	Exonic	CDS	Silent	LOC_Os07g12340	NAC domain-containing protein 67, putative, expressed
Chr7	7108434	7108434	A	A	C	SNP	OS07G0227200	Exonic	3UTR	NA	LOC_Os07g12470	expressed protein
Chr7	9038677	9038677	T	T	C	SNP	OS07G0259700	Exonic	CDS	Missense	LOC_Os07g15600	H-BTB6 - Bric-a-Brac, Tramtrack, Broad Complex BTB domain with H family conserved sequence, expressed
Chr7	9039215	9039215	A	A	C	SNP	OS07G0259700	Exonic	5UTR	NA	LOC_Os07g15600	H-BTB6 - Bric-a-Brac, Tramtrack, Broad Complex BTB domain with H family conserved sequence, expressed
Chr7	15604541	15604541	T	T	C	SNP	OS07G0452500	Exonic	5UTR	NA	LOC_Os07g26940	ORM1, putative, expressed
Chr7	18841140	18841140	T	T	C	SNP	OS07G0500300	Intronic	NA	NA	LOC_Os07g31720	GTPase activating protein, putative, expressed
Chr7	18841485	18841485	T	T	C	SNP	OS07G0500300	Intronic	NA	NA	LOC_Os07g31720	GTPase activating protein, putative, expressed
Chr7	19473789	19473789	A	A	C	SNP	OS07G0510800	Exonic	CDS	Missense	LOC_Os07g32650	retrotransposon protein, putative, unclassified, expressed

Chr7	19473860	19473860	T	T	C	SNP	OS07G0510800	Exonic	5UTR	NA	LOC_Os07g32650	retrotransposon protein, putative, unclassified, expressed
Chr7	20724687	20724687	T	T	C	SNP	OS07G0529600	Exonic	CDS	Silent	LOC_Os07g34570	FAD dependent oxidoreductase domain containing protein, expressed
Chr7	21246993	21246993	G	G	C	SNP	OS07G0539400	Exonic	CDS	Silent	LOC_Os07g35520	glucan endo-1,3-beta-glucosidase precursor, putative, expressed
Chr7	21280758	21280758	T	T	C	SNP	OS07G0539900	Exonic	CDS	Missense	LOC_Os07g35560	glucan endo-1,3-beta-glucosidase precursor, putative, expressed
Chr7	21281169	21281169	T	T	C	SNP	OS07G0539900	Exonic	CDS	Silent	LOC_Os07g35560	glucan endo-1,3-beta-glucosidase precursor, putative, expressed
Chr7	21281180	21281180	T	T	C	SNP	OS07G0539900	Exonic	CDS	Missense	LOC_Os07g35560	glucan endo-1,3-beta-glucosidase precursor, putative, expressed
Chr7	21281238	21281238	T	T	C	SNP	OS07G0539900	Exonic	CDS	Silent	LOC_Os07g35560	glucan endo-1,3-beta-glucosidase precursor, putative, expressed
Chr7	21281635	21281635	G	G	C	SNP	OS07G0539900	Exonic	CDS	Missense	LOC_Os07g35560	glucan endo-1,3-beta-glucosidase precursor, putative, expressed
Chr7	21378839	21378839	A	A	C	SNP	OS07G0541400	Exonic	CDS	Missense	LOC_Os07g35690	TKL_IRAK_DUF26-lc.3 - DUF26 kinases have homology to DUF26 containing loci, expressed
Chr7	21418469	21418469	T	T	C	SNP	OS07G0541900	Exonic	CDS	Silent	LOC_Os07g35750	TKL_IRAK_DUF26-ld.3 - DUF26 kinases have homology to DUF26 containing loci, expressed
Chr7	21418538	21418538	G	G	C	SNP	OS07G0541900	Exonic	CDS	Silent	LOC_Os07g35750	TKL_IRAK_DUF26-ld.3 - DUF26 kinases have homology to DUF26 containing loci, expressed
Chr7	21418811	21418811	G	G	C	SNP	OS07G0541900	Exonic	CDS	Silent	LOC_Os07g35750	TKL_IRAK_DUF26-ld.3 - DUF26 kinases have homology to DUF26 containing loci, expressed
Chr7	21419252	21419252	T	T	C	SNP	OS07G0541900	Exonic	5UTR	NA	LOC_Os07g35750	TKL_IRAK_DUF26-ld.3 - DUF26 kinases have homology to DUF26 containing loci, expressed
Chr7	22428899	22428899	T	T	C	SNP	OS07G0561800	Exonic	3UTR	NA	LOC_Os07g37454	urate anion exchanger, putative, expressed
Chr7	23059000	23059000	A	A	C	SNP	OS07G0571300	Exonic	3UTR	NA	LOC_Os07g38370	expressed protein
Chr7	23186774	23186774	T	T	C	SNP	OS07G0573600	Exonic	CDS	Missense	LOC_Os07g38600	REX1 DNA Repair family protein, expressed
Chr7	23283453	23283453	T	T	C	SNP	OS07G0575700	Exonic	CDS	Silent	LOC_Os07g38810	lectin receptor-type protein kinase, putative, expressed
Chr7	24398797	24398797	A	A	C	SNP	OS07G0598200	Intronic	NA	NA	LOC_Os07g40710	circadian clock coupling factor-related, putative, expressed
Chr7	24399996	24399996	T	T	C	SNP	OS07G0598200	Intronic	NA	NA	LOC_Os07g40710	circadian clock coupling factor-related, putative, expressed
Chr7	24700638	24700638	T	T	C	SNP	OS07G0603600	Intronic	NA	NA	LOC_Os07g41230	esterase, putative, expressed
Chr7	26104628	26104628	T	T	C	SNP	OS07G0629500	Exonic	5UTR	NA	LOC_Os07g43604	expressed protein
Chr7	26457400	26457400	G	G	C	SNP	OS07G0636800	Exonic	CDS	Silent	LOC_Os07g44280	dirigent, putative, expressed
Chr7	26603965	26603965	G	G	C	SNP	OS07G0639000	Intronic	NA	NA	LOC_Os07g44550	peroxidase precursor, putative, expressed
Chr7	26776722	26776722	T	T	C	SNP	OS07G0642900	Intronic	NA	NA	LOC_Os07g44840	bacterial transferase hexapeptide domain containing protein, expressed
Chr7	27172378	27172378	T	T	C	SNP	OS07G0650100	Exonic	CDS	Silent	LOC_Os07g45550	expressed protein
Chr7	27172402	27172402	A	A	C	SNP	OS07G0650100	Exonic	CDS	Silent	LOC_Os07g45550	expressed protein
Chr7	27174342	27174342	T	T	C	SNP	OS07G0650100	Exonic	CDS	Missense	LOC_Os07g45550	expressed protein
Chr7	27724014	27724014	A	A	C	SNP	OS07G0658400	Intronic	NA	NA	LOC_Os07g46460	ferredoxin-dependent glutamate synthase, chloroplast precursor, putative, expressed
Chr7	27725844	27725844	A	A	C	SNP	OS07G0658400	Intronic	NA	NA	LOC_Os07g46460	ferredoxin-dependent glutamate synthase, chloroplast precursor, putative, expressed
Chr7	27726736	27726736	A	A	C	SNP	OS07G0658400	Intronic	NA	NA	LOC_Os07g46460	ferredoxin-dependent glutamate synthase, chloroplast precursor, putative, expressed
Chr7	27727311	27727311	T	T	C	SNP	OS07G0658400	Exonic	CDS	NA	LOC_Os07g46460	ferredoxin-dependent glutamate synthase, chloroplast precursor, putative, expressed
Chr7	27727658	27727658	A	A	C	SNP	OS07G0658400	Intronic	NA	NA	LOC_Os07g46460	ferredoxin-dependent glutamate synthase, chloroplast precursor, putative, expressed
Chr7	27732205	27732205	A	A	C	SNP	OS07G0658400	Intronic	NA	NA	LOC_Os07g46460	ferredoxin-dependent glutamate synthase, chloroplast precursor, putative, expressed
Chr7	27736952	27736952	T	T	C	SNP	OS07G0658400	Exonic	CDS	NA	LOC_Os07g46460	ferredoxin-dependent glutamate synthase, chloroplast precursor, putative, expressed
Chr7	28670120	28670120	T	T	C	SNP	OS07G0677100	Intronic	NA	NA	LOC_Os07g48010	peroxidase precursor, putative, expressed
Chr7	28670126	28670126	T	T	C	SNP	OS07G0677100	Intronic	NA	NA	LOC_Os07g48010	peroxidase precursor, putative, expressed
Chr7	28670947	28670947	A	A	C	SNP	OS07G0677100	Exonic	CDS	Missense	LOC_Os07g48010	peroxidase precursor, putative, expressed
Chr7	6495521	6495523	CTT	CTT	C	INDEL	OS07G0217600	Intronic	NA	NA	LOC_Os07g11739	cytochrome P450, putative, expressed
Chr7	17410372	17410374	CAG	CAG	C	INDEL	OS07G0479300	Exonic	3UTR	NA	LOC_Os07g29620	OsSCP39 - Putative Serine Carboxypeptidase homologue, expressed
Chr7	17410707	17410708	CG	CG	C	INDEL	OS07G0479300	Intronic	NA	NA	LOC_Os07g29620	OsSCP39 - Putative Serine Carboxypeptidase homologue, expressed
Chr7	18840997	18840998	CT	CT	C	INDEL	OS07G0500300	Intronic	NA	NA	LOC_Os07g31720	GTPase activating protein, putative, expressed
Chr7	19656187	19656188	CT	CT	C	INDEL	OS07G0513000	Exonic	3UTR	NA	LOC_Os07g32880	ATP synthase gamma chain, putative, expressed
Chr7	21414575	21414576	CT	CT	C	INDEL	OS07G0541900	Exonic	3UTR	NA	LOC_Os07g35750	TKL_IRAK_DUF26-ld.3 - DUF26 kinases have homology to DUF26 containing loci, expressed
Chr7	26776232	26776233	CA	CA	C	INDEL	OS07G0642900	Intronic	NA	NA	LOC_Os07g44840	bacterial transferase hexapeptide domain containing protein, expressed
Chr7	27728992	27728993	CA	CA	C	INDEL	OS07G0658400	Intronic	NA	NA	LOC_Os07g46460	ferredoxin-dependent glutamate synthase, chloroplast precursor, putative, expressed
Chr7	27729138	27729139	CA	CA	C	INDEL	OS07G0658400	Intronic	NA	NA	LOC_Os07g46460	ferredoxin-dependent glutamate synthase, chloroplast precursor, putative, expressed
Chr7	27732532	27732534	CTT	CTT	C	INDEL	OS07G0658400	Intronic	NA	NA	LOC_Os07g46460	ferredoxin-dependent glutamate synthase, chloroplast precursor, putative, expressed
Chr.9	6996505	6996505	T	T	C	SNP	OS09G0294000	Intronic	NA	NA	LOC_Os09g12290	bifunctional aspartokinase/homoserine dehydrogenase, chloroplast precursor, putative, expressed
Chr.9	7003077	7003077	T	T	C	SNP	OS09G0294000	Intronic	NA	NA	LOC_Os09g12290	bifunctional aspartokinase/homoserine dehydrogenase, chloroplast precursor, putative, expressed
Chr.9	7199669	7199669	T	T	C	SNP	OS09G0297000	Intronic	NA	NA	LOC_Os09g12560	ferrochelatase-2, chloroplast precursor, putative, expressed
Chr.9	7281644	7281644	T	T	C	SNP	OS09G0298500	Exonic	3UTR	NA	LOC_Os09g12720	zinc finger, C3HC4 type domain containing protein, expressed
Chr.9	7279821	7279821	T	T	C	SNP	OS09G0298500	Intronic	NA	NA	LOC_Os09g12720	zinc finger, C3HC4 type domain containing protein, expressed
Chr.9	7280755	7280755	T	T	C	SNP	OS09G0298500	Intronic	NA	NA	LOC_Os09g12720	zinc finger, C3HC4 type domain containing protein, expressed
Chr.9	9359827	9359827	T	T	C	SNP	OS09G0321900	Intronic	NA	NA	LOC_Os09g15320	ubiquitin-conjugating enzyme, putative, expressed
Chr.9	9360067	9360067	T	T	C	SNP	OS09G0321900	Intronic	NA	NA	LOC_Os09g15320	ubiquitin-conjugating enzyme, putative, expressed
Chr.9	10254108	10254108	T	T	C	SNP	OS09G0337300	Exonic	CDS	Silent	LOC_Os09g16760	ankyrin repeat family protein, expressed
Chr.9	10523056	10523056	T	T	C	SNP	OS09G0341600	Exonic	3UTR	NA	LOC_Os09g17152	OsFBX319 - F-box domain containing protein, expressed
Chr.9	10523193	10523193	T	T	C	SNP	OS09G0341600	Exonic	CDS	Missense	LOC_Os09g17152	OsFBX319 - F-box domain containing protein, expressed
Chr.9	10523567	10523567	T	T	C	SNP	OS09G0341600	Exonic	CDS	Missense	LOC_Os09g17152	OsFBX319 - F-box domain containing protein, expressed
Chr.9	10523624	10523624	T	T	C	SNP	OS09G0341600	Exonic	CDS	Missense	LOC_Os09g17152	OsFBX319 - F-box domain containing protein, expressed
Chr.9	10523924	10523924	T	T	C	SNP	OS09G0341600	Exonic	CDS	Missense	LOC_Os09g17152	OsFBX319 - F-box domain containing protein, expressed
Chr.9	16960745	16960745	T	T	C	SNP	OS09G0452200	Intronic	NA	NA	LOC_Os09g27890	lysM domain-containing GPI-anchored protein precursor, putative, expressed
Chr.9	16961540	16961540	T	T	C	SNP	OS09G0452200	Intronic	NA	NA	LOC_Os09g27890	lysM domain-containing GPI-anchored protein precursor, putative, expressed
Chr.9	16980231	16980231	T	T	C	SNP	OS09G0452900	Exonic	3UTR	NA	LOC_Os09g27950	galactosyltransferase, putative, expressed
Chr.9	17082516	17082516	T	T	C	SNP	OS09G0454600	Intronic	NA	NA	LOC_Os09g28160	phosphate carrier protein, mitochondrial precursor, putative, expressed
Chr.9	18851499	18851499	T	T	C	SNP	OS09G0488700	Intronic	NA	NA	LOC_Os09g31360	expressed protein
Chr.9	10523791	10523791	G	G	C	SNP	OS09G0341600	Exonic	CDS	Missense	LOC_Os09g17152	OsFBX319 - F-box domain containing protein, expressed
Chr.9	21189653	21189653	G	G	C	SNP	OS09G0538400	Exonic	3UTR	NA	LOC_Os09g36730	MYB family transcription factor, putative, expressed
Chr.9	9369142	9369142	T	T	C	SNP	OS09G0322000	Intronic	NA	NA	LOC_Os09g15330	transporter family protein, putative, expressed
Chr.9	7005651	7005651	A	A	C	SNP	OS09G0294000	Intronic	NA	NA	LOC_Os09g12290	bifunctional aspartokinase/homoserine dehydrogenase, chloroplast precursor, putative, expressed
Chr.9	7201605	7201605	A	A	C	SNP	OS09G0297000	Intronic	NA	NA	LOC_Os09g12560	ferrochelatase-2, chloroplast precursor, putative, expressed
Chr.9	2132641	2132641	T	T	C	SNP	OS09G0127800	Exonic	5UTR	NA	LOC_Os09g04110	coatomer alpha subunit, putative, expressed
Chr.9	3052500	3052500	T	T	C	SNP	OS09G0240200	Intronic	NA	NA	LOC_Os09g06464	CCT/B-box zinc finger protein, putative, expressed
Chr.9	6389618	6389618	G	G	C	SNP	OS09G0286600	Exonic	CDS	Silent	LOC_Os09g11460	AP2 domain containing protein, expressed
Chr.9	6996088	6996088	A	A	C	SNP	OS09G0294000	Intronic	NA	NA	LOC_Os09g12290	bifunctional aspartokinase/homoserine dehydrogenase, chloroplast precursor, putative, expressed
Chr.9	6997458	6997458	T	T	C	SNP	OS09G0294000	Intronic	NA	NA	LOC_Os09g12290	bifunctional aspartokinase/homoserine dehydrogenase, chloroplast precursor, putative, expressed
Chr.9	6999755	6999755	G	G	C	SNP	OS09G0294000	Intronic	NA	NA	LOC_Os09g12290	bifunctional aspartokinase/homoserine dehydrogenase, chloroplast precursor, putative, expressed
Chr.9	9567471	9567471	A	A	C	SNP	OS09G0325700	Exonic	5UTR	NA	LOC_Os09g15670	protein phosphatase 2C, putative, expressed
Chr.9	10523500	10523500	T	T	C	SNP	OS09G0341600	Exonic	CDS	Silent	LOC_Os09g17152	OsFBX319 - F-box domain containing protein, expressed
Chr.9	10523761	10523761	A	A	C	SNP	OS09G0341600	Exonic	CDS	Silent	LOC_Os09g17152	OsFBX319 - F-box domain containing protein, expressed
Chr.9	10523992	10523992	T	T	C	SNP	OS09G0341600	Exonic	CDS	Silent	LOC_Os09g17152	OsFBX319 - F-box domain containing protein, expressed
Chr.9	10523891	10523891	T	T	C	SNP	OS09G0341600	Exonic	CDS	Missense	LOC_Os09g17152	OsFBX319 - F-box domain containing protein, expressed
Chr.9	10524188	10524188	T	T	C	SNP	OS09G0341600	Exonic	CDS	Missense	LOC_Os09g17152	OsFBX319 - F-box domain containing protein, expressed
Chr.9	14976796	14976796	G	G	C	SNP	OS09G0417600	Exonic	CDS	Missense	LOC_Os09g25060	WRKY76, expressed
Chr.9	3583569	3583585	CTCTACTCTTCGCTTGT	CTCTACTCTTCGCTTGT	C	INDEL	OS09G0247600	Intronic	NA	NA	LOC_Os09g07290	GDSL-like lipase/acylhydrolase, putative, expressed

Chr.9	4579773	4579775	CAT	CAT	C	INDEL	OS09G0261900	Exonic	3UTR	NA	LOC_Os09g08710	expressed protein
Chr.9	6389116	6389119	CCGG	CCGG	C	INDEL	OS09G0286600	Exonic	CDS	InFrame	LOC_Os09g11460	AP2 domain containing protein, expressed
Chr.9	7198685	7198686	CT	CT	C	INDEL	OS09G0297000	Exonic	5UTR	NA	LOC_Os09g12560	ferrochelatase-2, chloroplast precursor, putative, expressed
Chr.9	7249759	7249769	CCTCTTTTTTT	CCTCTTTTTTT	C	INDEL	OS09G0298200	Exonic	5UTR	NA	LOC_Os09g12660	glucose-1-phosphate adenylyltransferase large subunit, chloroplast precursor, putative, expressed
Chr.9	10338640	10338643	CCCG	CCCG	C	INDEL	OS09G0338500	Exonic	5UTR	NA	LOC_Os09g16920	desaturase/cytochrome b5 protein, putative, expressed
Chr.9	15960792	15960793	CT	CT	C	INDEL	OS09G0434500	Intronic	NA	NA	LOC_Os09g26420	AP2 domain containing protein, expressed
Chr.9	16618060	16618063	CTCT	CTCT	C	INDEL	OS09G0445600	Exonic	5UTR	NA	LOC_Os09g27330	oxidoreductase/ transition metal ion binding protein, putative, expressed
Chr.9	16930975	16930992	CTCCGACGTGGCGGTGAA	CTCCGACGTGGCGGTGAA	C	INDEL	OS09G0451500	Exonic	5UTR	NA	LOC_Os09g27830	OsPDIL2-3 protein disulfide isomerase PDIL2-3, expressed
Chr.9	16932422	16932424	CAT	CAT	C	INDEL	OS09G0451500	Intronic	NA	NA	LOC_Os09g27830	OsPDIL2-3 protein disulfide isomerase PDIL2-3, expressed
Chr.9	17133004	17133019	CCGCCGCCGCCACCGG	CCGCCGCCGCCACCGG	C	INDEL	OS09G0455300	Exonic	CDS	InFrame	LOC_Os09g28210	bHelix-loop-helix transcription factor, putative, expressed
Chr.9	18850474	18850475	CA	CA	C	INDEL	OS09G0488700	Intronic	NA	NA	LOC_Os09g31360	expressed protein
Chr.9	18983044	18983052	CTTCTTCTT	CTTCTTCTT	C	INDEL	OS09G0491740	Intronic	NA	NA	LOC_Os09g31478	auxin efflux carrier component, putative, expressed
Chr.9	22771475	22771476	CT	CT	C	INDEL	OS09G0570400	Exonic	3UTR	NA	LOC_Os09g39680	transporter, major facilitator family, putative, expressed
Chr.8	26467463	26467463	T	T	C	SNP	OS08G0531300	Exonic	3UTR	NA	LOC_Os08g41910	Sua5/YciO/YrdC/YwlC family protein, putative, expressed
Chr.8	26466692	26466692	T	T	C	SNP	OS08G0531300	Exonic	5UTR	NA	LOC_Os08g41910	Sua5/YciO/YrdC/YwlC family protein, putative, expressed
Chr.8	26466627	26466627	T	T	C	SNP	OS08G0531300	Exonic	5UTR	NA	LOC_Os08g41910	Sua5/YciO/YrdC/YwlC family protein, putative, expressed
Chr.8	26465753	26465753	T	T	C	SNP	OS08G0531300	Intronic	NA	NA	LOC_Os08g41910	Sua5/YciO/YrdC/YwlC family protein, putative, expressed
Chr.8	26421779	26421779	T	T	C	SNP	OS08G0530400	Intronic	NA	NA	LOC_Os08g41830	sulfite oxidase, putative, expressed
Chr.8	26419096	26419096	T	T	C	SNP	OS08G0530400	Intronic	NA	NA	LOC_Os08g41830	sulfite oxidase, putative, expressed
Chr.8	26418628	26418628	T	T	C	SNP	OS08G0530400	Intronic	NA	NA	LOC_Os08g41830	sulfite oxidase, putative, expressed
Chr.8	20252254	20252254	T	T	C	SNP	OS08G0422600	Intronic	NA	NA	LOC_Os08g32690	expressed protein
Chr.8	12268556	12268556	T	T	C	SNP	OS08G0299400	Intronic	NA	NA	LOC_Os08g20420	MGD2, putative, expressed
Chr.8	12268229	12268229	T	T	C	SNP	OS08G0299400	Intronic	NA	NA	LOC_Os08g20420	MGD2, putative, expressed
Chr.8	8329175	8329175	T	T	C	SNP	OS08G0237000	Exonic	3UTR	NA	LOC_Os08g13920	glycosyl hydrolases family 16, putative, expressed
Chr.8	7367795	7367795	T	T	C	SNP	OS08G0221300	Intronic	NA	NA	LOC_Os08g12449	expressed protein
Chr.8	6206046	6206046	T	T	C	SNP	OS08G0206400	Exonic	5UTR	NA	LOC_Os08g10550	potassium transporter, putative, expressed
Chr.8	3549057	3549057	T	T	C	SNP	OS08G0160500	Intronic	NA	NA	LOC_Os08g06380	CSLF6 - cellulose synthase-like family F; beta1,3;1,4 glucan synthase, expressed
Chr.8	3424200	3424200	T	T	C	SNP	OS08G0158500	Exonic	CDS	Missense	LOC_Os08g06200	stomatin-like protein 2, putative, expressed
Chr.8	3423519	3423519	T	T	C	SNP	OS08G0158500	Intronic	NA	NA	LOC_Os08g06200	stomatin-like protein 2, putative, expressed
Chr.8	3423428	3423428	T	T	C	SNP	OS08G0158500	Intronic	NA	NA	LOC_Os08g06200	stomatin-like protein 2, putative, expressed
Chr.8	3423271	3423271	T	T	C	SNP	OS08G0158500	Intronic	NA	NA	LOC_Os08g06200	stomatin-like protein 2, putative, expressed
Chr.8	3423008	3423008	T	T	C	SNP	OS08G0158500	Intronic	NA	NA	LOC_Os08g06200	stomatin-like protein 2, putative, expressed
Chr.8	3422982	3422982	T	T	C	SNP	OS08G0158500	Exonic	CDS	Silent	LOC_Os08g06200	stomatin-like protein 2, putative, expressed
Chr.8	3422686	3422686	T	T	C	SNP	OS08G0158500	Intronic	NA	NA	LOC_Os08g06200	stomatin-like protein 2, putative, expressed
Chr.8	3422639	3422639	T	T	C	SNP	OS08G0158500	Intronic	NA	NA	LOC_Os08g06200	stomatin-like protein 2, putative, expressed
Chr.8	3422610	3422610	T	T	C	SNP	OS08G0158500	Intronic	NA	NA	LOC_Os08g06200	stomatin-like protein 2, putative, expressed
Chr.8	3421561	3421561	T	T	C	SNP	OS08G0158500	Intronic	NA	NA	LOC_Os08g06200	stomatin-like protein 2, putative, expressed
Chr.8	3421538	3421538	T	T	C	SNP	OS08G0158500	Intronic	NA	NA	LOC_Os08g06200	stomatin-like protein 2, putative, expressed
Chr.8	3390709	3390709	T	T	C	SNP	OS08G0157900	Exonic	CDS	Missense	LOC_Os08g06140	no apical meristem protein, putative, expressed
Chr.8	3364762	3364762	T	T	C	SNP	OS08G0157600	Intronic	NA	NA	LOC_Os08g06110	MYB family transcription factor, putative, expressed
Chr.8	3363853	3363853	T	T	C	SNP	OS08G0157600	Intronic	NA	NA	LOC_Os08g06110	MYB family transcription factor, putative, expressed
Chr.8	3363189	3363189	T	T	C	SNP	OS08G0157600	Intronic	NA	NA	LOC_Os08g06110	MYB family transcription factor, putative, expressed
Chr.8	3282451	3282451	T	T	C	SNP	OS08G0156600	Exonic	CDS	Missense	LOC_Os08g06010	transporter, major facilitator family, putative, expressed
Chr.8	3281971	3281971	T	T	C	SNP	OS08G0156600	Intronic	NA	NA	LOC_Os08g06010	transporter, major facilitator family, putative, expressed
Chr.8	2508300	2508300	T	T	C	SNP	OS08G0144400	Intronic	NA	NA	LOC_Os08g04920	OsDegp11 - Putative Deg protease homologue, expressed
Chr.8	1836227	1836227	T	T	C	SNP	OS08G0132600	Exonic	3UTR	NA	LOC_Os08g03840	expressed protein
Chr.8	1835236	1835236	T	T	C	SNP	OS08G0132600	Intronic	NA	NA	LOC_Os08g03840	expressed protein
Chr.8	1566947	1566947	T	T	C	SNP	OS08G0127100	Exonic	3UTR	NA	LOC_Os08g03350	amino acid transporter, putative, expressed
Chr.8	582876	582876	T	T	C	SNP	OS08G0111200	Intronic	NA	NA	LOC_Os08g01940	non-lysosomal glucosylceramidase, putative, expressed
Chr.8	8760743	8760743	G	G	C	SNP	OS08G0243500	Intronic	NA	NA	LOC_Os08g14570	NADPH reductase, putative, expressed
Chr.8	3422309	3422309	G	G	C	SNP	OS08G0158500	Exonic	CDS	Silent	LOC_Os08g06200	stomatin-like protein 2, putative, expressed
Chr.8	3421496	3421496	G	G	C	SNP	OS08G0158500	Intronic	NA	NA	LOC_Os08g06200	stomatin-like protein 2, putative, expressed
Chr.8	1620713	1620713	G	G	C	SNP	OS08G0128500	Intronic	NA	NA	LOC_Os08g03450	ribosomal protein L37, putative, expressed
Chr.8	1564670	1564670	G	G	C	SNP	OS08G0127100	Intronic	NA	NA	LOC_Os08g03350	amino acid transporter, putative, expressed
Chr.8	14418243	14418243	T	T	C	SNP	OS08G0327400	Intronic	NA	NA	LOC_Os08g23810	enoyl-acyl-carrier-protein reductase NADH, chloroplast precursor, expressed
Chr.8	7940172	7940173	CT	CT	C	INDEL	OS08G0230200	Intronic	NA	NA	LOC_Os08g13350	expressed protein
Chr.8	26465777	26465777	A	A	C	SNP	OS08G0531300	Intronic	NA	NA	LOC_Os08g41910	Sua5/YciO/YrdC/YwlC family protein, putative, expressed
Chr.8	26418913	26418913	A	A	C	SNP	OS08G0530400	Intronic	NA	NA	LOC_Os08g41830	sulfite oxidase, putative, expressed
Chr.8	7941527	7941527	A	A	C	SNP	OS08G0230200	Intronic	NA	NA	LOC_Os08g13350	expressed protein
Chr.8	3424322	3424322	A	A	C	SNP	OS08G0158500	Exonic	CDS	Missense	LOC_Os08g06200	stomatin-like protein 2, putative, expressed
Chr.8	3423474	3423474	A	A	C	SNP	OS08G0158500	Intronic	NA	NA	LOC_Os08g06200	stomatin-like protein 2, putative, expressed
Chr.8	3422841	3422841	A	A	C	SNP	OS08G0158500	Intronic	NA	NA	LOC_Os08g06200	stomatin-like protein 2, putative, expressed
Chr.8	3422364	3422364	A	A	C	SNP	OS08G0158500	Intronic	NA	NA	LOC_Os08g06200	stomatin-like protein 2, putative, expressed
Chr.8	3422249	3422249	A	A	C	SNP	OS08G0158500	Exonic	CDS	Silent	LOC_Os08g06200	stomatin-like protein 2, putative, expressed
Chr.8	3421972	3421972	A	A	C	SNP	OS08G0158500	Intronic	NA	NA	LOC_Os08g06200	stomatin-like protein 2, putative, expressed
Chr.8	3421107	3421107	A	A	C	SNP	OS08G0158500	Intronic	NA	NA	LOC_Os08g06200	stomatin-like protein 2, putative, expressed
Chr.8	3367276	3367276	A	A	C	SNP	OS08G0157600	Intronic	NA	NA	LOC_Os08g06110	MYB family transcription factor, putative, expressed
Chr.8	3340118	3340118	A	A	C	SNP	OS08G0157500	Intronic	NA	NA	LOC_Os08g06100	O-methyltransferase, putative, expressed
Chr.8	3339047	3339047	A	A	C	SNP	OS08G0157500	Intronic	NA	NA	LOC_Os08g06100	O-methyltransferase, putative, expressed
Chr.8	3338770	3338770	T	T	C	SNP	OS08G0157500	Intronic	NA	NA	LOC_Os08g06100	O-methyltransferase, putative, expressed
Chr.8	3293878	3293878	A	A	C	SNP	OS08G0156800	Intronic	NA	NA	LOC_Os08g06040	60S ribosomal protein L34, putative, expressed
Chr.8	3283937	3283937	A	A	C	SNP	OS08G0156600	Exonic	CDS	Missense	LOC_Os08g06010	transporter, major facilitator family, putative, expressed
Chr.8	689307	689307	T	T	C	SNP	OS08G0112900	Intronic	NA	NA	LOC_Os08g02094	GDSL-like lipase/acylhydrolase, putative, expressed
Chr.8	690533	690533	T	T	C	SNP	OS08G0112900	Intronic	NA	NA	LOC_Os08g02094	GDSL-like lipase/acylhydrolase, putative, expressed
Chr.8	1565259	1565259	G	G	C	SNP	OS08G0127100	Exonic	CDS	Silent	LOC_Os08g03350	amino acid transporter, putative, expressed
Chr.8	1565520	1565520	G	G	C	SNP	OS08G0127100	Exonic	CDS	Silent	LOC_Os08g03350	amino acid transporter, putative, expressed
Chr.8	2508512	2508512	T	T	C	SNP	OS08G0144400	Exonic	3UTR	NA	LOC_Os08g04920	OsDegp11 - Putative Deg protease homologue, expressed
Chr.8	3243419	3243419	A	A	C	SNP	OS08G0155900	Exonic	3UTR	NA	LOC_Os08g05960	expressed protein
Chr.8	3366189	3366189	T	T	C	SNP	OS08G0157600	Intronic	NA	NA	LOC_Os08g06110	MYB family transcription factor, putative, expressed
Chr.8	3547332	3547332	G	G	C	SNP	OS08G0160500	Exonic	CDS	Silent	LOC_Os08g06380	CSLF6 - cellulose synthase-like family F; beta1,3;1,4 glucan synthase, expressed
Chr.8	6209311	6209311	T	T	C	SNP	OS08G0206400	Intronic	NA	NA	LOC_Os08g10550	potassium transporter, putative, expressed
Chr.8	8328847	8328847	T	T	C	SNP	OS08G0237000	Exonic	CDS	Silent	LOC_Os08g13920	glycosyl hydrolases family 16, putative, expressed
Chr.8	8328873	8328873	G	G	C	SNP	OS08G0237000	Exonic	CDS	Missense	LOC_Os08g13920	glycosyl hydrolases family 16, putative, expressed
Chr.8	9386628	9386628	G	G	C	SNP	OS08G0253800	Exonic	CDS	Silent	LOC_Os08g15420	CSLC3 - cellulose synthase-like family C, expressed
Chr.8	12267714	12267714	T	T	C	SNP	OS08G0299400	Intronic	NA	NA	LOC_Os08g20420	MGD2, putative, expressed

Chr.8	12268057	12268057	T	T	C	SNP	OS08G0299400	Intronic	NA	NA	LOC_Os08g20420	MGD2, putative, expressed
Chr.8	15658571	15658571	T	T	C	SNP	OS08G0345700	Exonic	5UTR	NA	LOC_Os08g25720	pyrophosphate--fructose 6-phosphate 1-phosphotransferase subunit alpha, putative, expressed
Chr.8	26918237	26918237	G	G	C	SNP	OS08G0538600	Intronic	NA	NA	LOC_Os08g42590	mtN19, putative, expressed
Chr.8	2507984	2507985	CT	CT	C	INDEL	OS08G0144400	Intronic	NA	NA	LOC_Os08g04920	OsDegp11 - Putative Deg protease homologue, expressed
Chr.8	3282062	3282069	CATCAAAT	CATCAAAT	C	INDEL	OS08G0156600	Intronic	NA	NA	LOC_Os08g06010	transporter, major facilitator family, putative, expressed
Chr.8	3363871	3363872	CT	CT	C	INDEL	OS08G0157600	Intronic	NA	NA	LOC_Os08g06110	MYB family transcription factor, putative, expressed
Chr.8	3366185	3366187	CTT	CTT	C	INDEL	OS08G0157600	Intronic	NA	NA	LOC_Os08g06110	MYB family transcription factor, putative, expressed
Chr.8	3548852	3548857	CTAAAA	CTAAAA	C	INDEL	OS08G0160500	Intronic	NA	NA	LOC_Os08g06380	CSLF6 - cellulose synthase-like family F; beta1,3;1,4 glucan synthase, expressed
Chr.6	297587	297587	T	T	C	SNP	OS06G0104300	Exonic	3UTR	NA	LOC_Os06g01490	monocopper oxidase, putative, expressed
Chr.6	1362187	1362187	T	T	C	SNP	OS06G0125200	Exonic	3UTR	NA	LOC_Os06g03520	DUF581 domain containing protein, expressed
Chr.6	1385599	1385599	T	T	C	SNP	OS06G0125800	Intronic	NA	NA	LOC_Os06g03580	zinc RING finger protein, putative, expressed
Chr.6	1556642	1556642	T	T	C	SNP	OS06G0129400	Intronic	NA	NA	LOC_Os06g03860	uncharacterized membrane protein, putative, expressed
Chr.6	1558283	1558283	T	T	C	SNP	OS06G0129400	Exonic	CDS	Silent	LOC_Os06g03860	uncharacterized membrane protein, putative, expressed
Chr.6	1558629	1558629	T	T	C	SNP	OS06G0129400	Intronic	NA	NA	LOC_Os06g03860	uncharacterized membrane protein, putative, expressed
Chr.6	1559536	1559536	T	T	C	SNP	OS06G0129400	Intronic	NA	NA	LOC_Os06g03860	uncharacterized membrane protein, putative, expressed
Chr.6	1559909	1559909	T	T	C	SNP	OS06G0129400	Intronic	NA	NA	LOC_Os06g03860	uncharacterized membrane protein, putative, expressed
Chr.6	1560739	1560739	T	T	C	SNP	OS06G0129400	Exonic	3UTR	NA	LOC_Os06g03860	uncharacterized membrane protein, putative, expressed
Chr.6	2323060	2323060	T	T	C	SNP	OS06G0143900	Intronic	NA	NA	LOC_Os06g05180	coatomer subunit beta, putative, expressed
Chr.6	4323640	4323640	T	T	C	SNP	OS06G0185900	Intronic	NA	NA	LOC_Os06g08670	glutathione peroxidase, putative, expressed
Chr.6	8761396	8761396	T	T	C	SNP	OS06G0265000	Intronic	NA	NA	LOC_Os06g15420	asparagine synthetase, putative, expressed
Chr.6	13481866	13481866	T	T	C	SNP	OS06G0338200	Intronic	NA	NA	LOC_Os06g23114	copper methylamine oxidase precursor, putative, expressed
Chr.6	13482871	13482871	T	T	C	SNP	OS06G0338200	Intronic	NA	NA	LOC_Os06g23114	copper methylamine oxidase precursor, putative, expressed
Chr.6	13484086	13484086	T	T	C	SNP	OS06G0338200	Exonic	CDS	Silent	LOC_Os06g23114	copper methylamine oxidase precursor, putative, expressed
Chr.6	17067460	17067460	T	T	C	SNP	OS06G0493100	Intronic	NA	NA	LOC_Os06g29730	RALFL28 - Rapid ALkalinization Factor RALF family protein precursor, expressed
Chr.6	20011947	20011947	T	T	C	SNP	OS06G0534800	Exonic	3UTR	NA	LOC_Os06g34390	zinc finger, C3HC4 type domain containing protein, expressed
Chr.6	20898371	20898371	T	T	C	SNP	OS06G0551400	Intronic	NA	NA	LOC_Os06g35814	ras-related protein, putative, expressed
Chr.6	21599706	21599706	T	T	C	SNP	OS06G0562600	Intronic	NA	NA	LOC_Os06g36700	T-complex protein, putative, expressed
Chr.6	21896469	21896469	T	T	C	SNP	OS06G0567200	Intronic	NA	NA	LOC_Os06g37080	L-ascorbate oxidase precursor, putative, expressed
Chr.6	21896483	21896483	T	T	C	SNP	OS06G0567200	Intronic	NA	NA	LOC_Os06g37080	L-ascorbate oxidase precursor, putative, expressed
Chr.6	21896697	21896697	T	T	C	SNP	OS06G0567200	Intronic	NA	NA	LOC_Os06g37080	L-ascorbate oxidase precursor, putative, expressed
Chr.6	22353141	22353141	T	T	C	SNP	OS06G0575400	Exonic	CDS	Silent	LOC_Os06g37750	S-locus-like receptor protein kinase, putative, expressed
Chr.6	22353554	22353554	T	T	C	SNP	OS06G0575400	Exonic	CDS	Missense	LOC_Os06g37750	S-locus-like receptor protein kinase, putative, expressed
Chr.6	23468616	23468616	T	T	C	SNP	OS06G0596300	Intronic	NA	NA	LOC_Os06g39520	myristoyl-acyl carrier protein thioesterase, chloroplast precursor, putative, expressed
Chr.6	24401255	24401255	T	T	C	SNP	OS06G0611900	Intronic	NA	NA	LOC_Os06g40940	glycine dehydrogenase, putative, expressed
Chr.6	24403444	24403444	T	T	C	SNP	OS06G0611900	Intronic	NA	NA	LOC_Os06g40940	glycine dehydrogenase, putative, expressed
Chr.6	28192955	28192955	T	T	C	SNP	OS06G0678200	Exonic	CDS	Silent	LOC_Os06g46450	polyprenyl synthetase, putative, expressed
Chr.6	682105	682105	G	G	C	SNP	OS06G0112100	Exonic	CDS	Missense	LOC_Os06g02210	bark storage protein A precursor, putative, expressed
Chr.6	1361764	1361764	G	G	C	SNP	OS06G0125200	Intronic	NA	NA	LOC_Os06g03520	DUF581 domain containing protein, expressed
Chr.6	1557220	1557220	G	G	C	SNP	OS06G0129400	Intronic	NA	NA	LOC_Os06g03860	uncharacterized membrane protein, putative, expressed
Chr.6	2005020	2005020	G	G	C	SNP	OS06G0137500	Exonic	CDS	Missense	LOC_Os06g04600	Brix domain containing protein, putative, expressed
Chr.6	7710734	7710734	G	G	C	SNP	OS06G0248500	Exonic	3UTR	NA	LOC_Os06g13870	U-box protein CMPG1, putative, expressed
Chr.6	9091548	9091548	G	G	C	SNP	OS06G0270900	Intronic	NA	NA	LOC_Os06g15990	aldehyde dehydrogenase, putative, expressed
Chr.6	10687248	10687248	G	G	C	SNP	OS06G0291500	Exonic	3UTR	NA	LOC_Os06g18820	serine threonine kinase, putative, expressed
Chr.6	13479424	13479424	G	G	C	SNP	OS06G0338200	Intronic	NA	NA	LOC_Os06g23114	copper methylamine oxidase precursor, putative, expressed
Chr.6	13481446	13481446	G	G	C	SNP	OS06G0338200	Intronic	NA	NA	LOC_Os06g23114	copper methylamine oxidase precursor, putative, expressed
Chr.6	13482082	13482082	G	G	C	SNP	OS06G0338200	Intronic	NA	NA	LOC_Os06g23114	copper methylamine oxidase precursor, putative, expressed
Chr.6	16265827	16265827	G	G	C	SNP	OS06G0480000	Exonic	CDS	Missense	LOC_Os06g28590	bg55, putative, expressed
Chr.6	21599513	21599513	G	G	C	SNP	OS06G0562600	Intronic	NA	NA	LOC_Os06g36700	T-complex protein, putative, expressed
Chr.6	21601657	21601657	G	G	C	SNP	OS06G0562600	Exonic	CDS	Silent	LOC_Os06g36700	T-complex protein, putative, expressed
Chr.6	21895723	21895723	G	G	C	SNP	OS06G0567200	Intronic	NA	NA	LOC_Os06g37080	L-ascorbate oxidase precursor, putative, expressed
Chr.6	22353518	22353518	G	G	C	SNP	OS06G0575400	Exonic	CDS	Missense	LOC_Os06g37750	S-locus-like receptor protein kinase, putative, expressed
Chr.6	30303197	30303197	G	G	C	SNP	OS06G0714200	Intronic	NA	NA	LOC_Os06g50030	CAMK_CAMK_like.30 - CAMK includes calcium/calmodulin depedent protein kinases, expressed
Chr.6	2323716	2323716	T	T	C	SNP	OS06G0143900	Intronic	NA	NA	LOC_Os06g05180	coatomer subunit beta, putative, expressed
Chr.6	2326427	2326427	T	T	C	SNP	OS06G0143900	Intronic	NA	NA	LOC_Os06g05180	coatomer subunit beta, putative, expressed
Chr.6	3363120	3363120	T	T	C	SNP	OS06G0166900	Exonic	CDS	Missense	LOC_Os06g07070	BRASSINOSTEROID INSENSITIVE 1-associated receptor kinase 1 precursor, putative, expressed
Chr.6	3454023	3454023	A	A	C	SNP	OS06G0168600	Intronic	NA	NA	LOC_Os06g07210	ribonucleoside-diphosphate reductase large subunit, putative, expressed
Chr.6	13480763	13480763	T	T	C	SNP	OS06G0338200	Intronic	NA	NA	LOC_Os06g23114	copper methylamine oxidase precursor, putative, expressed
Chr.6	17067549	17067549	T	T	C	SNP	OS06G0493100	Intronic	NA	NA	LOC_Os06g29730	RALFL28 - Rapid ALkalinization Factor RALF family protein precursor, expressed
Chr.6	27135996	27135996	T	T	C	SNP	OS06G0659300	Exonic	3UTR	NA	LOC_Os06g44890	villin protein, putative, expressed
Chr.6	27137899	27137899	T	T	C	SNP	OS06G0659300	Exonic	CDS	Silent	LOC_Os06g44890	villin protein, putative, expressed
Chr.6	27138881	27138881	A	A	C	SNP	OS06G0659300	Intronic	NA	NA	LOC_Os06g44890	villin protein, putative, expressed
Chr.6	27140529	27140529	T	T	C	SNP	OS06G0659300	Intronic	NA	NA	LOC_Os06g44890	villin protein, putative, expressed
Chr.6	27140787	27140787	T	T	C	SNP	OS06G0659300	Exonic	CDS	Silent	LOC_Os06g44890	villin protein, putative, expressed
Chr.6	27140851	27140851	A	A	C	SNP	OS06G0659300	Exonic	CDS	Missense	LOC_Os06g44890	villin protein, putative, expressed
Chr.6	27283395	27283395	G	G	C	SNP	OS06G0661900	Intronic	NA	NA	LOC_Os06g45110	DNA binding protein, putative, expressed
Chr.6	28191180	28191180	A	A	C	SNP	OS06G0678200	Exonic	3UTR	NA	LOC_Os06g46450	polyprenyl synthetase, putative, expressed
Chr.6	28192229	28192229	A	A	C	SNP	OS06G0678200	Intronic	NA	NA	LOC_Os06g46450	polyprenyl synthetase, putative, expressed
Chr.6	28194834	28194834	T	T	C	SNP	OS06G0678200	Intronic	NA	NA	LOC_Os06g46450	polyprenyl synthetase, putative, expressed
Chr.6	28194981	28194981	T	T	C	SNP	OS06G0678200	Intronic	NA	NA	LOC_Os06g46450	polyprenyl synthetase, putative, expressed
Chr.6	29145503	29145503	T	T	C	SNP	OS06G0696600	Exonic	CDS	Silent	LOC_Os06g48180	glycosyl hydrolases family 16, putative, expressed
Chr.6	29145778	29145778	T	T	C	SNP	OS06G0696600	Intronic	NA	NA	LOC_Os06g48180	glycosyl hydrolases family 16, putative, expressed
Chr.6	29145796	29145796	T	T	C	SNP	OS06G0696600	Intronic	NA	NA	LOC_Os06g48180	glycosyl hydrolases family 16, putative, expressed
Chr.6	31069366	31069366	T	T	C	SNP	OS06G0729300	Intronic	NA	NA	LOC_Os06g51310	PAZ domain containing protein, putative, expressed
Chr.6	31145383	31145383	T	T	C	SNP	OS06G0730600	Intronic	NA	NA	LOC_Os06g51410	OsPOP15 - Putative Prolyl Oligopeptidase homologue, expressed
Chr.6	1361785	1361785	A	A	C	SNP	OS06G0125200	Intronic	NA	NA	LOC_Os06g03520	DUF581 domain containing protein, expressed
Chr.6	1362294	1362294	A	A	C	SNP	OS06G0125200	Exonic	3UTR	NA	LOC_Os06g03520	DUF581 domain containing protein, expressed
Chr.6	1556888	1556888	A	A	C	SNP	OS06G0129400	Intronic	NA	NA	LOC_Os06g03860	uncharacterized membrane protein, putative, expressed
Chr.6	1559993	1559993	A	A	C	SNP	OS06G0129400	Intronic	NA	NA	LOC_Os06g03860	uncharacterized membrane protein, putative, expressed
Chr.6	23468650	23468650	A	A	C	SNP	OS06G0596300	Exonic	CDS	Silent	LOC_Os06g39520	myristoyl-acyl carrier protein thioesterase, chloroplast precursor, putative, expressed
Chr.6	24142673	24142673	A	A	C	SNP	OS06G0607200	Exonic	3UTR	NA	LOC_Os06g40510	CRAL/TRIO domain containing protein, expressed
Chr.6	27136531	27136531	A	A	C	SNP	OS06G0659300	Intronic	NA	NA	LOC_Os06g44890	villin protein, putative, expressed
Chr.6	27283014	27283014	A	A	C	SNP	OS06G0661900	Intronic	NA	NA	LOC_Os06g45110	DNA binding protein, putative, expressed
Chr.6	28195459	28195459	A	A	C	SNP	OS06G0678200	Intronic	NA	NA	LOC_Os06g46450	polyprenyl synthetase, putative, expressed
Chr.6	801797	801797	T	T	C	SNP	OS06G0113900	Intronic	NA	NA	LOC_Os06g02370	expressed protein
Chr.6	1362136	1362136	T	T	C	SNP	OS06G0125200	Exonic	3UTR	NA	LOC_Os06g03520	DUF581 domain containing protein, expressed

Chr.6	1361999	1361999	G	G	C	SNP	OS06G0125200	Exonic	CDS	Missense	LOC_Os06g03520	DUF581 domain containing protein, expressed
Chr.6	1414390	1414390	T	T	C	SNP	OS06G0126500	Exonic	5UTR	NA	LOC_Os06g03640	BAG domain containing protein, expressed
Chr.6	4331790	4331790	A	A	C	SNP	OS06G0186100	Exonic	CDS	Missense	LOC_Os06g08690	leucine-rich repeat receptor protein kinase EXS precursor, putative, expressed
Chr.6	4869589	4869589	G	G	C	SNP	OS06G0195800	Intronic	NA	NA	LOC_Os06g09560	heat shock protein DnaJ, putative, expressed
Chr.6	8184536	8184536	T	T	C	SNP	OS06G0256900	Exonic	5UTR	NA	LOC_Os06g14540	endoglucanase, putative, expressed
Chr.6	13476725	13476725	G	G	C	SNP	OS06G0338200	Exonic	CDS	Missense	LOC_Os06g23114	copper methylamine oxidase precursor, putative, expressed
Chr.6	13482482	13482482	T	T	C	SNP	OS06G0338200	Intronic	NA	NA	LOC_Os06g23114	copper methylamine oxidase precursor, putative, expressed
Chr.6	17067517	17067517	A	A	C	SNP	OS06G0493100	Intronic	NA	NA	LOC_Os06g29730	RALFL28 - Rapid ALkalinization Factor RALF family protein precursor, expressed
Chr.6	20677533	20677533	A	A	C	SNP	OS06G0546500	Exonic	CDS	Silent	LOC_Os06g35480	peroxidase precursor, putative, expressed
Chr.6	23113027	23113027	T	T	C	SNP	OS06G0589300	Exonic	5UTR	NA	LOC_Os06g38950	ABC transporter, ATP-binding protein, putative, expressed
Chr.6	23470351	23470351	G	G	C	SNP	OS06G0596300	Exonic	3UTR	NA	LOC_Os06g39520	myristoyl-acyl carrier protein thioesterase, chloroplast precursor, putative, expressed
Chr.6	23468249	23468249	T	T	C	SNP	OS06G0596300	Exonic	CDS	Silent	LOC_Os06g39520	myristoyl-acyl carrier protein thioesterase, chloroplast precursor, putative, expressed
Chr.6	27977851	27977851	T	T	C	SNP	OS06G0674100	Intronic	NA	NA	LOC_Os06g46160	expressed protein
Chr.6	29144968	29144968	T	T	C	SNP	OS06G0696600	Exonic	CDS	Missense	LOC_Os06g48180	glycosyl hydrolases family 16, putative, expressed
Chr.6	30106774	30106774	T	T	C	SNP	OS06G0711800	Exonic	5UTR	NA	LOC_Os06g49760	invertase/pectin methylesterase inhibitor family protein, putative, expressed
Chr.6	30301785	30301785	T	T	C	SNP	OS06G0714200	Exonic	5UTR	NA	LOC_Os06g50030	CAMK_CAMK_like.30 - CAMK includes calcium/calmodulin depedent protein kinases, expressed
Chr.6	30918772	30918772	T	T	C	SNP	OS06G0726800	Exonic	5UTR	NA	LOC_Os06g51110	cyclin, putative, expressed
Chr.6	31069645	31069645	A	A	C	SNP	OS06G0729300	Intronic	NA	NA	LOC_Os06g51310	PAZ domain containing protein, putative, expressed
Chr.6	300122	300123	CA	CA	C	INDEL	OS06G0104300	Intronic	NA	NA	LOC_Os06g01490	monocopper oxidase, putative, expressed
Chr.6	1362496	1362497	CA	CA	C	INDEL	OS06G0125200	Exonic	3UTR	NA	LOC_Os06g03520	DUF581 domain containing protein, expressed
Chr.6	1361836	1361840	CGTAT	CGTAT	C	INDEL	OS06G0125200	Intronic	NA	NA	LOC_Os06g03520	DUF581 domain containing protein, expressed
Chr.6	13482641	13482642	CT	CT	C	INDEL	OS06G0338200	Intronic	NA	NA	LOC_Os06g23114	copper methylamine oxidase precursor, putative, expressed
Chr.6	30993343	30993345	CCT	CCT	C	INDEL	OS06G0728000	Exonic	5UTR	NA	LOC_Os06g51220	HMG1/2, putative, expressed
Chr.5	27536164	27536164	G	G	C	SNP	OS05G0553800	Intronic	NA	NA	LOC_Os05g48030	anti-silencing protein, ASF1-like domain containing protein, expressed
Chr.5	25545279	25545279	T	T	C	SNP	OS05G0515200	Intronic	NA	NA	LOC_Os05g43910	cytochrome P450, putative, expressed
Chr.5	24781420	24781420	G	G	C	SNP	OS05G0503300	Intronic	NA	NA	LOC_Os05g42350	ferredoxin--nitrite reductase, putative, expressed
Chr.5	24607783	24607783	T	T	C	SNP	OS05G0499800	Exonic	3UTR	NA	LOC_Os05g42040	UDP-glucoronosyl and UDP-glucosyl transferase domain containing protein, expressed
Chr.5	24424741	24424741	T	T	C	SNP	OS05G0497150	Exonic	CDS	NA	LOC_Os05g41750	RecF/RecN/SMC N terminal domain containing protein, expressed
Chr.5	18844740	18844740	T	T	C	SNP	OS05G0389400	Exonic	CDS	Silent	LOC_Os05g32320	integral membrane transporter family protein, putative, expressed
Chr.5	17932306	17932306	A	A	C	SNP	OS05G0372200	Exonic	3UTR	NA	LOC_Os05g30880	nucleolar matrix protein-related, putative, expressed
Chr.5	15486564	15486564	T	T	C	SNP	OS05G0331200	Intronic	NA	NA	LOC_Os05g26660	NADH-ubiquinone oxidoreductase, mitochondrial precursor, putative, expressed
Chr.5	14308110	14308110	G	G	C	SNP	OS05G0312000	Intronic	NA	NA	LOC_Os05g24684	structural constituent of ribosome, putative, expressed
Chr.5	14246215	14246215	G	G	C	SNP	OS05G0311500	Exonic	3UTR	NA	LOC_Os05g24650	DUF567 domain containing protein, putative, expressed
Chr.5	14163987	14163987	T	T	C	SNP	OS05G0310500	Intronic	NA	NA	LOC_Os05g24550	Papain family cysteine protease domain containing protein, expressed
Chr.5	14162115	14162115	A	A	C	SNP	OS05G0310500	Intronic	NA	NA	LOC_Os05g24550	Papain family cysteine protease domain containing protein, expressed
Chr.5	5653781	5653781	A	A	C	SNP	OS05G0192100	Intronic	NA	NA	LOC_Os05g10370	acid phosphatase, putative, expressed
Chr.5	3758876	3758876	T	T	C	SNP	OS05G0163900	Intronic	NA	NA	LOC_Os05g07120	basic helix-loop-helix, putative, expressed
Chr.5	3758872	3758872	T	T	C	SNP	OS05G0163900	Intronic	NA	NA	LOC_Os05g07120	basic helix-loop-helix, putative, expressed
Chr.5	3757498	3757498	T	T	C	SNP	OS05G0163900	Intronic	NA	NA	LOC_Os05g07120	basic helix-loop-helix, putative, expressed
Chr.5	3757497	3757497	T	T	C	SNP	OS05G0163900	Intronic	NA	NA	LOC_Os05g07120	basic helix-loop-helix, putative, expressed
Chr.5	3733915	3733915	T	T	C	SNP	OS05G0163700	Intronic	NA	NA	LOC_Os05g07090	acyl-coenzyme A dehydrogenase, mitochondrial precursor, putative, expressed
Chr.5	3730666	3730666	A	A	C	SNP	OS05G0163700	Exonic	3UTR	NA	LOC_Os05g07090	acyl-coenzyme A dehydrogenase, mitochondrial precursor, putative, expressed
Chr.5	3610950	3610950	T	T	C	SNP	OS05G0161200	Intronic	NA	NA	LOC_Os05g06890	relA-SpoT like protein RSH4, putative, expressed
Chr.5	2721576	2721576	T	T	C	SNP	OS05G0147400	Intronic	NA	NA	LOC_Os05g05470	T-complex protein, putative, expressed
Chr.5	1002329	1002329	T	T	C	SNP	OS05G0119300	Exonic	5UTR	NA	LOC_Os05g02780	glycine-rich protein A3, putative, expressed
Chr.5	1002029	1002029	T	T	C	SNP	OS05G0119300	Exonic	5UTR	NA	LOC_Os05g02780	glycine-rich protein A3, putative, expressed
Chr.5	998383	998383	A	A	C	SNP	OS05G0119200	Intronic	NA	NA	LOC_Os05g02770	glycine-rich cell wall structural protein precursor, putative, expressed
Chr.5	742673	742673	T	T	C	SNP	OS05G0114000	Intronic	NA	NA	LOC_Os05g02310	soluble inorganic pyrophosphatase, putative, expressed
Chr.5	742412	742412	T	T	C	SNP	OS05G0114000	Intronic	NA	NA	LOC_Os05g02310	soluble inorganic pyrophosphatase, putative, expressed
Chr.5	741562	741562	G	G	C	SNP	OS05G0114000	Intronic	NA	NA	LOC_Os05g02310	soluble inorganic pyrophosphatase, putative, expressed
Chr.5	4863706	4863706	T	T	C	SNP	OS05G0180600	Intronic	NA	NA	LOC_Os05g08810	phosphatidylinositol 3-kinase, root isoform, putative, expressed
Chr.5	27959237	27959237	T	T	C	SNP	OS05G0562000	Exonic	CDS	Missense	LOC_Os05g48790	expressed protein
Chr.5	26826818	26826818	T	T	C	SNP	OS05G0540000	Exonic	CDS	Missense	LOC_Os05g46260	exostosin, putative, expressed
Chr.5	24777960	24777960	G	G	C	SNP	OS05G0503300	Intronic	NA	NA	LOC_Os05g42350	ferredoxin--nitrite reductase, putative, expressed
Chr.5	22070873	22070873	T	T	C	SNP	OS05G0449600	Intronic	NA	NA	LOC_Os05g37700	periplasmic beta-glucosidase precursor, putative, expressed
Chr.5	14297238	14297238	A	A	C	SNP	OS05G0312000	Intronic	NA	NA	LOC_Os05g24684	structural constituent of ribosome, putative, expressed
Chr.5	14164635	14164635	T	T	C	SNP	OS05G0310500	Intronic	NA	NA	LOC_Os05g24550	Papain family cysteine protease domain containing protein, expressed
Chr.5	2788639	2788639	T	T	C	SNP	OS05G0148700	Intronic	NA	NA	LOC_Os05g05600	ATA15 protein, putative, expressed
Chr.5	1995335	1995335	T	T	C	SNP	OS05G0134200	Intronic	NA	NA	LOC_Os05g04360	protein phosphatase 2C, putative, expressed
Chr.5	24671041	24671041	T	T	C	SNP	OS05G0501300	Intronic	NA	NA	LOC_Os05g42190	NADPH-dependent FMN reductase domain containing protein, expressed
Chr.5	26903310	26903310	T	T	C	SNP	OS05G0541900	Exonic	3UTR	NA	LOC_Os05g46430	60S ribosomal protein L28-1, putative, expressed
Chr.5	24104988	24104988	T	T	C	SNP	OS05G0490400	Exonic	CDS	Missense	LOC_Os05g41150	expressed protein
Chr.5	23749558	23749558	A	A	C	SNP	OS05G0482700	Intronic	NA	NA	LOC_Os05g40420	2,3-bisphosphoglycerate-independent phosphoglycerate mutase, putative, expressed
Chr.5	20277306	20277306	T	T	C	SNP	OS05G0414501	Exonic	3UTR	NA	LOC_Os05g34260	transposon protein, putative, Ac/Ds sub-class, expressed
Chr.5	14993605	14993605	T	T	C	SNP	OS05G0322900	Exonic	3UTR	NA	LOC_Os05g25770	WRKY45, expressed
Chr.5	14991644	14991644	G	G	C	SNP	OS05G0322900	Exonic	5UTR	NA	LOC_Os05g25770	WRKY45, expressed
Chr.5	2175040	2175040	T	T	C	SNP	OS05G0137400	Intronic	NA	NA	LOC_Os05g04630	retrotransposon protein, putative, SINE subclass, expressed
Chr.5	27700614	27700614	T	T	C	SNP	OS05G0557000	Intronic	NA	NA	LOC_Os05g48320	60S ribosomal protein L37a, putative, expressed
Chr.5	14295744	14295744	T	T	C	SNP	OS05G0312000	Exonic	CDS	Missense	LOC_Os05g24684	structural constituent of ribosome, putative, expressed
Chr.5	14165315	14165315	G	G	C	SNP	OS05G0310500	Exonic	3UTR	NA	LOC_Os05g24550	Papain family cysteine protease domain containing protein, expressed
Chr.5	14161521	14161521	T	T	C	SNP	OS05G0310500	Intronic	NA	NA	LOC_Os05g24550	Papain family cysteine protease domain containing protein, expressed
Chr.5	5653663	5653663	T	T	C	SNP	OS05G0192100	Intronic	NA	NA	LOC_Os05g10370	acid phosphatase, putative, expressed
Chr.5	3723998	3723998	T	T	C	SNP	OS05G0163400	Exonic	5UTR	NA	LOC_Os05g07070	zinc finger, C3HC4 type domain containing protein, expressed
Chr.5	340605	340605	G	G	C	SNP	OS05G0106200	Exonic	CDS	Missense	LOC_Os05g01570	auxin-induced protein 5NG4, putative, expressed
Chr.5	837802	837802	G	G	C	SNP	OS05G0115100	Exonic	3UTR	NA	LOC_Os05g02450	expressed protein
Chr.5	998526	998526	T	T	C	SNP	OS05G0119200	Intronic	NA	NA	LOC_Os05g02770	glycine-rich cell wall structural protein precursor, putative, expressed
Chr.5	1001031	1001031	T	T	C	SNP	OS05G0119300	Exonic	CDS	Missense	LOC_Os05g02780	glycine-rich protein A3, putative, expressed
Chr.5	1996676	1996676	T	T	C	SNP	OS05G0134200	Exonic	3UTR	NA	LOC_Os05g04360	protein phosphatase 2C, putative, expressed
Chr.5	1994320	1994320	T	T	C	SNP	OS05G0134200	Exonic	5UTR	NA	LOC_Os05g04360	protein phosphatase 2C, putative, expressed
Chr.5	3757882	3757882	T	T	C	SNP	OS05G0163900	Intronic	NA	NA	LOC_Os05g07120	basic helix-loop-helix, putative, expressed
Chr.5	4138236	4138236	T	T	C	SNP	OS05G0169000	Exonic	5UTR	NA	LOC_Os05g07690	thioredoxin, putative, expressed
Chr.5	5654640	5654640	T	T	C	SNP	OS05G0192100	Exonic	CDS	Missense	LOC_Os05g10370	acid phosphatase, putative, expressed
Chr.5	5653343	5653343	T	T	C	SNP	OS05G0192100	Intronic	NA	NA	LOC_Os05g10370	acid phosphatase, putative, expressed
Chr.5	13834242	13834242	A	A	C	SNP	OS05G0305900	Exonic	5UTR	NA	LOC_Os05g24010	receptor protein kinase-like, putative, expressed
Chr.5	21190039	21190039	T	T	C	SNP	OS05G0431700	Intronic	NA	NA	LOC_Os05g35650	peptide transporter PTR2, putative, expressed

Chr.5	22241613	22241613	G	G	C	SNP	OS05G0453300	Exonic	5UTR	NA	LOC_Os05g37930	expressed protein
Chr.5	23866172	23866172	A	A	C	SNP	OS05G0485300	Intronic	NA	NA	LOC_Os05g40700	transmembrane protein 56, putative, expressed
Chr.5	24281689	24281689	T	T	C	SNP	OS05G0494200	Exonic	CDS	Missense	LOC_Os05g41460	cysteine proteinase inhibitor precursor protein, putative, expressed
Chr.5	24326480	24326480	A	A	C	SNP	OS05G0495300	Exonic	3UTR	NA	LOC_Os05g41550	expressed protein
Chr.5	24327689	24327689	G	G	C	SNP	OS05G0495300	Exonic	5UTR	NA	LOC_Os05g41550	expressed protein
Chr.5	25546176	25546176	T	T	C	SNP	OS05G0515200	Exonic	3UTR	NA	LOC_Os05g43910	cytochrome P450, putative, expressed
Chr.5	25544448	25544448	G	G	C	SNP	OS05G0515200	Exonic	CDS	Missense	LOC_Os05g43910	cytochrome P450, putative, expressed
Chr.5	27959568	27959568	T	T	C	SNP	OS05G0562000	Intronic	NA	NA	LOC_Os05g48790	expressed protein
Chr.5	27959578	27959578	T	T	C	SNP	OS05G0562000	Intronic	NA	NA	LOC_Os05g48790	expressed protein
Chr.5	27959586	27959586	T	T	C	SNP	OS05G0562000	Intronic	NA	NA	LOC_Os05g48790	expressed protein
Chr.5	27959592	27959592	T	T	C	SNP	OS05G0562000	Intronic	NA	NA	LOC_Os05g48790	expressed protein
Chr.5	27959598	27959598	T	T	C	SNP	OS05G0562000	Intronic	NA	NA	LOC_Os05g48790	expressed protein
Chr.5	27959602	27959602	T	T	C	SNP	OS05G0562000	Intronic	NA	NA	LOC_Os05g48790	expressed protein
Chr.5	27959606	27959606	T	T	C	SNP	OS05G0562000	Intronic	NA	NA	LOC_Os05g48790	expressed protein
Chr.5	27959631	27959631	A	A	C	SNP	OS05G0562000	Intronic	NA	NA	LOC_Os05g48790	expressed protein
Chr.5	27959779	27959779	A	A	C	SNP	OS05G0562000	Intronic	NA	NA	LOC_Os05g48790	expressed protein
Chr.5	27960038	27960038	T	T	C	SNP	OS05G0562000	Intronic	NA	NA	LOC_Os05g48790	expressed protein
Chr.5	27960052	27960052	T	T	C	SNP	OS05G0562000	Intronic	NA	NA	LOC_Os05g48790	expressed protein
Chr.5	836332	836340	CCTTTCTCT	CCTTTCTCT	C	INDEL	OS05G0115100	Exonic	5UTR	NA	LOC_Os05g02450	expressed protein
Chr.5	873592	873593	CT	CT	C	INDEL	OS05G0116000	Intronic	NA	NA	LOC_Os05g02520	cupin domain containing protein, expressed
Chr.5	2722793	2722794	CA	CA	C	INDEL	OS05G0147400	Intronic	NA	NA	LOC_Os05g05470	T-complex protein, putative, expressed
Chr.5	3733563	3733564	CA	CA	C	INDEL	OS05G0163700	Intronic	NA	NA	LOC_Os05g07090	acyl-coenzyme A dehydrogenase, mitochondrial precursor, putative, expressed
Chr.5	7164866	7164868	CAT	CAT	C	INDEL	OS05G0215600	Intronic	NA	NA	LOC_Os05g12474	expressed protein
Chr.5	14993203	14993215	CGCGGCGGCGGCG	CGCGGCGGCGGCG	C	INDEL	OS05G0322900	Exonic	CDS	InFrame	LOC_Os05g25770	WRKY45, expressed
Chr.5	25644141	25644142	CA	CA	C	INDEL	OS05G0517200	Intronic	NA	NA	LOC_Os05g44100	trehalose synthase, putative, expressed
Chr.5	27602585	27602591	CCCGCCG	CCCGCCG	C	INDEL	OS05G0555100	Exonic	CDS	NA	LOC_Os05g48150	transposon protein, putative, unclassified, expressed
Chr.5	27959552	27959554	CAT	CAT	C	INDEL	OS05G0562000	Intronic	NA	NA	LOC_Os05g48790	expressed protein
Chr.5	28193712	28193713	CA	CA	C	INDEL	OS05G0566400	Intronic	NA	NA	LOC_Os05g49140	CGMC_MAPKCMGC_2.8 - CGMC includes CDA, MAPK, GSK3, and CLKC kinases, expressed
Chr.4	33532910	33532910	T	T	C	SNP	OS04G0657500	Intronic	NA	NA	LOC_Os04g56240	lipase, putative, expressed
Chr.4	33531984	33531984	T	T	C	SNP	OS04G0657500	Intronic	NA	NA	LOC_Os04g56240	lipase, putative, expressed
Chr.4	33256379	33256379	T	T	C	SNP	OS04G0652700	Exonic	3UTR	NA	LOC_Os04g55850	nuclease PA3, putative, expressed
Chr.4	31692383	31692383	T	T	C	SNP	OS04G0623500	Exonic	3UTR	NA	LOC_Os04g53210	hydroxyacid oxidase 1, putative, expressed
Chr.4	31441392	31441392	T	T	C	SNP	OS04G0619000	Intronic	NA	NA	LOC_Os04g52810	no apical meristem protein, putative, expressed
Chr.4	31441388	31441388	T	T	C	SNP	OS04G0619000	Intronic	NA	NA	LOC_Os04g52810	no apical meristem protein, putative, expressed
Chr.4	31441238	31441238	T	T	C	SNP	OS04G0619000	Intronic	NA	NA	LOC_Os04g52810	no apical meristem protein, putative, expressed
Chr.4	31440973	31440973	T	T	C	SNP	OS04G0619000	Intronic	NA	NA	LOC_Os04g52810	no apical meristem protein, putative, expressed
Chr.4	31439832	31439832	T	T	C	SNP	OS04G0619000	Exonic	3UTR	NA	LOC_Os04g52810	no apical meristem protein, putative, expressed
Chr.4	31439616	31439616	T	T	C	SNP	OS04G0619000	Exonic	3UTR	NA	LOC_Os04g52810	no apical meristem protein, putative, expressed
Chr.4	31241517	31241517	T	T	C	SNP	OS04G0615700	Intronic	NA	NA	LOC_Os04g52540	retrotransposon protein, putative, unclassified, expressed
Chr.4	31178256	31178256	T	T	C	SNP	OS04G0614600	Exonic	CDS	Missense	LOC_Os04g52450	aminotransferase, putative, expressed
Chr.4	29562809	29562809	T	T	C	SNP	OS04G0585100	Intronic	NA	NA	LOC_Os04g49560	zinc finger family protein, putative, expressed
Chr.4	29558939	29558939	T	T	C	SNP	OS04G0585100	Intronic	NA	NA	LOC_Os04g49560	zinc finger family protein, putative, expressed
Chr.4	29558835	29558835	T	T	C	SNP	OS04G0585100	Intronic	NA	NA	LOC_Os04g49560	zinc finger family protein, putative, expressed
Chr.4	29524079	29524079	T	T	C	SNP	OS04G0584500	Exonic	CDS	Silent	LOC_Os04g49500	U-box domain-containing protein, putative, expressed
Chr.4	29317604	29317604	T	T	C	SNP	OS04G0580800	Exonic	3UTR	NA	LOC_Os04g49160	zinc finger, C3HC4 type domain containing protein, expressed
Chr.4	29317483	29317483	T	T	C	SNP	OS04G0580800	Exonic	3UTR	NA	LOC_Os04g49160	zinc finger, C3HC4 type domain containing protein, expressed
Chr.4	29044745	29044745	T	T	C	SNP	OS04G0576500	Intronic	NA	NA	LOC_Os04g48710	expressed protein
Chr.4	28304545	28304545	T	T	C	SNP	OS04G0564700	Intronic	NA	NA	LOC_Os04g47700	expressed protein
Chr.4	27848633	27848633	T	T	C	SNP	OS04G0556500	Exonic	3UTR	NA	LOC_Os04g46980	cis-zeatin O-glucosyltransferase, putative, expressed
Chr.4	27642743	27642743	T	T	C	SNP	OS04G0551800	Exonic	CDS	Silent	LOC_Os04g46620	T-complex protein, putative, expressed
Chr.4	25195626	25195626	T	T	C	SNP	OS04G0504800	Intronic	NA	NA	LOC_Os04g42600	polyadenylate-binding protein, putative, expressed
Chr.4	24885386	24885386	T	T	C	SNP	OS04G0497600	Intronic	NA	NA	LOC_Os04g42010	RNA binding protein, putative, expressed
Chr.4	24883850	24883850	T	T	C	SNP	OS04G0497600	Intronic	NA	NA	LOC_Os04g42010	RNA binding protein, putative, expressed
Chr.4	24851445	24851445	T	T	C	SNP	OS04G0496800	Exonic	CDS	Silent	LOC_Os04g41950	calcium-binding mitochondrial protein anon-60Da, putative, expressed
Chr.4	23831298	23831298	T	T	C	SNP	OS04G0476100	Intronic	NA	NA	LOC_Os04g40040	copper methylamine oxidase precursor, putative, expressed
Chr.4	23409695	23409695	T	T	C	SNP	OS04G0468600	Exonic	CDS	Missense	LOC_Os04g39320	expressed protein
Chr.4	22626226	22626226	T	T	C	SNP	OS04G0453233	Intronic	NA	NA	LOC_Os04g38026	transporter family protein, putative, expressed
Chr.4	20426851	20426851	T	T	C	SNP	OS04G0413500	Exonic	3UTR	NA	LOC_Os04g33740	glycosyl hydrolases, putative, expressed
Chr.4	20389821	20389821	T	T	C	SNP	OS04G0412500	Exonic	3UTR	NA	LOC_Os04g33660	bifunctional monodehydroascorbate reductase and carbonic anhydrasenectarin-3 precursor, putative, expressed
Chr.4	16005051	16005051	T	T	C	SNP	OS04G0339400	Exonic	3UTR	NA	LOC_Os04g27060	oxidoreductase, aldo/keto reductase family protein, putative, expressed
Chr.4	16004177	16004177	T	T	C	SNP	OS04G0339400	Intronic	NA	NA	LOC_Os04g27060	oxidoreductase, aldo/keto reductase family protein, putative, expressed
Chr.4	16004167	16004167	T	T	C	SNP	OS04G0339400	Intronic	NA	NA	LOC_Os04g27060	oxidoreductase, aldo/keto reductase family protein, putative, expressed
Chr.4	16003962	16003962	T	T	C	SNP	OS04G0339400	Exonic	CDS	Missense	LOC_Os04g27060	oxidoreductase, aldo/keto reductase family protein, putative, expressed
Chr.4	11276789	11276789	T	T	C	SNP	OS04G0270100	Intronic	NA	NA	LOC_Os04g20220	elongation factor Tu, putative, expressed
Chr.4	7309814	7309814	T	T	C	SNP	OS04G0209200	Intronic	NA	NA	LOC_Os04g13210	multidrug resistance-associated protein, putative, expressed
Chr.4	6481137	6481137	T	T	C	SNP	OS04G0194500	Intronic	NA	NA	LOC_Os04g11820	white-brown complex homolog protein, putative, expressed
Chr.4	6479903	6479903	T	T	C	SNP	OS04G0194500	Intronic	NA	NA	LOC_Os04g11820	white-brown complex homolog protein, putative, expressed
Chr.4	6479046	6479046	T	T	C	SNP	OS04G0194500	Intronic	NA	NA	LOC_Os04g11820	white-brown complex homolog protein, putative, expressed
Chr.4	6479040	6479040	T	T	C	SNP	OS04G0194500	Intronic	NA	NA	LOC_Os04g11820	white-brown complex homolog protein, putative, expressed
Chr.4	6478937	6478937	T	T	C	SNP	OS04G0194500	Intronic	NA	NA	LOC_Os04g11820	white-brown complex homolog protein, putative, expressed
Chr.4	6478745	6478745	T	T	C	SNP	OS04G0194500	Intronic	NA	NA	LOC_Os04g11820	white-brown complex homolog protein, putative, expressed
Chr.4	6478334	6478334	T	T	C	SNP	OS04G0194500	Exonic	CDS	Silent	LOC_Os04g11820	white-brown complex homolog protein, putative, expressed
Chr.4	6232372	6232372	T	T	C	SNP	OS04G0191500	Exonic	CDS	NA	LOC_Os04g11400	expressed protein
Chr.4	1150290	1150290	T	T	C	SNP	OS04G0119500	Intronic	NA	NA	LOC_Os04g02910	membrane related protein CP5, putative, expressed
Chr.4	620896	620896	T	T	C	SNP	OS04G0110600	Intronic	NA	NA	LOC_Os04g02000	zinc finger family protein, putative, expressed
Chr.4	358817	358817	T	T	C	SNP	OS04G0105400	Intronic	NA	NA	LOC_Os04g01520	expressed protein
Chr.4	31441194	31441194	G	G	C	SNP	OS04G0619000	Intronic	NA	NA	LOC_Os04g52810	no apical meristem protein, putative, expressed
Chr.4	31181835	31181835	G	G	C	SNP	OS04G0614600	Intronic	NA	NA	LOC_Os04g52450	aminotransferase, putative, expressed
Chr.4	30077624	30077624	G	G	C	SNP	OS04G0596300	Exonic	3UTR	NA	LOC_Os04g50860	expressed protein
Chr.4	28303971	28303971	G	G	C	SNP	OS04G0564700	Intronic	NA	NA	LOC_Os04g47700	expressed protein
Chr.4	27642353	27642353	G	G	C	SNP	OS04G0551800	Intronic	NA	NA	LOC_Os04g46620	T-complex protein, putative, expressed
Chr.4	20423228	20423228	G	G	C	SNP	OS04G0413500	Intronic	NA	NA	LOC_Os04g33740	glycosyl hydrolases, putative, expressed
Chr.4	16005071	16005071	G	G	C	SNP	OS04G0339400	Exonic	3UTR	NA	LOC_Os04g27060	oxidoreductase, aldo/keto reductase family protein, putative, expressed
Chr.4	16004731	16004731	G	G	C	SNP	OS04G0339400	Exonic	CDS	Silent	LOC_Os04g27060	oxidoreductase, aldo/keto reductase family protein, putative, expressed
Chr.4	16004647	16004647	G	G	C	SNP	OS04G0339400	Intronic	NA	NA	LOC_Os04g27060	oxidoreductase, aldo/keto reductase family protein, putative, expressed

Chr.4	16003945	16003945	G	G	C	SNP	OS04G0339400	Exonic	CDS	Silent	LOC_Os04g27060	oxidoreductase, aldo/keto reductase family protein, putative, expressed
Chr.4	13495605	13495605	G	G	C	SNP	OS04G0301700	Intronic	NA	NA	LOC_Os04g23580	xylosyltransferase, putative, expressed
Chr.4	11273859	11273859	G	G	C	SNP	OS04G0270100	Intronic	NA	NA	LOC_Os04g20220	elongation factor Tu, putative, expressed
Chr.4	6478154	6478154	G	G	C	SNP	OS04G0194500	Exonic	CDS	Silent	LOC_Os04g11820	white-brown complex homolog protein, putative, expressed
Chr.4	335053	335053	G	G	C	SNP	OS04G0105100	Intronic	NA	NA	LOC_Os04g01490	zinc finger, C3HC4 type, domain containing protein, expressed
Chr.4	33418863	33418863	T	T	C	SNP	OS04G0655300	Exonic	5UTR	NA	LOC_Os04g56110	protein kinase, putative, expressed
Chr.4	33168747	33168747	G	G	C	SNP	OS04G0650800	Intronic	NA	NA	LOC_Os04g55720	D-3-phosphoglycerate dehydrogenase, chloroplast precursor, putative, expressed
Chr.4	33168182	33168182	A	A	C	SNP	OS04G0650800	Exonic	CDS	Missense	LOC_Os04g55720	D-3-phosphoglycerate dehydrogenase, chloroplast precursor, putative, expressed
Chr.4	33124698	33124698	A	A	C	SNP	OS04G0650000	Intronic	NA	NA	LOC_Os04g55650	oryzain alpha chain precursor, putative, expressed
Chr.4	33123800	33123800	A	A	C	SNP	OS04G0650000	Intronic	NA	NA	LOC_Os04g55650	oryzain alpha chain precursor, putative, expressed
Chr.4	31464461	31464461	T	T	C	SNP	OS04G0619400	Exonic	CDS	Silent	LOC_Os04g52840	tyrosine protein kinase domain containing protein, putative, expressed
Chr.4	31463411	31463411	T	T	C	SNP	OS04G0619400	Exonic	CDS	Silent	LOC_Os04g52840	tyrosine protein kinase domain containing protein, putative, expressed
Chr.4	31179593	31179593	A	A	C	SNP	OS04G0614600	Intronic	NA	NA	LOC_Os04g52450	aminotransferase, putative, expressed
Chr.4	31177697	31177697	A	A	C	SNP	OS04G0614600	Exonic	CDS	Missense	LOC_Os04g52450	aminotransferase, putative, expressed
Chr.4	31112555	31112555	T	T	C	SNP	OS04G0613700	Intronic	NA	NA	LOC_Os04g52370	UTP--glucose-1-phosphate uridylyltransferase, putative, expressed
Chr.4	29560098	29560098	T	T	C	SNP	OS04G0585100	Intronic	NA	NA	LOC_Os04g49560	zinc finger family protein, putative, expressed
Chr.4	29557507	29557507	T	T	C	SNP	OS04G0585100	Exonic	CDS	Silent	LOC_Os04g49560	zinc finger family protein, putative, expressed
Chr.4	29525290	29525290	T	T	C	SNP	OS04G0584500	Intronic	NA	NA	LOC_Os04g49500	U-box domain-containing protein, putative, expressed
Chr.4	29525256	29525256	T	T	C	SNP	OS04G0584500	Intronic	NA	NA	LOC_Os04g49500	U-box domain-containing protein, putative, expressed
Chr.4	29340031	29340031	T	T	C	SNP	OS04G0581100	Intronic	NA	NA	LOC_Os04g49210	naringenin,2-oxoglutarate 3-dioxygenase, putative, expressed
Chr.4	29317460	29317460	A	A	C	SNP	OS04G0580800	Exonic	3UTR	NA	LOC_Os04g49160	zinc finger, C3HC4 type domain containing protein, expressed
Chr.4	29134459	29134459	T	T	C	SNP	OS04G0578000	Exonic	CDS	Missense	LOC_Os04g48850	aminotransferase, classes I and II, domain containing protein, expressed
Chr.4	29045765	29045765	T	T	C	SNP	OS04G0576500	Intronic	NA	NA	LOC_Os04g48710	expressed protein
Chr.4	28342253	28342253	T	T	C	SNP	OS04G0565500	Exonic	CDS	Missense	LOC_Os04g47780	transmembrane amino acid transporter protein, putative, expressed
Chr.4	28303378	28303378	T	T	C	SNP	OS04G0564700	Intronic	NA	NA	LOC_Os04g47700	expressed protein
Chr.4	28302817	28302817	T	T	C	SNP	OS04G0564700	Exonic	CDS	Missense	LOC_Os04g47700	expressed protein
Chr.4	28300388	28300388	G	G	C	SNP	OS04G0564700	Exonic	CDS	Missense	LOC_Os04g47700	expressed protein
Chr.4	28298756	28298756	A	A	C	SNP	OS04G0564700	Exonic	CDS	Missense	LOC_Os04g47700	expressed protein
Chr.4	28298607	28298607	T	T	C	SNP	OS04G0564700	Intronic	NA	NA	LOC_Os04g47700	expressed protein
Chr.4	28084627	28084627	T	T	C	SNP	OS04G0560700	Exonic	5UTR	NA	LOC_Os04g47310	expressed protein
Chr.4	27849592	27849592	T	T	C	SNP	OS04G0556500	Exonic	CDS	Missense	LOC_Os04g46980	cis-zeatin O-glucosyltransferase, putative, expressed
Chr.4	27848787	27848787	A	A	C	SNP	OS04G0556500	Exonic	3UTR	NA	LOC_Os04g46980	cis-zeatin O-glucosyltransferase, putative, expressed
Chr.4	27848655	27848655	T	T	C	SNP	OS04G0556500	Exonic	3UTR	NA	LOC_Os04g46980	cis-zeatin O-glucosyltransferase, putative, expressed
Chr.4	27785421	27785421	T	T	C	SNP	OS04G0555300	Exonic	CDS	NA	LOC_Os04g46880	transporter, major facilitator family, putative, expressed
Chr.4	27785123	27785123	T	T	C	SNP	OS04G0555300	Exonic	CDS	NA	LOC_Os04g46880	transporter, major facilitator family, putative, expressed
Chr.4	27784875	27784875	T	T	C	SNP	OS04G0555300	Exonic	CDS	NA	LOC_Os04g46880	transporter, major facilitator family, putative, expressed
Chr.4	27640789	27640789	A	A	C	SNP	OS04G0551800	Intronic	NA	NA	LOC_Os04g46620	T-complex protein, putative, expressed
Chr.4	27639779	27639779	A	A	C	SNP	OS04G0551800	Intronic	NA	NA	LOC_Os04g46620	T-complex protein, putative, expressed
Chr.4	27236305	27236305	T	T	C	SNP	OS04G0543900	Exonic	3UTR	NA	LOC_Os04g45970	glutamate dehydrogenase protein, putative, expressed
Chr.4	27191730	27191730	T	T	C	SNP	OS04G0542900	Intronic	NA	NA	LOC_Os04g45910	placental protein 11 precursor, putative, expressed
Chr.4	25198479	25198479	T	T	C	SNP	OS04G0504800	Exonic	CDS	Missense	LOC_Os04g42600	polyadenylate-binding protein, putative, expressed
Chr.4	25195919	25195919	T	T	C	SNP	OS04G0504800	Exonic	CDS	Missense	LOC_Os04g42600	polyadenylate-binding protein, putative, expressed
Chr.4	25194730	25194730	G	G	C	SNP	OS04G0504800	Intronic	NA	NA	LOC_Os04g42600	polyadenylate-binding protein, putative, expressed
Chr.4	25194306	25194306	T	T	C	SNP	OS04G0504800	Exonic	3UTR	NA	LOC_Os04g42600	polyadenylate-binding protein, putative, expressed
Chr.4	25194132	25194132	T	T	C	SNP	OS04G0504800	Exonic	3UTR	NA	LOC_Os04g42600	polyadenylate-binding protein, putative, expressed
Chr.4	25194065	25194065	T	T	C	SNP	OS04G0504800	Exonic	3UTR	NA	LOC_Os04g42600	polyadenylate-binding protein, putative, expressed
Chr.4	25134242	25134242	T	T	C	SNP	OS04G0503500	Exonic	5UTR	NA	LOC_Os04g42470	regulatory subunit, putative, expressed
Chr.4	25133616	25133616	T	T	C	SNP	OS04G0503500	Exonic	5UTR	NA	LOC_Os04g42470	regulatory subunit, putative, expressed
Chr.4	25127341	25127341	G	G	C	SNP	OS04G0503500	Exonic	CDS	Missense	LOC_Os04g42470	regulatory subunit, putative, expressed
Chr.4	24886260	24886260	A	A	C	SNP	OS04G0497600	Exonic	CDS	Missense	LOC_Os04g42010	RNA binding protein, putative, expressed
Chr.4	24882802	24882802	T	T	C	SNP	OS04G0497600	Exonic	3UTR	NA	LOC_Os04g42010	RNA binding protein, putative, expressed
Chr.4	24858151	24858151	T	T	C	SNP	OS04G0497000	Exonic	3UTR	NA	LOC_Os04g41960	NADP-dependent oxidoreductase, putative, expressed
Chr.4	24853450	24853450	T	T	C	SNP	OS04G0496800	Intronic	NA	NA	LOC_Os04g41950	calcium-binding mitochondrial protein anon-60Da, putative, expressed
Chr.4	24853058	24853058	G	G	C	SNP	OS04G0496800	Intronic	NA	NA	LOC_Os04g41950	calcium-binding mitochondrial protein anon-60Da, putative, expressed
Chr.4	24851006	24851006	T	T	C	SNP	OS04G0496800	Intronic	NA	NA	LOC_Os04g41950	calcium-binding mitochondrial protein anon-60Da, putative, expressed
Chr.4	24849822	24849822	T	T	C	SNP	OS04G0496800	Exonic	CDS	Silent	LOC_Os04g41950	calcium-binding mitochondrial protein anon-60Da, putative, expressed
Chr.4	24848642	24848642	T	T	C	SNP	OS04G0496800	Exonic	CDS	Missense	LOC_Os04g41950	calcium-binding mitochondrial protein anon-60Da, putative, expressed
Chr.4	24529850	24529850	T	T	C	SNP	OS04G0490800	Exonic	CDS	Silent	LOC_Os04g41340	4-nitrophenylphosphatase, putative, expressed
Chr.4	24327434	24327434	T	T	C	SNP	OS04G0486950	Exonic	CDS	Missense	LOC_Os04g40990	malate synthase, glyoxysomal, putative, expressed
Chr.4	24325224	24325224	A	A	C	SNP	OS04G0486900	Exonic	CDS	Missense	LOC_Os04g40980	Divergent PAP2 family domain containing protein, expressed
Chr.4	24057090	24057090	T	T	C	SNP	OS04G0480900	Intronic	NA	NA	LOC_Os04g40490	glycosyl hydrolase family 5 protein, putative, expressed
Chr.4	24056774	24056774	T	T	C	SNP	OS04G0480900	Intronic	NA	NA	LOC_Os04g40490	glycosyl hydrolase family 5 protein, putative, expressed
Chr.4	24055620	24055620	T	T	C	SNP	OS04G0480900	Intronic	NA	NA	LOC_Os04g40490	glycosyl hydrolase family 5 protein, putative, expressed
Chr.4	23847228	23847228	A	A	C	SNP	OS04G0476400	Intronic	NA	NA	LOC_Os04g40050	ribonuclease H2 subunit B, putative, expressed
Chr.4	23847214	23847214	G	G	C	SNP	OS04G0476400	Intronic	NA	NA	LOC_Os04g40050	ribonuclease H2 subunit B, putative, expressed
Chr.4	23846293	23846293	T	T	C	SNP	OS04G0476400	Exonic	3UTR	NA	LOC_Os04g40050	ribonuclease H2 subunit B, putative, expressed
Chr.4	23409662	23409662	A	A	C	SNP	OS04G0468600	Exonic	CDS	Missense	LOC_Os04g39320	expressed protein
Chr.4	23141609	23141609	A	A	C	SNP	OS04G0463500	Intronic	NA	NA	LOC_Os04g38950	class I glutamine amidotransferase, putative, expressed
Chr.4	22261258	22261258	T	T	C	SNP	OS04G0446500	Intronic	NA	NA	LOC_Os04g36890	peptidyl-prolyl cis-trans isomerase, FKBP-type, putative, expressed
Chr.4	22259279	22259279	T	T	C	SNP	OS04G0446500	Exonic	CDS	Silent	LOC_Os04g36890	peptidyl-prolyl cis-trans isomerase, FKBP-type, putative, expressed
Chr.4	22258941	22258941	G	G	C	SNP	OS04G0446500	Exonic	CDS	Silent	LOC_Os04g36890	peptidyl-prolyl cis-trans isomerase, FKBP-type, putative, expressed
Chr.4	22258296	22258296	T	T	C	SNP	OS04G0446500	Exonic	5UTR	NA	LOC_Os04g36890	peptidyl-prolyl cis-trans isomerase, FKBP-type, putative, expressed
Chr.4	22207468	22207468	A	A	C	SNP	OS04G0445400	Intronic	NA	NA	LOC_Os04g36780	expressed protein
Chr.4	22186820	22186820	A	A	C	SNP	OS04G0444800	Exonic	3UTR	NA	LOC_Os04g36720	ferric-chelate reductase, putative, expressed
Chr.4	22184982	22184982	G	G	C	SNP	OS04G0444800	Exonic	CDS	Missense	LOC_Os04g36720	ferric-chelate reductase, putative, expressed
Chr.4	22184066	22184066	T	T	C	SNP	OS04G0444800	Intronic	NA	NA	LOC_Os04g36720	ferric-chelate reductase, putative, expressed
Chr.4	22183900	22183900	T	T	C	SNP	OS04G0444800	Intronic	NA	NA	LOC_Os04g36720	ferric-chelate reductase, putative, expressed
Chr.4	5326243	5326243	T	T	C	SNP	OS04G0178300	Exonic	3UTR	NA	LOC_Os04g09900	ent-kaurene synthase, chloroplast precursor, putative, expressed
Chr.4	5325403	5325403	T	T	C	SNP	OS04G0178300	Exonic	CDS	Missense	LOC_Os04g09900	ent-kaurene synthase, chloroplast precursor, putative, expressed
Chr.4	5325237	5325237	G	G	C	SNP	OS04G0178300	Exonic	CDS	Missense	LOC_Os04g09900	ent-kaurene synthase, chloroplast precursor, putative, expressed
Chr.4	5325235	5325235	T	T	C	SNP	OS04G0178300	Exonic	CDS	Silent	LOC_Os04g09900	ent-kaurene synthase, chloroplast precursor, putative, expressed
Chr.4	5325000	5325000	A	A	C	SNP	OS04G0178300	Intronic	NA	NA	LOC_Os04g09900	ent-kaurene synthase, chloroplast precursor, putative, expressed
Chr.4	5324611	5324611	T	T	C	SNP	OS04G0178300	Intronic	NA	NA	LOC_Os04g09900	ent-kaurene synthase, chloroplast precursor, putative, expressed
Chr.4	5324419	5324419	T	T	C	SNP	OS04G0178300	Intronic	NA	NA	LOC_Os04g09900	ent-kaurene synthase, chloroplast precursor, putative, expressed
Chr.4	5324377	5324377	A	A	C	SNP	OS04G0178300	Intronic	NA	NA	LOC_Os04g09900	ent-kaurene synthase, chloroplast precursor, putative, expressed

Chr.4	5324123	5324123	G	G	C	SNP	OS04G0178300	Intronic	NA	NA	LOC_Os04g09900	ent-kaurene synthase, chloroplast precursor, putative, expressed
Chr.4	5323048	5323048	T	T	C	SNP	OS04G0178300	Exonic	CDS	Silent	LOC_Os04g09900	ent-kaurene synthase, chloroplast precursor, putative, expressed
Chr.4	5322991	5322991	G	G	C	SNP	OS04G0178300	Intronic	NA	NA	LOC_Os04g09900	ent-kaurene synthase, chloroplast precursor, putative, expressed
Chr.4	5322182	5322182	A	A	C	SNP	OS04G0178300	Exonic	CDS	Silent	LOC_Os04g09900	ent-kaurene synthase, chloroplast precursor, putative, expressed
Chr.4	5321546	5321546	A	A	C	SNP	OS04G0178300	Intronic	NA	NA	LOC_Os04g09900	ent-kaurene synthase, chloroplast precursor, putative, expressed
Chr.4	483853	483853	A	A	C	SNP	OS04G0107900	Intronic	NA	NA	LOC_Os04g01740	heat shock protein, putative, expressed
Chr.4	24056248	24056249	CT	CT	C	INDEL	OS04G0480900	Intronic	NA	NA	LOC_Os04g40490	glycosyl hydrolase family 5 protein, putative, expressed
Chr.4	31109214	31109215	CT	CT	C	INDEL	OS04G0613700	Exonic	3UTR	NA	LOC_Os04g52370	UTP--glucose-1-phosphate uridylyltransferase, putative, expressed
Chr.4	24056761	24056764	CCTT	CCTT	C	INDEL	OS04G0480900	Intronic	NA	NA	LOC_Os04g40490	glycosyl hydrolase family 5 protein, putative, expressed
Chr.4	27764732	27764734	CCA	CCA	C	INDEL	OS04G0555000	Exonic	5UTR	NA	LOC_Os04g46860	scarecrow, putative, expressed
Chr.4	358681	358690	CACCGACAAA	CACCGACAAA	C	INDEL	OS04G0105400	Intronic	NA	NA	LOC_Os04g01520	expressed protein
Chr.4	27236577	27236579	CAA	CAA	C	INDEL	OS04G0543900	Exonic	3UTR	NA	LOC_Os04g45970	glutamate dehydrogenase protein, putative, expressed
Chr.4	33531657	33531657	A	A	C	SNP	OS04G0657500	Exonic	CDS	Missense	LOC_Os04g56240	lipase, putative, expressed
Chr.4	31441441	31441441	A	A	C	SNP	OS04G0619000	Intronic	NA	NA	LOC_Os04g52810	no apical meristem protein, putative, expressed
Chr.4	31441434	31441434	A	A	C	SNP	OS04G0619000	Intronic	NA	NA	LOC_Os04g52810	no apical meristem protein, putative, expressed
Chr.4	27194815	27194815	A	A	C	SNP	OS04G0542900	Exonic	3UTR	NA	LOC_Os04g45910	placental protein 11 precursor, putative, expressed
Chr.4	24324151	24324151	A	A	C	SNP	OS04G0486900	Intronic	NA	NA	LOC_Os04g40980	Divergent PAP2 family domain containing protein, expressed
Chr.4	23141513	23141513	A	A	C	SNP	OS04G0463500	Intronic	NA	NA	LOC_Os04g38950	class I glutamine amidotransferase, putative, expressed
Chr.4	22208479	22208479	A	A	C	SNP	OS04G0445400	Intronic	NA	NA	LOC_Os04g36780	expressed protein
Chr.4	20424778	20424778	A	A	C	SNP	OS04G0413500	Intronic	NA	NA	LOC_Os04g33740	glycosyl hydrolases, putative, expressed
Chr.4	13644942	13644942	A	A	C	SNP	OS04G0304200	Exonic	CDS	NA	LOC_Os04g23890	AGC_PVPK_like_kin82y.10 - ACG kinases include homologs to PKA, PKG and PKC, expressed
Chr.4	6481155	6481155	A	A	C	SNP	OS04G0194500	Intronic	NA	NA	LOC_Os04g11820	white-brown complex homolog protein, putative, expressed
Chr.4	6480739	6480739	A	A	C	SNP	OS04G0194500	Intronic	NA	NA	LOC_Os04g11820	white-brown complex homolog protein, putative, expressed
Chr.4	485809	485809	G	G	C	SNP	OS04G0107900	Exonic	3UTR	NA	LOC_Os04g01740	heat shock protein, putative, expressed
Chr.4	619418	619418	T	T	C	SNP	OS04G0110600	Intronic	NA	NA	LOC_Os04g02000	zinc finger family protein, putative, expressed
Chr.4	5326227	5326227	T	T	C	SNP	OS04G0178300	Exonic	3UTR	NA	LOC_Os04g09900	ent-kaurene synthase, chloroplast precursor, putative, expressed
Chr.4	5324383	5324383	G	G	C	SNP	OS04G0178300	Intronic	NA	NA	LOC_Os04g09900	ent-kaurene synthase, chloroplast precursor, putative, expressed
Chr.4	5325540	5325540	T	T	C	SNP	OS04G0178300	Intronic	NA	NA	LOC_Os04g09900	ent-kaurene synthase, chloroplast precursor, putative, expressed
Chr.4	5325556	5325556	T	T	C	SNP	OS04G0178300	Intronic	NA	NA	LOC_Os04g09900	ent-kaurene synthase, chloroplast precursor, putative, expressed
Chr.4	5325567	5325567	T	T	C	SNP	OS04G0178300	Intronic	NA	NA	LOC_Os04g09900	ent-kaurene synthase, chloroplast precursor, putative, expressed
Chr.4	5386186	5386186	G	G	C	SNP	OS04G0179200	Exonic	CDS	Silent	LOC_Os04g10010	sex determination protein tasselseed-2, putative, expressed
Chr.4	6479755	6479755	T	T	C	SNP	OS04G0194500	Exonic	CDS	Missense	LOC_Os04g11820	white-brown complex homolog protein, putative, expressed
Chr.4	6481260	6481260	T	T	C	SNP	OS04G0194500	Exonic	CDS	Missense	LOC_Os04g11820	white-brown complex homolog protein, putative, expressed
Chr.4	8949576	8949576	G	G	C	SNP	OS04G0233400	Exonic	CDS	Silent	LOC_Os04g16450	aquaporin protein, putative, expressed
Chr.4	13641904	13641904	T	T	C	SNP	OS04G0304200	Intronic	NA	NA	LOC_Os04g23890	AGC_PVPK_like_kin82y.10 - ACG kinases include homologs to PKA, PKG and PKC, expressed
Chr.4	13651014	13651014	T	T	C	SNP	OS04G0304200	Intronic	NA	NA	LOC_Os04g23890	AGC_PVPK_like_kin82y.10 - ACG kinases include homologs to PKA, PKG and PKC, expressed
Chr.4	16004848	16004848	A	A	C	SNP	OS04G0339400	Exonic	CDS	Silent	LOC_Os04g27060	oxidoreductase, aldo/keto reductase family protein, putative, expressed
Chr.4	16004158	16004158	T	T	C	SNP	OS04G0339400	Intronic	NA	NA	LOC_Os04g27060	oxidoreductase, aldo/keto reductase family protein, putative, expressed
Chr.4	20359176	20359176	G	G	C	SNP	OS04G0412100	Exonic	CDS	Silent	LOC_Os04g33610	expressed protein
Chr.4	20389788	20389788	T	T	C	SNP	OS04G0412500	Exonic	3UTR	NA	LOC_Os04g33660	bifunctional monodehydroascorbate reductase and carbonic anhydrasenectarin-3 precursor, putative, expressed
Chr.4	20424833	20424833	T	T	C	SNP	OS04G0413500	Intronic	NA	NA	LOC_Os04g33740	glycosyl hydrolases, putative, expressed
Chr.4	22022225	22022225	T	T	C	SNP	OS04G0442200	Intronic	NA	NA	LOC_Os04g36062	expressed protein
Chr.4	22182860	22182860	G	G	C	SNP	OS04G0444800	Exonic	CDS	Missense	LOC_Os04g36720	ferric-chelate reductase, putative, expressed
Chr.4	23843822	23843822	T	T	C	SNP	OS04G0476400	Intronic	NA	NA	LOC_Os04g40050	ribonuclease H2 subunit B, putative, expressed
Chr.4	24328068	24328068	A	A	C	SNP	OS04G0486950	Exonic	CDS	Missense	LOC_Os04g40990	malate synthase, glyoxysomal, putative, expressed
Chr.4	24530202	24530202	T	T	C	SNP	OS04G0490800	Exonic	CDS	Missense	LOC_Os04g41340	4-nitrophenylphosphatase, putative, expressed
Chr.4	24858641	24858641	T	T	C	SNP	OS04G0497000	Exonic	CDS	Silent	LOC_Os04g41960	NADP-dependent oxidoreductase, putative, expressed
Chr.4	25194357	25194357	T	T	C	SNP	OS04G0504800	Exonic	3UTR	NA	LOC_Os04g42600	polyadenylate-binding protein, putative, expressed
Chr.4	26559173	26559173	T	T	C	SNP	OS04G0531100	Intronic	NA	NA	LOC_Os04g44870	C2 domain containing protein, putative, expressed
Chr.4	27784102	27784102	A	A	C	SNP	OS04G0555300	Exonic	CDS	NA	LOC_Os04g46880	transporter, major facilitator family, putative, expressed
Chr.4	29045924	29045924	T	T	C	SNP	OS04G0576500	Exonic	CDS	Silent	LOC_Os04g48710	expressed protein
Chr.4	29046484	29046484	A	A	C	SNP	OS04G0576500	Exonic	CDS	Missense	LOC_Os04g48710	expressed protein
Chr.4	29340208	29340208	T	T	C	SNP	OS04G0581100	Exonic	CDS	Silent	LOC_Os04g49210	naringenin,2-oxoglutarate 3-dioxygenase, putative, expressed
Chr.4	29525690	29525690	T	T	C	SNP	OS04G0584500	Intronic	NA	NA	LOC_Os04g49500	U-box domain-containing protein, putative, expressed
Chr.4	30174083	30174083	T	T	C	SNP	OS04G0598000	Exonic	5UTR	NA	LOC_Os04g50970	seed specific protein Bn15D1B, putative, expressed
Chr.4	31062135	31062135	T	T	C	SNP	OS04G0612500	Exonic	CDS	Silent	LOC_Os04g52260	LTPL124 - Protease inhibitor/seed storage/LTP family protein precursor, expressed
Chr.4	31176824	31176824	T	T	C	SNP	OS04G0614600	Exonic	5UTR	NA	LOC_Os04g52450	aminotransferase, putative, expressed
Chr.4	31176843	31176843	G	G	C	SNP	OS04G0614600	Exonic	CDS	Silent	LOC_Os04g52450	aminotransferase, putative, expressed
Chr.4	31220521	31220521	A	A	C	SNP	OS04G0615200	Intronic	NA	NA	LOC_Os04g52504	adhesive/proline-rich protein, putative, expressed
Chr.4	31239372	31239372	G	G	C	SNP	OS04G0615700	Exonic	CDS	Missense	LOC_Os04g52540	retrotransposon protein, putative, unclassified, expressed
Chr.4	31440060	31440060	T	T	C	SNP	OS04G0619000	Exonic	3UTR	NA	LOC_Os04g52810	no apical meristem protein, putative, expressed
Chr.4	31442267	31442267	G	G	C	SNP	OS04G0619000	Exonic	3UTR	NA	LOC_Os04g52810	no apical meristem protein, putative, expressed
Chr.4	31443217	31443217	G	G	C	SNP	OS04G0619000	Exonic	CDS	Silent	LOC_Os04g52810	no apical meristem protein, putative, expressed
Chr.4	33163974	33163974	T	T	C	SNP	OS04G0650700	Exonic	CDS	Silent	LOC_Os04g55710	transposon protein, putative, unclassified, expressed
Chr.4	33164226	33164226	G	G	C	SNP	OS04G0650700	Exonic	CDS	Silent	LOC_Os04g55710	transposon protein, putative, unclassified, expressed
Chr.4	33255043	33255043	T	T	C	SNP	OS04G0652700	Intronic	NA	NA	LOC_Os04g55850	nuclease PA3, putative, expressed
Chr.4	33531373	33531373	T	T	C	SNP	OS04G0657500	Exonic	CDS	Silent	LOC_Os04g56240	lipase, putative, expressed
Chr.4	358760	358762	CTA	CTA	C	INDEL	OS04G0105400	Intronic	NA	NA	LOC_Os04g01520	expressed protein
Chr.4	5321556	5321557	CT	CT	C	INDEL	OS04G0178300	Intronic	NA	NA	LOC_Os04g09900	ent-kaurene synthase, chloroplast precursor, putative, expressed
Chr.4	5325032	5325033	CT	CT	C	INDEL	OS04G0178300	Intronic	NA	NA	LOC_Os04g09900	ent-kaurene synthase, chloroplast precursor, putative, expressed
Chr.4	6477588	6477592	CGTAA	CGTAA	C	INDEL	OS04G0194500	Intronic	NA	NA	LOC_Os04g11820	white-brown complex homolog protein, putative, expressed
Chr.4	6479412	6479413	CA	CA	C	INDEL	OS04G0194500	Intronic	NA	NA	LOC_Os04g11820	white-brown complex homolog protein, putative, expressed
Chr.4	11282021	11282023	CAA	CAA	C	INDEL	OS04G0270100	Intronic	NA	NA	LOC_Os04g20220	elongation factor Tu, putative, expressed
Chr.4	18603965	18603968	CAAG	CAAG	C	INDEL	OS04G0380300	Exonic	CDS	InFrame	LOC_Os04g31120	OsFBK14 - F-box domain and kelch repeat containing protein, expressed
Chr.4	20389841	20389843	CGT	CGT	C	INDEL	OS04G0412500	Exonic	3UTR	NA	LOC_Os04g33660	bifunctional monodehydroascorbate reductase and carbonic anhydrasenectarin-3 precursor, putative, expressed
Chr.4	22183108	22183109	CT	CT	C	INDEL	OS04G0444800	Intronic	NA	NA	LOC_Os04g36720	ferric-chelate reductase, putative, expressed
Chr.4	22258515	22258518	CGCT	CGCT	C	INDEL	OS04G0446500	Intronic	NA	NA	LOC_Os04g36890	peptidyl-prolyl cis-trans isomerase, FKBP-type, putative, expressed
Chr.4	23831640	23831641	CA	CA	C	INDEL	OS04G0476100	Intronic	NA	NA	LOC_Os04g40040	copper methylamine oxidase precursor, putative, expressed
Chr.4	24853901	24853905	CAGGA	CAGGA	C	INDEL	OS04G0496800	Exonic	5UTR	NA	LOC_Os04g41950	calcium-binding mitochondrial protein anon-60Da, putative, expressed
Chr.4	24853147	24853148	CT	CT	C	INDEL	OS04G0496800	Intronic	NA	NA	LOC_Os04g41950	calcium-binding mitochondrial protein anon-60Da, putative, expressed
Chr.4	27233842	27233843	CT	CT	C	INDEL	OS04G0543900	Exonic	5UTR	NA	LOC_Os04g45970	glutamate dehydrogenase protein, putative, expressed
Chr.4	31178127	31178128	CT	CT	C	INDEL	OS04G0614600	Intronic	NA	NA	LOC_Os04g52450	aminotransferase, putative, expressed
Chr.4	31440130	31440133	CGGT	CGGT	C	INDEL	OS04G0619000	Exonic	3UTR	NA	LOC_Os04g52810	no apical meristem protein, putative, expressed
Chr.4	31673596	31673597	CA	CA	C	INDEL	OS04G0623300	Intronic	NA	NA	LOC_Os04g53190	CPuORF12 - conserved peptide uORF-containing transcript, expressed
Chr.4	33418728	33418729	CA	CA	C	INDEL	OS04G0655300	Exonic	5UTR	NA	LOC_Os04g56110	protein kinase, putative, expressed

Chr.4	33418825	33418827	CTT	CTT	C	INDEL	OS04G0655300	Exonic	5UTR	NA	LOC_Os04g56110	protein kinase, putative, expressed
Chr.4	35032586	35032596	CATCGAATCGA	CATCGAATCGA	C	INDEL	OS04G0685700	Exonic	5UTR	NA	LOC_Os04g58890	expressed protein
Chr.3	1236478	1236478	T	T	C	SNP	OS03G0122300	Intronic	NA	NA	LOC_Os03g03034	flavonol synthase/flavanone 3-hydroxylase, putative, expressed
Chr.3	1236558	1236558	T	T	C	SNP	OS03G0122300	Intronic	NA	NA	LOC_Os03g03034	flavonol synthase/flavanone 3-hydroxylase, putative, expressed
Chr.3	1237693	1237693	T	T	C	SNP	OS03G0122300	Intronic	NA	NA	LOC_Os03g03034	flavonol synthase/flavanone 3-hydroxylase, putative, expressed
Chr.3	1238546	1238546	T	T	C	SNP	OS03G0122300	Intronic	NA	NA	LOC_Os03g03034	flavonol synthase/flavanone 3-hydroxylase, putative, expressed
Chr.3	1242891	1242891	T	T	C	SNP	OS03G0122300	Intronic	NA	NA	LOC_Os03g03034	flavonol synthase/flavanone 3-hydroxylase, putative, expressed
Chr.3	1243057	1243057	T	T	C	SNP	OS03G0122300	Intronic	NA	NA	LOC_Os03g03034	flavonol synthase/flavanone 3-hydroxylase, putative, expressed
Chr.3	1243364	1243364	T	T	C	SNP	OS03G0122300	Intronic	NA	NA	LOC_Os03g03034	flavonol synthase/flavanone 3-hydroxylase, putative, expressed
Chr.3	1243884	1243884	T	T	C	SNP	OS03G0122300	Intronic	NA	NA	LOC_Os03g03034	flavonol synthase/flavanone 3-hydroxylase, putative, expressed
Chr.3	1243914	1243914	T	T	C	SNP	OS03G0122300	Intronic	NA	NA	LOC_Os03g03034	flavonol synthase/flavanone 3-hydroxylase, putative, expressed
Chr.3	3168150	3168150	T	T	C	SNP	OS03G0159100	Intronic	NA	NA	LOC_Os03g06330	tyrosine protein kinase domain containing protein, putative, expressed
Chr.3	7129584	7129584	T	T	C	SNP	OS03G0235000	Exonic	CDS	NA	LOC_Os03g13210	peroxidase precursor, putative, expressed
Chr.3	8224120	8224120	T	T	C	SNP	OS03G0255500	Exonic	5UTR	NA	LOC_Os03g15050	phosphoenolpyruvate carboxykinase, putative, expressed
Chr.3	9014440	9014440	T	T	C	SNP	OS03G0269900	Intronic	NA	NA	LOC_Os03g16334	fringe-related protein, putative, expressed
Chr.3	9202586	9202586	T	T	C	SNP	OS03G0273800	Exonic	5UTR	NA	LOC_Os03g16670	haloacid dehalogenase-like hydrolase family protein, putative, expressed
Chr.3	9846586	9846586	T	T	C	SNP	OS03G0285700	Exonic	3UTR	NA	LOC_Os03g17690	OsAPx1 - Cytosolic Ascorbate Peroxidase encoding gene 1-8, expressed
Chr.3	15472290	15472290	T	T	C	SNP	OS03G0387900	Intronic	NA	NA	LOC_Os03g27019	expressed protein
Chr.3	16283978	16283978	T	T	C	SNP	OS03G0401100	Exonic	CDS	Missense	LOC_Os03g28300	protein kinase domain containing protein, expressed
Chr.3	16284553	16284553	T	T	C	SNP	OS03G0401100	Exonic	CDS	Missense	LOC_Os03g28300	protein kinase domain containing protein, expressed
Chr.3	16285237	16285237	T	T	C	SNP	OS03G0401100	Intronic	NA	NA	LOC_Os03g28300	protein kinase domain containing protein, expressed
Chr.3	16301454	16301454	T	T	C	SNP	OS03G0401300	Exonic	3UTR	NA	LOC_Os03g28330	sucrose synthase, putative, expressed
Chr.3	16592898	16592898	T	T	C	SNP	OS03G0405500	Intronic	NA	NA	LOC_Os03g29190	PDI, putative, expressed
Chr.3	22616469	22616469	T	T	C	SNP	OS03G0603600	Intronic	NA	NA	LOC_Os03g40670	glycerophosphoryl diester phosphodiesterase family protein, putative, expressed
Chr.3	23369810	23369810	T	T	C	SNP	OS03G0616700	Intronic	NA	NA	LOC_Os03g42050	exportin-7-A, putative, expressed
Chr.3	23375333	23375333	T	T	C	SNP	OS03G0616700	Intronic	NA	NA	LOC_Os03g42050	exportin-7-A, putative, expressed
Chr.3	23488066	23488066	T	T	C	SNP	OS03G0619400	Intronic	NA	NA	LOC_Os03g42220	T-complex protein, putative, expressed
Chr.3	24419453	24419453	T	T	C	SNP	OS03G0637800	Exonic	CDS	Silent	LOC_Os03g43670	TKL_IRAK_CR4L.1 - The CR4L subfamily has homology with Crinkly4, expressed
Chr.3	24470320	24470320	T	T	C	SNP	OS03G0638400	Intronic	NA	NA	LOC_Os03g43750	retrotransposon, putative, centromere-specific, expressed
Chr.3	24705032	24705032	T	T	C	SNP	OS03G0641300	Exonic	3UTR	NA	LOC_Os03g43990	BTBN8 - Bric-a-Brac, Tramtrack, Broad Complex BTB domain with non-phototropic hypocotyl 3 NPH3 and coiled-coil domains, ex
Chr.3	24705470	24705470	T	T	C	SNP	OS03G0641300	Exonic	3UTR	NA	LOC_Os03g43990	BTBN8 - Bric-a-Brac, Tramtrack, Broad Complex BTB domain with non-phototropic hypocotyl 3 NPH3 and coiled-coil domains, ex
Chr.3	24810247	24810247	T	T	C	SNP	OS03G0643300	Exonic	3UTR	NA	LOC_Os03g44150	aminotransferase, putative, expressed
Chr.3	24810314	24810314	T	T	C	SNP	OS03G0643300	Exonic	3UTR	NA	LOC_Os03g44150	aminotransferase, putative, expressed
Chr.3	24814362	24814362	T	T	C	SNP	OS03G0643300	Exonic	CDS	Silent	LOC_Os03g44150	aminotransferase, putative, expressed
Chr.3	24814640	24814640	T	T	C	SNP	OS03G0643300	Exonic	CDS	Silent	LOC_Os03g44150	aminotransferase, putative, expressed
Chr.3	24816634	24816634	T	T	C	SNP	OS03G0643300	Exonic	CDS	Silent	LOC_Os03g44150	aminotransferase, putative, expressed
Chr.3	24815481	24815481	T	T	C	SNP	OS03G0643300	Intronic	NA	NA	LOC_Os03g44150	aminotransferase, putative, expressed
Chr.3	24815550	24815550	T	T	C	SNP	OS03G0643300	Intronic	NA	NA	LOC_Os03g44150	aminotransferase, putative, expressed
Chr.3	24816279	24816279	T	T	C	SNP	OS03G0643300	Intronic	NA	NA	LOC_Os03g44150	aminotransferase, putative, expressed
Chr.3	24816287	24816287	T	T	C	SNP	OS03G0643300	Intronic	NA	NA	LOC_Os03g44150	aminotransferase, putative, expressed
Chr.3	24816313	24816313	T	T	C	SNP	OS03G0643300	Intronic	NA	NA	LOC_Os03g44150	aminotransferase, putative, expressed
Chr.3	24816398	24816398	T	T	C	SNP	OS03G0643300	Intronic	NA	NA	LOC_Os03g44150	aminotransferase, putative, expressed
Chr.3	26396797	26396797	T	T	C	SNP	OS03G0669100	Exonic	CDS	Silent	LOC_Os03g46640	deoxyuridine 5-triphosphate nucleotidohydrolase, putative, expressed
Chr.3	27491274	27491274	T	T	C	SNP	OS03G0689100	Exonic	CDS	Silent	LOC_Os03g48300	histidine acid phosphatase, putative, expressed
Chr.3	27493539	27493539	T	T	C	SNP	OS03G0689100	Intronic	NA	NA	LOC_Os03g48300	histidine acid phosphatase, putative, expressed
Chr.3	27493986	27493986	T	T	C	SNP	OS03G0689100	Intronic	NA	NA	LOC_Os03g48300	histidine acid phosphatase, putative, expressed
Chr.3	27497967	27497967	T	T	C	SNP	OS03G0689100	Intronic	NA	NA	LOC_Os03g48300	histidine acid phosphatase, putative, expressed
Chr.3	29144226	29144226	T	T	C	SNP	OS03G0719300	Intronic	NA	NA	LOC_Os03g51000	3,4-dihydroxy-2-butanone kinase, putative, expressed
Chr.3	29563733	29563733	T	T	C	SNP	OS03G0726500	Intronic	NA	NA	LOC_Os03g51650	membrane protein, putative, expressed
Chr.3	30078726	30078726	T	T	C	SNP	OS03G0734300	Exonic	3UTR	NA	LOC_Os03g52390	PIII1 - Proteinase inhibitor II family protein precursor, expressed
Chr.3	30225950	30225950	T	T	C	SNP	OS03G0737300	Exonic	CDS	Missense	LOC_Os03g52720	magnesium-dependent phosphatase 1, putative, expressed
Chr.3	30225721	30225721	T	T	C	SNP	OS03G0737300	Intronic	NA	NA	LOC_Os03g52720	magnesium-dependent phosphatase 1, putative, expressed
Chr.3	30534079	30534079	T	T	C	SNP	OS03G0743900	Intronic	NA	NA	LOC_Os03g53230	bifunctional 3-phosphoadenosine 5-phosphosulfate synthetase, putative, expressed
Chr.3	32934166	32934166	T	T	C	SNP	OS03G0791800	Intronic	NA	NA	LOC_Os03g57790	ubiquitin-conjugating enzyme, putative, expressed
Chr.3	33215663	33215663	T	T	C	SNP	OS03G0797400	Intronic	NA	NA	LOC_Os03g58300	indole-3-glycerol phosphate lyase, chloroplast precursor, putative, expressed
Chr.3	33348176	33348176	T	T	C	SNP	OS03G0799900	Exonic	CDS	Silent	LOC_Os03g58570	WD domain, G-beta repeat domain containing protein, expressed
Chr.3	33548039	33548039	T	T	C	SNP	OS03G0803900	Exonic	3UTR	NA	LOC_Os03g58920	galactosyltransferase family protein, putative, expressed
Chr.3	34911269	34911269	T	T	C	SNP	OS03G0831100	Intronic	NA	NA	LOC_Os03g61560	expressed protein
Chr.3	35661699	35661699	T	T	C	SNP	OS03G0848100	Exonic	3UTR	NA	LOC_Os03g63074	Ser/Thr protein phosphatase family protein, expressed
Chr.3	35869435	35869435	T	T	C	SNP	OS03G0851900	Intronic	NA	NA	LOC_Os03g63500	ATPase, AFG1 family domain containing protein, expressed
Chr.3	1876392	1876392	G	G	C	SNP	OS03G0133300	Intronic	NA	NA	LOC_Os03g04100	expressed protein
Chr.3	3525682	3525682	G	G	C	SNP	OS03G0165400	Exonic	CDS	Missense	LOC_Os03g06940	beta-galactosidase, putative, expressed
Chr.3	4546698	4546698	G	G	C	SNP	OS03G0187000	Intronic	NA	NA	LOC_Os03g08810	expressed protein
Chr.3	8789741	8789741	G	G	C	SNP	OS03G0265900	Exonic	CDS	Silent	LOC_Os03g15920	expressed protein
Chr.3	9016458	9016458	G	G	C	SNP	OS03G0269900	Exonic	3UTR	NA	LOC_Os03g16334	fringe-related protein, putative, expressed
Chr.3	10995386	10995386	G	G	C	SNP	OS03G0308800	Intronic	NA	NA	LOC_Os03g19560	tetratricopeptide repeat domain containing protein, expressed
Chr.3	11009345	11009345	G	G	C	SNP	OS03G0309000	Intronic	NA	NA	LOC_Os03g19580	expressed protein
Chr.3	12696483	12696483	G	G	C	SNP	OS03G0340900	Intronic	NA	NA	LOC_Os03g22160	relA-SpoT like protein RSH1, putative, expressed
Chr.3	15471101	15471101	G	G	C	SNP	OS03G0387900	Intronic	NA	NA	LOC_Os03g27019	expressed protein
Chr.3	16289690	16289690	G	G	C	SNP	OS03G0401100	Intronic	NA	NA	LOC_Os03g28300	protein kinase domain containing protein, expressed
Chr.3	21087058	21087058	G	G	C	SNP	OS03G0576700	Exonic	CDS	Silent	LOC_Os03g37970	ribosomal protein L13, putative, expressed
Chr.3	24705368	24705368	G	G	C	SNP	OS03G0641300	Exonic	3UTR	NA	LOC_Os03g43990	BTBN8 - Bric-a-Brac, Tramtrack, Broad Complex BTB domain with non-phototropic hypocotyl 3 NPH3 and coiled-coil domains, ex
Chr.3	24814871	24814871	G	G	C	SNP	OS03G0643300	Intronic	NA	NA	LOC_Os03g44150	aminotransferase, putative, expressed
Chr.3	24816841	24816841	G	G	C	SNP	OS03G0643300	Intronic	NA	NA	LOC_Os03g44150	aminotransferase, putative, expressed
Chr.3	28594691	28594691	G	G	C	SNP	OS03G0709000	Exonic	3UTR	NA	LOC_Os03g50130	microsomal glutathione S-transferase 3, putative, expressed
Chr.3	29386968	29386968	G	G	C	SNP	OS03G0723400	Intronic	NA	NA	LOC_Os03g51350	expressed protein
Chr.3	30518571	30518571	G	G	C	SNP	OS03G0743500	Exonic	5UTR	NA	LOC_Os03g53200	OsCML4 - Calmodulin-related calcium sensor protein, expressed
Chr.3	32176578	32176578	G	G	C	SNP	OS03G0776000	Exonic	CDS	Missense	LOC_Os03g56460	glucose-6-phosphate isomerase, putative, expressed
Chr.3	35162048	35162048	G	G	C	SNP	OS03G0836800	Intronic	NA	NA	LOC_Os03g62060	hydrolase, putative, expressed
Chr.3	541776	541776	T	T	C	SNP	OS03G0109300	Exonic	CDS	Silent	LOC_Os03g01880	possible lysine decarboxylase domain containing protein, expressed
Chr.3	538264	538264	T	T	C	SNP	OS03G0109300	Intronic	NA	NA	LOC_Os03g01880	possible lysine decarboxylase domain containing protein, expressed
Chr.3	538663	538663	A	A	C	SNP	OS03G0109300	Intronic	NA	NA	LOC_Os03g01880	possible lysine decarboxylase domain containing protein, expressed
Chr.3	538751	538751	G	G	C	SNP	OS03G0109300	Intronic	NA	NA	LOC_Os03g01880	possible lysine decarboxylase domain containing protein, expressed
Chr.3	540127	540127	T	T	C	SNP	OS03G0109300	Intronic	NA	NA	LOC_Os03g01880	possible lysine decarboxylase domain containing protein, expressed
Chr.3	540455	540455	G	G	C	SNP	OS03G0109300	Intronic	NA	NA	LOC_Os03g01880	possible lysine decarboxylase domain containing protein, expressed

Chr.3	540837	540837	T	T	C	SNP	OS03G0109300	Intronic	NA	NA	LOC_Os03g01880	possible lysine decarboxylase domain containing protein, expressed
Chr.3	541106	541106	T	T	C	SNP	OS03G0109300	Intronic	NA	NA	LOC_Os03g01880	possible lysine decarboxylase domain containing protein, expressed
Chr.3	541595	541595	G	G	C	SNP	OS03G0109300	Intronic	NA	NA	LOC_Os03g01880	possible lysine decarboxylase domain containing protein, expressed
Chr.3	541839	541839	G	G	C	SNP	OS03G0109300	Intronic	NA	NA	LOC_Os03g01880	possible lysine decarboxylase domain containing protein, expressed
Chr.3	542004	542004	G	G	C	SNP	OS03G0109300	Intronic	NA	NA	LOC_Os03g01880	possible lysine decarboxylase domain containing protein, expressed
Chr.3	908672	908672	T	T	C	SNP	OS03G0116000	Intronic	NA	NA	LOC_Os03g02480	inner membrane protein, putative, expressed
Chr.3	1612516	1612516	G	G	C	SNP	OS03G0128500	Exonic	3UTR	NA	LOC_Os03g03650	POLD2 - Putative DNA polymerase delta complex subunit, expressed
Chr.3	1629494	1629494	A	A	C	SNP	OS03G0128700	Exonic	3UTR	NA	LOC_Os03g03660	CAMK_CAMK_like.17 - CAMK includes calcium/calmodulin depedent protein kinases, expressed
Chr.3	1628064	1628064	T	T	C	SNP	OS03G0128700	Exonic	CDS	Silent	LOC_Os03g03660	CAMK_CAMK_like.17 - CAMK includes calcium/calmodulin depedent protein kinases, expressed
Chr.3	1628020	1628020	T	T	C	SNP	OS03G0128700	Intronic	NA	NA	LOC_Os03g03660	CAMK_CAMK_like.17 - CAMK includes calcium/calmodulin depedent protein kinases, expressed
Chr.3	1645315	1645315	A	A	C	SNP	OS03G0129100	Exonic	3UTR	NA	LOC_Os03g03700	MLO domain containing protein, putative, expressed
Chr.3	1738932	1738932	T	T	C	SNP	OS03G0130700	Exonic	CDS	NA	LOC_Os03g03860	expressed protein
Chr.3	1739712	1739712	T	T	C	SNP	OS03G0130700	Exonic	CDS	NA	LOC_Os03g03860	expressed protein
Chr.3	1790648	1790648	T	T	C	SNP	OS03G0131200	Exonic	5UTR	NA	LOC_Os03g03910	catalase domain containing protein, expressed
Chr.3	1788545	1788545	T	T	C	SNP	OS03G0131200	Intronic	NA	NA	LOC_Os03g03910	catalase domain containing protein, expressed
Chr.3	1788551	1788551	T	T	C	SNP	OS03G0131200	Intronic	NA	NA	LOC_Os03g03910	catalase domain containing protein, expressed
Chr.3	4630073	4630073	G	G	C	SNP	OS03G0188400	Intronic	NA	NA	LOC_Os03g08930	helix-loop-helix DNA-binding protein, putative, expressed
Chr.3	7349686	7349686	T	T	C	SNP	OS03G0239200	Intronic	NA	NA	LOC_Os03g13590	potyvirus VPg interacting protein, putative, expressed
Chr.3	7849836	7849836	T	T	C	SNP	OS03G0248600	Intronic	NA	NA	LOC_Os03g14450	enolase, putative, expressed
Chr.3	7851482	7851482	G	G	C	SNP	OS03G0248600	Intronic	NA	NA	LOC_Os03g14450	enolase, putative, expressed
Chr.3	10220620	10220620	T	T	C	SNP	OS03G0293500	Exonic	5UTR	NA	LOC_Os03g18220	pyruvate decarboxylase isozyme 2, putative, expressed
Chr.3	10220628	10220628	T	T	C	SNP	OS03G0293500	Exonic	5UTR	NA	LOC_Os03g18220	pyruvate decarboxylase isozyme 2, putative, expressed
Chr.3	10623483	10623483	T	T	C	SNP	OS03G0301700	Intronic	NA	NA	LOC_Os03g18970	protein phosphotase protein, putative, expressed
Chr.3	14482287	14482287	T	T	C	SNP	OS03G0369000	Exonic	3UTR	NA	LOC_Os03g25340	peroxidase precursor, putative, expressed
Chr.3	14480595	14480595	T	T	C	SNP	OS03G0369000	Exonic	5UTR	NA	LOC_Os03g25340	peroxidase precursor, putative, expressed
Chr.3	14481423	14481423	T	T	C	SNP	OS03G0369000	Intronic	NA	NA	LOC_Os03g25340	peroxidase precursor, putative, expressed
Chr.3	29145026	29145026	T	T	C	SNP	OS03G0719300	Exonic	CDS	Silent	LOC_Os03g51000	3,4-dihydroxy-2-butanone kinase, putative, expressed
Chr.3	538823	538825	CTA	CTA	C	INDEL	OS03G0109300	Intronic	NA	NA	LOC_Os03g01880	possible lysine decarboxylase domain containing protein, expressed
Chr.3	538299	538300	CT	CT	C	INDEL	OS03G0109300	Intronic	NA	NA	LOC_Os03g01880	possible lysine decarboxylase domain containing protein, expressed
Chr.3	1238639	1238639	A	A	C	SNP	OS03G0122300	Intronic	NA	NA	LOC_Os03g03034	flavonol synthase/flavanone 3-hydroxylase, putative, expressed
Chr.3	1242986	1242986	A	A	C	SNP	OS03G0122300	Intronic	NA	NA	LOC_Os03g03034	flavonol synthase/flavanone 3-hydroxylase, putative, expressed
Chr.3	1244089	1244089	A	A	C	SNP	OS03G0122300	Intronic	NA	NA	LOC_Os03g03034	flavonol synthase/flavanone 3-hydroxylase, putative, expressed
Chr.3	6460821	6460821	A	A	C	SNP	OS03G0223400	Intronic	NA	NA	LOC_Os03g12290	glutamine synthetase, catalytic domain containing protein, expressed
Chr.3	12349995	12349995	A	A	C	SNP	OS03G0334100	Intronic	NA	NA	LOC_Os03g21630	PRC-barrel domain protein, putative, expressed
Chr.3	24815848	24815848	A	A	C	SNP	OS03G0643300	Exonic	CDS	Missense	LOC_Os03g44150	aminotransferase, putative, expressed
Chr.3	24816498	24816498	A	A	C	SNP	OS03G0643300	Intronic	NA	NA	LOC_Os03g44150	aminotransferase, putative, expressed
Chr.3	24817137	24817137	A	A	C	SNP	OS03G0643300	Intronic	NA	NA	LOC_Os03g44150	aminotransferase, putative, expressed
Chr.3	25001894	25001894	A	A	C	SNP	OS03G0646200	Exonic	3UTR	NA	LOC_Os03g44430	ubiquitin carboxyl-terminal hydrolase, family 1, putative, expressed
Chr.3	27336165	27336165	A	A	C	SNP	OS03G0685300	Intronic	NA	NA	LOC_Os03g48060	class I glutamine amidotransferase, putative, expressed
Chr.3	32176679	32176679	A	A	C	SNP	OS03G0776000	Intronic	NA	NA	LOC_Os03g56460	glucose-6-phosphate isomerase, putative, expressed
Chr.3	32179846	32179846	A	A	C	SNP	OS03G0776000	Intronic	NA	NA	LOC_Os03g56460	glucose-6-phosphate isomerase, putative, expressed
Chr.3	35660387	35660387	A	A	C	SNP	OS03G0848100	Exonic	3UTR	NA	LOC_Os03g63074	Ser/Thr protein phosphatase family protein, expressed
Chr.3	540933	540933	T	T	C	SNP	OS03G0109300	Intronic	NA	NA	LOC_Os03g01880	possible lysine decarboxylase domain containing protein, expressed
Chr.3	540978	540978	T	T	C	SNP	OS03G0109300	Intronic	NA	NA	LOC_Os03g01880	possible lysine decarboxylase domain containing protein, expressed
Chr.3	541014	541014	G	G	C	SNP	OS03G0109300	Intronic	NA	NA	LOC_Os03g01880	possible lysine decarboxylase domain containing protein, expressed
Chr.3	541084	541084	T	T	C	SNP	OS03G0109300	Intronic	NA	NA	LOC_Os03g01880	possible lysine decarboxylase domain containing protein, expressed
Chr.3	1235465	1235465	G	G	C	SNP	OS03G0122300	Exonic	3UTR	NA	LOC_Os03g03034	flavonol synthase/flavanone 3-hydroxylase, putative, expressed
Chr.3	1235868	1235868	T	T	C	SNP	OS03G0122300	Exonic	CDS	Silent	LOC_Os03g03034	flavonol synthase/flavanone 3-hydroxylase, putative, expressed
Chr.3	1608960	1608960	G	G	C	SNP	OS03G0128300	Exonic	5UTR	NA	LOC_Os03g03630	expressed protein
Chr.3	1608614	1608614	T	T	C	SNP	OS03G0128300	Exonic	CDS	Missense	LOC_Os03g03630	expressed protein
Chr.3	1742398	1742398	T	T	C	SNP	OS03G0130700	Exonic	5UTR	NA	LOC_Os03g03860	expressed protein
Chr.3	1737934	1737934	A	A	C	SNP	OS03G0130700	Exonic	CDS	NA	LOC_Os03g03860	expressed protein
Chr.3	1788451	1788451	T	T	C	SNP	OS03G0131200	Intronic	NA	NA	LOC_Os03g03910	catalase domain containing protein, expressed
Chr.3	1788459	1788459	T	T	C	SNP	OS03G0131200	Intronic	NA	NA	LOC_Os03g03910	catalase domain containing protein, expressed
Chr.3	1790545	1790545	A	A	C	SNP	OS03G0131200	Intronic	NA	NA	LOC_Os03g03910	catalase domain containing protein, expressed
Chr.3	1876228	1876228	T	T	C	SNP	OS03G0133300	Intronic	NA	NA	LOC_Os03g04100	expressed protein
Chr.3	2750875	2750875	A	A	C	SNP	OS03G0149300	Intronic	NA	NA	LOC_Os03g05530	nodulin, putative, expressed
Chr.3	2751674	2751674	A	A	C	SNP	OS03G0149300	Intronic	NA	NA	LOC_Os03g05530	nodulin, putative, expressed
Chr.3	3529656	3529656	A	A	C	SNP	OS03G0165400	Intronic	NA	NA	LOC_Os03g06940	beta-galactosidase, putative, expressed
Chr.3	4387294	4387294	T	T	C	SNP	OS03G0183600	Intronic	NA	NA	LOC_Os03g08530	aminotransferase, classes I and II, domain containing protein, expressed
Chr.3	4387323	4387323	T	T	C	SNP	OS03G0183600	Intronic	NA	NA	LOC_Os03g08530	aminotransferase, classes I and II, domain containing protein, expressed
Chr.3	4387424	4387424	T	T	C	SNP	OS03G0183600	Intronic	NA	NA	LOC_Os03g08530	aminotransferase, classes I and II, domain containing protein, expressed
Chr.3	5979021	5979021	T	T	C	SNP	OS03G0214000	Exonic	3UTR	NA	LOC_Os03g11530	nucleotide pyrophosphatase/phosphodiesterase, putative, expressed
Chr.3	6625498	6625498	G	G	C	SNP	OS03G0226200	Exonic	CDS	Silent	LOC_Os03g12510	non-symbiotic hemoglobin 2, putative, expressed
Chr.3	7565687	7565687	G	G	C	SNP	OS03G0243100	Exonic	3UTR	NA	LOC_Os03g13950	actin-depolymerizing factor, putative, expressed
Chr.3	8200985	8200985	T	T	C	SNP	OS03G0255100	Intronic	NA	NA	LOC_Os03g15020	beta-galactosidase precursor, putative, expressed
Chr.3	8352450	8352450	T	T	C	SNP	OS03G0258200	Exonic	CDS	Missense	LOC_Os03g15270	gibberellin receptor GID1L2, putative, expressed
Chr.3	8842481	8842481	G	G	C	SNP	OS03G0267300	Intronic	NA	NA	LOC_Os03g16050	fructose-1,6-bisphosphatase, putative, expressed
Chr.3	13190001	13190001	G	G	C	SNP	OS03G0351700	Exonic	CDS	Missense	LOC_Os03g22820	expressed protein
Chr.3	15470212	15470212	G	G	C	SNP	OS03G0387900	Exonic	CDS	Missense	LOC_Os03g27019	expressed protein
Chr.3	15669361	15669361	G	G	C	SNP	OS03G0391100	Intronic	NA	NA	LOC_Os03g27360	RING-H2 finger protein ATL5H precursor, putative, expressed
Chr.3	15669750	15669750	T	T	C	SNP	OS03G0391100	Intronic	NA	NA	LOC_Os03g27360	RING-H2 finger protein ATL5H precursor, putative, expressed
Chr.3	16286379	16286379	T	T	C	SNP	OS03G0401100	Exonic	3UTR	NA	LOC_Os03g28300	protein kinase domain containing protein, expressed
Chr.3	16288990	16288990	T	T	C	SNP	OS03G0401100	Intronic	NA	NA	LOC_Os03g28300	protein kinase domain containing protein, expressed
Chr.3	16593522	16593522	A	A	C	SNP	OS03G0405500	Exonic	CDS	Silent	LOC_Os03g29190	PDI, putative, expressed
Chr.3	17288504	17288504	T	T	C	SNP	OS03G0416500	Exonic	CDS	Missense	LOC_Os03g30300	6-phosphogluconolactonase, putative, expressed
Chr.3	17289474	17289474	G	G	C	SNP	OS03G0416500	Intronic	NA	NA	LOC_Os03g30300	6-phosphogluconolactonase, putative, expressed
Chr.3	17292567	17292567	T	T	C	SNP	OS03G0416500	Intronic	NA	NA	LOC_Os03g30300	6-phosphogluconolactonase, putative, expressed
Chr.3	18687536	18687536	T	T	C	SNP	OS03G0441500	Exonic	CDS	Missense	LOC_Os03g32630	ABC transporter, ATP-binding protein, putative, expressed
Chr.3	20019247	20019247	T	T	C	SNP	OS03G0559700	Exonic	CDS	Missense	LOC_Os03g36080	expressed protein
Chr.3	21636823	21636823	T	T	C	SNP	OS03G0586500	Intronic	NA	NA	LOC_Os03g38950	chloroplast post-illumination chlorophyll fluorescence increase protein, putative, expressed
Chr.3	24471312	24471312	T	T	C	SNP	OS03G0638400	Intronic	NA	NA	LOC_Os03g43750	retrotransposon, putative, centromere-specific, expressed
Chr.3	24814689	24814689	A	A	C	SNP	OS03G0643300	Intronic	NA	NA	LOC_Os03g44150	aminotransferase, putative, expressed
Chr.3	24814788	24814788	A	A	C	SNP	OS03G0643300	Intronic	NA	NA	LOC_Os03g44150	aminotransferase, putative, expressed
Chr.3	25946377	25946377	A	A	C	SNP	OS03G0661300	Exonic	CDS	Missense	LOC_Os03g45920	tubulin/FtsZ domain containing protein, putative, expressed

Chr.3	28244550	28244550	A	A	C	SNP	OS03G0703000	Intronic	NA	NA	LOC_Os03g49600	Os3bglu7 - beta-glucosidase, exo-beta-glucanse, expressed
Chr.3	29388032	29388032	T	T	C	SNP	OS03G0723400	Exonic	3UTR	NA	LOC_Os03g51350	expressed protein
Chr.3	32567335	32567335	T	T	C	SNP	OS03G0784700	Exonic	CDS	Silent	LOC_Os03g57120	ferredoxin--NADP reductase, chloroplast precursor, putative, expressed
Chr.3	32668514	32668514	A	A	C	SNP	OS03G0786700	Exonic	CDS	Silent	LOC_Os03g57280	N-dimethylguanosine tRNA methyltransferase, putative, expressed
Chr.3	33217292	33217292	G	G	C	SNP	OS03G0797400	Exonic	5UTR	NA	LOC_Os03g58300	indole-3-glycerol phosphate lyase, chloroplast precursor, putative, expressed
Chr.3	33349143	33349143	T	T	C	SNP	OS03G0799900	Exonic	3UTR	NA	LOC_Os03g58570	WD domain, G-beta repeat domain containing protein, expressed
Chr.3	35380336	35380336	T	T	C	SNP	OS03G0841600	Exonic	3UTR	NA	LOC_Os03g62480	anthocyanidin 5,3-O-glucosyltransferase, putative, expressed
Chr.3	35790866	35790866	T	T	C	SNP	OS03G0850400	Intronic	NA	NA	LOC_Os03g63330	aspartokinase, chloroplast precursor, putative, expressed
Chr.3	1716613	1716619	CGCGGCG	CGCGGCG	C	INDEL	OS03G0130400	Exonic	CDS	InFrame	LOC_Os03g03820	adenylate kinase, putative, expressed
Chr.3	1790523	1790524	CA	CA	C	INDEL	OS03G0131200	Intronic	NA	NA	LOC_Os03g03910	catalase domain containing protein, expressed
Chr.3	3109077	3109089	CATGATTTAAGCT	CATGATTTAAGCT	C	INDEL	OS03G0157800	Intronic	NA	NA	LOC_Os03g06190	3-5 exonuclease family protein, putative, expressed
Chr.3	5001019	5001020	CT	CT	C	INDEL	OS03G0196000	Intronic	NA	NA	LOC_Os03g09980	sulfate transporter, putative, expressed
Chr.3	8223139	8223141	CTT	CTT	C	INDEL	OS03G0255500	Intronic	NA	NA	LOC_Os03g15050	phosphoenolpyruvate carboxykinase, putative, expressed
Chr.3	12588516	12588521	CTCTCT	CTCTCT	C	INDEL	OS03G0338400	Intronic	NA	NA	LOC_Os03g21970	basic helix-loop-helix domain containing protein, expressed
Chr.3	15669529	15669530	CT	CT	C	INDEL	OS03G0391100	Intronic	NA	NA	LOC_Os03g27360	RING-H2 finger protein ATL5H precursor, putative, expressed
Chr.3	15670064	15670069	CCCTGA	CCCTGA	C	INDEL	OS03G0391100	Intronic	NA	NA	LOC_Os03g27360	RING-H2 finger protein ATL5H precursor, putative, expressed
Chr.3	22947842	22947843	CT	CT	C	INDEL	OS03G0609000	Intronic	NA	NA	LOC_Os03g41280	expressed protein
Chr.3	23369956	23369958	CAA	CAA	C	INDEL	OS03G0616700	Intronic	NA	NA	LOC_Os03g42050	exportin-7-A, putative, expressed
Chr.3	23375165	23375166	CA	CA	C	INDEL	OS03G0616700	Intronic	NA	NA	LOC_Os03g42050	exportin-7-A, putative, expressed
Chr.3	23490390	23490392	CTG	CTG	C	INDEL	OS03G0619400	Exonic	3UTR	NA	LOC_Os03g42220	T-complex protein, putative, expressed
Chr.3	24449990	24449991	CT	CT	C	INDEL	OS03G0638200	Exonic	5UTR	NA	LOC_Os03g43720	transporter family protein, putative, expressed
Chr.3	24814575	24814576	CA	CA	C	INDEL	OS03G0643300	Intronic	NA	NA	LOC_Os03g44150	aminotransferase, putative, expressed
Chr.3	24814734	24814735	CA	CA	C	INDEL	OS03G0643300	Intronic	NA	NA	LOC_Os03g44150	aminotransferase, putative, expressed
Chr.3	24815494	24815496	CTG	CTG	C	INDEL	OS03G0643300	Intronic	NA	NA	LOC_Os03g44150	aminotransferase, putative, expressed
Chr.3	24816258	24816259	CT	CT	C	INDEL	OS03G0643300	Intronic	NA	NA	LOC_Os03g44150	aminotransferase, putative, expressed
Chr.3	24816827	24816829	CTT	CTT	C	INDEL	OS03G0643300	Intronic	NA	NA	LOC_Os03g44150	aminotransferase, putative, expressed
Chr.3	25062171	25062173	CTT	CTT	C	INDEL	OS03G0647400	Exonic	3UTR	NA	LOC_Os03g44520	expressed protein
Chr.3	28595094	28595108	CAGTCTGAAGTCTGA	CAGTCTGAAGTCTGA	C	INDEL	OS03G0709000	Intronic	NA	NA	LOC_Os03g50130	microsomal glutathione S-transferase 3, putative, expressed
Chr.3	29564777	29564778	CA	CA	C	INDEL	OS03G0726500	Intronic	NA	NA	LOC_Os03g51650	membrane protein, putative, expressed
Chr.3	33502597	33502603	CTCCACT	CTCCACT	C	INDEL	OS03G0802900	Exonic	CDS	InFrame	LOC_Os03g58830	BEE 1, putative, expressed
Chr.3	33502693	33502694	CT	CT	C	INDEL	OS03G0802900	Intronic	NA	NA	LOC_Os03g58830	BEE 1, putative, expressed
Chr.3	35871597	35871598	CA	CA	C	INDEL	OS03G0851900	Intronic	NA	NA	LOC_Os03g63500	ATPase, AFG1 family domain containing protein, expressed
Chr.3	36007441	36007444	CGGA	CGGA	C	INDEL	OS03G0854800	Exonic	CDS	NA	LOC_Os03g63770	RCD1, putative, expressed
Chr.2	3828409	3828409	T	T	C	SNP	OS02G0170100	Intronic	NA	NA	LOC_Os02g07410	glycine cleavage system H protein, putative, expressed
Chr.2	4293401	4293401	T	T	C	SNP	OS02G0177800	Exonic	3UTR	NA	LOC_Os02g08120	calmodulin binding protein, putative, expressed
Chr.2	4756219	4756219	T	T	C	SNP	OS02G0185200	Exonic	3UTR	NA	LOC_Os02g09240	cytochrome P450 71D8, putative, expressed
Chr.2	4755205	4755205	T	T	C	SNP	OS02G0185200	Intronic	NA	NA	LOC_Os02g09240	cytochrome P450 71D8, putative, expressed
Chr.2	4888557	4888557	T	T	C	SNP	OS02G0188000	Exonic	3UTR	NA	LOC_Os02g09510	limonoid UDP-glucosyltransferase, putative, expressed
Chr.2	5156711	5156711	T	T	C	SNP	OS02G0192500	Intronic	NA	NA	LOC_Os02g09930	CSLA1 - cellulose synthase-like family A; mannan synthase, expressed
Chr.2	5250301	5250301	T	T	C	SNP	OS02G0194200	Exonic	5UTR	NA	LOC_Os02g10080	zinc finger C-x8-C-x5-C-x3-H type family protein, expressed
Chr.2	5252664	5252664	T	T	C	SNP	OS02G0194200	Intronic	NA	NA	LOC_Os02g10080	zinc finger C-x8-C-x5-C-x3-H type family protein, expressed
Chr.2	5760291	5760291	T	T	C	SNP	OS02G0202950	Exonic	CDS	NA	LOC_Os02g10860	bZIP transcription factor domain containing protein, expressed
Chr.2	5760302	5760302	T	T	C	SNP	OS02G0202950	Exonic	CDS	NA	LOC_Os02g10860	bZIP transcription factor domain containing protein, expressed
Chr.2	5760343	5760343	T	T	C	SNP	OS02G0202950	Exonic	CDS	NA	LOC_Os02g10860	bZIP transcription factor domain containing protein, expressed
Chr.2	5760346	5760346	T	T	C	SNP	OS02G0202950	Exonic	CDS	NA	LOC_Os02g10860	bZIP transcription factor domain containing protein, expressed
Chr.2	6833312	6833312	T	T	C	SNP	OS02G0222500	Exonic	3UTR	NA	LOC_Os02g12939	expressed protein
Chr.2	6834587	6834587	T	T	C	SNP	OS02G0222500	Exonic	CDS	NA	LOC_Os02g12939	expressed protein
Chr.2	6834590	6834590	T	T	C	SNP	OS02G0222500	Exonic	CDS	NA	LOC_Os02g12939	expressed protein
Chr.2	6834611	6834611	T	T	C	SNP	OS02G0222500	Exonic	CDS	NA	LOC_Os02g12939	expressed protein
Chr.2	6835399	6835399	T	T	C	SNP	OS02G0222500	Intronic	NA	NA	LOC_Os02g12939	expressed protein
Chr.2	7221872	7221872	T	T	C	SNP	OS02G0228300	Exonic	CDS	Missense	LOC_Os02g13510	receptor-like protein kinase 5 precursor, putative, expressed
Chr.2	7224350	7224350	T	T	C	SNP	OS02G0228300	Exonic	CDS	Missense	LOC_Os02g13510	receptor-like protein kinase 5 precursor, putative, expressed
Chr.2	9650763	9650763	T	T	C	SNP	OS02G0269600	Exonic	CDS	Silent	LOC_Os02g16940	OsSub13 - Putative Subtilisin homologue, expressed
Chr.2	9651558	9651558	T	T	C	SNP	OS02G0269600	Exonic	CDS	Silent	LOC_Os02g16940	OsSub13 - Putative Subtilisin homologue, expressed
Chr.2	9651166	9651166	T	T	C	SNP	OS02G0269600	Exonic	CDS	Missense	LOC_Os02g16940	OsSub13 - Putative Subtilisin homologue, expressed
Chr.2	9651518	9651518	T	T	C	SNP	OS02G0269600	Exonic	CDS	Missense	LOC_Os02g16940	OsSub13 - Putative Subtilisin homologue, expressed
Chr.2	9654206	9654206	T	T	C	SNP	OS02G0269600	Exonic	CDS	Missense	LOC_Os02g16940	OsSub13 - Putative Subtilisin homologue, expressed
Chr.2	9927819	9927819	T	T	C	SNP	OS02G0272900	Exonic	CDS	Silent	LOC_Os02g17304	resistance protein, putative, expressed
Chr.2	9925605	9925605	T	T	C	SNP	OS02G0272900	Intronic	NA	NA	LOC_Os02g17304	resistance protein, putative, expressed
Chr.2	9972827	9972827	T	T	C	SNP	OS02G0273700	Exonic	5UTR	NA	LOC_Os02g17350	VHS and GAT domain containing protein, expressed
Chr.2	9975474	9975474	T	T	C	SNP	OS02G0273700	Exonic	CDS	Silent	LOC_Os02g17350	VHS and GAT domain containing protein, expressed
Chr.2	9976417	9976417	T	T	C	SNP	OS02G0273700	Exonic	CDS	Silent	LOC_Os02g17350	VHS and GAT domain containing protein, expressed
Chr.2	9973487	9973487	T	T	C	SNP	OS02G0273700	Intronic	NA	NA	LOC_Os02g17350	VHS and GAT domain containing protein, expressed
Chr.2	9973630	9973630	T	T	C	SNP	OS02G0273700	Intronic	NA	NA	LOC_Os02g17350	VHS and GAT domain containing protein, expressed
Chr.2	9974205	9974205	T	T	C	SNP	OS02G0273700	Intronic	NA	NA	LOC_Os02g17350	VHS and GAT domain containing protein, expressed
Chr.2	9988942	9988942	T	T	C	SNP	OS02G0273900	Exonic	3UTR	NA	LOC_Os02g17370	expressed protein
Chr.2	9989323	9989323	T	T	C	SNP	OS02G0273900	Intronic	NA	NA	LOC_Os02g17370	expressed protein
Chr.2	9989351	9989351	T	T	C	SNP	OS02G0273900	Intronic	NA	NA	LOC_Os02g17370	expressed protein
Chr.2	9990215	9990215	T	T	C	SNP	OS02G0273900	Intronic	NA	NA	LOC_Os02g17370	expressed protein
Chr.2	11484514	11484514	T	T	C	SNP	OS02G0299300	Exonic	3UTR	NA	LOC_Os02g19650	hydrolase, alpha/beta fold family domain containing protein, expressed
Chr.2	16115696	16115696	T	T	C	SNP	OS02G0473200	Exonic	CDS	Missense	LOC_Os02g27360	aspartic proteinase-like protein 2 precursor, putative, expressed
Chr.2	16115704	16115704	T	T	C	SNP	OS02G0473200	Exonic	CDS	Missense	LOC_Os02g27360	aspartic proteinase-like protein 2 precursor, putative, expressed
Chr.2	16117363	16117363	T	T	C	SNP	OS02G0473200	Intronic	NA	NA	LOC_Os02g27360	aspartic proteinase-like protein 2 precursor, putative, expressed
Chr.2	20815149	20815149	T	T	C	SNP	OS02G0552000	Exonic	CDS	Missense	LOC_Os02g34690	transmembrane 9 superfamily member, putative, expressed
Chr.2	21810480	21810480	T	T	C	SNP	OS02G0570700	Exonic	3UTR	NA	LOC_Os02g36190	cytochrome P450, putative, expressed
Chr.2	21842638	21842638	T	T	C	SNP	OS02G0570900	Exonic	CDS	Silent	LOC_Os02g36210	ent-kaurene synthase, chloroplast precursor, putative, expressed
Chr.2	21843263	21843263	T	T	C	SNP	OS02G0570900	Exonic	CDS	Silent	LOC_Os02g36210	ent-kaurene synthase, chloroplast precursor, putative, expressed
Chr.2	22298758	22298758	T	T	C	SNP	OS02G0579600	Intronic	NA	NA	LOC_Os02g36924	OsMADS27 - MADS-box family gene with MIKCc type-box, expressed
Chr.2	23530448	23530448	T	T	C	SNP	OS02G0601300	Exonic	3UTR	NA	LOC_Os02g38920	glyceraldehyde-3-phosphate dehydrogenase, putative, expressed
Chr.2	23528236	23528236	T	T	C	SNP	OS02G0601300	Intronic	NA	NA	LOC_Os02g38920	glyceraldehyde-3-phosphate dehydrogenase, putative, expressed
Chr.2	24901683	24901683	T	T	C	SNP	OS02G0624400	Exonic	CDS	Silent	LOC_Os02g41520	glycosyl transferase 8 domain containing protein, putative, expressed
Chr.2	26770303	26770303	T	T	C	SNP	OS02G0661100	Exonic	5UTR	NA	LOC_Os02g44230	CPuORF22 - conserved peptide uORF-containing transcript, expressed
Chr.2	30458401	30458401	T	T	C	SNP	OS02G0731200	Exonic	CDS	Silent	LOC_Os02g49840	OsMADS57 - MADS-box family gene with MIKCc type-box, expressed
Chr.2	33954414	33954414	T	T	C	SNP	OS02G0797600	Exonic	3UTR	NA	LOC_Os02g55430	alginate regulatory protein AlgP, putative, expressed
Chr.2	34042415	34042415	T	T	C	SNP	OS02G0799300	Intronic	NA	NA	LOC_Os02g55590	expressed protein

Chr.2	35098027	35098027	T	T	C	SNP	OS02G0817900	Exonic	CDS	Silent	LOC_Os02g57290	cytochrome P450, putative, expressed
Chr.2	35098099	35098099	T	T	C	SNP	OS02G0817900	Exonic	CDS	Silent	LOC_Os02g57290	cytochrome P450, putative, expressed
Chr.2	35098348	35098348	T	T	C	SNP	OS02G0817900	Exonic	CDS	Silent	LOC_Os02g57290	cytochrome P450, putative, expressed
Chr.2	35097776	35097776	T	T	C	SNP	OS02G0817900	Exonic	CDS	Missense	LOC_Os02g57290	cytochrome P450, putative, expressed
Chr.2	35095100	35095100	T	T	C	SNP	OS02G0817900	Intronic	NA	NA	LOC_Os02g57290	cytochrome P450, putative, expressed
Chr.2	35095492	35095492	T	T	C	SNP	OS02G0817900	Intronic	NA	NA	LOC_Os02g57290	cytochrome P450, putative, expressed
Chr.2	35096586	35096586	T	T	C	SNP	OS02G0817900	Intronic	NA	NA	LOC_Os02g57290	cytochrome P450, putative, expressed
Chr.2	35098398	35098398	T	T	C	SNP	OS02G0817900	Intronic	NA	NA	LOC_Os02g57290	cytochrome P450, putative, expressed
Chr.2	35350740	35350740	T	T	C	SNP	OS02G0823100	Intronic	NA	NA	LOC_Os02g57720	aquaporin protein, putative, expressed
Chr.2	2744395	2744395	G	G	C	SNP	OS02G0149800	Exonic	CDS	Silent	LOC_Os02g05630	protein phosphatase 2C, putative, expressed
Chr.2	5154727	5154727	G	G	C	SNP	OS02G0192500	Intronic	NA	NA	LOC_Os02g09930	CSLA1 - cellulose synthase-like family A; mannan synthase, expressed
Chr.2	9974378	9974378	G	G	C	SNP	OS02G0273700	Intronic	NA	NA	LOC_Os02g17350	VHS and GAT domain containing protein, expressed
Chr.2	9989765	9989765	G	G	C	SNP	OS02G0273900	Intronic	NA	NA	LOC_Os02g17370	expressed protein
Chr.2	9989891	9989891	G	G	C	SNP	OS02G0273900	Intronic	NA	NA	LOC_Os02g17370	expressed protein
Chr.2	9990214	9990214	G	G	C	SNP	OS02G0273900	Intronic	NA	NA	LOC_Os02g17370	expressed protein
Chr.2	21933236	21933236	G	G	C	SNP	OS02G0572400	Exonic	CDS	Missense	LOC_Os02g36340	riboflavin biosynthesis protein ribAB, chloroplast precursor, putative, expressed
Chr.2	35098385	35098385	G	G	C	SNP	OS02G0817900	Intronic	NA	NA	LOC_Os02g57290	cytochrome P450, putative, expressed
Chr.2	769890	769890	T	T	C	SNP	OS02G0114200	Exonic	CDS	Silent	LOC_Os02g02320	OsSCP6 - Putative Serine Carboxypeptidase homologue, expressed
Chr.2	2746654	2746654	T	T	C	SNP	OS02G0149800	Exonic	CDS	Silent	LOC_Os02g05630	protein phosphatase 2C, putative, expressed
Chr.2	3037755	3037755	T	T	C	SNP	OS02G0155400	Exonic	CDS	Silent	LOC_Os02g06090	phytosulfokine receptor precursor, putative, expressed
Chr.2	3037847	3037847	T	T	C	SNP	OS02G0155400	Exonic	CDS	Silent	LOC_Os02g06090	phytosulfokine receptor precursor, putative, expressed
Chr.2	3037944	3037944	T	T	C	SNP	OS02G0155400	Exonic	CDS	Silent	LOC_Os02g06090	phytosulfokine receptor precursor, putative, expressed
Chr.2	3038028	3038028	T	T	C	SNP	OS02G0155400	Exonic	CDS	Silent	LOC_Os02g06090	phytosulfokine receptor precursor, putative, expressed
Chr.2	3038048	3038048	T	T	C	SNP	OS02G0155400	Exonic	CDS	Silent	LOC_Os02g06090	phytosulfokine receptor precursor, putative, expressed
Chr.2	3038820	3038820	T	T	C	SNP	OS02G0155400	Exonic	CDS	Silent	LOC_Os02g06090	phytosulfokine receptor precursor, putative, expressed
Chr.2	3038930	3038930	T	T	C	SNP	OS02G0155400	Exonic	CDS	Silent	LOC_Os02g06090	phytosulfokine receptor precursor, putative, expressed
Chr.2	3039386	3039386	T	T	C	SNP	OS02G0155400	Exonic	CDS	Silent	LOC_Os02g06090	phytosulfokine receptor precursor, putative, expressed
Chr.2	3039464	3039464	T	T	C	SNP	OS02G0155400	Exonic	CDS	Silent	LOC_Os02g06090	phytosulfokine receptor precursor, putative, expressed
Chr.2	3038497	3038497	T	T	C	SNP	OS02G0155400	Exonic	CDS	Missense	LOC_Os02g06090	phytosulfokine receptor precursor, putative, expressed
Chr.2	3039217	3039217	T	T	C	SNP	OS02G0155400	Exonic	CDS	Missense	LOC_Os02g06090	phytosulfokine receptor precursor, putative, expressed
Chr.2	8837269	8837269	T	T	C	SNP	OS02G0256100	Exonic	3UTR	NA	LOC_Os02g15690	polygalacturonase, putative, expressed
Chr.2	18578262	18578262	T	T	C	SNP	OS02G0515200	Intronic	NA	NA	LOC_Os02g31080	hydroxyproline-rich glycoprotein family protein, putative, expressed
Chr.2	21930811	21930811	T	T	C	SNP	OS02G0572400	Exonic	3UTR	NA	LOC_Os02g36340	riboflavin biosynthesis protein ribAB, chloroplast precursor, putative, expressed
Chr.2	21931954	21931954	T	T	C	SNP	OS02G0572400	Intronic	NA	NA	LOC_Os02g36340	riboflavin biosynthesis protein ribAB, chloroplast precursor, putative, expressed
Chr.2	22510321	22510321	T	T	C	SNP	OS02G0583700	Intronic	NA	NA	LOC_Os02g37250	hypothetical protein
Chr.2	771434	771434	G	G	C	SNP	OS02G0114200	Intronic	NA	NA	LOC_Os02g02320	OsSCP6 - Putative Serine Carboxypeptidase homologue, expressed
Chr.2	3038540	3038540	G	G	C	SNP	OS02G0155400	Exonic	CDS	Silent	LOC_Os02g06090	phytosulfokine receptor precursor, putative, expressed
Chr.2	3037720	3037720	G	G	C	SNP	OS02G0155400	Exonic	CDS	Missense	LOC_Os02g06090	phytosulfokine receptor precursor, putative, expressed
Chr.2	3037740	3037740	G	G	C	SNP	OS02G0155400	Exonic	CDS	Missense	LOC_Os02g06090	phytosulfokine receptor precursor, putative, expressed
Chr.2	18578459	18578459	G	G	C	SNP	OS02G0515200	Exonic	CDS	Missense	LOC_Os02g31080	hydroxyproline-rich glycoprotein family protein, putative, expressed
Chr.2	22687831	22687831	G	G	C	SNP	OS02G0588500	Intronic	NA	NA	LOC_Os02g37590	glycerophosphoryl diester phosphodiesterase family protein, putative, expressed
Chr.2	2746074	2746074	A	A	C	SNP	OS02G0149800	Intronic	NA	NA	LOC_Os02g05630	protein phosphatase 2C, putative, expressed
Chr.2	18540400	18540400	A	A	C	SNP	OS02G0514500	Exonic	CDS	NA	LOC_Os02g31030	glycerophosphoryl diester phosphodiesterase family protein, putative, expressed
Chr.2	28268001	28268001	A	A	C	SNP	OS02G0689200	Exonic	5UTR	NA	LOC_Os02g46380	purine permease, putative, expressed
Chr.2	4390252	4390256	CTATA	CTATA	C	INDEL	OS02G0179200	Exonic	3UTR	NA	LOC_Os02g08270	class I glutamine amidotransferase, putative, expressed
Chr.2	2745062	2745064	CAA	CAA	C	INDEL	OS02G0149800	Intronic	NA	NA	LOC_Os02g05630	protein phosphatase 2C, putative, expressed
Chr.2	1308625	1308625	A	A	C	SNP	OS02G0125100	Intronic	NA	NA	LOC_Os02g03260	3-isopropylmalate dehydratase large subunit 2, putative, expressed
Chr.2	4452205	4452205	A	A	C	SNP	OS02G0180000	Exonic	CDS	Silent	LOC_Os02g08364	protein phosphatase 2C, putative, expressed
Chr.2	6832099	6832099	A	A	C	SNP	OS02G0222500	Exonic	3UTR	NA	LOC_Os02g12939	expressed protein
Chr.2	6834604	6834604	A	A	C	SNP	OS02G0222500	Exonic	CDS	NA	LOC_Os02g12939	expressed protein
Chr.2	7224348	7224348	A	A	C	SNP	OS02G0228300	Exonic	CDS	Silent	LOC_Os02g13510	receptor-like protein kinase 5 precursor, putative, expressed
Chr.2	9973021	9973021	A	A	C	SNP	OS02G0273700	Exonic	5UTR	NA	LOC_Os02g17350	VHS and GAT domain containing protein, expressed
Chr.2	9973607	9973607	A	A	C	SNP	OS02G0273700	Intronic	NA	NA	LOC_Os02g17350	VHS and GAT domain containing protein, expressed
Chr.2	9973613	9973613	A	A	C	SNP	OS02G0273700	Intronic	NA	NA	LOC_Os02g17350	VHS and GAT domain containing protein, expressed
Chr.2	9973997	9973997	A	A	C	SNP	OS02G0273700	Intronic	NA	NA	LOC_Os02g17350	VHS and GAT domain containing protein, expressed
Chr.2	9976032	9976032	A	A	C	SNP	OS02G0273700	Intronic	NA	NA	LOC_Os02g17350	VHS and GAT domain containing protein, expressed
Chr.2	9989259	9989259	A	A	C	SNP	OS02G0273900	Intronic	NA	NA	LOC_Os02g17370	expressed protein
Chr.2	9989269	9989269	A	A	C	SNP	OS02G0273900	Intronic	NA	NA	LOC_Os02g17370	expressed protein
Chr.2	9989611	9989611	A	A	C	SNP	OS02G0273900	Intronic	NA	NA	LOC_Os02g17370	expressed protein
Chr.2	17527956	17527956	A	A	C	SNP	OS02G0498300	Intronic	NA	NA	LOC_Os02g29510	DUF803 domain containing, putative, expressed
Chr.2	22300678	22300678	A	A	C	SNP	OS02G0579600	Intronic	NA	NA	LOC_Os02g36924	OsMADS27 - MADS-box family gene with MIKCc type-box, expressed
Chr.2	35098137	35098137	A	A	C	SNP	OS02G0817900	Exonic	CDS	Missense	LOC_Os02g57290	cytochrome P450, putative, expressed
Chr.2	82387	82387	T	T	C	SNP	OS02G0101500	Exonic	CDS	Silent	LOC_Os02g01150	erythronate-4-phosphate dehydrogenase domain containing protein, expressed
Chr.2	1083914	1083914	T	T	C	SNP	OS02G0120800	Exonic	5UTR	NA	LOC_Os02g02840	ras-related protein, putative, expressed
Chr.2	4298964	4298964	A	A	C	SNP	OS02G0177800	Exonic	5UTR	NA	LOC_Os02g08120	calmodulin binding protein, putative, expressed
Chr.2	4298799	4298799	T	T	C	SNP	OS02G0177800	Exonic	CDS	Missense	LOC_Os02g08120	calmodulin binding protein, putative, expressed
Chr.2	4298924	4298924	T	T	C	SNP	OS02G0177800	Intronic	NA	NA	LOC_Os02g08120	calmodulin binding protein, putative, expressed
Chr.2	4754555	4754555	T	T	C	SNP	OS02G0185200	Exonic	CDS	Silent	LOC_Os02g09240	cytochrome P450 71D8, putative, expressed
Chr.2	5155457	5155457	T	T	C	SNP	OS02G0192500	Intronic	NA	NA	LOC_Os02g09930	CSLA1 - cellulose synthase-like family A; mannan synthase, expressed
Chr.2	5156708	5156708	T	T	C	SNP	OS02G0192500	Intronic	NA	NA	LOC_Os02g09930	CSLA1 - cellulose synthase-like family A; mannan synthase, expressed
Chr.2	5195059	5195059	T	T	C	SNP	OS02G0193300	Exonic	CDS	Missense	LOC_Os02g09990	expressed protein
Chr.2	5195203	5195203	G	G	C	SNP	OS02G0193300	Exonic	CDS	Missense	LOC_Os02g09990	expressed protein
Chr.2	5250584	5250584	T	T	C	SNP	OS02G0194200	Exonic	5UTR	NA	LOC_Os02g10080	zinc finger C-x8-C-x5-C-x3-H type family protein, expressed
Chr.2	5251375	5251375	T	T	C	SNP	OS02G0194200	Intronic	NA	NA	LOC_Os02g10080	zinc finger C-x8-C-x5-C-x3-H type family protein, expressed
Chr.2	5760099	5760099	T	T	C	SNP	OS02G0202950	Exonic	CDS	NA	LOC_Os02g10860	bZIP transcription factor domain containing protein, expressed
Chr.2	6146371	6146371	T	T	C	SNP	OS02G0209300	Exonic	3UTR	NA	LOC_Os02g11859	expressed protein
Chr.2	6717760	6717760	T	T	C	SNP	OS02G0220500	Intronic	NA	NA	LOC_Os02g12794	elongation factor 1-gamma, putative, expressed
Chr.2	7224452	7224452	G	G	C	SNP	OS02G0228300	Exonic	5UTR	NA	LOC_Os02g13510	receptor-like protein kinase 5 precursor, putative, expressed
Chr.2	7222536	7222536	G	G	C	SNP	OS02G0228300	Exonic	CDS	Silent	LOC_Os02g13510	receptor-like protein kinase 5 precursor, putative, expressed
Chr.2	7223583	7223583	A	A	C	SNP	OS02G0228300	Exonic	CDS	Silent	LOC_Os02g13510	receptor-like protein kinase 5 precursor, putative, expressed
Chr.2	7223772	7223772	T	T	C	SNP	OS02G0228300	Exonic	CDS	Silent	LOC_Os02g13510	receptor-like protein kinase 5 precursor, putative, expressed
Chr.2	7221940	7221940	G	G	C	SNP	OS02G0228300	Exonic	CDS	Missense	LOC_Os02g13510	receptor-like protein kinase 5 precursor, putative, expressed
Chr.2	7222043	7222043	T	T	C	SNP	OS02G0228300	Exonic	CDS	Missense	LOC_Os02g13510	receptor-like protein kinase 5 precursor, putative, expressed
Chr.2	7222459	7222459	T	T	C	SNP	OS02G0228300	Exonic	CDS	Missense	LOC_Os02g13510	receptor-like protein kinase 5 precursor, putative, expressed
Chr.2	7222549	7222549	T	T	C	SNP	OS02G0228300	Exonic	CDS	Missense	LOC_Os02g13510	receptor-like protein kinase 5 precursor, putative, expressed

Chr.2	7222567	7222567	T	T	C	SNP	OS02G0228300	Exonic	CDS	Missense	LOC_Os02g13510	receptor-like protein kinase 5 precursor, putative, expressed
Chr.2	7222571	7222571	T	T	C	SNP	OS02G0228300	Exonic	CDS	Missense	LOC_Os02g13510	receptor-like protein kinase 5 precursor, putative, expressed
Chr.2	7223015	7223015	T	T	C	SNP	OS02G0228300	Exonic	CDS	Missense	LOC_Os02g13510	receptor-like protein kinase 5 precursor, putative, expressed
Chr.2	7223582	7223582	T	T	C	SNP	OS02G0228300	Exonic	CDS	Missense	LOC_Os02g13510	receptor-like protein kinase 5 precursor, putative, expressed
Chr.2	7223665	7223665	T	T	C	SNP	OS02G0228300	Exonic	CDS	Missense	LOC_Os02g13510	receptor-like protein kinase 5 precursor, putative, expressed
Chr.2	7223945	7223945	T	T	C	SNP	OS02G0228300	Exonic	CDS	Missense	LOC_Os02g13510	receptor-like protein kinase 5 precursor, putative, expressed
Chr.2	9651444	9651444	T	T	C	SNP	OS02G0269600	Exonic	CDS	Silent	LOC_Os02g16940	OsSub13 - Putative Subtilisin homologue, expressed
Chr.2	9925098	9925098	T	T	C	SNP	OS02G0272900	Intronic	NA	NA	LOC_Os02g17304	resistance protein, putative, expressed
Chr.2	9976863	9976863	T	T	C	SNP	OS02G0273700	Intronic	NA	NA	LOC_Os02g17350	VHS and GAT domain containing protein, expressed
Chr.2	9990363	9990363	T	T	C	SNP	OS02G0273900	Exonic	CDS	Missense	LOC_Os02g17370	expressed protein
Chr.2	17531339	17531339	T	T	C	SNP	OS02G0498300	Intronic	NA	NA	LOC_Os02g29510	DUF803 domain containing, putative, expressed
Chr.2	21843323	21843323	T	T	C	SNP	OS02G0570900	Exonic	CDS	Silent	LOC_Os02g36210	ent-kaurene synthase, chloroplast precursor, putative, expressed
Chr.2	21934754	21934754	T	T	C	SNP	OS02G0572400	Intronic	NA	NA	LOC_Os02g36340	riboflavin biosynthesis protein ribAB, chloroplast precursor, putative, expressed
Chr.2	24904684	24904684	T	T	C	SNP	OS02G0624400	Intronic	NA	NA	LOC_Os02g41520	glycosyl transferase 8 domain containing protein, putative, expressed
Chr.2	28803071	28803071	T	T	C	SNP	OS02G0700100	Exonic	5UTR	NA	LOC_Os02g47180	WD repeat-containing protein, putative, expressed
Chr.2	30458937	30458937	T	T	C	SNP	OS02G0731200	Intronic	NA	NA	LOC_Os02g49840	OsMADS57 - MADS-box family gene with MIKCc type-box, expressed
Chr.2	34614686	34614686	T	T	C	SNP	OS02G0809800	Exonic	3UTR	NA	LOC_Os02g56510	phosphate transporter 1, putative, expressed
Chr.2	34610979	34610979	G	G	C	SNP	OS02G0809800	Exonic	CDS	Missense	LOC_Os02g56510	phosphate transporter 1, putative, expressed
Chr.2	34675633	34675633	T	T	C	SNP	OS02G0810900	Exonic	CDS	Silent	LOC_Os02g56600	no apical meristem protein, putative, expressed
Chr.2	35098804	35098804	A	A	C	SNP	OS02G0817900	Exonic	CDS	Silent	LOC_Os02g57290	cytochrome P450, putative, expressed
Chr.2	35097921	35097921	A	A	C	SNP	OS02G0817900	Intronic	NA	NA	LOC_Os02g57290	cytochrome P450, putative, expressed
Chr.2	35097943	35097943	T	T	C	SNP	OS02G0817900	Intronic	NA	NA	LOC_Os02g57290	cytochrome P450, putative, expressed
Chr.2	35097957	35097957	T	T	C	SNP	OS02G0817900	Intronic	NA	NA	LOC_Os02g57290	cytochrome P450, putative, expressed
Chr.2	35098610	35098610	T	T	C	SNP	OS02G0817900	Intronic	NA	NA	LOC_Os02g57290	cytochrome P450, putative, expressed
Chr.2	35098870	35098870	A	A	C	SNP	OS02G0817900	Intronic	NA	NA	LOC_Os02g57290	cytochrome P450, putative, expressed
Chr.2	35098887	35098887	G	G	C	SNP	OS02G0817900	Intronic	NA	NA	LOC_Os02g57290	cytochrome P450, putative, expressed
Chr.2	82873	82884	CTACTACTAGTA	CTACTACTAGTA	C	INDEL	OS02G0101500	Exonic	5UTR	NA	LOC_Os02g01150	erythronate-4-phosphate dehydrogenase domain containing protein, expressed
Chr.2	80376	80380	CACAG	CACAG	C	INDEL	OS02G0101500	Intronic	NA	NA	LOC_Os02g01150	erythronate-4-phosphate dehydrogenase domain containing protein, expressed
Chr.2	82012	82020	CACATATAT	CACATATAT	C	INDEL	OS02G0101500	Intronic	NA	NA	LOC_Os02g01150	erythronate-4-phosphate dehydrogenase domain containing protein, expressed
Chr.2	82223	82233	CATGTGTACGT	CATGTGTACGT	C	INDEL	OS02G0101500	Intronic	NA	NA	LOC_Os02g01150	erythronate-4-phosphate dehydrogenase domain containing protein, expressed
Chr.2	82614	82632	CTCTCCACTACTAGTGTAA	CTCTCCACTACTAGTGTAA	C	INDEL	OS02G0101500	Intronic	NA	NA	LOC_Os02g01150	erythronate-4-phosphate dehydrogenase domain containing protein, expressed
Chr.2	183725	183735	CATCAGATGAG	CATCAGATGAG	C	INDEL	OS02G0103800	Exonic	3UTR	NA	LOC_Os02g01340	ferredoxin--NADP reductase, chloroplast precursor, putative, expressed
Chr.2	5551740	5551741	CT	CT	C	INDEL	OS02G0198900	Intronic	NA	NA	LOC_Os02g10540	ribosomal protein L7/L12 C-terminal domain containing protein, expressed
Chr.2	6719469	6719471	CAG	CAG	C	INDEL	OS02G0220500	Intronic	NA	NA	LOC_Os02g12794	elongation factor 1-gamma, putative, expressed
Chr.2	6835364	6835365	CA	CA	C	INDEL	OS02G0222500	Intronic	NA	NA	LOC_Os02g12939	expressed protein
Chr.2	9972895	9972900	CTCTCT	CTCTCT	C	INDEL	OS02G0273700	Intronic	NA	NA	LOC_Os02g17350	VHS and GAT domain containing protein, expressed
Chr.2	9973946	9973947	CT	CT	C	INDEL	OS02G0273700	Intronic	NA	NA	LOC_Os02g17350	VHS and GAT domain containing protein, expressed
Chr.2	18581258	18581262	CAAAA	CAAAA	C	INDEL	OS02G0515200	Exonic	3UTR	NA	LOC_Os02g31080	hydroxyproline-rich glycoprotein family protein, putative, expressed
Chr.2	22687466	22687477	CTCGCCTCCGGT	CTCGCCTCCGGT	C	INDEL	OS02G0588500	Exonic	5UTR	NA	LOC_Os02g37590	glycerophosphoryl diester phosphodiesterase family protein, putative, expressed
Chr.2	34227202	34227204	CTT	CTT	C	INDEL	OS02G0802700	Exonic	3UTR	NA	LOC_Os02g55910	monogalactosyldiacylglycerol synthase, putative, expressed
Chr.2	35081617	35081619	CAG	CAG	C	INDEL	OS02G0817700	Intronic	NA	NA	LOC_Os02g57260	3-ketoacyl-CoA thiolase, peroxisomal precursor, putative, expressed
Chr.2	35097946	35097947	CA	CA	C	INDEL	OS02G0817900	Intronic	NA	NA	LOC_Os02g57290	cytochrome P450, putative, expressed
Chr.2	35098441	35098442	CA	CA	C	INDEL	OS02G0817900	Intronic	NA	NA	LOC_Os02g57290	cytochrome P450, putative, expressed
Chr.2	35561541	35561549	CTCCTTCCG	CTCCTTCCG	C	INDEL	OS02G0827400	Exonic	3UTR	NA	LOC_Os02g58100	expressed protein
Chr.12	3130132	3130132	T	T	C	SNP	OS12G0162100	Exonic	CDS	Missense	LOC_Os12g06490	STE_PAK_Ste20_Slob_Wnk.6 - STE kinases include homologs to sterile 7, sterile 11 and sterile 20 from yeast, expressed
Chr.12	9918063	9918063	T	T	C	SNP	OS12G0271700	Intronic	NA	NA	LOC_Os12g17320	polyprenyl synthetase, putative, expressed
Chr.12	9918068	9918068	T	T	C	SNP	OS12G0271700	Intronic	NA	NA	LOC_Os12g17320	polyprenyl synthetase, putative, expressed
Chr.12	9918954	9918954	T	T	C	SNP	OS12G0271700	Intronic	NA	NA	LOC_Os12g17320	polyprenyl synthetase, putative, expressed
Chr.12	12987889	12987889	T	T	C	SNP	OS12G0418600	Intronic	NA	NA	LOC_Os12g22970	expressed protein
Chr.12	17574650	17574650	T	T	C	SNP	OS12G0479400	Exonic	CDS	Silent	LOC_Os12g29520	auxin response factor, putative, expressed
Chr.12	17576464	17576464	T	T	C	SNP	OS12G0479400	Exonic	CDS	Missense	LOC_Os12g29520	auxin response factor, putative, expressed
Chr.12	25519213	25519213	T	T	C	SNP	OS12G0604700	Intronic	NA	NA	LOC_Os12g41180	LSTK-1-like kinase, putative, expressed
Chr.12	25651830	25651830	T	T	C	SNP	OS12G0607100	Exonic	3UTR	NA	LOC_Os12g41400	eukaryotic translation initiation factor 2 subunit gamma, putative, expressed
Chr.12	25649757	25649757	T	T	C	SNP	OS12G0607100	Exonic	CDS	Silent	LOC_Os12g41400	eukaryotic translation initiation factor 2 subunit gamma, putative, expressed
Chr.12	25651420	25651420	T	T	C	SNP	OS12G0607100	Exonic	CDS	Silent	LOC_Os12g41400	eukaryotic translation initiation factor 2 subunit gamma, putative, expressed
Chr.12	25651727	25651727	T	T	C	SNP	OS12G0607100	Exonic	CDS	Silent	LOC_Os12g41400	eukaryotic translation initiation factor 2 subunit gamma, putative, expressed
Chr.12	25649330	25649330	T	T	C	SNP	OS12G0607100	Intronic	NA	NA	LOC_Os12g41400	eukaryotic translation initiation factor 2 subunit gamma, putative, expressed
Chr.12	25649624	25649624	T	T	C	SNP	OS12G0607100	Intronic	NA	NA	LOC_Os12g41400	eukaryotic translation initiation factor 2 subunit gamma, putative, expressed
Chr.12	25651462	25651462	T	T	C	SNP	OS12G0607100	Intronic	NA	NA	LOC_Os12g41400	eukaryotic translation initiation factor 2 subunit gamma, putative, expressed
Chr.12	9353004	9353004	G	G	C	SNP	OS12G0264500	Intronic	NA	NA	LOC_Os12g16350	enoyl-CoA hydratase/isomerase family protein, putative, expressed
Chr.12	12987863	12987863	G	G	C	SNP	OS12G0418600	Intronic	NA	NA	LOC_Os12g22970	expressed protein
Chr.12	23420644	23420644	G	G	C	SNP	OS12G0569200	Exonic	3UTR	NA	LOC_Os12g38140	expressed protein
Chr.12	23806568	23806568	T	T	C	SNP	OS12G0576600	Intronic	NA	NA	LOC_Os12g38750	nucleotide pyrophosphatase/phosphodiesterase, putative, expressed
Chr.12	25326426	25326428	CTA	CTA	C	INDEL	OS12G0601400	Intronic	NA	NA	LOC_Os12g40900	OsIAA31 - Auxin-responsive Aux/IAA gene family member, expressed
Chr.12	9917729	9917729	A	A	C	SNP	OS12G0271700	Intronic	NA	NA	LOC_Os12g17320	polyprenyl synthetase, putative, expressed
Chr.12	22531100	22531100	A	A	C	SNP	OS12G0554500	Exonic	CDS	Missense	LOC_Os12g36770	lipase class 3 family protein, putative, expressed
Chr.12	22592465	22592465	A	A	C	SNP	OS12G0555200	Intronic	NA	NA	LOC_Os12g36850	pathogenesis-related Bet v I family protein, putative, expressed
Chr.12	25651480	25651480	A	A	C	SNP	OS12G0607100	Intronic	NA	NA	LOC_Os12g41400	eukaryotic translation initiation factor 2 subunit gamma, putative, expressed
Chr.12	9918458	9918458	T	T	C	SNP	OS12G0271700	Intronic	NA	NA	LOC_Os12g17320	polyprenyl synthetase, putative, expressed
Chr.12	9918749	9918749	G	G	C	SNP	OS12G0271700	Intronic	NA	NA	LOC_Os12g17320	polyprenyl synthetase, putative, expressed
Chr.12	9918751	9918751	G	G	C	SNP	OS12G0271700	Intronic	NA	NA	LOC_Os12g17320	polyprenyl synthetase, putative, expressed
Chr.12	12987653	12987653	T	T	C	SNP	OS12G0418600	Intronic	NA	NA	LOC_Os12g22970	expressed protein
Chr.12	23420473	23420473	G	G	C	SNP	OS12G0569200	Exonic	CDS	Silent	LOC_Os12g38140	expressed protein
Chr.12	23420343	23420343	T	T	C	SNP	OS12G0569200	Exonic	CDS	Missense	LOC_Os12g38140	expressed protein
Chr.12	24872130	24872130	G	G	C	SNP	OS12G0592900	Exonic	3UTR	NA	LOC_Os12g40180	expressed protein
Chr.12	25445320	25445320	T	T	C	SNP	OS12G0603700	Exonic	CDS	Missense	LOC_Os12g41090	CAMK_KIN1/SNF1/Nim1_like.38 - CAMK includes calcium/calmodulin depedent protein kinases, expressed
Chr.12	25445665	25445665	G	G	C	SNP	OS12G0603700	Exonic	CDS	Missense	LOC_Os12g41090	CAMK_KIN1/SNF1/Nim1_like.38 - CAMK includes calcium/calmodulin depedent protein kinases, expressed
Chr.12	25651628	25651628	T	T	C	SNP	OS12G0607100	Exonic	CDS	Silent	LOC_Os12g41400	eukaryotic translation initiation factor 2 subunit gamma, putative, expressed
Chr.12	25651796	25651796	T	T	C	SNP	OS12G0607100	Exonic	CDS	Silent	LOC_Os12g41400	eukaryotic translation initiation factor 2 subunit gamma, putative, expressed
Chr.12	26780504	26780504	A	A	C	SNP	OS12G0626400	Exonic	3UTR	NA	LOC_Os12g43130	phytoene synthase, chloroplast precursor, putative, expressed
Chr.12	23502151	23502153	CTG	CTG	C	INDEL	OS12G0570700	Exonic	3UTR	NA	LOC_Os12g38270	metallothionein, putative, expressed
Chr.11	651903	651903	T	T	C	SNP	OS11G0114300	Exonic	3UTR	NA	LOC_Os11g02290	expressed protein
Chr.11	2424474	2424474	T	T	C	SNP	OS11G0151700	Intronic	NA	NA	LOC_Os11g05400	Ser/Thr protein phosphatase family protein, putative, expressed
Chr.11	2759954	2759954	T	T	C	SNP	OS11G0157400	Exonic	3UTR	NA	LOC_Os11g05880	exo70 exocyst complex subunit, putative, expressed
Chr.11	2760044	2760044	T	T	C	SNP	OS11G0157400	Exonic	3UTR	NA	LOC_Os11g05880	exo70 exocyst complex subunit, putative, expressed

Chr.11	4251449	4251449	T	T	C	SNP	OS11G0183900	Exonic	3UTR	NA	LOC_Os11g08100	eukaryotic aspartyl protease domain containing protein, expressed
Chr.11	4249317	4249317	T	T	C	SNP	OS11G0183900	Exonic	CDS	Silent	LOC_Os11g08100	eukaryotic aspartyl protease domain containing protein, expressed
Chr.11	4249367	4249367	T	T	C	SNP	OS11G0183900	Exonic	CDS	Missense	LOC_Os11g08100	eukaryotic aspartyl protease domain containing protein, expressed
Chr.11	4249717	4249717	T	T	C	SNP	OS11G0183900	Exonic	CDS	Missense	LOC_Os11g08100	eukaryotic aspartyl protease domain containing protein, expressed
Chr.11	4247782	4247782	T	T	C	SNP	OS11G0183900	Intronic	NA	NA	LOC_Os11g08100	eukaryotic aspartyl protease domain containing protein, expressed
Chr.11	4247998	4247998	T	T	C	SNP	OS11G0183900	Intronic	NA	NA	LOC_Os11g08100	eukaryotic aspartyl protease domain containing protein, expressed
Chr.11	4249476	4249476	T	T	C	SNP	OS11G0183900	Intronic	NA	NA	LOC_Os11g08100	eukaryotic aspartyl protease domain containing protein, expressed
Chr.11	4249959	4249959	T	T	C	SNP	OS11G0183900	Intronic	NA	NA	LOC_Os11g08100	eukaryotic aspartyl protease domain containing protein, expressed
Chr.11	4250004	4250004	T	T	C	SNP	OS11G0183900	Intronic	NA	NA	LOC_Os11g08100	eukaryotic aspartyl protease domain containing protein, expressed
Chr.11	5565843	5565843	T	T	C	SNP	OS11G0208600	Exonic	3UTR	NA	LOC_Os11g10250	expressed protein
Chr.11	5567186	5567186	T	T	C	SNP	OS11G0208600	Intronic	NA	NA	LOC_Os11g10250	expressed protein
Chr.11	6321887	6321887	T	T	C	SNP	OS11G0220800	Intronic	NA	NA	LOC_Os11g11390	ribosomal protein, putative, expressed
Chr.11	6639331	6639331	T	T	C	SNP	OS11G0226933	Exonic	CDS	NA	LOC_Os11g11960	disease resistance protein RPM1, putative, expressed
Chr.11	6639457	6639457	T	T	C	SNP	OS11G0226933	Exonic	CDS	NA	LOC_Os11g11960	disease resistance protein RPM1, putative, expressed
Chr.11	6639458	6639458	T	T	C	SNP	OS11G0226933	Exonic	CDS	NA	LOC_Os11g11960	disease resistance protein RPM1, putative, expressed
Chr.11	6640312	6640312	T	T	C	SNP	OS11G0226933	Exonic	CDS	NA	LOC_Os11g11960	disease resistance protein RPM1, putative, expressed
Chr.11	6650116	6650116	T	T	C	SNP	OS11G0227000	Exonic	3UTR	NA	LOC_Os11g11980	expressed protein
Chr.11	6650228	6650228	T	T	C	SNP	OS11G0227000	Exonic	3UTR	NA	LOC_Os11g11980	expressed protein
Chr.11	6649811	6649811	T	T	C	SNP	OS11G0227000	Exonic	CDS	Missense	LOC_Os11g11980	expressed protein
Chr.11	6649708	6649708	T	T	C	SNP	OS11G0227000	Intronic	NA	NA	LOC_Os11g11980	expressed protein
Chr.11	7127276	7127276	T	T	C	SNP	OS11G0234200	Exonic	CDS	Missense	LOC_Os11g12650	PHD-finger domain containing protein, putative, expressed
Chr.11	7939327	7939327	T	T	C	SNP	OS11G0247000	Exonic	5UTR	NA	LOC_Os11g14190	ARGOS, putative, expressed
Chr.11	9057696	9057696	T	T	C	SNP	OS11G0265200	Intronic	NA	NA	LOC_Os11g16390	expressed protein
Chr.11	10143495	10143495	T	T	C	SNP	OS11G0283500	Exonic	CDS	Silent	LOC_Os11g18044	peptide transporter PTR2, putative, expressed
Chr.11	10140360	10140360	T	T	C	SNP	OS11G0283500	Intronic	NA	NA	LOC_Os11g18044	peptide transporter PTR2, putative, expressed
Chr.11	10142580	10142580	T	T	C	SNP	OS11G0283500	Intronic	NA	NA	LOC_Os11g18044	peptide transporter PTR2, putative, expressed
Chr.11	14049176	14049176	T	T	C	SNP	OS11G0434000	Exonic	CDS	Missense	LOC_Os11g24630	magnesium-dependent phosphatase 1, putative, expressed
Chr.11	14049247	14049247	T	T	C	SNP	OS11G0434000	Intronic	NA	NA	LOC_Os11g24630	magnesium-dependent phosphatase 1, putative, expressed
Chr.11	14049258	14049258	T	T	C	SNP	OS11G0434000	Intronic	NA	NA	LOC_Os11g24630	magnesium-dependent phosphatase 1, putative, expressed
Chr.11	14049640	14049640	T	T	C	SNP	OS11G0434000	Intronic	NA	NA	LOC_Os11g24630	magnesium-dependent phosphatase 1, putative, expressed
Chr.11	14051249	14051249	T	T	C	SNP	OS11G0434000	Intronic	NA	NA	LOC_Os11g24630	magnesium-dependent phosphatase 1, putative, expressed
Chr.11	14051320	14051320	T	T	C	SNP	OS11G0434000	Intronic	NA	NA	LOC_Os11g24630	magnesium-dependent phosphatase 1, putative, expressed
Chr.11	15421435	15421435	T	T	C	SNP	OS11G0455500	Intronic	NA	NA	LOC_Os11g26850	erythronate-4-phosphate dehydrogenase, putative, expressed
Chr.11	17235194	17235194	T	T	C	SNP	OS11G0489250	Exonic	CDS	Missense	LOC_Os11g29720	cytochrome P450, putative, expressed
Chr.11	17352285	17352285	T	T	C	SNP	OS11G0490900	Exonic	5UTR	NA	LOC_Os11g29870	WRKY72, expressed
Chr.11	17352853	17352853	T	T	C	SNP	OS11G0490900	Intronic	NA	NA	LOC_Os11g29870	WRKY72, expressed
Chr.11	17353555	17353555	T	T	C	SNP	OS11G0490900	Intronic	NA	NA	LOC_Os11g29870	WRKY72, expressed
Chr.11	17353805	17353805	T	T	C	SNP	OS11G0490900	Intronic	NA	NA	LOC_Os11g29870	WRKY72, expressed
Chr.11	17354736	17354736	T	T	C	SNP	OS11G0490900	Intronic	NA	NA	LOC_Os11g29870	WRKY72, expressed
Chr.11	17354981	17354981	T	T	C	SNP	OS11G0490900	Intronic	NA	NA	LOC_Os11g29870	WRKY72, expressed
Chr.11	17354985	17354985	T	T	C	SNP	OS11G0490900	Intronic	NA	NA	LOC_Os11g29870	WRKY72, expressed
Chr.11	18938484	18938484	T	T	C	SNP	OS11G0523800	Exonic	CDS	Missense	LOC_Os11g32110	auxin response factor, putative, expressed
Chr.11	18939965	18939965	T	T	C	SNP	OS11G0523800	Exonic	CDS	Missense	LOC_Os11g32110	auxin response factor, putative, expressed
Chr.11	18939786	18939786	T	T	C	SNP	OS11G0523800	Intronic	NA	NA	LOC_Os11g32110	auxin response factor, putative, expressed
Chr.11	25424004	25424004	T	T	C	SNP	OS11G0641500	Exonic	CDS	Silent	LOC_Os11g42200	laccase precursor protein, putative, expressed
Chr.11	25424997	25424997	T	T	C	SNP	OS11G0641500	Exonic	CDS	Silent	LOC_Os11g42200	laccase precursor protein, putative, expressed
Chr.11	4972301	4972301	G	G	C	SNP	OS11G0199200	Intronic	NA	NA	LOC_Os11g09280	OsPDIL1-1 protein disulfide isomerase PDIL1-1, expressed
Chr.11	4974372	4974372	G	G	C	SNP	OS11G0199200	Intronic	NA	NA	LOC_Os11g09280	OsPDIL1-1 protein disulfide isomerase PDIL1-1, expressed
Chr.11	6639414	6639414	G	G	C	SNP	OS11G0226933	Exonic	CDS	NA	LOC_Os11g11960	disease resistance protein RPM1, putative, expressed
Chr.11	9058193	9058193	G	G	C	SNP	OS11G0265200	Intronic	NA	NA	LOC_Os11g16390	expressed protein
Chr.11	9188208	9188208	G	G	C	SNP	OS11G0267300	Intronic	NA	NA	LOC_Os11g16580	endonuclease III-like protein 1, putative, expressed
Chr.11	14051003	14051003	G	G	C	SNP	OS11G0434000	Intronic	NA	NA	LOC_Os11g24630	magnesium-dependent phosphatase 1, putative, expressed
Chr.11	17355379	17355379	G	G	C	SNP	OS11G0490900	Exonic	CDS	Silent	LOC_Os11g29870	WRKY72, expressed
Chr.11	17354628	17354628	G	G	C	SNP	OS11G0490900	Intronic	NA	NA	LOC_Os11g29870	WRKY72, expressed
Chr.11	26513110	26513110	G	G	C	SNP	OS11G0660500	Exonic	5UTR	NA	LOC_Os11g43900	translationally-controlled tumor protein, putative, expressed
Chr.11	28853667	28853667	G	G	C	SNP	OS11G0704800	Exonic	3UTR	NA	LOC_Os11g47840	OsRhmbd18 - Putative Rhomboid homologue, expressed
Chr.11	762068	762068	T	T	C	SNP	OS11G0116900	Intronic	NA	NA	LOC_Os11g02480	WRKY46, expressed
Chr.11	762487	762487	T	T	C	SNP	OS11G0116900	Intronic	NA	NA	LOC_Os11g02480	WRKY46, expressed
Chr.11	762516	762516	T	T	C	SNP	OS11G0116900	Intronic	NA	NA	LOC_Os11g02480	WRKY46, expressed
Chr.11	2423840	2423840	T	T	C	SNP	OS11G0151700	Exonic	3UTR	NA	LOC_Os11g05400	Ser/Thr protein phosphatase family protein, putative, expressed
Chr.11	2424313	2424313	T	T	C	SNP	OS11G0151700	Intronic	NA	NA	LOC_Os11g05400	Ser/Thr protein phosphatase family protein, putative, expressed
Chr.11	2424373	2424373	T	T	C	SNP	OS11G0151700	Intronic	NA	NA	LOC_Os11g05400	Ser/Thr protein phosphatase family protein, putative, expressed
Chr.11	2424402	2424402	T	T	C	SNP	OS11G0151700	Intronic	NA	NA	LOC_Os11g05400	Ser/Thr protein phosphatase family protein, putative, expressed
Chr.11	2607590	2607590	T	T	C	SNP	OS11G0155600	Exonic	3UTR	NA	LOC_Os11g05700	ABC transporter family protein, putative, expressed
Chr.11	8389546	8389546	T	T	C	SNP	OS11G0255500	Exonic	CDS	Silent	LOC_Os11g14910	NADP-dependent oxidoreductase, putative, expressed
Chr.11	8388311	8388311	T	T	C	SNP	OS11G0255500	Exonic	CDS	Missense	LOC_Os11g14910	NADP-dependent oxidoreductase, putative, expressed
Chr.11	8389572	8389572	T	T	C	SNP	OS11G0255500	Exonic	CDS	Missense	LOC_Os11g14910	NADP-dependent oxidoreductase, putative, expressed
Chr.11	8389667	8389667	T	T	C	SNP	OS11G0255500	Exonic	CDS	Missense	LOC_Os11g14910	NADP-dependent oxidoreductase, putative, expressed
Chr.11	8389004	8389004	T	T	C	SNP	OS11G0255500	Intronic	NA	NA	LOC_Os11g14910	NADP-dependent oxidoreductase, putative, expressed
Chr.11	8389196	8389196	T	T	C	SNP	OS11G0255500	Intronic	NA	NA	LOC_Os11g14910	NADP-dependent oxidoreductase, putative, expressed
Chr.11	8389262	8389262	T	T	C	SNP	OS11G0255500	Intronic	NA	NA	LOC_Os11g14910	NADP-dependent oxidoreductase, putative, expressed
Chr.11	8389370	8389370	T	T	C	SNP	OS11G0255500	Intronic	NA	NA	LOC_Os11g14910	NADP-dependent oxidoreductase, putative, expressed
Chr.11	8444866	8444866	T	T	C	SNP	OS11G0256200	Intronic	NA	NA	LOC_Os11g14990	expressed protein
Chr.11	21464289	21464289	T	T	C	SNP	OS11G0572200	Exonic	CDS	Silent	LOC_Os11g36400	expressed protein
Chr.11	22177354	22177354	T	T	C	SNP	OS11G0586300	Exonic	CDS	NA	LOC_Os11g37560	expressed protein
Chr.11	10865334	10865334	G	G	C	SNP	OS11G0295900	Intronic	NA	NA	LOC_Os11g19060	BABY BOOM 1, putative, expressed
Chr.11	19676125	19676125	A	A	C	SNP	OS11G0539200	Intronic	NA	NA	LOC_Os11g33270	glycosyl hydrolases family 16, putative, expressed
Chr.11	24652377	24652377	A	A	C	SNP	OS11G0629400	Intronic	NA	NA	LOC_Os11g41150	nitrilase-associated protein, putative, expressed
Chr.11	8444813	8444814	CA	CA	C	INDEL	OS11G0256200	Intronic	NA	NA	LOC_Os11g14990	expressed protein
Chr.11	17911163	17911166	CCCT	CCCT	C	INDEL	OS11G0502700	Exonic	5UTR	NA	LOC_Os11g30760	expressed protein
Chr.11	2763916	2763916	A	A	C	SNP	OS11G0157400	Intronic	NA	NA	LOC_Os11g05880	exo70 exocyst complex subunit, putative, expressed
Chr.11	4249302	4249302	A	A	C	SNP	OS11G0183900	Exonic	CDS	Silent	LOC_Os11g08100	eukaryotic aspartyl protease domain containing protein, expressed
Chr.11	6638568	6638568	A	A	C	SNP	OS11G0226933	Exonic	CDS	NA	LOC_Os11g11960	disease resistance protein RPM1, putative, expressed
Chr.11	6639440	6639440	A	A	C	SNP	OS11G0226933	Exonic	CDS	NA	LOC_Os11g11960	disease resistance protein RPM1, putative, expressed
Chr.11	6639571	6639571	A	A	C	SNP	OS11G0226933	Exonic	CDS	NA	LOC_Os11g11960	disease resistance protein RPM1, putative, expressed

Chr.11	7125575	7125575	A	A	C	SNP	OS11G0234200	Exonic	CDS	Silent	LOC_Os11g12650	PHD-finger domain containing protein, putative, expressed
Chr.11	8389344	8389344	A	A	C	SNP	OS11G0255500	Intronic	NA	NA	LOC_Os11g14910	NADP-dependent oxidoreductase, putative, expressed
Chr.11	10140565	10140565	A	A	C	SNP	OS11G0283500	Intronic	NA	NA	LOC_Os11g18044	peptide transporter PTR2, putative, expressed
Chr.11	10142830	10142830	A	A	C	SNP	OS11G0283500	Intronic	NA	NA	LOC_Os11g18044	peptide transporter PTR2, putative, expressed
Chr.11	14050903	14050903	A	A	C	SNP	OS11G0434000	Intronic	NA	NA	LOC_Os11g24630	magnesium-dependent phosphatase 1, putative, expressed
Chr.11	17353611	17353611	A	A	C	SNP	OS11G0490900	Intronic	NA	NA	LOC_Os11g29870	WRKY72, expressed
Chr.11	17354956	17354956	A	A	C	SNP	OS11G0490900	Intronic	NA	NA	LOC_Os11g29870	WRKY72, expressed
Chr.11	23080674	23080674	A	A	C	SNP	OS11G0600900	Intronic	NA	NA	LOC_Os11g38810	mannose-6-phosphate isomerase, putative, expressed
Chr.11	23080808	23080808	A	A	C	SNP	OS11G0600900	Intronic	NA	NA	LOC_Os11g38810	mannose-6-phosphate isomerase, putative, expressed
Chr.11	761981	761981	T	T	C	SNP	OS11G0116900	Intronic	NA	NA	LOC_Os11g02480	WRKY46, expressed
Chr.11	2424695	2424695	G	G	C	SNP	OS11G0151700	Exonic	CDS	NA	LOC_Os11g05400	Ser/Thr protein phosphatase family protein, putative, expressed
Chr.11	2426126	2426126	T	T	C	SNP	OS11G0151700	Exonic	CDS	NA	LOC_Os11g05400	Ser/Thr protein phosphatase family protein, putative, expressed
Chr.11	4247493	4247493	G	G	C	SNP	OS11G0183900	Exonic	CDS	Silent	LOC_Os11g08100	eukaryotic aspartyl protease domain containing protein, expressed
Chr.11	4247589	4247589	T	T	C	SNP	OS11G0183900	Exonic	CDS	Silent	LOC_Os11g08100	eukaryotic aspartyl protease domain containing protein, expressed
Chr.11	6649898	6649898	T	T	C	SNP	OS11G0227000	Exonic	3UTR	NA	LOC_Os11g11980	expressed protein
Chr.11	6650092	6650092	A	A	C	SNP	OS11G0227000	Exonic	3UTR	NA	LOC_Os11g11980	expressed protein
Chr.11	6649797	6649797	G	G	C	SNP	OS11G0227000	Exonic	CDS	Missense	LOC_Os11g11980	expressed protein
Chr.11	6649408	6649408	T	T	C	SNP	OS11G0227000	Intronic	NA	NA	LOC_Os11g11980	expressed protein
Chr.11	6649672	6649672	A	A	C	SNP	OS11G0227000	Intronic	NA	NA	LOC_Os11g11980	expressed protein
Chr.11	7128671	7128671	T	T	C	SNP	OS11G0234200	Exonic	CDS	Missense	LOC_Os11g12650	PHD-finger domain containing protein, putative, expressed
Chr.11	9187720	9187720	A	A	C	SNP	OS11G0267300	Exonic	5UTR	NA	LOC_Os11g16580	endonuclease III-like protein 1, putative, expressed
Chr.11	9187779	9187779	G	G	C	SNP	OS11G0267300	Exonic	5UTR	NA	LOC_Os11g16580	endonuclease III-like protein 1, putative, expressed
Chr.11	9188522	9188522	T	T	C	SNP	OS11G0267300	Intronic	NA	NA	LOC_Os11g16580	endonuclease III-like protein 1, putative, expressed
Chr.11	14052306	14052306	A	A	C	SNP	OS11G0434000	Exonic	CDS	Missense	LOC_Os11g24630	magnesium-dependent phosphatase 1, putative, expressed
Chr.11	14050746	14050746	T	T	C	SNP	OS11G0434000	Intronic	NA	NA	LOC_Os11g24630	magnesium-dependent phosphatase 1, putative, expressed
Chr.11	14051067	14051067	G	G	C	SNP	OS11G0434000	Intronic	NA	NA	LOC_Os11g24630	magnesium-dependent phosphatase 1, putative, expressed
Chr.11	15422335	15422335	G	G	C	SNP	OS11G0455500	Exonic	CDS	NA	LOC_Os11g26850	erythronate-4-phosphate dehydrogenase, putative, expressed
Chr.11	16938345	16938345	T	T	C	SNP	OS11G0482200	Intronic	NA	NA	LOC_Os11g29210	resistance-gene-interacting protein, putative, expressed
Chr.11	17284296	17284296	G	G	C	SNP	OS11G0490100	Exonic	CDS	Silent	LOC_Os11g29780	plant-specific domain TIGR01627 family protein, expressed
Chr.11	17284538	17284538	G	G	C	SNP	OS11G0490100	Exonic	CDS	Missense	LOC_Os11g29780	plant-specific domain TIGR01627 family protein, expressed
Chr.11	17352472	17352472	T	T	C	SNP	OS11G0490900	Exonic	CDS	Silent	LOC_Os11g29870	WRKY72, expressed
Chr.11	17352554	17352554	A	A	C	SNP	OS11G0490900	Exonic	CDS	Missense	LOC_Os11g29870	WRKY72, expressed
Chr.11	17352570	17352570	T	T	C	SNP	OS11G0490900	Exonic	CDS	Missense	LOC_Os11g29870	WRKY72, expressed
Chr.11	17352814	17352814	T	T	C	SNP	OS11G0490900	Intronic	NA	NA	LOC_Os11g29870	WRKY72, expressed
Chr.11	18941310	18941310	G	G	C	SNP	OS11G0523800	Intronic	NA	NA	LOC_Os11g32110	auxin response factor, putative, expressed
Chr.11	22176877	22176877	T	T	C	SNP	OS11G0586300	Exonic	CDS	NA	LOC_Os11g37560	expressed protein
Chr.11	22177712	22177712	T	T	C	SNP	OS11G0586300	Intronic	NA	NA	LOC_Os11g37560	expressed protein
Chr.11	22178194	22178194	A	A	C	SNP	OS11G0586300	Intronic	NA	NA	LOC_Os11g37560	expressed protein
Chr.11	25424088	25424088	T	T	C	SNP	OS11G0641500	Exonic	CDS	Silent	LOC_Os11g42200	laccase precursor protein, putative, expressed
Chr.11	26510888	26510888	T	T	C	SNP	OS11G0660500	Exonic	3UTR	NA	LOC_Os11g43900	translationally-controlled tumor protein, putative, expressed
Chr.11	27200408	27200408	G	G	C	SNP	OS11G0673100	Exonic	3UTR	NA	LOC_Os11g44940	seed maturation protein PM27, putative, expressed
Chr.11	27488306	27488306	T	T	C	SNP	OS11G0679700	Intronic	NA	NA	LOC_Os11g45400	glycerol-3-phosphate acyltransferase, putative, expressed
Chr.11	27488734	27488734	T	T	C	SNP	OS11G0679700	Intronic	NA	NA	LOC_Os11g45400	glycerol-3-phosphate acyltransferase, putative, expressed
Chr.11	27488787	27488787	T	T	C	SNP	OS11G0679700	Intronic	NA	NA	LOC_Os11g45400	glycerol-3-phosphate acyltransferase, putative, expressed
Chr.11	28766488	28766488	G	G	C	SNP	OS11G0702400	Exonic	CDS	Silent	LOC_Os11g47630	ZOS11-10 - C2H2 zinc finger protein, expressed
Chr.11	28954541	28954541	T	T	C	SNP	OS11G0707600	Intronic	NA	NA	LOC_Os11g48020	fatty acid hydroxylase, putative, expressed
Chr.11	4247369	4247371	CTA	CTA	C	INDEL	OS11G0183900	Exonic	5UTR	NA	LOC_Os11g08100	eukaryotic aspartyl protease domain containing protein, expressed
Chr.11	4247617	4247618	CT	CT	C	INDEL	OS11G0183900	Intronic	NA	NA	LOC_Os11g08100	eukaryotic aspartyl protease domain containing protein, expressed
Chr.11	4247898	4247899	CT	CT	C	INDEL	OS11G0183900	Intronic	NA	NA	LOC_Os11g08100	eukaryotic aspartyl protease domain containing protein, expressed
Chr.11	11594801	11594834	CGTGTAGCAAAACCGCTGAACAGAATAGGGAGAT	CGTGTAGCAAAACCGCTGAACAGAATAGGGAGAT	C	INDEL	OS11G0306400	Intronic	NA	NA	LOC_Os11g20090	O-methyltransferase, putative, expressed
Chr.11	17352877	17352879	CCT	CCT	C	INDEL	OS11G0490900	Intronic	NA	NA	LOC_Os11g29870	WRKY72, expressed
Chr.11	17353949	17353950	CT	CT	C	INDEL	OS11G0490900	Intronic	NA	NA	LOC_Os11g29870	WRKY72, expressed
Chr.11	18936775	18936777	CAT	CAT	C	INDEL	OS11G0523800	Intronic	NA	NA	LOC_Os11g32110	auxin response factor, putative, expressed
Chr.11	19676780	19676782	CAT	CAT	C	INDEL	OS11G0539200	Intronic	NA	NA	LOC_Os11g33270	glycosyl hydrolases family 16, putative, expressed
Chr.11	21298268	21298278	CTCAGCAGGCT	CTCAGCAGGCT	C	INDEL	OS11G0570000	Exonic	CDS	FrameShift	LOC_Os11g36200	receptor-like protein kinase 2 precursor, putative, expressed
Chr.11	23079733	23079735	CTG	CTG	C	INDEL	OS11G0600900	Intronic	NA	NA	LOC_Os11g38810	mannose-6-phosphate isomerase, putative, expressed
Chr.11	23909105	23909106	CT	CT	C	INDEL	OS11G0615000	Exonic	3UTR	NA	LOC_Os11g40080	lipin, N-terminal conserved region family protein, expressed
Chr.11	27489967	27489968	CA	CA	C	INDEL	OS11G0679700	Exonic	CDS	NA	LOC_Os11g45400	glycerol-3-phosphate acyltransferase, putative, expressed
Chr.10	20843585	20843585	T	T	C	SNP	OS10G0535800	Intronic	NA	NA	LOC_Os10g39100	uncharacterized Cys-rich domain containing protein, putative, expressed
Chr.10	19095296	19095296	T	T	C	SNP	OS10G0500600	Intronic	NA	NA	LOC_Os10g35710	expressed protein
Chr.10	18990626	18990626	T	T	C	SNP	OS10G0498000	Intronic	NA	NA	LOC_Os10g35490	hydrolase, alpha/beta fold family domain containing protein, expressed
Chr.10	15640244	15640244	T	T	C	SNP	OS10G0437200	Exonic	3UTR	NA	LOC_Os10g30100	3-5 exonuclease family protein, putative, expressed
Chr.10	15307517	15307517	T	T	C	SNP	OS10G0430200	Intronic	NA	NA	LOC_Os10g29470	dehydrogenase, putative, expressed
Chr.10	15306354	15306354	T	T	C	SNP	OS10G0430200	Intronic	NA	NA	LOC_Os10g29470	dehydrogenase, putative, expressed
Chr.10	7688937	7688937	T	T	C	SNP	OS10G0208500	Exonic	5UTR	NA	LOC_Os10g14180	expressed protein
Chr.10	6299722	6299722	T	T	C	SNP	OS10G0190900	Intronic	NA	NA	LOC_Os10g11354	MATE efflux family protein, putative, expressed
Chr.10	6276573	6276573	T	T	C	SNP	OS10G0190500	Exonic	5UTR	NA	LOC_Os10g11310	expressed protein
Chr.10	3755987	3755987	T	T	C	SNP	OS10G0159300	Exonic	5UTR	NA	LOC_Os10g07160	retrotransposon, putative, centromere-specific, expressed
Chr.10	3754753	3754753	T	T	C	SNP	OS10G0159300	Exonic	3UTR	NA	LOC_Os10g07160	retrotransposon, putative, centromere-specific, expressed
Chr.10	3753440	3753440	T	T	C	SNP	OS10G0159300	Exonic	3UTR	NA	LOC_Os10g07160	retrotransposon, putative, centromere-specific, expressed
Chr.10	19160953	19160953	G	G	C	SNP	OS10G0502400	Exonic	CDS	Missense	LOC_Os10g35840	shikimate/quinate 5-dehydrogenase, putative, expressed
Chr.10	15674622	15674622	G	G	C	SNP	OS10G0437600	Intronic	NA	NA	LOC_Os10g30156	starch synthase, putative, expressed
Chr.10	15639786	15639786	G	G	C	SNP	OS10G0437200	Intronic	NA	NA	LOC_Os10g30100	3-5 exonuclease family protein, putative, expressed
Chr.10	10295816	10295816	T	T	C	SNP	OS10G0344900	Exonic	3UTR	NA	LOC_Os10g20450	MATE efflux family protein, putative, expressed
Chr.10	10233187	10233187	A	A	C	SNP	OS10G0344000	Intronic	NA	NA	LOC_Os10g20350	MATE efflux family protein, putative, expressed
Chr.10	10232945	10232945	A	A	C	SNP	OS10G0344000	Intronic	NA	NA	LOC_Os10g20350	MATE efflux family protein, putative, expressed
Chr.10	12871621	12871621	A	A	C	SNP	OS10G0389000	Intronic	NA	NA	LOC_Os10g25010	OsCML8 - Calmodulin-related calcium sensor protein, expressed
Chr.10	2360689	2360689	A	A	C	SNP	OS10G0138100	Intronic	NA	NA	LOC_Os10g04860	aldehyde oxidase, putative, expressed
Chr.10	68622	68622	G	G	C	SNP	OS10G0100500	Intronic	NA	NA	LOC_Os10g01060	protein kinase family protein, putative, expressed
Chr.10	380137	380137	T	T	C	SNP	OS10G0104900	Intronic	NA	NA	LOC_Os10g01570	C-5 cytosine-specific DNA methylase, putative, expressed
Chr.10	2360438	2360438	A	A	C	SNP	OS10G0138100	Intronic	NA	NA	LOC_Os10g04860	aldehyde oxidase, putative, expressed
Chr.10	2913138	2913138	G	G	C	SNP	OS10G0148400	Exonic	CDS	Silent	LOC_Os10g05780	POT domain containing peptide transporter, putative, expressed
Chr.10	2913594	2913594	G	G	C	SNP	OS10G0148400	Exonic	CDS	Silent	LOC_Os10g05780	POT domain containing peptide transporter, putative, expressed
Chr.10	2913789	2913789	G	G	C	SNP	OS10G0148400	Exonic	CDS	Silent	LOC_Os10g05780	POT domain containing peptide transporter, putative, expressed
Chr.10	2914047	2914047	T	T	C	SNP	OS10G0148400	Exonic	CDS	Silent	LOC_Os10g05780	POT domain containing peptide transporter, putative, expressed

Chr.10	2912920	2912920	T	T	C	SNP	OS10G0148400	Exonic	CDS	Missense	LOC_Os10g05780	POT domain containing peptide transporter, putative, expressed
Chr.10	2913506	2913506	A	A	C	SNP	OS10G0148400	Exonic	CDS	Missense	LOC_Os10g05780	POT domain containing peptide transporter, putative, expressed
Chr.10	2914147	2914147	G	G	C	SNP	OS10G0148400	Exonic	CDS	Missense	LOC_Os10g05780	POT domain containing peptide transporter, putative, expressed
Chr.10	3753774	3753774	T	T	C	SNP	OS10G0159300	Exonic	3UTR	NA	LOC_Os10g07160	retrotransposon, putative, centromere-specific, expressed
Chr.10	3753829	3753829	G	G	C	SNP	OS10G0159300	Exonic	3UTR	NA	LOC_Os10g07160	retrotransposon, putative, centromere-specific, expressed
Chr.10	3756178	3756178	G	G	C	SNP	OS10G0159300	Exonic	5UTR	NA	LOC_Os10g07160	retrotransposon, putative, centromere-specific, expressed
Chr.10	3756188	3756188	T	T	C	SNP	OS10G0159300	Exonic	5UTR	NA	LOC_Os10g07160	retrotransposon, putative, centromere-specific, expressed
Chr.10	3756877	3756877	G	G	C	SNP	OS10G0159300	Intronic	NA	NA	LOC_Os10g07160	retrotransposon, putative, centromere-specific, expressed
Chr.10	10293599	10293599	A	A	C	SNP	OS10G0344900	Intronic	NA	NA	LOC_Os10g20450	MATE efflux family protein, putative, expressed
Chr.10	12968091	12968091	T	T	C	SNP	OS10G0390500	Exonic	5UTR	NA	LOC_Os10g25130	aminotransferase, classes I and II, domain containing protein, expressed
Chr.10	13070752	13070752	G	G	C	SNP	OS10G0392400	Exonic	CDS	Silent	LOC_Os10g25290	ZIM domain containing protein, putative, expressed
Chr.10	15306668	15306668	T	T	C	SNP	OS10G0430200	Intronic	NA	NA	LOC_Os10g29470	dehydrogenase, putative, expressed
Chr.10	15308069	15308069	A	A	C	SNP	OS10G0430200	Intronic	NA	NA	LOC_Os10g29470	dehydrogenase, putative, expressed
Chr.10	15673274	15673274	T	T	C	SNP	OS10G0437600	Exonic	5UTR	NA	LOC_Os10g30156	starch synthase, putative, expressed
Chr.10	18641451	18641451	T	T	C	SNP	OS10G0491000	Exonic	CDS	Silent	LOC_Os10g34930	secretory protein, putative, expressed
Chr.10	18641456	18641456	G	G	C	SNP	OS10G0491000	Exonic	CDS	Missense	LOC_Os10g34930	secretory protein, putative, expressed
Chr.10	19173825	19173825	G	G	C	SNP	OS10G0502600	Exonic	CDS	Missense	LOC_Os10g35870	cytochrome b5-like Heme/Steroid binding domain containing protein, expressed
Chr.10	20844177	20844177	T	T	C	SNP	OS10G0535800	Intronic	NA	NA	LOC_Os10g39100	uncharacterized Cys-rich domain containing protein, putative, expressed
Chr.10	20893765	20893765	A	A	C	SNP	OS10G0536500	Exonic	CDS	Silent	LOC_Os10g39150	expressed protein
Chr.10	67187	67193	CCTCCGG	CCTCCGG	C	INDEL	OS10G0100500	Exonic	CDS	InFrame	LOC_Os10g01060	protein kinase family protein, putative, expressed
Chr.10	2913521	2913536	CGCCGCCGCCGCCGCT	CGCCGCCGCCGCCGCT	C	INDEL	OS10G0148400	Exonic	CDS	InFrame	LOC_Os10g05780	POT domain containing peptide transporter, putative, expressed
Chr.10	12872901	12872902	CA	CA	C	INDEL	OS10G0389000	Intronic	NA	NA	LOC_Os10g25010	OsCML8 - Calmodulin-related calcium sensor protein, expressed
Chr.10	19358462	19358465	CAAA	CAAA	C	INDEL	OS10G0506200	Intronic	NA	NA	LOC_Os10g36210	valyl-tRNA synthetase, putative, expressed
Chr.1	29483416	29483416	T	T	C	SNP	OS01G0709500	Exonic	3UTR	NA	LOC_Os01g51290	protein kinase family protein, putative, expressed
Chr.1	262185	262185	T	T	C	SNP	OS01G0104800	Intronic	NA	NA	LOC_Os01g01510	sas10/Utp3 family protein, expressed
Chr.1	722853	722853	T	T	C	SNP	OS01G0113200	Exonic	CDS	Missense	LOC_Os01g02290	Ser/Thr receptor-like kinase, putative, expressed
Chr.1	6565548	6565548	T	T	C	SNP	OS01G0220100	Intronic	NA	NA	LOC_Os01g12070	endoglucanase precursor, putative, expressed
Chr.1	6969877	6969877	T	T	C	SNP	OS01G0226300	Exonic	3UTR	NA	LOC_Os01g12650	reticulon domain containing protein, putative, expressed
Chr.1	7092637	7092637	T	T	C	SNP	OS01G0228400	Intronic	NA	NA	LOC_Os01g12810	retrotransposon protein, putative, unclassified, expressed
Chr.1	7550454	7550454	T	T	C	SNP	OS01G0236300	Intronic	NA	NA	LOC_Os01g13520	auxin response factor 1, putative, expressed
Chr.1	8949707	8949707	T	T	C	SNP	OS01G0264000	Exonic	CDS	NA	LOC_Os01g15900	expressed protein
Chr.1	20742398	20742398	T	T	C	SNP	OS01G0552300	Exonic	3UTR	NA	LOC_Os01g37130	protein phosphatase 2C, putative, expressed
Chr.1	20739662	20739662	T	T	C	SNP	OS01G0552300	Exonic	CDS	Silent	LOC_Os01g37130	protein phosphatase 2C, putative, expressed
Chr.1	20739745	20739745	T	T	C	SNP	OS01G0552300	Intronic	NA	NA	LOC_Os01g37130	protein phosphatase 2C, putative, expressed
Chr.1	20739841	20739841	T	T	C	SNP	OS01G0552300	Intronic	NA	NA	LOC_Os01g37130	protein phosphatase 2C, putative, expressed
Chr.1	20740395	20740395	T	T	C	SNP	OS01G0552300	Intronic	NA	NA	LOC_Os01g37130	protein phosphatase 2C, putative, expressed
Chr.1	21632493	21632493	T	T	C	SNP	OS01G0565900	Exonic	CDS	Silent	LOC_Os01g38510	protein transport protein Sec61 subunit beta, putative, expressed
Chr.1	21637907	21637907	T	T	C	SNP	OS01G0566050	Exonic	CDS	NA	LOC_Os01g38530	ELF3 protein, putative, expressed
Chr.1	23344003	23344003	T	T	C	SNP	OS01G0595600	Intronic	NA	NA	LOC_Os01g41240	hydrolase, alpha/beta fold family domain containing protein, expressed
Chr.1	23344070	23344070	T	T	C	SNP	OS01G0595600	Intronic	NA	NA	LOC_Os01g41240	hydrolase, alpha/beta fold family domain containing protein, expressed
Chr.1	23344207	23344207	T	T	C	SNP	OS01G0595600	Intronic	NA	NA	LOC_Os01g41240	hydrolase, alpha/beta fold family domain containing protein, expressed
Chr.1	23673303	23673303	T	T	C	SNP	OS01G0602400	Exonic	CDS	Missense	LOC_Os01g41810	cytochrome P450 72A1, putative, expressed
Chr.1	23673306	23673306	T	T	C	SNP	OS01G0602400	Exonic	CDS	Missense	LOC_Os01g41810	cytochrome P450 72A1, putative, expressed
Chr.1	23673934	23673934	T	T	C	SNP	OS01G0602400	Exonic	CDS	Missense	LOC_Os01g41810	cytochrome P450 72A1, putative, expressed
Chr.1	23674038	23674038	T	T	C	SNP	OS01G0602400	Exonic	CDS	Missense	LOC_Os01g41810	cytochrome P450 72A1, putative, expressed
Chr.1	23674143	23674143	T	T	C	SNP	OS01G0602400	Exonic	CDS	Missense	LOC_Os01g41810	cytochrome P450 72A1, putative, expressed
Chr.1	24078542	24078542	T	T	C	SNP	OS01G0609300	Intronic	NA	NA	LOC_Os01g42380	pleiotropic drug resistance protein, putative, expressed
Chr.1	26120091	26120091	T	T	C	SNP	OS01G0648000	Exonic	CDS	Missense	LOC_Os01g45990	potassium channel AKT1, putative, expressed
Chr.1	26800693	26800693	T	T	C	SNP	OS01G0658900	Intronic	NA	NA	LOC_Os01g46970	transcription factor, putative, expressed
Chr.1	27174616	27174616	T	T	C	SNP	OS01G0665200	Exonic	3UTR	NA	LOC_Os01g47530	CGMC_MAPKCMGC_2.6 - CGMC includes CDA, MAPK, GSK3, and CLKC kinases, expressed
Chr.1	27174884	27174884	T	T	C	SNP	OS01G0665200	Exonic	3UTR	NA	LOC_Os01g47530	CGMC_MAPKCMGC_2.6 - CGMC includes CDA, MAPK, GSK3, and CLKC kinases, expressed
Chr.1	27180689	27180689	T	T	C	SNP	OS01G0665300	Exonic	3UTR	NA	LOC_Os01g47540	NLI interacting factor-like phosphatase, putative, expressed
Chr.1	29961599	29961599	T	T	C	SNP	OS01G0719000	Intronic	NA	NA	LOC_Os01g52100	DUF581 domain containing protein, expressed
Chr.1	30158423	30158423	T	T	C	SNP	OS01G0723200	Exonic	CDS	Silent	LOC_Os01g52490	40S ribosomal protein S24, putative, expressed
Chr.1	30158745	30158745	T	T	C	SNP	OS01G0723200	Intronic	NA	NA	LOC_Os01g52490	40S ribosomal protein S24, putative, expressed
Chr.1	30158960	30158960	T	T	C	SNP	OS01G0723200	Intronic	NA	NA	LOC_Os01g52490	40S ribosomal protein S24, putative, expressed
Chr.1	30159009	30159009	T	T	C	SNP	OS01G0723200	Intronic	NA	NA	LOC_Os01g52490	40S ribosomal protein S24, putative, expressed
Chr.1	30951293	30951293	T	T	C	SNP	OS01G0741200	Exonic	CDS	NA	LOC_Os01g53840	protein kinase family protein, putative, expressed
Chr.1	32002546	32002546	T	T	C	SNP	OS01G0760600	Intronic	NA	NA	LOC_Os01g55540	aminotransferase, classes I and II, domain containing protein, expressed
Chr.1	39830138	39830138	T	T	C	SNP	OS01G0914000	Intronic	NA	NA	LOC_Os01g68570	expressed protein
Chr.1	43075301	43075301	T	T	C	SNP	OS01G0974800	Exonic	5UTR	NA	LOC_Os01g74350	soluble inorganic pyrophosphatase, putative, expressed
Chr.1	604067	604067	G	G	C	SNP	OS01G0111500	Exonic	CDS	Silent	LOC_Os01g02110	helix-loop-helix DNA-binding domain containing protein, expressed
Chr.1	4215076	4215076	G	G	C	SNP	OS01G0180400	Exonic	3UTR	NA	LOC_Os01g08520	DUF581 domain containing protein, expressed
Chr.1	4215395	4215395	G	G	C	SNP	OS01G0180400	Exonic	3UTR	NA	LOC_Os01g08520	DUF581 domain containing protein, expressed
Chr.1	4337436	4337436	G	G	C	SNP	OS01G0182600	Intronic	NA	NA	LOC_Os01g08700	GIGANTEA, putative, expressed
Chr.1	5478785	5478785	G	G	C	SNP	OS01G0200700	Intronic	NA	NA	LOC_Os01g10400	expressed protein
Chr.1	6613132	6613132	G	G	C	SNP	OS01G0220700	Intronic	NA	NA	LOC_Os01g12130	nodulin MtN3 family protein, putative, expressed
Chr.1	7278956	7278956	G	G	C	SNP	OS01G0231700	Exonic	3UTR	NA	LOC_Os01g13080	60S acidic ribosomal protein, putative, expressed
Chr.1	7862079	7862079	G	G	C	SNP	OS01G0242600	Exonic	3UTR	NA	LOC_Os01g14050	C2 domain containing protein, putative, expressed
Chr.1	18127757	18127757	G	G	C	SNP	OS01G0513400	Intronic	NA	NA	LOC_Os01g33000	expressed protein
Chr.1	20742244	20742244	G	G	C	SNP	OS01G0552300	Exonic	3UTR	NA	LOC_Os01g37130	protein phosphatase 2C, putative, expressed
Chr.1	26894464	26894464	G	G	C	SNP	OS01G0660300	Intronic	NA	NA	LOC_Os01g47080	pyruvate kinase, putative, expressed
Chr.1	28575590	28575590	G	G	C	SNP	OS01G0692300	Exonic	3UTR	NA	LOC_Os01g49740	expressed protein
Chr.1	30158671	30158671	G	G	C	SNP	OS01G0723200	Intronic	NA	NA	LOC_Os01g52490	40S ribosomal protein S24, putative, expressed
Chr.1	30183920	30183920	G	G	C	SNP	OS01G0723600	Intronic	NA	NA	LOC_Os01g52530	phosphoribosyl transferase, putative, expressed
Chr.1	34550490	34550490	G	G	C	SNP	OS01G0812800	Exonic	3UTR	NA	LOC_Os01g59730	ribosomal protein L7Ae, putative, expressed
Chr.1	36083783	36083783	G	G	C	SNP	OS01G0840700	Intronic	NA	NA	LOC_Os01g62350	60S ribosomal protein L36-2, putative, expressed
Chr.1	38264465	38264465	G	G	C	SNP	OS01G0881500	Intronic	NA	NA	LOC_Os01g65900	chitin-inducible gibberellin-responsive protein, putative, expressed
Chr.1	651844	651844	G	G	C	SNP	OS01G0112500	Exonic	5UTR	NA	LOC_Os01g02200	armadillo/beta-catenin repeat family protein, putative, expressed
Chr.1	648546	648546	A	A	C	SNP	OS01G0112500	Exonic	CDS	Silent	LOC_Os01g02200	armadillo/beta-catenin repeat family protein, putative, expressed
Chr.1	649421	649421	T	T	C	SNP	OS01G0112500	Intronic	NA	NA	LOC_Os01g02200	armadillo/beta-catenin repeat family protein, putative, expressed
Chr.1	649627	649627	T	T	C	SNP	OS01G0112500	Intronic	NA	NA	LOC_Os01g02200	armadillo/beta-catenin repeat family protein, putative, expressed
Chr.1	650498	650498	A	A	C	SNP	OS01G0112500	Intronic	NA	NA	LOC_Os01g02200	armadillo/beta-catenin repeat family protein, putative, expressed
Chr.1	650528	650528	T	T	C	SNP	OS01G0112500	Intronic	NA	NA	LOC_Os01g02200	armadillo/beta-catenin repeat family protein, putative, expressed
Chr.1	650723	650723	T	T	C	SNP	OS01G0112500	Intronic	NA	NA	LOC_Os01g02200	armadillo/beta-catenin repeat family protein, putative, expressed

Chr.1	650739	650739	T	T	C	SNP	OS01G0112500	Intronic	NA	NA	LOC_Os01g02200	armadillo/beta-catenin repeat family protein, putative, expressed
Chr.1	650864	650864	T	T	C	SNP	OS01G0112500	Intronic	NA	NA	LOC_Os01g02200	armadillo/beta-catenin repeat family protein, putative, expressed
Chr.1	650882	650882	G	T	C	SNP	OS01G0112500	Intronic	NA	NA	LOC_Os01g02200	armadillo/beta-catenin repeat family protein, putative, expressed
Chr.1	650956	650956	T	T	C	SNP	OS01G0112500	Intronic	NA	NA	LOC_Os01g02200	armadillo/beta-catenin repeat family protein, putative, expressed
Chr.1	651124	651124	T	T	C	SNP	OS01G0112500	Intronic	NA	NA	LOC_Os01g02200	armadillo/beta-catenin repeat family protein, putative, expressed
Chr.1	744106	744106	T	T	C	SNP	OS01G0113700	Exonic	CDS	Missense	LOC_Os01g02350	receptor-like kinase ARK1AS, putative, expressed
Chr.1	744475	744475	T	T	C	SNP	OS01G0113700	Exonic	CDS	Missense	LOC_Os01g02350	receptor-like kinase ARK1AS, putative, expressed
Chr.1	744729	744729	T	T	C	SNP	OS01G0113700	Exonic	CDS	Missense	LOC_Os01g02350	receptor-like kinase ARK1AS, putative, expressed
Chr.1	744831	744831	G	G	C	SNP	OS01G0113700	Exonic	CDS	Missense	LOC_Os01g02350	receptor-like kinase ARK1AS, putative, expressed
Chr.1	4215574	4215574	G	G	C	SNP	OS01G0180400	Exonic	CDS	NA	LOC_Os01g08520	DUF581 domain containing protein, expressed
Chr.1	9229988	9229988	T	T	C	SNP	OS01G0268300	Intronic	NA	NA	LOC_Os01g16290	DNA gyrase subunit B, chloroplast/mitochondrial precursor, putative, expressed
Chr.1	9232895	9232895	T	T	C	SNP	OS01G0268300	Intronic	NA	NA	LOC_Os01g16290	DNA gyrase subunit B, chloroplast/mitochondrial precursor, putative, expressed
Chr.1	9234373	9234373	T	T	C	SNP	OS01G0268300	Intronic	NA	NA	LOC_Os01g16290	DNA gyrase subunit B, chloroplast/mitochondrial precursor, putative, expressed
Chr.1	9860128	9860128	A	A	C	SNP	OS01G0278900	Intronic	NA	NA	LOC_Os01g17150	plastid-specific 50S ribosomal protein 5, chloroplast precursor, putative, expressed
Chr.1	10200637	10200637	A	A	C	SNP	OS01G0284900	Exonic	3UTR	NA	LOC_Os01g18220	expressed protein
Chr.1	10200823	10200823	T	T	C	SNP	OS01G0284900	Exonic	3UTR	NA	LOC_Os01g18220	expressed protein
Chr.1	10342592	10342592	T	T	C	SNP	OS01G0287600	Intronic	NA	NA	LOC_Os01g18400	CHIT10 - Chitinase family protein precursor, expressed
Chr.1	10495411	10495411	G	G	C	SNP	OS01G0290000	Intronic	NA	NA	LOC_Os01g18630	aspartic proteinase oryzasin-1 precursor, putative, expressed
Chr.1	10655316	10655316	T	T	C	SNP	OS01G0292900	Exonic	CDS	Silent	LOC_Os01g18850	OsSPL1 - SBP-box gene family member, expressed
Chr.1	10653714	10653714	G	G	C	SNP	OS01G0292900	Exonic	CDS	Missense	LOC_Os01g18850	OsSPL1 - SBP-box gene family member, expressed
Chr.1	10654958	10654958	G	G	C	SNP	OS01G0292900	Intronic	NA	NA	LOC_Os01g18850	OsSPL1 - SBP-box gene family member, expressed
Chr.1	15294089	15294089	G	G	C	SNP	OS01G0371500	Exonic	CDS	Silent	LOC_Os01g27390	glutathione S-transferase, putative, expressed
Chr.1	15294107	15294107	T	T	C	SNP	OS01G0371500	Exonic	CDS	Silent	LOC_Os01g27390	glutathione S-transferase, putative, expressed
Chr.1	15293950	15293950	G	G	C	SNP	OS01G0371500	Exonic	CDS	Missense	LOC_Os01g27390	glutathione S-transferase, putative, expressed
Chr.1	15292114	15292114	T	T	C	SNP	OS01G0371500	Intronic	NA	NA	LOC_Os01g27390	glutathione S-transferase, putative, expressed
Chr.1	15292948	15292948	T	T	C	SNP	OS01G0371500	Intronic	NA	NA	LOC_Os01g27390	glutathione S-transferase, putative, expressed
Chr.1	15293289	15293289	T	T	C	SNP	OS01G0371500	Intronic	NA	NA	LOC_Os01g27390	glutathione S-transferase, putative, expressed
Chr.1	15293438	15293438	G	G	C	SNP	OS01G0371500	Intronic	NA	NA	LOC_Os01g27390	glutathione S-transferase, putative, expressed
Chr.1	15572300	15572300	G	G	C	SNP	OS01G0376700	Intronic	NA	NA	LOC_Os01g27880	sucrose-phosphatase, putative, expressed
Chr.1	20576095	20576095	G	G	C	SNP	OS01G0550000	Exonic	3UTR	NA	LOC_Os01g36920	DEAD-box ATP-dependent RNA helicase, putative, expressed
Chr.1	20576152	20576152	G	G	C	SNP	OS01G0550000	Exonic	3UTR	NA	LOC_Os01g36920	DEAD-box ATP-dependent RNA helicase, putative, expressed
Chr.1	20571941	20571941	T	T	C	SNP	OS01G0550000	Intronic	NA	NA	LOC_Os01g36920	DEAD-box ATP-dependent RNA helicase, putative, expressed
Chr.1	20572096	20572096	A	A	C	SNP	OS01G0550000	Intronic	NA	NA	LOC_Os01g36920	DEAD-box ATP-dependent RNA helicase, putative, expressed
Chr.1	20572445	20572445	G	G	C	SNP	OS01G0550000	Intronic	NA	NA	LOC_Os01g36920	DEAD-box ATP-dependent RNA helicase, putative, expressed
Chr.1	20572529	20572529	T	T	C	SNP	OS01G0550000	Intronic	NA	NA	LOC_Os01g36920	DEAD-box ATP-dependent RNA helicase, putative, expressed
Chr.1	20574661	20574661	T	T	C	SNP	OS01G0550000	Intronic	NA	NA	LOC_Os01g36920	DEAD-box ATP-dependent RNA helicase, putative, expressed
Chr.1	20582301	20582301	A	A	C	SNP	OS01G0550100	Exonic	CDS	Missense	LOC_Os01g36930	ubiquitin carboxyl-terminal hydrolase 6, putative, expressed
Chr.1	20583778	20583778	G	G	C	SNP	OS01G0550100	Intronic	NA	NA	LOC_Os01g36930	ubiquitin carboxyl-terminal hydrolase 6, putative, expressed
Chr.1	20585406	20585406	T	T	C	SNP	OS01G0550100	Intronic	NA	NA	LOC_Os01g36930	ubiquitin carboxyl-terminal hydrolase 6, putative, expressed
Chr.1	20585484	20585484	T	T	C	SNP	OS01G0550100	Intronic	NA	NA	LOC_Os01g36930	ubiquitin carboxyl-terminal hydrolase 6, putative, expressed
Chr.1	20589235	20589235	T	T	C	SNP	OS01G0550100	Intronic	NA	NA	LOC_Os01g36930	ubiquitin carboxyl-terminal hydrolase 6, putative, expressed
Chr.1	20601496	20601496	T	T	C	SNP	OS01G0550300	Exonic	CDS	Silent	LOC_Os01g36950	N-rich protein, putative, expressed
Chr.1	20601016	20601016	T	T	C	SNP	OS01G0550300	Intronic	NA	NA	LOC_Os01g36950	N-rich protein, putative, expressed
Chr.1	20601165	20601165	G	G	C	SNP	OS01G0550300	Intronic	NA	NA	LOC_Os01g36950	N-rich protein, putative, expressed
Chr.1	20740073	20740073	T	T	C	SNP	OS01G0552300	Intronic	NA	NA	LOC_Os01g37130	protein phosphatase 2C, putative, expressed
Chr.1	21125170	21125170	T	T	C	SNP	OS01G0558300	Exonic	3UTR	NA	LOC_Os01g37770	RWD domain containing protein, expressed
Chr.1	21127194	21127194	T	T	C	SNP	OS01G0558300	Exonic	CDS	Silent	LOC_Os01g37770	RWD domain containing protein, expressed
Chr.1	21125703	21125703	T	T	C	SNP	OS01G0558300	Exonic	CDS	Missense	LOC_Os01g37770	RWD domain containing protein, expressed
Chr.1	21125256	21125256	A	A	C	SNP	OS01G0558300	Intronic	NA	NA	LOC_Os01g37770	RWD domain containing protein, expressed
Chr.1	21126182	21126182	T	T	C	SNP	OS01G0558300	Intronic	NA	NA	LOC_Os01g37770	RWD domain containing protein, expressed
Chr.1	21126991	21126991	T	T	C	SNP	OS01G0558300	Intronic	NA	NA	LOC_Os01g37770	RWD domain containing protein, expressed
Chr.1	22494508	22494508	T	T	C	SNP	OS01G0580800	Exonic	3UTR	NA	LOC_Os01g39890	transposon protein, putative, unclassified, expressed
Chr.1	22495557	22495557	T	T	C	SNP	OS01G0580800	Exonic	CDS	Missense	LOC_Os01g39890	transposon protein, putative, unclassified, expressed
Chr.1	22496623	22496623	T	T	C	SNP	OS01G0580800	Intronic	NA	NA	LOC_Os01g39890	transposon protein, putative, unclassified, expressed
Chr.1	22626489	22626489	G	G	C	SNP	OS01G0583200	Intronic	NA	NA	LOC_Os01g40110	ZOS1-11 - C2H2 zinc finger protein, expressed
Chr.1	29490877	29490877	A	A	C	SNP	OS01G0709500	Exonic	CDS	Silent	LOC_Os01g51290	protein kinase family protein, putative, expressed
Chr.1	29483712	29483712	T	T	C	SNP	OS01G0709500	Exonic	CDS	Missense	LOC_Os01g51290	protein kinase family protein, putative, expressed
Chr.1	29896211	29896211	T	T	C	SNP	OS01G0717700	Intronic	NA	NA	LOC_Os01g52010	alliin lyase precursor, putative, expressed
Chr.1	41496513	41496513	T	T	C	SNP	OS01G0944100	Intronic	NA	NA	LOC_Os01g71624	expressed protein
Chr.1	41496858	41496858	A	A	C	SNP	OS01G0944100	Intronic	NA	NA	LOC_Os01g71624	expressed protein
Chr.1	41497339	41497339	T	T	C	SNP	OS01G0944100	Intronic	NA	NA	LOC_Os01g71624	expressed protein
Chr.1	41498198	41498198	T	T	C	SNP	OS01G0944100	Intronic	NA	NA	LOC_Os01g71624	expressed protein
Chr.1	41498793	41498793	T	T	C	SNP	OS01G0944100	Intronic	NA	NA	LOC_Os01g71624	expressed protein
Chr.1	41499144	41499144	T	T	C	SNP	OS01G0944100	Intronic	NA	NA	LOC_Os01g71624	expressed protein
Chr.1	744772	744778	CTCCAGG	CTCCAGG	C	INDEL	OS01G0113700	Exonic	CDS	InFrame	LOC_Os01g02350	receptor-like kinase ARK1AS, putative, expressed
Chr.1	9236929	9236942	CTAGTAGTAAGTAT	CTAGTAGTAAGTAT	C	INDEL	OS01G0268300	Exonic	3UTR	NA	LOC_Os01g16290	DNA gyrase subunit B, chloroplast/mitochondrial precursor, putative, expressed
Chr.1	10102622	10102623	CA	CA	C	INDEL	OS01G0282800	Intronic	NA	NA	LOC_Os01g18050	tubulin/FtsZ domain containing protein, putative, expressed
Chr.1	21125069	21125070	CA	CA	C	INDEL	OS01G0558300	Exonic	3UTR	NA	LOC_Os01g37770	RWD domain containing protein, expressed
Chr.1	21125676	21125678	CAG	CAG	C	INDEL	OS01G0558300	Intronic	NA	NA	LOC_Os01g37770	RWD domain containing protein, expressed
Chr.1	7559900	7559900	A	A	C	SNP	OS01G0236400	Intronic	NA	NA	LOC_Os01g13530	ABIL2, putative, expressed
Chr.1	17991851	17991851	A	A	C	SNP	OS01G0511100	Intronic	NA	NA	LOC_Os01g32780	universal stress protein domain containing protein, putative, expressed
Chr.1	20740148	20740148	A	A	C	SNP	OS01G0552300	Intronic	NA	NA	LOC_Os01g37130	protein phosphatase 2C, putative, expressed
Chr.1	21922186	21922186	A	A	C	SNP	OS01G0571100	Exonic	3UTR	NA	LOC_Os01g39000	ZmGR2c, putative, expressed
Chr.1	26891254	26891254	A	A	C	SNP	OS01G0660300	Intronic	NA	NA	LOC_Os01g47080	pyruvate kinase, putative, expressed
Chr.1	29844516	29844516	A	A	C	SNP	OS01G0717000	Intronic	NA	NA	LOC_Os01g51920	phosphotransferase, putative, expressed
Chr.1	34552121	34552121	A	A	C	SNP	OS01G0812800	Intronic	NA	NA	LOC_Os01g59730	ribosomal protein L7Ae, putative, expressed
Chr.1	34614714	34614714	A	A	C	SNP	OS01G0813900	Exonic	CDS	Missense	LOC_Os01g59850	GTPase-activating protein, putative, expressed
Chr.1	36083373	36083373	A	A	C	SNP	OS01G0840700	Intronic	NA	NA	LOC_Os01g62350	60S ribosomal protein L36-2, putative, expressed
Chr.1	38997055	38997055	A	A	C	SNP	OS01G0897300	Intronic	NA	NA	LOC_Os01g67190	ribonuclease T2 family domain containing protein, expressed
Chr.1	605400	605400	G	G	C	SNP	OS01G0111500	Exonic	CDS	Silent	LOC_Os01g02110	helix-loop-helix DNA-binding domain containing protein, expressed
Chr.1	605421	605421	A	A	C	SNP	OS01G0111500	Exonic	CDS	Silent	LOC_Os01g02110	helix-loop-helix DNA-binding domain containing protein, expressed
Chr.1	605047	605047	G	G	C	SNP	OS01G0111500	Exonic	CDS	Missense	LOC_Os01g02110	helix-loop-helix DNA-binding domain containing protein, expressed
Chr.1	605080	605080	T	T	C	SNP	OS01G0111500	Exonic	CDS	Missense	LOC_Os01g02110	helix-loop-helix DNA-binding domain containing protein, expressed
Chr.1	744656	744656	T	T	C	SNP	OS01G0113700	Exonic	CDS	Missense	LOC_Os01g02350	receptor-like kinase ARK1AS, putative, expressed
Chr.1	5904154	5904154	T	T	C	SNP	OS01G0208700	Intronic	NA	NA	LOC_Os01g11054	phosphoenolpyruvate carboxylase, putative, expressed

Chr.1	6788173	6788173	T	T	C	SNP	OS01G0223900	Exonic	CDS	Silent	LOC_Os01g12420	receptor-like protein kinase, putative, expressed
Chr.1	6787236	6787236	A	A	C	SNP	OS01G0223900	Exonic	CDS	Missense	LOC_Os01g12420	receptor-like protein kinase, putative, expressed
Chr.1	7026427	7026427	T	T	C	SNP	OS01G0227100	Intronic	NA	NA	LOC_Os01g12710	oxidoreductase, short chain dehydrogenase/reductase family domain containing protein, expressed
Chr.1	7092281	7092281	A	A	C	SNP	OS01G0228400	Exonic	5UTR	NA	LOC_Os01g12810	retrotransposon protein, putative, unclassified, expressed
Chr.1	7280207	7280207	T	T	C	SNP	OS01G0231700	Exonic	5UTR	NA	LOC_Os01g13080	60S acidic ribosomal protein, putative, expressed
Chr.1	7547414	7547414	G	G	C	SNP	OS01G0236300	Intronic	NA	NA	LOC_Os01g13520	auxin response factor 1, putative, expressed
Chr.1	9661272	9661272	G	G	C	SNP	OS01G0276000	Exonic	5UTR	NA	LOC_Os01g16890	ribosomal protein L7Ae, putative, expressed
Chr.1	10201296	10201296	T	T	C	SNP	OS01G0284900	Intronic	NA	NA	LOC_Os01g18220	expressed protein
Chr.1	10201302	10201302	T	T	C	SNP	OS01G0284900	Intronic	NA	NA	LOC_Os01g18220	expressed protein
Chr.1	10201316	10201316	T	T	C	SNP	OS01G0284900	Intronic	NA	NA	LOC_Os01g18220	expressed protein
Chr.1	10201322	10201322	T	T	C	SNP	OS01G0284900	Intronic	NA	NA	LOC_Os01g18220	expressed protein
Chr.1	15294044	15294044	T	T	C	SNP	OS01G0371500	Exonic	CDS	Silent	LOC_Os01g27390	glutathione S-transferase, putative, expressed
Chr.1	15294000	15294000	G	G	C	SNP	OS01G0371500	Exonic	CDS	Missense	LOC_Os01g27390	glutathione S-transferase, putative, expressed
Chr.1	15291909	15291909	T	T	C	SNP	OS01G0371500	Intronic	NA	NA	LOC_Os01g27390	glutathione S-transferase, putative, expressed
Chr.1	20571160	20571160	T	T	C	SNP	OS01G0550000	Exonic	CDS	Silent	LOC_Os01g36920	DEAD-box ATP-dependent RNA helicase, putative, expressed
Chr.1	20589403	20589403	A	A	C	SNP	OS01G0550100	Intronic	NA	NA	LOC_Os01g36930	ubiquitin carboxyl-terminal hydrolase 6, putative, expressed
Chr.1	20738944	20738944	A	A	C	SNP	OS01G0552300	Exonic	CDS	Silent	LOC_Os01g37130	protein phosphatase 2C, putative, expressed
Chr.1	21040769	21040769	G	G	C	SNP	OS01G0556700	Exonic	CDS	NA	LOC_Os01g37590	peptide transporter PTR2, putative, expressed
Chr.1	22747745	22747745	G	G	C	SNP	OS01G0585200	Exonic	CDS	Missense	LOC_Os01g40290	expressed protein
Chr.1	23343615	23343615	A	A	C	SNP	OS01G0595600	Exonic	CDS	Missense	LOC_Os01g41240	hydrolase, alpha/beta fold family domain containing protein, expressed
Chr.1	23343636	23343636	A	A	C	SNP	OS01G0595600	Exonic	CDS	Missense	LOC_Os01g41240	hydrolase, alpha/beta fold family domain containing protein, expressed
Chr.1	23344021	23344021	A	A	C	SNP	OS01G0595600	Intronic	NA	NA	LOC_Os01g41240	hydrolase, alpha/beta fold family domain containing protein, expressed
Chr.1	23675750	23675750	G	G	C	SNP	OS01G0602400	Exonic	CDS	Silent	LOC_Os01g41810	cytochrome P450 72A1, putative, expressed
Chr.1	24078834	24078834	A	A	C	SNP	OS01G0609300	Intronic	NA	NA	LOC_Os01g42380	pleiotropic drug resistance protein, putative, expressed
Chr.1	24785694	24785694	T	T	C	SNP	OS01G0621600	Exonic	CDS	Missense	LOC_Os01g43350	protein kinase family protein, putative, expressed
Chr.1	25599354	25599354	G	G	C	SNP	OS01G0638000	Exonic	CDS	Missense	LOC_Os01g45110	anthocyanin 3-O-beta-glucosyltransferase, putative, expressed
Chr.1	27171639	27171639	A	A	C	SNP	OS01G0665200	Exonic	5UTR	NA	LOC_Os01g47530	CGMC_MAPKCMGC_2.6 - CGMC includes CDA, MAPK, GSK3, and CLKC kinases, expressed
Chr.1	28574562	28574562	G	G	C	SNP	OS01G0692300	Exonic	CDS	Silent	LOC_Os01g49740	expressed protein
Chr.1	29188922	29188922	T	T	C	SNP	OS01G0704100	Exonic	5UTR	NA	LOC_Os01g50820	transporter, major facilitator family, putative, expressed
Chr.1	30261189	30261189	T	T	C	SNP	OS01G0725800	Exonic	CDS	Missense	LOC_Os01g52640	suppressor of phythchrome A, putative, expressed
Chr.1	32259279	32259279	A	A	C	SNP	OS01G0765600	Exonic	3UTR	NA	LOC_Os01g56030	EF hand family protein, putative, expressed
Chr.1	33078630	33078630	T	T	C	SNP	OS01G0780800	Exonic	5UTR	NA	LOC_Os01g57240	expressed protein
Chr.1	34550646	34550646	T	T	C	SNP	OS01G0812800	Exonic	3UTR	NA	LOC_Os01g59730	ribosomal protein L7Ae, putative, expressed
Chr.1	34552525	34552525	T	T	C	SNP	OS01G0812800	Intronic	NA	NA	LOC_Os01g59730	ribosomal protein L7Ae, putative, expressed
Chr.1	35032965	35032965	G	G	C	SNP	OS01G0821300	Exonic	3UTR	NA	LOC_Os01g60600	WRKY108, expressed
Chr.1	39565977	39565977	A	A	C	SNP	OS01G0908700	Exonic	3UTR	NA	LOC_Os01g68060	copine, putative, expressed
Chr.1	267038	267040	CAT	CAT	C	INDEL	OS01G0104800	Intronic	NA	NA	LOC_Os01g01510	sas10/Utp3 family protein, expressed
Chr.1	603793	603799	CAGGCTT	CAGGCTT	C	INDEL	OS01G0111500	Exonic	3UTR	NA	LOC_Os01g02110	helix-loop-helix DNA-binding domain containing protein, expressed
Chr.1	605207	605210	CGCT	CGCT	C	INDEL	OS01G0111500	Exonic	CDS	InFrame	LOC_Os01g02110	helix-loop-helix DNA-binding domain containing protein, expressed
Chr.1	4217005	4217017	CAATCCAAATCCA	CAATCCAAATCCA	C	INDEL	OS01G0180400	Exonic	CDS	NA	LOC_Os01g08520	DUF581 domain containing protein, expressed
Chr.1	7867314	7867315	CA	CA	C	INDEL	OS01G0242600	Intronic	NA	NA	LOC_Os01g14050	C2 domain containing protein, putative, expressed
Chr.1	9020307	9020308	CT	CT	C	INDEL	OS01G0265100	Intronic	NA	NA	LOC_Os01g16030	ADP-ribosylation factor, putative, expressed
Chr.1	13904535	13904537	CAA	CAA	C	INDEL	OS01G0348900	Intronic	NA	NA	LOC_Os01g24710	jacalin-like lectin domain containing protein, expressed
Chr.1	23343600	23343609	CCGGCGGCGG	CCGGCGGCGG	C	INDEL	OS01G0595600	Exonic	CDS	InFrame	LOC_Os01g41240	hydrolase, alpha/beta fold family domain containing protein, expressed
Chr.1	23674066	23674067	CT	CT	C	INDEL	OS01G0602400	Exonic	CDS	FrameShift	LOC_Os01g41810	cytochrome P450 72A1, putative, expressed
Chr.1	24113864	24113865	CT	CT	C	INDEL	OS01G0609900	Intronic	NA	NA	LOC_Os01g42410	pleiotropic drug resistance protein, putative, expressed
Chr.1	25236080	25236082	CGT	CGT	C	INDEL	OS01G0631200	Intronic	NA	NA	LOC_Os01g44050	siroheme synthase, putative, expressed
Chr.1	27084067	27084075	CTAGTACTA	CTAGTACTA	C	INDEL	OS01G0663300	Intronic	NA	NA	LOC_Os01g47400	OsMan01 - Endo-Beta-Mannanase, expressed
Chr.1	27171431	27171432	CA	CA	C	INDEL	OS01G0665200	Exonic	5UTR	NA	LOC_Os01g47530	CGMC_MAPKCMGC_2.6 - CGMC includes CDA, MAPK, GSK3, and CLKC kinases, expressed
Chr.1	28347372	28347378	CTAATTT	CTAATTT	C	INDEL	OS01G0687300	Intronic	NA	NA	LOC_Os01g49310	expressed protein
Chr.1	30158151	30158152	CA	CA	C	INDEL	OS01G0723200	Exonic	3UTR	NA	LOC_Os01g52490	40S ribosomal protein S24, putative, expressed
Chr.1	30159136	30159140	CTACA	CTACA	C	INDEL	OS01G0723200	Intronic	NA	NA	LOC_Os01g52490	40S ribosomal protein S24, putative, expressed
Chr.1	30159082	30159084	CCA	CCA	C	INDEL	OS01G0723200	Intronic	NA	NA	LOC_Os01g52490	40S ribosomal protein S24, putative, expressed
Chr.1	30159524	30159526	CGA	CGA	C	INDEL	OS01G0723200	Intronic	NA	NA	LOC_Os01g52490	40S ribosomal protein S24, putative, expressed
Chr.1	30158454	30158455	CA	CA	C	INDEL	OS01G0723200	Intronic	NA	NA	LOC_Os01g52490	40S ribosomal protein S24, putative, expressed
Chr.1	32135204	32135206	CAT	CAT	C	INDEL	OS01G0763600	Intronic	NA	NA	LOC_Os01g55780	glycerophosphoryl diester phosphodiesterase family protein, putative, expressed
Chr.1	33075666	33075667	CT	CT	C	INDEL	OS01G0780800	Intronic	NA	NA	LOC_Os01g57240	expressed protein
Chr.1	34617273	34617277	CAGTT	CAGTT	C	INDEL	OS01G0813900	Intronic	NA	NA	LOC_Os01g59850	GTPase-activating protein, putative, expressed
Chr.1	39840387	39840389	CTG	CTG	C	INDEL	OS01G0914300	Exonic	3UTR	NA	LOC_Os01g68589	LTPL39 - Protease inhibitor/seed storage/LTP family protein precursor, expressed
Chr.1	41005394	41005407	CAAATATATTATTA	CAAATATATTATTA	C	INDEL	OS01G0934800	Intronic	NA	NA	LOC_Os01g70850	esterase, putative, expressed
Chr.1	41384437	41384439	CTT	CTT	C	INDEL	OS01G0941800	Exonic	3UTR	NA	LOC_Os01g71420	Ser/Thr protein phosphatase family protein, putative, expressed
Chr.11	27490029	27490041	CCAAAAAAGTTCT	CCAAAAAAGTTCT	C	INDEL	OS11G0679700	Exonic	3UTR	NA	LOC_Os11g45400	glycerol-3-phosphate acyltransferase, putative, expressed
Chr12	3233346	3233350	CAAAA	C	CA	INDEL	OS12G0163700	Intronic	NA	NA	LOC_Os12g06660	actin, putative, expressed
Chr10	19433106	19433108	CAA	C	CA	INDEL	OS10G0507800	Intronic	NA	NA	LOC_Os10g36370	heat shock protein DnaJ, putative, expressed
Chr.6	1693550	1693550	C	CAA	CA	INDEL	OS06G0131500	Exonic	3UTR	NA	LOC_Os06g04080	glycosyl hydrolases family 17, putative, expressed
Chr.10	23092675	23092675	C	CAAAA	CA	INDEL	OS10G0579100	Intronic	NA	NA	LOC_Os10g42820	early-responsive to dehydration protein-related, putative, expressed
Chr.2	31959516	31959516	C	CAAAA	CA	INDEL	OS02G0759000	Intronic	NA	NA	LOC_Os02g52190	helix-loop-helix DNA-binding protein, putative, expressed
Chr7	26775508	26775508	C	C	CA	INDEL	OS07G0642900	Exonic	3UTR	NA	LOC_Os07g44840	bacterial transferase hexapeptide domain containing protein, expressed
Chr7	27726519	27726519	C	C	CA	INDEL	OS07G0658400	Intronic	NA	NA	LOC_Os07g46460	ferredoxin-dependent glutamate synthase, chloroplast precursor, putative, expressed
Chr.9	7470988	7470988	C	C	CA	INDEL	OS09G0300800	Intronic	NA	NA	LOC_Os09g12970	plant protein of unknown function domain containing protein, expressed
Chr.8	1620178	1620178	C	C	CA	INDEL	OS08G0128500	Intronic	NA	NA	LOC_Os08g03450	ribosomal protein L37, putative, expressed
Chr.8	3389151	3389151	C	C	CA	INDEL	OS08G0157900	Exonic	3UTR	NA	LOC_Os08g06140	no apical meristem protein, putative, expressed
Chr.8	3423679	3423679	C	C	CA	INDEL	OS08G0158500	Intronic	NA	NA	LOC_Os08g06200	stomatin-like protein 2, putative, expressed
Chr.8	23989680	23989680	C	C	CA	INDEL	OS08G0485400	Intronic	NA	NA	LOC_Os08g37874	2-nitropropane dioxygenase family, putative, expressed
Chr.8	7364104	7364104	C	C	CA	INDEL	OS08G0221300	Intronic	NA	NA	LOC_Os08g12449	expressed protein
Chr.6	23112449	23112449	C	C	CA	INDEL	OS06G0589300	Intronic	NA	NA	LOC_Os06g38950	ABC transporter, ATP-binding protein, putative, expressed
Chr.6	21601933	21601933	C	C	CA	INDEL	OS06G0562600	Intronic	NA	NA	LOC_Os06g36700	T-complex protein, putative, expressed
Chr.6	1385936	1385936	C	C	CA	INDEL	OS06G0125800	Exonic	3UTR	NA	LOC_Os06g03580	zinc RING finger protein, putative, expressed
Chr.5	15487075	15487075	C	C	CA	INDEL	OS05G0331200	Intronic	NA	NA	LOC_Os05g26660	NADH-ubiquinone oxidoreductase, mitochondrial precursor, putative, expressed
Chr.5	27746447	27746447	C	C	CA	INDEL	OS05G0557700	Intronic	NA	NA	LOC_Os05g48390	ubiquitin conjugating enzyme protein, putative, expressed
Chr.4	28084563	28084563	C	C	CA	INDEL	OS04G0560700	Exonic	5UTR	NA	LOC_Os04g47310	expressed protein
Chr.4	23832094	23832094	C	C	CA	INDEL	OS04G0476100	Intronic	NA	NA	LOC_Os04g40040	copper methylamine oxidase precursor, putative, expressed
Chr.4	31181873	31181873	C	C	CA	INDEL	OS04G0614600	Intronic	NA	NA	LOC_Os04g52450	aminotransferase, putative, expressed
Chr.4	20388408	20388408	C	C	CA	INDEL	OS04G0412500	Intronic	NA	NA	LOC_Os04g33660	bifunctional monodehydroascorbate reductase and carbonic anhydrasenectarin-3 precursor, putative, expressed
Chr.4	23315494	23315494	C	C	CA	INDEL	OS04G0466600	Intronic	NA	NA	LOC_Os04g39210	agmatine deiminase, putative, expressed

Chr.4	25843939	25843939	C	C	CA	INDEL	OS04G0517100	Exonic	5UTR	NA	LOC_Os04g43680	MYB family transcription factor, putative, expressed
Chr.4	29045397	29045397	C	C	CA	INDEL	OS04G0576500	Intronic	NA	NA	LOC_Os04g48710	expressed protein
Chr.4	32229675	32229675	C	C	CA	INDEL	OS04G0633300	Intronic	NA	NA	LOC_Os04g54080	serine/threonine-protein kinase receptor precursor, putative, expressed
Chr.4	34457591	34457591	C	C	CA	INDEL	OS04G0675000	Exonic	3UTR	NA	LOC_Os04g57870	expressed protein
Chr.3	35873899	35873899	C	C	CA	INDEL	OS03G0851900	Intronic	NA	NA	LOC_Os03g63500	ATPase, AFG1 family domain containing protein, expressed
Chr.3	10622967	10622967	C	C	CA	INDEL	OS03G0301700	Intronic	NA	NA	LOC_Os03g18970	protein phosphotase protein, putative, expressed
Chr.3	3530815	3530815	C	C	CA	INDEL	OS03G0165400	Intronic	NA	NA	LOC_Os03g06940	beta-galactosidase, putative, expressed
Chr.3	13137228	13137228	C	C	CA	INDEL	OS03G0350300	Intronic	NA	NA	LOC_Os03g22740	nucleolar protein NOP5-1, putative, expressed
Chr.3	29145232	29145232	C	C	CA	INDEL	OS03G0719300	Intronic	NA	NA	LOC_Os03g51000	3,4-dihydroxy-2-butanone kinase, putative, expressed
Chr.2	9924305	9924305	C	C	CA	INDEL	OS02G0272900	Exonic	CDS	FrameShift	LOC_Os02g17304	resistance protein, putative, expressed
Chr.2	18986812	18986812	C	C	CA	INDEL	OS02G0521100	Exonic	3UTR	NA	LOC_Os02g32140	AP2 domain containing protein, expressed
Chr.2	23996144	23996144	C	C	CA	INDEL	OS02G0610600	Intronic	NA	NA	LOC_Os02g39720	splicing factor, arginine/serine-rich 7, putative, expressed
Chr.11	2760514	2760514	C	C	CA	INDEL	OS11G0157400	Intronic	NA	NA	LOC_Os11g05880	exo70 exocyst complex subunit, putative, expressed
Chr.11	14051605	14051605	C	C	CA	INDEL	OS11G0434000	Intronic	NA	NA	LOC_Os11g24630	magnesium-dependent phosphatase 1, putative, expressed
Chr.11	18936629	18936629	C	C	CA	INDEL	OS11G0523800	Intronic	NA	NA	LOC_Os11g32110	auxin response factor, putative, expressed
Chr.11	17235682	17235682	C	C	CA	INDEL	OS11G0489250	Intronic	NA	NA	LOC_Os11g29720	cytochrome P450, putative, expressed
Chr.11	17284037	17284037	C	C	CA	INDEL	OS11G0490100	Exonic	5UTR	NA	LOC_Os11g29780	plant-specific domain TIGR01627 family protein, expressed
Chr.10	19362705	19362705	C	C	CA	INDEL	OS10G0506200	Intronic	NA	NA	LOC_Os10g36210	valyl-tRNA synthetase, putative, expressed
Chr.1	9140244	9140244	C	C	CA	INDEL	OS01G0266800	Intronic	NA	NA	LOC_Os01g16170	PQ loop repeat domain containing protein, expressed
Chr.1	26893994	26893994	C	C	CA	INDEL	OS01G0660300	Intronic	NA	NA	LOC_Os01g47080	pyruvate kinase, putative, expressed
Chr.1	26894465	26894465	C	C	CA	INDEL	OS01G0660300	Intronic	NA	NA	LOC_Os01g47080	pyruvate kinase, putative, expressed
Chr.1	27180459	27180459	C	C	CA	INDEL	OS01G0665300	Exonic	3UTR	NA	LOC_Os01g47540	NLI interacting factor-like phosphatase, putative, expressed
Chr.1	42353453	42353453	C	C	CA	INDEL	OS01G0960500	Intronic	NA	NA	LOC_Os01g73000	copine, putative, expressed
Chr.1	20738971	20738971	C	C	CA	INDEL	OS01G0552300	Intronic	NA	NA	LOC_Os01g37130	protein phosphatase 2C, putative, expressed
Chr.1	30183645	30183645	C	C	CA	INDEL	OS01G0723600	Intronic	NA	NA	LOC_Os01g52530	phosphoribosyl transferase, putative, expressed
Chr1	22498871	22498873	CAA	C	CAA	INDEL	OS01G0580800	Intronic	NA	NA	LOC_Os01g39890	transposon protein, putative, unclassified, expressed
Chr1	24075327	24075328	CA	C	CAA	INDEL	OS01G0609300	Exonic	3UTR	NA	LOC_Os01g42380	pleiotropic drug resistance protein, putative, expressed
Chr.3	27249533	27249533	C	CA	CAA	INDEL	OS03G0683700	Intronic	NA	NA	LOC_Os03g47930	expressed protein
Chr.1	38264986	38264986	C	CA	CAA	INDEL	OS01G0881500	Intronic	NA	NA	LOC_Os01g65900	chitin-inducible gibberellin-responsive protein, putative, expressed
Chr7	27726829	27726829	C	C	CAA	INDEL	OS07G0658400	Intronic	NA	NA	LOC_Os07g46460	ferredoxin-dependent glutamate synthase, chloroplast precursor, putative, expressed
Chr.6	28194446	28194446	C	C	CAA	INDEL	OS06G0678200	Intronic	NA	NA	LOC_Os06g46450	polyprenyl synthetase, putative, expressed
Chr.4	31220609	31220609	C	C	CAA	INDEL	OS04G0615200	Intronic	NA	NA	LOC_Os04g52504	adhesive/proline-rich protein, putative, expressed
Chr.3	1238204	1238204	C	C	CAA	INDEL	OS03G0122300	Intronic	NA	NA	LOC_Os03g03034	flavonol synthase/flavanone 3-hydroxylase, putative, expressed
Chr.2	5158378	5158378	C	C	CAA	INDEL	OS02G0192500	Intronic	NA	NA	LOC_Os02g09930	CSLA1 - cellulose synthase-like family A; mannan synthase, expressed
Chr.1	15291957	15291957	C	C	CAA	INDEL	OS01G0371500	Intronic	NA	NA	LOC_Os01g27390	glutathione S-transferase, putative, expressed
Chr8	10812103	10812104	CA	C	CAAA	INDEL	OS08G0278900	Intronic	NA	NA	LOC_Os08g17680	stromal cell-derived factor 2-like protein precursor, putative, expressed
Chr.4	27568961	27568962	CA	C	CAAA	INDEL	OS04G0550600	Intronic	NA	NA	LOC_Os04g46470	carotenoid cleavage dioxygenase 7, chloroplast precursor, putative, expressed
Chr.8	3392974	3392974	C	CA	CAAA	INDEL	OS08G0157900	Intronic	NA	NA	LOC_Os08g06140	no apical meristem protein, putative, expressed
Chr.1	7458691	7458691	C	CA	CAAA	INDEL	OS01G0235100	Intronic	NA	NA	LOC_Os01g13390	expressed protein
Chr.6	30445963	30445963	C	CA	CAAA	INDEL	OS06G0716700	Intronic	NA	NA	LOC_Os06g50300	heat shock protein, putative, expressed
Chr.6	31072404	31072404	C	CA	CAAA	INDEL	OS06G0729300	Intronic	NA	NA	LOC_Os06g51310	PAZ domain containing protein, putative, expressed
Chr7	29633990	29633990	C	CAA	CAAA	INDEL	OS07G0695400	Exonic	3UTR	NA	LOC_Os07g49480	KIP1, putative, expressed
Chr.6	27142614	27142614	C	C	CAAA	INDEL	OS06G0659300	Intronic	NA	NA	LOC_Os06g44890	villin protein, putative, expressed
Chr.3	10122373	10122373	C	C	CAAA	INDEL	OS03G0291500	Intronic	NA	NA	LOC_Os03g18130	asparagine synthetase, putative, expressed
Chr.3	33348515	33348515	C	C	CAAA	INDEL	OS03G0799900	Exonic	3UTR	NA	LOC_Os03g58570	WD domain, G-beta repeat domain containing protein, expressed
Chr.1	39831103	39831103	C	C	CAAA	INDEL	OS01G0914000	Intronic	NA	NA	LOC_Os01g68570	expressed protein
Chr12	25521870	25521871	CA	C	CAAAA	INDEL	OS12G0604700	Intronic	NA	NA	LOC_Os12g41180	LSTK-1-like kinase, putative, expressed
Chr.4	31281255	31281255	C	C	CAAAA	INDEL	OS04G0616300	Intronic	NA	NA	LOC_Os04g52600	SHR5-receptor-like kinase, putative, expressed
Chr.2	6835357	6835357	C	C	CAAGAA	INDEL	OS02G0222500	Intronic	NA	NA	LOC_Os02g12939	expressed protein
Chr7	27728890	27728890	C	C	CACAGATAA	INDEL	OS07G0658400	Intronic	NA	NA	LOC_Os07g46460	ferredoxin-dependent glutamate synthase, chloroplast precursor, putative, expressed
Chr7	4685985	4685985	C	CAGAG	CAG	INDEL	OS07G0187700	Intronic	NA	NA	LOC_Os07g09000	WD domain, G-beta repeat domain containing protein, expressed
Chr.5	19828948	19828948	C	C	CAG	INDEL	OS05G0406800	Exonic	5UTR	NA	LOC_Os05g33690	receptor-like protein kinase precursor, putative, expressed
Chr.3	30075076	30075076	C	C	CAG	INDEL	OS03G0734100	Intronic	NA	NA	LOC_Os03g52370	PIII4 - Proteinase inhibitor II family protein precursor, expressed
Chr.1	650958	650958	C	C	CAGA	INDEL	OS01G0112500	Intronic	NA	NA	LOC_Os01g02200	armadillo/beta-catenin repeat family protein, putative, expressed
Chr.5	836697	836707	CAGAGAGAGAG	C	CAGAGAGAG	INDEL	OS05G0115100	Exonic	CDS	FrameShift	LOC_Os05g02450	expressed protein
Chr.1	7458087	7458087	C	C	CAGT	INDEL	OS01G0235100	Exonic	CDS	InFrame	LOC_Os01g13390	expressed protein
Chr7	8967661	8967661	C	C	CAT	INDEL	OS07G0258400	Intronic	NA	NA	LOC_Os07g15460	metal transporter Nramp6, putative, expressed
Chr.4	23142333	23142333	C	C	CAT	INDEL	OS04G0463500	Intronic	NA	NA	LOC_Os04g38950	class I glutamine amidotransferase, putative, expressed
Chr.11	27487982	27487982	C	C	CAT	INDEL	OS11G0679700	Intronic	NA	NA	LOC_Os11g45400	glycerol-3-phosphate acyltransferase, putative, expressed
Chr.11	27488240	27488240	C	C	CAT	INDEL	OS11G0679700	Intronic	NA	NA	LOC_Os11g45400	glycerol-3-phosphate acyltransferase, putative, expressed
Chr.3	24816302	24816302	C	C	CATAAAGTG	INDEL	OS03G0643300	Intronic	NA	NA	LOC_Os03g44150	aminotransferase, putative, expressed
Chr.10	21864210	21864210	C	C	CATAT	INDEL	OS10G0556200	Intronic	NA	NA	LOC_Os10g40740	helix-loop-helix DNA-binding domain containing protein, expressed
Chr.1	7363928	7363928	C	CATAT	CATATAT	INDEL	OS01G0233000	Exonic	3UTR	NA	LOC_Os01g13210	salt stress root protein RS1, putative, expressed
Chr.2	81112	81112	C	C	CATATAT	INDEL	OS02G0101500	Intronic	NA	NA	LOC_Os02g01150	erythronate-4-phosphate dehydrogenase domain containing protein, expressed
Chr.2	80312	80312	C	C	CATT	INDEL	OS02G0101500	Intronic	NA	NA	LOC_Os02g01150	erythronate-4-phosphate dehydrogenase domain containing protein, expressed
Chr.11	28766272	28766272	C	C	CATTA	INDEL	OS11G0702400	Exonic	3UTR	NA	LOC_Os11g47630	ZOS11-10 - C2H2 zinc finger protein, expressed
Chr.4	7308646	7308646	C	C	CCA	INDEL	OS04G0209200	Intronic	NA	NA	LOC_Os04g13210	multidrug resistance-associated protein, putative, expressed
Chr.11	2764394	2764394	C	C	CCACA	INDEL	OS11G0157400	Intronic	NA	NA	LOC_Os11g05880	exo70 exocyst complex subunit, putative, expressed
Chr.12	25648906	25648906	C	C	CCACTATGG	INDEL	OS12G0607100	Intronic	NA	NA	LOC_Os12g41400	eukaryotic translation initiation factor 2 subunit gamma, putative, expressed
Chr.6	7230484	7230484	C	C	CCAG	INDEL	OS06G0239200	Exonic	CDS	InFrame	LOC_Os06g13190	expressed protein
Chr.4	33167103	33167103	C	C	CCCT	INDEL	OS04G0650800	Exonic	5UTR	NA	LOC_Os04g55720	D-3-phosphoglycerate dehydrogenase, chloroplast precursor, putative, expressed
Chr.2	2744335	2744335	C	C	CCCT	INDEL	OS02G0149800	Exonic	5UTR	NA	LOC_Os02g05630	protein phosphatase 2C, putative, expressed
Chr.11	2764731	2764731	C	CCCT	CCCTCCT	INDEL	OS11G0157400	Exonic	5UTR	NA	LOC_Os11g05880	exo70 exocyst complex subunit, putative, expressed
Chr7	24396361	24396361	C	C	CCG	INDEL	OS07G0598200	Exonic	5UTR	NA	LOC_Os07g40710	circadian clock coupling factor-related, putative, expressed
Chr.3	15669438	15669438	C	C	CCGCAG	INDEL	OS03G0391100	Intronic	NA	NA	LOC_Os03g27360	RING-H2 finger protein ATL5H precursor, putative, expressed
Chr.6	21896829	21896829	C	C	CCGCCGCCGG	INDEL	OS06G0567200	Exonic	CDS	InFrame	LOC_Os06g37080	L-ascorbate oxidase precursor, putative, expressed
Chr.11	22282027	22282027	C	C	CCGCCGT	INDEL	OS11G0587600	Exonic	CDS	InFrame	LOC_Os11g37700	pleiotropic drug resistance protein, putative, expressed
Chr.3	32694891	32694891	C	C	CCGG	INDEL	OS03G0787000	Exonic	CDS	InFrame	LOC_Os03g57310	syntaxin, putative, expressed
Chr.1	40796815	40796815	C	C	CCGG	INDEL	OS01G0929500	Exonic	CDS	InFrame	LOC_Os01g70430	oxidoreductase, short chain dehydrogenase/reductase family domain containing protein, expressed
Chr.5	29491280	29491286	CCTCTCT	C	CCT	INDEL	OS05G0591900	Exonic	5UTR	NA	LOC_Os05g51420	hypersensitive-induced response protein, putative, expressed
Chr.8	17576197	17576197	C	T	CCT	INDEL	OS08G0374800	Exonic	5UTR	NA	LOC_Os08g28730	NAD dependent epimerase/dehydratase family protein, putative, expressed
Chr.6	2472779	2472779	C	C	CCT	INDEL	OS06G0147300	Exonic	5UTR	NA	LOC_Os06g05470	expressed protein
Chr.3	1235618	1235618	C	C	CCT	INDEL	OS03G0122300	Exonic	3UTR	NA	LOC_Os03g03034	flavonol synthase/flavanone 3-hydroxylase, putative, expressed
Chr.3	16590369	16590369	C	C	CCT	INDEL	OS03G0405500	Intronic	NA	NA	LOC_Os03g29190	PDI, putative, expressed
Chr.1	27082455	27082455	C	C	CCT	INDEL	OS01G0663300	Intronic	NA	NA	LOC_Os01g47400	OsMan01 - Endo-Beta-Mannanase, expressed

Chr.1	41496774	41496774	C	C	CCTCGCTA	INDEL	OS01G0944100	Intronic	NA	NA	LOC_Os01g71624	expressed protein
Chr.2	18581627	18581627	C	CCTCT	CCTCTCT	INDEL	OS02G0515200	Exonic	5UTR	NA	LOC_Os02g31080	hydroxyproline-rich glycoprotein family protein, putative, expressed
Chr.9	13569466	13569466	C	C	CCTG	INDEL	OS09G0392400	Exonic	3UTR	NA	LOC_Os09g22500	expressed protein
Chr.4	31181718	31181718	C	C	CCTT	INDEL	OS04G0614600	Intronic	NA	NA	LOC_Os04g52450	aminotransferase, putative, expressed
Chr9	10128849	10128864	CCTTCTTCTTCTTCTT	C	CCTTCTTCTTCTTCTTCTT	INDEL	OS09G0334500	Exonic	5UTR	NA	LOC_Os09g16510	WRKY74, expressed
Chr.6	28389304	28389304	C	C	CG	INDEL	OS06G0681200	Intronic	NA	NA	LOC_Os06g46740	early nodulin 20 precursor, putative, expressed
Chr.4	31220589	31220589	C	C	CG	INDEL	OS04G0615200	Intronic	NA	NA	LOC_Os04g52504	adhesive/proline-rich protein, putative, expressed
Chr.4	11237461	11237461	C	C	CG	INDEL	OS04G0269600	Exonic	CDS	FrameShift	LOC_Os04g20164	amine oxidase precursor, putative, expressed
Chr.4	24853838	24853838	C	C	CG	INDEL	OS04G0496800	Exonic	5UTR	NA	LOC_Os04g41950	calcium-binding mitochondrial protein anon-60Da, putative, expressed
Chr.3	1740609	1740609	C	C	CG	INDEL	OS03G0130700	Intronic	NA	NA	LOC_Os03g03860	expressed protein
Chr.3	539562	539562	C	C	CG	INDEL	OS03G0109300	Intronic	NA	NA	LOC_Os03g01880	possible lysine decarboxylase domain containing protein, expressed
Chr.3	29451822	29451822	C	C	CG	INDEL	OS03G0724600	Intronic	NA	NA	LOC_Os03g51470	expressed protein
Chr.2	20811612	20811612	C	C	CG	INDEL	OS02G0552000	Intronic	NA	NA	LOC_Os02g34690	transmembrane 9 superfamily member, putative, expressed
Chr7	9039117	9039121	CGAGA	C	CGA	INDEL	OS07G0259700	Exonic	5UTR	NA	LOC_Os07g15600	H-BTB6 - Bric-a-Brac, Tramtrack, Broad Complex BTB domain with H family conserved sequence, expressed
Chr7	3606240	3606240	C	C	CGA	INDEL	OS07G0167100	Intronic	NA	NA	LOC_Os07g07260	glutamine-dependent NAD, putative, expressed
Chr.1	15293202	15293202	C	C	CGAATTCCT	INDEL	OS01G0371500	Intronic	NA	NA	LOC_Os01g27390	glutathione S-transferase, putative, expressed
Chr.5	22306170	22306170	C	C	CGAG	INDEL	OS05G0454500	Exonic	5UTR	NA	LOC_Os05g38040	expressed protein
Chr3	7322544	7322552	CGAGAGAGA	C	CGAGAGA	INDEL	OS03G0238600	Exonic	5UTR	NA	LOC_Os03g13540	Ser/Thr protein phosphatase family protein, putative, expressed
Chr.5	7165140	7165142	CGA	C	CGAGAGAGA	INDEL	OS05G0215600	Intronic	NA	NA	LOC_Os05g12474	expressed protein
Chr.3	8704155	8704155	C	CGAGAGA	CGAGAGAGAGA	INDEL	OS03G0264400	Exonic	3UTR	NA	LOC_Os03g15780	anthranilate synthase component I-1, chloroplast precursor, putative, expressed
Chr.3	10043173	10043173	C	CGAGGGATG	CGAGGGACG	INDEL	OS03G0289800	Intronic	NA	NA	LOC_Os03g18030	leucoanthocyanidin dioxygenase, putative, expressed
Chr1	7280175	7280181	CGCGGCG	C	CGCG	INDEL	OS01G0231700	Exonic	5UTR	NA	LOC_Os01g13080	60S acidic ribosomal protein, putative, expressed
Chr.6	21893426	21893426	C	C	CGCG	INDEL	OS06G0567200	Exonic	CDS	InFrame	LOC_Os06g37080	L-ascorbate oxidase precursor, putative, expressed
Chr.5	19768879	19768885	CGGAGGA	C	CGGA	INDEL	OS05G0405900	Exonic	5UTR	NA	LOC_Os05g33610	expressed protein
Chr.6	13482235	13482235	C	C	CGT	INDEL	OS06G0338200	Intronic	NA	NA	LOC_Os06g23114	copper methylamine oxidase precursor, putative, expressed
Chr.12	21488136	21488136	C	C	CGT	INDEL	OS12G0538800	Exonic	5UTR	NA	LOC_Os12g35350	kelch repeat protein, putative, expressed
Chr12	18340716	18340718	CTT	C	CT	INDEL	OS12G0489100	Intronic	NA	NA	LOC_Os12g30540	ubiquitin carboxyl-terminal hydrolase, putative, expressed
Chr.1	267211	267211	C	CTT	CT	INDEL	OS01G0104800	Intronic	NA	NA	LOC_Os01g01510	sas10/Utp3 family protein, expressed
Chr.8	687365	687365	C	CTT	CT	INDEL	OS08G0112900	Intronic	NA	NA	LOC_Os08g02094	GDSL-like lipase/acylhydrolase, putative, expressed
Chr.3	23087861	23087861	C	CTT	CT	INDEL	OS03G0611200	Intronic	NA	NA	LOC_Os03g41510	oxidoreductase, aldo/keto reductase family protein, putative, expressed
Chr.5	23866434	23866434	C	CTT	CT	INDEL	OS05G0485300	Intronic	NA	NA	LOC_Os05g40700	transmembrane protein 56, putative, expressed
Chr7	26770463	26770463	C	CTT	CT	INDEL	OS07G0642800	Intronic	NA	NA	LOC_Os07g44830	proline-rich family protein, putative, expressed
Chr.4	29662407	29662407	C	CTT	CT	INDEL	OS04G0587400	Exonic	3UTR	NA	LOC_Os04g49748	purine permease, putative, expressed
Chr.9	16933257	16933257	C	CTTT	CT	INDEL	OS09G0451500	Intronic	NA	NA	LOC_Os09g27830	OsPDIL2-3 protein disulfide isomerase PDIL2-3, expressed
Chr.10	19093138	19093138	C	CTTT	CT	INDEL	OS10G0500600	Intronic	NA	NA	LOC_Os10g35710	expressed protein
Chr.2	17537727	17537727	C	CTTTT	CT	INDEL	OS02G0498300	Intronic	NA	NA	LOC_Os02g29510	DUF803 domain containing, putative, expressed
Chr.8	2514503	2514503	C	T	CT	INDEL	OS08G0144400	Exonic	5UTR	NA	LOC_Os08g04920	OsDegp11 - Putative Deg protease homologue, expressed
Chr7	18841609	18841609	C	C	CT	INDEL	OS07G0500300	Intronic	NA	NA	LOC_Os07g31720	GTPase activating protein, putative, expressed
Chr7	23651065	23651065	C	C	CT	INDEL	OS07G0583600	Intronic	NA	NA	LOC_Os07g39470	gibberellin response modulator protein, putative, expressed
Chr7	1568397	1568397	C	C	CT	INDEL	OS07G0129700	Intronic	NA	NA	LOC_Os07g03770	Homeobox domain containing protein, expressed
Chr7	4503614	4503614	C	C	CT	INDEL	OS07G0185200	Exonic	3UTR	NA	LOC_Os07g08760	START domain containing protein, putative, expressed
Chr.9	7281509	7281509	C	C	CT	INDEL	OS09G0298500	Intronic	NA	NA	LOC_Os09g12720	zinc finger, C3HC4 type domain containing protein, expressed
Chr.9	3061995	3061995	C	C	CT	INDEL	OS09G0240200	Intronic	NA	NA	LOC_Os09g06464	CCT/B-box zinc finger protein, putative, expressed
Chr.9	7198822	7198822	C	C	CT	INDEL	OS09G0297000	Intronic	NA	NA	LOC_Os09g12560	ferrochelatase-2, chloroplast precursor, putative, expressed
Chr.9	9568705	9568705	C	C	CT	INDEL	OS09G0325700	Exonic	3UTR	NA	LOC_Os09g15670	protein phosphatase 2C, putative, expressed
Chr.8	8765011	8765011	C	C	CT	INDEL	OS08G0243500	Exonic	3UTR	NA	LOC_Os08g14570	NADPH reductase, putative, expressed
Chr.8	21823127	21823127	C	C	CT	INDEL	OS08G0447000	Exonic	5UTR	NA	LOC_Os08g34720	D-3-phosphoglycerate dehydrogenase, chloroplast precursor, putative, expressed
Chr.6	21893705	21893705	C	C	CT	INDEL	OS06G0567200	Intronic	NA	NA	LOC_Os06g37080	L-ascorbate oxidase precursor, putative, expressed
Chr.6	1362377	1362377	C	C	CT	INDEL	OS06G0125200	Exonic	3UTR	NA	LOC_Os06g03520	DUF581 domain containing protein, expressed
Chr.5	23867181	23867181	C	C	CT	INDEL	OS05G0485300	Intronic	NA	NA	LOC_Os05g40700	transmembrane protein 56, putative, expressed
Chr.5	22239237	22239237	C	C	CT	INDEL	OS05G0453300	Exonic	3UTR	NA	LOC_Os05g37930	expressed protein
Chr.5	22072034	22072034	C	C	CT	INDEL	OS05G0449600	Exonic	3UTR	NA	LOC_Os05g37700	periplasmic beta-glucosidase precursor, putative, expressed
Chr.5	20631657	20631657	C	C	CT	INDEL	OS05G0420600	Intronic	NA	NA	LOC_Os05g34770	cytochrome c, putative, expressed
Chr.4	619213	619213	C	C	CT	INDEL	OS04G0110600	Intronic	NA	NA	LOC_Os04g02000	zinc finger family protein, putative, expressed
Chr.4	23318005	23318005	C	C	CT	INDEL	OS04G0466600	Intronic	NA	NA	LOC_Os04g39210	agmatine deiminase, putative, expressed
Chr.4	29043577	29043577	C	C	CT	INDEL	OS04G0576500	Intronic	NA	NA	LOC_Os04g48710	expressed protein
Chr.4	16003722	16003722	C	C	CT	INDEL	OS04G0339400	Intronic	NA	NA	LOC_Os04g27060	oxidoreductase, aldo/keto reductase family protein, putative, expressed
Chr.4	31441447	31441447	C	C	CT	INDEL	OS04G0619000	Intronic	NA	NA	LOC_Os04g52810	no apical meristem protein, putative, expressed
Chr.3	27489477	27489477	C	C	CT	INDEL	OS03G0689100	Intronic	NA	NA	LOC_Os03g48300	histidine acid phosphatase, putative, expressed
Chr.3	23369050	23369050	C	C	CT	INDEL	OS03G0616700	Exonic	3UTR	NA	LOC_Os03g42050	exportin-7-A, putative, expressed
Chr.3	15454642	15454642	C	C	CT	INDEL	OS03G0387500	Intronic	NA	NA	LOC_Os03g27003	expressed protein
Chr.2	2747271	2747271	C	C	CT	INDEL	OS02G0149800	Exonic	3UTR	NA	LOC_Os02g05630	protein phosphatase 2C, putative, expressed
Chr.2	9927216	9927216	C	C	CT	INDEL	OS02G0272900	Intronic	NA	NA	LOC_Os02g17304	resistance protein, putative, expressed
Chr.2	34046049	34046049	C	C	CT	INDEL	OS02G0799300	Exonic	3UTR	NA	LOC_Os02g55590	expressed protein
Chr.2	7224460	7224460	C	C	CT	INDEL	OS02G0228300	Exonic	5UTR	NA	LOC_Os02g13510	receptor-like protein kinase 5 precursor, putative, expressed
Chr.2	9973187	9973187	C	C	CT	INDEL	OS02G0273700	Intronic	NA	NA	LOC_Os02g17350	VHS and GAT domain containing protein, expressed
Chr.2	22687958	22687958	C	C	CT	INDEL	OS02G0588500	Intronic	NA	NA	LOC_Os02g37590	glycerophosphoryl diester phosphodiesterase family protein, putative, expressed
Chr.11	21297857	21297857	C	C	CT	INDEL	OS11G0570000	Exonic	CDS	FrameShift	LOC_Os11g36200	receptor-like protein kinase 2 precursor, putative, expressed
Chr.11	28955413	28955413	C	C	CT	INDEL	OS11G0707600	Intronic	NA	NA	LOC_Os11g48020	fatty acid hydroxylase, putative, expressed
Chr.11	4250315	4250315	C	C	CT	INDEL	OS11G0183900	Intronic	NA	NA	LOC_Os11g08100	eukaryotic aspartyl protease domain containing protein, expressed
Chr.11	19676175	19676175	C	C	CT	INDEL	OS11G0539200	Intronic	NA	NA	LOC_Os11g33270	glycosyl hydrolases family 16, putative, expressed
Chr.11	27488091	27488091	C	C	CT	INDEL	OS11G0679700	Intronic	NA	NA	LOC_Os11g45400	glycerol-3-phosphate acyltransferase, putative, expressed
Chr.11	28933732	28933732	C	C	CT	INDEL	OS11G0707000	Intronic	NA	NA	LOC_Os11g47970	AAA-type ATPase family protein, putative, expressed
Chr.10	12969806	12969806	C	C	CT	INDEL	OS10G0390500	Intronic	NA	NA	LOC_Os10g25130	aminotransferase, classes I and II, domain containing protein, expressed
Chr.1	22497034	22497034	C	C	CT	INDEL	OS01G0580800	Intronic	NA	NA	LOC_Os01g39890	transposon protein, putative, unclassified, expressed
Chr.1	266462	266462	C	C	CT	INDEL	OS01G0104800	Intronic	NA	NA	LOC_Os01g01510	sas10/Utp3 family protein, expressed
Chr.1	605459	605459	C	C	CT	INDEL	OS01G0111500	Exonic	5UTR	NA	LOC_Os01g02110	helix-loop-helix DNA-binding domain containing protein, expressed
Chr.1	8792160	8792160	C	C	CT	INDEL	OS01G0261100	Exonic	3UTR	NA	LOC_Os01g15630	zinc finger, C3HC4 type domain containing protein, expressed
Chr.1	23344604	23344604	C	C	CT	INDEL	OS01G0595600	Intronic	NA	NA	LOC_Os01g41240	hydrolase, alpha/beta fold family domain containing protein, expressed
Chr.1	27105824	27105824	C	C	CT	INDEL	OS01G0663800	Intronic	NA	NA	LOC_Os01g47430	protein of unknown function DUF1296 domain containing protein, expressed
Chr.1	28347237	28347237	C	C	CT	INDEL	OS01G0687300	Intronic	NA	NA	LOC_Os01g49310	expressed protein
Chr.1	31455518	31455518	C	C	CT	INDEL	OS01G0750500	Intronic	NA	NA	LOC_Os01g54670	coiled-coil domain-containing protein 25, putative, expressed
Chr.1	40813827	40813827	C	C	CT	INDEL	OS01G0930200	Exonic	3UTR	NA	LOC_Os01g70470	expressed protein
Chr.2	9976052	9976052	C	C	CTA	INDEL	OS02G0273700	Intronic	NA	NA	LOC_Os02g17350	VHS and GAT domain containing protein, expressed
Chr.2	713457	713457	C	C	CTA	INDEL	OS02G0113200	Intronic	NA	NA	LOC_Os02g02230	cytochrome P450, putative, expressed

Chr.10	21864047	21864047	C	C	CTA	INDEL	OS10G0556200	Intronic	NA	NA	LOC_Os10g40740	helix-loop-helix DNA-binding domain containing protein, expressed
Chr7	4264391	4264391	C	C	CTAG	INDEL	OS07G0180900	Intronic	NA	NA	LOC_Os07g08330	ribosomal protein L4, putative, expressed
Chr.4	5325029	5325029	C	C	CTATATATA	INDEL	OS04G0178300	Intronic	NA	NA	LOC_Os04g09900	ent-kaurene synthase, chloroplast precursor, putative, expressed
Chr.3	25002171	25002171	C	C	CTCA	INDEL	OS03G0646200	Exonic	CDS	InFrame	LOC_Os03g44430	ubiquitin carboxyl-terminal hydrolase, family 1, putative, expressed
Chr.5	2764051	2764051	C	C	CTCACCATTG	INDEL	OS05G0148300	Intronic	NA	NA	LOC_Os05g05560	expressed protein
Chr.6	1560887	1560887	C	C	CTCCT	INDEL	OS06G0129400	Exonic	3UTR	NA	LOC_Os06g03860	uncharacterized membrane protein, putative, expressed
Chr.8	3422554	3422554	C	C	CTCT	INDEL	OS08G0158500	Intronic	NA	NA	LOC_Os08g06200	stomatin-like protein 2, putative, expressed
Chr.3	36366542	36366542	C	C	CTGG	INDEL	OS03G0861400	Exonic	CDS	InFrame	LOC_Os03g64340	heavy metal-associated domain containing protein, expressed
Chr.4	31439681	31439681	C	C	CTGTAAGG	INDEL	OS04G0619000	Exonic	3UTR	NA	LOC_Os04g52810	no apical meristem protein, putative, expressed
Chr3	2125565	2125566	CT	C	CTT	INDEL	OS03G0138500	Exonic	3UTR	NA	LOC_Os03g04550	expressed protein
Chr11	7127786	7127787	CT	C	CTT	INDEL	OS11G0234200	Intronic	NA	NA	LOC_Os11g12650	PHD-finger domain containing protein, putative, expressed
Chr2	8837300	8837301	CT	C	CTT	INDEL	OS02G0256100	Exonic	3UTR	NA	LOC_Os02g15690	polygalacturonase, putative, expressed
Chr1	9233238	9233239	CT	C	CTT	INDEL	OS01G0268300	Intronic	NA	NA	LOC_Os01g16290	DNA gyrase subunit B, chloroplast/mitochondrial precursor, putative, expressed
Chr9	9359175	9359176	CT	C	CTT	INDEL	OS09G0321900	Intronic	NA	NA	LOC_Os09g15320	ubiquitin-conjugating enzyme, putative, expressed
Chr7	6969172	6969172	C	CT	CTT	INDEL	OS07G0225300	Exonic	3UTR	NA	LOC_Os07g12340	NAC domain-containing protein 67, putative, expressed
Chr.8	20425353	20425353	C	CT	CTT	INDEL	OS08G0425200	Intronic	NA	NA	LOC_Os08g32930	expressed protein
Chr.2	3829163	3829163	C	CTTT	CTT	INDEL	OS02G0170100	Intronic	NA	NA	LOC_Os02g07410	glycine cleavage system H protein, putative, expressed
Chr.3	10996663	10996663	C	CTTT	CTT	INDEL	OS03G0308800	Intronic	NA	NA	LOC_Os03g19560	tetratricopeptide repeat domain containing protein, expressed
Chr.5	16269724	16269724	C	C	CTT	INDEL	OS05G0345500	Intronic	NA	NA	LOC_Os05g27880	BBF1 - 2 Bric-a-Brac, Tramtrack, Broad Complex BTB domains with a F5/8 type C discoidin domain, expressed
Chr.3	3104845	3104845	C	C	CTT	INDEL	OS03G0157800	Intronic	NA	NA	LOC_Os03g06190	3-5 exonuclease family protein, putative, expressed
Chr.11	4247912	4247912	C	C	CTT	INDEL	OS11G0183900	Intronic	NA	NA	LOC_Os11g08100	eukaryotic aspartyl protease domain containing protein, expressed
Chr.5	25546332	25546332	C	C	CTTAT	INDEL	OS05G0515200	Exonic	3UTR	NA	LOC_Os05g43910	cytochrome P450, putative, expressed
Chr.4	13645235	13645235	C	C	CTTCT	INDEL	OS04G0304200	Intronic	NA	NA	LOC_Os04g23890	AGC_PVPK_like_kin82y.10 - ACG kinases include homologs to PKA, PKG and PKC, expressed
Chr.4	25195499	25195499	C	C	CTTGAA	INDEL	OS04G0504800	Intronic	NA	NA	LOC_Os04g42600	polyadenylate-binding protein, putative, expressed
Chr6	1555467	1555479	CTTGGGGTTGGGG	C	CTTGGGG	INDEL	OS06G0129400	Intronic	NA	NA	LOC_Os06g03860	uncharacterized membrane protein, putative, expressed
Chr2	10739981	10739983	CTT	C	CTTT	INDEL	OS02G0285800	Intronic	NA	NA	LOC_Os02g18450	GTP-binding protein typA/bipA, putative, expressed
Chr.2	34223737	34223737	C	CT	CTTT	INDEL	OS02G0802700	Intronic	NA	NA	LOC_Os02g55910	monogalactosyldiacylglycerol synthase, putative, expressed
Chr.11	23079878	23079878	C	CTTTT	CTTT	INDEL	OS11G0600900	Intronic	NA	NA	LOC_Os11g38810	mannose-6-phosphate isomerase, putative, expressed
Chr.11	28855753	28855753	C	CTTTT	CTTT	INDEL	OS11G0704800	Intronic	NA	NA	LOC_Os11g47840	OsRhmbd18 - Putative Rhomboid homologue, expressed
Chr.5	7166337	7166337	C	C	CTTT	INDEL	OS05G0215600	Intronic	NA	NA	LOC_Os05g12474	expressed protein
Chr7	2647011	2647011	C	C	CTTTT	INDEL	OS07G0150700	Intronic	NA	NA	LOC_Os07g05620	CAMK_KIN1/SNF1/Nim1_like.28 - CAMK includes calcium/calmodulin depedent protein kinases, expressed
Chr.1	648289	648289	C	C	CTTTT	INDEL	OS01G0112500	Exonic	3UTR	NA	LOC_Os01g02200	armadillo/beta-catenin repeat family protein, putative, expressed
Chr.4	22018474	22018474	C	CTTTTTTTT	CTTTTTT	INDEL	OS04G0442200	Intronic	NA	NA	LOC_Os04g36062	expressed protein
Chr.10	2914104	2914104	C	A	G	SNP	OS10G0148400	Exonic	CDS	Silent	LOC_Os10g05780	POT domain containing peptide transporter, putative, expressed
Chr.10	3754509	3754509	T	A	G	SNP	OS10G0159300	Exonic	3UTR	NA	LOC_Os10g07160	retrotransposon, putative, centromere-specific, expressed
Chr.11	4972907	4972907	T	A	G	SNP	OS11G0199200	Intronic	NA	NA	LOC_Os11g09280	OsPDIL1-1 protein disulfide isomerase PDIL1-1, expressed
Chr.10	10303418	10303418	C	A	G	SNP	OS10G0345100	Intronic	NA	NA	LOC_Os10g20470	MATE efflux family protein, putative, expressed
Chr7	18763524	18763524	T	A	G	SNP	OS07G0499300	Exonic	3UTR	NA	LOC_Os07g31599	expressed protein
Chr.6	23112736	23112736	T	A	G	SNP	OS06G0589300	Intronic	NA	NA	LOC_Os06g38950	ABC transporter, ATP-binding protein, putative, expressed
Chr.1	34619005	34619005	T	A	G	SNP	OS01G0813900	Exonic	3UTR	NA	LOC_Os01g59850	GTPase-activating protein, putative, expressed
Chr.9	13568319	13568319	A	C	G	SNP	OS09G0392400	Intronic	NA	NA	LOC_Os09g22500	expressed protein
Chr.1	30159705	30159705	A	C	G	SNP	OS01G0723200	Exonic	5UTR	NA	LOC_Os01g52490	40S ribosomal protein S24, putative, expressed
Chr.3	21635505	21635505	A	G	G	SNP	OS03G0586500	Intronic	NA	NA	LOC_Os03g38950	chloroplast post-illumination chlorophyll fluorescence increase protein, putative, expressed
Chr7	26776370	26776372	GAA	GA	G	INDEL	OS07G0642900	Intronic	NA	NA	LOC_Os07g44840	bacterial transferase hexapeptide domain containing protein, expressed
Chr.9	18351832	18351833	GA	GAA	G	INDEL	OS09G0478600	Exonic	3UTR	NA	LOC_Os09g30160	zinc finger, C3HC4 type domain containing protein, expressed
Chr.4	33162414	33162415	GA	GAA	G	INDEL	OS04G0650700	Exonic	3UTR	NA	LOC_Os04g55710	transposon protein, putative, unclassified, expressed
Chr.1	42066583	42066584	GA	GAA	G	INDEL	OS01G0955000	Exonic	3UTR	NA	LOC_Os01g72520	phosphoesterase family protein, putative, expressed
Chr.1	27183814	27183818	GGAGA	GGA	G	INDEL	OS01G0665300	Exonic	5UTR	NA	LOC_Os01g47540	NLI interacting factor-like phosphatase, putative, expressed
Chr.5	1668176	1668182	GGACGAC	GGACGACGACGACGACGAC	G	INDEL	OS05G0128300	Exonic	CDS	InFrame	LOC_Os05g03770	expressed protein
Chr.1	37523786	37523788	GGA	GGAGAGAGAGA	G	INDEL	OS01G0866500	Intronic	NA	NA	LOC_Os01g64670	soluble inorganic pyrophosphatase, putative, expressed
Chr.3	24600190	24600196	GGCTGCT	GGCTGCT	G	INDEL	OS03G0639800	Exonic	5UTR	NA	LOC_Os03g43860	SNF7 domain containing protein, putative, expressed
Chr.4	20320102	20320104	GTA	GTATATATATATA	G	INDEL	OS04G0411400	Intronic	NA	NA	LOC_Os04g33570	RCN4 Centroradialis-like1 homologous to TFL1 gene; contains Pfam profile PF01161: Phosphatidylethanolamine-binding protein,
Chr.2	5154570	5154575	GTCAGC	GTCAGCTCAGC	G	INDEL	OS02G0192500	Intronic	NA	NA	LOC_Os02g09930	CSLA1 - cellulose synthase-like family A; mannan synthase, expressed
Chr.5	3757360	3757361	GT	GTT	G	INDEL	OS05G0163900	Intronic	NA	NA	LOC_Os05g07120	basic helix-loop-helix, putative, expressed
Chr.1	9229166	9229167	GT	GTT	G	INDEL	OS01G0268300	Intronic	NA	NA	LOC_Os01g16290	DNA gyrase subunit B, chloroplast/mitochondrial precursor, putative, expressed
Chr.5	13835193	13835195	GTT	GTTTT	G	INDEL	OS05G0305900	Intronic	NA	NA	LOC_Os05g24010	receptor protein kinase-like, putative, expressed
Chr.2	7221858	7221858	A	T	G	SNP	OS02G0228300	Exonic	CDS	Silent	LOC_Os02g13510	receptor-like protein kinase 5 precursor, putative, expressed
Chr.3	10039416	10039416	C	T	G	SNP	OS03G0289800	Exonic	3UTR	NA	LOC_Os03g18030	leucoanthocyanidin dioxygenase, putative, expressed
Chr.5	17583827	17583827	A	T	G	SNP	OS05G0366300	Intronic	NA	NA	LOC_Os05g30340	expressed protein
Chr7	21245684	21245684	A	T	G	SNP	OS07G0539400	Intronic	NA	NA	LOC_Os07g35520	glucan endo-1,3-beta-glucosidase precursor, putative, expressed
Chr.1	22498668	22498668	A	T	G	SNP	OS01G0580800	Intronic	NA	NA	LOC_Os01g39890	transposon protein, putative, unclassified, expressed
Chr.3	23372310	23372310	C	T	G	SNP	OS03G0616700	Intronic	NA	NA	LOC_Os03g42050	exportin-7-A, putative, expressed
Chr.5	25232472	25232472	A	T	G	SNP	OS05G0509500	Intronic	NA	NA	LOC_Os05g43390	signal recognition particle 54 kDa protein, putative, expressed
Chr.4	30288731	30288731	A	T	G	SNP	OS04G0600200	Intronic	NA	NA	LOC_Os04g51150	transposon protein, putative, unclassified, expressed
Chr.4	5847049	5847049	A	A	G	SNP	OS04G0186400	Exonic	5UTR	NA	LOC_Os04g10750	inorganic phosphate transporter, putative, expressed
Chr.4	5846959	5846959	A	A	G	SNP	OS04G0186400	Exonic	5UTR	NA	LOC_Os04g10750	inorganic phosphate transporter, putative, expressed
Chr.4	5846765	5846765	A	A	G	SNP	OS04G0186400	Exonic	5UTR	NA	LOC_Os04g10750	inorganic phosphate transporter, putative, expressed
Chr.4	5846511	5846511	A	A	G	SNP	OS04G0186400	Exonic	5UTR	NA	LOC_Os04g10750	inorganic phosphate transporter, putative, expressed
Chr.4	5846482	5846482	A	A	G	SNP	OS04G0186400	Exonic	5UTR	NA	LOC_Os04g10750	inorganic phosphate transporter, putative, expressed
Chr.4	5849133	5849133	C	C	G	SNP	OS04G0186400	Exonic	3UTR	NA	LOC_Os04g10750	inorganic phosphate transporter, putative, expressed
Chr.4	5849143	5849143	A	A	G	SNP	OS04G0186400	Exonic	3UTR	NA	LOC_Os04g10750	inorganic phosphate transporter, putative, expressed
Chr.4	5849004	5849004	A	A	G	SNP	OS04G0186400	Exonic	CDS	Silent	LOC_Os04g10750	inorganic phosphate transporter, putative, expressed
Chr.4	5846802	5846804	GAT	GAT	G	INDEL	OS04G0186400	Exonic	5UTR	NA	LOC_Os04g10750	inorganic phosphate transporter, putative, expressed
Chr7	4264808	4264808	T	T	G	SNP	OS07G0180900	Intronic	NA	NA	LOC_Os07g08330	ribosomal protein L4, putative, expressed
Chr7	4687396	4687396	T	T	G	SNP	OS07G0187700	Exonic	3UTR	NA	LOC_Os07g09000	WD domain, G-beta repeat domain containing protein, expressed
Chr7	8966532	8966532	T	T	G	SNP	OS07G0258400	Intronic	NA	NA	LOC_Os07g15460	metal transporter Nramp6, putative, expressed
Chr7	18336611	18336611	T	T	G	SNP	OS07G0492000	Intronic	NA	NA	LOC_Os07g30970	nucleoside diphosphate kinase, putative, expressed
Chr7	21246514	21246514	T	T	G	SNP	OS07G0539400	Exonic	CDS	Missense	LOC_Os07g35520	glucan endo-1,3-beta-glucosidase precursor, putative, expressed
Chr7	22889455	22889455	T	T	G	SNP	OS07G0569100	Exonic	3UTR	NA	LOC_Os07g38170	remorin, putative, expressed
Chr7	26776821	26776821	T	T	G	SNP	OS07G0642900	Exonic	CDS	Missense	LOC_Os07g44840	bacterial transferase hexapeptide domain containing protein, expressed
Chr7	27172894	27172894	T	T	G	SNP	OS07G0650100	Exonic	CDS	Missense	LOC_Os07g45550	expressed protein
Chr7	27725017	27725017	T	T	G	SNP	OS07G0658400	Exonic	CDS	NA	LOC_Os07g46460	ferredoxin-dependent glutamate synthase, chloroplast precursor, putative, expressed
Chr7	27725694	27725694	T	T	G	SNP	OS07G0658400	Intronic	NA	NA	LOC_Os07g46460	ferredoxin-dependent glutamate synthase, chloroplast precursor, putative, expressed
Chr7	27729683	27729683	T	T	G	SNP	OS07G0658400	Intronic	NA	NA	LOC_Os07g46460	ferredoxin-dependent glutamate synthase, chloroplast precursor, putative, expressed
Chr7	27729726	27729726	T	T	G	SNP	OS07G0658400	Intronic	NA	NA	LOC_Os07g46460	ferredoxin-dependent glutamate synthase, chloroplast precursor, putative, expressed

Chr7	28124395	28124395	T	T	G	SNP	OS07G0665900	Exonic	CDS	Missense	LOC_Os07g47040	expressed protein
Chr7	8968842	8968842	T	T	G	SNP	OS07G0258400	Intronic	NA	NA	LOC_Os07g15460	metal transporter Nramp6, putative, expressed
Chr7	18840604	18840604	T	T	G	SNP	OS07G0500300	Intronic	NA	NA	LOC_Os07g31720	GTPase activating protein, putative, expressed
Chr7	18840844	18840844	T	T	G	SNP	OS07G0500300	Intronic	NA	NA	LOC_Os07g31720	GTPase activating protein, putative, expressed
Chr7	18840889	18840889	T	T	G	SNP	OS07G0500300	Intronic	NA	NA	LOC_Os07g31720	GTPase activating protein, putative, expressed
Chr7	18841542	18841542	T	T	G	SNP	OS07G0500300	Intronic	NA	NA	LOC_Os07g31720	GTPase activating protein, putative, expressed
Chr7	7107870	7107870	C	C	G	SNP	OS07G0227200	Exonic	3UTR	NA	LOC_Os07g12470	expressed protein
Chr7	7109317	7109317	C	C	G	SNP	OS07G0227200	Exonic	3UTR	NA	LOC_Os07g12470	expressed protein
Chr7	8970392	8970392	C	C	G	SNP	OS07G0258400	Exonic	CDS	Missense	LOC_Os07g15460	metal transporter Nramp6, putative, expressed
Chr7	18840519	18840519	C	C	G	SNP	OS07G0500300	Intronic	NA	NA	LOC_Os07g31720	GTPase activating protein, putative, expressed
Chr7	18840587	18840587	C	C	G	SNP	OS07G0500300	Intronic	NA	NA	LOC_Os07g31720	GTPase activating protein, putative, expressed
Chr7	21380932	21380932	C	C	G	SNP	OS07G0541400	Exonic	CDS	Missense	LOC_Os07g35690	TKL_IRAK_DUF26-lc.3 - DUF26 kinases have homology to DUF26 containing loci, expressed
Chr7	21381131	21381131	C	C	G	SNP	OS07G0541400	Exonic	3UTR	NA	LOC_Os07g35690	TKL_IRAK_DUF26-lc.3 - DUF26 kinases have homology to DUF26 containing loci, expressed
Chr7	27172700	27172700	C	C	G	SNP	OS07G0650100	Exonic	CDS	Missense	LOC_Os07g45550	expressed protein
Chr7	7109972	7109972	A	A	G	SNP	OS07G0227200	Exonic	3UTR	NA	LOC_Os07g12470	expressed protein
Chr7	17412492	17412492	A	A	G	SNP	OS07G0479300	Exonic	CDS	Silent	LOC_Os07g29620	OsSCP39 - Putative Serine Carboxypeptidase homologue, expressed
Chr7	18840580	18840580	A	A	G	SNP	OS07G0500300	Intronic	NA	NA	LOC_Os07g31720	GTPase activating protein, putative, expressed
Chr7	18841107	18841107	A	A	G	SNP	OS07G0500300	Intronic	NA	NA	LOC_Os07g31720	GTPase activating protein, putative, expressed
Chr7	18841466	18841466	A	A	G	SNP	OS07G0500300	Intronic	NA	NA	LOC_Os07g31720	GTPase activating protein, putative, expressed
Chr7	18841713	18841713	A	A	G	SNP	OS07G0500300	Exonic	CDS	Silent	LOC_Os07g31720	GTPase activating protein, putative, expressed
Chr7	27174437	27174437	A	A	G	SNP	OS07G0650100	Exonic	CDS	Missense	LOC_Os07g45550	expressed protein
Chr7	27174544	27174544	A	A	G	SNP	OS07G0650100	Exonic	CDS	Silent	LOC_Os07g45550	expressed protein
Chr7	8075862	8075866	GCTCT	GCTCT	G	INDEL	OS07G0245100	Exonic	5UTR	NA	LOC_Os07g14150	cytidine deaminase, putative, expressed
Chr7	4265234	4265234	C	C	G	SNP	OS07G0180900	Intronic	NA	NA	LOC_Os07g08330	ribosomal protein L4, putative, expressed
Chr7	8967192	8967192	C	C	G	SNP	OS07G0258400	Intronic	NA	NA	LOC_Os07g15460	metal transporter Nramp6, putative, expressed
Chr7	13100576	13100576	C	C	G	SNP	OS07G0415200	Intronic	NA	NA	LOC_Os07g23244	ribosomal protein L23 family protein, putative, expressed
Chr7	26773123	26773123	C	C	G	SNP	OS07G0642800	Intronic	NA	NA	LOC_Os07g44830	proline-rich family protein, putative, expressed
Chr7	27725250	27725250	C	C	G	SNP	OS07G0658400	Intronic	NA	NA	LOC_Os07g46460	ferredoxin-dependent glutamate synthase, chloroplast precursor, putative, expressed
Chr7	27727709	27727709	C	C	G	SNP	OS07G0658400	Intronic	NA	NA	LOC_Os07g46460	ferredoxin-dependent glutamate synthase, chloroplast precursor, putative, expressed
Chr7	27727932	27727932	C	C	G	SNP	OS07G0658400	Intronic	NA	NA	LOC_Os07g46460	ferredoxin-dependent glutamate synthase, chloroplast precursor, putative, expressed
Chr7	27728566	27728566	C	C	G	SNP	OS07G0658400	Intronic	NA	NA	LOC_Os07g46460	ferredoxin-dependent glutamate synthase, chloroplast precursor, putative, expressed
Chr7	27730066	27730066	C	C	G	SNP	OS07G0658400	Intronic	NA	NA	LOC_Os07g46460	ferredoxin-dependent glutamate synthase, chloroplast precursor, putative, expressed
Chr7	27730700	27730700	C	C	G	SNP	OS07G0658400	Intronic	NA	NA	LOC_Os07g46460	ferredoxin-dependent glutamate synthase, chloroplast precursor, putative, expressed
Chr7	27732505	27732505	C	C	G	SNP	OS07G0658400	Intronic	NA	NA	LOC_Os07g46460	ferredoxin-dependent glutamate synthase, chloroplast precursor, putative, expressed
Chr7	27736674	27736674	C	C	G	SNP	OS07G0658400	Intronic	NA	NA	LOC_Os07g46460	ferredoxin-dependent glutamate synthase, chloroplast precursor, putative, expressed
Chr7	27737249	27737249	C	C	G	SNP	OS07G0658400	Intronic	NA	NA	LOC_Os07g46460	ferredoxin-dependent glutamate synthase, chloroplast precursor, putative, expressed
Chr7	1568456	1568456	A	A	G	SNP	OS07G0129700	Intronic	NA	NA	LOC_Os07g03770	Homeobox domain containing protein, expressed
Chr7	2194565	2194565	A	A	G	SNP	OS07G0142900	Intronic	NA	NA	LOC_Os07g04990	oxidoreductase, aldo/keto reductase family protein, putative, expressed
Chr7	2196768	2196768	A	A	G	SNP	OS07G0142900	Exonic	3UTR	NA	LOC_Os07g04990	oxidoreductase, aldo/keto reductase family protein, putative, expressed
Chr7	2314200	2314200	A	A	G	SNP	OS07G0145400	Exonic	CDS	Silent	LOC_Os07g05190	leucine-rich repeat family protein, putative, expressed
Chr7	3606626	3606626	A	A	G	SNP	OS07G0167100	Intronic	NA	NA	LOC_Os07g07260	glutamine-dependent NAD, putative, expressed
Chr7	4264381	4264381	A	A	G	SNP	OS07G0180900	Intronic	NA	NA	LOC_Os07g08330	ribosomal protein L4, putative, expressed
Chr7	4264387	4264387	A	A	G	SNP	OS07G0180900	Intronic	NA	NA	LOC_Os07g08330	ribosomal protein L4, putative, expressed
Chr7	4264959	4264959	A	A	G	SNP	OS07G0180900	Intronic	NA	NA	LOC_Os07g08330	ribosomal protein L4, putative, expressed
Chr7	4686664	4686664	A	A	G	SNP	OS07G0187700	Intronic	NA	NA	LOC_Os07g09000	WD domain, G-beta repeat domain containing protein, expressed
Chr7	19231775	19231775	A	A	G	SNP	OS07G0506700	Exonic	3UTR	NA	LOC_Os07g32350	WD domain, G-beta repeat domain containing protein, expressed
Chr7	21246505	21246505	A	A	G	SNP	OS07G0539400	Exonic	CDS	Missense	LOC_Os07g35520	glucan endo-1,3-beta-glucosidase precursor, putative, expressed
Chr7	21280677	21280677	A	A	G	SNP	OS07G0539900	Intronic	NA	NA	LOC_Os07g35560	glucan endo-1,3-beta-glucosidase precursor, putative, expressed
Chr7	21280683	21280683	A	A	G	SNP	OS07G0539900	Intronic	NA	NA	LOC_Os07g35560	glucan endo-1,3-beta-glucosidase precursor, putative, expressed
Chr7	21280849	21280849	A	A	G	SNP	OS07G0539900	Exonic	CDS	Silent	LOC_Os07g35560	glucan endo-1,3-beta-glucosidase precursor, putative, expressed
Chr7	21280948	21280948	A	A	G	SNP	OS07G0539900	Exonic	CDS	Silent	LOC_Os07g35560	glucan endo-1,3-beta-glucosidase precursor, putative, expressed
Chr7	21281071	21281071	A	A	G	SNP	OS07G0539900	Exonic	CDS	Missense	LOC_Os07g35560	glucan endo-1,3-beta-glucosidase precursor, putative, expressed
Chr7	21281370	21281370	A	A	G	SNP	OS07G0539900	Exonic	CDS	Silent	LOC_Os07g35560	glucan endo-1,3-beta-glucosidase precursor, putative, expressed
Chr7	21281861	21281861	A	A	G	SNP	OS07G0539900	Exonic	CDS	Silent	LOC_Os07g35560	glucan endo-1,3-beta-glucosidase precursor, putative, expressed
Chr7	21281913	21281913	A	A	G	SNP	OS07G0539900	Exonic	CDS	Silent	LOC_Os07g35560	glucan endo-1,3-beta-glucosidase precursor, putative, expressed
Chr7	21380036	21380036	A	A	G	SNP	OS07G0541400	Intronic	NA	NA	LOC_Os07g35690	TKL_IRAK_DUF26-lc.3 - DUF26 kinases have homology to DUF26 containing loci, expressed
Chr7	21415579	21415579	A	A	G	SNP	OS07G0541900	Exonic	CDS	Silent	LOC_Os07g35750	TKL_IRAK_DUF26-ld.3 - DUF26 kinases have homology to DUF26 containing loci, expressed
Chr7	22412746	22412746	A	A	G	SNP	OS07G0561300	Intronic	NA	NA	LOC_Os07g37400	OsFBX257 - F-box domain containing protein, expressed
Chr7	23652028	23652028	A	A	G	SNP	OS07G0583600	Exonic	5UTR	NA	LOC_Os07g39470	gibberellin response modulator protein, putative, expressed
Chr7	23929176	23929176	A	A	G	SNP	OS07G0588000	Exonic	CDS	Missense	LOC_Os07g39900	interferon-related developmental regulator, putative, expressed
Chr7	24398353	24398353	A	A	G	SNP	OS07G0598200	Intronic	NA	NA	LOC_Os07g40710	circadian clock coupling factor-related, putative, expressed
Chr7	24655376	24655376	A	A	G	SNP	OS07G0603100	Exonic	CDS	Silent	LOC_Os07g41180	RNA-binding protein-like, putative, expressed
Chr7	24656503	24656503	A	A	G	SNP	OS07G0603100	Intronic	NA	NA	LOC_Os07g41180	RNA-binding protein-like, putative, expressed
Chr7	24658103	24658103	A	A	G	SNP	OS07G0603100	Intronic	NA	NA	LOC_Os07g41180	RNA-binding protein-like, putative, expressed
Chr7	26104645	26104645	A	A	G	SNP	OS07G0629500	Exonic	5UTR	NA	LOC_Os07g43604	expressed protein
Chr7	26631807	26631807	A	A	G	SNP	OS07G0639900	Intronic	NA	NA	LOC_Os07g44630	thymidylate kinase, putative, expressed
Chr7	26632087	26632087	A	A	G	SNP	OS07G0639900	Intronic	NA	NA	LOC_Os07g44630	thymidylate kinase, putative, expressed
Chr7	26632279	26632279	A	A	G	SNP	OS07G0639900	Intronic	NA	NA	LOC_Os07g44630	thymidylate kinase, putative, expressed
Chr7	26771516	26771516	A	A	G	SNP	OS07G0642800	Intronic	NA	NA	LOC_Os07g44830	proline-rich family protein, putative, expressed
Chr7	27723386	27723386	A	A	G	SNP	OS07G0658400	Exonic	3UTR	NA	LOC_Os07g46460	ferredoxin-dependent glutamate synthase, chloroplast precursor, putative, expressed
Chr7	27723805	27723805	A	A	G	SNP	OS07G0658400	Intronic	NA	NA	LOC_Os07g46460	ferredoxin-dependent glutamate synthase, chloroplast precursor, putative, expressed
Chr7	27725069	27725069	A	A	G	SNP	OS07G0658400	Intronic	NA	NA	LOC_Os07g46460	ferredoxin-dependent glutamate synthase, chloroplast precursor, putative, expressed
Chr7	27725279	27725279	A	A	G	SNP	OS07G0658400	Exonic	CDS	NA	LOC_Os07g46460	ferredoxin-dependent glutamate synthase, chloroplast precursor, putative, expressed
Chr7	27725318	27725318	A	A	G	SNP	OS07G0658400	Exonic	CDS	NA	LOC_Os07g46460	ferredoxin-dependent glutamate synthase, chloroplast precursor, putative, expressed
Chr7	27725606	27725606	A	A	G	SNP	OS07G0658400	Intronic	NA	NA	LOC_Os07g46460	ferredoxin-dependent glutamate synthase, chloroplast precursor, putative, expressed
Chr7	27726370	27726370	A	A	G	SNP	OS07G0658400	Intronic	NA	NA	LOC_Os07g46460	ferredoxin-dependent glutamate synthase, chloroplast precursor, putative, expressed
Chr7	27727081	27727081	A	A	G	SNP	OS07G0658400	Intronic	NA	NA	LOC_Os07g46460	ferredoxin-dependent glutamate synthase, chloroplast precursor, putative, expressed
Chr7	27727461	27727461	A	A	G	SNP	OS07G0658400	Intronic	NA	NA	LOC_Os07g46460	ferredoxin-dependent glutamate synthase, chloroplast precursor, putative, expressed
Chr7	27727593	27727593	A	A	G	SNP	OS07G0658400	Intronic	NA	NA	LOC_Os07g46460	ferredoxin-dependent glutamate synthase, chloroplast precursor, putative, expressed
Chr7	27727733	27727733	A	A	G	SNP	OS07G0658400	Intronic	NA	NA	LOC_Os07g46460	ferredoxin-dependent glutamate synthase, chloroplast precursor, putative, expressed
Chr7	27728004	27728004	A	A	G	SNP	OS07G0658400	Intronic	NA	NA	LOC_Os07g46460	ferredoxin-dependent glutamate synthase, chloroplast precursor, putative, expressed
Chr7	27728152	27728152	A	A	G	SNP	OS07G0658400	Exonic	CDS	NA	LOC_Os07g46460	ferredoxin-dependent glutamate synthase, chloroplast precursor, putative, expressed
Chr7	27728404	27728404	A	A	G	SNP	OS07G0658400	Exonic	CDS	NA	LOC_Os07g46460	ferredoxin-dependent glutamate synthase, chloroplast precursor, putative, expressed
Chr7	27728486	27728486	A	A	G	SNP	OS07G0658400	Intronic	NA	NA	LOC_Os07g46460	ferredoxin-dependent glutamate synthase, chloroplast precursor, putative, expressed
Chr7	27728625	27728625	A	A	G	SNP	OS07G0658400	Intronic	NA	NA	LOC_Os07g46460	ferredoxin-dependent glutamate synthase, chloroplast precursor, putative, expressed

Chr7	27728663	27728663	A	A	G	SNP	OS07G0658400	Intronic	NA	NA	LOC_Os07g46460	ferredoxin-dependent glutamate synthase, chloroplast precursor, putative, expressed
Chr7	27729255	27729255	A	A	G	SNP	OS07G0658400	Intronic	NA	NA	LOC_Os07g46460	ferredoxin-dependent glutamate synthase, chloroplast precursor, putative, expressed
Chr7	27729664	27729664	A	A	G	SNP	OS07G0658400	Intronic	NA	NA	LOC_Os07g46460	ferredoxin-dependent glutamate synthase, chloroplast precursor, putative, expressed
Chr7	27729767	27729767	A	A	G	SNP	OS07G0658400	Intronic	NA	NA	LOC_Os07g46460	ferredoxin-dependent glutamate synthase, chloroplast precursor, putative, expressed
Chr7	27730182	27730182	A	A	G	SNP	OS07G0658400	Intronic	NA	NA	LOC_Os07g46460	ferredoxin-dependent glutamate synthase, chloroplast precursor, putative, expressed
Chr7	27730940	27730940	A	A	G	SNP	OS07G0658400	Intronic	NA	NA	LOC_Os07g46460	ferredoxin-dependent glutamate synthase, chloroplast precursor, putative, expressed
Chr7	27730961	27730961	A	A	G	SNP	OS07G0658400	Intronic	NA	NA	LOC_Os07g46460	ferredoxin-dependent glutamate synthase, chloroplast precursor, putative, expressed
Chr7	27731675	27731675	A	A	G	SNP	OS07G0658400	Intronic	NA	NA	LOC_Os07g46460	ferredoxin-dependent glutamate synthase, chloroplast precursor, putative, expressed
Chr7	27731817	27731817	A	A	G	SNP	OS07G0658400	Intronic	NA	NA	LOC_Os07g46460	ferredoxin-dependent glutamate synthase, chloroplast precursor, putative, expressed
Chr7	27732542	27732542	A	A	G	SNP	OS07G0658400	Intronic	NA	NA	LOC_Os07g46460	ferredoxin-dependent glutamate synthase, chloroplast precursor, putative, expressed
Chr7	27732759	27732759	A	A	G	SNP	OS07G0658400	Intronic	NA	NA	LOC_Os07g46460	ferredoxin-dependent glutamate synthase, chloroplast precursor, putative, expressed
Chr7	27733168	27733168	A	A	G	SNP	OS07G0658400	Intronic	NA	NA	LOC_Os07g46460	ferredoxin-dependent glutamate synthase, chloroplast precursor, putative, expressed
Chr7	27733396	27733396	A	A	G	SNP	OS07G0658400	Intronic	NA	NA	LOC_Os07g46460	ferredoxin-dependent glutamate synthase, chloroplast precursor, putative, expressed
Chr7	27734820	27734820	A	A	G	SNP	OS07G0658400	Exonic	CDS	NA	LOC_Os07g46460	ferredoxin-dependent glutamate synthase, chloroplast precursor, putative, expressed
Chr7	27735203	27735203	A	A	G	SNP	OS07G0658400	Intronic	NA	NA	LOC_Os07g46460	ferredoxin-dependent glutamate synthase, chloroplast precursor, putative, expressed
Chr7	27736775	27736775	A	A	G	SNP	OS07G0658400	Intronic	NA	NA	LOC_Os07g46460	ferredoxin-dependent glutamate synthase, chloroplast precursor, putative, expressed
Chr7	27736850	27736850	A	A	G	SNP	OS07G0658400	Exonic	CDS	NA	LOC_Os07g46460	ferredoxin-dependent glutamate synthase, chloroplast precursor, putative, expressed
Chr7	27737417	27737417	A	A	G	SNP	OS07G0658400	Intronic	NA	NA	LOC_Os07g46460	ferredoxin-dependent glutamate synthase, chloroplast precursor, putative, expressed
Chr7	27737475	27737475	A	A	G	SNP	OS07G0658400	Intronic	NA	NA	LOC_Os07g46460	ferredoxin-dependent glutamate synthase, chloroplast precursor, putative, expressed
Chr7	926212	926212	A	A	G	SNP	OS07G0116900	Intronic	NA	NA	LOC_Os07g02570	NB-ARC domain containing protein, expressed
Chr7	1567092	1567092	C	C	G	SNP	OS07G0129700	Exonic	CDS	Missense	LOC_Os07g03770	Homeobox domain containing protein, expressed
Chr7	3603125	3603125	A	A	G	SNP	OS07G0167100	Intronic	NA	NA	LOC_Os07g07260	glutamine-dependent NAD, putative, expressed
Chr7	4263476	4263476	C	C	G	SNP	OS07G0180900	Exonic	CDS	Silent	LOC_Os07g08330	ribosomal protein L4, putative, expressed
Chr7	4484179	4484179	A	A	G	SNP	OS07G0184800	Exonic	CDS	Missense	LOC_Os07g08710	AT hook-containing DNA-binding protein, putative, expressed
Chr7	6968847	6968847	C	C	G	SNP	OS07G0225300	Exonic	CDS	Missense	LOC_Os07g12340	NAC domain-containing protein 67, putative, expressed
Chr7	8968583	8968583	T	T	G	SNP	OS07G0258400	Intronic	NA	NA	LOC_Os07g15460	metal transporter Nramp6, putative, expressed
Chr7	8970704	8970704	A	A	G	SNP	OS07G0258400	Exonic	3UTR	NA	LOC_Os07g15460	metal transporter Nramp6, putative, expressed
Chr7	17413564	17413564	A	A	G	SNP	OS07G0479300	Exonic	CDS	Silent	LOC_Os07g29620	OsSCP39 - Putative Serine Carboxypeptidase homologue, expressed
Chr7	18841136	18841136	A	A	G	SNP	OS07G0500300	Intronic	NA	NA	LOC_Os07g31720	GTPase activating protein, putative, expressed
Chr7	19473363	19473363	A	A	G	SNP	OS07G0510800	Exonic	3UTR	NA	LOC_Os07g32650	retrotransposon protein, putative, unclassified, expressed
Chr7	19473450	19473450	A	A	G	SNP	OS07G0510800	Exonic	3UTR	NA	LOC_Os07g32650	retrotransposon protein, putative, unclassified, expressed
Chr7	19473489	19473489	A	A	G	SNP	OS07G0510800	Exonic	3UTR	NA	LOC_Os07g32650	retrotransposon protein, putative, unclassified, expressed
Chr7	19473888	19473888	A	A	G	SNP	OS07G0510800	Exonic	5UTR	NA	LOC_Os07g32650	retrotransposon protein, putative, unclassified, expressed
Chr7	19656237	19656237	A	A	G	SNP	OS07G0513000	Exonic	3UTR	NA	LOC_Os07g32880	ATP synthase gamma chain, putative, expressed
Chr7	20086287	20086287	T	T	G	SNP	OS07G0520300	Exonic	CDS	Missense	LOC_Os07g33620	cytochrome P450 domain containing protein, expressed
Chr7	20725593	20725593	A	A	G	SNP	OS07G0529600	Exonic	3UTR	NA	LOC_Os07g34570	FAD dependent oxidoreductase domain containing protein, expressed
Chr7	21281202	21281202	A	A	G	SNP	OS07G0539900	Exonic	CDS	Silent	LOC_Os07g35560	glucan endo-1,3-beta-glucosidase precursor, putative, expressed
Chr7	21281396	21281396	C	C	G	SNP	OS07G0539900	Exonic	CDS	Missense	LOC_Os07g35560	glucan endo-1,3-beta-glucosidase precursor, putative, expressed
Chr7	21281680	21281680	A	A	G	SNP	OS07G0539900	Exonic	CDS	Missense	LOC_Os07g35560	glucan endo-1,3-beta-glucosidase precursor, putative, expressed
Chr7	21378805	21378805	C	C	G	SNP	OS07G0541400	Exonic	CDS	Missense	LOC_Os07g35690	TKL_IRAK_DUF26-lc.3 - DUF26 kinases have homology to DUF26 containing loci, expressed
Chr7	21378830	21378830	A	A	G	SNP	OS07G0541400	Exonic	CDS	Silent	LOC_Os07g35690	TKL_IRAK_DUF26-lc.3 - DUF26 kinases have homology to DUF26 containing loci, expressed
Chr7	21378838	21378838	A	A	G	SNP	OS07G0541400	Exonic	CDS	Missense	LOC_Os07g35690	TKL_IRAK_DUF26-lc.3 - DUF26 kinases have homology to DUF26 containing loci, expressed
Chr7	21378971	21378971	T	T	G	SNP	OS07G0541400	Exonic	CDS	Silent	LOC_Os07g35690	TKL_IRAK_DUF26-lc.3 - DUF26 kinases have homology to DUF26 containing loci, expressed
Chr7	21378992	21378992	A	A	G	SNP	OS07G0541400	Exonic	CDS	Silent	LOC_Os07g35690	TKL_IRAK_DUF26-lc.3 - DUF26 kinases have homology to DUF26 containing loci, expressed
Chr7	21379028	21379028	T	T	G	SNP	OS07G0541400	Intronic	NA	NA	LOC_Os07g35690	TKL_IRAK_DUF26-lc.3 - DUF26 kinases have homology to DUF26 containing loci, expressed
Chr7	21417552	21417552	A	A	G	SNP	OS07G0541900	Exonic	CDS	Silent	LOC_Os07g35750	TKL_IRAK_DUF26-ld.3 - DUF26 kinases have homology to DUF26 containing loci, expressed
Chr7	21418402	21418402	C	C	G	SNP	OS07G0541900	Exonic	CDS	Missense	LOC_Os07g35750	TKL_IRAK_DUF26-ld.3 - DUF26 kinases have homology to DUF26 containing loci, expressed
Chr7	21418432	21418432	C	C	G	SNP	OS07G0541900	Exonic	CDS	Missense	LOC_Os07g35750	TKL_IRAK_DUF26-ld.3 - DUF26 kinases have homology to DUF26 containing loci, expressed
Chr7	21418475	21418475	A	A	G	SNP	OS07G0541900	Exonic	CDS	Silent	LOC_Os07g35750	TKL_IRAK_DUF26-ld.3 - DUF26 kinases have homology to DUF26 containing loci, expressed
Chr7	21418619	21418619	C	C	G	SNP	OS07G0541900	Exonic	CDS	Silent	LOC_Os07g35750	TKL_IRAK_DUF26-ld.3 - DUF26 kinases have homology to DUF26 containing loci, expressed
Chr7	21418736	21418736	C	C	G	SNP	OS07G0541900	Exonic	CDS	Silent	LOC_Os07g35750	TKL_IRAK_DUF26-ld.3 - DUF26 kinases have homology to DUF26 containing loci, expressed
Chr7	21418810	21418810	C	C	G	SNP	OS07G0541900	Exonic	CDS	Missense	LOC_Os07g35750	TKL_IRAK_DUF26-ld.3 - DUF26 kinases have homology to DUF26 containing loci, expressed
Chr7	21418835	21418835	A	A	G	SNP	OS07G0541900	Exonic	CDS	Silent	LOC_Os07g35750	TKL_IRAK_DUF26-ld.3 - DUF26 kinases have homology to DUF26 containing loci, expressed
Chr7	21419267	21419267	A	A	G	SNP	OS07G0541900	Exonic	5UTR	NA	LOC_Os07g35750	TKL_IRAK_DUF26-ld.3 - DUF26 kinases have homology to DUF26 containing loci, expressed
Chr7	22372460	22372460	A	A	G	SNP	OS07G0560300	Exonic	5UTR	NA	LOC_Os07g37350	armadillo/beta-catenin repeat family protein, putative, expressed
Chr7	23677870	23677870	A	A	G	SNP	OS07G0584100	Exonic	5UTR	NA	LOC_Os07g39520	STE_PAK_Ste20_Slob_Wnk.5 - STE kinases include homologs to sterile 7, sterile 11 and sterile 20 from yeast, expressed
Chr7	24398806	24398806	A	A	G	SNP	OS07G0598200	Intronic	NA	NA	LOC_Os07g40710	circadian clock coupling factor-related, putative, expressed
Chr7	24398928	24398928	C	C	G	SNP	OS07G0598200	Intronic	NA	NA	LOC_Os07g40710	circadian clock coupling factor-related, putative, expressed
Chr7	26484209	26484209	T	T	G	SNP	OS07G0637300	Intronic	NA	NA	LOC_Os07g44330	kinase, putative, expressed
Chr7	26776607	26776607	A	A	G	SNP	OS07G0642900	Intronic	NA	NA	LOC_Os07g44840	bacterial transferase hexapeptide domain containing protein, expressed
Chr7	27172245	27172245	A	A	G	SNP	OS07G0650100	Exonic	CDS	Missense	LOC_Os07g45550	expressed protein
Chr7	27172264	27172264	A	A	G	SNP	OS07G0650100	Exonic	CDS	Silent	LOC_Os07g45550	expressed protein
Chr7	27172348	27172348	A	A	G	SNP	OS07G0650100	Exonic	CDS	Silent	LOC_Os07g45550	expressed protein
Chr7	27172420	27172420	A	A	G	SNP	OS07G0650100	Exonic	CDS	Silent	LOC_Os07g45550	expressed protein
Chr7	27172429	27172429	A	A	G	SNP	OS07G0650100	Exonic	CDS	Silent	LOC_Os07g45550	expressed protein
Chr7	27172516	27172516	A	A	G	SNP	OS07G0650100	Exonic	CDS	Missense	LOC_Os07g45550	expressed protein
Chr7	27172527	27172527	A	A	G	SNP	OS07G0650100	Exonic	CDS	Missense	LOC_Os07g45550	expressed protein
Chr7	27174195	27174195	A	A	G	SNP	OS07G0650100	Exonic	CDS	Missense	LOC_Os07g45550	expressed protein
Chr7	27550994	27550994	A	A	G	SNP	OS07G0655400	Exonic	5UTR	NA	LOC_Os07g46170	OsRhmbd13 - Putative Rhomboid homologue, expressed
Chr7	27550995	27550995	A	A	G	SNP	OS07G0655400	Exonic	5UTR	NA	LOC_Os07g46170	OsRhmbd13 - Putative Rhomboid homologue, expressed
Chr7	27723907	27723907	A	A	G	SNP	OS07G0658400	Intronic	NA	NA	LOC_Os07g46460	ferredoxin-dependent glutamate synthase, chloroplast precursor, putative, expressed
Chr7	27724210	27724210	A	A	G	SNP	OS07G0658400	Intronic	NA	NA	LOC_Os07g46460	ferredoxin-dependent glutamate synthase, chloroplast precursor, putative, expressed
Chr7	27726582	27726582	A	A	G	SNP	OS07G0658400	Intronic	NA	NA	LOC_Os07g46460	ferredoxin-dependent glutamate synthase, chloroplast precursor, putative, expressed
Chr7	27732036	27732036	C	C	G	SNP	OS07G0658400	Exonic	CDS	NA	LOC_Os07g46460	ferredoxin-dependent glutamate synthase, chloroplast precursor, putative, expressed
Chr7	27732199	27732199	A	A	G	SNP	OS07G0658400	Intronic	NA	NA	LOC_Os07g46460	ferredoxin-dependent glutamate synthase, chloroplast precursor, putative, expressed
Chr7	27732335	27732335	A	A	G	SNP	OS07G0658400	Intronic	NA	NA	LOC_Os07g46460	ferredoxin-dependent glutamate synthase, chloroplast precursor, putative, expressed
Chr7	27732956	27732956	A	A	G	SNP	OS07G0658400	Exonic	CDS	NA	LOC_Os07g46460	ferredoxin-dependent glutamate synthase, chloroplast precursor, putative, expressed
Chr7	29172111	29172111	A	A	G	SNP	OS07G0686600	Exonic	3UTR	NA	LOC_Os07g48710	VQ domain containing protein, putative, expressed
Chr7	2318929	2318932	GAGA	GAGA	G	INDEL	OS07G0145400	Exonic	5UTR	NA	LOC_Os07g05190	leucine-rich repeat family protein, putative, expressed
Chr7	4686544	4686554	GGGTCGCCTCA	GGGTCGCCTCA	G	INDEL	OS07G0187700	Intronic	NA	NA	LOC_Os07g09000	WD domain, G-beta repeat domain containing protein, expressed
Chr7	8076996	8077006	GTAAAATAAGC	GTAAAATAAGC	G	INDEL	OS07G0245100	Exonic	5UTR	NA	LOC_Os07g14150	cytidine deaminase, putative, expressed
Chr7	8970709	8970710	GA	GA	G	INDEL	OS07G0258400	Exonic	3UTR	NA	LOC_Os07g15460	metal transporter Nramp6, putative, expressed
Chr7	12574783	12574784	GA	GA	G	INDEL	OS07G0406800	Exonic	3UTR	NA	LOC_Os07g22400	POLA3 - Putative DNA polymerase alpha complex subunit, expressed
Chr7	13367395	13367407	GCATACATACATA	GCATACATACATA	G	INDEL	OS07G0418700	Exonic	3UTR	NA	LOC_Os07g23660	retrotransposon protein, putative, unclassified, expressed
Chr7	20214869	20214870	GC	GC	G	INDEL	OS07G0522500	Intronic	NA	NA	LOC_Os07g33780	pleiotropic drug resistance protein 5, putative, expressed

Chr7	21419184	21419190	GCCGCGA	GCCGCGA	G	INDEL	OS07G0541900	Exonic	CDS	InFrame	LOC_Os07g35750	TKL_IRAK_DUF26-ld.3 - DUF26 kinases have homology to DUF26 containing loci, expressed
Chr7	26774022	26774024	GAC	GAC	G	INDEL	OS07G0642800	Exonic	3UTR	NA	LOC_Os07g44830	proline-rich family protein, putative, expressed
Chr7	27725859	27725860	GA	GA	G	INDEL	OS07G0658400	Intronic	NA	NA	LOC_Os07g46460	ferredoxin-dependent glutamate synthase, chloroplast precursor, putative, expressed
Chr7	27728699	27728700	GC	GC	G	INDEL	OS07G0658400	Intronic	NA	NA	LOC_Os07g46460	ferredoxin-dependent glutamate synthase, chloroplast precursor, putative, expressed
Chr7	27730616	27730617	GA	GA	G	INDEL	OS07G0658400	Intronic	NA	NA	LOC_Os07g46460	ferredoxin-dependent glutamate synthase, chloroplast precursor, putative, expressed
Chr7	27731488	27731492	GTCTT	GTCTT	G	INDEL	OS07G0658400	Intronic	NA	NA	LOC_Os07g46460	ferredoxin-dependent glutamate synthase, chloroplast precursor, putative, expressed
Chr7	27735510	27735511	GC	GC	G	INDEL	OS07G0658400	Intronic	NA	NA	LOC_Os07g46460	ferredoxin-dependent glutamate synthase, chloroplast precursor, putative, expressed
Chr7	27737450	27737458	GAACAGCGC	GAACAGCGC	G	INDEL	OS07G0658400	Intronic	NA	NA	LOC_Os07g46460	ferredoxin-dependent glutamate synthase, chloroplast precursor, putative, expressed
Chr7	28121754	28121755	GA	GA	G	INDEL	OS07G0665900	Intronic	NA	NA	LOC_Os07g47040	expressed protein
Chr.9	2130007	2130007	T	T	G	SNP	OS09G0127800	Exonic	CDS	Missense	LOC_Os09g04110	coatomer alpha subunit, putative, expressed
Chr.9	7204323	7204323	T	T	G	SNP	OS09G0297000	Exonic	CDS	NA	LOC_Os09g12560	ferrochelatase-2, chloroplast precursor, putative, expressed
Chr.9	10523661	10523661	T	T	G	SNP	OS09G0341600	Exonic	CDS	Missense	LOC_Os09g17152	OsFBX319 - F-box domain containing protein, expressed
Chr.9	16959746	16959746	T	T	G	SNP	OS09G0452200	Intronic	NA	NA	LOC_Os09g27890	lysM domain-containing GPI-anchored protein precursor, putative, expressed
Chr.9	5454878	5454878	A	A	G	SNP	OS09G0272600	Exonic	3UTR	NA	LOC_Os09g10010	expressed protein
Chr.9	5454191	5454191	A	A	G	SNP	OS09G0272600	Exonic	CDS	Missense	LOC_Os09g10010	expressed protein
Chr.9	18016613	18016613	A	A	G	SNP	OS09G0471900	Exonic	CDS	Silent	LOC_Os09g29630	NOL1/NOP2/sun family protein, putative, expressed
Chr.9	18017377	18017377	A	A	G	SNP	OS09G0471900	Exonic	CDS	Silent	LOC_Os09g29630	NOL1/NOP2/sun family protein, putative, expressed
Chr.9	3583890	3583890	C	C	G	SNP	OS09G0247600	Intronic	NA	NA	LOC_Os09g07290	GDSL-like lipase/acylhydrolase, putative, expressed
Chr.9	7203735	7203735	C	C	G	SNP	OS09G0297000	Intronic	NA	NA	LOC_Os09g12560	ferrochelatase-2, chloroplast precursor, putative, expressed
Chr.9	10523174	10523174	C	C	G	SNP	OS09G0341600	Exonic	CDS	Missense	LOC_Os09g17152	OsFBX319 - F-box domain containing protein, expressed
Chr.9	10523679	10523679	C	C	G	SNP	OS09G0341600	Exonic	CDS	Missense	LOC_Os09g17152	OsFBX319 - F-box domain containing protein, expressed
Chr.9	10523694	10523694	C	C	G	SNP	OS09G0341600	Exonic	CDS	Missense	LOC_Os09g17152	OsFBX319 - F-box domain containing protein, expressed
Chr.9	10523695	10523695	C	C	G	SNP	OS09G0341600	Exonic	CDS	Missense	LOC_Os09g17152	OsFBX319 - F-box domain containing protein, expressed
Chr.9	3055462	3055462	A	A	G	SNP	OS09G0240200	Intronic	NA	NA	LOC_Os09g06464	CCT/B-box zinc finger protein, putative, expressed
Chr.9	6996172	6996172	A	A	G	SNP	OS09G0294000	Intronic	NA	NA	LOC_Os09g12290	bifunctional aspartokinase/homoserine dehydrogenase, chloroplast precursor, putative, expressed
Chr.9	7202609	7202609	A	A	G	SNP	OS09G0297000	Intronic	NA	NA	LOC_Os09g12560	ferrochelatase-2, chloroplast precursor, putative, expressed
Chr.9	7277343	7277343	A	A	G	SNP	OS09G0298500	Exonic	3UTR	NA	LOC_Os09g12720	zinc finger, C3HC4 type domain containing protein, expressed
Chr.9	7281820	7281820	A	A	G	SNP	OS09G0298500	Exonic	3UTR	NA	LOC_Os09g12720	zinc finger, C3HC4 type domain containing protein, expressed
Chr.9	10523031	10523031	A	A	G	SNP	OS09G0341600	Exonic	3UTR	NA	LOC_Os09g17152	OsFBX319 - F-box domain containing protein, expressed
Chr.9	10523656	10523656	A	A	G	SNP	OS09G0341600	Exonic	CDS	Silent	LOC_Os09g17152	OsFBX319 - F-box domain containing protein, expressed
Chr.9	10523774	10523774	A	A	G	SNP	OS09G0341600	Exonic	CDS	Missense	LOC_Os09g17152	OsFBX319 - F-box domain containing protein, expressed
Chr.9	19583034	19583034	A	A	G	SNP	OS09G0506000	Exonic	CDS	Silent	LOC_Os09g32840	nucleotide pyrophosphatase/phosphodiesterase, putative, expressed
Chr.9	19868840	19868840	A	A	G	SNP	OS09G0511200	Exonic	3UTR	NA	LOC_Os09g33640	expressed protein
Chr.9	1537653	1537653	C	C	G	SNP	OS09G0120033	Exonic	CDS	Missense	LOC_Os09g03190	expressed protein
Chr.9	4201282	4201282	C	C	G	SNP	OS09G0255400	Exonic	3UTR	NA	LOC_Os09g08130	indole-3-glycerol phosphate synthase, chloroplast precursor, putative, expressed
Chr.9	6389077	6389077	T	T	G	SNP	OS09G0286600	Exonic	CDS	Missense	LOC_Os09g11460	AP2 domain containing protein, expressed
Chr.9	6995280	6995280	A	A	G	SNP	OS09G0294000	Exonic	3UTR	NA	LOC_Os09g12290	bifunctional aspartokinase/homoserine dehydrogenase, chloroplast precursor, putative, expressed
Chr.9	6997668	6997668	A	A	G	SNP	OS09G0294000	Intronic	NA	NA	LOC_Os09g12290	bifunctional aspartokinase/homoserine dehydrogenase, chloroplast precursor, putative, expressed
Chr.9	6998436	6998436	A	A	G	SNP	OS09G0294000	Intronic	NA	NA	LOC_Os09g12290	bifunctional aspartokinase/homoserine dehydrogenase, chloroplast precursor, putative, expressed
Chr.9	10524019	10524019	A	A	G	SNP	OS09G0341600	Exonic	CDS	Silent	LOC_Os09g17152	OsFBX319 - F-box domain containing protein, expressed
Chr.9	10523892	10523892	A	A	G	SNP	OS09G0341600	Exonic	CDS	Missense	LOC_Os09g17152	OsFBX319 - F-box domain containing protein, expressed
Chr.9	10523904	10523904	C	C	G	SNP	OS09G0341600	Exonic	CDS	Missense	LOC_Os09g17152	OsFBX319 - F-box domain containing protein, expressed
Chr.9	10524156	10524156	T	T	G	SNP	OS09G0341600	Exonic	CDS	Missense	LOC_Os09g17152	OsFBX319 - F-box domain containing protein, expressed
Chr.9	13782058	13782058	C	C	G	SNP	OS09G0395600	Exonic	3UTR	NA	LOC_Os09g23220	glycosyl hydrolases family 16, putative, expressed
Chr.9	3062159	3062160	GT	GT	G	INDEL	OS09G0240200	Intronic	NA	NA	LOC_Os09g06464	CCT/B-box zinc finger protein, putative, expressed
Chr.9	4202753	4202771	GTCAAATTTGGTCGAAACA	GTCAAATTTGGTCGAAACA	G	INDEL	OS09G0255400	Intronic	NA	NA	LOC_Os09g08130	indole-3-glycerol phosphate synthase, chloroplast precursor, putative, expressed
Chr.9	7203886	7203893	GTGTGTGT	GTGTGTGT	G	INDEL	OS09G0297000	Intronic	NA	NA	LOC_Os09g12560	ferrochelatase-2, chloroplast precursor, putative, expressed
Chr.9	9361899	9361901	GAT	GAT	G	INDEL	OS09G0321900	Intronic	NA	NA	LOC_Os09g15320	ubiquitin-conjugating enzyme, putative, expressed
Chr.9	10252183	10252196	GCTCTCTCGCCTCT	GCTCTCTCGCCTCT	G	INDEL	OS09G0337300	Exonic	CDS	NA	LOC_Os09g16760	ankyrin repeat family protein, putative, expressed
Chr.9	21344844	21344845	GA	GA	G	INDEL	OS09G0542000	Intronic	NA	NA	LOC_Os09g37006	protein of unknown function domain containing protein, expressed
Chr.8	26445329	26445329	T	T	G	SNP	OS08G0531000	Exonic	3UTR	NA	LOC_Os08g41880	nucleotide pyrophosphatase/phosphodiesterase, putative, expressed
Chr.8	26422075	26422075	T	T	G	SNP	OS08G0530400	Exonic	3UTR	NA	LOC_Os08g41830	sulfite oxidase, putative, expressed
Chr.8	26418645	26418645	T	T	G	SNP	OS08G0530400	Intronic	NA	NA	LOC_Os08g41830	sulfite oxidase, putative, expressed
Chr.8	13955496	13955496	T	T	G	SNP	OS08G0320400	Intronic	NA	NA	LOC_Os08g23150	indole-3-glycerol phosphate synthase, chloroplast precursor, putative, expressed
Chr.8	3424548	3424548	T	T	G	SNP	OS08G0158500	Exonic	3UTR	NA	LOC_Os08g06200	stomatin-like protein 2, putative, expressed
Chr.8	3424542	3424542	T	T	G	SNP	OS08G0158500	Exonic	3UTR	NA	LOC_Os08g06200	stomatin-like protein 2, putative, expressed
Chr.8	3423333	3423333	T	T	G	SNP	OS08G0158500	Intronic	NA	NA	LOC_Os08g06200	stomatin-like protein 2, putative, expressed
Chr.8	3422629	3422629	T	T	G	SNP	OS08G0158500	Intronic	NA	NA	LOC_Os08g06200	stomatin-like protein 2, putative, expressed
Chr.8	3422016	3422016	T	T	G	SNP	OS08G0158500	Intronic	NA	NA	LOC_Os08g06200	stomatin-like protein 2, putative, expressed
Chr.8	3421983	3421983	T	T	G	SNP	OS08G0158500	Intronic	NA	NA	LOC_Os08g06200	stomatin-like protein 2, putative, expressed
Chr.8	3389366	3389366	T	T	G	SNP	OS08G0157900	Exonic	3UTR	NA	LOC_Os08g06140	no apical meristem protein, putative, expressed
Chr.8	3365645	3365645	T	T	G	SNP	OS08G0157600	Intronic	NA	NA	LOC_Os08g06110	MYB family transcription factor, putative, expressed
Chr.8	3339325	3339325	T	T	G	SNP	OS08G0157500	Intronic	NA	NA	LOC_Os08g06100	O-methyltransferase, putative, expressed
Chr.8	1561857	1561857	T	T	G	SNP	OS08G0127100	Exonic	5UTR	NA	LOC_Os08g03350	amino acid transporter, putative, expressed
Chr.8	5985698	5985698	A	A	G	SNP	OS08G0203100	Intronic	NA	NA	LOC_Os08g10290	SHR5-receptor-like kinase, putative, expressed
Chr.8	5983818	5983818	T	T	G	SNP	OS08G0203100	Intronic	NA	NA	LOC_Os08g10290	SHR5-receptor-like kinase, putative, expressed
Chr.8	26418653	26418653	C	C	G	SNP	OS08G0530400	Intronic	NA	NA	LOC_Os08g41830	sulfite oxidase, putative, expressed
Chr.8	9785202	9785202	C	C	G	SNP	OS08G0260600	Intronic	NA	NA	LOC_Os08g16050	tetraspanin family protein, putative, expressed
Chr.8	8762649	8762649	C	C	G	SNP	OS08G0243500	Intronic	NA	NA	LOC_Os08g14570	NADPH reductase, putative, expressed
Chr.8	5234317	5234317	C	C	G	SNP	OS08G0189400	Exonic	CDS	Silent	LOC_Os08g08990	Cupin domain containing protein, expressed
Chr.8	3421223	3421223	C	C	G	SNP	OS08G0158500	Intronic	NA	NA	LOC_Os08g06200	stomatin-like protein 2, putative, expressed
Chr.8	26842814	26842814	A	A	G	SNP	OS08G0536800	Intronic	NA	NA	LOC_Os08g42470	BEE 1, putative, expressed
Chr.8	26728389	26728389	A	A	G	SNP	OS08G0535200	Intronic	NA	NA	LOC_Os08g42350	nodulin MtN3 family protein, putative, expressed
Chr.8	26466494	26466494	A	A	G	SNP	OS08G0531300	Exonic	5UTR	NA	LOC_Os08g41910	Sua5/YciO/YrdC/YwlC family protein, putative, expressed
Chr.8	26418667	26418667	A	A	G	SNP	OS08G0530400	Intronic	NA	NA	LOC_Os08g41830	sulfite oxidase, putative, expressed
Chr.8	26391683	26391683	A	A	G	SNP	OS08G0530000	Intronic	NA	NA	LOC_Os08g41790	phosphoribulokinase/Uridine kinase family protein, expressed
Chr.8	26390846	26390846	A	A	G	SNP	OS08G0530000	Intronic	NA	NA	LOC_Os08g41790	phosphoribulokinase/Uridine kinase family protein, expressed
Chr.8	26390301	26390301	A	A	G	SNP	OS08G0530000	Intronic	NA	NA	LOC_Os08g41790	phosphoribulokinase/Uridine kinase family protein, expressed
Chr.8	20464292	20464292	A	A	G	SNP	OS08G0425800	Exonic	3UTR	NA	LOC_Os08g32980	expressed protein
Chr.8	16064688	16064688	A	A	G	SNP	OS08G0352100	Exonic	3UTR	NA	LOC_Os08g26350	expressed protein
Chr.8	15659132	15659132	A	A	G	SNP	OS08G0345700	Intronic	NA	NA	LOC_Os08g25720	pyrophosphate--fructose 6-phosphate 1-phosphotransferase subunit alpha, putative, expressed
Chr.8	8764456	8764456	A	A	G	SNP	OS08G0243500	Intronic	NA	NA	LOC_Os08g14570	NADPH reductase, putative, expressed
Chr.8	8328589	8328589	A	A	G	SNP	OS08G0237000	Intronic	NA	NA	LOC_Os08g13920	glycosyl hydrolases family 16, putative, expressed
Chr.8	7364941	7364941	A	A	G	SNP	OS08G0221300	Intronic	NA	NA	LOC_Os08g12449	expressed protein
Chr.8	5265011	5265011	A	A	G	SNP	OS08G0190100	Exonic	CDS	NA	LOC_Os08g09080	Cupin domain containing protein, expressed

Chr.8	5142961	5142961	A	A	G	SNP	OS08G0187800	Intronic	NA	NA	LOC_Os08g08840	phosphate/phosphate translocator, putative, expressed
Chr.8	3544716	3544716	A	A	G	SNP	OS08G0160500	Exonic	3UTR	NA	LOC_Os08g06380	CSLF6 - cellulose synthase-like family F; beta1,3;1,4 glucan synthase, expressed
Chr.8	3461392	3461392	A	A	G	SNP	OS08G0159500	Exonic	3UTR	NA	LOC_Os08g06280	LSD1 zinc finger domain containing protein, expressed
Chr.8	3424213	3424213	A	A	G	SNP	OS08G0158500	Exonic	CDS	Silent	LOC_Os08g06200	stomatin-like protein 2, putative, expressed
Chr.8	3424182	3424182	A	A	G	SNP	OS08G0158500	Exonic	CDS	Missense	LOC_Os08g06200	stomatin-like protein 2, putative, expressed
Chr.8	3424069	3424069	A	A	G	SNP	OS08G0158500	Intronic	NA	NA	LOC_Os08g06200	stomatin-like protein 2, putative, expressed
Chr.8	3423644	3423644	A	A	G	SNP	OS08G0158500	Intronic	NA	NA	LOC_Os08g06200	stomatin-like protein 2, putative, expressed
Chr.8	3422801	3422801	A	A	G	SNP	OS08G0158500	Exonic	CDS	Silent	LOC_Os08g06200	stomatin-like protein 2, putative, expressed
Chr.8	3422668	3422668	A	A	G	SNP	OS08G0158500	Intronic	NA	NA	LOC_Os08g06200	stomatin-like protein 2, putative, expressed
Chr.8	3422383	3422383	A	A	G	SNP	OS08G0158500	Intronic	NA	NA	LOC_Os08g06200	stomatin-like protein 2, putative, expressed
Chr.8	3421543	3421543	A	A	G	SNP	OS08G0158500	Intronic	NA	NA	LOC_Os08g06200	stomatin-like protein 2, putative, expressed
Chr.8	3391786	3391786	A	A	G	SNP	OS08G0157900	Exonic	CDS	Silent	LOC_Os08g06140	no apical meristem protein, putative, expressed
Chr.8	3389596	3389596	A	A	G	SNP	OS08G0157900	Exonic	CDS	Missense	LOC_Os08g06140	no apical meristem protein, putative, expressed
Chr.8	3370545	3370545	A	A	G	SNP	OS08G0157600	Intronic	NA	NA	LOC_Os08g06110	MYB family transcription factor, putative, expressed
Chr.8	3364182	3364182	A	A	G	SNP	OS08G0157600	Intronic	NA	NA	LOC_Os08g06110	MYB family transcription factor, putative, expressed
Chr.8	3363892	3363892	A	A	G	SNP	OS08G0157600	Intronic	NA	NA	LOC_Os08g06110	MYB family transcription factor, putative, expressed
Chr.8	3339295	3339295	A	A	G	SNP	OS08G0157500	Intronic	NA	NA	LOC_Os08g06100	O-methyltransferase, putative, expressed
Chr.8	3295282	3295282	A	A	G	SNP	OS08G0156800	Intronic	NA	NA	LOC_Os08g06040	60S ribosomal protein L34, putative, expressed
Chr.8	3294890	3294890	A	A	G	SNP	OS08G0156800	Intronic	NA	NA	LOC_Os08g06040	60S ribosomal protein L34, putative, expressed
Chr.8	3283956	3283956	A	A	G	SNP	OS08G0156600	Exonic	CDS	Silent	LOC_Os08g06010	transporter, major facilitator family, putative, expressed
Chr.8	3282405	3282405	A	A	G	SNP	OS08G0156600	Exonic	CDS	Missense	LOC_Os08g06010	transporter, major facilitator family, putative, expressed
Chr.8	2173552	2173552	A	A	G	SNP	OS08G0139000	Exonic	3UTR	NA	LOC_Os08g04440	RNA recognition motif containing protein, putative, expressed
Chr.8	1835090	1835090	A	A	G	SNP	OS08G0132600	Intronic	NA	NA	LOC_Os08g03840	expressed protein
Chr.8	1619550	1619550	A	A	G	SNP	OS08G0128500	Intronic	NA	NA	LOC_Os08g03450	ribosomal protein L37, putative, expressed
Chr.8	1566252	1566252	A	A	G	SNP	OS08G0127100	Exonic	CDS	Silent	LOC_Os08g03350	amino acid transporter, putative, expressed
Chr.8	688105	688105	A	A	G	SNP	OS08G0112900	Intronic	NA	NA	LOC_Os08g02094	GDSL-like lipase/acylhydrolase, putative, expressed
Chr.8	581785	581785	A	A	G	SNP	OS08G0111200	Intronic	NA	NA	LOC_Os08g01940	non-lysosomal glucosylceramidase, putative, expressed
Chr.8	690881	690881	A	A	G	SNP	OS08G0112900	Intronic	NA	NA	LOC_Os08g02094	GDSL-like lipase/acylhydrolase, putative, expressed
Chr.8	691197	691197	A	A	G	SNP	OS08G0112900	Intronic	NA	NA	LOC_Os08g02094	GDSL-like lipase/acylhydrolase, putative, expressed
Chr.8	2130532	2130532	A	A	G	SNP	OS08G0137800	Exonic	3UTR	NA	LOC_Os08g04340	plastocyanin-like domain containing protein, putative, expressed
Chr.8	2514430	2514430	C	C	G	SNP	OS08G0144400	Exonic	CDS	StartLoss	LOC_Os08g04920	OsDegp11 - Putative Deg protease homologue, expressed
Chr.8	3243183	3243183	C	C	G	SNP	OS08G0155900	Exonic	CDS	Silent	LOC_Os08g05960	expressed protein
Chr.8	3243068	3243068	A	A	G	SNP	OS08G0155900	Exonic	CDS	Missense	LOC_Os08g05960	expressed protein
Chr.8	3424615	3424615	C	C	G	SNP	OS08G0158500	Exonic	3UTR	NA	LOC_Os08g06200	stomatin-like protein 2, putative, expressed
Chr.8	3421164	3421164	C	C	G	SNP	OS08G0158500	Intronic	NA	NA	LOC_Os08g06200	stomatin-like protein 2, putative, expressed
Chr.8	3421167	3421167	C	C	G	SNP	OS08G0158500	Intronic	NA	NA	LOC_Os08g06200	stomatin-like protein 2, putative, expressed
Chr.8	3421169	3421169	A	A	G	SNP	OS08G0158500	Intronic	NA	NA	LOC_Os08g06200	stomatin-like protein 2, putative, expressed
Chr.8	3421460	3421460	A	A	G	SNP	OS08G0158500	Intronic	NA	NA	LOC_Os08g06200	stomatin-like protein 2, putative, expressed
Chr.8	3421486	3421486	T	T	G	SNP	OS08G0158500	Intronic	NA	NA	LOC_Os08g06200	stomatin-like protein 2, putative, expressed
Chr.8	3421591	3421591	T	T	G	SNP	OS08G0158500	Intronic	NA	NA	LOC_Os08g06200	stomatin-like protein 2, putative, expressed
Chr.8	3423583	3423583	A	A	G	SNP	OS08G0158500	Intronic	NA	NA	LOC_Os08g06200	stomatin-like protein 2, putative, expressed
Chr.8	3423590	3423590	A	A	G	SNP	OS08G0158500	Intronic	NA	NA	LOC_Os08g06200	stomatin-like protein 2, putative, expressed
Chr.8	11608249	11608249	C	C	G	SNP	OS08G0290700	Exonic	CDS	Silent	LOC_Os08g19420	O-methyltransferase, putative, expressed
Chr.8	13954662	13954662	C	C	G	SNP	OS08G0320400	Exonic	CDS	Missense	LOC_Os08g23150	indole-3-glycerol phosphate synthase, chloroplast precursor, putative, expressed
Chr.8	14414342	14414342	T	T	G	SNP	OS08G0327400	Exonic	5UTR	NA	LOC_Os08g23810	enoyl-acyl-carrier-protein reductase NADH, chloroplast precursor, expressed
Chr.8	15658546	15658546	A	A	G	SNP	OS08G0345700	Exonic	5UTR	NA	LOC_Os08g25720	pyrophosphate--fructose 6-phosphate 1-phosphotransferase subunit alpha, putative, expressed
Chr.8	17511602	17511602	T	T	G	SNP	OS08G0374000	Exonic	5UTR	NA	LOC_Os08g28670	pathogenesis-related Bet v I family protein, putative, expressed
Chr.8	20249297	20249297	A	A	G	SNP	OS08G0422600	Exonic	CDS	NA	LOC_Os08g32690	expressed protein
Chr.8	2173580	2173583	GAGC	GAGC	G	INDEL	OS08G0139000	Exonic	3UTR	NA	LOC_Os08g04440	RNA recognition motif containing protein, putative, expressed
Chr.8	3294131	3294132	GT	GT	G	INDEL	OS08G0156800	Intronic	NA	NA	LOC_Os08g06040	60S ribosomal protein L34, putative, expressed
Chr.8	3366191	3366201	GCATTTTTTTT	GCATTTTTTTT	G	INDEL	OS08G0157600	Intronic	NA	NA	LOC_Os08g06110	MYB family transcription factor, putative, expressed
Chr.8	3423491	3423492	GT	GT	G	INDEL	OS08G0158500	Intronic	NA	NA	LOC_Os08g06200	stomatin-like protein 2, putative, expressed
Chr.8	5139950	5139951	GT	GT	G	INDEL	OS08G0187800	Intronic	NA	NA	LOC_Os08g08840	phosphate/phosphate translocator, putative, expressed
Chr.8	8329076	8329078	GGC	GGC	G	INDEL	OS08G0237000	Exonic	3UTR	NA	LOC_Os08g13920	glycosyl hydrolases family 16, putative, expressed
Chr.8	8329106	8329108	GTT	GTT	G	INDEL	OS08G0237000	Exonic	3UTR	NA	LOC_Os08g13920	glycosyl hydrolases family 16, putative, expressed
Chr.8	18323390	18323391	GA	GA	G	INDEL	OS08G0387700	Intronic	NA	NA	LOC_Os08g29809	resistance protein LR10, putative, expressed
Chr.6	1361823	1361823	T	T	G	SNP	OS06G0125200	Intronic	NA	NA	LOC_Os06g03520	DUF581 domain containing protein, expressed
Chr.6	7230629	7230629	T	T	G	SNP	OS06G0239200	Exonic	3UTR	NA	LOC_Os06g13190	expressed protein
Chr.6	13478889	13478889	T	T	G	SNP	OS06G0338200	Intronic	NA	NA	LOC_Os06g23114	copper methylamine oxidase precursor, putative, expressed
Chr.6	19619462	19619462	T	T	G	SNP	OS06G0528600	Exonic	3UTR	NA	LOC_Os06g33710	spermidine synthase, putative, expressed
Chr.6	21600673	21600673	T	T	G	SNP	OS06G0562600	Exonic	CDS	Missense	LOC_Os06g36700	T-complex protein, putative, expressed
Chr.6	21896487	21896487	T	T	G	SNP	OS06G0567200	Intronic	NA	NA	LOC_Os06g37080	L-ascorbate oxidase precursor, putative, expressed
Chr.6	23469036	23469036	T	T	G	SNP	OS06G0596300	Intronic	NA	NA	LOC_Os06g39520	myristoyl-acyl carrier protein thioesterase, chloroplast precursor, putative, expressed
Chr.6	24143049	24143049	T	T	G	SNP	OS06G0607200	Exonic	3UTR	NA	LOC_Os06g40510	CRAL/TRIO domain containing protein, expressed
Chr.6	24144992	24144992	T	T	G	SNP	OS06G0607200	Intronic	NA	NA	LOC_Os06g40510	CRAL/TRIO domain containing protein, expressed
Chr.6	3361920	3361920	T	T	G	SNP	OS06G0166900	Intronic	NA	NA	LOC_Os06g07070	BRASSINOSTEROID INSENSITIVE 1-associated receptor kinase 1 precursor, putative, expressed
Chr.6	3445054	3445054	T	T	G	SNP	OS06G0168500	Exonic	3UTR	NA	LOC_Os06g07200	syntaxin, putative, expressed
Chr.6	3453807	3453807	T	T	G	SNP	OS06G0168600	Intronic	NA	NA	LOC_Os06g07210	ribonucleoside-diphosphate reductase large subunit, putative, expressed
Chr.6	3454691	3454691	A	A	G	SNP	OS06G0168600	Exonic	5UTR	NA	LOC_Os06g07210	ribonucleoside-diphosphate reductase large subunit, putative, expressed
Chr.6	4654145	4654145	C	C	G	SNP	OS06G0192400	Intronic	NA	NA	LOC_Os06g09270	hypro1, putative, expressed
Chr.6	27135979	27135979	A	A	G	SNP	OS06G0659300	Exonic	3UTR	NA	LOC_Os06g44890	villin protein, putative, expressed
Chr.6	27137146	27137146	A	A	G	SNP	OS06G0659300	Intronic	NA	NA	LOC_Os06g44890	villin protein, putative, expressed
Chr.6	27138088	27138088	A	A	G	SNP	OS06G0659300	Intronic	NA	NA	LOC_Os06g44890	villin protein, putative, expressed
Chr.6	27279992	27279992	T	T	G	SNP	OS06G0661900	Exonic	3UTR	NA	LOC_Os06g45110	DNA binding protein, putative, expressed
Chr.6	27385825	27385825	A	A	G	SNP	OS06G0663400	Exonic	CDS	Silent	LOC_Os06g45300	serine/threonine-protein kinase HT1, putative, expressed
Chr.6	28196188	28196188	A	A	G	SNP	OS06G0678200	Exonic	5UTR	NA	LOC_Os06g46450	polyprenyl synthetase, putative, expressed
Chr.6	29145864	29145864	A	A	G	SNP	OS06G0696600	Intronic	NA	NA	LOC_Os06g48180	glycosyl hydrolases family 16, putative, expressed
Chr.6	29145872	29145872	A	A	G	SNP	OS06G0696600	Intronic	NA	NA	LOC_Os06g48180	glycosyl hydrolases family 16, putative, expressed
Chr.6	31070156	31070156	C	C	G	SNP	OS06G0729300	Intronic	NA	NA	LOC_Os06g51310	PAZ domain containing protein, expressed
Chr.6	31128652	31128652	A	A	G	SNP	OS06G0730300	Exonic	CDS	Missense	LOC_Os06g51390	expressed protein
Chr.6	3445764	3445765	GT	GT	G	INDEL	OS06G0168500	Intronic	NA	NA	LOC_Os06g07200	syntaxin, putative, expressed
Chr.6	679928	679928	C	C	G	SNP	OS06G0112100	Intronic	NA	NA	LOC_Os06g02210	bark storage protein A precursor, putative, expressed
Chr.6	1555921	1555921	C	C	G	SNP	OS06G0129400	Intronic	NA	NA	LOC_Os06g03860	uncharacterized membrane protein, putative, expressed
Chr.6	17067712	17067712	C	C	G	SNP	OS06G0493100	Intronic	NA	NA	LOC_Os06g29730	RALFL28 - Rapid ALkalinization Factor RALF family protein precursor, expressed
Chr.6	17068565	17068565	C	C	G	SNP	OS06G0493100	Exonic	CDS	Silent	LOC_Os06g29730	RALFL28 - Rapid ALkalinization Factor RALF family protein precursor, expressed

Chr.6	27137083	27137083	C	C	G	SNP	OS06G0659300	Intronic	NA	NA	LOC_Os06g44890	villin protein, putative, expressed
Chr.6	300686	300686	A	A	G	SNP	OS06G0104300	Exonic	CDS	Silent	LOC_Os06g01490	monocopper oxidase, putative, expressed
Chr.6	413702	413702	A	A	G	SNP	OS06G0106100	Exonic	CDS	Silent	LOC_Os06g01700	CWC15 homolog A, putative, expressed
Chr.6	1362189	1362189	A	A	G	SNP	OS06G0125200	Exonic	3UTR	NA	LOC_Os06g03520	DUF581 domain containing protein, expressed
Chr.6	1362316	1362316	A	A	G	SNP	OS06G0125200	Exonic	3UTR	NA	LOC_Os06g03520	DUF581 domain containing protein, expressed
Chr.6	1362347	1362347	A	A	G	SNP	OS06G0125200	Exonic	3UTR	NA	LOC_Os06g03520	DUF581 domain containing protein, expressed
Chr.6	1362364	1362364	A	A	G	SNP	OS06G0125200	Exonic	3UTR	NA	LOC_Os06g03520	DUF581 domain containing protein, expressed
Chr.6	1555676	1555676	A	A	G	SNP	OS06G0129400	Intronic	NA	NA	LOC_Os06g03860	uncharacterized membrane protein, putative, expressed
Chr.6	1556142	1556142	A	A	G	SNP	OS06G0129400	Intronic	NA	NA	LOC_Os06g03860	uncharacterized membrane protein, putative, expressed
Chr.6	2323415	2323415	A	A	G	SNP	OS06G0143900	Intronic	NA	NA	LOC_Os06g05180	coatomer subunit beta, putative, expressed
Chr.6	7230491	7230491	A	A	G	SNP	OS06G0239200	Exonic	CDS	Missense	LOC_Os06g13190	expressed protein
Chr.6	9096372	9096372	A	A	G	SNP	OS06G0270900	Exonic	3UTR	NA	LOC_Os06g15990	aldehyde dehydrogenase, putative, expressed
Chr.6	10687475	10687475	A	A	G	SNP	OS06G0291500	Exonic	3UTR	NA	LOC_Os06g18820	serine threonine kinase, putative, expressed
Chr.6	13478470	13478470	A	A	G	SNP	OS06G0338200	Intronic	NA	NA	LOC_Os06g23114	copper methylamine oxidase precursor, putative, expressed
Chr.6	13482282	13482282	A	A	G	SNP	OS06G0338200	Intronic	NA	NA	LOC_Os06g23114	copper methylamine oxidase precursor, putative, expressed
Chr.6	19619475	19619475	A	A	G	SNP	OS06G0528600	Exonic	3UTR	NA	LOC_Os06g33710	spermidine synthase, putative, expressed
Chr.6	21599654	21599654	A	A	G	SNP	OS06G0562600	Intronic	NA	NA	LOC_Os06g36700	T-complex protein, putative, expressed
Chr.6	21602259	21602259	A	A	G	SNP	OS06G0562600	Intronic	NA	NA	LOC_Os06g36700	T-complex protein, putative, expressed
Chr.6	22352773	22352773	A	A	G	SNP	OS06G0575400	Exonic	CDS	Missense	LOC_Os06g37750	S-locus-like receptor protein kinase, putative, expressed
Chr.6	22352815	22352815	A	A	G	SNP	OS06G0575400	Exonic	CDS	Missense	LOC_Os06g37750	S-locus-like receptor protein kinase, putative, expressed
Chr.6	22353290	22353290	A	A	G	SNP	OS06G0575400	Exonic	CDS	Missense	LOC_Os06g37750	S-locus-like receptor protein kinase, putative, expressed
Chr.6	23111319	23111319	A	A	G	SNP	OS06G0589300	Intronic	NA	NA	LOC_Os06g38950	ABC transporter, ATP-binding protein, putative, expressed
Chr.6	23112357	23112357	A	A	G	SNP	OS06G0589300	Intronic	NA	NA	LOC_Os06g38950	ABC transporter, ATP-binding protein, putative, expressed
Chr.6	23306872	23306872	A	A	G	SNP	OS06G0593100	Exonic	3UTR	NA	LOC_Os06g39260	solute carrier family 35 member B1, putative, expressed
Chr.6	23467853	23467853	A	A	G	SNP	OS06G0596300	Intronic	NA	NA	LOC_Os06g39520	myristoyl-acyl carrier protein thioesterase, chloroplast precursor, putative, expressed
Chr.6	23856625	23856625	A	A	G	SNP	OS06G0603000	Intronic	NA	NA	LOC_Os06g40080	heme oxygenase 1, putative, expressed
Chr.6	24401229	24401229	A	A	G	SNP	OS06G0611900	Intronic	NA	NA	LOC_Os06g40940	glycine dehydrogenase, putative, expressed
Chr.6	24402062	24402062	A	A	G	SNP	OS06G0611900	Intronic	NA	NA	LOC_Os06g40940	glycine dehydrogenase, putative, expressed
Chr.6	24402160	24402160	A	A	G	SNP	OS06G0611900	Intronic	NA	NA	LOC_Os06g40940	glycine dehydrogenase, putative, expressed
Chr.6	27140924	27140924	A	A	G	SNP	OS06G0659300	Intronic	NA	NA	LOC_Os06g44890	villin protein, putative, expressed
Chr.6	28754139	28754139	A	A	G	SNP	OS06G0689600	Intronic	NA	NA	LOC_Os06g47470	TKL_IRAK_DUF26-lh.2 - DUF26 kinases have homology to DUF26 containing loci, expressed
Chr.6	29145586	29145586	A	A	G	SNP	OS06G0696600	Intronic	NA	NA	LOC_Os06g48180	glycosyl hydrolases family 16, putative, expressed
Chr.6	29385275	29385275	A	A	G	SNP	OS06G0699200	Intronic	NA	NA	LOC_Os06g48570	Ser/Thr protein phosphatase family protein, putative, expressed
Chr.6	1361977	1361977	C	C	G	SNP	OS06G0125200	Exonic	CDS	Silent	LOC_Os06g03520	DUF581 domain containing protein, expressed
Chr.6	1361998	1361998	T	T	G	SNP	OS06G0125200	Exonic	CDS	Silent	LOC_Os06g03520	DUF581 domain containing protein, expressed
Chr.6	4331525	4331525	C	C	G	SNP	OS06G0186100	Exonic	CDS	Missense	LOC_Os06g08690	leucine-rich repeat receptor protein kinase EXS precursor, putative, expressed
Chr.6	4332730	4332730	C	C	G	SNP	OS06G0186100	Exonic	CDS	Missense	LOC_Os06g08690	leucine-rich repeat receptor protein kinase EXS precursor, putative, expressed
Chr.6	4654147	4654147	A	A	G	SNP	OS06G0192400	Intronic	NA	NA	LOC_Os06g09270	hypro1, putative, expressed
Chr.6	4868982	4868982	A	A	G	SNP	OS06G0195800	Exonic	CDS	NA	LOC_Os06g09560	heat shock protein DnaJ, putative, expressed
Chr.6	4868719	4868719	C	C	G	SNP	OS06G0195800	Intronic	NA	NA	LOC_Os06g09560	heat shock protein DnaJ, putative, expressed
Chr.6	8848345	8848345	A	A	G	SNP	OS06G0266800	Exonic	3UTR	NA	LOC_Os06g15620	GASR7 - Gibberellin-regulated GASA/GAST/Snakin family protein precursor, expressed
Chr.6	13478013	13478013	C	C	G	SNP	OS06G0338200	Intronic	NA	NA	LOC_Os06g23114	copper methylamine oxidase precursor, putative, expressed
Chr.6	13482378	13482378	C	C	G	SNP	OS06G0338200	Intronic	NA	NA	LOC_Os06g23114	copper methylamine oxidase precursor, putative, expressed
Chr.6	13482473	13482473	T	T	G	SNP	OS06G0338200	Intronic	NA	NA	LOC_Os06g23114	copper methylamine oxidase precursor, putative, expressed
Chr.6	17067502	17067502	A	A	G	SNP	OS06G0493100	Intronic	NA	NA	LOC_Os06g29730	RALFL28 - Rapid ALkalinization Factor RALF family protein precursor, expressed
Chr.6	17068033	17068033	A	A	G	SNP	OS06G0493100	Intronic	NA	NA	LOC_Os06g29730	RALFL28 - Rapid ALkalinization Factor RALF family protein precursor, expressed
Chr.6	21601221	21601221	A	A	G	SNP	OS06G0562600	Intronic	NA	NA	LOC_Os06g36700	T-complex protein, putative, expressed
Chr.6	23468174	23468174	C	C	G	SNP	OS06G0596300	Exonic	CDS	Silent	LOC_Os06g39520	myristoyl-acyl carrier protein thioesterase, chloroplast precursor, putative, expressed
Chr.6	23468214	23468214	A	A	G	SNP	OS06G0596300	Exonic	CDS	Missense	LOC_Os06g39520	myristoyl-acyl carrier protein thioesterase, chloroplast precursor, putative, expressed
Chr.6	23469697	23469697	A	A	G	SNP	OS06G0596300	Exonic	CDS	Missense	LOC_Os06g39520	myristoyl-acyl carrier protein thioesterase, chloroplast precursor, putative, expressed
Chr.6	23469855	23469855	A	A	G	SNP	OS06G0596300	Intronic	NA	NA	LOC_Os06g39520	myristoyl-acyl carrier protein thioesterase, chloroplast precursor, putative, expressed
Chr.6	24400547	24400547	T	T	G	SNP	OS06G0611900	Exonic	CDS	Silent	LOC_Os06g40940	glycine dehydrogenase, putative, expressed
Chr.6	26310877	26310877	C	C	G	SNP	OS06G0644600	Intronic	NA	NA	LOC_Os06g43690	WD repeat-containing protein, putative, expressed
Chr.6	28389186	28389186	A	A	G	SNP	OS06G0681200	Exonic	CDS	Missense	LOC_Os06g46740	early nodulin 20 precursor, putative, expressed
Chr.6	28617951	28617951	A	A	G	SNP	OS06G0686400	Exonic	3UTR	NA	LOC_Os06g47200	LTPL85 - Protease inhibitor/seed storage/LTP family protein precursor, expressed
Chr.6	30306312	30306312	A	A	G	SNP	OS06G0714200	Intronic	NA	NA	LOC_Os06g50030	CAMK_CAMK_like.30 - CAMK includes calcium/calmodulin depedent protein kinases, expressed
Chr.6	297499	297501	GAC	GAC	G	INDEL	OS06G0104300	Exonic	3UTR	NA	LOC_Os06g01490	monocopper oxidase, putative, expressed
Chr.6	414261	414270	GAGGTTTGGA	GAGGTTTGGA	G	INDEL	OS06G0106100	Intronic	NA	NA	LOC_Os06g01700	CWC15 homolog A, putative, expressed
Chr.6	1415899	1415911	GAGATGATGATGA	GAGATGATGATGA	G	INDEL	OS06G0126500	Exonic	3UTR	NA	LOC_Os06g03640	BAG domain containing protein, expressed
Chr.6	1555924	1555925	GT	GT	G	INDEL	OS06G0129400	Intronic	NA	NA	LOC_Os06g03860	uncharacterized membrane protein, putative, expressed
Chr.6	2262795	2262796	GA	GA	G	INDEL	OS06G0143100	Intronic	NA	NA	LOC_Os06g05120	expressed protein
Chr.6	4331783	4331784	GT	GT	G	INDEL	OS06G0186100	Exonic	CDS	FrameShift	LOC_Os06g08690	leucine-rich repeat receptor protein kinase EXS precursor, putative, expressed
Chr.6	5678500	5678501	GT	GT	G	INDEL	OS06G0211200	Intronic	NA	NA	LOC_Os06g10880	bZIP transcription factor, putative, expressed
Chr.6	8706304	8706310	GCCGCCA	GCCGCCA	G	INDEL	OS06G0264200	Exonic	CDS	InFrame	LOC_Os06g15330	CCT/B-box zinc finger protein, putative, expressed
Chr.6	8848688	8848690	GAT	GAT	G	INDEL	OS06G0266800	Exonic	5UTR	NA	LOC_Os06g15620	GASR7 - Gibberellin-regulated GASA/GAST/Snakin family protein precursor, expressed
Chr.6	20677271	20677273	GCA	GCA	G	INDEL	OS06G0546500	Intronic	NA	NA	LOC_Os06g35480	peroxidase precursor, putative, expressed
Chr.6	23468190	23468193	GCCC	GCCC	G	INDEL	OS06G0596300	Exonic	CDS	InFrame	LOC_Os06g39520	myristoyl-acyl carrier protein thioesterase, chloroplast precursor, putative, expressed
Chr.6	23467581	23467584	GTTC	GTTC	G	INDEL	OS06G0596300	Intronic	NA	NA	LOC_Os06g39520	myristoyl-acyl carrier protein thioesterase, chloroplast precursor, putative, expressed
Chr.6	23855450	23855467	GGATAATATGTTTGTTTT	GGATAATATGTTTGTTTT	G	INDEL	OS06G0603000	Intronic	NA	NA	LOC_Os06g40080	heme oxygenase 1, putative, expressed
Chr.6	28753513	28753536	GCAAGCTACCCCTGTCCGCAAACT	GCAAGCTACCCCTGTCCGCAAACT	G	INDEL	OS06G0689600	Exonic	CDS	FrameShift	LOC_Os06g47470	TKL_IRAK_DUF26-lh.2 - DUF26 kinases have homology to DUF26 containing loci, expressed
Chr.5	28193576	28193576	T	T	G	SNP	OS05G0566400	Intronic	NA	NA	LOC_Os05g49140	CGMC_MAPKCMGC_2.8 - CGMC includes CDA, MAPK, GSK3, and CLKC kinases, expressed
Chr.5	28190614	28190614	T	T	G	SNP	OS05G0566400	Intronic	NA	NA	LOC_Os05g49140	CGMC_MAPKCMGC_2.8 - CGMC includes CDA, MAPK, GSK3, and CLKC kinases, expressed
Chr.5	28190291	28190291	A	A	G	SNP	OS05G0566400	Intronic	NA	NA	LOC_Os05g49140	CGMC_MAPKCMGC_2.8 - CGMC includes CDA, MAPK, GSK3, and CLKC kinases, expressed
Chr.5	27960601	27960601	C	C	G	SNP	OS05G0562000	Exonic	CDS	Missense	LOC_Os05g48790	expressed protein
Chr.5	22072186	22072186	A	A	G	SNP	OS05G0449600	Exonic	3UTR	NA	LOC_Os05g37700	periplasmic beta-glucosidase precursor, putative, expressed
Chr.5	19736486	19736486	A	A	G	SNP	OS05G0405000	Intronic	NA	NA	LOC_Os05g33570	pyruvate, phosphate dikinase, chloroplast precursor, putative, expressed
Chr.5	14162307	14162307	T	T	G	SNP	OS05G0310500	Intronic	NA	NA	LOC_Os05g24550	Papain family cysteine protease domain containing protein, expressed
Chr.5	5653650	5653650	T	T	G	SNP	OS05G0192100	Intronic	NA	NA	LOC_Os05g10370	acid phosphatase, putative, expressed
Chr.5	5653393	5653393	C	C	G	SNP	OS05G0192100	Intronic	NA	NA	LOC_Os05g10370	acid phosphatase, putative, expressed
Chr.5	23753341	23753342	GT	GT	G	INDEL	OS05G0482700	Exonic	5UTR	NA	LOC_Os05g40420	2,3-bisphosphoglycerate-independent phosphoglycerate mutase, putative, expressed
Chr.5	25230698	25230698	C	C	G	SNP	OS05G0509500	Exonic	CDS	Missense	LOC_Os05g43390	signal recognition particle 54 kDa protein, putative, expressed
Chr.5	20631386	20631386	A	A	G	SNP	OS05G0420600	Intronic	NA	NA	LOC_Os05g34770	cytochrome c, putative, expressed
Chr.5	20630789	20630789	T	T	G	SNP	OS05G0420600	Intronic	NA	NA	LOC_Os05g34770	cytochrome c, putative, expressed
Chr.5	20316578	20316578	A	A	G	SNP	OS05G0415700	Exonic	CDS	Missense	LOC_Os05g34320	beta-hexosaminidase precursor, putative, expressed
Chr.5	875548	875548	A	A	G	SNP	OS05G0116000	Intronic	NA	NA	LOC_Os05g02520	cupin domain containing protein, expressed

Chr.5	27960235	27960235	A	A	G	SNP	OS05G0562000	Exonic	CDS	Missense	LOC_Os05g48790	expressed protein
Chr.5	13613131	13613131	A	A	G	SNP	OS05G0302700	Intronic	NA	NA	LOC_Os05g23720	mitochondrial carrier protein, putative, expressed
Chr.5	2721215	2721215	C	C	G	SNP	OS05G0147400	Intronic	NA	NA	LOC_Os05g05470	T-complex protein, putative, expressed
Chr.5	2718818	2718818	A	A	G	SNP	OS05G0147400	Exonic	3UTR	NA	LOC_Os05g05470	T-complex protein, putative, expressed
Chr.5	270716	270716	C	C	G	SNP	OS05G0105000	Intronic	NA	NA	LOC_Os05g01470	methionine S-methyltransferase, putative, expressed
Chr.5	2114970	2114970	A	A	G	SNP	OS05G0136200	Exonic	CDS	Silent	LOC_Os05g04550	CAMK_KIN1/SNF1/Nim1_like.3 - CAMK includes calcium/calmodulin depedent protein kinases, expressed
Chr.5	27973430	27973430	A	A	G	SNP	OS05G0562300	Intronic	NA	NA	LOC_Os05g48810	dnaJ domain containing protein, expressed
Chr.5	25644328	25644328	A	A	G	SNP	OS05G0517200	Intronic	NA	NA	LOC_Os05g44100	trehalose synthase, putative, expressed
Chr.5	25545965	25545965	A	A	G	SNP	OS05G0515200	Exonic	3UTR	NA	LOC_Os05g43910	cytochrome P450, putative, expressed
Chr.5	25545420	25545420	A	A	G	SNP	OS05G0515200	Intronic	NA	NA	LOC_Os05g43910	cytochrome P450, putative, expressed
Chr.5	25545078	25545078	A	A	G	SNP	OS05G0515200	Intronic	NA	NA	LOC_Os05g43910	cytochrome P450, putative, expressed
Chr.5	24584697	24584697	C	C	G	SNP	OS05G0499300	Exonic	3UTR	NA	LOC_Os05g41990	peroxidase precursor, putative, expressed
Chr.5	24327189	24327189	C	C	G	SNP	OS05G0495300	Exonic	CDS	Silent	LOC_Os05g41550	expressed protein
Chr.5	23420985	23420985	A	A	G	SNP	OS05G0476200	Intronic	NA	NA	LOC_Os05g39850	MCM3 - Putative minichromosome maintenance MCM complex subunit 3, expressed
Chr.5	23420454	23420454	A	A	G	SNP	OS05G0476200	Intronic	NA	NA	LOC_Os05g39850	MCM3 - Putative minichromosome maintenance MCM complex subunit 3, expressed
Chr.5	22305787	22305787	A	A	G	SNP	OS05G0454500	Intronic	NA	NA	LOC_Os05g38040	expressed protein
Chr.5	22304002	22304002	T	T	G	SNP	OS05G0454500	Intronic	NA	NA	LOC_Os05g38040	expressed protein
Chr.5	22072093	22072093	A	A	G	SNP	OS05G0449600	Exonic	3UTR	NA	LOC_Os05g37700	periplasmic beta-glucosidase precursor, putative, expressed
Chr.5	15487151	15487151	A	A	G	SNP	OS05G0331200	Intronic	NA	NA	LOC_Os05g26660	NADH-ubiquinone oxidoreductase, mitochondrial precursor, putative, expressed
Chr.5	14307666	14307666	A	A	G	SNP	OS05G0312000	Intronic	NA	NA	LOC_Os05g24684	structural constituent of ribosome, putative, expressed
Chr.5	14303940	14303940	C	C	G	SNP	OS05G0312000	Intronic	NA	NA	LOC_Os05g24684	structural constituent of ribosome, putative, expressed
Chr.5	14166900	14166900	A	A	G	SNP	OS05G0310500	Exonic	3UTR	NA	LOC_Os05g24550	Papain family cysteine protease domain containing protein, expressed
Chr.5	14163732	14163732	T	T	G	SNP	OS05G0310500	Intronic	NA	NA	LOC_Os05g24550	Papain family cysteine protease domain containing protein, expressed
Chr.5	13607458	13607458	A	A	G	SNP	OS05G0302600	Exonic	3UTR	NA	LOC_Os05g23700	DNA-binding storekeeper protein-related, putative, expressed
Chr.5	7165616	7165616	A	A	G	SNP	OS05G0215600	Intronic	NA	NA	LOC_Os05g12474	expressed protein
Chr.5	3734141	3734141	A	A	G	SNP	OS05G0163700	Intronic	NA	NA	LOC_Os05g07090	acyl-coenzyme A dehydrogenase, mitochondrial precursor, putative, expressed
Chr.5	2763944	2763944	T	T	G	SNP	OS05G0148300	Intronic	NA	NA	LOC_Os05g05560	expressed protein
Chr.5	2030010	2030010	C	C	G	SNP	OS05G0134700	Exonic	3UTR	NA	LOC_Os05g04410	peroxidase precursor, putative, expressed
Chr.5	1002513	1002513	A	A	G	SNP	OS05G0119300	Exonic	5UTR	NA	LOC_Os05g02780	glycine-rich protein A3, putative, expressed
Chr.5	1002628	1002628	A	A	G	SNP	OS05G0119300	Exonic	5UTR	NA	LOC_Os05g02780	glycine-rich protein A3, putative, expressed
Chr.5	4139006	4139006	T	T	G	SNP	OS05G0169000	Exonic	5UTR	NA	LOC_Os05g07690	thioredoxin, putative, expressed
Chr.5	9321385	9321385	A	A	G	SNP	OS05G0253200	Intronic	NA	NA	LOC_Os05g16420	SHR5-receptor-like kinase, putative, expressed
Chr.5	14295399	14295399	A	A	G	SNP	OS05G0312000	Exonic	CDS	Silent	LOC_Os05g24684	structural constituent of ribosome, putative, expressed
Chr.5	15484686	15484686	A	A	G	SNP	OS05G0331200	Intronic	NA	NA	LOC_Os05g26660	NADH-ubiquinone oxidoreductase, mitochondrial precursor, putative, expressed
Chr.5	20039343	20039343	T	T	G	SNP	OS05G0410200	Exonic	3UTR	NA	LOC_Os05g33940	alpha/beta hydrolase fold, putative, expressed
Chr.5	21189769	21189769	T	T	G	SNP	OS05G0431700	Intronic	NA	NA	LOC_Os05g35650	peptide transporter PTR2, putative, expressed
Chr.5	21189861	21189861	A	A	G	SNP	OS05G0431700	Intronic	NA	NA	LOC_Os05g35650	peptide transporter PTR2, putative, expressed
Chr.5	21190033	21190033	A	A	G	SNP	OS05G0431700	Intronic	NA	NA	LOC_Os05g35650	peptide transporter PTR2, putative, expressed
Chr.5	22306220	22306220	A	A	G	SNP	OS05G0454500	Exonic	5UTR	NA	LOC_Os05g38040	expressed protein
Chr.5	23424364	23424364	A	A	G	SNP	OS05G0476200	Exonic	3UTR	NA	LOC_Os05g39850	MCM3 - Putative minichromosome maintenance MCM complex subunit 3, expressed
Chr.5	23497511	23497511	T	T	G	SNP	OS05G0477600	Exonic	5UTR	NA	LOC_Os05g39990	expansin precursor, putative, expressed
Chr.5	23752600	23752600	C	C	G	SNP	OS05G0482700	Intronic	NA	NA	LOC_Os05g40420	2,3-bisphosphoglycerate-independent phosphoglycerate mutase, putative, expressed
Chr.5	24326630	24326630	A	A	G	SNP	OS05G0495300	Exonic	3UTR	NA	LOC_Os05g41550	expressed protein
Chr.5	25546026	25546026	A	A	G	SNP	OS05G0515200	Exonic	3UTR	NA	LOC_Os05g43910	cytochrome P450, putative, expressed
Chr.5	27122088	27122088	T	T	G	SNP	OS05G0546400	Exonic	CDS	Silent	LOC_Os05g46840	proline-rich protein, putative, expressed
Chr.5	27122226	27122226	A	A	G	SNP	OS05G0546400	Exonic	CDS	Silent	LOC_Os05g46840	proline-rich protein, putative, expressed
Chr.5	28803998	28803998	A	A	G	SNP	OS05G0578600	Exonic	CDS	NA	LOC_Os05g50260	polygalacturonase, putative, expressed
Chr.5	14304967	14304968	GA	GA	G	INDEL	OS05G0312000	Intronic	NA	NA	LOC_Os05g24684	structural constituent of ribosome, putative, expressed
Chr.5	15078257	15078260	GCGA	GCGA	G	INDEL	OS05G0324700	Exonic	5UTR	NA	LOC_Os05g25920	expressed protein
Chr.5	15483768	15483769	GT	GT	G	INDEL	OS05G0331200	Intronic	NA	NA	LOC_Os05g26660	NADH-ubiquinone oxidoreductase, mitochondrial precursor, putative, expressed
Chr.5	25545469	25545474	GTTTTT	GTTTTT	G	INDEL	OS05G0515200	Intronic	NA	NA	LOC_Os05g43910	cytochrome P450, putative, expressed
Chr.5	27122757	27122761	GTACT	GTACT	G	INDEL	OS05G0546400	Exonic	3UTR	NA	LOC_Os05g46840	proline-rich protein, putative, expressed
Chr.5	27122082	27122085	GCTT	GCTT	G	INDEL	OS05G0546400	Exonic	CDS	InFrame	LOC_Os05g46840	proline-rich protein, putative, expressed
Chr.5	29348402	29348414	GCGCCGCCGCCGC	GCGCCGCCGCCGC	G	INDEL	OS05G0589400	Exonic	CDS	InFrame	LOC_Os05g51160	Myb transcription factor, putative, expressed
Chr.4	31442862	31442862	T	T	G	SNP	OS04G0619000	Exonic	3UTR	NA	LOC_Os04g52810	no apical meristem protein, putative, expressed
Chr.4	31441013	31441013	T	T	G	SNP	OS04G0619000	Intronic	NA	NA	LOC_Os04g52810	no apical meristem protein, putative, expressed
Chr.4	31181113	31181113	T	T	G	SNP	OS04G0614600	Intronic	NA	NA	LOC_Os04g52450	aminotransferase, putative, expressed
Chr.4	30174188	30174188	T	T	G	SNP	OS04G0598000	Exonic	5UTR	NA	LOC_Os04g50970	seed specific protein Bn15D1B, putative, expressed
Chr.4	27848844	27848844	T	T	G	SNP	OS04G0556500	Exonic	3UTR	NA	LOC_Os04g46980	cis-zeatin O-glucosyltransferase, putative, expressed
Chr.4	22623455	22623455	T	T	G	SNP	OS04G0453233	Intronic	NA	NA	LOC_Os04g38026	transporter family protein, putative, expressed
Chr.4	16005142	16005142	T	T	G	SNP	OS04G0339400	Exonic	3UTR	NA	LOC_Os04g27060	oxidoreductase, aldo/keto reductase family protein, putative, expressed
Chr.4	16005121	16005121	T	T	G	SNP	OS04G0339400	Exonic	3UTR	NA	LOC_Os04g27060	oxidoreductase, aldo/keto reductase family protein, putative, expressed
Chr.4	16004749	16004749	T	T	G	SNP	OS04G0339400	Exonic	CDS	Silent	LOC_Os04g27060	oxidoreductase, aldo/keto reductase family protein, putative, expressed
Chr.4	13648864	13648864	T	T	G	SNP	OS04G0304200	Intronic	NA	NA	LOC_Os04g23890	AGC_PVPK_like_kin82y.10 - ACG kinases include homologs to PKA, PKG and PKC, expressed
Chr.4	11241449	11241449	T	T	G	SNP	OS04G0269600	Intronic	NA	NA	LOC_Os04g20164	amine oxidase precursor, putative, expressed
Chr.4	6478912	6478912	T	T	G	SNP	OS04G0194500	Intronic	NA	NA	LOC_Os04g11820	white-brown complex homolog protein, putative, expressed
Chr.4	33500717	33500717	C	C	G	SNP	OS04G0656800	Intronic	NA	NA	LOC_Os04g56180	peroxidase precursor, putative, expressed
Chr.4	33418974	33418974	A	A	G	SNP	OS04G0655300	Exonic	5UTR	NA	LOC_Os04g56110	protein kinase, putative, expressed
Chr.4	33254772	33254772	A	A	G	SNP	OS04G0652700	Intronic	NA	NA	LOC_Os04g55850	nuclease PA3, putative, expressed
Chr.4	33254558	33254558	A	A	G	SNP	OS04G0652700	Intronic	NA	NA	LOC_Os04g55850	nuclease PA3, putative, expressed
Chr.4	33181178	33181178	T	T	G	SNP	OS04G0651000	Exonic	CDS	Missense	LOC_Os04g55740	peroxidase precursor, putative, expressed
Chr.4	33181159	33181159	A	A	G	SNP	OS04G0651000	Exonic	CDS	Silent	LOC_Os04g55740	peroxidase precursor, putative, expressed
Chr.4	33163824	33163824	A	A	G	SNP	OS04G0650700	Exonic	CDS	Silent	LOC_Os04g55710	transposon protein, putative, unclassified, expressed
Chr.4	33162111	33162111	A	A	G	SNP	OS04G0650700	Exonic	CDS	Missense	LOC_Os04g55710	transposon protein, putative, unclassified, expressed
Chr.4	33125608	33125608	A	A	G	SNP	OS04G0650000	Intronic	NA	NA	LOC_Os04g55650	oryzain alpha chain precursor, putative, expressed
Chr.4	31670344	31670344	A	A	G	SNP	OS04G0623300	Exonic	CDS	Silent	LOC_Os04g53190	CPuORF12 - conserved peptide uORF-containing transcript, expressed
Chr.4	31281657	31281657	C	C	G	SNP	OS04G0616300	Intronic	NA	NA	LOC_Os04g52600	SHR5-receptor-like kinase, putative, expressed
Chr.4	31180599	31180599	T	T	G	SNP	OS04G0614600	Intronic	NA	NA	LOC_Os04g52450	aminotransferase, putative, expressed
Chr.4	31178002	31178002	T	T	G	SNP	OS04G0614600	Intronic	NA	NA	LOC_Os04g52450	aminotransferase, putative, expressed
Chr.4	29525219	29525219	A	A	G	SNP	OS04G0584500	Intronic	NA	NA	LOC_Os04g49500	U-box domain-containing protein, putative, expressed
Chr.4	28298785	28298785	A	A	G	SNP	OS04G0564700	Intronic	NA	NA	LOC_Os04g47700	expressed protein
Chr.4	27848764	27848764	A	A	G	SNP	OS04G0556500	Exonic	3UTR	NA	LOC_Os04g46980	cis-zeatin O-glucosyltransferase, putative, expressed
Chr.4	27785997	27785997	A	A	G	SNP	OS04G0555300	Intronic	NA	NA	LOC_Os04g46880	transporter, major facilitator family, putative, expressed
Chr.4	27785031	27785031	A	A	G	SNP	OS04G0555300	Exonic	CDS	NA	LOC_Os04g46880	transporter, major facilitator family, putative, expressed
Chr.4	27783959	27783959	A	A	G	SNP	OS04G0555300	Exonic	3UTR	NA	LOC_Os04g46880	transporter, major facilitator family, putative, expressed

Chr.4	27766786	27766786	A	A	G	SNP	OS04G0555000	Exonic	CDS	Silent	LOC_Os04g46860	scarecrow, putative, expressed
Chr.4	27641930	27641930	T	T	G	SNP	OS04G0551800	Exonic	CDS	Silent	LOC_Os04g46620	T-complex protein, putative, expressed
Chr.4	27641700	27641700	C	C	G	SNP	OS04G0551800	Exonic	CDS	Missense	LOC_Os04g46620	T-complex protein, putative, expressed
Chr.4	27640087	27640087	A	A	G	SNP	OS04G0551800	Intronic	NA	NA	LOC_Os04g46620	T-complex protein, putative, expressed
Chr.4	27639651	27639651	T	T	G	SNP	OS04G0551800	Intronic	NA	NA	LOC_Os04g46620	T-complex protein, putative, expressed
Chr.4	27236994	27236994	C	C	G	SNP	OS04G0543900	Intronic	NA	NA	LOC_Os04g45970	glutamate dehydrogenase protein, putative, expressed
Chr.4	27194777	27194777	C	C	G	SNP	OS04G0542900	Exonic	3UTR	NA	LOC_Os04g45910	placental protein 11 precursor, putative, expressed
Chr.4	27192908	27192908	C	C	G	SNP	OS04G0542900	Intronic	NA	NA	LOC_Os04g45910	placental protein 11 precursor, putative, expressed
Chr.4	27192735	27192735	A	A	G	SNP	OS04G0542900	Exonic	CDS	Missense	LOC_Os04g45910	placental protein 11 precursor, putative, expressed
Chr.4	27192449	27192449	A	A	G	SNP	OS04G0542900	Intronic	NA	NA	LOC_Os04g45910	placental protein 11 precursor, putative, expressed
Chr.4	27192324	27192324	T	T	G	SNP	OS04G0542900	Intronic	NA	NA	LOC_Os04g45910	placental protein 11 precursor, putative, expressed
Chr.4	25664103	25664103	A	A	G	SNP	OS04G0513400	Intronic	NA	NA	LOC_Os04g43390	Os4bglu16 - monolignol beta-glucoside homologue, expressed
Chr.4	25194638	25194638	C	C	G	SNP	OS04G0504800	Exonic	3UTR	NA	LOC_Os04g42600	polyadenylate-binding protein, putative, expressed
Chr.4	25194361	25194361	A	A	G	SNP	OS04G0504800	Exonic	3UTR	NA	LOC_Os04g42600	polyadenylate-binding protein, putative, expressed
Chr.4	25194155	25194155	A	A	G	SNP	OS04G0504800	Exonic	3UTR	NA	LOC_Os04g42600	polyadenylate-binding protein, putative, expressed
Chr.4	25194130	25194130	A	A	G	SNP	OS04G0504800	Exonic	3UTR	NA	LOC_Os04g42600	polyadenylate-binding protein, putative, expressed
Chr.4	25133999	25133999	C	C	G	SNP	OS04G0503500	Intronic	NA	NA	LOC_Os04g42470	regulatory subunit, putative, expressed
Chr.4	25133942	25133942	T	T	G	SNP	OS04G0503500	Intronic	NA	NA	LOC_Os04g42470	regulatory subunit, putative, expressed
Chr.4	25133498	25133498	A	A	G	SNP	OS04G0503500	Exonic	CDS	Silent	LOC_Os04g42470	regulatory subunit, putative, expressed
Chr.4	25131629	25131629	A	A	G	SNP	OS04G0503500	Exonic	CDS	Silent	LOC_Os04g42470	regulatory subunit, putative, expressed
Chr.4	24886259	24886259	A	A	G	SNP	OS04G0497600	Exonic	CDS	Missense	LOC_Os04g42010	RNA binding protein, putative, expressed
Chr.4	24885161	24885161	A	A	G	SNP	OS04G0497600	Exonic	CDS	Silent	LOC_Os04g42010	RNA binding protein, putative, expressed
Chr.4	24851562	24851562	A	A	G	SNP	OS04G0496800	Intronic	NA	NA	LOC_Os04g41950	calcium-binding mitochondrial protein anon-60Da, putative, expressed
Chr.4	24849246	24849246	A	A	G	SNP	OS04G0496800	Intronic	NA	NA	LOC_Os04g41950	calcium-binding mitochondrial protein anon-60Da, putative, expressed
Chr.4	24528957	24528957	A	A	G	SNP	OS04G0490800	Intronic	NA	NA	LOC_Os04g41340	4-nitrophenylphosphatase, putative, expressed
Chr.4	24528533	24528533	T	T	G	SNP	OS04G0490800	Intronic	NA	NA	LOC_Os04g41340	4-nitrophenylphosphatase, putative, expressed
Chr.4	24528400	24528400	A	A	G	SNP	OS04G0490800	Exonic	CDS	Silent	LOC_Os04g41340	4-nitrophenylphosphatase, putative, expressed
Chr.4	24353786	24353786	T	T	G	SNP	OS04G0487300	Exonic	3UTR	NA	LOC_Os04g41040	DNA-directed RNA polymerases I, II, and III subunit RPABC1, putative, expressed
Chr.4	24324287	24324287	T	T	G	SNP	OS04G0486900	Intronic	NA	NA	LOC_Os04g40980	Divergent PAP2 family domain containing protein, expressed
Chr.4	24055528	24055528	A	A	G	SNP	OS04G0480900	Intronic	NA	NA	LOC_Os04g40490	glycosyl hydrolase family 5 protein, putative, expressed
Chr.4	23139686	23139686	A	A	G	SNP	OS04G0463500	Exonic	CDS	Silent	LOC_Os04g38950	class I glutamine amidotransferase, putative, expressed
Chr.4	22185352	22185352	A	A	G	SNP	OS04G0444800	Exonic	CDS	Silent	LOC_Os04g36720	ferric-chelate reductase, putative, expressed
Chr.4	22184296	22184296	A	A	G	SNP	OS04G0444800	Exonic	CDS	Missense	LOC_Os04g36720	ferric-chelate reductase, putative, expressed
Chr.4	22183525	22183525	A	A	G	SNP	OS04G0444800	Intronic	NA	NA	LOC_Os04g36720	ferric-chelate reductase, putative, expressed
Chr.4	20319162	20319162	A	A	G	SNP	OS04G0411400	Exonic	3UTR	NA	LOC_Os04g33570	RCN4 Centroradialis-like1 homologous to TFL1 gene; contains Pfam profile PF01161: Phosphatidylethanolamine-binding protein,
Chr.4	20144204	20144204	C	C	G	SNP	OS04G0405900	Exonic	CDS	NA	LOC_Os04g33310	expressed protein
Chr.4	18564235	18564235	A	A	G	SNP	OS04G0379900	Exonic	CDS	Silent	LOC_Os04g31070	acyl-desaturase, chloroplast precursor, putative, expressed
Chr.4	5325613	5325613	T	T	G	SNP	OS04G0178300	Intronic	NA	NA	LOC_Os04g09900	ent-kaurene synthase, chloroplast precursor, putative, expressed
Chr.4	5325530	5325530	A	A	G	SNP	OS04G0178300	Intronic	NA	NA	LOC_Os04g09900	ent-kaurene synthase, chloroplast precursor, putative, expressed
Chr.4	5325500	5325500	A	A	G	SNP	OS04G0178300	Intronic	NA	NA	LOC_Os04g09900	ent-kaurene synthase, chloroplast precursor, putative, expressed
Chr.4	5325411	5325411	T	T	G	SNP	OS04G0178300	Exonic	CDS	Silent	LOC_Os04g09900	ent-kaurene synthase, chloroplast precursor, putative, expressed
Chr.4	5324999	5324999	C	C	G	SNP	OS04G0178300	Intronic	NA	NA	LOC_Os04g09900	ent-kaurene synthase, chloroplast precursor, putative, expressed
Chr.4	5324820	5324820	C	C	G	SNP	OS04G0178300	Exonic	CDS	Silent	LOC_Os04g09900	ent-kaurene synthase, chloroplast precursor, putative, expressed
Chr.4	5324353	5324353	A	A	G	SNP	OS04G0178300	Intronic	NA	NA	LOC_Os04g09900	ent-kaurene synthase, chloroplast precursor, putative, expressed
Chr.4	5324242	5324242	A	A	G	SNP	OS04G0178300	Exonic	CDS	Silent	LOC_Os04g09900	ent-kaurene synthase, chloroplast precursor, putative, expressed
Chr.4	5323813	5323813	T	T	G	SNP	OS04G0178300	Intronic	NA	NA	LOC_Os04g09900	ent-kaurene synthase, chloroplast precursor, putative, expressed
Chr.4	5323032	5323032	A	A	G	SNP	OS04G0178300	Intronic	NA	NA	LOC_Os04g09900	ent-kaurene synthase, chloroplast precursor, putative, expressed
Chr.4	5320794	5320794	A	A	G	SNP	OS04G0178300	Intronic	NA	NA	LOC_Os04g09900	ent-kaurene synthase, chloroplast precursor, putative, expressed
Chr.4	5320156	5320156	A	A	G	SNP	OS04G0178300	Intronic	NA	NA	LOC_Os04g09900	ent-kaurene synthase, chloroplast precursor, putative, expressed
Chr.4	5319945	5319945	A	A	G	SNP	OS04G0178300	Intronic	NA	NA	LOC_Os04g09900	ent-kaurene synthase, chloroplast precursor, putative, expressed
Chr.4	318567	318567	A	A	G	SNP	OS04G0104900	Intronic	NA	NA	LOC_Os04g01470	O-methyltransferase, putative, expressed
Chr.4	31062621	31062623	GTA	GTA	G	INDEL	OS04G0612500	Exonic	3UTR	NA	LOC_Os04g52260	LTPL124 - Protease inhibitor/seed storage/LTP family protein precursor, expressed
Chr.4	24055405	24055406	GT	GT	G	INDEL	OS04G0480900	Intronic	NA	NA	LOC_Os04g40490	glycosyl hydrolase family 5 protein, putative, expressed
Chr.4	27234941	27234942	GT	GT	G	INDEL	OS04G0543900	Exonic	3UTR	NA	LOC_Os04g45970	glutamate dehydrogenase protein, putative, expressed
Chr.4	27643183	27643184	GT	GT	G	INDEL	OS04G0551800	Intronic	NA	NA	LOC_Os04g46620	T-complex protein, putative, expressed
Chr.4	5324111	5324113	GAT	GAT	G	INDEL	OS04G0178300	Intronic	NA	NA	LOC_Os04g09900	ent-kaurene synthase, chloroplast precursor, putative, expressed
Chr.4	23315257	23315260	GAGA	GAGA	G	INDEL	OS04G0466600	Exonic	5UTR	NA	LOC_Os04g39210	agmatine deiminase, putative, expressed
Chr.4	24847147	24847148	GA	GA	G	INDEL	OS04G0496800	Exonic	3UTR	NA	LOC_Os04g41950	calcium-binding mitochondrial protein anon-60Da, putative, expressed
Chr.4	29562109	29562110	GA	GA	G	INDEL	OS04G0585100	Intronic	NA	NA	LOC_Os04g49560	zinc finger family protein, putative, expressed
Chr.4	31112825	31112826	GA	GA	G	INDEL	OS04G0613700	Intronic	NA	NA	LOC_Os04g52370	UTP--glucose-1-phosphate uridylyltransferase, putative, expressed
Chr.4	31242556	31242556	C	C	G	SNP	OS04G0615700	Exonic	CDS	Silent	LOC_Os04g52540	retrotransposon protein, putative, unclassified, expressed
Chr.4	31181422	31181422	C	C	G	SNP	OS04G0614600	Intronic	NA	NA	LOC_Os04g52450	aminotransferase, putative, expressed
Chr.4	31178449	31178449	C	C	G	SNP	OS04G0614600	Intronic	NA	NA	LOC_Os04g52450	aminotransferase, putative, expressed
Chr.4	29553073	29553073	C	C	G	SNP	OS04G0585000	Exonic	5UTR	NA	LOC_Os04g49550	RING-H2 finger protein ATL2A, putative, expressed
Chr.4	27643712	27643712	C	C	G	SNP	OS04G0551800	Exonic	3UTR	NA	LOC_Os04g46620	T-complex protein, putative, expressed
Chr.4	22261417	22261417	C	C	G	SNP	OS04G0446500	Intronic	NA	NA	LOC_Os04g36890	peptidyl-prolyl cis-trans isomerase, FKBP-type, putative, expressed
Chr.4	22019563	22019563	C	C	G	SNP	OS04G0442200	Intronic	NA	NA	LOC_Os04g36062	expressed protein
Chr.4	20388254	20388254	C	C	G	SNP	OS04G0412500	Intronic	NA	NA	LOC_Os04g33660	bifunctional monodehydroascorbate reductase and carbonic anhydrasenectarin-3 precursor, putative, expressed
Chr.4	13643763	13643763	C	C	G	SNP	OS04G0304200	Intronic	NA	NA	LOC_Os04g23890	AGC_PVPK_like_kin82y.10 - ACG kinases include homologs to PKA, PKG and PKC, expressed
Chr.4	6481043	6481043	C	C	G	SNP	OS04G0194500	Intronic	NA	NA	LOC_Os04g11820	white-brown complex homolog protein, putative, expressed
Chr.4	6480788	6480788	C	C	G	SNP	OS04G0194500	Intronic	NA	NA	LOC_Os04g11820	white-brown complex homolog protein, putative, expressed
Chr.4	6479880	6479880	C	C	G	SNP	OS04G0194500	Intronic	NA	NA	LOC_Os04g11820	white-brown complex homolog protein, putative, expressed
Chr.4	6479072	6479072	C	C	G	SNP	OS04G0194500	Intronic	NA	NA	LOC_Os04g11820	white-brown complex homolog protein, putative, expressed
Chr.4	5486697	5486697	C	C	G	SNP	OS04G0180400	Exonic	3UTR	NA	LOC_Os04g10160	cytochrome P450, putative, expressed
Chr.4	485738	485738	C	C	G	SNP	OS04G0107900	Exonic	3UTR	NA	LOC_Os04g01740	heat shock protein, putative, expressed
Chr.4	33533521	33533521	A	A	G	SNP	OS04G0657500	Exonic	3UTR	NA	LOC_Os04g56240	lipase, putative, expressed
Chr.4	33532189	33532189	A	A	G	SNP	OS04G0657500	Intronic	NA	NA	LOC_Os04g56240	lipase, putative, expressed
Chr.4	33531847	33531847	A	A	G	SNP	OS04G0657500	Intronic	NA	NA	LOC_Os04g56240	lipase, putative, expressed
Chr.4	31464800	31464800	A	A	G	SNP	OS04G0619400	Exonic	CDS	Silent	LOC_Os04g52840	tyrosine protein kinase domain containing protein, putative, expressed
Chr.4	31442624	31442624	A	A	G	SNP	OS04G0619000	Exonic	3UTR	NA	LOC_Os04g52810	no apical meristem protein, putative, expressed
Chr.4	31441460	31441460	A	A	G	SNP	OS04G0619000	Intronic	NA	NA	LOC_Os04g52810	no apical meristem protein, putative, expressed
Chr.4	31441410	31441410	A	A	G	SNP	OS04G0619000	Intronic	NA	NA	LOC_Os04g52810	no apical meristem protein, putative, expressed
Chr.4	31441405	31441405	A	A	G	SNP	OS04G0619000	Intronic	NA	NA	LOC_Os04g52810	no apical meristem protein, putative, expressed
Chr.4	31441394	31441394	A	A	G	SNP	OS04G0619000	Intronic	NA	NA	LOC_Os04g52810	no apical meristem protein, putative, expressed
Chr.4	31441387	31441387	A	A	G	SNP	OS04G0619000	Intronic	NA	NA	LOC_Os04g52810	no apical meristem protein, putative, expressed

Chr.4	31439692	31439692	A	A	G	SNP	OS04G0619000	Exonic	3UTR	NA	LOC_Os04g52810	no apical meristem protein, putative, expressed
Chr.4	31439687	31439687	A	A	G	SNP	OS04G0619000	Exonic	3UTR	NA	LOC_Os04g52810	no apical meristem protein, putative, expressed
Chr.4	31280009	31280009	A	A	G	SNP	OS04G0616300	Intronic	NA	NA	LOC_Os04g52600	SHR5-receptor-like kinase, putative, expressed
Chr.4	31181459	31181459	A	A	G	SNP	OS04G0614600	Intronic	NA	NA	LOC_Os04g52450	aminotransferase, putative, expressed
Chr.4	30910933	30910933	A	A	G	SNP	OS04G0609900	Intronic	NA	NA	LOC_Os04g52040	TNP1, putative, expressed
Chr.4	30288898	30288898	A	A	G	SNP	OS04G0600200	Intronic	NA	NA	LOC_Os04g51150	transposon protein, putative, unclassified, expressed
Chr.4	29340545	29340545	A	A	G	SNP	OS04G0581100	Exonic	CDS	Silent	LOC_Os04g49210	naringenin,2-oxoglutarate 3-dioxygenase, putative, expressed
Chr.4	29317417	29317417	A	A	G	SNP	OS04G0580800	Exonic	3UTR	NA	LOC_Os04g49160	zinc finger, C3HC4 type domain containing protein, expressed
Chr.4	29045681	29045681	A	A	G	SNP	OS04G0576500	Intronic	NA	NA	LOC_Os04g48710	expressed protein
Chr.4	27848772	27848772	A	A	G	SNP	OS04G0556500	Exonic	3UTR	NA	LOC_Os04g46980	cis-zeatin O-glucosyltransferase, putative, expressed
Chr.4	27639762	27639762	A	A	G	SNP	OS04G0551800	Intronic	NA	NA	LOC_Os04g46620	T-complex protein, putative, expressed
Chr.4	27508588	27508588	A	A	G	SNP	OS04G0549600	Exonic	CDS	Silent	LOC_Os04g46390	chaperone protein dnaJ, putative, expressed
Chr.4	27192467	27192467	A	A	G	SNP	OS04G0542900	Intronic	NA	NA	LOC_Os04g45910	placental protein 11 precursor, putative, expressed
Chr.4	25844339	25844339	A	A	G	SNP	OS04G0517100	Exonic	5UTR	NA	LOC_Os04g43680	MYB family transcription factor, putative, expressed
Chr.4	25198098	25198098	A	A	G	SNP	OS04G0504800	Exonic	CDS	Silent	LOC_Os04g42600	polyadenylate-binding protein, putative, expressed
Chr.4	24882925	24882925	A	A	G	SNP	OS04G0497600	Exonic	3UTR	NA	LOC_Os04g42010	RNA binding protein, putative, expressed
Chr.4	23829882	23829882	A	A	G	SNP	OS04G0476100	Exonic	3UTR	NA	LOC_Os04g40040	copper methylamine oxidase precursor, putative, expressed
Chr.4	23316596	23316596	A	A	G	SNP	OS04G0466600	Intronic	NA	NA	LOC_Os04g39210	agmatine deiminase, putative, expressed
Chr.4	20426252	20426252	A	A	G	SNP	OS04G0413500	Intronic	NA	NA	LOC_Os04g33740	glycosyl hydrolases, putative, expressed
Chr.4	20425065	20425065	A	A	G	SNP	OS04G0413500	Intronic	NA	NA	LOC_Os04g33740	glycosyl hydrolases, putative, expressed
Chr.4	20422597	20422597	A	A	G	SNP	OS04G0413500	Intronic	NA	NA	LOC_Os04g33740	glycosyl hydrolases, putative, expressed
Chr.4	20389439	20389439	A	A	G	SNP	OS04G0412500	Intronic	NA	NA	LOC_Os04g33660	bifunctional monodehydroascorbate reductase and carbonic anhydrasenectarin-3 precursor, putative, expressed
Chr.4	16005034	16005034	A	A	G	SNP	OS04G0339400	Exonic	CDS	Silent	LOC_Os04g27060	oxidoreductase, aldo/keto reductase family protein, putative, expressed
Chr.4	16004950	16004950	A	A	G	SNP	OS04G0339400	Exonic	CDS	Silent	LOC_Os04g27060	oxidoreductase, aldo/keto reductase family protein, putative, expressed
Chr.4	16004289	16004289	A	A	G	SNP	OS04G0339400	Intronic	NA	NA	LOC_Os04g27060	oxidoreductase, aldo/keto reductase family protein, putative, expressed
Chr.4	16002894	16002894	A	A	G	SNP	OS04G0339400	Intronic	NA	NA	LOC_Os04g27060	oxidoreductase, aldo/keto reductase family protein, putative, expressed
Chr.4	13654382	13654382	A	A	G	SNP	OS04G0304200	Intronic	NA	NA	LOC_Os04g23890	AGC_PVPK_like_kin82y.10 - ACG kinases include homologs to PKA, PKG and PKC, expressed
Chr.4	13650660	13650660	A	A	G	SNP	OS04G0304200	Intronic	NA	NA	LOC_Os04g23890	AGC_PVPK_like_kin82y.10 - ACG kinases include homologs to PKA, PKG and PKC, expressed
Chr.4	13641870	13641870	A	A	G	SNP	OS04G0304200	Exonic	3UTR	NA	LOC_Os04g23890	AGC_PVPK_like_kin82y.10 - ACG kinases include homologs to PKA, PKG and PKC, expressed
Chr.4	11281627	11281627	A	A	G	SNP	OS04G0270100	Intronic	NA	NA	LOC_Os04g20220	elongation factor Tu, putative, expressed
Chr.4	6481318	6481318	A	A	G	SNP	OS04G0194500	Exonic	CDS	Silent	LOC_Os04g11820	white-brown complex homolog protein, putative, expressed
Chr.4	6481074	6481074	A	A	G	SNP	OS04G0194500	Intronic	NA	NA	LOC_Os04g11820	white-brown complex homolog protein, putative, expressed
Chr.4	6480498	6480498	A	A	G	SNP	OS04G0194500	Intronic	NA	NA	LOC_Os04g11820	white-brown complex homolog protein, putative, expressed
Chr.4	6479819	6479819	A	A	G	SNP	OS04G0194500	Intronic	NA	NA	LOC_Os04g11820	white-brown complex homolog protein, putative, expressed
Chr.4	6479720	6479720	A	A	G	SNP	OS04G0194500	Exonic	CDS	Silent	LOC_Os04g11820	white-brown complex homolog protein, putative, expressed
Chr.4	6479212	6479212	A	A	G	SNP	OS04G0194500	Exonic	CDS	Silent	LOC_Os04g11820	white-brown complex homolog protein, putative, expressed
Chr.4	6478730	6478730	A	A	G	SNP	OS04G0194500	Intronic	NA	NA	LOC_Os04g11820	white-brown complex homolog protein, putative, expressed
Chr.4	6478293	6478293	A	A	G	SNP	OS04G0194500	Exonic	CDS	Missense	LOC_Os04g11820	white-brown complex homolog protein, putative, expressed
Chr.4	6478175	6478175	A	A	G	SNP	OS04G0194500	Exonic	CDS	Silent	LOC_Os04g11820	white-brown complex homolog protein, putative, expressed
Chr.4	6478004	6478004	A	A	G	SNP	OS04G0194500	Exonic	CDS	Silent	LOC_Os04g11820	white-brown complex homolog protein, putative, expressed
Chr.4	619380	619380	A	A	G	SNP	OS04G0110600	Intronic	NA	NA	LOC_Os04g02000	zinc finger family protein, putative, expressed
Chr.4	485897	485897	A	A	G	SNP	OS04G0107900	Exonic	3UTR	NA	LOC_Os04g01740	heat shock protein, putative, expressed
Chr.4	347030	347030	C	C	G	SNP	OS04G0105200	Exonic	CDS	Missense	LOC_Os04g01500	expressed protein
Chr.4	485913	485913	A	A	G	SNP	OS04G0107900	Exonic	3UTR	NA	LOC_Os04g01740	heat shock protein, putative, expressed
Chr.4	483338	483338	A	A	G	SNP	OS04G0107900	Exonic	5UTR	NA	LOC_Os04g01740	heat shock protein, putative, expressed
Chr.4	621870	621870	A	A	G	SNP	OS04G0110600	Exonic	3UTR	NA	LOC_Os04g02000	zinc finger family protein, putative, expressed
Chr.4	1151768	1151768	C	C	G	SNP	OS04G0119500	Exonic	5UTR	NA	LOC_Os04g02910	membrane related protein CP5, putative, expressed
Chr.4	2036614	2036614	C	C	G	SNP	OS04G0132500	Exonic	CDS	Silent	LOC_Os04g04330	receptor-like protein kinase 2 precursor, putative, expressed
Chr.4	4458477	4458477	A	A	G	SNP	OS04G0165700	Intronic	NA	NA	LOC_Os04g08350	cysteine synthase, chloroplast/chromoplast precursor, putative, expressed
Chr.4	4461338	4461338	A	A	G	SNP	OS04G0165700	Intronic	NA	NA	LOC_Os04g08350	cysteine synthase, chloroplast/chromoplast precursor, putative, expressed
Chr.4	5321463	5321463	A	A	G	SNP	OS04G0178300	Intronic	NA	NA	LOC_Os04g09900	ent-kaurene synthase, chloroplast precursor, putative, expressed
Chr.4	5321593	5321593	A	A	G	SNP	OS04G0178300	Intronic	NA	NA	LOC_Os04g09900	ent-kaurene synthase, chloroplast precursor, putative, expressed
Chr.4	5321613	5321613	A	A	G	SNP	OS04G0178300	Intronic	NA	NA	LOC_Os04g09900	ent-kaurene synthase, chloroplast precursor, putative, expressed
Chr.4	5321623	5321623	T	T	G	SNP	OS04G0178300	Intronic	NA	NA	LOC_Os04g09900	ent-kaurene synthase, chloroplast precursor, putative, expressed
Chr.4	5321634	5321634	A	A	G	SNP	OS04G0178300	Intronic	NA	NA	LOC_Os04g09900	ent-kaurene synthase, chloroplast precursor, putative, expressed
Chr.4	5323423	5323423	A	A	G	SNP	OS04G0178300	Intronic	NA	NA	LOC_Os04g09900	ent-kaurene synthase, chloroplast precursor, putative, expressed
Chr.4	5324333	5324333	C	C	G	SNP	OS04G0178300	Intronic	NA	NA	LOC_Os04g09900	ent-kaurene synthase, chloroplast precursor, putative, expressed
Chr.4	5324481	5324481	A	A	G	SNP	OS04G0178300	Intronic	NA	NA	LOC_Os04g09900	ent-kaurene synthase, chloroplast precursor, putative, expressed
Chr.4	5324507	5324507	T	T	G	SNP	OS04G0178300	Intronic	NA	NA	LOC_Os04g09900	ent-kaurene synthase, chloroplast precursor, putative, expressed
Chr.4	5325559	5325559	A	A	G	SNP	OS04G0178300	Intronic	NA	NA	LOC_Os04g09900	ent-kaurene synthase, chloroplast precursor, putative, expressed
Chr.4	5325669	5325669	T	T	G	SNP	OS04G0178300	Intronic	NA	NA	LOC_Os04g09900	ent-kaurene synthase, chloroplast precursor, putative, expressed
Chr.4	13650871	13650871	C	C	G	SNP	OS04G0304200	Intronic	NA	NA	LOC_Os04g23890	AGC_PVPK_like_kin82y.10 - ACG kinases include homologs to PKA, PKG and PKC, expressed
Chr.4	13651012	13651012	A	A	G	SNP	OS04G0304200	Intronic	NA	NA	LOC_Os04g23890	AGC_PVPK_like_kin82y.10 - ACG kinases include homologs to PKA, PKG and PKC, expressed
Chr.4	16004293	16004293	A	A	G	SNP	OS04G0339400	Intronic	NA	NA	LOC_Os04g27060	oxidoreductase, aldo/keto reductase family protein, putative, expressed
Chr.4	20424686	20424686	A	A	G	SNP	OS04G0413500	Intronic	NA	NA	LOC_Os04g33740	glycosyl hydrolases, putative, expressed
Chr.4	20424790	20424790	C	C	G	SNP	OS04G0413500	Intronic	NA	NA	LOC_Os04g33740	glycosyl hydrolases, putative, expressed
Chr.4	20935979	20935979	C	C	G	SNP	OS04G0423400	Exonic	5UTR	NA	LOC_Os04g34600	abscisic stress-ripening, putative, expressed
Chr.4	22182604	22182604	C	C	G	SNP	OS04G0444800	Exonic	5UTR	NA	LOC_Os04g36720	ferric-chelate reductase, putative, expressed
Chr.4	22624800	22624800	A	A	G	SNP	OS04G0453233	Intronic	NA	NA	LOC_Os04g38026	transporter family protein, putative, expressed
Chr.4	22626308	22626308	A	A	G	SNP	OS04G0453233	Intronic	NA	NA	LOC_Os04g38026	transporter family protein, putative, expressed
Chr.4	24321737	24321737	A	A	G	SNP	OS04G0486900	Intronic	NA	NA	LOC_Os04g40980	Divergent PAP2 family domain containing protein, expressed
Chr.4	27234032	27234032	T	T	G	SNP	OS04G0543900	Intronic	NA	NA	LOC_Os04g45970	glutamate dehydrogenase protein, putative, expressed
Chr.4	27237599	27237599	A	A	G	SNP	OS04G0543900	Intronic	NA	NA	LOC_Os04g45970	glutamate dehydrogenase protein, putative, expressed
Chr.4	27508130	27508130	A	A	G	SNP	OS04G0549600	Intronic	NA	NA	LOC_Os04g46390	chaperone protein dnaJ, putative, expressed
Chr.4	27568602	27568602	A	A	G	SNP	OS04G0550600	Exonic	CDS	Silent	LOC_Os04g46470	carotenoid cleavage dioxygenase 7, chloroplast precursor, putative, expressed
Chr.4	27569991	27569991	A	A	G	SNP	OS04G0550600	Exonic	CDS	Silent	LOC_Os04g46470	carotenoid cleavage dioxygenase 7, chloroplast precursor, putative, expressed
Chr.4	27638083	27638083	A	A	G	SNP	OS04G0551800	Exonic	5UTR	NA	LOC_Os04g46620	T-complex protein, putative, expressed
Chr.4	27784101	27784101	A	A	G	SNP	OS04G0555300	Exonic	CDS	NA	LOC_Os04g46880	transporter, major facilitator family, putative, expressed
Chr.4	27849687	27849687	C	C	G	SNP	OS04G0556500	Exonic	CDS	Missense	LOC_Os04g46980	cis-zeatin O-glucosyltransferase, putative, expressed
Chr.4	29044378	29044378	C	C	G	SNP	OS04G0576500	Intronic	NA	NA	LOC_Os04g48710	expressed protein
Chr.4	29132378	29132378	A	A	G	SNP	OS04G0578000	Exonic	CDS	Silent	LOC_Os04g48850	aminotransferase, classes I and II, domain containing protein, expressed
Chr.4	29317973	29317973	A	A	G	SNP	OS04G0580800	Exonic	CDS	Silent	LOC_Os04g49160	zinc finger, C3HC4 type domain containing protein, expressed
Chr.4	29339774	29339774	T	T	G	SNP	OS04G0581100	Exonic	CDS	Missense	LOC_Os04g49210	naringenin,2-oxoglutarate 3-dioxygenase, putative, expressed
Chr.4	29340367	29340367	A	A	G	SNP	OS04G0581100	Intronic	NA	NA	LOC_Os04g49210	naringenin,2-oxoglutarate 3-dioxygenase, putative, expressed
Chr.4	29340458	29340458	A	A	G	SNP	OS04G0581100	Intronic	NA	NA	LOC_Os04g49210	naringenin,2-oxoglutarate 3-dioxygenase, putative, expressed

Chr.4	29661861	29661861	C	C	G	SNP	OS04G0587400	Exonic	CDS	Silent	LOC_Os04g49748	purine permease, putative, expressed
Chr.4	31240614	31240614	C	C	G	SNP	OS04G0615700	Exonic	CDS	Silent	LOC_Os04g52540	retrotransposon protein, putative, unclassified, expressed
Chr.4	31440039	31440039	A	A	G	SNP	OS04G0619000	Exonic	3UTR	NA	LOC_Os04g52810	no apical meristem protein, putative, expressed
Chr.4	31440177	31440177	A	A	G	SNP	OS04G0619000	Exonic	3UTR	NA	LOC_Os04g52810	no apical meristem protein, putative, expressed
Chr.4	31440229	31440229	A	A	G	SNP	OS04G0619000	Exonic	3UTR	NA	LOC_Os04g52810	no apical meristem protein, putative, expressed
Chr.4	31442263	31442263	T	T	G	SNP	OS04G0619000	Exonic	3UTR	NA	LOC_Os04g52810	no apical meristem protein, putative, expressed
Chr.4	31442423	31442423	T	T	G	SNP	OS04G0619000	Exonic	3UTR	NA	LOC_Os04g52810	no apical meristem protein, putative, expressed
Chr.4	31442839	31442839	C	C	G	SNP	OS04G0619000	Exonic	3UTR	NA	LOC_Os04g52810	no apical meristem protein, putative, expressed
Chr.4	31443277	31443277	C	C	G	SNP	OS04G0619000	Exonic	CDS	Silent	LOC_Os04g52810	no apical meristem protein, putative, expressed
Chr.4	33125125	33125125	C	C	G	SNP	OS04G0650000	Intronic	NA	NA	LOC_Os04g55650	oryzain alpha chain precursor, putative, expressed
Chr.4	33164250	33164250	A	A	G	SNP	OS04G0650700	Exonic	CDS	Silent	LOC_Os04g55710	transposon protein, putative, unclassified, expressed
Chr.4	33167605	33167605	A	A	G	SNP	OS04G0650800	Exonic	CDS	Silent	LOC_Os04g55720	D-3-phosphoglycerate dehydrogenase, chloroplast precursor, putative, expressed
Chr.4	334529	334547	GAAGCACCAGCGTTGCCCC	GAAGCACCAGCGTTGCCCC	G	INDEL	OS04G0105100	Exonic	CDS	InFrame	LOC_Os04g01490	zinc finger, C3HC4 type, domain containing protein, expressed
Chr.4	6477461	6477462	GC	GC	G	INDEL	OS04G0194500	Intronic	NA	NA	LOC_Os04g11820	white-brown complex homolog protein, putative, expressed
Chr.4	6480936	6480944	GGCACCACC	GGCACCACC	G	INDEL	OS04G0194500	Intronic	NA	NA	LOC_Os04g11820	white-brown complex homolog protein, putative, expressed
Chr.4	6481410	6481411	GT	GT	G	INDEL	OS04G0194500	Intronic	NA	NA	LOC_Os04g11820	white-brown complex homolog protein, putative, expressed
Chr.4	13654680	13654686	GGGAAGA	GGGAAGA	G	INDEL	OS04G0304200	Exonic	5UTR	NA	LOC_Os04g23890	AGC_PVPK_like_kin82y.10 - ACG kinases include homologs to PKA, PKG and PKC, expressed
Chr.4	16003112	16003113	GT	GT	G	INDEL	OS04G0339400	Intronic	NA	NA	LOC_Os04g27060	oxidoreductase, aldo/keto reductase family protein, putative, expressed
Chr.4	22261754	22261755	GA	GA	G	INDEL	OS04G0446500	Intronic	NA	NA	LOC_Os04g36890	peptidyl-prolyl cis-trans isomerase, FKBP-type, putative, expressed
Chr.4	22623916	22623917	GA	GA	G	INDEL	OS04G0453233	Intronic	NA	NA	LOC_Os04g38026	transporter family protein, putative, expressed
Chr.4	23165757	23165758	GA	GA	G	INDEL	OS04G0464100	Exonic	5UTR	NA	LOC_Os04g39010	heavy metal associated domain containing protein, expressed
Chr.4	24354961	24354962	GA	GA	G	INDEL	OS04G0487300	Intronic	NA	NA	LOC_Os04g41040	DNA-directed RNA polymerases I, II, and III subunit RPABC1, putative, expressed
Chr.4	27371329	27371330	GT	GT	G	INDEL	OS04G0546800	Exonic	3UTR	NA	LOC_Os04g46220	ethylene-responsive transcription factor, putative, expressed
Chr.4	27505527	27505542	GCTCGCCTCCGTCCGC	GCTCGCCTCCGTCCGC	G	INDEL	OS04G0549600	Exonic	5UTR	NA	LOC_Os04g46390	chaperone protein dnaJ, putative, expressed
Chr.4	27567958	27567967	GGCCGCCGCC	GGCCGCCGCC	G	INDEL	OS04G0550600	Exonic	CDS	InFrame	LOC_Os04g46470	carotenoid cleavage dioxygenase 7, chloroplast precursor, putative, expressed
Chr.4	27787129	27787135	GAGTCGC	GAGTCGC	G	INDEL	OS04G0555300	Exonic	5UTR	NA	LOC_Os04g46880	transporter, major facilitator family, putative, expressed
Chr.4	31220527	31220528	GA	GA	G	INDEL	OS04G0615200	Intronic	NA	NA	LOC_Os04g52504	adhesive/proline-rich protein, putative, expressed
Chr.4	31239384	31239387	GTGC	GTGC	G	INDEL	OS04G0615700	Exonic	CDS	InFrame	LOC_Os04g52540	retrotransposon protein, putative, unclassified, expressed
Chr.4	31439729	31439730	GT	GT	G	INDEL	OS04G0619000	Exonic	3UTR	NA	LOC_Os04g52810	no apical meristem protein, putative, expressed
Chr.3	1238585	1238585	T	T	G	SNP	OS03G0122300	Intronic	NA	NA	LOC_Os03g03034	flavonol synthase/flavanone 3-hydroxylase, putative, expressed
Chr.3	3564960	3564960	T	T	G	SNP	OS03G0166000	Intronic	NA	NA	LOC_Os03g06980	nucleic acid binding protein, putative, expressed
Chr.3	12589242	12589242	T	T	G	SNP	OS03G0338400	Intronic	NA	NA	LOC_Os03g21970	basic helix-loop-helix domain containing protein, expressed
Chr.3	16289689	16289689	T	T	G	SNP	OS03G0401100	Intronic	NA	NA	LOC_Os03g28300	protein kinase domain containing protein, expressed
Chr.3	23486480	23486480	T	T	G	SNP	OS03G0619400	Intronic	NA	NA	LOC_Os03g42220	T-complex protein, putative, expressed
Chr.3	24705337	24705337	T	T	G	SNP	OS03G0641300	Exonic	3UTR	NA	LOC_Os03g43990	BTBN8 - Bric-a-Brac, Tramtrack, Broad Complex BTB domain with non-phototropic hypocotyl 3 NPH3 and coiled-coil domains, ex
Chr.3	24814852	24814852	T	T	G	SNP	OS03G0643300	Intronic	NA	NA	LOC_Os03g44150	aminotransferase, putative, expressed
Chr.3	24815622	24815622	T	T	G	SNP	OS03G0643300	Intronic	NA	NA	LOC_Os03g44150	aminotransferase, putative, expressed
Chr.3	24815650	24815650	T	T	G	SNP	OS03G0643300	Intronic	NA	NA	LOC_Os03g44150	aminotransferase, putative, expressed
Chr.3	24815922	24815922	T	T	G	SNP	OS03G0643300	Intronic	NA	NA	LOC_Os03g44150	aminotransferase, putative, expressed
Chr.3	24817346	24817346	T	T	G	SNP	OS03G0643300	Intronic	NA	NA	LOC_Os03g44150	aminotransferase, putative, expressed
Chr.3	25946634	25946634	T	T	G	SNP	OS03G0661300	Exonic	3UTR	NA	LOC_Os03g45920	tubulin/FtsZ domain containing protein, putative, expressed
Chr.3	26380711	26380711	T	T	G	SNP	OS03G0669000	Exonic	3UTR	NA	LOC_Os03g46610	DEAD-box ATP-dependent RNA helicase, putative, expressed
Chr.3	26953933	26953933	T	T	G	SNP	OS03G0679700	Intronic	NA	NA	LOC_Os03g47610	thiamine biosynthesis protein thiC, putative, expressed
Chr.3	30316732	30316732	T	T	G	SNP	OS03G0738600	Intronic	NA	NA	LOC_Os03g52860	lipoxygenase, putative, expressed
Chr.3	33502316	33502316	T	T	G	SNP	OS03G0802900	Intronic	NA	NA	LOC_Os03g58830	BEE 1, putative, expressed
Chr.3	541749	541749	A	A	G	SNP	OS03G0109300	Exonic	CDS	Silent	LOC_Os03g01880	possible lysine decarboxylase domain containing protein, expressed
Chr.3	538700	538700	A	A	G	SNP	OS03G0109300	Intronic	NA	NA	LOC_Os03g01880	possible lysine decarboxylase domain containing protein, expressed
Chr.3	538810	538810	A	A	G	SNP	OS03G0109300	Intronic	NA	NA	LOC_Os03g01880	possible lysine decarboxylase domain containing protein, expressed
Chr.3	540546	540546	A	A	G	SNP	OS03G0109300	Intronic	NA	NA	LOC_Os03g01880	possible lysine decarboxylase domain containing protein, expressed
Chr.3	906841	906841	T	T	G	SNP	OS03G0116000	Intronic	NA	NA	LOC_Os03g02480	inner membrane protein, putative, expressed
Chr.3	1613322	1613322	A	A	G	SNP	OS03G0128500	Exonic	CDS	Silent	LOC_Os03g03650	POLD2 - Putative DNA polymerase delta complex subunit, expressed
Chr.3	1614489	1614489	A	A	G	SNP	OS03G0128500	Exonic	CDS	Silent	LOC_Os03g03650	POLD2 - Putative DNA polymerase delta complex subunit, expressed
Chr.3	1613380	1613380	A	A	G	SNP	OS03G0128500	Exonic	CDS	Missense	LOC_Os03g03650	POLD2 - Putative DNA polymerase delta complex subunit, expressed
Chr.3	1615612	1615612	A	A	G	SNP	OS03G0128500	Intronic	NA	NA	LOC_Os03g03650	POLD2 - Putative DNA polymerase delta complex subunit, expressed
Chr.3	1615781	1615781	T	T	G	SNP	OS03G0128500	Intronic	NA	NA	LOC_Os03g03650	POLD2 - Putative DNA polymerase delta complex subunit, expressed
Chr.3	1627462	1627462	A	A	G	SNP	OS03G0128700	Intronic	NA	NA	LOC_Os03g03660	CAMK_CAMK_like.17 - CAMK includes calcium/calmodulin depedent protein kinases, expressed
Chr.3	1627700	1627700	A	A	G	SNP	OS03G0128700	Intronic	NA	NA	LOC_Os03g03660	CAMK_CAMK_like.17 - CAMK includes calcium/calmodulin depedent protein kinases, expressed
Chr.3	1646771	1646771	A	A	G	SNP	OS03G0129100	Intronic	NA	NA	LOC_Os03g03700	MLO domain containing protein, putative, expressed
Chr.3	1719458	1719458	T	T	G	SNP	OS03G0130400	Exonic	3UTR	NA	LOC_Os03g03820	adenylate kinase, putative, expressed
Chr.3	1717896	1717896	A	A	G	SNP	OS03G0130400	Intronic	NA	NA	LOC_Os03g03820	adenylate kinase, putative, expressed
Chr.3	1719029	1719029	A	A	G	SNP	OS03G0130400	Intronic	NA	NA	LOC_Os03g03820	adenylate kinase, putative, expressed
Chr.3	1719057	1719057	T	T	G	SNP	OS03G0130400	Intronic	NA	NA	LOC_Os03g03820	adenylate kinase, putative, expressed
Chr.3	1719205	1719205	A	A	G	SNP	OS03G0130400	Intronic	NA	NA	LOC_Os03g03820	adenylate kinase, putative, expressed
Chr.3	1740159	1740159	A	A	G	SNP	OS03G0130700	Exonic	CDS	NA	LOC_Os03g03860	expressed protein
Chr.3	1740784	1740784	A	A	G	SNP	OS03G0130700	Intronic	NA	NA	LOC_Os03g03860	expressed protein
Chr.3	1741046	1741046	T	T	G	SNP	OS03G0130700	Intronic	NA	NA	LOC_Os03g03860	expressed protein
Chr.3	1788946	1788946	T	T	G	SNP	OS03G0131200	Exonic	CDS	Silent	LOC_Os03g03910	catalase domain containing protein, expressed
Chr.3	1789444	1789444	A	A	G	SNP	OS03G0131200	Exonic	CDS	Silent	LOC_Os03g03910	catalase domain containing protein, expressed
Chr.3	3372035	3372035	A	A	G	SNP	OS03G0162200	Exonic	CDS	Silent	LOC_Os03g06670	Core histone H2A/H2B/H3/H4 domain containing protein, putative, expressed
Chr.3	4546540	4546540	T	T	G	SNP	OS03G0187000	Intronic	NA	NA	LOC_Os03g08810	expressed protein
Chr.3	7849199	7849199	A	A	G	SNP	OS03G0248600	Exonic	CDS	Silent	LOC_Os03g14450	enolase, putative, expressed
Chr.3	10395790	10395790	T	T	G	SNP	OS03G0297100	Exonic	3UTR	NA	LOC_Os03g18570	40S ribosomal protein S7, putative, expressed
Chr.3	10394843	10394843	A	A	G	SNP	OS03G0297100	Intronic	NA	NA	LOC_Os03g18570	40S ribosomal protein S7, putative, expressed
Chr.3	10774904	10774904	C	C	G	SNP	OS03G0304500	Intronic	NA	NA	LOC_Os03g19200	DNAJ heat shock N-terminal domain-containing protein, putative, expressed
Chr.3	10849920	10849920	A	A	G	SNP	OS03G0305600	Exonic	3UTR	NA	LOC_Os03g19290	mitochondrial import inner membrane translocase subunit Tim17, putative, expressed
Chr.3	29115895	29115895	T	T	G	SNP	OS03G0719000	Exonic	CDS	NA	LOC_Os03g50970	retrotransposon protein, putative, Ty1-copia subclass, expressed
Chr.3	29116615	29116615	A	A	G	SNP	OS03G0719000	Exonic	CDS	NA	LOC_Os03g50970	retrotransposon protein, putative, Ty1-copia subclass, expressed
Chr.3	29137881	29137881	A	A	G	SNP	OS03G0719300	Exonic	3UTR	NA	LOC_Os03g51000	3,4-dihydroxy-2-butanone kinase, putative, expressed
Chr.3	29138349	29138349	A	A	G	SNP	OS03G0719300	Intronic	NA	NA	LOC_Os03g51000	3,4-dihydroxy-2-butanone kinase, putative, expressed
Chr.3	3372045	3372047	GAA	GAA	G	INDEL	OS03G0162200	Exonic	3UTR	NA	LOC_Os03g06670	Core histone H2A/H2B/H3/H4 domain containing protein, putative, expressed
Chr.3	538081	538082	GC	GC	G	INDEL	OS03G0109300	Intronic	NA	NA	LOC_Os03g01880	possible lysine decarboxylase domain containing protein, expressed
Chr.3	4545331	4545331	C	C	G	SNP	OS03G0187000	Exonic	5UTR	NA	LOC_Os03g08810	expressed protein
Chr.3	541166	541166	C	C	G	SNP	OS03G0109300	Intronic	NA	NA	LOC_Os03g01880	possible lysine decarboxylase domain containing protein, expressed
Chr.3	1244228	1244228	C	C	G	SNP	OS03G0122300	Intronic	NA	NA	LOC_Os03g03034	flavonol synthase/flavanone 3-hydroxylase, putative, expressed
Chr.3	3527568	3527568	C	C	G	SNP	OS03G0165400	Intronic	NA	NA	LOC_Os03g06940	beta-galactosidase, putative, expressed

Chr.3	3530347	3530347	C	C	G	SNP	OS03G0165400	Intronic	NA	NA	LOC_Os03g06940	beta-galactosidase, putative, expressed
Chr.3	3564448	3564448	C	C	G	SNP	OS03G0166000	Intronic	NA	NA	LOC_Os03g06980	nucleic acid binding protein, putative, expressed
Chr.3	15669691	15669691	C	C	G	SNP	OS03G0391100	Intronic	NA	NA	LOC_Os03g27360	RING-H2 finger protein ATL5H precursor, putative, expressed
Chr.3	15669727	15669727	C	C	G	SNP	OS03G0391100	Intronic	NA	NA	LOC_Os03g27360	RING-H2 finger protein ATL5H precursor, putative, expressed
Chr.3	22616145	22616145	C	C	G	SNP	OS03G0603600	Intronic	NA	NA	LOC_Os03g40670	glycerophosphoryl diester phosphodiesterase family protein, putative, expressed
Chr.3	22949143	22949143	C	C	G	SNP	OS03G0609000	Exonic	5UTR	NA	LOC_Os03g41280	expressed protein
Chr.3	24470184	24470184	C	C	G	SNP	OS03G0638400	Exonic	5UTR	NA	LOC_Os03g43750	retrotransposon, putative, centromere-specific, expressed
Chr.3	24814240	24814240	C	C	G	SNP	OS03G0643300	Intronic	NA	NA	LOC_Os03g44150	aminotransferase, putative, expressed
Chr.3	24817168	24817168	C	C	G	SNP	OS03G0643300	Intronic	NA	NA	LOC_Os03g44150	aminotransferase, putative, expressed
Chr.3	24817428	24817428	C	C	G	SNP	OS03G0643300	Intronic	NA	NA	LOC_Os03g44150	aminotransferase, putative, expressed
Chr.3	24817431	24817431	C	C	G	SNP	OS03G0643300	Intronic	NA	NA	LOC_Os03g44150	aminotransferase, putative, expressed
Chr.3	25001885	25001885	C	C	G	SNP	OS03G0646200	Exonic	3UTR	NA	LOC_Os03g44430	ubiquitin carboxyl-terminal hydrolase, family 1, putative, expressed
Chr.3	28249094	28249094	C	C	G	SNP	OS03G0703100	Exonic	CDS	Missense	LOC_Os03g49610	Os3bglu8 - beta-glucosidase, exo-beta-glucansase, high similarity to Os3bglu7, expressed
Chr.3	32934698	32934698	C	C	G	SNP	OS03G0791800	Intronic	NA	NA	LOC_Os03g57790	ubiquitin-conjugating enzyme, putative, expressed
Chr.3	35659975	35659975	C	C	G	SNP	OS03G0848100	Exonic	3UTR	NA	LOC_Os03g63074	Ser/Thr protein phosphatase family protein, expressed
Chr.3	1238596	1238596	A	A	G	SNP	OS03G0122300	Intronic	NA	NA	LOC_Os03g03034	flavonol synthase/flavanone 3-hydroxylase, putative, expressed
Chr.3	1243242	1243242	A	A	G	SNP	OS03G0122300	Intronic	NA	NA	LOC_Os03g03034	flavonol synthase/flavanone 3-hydroxylase, putative, expressed
Chr.3	1243365	1243365	A	A	G	SNP	OS03G0122300	Intronic	NA	NA	LOC_Os03g03034	flavonol synthase/flavanone 3-hydroxylase, putative, expressed
Chr.3	1243987	1243987	A	A	G	SNP	OS03G0122300	Intronic	NA	NA	LOC_Os03g03034	flavonol synthase/flavanone 3-hydroxylase, putative, expressed
Chr.3	1244026	1244026	A	A	G	SNP	OS03G0122300	Intronic	NA	NA	LOC_Os03g03034	flavonol synthase/flavanone 3-hydroxylase, putative, expressed
Chr.3	1244061	1244061	A	A	G	SNP	OS03G0122300	Intronic	NA	NA	LOC_Os03g03034	flavonol synthase/flavanone 3-hydroxylase, putative, expressed
Chr.3	1876262	1876262	A	A	G	SNP	OS03G0133300	Intronic	NA	NA	LOC_Os03g04100	expressed protein
Chr.3	2749501	2749501	A	A	G	SNP	OS03G0149300	Intronic	NA	NA	LOC_Os03g05530	nodulin, putative, expressed
Chr.3	3107598	3107598	A	A	G	SNP	OS03G0157800	Intronic	NA	NA	LOC_Os03g06190	3-5 exonuclease family protein, putative, expressed
Chr.3	3528781	3528781	A	A	G	SNP	OS03G0165400	Exonic	CDS	Silent	LOC_Os03g06940	beta-galactosidase, putative, expressed
Chr.3	3683721	3683721	A	A	G	SNP	OS03G0168300	Intronic	NA	NA	LOC_Os03g07200	expressed protein
Chr.3	6370848	6370848	A	A	G	SNP	OS03G0221500	Intronic	NA	NA	LOC_Os03g12140	glucan endo-1,3-beta-glucosidase precursor, putative, expressed
Chr.3	8198521	8198521	A	A	G	SNP	OS03G0255100	Intronic	NA	NA	LOC_Os03g15020	beta-galactosidase precursor, putative, expressed
Chr.3	8833818	8833818	A	A	G	SNP	OS03G0266900	Exonic	3UTR	NA	LOC_Os03g16020	hsp20/alpha crystallin family protein, putative, expressed
Chr.3	10040157	10040157	A	A	G	SNP	OS03G0289800	Intronic	NA	NA	LOC_Os03g18030	leucoanthocyanidin dioxygenase, putative, expressed
Chr.3	12701626	12701626	A	A	G	SNP	OS03G0340900	Intronic	NA	NA	LOC_Os03g22160	relA-SpoT like protein RSH1, putative, expressed
Chr.3	12701828	12701828	A	A	G	SNP	OS03G0340900	Intronic	NA	NA	LOC_Os03g22160	relA-SpoT like protein RSH1, putative, expressed
Chr.3	15469857	15469857	A	A	G	SNP	OS03G0387900	Intronic	NA	NA	LOC_Os03g27019	expressed protein
Chr.3	15669666	15669666	A	A	G	SNP	OS03G0391100	Intronic	NA	NA	LOC_Os03g27360	RING-H2 finger protein ATL5H precursor, putative, expressed
Chr.3	15669700	15669700	A	A	G	SNP	OS03G0391100	Intronic	NA	NA	LOC_Os03g27360	RING-H2 finger protein ATL5H precursor, putative, expressed
Chr.3	16284368	16284368	A	A	G	SNP	OS03G0401100	Exonic	CDS	Missense	LOC_Os03g28300	protein kinase domain containing protein, expressed
Chr.3	16285559	16285559	A	A	G	SNP	OS03G0401100	Intronic	NA	NA	LOC_Os03g28300	protein kinase domain containing protein, expressed
Chr.3	16289305	16289305	A	A	G	SNP	OS03G0401100	Intronic	NA	NA	LOC_Os03g28300	protein kinase domain containing protein, expressed
Chr.3	16301453	16301453	A	A	G	SNP	OS03G0401300	Exonic	3UTR	NA	LOC_Os03g28330	sucrose synthase, putative, expressed
Chr.3	16301618	16301618	A	A	G	SNP	OS03G0401300	Exonic	3UTR	NA	LOC_Os03g28330	sucrose synthase, putative, expressed
Chr.3	21637218	21637218	A	A	G	SNP	OS03G0586500	Exonic	3UTR	NA	LOC_Os03g38950	chloroplast post-illumination chlorophyll fluorescence increase protein, putative, expressed
Chr.3	24449391	24449391	A	A	G	SNP	OS03G0638200	Exonic	5UTR	NA	LOC_Os03g43720	transporter family protein, putative, expressed
Chr.3	24451582	24451582	A	A	G	SNP	OS03G0638200	Intronic	NA	NA	LOC_Os03g43720	transporter family protein, putative, expressed
Chr.3	24705301	24705301	A	A	G	SNP	OS03G0641300	Exonic	3UTR	NA	LOC_Os03g43990	BTBN8 - Bric-a-Brac, Tramtrack, Broad Complex BTB domain with non-phototropic hypocotyl 3 NPH3 and coiled-coil domains, ex
Chr.3	24705322	24705322	A	A	G	SNP	OS03G0641300	Exonic	3UTR	NA	LOC_Os03g43990	BTBN8 - Bric-a-Brac, Tramtrack, Broad Complex BTB domain with non-phototropic hypocotyl 3 NPH3 and coiled-coil domains, ex
Chr.3	24705549	24705549	A	A	G	SNP	OS03G0641300	Exonic	3UTR	NA	LOC_Os03g43990	BTBN8 - Bric-a-Brac, Tramtrack, Broad Complex BTB domain with non-phototropic hypocotyl 3 NPH3 and coiled-coil domains, ex
Chr.3	24815262	24815262	A	A	G	SNP	OS03G0643300	Exonic	CDS	Silent	LOC_Os03g44150	aminotransferase, putative, expressed
Chr.3	24814216	24814216	A	A	G	SNP	OS03G0643300	Intronic	NA	NA	LOC_Os03g44150	aminotransferase, putative, expressed
Chr.3	24814518	24814518	A	A	G	SNP	OS03G0643300	Intronic	NA	NA	LOC_Os03g44150	aminotransferase, putative, expressed
Chr.3	24814526	24814526	A	A	G	SNP	OS03G0643300	Intronic	NA	NA	LOC_Os03g44150	aminotransferase, putative, expressed
Chr.3	24815087	24815087	A	A	G	SNP	OS03G0643300	Intronic	NA	NA	LOC_Os03g44150	aminotransferase, putative, expressed
Chr.3	24815519	24815519	A	A	G	SNP	OS03G0643300	Intronic	NA	NA	LOC_Os03g44150	aminotransferase, putative, expressed
Chr.3	24815592	24815592	A	A	G	SNP	OS03G0643300	Intronic	NA	NA	LOC_Os03g44150	aminotransferase, putative, expressed
Chr.3	24815627	24815627	A	A	G	SNP	OS03G0643300	Intronic	NA	NA	LOC_Os03g44150	aminotransferase, putative, expressed
Chr.3	24815739	24815739	A	A	G	SNP	OS03G0643300	Intronic	NA	NA	LOC_Os03g44150	aminotransferase, putative, expressed
Chr.3	24816449	24816449	A	A	G	SNP	OS03G0643300	Intronic	NA	NA	LOC_Os03g44150	aminotransferase, putative, expressed
Chr.3	24816822	24816822	A	A	G	SNP	OS03G0643300	Intronic	NA	NA	LOC_Os03g44150	aminotransferase, putative, expressed
Chr.3	24817268	24817268	A	A	G	SNP	OS03G0643300	Intronic	NA	NA	LOC_Os03g44150	aminotransferase, putative, expressed
Chr.3	24817441	24817441	A	A	G	SNP	OS03G0643300	Intronic	NA	NA	LOC_Os03g44150	aminotransferase, putative, expressed
Chr.3	25063244	25063244	A	A	G	SNP	OS03G0647500	Exonic	3UTR	NA	LOC_Os03g44530	GTPase of unknown function domain containing protein, putative, expressed
Chr.3	25946462	25946462	A	A	G	SNP	OS03G0661300	Exonic	3UTR	NA	LOC_Os03g45920	tubulin/FtsZ domain containing protein, putative, expressed
Chr.3	26953843	26953843	A	A	G	SNP	OS03G0679700	Intronic	NA	NA	LOC_Os03g47610	thiamine biosynthesis protein thiC, putative, expressed
Chr.3	27460554	27460554	A	A	G	SNP	OS03G0688200	Intronic	NA	NA	LOC_Os03g48260	expressed protein
Chr.3	27461480	27461480	A	A	G	SNP	OS03G0688200	Intronic	NA	NA	LOC_Os03g48260	expressed protein
Chr.3	27488708	27488708	A	A	G	SNP	OS03G0689100	Intronic	NA	NA	LOC_Os03g48300	histidine acid phosphatase, putative, expressed
Chr.3	27489970	27489970	A	A	G	SNP	OS03G0689100	Intronic	NA	NA	LOC_Os03g48300	histidine acid phosphatase, putative, expressed
Chr.3	28050754	28050754	A	A	G	SNP	OS03G0699700	Intronic	NA	NA	LOC_Os03g49260	lipoxygenase, putative, expressed
Chr.3	28336334	28336334	A	A	G	SNP	OS03G0704700	Intronic	NA	NA	LOC_Os03g49770	oxysterol-binding protein, putative, expressed
Chr.3	29138272	29138272	A	A	G	SNP	OS03G0719300	Exonic	CDS	Silent	LOC_Os03g51000	3,4-dihydroxy-2-butanone kinase, putative, expressed
Chr.3	29386981	29386981	A	A	G	SNP	OS03G0723400	Intronic	NA	NA	LOC_Os03g51350	expressed protein
Chr.3	29386995	29386995	A	A	G	SNP	OS03G0723400	Intronic	NA	NA	LOC_Os03g51350	expressed protein
Chr.3	30225560	30225560	A	A	G	SNP	OS03G0737300	Intronic	NA	NA	LOC_Os03g52720	magnesium-dependent phosphatase 1, putative, expressed
Chr.3	30518065	30518065	A	A	G	SNP	OS03G0743500	Exonic	5UTR	NA	LOC_Os03g53200	OsCML4 - Calmodulin-related calcium sensor protein, expressed
Chr.3	32709092	32709092	A	A	G	SNP	OS03G0787300	Intronic	NA	NA	LOC_Os03g57340	chaperone protein dnaJ, putative, expressed
Chr.3	32932926	32932926	A	A	G	SNP	OS03G0791800	Intronic	NA	NA	LOC_Os03g57790	ubiquitin-conjugating enzyme, putative, expressed
Chr.3	34192781	34192781	A	A	G	SNP	OS03G0815800	Exonic	CDS	Silent	LOC_Os03g60120	AP2 domain containing protein, expressed
Chr.3	34192759	34192759	A	A	G	SNP	OS03G0815800	Exonic	CDS	Missense	LOC_Os03g60120	AP2 domain containing protein, expressed
Chr.3	34911358	34911358	A	A	G	SNP	OS03G0831100	Intronic	NA	NA	LOC_Os03g61560	expressed protein
Chr.3	35663423	35663423	A	A	G	SNP	OS03G0848100	Exonic	3UTR	NA	LOC_Os03g63074	Ser/Thr protein phosphatase family protein, expressed
Chr.3	540973	540973	A	A	G	SNP	OS03G0109300	Intronic	NA	NA	LOC_Os03g01880	possible lysine decarboxylase domain containing protein, expressed
Chr.3	906138	906138	A	A	G	SNP	OS03G0116000	Exonic	5UTR	NA	LOC_Os03g02480	inner membrane protein, putative, expressed
Chr.3	1645427	1645427	A	A	G	SNP	OS03G0129100	Exonic	CDS	Missense	LOC_Os03g03700	MLO domain containing protein, putative, expressed
Chr.3	1716529	1716529	A	A	G	SNP	OS03G0130400	Exonic	5UTR	NA	LOC_Os03g03820	adenylate kinase, putative, expressed
Chr.3	1737981	1737981	C	C	G	SNP	OS03G0130700	Exonic	CDS	NA	LOC_Os03g03860	expressed protein
Chr.3	1790664	1790664	A	A	G	SNP	OS03G0131200	Exonic	5UTR	NA	LOC_Os03g03910	catalase domain containing protein, expressed

Chr.3	1790665	1790665	T	T	G	SNP	OS03G0131200	Exonic	5UTR	NA	LOC_Os03g03910	catalase domain containing protein, expressed
Chr.3	1789894	1789894	A	A	G	SNP	OS03G0131200	Exonic	CDS	Silent	LOC_Os03g03910	catalase domain containing protein, expressed
Chr.3	1788670	1788670	A	A	G	SNP	OS03G0131200	Intronic	NA	NA	LOC_Os03g03910	catalase domain containing protein, expressed
Chr.3	2751844	2751844	C	C	G	SNP	OS03G0149300	Intronic	NA	NA	LOC_Os03g05530	nodulin, putative, expressed
Chr.3	3564870	3564870	A	A	G	SNP	OS03G0166000	Intronic	NA	NA	LOC_Os03g06980	nucleic acid binding protein, putative, expressed
Chr.3	3683317	3683317	A	A	G	SNP	OS03G0168300	Exonic	3UTR	NA	LOC_Os03g07200	expressed protein
Chr.3	8200916	8200916	T	T	G	SNP	OS03G0255100	Intronic	NA	NA	LOC_Os03g15020	beta-galactosidase precursor, putative, expressed
Chr.3	8200968	8200968	A	A	G	SNP	OS03G0255100	Intronic	NA	NA	LOC_Os03g15020	beta-galactosidase precursor, putative, expressed
Chr.3	8200973	8200973	A	A	G	SNP	OS03G0255100	Intronic	NA	NA	LOC_Os03g15020	beta-galactosidase precursor, putative, expressed
Chr.3	8834328	8834328	A	A	G	SNP	OS03G0266900	Exonic	CDS	Missense	LOC_Os03g16020	hsp20/alpha crystallin family protein, putative, expressed
Chr.3	11959499	11959499	T	T	G	SNP	OS03G0327600	Exonic	5UTR	NA	LOC_Os03g21040	stress responsive protein, putative, expressed
Chr.3	13190126	13190126	C	C	G	SNP	OS03G0351700	Exonic	3UTR	NA	LOC_Os03g22820	expressed protein
Chr.3	13190130	13190130	A	A	G	SNP	OS03G0351700	Exonic	3UTR	NA	LOC_Os03g22820	expressed protein
Chr.3	15668335	15668335	A	A	G	SNP	OS03G0391100	Exonic	CDS	Silent	LOC_Os03g27360	RING-H2 finger protein ATL5H precursor, putative, expressed
Chr.3	15668842	15668842	C	C	G	SNP	OS03G0391100	Exonic	CDS	Silent	LOC_Os03g27360	RING-H2 finger protein ATL5H precursor, putative, expressed
Chr.3	15668914	15668914	A	A	G	SNP	OS03G0391100	Exonic	CDS	Silent	LOC_Os03g27360	RING-H2 finger protein ATL5H precursor, putative, expressed
Chr.3	15668911	15668911	C	C	G	SNP	OS03G0391100	Exonic	CDS	Missense	LOC_Os03g27360	RING-H2 finger protein ATL5H precursor, putative, expressed
Chr.3	15668969	15668969	T	T	G	SNP	OS03G0391100	Exonic	CDS	Missense	LOC_Os03g27360	RING-H2 finger protein ATL5H precursor, putative, expressed
Chr.3	15669436	15669436	A	A	G	SNP	OS03G0391100	Intronic	NA	NA	LOC_Os03g27360	RING-H2 finger protein ATL5H precursor, putative, expressed
Chr.3	16593523	16593523	A	A	G	SNP	OS03G0405500	Exonic	CDS	Missense	LOC_Os03g29190	PDI, putative, expressed
Chr.3	21416547	21416547	A	A	G	SNP	OS03G0582200	Exonic	CDS	Silent	LOC_Os03g38590	secretory carrier-associated membrane protein 4, putative, expressed
Chr.3	21635600	21635600	C	C	G	SNP	OS03G0586500	Intronic	NA	NA	LOC_Os03g38950	chloroplast post-illumination chlorophyll fluorescence increase protein, putative, expressed
Chr.3	24705238	24705238	A	A	G	SNP	OS03G0641300	Exonic	3UTR	NA	LOC_Os03g43990	BTBN8 - Bric-a-Brac, Tramtrack, Broad Complex BTB domain with non-phototropic hypocotyl 3 NPH3 and coiled-coil domains, ex
Chr.3	24810096	24810096	A	A	G	SNP	OS03G0643300	Exonic	3UTR	NA	LOC_Os03g44150	aminotransferase, putative, expressed
Chr.3	24814791	24814791	A	A	G	SNP	OS03G0643300	Intronic	NA	NA	LOC_Os03g44150	aminotransferase, putative, expressed
Chr.3	24815035	24815035	T	T	G	SNP	OS03G0643300	Intronic	NA	NA	LOC_Os03g44150	aminotransferase, putative, expressed
Chr.3	25946378	25946378	C	C	G	SNP	OS03G0661300	Exonic	CDS	Missense	LOC_Os03g45920	tubulin/FtsZ domain containing protein, putative, expressed
Chr.3	26033341	26033341	C	C	G	SNP	OS03G0663400	Exonic	CDS	Missense	LOC_Os03g46060	thaumatin family domain containing protein, expressed
Chr.3	26396906	26396906	A	A	G	SNP	OS03G0669100	Intronic	NA	NA	LOC_Os03g46640	deoxyuridine 5-triphosphate nucleotidohydrolase, putative, expressed
Chr.3	27490141	27490141	A	A	G	SNP	OS03G0689100	Intronic	NA	NA	LOC_Os03g48300	histidine acid phosphatase, putative, expressed
Chr.3	27494046	27494046	A	A	G	SNP	OS03G0689100	Intronic	NA	NA	LOC_Os03g48300	histidine acid phosphatase, putative, expressed
Chr.3	27497322	27497322	A	A	G	SNP	OS03G0689100	Intronic	NA	NA	LOC_Os03g48300	histidine acid phosphatase, putative, expressed
Chr.3	29018493	29018493	A	A	G	SNP	OS03G0717000	Exonic	CDS	NA	LOC_Os03g50810	receptor protein kinase TMK1 precursor, putative, expressed
Chr.3	29114908	29114908	A	A	G	SNP	OS03G0719000	Exonic	CDS	NA	LOC_Os03g50970	retrotransposon protein, putative, Ty1-copia subclass, expressed
Chr.3	30063913	30063913	A	A	G	SNP	OS03G0733800	Intronic	NA	NA	LOC_Os03g52340	endoplasmic oxidoreductin-1 precursor, putative, expressed
Chr.3	32008815	32008815	C	C	G	SNP	OS03G0772600	Exonic	CDS	NA	LOC_Os03g56160	lectin-like receptor kinase 7, putative, expressed
Chr.3	32933813	32933813	A	A	G	SNP	OS03G0791800	Intronic	NA	NA	LOC_Os03g57790	ubiquitin-conjugating enzyme, putative, expressed
Chr.3	32933909	32933909	T	T	G	SNP	OS03G0791800	Intronic	NA	NA	LOC_Os03g57790	ubiquitin-conjugating enzyme, putative, expressed
Chr.3	32933976	32933976	A	A	G	SNP	OS03G0791800	Intronic	NA	NA	LOC_Os03g57790	ubiquitin-conjugating enzyme, putative, expressed
Chr.3	35161652	35161652	A	A	G	SNP	OS03G0836800	Intronic	NA	NA	LOC_Os03g62060	hydrolase, putative, expressed
Chr.3	35161688	35161688	A	A	G	SNP	OS03G0836800	Intronic	NA	NA	LOC_Os03g62060	hydrolase, putative, expressed
Chr.3	537699	537723	GGCAGCAGCAGCAGCAGCAGCAGGA	GGCAGCAGCAGCAGCAGCAGCAGGA	G	INDEL	OS03G0109300	Exonic	CDS	InFrame	LOC_Os03g01880	possible lysine decarboxylase domain containing protein, expressed
Chr.3	1236858	1236859	GT	GT	G	INDEL	OS03G0122300	Intronic	NA	NA	LOC_Os03g03034	flavonol synthase/flavanone 3-hydroxylase, putative, expressed
Chr.3	1237836	1237843	GACATAAA	GACATAAA	G	INDEL	OS03G0122300	Intronic	NA	NA	LOC_Os03g03034	flavonol synthase/flavanone 3-hydroxylase, putative, expressed
Chr.3	3528439	3528444	GCTCAT	GCTCAT	G	INDEL	OS03G0165400	Intronic	NA	NA	LOC_Os03g06940	beta-galactosidase, putative, expressed
Chr.3	7566028	7566029	GC	GC	G	INDEL	OS03G0243100	Intronic	NA	NA	LOC_Os03g13950	actin-depolymerizing factor, putative, expressed
Chr.3	8201487	8201491	GAGAA	GAGAA	G	INDEL	OS03G0255100	Intronic	NA	NA	LOC_Os03g15020	beta-galactosidase precursor, putative, expressed
Chr.3	11730859	11730860	GA	GA	G	INDEL	OS03G0323600	Exonic	3UTR	NA	LOC_Os03g20730	chaperone protein dnaJ, putative, expressed
Chr.3	12349926	12349927	GT	GT	G	INDEL	OS03G0334100	Intronic	NA	NA	LOC_Os03g21630	PRC-barrel domain protein, putative, expressed
Chr.3	16283205	16283206	GC	GC	G	INDEL	OS03G0401100	Exonic	5UTR	NA	LOC_Os03g28300	protein kinase domain containing protein, expressed
Chr.3	17290669	17290670	GA	GA	G	INDEL	OS03G0416500	Exonic	5UTR	NA	LOC_Os03g30300	6-phosphogluconolactonase, putative, expressed
Chr.3	17291118	17291119	GT	GT	G	INDEL	OS03G0416500	Exonic	5UTR	NA	LOC_Os03g30300	6-phosphogluconolactonase, putative, expressed
Chr.3	17292121	17292122	GA	GA	G	INDEL	OS03G0416500	Exonic	5UTR	NA	LOC_Os03g30300	6-phosphogluconolactonase, putative, expressed
Chr.3	20794540	20794541	GA	GA	G	INDEL	OS03G0571900	Intronic	NA	NA	LOC_Os03g37490	MATE efflux family protein, putative, expressed
Chr.3	23371260	23371262	GAC	GAC	G	INDEL	OS03G0616700	Intronic	NA	NA	LOC_Os03g42050	exportin-7-A, putative, expressed
Chr.3	24416834	24416837	GAGA	GAGA	G	INDEL	OS03G0637800	Intronic	NA	NA	LOC_Os03g43670	TKL_IRAK_CR4L.1 - The CR4L subfamily has homology with Crinkly4, expressed
Chr.3	24816240	24816241	GA	GA	G	INDEL	OS03G0643300	Intronic	NA	NA	LOC_Os03g44150	aminotransferase, putative, expressed
Chr.3	24816495	24816496	GA	GA	G	INDEL	OS03G0643300	Intronic	NA	NA	LOC_Os03g44150	aminotransferase, putative, expressed
Chr.3	24817534	24817536	GAA	GAA	G	INDEL	OS03G0643300	Intronic	NA	NA	LOC_Os03g44150	aminotransferase, putative, expressed
Chr.3	25002258	25002261	GTCT	GTCT	G	INDEL	OS03G0646200	Exonic	CDS	InFrame	LOC_Os03g44430	ubiquitin carboxyl-terminal hydrolase, family 1, putative, expressed
Chr.3	28337209	28337212	GTTC	GTTC	G	INDEL	OS03G0704700	Intronic	NA	NA	LOC_Os03g49770	oxysterol-binding protein, putative, expressed
Chr.3	28337398	28337400	GAA	GAA	G	INDEL	OS03G0704700	Intronic	NA	NA	LOC_Os03g49770	oxysterol-binding protein, putative, expressed
Chr.3	29564699	29564700	GA	GA	G	INDEL	OS03G0726500	Intronic	NA	NA	LOC_Os03g51650	membrane protein, putative, expressed
Chr.3	32666219	32666224	GTTGGA	GTTGGA	G	INDEL	OS03G0786700	Exonic	5UTR	NA	LOC_Os03g57280	N-dimethylguanosine tRNA methyltransferase, putative, expressed
Chr.3	33907334	33907335	GT	GT	G	INDEL	OS03G0810100	Exonic	3UTR	NA	LOC_Os03g59570	IPP transferase, putative, expressed
Chr.2	81907	81907	T	T	G	SNP	OS02G0101500	Intronic	NA	NA	LOC_Os02g01150	erythronate-4-phosphate dehydrogenase domain containing protein, expressed
Chr.2	119521	119521	T	T	G	SNP	OS02G0102200	Intronic	NA	NA	LOC_Os02g01210	amino acid transporter, putative, expressed
Chr.2	317353	317353	T	T	G	SNP	OS02G0105900	Exonic	CDS	Silent	LOC_Os02g01560	40S ribosomal protein S4, putative, expressed
Chr.2	5551335	5551335	T	T	G	SNP	OS02G0198900	Intronic	NA	NA	LOC_Os02g10540	ribosomal protein L7/L12 C-terminal domain containing protein, expressed
Chr.2	5760303	5760303	T	T	G	SNP	OS02G0202950	Exonic	CDS	NA	LOC_Os02g10860	bZIP transcription factor domain containing protein, expressed
Chr.2	6835306	6835306	T	T	G	SNP	OS02G0222500	Intronic	NA	NA	LOC_Os02g12939	expressed protein
Chr.2	9927241	9927241	T	T	G	SNP	OS02G0272900	Intronic	NA	NA	LOC_Os02g17304	resistance protein, putative, expressed
Chr.2	9977756	9977756	T	T	G	SNP	OS02G0273700	Exonic	3UTR	NA	LOC_Os02g17350	VHS and GAT domain containing protein, expressed
Chr.2	9972906	9972906	T	T	G	SNP	OS02G0273700	Intronic	NA	NA	LOC_Os02g17350	VHS and GAT domain containing protein, expressed
Chr.2	9973257	9973257	T	T	G	SNP	OS02G0273700	Intronic	NA	NA	LOC_Os02g17350	VHS and GAT domain containing protein, expressed
Chr.2	9989586	9989586	T	T	G	SNP	OS02G0273900	Intronic	NA	NA	LOC_Os02g17370	expressed protein
Chr.2	34028507	34028507	T	T	G	SNP	OS02G0799000	Exonic	CDS	Missense	LOC_Os02g55560	protein phosphatase 2C, putative, expressed
Chr.2	35094024	35094024	T	T	G	SNP	OS02G0817900	Intronic	NA	NA	LOC_Os02g57290	cytochrome P450, putative, expressed
Chr.2	35096546	35096546	T	T	G	SNP	OS02G0817900	Intronic	NA	NA	LOC_Os02g57290	cytochrome P450, putative, expressed
Chr.2	4450989	4450989	T	T	G	SNP	OS02G0180000	Intronic	NA	NA	LOC_Os02g08364	protein phosphatase 2C, putative, expressed
Chr.2	3037751	3037751	C	C	G	SNP	OS02G0155400	Exonic	CDS	Silent	LOC_Os02g06090	phytosulfokine receptor precursor, putative, expressed
Chr.2	3039482	3039482	C	C	G	SNP	OS02G0155400	Exonic	CDS	Silent	LOC_Os02g06090	phytosulfokine receptor precursor, putative, expressed
Chr.2	8837413	8837413	C	C	G	SNP	OS02G0256100	Exonic	3UTR	NA	LOC_Os02g15690	polygalacturonase, putative, expressed
Chr.2	21933297	21933297	C	C	G	SNP	OS02G0572400	Exonic	CDS	Missense	LOC_Os02g36340	riboflavin biosynthesis protein ribAB, chloroplast precursor, putative, expressed
Chr.2	21934560	21934560	C	C	G	SNP	OS02G0572400	Exonic	CDS	Missense	LOC_Os02g36340	riboflavin biosynthesis protein ribAB, chloroplast precursor, putative, expressed

Chr.2	2747069	2747069	A	A	G	SNP	OS02G0149800	Exonic	3UTR	NA	LOC_Os02g05630	protein phosphatase 2C, putative, expressed
Chr.2	3037793	3037793	A	A	G	SNP	OS02G0155400	Exonic	CDS	Silent	LOC_Os02g06090	phytosulfokine receptor precursor, putative, expressed
Chr.2	3039431	3039431	A	A	G	SNP	OS02G0155400	Exonic	CDS	Silent	LOC_Os02g06090	phytosulfokine receptor precursor, putative, expressed
Chr.2	3039815	3039815	A	A	G	SNP	OS02G0155400	Exonic	CDS	Silent	LOC_Os02g06090	phytosulfokine receptor precursor, putative, expressed
Chr.2	3037675	3037675	A	A	G	SNP	OS02G0155400	Exonic	CDS	Missense	LOC_Os02g06090	phytosulfokine receptor precursor, putative, expressed
Chr.2	3037713	3037713	A	A	G	SNP	OS02G0155400	Exonic	CDS	Missense	LOC_Os02g06090	phytosulfokine receptor precursor, putative, expressed
Chr.2	3037929	3037929	A	A	G	SNP	OS02G0155400	Exonic	CDS	Missense	LOC_Os02g06090	phytosulfokine receptor precursor, putative, expressed
Chr.2	3038307	3038307	A	A	G	SNP	OS02G0155400	Exonic	CDS	Missense	LOC_Os02g06090	phytosulfokine receptor precursor, putative, expressed
Chr.2	3038667	3038667	A	A	G	SNP	OS02G0155400	Exonic	CDS	Missense	LOC_Os02g06090	phytosulfokine receptor precursor, putative, expressed
Chr.2	3039088	3039088	A	A	G	SNP	OS02G0155400	Exonic	CDS	Missense	LOC_Os02g06090	phytosulfokine receptor precursor, putative, expressed
Chr.2	3039231	3039231	A	A	G	SNP	OS02G0155400	Exonic	CDS	Missense	LOC_Os02g06090	phytosulfokine receptor precursor, putative, expressed
Chr.2	3039405	3039405	A	A	G	SNP	OS02G0155400	Exonic	CDS	Missense	LOC_Os02g06090	phytosulfokine receptor precursor, putative, expressed
Chr.2	3039568	3039568	A	A	G	SNP	OS02G0155400	Exonic	CDS	Missense	LOC_Os02g06090	phytosulfokine receptor precursor, putative, expressed
Chr.2	3039804	3039804	A	A	G	SNP	OS02G0155400	Exonic	CDS	Missense	LOC_Os02g06090	phytosulfokine receptor precursor, putative, expressed
Chr.2	4390396	4390396	A	A	G	SNP	OS02G0179200	Exonic	3UTR	NA	LOC_Os02g08270	class I glutamine amidotransferase, putative, expressed
Chr.2	8977441	8977441	A	A	G	SNP	OS02G0258900	Exonic	3UTR	NA	LOC_Os02g15870	molybdenum cofactor biosynthesis protein 1, putative, expressed
Chr.2	18575481	18575481	A	A	G	SNP	OS02G0515200	Exonic	3UTR	NA	LOC_Os02g31080	hydroxyproline-rich glycoprotein family protein, putative, expressed
Chr.2	21931456	21931456	A	A	G	SNP	OS02G0572400	Intronic	NA	NA	LOC_Os02g36340	riboflavin biosynthesis protein ribAB, chloroplast precursor, putative, expressed
Chr.2	22688523	22688523	A	A	G	SNP	OS02G0588500	Exonic	CDS	Missense	LOC_Os02g37590	glycerophosphoryl diester phosphodiesterase family protein, putative, expressed
Chr.2	21933970	21933976	GAGAATA	GAGAATA	G	INDEL	OS02G0572400	Intronic	NA	NA	LOC_Os02g36340	riboflavin biosynthesis protein ribAB, chloroplast precursor, putative, expressed
Chr.2	3829349	3829349	C	C	G	SNP	OS02G0170100	Intronic	NA	NA	LOC_Os02g07410	glycine cleavage system H protein, putative, expressed
Chr.2	4297616	4297616	C	C	G	SNP	OS02G0177800	Intronic	NA	NA	LOC_Os02g08120	calmodulin binding protein, putative, expressed
Chr.2	6812236	6812236	C	C	G	SNP	OS02G0222100	Intronic	NA	NA	LOC_Os02g12900	cysteine synthase, putative, expressed
Chr.2	9651260	9651260	C	C	G	SNP	OS02G0269600	Exonic	CDS	Missense	LOC_Os02g16940	OsSub13 - Putative Subtilisin homologue, expressed
Chr.2	9651715	9651715	C	C	G	SNP	OS02G0269600	Exonic	CDS	Missense	LOC_Os02g16940	OsSub13 - Putative Subtilisin homologue, expressed
Chr.2	9923657	9923657	C	C	G	SNP	OS02G0272900	Exonic	CDS	Missense	LOC_Os02g17304	resistance protein, putative, expressed
Chr.2	9925846	9925846	C	C	G	SNP	OS02G0272900	Intronic	NA	NA	LOC_Os02g17304	resistance protein, putative, expressed
Chr.2	9973812	9973812	C	C	G	SNP	OS02G0273700	Intronic	NA	NA	LOC_Os02g17350	VHS and GAT domain containing protein, expressed
Chr.2	9974446	9974446	C	C	G	SNP	OS02G0273700	Intronic	NA	NA	LOC_Os02g17350	VHS and GAT domain containing protein, expressed
Chr.2	9974478	9974478	C	C	G	SNP	OS02G0273700	Intronic	NA	NA	LOC_Os02g17350	VHS and GAT domain containing protein, expressed
Chr.2	9974513	9974513	C	C	G	SNP	OS02G0273700	Intronic	NA	NA	LOC_Os02g17350	VHS and GAT domain containing protein, expressed
Chr.2	9989917	9989917	C	C	G	SNP	OS02G0273900	Intronic	NA	NA	LOC_Os02g17370	expressed protein
Chr.2	9990051	9990051	C	C	G	SNP	OS02G0273900	Intronic	NA	NA	LOC_Os02g17370	expressed protein
Chr.2	28783860	28783860	C	C	G	SNP	OS02G0699700	Intronic	NA	NA	LOC_Os02g47150	DNA topoisomerase 2, putative, expressed
Chr.2	34835918	34835918	C	C	G	SNP	OS02G0813350	Exonic	3UTR	NA	LOC_Os02g56840	OsFBX70 - F-box domain containing protein, expressed
Chr.2	35098188	35098188	C	C	G	SNP	OS02G0817900	Intronic	NA	NA	LOC_Os02g57290	cytochrome P450, putative, expressed
Chr.2	35098193	35098193	C	C	G	SNP	OS02G0817900	Intronic	NA	NA	LOC_Os02g57290	cytochrome P450, putative, expressed
Chr.2	80255	80255	A	A	G	SNP	OS02G0101500	Exonic	CDS	Silent	LOC_Os02g01150	erythronate-4-phosphate dehydrogenase domain containing protein, expressed
Chr.2	81419	81419	A	A	G	SNP	OS02G0101500	Intronic	NA	NA	LOC_Os02g01150	erythronate-4-phosphate dehydrogenase domain containing protein, expressed
Chr.2	81643	81643	A	A	G	SNP	OS02G0101500	Intronic	NA	NA	LOC_Os02g01150	erythronate-4-phosphate dehydrogenase domain containing protein, expressed
Chr.2	81681	81681	A	A	G	SNP	OS02G0101500	Intronic	NA	NA	LOC_Os02g01150	erythronate-4-phosphate dehydrogenase domain containing protein, expressed
Chr.2	119615	119615	A	A	G	SNP	OS02G0102200	Intronic	NA	NA	LOC_Os02g01210	amino acid transporter, putative, expressed
Chr.2	713286	713286	A	A	G	SNP	OS02G0113200	Intronic	NA	NA	LOC_Os02g02230	cytochrome P450, putative, expressed
Chr.2	1660456	1660456	A	A	G	SNP	OS02G0131800	Intronic	NA	NA	LOC_Os02g03900	metal transporter Nramp6, putative, expressed
Chr.2	1825547	1825547	A	A	G	SNP	OS02G0134400	Exonic	CDS	Missense	LOC_Os02g04170	L-aspartate oxidase 1, putative, expressed
Chr.2	1823800	1823800	A	A	G	SNP	OS02G0134400	Intronic	NA	NA	LOC_Os02g04170	L-aspartate oxidase 1, putative, expressed
Chr.2	4293365	4293365	A	A	G	SNP	OS02G0177800	Exonic	3UTR	NA	LOC_Os02g08120	calmodulin binding protein, putative, expressed
Chr.2	4296359	4296359	A	A	G	SNP	OS02G0177800	Intronic	NA	NA	LOC_Os02g08120	calmodulin binding protein, putative, expressed
Chr.2	5158294	5158294	A	A	G	SNP	OS02G0192500	Intronic	NA	NA	LOC_Os02g09930	CSLA1 - cellulose synthase-like family A; mannan synthase, expressed
Chr.2	5250310	5250310	A	A	G	SNP	OS02G0194200	Exonic	5UTR	NA	LOC_Os02g10080	zinc finger C-x8-C-x5-C-x3-H type family protein, expressed
Chr.2	5551843	5551843	A	A	G	SNP	OS02G0198900	Intronic	NA	NA	LOC_Os02g10540	ribosomal protein L7/L12 C-terminal domain containing protein, expressed
Chr.2	6813624	6813624	A	A	G	SNP	OS02G0222100	Exonic	3UTR	NA	LOC_Os02g12900	cysteine synthase, putative, expressed
Chr.2	6833754	6833754	A	A	G	SNP	OS02G0222500	Exonic	3UTR	NA	LOC_Os02g12939	expressed protein
Chr.2	6834071	6834071	A	A	G	SNP	OS02G0222500	Exonic	3UTR	NA	LOC_Os02g12939	expressed protein
Chr.2	6834599	6834599	A	A	G	SNP	OS02G0222500	Exonic	CDS	NA	LOC_Os02g12939	expressed protein
Chr.2	6834814	6834814	A	A	G	SNP	OS02G0222500	Intronic	NA	NA	LOC_Os02g12939	expressed protein
Chr.2	6834927	6834927	A	A	G	SNP	OS02G0222500	Intronic	NA	NA	LOC_Os02g12939	expressed protein
Chr.2	6835367	6835367	A	A	G	SNP	OS02G0222500	Intronic	NA	NA	LOC_Os02g12939	expressed protein
Chr.2	9651411	9651411	A	A	G	SNP	OS02G0269600	Exonic	CDS	Silent	LOC_Os02g16940	OsSub13 - Putative Subtilisin homologue, expressed
Chr.2	9651417	9651417	A	A	G	SNP	OS02G0269600	Exonic	CDS	Silent	LOC_Os02g16940	OsSub13 - Putative Subtilisin homologue, expressed
Chr.2	9651534	9651534	A	A	G	SNP	OS02G0269600	Exonic	CDS	Silent	LOC_Os02g16940	OsSub13 - Putative Subtilisin homologue, expressed
Chr.2	9651711	9651711	A	A	G	SNP	OS02G0269600	Exonic	CDS	Silent	LOC_Os02g16940	OsSub13 - Putative Subtilisin homologue, expressed
Chr.2	9925344	9925344	A	A	G	SNP	OS02G0272900	Intronic	NA	NA	LOC_Os02g17304	resistance protein, putative, expressed
Chr.2	9925644	9925644	A	A	G	SNP	OS02G0272900	Intronic	NA	NA	LOC_Os02g17304	resistance protein, putative, expressed
Chr.2	9926034	9926034	A	A	G	SNP	OS02G0272900	Intronic	NA	NA	LOC_Os02g17304	resistance protein, putative, expressed
Chr.2	9926060	9926060	A	A	G	SNP	OS02G0272900	Intronic	NA	NA	LOC_Os02g17304	resistance protein, putative, expressed
Chr.2	9977809	9977809	A	A	G	SNP	OS02G0273700	Exonic	3UTR	NA	LOC_Os02g17350	VHS and GAT domain containing protein, expressed
Chr.2	9972735	9972735	A	A	G	SNP	OS02G0273700	Exonic	5UTR	NA	LOC_Os02g17350	VHS and GAT domain containing protein, expressed
Chr.2	9975962	9975962	A	A	G	SNP	OS02G0273700	Exonic	CDS	Silent	LOC_Os02g17350	VHS and GAT domain containing protein, expressed
Chr.2	9973582	9973582	A	A	G	SNP	OS02G0273700	Intronic	NA	NA	LOC_Os02g17350	VHS and GAT domain containing protein, expressed
Chr.2	9973783	9973783	A	A	G	SNP	OS02G0273700	Intronic	NA	NA	LOC_Os02g17350	VHS and GAT domain containing protein, expressed
Chr.2	9973978	9973978	A	A	G	SNP	OS02G0273700	Intronic	NA	NA	LOC_Os02g17350	VHS and GAT domain containing protein, expressed
Chr.2	9974258	9974258	A	A	G	SNP	OS02G0273700	Intronic	NA	NA	LOC_Os02g17350	VHS and GAT domain containing protein, expressed
Chr.2	9974316	9974316	A	A	G	SNP	OS02G0273700	Intronic	NA	NA	LOC_Os02g17350	VHS and GAT domain containing protein, expressed
Chr.2	9974428	9974428	A	A	G	SNP	OS02G0273700	Intronic	NA	NA	LOC_Os02g17350	VHS and GAT domain containing protein, expressed
Chr.2	9988938	9988938	A	A	G	SNP	OS02G0273900	Exonic	3UTR	NA	LOC_Os02g17370	expressed protein
Chr.2	9989135	9989135	A	A	G	SNP	OS02G0273900	Intronic	NA	NA	LOC_Os02g17370	expressed protein
Chr.2	9989255	9989255	A	A	G	SNP	OS02G0273900	Intronic	NA	NA	LOC_Os02g17370	expressed protein
Chr.2	9990063	9990063	A	A	G	SNP	OS02G0273900	Intronic	NA	NA	LOC_Os02g17370	expressed protein
Chr.2	16013092	16013092	A	A	G	SNP	OS02G0471500	Intronic	NA	NA	LOC_Os02g27220	protein phosphatase 2C, putative, expressed
Chr.2	17528256	17528256	A	A	G	SNP	OS02G0498300	Intronic	NA	NA	LOC_Os02g29510	DUF803 domain containing, putative, expressed
Chr.2	21810551	21810551	A	A	G	SNP	OS02G0570700	Exonic	3UTR	NA	LOC_Os02g36190	cytochrome P450, putative, expressed
Chr.2	21810555	21810555	A	A	G	SNP	OS02G0570700	Exonic	3UTR	NA	LOC_Os02g36190	cytochrome P450, putative, expressed
Chr.2	21843278	21843278	A	A	G	SNP	OS02G0570900	Exonic	CDS	Silent	LOC_Os02g36210	ent-kaurene synthase, chloroplast precursor, putative, expressed
Chr.2	21843320	21843320	A	A	G	SNP	OS02G0570900	Exonic	CDS	Silent	LOC_Os02g36210	ent-kaurene synthase, chloroplast precursor, putative, expressed

Chr.2	21843228	21843228	A	A	G	SNP	OS02G0570900	Exonic	CDS	Missense	LOC_Os02g36210	ent-kaurene synthase, chloroplast precursor, putative, expressed
Chr.2	23992797	23992797	A	A	G	SNP	OS02G0610600	Exonic	3UTR	NA	LOC_Os02g39720	splicing factor, arginine/serine-rich 7, putative, expressed
Chr.2	25190661	25190661	A	A	G	SNP	OS02G0629800	Intronic	NA	NA	LOC_Os02g41904	DEF7 - Defensin and Defensin-like DEFL family, expressed
Chr.2	28749837	28749837	A	A	G	SNP	OS02G0699000	Intronic	NA	NA	LOC_Os02g47090	peptide transporter PTR2, putative, expressed
Chr.2	28750442	28750442	A	A	G	SNP	OS02G0699000	Intronic	NA	NA	LOC_Os02g47090	peptide transporter PTR2, putative, expressed
Chr.2	34027402	34027402	A	A	G	SNP	OS02G0799000	Intronic	NA	NA	LOC_Os02g55560	protein phosphatase 2C, putative, expressed
Chr.2	34042330	34042330	A	A	G	SNP	OS02G0799300	Intronic	NA	NA	LOC_Os02g55590	expressed protein
Chr.2	34043213	34043213	A	A	G	SNP	OS02G0799300	Intronic	NA	NA	LOC_Os02g55590	expressed protein
Chr.2	34635883	34635883	A	A	G	SNP	OS02G0810200	Exonic	CDS	NA	LOC_Os02g56540	kinesin motor domain containing protein, putative, expressed
Chr.2	35098651	35098651	A	A	G	SNP	OS02G0817900	Exonic	CDS	Silent	LOC_Os02g57290	cytochrome P450, putative, expressed
Chr.2	35098657	35098657	A	A	G	SNP	OS02G0817900	Exonic	CDS	Silent	LOC_Os02g57290	cytochrome P450, putative, expressed
Chr.2	35095097	35095097	A	A	G	SNP	OS02G0817900	Intronic	NA	NA	LOC_Os02g57290	cytochrome P450, putative, expressed
Chr.2	35096240	35096240	A	A	G	SNP	OS02G0817900	Intronic	NA	NA	LOC_Os02g57290	cytochrome P450, putative, expressed
Chr.2	35096770	35096770	A	A	G	SNP	OS02G0817900	Intronic	NA	NA	LOC_Os02g57290	cytochrome P450, putative, expressed
Chr.2	35097963	35097963	A	A	G	SNP	OS02G0817900	Intronic	NA	NA	LOC_Os02g57290	cytochrome P450, putative, expressed
Chr.2	35098399	35098399	A	A	G	SNP	OS02G0817900	Intronic	NA	NA	LOC_Os02g57290	cytochrome P450, putative, expressed
Chr.2	35674964	35674964	A	A	G	SNP	OS02G0829800	Exonic	3UTR	NA	LOC_Os02g58300	expressed protein
Chr.2	81574	81574	A	A	G	SNP	OS02G0101500	Intronic	NA	NA	LOC_Os02g01150	erythronate-4-phosphate dehydrogenase domain containing protein, expressed
Chr.2	81594	81594	A	A	G	SNP	OS02G0101500	Intronic	NA	NA	LOC_Os02g01150	erythronate-4-phosphate dehydrogenase domain containing protein, expressed
Chr.2	714030	714030	C	C	G	SNP	OS02G0113200	Intronic	NA	NA	LOC_Os02g02230	cytochrome P450, putative, expressed
Chr.2	5155233	5155233	C	C	G	SNP	OS02G0192500	Intronic	NA	NA	LOC_Os02g09930	CSLA1 - cellulose synthase-like family A; mannan synthase, expressed
Chr.2	5195072	5195072	C	C	G	SNP	OS02G0193300	Exonic	CDS	Silent	LOC_Os02g09990	expressed protein
Chr.2	5760380	5760380	A	A	G	SNP	OS02G0202950	Exonic	CDS	NA	LOC_Os02g10860	bZIP transcription factor domain containing protein, expressed
Chr.2	6717370	6717370	A	A	G	SNP	OS02G0220500	Intronic	NA	NA	LOC_Os02g12794	elongation factor 1-gamma, putative, expressed
Chr.2	6812814	6812814	A	A	G	SNP	OS02G0222100	Exonic	3UTR	NA	LOC_Os02g12900	cysteine synthase, putative, expressed
Chr.2	6834641	6834641	T	T	G	SNP	OS02G0222500	Exonic	CDS	NA	LOC_Os02g12939	expressed protein
Chr.2	6834648	6834648	A	A	G	SNP	OS02G0222500	Exonic	CDS	NA	LOC_Os02g12939	expressed protein
Chr.2	7220989	7220989	A	A	G	SNP	OS02G0228300	Exonic	3UTR	NA	LOC_Os02g13510	receptor-like protein kinase 5 precursor, putative, expressed
Chr.2	7220993	7220993	A	A	G	SNP	OS02G0228300	Exonic	3UTR	NA	LOC_Os02g13510	receptor-like protein kinase 5 precursor, putative, expressed
Chr.2	7222212	7222212	C	C	G	SNP	OS02G0228300	Exonic	CDS	Silent	LOC_Os02g13510	receptor-like protein kinase 5 precursor, putative, expressed
Chr.2	7222254	7222254	C	C	G	SNP	OS02G0228300	Exonic	CDS	Silent	LOC_Os02g13510	receptor-like protein kinase 5 precursor, putative, expressed
Chr.2	7222938	7222938	A	A	G	SNP	OS02G0228300	Exonic	CDS	Silent	LOC_Os02g13510	receptor-like protein kinase 5 precursor, putative, expressed
Chr.2	7223699	7223699	A	A	G	SNP	OS02G0228300	Exonic	CDS	Silent	LOC_Os02g13510	receptor-like protein kinase 5 precursor, putative, expressed
Chr.2	7223901	7223901	A	A	G	SNP	OS02G0228300	Exonic	CDS	Silent	LOC_Os02g13510	receptor-like protein kinase 5 precursor, putative, expressed
Chr.2	7222207	7222207	A	A	G	SNP	OS02G0228300	Exonic	CDS	Missense	LOC_Os02g13510	receptor-like protein kinase 5 precursor, putative, expressed
Chr.2	7222671	7222671	C	C	G	SNP	OS02G0228300	Exonic	CDS	Missense	LOC_Os02g13510	receptor-like protein kinase 5 precursor, putative, expressed
Chr.2	7223493	7223493	C	C	G	SNP	OS02G0228300	Exonic	CDS	Missense	LOC_Os02g13510	receptor-like protein kinase 5 precursor, putative, expressed
Chr.2	7223534	7223534	A	A	G	SNP	OS02G0228300	Exonic	CDS	Missense	LOC_Os02g13510	receptor-like protein kinase 5 precursor, putative, expressed
Chr.2	7223558	7223558	C	C	G	SNP	OS02G0228300	Exonic	CDS	Missense	LOC_Os02g13510	receptor-like protein kinase 5 precursor, putative, expressed
Chr.2	7223710	7223710	A	A	G	SNP	OS02G0228300	Exonic	CDS	Missense	LOC_Os02g13510	receptor-like protein kinase 5 precursor, putative, expressed
Chr.2	7223770	7223770	A	A	G	SNP	OS02G0228300	Exonic	CDS	Missense	LOC_Os02g13510	receptor-like protein kinase 5 precursor, putative, expressed
Chr.2	9923909	9923909	A	A	G	SNP	OS02G0272900	Exonic	CDS	Silent	LOC_Os02g17304	resistance protein, putative, expressed
Chr.2	9974058	9974058	A	A	G	SNP	OS02G0273700	Intronic	NA	NA	LOC_Os02g17350	VHS and GAT domain containing protein, expressed
Chr.2	9975040	9975040	A	A	G	SNP	OS02G0273700	Intronic	NA	NA	LOC_Os02g17350	VHS and GAT domain containing protein, expressed
Chr.2	9976727	9976727	A	A	G	SNP	OS02G0273700	Intronic	NA	NA	LOC_Os02g17350	VHS and GAT domain containing protein, expressed
Chr.2	9976738	9976738	A	A	G	SNP	OS02G0273700	Intronic	NA	NA	LOC_Os02g17350	VHS and GAT domain containing protein, expressed
Chr.2	9976874	9976874	A	A	G	SNP	OS02G0273700	Intronic	NA	NA	LOC_Os02g17350	VHS and GAT domain containing protein, expressed
Chr.2	17535900	17535900	T	T	G	SNP	OS02G0498300	Intronic	NA	NA	LOC_Os02g29510	DUF803 domain containing, putative, expressed
Chr.2	18581390	18581390	T	T	G	SNP	OS02G0515200	Exonic	CDS	Missense	LOC_Os02g31080	hydroxyproline-rich glycoprotein family protein, putative, expressed
Chr.2	21843776	21843776	A	A	G	SNP	OS02G0570900	Exonic	CDS	Silent	LOC_Os02g36210	ent-kaurene synthase, chloroplast precursor, putative, expressed
Chr.2	22477799	22477799	A	A	G	SNP	OS02G0582900	Exonic	3UTR	NA	LOC_Os02g37190	expressed protein
Chr.2	22687937	22687937	T	T	G	SNP	OS02G0588500	Intronic	NA	NA	LOC_Os02g37590	glycerophosphoryl diester phosphodiesterase family protein, putative, expressed
Chr.2	23994985	23994985	A	A	G	SNP	OS02G0610600	Intronic	NA	NA	LOC_Os02g39720	splicing factor, arginine/serine-rich 7, putative, expressed
Chr.2	24569764	24569764	A	A	G	SNP	OS02G0618200	Exonic	5UTR	NA	LOC_Os02g40510	response regulator receiver domain containing protein, expressed
Chr.2	25153906	25153906	C	C	G	SNP	OS02G0629000	Exonic	CDS	Missense	LOC_Os02g41840	DUF584 domain containing protein, putative, expressed
Chr.2	28267320	28267320	A	A	G	SNP	OS02G0689200	Exonic	5UTR	NA	LOC_Os02g46380	purine permease, putative, expressed
Chr.2	33777486	33777486	C	C	G	SNP	OS02G0794600	Exonic	5UTR	NA	LOC_Os02g55134	cytochrome c oxidase copper chaperone, putative, expressed
Chr.2	34223335	34223335	T	T	G	SNP	OS02G0802700	Exonic	CDS	NA	LOC_Os02g55910	monogalactosyldiacylglycerol synthase, putative, expressed
Chr.2	34673830	34673830	A	A	G	SNP	OS02G0810900	Intronic	NA	NA	LOC_Os02g56600	no apical meristem protein, putative, expressed
Chr.2	35099274	35099274	A	A	G	SNP	OS02G0817900	Exonic	5UTR	NA	LOC_Os02g57290	cytochrome P450, putative, expressed
Chr.2	35099189	35099189	A	A	G	SNP	OS02G0817900	Exonic	CDS	Silent	LOC_Os02g57290	cytochrome P450, putative, expressed
Chr.2	35098811	35098811	A	A	G	SNP	OS02G0817900	Exonic	CDS	Missense	LOC_Os02g57290	cytochrome P450, putative, expressed
Chr.2	35097922	35097922	A	A	G	SNP	OS02G0817900	Intronic	NA	NA	LOC_Os02g57290	cytochrome P450, putative, expressed
Chr.2	80603	80606	GAGT	GAGT	G	INDEL	OS02G0101500	Intronic	NA	NA	LOC_Os02g01150	erythronate-4-phosphate dehydrogenase domain containing protein, expressed
Chr.2	121669	121682	GACACGGTTCATCC	GACACGGTTCATCC	G	INDEL	OS02G0102200	Exonic	3UTR	NA	LOC_Os02g01210	amino acid transporter, putative, expressed
Chr.2	3829422	3829423	GT	GT	G	INDEL	OS02G0170100	Intronic	NA	NA	LOC_Os02g07410	glycine cleavage system H protein, putative, expressed
Chr.2	5157567	5157569	GGT	GGT	G	INDEL	OS02G0192500	Intronic	NA	NA	LOC_Os02g09930	CSLA1 - cellulose synthase-like family A; mannan synthase, expressed
Chr.2	5252554	5252570	GTGGTTCCAGAAATCTC	GTGGTTCCAGAAATCTC	G	INDEL	OS02G0194200	Intronic	NA	NA	LOC_Os02g10080	zinc finger C-x8-C-x5-C-x3-H type family protein, expressed
Chr.2	5760065	5760066	GC	GC	G	INDEL	OS02G0202950	Exonic	CDS	NA	LOC_Os02g10860	bZIP transcription factor domain containing protein, expressed
Chr.2	7438194	7438214	GTTTGGGCTTGAAAGATGTGA	GTTTGGGCTTGAAAGATGTGA	G	INDEL	OS02G0231700	Exonic	CDS	FrameShift	LOC_Os02g13780	receptor-like protein kinase 2 precursor, putative, expressed
Chr.2	9923750	9923751	GT	GT	G	INDEL	OS02G0272900	Exonic	CDS	FrameShift	LOC_Os02g17304	resistance protein, putative, expressed
Chr.2	9972761	9972764	GAAA	GAAA	G	INDEL	OS02G0273700	Exonic	5UTR	NA	LOC_Os02g17350	VHS and GAT domain containing protein, expressed
Chr.2	9990170	9990178	GTTACAAAC	GTTACAAAC	G	INDEL	OS02G0273900	Intronic	NA	NA	LOC_Os02g17370	expressed protein
Chr.2	22298932	22298935	GTCC	GTCC	G	INDEL	OS02G0579600	Intronic	NA	NA	LOC_Os02g36924	OsMADS27 - MADS-box family gene with MIKCc type-box, expressed
Chr.2	24569586	24569598	GCCGCCACCACCA	GCCGCCACCACCA	G	INDEL	OS02G0618200	Exonic	5UTR	NA	LOC_Os02g40510	response regulator receiver domain containing protein, expressed
Chr.2	24879890	24879892	GGT	GGT	G	INDEL	OS02G0624300	Exonic	5UTR	NA	LOC_Os02g41510	MYB family transcription factor, putative, expressed
Chr.2	25779446	25779448	GAC	GAC	G	INDEL	OS02G0641900	Intronic	NA	NA	LOC_Os02g42870	MYB family transcription factor, putative, expressed
Chr.2	28268183	28268184	GA	GA	G	INDEL	OS02G0689200	Exonic	5UTR	NA	LOC_Os02g46380	purine permease, putative, expressed
Chr.2	35098573	35098581	GGGAGTTGC	GGGAGTTGC	G	INDEL	OS02G0817900	Intronic	NA	NA	LOC_Os02g57290	cytochrome P450, putative, expressed
Chr.12	25648265	25648265	T	T	G	SNP	OS12G0607100	Intronic	NA	NA	LOC_Os12g41400	eukaryotic translation initiation factor 2 subunit gamma, putative, expressed
Chr.12	23807344	23807345	GA	GA	G	INDEL	OS12G0576600	Intronic	NA	NA	LOC_Os12g38750	nucleotide pyrophosphatase/phosphodiesterase, putative, expressed
Chr.12	9918939	9918939	C	C	G	SNP	OS12G0271700	Intronic	NA	NA	LOC_Os12g17320	polyprenyl synthetase, putative, expressed
Chr.12	8963999	8963999	A	A	G	SNP	OS12G0258700	Exonic	3UTR	NA	LOC_Os12g15680	laccase precursor protein, putative, expressed
Chr.12	9915600	9915600	A	A	G	SNP	OS12G0271700	Exonic	3UTR	NA	LOC_Os12g17320	polyprenyl synthetase, putative, expressed
Chr.12	9918988	9918988	A	A	G	SNP	OS12G0271700	Intronic	NA	NA	LOC_Os12g17320	polyprenyl synthetase, putative, expressed

Chr.12	12987886	12987886	A	A	G	SNP	OS12G0418600	Intronic	NA	NA	LOC_Os12g22970	expressed protein
Chr.12	24872298	24872298	A	A	G	SNP	OS12G0592900	Exonic	3UTR	NA	LOC_Os12g40180	expressed protein
Chr.12	25651393	25651393	A	A	G	SNP	OS12G0607100	Exonic	CDS	Silent	LOC_Os12g41400	eukaryotic translation initiation factor 2 subunit gamma, putative, expressed
Chr.12	25648916	25648916	A	A	G	SNP	OS12G0607100	Intronic	NA	NA	LOC_Os12g41400	eukaryotic translation initiation factor 2 subunit gamma, putative, expressed
Chr.12	83601	83601	A	A	G	SNP	OS12G0101800	Intronic	NA	NA	LOC_Os12g01140	AGC_PVPK_like_kin82y.2 - ACG kinases include homologs to PKA, PKG and PKC, expressed
Chr.12	3131505	3131505	A	A	G	SNP	OS12G0162100	Intronic	NA	NA	LOC_Os12g06490	STE_PAK_Ste20_Slob_Wnk.6 - STE kinases include homologs to sterile 7, sterile 11 and sterile 20 from yeast, expressed
Chr.12	23420221	23420221	C	C	G	SNP	OS12G0569200	Exonic	CDS	Silent	LOC_Os12g38140	expressed protein
Chr.12	24872139	24872139	C	C	G	SNP	OS12G0592900	Exonic	3UTR	NA	LOC_Os12g40180	expressed protein
Chr.12	24872080	24872080	A	A	G	SNP	OS12G0592900	Exonic	CDS	Missense	LOC_Os12g40180	expressed protein
Chr.12	25103731	25103731	C	C	G	SNP	OS12G0597700	Intronic	NA	NA	LOC_Os12g40570	WRKY94, expressed
Chr.12	25445777	25445777	A	A	G	SNP	OS12G0603700	Exonic	CDS	Silent	LOC_Os12g41090	CAMK_KIN1/SNF1/Nim1_like.38 - CAMK includes calcium/calmodulin depedent protein kinases, expressed
Chr.12	25445858	25445858	C	C	G	SNP	OS12G0603700	Exonic	CDS	Silent	LOC_Os12g41090	CAMK_KIN1/SNF1/Nim1_like.38 - CAMK includes calcium/calmodulin depedent protein kinases, expressed
Chr.12	25445203	25445203	T	T	G	SNP	OS12G0603700	Exonic	CDS	Missense	LOC_Os12g41090	CAMK_KIN1/SNF1/Nim1_like.38 - CAMK includes calcium/calmodulin depedent protein kinases, expressed
Chr.12	25445220	25445220	C	C	G	SNP	OS12G0603700	Exonic	CDS	Missense	LOC_Os12g41090	CAMK_KIN1/SNF1/Nim1_like.38 - CAMK includes calcium/calmodulin depedent protein kinases, expressed
Chr.12	25445479	25445479	C	C	G	SNP	OS12G0603700	Exonic	CDS	Missense	LOC_Os12g41090	CAMK_KIN1/SNF1/Nim1_like.38 - CAMK includes calcium/calmodulin depedent protein kinases, expressed
Chr.12	25647490	25647490	T	T	G	SNP	OS12G0607100	Exonic	5UTR	NA	LOC_Os12g41400	eukaryotic translation initiation factor 2 subunit gamma, putative, expressed
Chr.12	25648876	25648876	T	T	G	SNP	OS12G0607100	Intronic	NA	NA	LOC_Os12g41400	eukaryotic translation initiation factor 2 subunit gamma, putative, expressed
Chr.12	25521039	25521041	GAA	GAA	G	INDEL	OS12G0604700	Intronic	NA	NA	LOC_Os12g41180	LSTK-1-like kinase, putative, expressed
Chr.12	25648751	25648752	GT	GT	G	INDEL	OS12G0607100	Intronic	NA	NA	LOC_Os12g41400	eukaryotic translation initiation factor 2 subunit gamma, putative, expressed
Chr.12	25649640	25649641	GT	GT	G	INDEL	OS12G0607100	Intronic	NA	NA	LOC_Os12g41400	eukaryotic translation initiation factor 2 subunit gamma, putative, expressed
Chr.11	4249575	4249575	T	T	G	SNP	OS11G0183900	Exonic	CDS	Silent	LOC_Os11g08100	eukaryotic aspartyl protease domain containing protein, expressed
Chr.11	4250873	4250873	T	T	G	SNP	OS11G0183900	Exonic	CDS	Missense	LOC_Os11g08100	eukaryotic aspartyl protease domain containing protein, expressed
Chr.11	4248528	4248528	T	T	G	SNP	OS11G0183900	Intronic	NA	NA	LOC_Os11g08100	eukaryotic aspartyl protease domain containing protein, expressed
Chr.11	6639441	6639441	T	T	G	SNP	OS11G0226933	Exonic	CDS	NA	LOC_Os11g11960	disease resistance protein RPM1, putative, expressed
Chr.11	6639471	6639471	T	T	G	SNP	OS11G0226933	Exonic	CDS	NA	LOC_Os11g11960	disease resistance protein RPM1, putative, expressed
Chr.11	11593533	11593533	T	T	G	SNP	OS11G0306400	Intronic	NA	NA	LOC_Os11g20090	O-methyltransferase, putative, expressed
Chr.11	14049689	14049689	T	T	G	SNP	OS11G0434000	Intronic	NA	NA	LOC_Os11g24630	magnesium-dependent phosphatase 1, putative, expressed
Chr.11	14051240	14051240	T	T	G	SNP	OS11G0434000	Intronic	NA	NA	LOC_Os11g24630	magnesium-dependent phosphatase 1, putative, expressed
Chr.11	17353552	17353552	T	T	G	SNP	OS11G0490900	Intronic	NA	NA	LOC_Os11g29870	WRKY72, expressed
Chr.11	21300180	21300180	T	T	G	SNP	OS11G0570000	Exonic	3UTR	NA	LOC_Os11g36200	receptor-like protein kinase 2 precursor, putative, expressed
Chr.11	23185590	23185590	T	T	G	SNP	OS11G0602600	Intronic	NA	NA	LOC_Os11g38959	40S ribosomal protein S9-2, putative, expressed
Chr.11	27488592	27488592	T	T	G	SNP	OS11G0679700	Intronic	NA	NA	LOC_Os11g45400	glycerol-3-phosphate acyltransferase, putative, expressed
Chr.11	2425621	2425621	T	T	G	SNP	OS11G0151700	Intronic	NA	NA	LOC_Os11g05400	Ser/Thr protein phosphatase family protein, putative, expressed
Chr.11	2606043	2606043	T	T	G	SNP	OS11G0155600	Exonic	3UTR	NA	LOC_Os11g05700	ABC transporter family protein, putative, expressed
Chr.11	2611594	2611594	T	T	G	SNP	OS11G0155600	Intronic	NA	NA	LOC_Os11g05700	ABC transporter family protein, putative, expressed
Chr.11	6638383	6638383	T	T	G	SNP	OS11G0226933	Exonic	3UTR	NA	LOC_Os11g11960	disease resistance protein RPM1, putative, expressed
Chr.11	7126795	7126795	T	T	G	SNP	OS11G0234200	Intronic	NA	NA	LOC_Os11g12650	PHD-finger domain containing protein, putative, expressed
Chr.11	7963276	7963276	T	T	G	SNP	OS11G0247300	Exonic	3UTR	NA	LOC_Os11g14220	tubulin/FtsZ domain containing protein, putative, expressed
Chr.11	8389410	8389410	T	T	G	SNP	OS11G0255500	Intronic	NA	NA	LOC_Os11g14910	NADP-dependent oxidoreductase, putative, expressed
Chr.11	8444091	8444091	T	T	G	SNP	OS11G0256200	Intronic	NA	NA	LOC_Os11g14990	expressed protein
Chr.11	10865295	10865295	T	T	G	SNP	OS11G0295900	Intronic	NA	NA	LOC_Os11g19060	BABY BOOM 1, putative, expressed
Chr.11	8443336	8443336	C	C	G	SNP	OS11G0256200	Exonic	3UTR	NA	LOC_Os11g14990	expressed protein
Chr.11	10867437	10867437	C	C	G	SNP	OS11G0295900	Intronic	NA	NA	LOC_Os11g19060	BABY BOOM 1, putative, expressed
Chr.11	762245	762245	A	A	G	SNP	OS11G0116900	Intronic	NA	NA	LOC_Os11g02480	WRKY46, expressed
Chr.11	2425512	2425512	A	A	G	SNP	OS11G0151700	Exonic	CDS	NA	LOC_Os11g05400	Ser/Thr protein phosphatase family protein, putative, expressed
Chr.11	2424412	2424412	A	A	G	SNP	OS11G0151700	Intronic	NA	NA	LOC_Os11g05400	Ser/Thr protein phosphatase family protein, putative, expressed
Chr.11	2425654	2425654	A	A	G	SNP	OS11G0151700	Intronic	NA	NA	LOC_Os11g05400	Ser/Thr protein phosphatase family protein, putative, expressed
Chr.11	2425708	2425708	A	A	G	SNP	OS11G0151700	Intronic	NA	NA	LOC_Os11g05400	Ser/Thr protein phosphatase family protein, putative, expressed
Chr.11	2607602	2607602	A	A	G	SNP	OS11G0155600	Exonic	3UTR	NA	LOC_Os11g05700	ABC transporter family protein, putative, expressed
Chr.11	2611306	2611306	A	A	G	SNP	OS11G0155600	Intronic	NA	NA	LOC_Os11g05700	ABC transporter family protein, putative, expressed
Chr.11	2613717	2613717	A	A	G	SNP	OS11G0155600	Intronic	NA	NA	LOC_Os11g05700	ABC transporter family protein, putative, expressed
Chr.11	6638483	6638483	A	A	G	SNP	OS11G0226933	Exonic	CDS	NA	LOC_Os11g11960	disease resistance protein RPM1, putative, expressed
Chr.11	6638748	6638748	A	A	G	SNP	OS11G0226933	Exonic	CDS	NA	LOC_Os11g11960	disease resistance protein RPM1, putative, expressed
Chr.11	7127755	7127755	A	A	G	SNP	OS11G0234200	Intronic	NA	NA	LOC_Os11g12650	PHD-finger domain containing protein, putative, expressed
Chr.11	7127890	7127890	A	A	G	SNP	OS11G0234200	Intronic	NA	NA	LOC_Os11g12650	PHD-finger domain containing protein, putative, expressed
Chr.11	8387958	8387958	A	A	G	SNP	OS11G0255500	Exonic	CDS	Silent	LOC_Os11g14910	NADP-dependent oxidoreductase, putative, expressed
Chr.11	8443404	8443404	A	A	G	SNP	OS11G0256200	Exonic	3UTR	NA	LOC_Os11g14990	expressed protein
Chr.11	8444409	8444409	A	A	G	SNP	OS11G0256200	Intronic	NA	NA	LOC_Os11g14990	expressed protein
Chr.11	8444428	8444428	A	A	G	SNP	OS11G0256200	Intronic	NA	NA	LOC_Os11g14990	expressed protein
Chr.11	22177486	22177486	A	A	G	SNP	OS11G0586300	Exonic	CDS	NA	LOC_Os11g37560	expressed protein
Chr.11	28935426	28935446	GAGGTATGTATGCATATATCC	GAGGTATGTATGCATATATCC	G	INDEL	OS11G0707000	Intronic	NA	NA	LOC_Os11g47970	AAA-type ATPase family protein, putative, expressed
Chr.11	2763319	2763319	C	C	G	SNP	OS11G0157400	Intronic	NA	NA	LOC_Os11g05880	exo70 exocyst complex subunit, putative, expressed
Chr.11	4247902	4247902	C	C	G	SNP	OS11G0183900	Intronic	NA	NA	LOC_Os11g08100	eukaryotic aspartyl protease domain containing protein, expressed
Chr.11	6639558	6639558	C	C	G	SNP	OS11G0226933	Exonic	CDS	NA	LOC_Os11g11960	disease resistance protein RPM1, putative, expressed
Chr.11	6649210	6649210	C	C	G	SNP	OS11G0227000	Exonic	5UTR	NA	LOC_Os11g11980	expressed protein
Chr.11	9055920	9055920	C	C	G	SNP	OS11G0265200	Exonic	3UTR	NA	LOC_Os11g16390	expressed protein
Chr.11	9056038	9056038	C	C	G	SNP	OS11G0265200	Exonic	CDS	Missense	LOC_Os11g16390	expressed protein
Chr.11	10143414	10143414	C	C	G	SNP	OS11G0283500	Exonic	CDS	Silent	LOC_Os11g18044	peptide transporter PTR2, putative, expressed
Chr.11	11593032	11593032	C	C	G	SNP	OS11G0306400	Intronic	NA	NA	LOC_Os11g20090	O-methyltransferase, putative, expressed
Chr.11	17354181	17354181	C	C	G	SNP	OS11G0490900	Intronic	NA	NA	LOC_Os11g29870	WRKY72, expressed
Chr.11	23906799	23906799	C	C	G	SNP	OS11G0615000	Intronic	NA	NA	LOC_Os11g40080	lipin, N-terminal conserved region family protein, expressed
Chr.11	25425258	25425258	C	C	G	SNP	OS11G0641500	Exonic	CDS	Silent	LOC_Os11g42200	laccase precursor protein, putative, expressed
Chr.11	25424313	25424313	C	C	G	SNP	OS11G0641500	Intronic	NA	NA	LOC_Os11g42200	laccase precursor protein, putative, expressed
Chr.11	27488294	27488294	C	C	G	SNP	OS11G0679700	Intronic	NA	NA	LOC_Os11g45400	glycerol-3-phosphate acyltransferase, putative, expressed
Chr.11	759767	759767	A	A	G	SNP	OS11G0116900	Exonic	3UTR	NA	LOC_Os11g02480	WRKY46, expressed
Chr.11	4251620	4251620	A	A	G	SNP	OS11G0183900	Exonic	3UTR	NA	LOC_Os11g08100	eukaryotic aspartyl protease domain containing protein, expressed
Chr.11	4251633	4251633	A	A	G	SNP	OS11G0183900	Exonic	3UTR	NA	LOC_Os11g08100	eukaryotic aspartyl protease domain containing protein, expressed
Chr.11	4249398	4249398	A	A	G	SNP	OS11G0183900	Exonic	CDS	Silent	LOC_Os11g08100	eukaryotic aspartyl protease domain containing protein, expressed
Chr.11	4249584	4249584	A	A	G	SNP	OS11G0183900	Exonic	CDS	Silent	LOC_Os11g08100	eukaryotic aspartyl protease domain containing protein, expressed
Chr.11	4249716	4249716	A	A	G	SNP	OS11G0183900	Exonic	CDS	Silent	LOC_Os11g08100	eukaryotic aspartyl protease domain containing protein, expressed
Chr.11	4250898	4250898	A	A	G	SNP	OS11G0183900	Exonic	CDS	Missense	LOC_Os11g08100	eukaryotic aspartyl protease domain containing protein, expressed
Chr.11	4248020	4248020	A	A	G	SNP	OS11G0183900	Intronic	NA	NA	LOC_Os11g08100	eukaryotic aspartyl protease domain containing protein, expressed
Chr.11	4249020	4249020	A	A	G	SNP	OS11G0183900	Intronic	NA	NA	LOC_Os11g08100	eukaryotic aspartyl protease domain containing protein, expressed
Chr.11	4250479	4250479	A	A	G	SNP	OS11G0183900	Intronic	NA	NA	LOC_Os11g08100	eukaryotic aspartyl protease domain containing protein, expressed
Chr.11	4251130	4251130	A	A	G	SNP	OS11G0183900	Intronic	NA	NA	LOC_Os11g08100	eukaryotic aspartyl protease domain containing protein, expressed

Chr.11	6638566	6638566	A	A	G	SNP	OS11G0226933	Exonic	CDS	NA	LOC_Os11g11960	disease resistance protein RPM1, putative, expressed
Chr.11	6638905	6638905	A	A	G	SNP	OS11G0226933	Exonic	CDS	NA	LOC_Os11g11960	disease resistance protein RPM1, putative, expressed
Chr.11	6639432	6639432	A	A	G	SNP	OS11G0226933	Exonic	CDS	NA	LOC_Os11g11960	disease resistance protein RPM1, putative, expressed
Chr.11	6639436	6639436	A	A	G	SNP	OS11G0226933	Exonic	CDS	NA	LOC_Os11g11960	disease resistance protein RPM1, putative, expressed
Chr.11	6639485	6639485	A	A	G	SNP	OS11G0226933	Exonic	CDS	NA	LOC_Os11g11960	disease resistance protein RPM1, putative, expressed
Chr.11	6650103	6650103	A	A	G	SNP	OS11G0227000	Exonic	3UTR	NA	LOC_Os11g11980	expressed protein
Chr.11	6649862	6649862	A	A	G	SNP	OS11G0227000	Exonic	CDS	Missense	LOC_Os11g11980	expressed protein
Chr.11	6649705	6649705	A	A	G	SNP	OS11G0227000	Intronic	NA	NA	LOC_Os11g11980	expressed protein
Chr.11	9189278	9189278	A	A	G	SNP	OS11G0267300	Exonic	CDS	Silent	LOC_Os11g16580	endonuclease III-like protein 1, putative, expressed
Chr.11	9188185	9188185	A	A	G	SNP	OS11G0267300	Intronic	NA	NA	LOC_Os11g16580	endonuclease III-like protein 1, putative, expressed
Chr.11	10141281	10141281	A	A	G	SNP	OS11G0283500	Intronic	NA	NA	LOC_Os11g18044	peptide transporter PTR2, putative, expressed
Chr.11	14048997	14048997	A	A	G	SNP	OS11G0434000	Intronic	NA	NA	LOC_Os11g24630	magnesium-dependent phosphatase 1, putative, expressed
Chr.11	14049317	14049317	A	A	G	SNP	OS11G0434000	Intronic	NA	NA	LOC_Os11g24630	magnesium-dependent phosphatase 1, putative, expressed
Chr.11	14050917	14050917	A	A	G	SNP	OS11G0434000	Intronic	NA	NA	LOC_Os11g24630	magnesium-dependent phosphatase 1, putative, expressed
Chr.11	14051382	14051382	A	A	G	SNP	OS11G0434000	Intronic	NA	NA	LOC_Os11g24630	magnesium-dependent phosphatase 1, putative, expressed
Chr.11	17355355	17355355	A	A	G	SNP	OS11G0490900	Exonic	CDS	Silent	LOC_Os11g29870	WRKY72, expressed
Chr.11	17352825	17352825	A	A	G	SNP	OS11G0490900	Intronic	NA	NA	LOC_Os11g29870	WRKY72, expressed
Chr.11	17352861	17352861	A	A	G	SNP	OS11G0490900	Intronic	NA	NA	LOC_Os11g29870	WRKY72, expressed
Chr.11	17353603	17353603	A	A	G	SNP	OS11G0490900	Intronic	NA	NA	LOC_Os11g29870	WRKY72, expressed
Chr.11	17353687	17353687	A	A	G	SNP	OS11G0490900	Intronic	NA	NA	LOC_Os11g29870	WRKY72, expressed
Chr.11	17353721	17353721	A	A	G	SNP	OS11G0490900	Intronic	NA	NA	LOC_Os11g29870	WRKY72, expressed
Chr.11	17354926	17354926	A	A	G	SNP	OS11G0490900	Intronic	NA	NA	LOC_Os11g29870	WRKY72, expressed
Chr.11	18935745	18935745	A	A	G	SNP	OS11G0523800	Exonic	3UTR	NA	LOC_Os11g32110	auxin response factor, putative, expressed
Chr.11	18936099	18936099	A	A	G	SNP	OS11G0523800	Exonic	3UTR	NA	LOC_Os11g32110	auxin response factor, putative, expressed
Chr.11	18937657	18937657	A	A	G	SNP	OS11G0523800	Exonic	3UTR	NA	LOC_Os11g32110	auxin response factor, putative, expressed
Chr.11	18939571	18939571	A	A	G	SNP	OS11G0523800	Intronic	NA	NA	LOC_Os11g32110	auxin response factor, putative, expressed
Chr.11	21299810	21299810	A	A	G	SNP	OS11G0570000	Exonic	3UTR	NA	LOC_Os11g36200	receptor-like protein kinase 2 precursor, putative, expressed
Chr.11	22276949	22276949	A	A	G	SNP	OS11G0587600	Exonic	3UTR	NA	LOC_Os11g37700	pleiotropic drug resistance protein, putative, expressed
Chr.11	23187446	23187446	A	A	G	SNP	OS11G0602600	Exonic	3UTR	NA	LOC_Os11g38959	40S ribosomal protein S9-2, putative, expressed
Chr.11	23232371	23232371	A	A	G	SNP	OS11G0603200	Exonic	CDS	Silent	LOC_Os11g39020	ABC transporter, ATP-binding protein, putative, expressed
Chr.11	23907550	23907550	A	A	G	SNP	OS11G0615000	Intronic	NA	NA	LOC_Os11g40080	lipin, N-terminal conserved region family protein, expressed
Chr.11	23907910	23907910	A	A	G	SNP	OS11G0615000	Intronic	NA	NA	LOC_Os11g40080	lipin, N-terminal conserved region family protein, expressed
Chr.11	25425784	25425784	A	A	G	SNP	OS11G0641500	Exonic	CDS	Silent	LOC_Os11g42200	laccase precursor protein, putative, expressed
Chr.11	25424410	25424410	A	A	G	SNP	OS11G0641500	Intronic	NA	NA	LOC_Os11g42200	laccase precursor protein, putative, expressed
Chr.11	651682	651682	A	A	G	SNP	OS11G0114300	Exonic	CDS	NA	LOC_Os11g02290	expressed protein
Chr.11	2424465	2424465	A	A	G	SNP	OS11G0151700	Intronic	NA	NA	LOC_Os11g05400	Ser/Thr protein phosphatase family protein, putative, expressed
Chr.11	2762033	2762033	A	A	G	SNP	OS11G0157400	Intronic	NA	NA	LOC_Os11g05880	exo70 exocyst complex subunit, putative, expressed
Chr.11	2762755	2762755	A	A	G	SNP	OS11G0157400	Intronic	NA	NA	LOC_Os11g05880	exo70 exocyst complex subunit, putative, expressed
Chr.11	4251454	4251454	T	T	G	SNP	OS11G0183900	Exonic	3UTR	NA	LOC_Os11g08100	eukaryotic aspartyl protease domain containing protein, expressed
Chr.11	4247592	4247592	C	C	G	SNP	OS11G0183900	Exonic	CDS	Silent	LOC_Os11g08100	eukaryotic aspartyl protease domain containing protein, expressed
Chr.11	4248795	4248795	A	A	G	SNP	OS11G0183900	Intronic	NA	NA	LOC_Os11g08100	eukaryotic aspartyl protease domain containing protein, expressed
Chr.11	4248972	4248972	A	A	G	SNP	OS11G0183900	Intronic	NA	NA	LOC_Os11g08100	eukaryotic aspartyl protease domain containing protein, expressed
Chr.11	6639532	6639532	C	C	G	SNP	OS11G0226933	Exonic	CDS	NA	LOC_Os11g11960	disease resistance protein RPM1, putative, expressed
Chr.11	6649530	6649530	A	A	G	SNP	OS11G0227000	Exonic	CDS	Missense	LOC_Os11g11980	expressed protein
Chr.11	6649595	6649595	C	C	G	SNP	OS11G0227000	Intronic	NA	NA	LOC_Os11g11980	expressed protein
Chr.11	7128574	7128574	A	A	G	SNP	OS11G0234200	Exonic	CDS	Missense	LOC_Os11g12650	PHD-finger domain containing protein, putative, expressed
Chr.11	7552060	7552060	A	A	G	SNP	OS11G0241200	Exonic	3UTR	NA	LOC_Os11g13720	expressed protein
Chr.11	7552592	7552592	T	T	G	SNP	OS11G0241200	Exonic	CDS	Missense	LOC_Os11g13720	expressed protein
Chr.11	7961104	7961104	T	T	G	SNP	OS11G0247300	Intronic	NA	NA	LOC_Os11g14220	tubulin/FtsZ domain containing protein, putative, expressed
Chr.11	8446043	8446043	C	C	G	SNP	OS11G0256200	Exonic	5UTR	NA	LOC_Os11g14990	expressed protein
Chr.11	9190223	9190223	A	A	G	SNP	OS11G0267300	Exonic	3UTR	NA	LOC_Os11g16580	endonuclease III-like protein 1, putative, expressed
Chr.11	9188389	9188389	A	A	G	SNP	OS11G0267300	Intronic	NA	NA	LOC_Os11g16580	endonuclease III-like protein 1, putative, expressed
Chr.11	9188704	9188704	T	T	G	SNP	OS11G0267300	Intronic	NA	NA	LOC_Os11g16580	endonuclease III-like protein 1, putative, expressed
Chr.11	10140964	10140964	A	A	G	SNP	OS11G0283500	Intronic	NA	NA	LOC_Os11g18044	peptide transporter PTR2, putative, expressed
Chr.11	14050884	14050884	T	T	G	SNP	OS11G0434000	Intronic	NA	NA	LOC_Os11g24630	magnesium-dependent phosphatase 1, putative, expressed
Chr.11	17284299	17284299	C	C	G	SNP	OS11G0490100	Exonic	CDS	Silent	LOC_Os11g29780	plant-specific domain TIGR01627 family protein, expressed
Chr.11	17284557	17284557	C	C	G	SNP	OS11G0490100	Exonic	CDS	Silent	LOC_Os11g29780	plant-specific domain TIGR01627 family protein, expressed
Chr.11	17353795	17353795	A	A	G	SNP	OS11G0490900	Intronic	NA	NA	LOC_Os11g29870	WRKY72, expressed
Chr.11	18936331	18936331	A	A	G	SNP	OS11G0523800	Exonic	3UTR	NA	LOC_Os11g32110	auxin response factor, putative, expressed
Chr.11	18937213	18937213	A	A	G	SNP	OS11G0523800	Exonic	3UTR	NA	LOC_Os11g32110	auxin response factor, putative, expressed
Chr.11	18941272	18941272	T	T	G	SNP	OS11G0523800	Exonic	CDS	Silent	LOC_Os11g32110	auxin response factor, putative, expressed
Chr.11	21298888	21298888	A	A	G	SNP	OS11G0570000	Exonic	CDS	Silent	LOC_Os11g36200	receptor-like protein kinase 2 precursor, putative, expressed
Chr.11	21464124	21464124	A	A	G	SNP	OS11G0572200	Exonic	CDS	Silent	LOC_Os11g36400	expressed protein
Chr.11	22178281	22178281	C	C	G	SNP	OS11G0586300	Exonic	CDS	NA	LOC_Os11g37560	expressed protein
Chr.11	22177624	22177624	A	A	G	SNP	OS11G0586300	Intronic	NA	NA	LOC_Os11g37560	expressed protein
Chr.11	22177653	22177653	A	A	G	SNP	OS11G0586300	Intronic	NA	NA	LOC_Os11g37560	expressed protein
Chr.11	22178518	22178518	C	C	G	SNP	OS11G0586300	Intronic	NA	NA	LOC_Os11g37560	expressed protein
Chr.11	22282054	22282054	C	C	G	SNP	OS11G0587600	Exonic	CDS	Missense	LOC_Os11g37700	pleiotropic drug resistance protein, putative, expressed
Chr.11	22281585	22281585	A	A	G	SNP	OS11G0587600	Intronic	NA	NA	LOC_Os11g37700	pleiotropic drug resistance protein, putative, expressed
Chr.11	23187392	23187392	C	C	G	SNP	OS11G0602600	Exonic	3UTR	NA	LOC_Os11g38959	40S ribosomal protein S9-2, putative, expressed
Chr.11	23230465	23230465	A	A	G	SNP	OS11G0603200	Exonic	CDS	Missense	LOC_Os11g39020	ABC transporter, ATP-binding protein, putative, expressed
Chr.11	24227220	24227220	C	C	G	SNP	OS11G0621400	Intronic	NA	NA	LOC_Os11g40600	expressed protein
Chr.11	25424863	25424863	A	A	G	SNP	OS11G0641500	Exonic	CDS	Missense	LOC_Os11g42200	laccase precursor protein, putative, expressed
Chr.11	27487346	27487346	C	C	G	SNP	OS11G0679700	Exonic	CDS	NA	LOC_Os11g45400	glycerol-3-phosphate acyltransferase, putative, expressed
Chr.11	27488783	27488783	C	C	G	SNP	OS11G0679700	Intronic	NA	NA	LOC_Os11g45400	glycerol-3-phosphate acyltransferase, putative, expressed
Chr.11	27488997	27488997	A	A	G	SNP	OS11G0679700	Intronic	NA	NA	LOC_Os11g45400	glycerol-3-phosphate acyltransferase, putative, expressed
Chr.11	27489007	27489007	A	A	G	SNP	OS11G0679700	Intronic	NA	NA	LOC_Os11g45400	glycerol-3-phosphate acyltransferase, putative, expressed
Chr.11	2609040	2609045	GAAATA	GAAATA	G	INDEL	OS11G0155600	Intronic	NA	NA	LOC_Os11g05700	ABC transporter family protein, putative, expressed
Chr.11	4973705	4973706	GA	GA	G	INDEL	OS11G0199200	Intronic	NA	NA	LOC_Os11g09280	OsPDIL1-1 protein disulfide isomerase PDIL1-1, expressed
Chr.11	6323481	6323482	GT	GT	G	INDEL	OS11G0220800	Intronic	NA	NA	LOC_Os11g11390	ribosomal protein, putative, expressed
Chr.11	6649907	6649908	GA	GA	G	INDEL	OS11G0227000	Exonic	3UTR	NA	LOC_Os11g11980	expressed protein
Chr.11	8446000	8446002	GGA	GGA	G	INDEL	OS11G0256200	Exonic	5UTR	NA	LOC_Os11g14990	expressed protein
Chr.11	14049231	14049234	GACA	GACA	G	INDEL	OS11G0434000	Intronic	NA	NA	LOC_Os11g24630	magnesium-dependent phosphatase 1, putative, expressed
Chr.11	17235650	17235654	GATCT	GATCT	G	INDEL	OS11G0489250	Intronic	NA	NA	LOC_Os11g29720	cytochrome P450, putative, expressed
Chr.11	18835218	18835221	GAGA	GAGA	G	INDEL	OS11G0522900	Exonic	3UTR	NA	LOC_Os11g31980	OsSCP63 - Putative Serine Carboxypeptidase homologue, expressed

Chr.11	20047542	20047543	GA	GA	G	INDEL	OS11G0545000	Exonic	3UTR	NA	LOC_Os11g34230	expressed protein
Chr.11	27489993	27489996	GGAA	GGAA	G	INDEL	OS11G0679700	Exonic	CDS	NA	LOC_Os11g45400	glycerol-3-phosphate acyltransferase, putative, expressed
Chr.11	28956660	28956661	GA	GA	G	INDEL	OS11G0707600	Intronic	NA	NA	LOC_Os11g48020	fatty acid hydroxylase, putative, expressed
Chr.10	19361343	19361343	T	T	G	SNP	OS10G0506200	Intronic	NA	NA	LOC_Os10g36210	valyl-tRNA synthetase, putative, expressed
Chr.10	6272893	6272893	T	T	G	SNP	OS10G0190500	Exonic	3UTR	NA	LOC_Os10g11310	expressed protein
Chr.10	2360037	2360037	T	T	G	SNP	OS10G0138100	Intronic	NA	NA	LOC_Os10g04860	aldehyde oxidase, putative, expressed
Chr.10	18640937	18640937	A	A	G	SNP	OS10G0491000	Intronic	NA	NA	LOC_Os10g34930	secretory protein, putative, expressed
Chr.10	18533508	18533508	T	T	G	SNP	OS10G0488800	Intronic	NA	NA	LOC_Os10g34710	myosin-2 heavy chain, non muscle, putative, expressed
Chr.10	21758108	21758108	C	C	G	SNP	OS10G0554200	Exonic	3UTR	NA	LOC_Os10g40600	peptide transporter PTR2, putative, expressed
Chr.10	15307871	15307871	C	C	G	SNP	OS10G0430200	Intronic	NA	NA	LOC_Os10g29470	dehydrogenase, putative, expressed
Chr.10	12971407	12971407	C	C	G	SNP	OS10G0390500	Intronic	NA	NA	LOC_Os10g25130	aminotransferase, classes I and II, domain containing protein, expressed
Chr.10	12969300	12969300	C	C	G	SNP	OS10G0390500	Intronic	NA	NA	LOC_Os10g25130	aminotransferase, classes I and II, domain containing protein, expressed
Chr.10	6299835	6299835	C	C	G	SNP	OS10G0190900	Intronic	NA	NA	LOC_Os10g11354	MATE efflux family protein, putative, expressed
Chr.10	6274444	6274444	C	C	G	SNP	OS10G0190500	Exonic	CDS	Silent	LOC_Os10g11310	expressed protein
Chr.10	2362565	2362565	C	C	G	SNP	OS10G0138100	Exonic	CDS	Silent	LOC_Os10g04860	aldehyde oxidase, putative, expressed
Chr.10	23088894	23088894	A	A	G	SNP	OS10G0579100	Exonic	CDS	Silent	LOC_Os10g42820	early-responsive to dehydration protein-related, putative, expressed
Chr.10	19607715	19607715	A	A	G	SNP	OS10G0510000	Intronic	NA	NA	LOC_Os10g36650	actin, putative, expressed
Chr.10	19607584	19607584	A	A	G	SNP	OS10G0510000	Intronic	NA	NA	LOC_Os10g36650	actin, putative, expressed
Chr.10	15638119	15638119	A	A	G	SNP	OS10G0437200	Exonic	3UTR	NA	LOC_Os10g30100	3-5 exonuclease family protein, putative, expressed
Chr.10	15308139	15308139	A	A	G	SNP	OS10G0430200	Intronic	NA	NA	LOC_Os10g29470	dehydrogenase, putative, expressed
Chr.10	6299760	6299760	A	A	G	SNP	OS10G0190900	Intronic	NA	NA	LOC_Os10g11354	MATE efflux family protein, putative, expressed
Chr.10	6299753	6299753	A	A	G	SNP	OS10G0190900	Intronic	NA	NA	LOC_Os10g11354	MATE efflux family protein, putative, expressed
Chr.10	6298635	6298635	A	A	G	SNP	OS10G0190900	Intronic	NA	NA	LOC_Os10g11354	MATE efflux family protein, putative, expressed
Chr.10	6298557	6298557	A	A	G	SNP	OS10G0190900	Intronic	NA	NA	LOC_Os10g11354	MATE efflux family protein, putative, expressed
Chr.10	3755338	3755338	A	A	G	SNP	OS10G0159300	Exonic	5UTR	NA	LOC_Os10g07160	retrotransposon, putative, centromere-specific, expressed
Chr.10	2360844	2360844	A	A	G	SNP	OS10G0138100	Intronic	NA	NA	LOC_Os10g04860	aldehyde oxidase, putative, expressed
Chr.10	2360779	2360779	A	A	G	SNP	OS10G0138100	Intronic	NA	NA	LOC_Os10g04860	aldehyde oxidase, putative, expressed
Chr.10	2360055	2360055	A	A	G	SNP	OS10G0138100	Intronic	NA	NA	LOC_Os10g04860	aldehyde oxidase, putative, expressed
Chr.10	1084483	1084483	A	A	G	SNP	OS10G0116800	Exonic	CDS	Missense	LOC_Os10g02750	Ser/Thr protein phosphatase family protein, putative, expressed
Chr.10	378930	378930	A	A	G	SNP	OS10G0104900	Intronic	NA	NA	LOC_Os10g01570	C-5 cytosine-specific DNA methylase, putative, expressed
Chr.10	378471	378471	A	A	G	SNP	OS10G0104900	Intronic	NA	NA	LOC_Os10g01570	C-5 cytosine-specific DNA methylase, putative, expressed
Chr.10	379819	379819	T	T	G	SNP	OS10G0104900	Intronic	NA	NA	LOC_Os10g01570	C-5 cytosine-specific DNA methylase, putative, expressed
Chr.10	1083378	1083378	A	A	G	SNP	OS10G0116800	Exonic	CDS	Missense	LOC_Os10g02750	Ser/Thr protein phosphatase family protein, putative, expressed
Chr.10	2365453	2365453	A	A	G	SNP	OS10G0138100	Exonic	CDS	Missense	LOC_Os10g04860	aldehyde oxidase, putative, expressed
Chr.10	2359250	2359250	A	A	G	SNP	OS10G0138100	Intronic	NA	NA	LOC_Os10g04860	aldehyde oxidase, putative, expressed
Chr.10	2359604	2359604	C	C	G	SNP	OS10G0138100	Intronic	NA	NA	LOC_Os10g04860	aldehyde oxidase, putative, expressed
Chr.10	2361644	2361644	A	A	G	SNP	OS10G0138100	Intronic	NA	NA	LOC_Os10g04860	aldehyde oxidase, putative, expressed
Chr.10	2913453	2913453	C	C	G	SNP	OS10G0148400	Exonic	CDS	Silent	LOC_Os10g05780	POT domain containing peptide transporter, putative, expressed
Chr.10	2912918	2912918	T	T	G	SNP	OS10G0148400	Exonic	CDS	Missense	LOC_Os10g05780	POT domain containing peptide transporter, putative, expressed
Chr.10	2913691	2913691	A	A	G	SNP	OS10G0148400	Exonic	CDS	Missense	LOC_Os10g05780	POT domain containing peptide transporter, putative, expressed
Chr.10	3753586	3753586	A	A	G	SNP	OS10G0159300	Exonic	3UTR	NA	LOC_Os10g07160	retrotransposon, putative, centromere-specific, expressed
Chr.10	12015946	12015946	C	C	G	SNP	OS10G0376900	Exonic	5UTR	NA	LOC_Os10g23050	helix-loop-helix DNA-binding protein, putative, expressed
Chr.10	12971195	12971195	A	A	G	SNP	OS10G0390500	Intronic	NA	NA	LOC_Os10g25130	aminotransferase, classes I and II, domain containing protein, expressed
Chr.10	15309202	15309202	C	C	G	SNP	OS10G0430200	Exonic	5UTR	NA	LOC_Os10g29470	dehydrogenase, putative, expressed
Chr.10	15305345	15305345	A	A	G	SNP	OS10G0430200	Intronic	NA	NA	LOC_Os10g29470	dehydrogenase, putative, expressed
Chr.10	15308335	15308335	C	C	G	SNP	OS10G0430200	Intronic	NA	NA	LOC_Os10g29470	dehydrogenase, putative, expressed
Chr.10	21445021	21445021	C	C	G	SNP	OS10G0548100	Exonic	CDS	Missense	LOC_Os10g40040	expressed protein
Chr.10	72011	72029	GGAAGATGCCAAGGACGCA	GGAAGATGCCAAGGACGCA	G	INDEL	OS10G0100500	Exonic	CDS	InFrame	LOC_Os10g01060	protein kinase family protein, putative, expressed
Chr.10	379420	379421	GT	GT	G	INDEL	OS10G0104900	Intronic	NA	NA	LOC_Os10g01570	C-5 cytosine-specific DNA methylase, putative, expressed
Chr.10	7694324	7694325	GT	GT	G	INDEL	OS10G0208500	Exonic	3UTR	NA	LOC_Os10g14180	expressed protein
Chr.10	12016237	12016240	GCTT	GCTT	G	INDEL	OS10G0376900	Exonic	CDS	InFrame	LOC_Os10g23050	helix-loop-helix DNA-binding protein, putative, expressed
Chr.10	15308700	15308701	GC	GC	G	INDEL	OS10G0430200	Intronic	NA	NA	LOC_Os10g29470	dehydrogenase, putative, expressed
Chr.10	18637602	18637622	GATGCATATATTTAACAACTT	GATGCATATATTTAACAACTT	G	INDEL	OS10G0490900	Exonic	3UTR	NA	LOC_Os10g34920	secretory protein, putative, expressed
Chr.10	20894522	20894524	GTT	GTT	G	INDEL	OS10G0536500	Intronic	NA	NA	LOC_Os10g39150	expressed protein
Chr.1	28346106	28346106	T	T	G	SNP	OS01G0687300	Exonic	5UTR	NA	LOC_Os01g49310	expressed protein
Chr.1	604383	604383	T	T	G	SNP	OS01G0111500	Intronic	NA	NA	LOC_Os01g02110	helix-loop-helix DNA-binding domain containing protein, expressed
Chr.1	15171579	15171579	T	T	G	SNP	OS01G0369700	Intronic	NA	NA	LOC_Os01g27210	glutathione S-transferase, putative, expressed
Chr.1	20741575	20741575	T	T	G	SNP	OS01G0552300	Intronic	NA	NA	LOC_Os01g37130	protein phosphatase 2C, putative, expressed
Chr.1	20741930	20741930	T	T	G	SNP	OS01G0552300	Intronic	NA	NA	LOC_Os01g37130	protein phosphatase 2C, putative, expressed
Chr.1	23345274	23345274	T	T	G	SNP	OS01G0595600	Exonic	3UTR	NA	LOC_Os01g41240	hydrolase, alpha/beta fold family domain containing protein, expressed
Chr.1	23675940	23675940	T	T	G	SNP	OS01G0602400	Exonic	5UTR	NA	LOC_Os01g41810	cytochrome P450 72A1, putative, expressed
Chr.1	27178896	27178896	T	T	G	SNP	OS01G0665300	Exonic	3UTR	NA	LOC_Os01g47540	NLI interacting factor-like phosphatase, putative, expressed
Chr.1	30025413	30025413	T	T	G	SNP	OS01G0720400	Exonic	3UTR	NA	LOC_Os01g52230	phosphoethanolamine/phosphocholine phosphatase, putative, expressed
Chr.1	30158469	30158469	T	T	G	SNP	OS01G0723200	Intronic	NA	NA	LOC_Os01g52490	40S ribosomal protein S24, putative, expressed
Chr.1	30158774	30158774	T	T	G	SNP	OS01G0723200	Intronic	NA	NA	LOC_Os01g52490	40S ribosomal protein S24, putative, expressed
Chr.1	34550484	34550484	T	T	G	SNP	OS01G0812800	Exonic	3UTR	NA	LOC_Os01g59730	ribosomal protein L7Ae, putative, expressed
Chr.1	34550896	34550896	T	T	G	SNP	OS01G0812800	Intronic	NA	NA	LOC_Os01g59730	ribosomal protein L7Ae, putative, expressed
Chr.1	34551373	34551373	T	T	G	SNP	OS01G0812800	Intronic	NA	NA	LOC_Os01g59730	ribosomal protein L7Ae, putative, expressed
Chr.1	39549768	39549768	T	T	G	SNP	OS01G0908400	Intronic	NA	NA	LOC_Os01g68030	expressed protein
Chr.1	650417	650417	A	A	G	SNP	OS01G0112500	Intronic	NA	NA	LOC_Os01g02200	armadillo/beta-catenin repeat family protein, putative, expressed
Chr.1	651376	651376	T	T	G	SNP	OS01G0112500	Intronic	NA	NA	LOC_Os01g02200	armadillo/beta-catenin repeat family protein, putative, expressed
Chr.1	744272	744272	A	A	G	SNP	OS01G0113700	Exonic	CDS	Silent	LOC_Os01g02350	receptor-like kinase ARK1AS, putative, expressed
Chr.1	4215441	4215441	A	A	G	SNP	OS01G0180400	Exonic	3UTR	NA	LOC_Os01g08520	DUF581 domain containing protein, expressed
Chr.1	9226643	9226643	T	T	G	SNP	OS01G0268300	Intronic	NA	NA	LOC_Os01g16290	DNA gyrase subunit B, chloroplast/mitochondrial precursor, putative, expressed
Chr.1	9227405	9227405	A	A	G	SNP	OS01G0268300	Intronic	NA	NA	LOC_Os01g16290	DNA gyrase subunit B, chloroplast/mitochondrial precursor, putative, expressed
Chr.1	9227653	9227653	A	A	G	SNP	OS01G0268300	Intronic	NA	NA	LOC_Os01g16290	DNA gyrase subunit B, chloroplast/mitochondrial precursor, putative, expressed
Chr.1	9230860	9230860	A	A	G	SNP	OS01G0268300	Intronic	NA	NA	LOC_Os01g16290	DNA gyrase subunit B, chloroplast/mitochondrial precursor, putative, expressed
Chr.1	9234139	9234139	A	A	G	SNP	OS01G0268300	Intronic	NA	NA	LOC_Os01g16290	DNA gyrase subunit B, chloroplast/mitochondrial precursor, putative, expressed
Chr.1	9234162	9234162	A	A	G	SNP	OS01G0268300	Intronic	NA	NA	LOC_Os01g16290	DNA gyrase subunit B, chloroplast/mitochondrial precursor, putative, expressed
Chr.1	10102427	10102427	A	A	G	SNP	OS01G0282800	Intronic	NA	NA	LOC_Os01g18050	tubulin/FtsZ domain containing protein, putative, expressed
Chr.1	10201418	10201418	T	T	G	SNP	OS01G0284900	Intronic	NA	NA	LOC_Os01g18220	expressed protein
Chr.1	10201815	10201815	C	C	G	SNP	OS01G0284900	Intronic	NA	NA	LOC_Os01g18220	expressed protein
Chr.1	10342624	10342624	A	A	G	SNP	OS01G0287600	Intronic	NA	NA	LOC_Os01g18400	CHIT10 - Chitinase family protein precursor, expressed
Chr.1	15292178	15292178	A	A	G	SNP	OS01G0371500	Intronic	NA	NA	LOC_Os01g27390	glutathione S-transferase, putative, expressed
Chr.1	15292328	15292328	A	A	G	SNP	OS01G0371500	Intronic	NA	NA	LOC_Os01g27390	glutathione S-transferase, putative, expressed

Chr.1	15292808	15292808	T	T	G	SNP	OS01G0371500	Intronic	NA	NA	LOC_Os01g27390	glutathione S-transferase, putative, expressed
Chr.1	15292876	15292876	A	A	G	SNP	OS01G0371500	Intronic	NA	NA	LOC_Os01g27390	glutathione S-transferase, putative, expressed
Chr.1	15292999	15292999	A	A	G	SNP	OS01G0371500	Intronic	NA	NA	LOC_Os01g27390	glutathione S-transferase, putative, expressed
Chr.1	15293097	15293097	A	A	G	SNP	OS01G0371500	Intronic	NA	NA	LOC_Os01g27390	glutathione S-transferase, putative, expressed
Chr.1	20575929	20575929	C	C	G	SNP	OS01G0550000	Exonic	3UTR	NA	LOC_Os01g36920	DEAD-box ATP-dependent RNA helicase, putative, expressed
Chr.1	20574000	20574000	T	T	G	SNP	OS01G0550000	Exonic	5UTR	NA	LOC_Os01g36920	DEAD-box ATP-dependent RNA helicase, putative, expressed
Chr.1	20573559	20573559	A	A	G	SNP	OS01G0550000	Exonic	CDS	Missense	LOC_Os01g36920	DEAD-box ATP-dependent RNA helicase, putative, expressed
Chr.1	20581791	20581791	T	T	G	SNP	OS01G0550100	Exonic	3UTR	NA	LOC_Os01g36930	ubiquitin carboxyl-terminal hydrolase 6, putative, expressed
Chr.1	20600985	20600985	A	A	G	SNP	OS01G0550300	Intronic	NA	NA	LOC_Os01g36950	N-rich protein, putative, expressed
Chr.1	20740909	20740909	A	A	G	SNP	OS01G0552300	Intronic	NA	NA	LOC_Os01g37130	protein phosphatase 2C, putative, expressed
Chr.1	20741104	20741104	A	A	G	SNP	OS01G0552300	Intronic	NA	NA	LOC_Os01g37130	protein phosphatase 2C, putative, expressed
Chr.1	21125423	21125423	A	A	G	SNP	OS01G0558300	Intronic	NA	NA	LOC_Os01g37770	RWD domain containing protein, expressed
Chr.1	21127525	21127525	T	T	G	SNP	OS01G0558300	Intronic	NA	NA	LOC_Os01g37770	RWD domain containing protein, expressed
Chr.1	22139794	22139794	C	C	G	SNP	OS01G0575200	Intronic	NA	NA	LOC_Os01g39330	helix-loop-helix DNA-binding domain containing protein, expressed
Chr.1	22498437	22498437	A	A	G	SNP	OS01G0580800	Intronic	NA	NA	LOC_Os01g39890	transposon protein, putative, unclassified, expressed
Chr.1	29483825	29483825	A	A	G	SNP	OS01G0709500	Exonic	CDS	Missense	LOC_Os01g51290	protein kinase family protein, putative, expressed
Chr.1	31837771	31837771	C	C	G	SNP	OS01G0757900	Exonic	3UTR	NA	LOC_Os01g55310	haloacid dehalogenase-like hydrolase domain-containing protein 1A, putative, expressed
Chr.1	41497988	41497988	A	A	G	SNP	OS01G0944100	Exonic	3UTR	NA	LOC_Os01g71624	expressed protein
Chr.1	41502410	41502410	T	T	G	SNP	OS01G0944100	Exonic	3UTR	NA	LOC_Os01g71624	expressed protein
Chr.1	41499228	41499228	T	T	G	SNP	OS01G0944100	Intronic	NA	NA	LOC_Os01g71624	expressed protein
Chr.1	41500030	41500030	T	T	G	SNP	OS01G0944100	Intronic	NA	NA	LOC_Os01g71624	expressed protein
Chr.1	41500213	41500213	T	T	G	SNP	OS01G0944100	Intronic	NA	NA	LOC_Os01g71624	expressed protein
Chr.1	41500576	41500576	A	A	G	SNP	OS01G0944100	Intronic	NA	NA	LOC_Os01g71624	expressed protein
Chr.1	41502270	41502270	A	A	G	SNP	OS01G0944100	Intronic	NA	NA	LOC_Os01g71624	expressed protein
Chr.1	650507	650508	GA	GA	G	INDEL	OS01G0112500	Intronic	NA	NA	LOC_Os01g02200	armadillo/beta-catenin repeat family protein, putative, expressed
Chr.1	9233054	9233055	GT	GT	G	INDEL	OS01G0268300	Intronic	NA	NA	LOC_Os01g16290	DNA gyrase subunit B, chloroplast/mitochondrial precursor, putative, expressed
Chr.1	10099548	10099551	GTAC	GTAC	G	INDEL	OS01G0282800	Exonic	3UTR	NA	LOC_Os01g18050	tubulin/FtsZ domain containing protein, putative, expressed
Chr.1	21125327	21125331	GAAGT	GAAGT	G	INDEL	OS01G0558300	Intronic	NA	NA	LOC_Os01g37770	RWD domain containing protein, expressed
Chr.1	263790	263790	C	C	G	SNP	OS01G0104800	Intronic	NA	NA	LOC_Os01g01510	sas10/Utp3 family protein, expressed
Chr.1	604624	604624	C	C	G	SNP	OS01G0111500	Intronic	NA	NA	LOC_Os01g02110	helix-loop-helix DNA-binding domain containing protein, expressed
Chr.1	7028252	7028252	C	C	G	SNP	OS01G0227100	Intronic	NA	NA	LOC_Os01g12710	oxidoreductase, short chain dehydrogenase/reductase family domain containing protein, expressed
Chr.1	7864770	7864770	C	C	G	SNP	OS01G0242600	Exonic	CDS	Missense	LOC_Os01g14050	C2 domain containing protein, putative, expressed
Chr.1	24076571	24076571	C	C	G	SNP	OS01G0609300	Exonic	CDS	NA	LOC_Os01g42380	pleiotropic drug resistance protein, putative, expressed
Chr.1	28848045	28848045	C	C	G	SNP	OS01G0697100	Exonic	3UTR	NA	LOC_Os01g50200	UDP-glucoronosyl and UDP-glucosyl transferase domain containing protein, expressed
Chr.1	34550456	34550456	C	C	G	SNP	OS01G0812800	Exonic	3UTR	NA	LOC_Os01g59730	ribosomal protein L7Ae, putative, expressed
Chr.1	36008541	36008541	C	C	G	SNP	OS01G0839300	Intronic	NA	NA	LOC_Os01g62210	50S ribosomal protein L17, putative, expressed
Chr.1	41099909	41099909	C	C	G	SNP	OS01G0936100	Exonic	3UTR	NA	LOC_Os01g71000	protein kinase APK1B, chloroplast precursor, putative, expressed
Chr.1	43075079	43075079	C	C	G	SNP	OS01G0974800	Exonic	5UTR	NA	LOC_Os01g74350	soluble inorganic pyrophosphatase, putative, expressed
Chr.1	265869	265869	A	A	G	SNP	OS01G0104800	Intronic	NA	NA	LOC_Os01g01510	sas10/Utp3 family protein, expressed
Chr.1	603729	603729	A	A	G	SNP	OS01G0111500	Exonic	3UTR	NA	LOC_Os01g02110	helix-loop-helix DNA-binding domain containing protein, expressed
Chr.1	603948	603948	A	A	G	SNP	OS01G0111500	Exonic	3UTR	NA	LOC_Os01g02110	helix-loop-helix DNA-binding domain containing protein, expressed
Chr.1	604073	604073	A	A	G	SNP	OS01G0111500	Exonic	CDS	Silent	LOC_Os01g02110	helix-loop-helix DNA-binding domain containing protein, expressed
Chr.1	604501	604501	A	A	G	SNP	OS01G0111500	Exonic	CDS	Silent	LOC_Os01g02110	helix-loop-helix DNA-binding domain containing protein, expressed
Chr.1	604461	604461	A	A	G	SNP	OS01G0111500	Intronic	NA	NA	LOC_Os01g02110	helix-loop-helix DNA-binding domain containing protein, expressed
Chr.1	604463	604463	A	A	G	SNP	OS01G0111500	Intronic	NA	NA	LOC_Os01g02110	helix-loop-helix DNA-binding domain containing protein, expressed
Chr.1	743281	743281	A	A	G	SNP	OS01G0113700	Exonic	CDS	Missense	LOC_Os01g02350	receptor-like kinase ARK1AS, putative, expressed
Chr.1	2384649	2384649	A	A	G	SNP	OS01G0144100	Intronic	NA	NA	LOC_Os01g05080	thylakoid lumenal protein, putative, expressed
Chr.1	7025047	7025047	A	A	G	SNP	OS01G0227100	Intronic	NA	NA	LOC_Os01g12710	oxidoreductase, short chain dehydrogenase/reductase family domain containing protein, expressed
Chr.1	7365250	7365250	A	A	G	SNP	OS01G0233000	Intronic	NA	NA	LOC_Os01g13210	salt stress root protein RS1, putative, expressed
Chr.1	7558666	7558666	A	A	G	SNP	OS01G0236400	Intronic	NA	NA	LOC_Os01g13530	ABIL2, putative, expressed
Chr.1	9671467	9671467	A	A	G	SNP	OS01G0276200	Intronic	NA	NA	LOC_Os01g16910	outer mitochondrial membrane porin, putative, expressed
Chr.1	9674742	9674742	A	A	G	SNP	OS01G0276200	Intronic	NA	NA	LOC_Os01g16910	outer mitochondrial membrane porin, putative, expressed
Chr.1	15171283	15171283	A	A	G	SNP	OS01G0369700	Exonic	CDS	Silent	LOC_Os01g27210	glutathione S-transferase, putative, expressed
Chr.1	15171065	15171065	A	A	G	SNP	OS01G0369700	Intronic	NA	NA	LOC_Os01g27210	glutathione S-transferase, putative, expressed
Chr.1	20739892	20739892	A	A	G	SNP	OS01G0552300	Intronic	NA	NA	LOC_Os01g37130	protein phosphatase 2C, putative, expressed
Chr.1	20740346	20740346	A	A	G	SNP	OS01G0552300	Intronic	NA	NA	LOC_Os01g37130	protein phosphatase 2C, putative, expressed
Chr.1	20741506	20741506	A	A	G	SNP	OS01G0552300	Intronic	NA	NA	LOC_Os01g37130	protein phosphatase 2C, putative, expressed
Chr.1	20741750	20741750	A	A	G	SNP	OS01G0552300	Intronic	NA	NA	LOC_Os01g37130	protein phosphatase 2C, putative, expressed
Chr.1	20741921	20741921	A	A	G	SNP	OS01G0552300	Intronic	NA	NA	LOC_Os01g37130	protein phosphatase 2C, putative, expressed
Chr.1	20741937	20741937	A	A	G	SNP	OS01G0552300	Intronic	NA	NA	LOC_Os01g37130	protein phosphatase 2C, putative, expressed
Chr.1	20742039	20742039	A	A	G	SNP	OS01G0552300	Intronic	NA	NA	LOC_Os01g37130	protein phosphatase 2C, putative, expressed
Chr.1	21632892	21632892	A	A	G	SNP	OS01G0565900	Exonic	3UTR	NA	LOC_Os01g38510	protein transport protein Sec61 subunit beta, putative, expressed
Chr.1	21633025	21633025	A	A	G	SNP	OS01G0565900	Exonic	3UTR	NA	LOC_Os01g38510	protein transport protein Sec61 subunit beta, putative, expressed
Chr.1	21632268	21632268	A	A	G	SNP	OS01G0565900	Exonic	5UTR	NA	LOC_Os01g38510	protein transport protein Sec61 subunit beta, putative, expressed
Chr.1	21632281	21632281	A	A	G	SNP	OS01G0565900	Exonic	5UTR	NA	LOC_Os01g38510	protein transport protein Sec61 subunit beta, putative, expressed
Chr.1	23343986	23343986	A	A	G	SNP	OS01G0595600	Intronic	NA	NA	LOC_Os01g41240	hydrolase, alpha/beta fold family domain containing protein, expressed
Chr.1	23344004	23344004	A	A	G	SNP	OS01G0595600	Intronic	NA	NA	LOC_Os01g41240	hydrolase, alpha/beta fold family domain containing protein, expressed
Chr.1	23344566	23344566	A	A	G	SNP	OS01G0595600	Intronic	NA	NA	LOC_Os01g41240	hydrolase, alpha/beta fold family domain containing protein, expressed
Chr.1	23673198	23673198	A	A	G	SNP	OS01G0602400	Exonic	3UTR	NA	LOC_Os01g41810	cytochrome P450 72A1, putative, expressed
Chr.1	23673298	23673298	A	A	G	SNP	OS01G0602400	Exonic	CDS	Silent	LOC_Os01g41810	cytochrome P450 72A1, putative, expressed
Chr.1	23673987	23673987	A	A	G	SNP	OS01G0602400	Exonic	CDS	Silent	LOC_Os01g41810	cytochrome P450 72A1, putative, expressed
Chr.1	23673984	23673984	A	A	G	SNP	OS01G0602400	Exonic	CDS	Missense	LOC_Os01g41810	cytochrome P450 72A1, putative, expressed
Chr.1	25236704	25236704	A	A	G	SNP	OS01G0631200	Intronic	NA	NA	LOC_Os01g44050	siroheme synthase, putative, expressed
Chr.1	27181653	27181653	A	A	G	SNP	OS01G0665300	Intronic	NA	NA	LOC_Os01g47540	NLI interacting factor-like phosphatase, putative, expressed
Chr.1	28348436	28348436	A	A	G	SNP	OS01G0687300	Exonic	CDS	Missense	LOC_Os01g49310	expressed protein
Chr.1	30158165	30158165	A	A	G	SNP	OS01G0723200	Exonic	3UTR	NA	LOC_Os01g52490	40S ribosomal protein S24, putative, expressed
Chr.1	30158195	30158195	A	A	G	SNP	OS01G0723200	Exonic	3UTR	NA	LOC_Os01g52490	40S ribosomal protein S24, putative, expressed
Chr.1	30158275	30158275	A	A	G	SNP	OS01G0723200	Intronic	NA	NA	LOC_Os01g52490	40S ribosomal protein S24, putative, expressed
Chr.1	30158811	30158811	A	A	G	SNP	OS01G0723200	Intronic	NA	NA	LOC_Os01g52490	40S ribosomal protein S24, putative, expressed
Chr.1	30158855	30158855	A	A	G	SNP	OS01G0723200	Intronic	NA	NA	LOC_Os01g52490	40S ribosomal protein S24, putative, expressed
Chr.1	30159132	30159132	A	A	G	SNP	OS01G0723200	Intronic	NA	NA	LOC_Os01g52490	40S ribosomal protein S24, putative, expressed
Chr.1	32463482	32463482	A	A	G	SNP	OS01G0769700	Exonic	CDS	Silent	LOC_Os01g56330	TKL_IRAK_CrRLK1L-1.5 - The CrRLK1L-1 subfamily has homology to the CrRLK1L homolog, expressed
Chr.1	33076558	33076558	A	A	G	SNP	OS01G0780800	Intronic	NA	NA	LOC_Os01g57240	expressed protein
Chr.1	34615717	34615717	A	A	G	SNP	OS01G0813900	Intronic	NA	NA	LOC_Os01g59850	GTPase-activating protein, putative, expressed
Chr.1	34617969	34617969	A	A	G	SNP	OS01G0813900	Intronic	NA	NA	LOC_Os01g59850	GTPase-activating protein, putative, expressed

Chr.1	36083087	36083087	A	A	G	SNP	OS01G0840700	Exonic	3UTR	NA	LOC_Os01g62350	60S ribosomal protein L36-2, putative, expressed
Chr.1	38400784	38400784	A	A	G	SNP	OS01G0884300	Intronic	NA	NA	LOC_Os01g66120	No apical meristem protein, putative, expressed
Chr.1	39550010	39550010	A	A	G	SNP	OS01G0908400	Intronic	NA	NA	LOC_Os01g68030	expressed protein
Chr.1	39840080	39840080	A	A	G	SNP	OS01G0914300	Exonic	3UTR	NA	LOC_Os01g68589	LTPL39 - Protease inhibitor/seed storage/LTP family protein precursor, expressed
Chr.1	41384597	41384597	A	A	G	SNP	OS01G0941800	Exonic	3UTR	NA	LOC_Os01g71420	Ser/Thr protein phosphatase family protein, putative, expressed
Chr.1	42978404	42978404	A	A	G	SNP	OS01G0973400	Exonic	CDS	Silent	LOC_Os01g74190	radical SAM enzyme, putative, expressed
Chr.1	42978271	42978271	A	A	G	SNP	OS01G0973400	Intronic	NA	NA	LOC_Os01g74190	radical SAM enzyme, putative, expressed
Chr.1	43047405	43047405	A	A	G	SNP	OS01G0974200	Exonic	CDS	Silent	LOC_Os01g74300	metallothionein, putative, expressed
Chr.1	43073852	43073852	A	A	G	SNP	OS01G0974800	Intronic	NA	NA	LOC_Os01g74350	soluble inorganic pyrophosphatase, putative, expressed
Chr.1	264568	264568	A	A	G	SNP	OS01G0104800	Intronic	NA	NA	LOC_Os01g01510	sas10/Utp3 family protein, expressed
Chr.1	266806	266806	A	A	G	SNP	OS01G0104800	Intronic	NA	NA	LOC_Os01g01510	sas10/Utp3 family protein, expressed
Chr.1	605073	605073	A	A	G	SNP	OS01G0111500	Exonic	CDS	Silent	LOC_Os01g02110	helix-loop-helix DNA-binding domain containing protein, expressed
Chr.1	605298	605298	A	A	G	SNP	OS01G0111500	Exonic	CDS	Silent	LOC_Os01g02110	helix-loop-helix DNA-binding domain containing protein, expressed
Chr.1	651665	651665	C	C	G	SNP	OS01G0112500	Exonic	CDS	Silent	LOC_Os01g02200	armadillo/beta-catenin repeat family protein, putative, expressed
Chr.1	744494	744494	T	T	G	SNP	OS01G0113700	Exonic	CDS	Silent	LOC_Os01g02350	receptor-like kinase ARK1AS, putative, expressed
Chr.1	744496	744496	T	T	G	SNP	OS01G0113700	Exonic	CDS	Missense	LOC_Os01g02350	receptor-like kinase ARK1AS, putative, expressed
Chr.1	3804895	3804895	A	A	G	SNP	OS01G0173900	Exonic	CDS	Silent	LOC_Os01g07870	ABC transporter family protein, putative, expressed
Chr.1	7024543	7024543	C	C	G	SNP	OS01G0227100	Exonic	CDS	Missense	LOC_Os01g12710	oxidoreductase, short chain dehydrogenase/reductase family domain containing protein, expressed
Chr.1	8788029	8788029	A	A	G	SNP	OS01G0261100	Intronic	NA	NA	LOC_Os01g15630	zinc finger, C3HC4 type domain containing protein, expressed
Chr.1	9114595	9114595	A	A	G	SNP	OS01G0266500	Exonic	3UTR	NA	LOC_Os01g16146	phenazine biosynthesis protein, putative, expressed
Chr.1	9114761	9114761	A	A	G	SNP	OS01G0266500	Exonic	CDS	Silent	LOC_Os01g16146	phenazine biosynthesis protein, putative, expressed
Chr.1	11634235	11634235	A	A	G	SNP	OS01G0310100	Intronic	NA	NA	LOC_Os01g20860	phospholipase D. Active site motif family protein, expressed
Chr.1	12365228	12365228	C	C	G	SNP	OS01G0323600	Exonic	CDS	Silent	LOC_Os01g22010	S-adenosylmethionine synthetase, putative, expressed
Chr.1	13904093	13904093	A	A	G	SNP	OS01G0348900	Intronic	NA	NA	LOC_Os01g24710	jacalin-like lectin domain containing protein, expressed
Chr.1	15294295	15294295	A	A	G	SNP	OS01G0371500	Exonic	3UTR	NA	LOC_Os01g27390	glutathione S-transferase, putative, expressed
Chr.1	15294298	15294298	A	A	G	SNP	OS01G0371500	Exonic	3UTR	NA	LOC_Os01g27390	glutathione S-transferase, putative, expressed
Chr.1	15294182	15294182	A	A	G	SNP	OS01G0371500	Exonic	CDS	Silent	LOC_Os01g27390	glutathione S-transferase, putative, expressed
Chr.1	15293948	15293948	T	T	G	SNP	OS01G0371500	Exonic	CDS	Missense	LOC_Os01g27390	glutathione S-transferase, putative, expressed
Chr.1	15294136	15294136	C	C	G	SNP	OS01G0371500	Exonic	CDS	Missense	LOC_Os01g27390	glutathione S-transferase, putative, expressed
Chr.1	15293658	15293658	A	A	G	SNP	OS01G0371500	Intronic	NA	NA	LOC_Os01g27390	glutathione S-transferase, putative, expressed
Chr.1	20589402	20589402	A	A	G	SNP	OS01G0550100	Intronic	NA	NA	LOC_Os01g36930	ubiquitin carboxyl-terminal hydrolase 6, putative, expressed
Chr.1	20601113	20601113	A	A	G	SNP	OS01G0550300	Intronic	NA	NA	LOC_Os01g36950	N-rich protein, putative, expressed
Chr.1	20601193	20601193	A	A	G	SNP	OS01G0550300	Intronic	NA	NA	LOC_Os01g36950	N-rich protein, putative, expressed
Chr.1	20739002	20739002	T	T	G	SNP	OS01G0552300	Intronic	NA	NA	LOC_Os01g37130	protein phosphatase 2C, putative, expressed
Chr.1	20739042	20739042	A	A	G	SNP	OS01G0552300	Intronic	NA	NA	LOC_Os01g37130	protein phosphatase 2C, putative, expressed
Chr.1	21040847	21040847	C	C	G	SNP	OS01G0556700	Exonic	3UTR	NA	LOC_Os01g37590	peptide transporter PTR2, putative, expressed
Chr.1	23343760	23343760	C	C	G	SNP	OS01G0595600	Exonic	CDS	Silent	LOC_Os01g41240	hydrolase, alpha/beta fold family domain containing protein, expressed
Chr.1	23343648	23343648	A	A	G	SNP	OS01G0595600	Exonic	CDS	Missense	LOC_Os01g41240	hydrolase, alpha/beta fold family domain containing protein, expressed
Chr.1	23345120	23345120	A	A	G	SNP	OS01G0595600	Exonic	CDS	Missense	LOC_Os01g41240	hydrolase, alpha/beta fold family domain containing protein, expressed
Chr.1	23345126	23345126	A	A	G	SNP	OS01G0595600	Exonic	CDS	Missense	LOC_Os01g41240	hydrolase, alpha/beta fold family domain containing protein, expressed
Chr.1	23343564	23343564	T	T	G	SNP	OS01G0595600	Intronic	NA	NA	LOC_Os01g41240	hydrolase, alpha/beta fold family domain containing protein, expressed
Chr.1	23344042	23344042	A	A	G	SNP	OS01G0595600	Intronic	NA	NA	LOC_Os01g41240	hydrolase, alpha/beta fold family domain containing protein, expressed
Chr.1	23344438	23344438	A	A	G	SNP	OS01G0595600	Intronic	NA	NA	LOC_Os01g41240	hydrolase, alpha/beta fold family domain containing protein, expressed
Chr.1	24786317	24786317	A	A	G	SNP	OS01G0621600	Exonic	CDS	Silent	LOC_Os01g43350	protein kinase family protein, putative, expressed
Chr.1	27083698	27083698	A	A	G	SNP	OS01G0663300	Intronic	NA	NA	LOC_Os01g47400	OsMan01 - Endo-Beta-Mannanase, expressed
Chr.1	28348202	28348202	A	A	G	SNP	OS01G0687300	Exonic	CDS	Missense	LOC_Os01g49310	expressed protein
Chr.1	28848156	28848156	A	A	G	SNP	OS01G0697100	Exonic	3UTR	NA	LOC_Os01g50200	UDP-glucoronosyl and UDP-glucosyl transferase domain containing protein, expressed
Chr.1	29458865	29458865	T	T	G	SNP	OS01G0708900	Exonic	5UTR	NA	LOC_Os01g51250	mitochondrial carrier protein, putative, expressed
Chr.1	29458866	29458866	T	T	G	SNP	OS01G0708900	Exonic	5UTR	NA	LOC_Os01g51250	mitochondrial carrier protein, putative, expressed
Chr.1	29961427	29961427	T	T	G	SNP	OS01G0719000	Intronic	NA	NA	LOC_Os01g52100	DUF581 domain containing protein, expressed
Chr.1	30263103	30263103	A	A	G	SNP	OS01G0725800	Exonic	3UTR	NA	LOC_Os01g52640	suppressor of phythchrome A, putative, expressed
Chr.1	31728411	31728411	A	A	G	SNP	OS01G0756200	Exonic	CDS	Missense	LOC_Os01g55150	bZIP transcription factor domain containing protein, expressed
Chr.1	31837215	31837215	C	C	G	SNP	OS01G0757900	Exonic	3UTR	NA	LOC_Os01g55310	haloacid dehalogenase-like hydrolase domain-containing protein 1A, putative, expressed
Chr.1	32259184	32259184	A	A	G	SNP	OS01G0765600	Exonic	3UTR	NA	LOC_Os01g56030	EF hand family protein, putative, expressed
Chr.1	33410929	33410929	A	A	G	SNP	OS01G0787600	Intronic	NA	NA	LOC_Os01g57770	OsPOP4 - Putative Prolyl Oligopeptidase homologue, expressed
Chr.1	34550631	34550631	C	C	G	SNP	OS01G0812800	Exonic	3UTR	NA	LOC_Os01g59730	ribosomal protein L7Ae, putative, expressed
Chr.1	34552584	34552584	T	T	G	SNP	OS01G0812800	Exonic	5UTR	NA	LOC_Os01g59730	ribosomal protein L7Ae, putative, expressed
Chr.1	34552590	34552590	A	A	G	SNP	OS01G0812800	Exonic	5UTR	NA	LOC_Os01g59730	ribosomal protein L7Ae, putative, expressed
Chr.1	34552565	34552565	A	A	G	SNP	OS01G0812800	Exonic	CDS	Silent	LOC_Os01g59730	ribosomal protein L7Ae, putative, expressed
Chr.1	34552567	34552567	C	C	G	SNP	OS01G0812800	Exonic	CDS	Missense	LOC_Os01g59730	ribosomal protein L7Ae, putative, expressed
Chr.1	34552473	34552473	A	A	G	SNP	OS01G0812800	Intronic	NA	NA	LOC_Os01g59730	ribosomal protein L7Ae, putative, expressed
Chr.1	34552494	34552494	A	A	G	SNP	OS01G0812800	Intronic	NA	NA	LOC_Os01g59730	ribosomal protein L7Ae, putative, expressed
Chr.1	42025853	42025853	C	C	G	SNP	OS01G0954400	Exonic	CDS	Silent	LOC_Os01g72480	zinc finger, C3HC4 type domain containing protein, expressed
Chr.1	42219590	42219590	A	A	G	SNP	OS01G0957900	Intronic	NA	NA	LOC_Os01g72780	expressed protein
Chr.1	42977253	42977253	A	A	G	SNP	OS01G0973400	Exonic	3UTR	NA	LOC_Os01g74190	radical SAM enzyme, putative, expressed
Chr.1	266345	266373	GTTATAATATGTAAGCTTGAAATGATTTA	GTTATAATATGTAAGCTTGAAATGATTTA	G	INDEL	OS01G0104800	Intronic	NA	NA	LOC_Os01g01510	sas10/Utp3 family protein, expressed
Chr.1	603721	603722	GT	GT	G	INDEL	OS01G0111500	Exonic	3UTR	NA	LOC_Os01g02110	helix-loop-helix DNA-binding domain containing protein, expressed
Chr.1	7364481	7364482	GA	GA	G	INDEL	OS01G0233000	Intronic	NA	NA	LOC_Os01g13210	salt stress root protein RS1, putative, expressed
Chr.1	7863348	7863349	GA	GA	G	INDEL	OS01G0242600	Intronic	NA	NA	LOC_Os01g14050	C2 domain containing protein, putative, expressed
Chr.1	13904074	13904081	GTTTGAAC	GTTTGAAC	G	INDEL	OS01G0348900	Intronic	NA	NA	LOC_Os01g24710	jacalin-like lectin domain containing protein, expressed
Chr.1	15292531	15292532	GA	GA	G	INDEL	OS01G0371500	Intronic	NA	NA	LOC_Os01g27390	glutathione S-transferase, putative, expressed
Chr.1	20571283	20571286	GCCC	GCCC	G	INDEL	OS01G0550000	Intronic	NA	NA	LOC_Os01g36920	DEAD-box ATP-dependent RNA helicase, putative, expressed
Chr.1	21074019	21074031	GAAAAAATTTTGA	GAAAAAATTTTGA	G	INDEL	OS01G0557500	Intronic	NA	NA	LOC_Os01g37690	sodium/calcium exchanger protein, putative, expressed
Chr.1	21076183	21076184	GA	GA	G	INDEL	OS01G0557500	Intronic	NA	NA	LOC_Os01g37690	sodium/calcium exchanger protein, putative, expressed
Chr.1	23345093	23345102	GCGGCCGCCA	GCGGCCGCCA	G	INDEL	OS01G0595600	Exonic	CDS	InFrame	LOC_Os01g41240	hydrolase, alpha/beta fold family domain containing protein, expressed
Chr.1	23344086	23344087	GC	GC	G	INDEL	OS01G0595600	Intronic	NA	NA	LOC_Os01g41240	hydrolase, alpha/beta fold family domain containing protein, expressed
Chr.1	24114421	24114423	GGT	GGT	G	INDEL	OS01G0609900	Intronic	NA	NA	LOC_Os01g42410	pleiotropic drug resistance protein, putative, expressed
Chr.1	25235738	25235739	GT	GT	G	INDEL	OS01G0631200	Intronic	NA	NA	LOC_Os01g44050	siroheme synthase, putative, expressed
Chr.1	25600125	25600127	GTT	GTT	G	INDEL	OS01G0638000	Exonic	3UTR	NA	LOC_Os01g45110	anthocyanin 3-O-beta-glucosyltransferase, putative, expressed
Chr.1	30181132	30181133	GT	GT	G	INDEL	OS01G0723600	Intronic	NA	NA	LOC_Os01g52530	phosphoribosyl transferase, putative, expressed
Chr.1	30952103	30952104	GC	GC	G	INDEL	OS01G0741200	Intronic	NA	NA	LOC_Os01g53840	protein kinase family protein, putative, expressed
Chr.1	31851350	31851353	GCTC	GCTC	G	INDEL	OS01G0758200	Exonic	CDS	InFrame	LOC_Os01g55340	dof zinc finger domain containing protein, putative, expressed
Chr.1	32259065	32259073	GGCGTGCAC	GGCGTGCAC	G	INDEL	OS01G0765600	Exonic	3UTR	NA	LOC_Os01g56030	EF hand family protein, putative, expressed
Chr.1	34550620	34550625	GAAGAT	GAAGAT	G	INDEL	OS01G0812800	Exonic	3UTR	NA	LOC_Os01g59730	ribosomal protein L7Ae, putative, expressed
Chr7	18762653	18762655	GAA	GAA	G	INDEL	OS07G0499300	Exonic	3UTR	NA	LOC_Os07g31599	expressed protein

Chr2	22297490	22297492	GAA	G	GA	INDEL	OS02G0579600	Intronic	NA	NA	LOC_Os02g36924	OsMADS27 - MADS-box family gene with MIKCc type-box, expressed
Chr.4	618021	618023	GAA	G	GA	INDEL	OS04G0110600	Intronic	NA	NA	LOC_Os04g02000	zinc finger family protein, putative, expressed
Chr.5	15077846	15077848	GAA	G	GA	INDEL	OS05G0324700	Exonic	3UTR	NA	LOC_Os05g25920	expressed protein
Chr10	15404087	15404089	GAA	G	GA	INDEL	OS10G0431900	Intronic	NA	NA	LOC_Os10g29620	tyrosine protein kinase domain containing protein, putative, expressed
Chr7	2200782	2200782	G	GAA	GA	INDEL	OS07G0143000	Intronic	NA	NA	LOC_Os07g05000	oxidoreductase, aldo/keto reductase family protein, putative, expressed
Chr.5	3730783	3730783	G	GAA	GA	INDEL	OS05G0163700	Exonic	3UTR	NA	LOC_Os05g07090	acyl-coenzyme A dehydrogenase, mitochondrial precursor, putative, expressed
Chr.8	20425948	20425948	G	GAA	GA	INDEL	OS08G0425200	Intronic	NA	NA	LOC_Os08g32930	expressed protein
Chr7	21147584	21147584	G	GAA	GA	INDEL	OS07G0537900	Intronic	NA	NA	LOC_Os07g35340	TKL_IRAK_DUF26-lc.14 - DUF26 kinases have homology to DUF26 containing loci, expressed
Chr.1	27108713	27108713	G	GAA	GA	INDEL	OS01G0663800	Intronic	NA	NA	LOC_Os01g47430	protein of unknown function DUF1296 domain containing protein, expressed
Chr7	26773685	26773685	G	GAT	GA	INDEL	OS07G0642800	Intronic	NA	NA	LOC_Os07g44830	proline-rich family protein, putative, expressed
Chr7	27727725	27727725	G	G	GA	INDEL	OS07G0658400	Intronic	NA	NA	LOC_Os07g46460	ferredoxin-dependent glutamate synthase, chloroplast precursor, putative, expressed
Chr7	27731741	27731741	G	G	GA	INDEL	OS07G0658400	Intronic	NA	NA	LOC_Os07g46460	ferredoxin-dependent glutamate synthase, chloroplast precursor, putative, expressed
Chr7	27737679	27737679	G	G	GA	INDEL	OS07G0658400	Intronic	NA	NA	LOC_Os07g46460	ferredoxin-dependent glutamate synthase, chloroplast precursor, putative, expressed
Chr7	18337411	18337411	G	G	GA	INDEL	OS07G0492000	Exonic	5UTR	NA	LOC_Os07g30970	nucleoside diphosphate kinase, putative, expressed
Chr.9	6388158	6388158	G	G	GA	INDEL	OS09G0286600	Exonic	3UTR	NA	LOC_Os09g11460	AP2 domain containing protein, expressed
Chr.8	1563011	1563011	G	G	GA	INDEL	OS08G0127100	Intronic	NA	NA	LOC_Os08g03350	amino acid transporter, putative, expressed
Chr.8	3281856	3281856	G	G	GA	INDEL	OS08G0156600	Intronic	NA	NA	LOC_Os08g06010	transporter, major facilitator family, putative, expressed
Chr.8	3423232	3423232	G	G	GA	INDEL	OS08G0158500	Intronic	NA	NA	LOC_Os08g06200	stomatin-like protein 2, putative, expressed
Chr.6	31076284	31076284	G	G	GA	INDEL	OS06G0729300	Intronic	NA	NA	LOC_Os06g51310	PAZ domain containing protein, putative, expressed
Chr.6	27144353	27144353	G	G	GA	INDEL	OS06G0659300	Intronic	NA	NA	LOC_Os06g44890	villin protein, putative, expressed
Chr.6	23112413	23112413	G	G	GA	INDEL	OS06G0589300	Intronic	NA	NA	LOC_Os06g38950	ABC transporter, ATP-binding protein, putative, expressed
Chr.5	20630059	20630059	G	G	GA	INDEL	OS05G0420600	Exonic	5UTR	NA	LOC_Os05g34770	cytochrome c, putative, expressed
Chr.5	27536214	27536214	G	G	GA	INDEL	OS05G0553800	Intronic	NA	NA	LOC_Os05g48030	anti-silencing protein, ASF1-like domain containing protein, expressed
Chr.5	25229793	25229793	G	G	GA	INDEL	OS05G0509500	Exonic	3UTR	NA	LOC_Os05g43390	signal recognition particle 54 kDa protein, putative, expressed
Chr.5	23868914	23868914	G	G	GA	INDEL	OS05G0485300	Intronic	NA	NA	LOC_Os05g40700	transmembrane protein 56, putative, expressed
Chr.5	2116238	2116238	G	G	GA	INDEL	OS05G0136200	Intronic	NA	NA	LOC_Os05g04550	CAMK_KIN1/SNF1/Nim1_like.3 - CAMK includes calcium/calmodulin depedent protein kinases, expressed
Chr.5	1994940	1994940	G	G	GA	INDEL	OS05G0134200	Intronic	NA	NA	LOC_Os05g04360	protein phosphatase 2C, putative, expressed
Chr.5	13839127	13839127	G	G	GA	INDEL	OS05G0305900	Intronic	NA	NA	LOC_Os05g24010	receptor protein kinase-like, putative, expressed
Chr.4	1150460	1150460	G	G	GA	INDEL	OS04G0119500	Intronic	NA	NA	LOC_Os04g02910	membrane related protein CP5, putative, expressed
Chr.4	22209481	22209481	G	G	GA	INDEL	OS04G0445400	Exonic	3UTR	NA	LOC_Os04g36780	expressed protein
Chr.4	24886005	24886005	G	G	GA	INDEL	OS04G0497600	Intronic	NA	NA	LOC_Os04g42010	RNA binding protein, putative, expressed
Chr.4	27237219	27237219	G	G	GA	INDEL	OS04G0543900	Intronic	NA	NA	LOC_Os04g45970	glutamate dehydrogenase protein, putative, expressed
Chr.4	31111260	31111260	G	G	GA	INDEL	OS04G0613700	Intronic	NA	NA	LOC_Os04g52370	UTP--glucose-1-phosphate uridylyltransferase, putative, expressed
Chr.4	30288611	30288611	G	G	GA	INDEL	OS04G0600200	Intronic	NA	NA	LOC_Os04g51150	transposon protein, putative, unclassified, expressed
Chr.4	31274624	31274624	G	G	GA	INDEL	OS04G0616300	Exonic	3UTR	NA	LOC_Os04g52600	SHR5-receptor-like kinase, putative, expressed
Chr.4	31439778	31439778	G	G	GA	INDEL	OS04G0619000	Exonic	3UTR	NA	LOC_Os04g52810	no apical meristem protein, putative, expressed
Chr.4	31463252	31463252	G	G	GA	INDEL	OS04G0619400	Exonic	3UTR	NA	LOC_Os04g52840	tyrosine protein kinase domain containing protein, putative, expressed
Chr.4	22624072	22624072	G	G	GA	INDEL	OS04G0453233	Intronic	NA	NA	LOC_Os04g38026	transporter family protein, putative, expressed
Chr.4	29134588	29134588	G	G	GA	INDEL	OS04G0578000	Exonic	5UTR	NA	LOC_Os04g48850	aminotransferase, classes I and II, domain containing protein, expressed
Chr.3	35869498	35869498	G	G	GA	INDEL	OS03G0851900	Intronic	NA	NA	LOC_Os03g63500	ATPase, AFG1 family domain containing protein, expressed
Chr.3	32566614	32566614	G	G	GA	INDEL	OS03G0784700	Exonic	3UTR	NA	LOC_Os03g57120	ferredoxin--NADP reductase, chloroplast precursor, putative, expressed
Chr.3	29144229	29144229	G	G	GA	INDEL	OS03G0719300	Intronic	NA	NA	LOC_Os03g51000	3,4-dihydroxy-2-butanone kinase, putative, expressed
Chr.3	27461228	27461228	G	G	GA	INDEL	OS03G0688200	Intronic	NA	NA	LOC_Os03g48260	expressed protein
Chr.3	23372555	23372555	G	G	GA	INDEL	OS03G0616700	Intronic	NA	NA	LOC_Os03g42050	exportin-7-A, putative, expressed
Chr.3	16288138	16288138	G	G	GA	INDEL	OS03G0401100	Intronic	NA	NA	LOC_Os03g28300	protein kinase domain containing protein, expressed
Chr.3	16287806	16287806	G	G	GA	INDEL	OS03G0401100	Intronic	NA	NA	LOC_Os03g28300	protein kinase domain containing protein, expressed
Chr.3	16287666	16287666	G	G	GA	INDEL	OS03G0401100	Intronic	NA	NA	LOC_Os03g28300	protein kinase domain containing protein, expressed
Chr.3	1243770	1243770	G	G	GA	INDEL	OS03G0122300	Intronic	NA	NA	LOC_Os03g03034	flavonol synthase/flavanone 3-hydroxylase, putative, expressed
Chr.3	1737491	1737491	G	G	GA	INDEL	OS03G0130700	Exonic	3UTR	NA	LOC_Os03g03860	expressed protein
Chr.3	10573209	10573209	G	G	GA	INDEL	OS03G0300600	Exonic	5UTR	NA	LOC_Os03g18870	heat shock protein DnaJ, putative, expressed
Chr.3	24470813	24470813	G	G	GA	INDEL	OS03G0638400	Intronic	NA	NA	LOC_Os03g43750	retrotransposon, putative, centromere-specific, expressed
Chr.3	26397026	26397026	G	G	GA	INDEL	OS03G0669100	Intronic	NA	NA	LOC_Os03g46640	deoxyuridine 5-triphosphate nucleotidohydrolase, putative, expressed
Chr.3	28329976	28329976	G	G	GA	INDEL	OS03G0704700	Exonic	5UTR	NA	LOC_Os03g49770	oxysterol-binding protein, putative, expressed
Chr.2	767399	767399	G	G	GA	INDEL	OS02G0114200	Exonic	3UTR	NA	LOC_Os02g02320	OsSCP6 - Putative Serine Carboxypeptidase homologue, expressed
Chr.2	9927149	9927149	G	G	GA	INDEL	OS02G0272900	Intronic	NA	NA	LOC_Os02g17304	resistance protein, putative, expressed
Chr.2	14950063	14950063	G	G	GA	INDEL	OS02G0452500	Intronic	NA	NA	LOC_Os02g25580	Sec1 family transport protein, putative, expressed
Chr.2	24738362	24738362	G	G	GA	INDEL	OS02G0621600	Exonic	3UTR	NA	LOC_Os02g40820	expressed protein
Chr.12	22600453	22600453	G	G	GA	INDEL	OS12G0555500	Intronic	NA	NA	LOC_Os12g36880	pathogenesis-related Bet v I family protein, putative, expressed
Chr.12	25519297	25519297	G	G	GA	INDEL	OS12G0604700	Intronic	NA	NA	LOC_Os12g41180	LSTK-1-like kinase, putative, expressed
Chr.11	4247750	4247750	G	G	GA	INDEL	OS11G0183900	Intronic	NA	NA	LOC_Os11g08100	eukaryotic aspartyl protease domain containing protein, expressed
Chr.11	4248550	4248550	G	G	GA	INDEL	OS11G0183900	Intronic	NA	NA	LOC_Os11g08100	eukaryotic aspartyl protease domain containing protein, expressed
Chr.11	14049688	14049688	G	G	GA	INDEL	OS11G0434000	Intronic	NA	NA	LOC_Os11g24630	magnesium-dependent phosphatase 1, putative, expressed
Chr.11	17354576	17354576	G	G	GA	INDEL	OS11G0490900	Intronic	NA	NA	LOC_Os11g29870	WRKY72, expressed
Chr.11	7128329	7128329	G	G	GA	INDEL	OS11G0234200	Intronic	NA	NA	LOC_Os11g12650	PHD-finger domain containing protein, putative, expressed
Chr.10	21697193	21697193	G	G	GA	INDEL	OS10G0552800	Exonic	3UTR	NA	LOC_Os10g40530	LTPL146 - Protease inhibitor/seed storage/LTP family protein precursor, expressed
Chr.1	650240	650240	G	G	GA	INDEL	OS01G0112500	Intronic	NA	NA	LOC_Os01g02200	armadillo/beta-catenin repeat family protein, putative, expressed
Chr.1	603841	603841	G	G	GA	INDEL	OS01G0111500	Exonic	3UTR	NA	LOC_Os01g02110	helix-loop-helix DNA-binding domain containing protein, expressed
Chr.1	648344	648344	G	G	GA	INDEL	OS01G0112500	Exonic	3UTR	NA	LOC_Os01g02200	armadillo/beta-catenin repeat family protein, putative, expressed
Chr.1	7865597	7865597	G	G	GA	INDEL	OS01G0242600	Intronic	NA	NA	LOC_Os01g14050	C2 domain containing protein, putative, expressed
Chr.1	9671782	9671782	G	G	GA	INDEL	OS01G0276200	Intronic	NA	NA	LOC_Os01g16910	outer mitochondrial membrane porin, putative, expressed
Chr.1	10493395	10493395	G	G	GA	INDEL	OS01G0290000	Intronic	NA	NA	LOC_Os01g18630	aspartic proteinase oryzasin-1 precursor, putative, expressed
Chr.1	29831642	29831642	G	G	GA	INDEL	OS01G0716800	Intronic	NA	NA	LOC_Os01g51890	endonuclease/exonuclease/phosphatase family domain containing protein, expressed
Chr.1	38264827	38264827	G	G	GA	INDEL	OS01G0881500	Intronic	NA	NA	LOC_Os01g65900	chitin-inducible gibberellin-responsive protein, putative, expressed
Chr.1	40504054	40504054	G	G	GA	INDEL	OS01G0924900	Intronic	NA	NA	LOC_Os01g70020	DEK C terminal domain containing protein, expressed
Chr1	8429441	8429442	GA	G	GAA	INDEL	OS01G0254100	Intronic	NA	NA	LOC_Os01g15020	lissencephaly type-1-like homology motif, putative, expressed
Chr1	10201694	10201695	GA	G	GAA	INDEL	OS01G0284900	Intronic	NA	NA	LOC_Os01g18220	expressed protein
Chr3	15467958	15467959	GA	G	GAA	INDEL	OS03G0387900	Exonic	5UTR	NA	LOC_Os03g27019	expressed protein
Chr1	22497672	22497673	GA	G	GAA	INDEL	OS01G0580800	Intronic	NA	NA	LOC_Os01g39890	transposon protein, putative, unclassified, expressed
Chr.6	2262537	2262537	G	GA	GAA	INDEL	OS06G0143100	Intronic	NA	NA	LOC_Os06g05120	expressed protein
Chr.1	9134953	9134953	G	GA	GAA	INDEL	OS01G0266800	Intronic	NA	NA	LOC_Os01g16170	PQ loop repeat domain containing protein, expressed
Chr.5	27633179	27633179	G	GA	GAA	INDEL	OS05G0555600	Intronic	NA	NA	LOC_Os05g48200	glutamate synthase, chloroplast precursor, putative, expressed
Chr.3	28252478	28252478	G	GA	GAA	INDEL	OS03G0703100	Intronic	NA	NA	LOC_Os03g49610	Os3bglu8 - beta-glucosidase, exo-beta-glucansase, high similarity to Os3bglu7, expressed
Chr.1	29844176	29844176	G	GA	GAA	INDEL	OS01G0717000	Intronic	NA	NA	LOC_Os01g51920	phosphotransferase, putative, expressed
Chr.1	31850715	31850715	G	GA	GAA	INDEL	OS01G0758200	Exonic	3UTR	NA	LOC_Os01g55340	dof zinc finger domain containing protein, putative, expressed
Chr.3	36002446	36002446	G	GA	GAA	INDEL	OS03G0854800	Intronic	NA	NA	LOC_Os03g63770	RCD1, putative, expressed

Chr.6	1555714	1555714	G	G	GAA	INDEL	OS06G0129400	Intronic	NA	NA	LOC_Os06g03860	uncharacterized membrane protein, putative, expressed
Chr.5	25229903	25229903	G	G	GAA	INDEL	OS05G0509500	Exonic	3UTR	NA	LOC_Os05g43390	signal recognition particle 54 kDa protein, putative, expressed
Chr.4	28296613	28296613	G	G	GAA	INDEL	OS04G0564700	Exonic	3UTR	NA	LOC_Os04g47700	expressed protein
Chr.4	6478997	6478997	G	G	GAA	INDEL	OS04G0194500	Intronic	NA	NA	LOC_Os04g11820	white-brown complex homolog protein, putative, expressed
Chr.4	28303761	28303761	G	G	GAA	INDEL	OS04G0564700	Intronic	NA	NA	LOC_Os04g47700	expressed protein
Chr.3	26384816	26384816	G	G	GAA	INDEL	OS03G0669000	Intronic	NA	NA	LOC_Os03g46610	DEAD-box ATP-dependent RNA helicase, putative, expressed
Chr.11	27399654	27399654	G	G	GAA	INDEL	OS11G0678000	Exonic	5UTR	NA	LOC_Os11g45280	protein kinase family protein, putative, expressed
Chr.1	24787483	24787483	G	G	GAA	INDEL	OS01G0621600	Intronic	NA	NA	LOC_Os01g43350	protein kinase family protein, putative, expressed
Chr.1	25682768	25682768	G	G	GAA	INDEL	OS01G0639600	Exonic	3UTR	NA	LOC_Os01g45250	DUF1645 domain containing protein, putative, expressed
Chr1	27341083	27341085	GAA	G	GAAA	INDEL	OS01G0668100	Exonic	3UTR	NA	LOC_Os01g47780	fasciclin domain containing protein, expressed
Chr.9	17903186	17903186	G	G	GAAAAAGAA	INDEL	OS09G0470500	Exonic	5UTR	NA	LOC_Os09g29460	homeobox associated leucine zipper, putative, expressed
Chr.9	7281955	7281955	G	G	GAAAAT	INDEL	OS09G0298500	Exonic	CDS	FrameShift	LOC_Os09g12720	zinc finger, C3HC4 type domain containing protein, expressed
Chr.1	43116109	43116109	G	G	GACAGC	INDEL	OS01G0975900	Exonic	3UTR	NA	LOC_Os01g74450	aquaporin protein, putative, expressed
Chr2	82071	82073	AAG	A	GAG	INDEL	OS02G0101500	Intronic	NA	NA	LOC_Os02g01150	erythronate-4-phosphate dehydrogenase domain containing protein, expressed
Chr.8	3243208	3243208	G	G	GAGA	INDEL	OS08G0155900	Exonic	CDS	InFrame	LOC_Os08g05960	expressed protein
Chr.1	43075192	43075192	G	G	GAGAGGC	INDEL	OS01G0974800	Exonic	5UTR	NA	LOC_Os01g74350	soluble inorganic pyrophosphatase, putative, expressed
Chr.2	35099173	35099173	G	G	GAGC	INDEL	OS02G0817900	Exonic	CDS	InFrame	LOC_Os02g57290	cytochrome P450, putative, expressed
Chr.9	10252155	10252155	G	GAGCCGCTC	GAGTCGCTC	INDEL	OS09G0337300	Exonic	CDS	NA	LOC_Os09g16760	ankyrin repeat family protein, putative, expressed
Chr.6	17068209	17068209	G	G	GAT	INDEL	OS06G0493100	Intronic	NA	NA	LOC_Os06g29730	RALFL28 - Rapid ALkalinization Factor RALF family protein precursor, expressed
Chr.4	23409674	23409674	G	G	GAT	INDEL	OS04G0468600	Exonic	CDS	FrameShift	LOC_Os04g39320	expressed protein
Chr.1	39690673	39690673	G	G	GAT	INDEL	OS01G0911100	Intronic	NA	NA	LOC_Os01g68320	DEAD-box ATP-dependent RNA helicase 30, putative, expressed
Chr.4	16005150	16005150	G	G	GATA	INDEL	OS04G0339400	Exonic	3UTR	NA	LOC_Os04g27060	oxidoreductase, aldo/keto reductase family protein, putative, expressed
Chr.8	3244516	3244516	G	G	GATAT	INDEL	OS08G0156000	Exonic	5UTR	NA	LOC_Os08g05970	expressed protein
Chr7	1788910	1788910	G	G	GATC	INDEL	OS07G0134000	Exonic	3UTR	NA	LOC_Os07g04180	amino acid transporter, putative, expressed
Chr1	43075279	43075299	GATCTATCTATCTATCTATCT	G	GATCTATCTATCT	INDEL	OS01G0974800	Exonic	5UTR	NA	LOC_Os01g74350	soluble inorganic pyrophosphatase, putative, expressed
Chr.4	31442955	31442955	G	G	GATGGGTC	INDEL	OS04G0619000	Exonic	3UTR	NA	LOC_Os04g52810	no apical meristem protein, putative, expressed
Chr.11	19677814	19677814	G	G	GATGT	INDEL	OS11G0539200	Exonic	3UTR	NA	LOC_Os11g33270	glycosyl hydrolases family 16, putative, expressed
Chr.4	24301587	24301587	G	G	GATT	INDEL	OS04G0486500	Exonic	3UTR	NA	LOC_Os04g40940	mitotic spindle checkpoint protein MAD2, putative, expressed
Chr.4	6479893	6479893	G	G	GATTT	INDEL	OS04G0194500	Intronic	NA	NA	LOC_Os04g11820	white-brown complex homolog protein, putative, expressed
Chr.2	3868224	3868224	G	GCC	GC	INDEL	OS02G0171100	Exonic	5UTR	NA	LOC_Os02g07490	glyceraldehyde-3-phosphate dehydrogenase, putative, expressed
Chr7	23059233	23059233	G	GCC	GC	INDEL	OS07G0571300	Exonic	CDS	FrameShift	LOC_Os07g38370	expressed protein
Chr.3	6136665	6136665	G	G	GC	INDEL	OS03G0216700	Intronic	NA	NA	LOC_Os03g11734	MATE efflux protein, putative, expressed
Chr.3	1717901	1717901	G	G	GC	INDEL	OS03G0130400	Intronic	NA	NA	LOC_Os03g03820	adenylate kinase, putative, expressed
Chr.3	17292701	17292701	G	G	GC	INDEL	OS03G0416500	Exonic	5UTR	NA	LOC_Os03g30300	6-phosphogluconolactonase, putative, expressed
Chr.2	32569969	32569969	G	G	GC	INDEL	OS02G0771700	Exonic	5UTR	NA	LOC_Os02g53200	glucan endo-1,3-beta-glucosidase precursor, putative, expressed
Chr.5	27121991	27121991	G	G	GCAA	INDEL	OS05G0546400	Exonic	5UTR	NA	LOC_Os05g46840	proline-rich protein, putative, expressed
Chr.6	1415608	1415608	G	G	GCAACAGCAA	INDEL	OS06G0126500	Exonic	CDS	InFrame	LOC_Os06g03640	BAG domain containing protein, expressed
Chr.3	29145058	29145058	G	G	GCACA	INDEL	OS03G0719300	Intronic	NA	NA	LOC_Os03g51000	3,4-dihydroxy-2-butanone kinase, putative, expressed
Chr.5	2027824	2027824	G	GCACA	GCACACA	INDEL	OS05G0134700	Exonic	5UTR	NA	LOC_Os05g04410	peroxidase precursor, putative, expressed
Chr.1	21127810	21127810	G	G	GCATCAT	INDEL	OS01G0558300	Intronic	NA	NA	LOC_Os01g37770	RWD domain containing protein, expressed
Chr7	5128746	5128746	G	G	GCCA	INDEL	OS07G0194800	Exonic	3UTR	NA	LOC_Os07g09670	galactosyltransferase family protein, putative, expressed
Chr.4	32385931	32385933	GCC	G	GCCCCC	INDEL	OS04G0636800	Intronic	NA	NA	LOC_Os04g54430	immediate-early protein RSP40, putative, expressed
Chr.8	26464593	26464593	G	G	GCCT	INDEL	OS08G0531300	Exonic	CDS	InFrame	LOC_Os08g41910	Sua5/YciO/YrdC/YwlC family protein, putative, expressed
Chr.6	21602545	21602545	G	G	GCCT	INDEL	OS06G0562600	Exonic	5UTR	NA	LOC_Os06g36700	T-complex protein, putative, expressed
Chr.1	29960846	29960846	G	G	GCCT	INDEL	OS01G0719000	Exonic	5UTR	NA	LOC_Os01g52100	DUF581 domain containing protein, expressed
Chr.1	605351	605351	G	G	GCGC	INDEL	OS01G0111500	Exonic	CDS	InFrame	LOC_Os01g02110	helix-loop-helix DNA-binding domain containing protein, expressed
Chr.1	4852464	4852464	G	G	GCGCCGC	INDEL	OS01G0191100	Exonic	5UTR	NA	LOC_Os01g09510	60S acidic ribosomal protein, putative, expressed
Chr.10	2914012	2914012	G	G	GCGT	INDEL	OS10G0148400	Exonic	CDS	InFrame	LOC_Os10g05780	POT domain containing peptide transporter, putative, expressed
Chr.5	27122515	27122515	G	G	GCTC	INDEL	OS05G0546400	Exonic	CDS	InFrame	LOC_Os05g46840	proline-rich protein, putative, expressed
Chr.1	30026403	30026403	G	G	GCTCT	INDEL	OS01G0720400	Exonic	5UTR	NA	LOC_Os01g52230	phosphoethanolamine/phosphocholine phosphatase, putative, expressed
Chr.8	26390076	26390076	G	GCT	GCTCTCT	INDEL	OS08G0530000	Intronic	NA	NA	LOC_Os08g41790	phosphoribulokinase/Uridine kinase family protein, expressed
Chr.1	29830523	29830523	G	GCTCT	GCTCTCT	INDEL	OS01G0716800	Intronic	NA	NA	LOC_Os01g51890	endonuclease/exonuclease/phosphatase family domain containing protein, expressed
Chr.5	21191674	21191674	G	G	GCTCTCTCT	INDEL	OS05G0431700	Exonic	5UTR	NA	LOC_Os05g35650	peptide transporter PTR2, putative, expressed
Chr7	6968889	6968901	GCTCTCTCTCTCT	G	GCTCTCTCTCT	INDEL	OS07G0225300	Exonic	3UTR	NA	LOC_Os07g12340	NAC domain-containing protein 67, putative, expressed
Chr.6	8848601	8848601	G	G	GCTGCTT	INDEL	OS06G0266800	Exonic	CDS	InFrame	LOC_Os06g15620	GASR7 - Gibberellin-regulated GASA/GAST/Snakin family protein precursor, expressed
Chr.1	24115168	24115168	G	G	GCTTCCT	INDEL	OS01G0609900	Exonic	5UTR	NA	LOC_Os01g42410	pleiotropic drug resistance protein, putative, expressed
Chr1	38262409	38262413	GGAGA	G	GGA	INDEL	OS01G0881500	Intronic	NA	NA	LOC_Os01g65900	chitin-inducible gibberellin-responsive protein, putative, expressed
Chr.3	16754030	16754030	G	G	GGA	INDEL	OS03G0407900	Exonic	CDS	FrameShift	LOC_Os03g29410	tyrosine protein kinase domain containing protein, putative, expressed
Chr.2	4294288	4294288	G	G	GGA	INDEL	OS02G0177800	Intronic	NA	NA	LOC_Os02g08120	calmodulin binding protein, putative, expressed
Chr.1	26895333	26895333	G	G	GGA	INDEL	OS01G0660300	Intronic	NA	NA	LOC_Os01g47080	pyruvate kinase, putative, expressed
Chr.6	23737216	23737216	G	G	GGAA	INDEL	OS06G0601100	Exonic	CDS	InFrame	LOC_Os06g39912	expressed protein
Chr.4	31466279	31466279	G	G	GGAA	INDEL	OS04G0619400	Exonic	5UTR	NA	LOC_Os04g52840	tyrosine protein kinase domain containing protein, putative, expressed
Chr.3	2748664	2748664	G	G	GGAGCA	INDEL	OS03G0149300	Intronic	NA	NA	LOC_Os03g05530	nodulin, putative, expressed
Chr.4	318657	318657	G	C	GGAGGGAGTATATTAC	INDEL	OS04G0104900	Intronic	NA	NA	LOC_Os04g01470	O-methyltransferase, putative, expressed
Chr.2	22299430	22299430	G	G	GGATAAGCAGCTTGGAACGCTCA	INDEL	OS02G0579600	Intronic	NA	NA	LOC_Os02g36924	OsMADS27 - MADS-box family gene with MIKCc type-box, expressed
Chr.2	5155690	5155690	G	G	GGATC	INDEL	OS02G0192500	Intronic	NA	NA	LOC_Os02g09930	CSLA1 - cellulose synthase-like family A; mannan synthase, expressed
Chr.8	6168686	6168686	G	G	GGCA	INDEL	OS08G0205800	Exonic	CDS	NA	LOC_Os08g10500	expressed protein
Chr.3	11667811	11667811	G	G	GGCGGCGGCA	INDEL	OS03G0322200	Exonic	CDS	InFrame	LOC_Os03g20600	expressed protein
Chr.5	29492094	29492101	GGGACGGC	G	GGGACGGCGGACGGC	INDEL	OS05G0591900	Exonic	5UTR	NA	LOC_Os05g51420	hypersensitive-induced response protein, putative, expressed
Chr.6	20901584	20901584	G	G	GGGTGCA	INDEL	OS06G0551400	Exonic	5UTR	NA	LOC_Os06g35814	ras-related protein, putative, expressed
Chr.5	29227367	29227377	GGTGTGTGTGT	G	GGT	INDEL	OS05G0586600	Exonic	3UTR	NA	LOC_Os05g50930	RNA polymerase sigma factor, putative, expressed
Chr7	4332188	4332188	G	G	GGT	INDEL	OS07G0182100	Intronic	NA	NA	LOC_Os07g08430	indole-3-glycerol phosphate lyase, chloroplast precursor, putative, expressed
Chr7	18841148	18841148	G	G	GGTA	INDEL	OS07G0500300	Intronic	NA	NA	LOC_Os07g31720	GTPase activating protein, putative, expressed
Chr10	378507	378508	GT	G	GT	INDEL	OS10G0104900	Intronic	NA	NA	LOC_Os10g01570	C-5 cytosine-specific DNA methylase, putative, expressed
Chr8	3293846	3293849	GTAT	G	GT	INDEL	OS08G0156800	Intronic	NA	NA	LOC_Os08g06040	60S ribosomal protein L34, putative, expressed
Chr9	3658433	3658435	GTT	G	GT	INDEL	OS09G0248100	Exonic	3UTR	NA	LOC_Os09g07350	fasciclin-like arabinogalactan protein 8 precursor, putative, expressed
Chr6	5421300	5421302	GTT	G	GT	INDEL	OS06G0207000	Intronic	NA	NA	LOC_Os06g10520	pantothenate kinase, putative, expressed
Chr.4	11277331	11277333	GTT	G	GT	INDEL	OS04G0270100	Intronic	NA	NA	LOC_Os04g20220	elongation factor Tu, putative, expressed
Chr10	15673826	15673828	GTT	G	GT	INDEL	OS10G0437600	Intronic	NA	NA	LOC_Os10g30156	starch synthase, putative, expressed
Chr7	20217410	20217413	GTTT	G	GT	INDEL	OS07G0522500	Intronic	NA	NA	LOC_Os07g33780	pleiotropic drug resistance protein 5, putative, expressed
Chr9	21678585	21678587	GTT	G	GT	INDEL	OS09G0548200	Intronic	NA	NA	LOC_Os09g37600	lysM domain-containing GPI-anchored protein precursor, putative, expressed
Chr2	29608332	29608334	GTT	G	GT	INDEL	OS02G0714200	Intronic	NA	NA	LOC_Os02g48360	pyrophosphate--fructose 6-phosphate 1-phosphotransferase subunit alpha, putative, expressed
Chr3	35162481	35162484	GTTT	G	GT	INDEL	OS03G0836800	Intronic	NA	NA	LOC_Os03g62060	hydrolase, putative, expressed
Chr.2	4454558	4454558	G	GTT	GT	INDEL	OS02G0180000	Exonic	3UTR	NA	LOC_Os02g08364	protein phosphatase 2C, putative, expressed
Chr.8	14414707	14414707	G	GTT	GT	INDEL	OS08G0327400	Intronic	NA	NA	LOC_Os08g23810	enoyl-acyl-carrier-protein reductase NADH, chloroplast precursor, expressed

Chr.6	23737581	23737581	G	GTT	GT	INDEL	OS06G0601100	Exonic	3UTR	NA	LOC_Os06g39912	expressed protein
Chr.5	29488145	29488145	G	GTT	GT	INDEL	OS05G0591900	Intronic	NA	NA	LOC_Os05g51420	hypersensitive-induced response protein, putative, expressed
Chr.3	7063090	7063090	G	T	GT	INDEL	OS03G0233000	Exonic	3UTR	NA	LOC_Os03g13050	E2F-related protein, putative, expressed
Chr7	22429134	22429134	G	G	GT	INDEL	OS07G0561800	Exonic	3UTR	NA	LOC_Os07g37454	urate anion exchanger, putative, expressed
Chr.9	3656484	3656484	G	G	GT	INDEL	OS09G0248100	Intronic	NA	NA	LOC_Os09g07350	fasciclin-like arabinogalactan protein 8 precursor, putative, expressed
Chr.8	3423834	3423834	G	G	GT	INDEL	OS08G0158500	Intronic	NA	NA	LOC_Os08g06200	stomatin-like protein 2, putative, expressed
Chr.8	3463572	3463572	G	G	GT	INDEL	OS08G0159500	Intronic	NA	NA	LOC_Os08g06280	LSD1 zinc finger domain containing protein, expressed
Chr.8	26420293	26420293	G	G	GT	INDEL	OS08G0530400	Intronic	NA	NA	LOC_Os08g41830	sulfite oxidase, putative, expressed
Chr.8	26421276	26421276	G	G	GT	INDEL	OS08G0530400	Intronic	NA	NA	LOC_Os08g41830	sulfite oxidase, putative, expressed
Chr.8	12267676	12267676	G	G	GT	INDEL	OS08G0299400	Intronic	NA	NA	LOC_Os08g20420	MGD2, putative, expressed
Chr.6	31074251	31074251	G	G	GT	INDEL	OS06G0729300	Intronic	NA	NA	LOC_Os06g51310	PAZ domain containing protein, putative, expressed
Chr.6	23856670	23856670	G	G	GT	INDEL	OS06G0603000	Intronic	NA	NA	LOC_Os06g40080	heme oxygenase 1, putative, expressed
Chr.6	23469037	23469037	G	G	GT	INDEL	OS06G0596300	Intronic	NA	NA	LOC_Os06g39520	myristoyl-acyl carrier protein thioesterase, chloroplast precursor, putative, expressed
Chr.6	23468623	23468623	G	G	GT	INDEL	OS06G0596300	Intronic	NA	NA	LOC_Os06g39520	myristoyl-acyl carrier protein thioesterase, chloroplast precursor, putative, expressed
Chr.6	23467572	23467572	G	G	GT	INDEL	OS06G0596300	Intronic	NA	NA	LOC_Os06g39520	myristoyl-acyl carrier protein thioesterase, chloroplast precursor, putative, expressed
Chr.6	23468812	23468812	G	G	GT	INDEL	OS06G0596300	Intronic	NA	NA	LOC_Os06g39520	myristoyl-acyl carrier protein thioesterase, chloroplast precursor, putative, expressed
Chr.6	26304471	26304471	G	G	GT	INDEL	OS06G0644600	Intronic	NA	NA	LOC_Os06g43690	WD repeat-containing protein, putative, expressed
Chr.5	14619239	14619239	G	G	GT	INDEL	OS05G0316200	Intronic	NA	NA	LOC_Os05g25210	expressed protein
Chr.5	23422840	23422840	G	G	GT	INDEL	OS05G0476200	Intronic	NA	NA	LOC_Os05g39850	MCM3 - Putative minichromosome maintenance MCM complex subunit 3, expressed
Chr.5	7163884	7163884	G	G	GT	INDEL	OS05G0215600	Exonic	3UTR	NA	LOC_Os05g12474	expressed protein
Chr.4	24883007	24883007	G	G	GT	INDEL	OS04G0497600	Exonic	3UTR	NA	LOC_Os04g42010	RNA binding protein, putative, expressed
Chr.4	27508381	27508381	G	G	GT	INDEL	OS04G0549600	Intronic	NA	NA	LOC_Os04g46390	chaperone protein dnaJ, putative, expressed
Chr.4	31177080	31177080	G	G	GT	INDEL	OS04G0614600	Intronic	NA	NA	LOC_Os04g52450	aminotransferase, putative, expressed
Chr.4	6481048	6481048	G	G	GT	INDEL	OS04G0194500	Intronic	NA	NA	LOC_Os04g11820	white-brown complex homolog protein, putative, expressed
Chr.4	25194430	25194430	G	G	GT	INDEL	OS04G0504800	Exonic	3UTR	NA	LOC_Os04g42600	polyadenylate-binding protein, putative, expressed
Chr.4	27192640	27192640	G	G	GT	INDEL	OS04G0542900	Intronic	NA	NA	LOC_Os04g45910	placental protein 11 precursor, putative, expressed
Chr.4	29043808	29043808	G	G	GT	INDEL	OS04G0576500	Intronic	NA	NA	LOC_Os04g48710	expressed protein
Chr.4	5321621	5321621	G	G	GT	INDEL	OS04G0178300	Intronic	NA	NA	LOC_Os04g09900	ent-kaurene synthase, chloroplast precursor, putative, expressed
Chr.4	31463352	31463352	G	G	GT	INDEL	OS04G0619400	Exonic	3UTR	NA	LOC_Os04g52840	tyrosine protein kinase domain containing protein, putative, expressed
Chr.3	27491741	27491741	G	G	GT	INDEL	OS03G0689100	Intronic	NA	NA	LOC_Os03g48300	histidine acid phosphatase, putative, expressed
Chr.3	24815749	24815749	G	G	GT	INDEL	OS03G0643300	Intronic	NA	NA	LOC_Os03g44150	aminotransferase, putative, expressed
Chr.3	24815417	24815417	G	G	GT	INDEL	OS03G0643300	Intronic	NA	NA	LOC_Os03g44150	aminotransferase, putative, expressed
Chr.3	16593429	16593429	G	G	GT	INDEL	OS03G0405500	Intronic	NA	NA	LOC_Os03g29190	PDI, putative, expressed
Chr.3	1243553	1243553	G	G	GT	INDEL	OS03G0122300	Intronic	NA	NA	LOC_Os03g03034	flavonol synthase/flavanone 3-hydroxylase, putative, expressed
Chr.3	9014821	9014821	G	G	GT	INDEL	OS03G0269900	Intronic	NA	NA	LOC_Os03g16334	fringe-related protein, putative, expressed
Chr.3	9879742	9879742	G	G	GT	INDEL	OS03G0286500	Intronic	NA	NA	LOC_Os03g17760	RNA recognition motif containing protein, putative, expressed
Chr.2	5251998	5251998	G	G	GT	INDEL	OS02G0194200	Intronic	NA	NA	LOC_Os02g10080	zinc finger C-x8-C-x5-C-x3-H type family protein, expressed
Chr.11	23185086	23185086	G	G	GT	INDEL	OS11G0602600	Intronic	NA	NA	LOC_Os11g38959	40S ribosomal protein S9-2, putative, expressed
Chr.11	26510896	26510896	G	G	GT	INDEL	OS11G0660500	Exonic	3UTR	NA	LOC_Os11g43900	translationally-controlled tumor protein, putative, expressed
Chr.10	12869182	12869182	G	G	GT	INDEL	OS10G0388900	Intronic	NA	NA	LOC_Os10g25000	expressed protein
Chr.10	2368568	2368568	G	G	GT	INDEL	OS10G0138100	Exonic	3UTR	NA	LOC_Os10g04860	aldehyde oxidase, putative, expressed
Chr.10	69365	69365	G	G	GT	INDEL	OS10G0100500	Intronic	NA	NA	LOC_Os10g01060	protein kinase family protein, putative, expressed
Chr.1	266783	266783	G	G	GT	INDEL	OS01G0104800	Intronic	NA	NA	LOC_Os01g01510	sas10/Utp3 family protein, expressed
Chr.1	7025752	7025752	G	G	GT	INDEL	OS01G0227100	Intronic	NA	NA	LOC_Os01g12710	oxidoreductase, short chain dehydrogenase/reductase family domain containing protein, expressed
Chr.1	26890031	26890031	G	G	GT	INDEL	OS01G0660300	Intronic	NA	NA	LOC_Os01g47080	pyruvate kinase, putative, expressed
Chr.1	28347046	28347046	G	G	GT	INDEL	OS01G0687300	Intronic	NA	NA	LOC_Os01g49310	expressed protein
Chr.1	41729278	41729278	G	G	GT	INDEL	OS01G0948600	Exonic	CDS	NA	LOC_Os01g72009	expressed protein
Chr.1	9226269	9226269	G	G	GT	INDEL	OS01G0268300	Intronic	NA	NA	LOC_Os01g16290	DNA gyrase subunit B, chloroplast/mitochondrial precursor, putative, expressed
Chr.1	7029403	7029403	G	G	GT	INDEL	OS01G0227100	Intronic	NA	NA	LOC_Os01g12710	oxidoreductase, short chain dehydrogenase/reductase family domain containing protein, expressed
Chr7	8076756	8076756	G	G	GTA	INDEL	OS07G0245100	Exonic	5UTR	NA	LOC_Os07g14150	cytidine deaminase, putative, expressed
Chr7	27726447	27726447	G	G	GTA	INDEL	OS07G0658400	Intronic	NA	NA	LOC_Os07g46460	ferredoxin-dependent glutamate synthase, chloroplast precursor, putative, expressed
Chr.9	22427499	22427499	G	G	GTA	INDEL	OS09G0564200	Exonic	3UTR	NA	LOC_Os09g39070	thiol protease SEN102 precursor, putative, expressed
Chr.3	29452014	29452014	G	G	GTA	INDEL	OS03G0724600	Intronic	NA	NA	LOC_Os03g51470	expressed protein
Chr.3	29140103	29140103	G	G	GTA	INDEL	OS03G0719300	Intronic	NA	NA	LOC_Os03g51000	3,4-dihydroxy-2-butanone kinase, putative, expressed
Chr2	5156704	5156710	GTACTAC	G	GTAC	INDEL	OS02G0192500	Intronic	NA	NA	LOC_Os02g09930	CSLA1 - cellulose synthase-like family A; mannan synthase, expressed
Chr7	27729288	27729288	G	G	GTATGCTTGCT	INDEL	OS07G0658400	Intronic	NA	NA	LOC_Os07g46460	ferredoxin-dependent glutamate synthase, chloroplast precursor, putative, expressed
Chr.3	10393825	10393825	G	G	GTC	INDEL	OS03G0297100	Intronic	NA	NA	LOC_Os03g18570	40S ribosomal protein S7, putative, expressed
Chr.6	21601448	21601448	G	G	GTCA	INDEL	OS06G0562600	Intronic	NA	NA	LOC_Os06g36700	T-complex protein, putative, expressed
Chr.4	5325687	5325687	G	G	GTCCCCGGC	INDEL	OS04G0178300	Intronic	NA	NA	LOC_Os04g09900	ent-kaurene synthase, chloroplast precursor, putative, expressed
Chr.4	22021999	22021999	G	GTCCTATTT	GTCCTATAT	INDEL	OS04G0442200	Intronic	NA	NA	LOC_Os04g36062	expressed protein
Chr.6	7230573	7230573	G	G	GTCGA	INDEL	OS06G0239200	Exonic	3UTR	NA	LOC_Os06g13190	expressed protein
Chr.8	8329124	8329124	G	G	GTCT	INDEL	OS08G0237000	Exonic	3UTR	NA	LOC_Os08g13920	glycosyl hydrolases family 16, putative, expressed
Chr.12	23420563	23420563	G	G	GTGATTT	INDEL	OS12G0569200	Exonic	CDS	InFrame	LOC_Os12g38140	expressed protein
Chr.1	23345072	23345072	G	G	GTGC	INDEL	OS01G0595600	Exonic	CDS	InFrame	LOC_Os01g41240	hydrolase, alpha/beta fold family domain containing protein, expressed
Chr10	19607586	19607586	G	A	GTGCTAATGATGGAT	INDEL	OS10G0510000	Intronic	NA	NA	LOC_Os10g36650	actin, putative, expressed
Chr.5	22306317	22306317	G	G	GTGGGAAC	INDEL	OS05G0454500	Exonic	5UTR	NA	LOC_Os05g38040	expressed protein
Chr.3	29117565	29117565	G	G	GTGT	INDEL	OS03G0719000	Intronic	NA	NA	LOC_Os03g50970	retrotransposon protein, putative, Ty1-copia subclass, expressed
Chr.5	27122494	27122494	G	G	GTGTC	INDEL	OS05G0546400	Exonic	CDS	FrameShift	LOC_Os05g46840	proline-rich protein, putative, expressed
Chr1	10200780	10200782	CTT	C	GTT	INDEL	OS01G0284900	Exonic	3UTR	NA	LOC_Os01g18220	expressed protein
Chr1	24113791	24113792	GT	G	GTT	INDEL	OS01G0609900	Intronic	NA	NA	LOC_Os01g42410	pleiotropic drug resistance protein, putative, expressed
Chr6	26658701	26658702	GT	G	GTT	INDEL	OS06G0651100	Intronic	NA	NA	LOC_Os06g44180	NAD dependent epimerase/dehydratase family protein, putative, expressed
Chr6	30823435	30823438	GTTT	G	GTT	INDEL	OS06G0725000	Exonic	3UTR	NA	LOC_Os06g50930	rhodanese-like domain containing protein, putative, expressed
Chr1	37506002	37506003	GT	G	GTT	INDEL	OS01G0866000	Exonic	3UTR	NA	LOC_Os01g64620	zinc finger, C3HC4 type domain containing protein, expressed
Chr.6	5680288	5680288	G	GT	GTT	INDEL	OS06G0211200	Intronic	NA	NA	LOC_Os06g10880	bZIP transcription factor, putative, expressed
Chr.1	4905728	4905728	G	T	GTT	INDEL	OS01G0191700	Exonic	5UTR	NA	LOC_Os01g09570	6-phosphofructokinase, putative, expressed
Chr7	16898433	16898433	G	G	GTT	INDEL	OS07G0471300	Intronic	NA	NA	LOC_Os07g28850	retrotransposon protein, putative, unclassified, expressed
Chr7	27724076	27724076	G	G	GTT	INDEL	OS07G0658400	Intronic	NA	NA	LOC_Os07g46460	ferredoxin-dependent glutamate synthase, chloroplast precursor, putative, expressed
Chr.4	24324995	24324995	G	G	GTT	INDEL	OS04G0486900	Intronic	NA	NA	LOC_Os04g40980	Divergent PAP2 family domain containing protein, expressed
Chr.3	538947	538947	G	G	GTT	INDEL	OS03G0109300	Intronic	NA	NA	LOC_Os03g01880	possible lysine decarboxylase domain containing protein, expressed
Chr.3	23086597	23086597	G	G	GTT	INDEL	OS03G0611200	Intronic	NA	NA	LOC_Os03g41510	oxidoreductase, aldo/keto reductase family protein, putative, expressed
Chr.2	767954	767954	G	G	GTT	INDEL	OS02G0114200	Intronic	NA	NA	LOC_Os02g02320	OsSCP6 - Putative Serine Carboxypeptidase homologue, expressed
Chr.1	39830611	39830611	G	G	GTT	INDEL	OS01G0914000	Intronic	NA	NA	LOC_Os01g68570	expressed protein
Chr.3	28595508	28595508	G	G	GTTATTGGAC	INDEL	OS03G0709000	Intronic	NA	NA	LOC_Os03g50130	microsomal glutathione S-transferase 3, putative, expressed
Chr.2	9973639	9973639	G	G	GTTC	INDEL	OS02G0273700	Intronic	NA	NA	LOC_Os02g17350	VHS and GAT domain containing protein, expressed
Chr.5	22303590	22303590	G	G	GTTGCTGGC	INDEL	OS05G0454500	Exonic	3UTR	NA	LOC_Os05g38040	expressed protein

Chr7	16897585	16897585	G	GT	GTTT	INDEL	OS07G0471300	Intronic	NA	NA	LOC_Os07g28850	retrotransposon protein, putative, unclassified, expressed
Chr.2	31408220	31408220	G	G	GTTT	INDEL	OS02G0747500	Intronic	NA	NA	LOC_Os02g51290	HVA22, putative, expressed
Chr.8	8329239	8329239	G	G	GTTTA	INDEL	OS08G0237000	Exonic	3UTR	NA	LOC_Os08g13920	glycosyl hydrolases family 16, putative, expressed
Chr.11	24652138	24652138	G	GTT	GTTTT	INDEL	OS11G0629400	Exonic	3UTR	NA	LOC_Os11g41150	nitrilase-associated protein, putative, expressed
Chr.5	3757410	3757410	G	A	T	SNP	OS05G0163900	Intronic	NA	NA	LOC_Os05g07120	basic helix-loop-helix, putative, expressed
Chr.4	16004183	16004183	G	A	T	SNP	OS04G0339400	Intronic	NA	NA	LOC_Os04g27060	oxidoreductase, aldo/keto reductase family protein, putative, expressed
Chr.11	22279256	22279256	G	A	T	SNP	OS11G0587600	Intronic	NA	NA	LOC_Os11g37700	pleiotropic drug resistance protein, putative, expressed
Chr.1	24114432	24114432	C	A	T	SNP	OS01G0609900	Intronic	NA	NA	LOC_Os01g42410	pleiotropic drug resistance protein, putative, expressed
Chr.3	33216033	33216033	G	A	T	SNP	OS03G0797400	Intronic	NA	NA	LOC_Os03g58300	indole-3-glycerol phosphate lyase, chloroplast precursor, putative, expressed
Chr.9	18709025	18709026	TA	AA	T	INDEL	OS09G0484800	Intronic	NA	NA	LOC_Os09g31120	pirin, putative, expressed
Chr.3	20793679	20793679	A	AAT	T	INDEL	OS03G0571900	Intronic	NA	NA	LOC_Os03g37490	MATE efflux family protein, putative, expressed
Chr.12	9918331	9918331	A	C	T	SNP	OS12G0271700	Intronic	NA	NA	LOC_Os12g17320	polyprenyl synthetase, putative, expressed
Chr.11	10865224	10865224	G	C	T	SNP	OS11G0295900	Intronic	NA	NA	LOC_Os11g19060	BABY BOOM 1, putative, expressed
Chr.4	33254771	33254771	C	CA	T	INDEL	OS04G0652700	Intronic	NA	NA	LOC_Os04g55850	nuclease PA3, putative, expressed
Chr.8	2256045	2256045	C	CACTT	T	INDEL	OS08G0140300	Exonic	5UTR	NA	LOC_Os08g04540	decarboxylase, putative, expressed
Chr.5	16269726	16269726	C	CTTT	T	INDEL	OS05G0345500	Intronic	NA	NA	LOC_Os05g27880	BBF1 - 2 Bric-a-Brac, Tramtrack, Broad Complex BTB domains with a F5/8 type C discoidin domain, expressed
Chr7	21246274	21246274	C	G	T	SNP	OS07G0539400	Exonic	CDS	Missense	LOC_Os07g35520	glucan endo-1,3-beta-glucosidase precursor, putative, expressed
Chr7	28781248	28781250	TAA	TA	T	INDEL	OS07G0679500	Intronic	NA	NA	LOC_Os07g48180	bZIP transcription factor domain containing protein, expressed
Chr.2	35082780	35082782	TAA	TA	T	INDEL	OS02G0817700	Intronic	NA	NA	LOC_Os02g57260	3-ketoacyl-CoA thiolase, peroxisomal precursor, putative, expressed
Chr.1	41005497	41005499	TAA	TA	T	INDEL	OS01G0934800	Intronic	NA	NA	LOC_Os01g70850	esterase, putative, expressed
Chr7	2646535	2646536	TA	TAA	T	INDEL	OS07G0150700	Intronic	NA	NA	LOC_Os07g05620	CAMK_KIN1/SNF1/Nim1_like.28 - CAMK includes calcium/calmodulin depedent protein kinases, expressed
Chr.1	22497731	22497732	TA	TAAA	T	INDEL	OS01G0580800	Intronic	NA	NA	LOC_Os01g39890	transposon protein, putative, unclassified, expressed
Chr.4	27235179	27235180	TA	TAAA	T	INDEL	OS04G0543900	Exonic	3UTR	NA	LOC_Os04g45970	glutamate dehydrogenase protein, putative, expressed
Chr7	27724892	27724894	TAA	TAAA	T	INDEL	OS07G0658400	Intronic	NA	NA	LOC_Os07g46460	ferredoxin-dependent glutamate synthase, chloroplast precursor, putative, expressed
Chr.4	33126214	33126217	TCCC	TCC	T	INDEL	OS04G0650000	Intronic	NA	NA	LOC_Os04g55650	oryzain alpha chain precursor, putative, expressed
Chr.9	14030822	14030824	TGA	TGAGA	T	INDEL	OS09G0401000	Exonic	3UTR	NA	LOC_Os09g23620	MYB family transcription factor, putative, expressed
Chr.2	35098896	35098908	TGAGAGAGAGAGA	TGAGAGAGA	T	INDEL	OS02G0817900	Intronic	NA	NA	LOC_Os02g57290	cytochrome P450, putative, expressed
Chr.4	5847266	5847266	G	G	T	SNP	OS04G0186400	Exonic	5UTR	NA	LOC_Os04g10750	inorganic phosphate transporter, putative, expressed
Chr.4	5847027	5847027	G	G	T	SNP	OS04G0186400	Exonic	5UTR	NA	LOC_Os04g10750	inorganic phosphate transporter, putative, expressed
Chr.4	5847005	5847005	G	G	T	SNP	OS04G0186400	Exonic	5UTR	NA	LOC_Os04g10750	inorganic phosphate transporter, putative, expressed
Chr.4	5846843	5846843	G	G	T	SNP	OS04G0186400	Exonic	5UTR	NA	LOC_Os04g10750	inorganic phosphate transporter, putative, expressed
Chr.4	5847182	5847182	A	A	T	SNP	OS04G0186400	Exonic	5UTR	NA	LOC_Os04g10750	inorganic phosphate transporter, putative, expressed
Chr.4	5846618	5846618	A	A	T	SNP	OS04G0186400	Exonic	5UTR	NA	LOC_Os04g10750	inorganic phosphate transporter, putative, expressed
Chr.4	5849011	5849011	G	G	T	SNP	OS04G0186400	Exonic	CDS	Missense	LOC_Os04g10750	inorganic phosphate transporter, putative, expressed
Chr.4	5849264	5849265	TA	TA	T	INDEL	OS04G0186400	Exonic	3UTR	NA	LOC_Os04g10750	inorganic phosphate transporter, putative, expressed
Chr.4	5846743	5846744	TA	TA	T	INDEL	OS04G0186400	Exonic	5UTR	NA	LOC_Os04g10750	inorganic phosphate transporter, putative, expressed
Chr.4	5846758	5846759	TG	TG	T	INDEL	OS04G0186400	Exonic	5UTR	NA	LOC_Os04g10750	inorganic phosphate transporter, putative, expressed
Chr7	8076174	8076174	G	G	T	SNP	OS07G0245100	Exonic	5UTR	NA	LOC_Os07g14150	cytidine deaminase, putative, expressed
Chr7	10232335	10232335	G	G	T	SNP	OS07G0274700	Intronic	NA	NA	LOC_Os07g17330	B12D protein, putative, expressed
Chr7	18841826	18841826	G	G	T	SNP	OS07G0500300	Intronic	NA	NA	LOC_Os07g31720	GTPase activating protein, putative, expressed
Chr7	21381139	21381139	G	G	T	SNP	OS07G0541400	Exonic	3UTR	NA	LOC_Os07g35690	TKL_IRAK_DUF26-lc.3 - DUF26 kinases have homology to DUF26 containing loci, expressed
Chr7	21381142	21381142	G	G	T	SNP	OS07G0541400	Exonic	3UTR	NA	LOC_Os07g35690	TKL_IRAK_DUF26-lc.3 - DUF26 kinases have homology to DUF26 containing loci, expressed
Chr7	27172762	27172762	G	G	T	SNP	OS07G0650100	Exonic	CDS	Silent	LOC_Os07g45550	expressed protein
Chr7	29172077	29172077	G	G	T	SNP	OS07G0686600	Exonic	3UTR	NA	LOC_Os07g48710	VQ domain containing protein, putative, expressed
Chr7	7108725	7108725	C	C	T	SNP	OS07G0227200	Exonic	3UTR	NA	LOC_Os07g12470	expressed protein
Chr7	8967372	8967372	C	C	T	SNP	OS07G0258400	Intronic	NA	NA	LOC_Os07g15460	metal transporter Nramp6, putative, expressed
Chr7	15408062	15408062	C	C	T	SNP	OS07G0448800	Intronic	NA	NA	LOC_Os07g26690	aquaporin protein, putative, expressed
Chr7	16503503	16503503	C	C	T	SNP	OS07G0466000	Intronic	NA	NA	LOC_Os07g28250	lipase class 3 family protein, putative, expressed
Chr7	17412788	17412788	C	C	T	SNP	OS07G0479300	Intronic	NA	NA	LOC_Os07g29620	OsSCP39 - Putative Serine Carboxypeptidase homologue, expressed
Chr7	18840845	18840845	C	C	T	SNP	OS07G0500300	Intronic	NA	NA	LOC_Os07g31720	GTPase activating protein, putative, expressed
Chr7	18841082	18841082	C	C	T	SNP	OS07G0500300	Intronic	NA	NA	LOC_Os07g31720	GTPase activating protein, putative, expressed
Chr7	18841597	18841597	C	C	T	SNP	OS07G0500300	Intronic	NA	NA	LOC_Os07g31720	GTPase activating protein, putative, expressed
Chr7	18842077	18842077	C	C	T	SNP	OS07G0500300	Exonic	CDS	Silent	LOC_Os07g31720	GTPase activating protein, putative, expressed
Chr7	27172758	27172758	C	C	T	SNP	OS07G0650100	Exonic	CDS	Missense	LOC_Os07g45550	expressed protein
Chr7	27174317	27174317	C	C	T	SNP	OS07G0650100	Exonic	CDS	Missense	LOC_Os07g45550	expressed protein
Chr7	2644886	2644886	A	A	T	SNP	OS07G0150700	Exonic	CDS	Silent	LOC_Os07g05620	CAMK_KIN1/SNF1/Nim1_like.28 - CAMK includes calcium/calmodulin depedent protein kinases, expressed
Chr7	8969233	8969233	A	A	T	SNP	OS07G0258400	Exonic	CDS	Silent	LOC_Os07g15460	metal transporter Nramp6, putative, expressed
Chr7	17410452	17410452	A	A	T	SNP	OS07G0479300	Exonic	CDS	Missense	LOC_Os07g29620	OsSCP39 - Putative Serine Carboxypeptidase homologue, expressed
Chr7	17412075	17412075	A	A	T	SNP	OS07G0479300	Intronic	NA	NA	LOC_Os07g29620	OsSCP39 - Putative Serine Carboxypeptidase homologue, expressed
Chr7	18841827	18841827	A	A	T	SNP	OS07G0500300	Intronic	NA	NA	LOC_Os07g31720	GTPase activating protein, putative, expressed
Chr7	27172478	27172478	A	A	T	SNP	OS07G0650100	Exonic	CDS	Missense	LOC_Os07g45550	expressed protein
Chr7	1615042	1615042	G	G	T	SNP	OS07G0130800	Exonic	CDS	NA	LOC_Os07g03870	receptor like protein kinase, putative, expressed
Chr7	3610537	3610537	G	G	T	SNP	OS07G0167100	Exonic	3UTR	NA	LOC_Os07g07260	glutamine-dependent NAD, putative, expressed
Chr7	4264328	4264328	G	G	T	SNP	OS07G0180900	Exonic	CDS	Missense	LOC_Os07g08330	ribosomal protein L4, putative, expressed
Chr7	4264988	4264988	G	G	T	SNP	OS07G0180900	Intronic	NA	NA	LOC_Os07g08330	ribosomal protein L4, putative, expressed
Chr7	4683525	4683525	G	G	T	SNP	OS07G0187700	Intronic	NA	NA	LOC_Os07g09000	WD domain, G-beta repeat domain containing protein, expressed
Chr7	18763583	18763583	G	G	T	SNP	OS07G0499300	Exonic	3UTR	NA	LOC_Os07g31599	expressed protein
Chr7	21281811	21281811	G	G	T	SNP	OS07G0539900	Exonic	CDS	Silent	LOC_Os07g35560	glucan endo-1,3-beta-glucosidase precursor, putative, expressed
Chr7	21415417	21415417	G	G	T	SNP	OS07G0541900	Exonic	CDS	Silent	LOC_Os07g35750	TKL_IRAK_DUF26-ld.3 - DUF26 kinases have homology to DUF26 containing loci, expressed
Chr7	22429327	22429327	G	G	T	SNP	OS07G0561800	Exonic	3UTR	NA	LOC_Os07g37454	urate anion exchanger, putative, expressed
Chr7	26769075	26769075	G	G	T	SNP	OS07G0642800	Intronic	NA	NA	LOC_Os07g44830	proline-rich family protein, putative, expressed
Chr7	27723604	27723604	G	G	T	SNP	OS07G0658400	Exonic	CDS	NA	LOC_Os07g46460	ferredoxin-dependent glutamate synthase, chloroplast precursor, putative, expressed
Chr7	27724188	27724188	G	G	T	SNP	OS07G0658400	Intronic	NA	NA	LOC_Os07g46460	ferredoxin-dependent glutamate synthase, chloroplast precursor, putative, expressed
Chr7	27733729	27733729	G	G	T	SNP	OS07G0658400	Intronic	NA	NA	LOC_Os07g46460	ferredoxin-dependent glutamate synthase, chloroplast precursor, putative, expressed
Chr7	27734795	27734795	G	G	T	SNP	OS07G0658400	Intronic	NA	NA	LOC_Os07g46460	ferredoxin-dependent glutamate synthase, chloroplast precursor, putative, expressed
Chr7	27736883	27736883	G	G	T	SNP	OS07G0658400	Exonic	CDS	NA	LOC_Os07g46460	ferredoxin-dependent glutamate synthase, chloroplast precursor, putative, expressed
Chr7	28121260	28121260	G	G	T	SNP	OS07G0665900	Intronic	NA	NA	LOC_Os07g47040	expressed protein
Chr7	925342	925342	C	C	T	SNP	OS07G0116900	Exonic	3UTR	NA	LOC_Os07g02570	NB-ARC domain containing protein, expressed
Chr7	925962	925962	C	C	T	SNP	OS07G0116900	Exonic	3UTR	NA	LOC_Os07g02570	NB-ARC domain containing protein, expressed
Chr7	2195642	2195642	C	C	T	SNP	OS07G0142900	Intronic	NA	NA	LOC_Os07g04990	oxidoreductase, aldo/keto reductase family protein, putative, expressed
Chr7	3606268	3606268	C	C	T	SNP	OS07G0167100	Intronic	NA	NA	LOC_Os07g07260	glutamine-dependent NAD, putative, expressed
Chr7	3606658	3606658	C	C	T	SNP	OS07G0167100	Intronic	NA	NA	LOC_Os07g07260	glutamine-dependent NAD, putative, expressed
Chr7	3608939	3608939	C	C	T	SNP	OS07G0167100	Intronic	NA	NA	LOC_Os07g07260	glutamine-dependent NAD, putative, expressed
Chr7	3609036	3609036	C	C	T	SNP	OS07G0167100	Intronic	NA	NA	LOC_Os07g07260	glutamine-dependent NAD, putative, expressed
Chr7	3610653	3610653	C	C	T	SNP	OS07G0167100	Exonic	3UTR	NA	LOC_Os07g07260	glutamine-dependent NAD, putative, expressed

Chr7	4263926	4263926	C	C	T	SNP	OS07G0180900	Exonic	CDS	Silent	LOC_Os07g08330	ribosomal protein L4, putative, expressed
Chr7	4264214	4264214	C	C	T	SNP	OS07G0180900	Exonic	CDS	Silent	LOC_Os07g08330	ribosomal protein L4, putative, expressed
Chr7	4265340	4265340	C	C	T	SNP	OS07G0180900	Exonic	CDS	Silent	LOC_Os07g08330	ribosomal protein L4, putative, expressed
Chr7	4683821	4683821	C	C	T	SNP	OS07G0187700	Exonic	CDS	Silent	LOC_Os07g09000	WD domain, G-beta repeat domain containing protein, expressed
Chr7	4685184	4685184	C	C	T	SNP	OS07G0187700	Intronic	NA	NA	LOC_Os07g09000	WD domain, G-beta repeat domain containing protein, expressed
Chr7	8076243	8076243	C	C	T	SNP	OS07G0245100	Exonic	5UTR	NA	LOC_Os07g14150	cytidine deaminase, putative, expressed
Chr7	8076589	8076589	C	C	T	SNP	OS07G0245100	Exonic	5UTR	NA	LOC_Os07g14150	cytidine deaminase, putative, expressed
Chr7	9036287	9036287	C	C	T	SNP	OS07G0259700	Exonic	CDS	Silent	LOC_Os07g15600	H-BTB6 - Bric-a-Brac, Tramtrack, Broad Complex BTB domain with H family conserved sequence, expressed
Chr7	13101671	13101671	C	C	T	SNP	OS07G0415200	Intronic	NA	NA	LOC_Os07g23244	ribosomal protein L23 family protein, putative, expressed
Chr7	13103614	13103614	C	C	T	SNP	OS07G0415200	Exonic	3UTR	NA	LOC_Os07g23244	ribosomal protein L23 family protein, putative, expressed
Chr7	16899850	16899850	C	C	T	SNP	OS07G0471300	Exonic	CDS	NA	LOC_Os07g28850	retrotransposon protein, putative, unclassified, expressed
Chr7	21245526	21245526	C	C	T	SNP	OS07G0539400	Exonic	CDS	Missense	LOC_Os07g35520	glucan endo-1,3-beta-glucosidase precursor, putative, expressed
Chr7	21245986	21245986	C	C	T	SNP	OS07G0539400	Exonic	CDS	Missense	LOC_Os07g35520	glucan endo-1,3-beta-glucosidase precursor, putative, expressed
Chr7	21280844	21280844	C	C	T	SNP	OS07G0539900	Exonic	CDS	Missense	LOC_Os07g35560	glucan endo-1,3-beta-glucosidase precursor, putative, expressed
Chr7	21280936	21280936	C	C	T	SNP	OS07G0539900	Exonic	CDS	Silent	LOC_Os07g35560	glucan endo-1,3-beta-glucosidase precursor, putative, expressed
Chr7	21280969	21280969	C	C	T	SNP	OS07G0539900	Intronic	NA	NA	LOC_Os07g35560	glucan endo-1,3-beta-glucosidase precursor, putative, expressed
Chr7	21281889	21281889	C	C	T	SNP	OS07G0539900	Exonic	CDS	Silent	LOC_Os07g35560	glucan endo-1,3-beta-glucosidase precursor, putative, expressed
Chr7	21415423	21415423	C	C	T	SNP	OS07G0541900	Exonic	CDS	Silent	LOC_Os07g35750	TKL_IRAK_DUF26-ld.3 - DUF26 kinases have homology to DUF26 containing loci, expressed
Chr7	21415750	21415750	C	C	T	SNP	OS07G0541900	Exonic	CDS	Silent	LOC_Os07g35750	TKL_IRAK_DUF26-ld.3 - DUF26 kinases have homology to DUF26 containing loci, expressed
Chr7	21417725	21417725	C	C	T	SNP	OS07G0541900	Exonic	CDS	Missense	LOC_Os07g35750	TKL_IRAK_DUF26-ld.3 - DUF26 kinases have homology to DUF26 containing loci, expressed
Chr7	22859183	22859183	C	C	T	SNP	OS07G0568600	Intronic	NA	NA	LOC_Os07g38120	CAMK_CAMK_like.34 - CAMK includes calcium/calmodulin depedent protein kinases, expressed
Chr7	24397114	24397114	C	C	T	SNP	OS07G0598200	Exonic	CDS	Silent	LOC_Os07g40710	circadian clock coupling factor-related, putative, expressed
Chr7	24397257	24397257	C	C	T	SNP	OS07G0598200	Exonic	CDS	Missense	LOC_Os07g40710	circadian clock coupling factor-related, putative, expressed
Chr7	24397476	24397476	C	C	T	SNP	OS07G0598200	Exonic	CDS	Missense	LOC_Os07g40710	circadian clock coupling factor-related, putative, expressed
Chr7	24398608	24398608	C	C	T	SNP	OS07G0598200	Intronic	NA	NA	LOC_Os07g40710	circadian clock coupling factor-related, putative, expressed
Chr7	24656263	24656263	C	C	T	SNP	OS07G0603100	Intronic	NA	NA	LOC_Os07g41180	RNA-binding protein-like, putative, expressed
Chr7	24658400	24658400	C	C	T	SNP	OS07G0603100	Intronic	NA	NA	LOC_Os07g41180	RNA-binding protein-like, putative, expressed
Chr7	24658570	24658570	C	C	T	SNP	OS07G0603100	Intronic	NA	NA	LOC_Os07g41180	RNA-binding protein-like, putative, expressed
Chr7	24700325	24700325	C	C	T	SNP	OS07G0603600	Intronic	NA	NA	LOC_Os07g41230	esterase, putative, expressed
Chr7	24700645	24700645	C	C	T	SNP	OS07G0603600	Intronic	NA	NA	LOC_Os07g41230	esterase, putative, expressed
Chr7	24701043	24701043	C	C	T	SNP	OS07G0603600	Intronic	NA	NA	LOC_Os07g41230	esterase, putative, expressed
Chr7	24702485	24702485	C	C	T	SNP	OS07G0603600	Intronic	NA	NA	LOC_Os07g41230	esterase, putative, expressed
Chr7	24702633	24702633	C	C	T	SNP	OS07G0603600	Exonic	CDS	NA	LOC_Os07g41230	esterase, putative, expressed
Chr7	26632026	26632026	C	C	T	SNP	OS07G0639900	Exonic	CDS	Missense	LOC_Os07g44630	thymidylate kinase, putative, expressed
Chr7	26769013	26769013	C	C	T	SNP	OS07G0642800	Intronic	NA	NA	LOC_Os07g44830	proline-rich family protein, putative, expressed
Chr7	27723698	27723698	C	C	T	SNP	OS07G0658400	Exonic	CDS	NA	LOC_Os07g46460	ferredoxin-dependent glutamate synthase, chloroplast precursor, putative, expressed
Chr7	27724095	27724095	C	C	T	SNP	OS07G0658400	Intronic	NA	NA	LOC_Os07g46460	ferredoxin-dependent glutamate synthase, chloroplast precursor, putative, expressed
Chr7	27724719	27724719	C	C	T	SNP	OS07G0658400	Intronic	NA	NA	LOC_Os07g46460	ferredoxin-dependent glutamate synthase, chloroplast precursor, putative, expressed
Chr7	27724724	27724724	C	C	T	SNP	OS07G0658400	Intronic	NA	NA	LOC_Os07g46460	ferredoxin-dependent glutamate synthase, chloroplast precursor, putative, expressed
Chr7	27724775	27724775	C	C	T	SNP	OS07G0658400	Exonic	CDS	NA	LOC_Os07g46460	ferredoxin-dependent glutamate synthase, chloroplast precursor, putative, expressed
Chr7	27725067	27725067	C	C	T	SNP	OS07G0658400	Intronic	NA	NA	LOC_Os07g46460	ferredoxin-dependent glutamate synthase, chloroplast precursor, putative, expressed
Chr7	27726741	27726741	C	C	T	SNP	OS07G0658400	Intronic	NA	NA	LOC_Os07g46460	ferredoxin-dependent glutamate synthase, chloroplast precursor, putative, expressed
Chr7	27726784	27726784	C	C	T	SNP	OS07G0658400	Intronic	NA	NA	LOC_Os07g46460	ferredoxin-dependent glutamate synthase, chloroplast precursor, putative, expressed
Chr7	27727117	27727117	C	C	T	SNP	OS07G0658400	Intronic	NA	NA	LOC_Os07g46460	ferredoxin-dependent glutamate synthase, chloroplast precursor, putative, expressed
Chr7	27727490	27727490	C	C	T	SNP	OS07G0658400	Intronic	NA	NA	LOC_Os07g46460	ferredoxin-dependent glutamate synthase, chloroplast precursor, putative, expressed
Chr7	27728709	27728709	C	C	T	SNP	OS07G0658400	Intronic	NA	NA	LOC_Os07g46460	ferredoxin-dependent glutamate synthase, chloroplast precursor, putative, expressed
Chr7	27728922	27728922	C	C	T	SNP	OS07G0658400	Intronic	NA	NA	LOC_Os07g46460	ferredoxin-dependent glutamate synthase, chloroplast precursor, putative, expressed
Chr7	27729525	27729525	C	C	T	SNP	OS07G0658400	Intronic	NA	NA	LOC_Os07g46460	ferredoxin-dependent glutamate synthase, chloroplast precursor, putative, expressed
Chr7	27729812	27729812	C	C	T	SNP	OS07G0658400	Intronic	NA	NA	LOC_Os07g46460	ferredoxin-dependent glutamate synthase, chloroplast precursor, putative, expressed
Chr7	27729931	27729931	C	C	T	SNP	OS07G0658400	Exonic	CDS	NA	LOC_Os07g46460	ferredoxin-dependent glutamate synthase, chloroplast precursor, putative, expressed
Chr7	27730039	27730039	C	C	T	SNP	OS07G0658400	Intronic	NA	NA	LOC_Os07g46460	ferredoxin-dependent glutamate synthase, chloroplast precursor, putative, expressed
Chr7	27730286	27730286	C	C	T	SNP	OS07G0658400	Intronic	NA	NA	LOC_Os07g46460	ferredoxin-dependent glutamate synthase, chloroplast precursor, putative, expressed
Chr7	27730539	27730539	C	C	T	SNP	OS07G0658400	Intronic	NA	NA	LOC_Os07g46460	ferredoxin-dependent glutamate synthase, chloroplast precursor, putative, expressed
Chr7	27730730	27730730	C	C	T	SNP	OS07G0658400	Intronic	NA	NA	LOC_Os07g46460	ferredoxin-dependent glutamate synthase, chloroplast precursor, putative, expressed
Chr7	27730851	27730851	C	C	T	SNP	OS07G0658400	Intronic	NA	NA	LOC_Os07g46460	ferredoxin-dependent glutamate synthase, chloroplast precursor, putative, expressed
Chr7	27731681	27731681	C	C	T	SNP	OS07G0658400	Intronic	NA	NA	LOC_Os07g46460	ferredoxin-dependent glutamate synthase, chloroplast precursor, putative, expressed
Chr7	27732393	27732393	C	C	T	SNP	OS07G0658400	Intronic	NA	NA	LOC_Os07g46460	ferredoxin-dependent glutamate synthase, chloroplast precursor, putative, expressed
Chr7	27733114	27733114	C	C	T	SNP	OS07G0658400	Intronic	NA	NA	LOC_Os07g46460	ferredoxin-dependent glutamate synthase, chloroplast precursor, putative, expressed
Chr7	27733167	27733167	C	C	T	SNP	OS07G0658400	Intronic	NA	NA	LOC_Os07g46460	ferredoxin-dependent glutamate synthase, chloroplast precursor, putative, expressed
Chr7	27733906	27733906	C	C	T	SNP	OS07G0658400	Intronic	NA	NA	LOC_Os07g46460	ferredoxin-dependent glutamate synthase, chloroplast precursor, putative, expressed
Chr7	27735367	27735367	C	C	T	SNP	OS07G0658400	Intronic	NA	NA	LOC_Os07g46460	ferredoxin-dependent glutamate synthase, chloroplast precursor, putative, expressed
Chr7	27735391	27735391	C	C	T	SNP	OS07G0658400	Intronic	NA	NA	LOC_Os07g46460	ferredoxin-dependent glutamate synthase, chloroplast precursor, putative, expressed
Chr7	27735498	27735498	C	C	T	SNP	OS07G0658400	Intronic	NA	NA	LOC_Os07g46460	ferredoxin-dependent glutamate synthase, chloroplast precursor, putative, expressed
Chr7	27736364	27736364	C	C	T	SNP	OS07G0658400	Intronic	NA	NA	LOC_Os07g46460	ferredoxin-dependent glutamate synthase, chloroplast precursor, putative, expressed
Chr7	27736636	27736636	C	C	T	SNP	OS07G0658400	Intronic	NA	NA	LOC_Os07g46460	ferredoxin-dependent glutamate synthase, chloroplast precursor, putative, expressed
Chr7	27737546	27737546	C	C	T	SNP	OS07G0658400	Intronic	NA	NA	LOC_Os07g46460	ferredoxin-dependent glutamate synthase, chloroplast precursor, putative, expressed
Chr7	27737641	27737641	C	C	T	SNP	OS07G0658400	Intronic	NA	NA	LOC_Os07g46460	ferredoxin-dependent glutamate synthase, chloroplast precursor, putative, expressed
Chr7	27737660	27737660	C	C	T	SNP	OS07G0658400	Intronic	NA	NA	LOC_Os07g46460	ferredoxin-dependent glutamate synthase, chloroplast precursor, putative, expressed
Chr7	28671118	28671118	C	C	T	SNP	OS07G0677100	Exonic	CDS	Missense	LOC_Os07g48010	peroxidase precursor, putative, expressed
Chr7	28781349	28781349	C	C	T	SNP	OS07G0679500	Intronic	NA	NA	LOC_Os07g48180	bZIP transcription factor domain containing protein, expressed
Chr7	28782978	28782978	C	C	T	SNP	OS07G0679500	Intronic	NA	NA	LOC_Os07g48180	bZIP transcription factor domain containing protein, expressed
Chr7	29636252	29636252	C	C	T	SNP	OS07G0695400	Exonic	CDS	Missense	LOC_Os07g49480	KIP1, putative, expressed
Chr7	27555101	27555101	A	A	T	SNP	OS07G0655400	Exonic	3UTR	NA	LOC_Os07g46170	OsRhmbd13 - Putative Rhomboid homologue, expressed
Chr7	19943	19943	A	A	T	SNP	OS07G0100300	Intronic	NA	NA	LOC_Os07g01030	glycosyl transferase, group 1 domain containing protein, expressed
Chr7	4264327	4264327	A	A	T	SNP	OS07G0180900	Exonic	CDS	Missense	LOC_Os07g08330	ribosomal protein L4, putative, expressed
Chr7	4265220	4265220	A	A	T	SNP	OS07G0180900	Intronic	NA	NA	LOC_Os07g08330	ribosomal protein L4, putative, expressed
Chr7	4265546	4265546	A	A	T	SNP	OS07G0180900	Exonic	3UTR	NA	LOC_Os07g08330	ribosomal protein L4, putative, expressed
Chr7	4504320	4504320	A	A	T	SNP	OS07G0185200	Exonic	CDS	Silent	LOC_Os07g08760	START domain containing protein, putative, expressed
Chr7	4687038	4687038	A	A	T	SNP	OS07G0187700	Intronic	NA	NA	LOC_Os07g09000	WD domain, G-beta repeat domain containing protein, expressed
Chr7	13100290	13100290	A	A	T	SNP	OS07G0415200	Intronic	NA	NA	LOC_Os07g23244	ribosomal protein L23 family protein, putative, expressed
Chr7	15376923	15376923	A	A	T	SNP	OS07G0448400	Exonic	CDS	NA	LOC_Os07g26660	aquaporin protein, putative, expressed
Chr7	22412816	22412816	A	A	T	SNP	OS07G0561300	Intronic	NA	NA	LOC_Os07g37400	OsFBX257 - F-box domain containing protein, expressed
Chr7	26406866	26406866	A	A	T	SNP	OS07G0636000	Exonic	3UTR	NA	LOC_Os07g44190	h/ACA ribonucleoprotein complex subunit 4, putative, expressed
Chr7	27723557	27723557	A	A	T	SNP	OS07G0658400	Exonic	3UTR	NA	LOC_Os07g46460	ferredoxin-dependent glutamate synthase, chloroplast precursor, putative, expressed
Chr7	27723926	27723926	A	A	T	SNP	OS07G0658400	Intronic	NA	NA	LOC_Os07g46460	ferredoxin-dependent glutamate synthase, chloroplast precursor, putative, expressed

Chr7	27726632	27726632	A	A	T	SNP	OS07G0658400	Intronic	NA	NA	LOC_Os07g46460	ferredoxin-dependent glutamate synthase, chloroplast precursor, putative, expressed
Chr7	27726634	27726634	A	A	T	SNP	OS07G0658400	Intronic	NA	NA	LOC_Os07g46460	ferredoxin-dependent glutamate synthase, chloroplast precursor, putative, expressed
Chr7	27730314	27730314	A	A	T	SNP	OS07G0658400	Exonic	CDS	NA	LOC_Os07g46460	ferredoxin-dependent glutamate synthase, chloroplast precursor, putative, expressed
Chr7	27730788	27730788	A	A	T	SNP	OS07G0658400	Intronic	NA	NA	LOC_Os07g46460	ferredoxin-dependent glutamate synthase, chloroplast precursor, putative, expressed
Chr7	27733435	27733435	A	A	T	SNP	OS07G0658400	Intronic	NA	NA	LOC_Os07g46460	ferredoxin-dependent glutamate synthase, chloroplast precursor, putative, expressed
Chr7	27734451	27734451	A	A	T	SNP	OS07G0658400	Intronic	NA	NA	LOC_Os07g46460	ferredoxin-dependent glutamate synthase, chloroplast precursor, putative, expressed
Chr7	27735507	27735507	A	A	T	SNP	OS07G0658400	Intronic	NA	NA	LOC_Os07g46460	ferredoxin-dependent glutamate synthase, chloroplast precursor, putative, expressed
Chr7	28124400	28124400	A	A	T	SNP	OS07G0665900	Exonic	CDS	Missense	LOC_Os07g47040	expressed protein
Chr7	28782740	28782740	A	A	T	SNP	OS07G0679500	Intronic	NA	NA	LOC_Os07g48180	bZIP transcription factor domain containing protein, expressed
Chr7	17741	17741	C	C	T	SNP	OS07G0100300	Exonic	CDS	Missense	LOC_Os07g01030	glycosyl transferase, group 1 domain containing protein, expressed
Chr7	926726	926726	C	C	T	SNP	OS07G0116900	Exonic	CDS	Missense	LOC_Os07g02570	NB-ARC domain containing protein, expressed
Chr7	926731	926731	C	C	T	SNP	OS07G0116900	Exonic	CDS	Silent	LOC_Os07g02570	NB-ARC domain containing protein, expressed
Chr7	1567211	1567211	G	G	T	SNP	OS07G0129700	Intronic	NA	NA	LOC_Os07g03770	Homeobox domain containing protein, expressed
Chr7	1791607	1791607	C	C	T	SNP	OS07G0134000	Exonic	CDS	Missense	LOC_Os07g04180	amino acid transporter, putative, expressed
Chr7	1794380	1794380	G	G	T	SNP	OS07G0134000	Exonic	CDS	Silent	LOC_Os07g04180	amino acid transporter, putative, expressed
Chr7	3606168	3606168	A	A	T	SNP	OS07G0167100	Intronic	NA	NA	LOC_Os07g07260	glutamine-dependent NAD, putative, expressed
Chr7	3606444	3606444	A	A	T	SNP	OS07G0167100	Intronic	NA	NA	LOC_Os07g07260	glutamine-dependent NAD, putative, expressed
Chr7	4263193	4263193	A	A	T	SNP	OS07G0180900	Exonic	5UTR	NA	LOC_Os07g08330	ribosomal protein L4, putative, expressed
Chr7	4263503	4263503	C	C	T	SNP	OS07G0180900	Exonic	CDS	Silent	LOC_Os07g08330	ribosomal protein L4, putative, expressed
Chr7	4265049	4265049	C	C	T	SNP	OS07G0180900	Intronic	NA	NA	LOC_Os07g08330	ribosomal protein L4, putative, expressed
Chr7	4505237	4505237	A	A	T	SNP	OS07G0185200	Intronic	NA	NA	LOC_Os07g08760	START domain containing protein, putative, expressed
Chr7	4507634	4507634	C	C	T	SNP	OS07G0185200	Exonic	5UTR	NA	LOC_Os07g08760	START domain containing protein, putative, expressed
Chr7	4686111	4686111	G	G	T	SNP	OS07G0187700	Intronic	NA	NA	LOC_Os07g09000	WD domain, G-beta repeat domain containing protein, expressed
Chr7	6968832	6968832	A	A	T	SNP	OS07G0225300	Exonic	CDS	Missense	LOC_Os07g12340	NAC domain-containing protein 67, putative, expressed
Chr7	7107461	7107461	C	C	T	SNP	OS07G0227200	Exonic	CDS	Missense	LOC_Os07g12470	expressed protein
Chr7	8970706	8970706	A	A	T	SNP	OS07G0258400	Exonic	3UTR	NA	LOC_Os07g15460	metal transporter Nramp6, putative, expressed
Chr7	13099148	13099148	C	C	T	SNP	OS07G0415200	Exonic	CDS	Missense	LOC_Os07g23244	ribosomal protein L23 family protein, putative, expressed
Chr7	13366675	13366675	C	C	T	SNP	OS07G0418700	Exonic	CDS	NA	LOC_Os07g23660	retrotransposon protein, putative, unclassified, expressed
Chr7	16503485	16503485	G	G	T	SNP	OS07G0466000	Intronic	NA	NA	LOC_Os07g28250	lipase class 3 family protein, putative, expressed
Chr7	18840543	18840543	C	C	T	SNP	OS07G0500300	Intronic	NA	NA	LOC_Os07g31720	GTPase activating protein, putative, expressed
Chr7	19473707	19473707	G	G	T	SNP	OS07G0510800	Exonic	CDS	Silent	LOC_Os07g32650	retrotransposon protein, putative, unclassified, expressed
Chr7	20085797	20085797	C	C	T	SNP	OS07G0520300	Exonic	CDS	Silent	LOC_Os07g33620	cytochrome P450 domain containing protein, expressed
Chr7	20724692	20724692	C	C	T	SNP	OS07G0529600	Exonic	CDS	Missense	LOC_Os07g34570	FAD dependent oxidoreductase domain containing protein, expressed
Chr7	21281201	21281201	C	C	T	SNP	OS07G0539900	Exonic	CDS	Missense	LOC_Os07g35560	glucan endo-1,3-beta-glucosidase precursor, putative, expressed
Chr7	21281232	21281232	C	C	T	SNP	OS07G0539900	Exonic	CDS	Silent	LOC_Os07g35560	glucan endo-1,3-beta-glucosidase precursor, putative, expressed
Chr7	21281235	21281235	C	C	T	SNP	OS07G0539900	Exonic	CDS	Silent	LOC_Os07g35560	glucan endo-1,3-beta-glucosidase precursor, putative, expressed
Chr7	21281672	21281672	C	C	T	SNP	OS07G0539900	Exonic	CDS	Missense	LOC_Os07g35560	glucan endo-1,3-beta-glucosidase precursor, putative, expressed
Chr7	21414645	21414645	C	C	T	SNP	OS07G0541900	Exonic	CDS	Missense	LOC_Os07g35750	TKL_IRAK_DUF26-ld.3 - DUF26 kinases have homology to DUF26 containing loci, expressed
Chr7	21418620	21418620	G	G	T	SNP	OS07G0541900	Exonic	CDS	Missense	LOC_Os07g35750	TKL_IRAK_DUF26-ld.3 - DUF26 kinases have homology to DUF26 containing loci, expressed
Chr7	21418834	21418834	C	C	T	SNP	OS07G0541900	Exonic	CDS	Missense	LOC_Os07g35750	TKL_IRAK_DUF26-ld.3 - DUF26 kinases have homology to DUF26 containing loci, expressed
Chr7	21419069	21419069	C	C	T	SNP	OS07G0541900	Exonic	CDS	Silent	LOC_Os07g35750	TKL_IRAK_DUF26-ld.3 - DUF26 kinases have homology to DUF26 containing loci, expressed
Chr7	22412508	22412508	A	A	T	SNP	OS07G0561300	Intronic	NA	NA	LOC_Os07g37400	OsFBX257 - F-box domain containing protein, expressed
Chr7	22429216	22429216	A	A	T	SNP	OS07G0561800	Exonic	3UTR	NA	LOC_Os07g37454	urate anion exchanger, putative, expressed
Chr7	22544461	22544461	C	C	T	SNP	OS07G0563400	Exonic	CDS	Silent	LOC_Os07g37620	fiber expressed protein, putative, expressed
Chr7	22874941	22874941	C	C	T	SNP	OS07G0568700	Exonic	CDS	Missense	LOC_Os07g38130	polygalacturonase inhibitor 1 precursor, putative, expressed
Chr7	23185719	23185719	C	C	T	SNP	OS07G0573600	Exonic	CDS	Silent	LOC_Os07g38600	REX1 DNA Repair family protein, expressed
Chr7	23283461	23283461	C	C	T	SNP	OS07G0575700	Exonic	CDS	Missense	LOC_Os07g38810	lectin receptor-type protein kinase, putative, expressed
Chr7	23678030	23678030	G	G	T	SNP	OS07G0584100	Exonic	CDS	Missense	LOC_Os07g39520	STE_PAK_Ste20_Slob_Wnk.5 - STE kinases include homologs to sterile 7, sterile 11 and sterile 20 from yeast, expressed
Chr7	24398777	24398777	C	C	T	SNP	OS07G0598200	Intronic	NA	NA	LOC_Os07g40710	circadian clock coupling factor-related, putative, expressed
Chr7	24398848	24398848	C	C	T	SNP	OS07G0598200	Intronic	NA	NA	LOC_Os07g40710	circadian clock coupling factor-related, putative, expressed
Chr7	24399755	24399755	C	C	T	SNP	OS07G0598200	Intronic	NA	NA	LOC_Os07g40710	circadian clock coupling factor-related, putative, expressed
Chr7	26776295	26776295	C	C	T	SNP	OS07G0642900	Intronic	NA	NA	LOC_Os07g44840	bacterial transferase hexapeptide domain containing protein, expressed
Chr7	26776879	26776879	A	A	T	SNP	OS07G0642900	Exonic	CDS	Silent	LOC_Os07g44840	bacterial transferase hexapeptide domain containing protein, expressed
Chr7	27172525	27172525	G	G	T	SNP	OS07G0650100	Exonic	CDS	Missense	LOC_Os07g45550	expressed protein
Chr7	27172770	27172770	C	C	T	SNP	OS07G0650100	Exonic	CDS	Missense	LOC_Os07g45550	expressed protein
Chr7	27551126	27551126	C	C	T	SNP	OS07G0655400	Exonic	5UTR	NA	LOC_Os07g46170	OsRhmbd13 - Putative Rhomboid homologue, expressed
Chr7	27551212	27551212	G	G	T	SNP	OS07G0655400	Intronic	NA	NA	LOC_Os07g46170	OsRhmbd13 - Putative Rhomboid homologue, expressed
Chr7	27724909	27724909	C	C	T	SNP	OS07G0658400	Intronic	NA	NA	LOC_Os07g46460	ferredoxin-dependent glutamate synthase, chloroplast precursor, putative, expressed
Chr7	27725880	27725880	C	C	T	SNP	OS07G0658400	Intronic	NA	NA	LOC_Os07g46460	ferredoxin-dependent glutamate synthase, chloroplast precursor, putative, expressed
Chr7	27726166	27726166	C	C	T	SNP	OS07G0658400	Exonic	CDS	NA	LOC_Os07g46460	ferredoxin-dependent glutamate synthase, chloroplast precursor, putative, expressed
Chr7	27732186	27732186	C	C	T	SNP	OS07G0658400	Intronic	NA	NA	LOC_Os07g46460	ferredoxin-dependent glutamate synthase, chloroplast precursor, putative, expressed
Chr7	27737777	27737777	A	A	T	SNP	OS07G0658400	Intronic	NA	NA	LOC_Os07g46460	ferredoxin-dependent glutamate synthase, chloroplast precursor, putative, expressed
Chr7	28383415	28383415	C	C	T	SNP	OS07G0671400	Exonic	CDS	Silent	LOC_Os07g47480	heavy metal-associated domain containing protein, expressed
Chr7	28383819	28383819	C	C	T	SNP	OS07G0671400	Exonic	CDS	Missense	LOC_Os07g47480	heavy metal-associated domain containing protein, expressed
Chr7	28678515	28678515	G	G	T	SNP	OS07G0677300	Exonic	CDS	Silent	LOC_Os07g48030	peroxidase precursor, putative, expressed
Chr7	28711556	28711556	C	C	T	SNP	OS07G0678300	Exonic	CDS	Silent	LOC_Os07g48090	CAMK_KIN1/SNF1/Nim1_like.30 - CAMK includes calcium/calmodulin depedent protein kinases, expressed
Chr7	28781539	28781539	C	C	T	SNP	OS07G0679500	Intronic	NA	NA	LOC_Os07g48180	bZIP transcription factor domain containing protein, expressed
Chr7	28859118	28859118	G	G	T	SNP	OS07G0680900	Exonic	CDS	NA	LOC_Os07g48290	AGC_AGC_other_RS6K_like.2 - ACG kinases include homologs to PKA, PKG and PKC, expressed
Chr7	29324009	29324009	C	C	T	SNP	OS07G0689600	Exonic	CDS	Silent	LOC_Os07g48980	nicotianamine synthase, putative, expressed
Chr7	699885	699886	TA	TA	T	INDEL	OS07G0112700	Exonic	3UTR	NA	LOC_Os07g02200	plastocyanin-like domain containing protein, putative, expressed
Chr7	1567139	1567142	TCCC	TCCC	T	INDEL	OS07G0129700	Intronic	NA	NA	LOC_Os07g03770	Homeobox domain containing protein, expressed
Chr7	1567202	1567204	TTC	TTC	T	INDEL	OS07G0129700	Intronic	NA	NA	LOC_Os07g03770	Homeobox domain containing protein, expressed
Chr7	1567696	1567698	TTC	TTC	T	INDEL	OS07G0129700	Intronic	NA	NA	LOC_Os07g03770	Homeobox domain containing protein, expressed
Chr7	4265134	4265135	TG	TG	T	INDEL	OS07G0180900	Intronic	NA	NA	LOC_Os07g08330	ribosomal protein L4, putative, expressed
Chr7	4682886	4682898	TCTCCTCCTCCTC	TCTCCTCCTCCTC	T	INDEL	OS07G0187700	Intronic	NA	NA	LOC_Os07g09000	WD domain, G-beta repeat domain containing protein, expressed
Chr7	21415598	21415599	TA	TA	T	INDEL	OS07G0541900	Exonic	CDS	FrameShift	LOC_Os07g35750	TKL_IRAK_DUF26-ld.3 - DUF26 kinases have homology to DUF26 containing loci, expressed
Chr7	26768724	26768725	TC	TC	T	INDEL	OS07G0642800	Intronic	NA	NA	LOC_Os07g44830	proline-rich family protein, putative, expressed
Chr7	26769612	26769617	TTTTTA	TTTTTA	T	INDEL	OS07G0642800	Intronic	NA	NA	LOC_Os07g44830	proline-rich family protein, putative, expressed
Chr7	26774086	26774088	TTA	TTA	T	INDEL	OS07G0642800	Exonic	3UTR	NA	LOC_Os07g44830	proline-rich family protein, putative, expressed
Chr7	27723374	27723375	TC	TC	T	INDEL	OS07G0658400	Exonic	3UTR	NA	LOC_Os07g46460	ferredoxin-dependent glutamate synthase, chloroplast precursor, putative, expressed
Chr7	27724670	27724672	TAA	TAA	T	INDEL	OS07G0658400	Intronic	NA	NA	LOC_Os07g46460	ferredoxin-dependent glutamate synthase, chloroplast precursor, putative, expressed
Chr7	27725101	27725102	TA	TA	T	INDEL	OS07G0658400	Intronic	NA	NA	LOC_Os07g46460	ferredoxin-dependent glutamate synthase, chloroplast precursor, putative, expressed
Chr7	27726347	27726351	TTATA	TTATA	T	INDEL	OS07G0658400	Intronic	NA	NA	LOC_Os07g46460	ferredoxin-dependent glutamate synthase, chloroplast precursor, putative, expressed
Chr7	27732734	27732735	TA	TA	T	INDEL	OS07G0658400	Intronic	NA	NA	LOC_Os07g46460	ferredoxin-dependent glutamate synthase, chloroplast precursor, putative, expressed
Chr7	27733288	27733291	TAAA	TAAA	T	INDEL	OS07G0658400	Intronic	NA	NA	LOC_Os07g46460	ferredoxin-dependent glutamate synthase, chloroplast precursor, putative, expressed

Chr7	27733374	27733375	TA	TA	T	INDEL	OS07G0658400	Intronic	NA	NA	LOC_Os07g46460	ferredoxin-dependent glutamate synthase, chloroplast precursor, putative, expressed
Chr7	27734412	27734428	TAGTTCAGCAATATTTC	TAGTTCAGCAATATTTC	T	INDEL	OS07G0658400	Intronic	NA	NA	LOC_Os07g46460	ferredoxin-dependent glutamate synthase, chloroplast precursor, putative, expressed
Chr7	27736401	27736407	TTTTTAA	TTTTTAA	T	INDEL	OS07G0658400	Intronic	NA	NA	LOC_Os07g46460	ferredoxin-dependent glutamate synthase, chloroplast precursor, putative, expressed
Chr.9	18018845	18018845	C	C	T	SNP	OS09G0471900	Exonic	CDS	Silent	LOC_Os09g29630	NOL1/NOP2/sun family protein, putative, expressed
Chr.9	19012771	19012771	G	G	T	SNP	OS09G0491820	Intronic	NA	NA	LOC_Os09g31502	dehydrogenase, putative, expressed
Chr.9	3585250	3585250	G	G	T	SNP	OS09G0247600	Intronic	NA	NA	LOC_Os09g07290	GDSL-like lipase/acylhydrolase, putative, expressed
Chr.9	4578879	4578879	G	G	T	SNP	OS09G0261900	Intronic	NA	NA	LOC_Os09g08710	expressed protein
Chr.9	9359529	9359529	G	G	T	SNP	OS09G0321900	Intronic	NA	NA	LOC_Os09g15320	ubiquitin-conjugating enzyme, putative, expressed
Chr.9	10523925	10523925	G	G	T	SNP	OS09G0341600	Exonic	CDS	Missense	LOC_Os09g17152	OsFBX319 - F-box domain containing protein, expressed
Chr.9	15927953	15927953	G	G	T	SNP	OS09G0433800	Exonic	3UTR	NA	LOC_Os09g26370	DUF581 domain containing protein, expressed
Chr.9	18979553	18979553	G	G	T	SNP	OS09G0491740	Intronic	NA	NA	LOC_Os09g31478	auxin efflux carrier component, putative, expressed
Chr.9	3048976	3048976	C	C	T	SNP	OS09G0240200	Intronic	NA	NA	LOC_Os09g06464	CCT/B-box zinc finger protein, putative, expressed
Chr.9	3050164	3050164	C	C	T	SNP	OS09G0240200	Intronic	NA	NA	LOC_Os09g06464	CCT/B-box zinc finger protein, putative, expressed
Chr.9	3051639	3051639	C	C	T	SNP	OS09G0240200	Intronic	NA	NA	LOC_Os09g06464	CCT/B-box zinc finger protein, putative, expressed
Chr.9	3055426	3055426	C	C	T	SNP	OS09G0240200	Intronic	NA	NA	LOC_Os09g06464	CCT/B-box zinc finger protein, putative, expressed
Chr.9	3582104	3582104	C	C	T	SNP	OS09G0247600	Intronic	NA	NA	LOC_Os09g07290	GDSL-like lipase/acylhydrolase, putative, expressed
Chr.9	4580441	4580441	C	C	T	SNP	OS09G0261900	Exonic	3UTR	NA	LOC_Os09g08710	expressed protein
Chr.9	4578294	4578294	C	C	T	SNP	OS09G0261900	Exonic	CDS	Missense	LOC_Os09g08710	expressed protein
Chr.9	7199589	7199589	C	C	T	SNP	OS09G0297000	Intronic	NA	NA	LOC_Os09g12560	ferrochelatase-2, chloroplast precursor, putative, expressed
Chr.9	7278730	7278730	C	C	T	SNP	OS09G0298500	Exonic	CDS	Silent	LOC_Os09g12720	zinc finger, C3HC4 type domain containing protein, expressed
Chr.9	8692915	8692915	C	C	T	SNP	OS09G0315700	Exonic	CDS	Silent	LOC_Os09g14670	phosphoenolpyruvate carboxylase, putative, expressed
Chr.9	9358440	9358440	C	C	T	SNP	OS09G0321900	Intronic	NA	NA	LOC_Os09g15320	ubiquitin-conjugating enzyme, putative, expressed
Chr.9	9360401	9360401	C	C	T	SNP	OS09G0321900	Intronic	NA	NA	LOC_Os09g15320	ubiquitin-conjugating enzyme, putative, expressed
Chr.9	14531538	14531538	C	C	T	SNP	OS09G0409950	Intronic	NA	NA	LOC_Os09g24440	expressed protein
Chr.9	16959740	16959740	C	C	T	SNP	OS09G0452200	Intronic	NA	NA	LOC_Os09g27890	lysM domain-containing GPI-anchored protein precursor, putative, expressed
Chr.9	22884365	22884365	C	C	T	SNP	OS09G0572400	Exonic	CDS	Missense	LOC_Os09g39910	ABC transporter, ATP-binding protein, putative, expressed
Chr.9	3581604	3581604	A	A	T	SNP	OS09G0247600	Intronic	NA	NA	LOC_Os09g07290	GDSL-like lipase/acylhydrolase, putative, expressed
Chr.9	3655817	3655817	A	A	T	SNP	OS09G0248100	Intronic	NA	NA	LOC_Os09g07350	fasciclin-like arabinogalactan protein 8 precursor, putative, expressed
Chr.9	7003589	7003589	A	A	T	SNP	OS09G0294000	Intronic	NA	NA	LOC_Os09g12290	bifunctional aspartokinase/homoserine dehydrogenase, chloroplast precursor, putative, expressed
Chr.9	7200447	7200447	A	A	T	SNP	OS09G0297000	Intronic	NA	NA	LOC_Os09g12560	ferrochelatase-2, chloroplast precursor, putative, expressed
Chr.9	7203408	7203408	A	A	T	SNP	OS09G0297000	Intronic	NA	NA	LOC_Os09g12560	ferrochelatase-2, chloroplast precursor, putative, expressed
Chr.9	7280323	7280323	A	A	T	SNP	OS09G0298500	Intronic	NA	NA	LOC_Os09g12720	zinc finger, C3HC4 type domain containing protein, expressed
Chr.9	907368	907368	C	C	T	SNP	OS09G0110100	Exonic	CDS	Silent	LOC_Os09g02250	transposon protein, putative, unclassified, expressed
Chr.9	1537485	1537485	C	C	T	SNP	OS09G0120033	Intronic	NA	NA	LOC_Os09g03190	expressed protein
Chr.9	7009354	7009354	C	C	T	SNP	OS09G0294000	Exonic	CDS	Missense	LOC_Os09g12290	bifunctional aspartokinase/homoserine dehydrogenase, chloroplast precursor, putative, expressed
Chr.9	6997510	6997510	C	C	T	SNP	OS09G0294000	Intronic	NA	NA	LOC_Os09g12290	bifunctional aspartokinase/homoserine dehydrogenase, chloroplast precursor, putative, expressed
Chr.9	6999911	6999911	G	G	T	SNP	OS09G0294000	Intronic	NA	NA	LOC_Os09g12290	bifunctional aspartokinase/homoserine dehydrogenase, chloroplast precursor, putative, expressed
Chr.9	6999992	6999992	C	C	T	SNP	OS09G0294000	Intronic	NA	NA	LOC_Os09g12290	bifunctional aspartokinase/homoserine dehydrogenase, chloroplast precursor, putative, expressed
Chr.9	7009151	7009151	C	C	T	SNP	OS09G0294000	Intronic	NA	NA	LOC_Os09g12290	bifunctional aspartokinase/homoserine dehydrogenase, chloroplast precursor, putative, expressed
Chr.9	7247864	7247864	C	C	T	SNP	OS09G0298200	Exonic	CDS	Missense	LOC_Os09g12660	glucose-1-phosphate adenylyltransferase large subunit, chloroplast precursor, putative, expressed
Chr.9	9367918	9367918	G	G	T	SNP	OS09G0322000	Exonic	CDS	Silent	LOC_Os09g15330	transporter family protein, putative, expressed
Chr.9	9367511	9367511	C	C	T	SNP	OS09G0322000	Exonic	CDS	Missense	LOC_Os09g15330	transporter family protein, putative, expressed
Chr.9	9568215	9568215	C	C	T	SNP	OS09G0325700	Exonic	CDS	Missense	LOC_Os09g15670	protein phosphatase 2C, putative, expressed
Chr.9	9979107	9979107	C	C	T	SNP	OS09G0332700	Exonic	CDS	Silent	LOC_Os09g16330	pleiotropic drug resistance protein, putative, expressed
Chr.9	10523149	10523149	C	C	T	SNP	OS09G0341600	Exonic	CDS	Silent	LOC_Os09g17152	OsFBX319 - F-box domain containing protein, expressed
Chr.9	10524151	10524151	G	G	T	SNP	OS09G0341600	Exonic	CDS	Silent	LOC_Os09g17152	OsFBX319 - F-box domain containing protein, expressed
Chr.9	10524185	10524185	C	C	T	SNP	OS09G0341600	Exonic	CDS	Missense	LOC_Os09g17152	OsFBX319 - F-box domain containing protein, expressed
Chr.9	13569162	13569162	C	C	T	SNP	OS09G0392400	Exonic	3UTR	NA	LOC_Os09g22500	expressed protein
Chr.9	13569306	13569306	C	C	T	SNP	OS09G0392400	Exonic	3UTR	NA	LOC_Os09g22500	expressed protein
Chr.9	17133002	17133002	C	C	T	SNP	OS09G0455300	Exonic	CDS	Silent	LOC_Os09g28210	bHelix-loop-helix transcription factor, putative, expressed
Chr.9	17133037	17133037	C	C	T	SNP	OS09G0455300	Exonic	CDS	Missense	LOC_Os09g28210	bHelix-loop-helix transcription factor, putative, expressed
Chr.9	17903560	17903560	C	C	T	SNP	OS09G0470500	Exonic	CDS	Missense	LOC_Os09g29460	homeobox associated leucine zipper, putative, expressed
Chr.9	3656624	3656625	TA	TA	T	INDEL	OS09G0248100	Intronic	NA	NA	LOC_Os09g07350	fasciclin-like arabinogalactan protein 8 precursor, putative, expressed
Chr.9	7006258	7006259	TC	TC	T	INDEL	OS09G0294000	Intronic	NA	NA	LOC_Os09g12290	bifunctional aspartokinase/homoserine dehydrogenase, chloroplast precursor, putative, expressed
Chr.9	9369875	9369893	TTCTCTCTCTCTCTCTCTC	TTCTCTCTCTCTCTCTCTC	T	INDEL	OS09G0322000	Exonic	5UTR	NA	LOC_Os09g15330	transporter family protein, putative, expressed
Chr.9	13568833	13568838	TTTCCG	TTTCCG	T	INDEL	OS09G0392400	Intronic	NA	NA	LOC_Os09g22500	expressed protein
Chr.9	16934074	16934075	TA	TA	T	INDEL	OS09G0451500	Intronic	NA	NA	LOC_Os09g27830	OsPDIL2-3 protein disulfide isomerase PDIL2-3, expressed
Chr.9	16961373	16961376	TGTG	TGTG	T	INDEL	OS09G0452200	Intronic	NA	NA	LOC_Os09g27890	lysM domain-containing GPI-anchored protein precursor, putative, expressed
Chr.9	18671086	18671096	TTTGTATTGGA	TTTGTATTGGA	T	INDEL	OS09G0483400	Intronic	NA	NA	LOC_Os09g31031	ubiquitin family protein, putative, expressed
Chr.9	18944606	18944607	TA	TA	T	INDEL	OS09G0491596	Exonic	3UTR	NA	LOC_Os09g31446	expressed protein
Chr.8	23839230	23839230	A	A	T	SNP	OS08G0482100	Intronic	NA	NA	LOC_Os08g37610	expressed protein
Chr.8	11607462	11607462	C	C	T	SNP	OS08G0290700	Exonic	CDS	Missense	LOC_Os08g19420	O-methyltransferase, putative, expressed
Chr.8	5984795	5984795	G	G	T	SNP	OS08G0203100	Exonic	3UTR	NA	LOC_Os08g10290	SHR5-receptor-like kinase, putative, expressed
Chr.8	5984027	5984027	A	A	T	SNP	OS08G0203100	Intronic	NA	NA	LOC_Os08g10290	SHR5-receptor-like kinase, putative, expressed
Chr.8	26421163	26421163	G	G	T	SNP	OS08G0530400	Intronic	NA	NA	LOC_Os08g41830	sulfite oxidase, putative, expressed
Chr.8	26419208	26419208	G	G	T	SNP	OS08G0530400	Exonic	CDS	Silent	LOC_Os08g41830	sulfite oxidase, putative, expressed
Chr.8	14417543	14417543	G	G	T	SNP	OS08G0327400	Intronic	NA	NA	LOC_Os08g23810	enoyl-acyl-carrier-protein reductase NADH, chloroplast precursor, expressed
Chr.8	13955285	13955285	G	G	T	SNP	OS08G0320400	Intronic	NA	NA	LOC_Os08g23150	indole-3-glycerol phosphate synthase, chloroplast precursor, putative, expressed
Chr.8	11607768	11607768	G	G	T	SNP	OS08G0290700	Exonic	CDS	Missense	LOC_Os08g19420	O-methyltransferase, putative, expressed
Chr.8	11607532	11607532	G	G	T	SNP	OS08G0290700	Exonic	CDS	Missense	LOC_Os08g19420	O-methyltransferase, putative, expressed
Chr.8	9386295	9386295	G	G	T	SNP	OS08G0253800	Intronic	NA	NA	LOC_Os08g15420	CSLC3 - cellulose synthase-like family C, expressed
Chr.8	7367317	7367317	G	G	T	SNP	OS08G0221300	Exonic	CDS	NA	LOC_Os08g12449	expressed protein
Chr.8	5422387	5422387	G	G	T	SNP	OS08G0192900	Exonic	3UTR	NA	LOC_Os08g09350	gar2, putative, expressed
Chr.8	3423822	3423822	G	G	T	SNP	OS08G0158500	Intronic	NA	NA	LOC_Os08g06200	stomatin-like protein 2, putative, expressed
Chr.8	3422913	3422913	G	G	T	SNP	OS08G0158500	Exonic	CDS	Silent	LOC_Os08g06200	stomatin-like protein 2, putative, expressed
Chr.8	3422718	3422718	G	G	T	SNP	OS08G0158500	Intronic	NA	NA	LOC_Os08g06200	stomatin-like protein 2, putative, expressed
Chr.8	3422188	3422188	G	G	T	SNP	OS08G0158500	Intronic	NA	NA	LOC_Os08g06200	stomatin-like protein 2, putative, expressed
Chr.8	3422168	3422168	G	G	T	SNP	OS08G0158500	Intronic	NA	NA	LOC_Os08g06200	stomatin-like protein 2, putative, expressed
Chr.8	3421999	3421999	G	G	T	SNP	OS08G0158500	Intronic	NA	NA	LOC_Os08g06200	stomatin-like protein 2, putative, expressed
Chr.8	3421970	3421970	G	G	T	SNP	OS08G0158500	Intronic	NA	NA	LOC_Os08g06200	stomatin-like protein 2, putative, expressed
Chr.8	3421160	3421160	G	G	T	SNP	OS08G0158500	Intronic	NA	NA	LOC_Os08g06200	stomatin-like protein 2, putative, expressed
Chr.8	3421140	3421140	G	G	T	SNP	OS08G0158500	Intronic	NA	NA	LOC_Os08g06200	stomatin-like protein 2, putative, expressed
Chr.8	3281640	3281640	G	G	T	SNP	OS08G0156600	Intronic	NA	NA	LOC_Os08g06010	transporter, major facilitator family, putative, expressed
Chr.8	2130036	2130036	G	G	T	SNP	OS08G0137800	Exonic	CDS	Silent	LOC_Os08g04340	plastocyanin-like domain containing protein, putative, expressed
Chr.8	2129936	2129936	G	G	T	SNP	OS08G0137800	Intronic	NA	NA	LOC_Os08g04340	plastocyanin-like domain containing protein, putative, expressed

Chr.8	753152	753152	G	G	T	SNP	OS08G0114100	Exonic	3UTR	NA	LOC_Os08g02210	expressed protein
Chr.8	26422130	26422130	C	C	T	SNP	OS08G0530400	Exonic	3UTR	NA	LOC_Os08g41830	sulfite oxidase, putative, expressed
Chr.8	26420662	26420662	C	C	T	SNP	OS08G0530400	Intronic	NA	NA	LOC_Os08g41830	sulfite oxidase, putative, expressed
Chr.8	26392532	26392532	C	C	T	SNP	OS08G0530000	Exonic	CDS	Silent	LOC_Os08g41790	phosphoribulokinase/Uridine kinase family protein, expressed
Chr.8	26391797	26391797	C	C	T	SNP	OS08G0530000	Intronic	NA	NA	LOC_Os08g41790	phosphoribulokinase/Uridine kinase family protein, expressed
Chr.8	13956216	13956216	C	C	T	SNP	OS08G0320400	Exonic	3UTR	NA	LOC_Os08g23150	indole-3-glycerol phosphate synthase, chloroplast precursor, putative, expressed
Chr.8	12268373	12268373	C	C	T	SNP	OS08G0299400	Intronic	NA	NA	LOC_Os08g20420	MGD2, putative, expressed
Chr.8	12268216	12268216	C	C	T	SNP	OS08G0299400	Intronic	NA	NA	LOC_Os08g20420	MGD2, putative, expressed
Chr.8	11608557	11608557	C	C	T	SNP	OS08G0290700	Exonic	CDS	Silent	LOC_Os08g19420	O-methyltransferase, putative, expressed
Chr.8	11608292	11608292	C	C	T	SNP	OS08G0290700	Intronic	NA	NA	LOC_Os08g19420	O-methyltransferase, putative, expressed
Chr.8	8762316	8762316	C	C	T	SNP	OS08G0243500	Intronic	NA	NA	LOC_Os08g14570	NADPH reductase, putative, expressed
Chr.8	8329230	8329230	C	C	T	SNP	OS08G0237000	Exonic	3UTR	NA	LOC_Os08g13920	glycosyl hydrolases family 16, putative, expressed
Chr.8	7941232	7941232	C	C	T	SNP	OS08G0230200	Intronic	NA	NA	LOC_Os08g13350	expressed protein
Chr.8	7366854	7366854	C	C	T	SNP	OS08G0221300	Intronic	NA	NA	LOC_Os08g12449	expressed protein
Chr.8	7366662	7366662	C	C	T	SNP	OS08G0221300	Intronic	NA	NA	LOC_Os08g12449	expressed protein
Chr.8	7366222	7366222	C	C	T	SNP	OS08G0221300	Intronic	NA	NA	LOC_Os08g12449	expressed protein
Chr.8	6208355	6208355	C	C	T	SNP	OS08G0206400	Intronic	NA	NA	LOC_Os08g10550	potassium transporter, putative, expressed
Chr.8	6206040	6206040	C	C	T	SNP	OS08G0206400	Exonic	5UTR	NA	LOC_Os08g10550	potassium transporter, putative, expressed
Chr.8	5142324	5142324	C	C	T	SNP	OS08G0187800	Intronic	NA	NA	LOC_Os08g08840	phosphate/phosphate translocator, putative, expressed
Chr.8	3724478	3724478	C	C	T	SNP	OS08G0163100	Intronic	NA	NA	LOC_Os08g06600	nuf2 family protein, expressed
Chr.8	3723351	3723351	C	C	T	SNP	OS08G0163100	Intronic	NA	NA	LOC_Os08g06600	nuf2 family protein, expressed
Chr.8	3424521	3424521	C	C	T	SNP	OS08G0158500	Exonic	3UTR	NA	LOC_Os08g06200	stomatin-like protein 2, putative, expressed
Chr.8	3424467	3424467	C	C	T	SNP	OS08G0158500	Exonic	3UTR	NA	LOC_Os08g06200	stomatin-like protein 2, putative, expressed
Chr.8	3424284	3424284	C	C	T	SNP	OS08G0158500	Exonic	CDS	Missense	LOC_Os08g06200	stomatin-like protein 2, putative, expressed
Chr.8	3424276	3424276	C	C	T	SNP	OS08G0158500	Exonic	CDS	Silent	LOC_Os08g06200	stomatin-like protein 2, putative, expressed
Chr.8	3423812	3423812	C	C	T	SNP	OS08G0158500	Intronic	NA	NA	LOC_Os08g06200	stomatin-like protein 2, putative, expressed
Chr.8	3423608	3423608	C	C	T	SNP	OS08G0158500	Intronic	NA	NA	LOC_Os08g06200	stomatin-like protein 2, putative, expressed
Chr.8	3423309	3423309	C	C	T	SNP	OS08G0158500	Intronic	NA	NA	LOC_Os08g06200	stomatin-like protein 2, putative, expressed
Chr.8	3423290	3423290	C	C	T	SNP	OS08G0158500	Intronic	NA	NA	LOC_Os08g06200	stomatin-like protein 2, putative, expressed
Chr.8	3423213	3423213	C	C	T	SNP	OS08G0158500	Intronic	NA	NA	LOC_Os08g06200	stomatin-like protein 2, putative, expressed
Chr.8	3423020	3423020	C	C	T	SNP	OS08G0158500	Intronic	NA	NA	LOC_Os08g06200	stomatin-like protein 2, putative, expressed
Chr.8	3422892	3422892	C	C	T	SNP	OS08G0158500	Intronic	NA	NA	LOC_Os08g06200	stomatin-like protein 2, putative, expressed
Chr.8	3421992	3421992	C	C	T	SNP	OS08G0158500	Intronic	NA	NA	LOC_Os08g06200	stomatin-like protein 2, putative, expressed
Chr.8	3421971	3421971	C	C	T	SNP	OS08G0158500	Intronic	NA	NA	LOC_Os08g06200	stomatin-like protein 2, putative, expressed
Chr.8	3421788	3421788	C	C	T	SNP	OS08G0158500	Intronic	NA	NA	LOC_Os08g06200	stomatin-like protein 2, putative, expressed
Chr.8	3421492	3421492	C	C	T	SNP	OS08G0158500	Intronic	NA	NA	LOC_Os08g06200	stomatin-like protein 2, putative, expressed
Chr.8	3421449	3421449	C	C	T	SNP	OS08G0158500	Intronic	NA	NA	LOC_Os08g06200	stomatin-like protein 2, putative, expressed
Chr.8	3390734	3390734	C	C	T	SNP	OS08G0157900	Exonic	CDS	Missense	LOC_Os08g06140	no apical meristem protein, putative, expressed
Chr.8	3370617	3370617	C	C	T	SNP	OS08G0157600	Intronic	NA	NA	LOC_Os08g06110	MYB family transcription factor, putative, expressed
Chr.8	3368840	3368840	C	C	T	SNP	OS08G0157600	Intronic	NA	NA	LOC_Os08g06110	MYB family transcription factor, putative, expressed
Chr.8	3366428	3366428	C	C	T	SNP	OS08G0157600	Exonic	5UTR	NA	LOC_Os08g06110	MYB family transcription factor, putative, expressed
Chr.8	3364591	3364591	C	C	T	SNP	OS08G0157600	Intronic	NA	NA	LOC_Os08g06110	MYB family transcription factor, putative, expressed
Chr.8	3363319	3363319	C	C	T	SNP	OS08G0157600	Intronic	NA	NA	LOC_Os08g06110	MYB family transcription factor, putative, expressed
Chr.8	3339179	3339179	C	C	T	SNP	OS08G0157500	Intronic	NA	NA	LOC_Os08g06100	O-methyltransferase, putative, expressed
Chr.8	3338851	3338851	C	C	T	SNP	OS08G0157500	Intronic	NA	NA	LOC_Os08g06100	O-methyltransferase, putative, expressed
Chr.8	3295563	3295563	C	C	T	SNP	OS08G0156800	Intronic	NA	NA	LOC_Os08g06040	60S ribosomal protein L34, putative, expressed
Chr.8	3295459	3295459	C	C	T	SNP	OS08G0156800	Exonic	CDS	Silent	LOC_Os08g06040	60S ribosomal protein L34, putative, expressed
Chr.8	3295281	3295281	C	C	T	SNP	OS08G0156800	Intronic	NA	NA	LOC_Os08g06040	60S ribosomal protein L34, putative, expressed
Chr.8	3294885	3294885	C	C	T	SNP	OS08G0156800	Intronic	NA	NA	LOC_Os08g06040	60S ribosomal protein L34, putative, expressed
Chr.8	2513415	2513415	C	C	T	SNP	OS08G0144400	Exonic	3UTR	NA	LOC_Os08g04920	OsDegp11 - Putative Deg protease homologue, expressed
Chr.8	2511186	2511186	C	C	T	SNP	OS08G0144400	Intronic	NA	NA	LOC_Os08g04920	OsDegp11 - Putative Deg protease homologue, expressed
Chr.8	2511139	2511139	C	C	T	SNP	OS08G0144400	Intronic	NA	NA	LOC_Os08g04920	OsDegp11 - Putative Deg protease homologue, expressed
Chr.8	2175743	2175743	C	C	T	SNP	OS08G0139000	Exonic	CDS	Missense	LOC_Os08g04440	RNA recognition motif containing protein, putative, expressed
Chr.8	2175385	2175385	C	C	T	SNP	OS08G0139000	Exonic	3UTR	NA	LOC_Os08g04440	RNA recognition motif containing protein, putative, expressed
Chr.8	1564740	1564740	C	C	T	SNP	OS08G0127100	Intronic	NA	NA	LOC_Os08g03350	amino acid transporter, putative, expressed
Chr.8	1561853	1561853	C	C	T	SNP	OS08G0127100	Exonic	5UTR	NA	LOC_Os08g03350	amino acid transporter, putative, expressed
Chr.8	690455	690455	C	C	T	SNP	OS08G0112900	Intronic	NA	NA	LOC_Os08g02094	GDSL-like lipase/acylhydrolase, putative, expressed
Chr.8	690317	690317	C	C	T	SNP	OS08G0112900	Intronic	NA	NA	LOC_Os08g02094	GDSL-like lipase/acylhydrolase, putative, expressed
Chr.8	688155	688155	C	C	T	SNP	OS08G0112900	Intronic	NA	NA	LOC_Os08g02094	GDSL-like lipase/acylhydrolase, putative, expressed
Chr.8	26391401	26391401	A	A	T	SNP	OS08G0530000	Intronic	NA	NA	LOC_Os08g41790	phosphoribulokinase/Uridine kinase family protein, expressed
Chr.8	13955971	13955971	A	A	T	SNP	OS08G0320400	Exonic	3UTR	NA	LOC_Os08g23150	indole-3-glycerol phosphate synthase, chloroplast precursor, putative, expressed
Chr.8	8764691	8764691	A	A	T	SNP	OS08G0243500	Intronic	NA	NA	LOC_Os08g14570	NADPH reductase, putative, expressed
Chr.8	5422426	5422426	A	A	T	SNP	OS08G0192900	Exonic	3UTR	NA	LOC_Os08g09350	gar2, putative, expressed
Chr.8	5366279	5366279	A	A	T	SNP	OS08G0191600	Exonic	3UTR	NA	LOC_Os08g09240	autophagy-related protein, putative, expressed
Chr.8	3548190	3548190	A	A	T	SNP	OS08G0160500	Exonic	5UTR	NA	LOC_Os08g06380	CSLF6 - cellulose synthase-like family F; beta1,3;1,4 glucan synthase, expressed
Chr.8	3424549	3424549	A	A	T	SNP	OS08G0158500	Exonic	3UTR	NA	LOC_Os08g06200	stomatin-like protein 2, putative, expressed
Chr.8	3424246	3424246	A	A	T	SNP	OS08G0158500	Exonic	CDS	Silent	LOC_Os08g06200	stomatin-like protein 2, putative, expressed
Chr.8	3423986	3423986	A	A	T	SNP	OS08G0158500	Intronic	NA	NA	LOC_Os08g06200	stomatin-like protein 2, putative, expressed
Chr.8	3423286	3423286	A	A	T	SNP	OS08G0158500	Intronic	NA	NA	LOC_Os08g06200	stomatin-like protein 2, putative, expressed
Chr.8	3422015	3422015	A	A	T	SNP	OS08G0158500	Intronic	NA	NA	LOC_Os08g06200	stomatin-like protein 2, putative, expressed
Chr.8	3421995	3421995	A	A	T	SNP	OS08G0158500	Intronic	NA	NA	LOC_Os08g06200	stomatin-like protein 2, putative, expressed
Chr.8	3421501	3421501	A	A	T	SNP	OS08G0158500	Intronic	NA	NA	LOC_Os08g06200	stomatin-like protein 2, putative, expressed
Chr.8	3370887	3370887	A	A	T	SNP	OS08G0157600	Intronic	NA	NA	LOC_Os08g06110	MYB family transcription factor, putative, expressed
Chr.8	3368626	3368626	A	A	T	SNP	OS08G0157600	Intronic	NA	NA	LOC_Os08g06110	MYB family transcription factor, putative, expressed
Chr.8	3295536	3295536	A	A	T	SNP	OS08G0156800	Intronic	NA	NA	LOC_Os08g06040	60S ribosomal protein L34, putative, expressed
Chr.8	3293821	3293821	A	A	T	SNP	OS08G0156800	Intronic	NA	NA	LOC_Os08g06040	60S ribosomal protein L34, putative, expressed
Chr.8	2176759	2176759	A	A	T	SNP	OS08G0139000	Intronic	NA	NA	LOC_Os08g04440	RNA recognition motif containing protein, putative, expressed
Chr.8	582357	582357	C	C	T	SNP	OS08G0111200	Exonic	CDS	Silent	LOC_Os08g01940	non-lysosomal glucosylceramidase, putative, expressed
Chr.8	688899	688899	G	G	T	SNP	OS08G0112900	Intronic	NA	NA	LOC_Os08g02094	GDSL-like lipase/acylhydrolase, putative, expressed
Chr.8	692171	692171	G	G	T	SNP	OS08G0112900	Intronic	NA	NA	LOC_Os08g02094	GDSL-like lipase/acylhydrolase, putative, expressed
Chr.8	692332	692332	C	C	T	SNP	OS08G0112900	Intronic	NA	NA	LOC_Os08g02094	GDSL-like lipase/acylhydrolase, putative, expressed
Chr.8	692361	692361	C	C	T	SNP	OS08G0112900	Intronic	NA	NA	LOC_Os08g02094	GDSL-like lipase/acylhydrolase, putative, expressed
Chr.8	752665	752665	G	G	T	SNP	OS08G0114100	Intronic	NA	NA	LOC_Os08g02210	expressed protein
Chr.8	1562487	1562487	A	A	T	SNP	OS08G0127100	Intronic	NA	NA	LOC_Os08g03350	amino acid transporter, putative, expressed
Chr.8	2130566	2130566	G	G	T	SNP	OS08G0137800	Exonic	3UTR	NA	LOC_Os08g04340	plastocyanin-like domain containing protein, putative, expressed

Chr.8	3295574	3295574	C	C	T	SNP	OS08G0156800	Exonic	CDS	Silent	LOC_Os08g06040	60S ribosomal protein L34, putative, expressed
Chr.8	3338505	3338505	G	G	T	SNP	OS08G0157500	Exonic	CDS	Silent	LOC_Os08g06100	O-methyltransferase, putative, expressed
Chr.8	3338360	3338360	C	C	T	SNP	OS08G0157500	Exonic	CDS	Missense	LOC_Os08g06100	O-methyltransferase, putative, expressed
Chr.8	3339321	3339321	C	C	T	SNP	OS08G0157500	Intronic	NA	NA	LOC_Os08g06100	O-methyltransferase, putative, expressed
Chr.8	3361372	3361372	A	A	T	SNP	OS08G0157600	Exonic	5UTR	NA	LOC_Os08g06110	MYB family transcription factor, putative, expressed
Chr.8	3424617	3424617	G	G	T	SNP	OS08G0158500	Exonic	3UTR	NA	LOC_Os08g06200	stomatin-like protein 2, putative, expressed
Chr.8	3424658	3424658	A	A	T	SNP	OS08G0158500	Exonic	3UTR	NA	LOC_Os08g06200	stomatin-like protein 2, putative, expressed
Chr.8	3424662	3424662	G	G	T	SNP	OS08G0158500	Exonic	3UTR	NA	LOC_Os08g06200	stomatin-like protein 2, putative, expressed
Chr.8	3423777	3423777	C	C	T	SNP	OS08G0158500	Exonic	CDS	Silent	LOC_Os08g06200	stomatin-like protein 2, putative, expressed
Chr.8	3421220	3421220	C	C	T	SNP	OS08G0158500	Intronic	NA	NA	LOC_Os08g06200	stomatin-like protein 2, putative, expressed
Chr.8	3421342	3421342	G	G	T	SNP	OS08G0158500	Intronic	NA	NA	LOC_Os08g06200	stomatin-like protein 2, putative, expressed
Chr.8	3421466	3421466	G	G	T	SNP	OS08G0158500	Intronic	NA	NA	LOC_Os08g06200	stomatin-like protein 2, putative, expressed
Chr.8	3421593	3421593	C	C	T	SNP	OS08G0158500	Intronic	NA	NA	LOC_Os08g06200	stomatin-like protein 2, putative, expressed
Chr.8	3423233	3423233	C	C	T	SNP	OS08G0158500	Intronic	NA	NA	LOC_Os08g06200	stomatin-like protein 2, putative, expressed
Chr.8	3461886	3461886	C	C	T	SNP	OS08G0159500	Exonic	CDS	Silent	LOC_Os08g06280	LSD1 zinc finger domain containing protein, expressed
Chr.8	3548456	3548456	G	G	T	SNP	OS08G0160500	Exonic	5UTR	NA	LOC_Os08g06380	CSLF6 - cellulose synthase-like family F; beta1,3;1,4 glucan synthase, expressed
Chr.8	7364760	7364760	C	C	T	SNP	OS08G0221300	Intronic	NA	NA	LOC_Os08g12449	expressed protein
Chr.8	7367115	7367115	G	G	T	SNP	OS08G0221300	Intronic	NA	NA	LOC_Os08g12449	expressed protein
Chr.8	8328655	8328655	C	C	T	SNP	OS08G0237000	Exonic	CDS	Silent	LOC_Os08g13920	glycosyl hydrolases family 16, putative, expressed
Chr.8	12269472	12269472	C	C	T	SNP	OS08G0299400	Exonic	3UTR	NA	LOC_Os08g20420	MGD2, putative, expressed
Chr.8	26421985	26421985	A	A	T	SNP	OS08G0530400	Exonic	3UTR	NA	LOC_Os08g41830	sulfite oxidase, putative, expressed
Chr.8	27479531	27479531	C	C	T	SNP	OS08G0547900	Exonic	CDS	Silent	LOC_Os08g43440	cytochrome P450, putative, expressed
Chr.8	27479588	27479588	C	C	T	SNP	OS08G0547900	Exonic	CDS	Silent	LOC_Os08g43440	cytochrome P450, putative, expressed
Chr.8	27479743	27479743	C	C	T	SNP	OS08G0547900	Exonic	CDS	Missense	LOC_Os08g43440	cytochrome P450, putative, expressed
Chr.8	687246	687247	TC	TC	T	INDEL	OS08G0112900	Intronic	NA	NA	LOC_Os08g02094	GDSL-like lipase/acylhydrolase, putative, expressed
Chr.8	1561872	1561877	TCCTCC	TCCTCC	T	INDEL	OS08G0127100	Exonic	5UTR	NA	LOC_Os08g03350	amino acid transporter, putative, expressed
Chr.8	2030575	2030579	TATAG	TATAG	T	INDEL	OS08G0135900	Intronic	NA	NA	LOC_Os08g04180	tryptophan synthase beta chain 1, putative, expressed
Chr.8	3281341	3281343	TTA	TTA	T	INDEL	OS08G0156600	Intronic	NA	NA	LOC_Os08g06010	transporter, major facilitator family, putative, expressed
Chr.8	3424611	3424612	TA	TA	T	INDEL	OS08G0158500	Exonic	3UTR	NA	LOC_Os08g06200	stomatin-like protein 2, putative, expressed
Chr.8	3421101	3421103	TTA	TTA	T	INDEL	OS08G0158500	Intronic	NA	NA	LOC_Os08g06200	stomatin-like protein 2, putative, expressed
Chr.8	3423870	3423872	TTC	TTC	T	INDEL	OS08G0158500	Intronic	NA	NA	LOC_Os08g06200	stomatin-like protein 2, putative, expressed
Chr.8	9386154	9386177	TAACCATGCATCCAGCCATCCACA	TAACCATGCATCCAGCCATCCACA	T	INDEL	OS08G0253800	Intronic	NA	NA	LOC_Os08g15420	CSLC3 - cellulose synthase-like family C, expressed
Chr.8	18320139	18320140	TA	TA	T	INDEL	OS08G0387700	Intronic	NA	NA	LOC_Os08g29809	resistance protein LR10, putative, expressed
Chr.8	21052555	21052566	TTCACTTGTTCA	TTCACTTGTTCA	T	INDEL	OS08G0434100	Exonic	3UTR	NA	LOC_Os08g33710	ribonuclease T2 family domain containing protein, expressed
Chr.6	2321422	2321422	C	C	T	SNP	OS06G0143900	Intronic	NA	NA	LOC_Os06g05180	coatomer subunit beta, putative, expressed
Chr.6	2322998	2322998	A	A	T	SNP	OS06G0143900	Intronic	NA	NA	LOC_Os06g05180	coatomer subunit beta, putative, expressed
Chr.6	2325882	2325882	A	A	T	SNP	OS06G0143900	Intronic	NA	NA	LOC_Os06g05180	coatomer subunit beta, putative, expressed
Chr.6	3362081	3362081	C	C	T	SNP	OS06G0166900	Intronic	NA	NA	LOC_Os06g07070	BRASSINOSTEROID INSENSITIVE 1-associated receptor kinase 1 precursor, putative, expressed
Chr.6	3643011	3643011	G	G	T	SNP	OS06G0172000	Exonic	3UTR	NA	LOC_Os06g07550	PPR repeat domain containing protein, putative, expressed
Chr.6	27137253	27137253	C	C	T	SNP	OS06G0659300	Intronic	NA	NA	LOC_Os06g44890	villin protein, putative, expressed
Chr.6	27137309	27137309	C	C	T	SNP	OS06G0659300	Intronic	NA	NA	LOC_Os06g44890	villin protein, putative, expressed
Chr.6	27140954	27140954	A	A	T	SNP	OS06G0659300	Intronic	NA	NA	LOC_Os06g44890	villin protein, putative, expressed
Chr.6	27141897	27141897	C	C	T	SNP	OS06G0659300	Intronic	NA	NA	LOC_Os06g44890	villin protein, putative, expressed
Chr.6	29762555	29762555	C	C	T	SNP	OS06G0704700	Exonic	3UTR	NA	LOC_Os06g49120	Complex I intermediate-associated protein 30 domain containing protein, putative, expressed
Chr.6	29765578	29765578	A	A	T	SNP	OS06G0704700	Intronic	NA	NA	LOC_Os06g49120	Complex I intermediate-associated protein 30 domain containing protein, putative, expressed
Chr.6	31139962	31139962	C	C	T	SNP	OS06G0730600	Intronic	NA	NA	LOC_Os06g51410	OsPOP15 - Putative Prolyl Oligopeptidase homologue, expressed
Chr.6	31142963	31142963	C	C	T	SNP	OS06G0730600	Intronic	NA	NA	LOC_Os06g51410	OsPOP15 - Putative Prolyl Oligopeptidase homologue, expressed
Chr.6	3447982	3447988	TAATAAA	TAATAAA	T	INDEL	OS06G0168500	Intronic	NA	NA	LOC_Os06g07200	syntaxin, putative, expressed
Chr.6	679725	679725	G	G	T	SNP	OS06G0112100	Exonic	CDS	Missense	LOC_Os06g02210	bark storage protein A precursor, putative, expressed
Chr.6	1362190	1362190	G	G	T	SNP	OS06G0125200	Exonic	3UTR	NA	LOC_Os06g03520	DUF581 domain containing protein, expressed
Chr.6	1555588	1555588	G	G	T	SNP	OS06G0129400	Intronic	NA	NA	LOC_Os06g03860	uncharacterized membrane protein, putative, expressed
Chr.6	1556464	1556464	G	G	T	SNP	OS06G0129400	Exonic	5UTR	NA	LOC_Os06g03860	uncharacterized membrane protein, putative, expressed
Chr.6	1558527	1558527	G	G	T	SNP	OS06G0129400	Intronic	NA	NA	LOC_Os06g03860	uncharacterized membrane protein, putative, expressed
Chr.6	2000875	2000875	G	G	T	SNP	OS06G0137500	Exonic	3UTR	NA	LOC_Os06g04600	Brix domain containing protein, putative, expressed
Chr.6	2987510	2987510	G	G	T	SNP	OS06G0158500	Intronic	NA	NA	LOC_Os06g06400	NBS-LRR type disease resistance protein, putative, expressed
Chr.6	19614682	19614682	G	G	T	SNP	OS06G0528600	Intronic	NA	NA	LOC_Os06g33710	spermidine synthase, putative, expressed
Chr.6	19619314	19619314	G	G	T	SNP	OS06G0528600	Exonic	CDS	Missense	LOC_Os06g33710	spermidine synthase, putative, expressed
Chr.6	21895898	21895898	G	G	T	SNP	OS06G0567200	Intronic	NA	NA	LOC_Os06g37080	L-ascorbate oxidase precursor, putative, expressed
Chr.6	23468021	23468021	G	G	T	SNP	OS06G0596300	Exonic	5UTR	NA	LOC_Os06g39520	myristoyl-acyl carrier protein thioesterase, chloroplast precursor, putative, expressed
Chr.6	23470209	23470209	G	G	T	SNP	OS06G0596300	Exonic	3UTR	NA	LOC_Os06g39520	myristoyl-acyl carrier protein thioesterase, chloroplast precursor, putative, expressed
Chr.6	23470556	23470556	G	G	T	SNP	OS06G0596300	Exonic	3UTR	NA	LOC_Os06g39520	myristoyl-acyl carrier protein thioesterase, chloroplast precursor, putative, expressed
Chr.6	23855470	23855470	G	G	T	SNP	OS06G0603000	Intronic	NA	NA	LOC_Os06g40080	heme oxygenase 1, putative, expressed
Chr.6	30306771	30306771	G	G	T	SNP	OS06G0714200	Intronic	NA	NA	LOC_Os06g50030	CAMK_CAMK_like.30 - CAMK includes calcium/calmodulin depedent protein kinases, expressed
Chr.6	299549	299549	C	C	T	SNP	OS06G0104300	Intronic	NA	NA	LOC_Os06g01490	monocopper oxidase, putative, expressed
Chr.6	415441	415441	C	C	T	SNP	OS06G0106100	Intronic	NA	NA	LOC_Os06g01700	CWC15 homolog A, putative, expressed
Chr.6	1556156	1556156	C	C	T	SNP	OS06G0129400	Intronic	NA	NA	LOC_Os06g03860	uncharacterized membrane protein, putative, expressed
Chr.6	1556787	1556787	C	C	T	SNP	OS06G0129400	Exonic	CDS	Silent	LOC_Os06g03860	uncharacterized membrane protein, putative, expressed
Chr.6	1556956	1556956	C	C	T	SNP	OS06G0129400	Intronic	NA	NA	LOC_Os06g03860	uncharacterized membrane protein, putative, expressed
Chr.6	1557745	1557745	C	C	T	SNP	OS06G0129400	Intronic	NA	NA	LOC_Os06g03860	uncharacterized membrane protein, putative, expressed
Chr.6	1559840	1559840	C	C	T	SNP	OS06G0129400	Intronic	NA	NA	LOC_Os06g03860	uncharacterized membrane protein, putative, expressed
Chr.6	1560319	1560319	C	C	T	SNP	OS06G0129400	Exonic	CDS	Missense	LOC_Os06g03860	uncharacterized membrane protein, putative, expressed
Chr.6	1560862	1560862	C	C	T	SNP	OS06G0129400	Exonic	3UTR	NA	LOC_Os06g03860	uncharacterized membrane protein, putative, expressed
Chr.6	3197725	3197725	C	C	T	SNP	OS06G0163300	Intronic	NA	NA	LOC_Os06g06780	harpin-induced protein, putative, expressed
Chr.6	4869572	4869572	C	C	T	SNP	OS06G0195800	Intronic	NA	NA	LOC_Os06g09560	heat shock protein DnaJ, putative, expressed
Chr.6	5681932	5681932	C	C	T	SNP	OS06G0211200	Exonic	3UTR	NA	LOC_Os06g10880	bZIP transcription factor, putative, expressed
Chr.6	6103027	6103027	C	C	T	SNP	OS06G0218900	Exonic	CDS	NA	LOC_Os06g11520	LMBR1 integral membrane protein, putative, expressed
Chr.6	6103259	6103259	C	C	T	SNP	OS06G0218900	Intronic	NA	NA	LOC_Os06g11520	LMBR1 integral membrane protein, putative, expressed
Chr.6	6104256	6104256	C	C	T	SNP	OS06G0218900	Intronic	NA	NA	LOC_Os06g11520	LMBR1 integral membrane protein, putative, expressed
Chr.6	6205629	6205629	C	C	T	SNP	OS06G0220500	Exonic	CDS	Silent	LOC_Os06g11720	cytokinin-O-glucosyltransferase 2, putative, expressed
Chr.6	8179375	8179375	C	C	T	SNP	OS06G0256900	Exonic	3UTR	NA	LOC_Os06g14540	endoglucanase, putative, expressed
Chr.6	8763272	8763272	C	C	T	SNP	OS06G0265000	Intronic	NA	NA	LOC_Os06g15420	asparagine synthetase, putative, expressed
Chr.6	10686772	10686772	C	C	T	SNP	OS06G0291500	Exonic	3UTR	NA	LOC_Os06g18820	serine threonine kinase, putative, expressed
Chr.6	10781412	10781412	C	C	T	SNP	OS06G0293500	Exonic	CDS	Missense	LOC_Os06g18960	embryogenesis transmembrane protein, putative, expressed
Chr.6	13477896	13477896	C	C	T	SNP	OS06G0338200	Intronic	NA	NA	LOC_Os06g23114	copper methylamine oxidase precursor, putative, expressed
Chr.6	13483132	13483132	C	C	T	SNP	OS06G0338200	Intronic	NA	NA	LOC_Os06g23114	copper methylamine oxidase precursor, putative, expressed

Chr.6	13483582	13483582	C	C	T	SNP	OS06G0338200	Exonic	CDS	Missense	LOC_Os06g23114	copper methylamine oxidase precursor, putative, expressed
Chr.6	13484043	13484043	C	C	T	SNP	OS06G0338200	Intronic	NA	NA	LOC_Os06g23114	copper methylamine oxidase precursor, putative, expressed
Chr.6	15781555	15781555	C	C	T	SNP	OS06G0473000	Exonic	3UTR	NA	LOC_Os06g27860	poor homologous synapsis 1 protein, putative, expressed
Chr.6	16270610	16270610	C	C	T	SNP	OS06G0480000	Exonic	5UTR	NA	LOC_Os06g28590	bg55, putative, expressed
Chr.6	19617175	19617175	C	C	T	SNP	OS06G0528600	Intronic	NA	NA	LOC_Os06g33710	spermidine synthase, putative, expressed
Chr.6	19619566	19619566	C	C	T	SNP	OS06G0528600	Exonic	3UTR	NA	LOC_Os06g33710	spermidine synthase, putative, expressed
Chr.6	21601510	21601510	C	C	T	SNP	OS06G0562600	Exonic	CDS	Silent	LOC_Os06g36700	T-complex protein, putative, expressed
Chr.6	21893721	21893721	C	C	T	SNP	OS06G0567200	Intronic	NA	NA	LOC_Os06g37080	L-ascorbate oxidase precursor, putative, expressed
Chr.6	21896356	21896356	C	C	T	SNP	OS06G0567200	Intronic	NA	NA	LOC_Os06g37080	L-ascorbate oxidase precursor, putative, expressed
Chr.6	21896447	21896447	C	C	T	SNP	OS06G0567200	Intronic	NA	NA	LOC_Os06g37080	L-ascorbate oxidase precursor, putative, expressed
Chr.6	22352803	22352803	C	C	T	SNP	OS06G0575400	Exonic	CDS	Missense	LOC_Os06g37750	S-locus-like receptor protein kinase, putative, expressed
Chr.6	23105950	23105950	C	C	T	SNP	OS06G0589300	Intronic	NA	NA	LOC_Os06g38950	ABC transporter, ATP-binding protein, putative, expressed
Chr.6	23855607	23855607	C	C	T	SNP	OS06G0603000	Intronic	NA	NA	LOC_Os06g40080	heme oxygenase 1, putative, expressed
Chr.6	24145318	24145318	C	C	T	SNP	OS06G0607200	Intronic	NA	NA	LOC_Os06g40510	CRAL/TRIO domain containing protein, expressed
Chr.6	26377284	26377284	C	C	T	SNP	OS06G0646000	Intronic	NA	NA	LOC_Os06g43800	methyltransferase domain containing protein, putative, expressed
Chr.6	27142488	27142488	C	C	T	SNP	OS06G0659300	Intronic	NA	NA	LOC_Os06g44890	villin protein, putative, expressed
Chr.6	28193708	28193708	C	C	T	SNP	OS06G0678200	Exonic	CDS	Silent	LOC_Os06g46450	polyprenyl synthetase, putative, expressed
Chr.6	28826692	28826692	C	C	T	SNP	OS06G0691200	Exonic	3UTR	NA	LOC_Os06g47600	thaumatin family domain containing protein, expressed
Chr.6	30306604	30306604	C	C	T	SNP	OS06G0714200	Intronic	NA	NA	LOC_Os06g50030	CAMK_CAMK_like.30 - CAMK includes calcium/calmodulin depedent protein kinases, expressed
Chr.6	1559712	1559712	A	A	T	SNP	OS06G0129400	Intronic	NA	NA	LOC_Os06g03860	uncharacterized membrane protein, putative, expressed
Chr.6	3198141	3198141	A	A	T	SNP	OS06G0163300	Intronic	NA	NA	LOC_Os06g06780	harpin-induced protein, putative, expressed
Chr.6	13480568	13480568	A	A	T	SNP	OS06G0338200	Intronic	NA	NA	LOC_Os06g23114	copper methylamine oxidase precursor, putative, expressed
Chr.6	15781523	15781523	A	A	T	SNP	OS06G0473000	Exonic	3UTR	NA	LOC_Os06g27860	poor homologous synapsis 1 protein, putative, expressed
Chr.6	16269629	16269629	A	A	T	SNP	OS06G0480000	Intronic	NA	NA	LOC_Os06g28590	bg55, putative, expressed
Chr.6	19614943	19614943	A	A	T	SNP	OS06G0528600	Intronic	NA	NA	LOC_Os06g33710	spermidine synthase, putative, expressed
Chr.6	19617931	19617931	A	A	T	SNP	OS06G0528600	Exonic	CDS	Missense	LOC_Os06g33710	spermidine synthase, putative, expressed
Chr.6	19619393	19619393	A	A	T	SNP	OS06G0528600	Exonic	3UTR	NA	LOC_Os06g33710	spermidine synthase, putative, expressed
Chr.6	21598896	21598896	A	A	T	SNP	OS06G0562600	Intronic	NA	NA	LOC_Os06g36700	T-complex protein, putative, expressed
Chr.6	21599697	21599697	A	A	T	SNP	OS06G0562600	Intronic	NA	NA	LOC_Os06g36700	T-complex protein, putative, expressed
Chr.6	22352789	22352789	A	A	T	SNP	OS06G0575400	Exonic	CDS	Missense	LOC_Os06g37750	S-locus-like receptor protein kinase, putative, expressed
Chr.6	23467899	23467899	A	A	T	SNP	OS06G0596300	Intronic	NA	NA	LOC_Os06g39520	myristoyl-acyl carrier protein thioesterase, chloroplast precursor, putative, expressed
Chr.6	23470621	23470621	A	A	T	SNP	OS06G0596300	Exonic	3UTR	NA	LOC_Os06g39520	myristoyl-acyl carrier protein thioesterase, chloroplast precursor, putative, expressed
Chr.6	23856872	23856872	A	A	T	SNP	OS06G0603000	Intronic	NA	NA	LOC_Os06g40080	heme oxygenase 1, putative, expressed
Chr.6	24399590	24399590	A	A	T	SNP	OS06G0611900	Exonic	3UTR	NA	LOC_Os06g40940	glycine dehydrogenase, putative, expressed
Chr.6	25925011	25925011	A	A	T	SNP	OS06G0638000	Intronic	NA	NA	LOC_Os06g43120	zinc finger C-x8-C-x5-C-x3-H type family protein, expressed
Chr.6	27140881	27140881	A	A	T	SNP	OS06G0659300	Intronic	NA	NA	LOC_Os06g44890	villin protein, putative, expressed
Chr.6	643735	643735	G	G	T	SNP	OS06G0111500	Exonic	5UTR	NA	LOC_Os06g02144	6-phosphogluconate dehydrogenase, decarboxylating, putative, expressed
Chr.6	1415542	1415542	C	C	T	SNP	OS06G0126500	Exonic	CDS	Silent	LOC_Os06g03640	BAG domain containing protein, expressed
Chr.6	1470901	1470901	C	C	T	SNP	OS06G0127900	Exonic	5UTR	NA	LOC_Os06g03720	ribonucleoside-diphosphate reductase small chain, putative, expressed
Chr.6	1556203	1556203	A	A	T	SNP	OS06G0129400	Intronic	NA	NA	LOC_Os06g03860	uncharacterized membrane protein, putative, expressed
Chr.6	1784605	1784605	G	G	T	SNP	OS06G0133400	Exonic	CDS	NA	LOC_Os06g04230	expressed protein
Chr.6	2323253	2323253	A	A	T	SNP	OS06G0143900	Intronic	NA	NA	LOC_Os06g05180	coatomer subunit beta, putative, expressed
Chr.6	4333520	4333520	C	C	T	SNP	OS06G0186100	Exonic	CDS	Silent	LOC_Os06g08690	leucine-rich repeat receptor protein kinase EXS precursor, putative, expressed
Chr.6	4333053	4333053	G	G	T	SNP	OS06G0186100	Exonic	CDS	Missense	LOC_Os06g08690	leucine-rich repeat receptor protein kinase EXS precursor, putative, expressed
Chr.6	4679647	4679647	C	C	T	SNP	OS06G0192800	Exonic	CDS	Missense	LOC_Os06g09310	zinc finger, C3HC4 type domain containing protein, expressed
Chr.6	4869361	4869361	G	G	T	SNP	OS06G0195800	Intronic	NA	NA	LOC_Os06g09560	heat shock protein DnaJ, putative, expressed
Chr.6	5678115	5678115	C	C	T	SNP	OS06G0211200	Exonic	CDS	Silent	LOC_Os06g10880	bZIP transcription factor, putative, expressed
Chr.6	5886035	5886035	C	C	T	SNP	OS06G0215600	Exonic	CDS	Missense	LOC_Os06g11210	12-oxophytodienoate reductase, putative, expressed
Chr.6	7230299	7230299	C	C	T	SNP	OS06G0239200	Exonic	CDS	Missense	LOC_Os06g13190	expressed protein
Chr.6	9094665	9094665	G	G	T	SNP	OS06G0270900	Intronic	NA	NA	LOC_Os06g15990	aldehyde dehydrogenase, putative, expressed
Chr.6	9284780	9284780	C	C	T	SNP	OS06G0274200	Intronic	NA	NA	LOC_Os06g16290	ribosomal protein L7Ae, putative, expressed
Chr.6	10784340	10784340	C	C	T	SNP	OS06G0293500	Exonic	CDS	Missense	LOC_Os06g18960	embryogenesis transmembrane protein, putative, expressed
Chr.6	13477650	13477650	A	A	T	SNP	OS06G0338200	Intronic	NA	NA	LOC_Os06g23114	copper methylamine oxidase precursor, putative, expressed
Chr.6	13478214	13478214	C	C	T	SNP	OS06G0338200	Intronic	NA	NA	LOC_Os06g23114	copper methylamine oxidase precursor, putative, expressed
Chr.6	13479095	13479095	C	C	T	SNP	OS06G0338200	Intronic	NA	NA	LOC_Os06g23114	copper methylamine oxidase precursor, putative, expressed
Chr.6	13480573	13480573	C	C	T	SNP	OS06G0338200	Intronic	NA	NA	LOC_Os06g23114	copper methylamine oxidase precursor, putative, expressed
Chr.6	13480715	13480715	A	A	T	SNP	OS06G0338200	Intronic	NA	NA	LOC_Os06g23114	copper methylamine oxidase precursor, putative, expressed
Chr.6	13480810	13480810	C	C	T	SNP	OS06G0338200	Intronic	NA	NA	LOC_Os06g23114	copper methylamine oxidase precursor, putative, expressed
Chr.6	13480971	13480971	C	C	T	SNP	OS06G0338200	Intronic	NA	NA	LOC_Os06g23114	copper methylamine oxidase precursor, putative, expressed
Chr.6	13481066	13481066	C	C	T	SNP	OS06G0338200	Intronic	NA	NA	LOC_Os06g23114	copper methylamine oxidase precursor, putative, expressed
Chr.6	13481175	13481175	C	C	T	SNP	OS06G0338200	Intronic	NA	NA	LOC_Os06g23114	copper methylamine oxidase precursor, putative, expressed
Chr.6	13481346	13481346	C	C	T	SNP	OS06G0338200	Intronic	NA	NA	LOC_Os06g23114	copper methylamine oxidase precursor, putative, expressed
Chr.6	23113013	23113013	G	G	T	SNP	OS06G0589300	Exonic	5UTR	NA	LOC_Os06g38950	ABC transporter, ATP-binding protein, putative, expressed
Chr.6	23470554	23470554	C	C	T	SNP	OS06G0596300	Exonic	3UTR	NA	LOC_Os06g39520	myristoyl-acyl carrier protein thioesterase, chloroplast precursor, putative, expressed
Chr.6	23468045	23468045	C	C	T	SNP	OS06G0596300	Exonic	5UTR	NA	LOC_Os06g39520	myristoyl-acyl carrier protein thioesterase, chloroplast precursor, putative, expressed
Chr.6	23468647	23468647	C	C	T	SNP	OS06G0596300	Exonic	CDS	Silent	LOC_Os06g39520	myristoyl-acyl carrier protein thioesterase, chloroplast precursor, putative, expressed
Chr.6	23468813	23468813	G	G	T	SNP	OS06G0596300	Intronic	NA	NA	LOC_Os06g39520	myristoyl-acyl carrier protein thioesterase, chloroplast precursor, putative, expressed
Chr.6	23857780	23857780	C	C	T	SNP	OS06G0603000	Exonic	5UTR	NA	LOC_Os06g40080	heme oxygenase 1, putative, expressed
Chr.6	24402294	24402294	C	C	T	SNP	OS06G0611900	Intronic	NA	NA	LOC_Os06g40940	glycine dehydrogenase, putative, expressed
Chr.6	28617816	28617816	C	C	T	SNP	OS06G0686400	Exonic	CDS	Missense	LOC_Os06g47200	LTPL85 - Protease inhibitor/seed storage/LTP family protein precursor, expressed
Chr.6	28752630	28752630	C	C	T	SNP	OS06G0689600	Exonic	3UTR	NA	LOC_Os06g47470	TKL_IRAK_DUF26-lh.2 - DUF26 kinases have homology to DUF26 containing loci, expressed
Chr.6	30106360	30106360	C	C	T	SNP	OS06G0711800	Exonic	CDS	Missense	LOC_Os06g49760	invertase/pectin methylesterase inhibitor family protein, putative, expressed
Chr.6	30106689	30106689	G	G	T	SNP	OS06G0711800	Exonic	CDS	Missense	LOC_Os06g49760	invertase/pectin methylesterase inhibitor family protein, putative, expressed
Chr.6	30301912	30301912	C	C	T	SNP	OS06G0714200	Exonic	5UTR	NA	LOC_Os06g50030	CAMK_CAMK_like.30 - CAMK includes calcium/calmodulin depedent protein kinases, expressed
Chr.6	31070068	31070068	A	A	T	SNP	OS06G0729300	Intronic	NA	NA	LOC_Os06g51310	PAZ domain containing protein, putative, expressed
Chr.6	1472152	1472155	TCGA	TCGA	T	INDEL	OS06G0127900	Exonic	3UTR	NA	LOC_Os06g03720	ribonucleoside-diphosphate reductase small chain, putative, expressed
Chr.6	1556237	1556238	TC	TC	T	INDEL	OS06G0129400	Intronic	NA	NA	LOC_Os06g03860	uncharacterized membrane protein, putative, expressed
Chr.6	3198232	3198234	TAC	TAC	T	INDEL	OS06G0163300	Intronic	NA	NA	LOC_Os06g06780	harpin-induced protein, putative, expressed
Chr.6	4869641	4869642	TA	TA	T	INDEL	OS06G0195800	Intronic	NA	NA	LOC_Os06g09560	heat shock protein DnaJ, putative, expressed
Chr.6	9566088	9566094	TATAATA	TATAATA	T	INDEL	OS06G0278000	Intronic	NA	NA	LOC_Os06g16640	carboxyl-terminal peptidase, putative, expressed
Chr.6	10685857	10685866	TGCCGCCGCC	TGCCGCCGCC	T	INDEL	OS06G0291500	Exonic	CDS	InFrame	LOC_Os06g18820	serine threonine kinase, putative, expressed
Chr.6	21600083	21600087	TAAAA	TAAAA	T	INDEL	OS06G0562600	Intronic	NA	NA	LOC_Os06g36700	T-complex protein, putative, expressed
Chr.6	27977228	27977230	TTG	TTG	T	INDEL	OS06G0674100	Intronic	NA	NA	LOC_Os06g46160	expressed protein
Chr.6	30302615	30302620	TTTTTG	TTTTTG	T	INDEL	OS06G0714200	Intronic	NA	NA	LOC_Os06g50030	CAMK_CAMK_like.30 - CAMK includes calcium/calmodulin depedent protein kinases, expressed
Chr.5	26954700	26954700	G	G	T	SNP	OS05G0543100	Exonic	CDS	Silent	LOC_Os05g46550	adaptor complexes medium subunit family domain containing protein, expressed

Chr.5	26952831	26952831	G	G	T	SNP	OS05G0543100	Exonic	3UTR	NA	LOC_Os05g46550	adaptor complexes medium subunit family domain containing protein, expressed
Chr.5	26952621	26952621	G	G	T	SNP	OS05G0543100	Exonic	3UTR	NA	LOC_Os05g46550	adaptor complexes medium subunit family domain containing protein, expressed
Chr.5	26928368	26928368	C	C	T	SNP	OS05G0542500	Exonic	3UTR	NA	LOC_Os05g46480	late embryogenesis abundant protein, group 3, putative, expressed
Chr.5	26922797	26922797	C	C	T	SNP	OS05G0542300	Intronic	NA	NA	LOC_Os05g46470	expressed protein
Chr.5	21189895	21189895	C	C	T	SNP	OS05G0431700	Intronic	NA	NA	LOC_Os05g35650	peptide transporter PTR2, putative, expressed
Chr.5	20631349	20631349	C	C	T	SNP	OS05G0420600	Intronic	NA	NA	LOC_Os05g34770	cytochrome c, putative, expressed
Chr.5	20631264	20631264	C	C	T	SNP	OS05G0420600	Intronic	NA	NA	LOC_Os05g34770	cytochrome c, putative, expressed
Chr.5	20278154	20278154	C	C	T	SNP	OS05G0414501	Intronic	NA	NA	LOC_Os05g34260	transposon protein, putative, Ac/Ds sub-class, expressed
Chr.5	2790030	2790030	C	C	T	SNP	OS05G0148700	Intronic	NA	NA	LOC_Os05g05600	ATA15 protein, putative, expressed
Chr.5	14619747	14619749	TTG	TTG	T	INDEL	OS05G0316200	Exonic	3UTR	NA	LOC_Os05g25210	expressed protein
Chr.5	875349	875356	TCACATCA	TCACATCA	T	INDEL	OS05G0116000	Intronic	NA	NA	LOC_Os05g02520	cupin domain containing protein, expressed
Chr.5	29490231	29490231	G	G	T	SNP	OS05G0591900	Exonic	5UTR	NA	LOC_Os05g51420	hypersensitive-induced response protein, putative, expressed
Chr.5	28857710	28857710	G	G	T	SNP	OS05G0579800	Exonic	3UTR	NA	LOC_Os05g50360	anaphase-promoting complex subunit 10, putative, expressed
Chr.5	28855149	28855149	A	A	T	SNP	OS05G0579800	Intronic	NA	NA	LOC_Os05g50360	anaphase-promoting complex subunit 10, putative, expressed
Chr.5	27959135	27959135	C	C	T	SNP	OS05G0562000	Exonic	3UTR	NA	LOC_Os05g48790	expressed protein
Chr.5	25382910	25382910	C	C	T	SNP	OS05G0512200	Intronic	NA	NA	LOC_Os05g43650	expressed protein
Chr.5	23867516	23867516	G	G	T	SNP	OS05G0485300	Intronic	NA	NA	LOC_Os05g40700	transmembrane protein 56, putative, expressed
Chr.5	23865803	23865803	C	C	T	SNP	OS05G0485300	Exonic	5UTR	NA	LOC_Os05g40700	transmembrane protein 56, putative, expressed
Chr.5	22070477	22070477	C	C	T	SNP	OS05G0449600	Intronic	NA	NA	LOC_Os05g37700	periplasmic beta-glucosidase precursor, putative, expressed
Chr.5	14162887	14162887	C	C	T	SNP	OS05G0310500	Exonic	CDS	NA	LOC_Os05g24550	Papain family cysteine protease domain containing protein, expressed
Chr.5	13839587	13839587	C	C	T	SNP	OS05G0305900	Exonic	CDS	Missense	LOC_Os05g24010	receptor protein kinase-like, putative, expressed
Chr.5	5653653	5653653	A	A	T	SNP	OS05G0192100	Intronic	NA	NA	LOC_Os05g10370	acid phosphatase, putative, expressed
Chr.5	28192978	28192978	C	C	T	SNP	OS05G0566400	Intronic	NA	NA	LOC_Os05g49140	CGMC_MAPKCMGC_2.8 - CGMC includes CDA, MAPK, GSK3, and CLKC kinases, expressed
Chr.5	27959373	27959373	G	G	T	SNP	OS05G0562000	Exonic	CDS	Missense	LOC_Os05g48790	expressed protein
Chr.5	27958502	27958502	C	C	T	SNP	OS05G0562000	Exonic	3UTR	NA	LOC_Os05g48790	expressed protein
Chr.5	27714897	27714897	A	A	T	SNP	OS05G0557200	Intronic	NA	NA	LOC_Os05g48340	proteasome-related, putative, expressed
Chr.5	26171177	26171177	C	C	T	SNP	OS05G0525900	Exonic	3UTR	NA	LOC_Os05g45020	zinc finger/CCCH transcription factor, putative, expressed
Chr.5	25546079	25546079	C	C	T	SNP	OS05G0515200	Exonic	3UTR	NA	LOC_Os05g43910	cytochrome P450, putative, expressed
Chr.5	25546061	25546061	C	C	T	SNP	OS05G0515200	Exonic	3UTR	NA	LOC_Os05g43910	cytochrome P450, putative, expressed
Chr.5	25545774	25545774	C	C	T	SNP	OS05G0515200	Exonic	3UTR	NA	LOC_Os05g43910	cytochrome P450, putative, expressed
Chr.5	25545151	25545151	A	A	T	SNP	OS05G0515200	Intronic	NA	NA	LOC_Os05g43910	cytochrome P450, putative, expressed
Chr.5	25544973	25544973	A	A	T	SNP	OS05G0515200	Intronic	NA	NA	LOC_Os05g43910	cytochrome P450, putative, expressed
Chr.5	24633830	24633830	C	C	T	SNP	OS05G0500700	Exonic	3UTR	NA	LOC_Os05g42140	methyltransferase domain containing protein, expressed
Chr.5	24425753	24425753	C	C	T	SNP	OS05G0497150	Exonic	CDS	NA	LOC_Os05g41750	RecF/RecN/SMC N terminal domain containing protein, expressed
Chr.5	24425483	24425483	C	C	T	SNP	OS05G0497150	Intronic	NA	NA	LOC_Os05g41750	RecF/RecN/SMC N terminal domain containing protein, expressed
Chr.5	24424047	24424047	G	G	T	SNP	OS05G0497150	Intronic	NA	NA	LOC_Os05g41750	RecF/RecN/SMC N terminal domain containing protein, expressed
Chr.5	24329447	24329447	G	G	T	SNP	OS05G0495300	Exonic	5UTR	NA	LOC_Os05g41550	expressed protein
Chr.5	24281170	24281170	C	C	T	SNP	OS05G0494200	Exonic	3UTR	NA	LOC_Os05g41460	cysteine proteinase inhibitor precursor protein, putative, expressed
Chr.5	23422185	23422185	C	C	T	SNP	OS05G0476200	Exonic	CDS	Silent	LOC_Os05g39850	MCM3 - Putative minichromosome maintenance MCM complex subunit 3, expressed
Chr.5	22305864	22305864	C	C	T	SNP	OS05G0454500	Intronic	NA	NA	LOC_Os05g38040	expressed protein
Chr.5	22304671	22304671	A	A	T	SNP	OS05G0454500	Intronic	NA	NA	LOC_Os05g38040	expressed protein
Chr.5	22161470	22161470	C	C	T	SNP	OS05G0451300	Intronic	NA	NA	LOC_Os05g37830	expressed protein
Chr.5	22072644	22072644	C	C	T	SNP	OS05G0449600	Exonic	3UTR	NA	LOC_Os05g37700	periplasmic beta-glucosidase precursor, putative, expressed
Chr.5	22071781	22071781	G	G	T	SNP	OS05G0449600	Exonic	CDS	Missense	LOC_Os05g37700	periplasmic beta-glucosidase precursor, putative, expressed
Chr.5	19960532	19960532	C	C	T	SNP	OS05G0408900	Intronic	NA	NA	LOC_Os05g33840	transketolase, putative, expressed
Chr.5	19769343	19769343	C	C	T	SNP	OS05G0405900	Intronic	NA	NA	LOC_Os05g33610	expressed protein
Chr.5	14307403	14307403	C	C	T	SNP	OS05G0312000	Intronic	NA	NA	LOC_Os05g24684	structural constituent of ribosome, putative, expressed
Chr.5	14295215	14295215	C	C	T	SNP	OS05G0312000	Exonic	3UTR	NA	LOC_Os05g24684	structural constituent of ribosome, putative, expressed
Chr.5	14294948	14294948	A	A	T	SNP	OS05G0312000	Exonic	3UTR	NA	LOC_Os05g24684	structural constituent of ribosome, putative, expressed
Chr.5	14167280	14167280	C	C	T	SNP	OS05G0310500	Exonic	3UTR	NA	LOC_Os05g24550	Papain family cysteine protease domain containing protein, expressed
Chr.5	14164435	14164435	C	C	T	SNP	OS05G0310500	Intronic	NA	NA	LOC_Os05g24550	Papain family cysteine protease domain containing protein, expressed
Chr.5	14164125	14164125	G	G	T	SNP	OS05G0310500	Exonic	3UTR	NA	LOC_Os05g24550	Papain family cysteine protease domain containing protein, expressed
Chr.5	13839090	13839090	C	C	T	SNP	OS05G0305900	Intronic	NA	NA	LOC_Os05g24010	receptor protein kinase-like, putative, expressed
Chr.5	13837530	13837530	C	C	T	SNP	OS05G0305900	Exonic	CDS	Silent	LOC_Os05g24010	receptor protein kinase-like, putative, expressed
Chr.5	13834979	13834979	C	C	T	SNP	OS05G0305900	Intronic	NA	NA	LOC_Os05g24010	receptor protein kinase-like, putative, expressed
Chr.5	13613928	13613928	G	G	T	SNP	OS05G0302700	Intronic	NA	NA	LOC_Os05g23720	mitochondrial carrier protein, putative, expressed
Chr.5	7167061	7167061	C	C	T	SNP	OS05G0215600	Intronic	NA	NA	LOC_Os05g12474	expressed protein
Chr.5	5653941	5653941	C	C	T	SNP	OS05G0192100	Intronic	NA	NA	LOC_Os05g10370	acid phosphatase, putative, expressed
Chr.5	5653910	5653910	C	C	T	SNP	OS05G0192100	Intronic	NA	NA	LOC_Os05g10370	acid phosphatase, putative, expressed
Chr.5	5653898	5653898	G	G	T	SNP	OS05G0192100	Intronic	NA	NA	LOC_Os05g10370	acid phosphatase, putative, expressed
Chr.5	5653465	5653465	C	C	T	SNP	OS05G0192100	Intronic	NA	NA	LOC_Os05g10370	acid phosphatase, putative, expressed
Chr.5	5653396	5653396	A	A	T	SNP	OS05G0192100	Intronic	NA	NA	LOC_Os05g10370	acid phosphatase, putative, expressed
Chr.5	3758292	3758292	C	C	T	SNP	OS05G0163900	Intronic	NA	NA	LOC_Os05g07120	basic helix-loop-helix, putative, expressed
Chr.5	3757463	3757463	C	C	T	SNP	OS05G0163900	Intronic	NA	NA	LOC_Os05g07120	basic helix-loop-helix, putative, expressed
Chr.5	3720619	3720619	C	C	T	SNP	OS05G0163400	Exonic	CDS	Missense	LOC_Os05g07070	zinc finger, C3HC4 type domain containing protein, expressed
Chr.5	3608609	3608609	A	A	T	SNP	OS05G0161200	Intronic	NA	NA	LOC_Os05g06890	relA-SpoT like protein RSH4, putative, expressed
Chr.5	2719152	2719152	A	A	T	SNP	OS05G0147400	Intronic	NA	NA	LOC_Os05g05470	T-complex protein, putative, expressed
Chr.5	2115020	2115020	C	C	T	SNP	OS05G0136200	Exonic	CDS	Missense	LOC_Os05g04550	CAMK_KIN1/SNF1/Nim1_like.3 - CAMK includes calcium/calmodulin depedent protein kinases, expressed
Chr.5	2101865	2101865	A	A	T	SNP	OS05G0135900	Exonic	CDS	Missense	LOC_Os05g04530	IF, putative, expressed
Chr.5	2029991	2029991	C	C	T	SNP	OS05G0134700	Exonic	3UTR	NA	LOC_Os05g04410	peroxidase precursor, putative, expressed
Chr.5	875421	875421	C	C	T	SNP	OS05G0116000	Intronic	NA	NA	LOC_Os05g02520	cupin domain containing protein, expressed
Chr.5	836665	836665	C	C	T	SNP	OS05G0115100	Exonic	CDS	Missense	LOC_Os05g02450	expressed protein
Chr.5	836558	836558	A	A	T	SNP	OS05G0115100	Exonic	CDS	Missense	LOC_Os05g02450	expressed protein
Chr.5	739907	739907	C	C	T	SNP	OS05G0114000	Exonic	3UTR	NA	LOC_Os05g02310	soluble inorganic pyrophosphatase, putative, expressed
Chr.5	28985809	28985809	G	G	T	SNP	OS05G0582300	Intronic	NA	NA	LOC_Os05g50550	polyprenyl synthetase, putative, expressed
Chr.5	25545016	25545016	C	C	T	SNP	OS05G0515200	Intronic	NA	NA	LOC_Os05g43910	cytochrome P450, putative, expressed
Chr.5	5653497	5653497	C	C	T	SNP	OS05G0192100	Intronic	NA	NA	LOC_Os05g10370	acid phosphatase, putative, expressed
Chr.5	5653361	5653361	C	C	T	SNP	OS05G0192100	Intronic	NA	NA	LOC_Os05g10370	acid phosphatase, putative, expressed
Chr.5	4304037	4304037	C	C	T	SNP	OS05G0171900	Intronic	NA	NA	LOC_Os05g07940	glyoxalase family protein, putative, expressed
Chr.5	741909	741909	C	C	T	SNP	OS05G0114000	Intronic	NA	NA	LOC_Os05g02310	soluble inorganic pyrophosphatase, putative, expressed
Chr.5	340482	340482	C	C	T	SNP	OS05G0106200	Exonic	CDS	Silent	LOC_Os05g01570	auxin-induced protein 5NG4, putative, expressed
Chr.5	738563	738563	G	G	T	SNP	OS05G0113900	Intronic	NA	NA	LOC_Os05g02300	Core histone H2A/H2B/H3/H4 domain containing protein, putative, expressed
Chr.5	836153	836153	C	C	T	SNP	OS05G0115100	Exonic	5UTR	NA	LOC_Os05g02450	expressed protein
Chr.5	1001837	1001837	C	C	T	SNP	OS05G0119300	Exonic	CDS	Missense	LOC_Os05g02780	glycine-rich protein A3, putative, expressed
Chr.5	2177208	2177208	C	C	T	SNP	OS05G0137400	Intronic	NA	NA	LOC_Os05g04630	retrotransposon protein, putative, SINE subclass, expressed

Chr.5	2763407	2763407	C	C	T	SNP	OS05G0148300	Exonic	CDS	Silent	LOC_Os05g05560	expressed protein
Chr.5	3734924	3734924	G	G	T	SNP	OS05G0163700	Exonic	CDS	Missense	LOC_Os05g07090	acyl-coenzyme A dehydrogenase, mitochondrial precursor, putative, expressed
Chr.5	3758263	3758263	C	C	T	SNP	OS05G0163900	Intronic	NA	NA	LOC_Os05g07120	basic helix-loop-helix, putative, expressed
Chr.5	3758613	3758613	C	C	T	SNP	OS05G0163900	Intronic	NA	NA	LOC_Os05g07120	basic helix-loop-helix, putative, expressed
Chr.5	3758988	3758988	C	C	T	SNP	OS05G0163900	Intronic	NA	NA	LOC_Os05g07120	basic helix-loop-helix, putative, expressed
Chr.5	4138977	4138977	C	C	T	SNP	OS05G0169000	Exonic	5UTR	NA	LOC_Os05g07690	thioredoxin, putative, expressed
Chr.5	5653152	5653152	G	G	T	SNP	OS05G0192100	Exonic	3UTR	NA	LOC_Os05g10370	acid phosphatase, putative, expressed
Chr.5	5653194	5653194	C	C	T	SNP	OS05G0192100	Exonic	3UTR	NA	LOC_Os05g10370	acid phosphatase, putative, expressed
Chr.5	7162624	7162624	C	C	T	SNP	OS05G0215600	Exonic	3UTR	NA	LOC_Os05g12474	expressed protein
Chr.5	7161684	7161684	C	C	T	SNP	OS05G0215600	Exonic	5UTR	NA	LOC_Os05g12474	expressed protein
Chr.5	7164391	7164391	C	C	T	SNP	OS05G0215600	Intronic	NA	NA	LOC_Os05g12474	expressed protein
Chr.5	8002507	8002507	C	C	T	SNP	OS05G0231700	Exonic	CDS	NA	LOC_Os05g14240	aquaporin protein, putative, expressed
Chr.5	8902524	8902524	A	A	T	SNP	OS05G0247100	Exonic	3UTR	NA	LOC_Os05g15770	glycosyl hydrolase, putative, expressed
Chr.5	8902528	8902528	A	A	T	SNP	OS05G0247100	Exonic	3UTR	NA	LOC_Os05g15770	glycosyl hydrolase, putative, expressed
Chr.5	14246104	14246104	C	C	T	SNP	OS05G0311500	Exonic	3UTR	NA	LOC_Os05g24650	DUF567 domain containing protein, putative, expressed
Chr.5	15078055	15078055	C	C	T	SNP	OS05G0324700	Exonic	CDS	Silent	LOC_Os05g25920	expressed protein
Chr.5	17175860	17175860	G	G	T	SNP	OS05G0360400	Exonic	5UTR	NA	LOC_Os05g29710	RING-H2 finger protein, putative, expressed
Chr.5	18489014	18489014	A	A	T	SNP	OS05G0382500	Exonic	3UTR	NA	LOC_Os05g31740	cytochrome P450 86A2, putative, expressed
Chr.5	19768801	19768801	G	G	T	SNP	OS05G0405900	Exonic	5UTR	NA	LOC_Os05g33610	expressed protein
Chr.5	19959594	19959594	C	C	T	SNP	OS05G0408900	Intronic	NA	NA	LOC_Os05g33840	transketolase, putative, expressed
Chr.5	19959683	19959683	C	C	T	SNP	OS05G0408900	Intronic	NA	NA	LOC_Os05g33840	transketolase, putative, expressed
Chr.5	21141624	21141624	C	C	T	SNP	OS05G0430800	Exonic	CDS	Missense	LOC_Os05g35580	amidophosphoribosyltransferase, chloroplast precursor, putative, expressed
Chr.5	23497394	23497394	G	G	T	SNP	OS05G0477600	Exonic	CDS	Silent	LOC_Os05g39990	expansin precursor, putative, expressed
Chr.5	23866275	23866275	A	A	T	SNP	OS05G0485300	Intronic	NA	NA	LOC_Os05g40700	transmembrane protein 56, putative, expressed
Chr.5	23873837	23873837	C	C	T	SNP	OS05G0485500	Exonic	3UTR	NA	LOC_Os05g40720	glycosyl transferase, family 8, putative, expressed
Chr.5	24281138	24281138	A	A	T	SNP	OS05G0494200	Exonic	3UTR	NA	LOC_Os05g41460	cysteine proteinase inhibitor precursor protein, putative, expressed
Chr.5	24281561	24281561	C	C	T	SNP	OS05G0494200	Exonic	CDS	Silent	LOC_Os05g41460	cysteine proteinase inhibitor precursor protein, putative, expressed
Chr.5	24327690	24327690	A	A	T	SNP	OS05G0495300	Exonic	5UTR	NA	LOC_Os05g41550	expressed protein
Chr.5	24606910	24606910	C	C	T	SNP	OS05G0499800	Exonic	CDS	Silent	LOC_Os05g42040	UDP-glucoronosyl and UDP-glucosyl transferase domain containing protein, expressed
Chr.5	24632061	24632061	C	C	T	SNP	OS05G0500600	Exonic	CDS	Silent	LOC_Os05g42130	MONOCULM 1, putative, expressed
Chr.5	24631276	24631276	G	G	T	SNP	OS05G0500600	Exonic	CDS	Missense	LOC_Os05g42130	MONOCULM 1, putative, expressed
Chr.5	24631994	24631994	C	C	T	SNP	OS05G0500600	Exonic	CDS	Missense	LOC_Os05g42130	MONOCULM 1, putative, expressed
Chr.5	24633158	24633158	C	C	T	SNP	OS05G0500700	Exonic	CDS	NA	LOC_Os05g42140	methyltransferase domain containing protein, expressed
Chr.5	24633500	24633500	C	C	T	SNP	OS05G0500700	Exonic	CDS	NA	LOC_Os05g42140	methyltransferase domain containing protein, expressed
Chr.5	25385340	25385340	C	C	T	SNP	OS05G0512200	Exonic	3UTR	NA	LOC_Os05g43650	expressed protein
Chr.5	25384992	25384992	C	C	T	SNP	OS05G0512200	Exonic	CDS	Silent	LOC_Os05g43650	expressed protein
Chr.5	25546053	25546053	C	C	T	SNP	OS05G0515200	Exonic	3UTR	NA	LOC_Os05g43910	cytochrome P450, putative, expressed
Chr.5	25543299	25543299	C	C	T	SNP	OS05G0515200	Exonic	CDS	Missense	LOC_Os05g43910	cytochrome P450, putative, expressed
Chr.5	26368374	26368374	C	C	T	SNP	OS05G0531100	Exonic	CDS	Silent	LOC_Os05g45450	DUF584 domain containing protein, putative, expressed
Chr.5	27122074	27122074	C	C	T	SNP	OS05G0546400	Exonic	CDS	Silent	LOC_Os05g46840	proline-rich protein, putative, expressed
Chr.5	27122547	27122547	C	C	T	SNP	OS05G0546400	Exonic	CDS	Silent	LOC_Os05g46840	proline-rich protein, putative, expressed
Chr.5	27122215	27122215	A	A	T	SNP	OS05G0546400	Exonic	CDS	Missense	LOC_Os05g46840	proline-rich protein, putative, expressed
Chr.5	27122443	27122443	C	C	T	SNP	OS05G0546400	Exonic	CDS	Missense	LOC_Os05g46840	proline-rich protein, putative, expressed
Chr.5	27122470	27122470	C	C	T	SNP	OS05G0546400	Exonic	CDS	Missense	LOC_Os05g46840	proline-rich protein, putative, expressed
Chr.5	27535936	27535936	C	C	T	SNP	OS05G0553800	Intronic	NA	NA	LOC_Os05g48030	anti-silencing protein, ASF1-like domain containing protein, expressed
Chr.5	27959570	27959570	C	C	T	SNP	OS05G0562000	Intronic	NA	NA	LOC_Os05g48790	expressed protein
Chr.5	27959610	27959610	C	C	T	SNP	OS05G0562000	Intronic	NA	NA	LOC_Os05g48790	expressed protein
Chr.5	28311330	28311330	G	G	T	SNP	OS05G0568800	Exonic	CDS	Silent	LOC_Os05g49370	DUF1645 domain containing protein, putative, expressed
Chr.5	998385	998386	TA	TA	T	INDEL	OS05G0119200	Intronic	NA	NA	LOC_Os05g02770	glycine-rich cell wall structural protein precursor, putative, expressed
Chr.5	4137897	4137898	TC	TC	T	INDEL	OS05G0169000	Exonic	CDS	NA	LOC_Os05g07690	thioredoxin, putative, expressed
Chr.5	8902500	8902507	TGGCGATG	TGGCGATG	T	INDEL	OS05G0247100	Exonic	3UTR	NA	LOC_Os05g15770	glycosyl hydrolase, putative, expressed
Chr.5	14306865	14306870	TGAGGG	TGAGGG	T	INDEL	OS05G0312000	Intronic	NA	NA	LOC_Os05g24684	structural constituent of ribosome, putative, expressed
Chr.5	15482560	15482567	TCTAATAG	TCTAATAG	T	INDEL	OS05G0331200	Exonic	3UTR	NA	LOC_Os05g26660	NADH-ubiquinone oxidoreductase, mitochondrial precursor, putative, expressed
Chr.5	15486528	15486530	TGA	TGA	T	INDEL	OS05G0331200	Intronic	NA	NA	LOC_Os05g26660	NADH-ubiquinone oxidoreductase, mitochondrial precursor, putative, expressed
Chr.5	23495731	23495734	TACA	TACA	T	INDEL	OS05G0477600	Exonic	3UTR	NA	LOC_Os05g39990	expansin precursor, putative, expressed
Chr.5	23873723	23873736	TATGAGTACTATCA	TATGAGTACTATCA	T	INDEL	OS05G0485500	Exonic	3UTR	NA	LOC_Os05g40720	glycosyl transferase, family 8, putative, expressed
Chr.5	24328401	24328403	TGA	TGA	T	INDEL	OS05G0495300	Exonic	5UTR	NA	LOC_Os05g41550	expressed protein
Chr.5	25384806	25384807	TA	TA	T	INDEL	OS05G0512200	Intronic	NA	NA	LOC_Os05g43650	expressed protein
Chr.5	26903528	26903542	TCTTATGTGTTACGG	TCTTATGTGTTACGG	T	INDEL	OS05G0541900	Exonic	3UTR	NA	LOC_Os05g46430	60S ribosomal protein L28-1, putative, expressed
Chr.5	27701087	27701092	TGAATA	TGAATA	T	INDEL	OS05G0557000	Intronic	NA	NA	LOC_Os05g48320	60S ribosomal protein L37a, putative, expressed
Chr.4	33168082	33168082	C	C	T	SNP	OS04G0650800	Intronic	NA	NA	LOC_Os04g55720	D-3-phosphoglycerate dehydrogenase, chloroplast precursor, putative, expressed
Chr.4	33126225	33126225	C	C	T	SNP	OS04G0650000	Intronic	NA	NA	LOC_Os04g55650	oryzain alpha chain precursor, putative, expressed
Chr.4	33101937	33101937	C	C	T	SNP	OS04G0649500	Exonic	CDS	Silent	LOC_Os04g55600	expressed protein
Chr.4	31180196	31180196	C	C	T	SNP	OS04G0614600	Intronic	NA	NA	LOC_Os04g52450	aminotransferase, putative, expressed
Chr.4	31113356	31113356	G	G	T	SNP	OS04G0613700	Exonic	5UTR	NA	LOC_Os04g52370	UTP--glucose-1-phosphate uridylyltransferase, putative, expressed
Chr.4	28305239	28305239	A	A	T	SNP	OS04G0564700	Exonic	5UTR	NA	LOC_Os04g47700	expressed protein
Chr.4	28296571	28296571	C	C	T	SNP	OS04G0564700	Exonic	3UTR	NA	LOC_Os04g47700	expressed protein
Chr.4	28085137	28085137	G	G	T	SNP	OS04G0560700	Intronic	NA	NA	LOC_Os04g47310	expressed protein
Chr.4	28084266	28084266	G	G	T	SNP	OS04G0560700	Exonic	5UTR	NA	LOC_Os04g47310	expressed protein
Chr.4	27848783	27848783	C	C	T	SNP	OS04G0556500	Exonic	3UTR	NA	LOC_Os04g46980	cis-zeatin O-glucosyltransferase, putative, expressed
Chr.4	27848709	27848709	C	C	T	SNP	OS04G0556500	Exonic	3UTR	NA	LOC_Os04g46980	cis-zeatin O-glucosyltransferase, putative, expressed
Chr.4	27786984	27786984	G	G	T	SNP	OS04G0555300	Intronic	NA	NA	LOC_Os04g46880	transporter, major facilitator family, putative, expressed
Chr.4	27642300	27642300	G	G	T	SNP	OS04G0551800	Intronic	NA	NA	LOC_Os04g46620	T-complex protein, putative, expressed
Chr.4	27641342	27641342	A	A	T	SNP	OS04G0551800	Exonic	CDS	Silent	LOC_Os04g46620	T-complex protein, putative, expressed
Chr.4	27640116	27640116	C	C	T	SNP	OS04G0551800	Intronic	NA	NA	LOC_Os04g46620	T-complex protein, putative, expressed
Chr.4	27639299	27639299	C	C	T	SNP	OS04G0551800	Intronic	NA	NA	LOC_Os04g46620	T-complex protein, putative, expressed
Chr.4	27507605	27507605	C	C	T	SNP	OS04G0549600	Intronic	NA	NA	LOC_Os04g46390	chaperone protein dnaJ, putative, expressed
Chr.4	27506988	27506988	C	C	T	SNP	OS04G0549600	Exonic	CDS	Missense	LOC_Os04g46390	chaperone protein dnaJ, putative, expressed
Chr.4	27194589	27194589	C	C	T	SNP	OS04G0542900	Exonic	3UTR	NA	LOC_Os04g45910	placental protein 11 precursor, putative, expressed
Chr.4	27193217	27193217	C	C	T	SNP	OS04G0542900	Intronic	NA	NA	LOC_Os04g45910	placental protein 11 precursor, putative, expressed
Chr.4	27192336	27192336	C	C	T	SNP	OS04G0542900	Intronic	NA	NA	LOC_Os04g45910	placental protein 11 precursor, putative, expressed
Chr.4	25195891	25195891	C	C	T	SNP	OS04G0504800	Exonic	CDS	Silent	LOC_Os04g42600	polyadenylate-binding protein, putative, expressed
Chr.4	25194368	25194368	C	C	T	SNP	OS04G0504800	Exonic	3UTR	NA	LOC_Os04g42600	polyadenylate-binding protein, putative, expressed
Chr.4	25194310	25194310	C	C	T	SNP	OS04G0504800	Exonic	3UTR	NA	LOC_Os04g42600	polyadenylate-binding protein, putative, expressed
Chr.4	25134123	25134123	C	C	T	SNP	OS04G0503500	Intronic	NA	NA	LOC_Os04g42470	regulatory subunit, putative, expressed

Chr.4	25133597	25133597	C	C	T	SNP	OS04G0503500	Exonic	5UTR	NA	LOC_Os04g42470	regulatory subunit, putative, expressed
Chr.4	25126765	25126765	A	A	T	SNP	OS04G0503500	Exonic	3UTR	NA	LOC_Os04g42470	regulatory subunit, putative, expressed
Chr.4	24886706	24886706	A	A	T	SNP	OS04G0497600	Exonic	5UTR	NA	LOC_Os04g42010	RNA binding protein, putative, expressed
Chr.4	24850797	24850797	C	C	T	SNP	OS04G0496800	Intronic	NA	NA	LOC_Os04g41950	calcium-binding mitochondrial protein anon-60Da, putative, expressed
Chr.4	24529424	24529424	A	A	T	SNP	OS04G0490800	Intronic	NA	NA	LOC_Os04g41340	4-nitrophenylphosphatase, putative, expressed
Chr.4	24528532	24528532	C	C	T	SNP	OS04G0490800	Intronic	NA	NA	LOC_Os04g41340	4-nitrophenylphosphatase, putative, expressed
Chr.4	24354158	24354158	C	C	T	SNP	OS04G0487300	Exonic	3UTR	NA	LOC_Os04g41040	DNA-directed RNA polymerases I, II, and III subunit RPABC1, putative, expressed
Chr.4	24353651	24353651	C	C	T	SNP	OS04G0487300	Exonic	3UTR	NA	LOC_Os04g41040	DNA-directed RNA polymerases I, II, and III subunit RPABC1, putative, expressed
Chr.4	24322666	24322666	A	A	T	SNP	OS04G0486900	Intronic	NA	NA	LOC_Os04g40980	Divergent PAP2 family domain containing protein, expressed
Chr.4	24057437	24057437	C	C	T	SNP	OS04G0480900	Exonic	CDS	Silent	LOC_Os04g40490	glycosyl hydrolase family 5 protein, putative, expressed
Chr.4	24057063	24057063	C	C	T	SNP	OS04G0480900	Intronic	NA	NA	LOC_Os04g40490	glycosyl hydrolase family 5 protein, putative, expressed
Chr.4	24055786	24055786	C	C	T	SNP	OS04G0480900	Intronic	NA	NA	LOC_Os04g40490	glycosyl hydrolase family 5 protein, putative, expressed
Chr.4	24055624	24055624	G	G	T	SNP	OS04G0480900	Intronic	NA	NA	LOC_Os04g40490	glycosyl hydrolase family 5 protein, putative, expressed
Chr.4	24055595	24055595	C	C	T	SNP	OS04G0480900	Intronic	NA	NA	LOC_Os04g40490	glycosyl hydrolase family 5 protein, putative, expressed
Chr.4	23847765	23847765	G	G	T	SNP	OS04G0476400	Exonic	3UTR	NA	LOC_Os04g40050	ribonuclease H2 subunit B, putative, expressed
Chr.4	23847248	23847248	C	C	T	SNP	OS04G0476400	Intronic	NA	NA	LOC_Os04g40050	ribonuclease H2 subunit B, putative, expressed
Chr.4	23846682	23846682	C	C	T	SNP	OS04G0476400	Intronic	NA	NA	LOC_Os04g40050	ribonuclease H2 subunit B, putative, expressed
Chr.4	23846011	23846011	G	G	T	SNP	OS04G0476400	Exonic	3UTR	NA	LOC_Os04g40050	ribonuclease H2 subunit B, putative, expressed
Chr.4	23844360	23844360	C	C	T	SNP	OS04G0476400	Intronic	NA	NA	LOC_Os04g40050	ribonuclease H2 subunit B, putative, expressed
Chr.4	23409663	23409663	A	A	T	SNP	OS04G0468600	Exonic	CDS	Missense	LOC_Os04g39320	expressed protein
Chr.4	23318648	23318648	A	A	T	SNP	OS04G0466600	Exonic	3UTR	NA	LOC_Os04g39210	agmatine deiminase, putative, expressed
Chr.4	23141592	23141592	C	C	T	SNP	OS04G0463500	Intronic	NA	NA	LOC_Os04g38950	class I glutamine amidotransferase, putative, expressed
Chr.4	23140894	23140894	C	C	T	SNP	OS04G0463500	Intronic	NA	NA	LOC_Os04g38950	class I glutamine amidotransferase, putative, expressed
Chr.4	22625054	22625054	C	C	T	SNP	OS04G0453233	Intronic	NA	NA	LOC_Os04g38026	transporter family protein, putative, expressed
Chr.4	22260801	22260801	G	G	T	SNP	OS04G0446500	Intronic	NA	NA	LOC_Os04g36890	peptidyl-prolyl cis-trans isomerase, FKBP-type, putative, expressed
Chr.4	22259926	22259926	C	C	T	SNP	OS04G0446500	Intronic	NA	NA	LOC_Os04g36890	peptidyl-prolyl cis-trans isomerase, FKBP-type, putative, expressed
Chr.4	22258313	22258313	C	C	T	SNP	OS04G0446500	Exonic	5UTR	NA	LOC_Os04g36890	peptidyl-prolyl cis-trans isomerase, FKBP-type, putative, expressed
Chr.4	22185391	22185391	C	C	T	SNP	OS04G0444800	Exonic	CDS	Silent	LOC_Os04g36720	ferric-chelate reductase, putative, expressed
Chr.4	20321306	20321306	A	A	T	SNP	OS04G0411400	Exonic	5UTR	NA	LOC_Os04g33570	RCN4 Centroradialis-like1 homologous to TFL1 gene; contains Pfam profile PF01161: Phosphatidylethanolamine-binding protein,
Chr.4	5325501	5325501	C	C	T	SNP	OS04G0178300	Intronic	NA	NA	LOC_Os04g09900	ent-kaurene synthase, chloroplast precursor, putative, expressed
Chr.4	5325447	5325447	G	G	T	SNP	OS04G0178300	Intronic	NA	NA	LOC_Os04g09900	ent-kaurene synthase, chloroplast precursor, putative, expressed
Chr.4	5325342	5325342	C	C	T	SNP	OS04G0178300	Exonic	CDS	Silent	LOC_Os04g09900	ent-kaurene synthase, chloroplast precursor, putative, expressed
Chr.4	5324742	5324742	C	C	T	SNP	OS04G0178300	Exonic	CDS	Silent	LOC_Os04g09900	ent-kaurene synthase, chloroplast precursor, putative, expressed
Chr.4	5324648	5324648	A	A	T	SNP	OS04G0178300	Intronic	NA	NA	LOC_Os04g09900	ent-kaurene synthase, chloroplast precursor, putative, expressed
Chr.4	5324275	5324275	A	A	T	SNP	OS04G0178300	Intronic	NA	NA	LOC_Os04g09900	ent-kaurene synthase, chloroplast precursor, putative, expressed
Chr.4	5324100	5324100	C	C	T	SNP	OS04G0178300	Intronic	NA	NA	LOC_Os04g09900	ent-kaurene synthase, chloroplast precursor, putative, expressed
Chr.4	5324089	5324089	C	C	T	SNP	OS04G0178300	Intronic	NA	NA	LOC_Os04g09900	ent-kaurene synthase, chloroplast precursor, putative, expressed
Chr.4	5323912	5323912	C	C	T	SNP	OS04G0178300	Intronic	NA	NA	LOC_Os04g09900	ent-kaurene synthase, chloroplast precursor, putative, expressed
Chr.4	5321739	5321739	C	C	T	SNP	OS04G0178300	Intronic	NA	NA	LOC_Os04g09900	ent-kaurene synthase, chloroplast precursor, putative, expressed
Chr.4	5321696	5321696	G	G	T	SNP	OS04G0178300	Intronic	NA	NA	LOC_Os04g09900	ent-kaurene synthase, chloroplast precursor, putative, expressed
Chr.4	5320780	5320780	C	C	T	SNP	OS04G0178300	Intronic	NA	NA	LOC_Os04g09900	ent-kaurene synthase, chloroplast precursor, putative, expressed
Chr.4	318574	318574	C	C	T	SNP	OS04G0104900	Intronic	NA	NA	LOC_Os04g01470	O-methyltransferase, putative, expressed
Chr.4	317427	317427	G	G	T	SNP	OS04G0104900	Intronic	NA	NA	LOC_Os04g01470	O-methyltransferase, putative, expressed
Chr.4	24057048	24057050	TGA	TGA	T	INDEL	OS04G0480900	Intronic	NA	NA	LOC_Os04g40490	glycosyl hydrolase family 5 protein, putative, expressed
Chr.4	24056558	24056560	TAA	TAA	T	INDEL	OS04G0480900	Intronic	NA	NA	LOC_Os04g40490	glycosyl hydrolase family 5 protein, putative, expressed
Chr.4	23409993	23409994	TA	TA	T	INDEL	OS04G0468600	Exonic	5UTR	NA	LOC_Os04g39320	expressed protein
Chr.4	24884495	24884496	TA	TA	T	INDEL	OS04G0497600	Intronic	NA	NA	LOC_Os04g42010	RNA binding protein, putative, expressed
Chr.4	33254624	33254624	G	G	T	SNP	OS04G0652700	Intronic	NA	NA	LOC_Os04g55850	nuclease PA3, putative, expressed
Chr.4	31441432	31441432	G	G	T	SNP	OS04G0619000	Intronic	NA	NA	LOC_Os04g52810	no apical meristem protein, putative, expressed
Chr.4	31441365	31441365	G	G	T	SNP	OS04G0619000	Intronic	NA	NA	LOC_Os04g52810	no apical meristem protein, putative, expressed
Chr.4	30911398	30911398	G	G	T	SNP	OS04G0609900	Exonic	CDS	Missense	LOC_Os04g52040	TNP1, putative, expressed
Chr.4	29976513	29976513	G	G	T	SNP	OS04G0592600	Intronic	NA	NA	LOC_Os04g50216	SNARE associated Golgi protein, putative, expressed
Chr.4	29560546	29560546	G	G	T	SNP	OS04G0585100	Intronic	NA	NA	LOC_Os04g49560	zinc finger family protein, putative, expressed
Chr.4	28304196	28304196	G	G	T	SNP	OS04G0564700	Intronic	NA	NA	LOC_Os04g47700	expressed protein
Chr.4	28303969	28303969	G	G	T	SNP	OS04G0564700	Intronic	NA	NA	LOC_Os04g47700	expressed protein
Chr.4	26237930	26237930	G	G	T	SNP	OS04G0524500	Exonic	CDS	NA	LOC_Os04g44300	transposon protein, putative, unclassified, expressed
Chr.4	22260247	22260247	G	G	T	SNP	OS04G0446500	Exonic	CDS	Missense	LOC_Os04g36890	peptidyl-prolyl cis-trans isomerase, FKBP-type, putative, expressed
Chr.4	20425565	20425565	G	G	T	SNP	OS04G0413500	Intronic	NA	NA	LOC_Os04g33740	glycosyl hydrolases, putative, expressed
Chr.4	20389371	20389371	G	G	T	SNP	OS04G0412500	Exonic	CDS	Silent	LOC_Os04g33660	bifunctional monodehydroascorbate reductase and carbonic anhydrasenectarin-3 precursor, putative, expressed
Chr.4	13650412	13650412	G	G	T	SNP	OS04G0304200	Intronic	NA	NA	LOC_Os04g23890	AGC_PVPK_like_kin82y.10 - ACG kinases include homologs to PKA, PKG and PKC, expressed
Chr.4	11238352	11238352	G	G	T	SNP	OS04G0269600	Exonic	5UTR	NA	LOC_Os04g20164	amine oxidase precursor, putative, expressed
Chr.4	8948429	8948429	G	G	T	SNP	OS04G0233400	Exonic	3UTR	NA	LOC_Os04g16450	aquaporin protein, putative, expressed
Chr.4	7307866	7307866	G	G	T	SNP	OS04G0209200	Intronic	NA	NA	LOC_Os04g13210	multidrug resistance-associated protein, putative, expressed
Chr.4	6480072	6480072	G	G	T	SNP	OS04G0194500	Intronic	NA	NA	LOC_Os04g11820	white-brown complex homolog protein, putative, expressed
Chr.4	25662561	25662561	C	C	T	SNP	OS04G0513400	Exonic	3UTR	NA	LOC_Os04g43390	Os4bglu16 - monolignol beta-glucoside homologue, expressed
Chr.4	34459242	34459242	C	C	T	SNP	OS04G0675000	Intronic	NA	NA	LOC_Os04g57870	expressed protein
Chr.4	33532244	33532244	C	C	T	SNP	OS04G0657500	Intronic	NA	NA	LOC_Os04g56240	lipase, putative, expressed
Chr.4	33532243	33532243	C	C	T	SNP	OS04G0657500	Intronic	NA	NA	LOC_Os04g56240	lipase, putative, expressed
Chr.4	33532193	33532193	C	C	T	SNP	OS04G0657500	Intronic	NA	NA	LOC_Os04g56240	lipase, putative, expressed
Chr.4	33418674	33418674	C	C	T	SNP	OS04G0655300	Exonic	5UTR	NA	LOC_Os04g56110	protein kinase, putative, expressed
Chr.4	33255540	33255540	C	C	T	SNP	OS04G0652700	Exonic	CDS	Silent	LOC_Os04g55850	nuclease PA3, putative, expressed
Chr.4	33255353	33255353	C	C	T	SNP	OS04G0652700	Intronic	NA	NA	LOC_Os04g55850	nuclease PA3, putative, expressed
Chr.4	31691626	31691626	C	C	T	SNP	OS04G0623500	Exonic	CDS	Missense	LOC_Os04g53210	hydroxyacid oxidase 1, putative, expressed
Chr.4	31674441	31674441	C	C	T	SNP	OS04G0623300	Exonic	5UTR	NA	LOC_Os04g53190	CPuORF12 - conserved peptide uORF-containing transcript, expressed
Chr.4	31465488	31465488	C	C	T	SNP	OS04G0619400	Intronic	NA	NA	LOC_Os04g52840	tyrosine protein kinase domain containing protein, putative, expressed
Chr.4	31443109	31443109	C	C	T	SNP	OS04G0619000	Exonic	3UTR	NA	LOC_Os04g52810	no apical meristem protein, putative, expressed
Chr.4	31443072	31443072	C	C	T	SNP	OS04G0619000	Exonic	3UTR	NA	LOC_Os04g52810	no apical meristem protein, putative, expressed
Chr.4	31441464	31441464	C	C	T	SNP	OS04G0619000	Intronic	NA	NA	LOC_Os04g52810	no apical meristem protein, putative, expressed
Chr.4	31441411	31441411	C	C	T	SNP	OS04G0619000	Intronic	NA	NA	LOC_Os04g52810	no apical meristem protein, putative, expressed
Chr.4	31280273	31280273	C	C	T	SNP	OS04G0616300	Intronic	NA	NA	LOC_Os04g52600	SHR5-receptor-like kinase, putative, expressed
Chr.4	31181804	31181804	C	C	T	SNP	OS04G0614600	Intronic	NA	NA	LOC_Os04g52450	aminotransferase, putative, expressed
Chr.4	31181151	31181151	C	C	T	SNP	OS04G0614600	Intronic	NA	NA	LOC_Os04g52450	aminotransferase, putative, expressed
Chr.4	31180189	31180189	C	C	T	SNP	OS04G0614600	Intronic	NA	NA	LOC_Os04g52450	aminotransferase, putative, expressed
Chr.4	30079242	30079242	C	C	T	SNP	OS04G0596300	Exonic	5UTR	NA	LOC_Os04g50860	expressed protein
Chr.4	29523760	29523760	C	C	T	SNP	OS04G0584500	Exonic	CDS	Silent	LOC_Os04g49500	U-box domain-containing protein, putative, expressed

Chr.4	29317587	29317587	C	C	T	SNP	OS04G0580800	Exonic	3UTR	NA	LOC_Os04g49160	zinc finger, C3HC4 type domain containing protein, expressed
Chr.4	29317557	29317557	C	C	T	SNP	OS04G0580800	Exonic	3UTR	NA	LOC_Os04g49160	zinc finger, C3HC4 type domain containing protein, expressed
Chr.4	28302393	28302393	C	C	T	SNP	OS04G0564700	Intronic	NA	NA	LOC_Os04g47700	expressed protein
Chr.4	27848792	27848792	C	C	T	SNP	OS04G0556500	Exonic	3UTR	NA	LOC_Os04g46980	cis-zeatin O-glucosyltransferase, putative, expressed
Chr.4	27848727	27848727	C	C	T	SNP	OS04G0556500	Exonic	3UTR	NA	LOC_Os04g46980	cis-zeatin O-glucosyltransferase, putative, expressed
Chr.4	24848356	24848356	C	C	T	SNP	OS04G0496800	Intronic	NA	NA	LOC_Os04g41950	calcium-binding mitochondrial protein anon-60Da, putative, expressed
Chr.4	24323203	24323203	C	C	T	SNP	OS04G0486900	Intronic	NA	NA	LOC_Os04g40980	Divergent PAP2 family domain containing protein, expressed
Chr.4	22623392	22623392	C	C	T	SNP	OS04G0453233	Intronic	NA	NA	LOC_Os04g38026	transporter family protein, putative, expressed
Chr.4	22185220	22185220	C	C	T	SNP	OS04G0444800	Exonic	CDS	Silent	LOC_Os04g36720	ferric-chelate reductase, putative, expressed
Chr.4	22019408	22019408	C	C	T	SNP	OS04G0442200	Intronic	NA	NA	LOC_Os04g36062	expressed protein
Chr.4	20426432	20426432	C	C	T	SNP	OS04G0413500	Intronic	NA	NA	LOC_Os04g33740	glycosyl hydrolases, putative, expressed
Chr.4	20388338	20388338	C	C	T	SNP	OS04G0412500	Intronic	NA	NA	LOC_Os04g33660	bifunctional monodehydroascorbate reductase and carbonic anhydrasenectarin-3 precursor, putative, expressed
Chr.4	18602943	18602943	C	C	T	SNP	OS04G0380300	Exonic	3UTR	NA	LOC_Os04g31120	OsFBK14 - F-box domain and kelch repeat containing protein, expressed
Chr.4	16004550	16004550	C	C	T	SNP	OS04G0339400	Intronic	NA	NA	LOC_Os04g27060	oxidoreductase, aldo/keto reductase family protein, putative, expressed
Chr.4	16004535	16004535	C	C	T	SNP	OS04G0339400	Intronic	NA	NA	LOC_Os04g27060	oxidoreductase, aldo/keto reductase family protein, putative, expressed
Chr.4	16004283	16004283	C	C	T	SNP	OS04G0339400	Intronic	NA	NA	LOC_Os04g27060	oxidoreductase, aldo/keto reductase family protein, putative, expressed
Chr.4	16002159	16002159	C	C	T	SNP	OS04G0339400	Intronic	NA	NA	LOC_Os04g27060	oxidoreductase, aldo/keto reductase family protein, putative, expressed
Chr.4	13642006	13642006	C	C	T	SNP	OS04G0304200	Intronic	NA	NA	LOC_Os04g23890	AGC_PVPK_like_kin82y.10 - ACG kinases include homologs to PKA, PKG and PKC, expressed
Chr.4	11282400	11282400	C	C	T	SNP	OS04G0270100	Intronic	NA	NA	LOC_Os04g20220	elongation factor Tu, putative, expressed
Chr.4	8948614	8948614	C	C	T	SNP	OS04G0233400	Exonic	3UTR	NA	LOC_Os04g16450	aquaporin protein, putative, expressed
Chr.4	7308206	7308206	C	C	T	SNP	OS04G0209200	Intronic	NA	NA	LOC_Os04g13210	multidrug resistance-associated protein, putative, expressed
Chr.4	7308072	7308072	C	C	T	SNP	OS04G0209200	Intronic	NA	NA	LOC_Os04g13210	multidrug resistance-associated protein, putative, expressed
Chr.4	7308029	7308029	C	C	T	SNP	OS04G0209200	Intronic	NA	NA	LOC_Os04g13210	multidrug resistance-associated protein, putative, expressed
Chr.4	6481340	6481340	C	C	T	SNP	OS04G0194500	Intronic	NA	NA	LOC_Os04g11820	white-brown complex homolog protein, putative, expressed
Chr.4	6481132	6481132	C	C	T	SNP	OS04G0194500	Intronic	NA	NA	LOC_Os04g11820	white-brown complex homolog protein, putative, expressed
Chr.4	6480844	6480844	C	C	T	SNP	OS04G0194500	Intronic	NA	NA	LOC_Os04g11820	white-brown complex homolog protein, putative, expressed
Chr.4	6480835	6480835	C	C	T	SNP	OS04G0194500	Intronic	NA	NA	LOC_Os04g11820	white-brown complex homolog protein, putative, expressed
Chr.4	6479577	6479577	C	C	T	SNP	OS04G0194500	Exonic	CDS	Missense	LOC_Os04g11820	white-brown complex homolog protein, putative, expressed
Chr.4	6479068	6479068	C	C	T	SNP	OS04G0194500	Intronic	NA	NA	LOC_Os04g11820	white-brown complex homolog protein, putative, expressed
Chr.4	6478484	6478484	C	C	T	SNP	OS04G0194500	Exonic	CDS	Silent	LOC_Os04g11820	white-brown complex homolog protein, putative, expressed
Chr.4	6475626	6475626	C	C	T	SNP	OS04G0194500	Intronic	NA	NA	LOC_Os04g11820	white-brown complex homolog protein, putative, expressed
Chr.4	621134	621134	C	C	T	SNP	OS04G0110600	Intronic	NA	NA	LOC_Os04g02000	zinc finger family protein, putative, expressed
Chr.4	617873	617873	C	C	T	SNP	OS04G0110600	Exonic	3UTR	NA	LOC_Os04g02000	zinc finger family protein, putative, expressed
Chr.4	25201130	25201130	A	A	T	SNP	OS04G0504800	Exonic	5UTR	NA	LOC_Os04g42600	polyadenylate-binding protein, putative, expressed
Chr.4	33533706	33533706	A	A	T	SNP	OS04G0657500	Exonic	3UTR	NA	LOC_Os04g56240	lipase, putative, expressed
Chr.4	33418730	33418730	A	A	T	SNP	OS04G0655300	Exonic	5UTR	NA	LOC_Os04g56110	protein kinase, putative, expressed
Chr.4	33254394	33254394	A	A	T	SNP	OS04G0652700	Intronic	NA	NA	LOC_Os04g55850	nuclease PA3, putative, expressed
Chr.4	31442880	31442880	A	A	T	SNP	OS04G0619000	Exonic	3UTR	NA	LOC_Os04g52810	no apical meristem protein, putative, expressed
Chr.4	31442639	31442639	A	A	T	SNP	OS04G0619000	Exonic	3UTR	NA	LOC_Os04g52810	no apical meristem protein, putative, expressed
Chr.4	31441378	31441378	A	A	T	SNP	OS04G0619000	Intronic	NA	NA	LOC_Os04g52810	no apical meristem protein, putative, expressed
Chr.4	31439833	31439833	A	A	T	SNP	OS04G0619000	Exonic	3UTR	NA	LOC_Os04g52810	no apical meristem protein, putative, expressed
Chr.4	31439803	31439803	A	A	T	SNP	OS04G0619000	Exonic	3UTR	NA	LOC_Os04g52810	no apical meristem protein, putative, expressed
Chr.4	31439615	31439615	A	A	T	SNP	OS04G0619000	Exonic	3UTR	NA	LOC_Os04g52810	no apical meristem protein, putative, expressed
Chr.4	31220633	31220633	A	A	T	SNP	OS04G0615200	Intronic	NA	NA	LOC_Os04g52504	adhesive/proline-rich protein, putative, expressed
Chr.4	31182425	31182425	A	A	T	SNP	OS04G0614600	Exonic	3UTR	NA	LOC_Os04g52450	aminotransferase, putative, expressed
Chr.4	29559533	29559533	A	A	T	SNP	OS04G0585100	Intronic	NA	NA	LOC_Os04g49560	zinc finger family protein, putative, expressed
Chr.4	29317482	29317482	C	C	T	SNP	OS04G0580800	Exonic	3UTR	NA	LOC_Os04g49160	zinc finger, C3HC4 type domain containing protein, expressed
Chr.4	28299360	28299360	A	A	T	SNP	OS04G0564700	Intronic	NA	NA	LOC_Os04g47700	expressed protein
Chr.4	27639045	27639045	A	A	T	SNP	OS04G0551800	Exonic	CDS	Silent	LOC_Os04g46620	T-complex protein, putative, expressed
Chr.4	27237244	27237244	A	A	T	SNP	OS04G0543900	Intronic	NA	NA	LOC_Os04g45970	glutamate dehydrogenase protein, putative, expressed
Chr.4	27234771	27234771	A	A	T	SNP	OS04G0543900	Exonic	3UTR	NA	LOC_Os04g45970	glutamate dehydrogenase protein, putative, expressed
Chr.4	24325861	24325861	A	A	T	SNP	OS04G0486950	Exonic	3UTR	NA	LOC_Os04g40990	malate synthase, glyoxysomal, putative, expressed
Chr.4	22624620	22624620	A	A	T	SNP	OS04G0453233	Intronic	NA	NA	LOC_Os04g38026	transporter family protein, putative, expressed
Chr.4	22208973	22208973	A	A	T	SNP	OS04G0445400	Intronic	NA	NA	LOC_Os04g36780	expressed protein
Chr.4	16004591	16004591	A	A	T	SNP	OS04G0339400	Intronic	NA	NA	LOC_Os04g27060	oxidoreductase, aldo/keto reductase family protein, putative, expressed
Chr.4	7307927	7307927	A	A	T	SNP	OS04G0209200	Intronic	NA	NA	LOC_Os04g13210	multidrug resistance-associated protein, putative, expressed
Chr.4	6479041	6479041	A	A	T	SNP	OS04G0194500	Intronic	NA	NA	LOC_Os04g11820	white-brown complex homolog protein, putative, expressed
Chr.4	619900	619900	A	A	T	SNP	OS04G0110600	Exonic	3UTR	NA	LOC_Os04g02000	zinc finger family protein, putative, expressed
Chr.4	317564	317564	C	C	T	SNP	OS04G0104900	Intronic	NA	NA	LOC_Os04g01470	O-methyltransferase, putative, expressed
Chr.4	483900	483900	A	A	T	SNP	OS04G0107900	Intronic	NA	NA	LOC_Os04g01740	heat shock protein, putative, expressed
Chr.4	484829	484829	G	G	T	SNP	OS04G0107900	Intronic	NA	NA	LOC_Os04g01740	heat shock protein, putative, expressed
Chr.4	619511	619511	C	C	T	SNP	OS04G0110600	Intronic	NA	NA	LOC_Os04g02000	zinc finger family protein, putative, expressed
Chr.4	5325061	5325061	C	C	T	SNP	OS04G0178300	Exonic	CDS	Silent	LOC_Os04g09900	ent-kaurene synthase, chloroplast precursor, putative, expressed
Chr.4	5325054	5325054	C	C	T	SNP	OS04G0178300	Exonic	CDS	Missense	LOC_Os04g09900	ent-kaurene synthase, chloroplast precursor, putative, expressed
Chr.4	5321459	5321459	C	C	T	SNP	OS04G0178300	Intronic	NA	NA	LOC_Os04g09900	ent-kaurene synthase, chloroplast precursor, putative, expressed
Chr.4	5321507	5321507	C	C	T	SNP	OS04G0178300	Intronic	NA	NA	LOC_Os04g09900	ent-kaurene synthase, chloroplast precursor, putative, expressed
Chr.4	5321582	5321582	C	C	T	SNP	OS04G0178300	Intronic	NA	NA	LOC_Os04g09900	ent-kaurene synthase, chloroplast precursor, putative, expressed
Chr.4	5321609	5321609	C	C	T	SNP	OS04G0178300	Intronic	NA	NA	LOC_Os04g09900	ent-kaurene synthase, chloroplast precursor, putative, expressed
Chr.4	5321628	5321628	C	C	T	SNP	OS04G0178300	Intronic	NA	NA	LOC_Os04g09900	ent-kaurene synthase, chloroplast precursor, putative, expressed
Chr.4	5321630	5321630	C	C	T	SNP	OS04G0178300	Intronic	NA	NA	LOC_Os04g09900	ent-kaurene synthase, chloroplast precursor, putative, expressed
Chr.4	5324392	5324392	C	C	T	SNP	OS04G0178300	Intronic	NA	NA	LOC_Os04g09900	ent-kaurene synthase, chloroplast precursor, putative, expressed
Chr.4	5324472	5324472	G	G	T	SNP	OS04G0178300	Intronic	NA	NA	LOC_Os04g09900	ent-kaurene synthase, chloroplast precursor, putative, expressed
Chr.4	5324973	5324973	C	C	T	SNP	OS04G0178300	Intronic	NA	NA	LOC_Os04g09900	ent-kaurene synthase, chloroplast precursor, putative, expressed
Chr.4	5522438	5522438	C	C	T	SNP	OS04G0180900	Exonic	3UTR	NA	LOC_Os04g10214	expressed protein
Chr.4	5522087	5522087	C	C	T	SNP	OS04G0180900	Exonic	CDS	Missense	LOC_Os04g10214	expressed protein
Chr.4	6477412	6477412	C	C	T	SNP	OS04G0194500	Exonic	CDS	Silent	LOC_Os04g11820	white-brown complex homolog protein, putative, expressed
Chr.4	6479639	6479639	C	C	T	SNP	OS04G0194500	Exonic	CDS	Silent	LOC_Os04g11820	white-brown complex homolog protein, putative, expressed
Chr.4	6479994	6479994	C	C	T	SNP	OS04G0194500	Intronic	NA	NA	LOC_Os04g11820	white-brown complex homolog protein, putative, expressed
Chr.4	7308113	7308113	G	G	T	SNP	OS04G0209200	Intronic	NA	NA	LOC_Os04g13210	multidrug resistance-associated protein, putative, expressed
Chr.4	7309885	7309885	C	C	T	SNP	OS04G0209200	Intronic	NA	NA	LOC_Os04g13210	multidrug resistance-associated protein, putative, expressed
Chr.4	8587903	8587903	G	G	T	SNP	OS04G0228100	Exonic	CDS	Silent	LOC_Os04g15800	expressed protein
Chr.4	8588037	8588037	C	C	T	SNP	OS04G0228100	Intronic	NA	NA	LOC_Os04g15800	expressed protein
Chr.4	11237232	11237232	G	G	T	SNP	OS04G0269600	Exonic	CDS	Missense	LOC_Os04g20164	amine oxidase precursor, putative, expressed
Chr.4	13647482	13647482	G	G	T	SNP	OS04G0304200	Intronic	NA	NA	LOC_Os04g23890	AGC_PVPK_like_kin82y.10 - ACG kinases include homologs to PKA, PKG and PKC, expressed
Chr.4	13650727	13650727	C	C	T	SNP	OS04G0304200	Intronic	NA	NA	LOC_Os04g23890	AGC_PVPK_like_kin82y.10 - ACG kinases include homologs to PKA, PKG and PKC, expressed

Chr.4	13651234	13651234	A	A	T	SNP	OS04G0304200	Intronic	NA	NA	LOC_Os04g23890	AGC_PVPK_like_kin82y.10 - ACG kinases include homologs to PKA, PKG and PKC, expressed
Chr.4	16004797	16004797	G	G	T	SNP	OS04G0339400	Exonic	CDS	Silent	LOC_Os04g27060	oxidoreductase, aldo/keto reductase family protein, putative, expressed
Chr.4	16002083	16002083	G	G	T	SNP	OS04G0339400	Intronic	NA	NA	LOC_Os04g27060	oxidoreductase, aldo/keto reductase family protein, putative, expressed
Chr.4	16002229	16002229	C	C	T	SNP	OS04G0339400	Intronic	NA	NA	LOC_Os04g27060	oxidoreductase, aldo/keto reductase family protein, putative, expressed
Chr.4	20359031	20359031	C	C	T	SNP	OS04G0412100	Exonic	CDS	Missense	LOC_Os04g33610	expressed protein
Chr.4	20359349	20359349	C	C	T	SNP	OS04G0412100	Exonic	CDS	Missense	LOC_Os04g33610	expressed protein
Chr.4	20425931	20425931	C	C	T	SNP	OS04G0413500	Exonic	CDS	Silent	LOC_Os04g33740	glycosyl hydrolases, putative, expressed
Chr.4	20425861	20425861	G	G	T	SNP	OS04G0413500	Exonic	CDS	Missense	LOC_Os04g33740	glycosyl hydrolases, putative, expressed
Chr.4	20424593	20424593	C	C	T	SNP	OS04G0413500	Intronic	NA	NA	LOC_Os04g33740	glycosyl hydrolases, putative, expressed
Chr.4	20426385	20426385	C	C	T	SNP	OS04G0413500	Intronic	NA	NA	LOC_Os04g33740	glycosyl hydrolases, putative, expressed
Chr.4	20708141	20708141	C	C	T	SNP	OS04G0418800	Exonic	CDS	Missense	LOC_Os04g34170	retrotransposon protein, putative, unclassified, expressed
Chr.4	22018016	22018016	G	G	T	SNP	OS04G0442200	Exonic	CDS	Silent	LOC_Os04g36062	expressed protein
Chr.4	22258608	22258608	G	G	T	SNP	OS04G0446500	Exonic	CDS	Silent	LOC_Os04g36890	peptidyl-prolyl cis-trans isomerase, FKBP-type, putative, expressed
Chr.4	22624796	22624796	A	A	T	SNP	OS04G0453233	Intronic	NA	NA	LOC_Os04g38026	transporter family protein, putative, expressed
Chr.4	22625428	22625428	C	C	T	SNP	OS04G0453233	Intronic	NA	NA	LOC_Os04g38026	transporter family protein, putative, expressed
Chr.4	22625614	22625614	C	C	T	SNP	OS04G0453233	Intronic	NA	NA	LOC_Os04g38026	transporter family protein, putative, expressed
Chr.4	23409229	23409229	C	C	T	SNP	OS04G0468600	Exonic	CDS	Missense	LOC_Os04g39320	expressed protein
Chr.4	24302460	24302460	A	A	T	SNP	OS04G0486500	Intronic	NA	NA	LOC_Os04g40940	mitotic spindle checkpoint protein MAD2, putative, expressed
Chr.4	24326664	24326664	C	C	T	SNP	OS04G0486950	Exonic	CDS	Silent	LOC_Os04g40990	malate synthase, glyoxysomal, putative, expressed
Chr.4	24326221	24326221	A	A	T	SNP	OS04G0486950	Exonic	CDS	Missense	LOC_Os04g40990	malate synthase, glyoxysomal, putative, expressed
Chr.4	25194032	25194032	C	C	T	SNP	OS04G0504800	Exonic	3UTR	NA	LOC_Os04g42600	polyadenylate-binding protein, putative, expressed
Chr.4	27567935	27567935	C	C	T	SNP	OS04G0550600	Exonic	CDS	Missense	LOC_Os04g46470	carotenoid cleavage dioxygenase 7, chloroplast precursor, putative, expressed
Chr.4	27568734	27568734	G	G	T	SNP	OS04G0550600	Intronic	NA	NA	LOC_Os04g46470	carotenoid cleavage dioxygenase 7, chloroplast precursor, putative, expressed
Chr.4	28304277	28304277	A	A	T	SNP	OS04G0564700	Intronic	NA	NA	LOC_Os04g47700	expressed protein
Chr.4	29043026	29043026	C	C	T	SNP	OS04G0576500	Exonic	5UTR	NA	LOC_Os04g48710	expressed protein
Chr.4	29045917	29045917	C	C	T	SNP	OS04G0576500	Exonic	CDS	Missense	LOC_Os04g48710	expressed protein
Chr.4	29523458	29523458	G	G	T	SNP	OS04G0584500	Exonic	CDS	Missense	LOC_Os04g49500	U-box domain-containing protein, putative, expressed
Chr.4	29525730	29525730	C	C	T	SNP	OS04G0584500	Intronic	NA	NA	LOC_Os04g49500	U-box domain-containing protein, putative, expressed
Chr.4	29557939	29557939	C	C	T	SNP	OS04G0585100	Intronic	NA	NA	LOC_Os04g49560	zinc finger family protein, putative, expressed
Chr.4	29557953	29557953	G	G	T	SNP	OS04G0585100	Intronic	NA	NA	LOC_Os04g49560	zinc finger family protein, putative, expressed
Chr.4	29661444	29661444	G	G	T	SNP	OS04G0587400	Exonic	CDS	Silent	LOC_Os04g49748	purine permease, putative, expressed
Chr.4	29661627	29661627	G	G	T	SNP	OS04G0587400	Exonic	CDS	Silent	LOC_Os04g49748	purine permease, putative, expressed
Chr.4	29661960	29661960	G	G	T	SNP	OS04G0587400	Exonic	CDS	Silent	LOC_Os04g49748	purine permease, putative, expressed
Chr.4	30172973	30172973	C	C	T	SNP	OS04G0598000	Intronic	NA	NA	LOC_Os04g50970	seed specific protein Bn15D1B, putative, expressed
Chr.4	30939418	30939418	C	C	T	SNP	OS04G0610400	Exonic	CDS	Silent	LOC_Os04g52090	AP2 domain containing protein, expressed
Chr.4	31062206	31062206	C	C	T	SNP	OS04G0612500	Exonic	3UTR	NA	LOC_Os04g52260	LTPL124 - Protease inhibitor/seed storage/LTP family protein precursor, expressed
Chr.4	31182409	31182409	G	G	T	SNP	OS04G0614600	Exonic	3UTR	NA	LOC_Os04g52450	aminotransferase, putative, expressed
Chr.4	31182411	31182411	A	A	T	SNP	OS04G0614600	Exonic	3UTR	NA	LOC_Os04g52450	aminotransferase, putative, expressed
Chr.4	31242782	31242782	G	G	T	SNP	OS04G0615700	Exonic	CDS	Missense	LOC_Os04g52540	retrotransposon protein, putative, unclassified, expressed
Chr.4	31281938	31281938	G	G	T	SNP	OS04G0616300	Intronic	NA	NA	LOC_Os04g52600	SHR5-receptor-like kinase, putative, expressed
Chr.4	31440122	31440122	G	G	T	SNP	OS04G0619000	Exonic	3UTR	NA	LOC_Os04g52810	no apical meristem protein, putative, expressed
Chr.4	31442260	31442260	C	C	T	SNP	OS04G0619000	Exonic	3UTR	NA	LOC_Os04g52810	no apical meristem protein, putative, expressed
Chr.4	33126775	33126775	C	C	T	SNP	OS04G0650000	Exonic	CDS	Missense	LOC_Os04g55650	oryzain alpha chain precursor, putative, expressed
Chr.4	33180567	33180567	G	G	T	SNP	OS04G0651000	Intronic	NA	NA	LOC_Os04g55740	peroxidase precursor, putative, expressed
Chr.4	33255007	33255007	C	C	T	SNP	OS04G0652700	Intronic	NA	NA	LOC_Os04g55850	nuclease PA3, putative, expressed
Chr.4	33255068	33255068	C	C	T	SNP	OS04G0652700	Intronic	NA	NA	LOC_Os04g55850	nuclease PA3, putative, expressed
Chr.4	34598330	34598330	C	C	T	SNP	OS04G0677300	Exonic	CDS	Missense	LOC_Os04g58090	harpin-induced protein 1 domain containing protein, expressed
Chr.4	35444439	35444439	C	C	T	SNP	OS04G0692600	Intronic	NA	NA	LOC_Os04g59610	expressed protein
Chr.4	4458260	4458262	TAA	TAA	T	INDEL	OS04G0165700	Intronic	NA	NA	LOC_Os04g08350	cysteine synthase, chloroplast/chromoplast precursor, putative, expressed
Chr.4	4458312	4458322	TATTTTTTTTA	TATTTTTTTTA	T	INDEL	OS04G0165700	Intronic	NA	NA	LOC_Os04g08350	cysteine synthase, chloroplast/chromoplast precursor, putative, expressed
Chr.4	4458797	4458798	TA	TA	T	INDEL	OS04G0165700	Intronic	NA	NA	LOC_Os04g08350	cysteine synthase, chloroplast/chromoplast precursor, putative, expressed
Chr.4	5325714	5325715	TG	TG	T	INDEL	OS04G0178300	Intronic	NA	NA	LOC_Os04g09900	ent-kaurene synthase, chloroplast precursor, putative, expressed
Chr.4	5325779	5325780	TA	TA	T	INDEL	OS04G0178300	Intronic	NA	NA	LOC_Os04g09900	ent-kaurene synthase, chloroplast precursor, putative, expressed
Chr.4	7308087	7308088	TG	TG	T	INDEL	OS04G0209200	Intronic	NA	NA	LOC_Os04g13210	multidrug resistance-associated protein, putative, expressed
Chr.4	11240241	11240242	TG	TG	T	INDEL	OS04G0269600	Intronic	NA	NA	LOC_Os04g20164	amine oxidase precursor, putative, expressed
Chr.4	11276081	11276082	TG	TG	T	INDEL	OS04G0270100	Intronic	NA	NA	LOC_Os04g20220	elongation factor Tu, putative, expressed
Chr.4	16002882	16002883	TA	TA	T	INDEL	OS04G0339400	Intronic	NA	NA	LOC_Os04g27060	oxidoreductase, aldo/keto reductase family protein, putative, expressed
Chr.4	16004587	16004588	TC	TC	T	INDEL	OS04G0339400	Intronic	NA	NA	LOC_Os04g27060	oxidoreductase, aldo/keto reductase family protein, putative, expressed
Chr.4	20389208	20389211	TGTG	TGTG	T	INDEL	OS04G0412500	Intronic	NA	NA	LOC_Os04g33660	bifunctional monodehydroascorbate reductase and carbonic anhydrasenectarin-3 precursor, putative, expressed
Chr.4	20424624	20424625	TA	TA	T	INDEL	OS04G0413500	Intronic	NA	NA	LOC_Os04g33740	glycosyl hydrolases, putative, expressed
Chr.4	20425409	20425410	TG	TG	T	INDEL	OS04G0413500	Intronic	NA	NA	LOC_Os04g33740	glycosyl hydrolases, putative, expressed
Chr.4	22022509	22022511	TTC	TTC	T	INDEL	OS04G0442200	Intronic	NA	NA	LOC_Os04g36062	expressed protein
Chr.4	22624132	22624133	TA	TA	T	INDEL	OS04G0453233	Intronic	NA	NA	LOC_Os04g38026	transporter family protein, putative, expressed
Chr.4	23422666	23422667	TC	TC	T	INDEL	OS04G0469000	Exonic	3UTR	NA	LOC_Os04g39350	heavy metal associated domain containing protein, expressed
Chr.4	24302696	24302697	TA	TA	T	INDEL	OS04G0486500	Intronic	NA	NA	LOC_Os04g40940	mitotic spindle checkpoint protein MAD2, putative, expressed
Chr.4	27508204	27508213	TTCATCCCAG	TTCATCCCAG	T	INDEL	OS04G0549600	Intronic	NA	NA	LOC_Os04g46390	chaperone protein dnaJ, putative, expressed
Chr.4	27787137	27787146	TCGACCGTGG	TCGACCGTGG	T	INDEL	OS04G0555300	Exonic	5UTR	NA	LOC_Os04g46880	transporter, major facilitator family, putative, expressed
Chr.4	28085736	28085737	TA	TA	T	INDEL	OS04G0560700	Exonic	3UTR	NA	LOC_Os04g47310	expressed protein
Chr.4	29134477	29134501	TCGGACTCCGAGGATATCCGAAGCC	TCGGACTCCGAGGATATCCGAAGCC	T	INDEL	OS04G0578000	Exonic	5UTR	NA	LOC_Os04g48850	aminotransferase, classes I and II, domain containing protein, expressed
Chr.4	29317543	29317552	TGCCATTGTA	TGCCATTGTA	T	INDEL	OS04G0580800	Exonic	3UTR	NA	LOC_Os04g49160	zinc finger, C3HC4 type domain containing protein, expressed
Chr.4	29340369	29340370	TA	TA	T	INDEL	OS04G0581100	Intronic	NA	NA	LOC_Os04g49210	naringenin,2-oxoglutarate 3-dioxygenase, putative, expressed
Chr.4	29976699	29976701	TCA	TCA	T	INDEL	OS04G0592600	Intronic	NA	NA	LOC_Os04g50216	SNARE associated Golgi protein, putative, expressed
Chr.4	30287650	30287652	TCA	TCA	T	INDEL	OS04G0600200	Intronic	NA	NA	LOC_Os04g51150	transposon protein, putative, unclassified, expressed
Chr.4	31442514	31442515	TA	TA	T	INDEL	OS04G0619000	Exonic	3UTR	NA	LOC_Os04g52810	no apical meristem protein, putative, expressed
Chr.4	31442977	31442978	TA	TA	T	INDEL	OS04G0619000	Exonic	3UTR	NA	LOC_Os04g52810	no apical meristem protein, putative, expressed
Chr.4	33418717	33418725	TGAGATGAG	TGAGATGAG	T	INDEL	OS04G0655300	Exonic	5UTR	NA	LOC_Os04g56110	protein kinase, putative, expressed
Chr.4	33532351	33532365	TTTTTAATATATATA	TTTTTAATATATATA	T	INDEL	OS04G0657500	Intronic	NA	NA	LOC_Os04g56240	lipase, putative, expressed
Chr.3	538360	538360	C	C	T	SNP	OS03G0109300	Intronic	NA	NA	LOC_Os03g01880	possible lysine decarboxylase domain containing protein, expressed
Chr.3	538366	538366	A	A	T	SNP	OS03G0109300	Intronic	NA	NA	LOC_Os03g01880	possible lysine decarboxylase domain containing protein, expressed
Chr.3	538616	538616	A	A	T	SNP	OS03G0109300	Intronic	NA	NA	LOC_Os03g01880	possible lysine decarboxylase domain containing protein, expressed
Chr.3	538709	538709	C	C	T	SNP	OS03G0109300	Intronic	NA	NA	LOC_Os03g01880	possible lysine decarboxylase domain containing protein, expressed
Chr.3	538763	538763	C	C	T	SNP	OS03G0109300	Intronic	NA	NA	LOC_Os03g01880	possible lysine decarboxylase domain containing protein, expressed
Chr.3	541276	541276	C	C	T	SNP	OS03G0109300	Intronic	NA	NA	LOC_Os03g01880	possible lysine decarboxylase domain containing protein, expressed
Chr.3	908521	908521	C	C	T	SNP	OS03G0116000	Exonic	CDS	Silent	LOC_Os03g02480	inner membrane protein, putative, expressed
Chr.3	908174	908174	C	C	T	SNP	OS03G0116000	Intronic	NA	NA	LOC_Os03g02480	inner membrane protein, putative, expressed

Chr.3	1608449	1608449	C	C	T	SNP	OS03G0128300	Exonic	CDS	Silent	LOC_Os03g03630	expressed protein
Chr.3	1612497	1612497	A	A	T	SNP	OS03G0128500	Exonic	3UTR	NA	LOC_Os03g03650	POLD2 - Putative DNA polymerase delta complex subunit, expressed
Chr.3	1627188	1627188	A	A	T	SNP	OS03G0128700	Intronic	NA	NA	LOC_Os03g03660	CAMK_CAMK_like.17 - CAMK includes calcium/calmodulin depedent protein kinases, expressed
Chr.3	1628235	1628235	C	C	T	SNP	OS03G0128700	Intronic	NA	NA	LOC_Os03g03660	CAMK_CAMK_like.17 - CAMK includes calcium/calmodulin depedent protein kinases, expressed
Chr.3	1628682	1628682	C	C	T	SNP	OS03G0128700	Intronic	NA	NA	LOC_Os03g03660	CAMK_CAMK_like.17 - CAMK includes calcium/calmodulin depedent protein kinases, expressed
Chr.3	1718132	1718132	C	C	T	SNP	OS03G0130400	Intronic	NA	NA	LOC_Os03g03820	adenylate kinase, putative, expressed
Chr.3	1742135	1742135	C	C	T	SNP	OS03G0130700	Exonic	5UTR	NA	LOC_Os03g03860	expressed protein
Chr.3	1738953	1738953	C	C	T	SNP	OS03G0130700	Exonic	CDS	NA	LOC_Os03g03860	expressed protein
Chr.3	1741318	1741318	C	C	T	SNP	OS03G0130700	Exonic	CDS	NA	LOC_Os03g03860	expressed protein
Chr.3	1741098	1741098	C	C	T	SNP	OS03G0130700	Intronic	NA	NA	LOC_Os03g03860	expressed protein
Chr.3	1790724	1790724	C	C	T	SNP	OS03G0131200	Exonic	5UTR	NA	LOC_Os03g03910	catalase domain containing protein, expressed
Chr.3	1788535	1788535	C	C	T	SNP	OS03G0131200	Intronic	NA	NA	LOC_Os03g03910	catalase domain containing protein, expressed
Chr.3	1984508	1984508	G	G	T	SNP	OS03G0135700	Intronic	NA	NA	LOC_Os03g04310	BHLH transcription factor, putative, expressed
Chr.3	3371870	3371870	G	G	T	SNP	OS03G0162200	Exonic	CDS	Silent	LOC_Os03g06670	Core histone H2A/H2B/H3/H4 domain containing protein, putative, expressed
Chr.3	4417841	4417841	C	C	T	SNP	OS03G0184100	Exonic	CDS	Silent	LOC_Os03g08580	expressed protein
Chr.3	5113784	5113784	G	G	T	SNP	OS03G0197200	Exonic	CDS	NA	LOC_Os03g10100	transporter family protein, putative, expressed
Chr.3	7349377	7349377	C	C	T	SNP	OS03G0239200	Intronic	NA	NA	LOC_Os03g13590	potyvirus VPg interacting protein, putative, expressed
Chr.3	7852222	7852222	G	G	T	SNP	OS03G0248600	Intronic	NA	NA	LOC_Os03g14450	enolase, putative, expressed
Chr.3	10123997	10123997	A	A	T	SNP	OS03G0291500	Intronic	NA	NA	LOC_Os03g18130	asparagine synthetase, putative, expressed
Chr.3	10124042	10124042	C	C	T	SNP	OS03G0291500	Intronic	NA	NA	LOC_Os03g18130	asparagine synthetase, putative, expressed
Chr.3	10124075	10124075	G	G	T	SNP	OS03G0291500	Intronic	NA	NA	LOC_Os03g18130	asparagine synthetase, putative, expressed
Chr.3	10601193	10601193	C	C	T	SNP	OS03G0301200	Exonic	3UTR	NA	LOC_Os03g18910	COBRA-like protein 7 precursor, putative, expressed
Chr.3	11405008	11405008	C	C	T	SNP	OS03G0317000	Exonic	CDS	Missense	LOC_Os03g20180	ECT protein, putative, expressed
Chr.3	11524261	11524261	G	G	T	SNP	OS03G0319300	Exonic	3UTR	NA	LOC_Os03g20370	OsCam1-1 - Calmodulin, expressed
Chr.3	14480919	14480919	C	C	T	SNP	OS03G0369000	Exonic	CDS	Silent	LOC_Os03g25340	peroxidase precursor, putative, expressed
Chr.3	14481228	14481228	G	G	T	SNP	OS03G0369000	Intronic	NA	NA	LOC_Os03g25340	peroxidase precursor, putative, expressed
Chr.3	29115907	29115907	C	C	T	SNP	OS03G0719000	Intronic	NA	NA	LOC_Os03g50970	retrotransposon protein, putative, Ty1-copia subclass, expressed
Chr.3	29140944	29140944	C	C	T	SNP	OS03G0719300	Intronic	NA	NA	LOC_Os03g51000	3,4-dihydroxy-2-butanone kinase, putative, expressed
Chr.3	29145339	29145339	C	C	T	SNP	OS03G0719300	Intronic	NA	NA	LOC_Os03g51000	3,4-dihydroxy-2-butanone kinase, putative, expressed
Chr.3	35792695	35792695	G	G	T	SNP	OS03G0850400	Intronic	NA	NA	LOC_Os03g63330	aspartokinase, chloroplast precursor, putative, expressed
Chr.3	35874922	35874922	C	C	T	SNP	OS03G0851900	Exonic	CDS	Missense	LOC_Os03g63500	ATPase, AFG1 family domain containing protein, expressed
Chr.3	35869235	35869235	C	C	T	SNP	OS03G0851900	Intronic	NA	NA	LOC_Os03g63500	ATPase, AFG1 family domain containing protein, expressed
Chr.3	35869251	35869251	C	C	T	SNP	OS03G0851900	Intronic	NA	NA	LOC_Os03g63500	ATPase, AFG1 family domain containing protein, expressed
Chr.3	35869267	35869267	C	C	T	SNP	OS03G0851900	Intronic	NA	NA	LOC_Os03g63500	ATPase, AFG1 family domain containing protein, expressed
Chr.3	29140932	29140933	TA	TA	T	INDEL	OS03G0719300	Intronic	NA	NA	LOC_Os03g51000	3,4-dihydroxy-2-butanone kinase, putative, expressed
Chr.3	14850354	14850355	TC	TC	T	INDEL	OS03G0376100	Exonic	3UTR	NA	LOC_Os03g25940	cystathionine gamma-synthase, putative, expressed
Chr.3	12007673	12007674	TG	TG	T	INDEL	OS03G0328000	Exonic	3UTR	NA	LOC_Os03g21080	guanine nucleotide exchange factor, putative, expressed
Chr.3	4418616	4418617	TA	TA	T	INDEL	OS03G0184100	Intronic	NA	NA	LOC_Os03g08580	expressed protein
Chr.3	907590	907591	TA	TA	T	INDEL	OS03G0116000	Intronic	NA	NA	LOC_Os03g02480	inner membrane protein, putative, expressed
Chr.3	656646	656648	TTC	TTC	T	INDEL	OS03G0111400	Exonic	3UTR	NA	LOC_Os03g02070	heavy-metal-associated domain-containing protein, putative, expressed
Chr.3	1237863	1237863	G	G	T	SNP	OS03G0122300	Intronic	NA	NA	LOC_Os03g03034	flavonol synthase/flavanone 3-hydroxylase, putative, expressed
Chr.3	2748798	2748798	G	G	T	SNP	OS03G0149300	Exonic	CDS	Silent	LOC_Os03g05530	nodulin, putative, expressed
Chr.3	3529336	3529336	G	G	T	SNP	OS03G0165400	Intronic	NA	NA	LOC_Os03g06940	beta-galactosidase, putative, expressed
Chr.3	3563731	3563731	G	G	T	SNP	OS03G0166000	Intronic	NA	NA	LOC_Os03g06980	nucleic acid binding protein, putative, expressed
Chr.3	6777043	6777043	G	G	T	SNP	OS03G0228200	Exonic	CDS	Missense	LOC_Os03g12700	expressed protein
Chr.3	8201235	8201235	G	G	T	SNP	OS03G0255100	Intronic	NA	NA	LOC_Os03g15020	beta-galactosidase precursor, putative, expressed
Chr.3	8223152	8223152	G	G	T	SNP	OS03G0255500	Intronic	NA	NA	LOC_Os03g15050	phosphoenolpyruvate carboxykinase, putative, expressed
Chr.3	10996387	10996387	G	G	T	SNP	OS03G0308800	Intronic	NA	NA	LOC_Os03g19560	tetratricopeptide repeat domain containing protein, expressed
Chr.3	12411458	12411458	G	G	T	SNP	OS03G0335500	Intronic	NA	NA	LOC_Os03g21730	receptor-like protein kinase precursor, putative, expressed
Chr.3	16286694	16286694	G	G	T	SNP	OS03G0401100	Intronic	NA	NA	LOC_Os03g28300	protein kinase domain containing protein, expressed
Chr.3	16595768	16595768	G	G	T	SNP	OS03G0405500	Exonic	3UTR	NA	LOC_Os03g29190	PDI, putative, expressed
Chr.3	16594294	16594294	G	G	T	SNP	OS03G0405500	Exonic	CDS	Missense	LOC_Os03g29190	PDI, putative, expressed
Chr.3	16590798	16590798	G	G	T	SNP	OS03G0405500	Intronic	NA	NA	LOC_Os03g29190	PDI, putative, expressed
Chr.3	23372311	23372311	G	G	T	SNP	OS03G0616700	Intronic	NA	NA	LOC_Os03g42050	exportin-7-A, putative, expressed
Chr.3	23486704	23486704	G	G	T	SNP	OS03G0619400	Intronic	NA	NA	LOC_Os03g42220	T-complex protein, putative, expressed
Chr.3	24467370	24467370	G	G	T	SNP	OS03G0638400	Intronic	NA	NA	LOC_Os03g43750	retrotransposon, putative, centromere-specific, expressed
Chr.3	24597114	24597114	G	G	T	SNP	OS03G0639800	Intronic	NA	NA	LOC_Os03g43860	SNF7 domain containing protein, putative, expressed
Chr.3	24814813	24814813	G	G	T	SNP	OS03G0643300	Intronic	NA	NA	LOC_Os03g44150	aminotransferase, putative, expressed
Chr.3	24816169	24816169	G	G	T	SNP	OS03G0643300	Intronic	NA	NA	LOC_Os03g44150	aminotransferase, putative, expressed
Chr.3	24816808	24816808	G	G	T	SNP	OS03G0643300	Intronic	NA	NA	LOC_Os03g44150	aminotransferase, putative, expressed
Chr.3	27461260	27461260	G	G	T	SNP	OS03G0688200	Intronic	NA	NA	LOC_Os03g48260	expressed protein
Chr.3	27495775	27495775	G	G	T	SNP	OS03G0689100	Intronic	NA	NA	LOC_Os03g48300	histidine acid phosphatase, putative, expressed
Chr.3	27499018	27499018	G	G	T	SNP	OS03G0689100	Intronic	NA	NA	LOC_Os03g48300	histidine acid phosphatase, putative, expressed
Chr.3	28241243	28241243	G	G	T	SNP	OS03G0703000	Intronic	NA	NA	LOC_Os03g49600	Os3bglu7 - beta-glucosidase, exo-beta-glucanse, expressed
Chr.3	28261575	28261575	G	G	T	SNP	OS03G0703300	Exonic	CDS	Missense	LOC_Os03g49630	expressed protein
Chr.3	29387110	29387110	G	G	T	SNP	OS03G0723400	Intronic	NA	NA	LOC_Os03g51350	expressed protein
Chr.3	29455859	29455859	G	G	T	SNP	OS03G0724700	Intronic	NA	NA	LOC_Os03g51479	maf, putative, expressed
Chr.3	32625867	32625867	G	G	T	SNP	OS03G0786000	Intronic	NA	NA	LOC_Os03g57210	glycine-rich protein, putative, expressed
Chr.3	32666184	32666184	G	G	T	SNP	OS03G0786700	Intronic	NA	NA	LOC_Os03g57280	N-dimethylguanosine tRNA methyltransferase, putative, expressed
Chr.3	33347693	33347693	G	G	T	SNP	OS03G0799900	Intronic	NA	NA	LOC_Os03g58570	WD domain, G-beta repeat domain containing protein, expressed
Chr.3	541096	541096	C	C	T	SNP	OS03G0109300	Intronic	NA	NA	LOC_Os03g01880	possible lysine decarboxylase domain containing protein, expressed
Chr.3	541101	541101	C	C	T	SNP	OS03G0109300	Intronic	NA	NA	LOC_Os03g01880	possible lysine decarboxylase domain containing protein, expressed
Chr.3	1236762	1236762	C	C	T	SNP	OS03G0122300	Intronic	NA	NA	LOC_Os03g03034	flavonol synthase/flavanone 3-hydroxylase, putative, expressed
Chr.3	1237405	1237405	C	C	T	SNP	OS03G0122300	Intronic	NA	NA	LOC_Os03g03034	flavonol synthase/flavanone 3-hydroxylase, putative, expressed
Chr.3	1237542	1237542	C	C	T	SNP	OS03G0122300	Intronic	NA	NA	LOC_Os03g03034	flavonol synthase/flavanone 3-hydroxylase, putative, expressed
Chr.3	1238942	1238942	C	C	T	SNP	OS03G0122300	Intronic	NA	NA	LOC_Os03g03034	flavonol synthase/flavanone 3-hydroxylase, putative, expressed
Chr.3	1242890	1242890	C	C	T	SNP	OS03G0122300	Intronic	NA	NA	LOC_Os03g03034	flavonol synthase/flavanone 3-hydroxylase, putative, expressed
Chr.3	1243922	1243922	C	C	T	SNP	OS03G0122300	Intronic	NA	NA	LOC_Os03g03034	flavonol synthase/flavanone 3-hydroxylase, putative, expressed
Chr.3	1244027	1244027	C	C	T	SNP	OS03G0122300	Intronic	NA	NA	LOC_Os03g03034	flavonol synthase/flavanone 3-hydroxylase, putative, expressed
Chr.3	1244102	1244102	C	C	T	SNP	OS03G0122300	Intronic	NA	NA	LOC_Os03g03034	flavonol synthase/flavanone 3-hydroxylase, putative, expressed
Chr.3	1876432	1876432	C	C	T	SNP	OS03G0133300	Intronic	NA	NA	LOC_Os03g04100	expressed protein
Chr.3	2750586	2750586	C	C	T	SNP	OS03G0149300	Intronic	NA	NA	LOC_Os03g05530	nodulin, putative, expressed
Chr.3	3529579	3529579	C	C	T	SNP	OS03G0165400	Intronic	NA	NA	LOC_Os03g06940	beta-galactosidase, putative, expressed
Chr.3	3530762	3530762	C	C	T	SNP	OS03G0165400	Intronic	NA	NA	LOC_Os03g06940	beta-galactosidase, putative, expressed
Chr.3	3564334	3564334	C	C	T	SNP	OS03G0166000	Intronic	NA	NA	LOC_Os03g06980	nucleic acid binding protein, putative, expressed

Chr.3	4002277	4002277	C	C	T	SNP	OS03G0174900	Exonic	3UTR	NA	LOC_Os03g07880	nuclear transcription factor Y subunit, putative, expressed
Chr.3	5980025	5980025	C	C	T	SNP	OS03G0214100	Exonic	CDS	Missense	LOC_Os03g11540	RPA1B - Putative single-stranded DNA binding complex subunit 1, expressed
Chr.3	6369979	6369979	C	C	T	SNP	OS03G0221500	Intronic	NA	NA	LOC_Os03g12140	glucan endo-1,3-beta-glucosidase precursor, putative, expressed
Chr.3	6776572	6776572	C	C	T	SNP	OS03G0228200	Exonic	3UTR	NA	LOC_Os03g12700	expressed protein
Chr.3	7566599	7566599	C	C	T	SNP	OS03G0243100	Intronic	NA	NA	LOC_Os03g13950	actin-depolymerizing factor, putative, expressed
Chr.3	8789401	8789401	C	C	T	SNP	OS03G0265900	Exonic	3UTR	NA	LOC_Os03g15920	expressed protein
Chr.3	9014452	9014452	C	C	T	SNP	OS03G0269900	Intronic	NA	NA	LOC_Os03g16334	fringe-related protein, putative, expressed
Chr.3	9878813	9878813	C	C	T	SNP	OS03G0286500	Intronic	NA	NA	LOC_Os03g17760	RNA recognition motif containing protein, putative, expressed
Chr.3	9957686	9957686	C	C	T	SNP	OS03G0288000	Intronic	NA	NA	LOC_Os03g17870	metallothionein, putative, expressed
Chr.3	10041603	10041603	C	C	T	SNP	OS03G0289800	Intronic	NA	NA	LOC_Os03g18030	leucoanthocyanidin dioxygenase, putative, expressed
Chr.3	10906116	10906116	C	C	T	SNP	OS03G0306700	Exonic	5UTR	NA	LOC_Os03g19370	CPuORF4 - conserved peptide uORF-containing transcript, expressed
Chr.3	10996042	10996042	C	C	T	SNP	OS03G0308800	Exonic	CDS	Silent	LOC_Os03g19560	tetratricopeptide repeat domain containing protein, expressed
Chr.3	10996885	10996885	C	C	T	SNP	OS03G0308800	Intronic	NA	NA	LOC_Os03g19560	tetratricopeptide repeat domain containing protein, expressed
Chr.3	12352143	12352143	C	C	T	SNP	OS03G0334100	Exonic	3UTR	NA	LOC_Os03g21630	PRC-barrel domain protein, putative, expressed
Chr.3	12349670	12349670	C	C	T	SNP	OS03G0334100	Intronic	NA	NA	LOC_Os03g21630	PRC-barrel domain protein, putative, expressed
Chr.3	12412994	12412994	C	C	T	SNP	OS03G0335500	Exonic	CDS	Silent	LOC_Os03g21730	receptor-like protein kinase precursor, putative, expressed
Chr.3	12587922	12587922	C	C	T	SNP	OS03G0338400	Intronic	NA	NA	LOC_Os03g21970	basic helix-loop-helix domain containing protein, expressed
Chr.3	12701694	12701694	C	C	T	SNP	OS03G0340900	Exonic	CDS	Missense	LOC_Os03g22160	relA-SpoT like protein RSH1, putative, expressed
Chr.3	15669519	15669519	C	C	T	SNP	OS03G0391100	Intronic	NA	NA	LOC_Os03g27360	RING-H2 finger protein ATL5H precursor, putative, expressed
Chr.3	16288380	16288380	C	C	T	SNP	OS03G0401100	Intronic	NA	NA	LOC_Os03g28300	protein kinase domain containing protein, expressed
Chr.3	16590794	16590794	C	C	T	SNP	OS03G0405500	Intronic	NA	NA	LOC_Os03g29190	PDI, putative, expressed
Chr.3	16591566	16591566	C	C	T	SNP	OS03G0405500	Intronic	NA	NA	LOC_Os03g29190	PDI, putative, expressed
Chr.3	16592196	16592196	C	C	T	SNP	OS03G0405500	Intronic	NA	NA	LOC_Os03g29190	PDI, putative, expressed
Chr.3	16592392	16592392	C	C	T	SNP	OS03G0405500	Intronic	NA	NA	LOC_Os03g29190	PDI, putative, expressed
Chr.3	16592393	16592393	C	C	T	SNP	OS03G0405500	Intronic	NA	NA	LOC_Os03g29190	PDI, putative, expressed
Chr.3	16839657	16839657	C	C	T	SNP	OS03G0409100	Exonic	5UTR	NA	LOC_Os03g29540	ATP-dependent protease, putative, expressed
Chr.3	20793671	20793671	C	C	T	SNP	OS03G0571900	Intronic	NA	NA	LOC_Os03g37490	MATE efflux family protein, putative, expressed
Chr.3	20795422	20795422	C	C	T	SNP	OS03G0571900	Intronic	NA	NA	LOC_Os03g37490	MATE efflux family protein, putative, expressed
Chr.3	21637152	21637152	C	C	T	SNP	OS03G0586500	Exonic	3UTR	NA	LOC_Os03g38950	chloroplast post-illumination chlorophyll fluorescence increase protein, putative, expressed
Chr.3	21635516	21635516	C	C	T	SNP	OS03G0586500	Intronic	NA	NA	LOC_Os03g38950	chloroplast post-illumination chlorophyll fluorescence increase protein, putative, expressed
Chr.3	23370293	23370293	C	C	T	SNP	OS03G0616700	Intronic	NA	NA	LOC_Os03g42050	exportin-7-A, putative, expressed
Chr.3	23490273	23490273	C	C	T	SNP	OS03G0619400	Exonic	CDS	Silent	LOC_Os03g42220	T-complex protein, putative, expressed
Chr.3	24451144	24451144	C	C	T	SNP	OS03G0638200	Intronic	NA	NA	LOC_Os03g43720	transporter family protein, putative, expressed
Chr.3	24473333	24473333	C	C	T	SNP	OS03G0638400	Exonic	3UTR	NA	LOC_Os03g43750	retrotransposon, putative, centromere-specific, expressed
Chr.3	24705047	24705047	C	C	T	SNP	OS03G0641300	Exonic	3UTR	NA	LOC_Os03g43990	BTBN8 - Bric-a-Brac, Tramtrack, Broad Complex BTB domain with non-phototropic hypocotyl 3 NPH3 and coiled-coil domains, ex
Chr.3	24705171	24705171	C	C	T	SNP	OS03G0641300	Exonic	3UTR	NA	LOC_Os03g43990	BTBN8 - Bric-a-Brac, Tramtrack, Broad Complex BTB domain with non-phototropic hypocotyl 3 NPH3 and coiled-coil domains, ex
Chr.3	24705362	24705362	C	C	T	SNP	OS03G0641300	Exonic	3UTR	NA	LOC_Os03g43990	BTBN8 - Bric-a-Brac, Tramtrack, Broad Complex BTB domain with non-phototropic hypocotyl 3 NPH3 and coiled-coil domains, ex
Chr.3	24814221	24814221	C	C	T	SNP	OS03G0643300	Intronic	NA	NA	LOC_Os03g44150	aminotransferase, putative, expressed
Chr.3	24814840	24814840	C	C	T	SNP	OS03G0643300	Intronic	NA	NA	LOC_Os03g44150	aminotransferase, putative, expressed
Chr.3	24815700	24815700	C	C	T	SNP	OS03G0643300	Intronic	NA	NA	LOC_Os03g44150	aminotransferase, putative, expressed
Chr.3	24816152	24816152	C	C	T	SNP	OS03G0643300	Intronic	NA	NA	LOC_Os03g44150	aminotransferase, putative, expressed
Chr.3	24816325	24816325	C	C	T	SNP	OS03G0643300	Intronic	NA	NA	LOC_Os03g44150	aminotransferase, putative, expressed
Chr.3	24816786	24816786	C	C	T	SNP	OS03G0643300	Intronic	NA	NA	LOC_Os03g44150	aminotransferase, putative, expressed
Chr.3	24817275	24817275	C	C	T	SNP	OS03G0643300	Intronic	NA	NA	LOC_Os03g44150	aminotransferase, putative, expressed
Chr.3	24817384	24817384	C	C	T	SNP	OS03G0643300	Intronic	NA	NA	LOC_Os03g44150	aminotransferase, putative, expressed
Chr.3	26396541	26396541	C	C	T	SNP	OS03G0669100	Exonic	CDS	Nonsense	LOC_Os03g46640	deoxyuridine 5-triphosphate nucleotidohydrolase, putative, expressed
Chr.3	26532662	26532662	C	C	T	SNP	OS03G0672400	Exonic	CDS	Missense	LOC_Os03g46920	expressed protein
Chr.3	27462009	27462009	C	C	T	SNP	OS03G0688200	Intronic	NA	NA	LOC_Os03g48260	expressed protein
Chr.3	27503690	27503690	C	C	T	SNP	OS03G0689100	Exonic	CDS	Missense	LOC_Os03g48300	histidine acid phosphatase, putative, expressed
Chr.3	27494934	27494934	C	C	T	SNP	OS03G0689100	Intronic	NA	NA	LOC_Os03g48300	histidine acid phosphatase, putative, expressed
Chr.3	27495364	27495364	C	C	T	SNP	OS03G0689100	Intronic	NA	NA	LOC_Os03g48300	histidine acid phosphatase, putative, expressed
Chr.3	27495504	27495504	C	C	T	SNP	OS03G0689100	Intronic	NA	NA	LOC_Os03g48300	histidine acid phosphatase, putative, expressed
Chr.3	27495536	27495536	C	C	T	SNP	OS03G0689100	Intronic	NA	NA	LOC_Os03g48300	histidine acid phosphatase, putative, expressed
Chr.3	27495734	27495734	C	C	T	SNP	OS03G0689100	Intronic	NA	NA	LOC_Os03g48300	histidine acid phosphatase, putative, expressed
Chr.3	27497787	27497787	C	C	T	SNP	OS03G0689100	Intronic	NA	NA	LOC_Os03g48300	histidine acid phosphatase, putative, expressed
Chr.3	27498231	27498231	C	C	T	SNP	OS03G0689100	Intronic	NA	NA	LOC_Os03g48300	histidine acid phosphatase, putative, expressed
Chr.3	27500809	27500809	C	C	T	SNP	OS03G0689100	Intronic	NA	NA	LOC_Os03g48300	histidine acid phosphatase, putative, expressed
Chr.3	27738525	27738525	C	C	T	SNP	OS03G0693000	Intronic	NA	NA	LOC_Os03g48660	DUF1336 domain containing protein, expressed
Chr.3	28051536	28051536	C	C	T	SNP	OS03G0699700	Exonic	CDS	Silent	LOC_Os03g49260	lipoxygenase, putative, expressed
Chr.3	28252897	28252897	C	C	T	SNP	OS03G0703100	Intronic	NA	NA	LOC_Os03g49610	Os3bglu8 - beta-glucosidase, exo-beta-glucansase, high similarity to Os3bglu7, expressed
Chr.3	29016198	29016198	C	C	T	SNP	OS03G0717000	Exonic	3UTR	NA	LOC_Os03g50810	receptor protein kinase TMK1 precursor, putative, expressed
Chr.3	29017309	29017309	C	C	T	SNP	OS03G0717000	Intronic	NA	NA	LOC_Os03g50810	receptor protein kinase TMK1 precursor, putative, expressed
Chr.3	29138921	29138921	C	C	T	SNP	OS03G0719300	Intronic	NA	NA	LOC_Os03g51000	3,4-dihydroxy-2-butanone kinase, putative, expressed
Chr.3	29139922	29139922	C	C	T	SNP	OS03G0719300	Intronic	NA	NA	LOC_Os03g51000	3,4-dihydroxy-2-butanone kinase, putative, expressed
Chr.3	29386862	29386862	C	C	T	SNP	OS03G0723400	Intronic	NA	NA	LOC_Os03g51350	expressed protein
Chr.3	29387099	29387099	C	C	T	SNP	OS03G0723400	Intronic	NA	NA	LOC_Os03g51350	expressed protein
Chr.3	29387415	29387415	C	C	T	SNP	OS03G0723400	Intronic	NA	NA	LOC_Os03g51350	expressed protein
Chr.3	29387545	29387545	C	C	T	SNP	OS03G0723400	Intronic	NA	NA	LOC_Os03g51350	expressed protein
Chr.3	29455521	29455521	C	C	T	SNP	OS03G0724700	Exonic	CDS	Silent	LOC_Os03g51479	maf, putative, expressed
Chr.3	30063630	30063630	C	C	T	SNP	OS03G0733800	Exonic	CDS	Silent	LOC_Os03g52340	endoplasmic oxidoreductin-1 precursor, putative, expressed
Chr.3	30063442	30063442	C	C	T	SNP	OS03G0733800	Intronic	NA	NA	LOC_Os03g52340	endoplasmic oxidoreductin-1 precursor, putative, expressed
Chr.3	30075133	30075133	C	C	T	SNP	OS03G0734100	Intronic	NA	NA	LOC_Os03g52370	PIII4 - Proteinase inhibitor II family protein precursor, expressed
Chr.3	30075172	30075172	C	C	T	SNP	OS03G0734100	Intronic	NA	NA	LOC_Os03g52370	PIII4 - Proteinase inhibitor II family protein precursor, expressed
Chr.3	30318720	30318720	C	C	T	SNP	OS03G0738600	Exonic	CDS	Silent	LOC_Os03g52860	lipoxygenase, putative, expressed
Chr.3	32181973	32181973	C	C	T	SNP	OS03G0776000	Exonic	3UTR	NA	LOC_Os03g56460	glucose-6-phosphate isomerase, putative, expressed
Chr.3	32376968	32376968	C	C	T	SNP	OS03G0780800	Intronic	NA	NA	LOC_Os03g56820	fatty acid hydroxylase, putative, expressed
Chr.3	32624894	32624894	C	C	T	SNP	OS03G0786000	Intronic	NA	NA	LOC_Os03g57210	glycine-rich protein, putative, expressed
Chr.3	32933035	32933035	C	C	T	SNP	OS03G0791800	Intronic	NA	NA	LOC_Os03g57790	ubiquitin-conjugating enzyme, putative, expressed
Chr.3	33215692	33215692	C	C	T	SNP	OS03G0797400	Intronic	NA	NA	LOC_Os03g58300	indole-3-glycerol phosphate lyase, chloroplast precursor, putative, expressed
Chr.3	33216107	33216107	C	C	T	SNP	OS03G0797400	Intronic	NA	NA	LOC_Os03g58300	indole-3-glycerol phosphate lyase, chloroplast precursor, putative, expressed
Chr.3	33216622	33216622	C	C	T	SNP	OS03G0797400	Intronic	NA	NA	LOC_Os03g58300	indole-3-glycerol phosphate lyase, chloroplast precursor, putative, expressed
Chr.3	33216632	33216632	C	C	T	SNP	OS03G0797400	Intronic	NA	NA	LOC_Os03g58300	indole-3-glycerol phosphate lyase, chloroplast precursor, putative, expressed
Chr.3	33349390	33349390	C	C	T	SNP	OS03G0799900	Exonic	3UTR	NA	LOC_Os03g58570	WD domain, G-beta repeat domain containing protein, expressed
Chr.3	33347347	33347347	C	C	T	SNP	OS03G0799900	Intronic	NA	NA	LOC_Os03g58570	WD domain, G-beta repeat domain containing protein, expressed
Chr.3	35162193	35162193	C	C	T	SNP	OS03G0836800	Intronic	NA	NA	LOC_Os03g62060	hydrolase, putative, expressed

Chr.3	35661610	35661610	C	C	T	SNP	OS03G0848100	Exonic	3UTR	NA	LOC_Os03g63074	Ser/Thr protein phosphatase family protein, expressed
Chr.3	35662804	35662804	C	C	T	SNP	OS03G0848100	Exonic	3UTR	NA	LOC_Os03g63074	Ser/Thr protein phosphatase family protein, expressed
Chr.3	35791647	35791647	C	C	T	SNP	OS03G0850400	Intronic	NA	NA	LOC_Os03g63330	aspartokinase, chloroplast precursor, putative, expressed
Chr.3	35869430	35869430	C	C	T	SNP	OS03G0851900	Intronic	NA	NA	LOC_Os03g63500	ATPase, AFG1 family domain containing protein, expressed
Chr.3	35869478	35869478	C	C	T	SNP	OS03G0851900	Intronic	NA	NA	LOC_Os03g63500	ATPase, AFG1 family domain containing protein, expressed
Chr.3	1237971	1237971	A	A	T	SNP	OS03G0122300	Intronic	NA	NA	LOC_Os03g03034	flavonol synthase/flavanone 3-hydroxylase, putative, expressed
Chr.3	1238822	1238822	A	A	T	SNP	OS03G0122300	Intronic	NA	NA	LOC_Os03g03034	flavonol synthase/flavanone 3-hydroxylase, putative, expressed
Chr.3	3166248	3166248	A	A	T	SNP	OS03G0159100	Intronic	NA	NA	LOC_Os03g06330	tyrosine protein kinase domain containing protein, putative, expressed
Chr.3	3531343	3531343	A	A	T	SNP	OS03G0165400	Exonic	5UTR	NA	LOC_Os03g06940	beta-galactosidase, putative, expressed
Chr.3	15473418	15473418	A	A	T	SNP	OS03G0387900	Exonic	CDS	Nonsense	LOC_Os03g27019	expressed protein
Chr.3	15669573	15669573	A	A	T	SNP	OS03G0391100	Intronic	NA	NA	LOC_Os03g27360	RING-H2 finger protein ATL5H precursor, putative, expressed
Chr.3	15669699	15669699	A	A	T	SNP	OS03G0391100	Intronic	NA	NA	LOC_Os03g27360	RING-H2 finger protein ATL5H precursor, putative, expressed
Chr.3	15669733	15669733	A	A	T	SNP	OS03G0391100	Intronic	NA	NA	LOC_Os03g27360	RING-H2 finger protein ATL5H precursor, putative, expressed
Chr.3	16590643	16590643	A	A	T	SNP	OS03G0405500	Intronic	NA	NA	LOC_Os03g29190	PDI, putative, expressed
Chr.3	16591187	16591187	A	A	T	SNP	OS03G0405500	Intronic	NA	NA	LOC_Os03g29190	PDI, putative, expressed
Chr.3	21419158	21419158	A	A	T	SNP	OS03G0582200	Intronic	NA	NA	LOC_Os03g38590	secretory carrier-associated membrane protein 4, putative, expressed
Chr.3	23372209	23372209	A	A	T	SNP	OS03G0616700	Intronic	NA	NA	LOC_Os03g42050	exportin-7-A, putative, expressed
Chr.3	24471489	24471489	A	A	T	SNP	OS03G0638400	Exonic	5UTR	NA	LOC_Os03g43750	retrotransposon, putative, centromere-specific, expressed
Chr.3	24814577	24814577	A	A	T	SNP	OS03G0643300	Intronic	NA	NA	LOC_Os03g44150	aminotransferase, putative, expressed
Chr.3	24815071	24815071	A	A	T	SNP	OS03G0643300	Intronic	NA	NA	LOC_Os03g44150	aminotransferase, putative, expressed
Chr.3	24815473	24815473	A	A	T	SNP	OS03G0643300	Intronic	NA	NA	LOC_Os03g44150	aminotransferase, putative, expressed
Chr.3	24815530	24815530	A	A	T	SNP	OS03G0643300	Intronic	NA	NA	LOC_Os03g44150	aminotransferase, putative, expressed
Chr.3	24815587	24815587	A	A	T	SNP	OS03G0643300	Intronic	NA	NA	LOC_Os03g44150	aminotransferase, putative, expressed
Chr.3	24815930	24815930	A	A	T	SNP	OS03G0643300	Intronic	NA	NA	LOC_Os03g44150	aminotransferase, putative, expressed
Chr.3	24816276	24816276	A	A	T	SNP	OS03G0643300	Intronic	NA	NA	LOC_Os03g44150	aminotransferase, putative, expressed
Chr.3	26378949	26378949	A	A	T	SNP	OS03G0668900	Intronic	NA	NA	LOC_Os03g46600	acidic leucine-rich nuclear phosphoprotein 32-related protein 2, putative, expressed
Chr.3	29016199	29016199	A	A	T	SNP	OS03G0717000	Exonic	3UTR	NA	LOC_Os03g50810	receptor protein kinase TMK1 precursor, putative, expressed
Chr.3	29140541	29140541	A	A	T	SNP	OS03G0719300	Exonic	3UTR	NA	LOC_Os03g51000	3,4-dihydroxy-2-butanone kinase, putative, expressed
Chr.3	29141030	29141030	A	A	T	SNP	OS03G0719300	Intronic	NA	NA	LOC_Os03g51000	3,4-dihydroxy-2-butanone kinase, putative, expressed
Chr.3	32667543	32667543	A	A	T	SNP	OS03G0786700	Intronic	NA	NA	LOC_Os03g57280	N-dimethylguanosine tRNA methyltransferase, putative, expressed
Chr.3	33347119	33347119	A	A	T	SNP	OS03G0799900	Exonic	CDS	Missense	LOC_Os03g58570	WD domain, G-beta repeat domain containing protein, expressed
Chr.3	33549730	33549730	A	A	T	SNP	OS03G0803900	Exonic	CDS	Missense	LOC_Os03g58920	galactosyltransferase family protein, putative, expressed
Chr.3	34911228	34911228	A	A	T	SNP	OS03G0831100	Intronic	NA	NA	LOC_Os03g61560	expressed protein
Chr.3	35659888	35659888	A	A	T	SNP	OS03G0848100	Exonic	3UTR	NA	LOC_Os03g63074	Ser/Thr protein phosphatase family protein, expressed
Chr.3	540920	540920	G	G	T	SNP	OS03G0109300	Intronic	NA	NA	LOC_Os03g01880	possible lysine decarboxylase domain containing protein, expressed
Chr.3	1244354	1244354	G	G	T	SNP	OS03G0122300	Exonic	CDS	Missense	LOC_Os03g03034	flavonol synthase/flavanone 3-hydroxylase, putative, expressed
Chr.3	1238330	1238330	C	C	T	SNP	OS03G0122300	Intronic	NA	NA	LOC_Os03g03034	flavonol synthase/flavanone 3-hydroxylase, putative, expressed
Chr.3	1244137	1244137	A	A	T	SNP	OS03G0122300	Intronic	NA	NA	LOC_Os03g03034	flavonol synthase/flavanone 3-hydroxylase, putative, expressed
Chr.3	1737794	1737794	C	C	T	SNP	OS03G0130700	Exonic	CDS	NA	LOC_Os03g03860	expressed protein
Chr.3	2752554	2752554	C	C	T	SNP	OS03G0149300	Exonic	CDS	Missense	LOC_Os03g05530	nodulin, putative, expressed
Chr.3	3627132	3627132	C	C	T	SNP	OS03G0167000	Intronic	NA	NA	LOC_Os03g07100	LTPL82 - Protease inhibitor/seed storage/LTP family protein precursor, expressed
Chr.3	4387011	4387011	C	C	T	SNP	OS03G0183600	Intronic	NA	NA	LOC_Os03g08530	aminotransferase, classes I and II, domain containing protein, expressed
Chr.3	4387023	4387023	G	G	T	SNP	OS03G0183600	Intronic	NA	NA	LOC_Os03g08530	aminotransferase, classes I and II, domain containing protein, expressed
Chr.3	4387047	4387047	C	C	T	SNP	OS03G0183600	Intronic	NA	NA	LOC_Os03g08530	aminotransferase, classes I and II, domain containing protein, expressed
Chr.3	4387182	4387182	C	C	T	SNP	OS03G0183600	Intronic	NA	NA	LOC_Os03g08530	aminotransferase, classes I and II, domain containing protein, expressed
Chr.3	4387418	4387418	C	C	T	SNP	OS03G0183600	Intronic	NA	NA	LOC_Os03g08530	aminotransferase, classes I and II, domain containing protein, expressed
Chr.3	5114031	5114031	C	C	T	SNP	OS03G0197200	Exonic	3UTR	NA	LOC_Os03g10100	transporter family protein, putative, expressed
Chr.3	6371476	6371476	A	A	T	SNP	OS03G0221500	Intronic	NA	NA	LOC_Os03g12140	glucan endo-1,3-beta-glucosidase precursor, putative, expressed
Chr.3	7441372	7441372	C	C	T	SNP	OS03G0240600	Exonic	CDS	Silent	LOC_Os03g13740	immediate-early fungal elicitor protein CMPG1, putative, expressed
Chr.3	8707003	8707003	C	C	T	SNP	OS03G0264400	Exonic	CDS	Missense	LOC_Os03g15780	anthranilate synthase component I-1, chloroplast precursor, putative, expressed
Chr.3	8706796	8706796	A	A	T	SNP	OS03G0264400	Intronic	NA	NA	LOC_Os03g15780	anthranilate synthase component I-1, chloroplast precursor, putative, expressed
Chr.3	9013353	9013353	C	C	T	SNP	OS03G0269900	Intronic	NA	NA	LOC_Os03g16334	fringe-related protein, putative, expressed
Chr.3	9014243	9014243	G	G	T	SNP	OS03G0269900	Intronic	NA	NA	LOC_Os03g16334	fringe-related protein, putative, expressed
Chr.3	10599297	10599297	G	G	T	SNP	OS03G0301200	Exonic	CDS	Silent	LOC_Os03g18910	COBRA-like protein 7 precursor, putative, expressed
Chr.3	10623343	10623343	C	C	T	SNP	OS03G0301700	Intronic	NA	NA	LOC_Os03g18970	protein phosphatase protein, putative, expressed
Chr.3	10624892	10624892	C	C	T	SNP	OS03G0301700	Intronic	NA	NA	LOC_Os03g18970	protein phosphatase protein, putative, expressed
Chr.3	10772998	10772998	C	C	T	SNP	OS03G0304500	Exonic	CDS	Missense	LOC_Os03g19200	DNAJ heat shock N-terminal domain-containing protein, putative, expressed
Chr.3	10774856	10774856	C	C	T	SNP	OS03G0304500	Intronic	NA	NA	LOC_Os03g19200	DNAJ heat shock N-terminal domain-containing protein, putative, expressed
Chr.3	12589574	12589574	C	C	T	SNP	OS03G0338400	Exonic	3UTR	NA	LOC_Os03g21970	basic helix-loop-helix domain containing protein, expressed
Chr.3	15668371	15668371	C	C	T	SNP	OS03G0391100	Exonic	CDS	Silent	LOC_Os03g27360	RING-H2 finger protein ATL5H precursor, putative, expressed
Chr.3	15668890	15668890	C	C	T	SNP	OS03G0391100	Exonic	CDS	Silent	LOC_Os03g27360	RING-H2 finger protein ATL5H precursor, putative, expressed
Chr.3	15669166	15669166	G	G	T	SNP	OS03G0391100	Intronic	NA	NA	LOC_Os03g27360	RING-H2 finger protein ATL5H precursor, putative, expressed
Chr.3	15669304	15669304	G	G	T	SNP	OS03G0391100	Intronic	NA	NA	LOC_Os03g27360	RING-H2 finger protein ATL5H precursor, putative, expressed
Chr.3	15669746	15669746	C	C	T	SNP	OS03G0391100	Intronic	NA	NA	LOC_Os03g27360	RING-H2 finger protein ATL5H precursor, putative, expressed
Chr.3	16285485	16285485	C	C	T	SNP	OS03G0401100	Intronic	NA	NA	LOC_Os03g28300	protein kinase domain containing protein, expressed
Chr.3	16592503	16592503	C	C	T	SNP	OS03G0405500	Intronic	NA	NA	LOC_Os03g29190	PDI, putative, expressed
Chr.3	17291479	17291479	C	C	T	SNP	OS03G0416500	Exonic	5UTR	NA	LOC_Os03g30300	6-phosphogluconolactonase, putative, expressed
Chr.3	17449620	17449620	G	G	T	SNP	OS03G0419100	Exonic	5UTR	NA	LOC_Os03g30570	protein of unknown function DUF1675 domain containing protein, expressed
Chr.3	18577356	18577356	C	C	T	SNP	OS03G0439700	Exonic	CDS	Silent	LOC_Os03g32490	DUF1230 domain containing protein, expressed
Chr.3	21105925	21105925	C	C	T	SNP	OS03G0577100	Exonic	5UTR	NA	LOC_Os03g38010	nuf2 family protein, expressed
Chr.3	23486274	23486274	C	C	T	SNP	OS03G0619400	Intronic	NA	NA	LOC_Os03g42220	T-complex protein, putative, expressed
Chr.3	24471775	24471775	C	C	T	SNP	OS03G0638400	Exonic	5UTR	NA	LOC_Os03g43750	retrotransposon, putative, centromere-specific, expressed
Chr.3	24469561	24469561	C	C	T	SNP	OS03G0638400	Intronic	NA	NA	LOC_Os03g43750	retrotransposon, putative, centromere-specific, expressed
Chr.3	24469887	24469887	C	C	T	SNP	OS03G0638400	Intronic	NA	NA	LOC_Os03g43750	retrotransposon, putative, centromere-specific, expressed
Chr.3	24470659	24470659	C	C	T	SNP	OS03G0638400	Intronic	NA	NA	LOC_Os03g43750	retrotransposon, putative, centromere-specific, expressed
Chr.3	24471005	24471005	C	C	T	SNP	OS03G0638400	Intronic	NA	NA	LOC_Os03g43750	retrotransposon, putative, centromere-specific, expressed
Chr.3	24705214	24705214	C	C	T	SNP	OS03G0641300	Exonic	3UTR	NA	LOC_Os03g43990	BTBN8 - Bric-a-Brac, Tramtrack, Broad Complex BTB domain with non-phototropic hypocotyl 3 NPH3 and coiled-coil domains, ex
Chr.3	24816610	24816610	C	C	T	SNP	OS03G0643300	Exonic	CDS	Silent	LOC_Os03g44150	aminotransferase, putative, expressed
Chr.3	24814693	24814693	G	G	T	SNP	OS03G0643300	Intronic	NA	NA	LOC_Os03g44150	aminotransferase, putative, expressed
Chr.3	24814745	24814745	C	C	T	SNP	OS03G0643300	Intronic	NA	NA	LOC_Os03g44150	aminotransferase, putative, expressed
Chr.3	24814789	24814789	C	C	T	SNP	OS03G0643300	Intronic	NA	NA	LOC_Os03g44150	aminotransferase, putative, expressed
Chr.3	24814795	24814795	C	C	T	SNP	OS03G0643300	Intronic	NA	NA	LOC_Os03g44150	aminotransferase, putative, expressed
Chr.3	24815377	24815377	A	A	T	SNP	OS03G0643300	Intronic	NA	NA	LOC_Os03g44150	aminotransferase, putative, expressed
Chr.3	26385127	26385127	C	C	T	SNP	OS03G0669000	Exonic	CDS	Silent	LOC_Os03g46610	DEAD-box ATP-dependent RNA helicase, putative, expressed
Chr.3	26385269	26385269	G	G	T	SNP	OS03G0669000	Exonic	CDS	Missense	LOC_Os03g46610	DEAD-box ATP-dependent RNA helicase, putative, expressed

Chr.3	27336580	27336580	C	C	T	SNP	OS03G0685300	Intronic	NA	NA	LOC_Os03g48060	class I glutamine amidotransferase, putative, expressed
Chr.3	27493191	27493191	C	C	T	SNP	OS03G0689100	Intronic	NA	NA	LOC_Os03g48300	histidine acid phosphatase, putative, expressed
Chr.3	28243705	28243705	C	C	T	SNP	OS03G0703000	Intronic	NA	NA	LOC_Os03g49600	Os3bglu7 - beta-glucosidase, exo-beta-glucanse, expressed
Chr.3	28595471	28595471	G	G	T	SNP	OS03G0709000	Intronic	NA	NA	LOC_Os03g50130	microsomal glutathione S-transferase 3, putative, expressed
Chr.3	30075207	30075207	G	G	T	SNP	OS03G0734100	Intronic	NA	NA	LOC_Os03g52370	PIII4 - Proteinase inhibitor II family protein precursor, expressed
Chr.3	30204430	30204430	G	G	T	SNP	OS03G0736900	Exonic	CDS	NA	LOC_Os03g52680	expressed protein
Chr.3	30317556	30317556	C	C	T	SNP	OS03G0738600	Exonic	CDS	Silent	LOC_Os03g52860	lipoxygenase, putative, expressed
Chr.3	32008807	32008807	C	C	T	SNP	OS03G0772600	Exonic	CDS	NA	LOC_Os03g56160	lectin-like receptor kinase 7, putative, expressed
Chr.3	32695975	32695975	C	C	T	SNP	OS03G0787000	Exonic	3UTR	NA	LOC_Os03g57310	syntaxin, putative, expressed
Chr.3	32711162	32711162	C	C	T	SNP	OS03G0787300	Exonic	3UTR	NA	LOC_Os03g57340	chaperone protein dnaJ, putative, expressed
Chr.3	32933382	32933382	C	C	T	SNP	OS03G0791800	Intronic	NA	NA	LOC_Os03g57790	ubiquitin-conjugating enzyme, putative, expressed
Chr.3	33215866	33215866	C	C	T	SNP	OS03G0797400	Intronic	NA	NA	LOC_Os03g58300	indole-3-glycerol phosphate lyase, chloroplast precursor, putative, expressed
Chr.3	33349922	33349922	C	C	T	SNP	OS03G0799900	Exonic	3UTR	NA	LOC_Os03g58570	WD domain, G-beta repeat domain containing protein, expressed
Chr.3	33346844	33346844	C	C	T	SNP	OS03G0799900	Exonic	CDS	Missense	LOC_Os03g58570	WD domain, G-beta repeat domain containing protein, expressed
Chr.3	35161671	35161671	C	C	T	SNP	OS03G0836800	Intronic	NA	NA	LOC_Os03g62060	hydrolase, putative, expressed
Chr.3	35161783	35161783	C	C	T	SNP	OS03G0836800	Intronic	NA	NA	LOC_Os03g62060	hydrolase, putative, expressed
Chr.3	35161917	35161917	C	C	T	SNP	OS03G0836800	Intronic	NA	NA	LOC_Os03g62060	hydrolase, putative, expressed
Chr.3	35161928	35161928	C	C	T	SNP	OS03G0836800	Intronic	NA	NA	LOC_Os03g62060	hydrolase, putative, expressed
Chr.3	35161979	35161979	A	A	T	SNP	OS03G0836800	Intronic	NA	NA	LOC_Os03g62060	hydrolase, putative, expressed
Chr.3	35162312	35162312	C	C	T	SNP	OS03G0836800	Intronic	NA	NA	LOC_Os03g62060	hydrolase, putative, expressed
Chr.3	35332111	35332111	C	C	T	SNP	OS03G0840200	Exonic	5UTR	NA	LOC_Os03g62370	WD40-like Beta Propeller Repeat family protein, expressed
Chr.3	35381142	35381142	C	C	T	SNP	OS03G0841600	Exonic	CDS	NA	LOC_Os03g62480	anthocyanidin 5,3-O-glucosyltransferase, putative, expressed
Chr.3	35663804	35663804	C	C	T	SNP	OS03G0848100	Exonic	CDS	NA	LOC_Os03g63074	Ser/Thr protein phosphatase family protein, expressed
Chr.3	1243892	1243894	TTC	TTC	T	INDEL	OS03G0122300	Intronic	NA	NA	LOC_Os03g03034	flavonol synthase/flavanone 3-hydroxylase, putative, expressed
Chr.3	2750366	2750367	TC	TC	T	INDEL	OS03G0149300	Intronic	NA	NA	LOC_Os03g05530	nodulin, putative, expressed
Chr.3	4387056	4387057	TC	TC	T	INDEL	OS03G0183600	Intronic	NA	NA	LOC_Os03g08530	aminotransferase, classes I and II, domain containing protein, expressed
Chr.3	6369548	6369569	TTGGTCTGCTCTTCTTGGTATC	TTGGTCTGCTCTTCTTGGTATC	T	INDEL	OS03G0221500	Exonic	5UTR	NA	LOC_Os03g12140	glucan endo-1,3-beta-glucosidase precursor, putative, expressed
Chr.3	6369571	6369573	TTC	TTC	T	INDEL	OS03G0221500	Exonic	5UTR	NA	LOC_Os03g12140	glucan endo-1,3-beta-glucosidase precursor, putative, expressed
Chr.3	9845397	9845398	TA	TA	T	INDEL	OS03G0285700	Intronic	NA	NA	LOC_Os03g17690	OsAPx1 - Cytosolic Ascorbate Peroxidase encoding gene 1-8, expressed
Chr.3	10120949	10120955	TTAGTAC	TTAGTAC	T	INDEL	OS03G0291500	Intronic	NA	NA	LOC_Os03g18130	asparagine synthetase, putative, expressed
Chr.3	15669768	15669769	TC	TC	T	INDEL	OS03G0391100	Intronic	NA	NA	LOC_Os03g27360	RING-H2 finger protein ATL5H precursor, putative, expressed
Chr.3	17290566	17290578	TCGGAAACATCAA	TCGGAAACATCAA	T	INDEL	OS03G0416500	Exonic	5UTR	NA	LOC_Os03g30300	6-phosphogluconolactonase, putative, expressed
Chr.3	20795164	20795165	TG	TG	T	INDEL	OS03G0571900	Intronic	NA	NA	LOC_Os03g37490	MATE efflux family protein, putative, expressed
Chr.3	24817162	24817166	TATTA	TATTA	T	INDEL	OS03G0643300	Intronic	NA	NA	LOC_Os03g44150	aminotransferase, putative, expressed
Chr.3	25946547	25946548	TA	TA	T	INDEL	OS03G0661300	Exonic	3UTR	NA	LOC_Os03g45920	tubulin/FtsZ domain containing protein, putative, expressed
Chr.3	26383178	26383179	TA	TA	T	INDEL	OS03G0669000	Exonic	3UTR	NA	LOC_Os03g46610	DEAD-box ATP-dependent RNA helicase, putative, expressed
Chr.3	27490026	27490027	TG	TG	T	INDEL	OS03G0689100	Intronic	NA	NA	LOC_Os03g48300	histidine acid phosphatase, putative, expressed
Chr.3	28243807	28243808	TC	TC	T	INDEL	OS03G0703000	Intronic	NA	NA	LOC_Os03g49600	Os3bglu7 - beta-glucosidase, exo-beta-glucanse, expressed
Chr.3	29451513	29451519	TTGGTAC	TTGGTAC	T	INDEL	OS03G0724600	Exonic	3UTR	NA	LOC_Os03g51470	expressed protein
Chr.3	30075161	30075163	TTA	TTA	T	INDEL	OS03G0734100	Intronic	NA	NA	LOC_Os03g52370	PIII4 - Proteinase inhibitor II family protein precursor, expressed
Chr.3	32624942	32624944	TGA	TGA	T	INDEL	OS03G0786000	Intronic	NA	NA	LOC_Os03g57210	glycine-rich protein, putative, expressed
Chr.3	33215725	33215726	TG	TG	T	INDEL	OS03G0797400	Intronic	NA	NA	LOC_Os03g58300	indole-3-glycerol phosphate lyase, chloroplast precursor, putative, expressed
Chr.3	33216666	33216667	TA	TA	T	INDEL	OS03G0797400	Intronic	NA	NA	LOC_Os03g58300	indole-3-glycerol phosphate lyase, chloroplast precursor, putative, expressed
Chr.3	34193541	34193542	TG	TG	T	INDEL	OS03G0815800	Exonic	5UTR	NA	LOC_Os03g60120	AP2 domain containing protein, expressed
Chr.3	35161679	35161681	TAA	TAA	T	INDEL	OS03G0836800	Intronic	NA	NA	LOC_Os03g62060	hydrolase, putative, expressed
Chr.2	3039404	3039404	G	G	T	SNP	OS02G0155400	Exonic	CDS	Silent	LOC_Os02g06090	phytosulfokine receptor precursor, putative, expressed
Chr.2	3037724	3037724	G	G	T	SNP	OS02G0155400	Exonic	CDS	Missense	LOC_Os02g06090	phytosulfokine receptor precursor, putative, expressed
Chr.2	3038309	3038309	G	G	T	SNP	OS02G0155400	Exonic	CDS	Missense	LOC_Os02g06090	phytosulfokine receptor precursor, putative, expressed
Chr.2	21930840	21930840	G	G	T	SNP	OS02G0572400	Exonic	3UTR	NA	LOC_Os02g36340	riboflavin biosynthesis protein ribAB, chloroplast precursor, putative, expressed
Chr.2	768771	768771	C	C	T	SNP	OS02G0114200	Intronic	NA	NA	LOC_Os02g02320	OsSCP6 - Putative Serine Carboxypeptidase homologue, expressed
Chr.2	768897	768897	C	C	T	SNP	OS02G0114200	Intronic	NA	NA	LOC_Os02g02320	OsSCP6 - Putative Serine Carboxypeptidase homologue, expressed
Chr.2	3037922	3037922	C	C	T	SNP	OS02G0155400	Exonic	CDS	Silent	LOC_Os02g06090	phytosulfokine receptor precursor, putative, expressed
Chr.2	3038240	3038240	C	C	T	SNP	OS02G0155400	Exonic	CDS	Silent	LOC_Os02g06090	phytosulfokine receptor precursor, putative, expressed
Chr.2	3038306	3038306	C	C	T	SNP	OS02G0155400	Exonic	CDS	Silent	LOC_Os02g06090	phytosulfokine receptor precursor, putative, expressed
Chr.2	3038516	3038516	C	C	T	SNP	OS02G0155400	Exonic	CDS	Silent	LOC_Os02g06090	phytosulfokine receptor precursor, putative, expressed
Chr.2	3038891	3038891	C	C	T	SNP	OS02G0155400	Exonic	CDS	Silent	LOC_Os02g06090	phytosulfokine receptor precursor, putative, expressed
Chr.2	3037744	3037744	C	C	T	SNP	OS02G0155400	Exonic	CDS	Missense	LOC_Os02g06090	phytosulfokine receptor precursor, putative, expressed
Chr.2	3039468	3039468	C	C	T	SNP	OS02G0155400	Exonic	CDS	Missense	LOC_Os02g06090	phytosulfokine receptor precursor, putative, expressed
Chr.2	3039742	3039742	C	C	T	SNP	OS02G0155400	Exonic	CDS	Missense	LOC_Os02g06090	phytosulfokine receptor precursor, putative, expressed
Chr.2	4390234	4390234	C	C	T	SNP	OS02G0179200	Exonic	3UTR	NA	LOC_Os02g08270	class I glutamine amidotransferase, putative, expressed
Chr.2	4390533	4390533	C	C	T	SNP	OS02G0179200	Exonic	CDS	Missense	LOC_Os02g08270	class I glutamine amidotransferase, putative, expressed
Chr.2	8977390	8977390	C	C	T	SNP	OS02G0258900	Exonic	3UTR	NA	LOC_Os02g15870	molybdenum cofactor biosynthesis protein 1, putative, expressed
Chr.2	17160895	17160895	C	C	T	SNP	OS02G0491400	Exonic	5UTR	NA	LOC_Os02g28980	peptidyl-prolyl isomerase, putative, expressed
Chr.2	21931667	21931667	C	C	T	SNP	OS02G0572400	Intronic	NA	NA	LOC_Os02g36340	riboflavin biosynthesis protein ribAB, chloroplast precursor, putative, expressed
Chr.2	22510304	22510304	C	C	T	SNP	OS02G0583700	Intronic	NA	NA	LOC_Os02g37250	hypothetical protein
Chr.2	22510378	22510378	C	C	T	SNP	OS02G0583700	Intronic	NA	NA	LOC_Os02g37250	hypothetical protein
Chr.2	22689566	22689566	C	C	T	SNP	OS02G0588500	Exonic	CDS	Silent	LOC_Os02g37590	glycerophosphoryl diester phosphodiesterase family protein, putative, expressed
Chr.2	767964	767964	A	A	T	SNP	OS02G0114200	Intronic	NA	NA	LOC_Os02g02320	OsSCP6 - Putative Serine Carboxypeptidase homologue, expressed
Chr.2	768814	768814	A	A	T	SNP	OS02G0114200	Intronic	NA	NA	LOC_Os02g02320	OsSCP6 - Putative Serine Carboxypeptidase homologue, expressed
Chr.2	2747294	2747294	A	A	T	SNP	OS02G0149800	Exonic	3UTR	NA	LOC_Os02g05630	protein phosphatase 2C, putative, expressed
Chr.2	3038658	3038658	A	A	T	SNP	OS02G0155400	Exonic	CDS	Missense	LOC_Os02g06090	phytosulfokine receptor precursor, putative, expressed
Chr.2	3039109	3039109	A	A	T	SNP	OS02G0155400	Exonic	CDS	Missense	LOC_Os02g06090	phytosulfokine receptor precursor, putative, expressed
Chr.2	4390171	4390171	A	A	T	SNP	OS02G0179200	Exonic	3UTR	NA	LOC_Os02g08270	class I glutamine amidotransferase, putative, expressed
Chr.2	8583593	8583593	A	A	T	SNP	OS02G0252200	Intronic	NA	NA	LOC_Os02g15340	no apical meristem protein, putative, expressed
Chr.2	18580358	18580358	A	A	T	SNP	OS02G0515200	Intronic	NA	NA	LOC_Os02g31080	hydroxyproline-rich glycoprotein family protein, putative, expressed
Chr.2	34224073	34224073	A	A	T	SNP	OS02G0802700	Intronic	NA	NA	LOC_Os02g55910	monogalactosyldiacylglycerol synthase, putative, expressed
Chr.2	34224074	34224074	A	A	T	SNP	OS02G0802700	Intronic	NA	NA	LOC_Os02g55910	monogalactosyldiacylglycerol synthase, putative, expressed
Chr.2	34224075	34224075	A	A	T	SNP	OS02G0802700	Intronic	NA	NA	LOC_Os02g55910	monogalactosyldiacylglycerol synthase, putative, expressed
Chr.2	81410	81410	G	G	T	SNP	OS02G0101500	Intronic	NA	NA	LOC_Os02g01150	erythronate-4-phosphate dehydrogenase domain containing protein, expressed
Chr.2	4294649	4294649	G	G	T	SNP	OS02G0177800	Exonic	CDS	Missense	LOC_Os02g08120	calmodulin binding protein, putative, expressed
Chr.2	6813897	6813897	G	G	T	SNP	OS02G0222100	Exonic	3UTR	NA	LOC_Os02g12900	cysteine synthase, putative, expressed
Chr.2	6812197	6812197	G	G	T	SNP	OS02G0222100	Intronic	NA	NA	LOC_Os02g12900	cysteine synthase, putative, expressed
Chr.2	6835269	6835269	G	G	T	SNP	OS02G0222500	Intronic	NA	NA	LOC_Os02g12939	expressed protein
Chr.2	6835321	6835321	G	G	T	SNP	OS02G0222500	Intronic	NA	NA	LOC_Os02g12939	expressed protein
Chr.2	7221930	7221930	G	G	T	SNP	OS02G0228300	Exonic	CDS	Silent	LOC_Os02g13510	receptor-like protein kinase 5 precursor, putative, expressed

Chr.2	9973367	9973367	G	G	T	SNP	OS02G0273700	Intronic	NA	NA	LOC_Os02g17350	VHS and GAT domain containing protein, expressed
Chr.2	9973608	9973608	G	G	T	SNP	OS02G0273700	Intronic	NA	NA	LOC_Os02g17350	VHS and GAT domain containing protein, expressed
Chr.2	9973612	9973612	G	G	T	SNP	OS02G0273700	Intronic	NA	NA	LOC_Os02g17350	VHS and GAT domain containing protein, expressed
Chr.2	9974037	9974037	G	G	T	SNP	OS02G0273700	Intronic	NA	NA	LOC_Os02g17350	VHS and GAT domain containing protein, expressed
Chr.2	9974070	9974070	G	G	T	SNP	OS02G0273700	Intronic	NA	NA	LOC_Os02g17350	VHS and GAT domain containing protein, expressed
Chr.2	9975808	9975808	G	G	T	SNP	OS02G0273700	Intronic	NA	NA	LOC_Os02g17350	VHS and GAT domain containing protein, expressed
Chr.2	9976009	9976009	G	G	T	SNP	OS02G0273700	Intronic	NA	NA	LOC_Os02g17350	VHS and GAT domain containing protein, expressed
Chr.2	9989296	9989296	G	G	T	SNP	OS02G0273900	Intronic	NA	NA	LOC_Os02g17370	expressed protein
Chr.2	28804768	28804768	G	G	T	SNP	OS02G0700100	Intronic	NA	NA	LOC_Os02g47180	WD repeat-containing protein, putative, expressed
Chr.2	34029066	34029066	G	G	T	SNP	OS02G0799000	Intronic	NA	NA	LOC_Os02g55560	protein phosphatase 2C, putative, expressed
Chr.2	34044390	34044390	G	G	T	SNP	OS02G0799300	Intronic	NA	NA	LOC_Os02g55590	expressed protein
Chr.2	34044666	34044666	G	G	T	SNP	OS02G0799300	Intronic	NA	NA	LOC_Os02g55590	expressed protein
Chr.2	34613134	34613134	G	G	T	SNP	OS02G0809800	Exonic	CDS	Silent	LOC_Os02g56510	phosphate transporter 1, putative, expressed
Chr.2	35081772	35081772	G	G	T	SNP	OS02G0817700	Intronic	NA	NA	LOC_Os02g57260	3-ketoacyl-CoA thiolase, peroxisomal precursor, putative, expressed
Chr.2	35092097	35092097	G	G	T	SNP	OS02G0817900	Exonic	3UTR	NA	LOC_Os02g57290	cytochrome P450, putative, expressed
Chr.2	35093037	35093037	G	G	T	SNP	OS02G0817900	Exonic	3UTR	NA	LOC_Os02g57290	cytochrome P450, putative, expressed
Chr.2	35095970	35095970	G	G	T	SNP	OS02G0817900	Intronic	NA	NA	LOC_Os02g57290	cytochrome P450, putative, expressed
Chr.2	35096020	35096020	G	G	T	SNP	OS02G0817900	Intronic	NA	NA	LOC_Os02g57290	cytochrome P450, putative, expressed
Chr.2	35096234	35096234	G	G	T	SNP	OS02G0817900	Intronic	NA	NA	LOC_Os02g57290	cytochrome P450, putative, expressed
Chr.2	80330	80330	C	C	T	SNP	OS02G0101500	Intronic	NA	NA	LOC_Os02g01150	erythronate-4-phosphate dehydrogenase domain containing protein, expressed
Chr.2	317383	317383	C	C	T	SNP	OS02G0105900	Exonic	CDS	Silent	LOC_Os02g01560	40S ribosomal protein S4, putative, expressed
Chr.2	1662532	1662532	C	C	T	SNP	OS02G0131800	Exonic	CDS	Missense	LOC_Os02g03900	metal transporter Nramp6, putative, expressed
Chr.2	4295635	4295635	C	C	T	SNP	OS02G0177800	Intronic	NA	NA	LOC_Os02g08120	calmodulin binding protein, putative, expressed
Chr.2	5155599	5155599	C	C	T	SNP	OS02G0192500	Intronic	NA	NA	LOC_Os02g09930	CSLA1 - cellulose synthase-like family A; mannan synthase, expressed
Chr.2	5157564	5157564	C	C	T	SNP	OS02G0192500	Intronic	NA	NA	LOC_Os02g09930	CSLA1 - cellulose synthase-like family A; mannan synthase, expressed
Chr.2	5707805	5707805	C	C	T	SNP	OS02G0202200	Intronic	NA	NA	LOC_Os02g10780	SPX domain containing protein, putative, expressed
Chr.2	5760238	5760238	C	C	T	SNP	OS02G0202950	Exonic	CDS	NA	LOC_Os02g10860	bZIP transcription factor domain containing protein, expressed
Chr.2	5760342	5760342	C	C	T	SNP	OS02G0202950	Exonic	CDS	NA	LOC_Os02g10860	bZIP transcription factor domain containing protein, expressed
Chr.2	6071970	6071970	C	C	T	SNP	OS02G0208300	Exonic	CDS	NA	LOC_Os02g11760	pleiotropic drug resistance protein, putative, expressed
Chr.2	6079027	6079027	C	C	T	SNP	OS02G0208500	Exonic	5UTR	NA	LOC_Os02g11770	expressed protein
Chr.2	6719766	6719766	C	C	T	SNP	OS02G0220500	Exonic	CDS	Silent	LOC_Os02g12794	elongation factor 1-gamma, putative, expressed
Chr.2	6834560	6834560	C	C	T	SNP	OS02G0222500	Exonic	CDS	NA	LOC_Os02g12939	expressed protein
Chr.2	6834580	6834580	C	C	T	SNP	OS02G0222500	Exonic	CDS	NA	LOC_Os02g12939	expressed protein
Chr.2	6834596	6834596	C	C	T	SNP	OS02G0222500	Exonic	CDS	NA	LOC_Os02g12939	expressed protein
Chr.2	6834892	6834892	C	C	T	SNP	OS02G0222500	Intronic	NA	NA	LOC_Os02g12939	expressed protein
Chr.2	7220652	7220652	C	C	T	SNP	OS02G0228300	Exonic	3UTR	NA	LOC_Os02g13510	receptor-like protein kinase 5 precursor, putative, expressed
Chr.2	7221860	7221860	C	C	T	SNP	OS02G0228300	Exonic	CDS	Missense	LOC_Os02g13510	receptor-like protein kinase 5 precursor, putative, expressed
Chr.2	7224332	7224332	C	C	T	SNP	OS02G0228300	Exonic	CDS	Missense	LOC_Os02g13510	receptor-like protein kinase 5 precursor, putative, expressed
Chr.2	9504201	9504201	C	C	T	SNP	OS02G0266000	Intronic	NA	NA	LOC_Os02g16630	tryptophan biosynthesis protein trpCF, putative, expressed
Chr.2	9651183	9651183	C	C	T	SNP	OS02G0269600	Exonic	CDS	Silent	LOC_Os02g16940	OsSub13 - Putative Subtilisin homologue, expressed
Chr.2	9650920	9650920	C	C	T	SNP	OS02G0269600	Exonic	CDS	Missense	LOC_Os02g16940	OsSub13 - Putative Subtilisin homologue, expressed
Chr.2	9651292	9651292	C	C	T	SNP	OS02G0269600	Exonic	CDS	Missense	LOC_Os02g16940	OsSub13 - Putative Subtilisin homologue, expressed
Chr.2	9925282	9925282	C	C	T	SNP	OS02G0272900	Intronic	NA	NA	LOC_Os02g17304	resistance protein, putative, expressed
Chr.2	9925314	9925314	C	C	T	SNP	OS02G0272900	Intronic	NA	NA	LOC_Os02g17304	resistance protein, putative, expressed
Chr.2	9926427	9926427	C	C	T	SNP	OS02G0272900	Intronic	NA	NA	LOC_Os02g17304	resistance protein, putative, expressed
Chr.2	9977774	9977774	C	C	T	SNP	OS02G0273700	Exonic	3UTR	NA	LOC_Os02g17350	VHS and GAT domain containing protein, expressed
Chr.2	9972829	9972829	C	C	T	SNP	OS02G0273700	Exonic	5UTR	NA	LOC_Os02g17350	VHS and GAT domain containing protein, expressed
Chr.2	9973567	9973567	C	C	T	SNP	OS02G0273700	Intronic	NA	NA	LOC_Os02g17350	VHS and GAT domain containing protein, expressed
Chr.2	9973815	9973815	C	C	T	SNP	OS02G0273700	Intronic	NA	NA	LOC_Os02g17350	VHS and GAT domain containing protein, expressed
Chr.2	9974166	9974166	C	C	T	SNP	OS02G0273700	Intronic	NA	NA	LOC_Os02g17350	VHS and GAT domain containing protein, expressed
Chr.2	9974202	9974202	C	C	T	SNP	OS02G0273700	Intronic	NA	NA	LOC_Os02g17350	VHS and GAT domain containing protein, expressed
Chr.2	9974485	9974485	C	C	T	SNP	OS02G0273700	Intronic	NA	NA	LOC_Os02g17350	VHS and GAT domain containing protein, expressed
Chr.2	9974890	9974890	C	C	T	SNP	OS02G0273700	Intronic	NA	NA	LOC_Os02g17350	VHS and GAT domain containing protein, expressed
Chr.2	9975503	9975503	C	C	T	SNP	OS02G0273700	Intronic	NA	NA	LOC_Os02g17350	VHS and GAT domain containing protein, expressed
Chr.2	9989003	9989003	C	C	T	SNP	OS02G0273900	Exonic	3UTR	NA	LOC_Os02g17370	expressed protein
Chr.2	9989490	9989490	C	C	T	SNP	OS02G0273900	Intronic	NA	NA	LOC_Os02g17370	expressed protein
Chr.2	9989561	9989561	C	C	T	SNP	OS02G0273900	Intronic	NA	NA	LOC_Os02g17370	expressed protein
Chr.2	9989654	9989654	C	C	T	SNP	OS02G0273900	Intronic	NA	NA	LOC_Os02g17370	expressed protein
Chr.2	9989715	9989715	C	C	T	SNP	OS02G0273900	Intronic	NA	NA	LOC_Os02g17370	expressed protein
Chr.2	9989859	9989859	C	C	T	SNP	OS02G0273900	Intronic	NA	NA	LOC_Os02g17370	expressed protein
Chr.2	9989883	9989883	C	C	T	SNP	OS02G0273900	Intronic	NA	NA	LOC_Os02g17370	expressed protein
Chr.2	9989988	9989988	C	C	T	SNP	OS02G0273900	Intronic	NA	NA	LOC_Os02g17370	expressed protein
Chr.2	9990236	9990236	C	C	T	SNP	OS02G0273900	Intronic	NA	NA	LOC_Os02g17370	expressed protein
Chr.2	11487169	11487169	C	C	T	SNP	OS02G0299300	Intronic	NA	NA	LOC_Os02g19650	hydrolase, alpha/beta fold family domain containing protein, expressed
Chr.2	16009860	16009860	C	C	T	SNP	OS02G0471500	Exonic	3UTR	NA	LOC_Os02g27220	protein phosphatase 2C, putative, expressed
Chr.2	16010114	16010114	C	C	T	SNP	OS02G0471500	Exonic	CDS	Silent	LOC_Os02g27220	protein phosphatase 2C, putative, expressed
Chr.2	17534233	17534233	C	C	T	SNP	OS02G0498300	Intronic	NA	NA	LOC_Os02g29510	DUF803 domain containing, putative, expressed
Chr.2	17538710	17538710	C	C	T	SNP	OS02G0498300	Intronic	NA	NA	LOC_Os02g29510	DUF803 domain containing, putative, expressed
Chr.2	19104643	19104643	C	C	T	SNP	OS02G0523500	Intronic	NA	NA	LOC_Os02g32350	TUDOR protein with multiple SNc domains, putative, expressed
Chr.2	21843686	21843686	C	C	T	SNP	OS02G0570900	Exonic	CDS	Silent	LOC_Os02g36210	ent-kaurene synthase, chloroplast precursor, putative, expressed
Chr.2	22299264	22299264	C	C	T	SNP	OS02G0579600	Intronic	NA	NA	LOC_Os02g36924	OsMADS27 - MADS-box family gene with MIKCc type-box, expressed
Chr.2	22300804	22300804	C	C	T	SNP	OS02G0579600	Intronic	NA	NA	LOC_Os02g36924	OsMADS27 - MADS-box family gene with MIKCc type-box, expressed
Chr.2	23564256	23564256	C	C	T	SNP	OS02G0602100	Exonic	CDS	Silent	LOC_Os02g39010	cyclin-dependent kinase G-1, putative, expressed
Chr.2	24566405	24566405	C	C	T	SNP	OS02G0618100	Exonic	CDS	NA	LOC_Os02g40500	OsGrx_C2.1 - glutaredoxin subgroup I, expressed
Chr.2	26769264	26769264	C	C	T	SNP	OS02G0661100	Intronic	NA	NA	LOC_Os02g44230	CPuORF22 - conserved peptide uORF-containing transcript, expressed
Chr.2	34026583	34026583	C	C	T	SNP	OS02G0799000	Exonic	CDS	Silent	LOC_Os02g55560	protein phosphatase 2C, putative, expressed
Chr.2	34044380	34044380	C	C	T	SNP	OS02G0799300	Intronic	NA	NA	LOC_Os02g55590	expressed protein
Chr.2	34674113	34674113	C	C	T	SNP	OS02G0810900	Intronic	NA	NA	LOC_Os02g56600	no apical meristem protein, putative, expressed
Chr.2	34835171	34835171	C	C	T	SNP	OS02G0813350	Intronic	NA	NA	LOC_Os02g56840	OsFBX70 - F-box domain containing protein, expressed
Chr.2	35082337	35082337	C	C	T	SNP	OS02G0817700	Intronic	NA	NA	LOC_Os02g57260	3-ketoacyl-CoA thiolase, peroxisomal precursor, putative, expressed
Chr.2	35095531	35095531	C	C	T	SNP	OS02G0817900	Intronic	NA	NA	LOC_Os02g57290	cytochrome P450, putative, expressed
Chr.2	35096448	35096448	C	C	T	SNP	OS02G0817900	Intronic	NA	NA	LOC_Os02g57290	cytochrome P450, putative, expressed
Chr.2	35098197	35098197	C	C	T	SNP	OS02G0817900	Intronic	NA	NA	LOC_Os02g57290	cytochrome P450, putative, expressed
Chr.2	1397067	1397067	A	A	T	SNP	OS02G0126500	Exonic	3UTR	NA	LOC_Os02g03420	pentatricopeptide repeat-containing protein, putative, expressed
Chr.2	4297487	4297487	A	A	T	SNP	OS02G0177800	Intronic	NA	NA	LOC_Os02g08120	calmodulin binding protein, putative, expressed

Chr.2	4453236	4453236	A	A	T	SNP	OS02G0180000	Intronic	NA	NA	LOC_Os02g08364	protein phosphatase 2C, putative, expressed
Chr.2	5252362	5252362	A	A	T	SNP	OS02G0194200	Intronic	NA	NA	LOC_Os02g10080	zinc finger C-x8-C-x5-C-x3-H type family protein, expressed
Chr.2	5551309	5551309	A	A	T	SNP	OS02G0198900	Intronic	NA	NA	LOC_Os02g10540	ribosomal protein L7/L12 C-terminal domain containing protein, expressed
Chr.2	5760328	5760328	A	A	T	SNP	OS02G0202950	Exonic	CDS	NA	LOC_Os02g10860	bZIP transcription factor domain containing protein, expressed
Chr.2	6832513	6832513	A	A	T	SNP	OS02G0222500	Exonic	3UTR	NA	LOC_Os02g12939	expressed protein
Chr.2	6835307	6835307	A	A	T	SNP	OS02G0222500	Intronic	NA	NA	LOC_Os02g12939	expressed protein
Chr.2	9504202	9504202	A	A	T	SNP	OS02G0266000	Intronic	NA	NA	LOC_Os02g16630	tryptophan biosynthesis protein trpCF, putative, expressed
Chr.2	9925425	9925425	A	A	T	SNP	OS02G0272900	Intronic	NA	NA	LOC_Os02g17304	resistance protein, putative, expressed
Chr.2	9925862	9925862	A	A	T	SNP	OS02G0272900	Intronic	NA	NA	LOC_Os02g17304	resistance protein, putative, expressed
Chr.2	9926007	9926007	A	A	T	SNP	OS02G0272900	Intronic	NA	NA	LOC_Os02g17304	resistance protein, putative, expressed
Chr.2	9973316	9973316	A	A	T	SNP	OS02G0273700	Intronic	NA	NA	LOC_Os02g17350	VHS and GAT domain containing protein, expressed
Chr.2	9973697	9973697	A	A	T	SNP	OS02G0273700	Intronic	NA	NA	LOC_Os02g17350	VHS and GAT domain containing protein, expressed
Chr.2	9989745	9989745	A	A	T	SNP	OS02G0273900	Intronic	NA	NA	LOC_Os02g17370	expressed protein
Chr.2	17158191	17158191	A	A	T	SNP	OS02G0491400	Exonic	CDS	Missense	LOC_Os02g28980	peptidyl-prolyl isomerase, putative, expressed
Chr.2	18828608	18828608	A	A	T	SNP	OS02G0518100	Exonic	CDS	Missense	LOC_Os02g31874	DUF803 domain containing, putative, expressed
Chr.2	34833978	34833978	A	A	T	SNP	OS02G0813350	Exonic	CDS	Missense	LOC_Os02g56840	OsFBX70 - F-box domain containing protein, expressed
Chr.2	35098135	35098135	A	A	T	SNP	OS02G0817900	Exonic	CDS	Nonsense	LOC_Os02g57290	cytochrome P450, putative, expressed
Chr.2	35097669	35097669	A	A	T	SNP	OS02G0817900	Intronic	NA	NA	LOC_Os02g57290	cytochrome P450, putative, expressed
Chr.2	80610	80610	A	A	T	SNP	OS02G0101500	Intronic	NA	NA	LOC_Os02g01150	erythronate-4-phosphate dehydrogenase domain containing protein, expressed
Chr.2	1083919	1083919	C	C	T	SNP	OS02G0120800	Exonic	5UTR	NA	LOC_Os02g02840	ras-related protein, putative, expressed
Chr.2	3038145	3038145	C	C	T	SNP	OS02G0155400	Exonic	CDS	Missense	LOC_Os02g06090	phytosulfokine receptor precursor, putative, expressed
Chr.2	4298989	4298989	C	C	T	SNP	OS02G0177800	Exonic	5UTR	NA	LOC_Os02g08120	calmodulin binding protein, putative, expressed
Chr.2	4298713	4298713	C	C	T	SNP	OS02G0177800	Intronic	NA	NA	LOC_Os02g08120	calmodulin binding protein, putative, expressed
Chr.2	4390486	4390486	C	C	T	SNP	OS02G0179200	Exonic	3UTR	NA	LOC_Os02g08270	class I glutamine amidotransferase, putative, expressed
Chr.2	4889626	4889626	C	C	T	SNP	OS02G0188000	Exonic	CDS	Silent	LOC_Os02g09510	limonoid UDP-glucosyltransferase, putative, expressed
Chr.2	4889934	4889934	C	C	T	SNP	OS02G0188000	Exonic	CDS	Missense	LOC_Os02g09510	limonoid UDP-glucosyltransferase, putative, expressed
Chr.2	5156512	5156512	C	C	T	SNP	OS02G0192500	Intronic	NA	NA	LOC_Os02g09930	CSLA1 - cellulose synthase-like family A; mannan synthase, expressed
Chr.2	5157484	5157484	C	C	T	SNP	OS02G0192500	Intronic	NA	NA	LOC_Os02g09930	CSLA1 - cellulose synthase-like family A; mannan synthase, expressed
Chr.2	5157723	5157723	C	C	T	SNP	OS02G0192500	Intronic	NA	NA	LOC_Os02g09930	CSLA1 - cellulose synthase-like family A; mannan synthase, expressed
Chr.2	5194939	5194939	C	C	T	SNP	OS02G0193300	Exonic	CDS	Missense	LOC_Os02g09990	expressed protein
Chr.2	5250343	5250343	G	G	T	SNP	OS02G0194200	Exonic	5UTR	NA	LOC_Os02g10080	zinc finger C-x8-C-x5-C-x3-H type family protein, expressed
Chr.2	5250571	5250571	A	A	T	SNP	OS02G0194200	Exonic	5UTR	NA	LOC_Os02g10080	zinc finger C-x8-C-x5-C-x3-H type family protein, expressed
Chr.2	5250604	5250604	C	C	T	SNP	OS02G0194200	Exonic	5UTR	NA	LOC_Os02g10080	zinc finger C-x8-C-x5-C-x3-H type family protein, expressed
Chr.2	5706788	5706788	C	C	T	SNP	OS02G0202200	Exonic	CDS	Silent	LOC_Os02g10780	SPX domain containing protein, putative, expressed
Chr.2	5760043	5760043	C	C	T	SNP	OS02G0202950	Exonic	CDS	NA	LOC_Os02g10860	bZIP transcription factor domain containing protein, expressed
Chr.2	5760379	5760379	C	C	T	SNP	OS02G0202950	Exonic	CDS	NA	LOC_Os02g10860	bZIP transcription factor domain containing protein, expressed
Chr.2	5760432	5760432	C	C	T	SNP	OS02G0202950	Exonic	CDS	NA	LOC_Os02g10860	bZIP transcription factor domain containing protein, expressed
Chr.2	5760448	5760448	G	G	T	SNP	OS02G0202950	Exonic	CDS	NA	LOC_Os02g10860	bZIP transcription factor domain containing protein, expressed
Chr.2	5760469	5760469	C	C	T	SNP	OS02G0202950	Exonic	CDS	NA	LOC_Os02g10860	bZIP transcription factor domain containing protein, expressed
Chr.2	6610143	6610143	C	C	T	SNP	OS02G0218400	Exonic	CDS	Silent	LOC_Os02g12660	protein kinase domain containing protein, putative, expressed
Chr.2	7220908	7220908	C	C	T	SNP	OS02G0228300	Exonic	3UTR	NA	LOC_Os02g13510	receptor-like protein kinase 5 precursor, putative, expressed
Chr.2	7222428	7222428	C	C	T	SNP	OS02G0228300	Exonic	CDS	Silent	LOC_Os02g13510	receptor-like protein kinase 5 precursor, putative, expressed
Chr.2	7222788	7222788	G	G	T	SNP	OS02G0228300	Exonic	CDS	Silent	LOC_Os02g13510	receptor-like protein kinase 5 precursor, putative, expressed
Chr.2	7223151	7223151	C	C	T	SNP	OS02G0228300	Exonic	CDS	Silent	LOC_Os02g13510	receptor-like protein kinase 5 precursor, putative, expressed
Chr.2	7223445	7223445	C	C	T	SNP	OS02G0228300	Exonic	CDS	Silent	LOC_Os02g13510	receptor-like protein kinase 5 precursor, putative, expressed
Chr.2	7223643	7223643	C	C	T	SNP	OS02G0228300	Exonic	CDS	Silent	LOC_Os02g13510	receptor-like protein kinase 5 precursor, putative, expressed
Chr.2	7222448	7222448	C	C	T	SNP	OS02G0228300	Exonic	CDS	Missense	LOC_Os02g13510	receptor-like protein kinase 5 precursor, putative, expressed
Chr.2	7222658	7222658	C	C	T	SNP	OS02G0228300	Exonic	CDS	Missense	LOC_Os02g13510	receptor-like protein kinase 5 precursor, putative, expressed
Chr.2	7222762	7222762	C	C	T	SNP	OS02G0228300	Exonic	CDS	Missense	LOC_Os02g13510	receptor-like protein kinase 5 precursor, putative, expressed
Chr.2	7222763	7222763	C	C	T	SNP	OS02G0228300	Exonic	CDS	Missense	LOC_Os02g13510	receptor-like protein kinase 5 precursor, putative, expressed
Chr.2	7222955	7222955	C	C	T	SNP	OS02G0228300	Exonic	CDS	Missense	LOC_Os02g13510	receptor-like protein kinase 5 precursor, putative, expressed
Chr.2	7223608	7223608	C	C	T	SNP	OS02G0228300	Exonic	CDS	Missense	LOC_Os02g13510	receptor-like protein kinase 5 precursor, putative, expressed
Chr.2	7223731	7223731	G	G	T	SNP	OS02G0228300	Exonic	CDS	Missense	LOC_Os02g13510	receptor-like protein kinase 5 precursor, putative, expressed
Chr.2	7223769	7223769	C	C	T	SNP	OS02G0228300	Exonic	CDS	Missense	LOC_Os02g13510	receptor-like protein kinase 5 precursor, putative, expressed
Chr.2	7465143	7465143	G	G	T	SNP	OS02G0232000	Exonic	3UTR	NA	LOC_Os02g13800	HSF-type DNA-binding domain containing protein, expressed
Chr.2	8584044	8584044	G	G	T	SNP	OS02G0252200	Exonic	CDS	Missense	LOC_Os02g15340	no apical meristem protein, putative, expressed
Chr.2	8978454	8978454	A	A	T	SNP	OS02G0258900	Intronic	NA	NA	LOC_Os02g15870	molybdenum cofactor biosynthesis protein 1, putative, expressed
Chr.2	9504203	9504203	G	G	T	SNP	OS02G0266000	Intronic	NA	NA	LOC_Os02g16630	tryptophan biosynthesis protein trpCF, putative, expressed
Chr.2	9926339	9926339	C	C	T	SNP	OS02G0272900	Intronic	NA	NA	LOC_Os02g17304	resistance protein, putative, expressed
Chr.2	9973220	9973220	C	C	T	SNP	OS02G0273700	Intronic	NA	NA	LOC_Os02g17350	VHS and GAT domain containing protein, expressed
Chr.2	9976675	9976675	A	A	T	SNP	OS02G0273700	Intronic	NA	NA	LOC_Os02g17350	VHS and GAT domain containing protein, expressed
Chr.2	16014123	16014123	C	C	T	SNP	OS02G0471500	Exonic	5UTR	NA	LOC_Os02g27220	protein phosphatase 2C, putative, expressed
Chr.2	17527660	17527660	G	G	T	SNP	OS02G0498300	Exonic	CDS	NA	LOC_Os02g29510	DUF803 domain containing, putative, expressed
Chr.2	17535898	17535898	C	C	T	SNP	OS02G0498300	Intronic	NA	NA	LOC_Os02g29510	DUF803 domain containing, putative, expressed
Chr.2	22468761	22468761	C	C	T	SNP	OS02G0582600	Exonic	CDS	Missense	LOC_Os02g37160	heavy metal transport/detoxification protein, putative, expressed
Chr.2	22469577	22469577	C	C	T	SNP	OS02G0582600	Intronic	NA	NA	LOC_Os02g37160	heavy metal transport/detoxification protein, putative, expressed
Chr.2	22688175	22688175	G	G	T	SNP	OS02G0588500	Intronic	NA	NA	LOC_Os02g37590	glycerophosphoryl diester phosphodiesterase family protein, putative, expressed
Chr.2	23527315	23527315	C	C	T	SNP	OS02G0601300	Exonic	5UTR	NA	LOC_Os02g38920	glyceraldehyde-3-phosphate dehydrogenase, putative, expressed
Chr.2	23562368	23562368	C	C	T	SNP	OS02G0602100	Exonic	5UTR	NA	LOC_Os02g39010	cyclin-dependent kinase G-1, putative, expressed
Chr.2	23563474	23563474	G	G	T	SNP	OS02G0602100	Exonic	CDS	Missense	LOC_Os02g39010	cyclin-dependent kinase G-1, putative, expressed
Chr.2	24738814	24738814	G	G	T	SNP	OS02G0621600	Exonic	CDS	Missense	LOC_Os02g40820	expressed protein
Chr.2	24904685	24904685	G	G	T	SNP	OS02G0624400	Intronic	NA	NA	LOC_Os02g41520	glycosyl transferase 8 domain containing protein, putative, expressed
Chr.2	27436757	27436757	C	C	T	SNP	OS02G0673700	Exonic	CDS	Missense	LOC_Os02g45200	dof zinc finger domain containing protein, putative, expressed
Chr.2	28779485	28779485	A	A	T	SNP	OS02G0699700	Intronic	NA	NA	LOC_Os02g47150	DNA topoisomerase 2, putative, expressed
Chr.2	30458966	30458966	C	C	T	SNP	OS02G0731200	Intronic	NA	NA	LOC_Os02g49840	OsMADS57 - MADS-box family gene with MIKCc type-box, expressed
Chr.2	33954062	33954062	C	C	T	SNP	OS02G0797600	Exonic	CDS	Silent	LOC_Os02g55430	alginate regulatory protein AlgP, putative, expressed
Chr.2	34043292	34043292	A	A	T	SNP	OS02G0799300	Intronic	NA	NA	LOC_Os02g55590	expressed protein
Chr.2	35083504	35083504	C	C	T	SNP	OS02G0817700	Intronic	NA	NA	LOC_Os02g57260	3-ketoacyl-CoA thiolase, peroxisomal precursor, putative, expressed
Chr.2	35084693	35084693	C	C	T	SNP	OS02G0817700	Intronic	NA	NA	LOC_Os02g57260	3-ketoacyl-CoA thiolase, peroxisomal precursor, putative, expressed
Chr.2	35099011	35099011	C	C	T	SNP	OS02G0817900	Exonic	CDS	Missense	LOC_Os02g57290	cytochrome P450, putative, expressed
Chr.2	35096606	35096606	C	C	T	SNP	OS02G0817900	Intronic	NA	NA	LOC_Os02g57290	cytochrome P450, putative, expressed
Chr.2	35098543	35098543	A	A	T	SNP	OS02G0817900	Intronic	NA	NA	LOC_Os02g57290	cytochrome P450, putative, expressed
Chr.2	79615	79625	TGTATGTGCTA	TGTATGTGCTA	T	INDEL	OS02G0101500	Exonic	3UTR	NA	LOC_Os02g01150	erythronate-4-phosphate dehydrogenase domain containing protein, expressed
Chr.2	119794	119796	TGC	TGC	T	INDEL	OS02G0102200	Intronic	NA	NA	LOC_Os02g01210	amino acid transporter, putative, expressed
Chr.2	2744903	2744906	TGAG	TGAG	T	INDEL	OS02G0149800	Intronic	NA	NA	LOC_Os02g05630	protein phosphatase 2C, putative, expressed

Chr.2	6720155	6720156	TG	TG	T	INDEL	OS02G0220500	Exonic	3UTR	NA	LOC_Os02g12794	elongation factor 1-gamma, putative, expressed
Chr.2	9925369	9925377	TAAAAGCCC	TAAAAGCCC	T	INDEL	OS02G0272900	Intronic	NA	NA	LOC_Os02g17304	resistance protein, putative, expressed
Chr.2	11487630	11487631	TA	TA	T	INDEL	OS02G0299300	Intronic	NA	NA	LOC_Os02g19650	hydrolase, alpha/beta fold family domain containing protein, expressed
Chr.2	17536125	17536126	TG	TG	T	INDEL	OS02G0498300	Intronic	NA	NA	LOC_Os02g29510	DUF803 domain containing, putative, expressed
Chr.2	22300205	22300210	TTAAGA	TTAAGA	T	INDEL	OS02G0579600	Intronic	NA	NA	LOC_Os02g36924	OsMADS27 - MADS-box family gene with MIKCc type-box, expressed
Chr.2	24737023	24737025	TTG	TTG	T	INDEL	OS02G0621600	Exonic	3UTR	NA	LOC_Os02g40820	expressed protein
Chr.2	33954415	33954418	TGTC	TGTC	T	INDEL	OS02G0797600	Exonic	3UTR	NA	LOC_Os02g55430	alginate regulatory protein AlgP, putative, expressed
Chr.2	34224833	34224835	TCG	TCG	T	INDEL	OS02G0802700	Intronic	NA	NA	LOC_Os02g55910	monogalactosyldiacylglycerol synthase, putative, expressed
Chr.2	35098520	35098521	TG	TG	T	INDEL	OS02G0817900	Intronic	NA	NA	LOC_Os02g57290	cytochrome P450, putative, expressed
Chr.12	23806836	23806836	C	C	T	SNP	OS12G0576600	Intronic	NA	NA	LOC_Os12g38750	nucleotide pyrophosphatase/phosphodiesterase, putative, expressed
Chr.12	23807090	23807090	C	C	T	SNP	OS12G0576600	Intronic	NA	NA	LOC_Os12g38750	nucleotide pyrophosphatase/phosphodiesterase, putative, expressed
Chr.12	25326439	25326439	C	C	T	SNP	OS12G0601400	Intronic	NA	NA	LOC_Os12g40900	OsIAA31 - Auxin-responsive Aux/IAA gene family member, expressed
Chr.12	10081340	10081340	A	A	T	SNP	OS12G0274700	Exonic	CDS	Silent	LOC_Os12g17600	ribulose bisphosphate carboxylase small chain, chloroplast precursor, putative, expressed
Chr.12	24184349	24184349	A	A	T	SNP	OS12G0582700	Exonic	3UTR	NA	LOC_Os12g39310	cytochrome P450, putative, expressed
Chr.12	24185242	24185242	A	A	T	SNP	OS12G0582700	Intronic	NA	NA	LOC_Os12g39310	cytochrome P450, putative, expressed
Chr.12	24185345	24185346	TA	TA	T	INDEL	OS12G0582700	Intronic	NA	NA	LOC_Os12g39310	cytochrome P450, putative, expressed
Chr.12	17575494	17575494	G	G	T	SNP	OS12G0479400	Intronic	NA	NA	LOC_Os12g29520	auxin response factor, putative, expressed
Chr.12	18337397	18337397	G	G	T	SNP	OS12G0489100	Intronic	NA	NA	LOC_Os12g30540	ubiquitin carboxyl-terminal hydrolase, putative, expressed
Chr.12	9353002	9353002	C	C	T	SNP	OS12G0264500	Intronic	NA	NA	LOC_Os12g16350	enoyl-CoA hydratase/isomerase family protein, putative, expressed
Chr.12	9918066	9918066	C	C	T	SNP	OS12G0271700	Intronic	NA	NA	LOC_Os12g17320	polyprenyl synthetase, putative, expressed
Chr.12	9918896	9918896	C	C	T	SNP	OS12G0271700	Intronic	NA	NA	LOC_Os12g17320	polyprenyl synthetase, putative, expressed
Chr.12	12987872	12987872	C	C	T	SNP	OS12G0418600	Intronic	NA	NA	LOC_Os12g22970	expressed protein
Chr.12	12987899	12987899	C	C	T	SNP	OS12G0418600	Intronic	NA	NA	LOC_Os12g22970	expressed protein
Chr.12	22133768	22133768	C	C	T	SNP	OS12G0547600	Exonic	3UTR	NA	LOC_Os12g36110	calmodulin binding protein, putative, expressed
Chr.12	22591686	22591686	C	C	T	SNP	OS12G0555200	Exonic	5UTR	NA	LOC_Os12g36850	pathogenesis-related Bet v I family protein, putative, expressed
Chr.12	25521813	25521813	C	C	T	SNP	OS12G0604700	Intronic	NA	NA	LOC_Os12g41180	LSTK-1-like kinase, putative, expressed
Chr.12	25651895	25651895	C	C	T	SNP	OS12G0607100	Exonic	3UTR	NA	LOC_Os12g41400	eukaryotic translation initiation factor 2 subunit gamma, putative, expressed
Chr.12	25649502	25649502	C	C	T	SNP	OS12G0607100	Intronic	NA	NA	LOC_Os12g41400	eukaryotic translation initiation factor 2 subunit gamma, putative, expressed
Chr.12	25649526	25649526	C	C	T	SNP	OS12G0607100	Intronic	NA	NA	LOC_Os12g41400	eukaryotic translation initiation factor 2 subunit gamma, putative, expressed
Chr.12	25651438	25651438	C	C	T	SNP	OS12G0607100	Intronic	NA	NA	LOC_Os12g41400	eukaryotic translation initiation factor 2 subunit gamma, putative, expressed
Chr.12	25651492	25651492	C	C	T	SNP	OS12G0607100	Intronic	NA	NA	LOC_Os12g41400	eukaryotic translation initiation factor 2 subunit gamma, putative, expressed
Chr.12	3231943	3231943	A	A	T	SNP	OS12G0163700	Intronic	NA	NA	LOC_Os12g06660	actin, putative, expressed
Chr.12	25650015	25650015	A	A	T	SNP	OS12G0607100	Intronic	NA	NA	LOC_Os12g41400	eukaryotic translation initiation factor 2 subunit gamma, putative, expressed
Chr.12	25651432	25651432	A	A	T	SNP	OS12G0607100	Intronic	NA	NA	LOC_Os12g41400	eukaryotic translation initiation factor 2 subunit gamma, putative, expressed
Chr.12	25651468	25651468	A	A	T	SNP	OS12G0607100	Intronic	NA	NA	LOC_Os12g41400	eukaryotic translation initiation factor 2 subunit gamma, putative, expressed
Chr.12	83435	83435	C	C	T	SNP	OS12G0101800	Exonic	CDS	Missense	LOC_Os12g01140	AGC_PVPK_like_kin82y.2 - ACG kinases include homologs to PKA, PKG and PKC, expressed
Chr.12	9353463	9353463	G	G	T	SNP	OS12G0264500	Intronic	NA	NA	LOC_Os12g16350	enoyl-CoA hydratase/isomerase family protein, putative, expressed
Chr.12	9918100	9918100	C	C	T	SNP	OS12G0271700	Intronic	NA	NA	LOC_Os12g17320	polyprenyl synthetase, putative, expressed
Chr.12	9918192	9918192	C	C	T	SNP	OS12G0271700	Intronic	NA	NA	LOC_Os12g17320	polyprenyl synthetase, putative, expressed
Chr.12	9918718	9918718	C	C	T	SNP	OS12G0271700	Intronic	NA	NA	LOC_Os12g17320	polyprenyl synthetase, putative, expressed
Chr.12	9919199	9919199	C	C	T	SNP	OS12G0271700	Intronic	NA	NA	LOC_Os12g17320	polyprenyl synthetase, putative, expressed
Chr.12	9919701	9919701	G	G	T	SNP	OS12G0271700	Intronic	NA	NA	LOC_Os12g17320	polyprenyl synthetase, putative, expressed
Chr.12	24185899	24185899	C	C	T	SNP	OS12G0582700	Exonic	CDS	Missense	LOC_Os12g39310	cytochrome P450, putative, expressed
Chr.12	25445839	25445839	G	G	T	SNP	OS12G0603700	Exonic	CDS	Silent	LOC_Os12g41090	CAMK_KIN1/SNF1/Nim1_like.38 - CAMK includes calcium/calmodulin depedent protein kinases, expressed
Chr.12	25445380	25445380	C	C	T	SNP	OS12G0603700	Exonic	CDS	Missense	LOC_Os12g41090	CAMK_KIN1/SNF1/Nim1_like.38 - CAMK includes calcium/calmodulin depedent protein kinases, expressed
Chr.12	25445923	25445923	C	C	T	SNP	OS12G0603700	Exonic	CDS	Missense	LOC_Os12g41090	CAMK_KIN1/SNF1/Nim1_like.38 - CAMK includes calcium/calmodulin depedent protein kinases, expressed
Chr.12	25519940	25519940	A	A	T	SNP	OS12G0604700	Intronic	NA	NA	LOC_Os12g41180	LSTK-1-like kinase, putative, expressed
Chr.12	25651598	25651598	C	C	T	SNP	OS12G0607100	Exonic	CDS	Silent	LOC_Os12g41400	eukaryotic translation initiation factor 2 subunit gamma, putative, expressed
Chr.12	26903665	26903665	C	C	T	SNP	OS12G0628400	Intronic	NA	NA	LOC_Os12g43363	fatty acid hydroxylase, putative, expressed
Chr.12	25446171	25446177	TCGCCGC	TCGCCGC	T	INDEL	OS12G0603700	Exonic	CDS	InFrame	LOC_Os12g41090	CAMK_KIN1/SNF1/Nim1_like.38 - CAMK includes calcium/calmodulin depedent protein kinases, expressed
Chr.12	25648687	25648691	TATTG	TATTG	T	INDEL	OS12G0607100	Intronic	NA	NA	LOC_Os12g41400	eukaryotic translation initiation factor 2 subunit gamma, putative, expressed
Chr.11	2613139	2613139	G	G	T	SNP	OS11G0155600	Exonic	CDS	Silent	LOC_Os11g05700	ABC transporter family protein, putative, expressed
Chr.11	2606707	2606707	G	G	T	SNP	OS11G0155600	Intronic	NA	NA	LOC_Os11g05700	ABC transporter family protein, putative, expressed
Chr.11	762582	762582	C	C	T	SNP	OS11G0116900	Intronic	NA	NA	LOC_Os11g02480	WRKY46, expressed
Chr.11	2424060	2424060	C	C	T	SNP	OS11G0151700	Exonic	CDS	NA	LOC_Os11g05400	Ser/Thr protein phosphatase family protein, putative, expressed
Chr.11	2424385	2424385	C	C	T	SNP	OS11G0151700	Intronic	NA	NA	LOC_Os11g05400	Ser/Thr protein phosphatase family protein, putative, expressed
Chr.11	2424398	2424398	C	C	T	SNP	OS11G0151700	Intronic	NA	NA	LOC_Os11g05400	Ser/Thr protein phosphatase family protein, putative, expressed
Chr.11	2424435	2424435	C	C	T	SNP	OS11G0151700	Intronic	NA	NA	LOC_Os11g05400	Ser/Thr protein phosphatase family protein, putative, expressed
Chr.11	2608381	2608381	C	C	T	SNP	OS11G0155600	Intronic	NA	NA	LOC_Os11g05700	ABC transporter family protein, putative, expressed
Chr.11	2610121	2610121	C	C	T	SNP	OS11G0155600	Intronic	NA	NA	LOC_Os11g05700	ABC transporter family protein, putative, expressed
Chr.11	2612168	2612168	C	C	T	SNP	OS11G0155600	Intronic	NA	NA	LOC_Os11g05700	ABC transporter family protein, putative, expressed
Chr.11	8388554	8388554	C	C	T	SNP	OS11G0255500	Intronic	NA	NA	LOC_Os11g14910	NADP-dependent oxidoreductase, putative, expressed
Chr.11	10867107	10867107	C	C	T	SNP	OS11G0295900	Intronic	NA	NA	LOC_Os11g19060	BABY BOOM 1, putative, expressed
Chr.11	22176865	22176865	C	C	T	SNP	OS11G0586300	Exonic	CDS	NA	LOC_Os11g37560	expressed protein
Chr.11	22177423	22177423	C	C	T	SNP	OS11G0586300	Exonic	CDS	NA	LOC_Os11g37560	expressed protein
Chr.11	22177485	22177485	C	C	T	SNP	OS11G0586300	Exonic	CDS	NA	LOC_Os11g37560	expressed protein
Chr.11	26878565	26878565	C	C	T	SNP	OS11G0666200	Exonic	CDS	Missense	LOC_Os11g44430	protein kinase, putative, expressed
Chr.11	2605932	2605932	A	A	T	SNP	OS11G0155600	Exonic	3UTR	NA	LOC_Os11g05700	ABC transporter family protein, putative, expressed
Chr.11	2609054	2609054	A	A	T	SNP	OS11G0155600	Intronic	NA	NA	LOC_Os11g05700	ABC transporter family protein, putative, expressed
Chr.11	7127396	7127396	A	A	T	SNP	OS11G0234200	Intronic	NA	NA	LOC_Os11g12650	PHD-finger domain containing protein, putative, expressed
Chr.11	8387897	8387897	A	A	T	SNP	OS11G0255500	Exonic	3UTR	NA	LOC_Os11g14910	NADP-dependent oxidoreductase, putative, expressed
Chr.11	8443992	8443992	A	A	T	SNP	OS11G0256200	Intronic	NA	NA	LOC_Os11g14990	expressed protein
Chr.11	761395	761397	TTA	TTA	T	INDEL	OS11G0116900	Intronic	NA	NA	LOC_Os11g02480	WRKY46, expressed
Chr.11	2491665	2491665	G	G	T	SNP	OS11G0153300	Exonic	5UTR	NA	LOC_Os11g05530	expressed protein
Chr.11	4251601	4251601	G	G	T	SNP	OS11G0183900	Exonic	3UTR	NA	LOC_Os11g08100	eukaryotic aspartyl protease domain containing protein, expressed
Chr.11	4247956	4247956	G	G	T	SNP	OS11G0183900	Intronic	NA	NA	LOC_Os11g08100	eukaryotic aspartyl protease domain containing protein, expressed
Chr.11	4249851	4249851	G	G	T	SNP	OS11G0183900	Intronic	NA	NA	LOC_Os11g08100	eukaryotic aspartyl protease domain containing protein, expressed
Chr.11	6323579	6323579	G	G	T	SNP	OS11G0220800	Exonic	CDS	Missense	LOC_Os11g11390	ribosomal protein, putative, expressed
Chr.11	6322277	6322277	G	G	T	SNP	OS11G0220800	Intronic	NA	NA	LOC_Os11g11390	ribosomal protein, putative, expressed
Chr.11	6638407	6638407	G	G	T	SNP	OS11G0226933	Exonic	CDS	NA	LOC_Os11g11960	disease resistance protein RPM1, putative, expressed
Chr.11	6639412	6639412	G	G	T	SNP	OS11G0226933	Exonic	CDS	NA	LOC_Os11g11960	disease resistance protein RPM1, putative, expressed
Chr.11	6650234	6650234	G	G	T	SNP	OS11G0227000	Exonic	3UTR	NA	LOC_Os11g11980	expressed protein
Chr.11	7125809	7125809	G	G	T	SNP	OS11G0234200	Exonic	CDS	Missense	LOC_Os11g12650	PHD-finger domain containing protein, putative, expressed
Chr.11	14049406	14049406	G	G	T	SNP	OS11G0434000	Intronic	NA	NA	LOC_Os11g24630	magnesium-dependent phosphatase 1, putative, expressed
Chr.11	14049709	14049709	G	G	T	SNP	OS11G0434000	Intronic	NA	NA	LOC_Os11g24630	magnesium-dependent phosphatase 1, putative, expressed

Chr.11	14051191	14051191	G	G	T	SNP	OS11G0434000	Intronic	NA	NA	LOC_Os11g24630	magnesium-dependent phosphatase 1, putative, expressed
Chr.11	17285208	17285208	G	G	T	SNP	OS11G0490100	Exonic	3UTR	NA	LOC_Os11g29780	plant-specific domain TIGR01627 family protein, expressed
Chr.11	17285353	17285353	G	G	T	SNP	OS11G0490100	Exonic	3UTR	NA	LOC_Os11g29780	plant-specific domain TIGR01627 family protein, expressed
Chr.11	17353553	17353553	G	G	T	SNP	OS11G0490900	Intronic	NA	NA	LOC_Os11g29870	WRKY72, expressed
Chr.11	17353766	17353766	G	G	T	SNP	OS11G0490900	Intronic	NA	NA	LOC_Os11g29870	WRKY72, expressed
Chr.11	21301074	21301074	G	G	T	SNP	OS11G0570000	Exonic	3UTR	NA	LOC_Os11g36200	receptor-like protein kinase 2 precursor, putative, expressed
Chr.11	23231891	23231891	G	G	T	SNP	OS11G0603200	Intronic	NA	NA	LOC_Os11g39020	ABC transporter, ATP-binding protein, putative, expressed
Chr.11	23232221	23232221	G	G	T	SNP	OS11G0603200	Intronic	NA	NA	LOC_Os11g39020	ABC transporter, ATP-binding protein, putative, expressed
Chr.11	27488349	27488349	G	G	T	SNP	OS11G0679700	Intronic	NA	NA	LOC_Os11g45400	glycerol-3-phosphate acyltransferase, putative, expressed
Chr.11	28121892	28121892	G	G	T	SNP	OS11G0691100	Exonic	CDS	Missense	LOC_Os11g46860	wall-associated receptor kinase-like 4 precursor, putative, expressed
Chr.11	28854219	28854219	G	G	T	SNP	OS11G0704800	Exonic	CDS	Silent	LOC_Os11g47840	OsRhmbd18 - Putative Rhomboid homologue, expressed
Chr.11	1226007	1226007	C	C	T	SNP	OS11G0126800	Intronic	NA	NA	LOC_Os11g03290	nucleoside-triphosphatase, putative, expressed
Chr.11	4251664	4251664	C	C	T	SNP	OS11G0183900	Exonic	3UTR	NA	LOC_Os11g08100	eukaryotic aspartyl protease domain containing protein, expressed
Chr.11	4251701	4251701	C	C	T	SNP	OS11G0183900	Exonic	3UTR	NA	LOC_Os11g08100	eukaryotic aspartyl protease domain containing protein, expressed
Chr.11	4251707	4251707	C	C	T	SNP	OS11G0183900	Exonic	3UTR	NA	LOC_Os11g08100	eukaryotic aspartyl protease domain containing protein, expressed
Chr.11	4250876	4250876	C	C	T	SNP	OS11G0183900	Exonic	CDS	Silent	LOC_Os11g08100	eukaryotic aspartyl protease domain containing protein, expressed
Chr.11	4247926	4247926	C	C	T	SNP	OS11G0183900	Intronic	NA	NA	LOC_Os11g08100	eukaryotic aspartyl protease domain containing protein, expressed
Chr.11	4248530	4248530	C	C	T	SNP	OS11G0183900	Intronic	NA	NA	LOC_Os11g08100	eukaryotic aspartyl protease domain containing protein, expressed
Chr.11	4248542	4248542	C	C	T	SNP	OS11G0183900	Intronic	NA	NA	LOC_Os11g08100	eukaryotic aspartyl protease domain containing protein, expressed
Chr.11	6639423	6639423	C	C	T	SNP	OS11G0226933	Exonic	CDS	NA	LOC_Os11g11960	disease resistance protein RPM1, putative, expressed
Chr.11	6639504	6639504	C	C	T	SNP	OS11G0226933	Exonic	CDS	NA	LOC_Os11g11960	disease resistance protein RPM1, putative, expressed
Chr.11	6639547	6639547	C	C	T	SNP	OS11G0226933	Exonic	CDS	NA	LOC_Os11g11960	disease resistance protein RPM1, putative, expressed
Chr.11	6639561	6639561	C	C	T	SNP	OS11G0226933	Exonic	CDS	NA	LOC_Os11g11960	disease resistance protein RPM1, putative, expressed
Chr.11	6650184	6650184	C	C	T	SNP	OS11G0227000	Exonic	3UTR	NA	LOC_Os11g11980	expressed protein
Chr.11	8388165	8388165	C	C	T	SNP	OS11G0255500	Exonic	CDS	Silent	LOC_Os11g14910	NADP-dependent oxidoreductase, putative, expressed
Chr.11	8388974	8388974	C	C	T	SNP	OS11G0255500	Intronic	NA	NA	LOC_Os11g14910	NADP-dependent oxidoreductase, putative, expressed
Chr.11	9055929	9055929	C	C	T	SNP	OS11G0265200	Exonic	3UTR	NA	LOC_Os11g16390	expressed protein
Chr.11	10140463	10140463	C	C	T	SNP	OS11G0283500	Intronic	NA	NA	LOC_Os11g18044	peptide transporter PTR2, putative, expressed
Chr.11	10140651	10140651	C	C	T	SNP	OS11G0283500	Intronic	NA	NA	LOC_Os11g18044	peptide transporter PTR2, putative, expressed
Chr.11	10140764	10140764	C	C	T	SNP	OS11G0283500	Intronic	NA	NA	LOC_Os11g18044	peptide transporter PTR2, putative, expressed
Chr.11	14049105	14049105	C	C	T	SNP	OS11G0434000	Exonic	CDS	Missense	LOC_Os11g24630	magnesium-dependent phosphatase 1, putative, expressed
Chr.11	14049249	14049249	C	C	T	SNP	OS11G0434000	Intronic	NA	NA	LOC_Os11g24630	magnesium-dependent phosphatase 1, putative, expressed
Chr.11	14049254	14049254	C	C	T	SNP	OS11G0434000	Intronic	NA	NA	LOC_Os11g24630	magnesium-dependent phosphatase 1, putative, expressed
Chr.11	14049320	14049320	C	C	T	SNP	OS11G0434000	Intronic	NA	NA	LOC_Os11g24630	magnesium-dependent phosphatase 1, putative, expressed
Chr.11	14049430	14049430	C	C	T	SNP	OS11G0434000	Intronic	NA	NA	LOC_Os11g24630	magnesium-dependent phosphatase 1, putative, expressed
Chr.11	14051655	14051655	C	C	T	SNP	OS11G0434000	Intronic	NA	NA	LOC_Os11g24630	magnesium-dependent phosphatase 1, putative, expressed
Chr.11	14051962	14051962	C	C	T	SNP	OS11G0434000	Intronic	NA	NA	LOC_Os11g24630	magnesium-dependent phosphatase 1, putative, expressed
Chr.11	14051982	14051982	C	C	T	SNP	OS11G0434000	Intronic	NA	NA	LOC_Os11g24630	magnesium-dependent phosphatase 1, putative, expressed
Chr.11	17049465	17049465	C	C	T	SNP	OS11G0484300	Exonic	CDS	Silent	LOC_Os11g29380	MCM2 - Putative minichromosome maintenance MCM complex subunit 2, expressed
Chr.11	17050362	17050362	C	C	T	SNP	OS11G0484300	Exonic	CDS	Silent	LOC_Os11g29380	MCM2 - Putative minichromosome maintenance MCM complex subunit 2, expressed
Chr.11	17047762	17047762	C	C	T	SNP	OS11G0484300	Intronic	NA	NA	LOC_Os11g29380	MCM2 - Putative minichromosome maintenance MCM complex subunit 2, expressed
Chr.11	17236031	17236031	C	C	T	SNP	OS11G0489250	Intronic	NA	NA	LOC_Os11g29720	cytochrome P450, putative, expressed
Chr.11	17236624	17236624	C	C	T	SNP	OS11G0489250	Intronic	NA	NA	LOC_Os11g29720	cytochrome P450, putative, expressed
Chr.11	17236745	17236745	C	C	T	SNP	OS11G0489250	Intronic	NA	NA	LOC_Os11g29720	cytochrome P450, putative, expressed
Chr.11	17352869	17352869	C	C	T	SNP	OS11G0490900	Intronic	NA	NA	LOC_Os11g29870	WRKY72, expressed
Chr.11	17353485	17353485	C	C	T	SNP	OS11G0490900	Intronic	NA	NA	LOC_Os11g29870	WRKY72, expressed
Chr.11	17353695	17353695	C	C	T	SNP	OS11G0490900	Intronic	NA	NA	LOC_Os11g29870	WRKY72, expressed
Chr.11	17353763	17353763	C	C	T	SNP	OS11G0490900	Intronic	NA	NA	LOC_Os11g29870	WRKY72, expressed
Chr.11	17353882	17353882	C	C	T	SNP	OS11G0490900	Intronic	NA	NA	LOC_Os11g29870	WRKY72, expressed
Chr.11	17353932	17353932	C	C	T	SNP	OS11G0490900	Intronic	NA	NA	LOC_Os11g29870	WRKY72, expressed
Chr.11	17354161	17354161	C	C	T	SNP	OS11G0490900	Intronic	NA	NA	LOC_Os11g29870	WRKY72, expressed
Chr.11	17354452	17354452	C	C	T	SNP	OS11G0490900	Intronic	NA	NA	LOC_Os11g29870	WRKY72, expressed
Chr.11	17509735	17509735	C	C	T	SNP	OS11G0493100	Exonic	CDS	Silent	LOC_Os11g30110	disease resistance RPP13-like protein 1, putative, expressed
Chr.11	18430818	18430818	C	C	T	SNP	OS11G0514500	Exonic	CDS	Missense	LOC_Os11g31540	BRASSINOSTEROID INSENSITIVE 1-associated receptor kinase 1 precursor, putative, expressed
Chr.11	18834079	18834079	C	C	T	SNP	OS11G0522900	Exonic	CDS	Silent	LOC_Os11g31980	OsSCP63 - Putative Serine Carboxypeptidase homologue, expressed
Chr.11	18935820	18935820	C	C	T	SNP	OS11G0523800	Exonic	3UTR	NA	LOC_Os11g32110	auxin response factor, putative, expressed
Chr.11	18938443	18938443	C	C	T	SNP	OS11G0523800	Exonic	CDS	Missense	LOC_Os11g32110	auxin response factor, putative, expressed
Chr.11	19677003	19677003	C	C	T	SNP	OS11G0539200	Intronic	NA	NA	LOC_Os11g33270	glycosyl hydrolases family 16, putative, expressed
Chr.11	21300087	21300087	C	C	T	SNP	OS11G0570000	Exonic	3UTR	NA	LOC_Os11g36200	receptor-like protein kinase 2 precursor, putative, expressed
Chr.11	21298764	21298764	C	C	T	SNP	OS11G0570000	Exonic	CDS	Missense	LOC_Os11g36200	receptor-like protein kinase 2 precursor, putative, expressed
Chr.11	21993284	21993284	C	C	T	SNP	OS11G0582100	Intronic	NA	NA	LOC_Os11g37230	zinc finger, C3HC4 type domain containing protein, expressed
Chr.11	22273328	22273328	C	C	T	SNP	OS11G0587600	Exonic	3UTR	NA	LOC_Os11g37700	pleiotropic drug resistance protein, putative, expressed
Chr.11	22273448	22273448	C	C	T	SNP	OS11G0587600	Intronic	NA	NA	LOC_Os11g37700	pleiotropic drug resistance protein, putative, expressed
Chr.11	22281629	22281629	C	C	T	SNP	OS11G0587600	Intronic	NA	NA	LOC_Os11g37700	pleiotropic drug resistance protein, putative, expressed
Chr.11	22512015	22512015	C	C	T	SNP	OS11G0592200	Intronic	NA	NA	LOC_Os11g37970	WIP5 - Wound-induced protein precursor, expressed
Chr.11	23185372	23185372	C	C	T	SNP	OS11G0602600	Intronic	NA	NA	LOC_Os11g38959	40S ribosomal protein S9-2, putative, expressed
Chr.11	25424637	25424637	C	C	T	SNP	OS11G0641500	Exonic	CDS	Silent	LOC_Os11g42200	laccase precursor protein, putative, expressed
Chr.11	25425030	25425030	C	C	T	SNP	OS11G0641500	Exonic	CDS	Silent	LOC_Os11g42200	laccase precursor protein, putative, expressed
Chr.11	25425087	25425087	C	C	T	SNP	OS11G0641500	Exonic	CDS	Silent	LOC_Os11g42200	laccase precursor protein, putative, expressed
Chr.11	25424372	25424372	C	C	T	SNP	OS11G0641500	Intronic	NA	NA	LOC_Os11g42200	laccase precursor protein, putative, expressed
Chr.11	26314985	26314985	C	C	T	SNP	OS11G0657300	Exonic	3UTR	NA	LOC_Os11g43620	LSM domain containing protein, expressed
Chr.11	26511180	26511180	C	C	T	SNP	OS11G0660500	Exonic	3UTR	NA	LOC_Os11g43900	translationally-controlled tumor protein, putative, expressed
Chr.11	26879340	26879340	C	C	T	SNP	OS11G0666200	Intronic	NA	NA	LOC_Os11g44430	protein kinase, putative, expressed
Chr.11	27200499	27200499	C	C	T	SNP	OS11G0673100	Exonic	3UTR	NA	LOC_Os11g44940	seed maturation protein PM27, putative, expressed
Chr.11	27488191	27488191	C	C	T	SNP	OS11G0679700	Intronic	NA	NA	LOC_Os11g45400	glycerol-3-phosphate acyltransferase, putative, expressed
Chr.11	28934093	28934093	C	C	T	SNP	OS11G0707000	Intronic	NA	NA	LOC_Os11g47970	AAA-type ATPase family protein, putative, expressed
Chr.11	2762911	2762911	A	A	T	SNP	OS11G0157400	Exonic	CDS	Silent	LOC_Os11g05880	exo70 exocyst complex subunit, putative, expressed
Chr.11	4251430	4251430	A	A	T	SNP	OS11G0183900	Exonic	3UTR	NA	LOC_Os11g08100	eukaryotic aspartyl protease domain containing protein, expressed
Chr.11	4247771	4247771	A	A	T	SNP	OS11G0183900	Intronic	NA	NA	LOC_Os11g08100	eukaryotic aspartyl protease domain containing protein, expressed
Chr.11	4250320	4250320	A	A	T	SNP	OS11G0183900	Intronic	NA	NA	LOC_Os11g08100	eukaryotic aspartyl protease domain containing protein, expressed
Chr.11	4250321	4250321	A	A	T	SNP	OS11G0183900	Intronic	NA	NA	LOC_Os11g08100	eukaryotic aspartyl protease domain containing protein, expressed
Chr.11	4250322	4250322	A	A	T	SNP	OS11G0183900	Intronic	NA	NA	LOC_Os11g08100	eukaryotic aspartyl protease domain containing protein, expressed
Chr.11	4250323	4250323	A	A	T	SNP	OS11G0183900	Intronic	NA	NA	LOC_Os11g08100	eukaryotic aspartyl protease domain containing protein, expressed
Chr.11	4787062	4787062	A	A	T	SNP	OS11G0195500	Intronic	NA	NA	LOC_Os11g09010	lipase, putative, expressed
Chr.11	6639439	6639439	A	A	T	SNP	OS11G0226933	Exonic	CDS	NA	LOC_Os11g11960	disease resistance protein RPM1, putative, expressed

Chr.11	6640129	6640129	A	A	T	SNP	OS11G0226933	Exonic	CDS	NA	LOC_Os11g11960	disease resistance protein RPM1, putative, expressed
Chr.11	14049653	14049653	A	A	T	SNP	OS11G0434000	Intronic	NA	NA	LOC_Os11g24630	magnesium-dependent phosphatase 1, putative, expressed
Chr.11	14049725	14049725	A	A	T	SNP	OS11G0434000	Intronic	NA	NA	LOC_Os11g24630	magnesium-dependent phosphatase 1, putative, expressed
Chr.11	14050786	14050786	A	A	T	SNP	OS11G0434000	Intronic	NA	NA	LOC_Os11g24630	magnesium-dependent phosphatase 1, putative, expressed
Chr.11	14050821	14050821	A	A	T	SNP	OS11G0434000	Intronic	NA	NA	LOC_Os11g24630	magnesium-dependent phosphatase 1, putative, expressed
Chr.11	14050940	14050940	A	A	T	SNP	OS11G0434000	Intronic	NA	NA	LOC_Os11g24630	magnesium-dependent phosphatase 1, putative, expressed
Chr.11	17285202	17285202	A	A	T	SNP	OS11G0490100	Exonic	3UTR	NA	LOC_Os11g29780	plant-specific domain TIGR01627 family protein, expressed
Chr.11	17352851	17352851	A	A	T	SNP	OS11G0490900	Intronic	NA	NA	LOC_Os11g29870	WRKY72, expressed
Chr.11	17353477	17353477	A	A	T	SNP	OS11G0490900	Intronic	NA	NA	LOC_Os11g29870	WRKY72, expressed
Chr.11	17353639	17353639	A	A	T	SNP	OS11G0490900	Intronic	NA	NA	LOC_Os11g29870	WRKY72, expressed
Chr.11	17353643	17353643	A	A	T	SNP	OS11G0490900	Intronic	NA	NA	LOC_Os11g29870	WRKY72, expressed
Chr.11	17353883	17353883	A	A	T	SNP	OS11G0490900	Intronic	NA	NA	LOC_Os11g29870	WRKY72, expressed
Chr.11	17354211	17354211	A	A	T	SNP	OS11G0490900	Intronic	NA	NA	LOC_Os11g29870	WRKY72, expressed
Chr.11	17354768	17354768	A	A	T	SNP	OS11G0490900	Intronic	NA	NA	LOC_Os11g29870	WRKY72, expressed
Chr.11	18831958	18831958	A	A	T	SNP	OS11G0522900	Exonic	5UTR	NA	LOC_Os11g31980	OsSCP63 - Putative Serine Carboxypeptidase homologue, expressed
Chr.11	18936739	18936739	A	A	T	SNP	OS11G0523800	Intronic	NA	NA	LOC_Os11g32110	auxin response factor, putative, expressed
Chr.11	18938363	18938363	A	A	T	SNP	OS11G0523800	Intronic	NA	NA	LOC_Os11g32110	auxin response factor, putative, expressed
Chr.11	19675733	19675733	A	A	T	SNP	OS11G0539200	Intronic	NA	NA	LOC_Os11g33270	glycosyl hydrolases family 16, putative, expressed
Chr.11	19675977	19675977	A	A	T	SNP	OS11G0539200	Intronic	NA	NA	LOC_Os11g33270	glycosyl hydrolases family 16, putative, expressed
Chr.11	23184800	23184800	A	A	T	SNP	OS11G0602600	Intronic	NA	NA	LOC_Os11g38959	40S ribosomal protein S9-2, putative, expressed
Chr.11	23906844	23906844	A	A	T	SNP	OS11G0615000	Intronic	NA	NA	LOC_Os11g40080	lipin, N-terminal conserved region family protein, expressed
Chr.11	25425582	25425582	A	A	T	SNP	OS11G0641500	Intronic	NA	NA	LOC_Os11g42200	laccase precursor protein, putative, expressed
Chr.11	26314919	26314919	A	A	T	SNP	OS11G0657300	Exonic	3UTR	NA	LOC_Os11g43620	LSM domain containing protein, expressed
Chr.11	2425205	2425205	C	C	T	SNP	OS11G0151700	Exonic	CDS	NA	LOC_Os11g05400	Ser/Thr protein phosphatase family protein, putative, expressed
Chr.11	2426111	2426111	C	C	T	SNP	OS11G0151700	Exonic	CDS	NA	LOC_Os11g05400	Ser/Thr protein phosphatase family protein, putative, expressed
Chr.11	2426348	2426348	C	C	T	SNP	OS11G0151700	Exonic	CDS	NA	LOC_Os11g05400	Ser/Thr protein phosphatase family protein, putative, expressed
Chr.11	4108350	4108350	G	G	T	SNP	OS11G0181900	Intronic	NA	NA	LOC_Os11g07940	expressed protein
Chr.11	4108360	4108360	C	C	T	SNP	OS11G0181900	Intronic	NA	NA	LOC_Os11g07940	expressed protein
Chr.11	4247625	4247625	C	C	T	SNP	OS11G0183900	Intronic	NA	NA	LOC_Os11g08100	eukaryotic aspartyl protease domain containing protein, expressed
Chr.11	4247626	4247626	C	C	T	SNP	OS11G0183900	Intronic	NA	NA	LOC_Os11g08100	eukaryotic aspartyl protease domain containing protein, expressed
Chr.11	4247630	4247630	C	C	T	SNP	OS11G0183900	Intronic	NA	NA	LOC_Os11g08100	eukaryotic aspartyl protease domain containing protein, expressed
Chr.11	4250319	4250319	A	A	T	SNP	OS11G0183900	Intronic	NA	NA	LOC_Os11g08100	eukaryotic aspartyl protease domain containing protein, expressed
Chr.11	4973923	4973923	C	C	T	SNP	OS11G0199200	Exonic	CDS	Silent	LOC_Os11g09280	OsPDIL1-1 protein disulfide isomerase PDIL1-1, expressed
Chr.11	6321461	6321461	C	C	T	SNP	OS11G0220800	Intronic	NA	NA	LOC_Os11g11390	ribosomal protein, putative, expressed
Chr.11	6322347	6322347	C	C	T	SNP	OS11G0220800	Intronic	NA	NA	LOC_Os11g11390	ribosomal protein, putative, expressed
Chr.11	7127519	7127519	G	G	T	SNP	OS11G0234200	Intronic	NA	NA	LOC_Os11g12650	PHD-finger domain containing protein, putative, expressed
Chr.11	8446041	8446041	G	G	T	SNP	OS11G0256200	Exonic	5UTR	NA	LOC_Os11g14990	expressed protein
Chr.11	10140166	10140166	G	G	T	SNP	OS11G0283500	Intronic	NA	NA	LOC_Os11g18044	peptide transporter PTR2, putative, expressed
Chr.11	10863716	10863716	C	C	T	SNP	OS11G0295900	Exonic	CDS	Missense	LOC_Os11g19060	BABY BOOM 1, putative, expressed
Chr.11	11594799	11594799	A	A	T	SNP	OS11G0306400	Intronic	NA	NA	LOC_Os11g20090	O-methyltransferase, putative, expressed
Chr.11	14052263	14052263	C	C	T	SNP	OS11G0434000	Exonic	CDS	Silent	LOC_Os11g24630	magnesium-dependent phosphatase 1, putative, expressed
Chr.11	14051038	14051038	A	A	T	SNP	OS11G0434000	Intronic	NA	NA	LOC_Os11g24630	magnesium-dependent phosphatase 1, putative, expressed
Chr.11	14739459	14739459	C	C	T	SNP	OS11G0445300	Exonic	CDS	Silent	LOC_Os11g25860	protein Kinase, putative, expressed
Chr.11	17051511	17051511	C	C	T	SNP	OS11G0484300	Intronic	NA	NA	LOC_Os11g29380	MCM2 - Putative minichromosome maintenance MCM complex subunit 2, expressed
Chr.11	17284411	17284411	C	C	T	SNP	OS11G0490100	Exonic	CDS	Silent	LOC_Os11g29780	plant-specific domain TIGR01627 family protein, expressed
Chr.11	17353061	17353061	G	G	T	SNP	OS11G0490900	Intronic	NA	NA	LOC_Os11g29870	WRKY72, expressed
Chr.11	18941052	18941052	A	A	T	SNP	OS11G0523800	Intronic	NA	NA	LOC_Os11g32110	auxin response factor, putative, expressed
Chr.11	19676083	19676083	A	A	T	SNP	OS11G0539200	Intronic	NA	NA	LOC_Os11g33270	glycosyl hydrolases family 16, putative, expressed
Chr.11	19676086	19676086	G	G	T	SNP	OS11G0539200	Intronic	NA	NA	LOC_Os11g33270	glycosyl hydrolases family 16, putative, expressed
Chr.11	22175810	22175810	A	A	T	SNP	OS11G0586300	Exonic	CDS	NA	LOC_Os11g37560	expressed protein
Chr.11	22175957	22175957	C	C	T	SNP	OS11G0586300	Exonic	CDS	NA	LOC_Os11g37560	expressed protein
Chr.11	22176723	22176723	C	C	T	SNP	OS11G0586300	Intronic	NA	NA	LOC_Os11g37560	expressed protein
Chr.11	22177741	22177741	C	C	T	SNP	OS11G0586300	Intronic	NA	NA	LOC_Os11g37560	expressed protein
Chr.11	22178173	22178173	C	C	T	SNP	OS11G0586300	Intronic	NA	NA	LOC_Os11g37560	expressed protein
Chr.11	22277683	22277683	C	C	T	SNP	OS11G0587600	Exonic	CDS	Silent	LOC_Os11g37700	pleiotropic drug resistance protein, putative, expressed
Chr.11	22502161	22502161	C	C	T	SNP	OS11G0592000	Exonic	CDS	Silent	LOC_Os11g37950	WIP3 - Wound-induced protein precursor, expressed
Chr.11	25423839	25423839	C	C	T	SNP	OS11G0641500	Exonic	CDS	Silent	LOC_Os11g42200	laccase precursor protein, putative, expressed
Chr.11	26510910	26510910	G	G	T	SNP	OS11G0660500	Exonic	3UTR	NA	LOC_Os11g43900	translationally-controlled tumor protein, putative, expressed
Chr.11	27489262	27489262	C	C	T	SNP	OS11G0679700	Exonic	CDS	NA	LOC_Os11g45400	glycerol-3-phosphate acyltransferase, putative, expressed
Chr.11	27489377	27489377	G	G	T	SNP	OS11G0679700	Exonic	CDS	NA	LOC_Os11g45400	glycerol-3-phosphate acyltransferase, putative, expressed
Chr.11	27489378	27489378	C	C	T	SNP	OS11G0679700	Exonic	CDS	NA	LOC_Os11g45400	glycerol-3-phosphate acyltransferase, putative, expressed
Chr.11	27489751	27489751	C	C	T	SNP	OS11G0679700	Exonic	CDS	NA	LOC_Os11g45400	glycerol-3-phosphate acyltransferase, putative, expressed
Chr.11	27488822	27488822	A	A	T	SNP	OS11G0679700	Intronic	NA	NA	LOC_Os11g45400	glycerol-3-phosphate acyltransferase, putative, expressed
Chr.11	28766412	28766412	C	C	T	SNP	OS11G0702400	Exonic	CDS	Missense	LOC_Os11g47630	ZOS11-10 - C2H2 zinc finger protein, expressed
Chr.11	28933387	28933387	A	A	T	SNP	OS11G0707000	Intronic	NA	NA	LOC_Os11g47970	AAA-type ATPase family protein, putative, expressed
Chr.11	2424469	2424473	TGAGA	TGAGA	T	INDEL	OS11G0151700	Intronic	NA	NA	LOC_Os11g05400	Ser/Thr protein phosphatase family protein, putative, expressed
Chr.11	2614104	2614116	TCGCCGCCGCCGC	TCGCCGCCGCCGC	T	INDEL	OS11G0155600	Exonic	5UTR	NA	LOC_Os11g05700	ABC transporter family protein, putative, expressed
Chr.11	3672894	3672903	TCGCGGAGAG	TCGCGGAGAG	T	INDEL	OS11G0174000	Exonic	CDS	InFrame	LOC_Os11g07280	coatomer subunit beta-1, putative, expressed
Chr.11	4251471	4251477	TGTAATA	TGTAATA	T	INDEL	OS11G0183900	Exonic	3UTR	NA	LOC_Os11g08100	eukaryotic aspartyl protease domain containing protein, expressed
Chr.11	6649730	6649731	TG	TG	T	INDEL	OS11G0227000	Intronic	NA	NA	LOC_Os11g11980	expressed protein
Chr.11	10135317	10135333	TGGCCTTATGGGAAATG	TGGCCTTATGGGAAATG	T	INDEL	OS11G0283500	Intronic	NA	NA	LOC_Os11g18044	peptide transporter PTR2, putative, expressed
Chr.11	14049211	14049213	TTA	TTA	T	INDEL	OS11G0434000	Intronic	NA	NA	LOC_Os11g24630	magnesium-dependent phosphatase 1, putative, expressed
Chr.11	14051188	14051189	TG	TG	T	INDEL	OS11G0434000	Intronic	NA	NA	LOC_Os11g24630	magnesium-dependent phosphatase 1, putative, expressed
Chr.11	16938235	16938239	TACTC	TACTC	T	INDEL	OS11G0482200	Intronic	NA	NA	LOC_Os11g29210	resistance-gene-interacting protein, putative, expressed
Chr.11	17236656	17236657	TA	TA	T	INDEL	OS11G0489250	Intronic	NA	NA	LOC_Os11g29720	cytochrome P450, putative, expressed
Chr.11	17353702	17353703	TC	TC	T	INDEL	OS11G0490900	Intronic	NA	NA	LOC_Os11g29870	WRKY72, expressed
Chr.11	17354722	17354724	TTA	TTA	T	INDEL	OS11G0490900	Intronic	NA	NA	LOC_Os11g29870	WRKY72, expressed
Chr.11	18451458	18451460	TAC	TAC	T	INDEL	OS11G0514800	Intronic	NA	NA	LOC_Os11g31570	expressed protein
Chr.11	18935566	18935568	TGA	TGA	T	INDEL	OS11G0523800	Exonic	3UTR	NA	LOC_Os11g32110	auxin response factor, putative, expressed
Chr.11	21300001	21300002	TA	TA	T	INDEL	OS11G0570000	Exonic	3UTR	NA	LOC_Os11g36200	receptor-like protein kinase 2 precursor, putative, expressed
Chr.11	22279216	22279226	TACTCTCTCCG	TACTCTCTCCG	T	INDEL	OS11G0587600	Intronic	NA	NA	LOC_Os11g37700	pleiotropic drug resistance protein, putative, expressed
Chr.10	18539265	18539265	C	C	T	SNP	OS10G0488800	Exonic	CDS	NA	LOC_Os10g34710	myosin-2 heavy chain, non muscle, putative, expressed
Chr.10	18538754	18538754	C	C	T	SNP	OS10G0488800	Exonic	CDS	NA	LOC_Os10g34710	myosin-2 heavy chain, non muscle, putative, expressed
Chr.10	10301819	10301819	C	C	T	SNP	OS10G0345100	Exonic	3UTR	NA	LOC_Os10g20470	MATE efflux family protein, putative, expressed
Chr.10	3756947	3756947	C	C	T	SNP	OS10G0159300	Intronic	NA	NA	LOC_Os10g07160	retrotransposon, putative, centromere-specific, expressed

Chr.10	10295536	10295547	TTCCTTTCATTG	TTCCTTTCATTG	T	INDEL	OS10G0344900	Intronic	NA	NA	LOC_Os10g20450	MATE efflux family protein, putative, expressed
Chr.10	6298591	6298591	G	G	T	SNP	OS10G0190900	Intronic	NA	NA	LOC_Os10g11354	MATE efflux family protein, putative, expressed
Chr.10	20843618	20843618	G	G	T	SNP	OS10G0535800	Intronic	NA	NA	LOC_Os10g39100	uncharacterized Cys-rich domain containing protein, putative, expressed
Chr.10	15640245	15640245	G	G	T	SNP	OS10G0437200	Exonic	3UTR	NA	LOC_Os10g30100	3-5 exonuclease family protein, putative, expressed
Chr.10	12023407	12023407	G	G	T	SNP	OS10G0377000	Intronic	NA	NA	LOC_Os10g23060	expressed protein
Chr.10	12017289	12017289	G	G	T	SNP	OS10G0376900	Intronic	NA	NA	LOC_Os10g23050	helix-loop-helix DNA-binding protein, putative, expressed
Chr.10	7687636	7687636	G	G	T	SNP	OS10G0208500	Exonic	5UTR	NA	LOC_Os10g14180	expressed protein
Chr.10	2360708	2360708	G	G	T	SNP	OS10G0138100	Intronic	NA	NA	LOC_Os10g04860	aldehyde oxidase, putative, expressed
Chr.10	1084943	1084943	G	G	T	SNP	OS10G0116800	Exonic	3UTR	NA	LOC_Os10g02750	Ser/Thr protein phosphatase family protein, putative, expressed
Chr.10	72785	72785	G	G	T	SNP	OS10G0100500	Exonic	3UTR	NA	LOC_Os10g01060	protein kinase family protein, putative, expressed
Chr.10	20211153	20211153	C	C	T	SNP	OS10G0522000	Exonic	3UTR	NA	LOC_Os10g37770	dehydration response related protein, putative, expressed
Chr.10	19607677	19607677	C	C	T	SNP	OS10G0510000	Intronic	NA	NA	LOC_Os10g36650	actin, putative, expressed
Chr.10	19172099	19172099	C	C	T	SNP	OS10G0502600	Exonic	CDS	Missense	LOC_Os10g35870	cytochrome b5-like Heme/Steroid binding domain containing protein, expressed
Chr.10	19094603	19094603	C	C	T	SNP	OS10G0500600	Intronic	NA	NA	LOC_Os10g35710	expressed protein
Chr.10	18989951	18989951	C	C	T	SNP	OS10G0498000	Exonic	CDS	Missense	LOC_Os10g35490	hydrolase, alpha/beta fold family domain containing protein, expressed
Chr.10	15677472	15677472	C	C	T	SNP	OS10G0437600	Intronic	NA	NA	LOC_Os10g30156	starch synthase, putative, expressed
Chr.10	15675647	15675647	C	C	T	SNP	OS10G0437600	Intronic	NA	NA	LOC_Os10g30156	starch synthase, putative, expressed
Chr.10	15642431	15642431	C	C	T	SNP	OS10G0437200	Intronic	NA	NA	LOC_Os10g30100	3-5 exonuclease family protein, putative, expressed
Chr.10	15642324	15642324	C	C	T	SNP	OS10G0437200	Intronic	NA	NA	LOC_Os10g30100	3-5 exonuclease family protein, putative, expressed
Chr.10	15405731	15405731	C	C	T	SNP	OS10G0431900	Exonic	5UTR	NA	LOC_Os10g29620	tyrosine protein kinase domain containing protein, putative, expressed
Chr.10	15308384	15308384	C	C	T	SNP	OS10G0430200	Intronic	NA	NA	LOC_Os10g29470	dehydrogenase, putative, expressed
Chr.10	15308121	15308121	C	C	T	SNP	OS10G0430200	Intronic	NA	NA	LOC_Os10g29470	dehydrogenase, putative, expressed
Chr.10	15306566	15306566	C	C	T	SNP	OS10G0430200	Intronic	NA	NA	LOC_Os10g29470	dehydrogenase, putative, expressed
Chr.10	12973451	12973451	C	C	T	SNP	OS10G0390500	Intronic	NA	NA	LOC_Os10g25130	aminotransferase, classes I and II, domain containing protein, expressed
Chr.10	12973397	12973397	C	C	T	SNP	OS10G0390500	Intronic	NA	NA	LOC_Os10g25130	aminotransferase, classes I and II, domain containing protein, expressed
Chr.10	12971265	12971265	C	C	T	SNP	OS10G0390500	Exonic	CDS	NA	LOC_Os10g25130	aminotransferase, classes I and II, domain containing protein, expressed
Chr.10	12970258	12970258	C	C	T	SNP	OS10G0390500	Intronic	NA	NA	LOC_Os10g25130	aminotransferase, classes I and II, domain containing protein, expressed
Chr.10	12968870	12968870	C	C	T	SNP	OS10G0390500	Intronic	NA	NA	LOC_Os10g25130	aminotransferase, classes I and II, domain containing protein, expressed
Chr.10	12869208	12869208	C	C	T	SNP	OS10G0388900	Intronic	NA	NA	LOC_Os10g25000	expressed protein
Chr.10	12017478	12017478	C	C	T	SNP	OS10G0376900	Exonic	3UTR	NA	LOC_Os10g23050	helix-loop-helix DNA-binding protein, putative, expressed
Chr.10	12017282	12017282	C	C	T	SNP	OS10G0376900	Intronic	NA	NA	LOC_Os10g23050	helix-loop-helix DNA-binding protein, putative, expressed
Chr.10	7689043	7689043	C	C	T	SNP	OS10G0208500	Exonic	5UTR	NA	LOC_Os10g14180	expressed protein
Chr.10	6300962	6300962	C	C	T	SNP	OS10G0190900	Intronic	NA	NA	LOC_Os10g11354	MATE efflux family protein, putative, expressed
Chr.10	6298744	6298744	C	C	T	SNP	OS10G0190900	Intronic	NA	NA	LOC_Os10g11354	MATE efflux family protein, putative, expressed
Chr.10	6298736	6298736	C	C	T	SNP	OS10G0190900	Intronic	NA	NA	LOC_Os10g11354	MATE efflux family protein, putative, expressed
Chr.10	6298587	6298587	C	C	T	SNP	OS10G0190900	Intronic	NA	NA	LOC_Os10g11354	MATE efflux family protein, putative, expressed
Chr.10	6298527	6298527	C	C	T	SNP	OS10G0190900	Intronic	NA	NA	LOC_Os10g11354	MATE efflux family protein, putative, expressed
Chr.10	6298075	6298075	C	C	T	SNP	OS10G0190900	Intronic	NA	NA	LOC_Os10g11354	MATE efflux family protein, putative, expressed
Chr.10	6272738	6272738	C	C	T	SNP	OS10G0190500	Exonic	3UTR	NA	LOC_Os10g11310	expressed protein
Chr.10	3756433	3756433	C	C	T	SNP	OS10G0159300	Intronic	NA	NA	LOC_Os10g07160	retrotransposon, putative, centromere-specific, expressed
Chr.10	3756067	3756067	C	C	T	SNP	OS10G0159300	Exonic	5UTR	NA	LOC_Os10g07160	retrotransposon, putative, centromere-specific, expressed
Chr.10	3755993	3755993	C	C	T	SNP	OS10G0159300	Exonic	5UTR	NA	LOC_Os10g07160	retrotransposon, putative, centromere-specific, expressed
Chr.10	3755796	3755796	C	C	T	SNP	OS10G0159300	Exonic	5UTR	NA	LOC_Os10g07160	retrotransposon, putative, centromere-specific, expressed
Chr.10	3755640	3755640	C	C	T	SNP	OS10G0159300	Exonic	5UTR	NA	LOC_Os10g07160	retrotransposon, putative, centromere-specific, expressed
Chr.10	3755572	3755572	C	C	T	SNP	OS10G0159300	Exonic	CDS	NA	LOC_Os10g07160	retrotransposon, putative, centromere-specific, expressed
Chr.10	3755314	3755314	C	C	T	SNP	OS10G0159300	Exonic	5UTR	NA	LOC_Os10g07160	retrotransposon, putative, centromere-specific, expressed
Chr.10	3754674	3754674	C	C	T	SNP	OS10G0159300	Exonic	3UTR	NA	LOC_Os10g07160	retrotransposon, putative, centromere-specific, expressed
Chr.10	2366090	2366090	C	C	T	SNP	OS10G0138100	Intronic	NA	NA	LOC_Os10g04860	aldehyde oxidase, putative, expressed
Chr.10	2366025	2366025	C	C	T	SNP	OS10G0138100	Intronic	NA	NA	LOC_Os10g04860	aldehyde oxidase, putative, expressed
Chr.10	2361612	2361612	C	C	T	SNP	OS10G0138100	Intronic	NA	NA	LOC_Os10g04860	aldehyde oxidase, putative, expressed
Chr.10	2360895	2360895	C	C	T	SNP	OS10G0138100	Intronic	NA	NA	LOC_Os10g04860	aldehyde oxidase, putative, expressed
Chr.10	2360891	2360891	C	C	T	SNP	OS10G0138100	Intronic	NA	NA	LOC_Os10g04860	aldehyde oxidase, putative, expressed
Chr.10	381167	381167	C	C	T	SNP	OS10G0104900	Intronic	NA	NA	LOC_Os10g01570	C-5 cytosine-specific DNA methylase, putative, expressed
Chr.10	379160	379160	C	C	T	SNP	OS10G0104900	Intronic	NA	NA	LOC_Os10g01570	C-5 cytosine-specific DNA methylase, putative, expressed
Chr.10	378996	378996	C	C	T	SNP	OS10G0104900	Intronic	NA	NA	LOC_Os10g01570	C-5 cytosine-specific DNA methylase, putative, expressed
Chr.10	71825	71825	C	C	T	SNP	OS10G0100500	Exonic	CDS	Silent	LOC_Os10g01060	protein kinase family protein, putative, expressed
Chr.10	6298590	6298590	A	A	T	SNP	OS10G0190900	Intronic	NA	NA	LOC_Os10g11354	MATE efflux family protein, putative, expressed
Chr.10	15308070	15308070	A	A	T	SNP	OS10G0430200	Intronic	NA	NA	LOC_Os10g29470	dehydrogenase, putative, expressed
Chr.10	15305391	15305391	A	A	T	SNP	OS10G0430200	Intronic	NA	NA	LOC_Os10g29470	dehydrogenase, putative, expressed
Chr.10	6298643	6298643	A	A	T	SNP	OS10G0190900	Intronic	NA	NA	LOC_Os10g11354	MATE efflux family protein, putative, expressed
Chr.10	6298611	6298611	A	A	T	SNP	OS10G0190900	Intronic	NA	NA	LOC_Os10g11354	MATE efflux family protein, putative, expressed
Chr.10	3754530	3754530	A	A	T	SNP	OS10G0159300	Exonic	3UTR	NA	LOC_Os10g07160	retrotransposon, putative, centromere-specific, expressed
Chr.10	72786	72786	A	A	T	SNP	OS10G0100500	Exonic	3UTR	NA	LOC_Os10g01060	protein kinase family protein, putative, expressed
Chr.10	69613	69613	A	A	T	SNP	OS10G0100500	Intronic	NA	NA	LOC_Os10g01060	protein kinase family protein, putative, expressed
Chr.10	71876	71876	C	C	T	SNP	OS10G0100500	Exonic	CDS	Silent	LOC_Os10g01060	protein kinase family protein, putative, expressed
Chr.10	69360	69360	C	C	T	SNP	OS10G0100500	Intronic	NA	NA	LOC_Os10g01060	protein kinase family protein, putative, expressed
Chr.10	81478	81478	C	C	T	SNP	OS10G0100700	Exonic	CDS	Silent	LOC_Os10g01080	SOR/SNZ family protein, putative, expressed
Chr.10	2367840	2367840	C	C	T	SNP	OS10G0138100	Exonic	3UTR	NA	LOC_Os10g04860	aldehyde oxidase, putative, expressed
Chr.10	2359003	2359003	C	C	T	SNP	OS10G0138100	Exonic	CDS	Silent	LOC_Os10g04860	aldehyde oxidase, putative, expressed
Chr.10	2365292	2365292	C	C	T	SNP	OS10G0138100	Exonic	CDS	Silent	LOC_Os10g04860	aldehyde oxidase, putative, expressed
Chr.10	2367670	2367670	C	C	T	SNP	OS10G0138100	Exonic	CDS	Silent	LOC_Os10g04860	aldehyde oxidase, putative, expressed
Chr.10	2365177	2365177	C	C	T	SNP	OS10G0138100	Exonic	CDS	Missense	LOC_Os10g04860	aldehyde oxidase, putative, expressed
Chr.10	2508116	2508116	C	C	T	SNP	OS10G0140700	Exonic	3UTR	NA	LOC_Os10g05130	expressed protein
Chr.10	2913664	2913664	C	C	T	SNP	OS10G0148400	Exonic	CDS	Missense	LOC_Os10g05780	POT domain containing peptide transporter, putative, expressed
Chr.10	2914009	2914009	C	C	T	SNP	OS10G0148400	Exonic	CDS	Missense	LOC_Os10g05780	POT domain containing peptide transporter, putative, expressed
Chr.10	3753507	3753507	C	C	T	SNP	OS10G0159300	Exonic	3UTR	NA	LOC_Os10g07160	retrotransposon, putative, centromere-specific, expressed
Chr.10	3753762	3753762	C	C	T	SNP	OS10G0159300	Exonic	3UTR	NA	LOC_Os10g07160	retrotransposon, putative, centromere-specific, expressed
Chr.10	3754134	3754134	C	C	T	SNP	OS10G0159300	Exonic	3UTR	NA	LOC_Os10g07160	retrotransposon, putative, centromere-specific, expressed
Chr.10	3756658	3756658	C	C	T	SNP	OS10G0159300	Intronic	NA	NA	LOC_Os10g07160	retrotransposon, putative, centromere-specific, expressed
Chr.10	6272697	6272697	C	C	T	SNP	OS10G0190500	Exonic	3UTR	NA	LOC_Os10g11310	expressed protein
Chr.10	10293298	10293298	C	C	T	SNP	OS10G0344900	Exonic	CDS	Missense	LOC_Os10g20450	MATE efflux family protein, putative, expressed
Chr.10	12016806	12016806	C	C	T	SNP	OS10G0376900	Exonic	CDS	Missense	LOC_Os10g23050	helix-loop-helix DNA-binding protein, putative, expressed
Chr.10	15308605	15308605	C	C	T	SNP	OS10G0430200	Intronic	NA	NA	LOC_Os10g29470	dehydrogenase, putative, expressed
Chr.10	15404209	15404209	C	C	T	SNP	OS10G0431900	Exonic	CDS	Silent	LOC_Os10g29620	tyrosine protein kinase domain containing protein, putative, expressed
Chr.10	15404014	15404014	A	A	T	SNP	OS10G0431900	Intronic	NA	NA	LOC_Os10g29620	tyrosine protein kinase domain containing protein, putative, expressed

Chr.10	15404352	15404352	C	C	T	SNP	OS10G0431900	Intronic	NA	NA	LOC_Os10g29620	tyrosine protein kinase domain containing protein, putative, expressed
Chr.10	15673341	15673341	C	C	T	SNP	OS10G0437600	Exonic	CDS	Missense	LOC_Os10g30156	starch synthase, putative, expressed
Chr.10	18638312	18638312	C	C	T	SNP	OS10G0490900	Exonic	CDS	NA	LOC_Os10g34920	secretory protein, putative, expressed
Chr.10	19362077	19362077	A	A	T	SNP	OS10G0506200	Intronic	NA	NA	LOC_Os10g36210	valyl-tRNA synthetase, putative, expressed
Chr.10	20211594	20211594	C	C	T	SNP	OS10G0522000	Intronic	NA	NA	LOC_Os10g37770	dehydration response related protein, putative, expressed
Chr.10	378721	378723	TTA	TTA	T	INDEL	OS10G0104900	Intronic	NA	NA	LOC_Os10g01570	C-5 cytosine-specific DNA methylase, putative, expressed
Chr.10	2359617	2359621	TAATA	TAATA	T	INDEL	OS10G0138100	Intronic	NA	NA	LOC_Os10g04860	aldehyde oxidase, putative, expressed
Chr.10	12017241	12017243	TTG	TTG	T	INDEL	OS10G0376900	Intronic	NA	NA	LOC_Os10g23050	helix-loop-helix DNA-binding protein, putative, expressed
Chr.10	12021759	12021777	TATATAGGCCGGTGATCAA	TATATAGGCCGGTGATCAA	T	INDEL	OS10G0377000	Exonic	5UTR	NA	LOC_Os10g23060	expressed protein
Chr.10	12970037	12970038	TG	TG	T	INDEL	OS10G0390500	Intronic	NA	NA	LOC_Os10g25130	aminotransferase, classes I and II, domain containing protein, expressed
Chr.10	12973307	12973308	TC	TC	T	INDEL	OS10G0390500	Intronic	NA	NA	LOC_Os10g25130	aminotransferase, classes I and II, domain containing protein, expressed
Chr.10	15308065	15308068	TTCA	TTCA	T	INDEL	OS10G0430200	Intronic	NA	NA	LOC_Os10g29470	dehydrogenase, putative, expressed
Chr.10	15308127	15308131	TTACG	TTACG	T	INDEL	OS10G0430200	Intronic	NA	NA	LOC_Os10g29470	dehydrogenase, putative, expressed
Chr.10	15308719	15308720	TG	TG	T	INDEL	OS10G0430200	Intronic	NA	NA	LOC_Os10g29470	dehydrogenase, putative, expressed
Chr.10	19359753	19359757	TTCTC	TTCTC	T	INDEL	OS10G0506200	Intronic	NA	NA	LOC_Os10g36210	valyl-tRNA synthetase, putative, expressed
Chr.10	19362681	19362683	TTA	TTA	T	INDEL	OS10G0506200	Intronic	NA	NA	LOC_Os10g36210	valyl-tRNA synthetase, putative, expressed
Chr.1	650496	650496	A	A	T	SNP	OS01G0112500	Intronic	NA	NA	LOC_Os01g02200	armadillo/beta-catenin repeat family protein, putative, expressed
Chr.1	650680	650680	C	C	T	SNP	OS01G0112500	Intronic	NA	NA	LOC_Os01g02200	armadillo/beta-catenin repeat family protein, putative, expressed
Chr.1	650919	650919	C	C	T	SNP	OS01G0112500	Intronic	NA	NA	LOC_Os01g02200	armadillo/beta-catenin repeat family protein, putative, expressed
Chr.1	744577	744577	A	A	T	SNP	OS01G0113700	Exonic	CDS	Missense	LOC_Os01g02350	receptor-like kinase ARK1AS, putative, expressed
Chr.1	744627	744627	C	C	T	SNP	OS01G0113700	Exonic	CDS	Missense	LOC_Os01g02350	receptor-like kinase ARK1AS, putative, expressed
Chr.1	744793	744793	C	C	T	SNP	OS01G0113700	Exonic	CDS	Missense	LOC_Os01g02350	receptor-like kinase ARK1AS, putative, expressed
Chr.1	744829	744829	C	C	T	SNP	OS01G0113700	Exonic	CDS	Missense	LOC_Os01g02350	receptor-like kinase ARK1AS, putative, expressed
Chr.1	744837	744837	A	A	T	SNP	OS01G0113700	Exonic	CDS	Missense	LOC_Os01g02350	receptor-like kinase ARK1AS, putative, expressed
Chr.1	744361	744361	G	G	T	SNP	OS01G0113700	Intronic	NA	NA	LOC_Os01g02350	receptor-like kinase ARK1AS, putative, expressed
Chr.1	9225756	9225756	A	A	T	SNP	OS01G0268300	Intronic	NA	NA	LOC_Os01g16290	DNA gyrase subunit B, chloroplast/mitochondrial precursor, putative, expressed
Chr.1	9227473	9227473	A	A	T	SNP	OS01G0268300	Intronic	NA	NA	LOC_Os01g16290	DNA gyrase subunit B, chloroplast/mitochondrial precursor, putative, expressed
Chr.1	9228008	9228008	C	C	T	SNP	OS01G0268300	Intronic	NA	NA	LOC_Os01g16290	DNA gyrase subunit B, chloroplast/mitochondrial precursor, putative, expressed
Chr.1	9231726	9231726	C	C	T	SNP	OS01G0268300	Intronic	NA	NA	LOC_Os01g16290	DNA gyrase subunit B, chloroplast/mitochondrial precursor, putative, expressed
Chr.1	9232881	9232881	C	C	T	SNP	OS01G0268300	Intronic	NA	NA	LOC_Os01g16290	DNA gyrase subunit B, chloroplast/mitochondrial precursor, putative, expressed
Chr.1	9234865	9234865	G	G	T	SNP	OS01G0268300	Intronic	NA	NA	LOC_Os01g16290	DNA gyrase subunit B, chloroplast/mitochondrial precursor, putative, expressed
Chr.1	9859926	9859926	C	C	T	SNP	OS01G0278900	Intronic	NA	NA	LOC_Os01g17150	plastid-specific 50S ribosomal protein 5, chloroplast precursor, putative, expressed
Chr.1	10010730	10010730	A	A	T	SNP	OS01G0281100	Intronic	NA	NA	LOC_Os01g17396	expressed protein
Chr.1	10011050	10011050	C	C	T	SNP	OS01G0281100	Intronic	NA	NA	LOC_Os01g17396	expressed protein
Chr.1	10102477	10102477	C	C	T	SNP	OS01G0282800	Intronic	NA	NA	LOC_Os01g18050	tubulin/FtsZ domain containing protein, putative, expressed
Chr.1	10202238	10202238	A	A	T	SNP	OS01G0284900	Intronic	NA	NA	LOC_Os01g18220	expressed protein
Chr.1	10342605	10342605	C	C	T	SNP	OS01G0287600	Intronic	NA	NA	LOC_Os01g18400	CHIT10 - Chitinase family protein precursor, expressed
Chr.1	10342781	10342781	C	C	T	SNP	OS01G0287600	Intronic	NA	NA	LOC_Os01g18400	CHIT10 - Chitinase family protein precursor, expressed
Chr.1	10342835	10342835	G	G	T	SNP	OS01G0287600	Intronic	NA	NA	LOC_Os01g18400	CHIT10 - Chitinase family protein precursor, expressed
Chr.1	10342842	10342842	C	C	T	SNP	OS01G0287600	Intronic	NA	NA	LOC_Os01g18400	CHIT10 - Chitinase family protein precursor, expressed
Chr.1	10342856	10342856	C	C	T	SNP	OS01G0287600	Intronic	NA	NA	LOC_Os01g18400	CHIT10 - Chitinase family protein precursor, expressed
Chr.1	10493764	10493764	A	A	T	SNP	OS01G0290000	Intronic	NA	NA	LOC_Os01g18630	aspartic proteinase oryzasin-1 precursor, putative, expressed
Chr.1	10652671	10652671	C	C	T	SNP	OS01G0292900	Intronic	NA	NA	LOC_Os01g18850	OsSPL1 - SBP-box gene family member, expressed
Chr.1	15294119	15294119	C	C	T	SNP	OS01G0371500	Exonic	CDS	Silent	LOC_Os01g27390	glutathione S-transferase, putative, expressed
Chr.1	15294116	15294116	A	A	T	SNP	OS01G0371500	Exonic	CDS	Missense	LOC_Os01g27390	glutathione S-transferase, putative, expressed
Chr.1	15292080	15292080	A	A	T	SNP	OS01G0371500	Intronic	NA	NA	LOC_Os01g27390	glutathione S-transferase, putative, expressed
Chr.1	15292096	15292096	G	G	T	SNP	OS01G0371500	Intronic	NA	NA	LOC_Os01g27390	glutathione S-transferase, putative, expressed
Chr.1	15292309	15292309	C	C	T	SNP	OS01G0371500	Intronic	NA	NA	LOC_Os01g27390	glutathione S-transferase, putative, expressed
Chr.1	15292480	15292480	A	A	T	SNP	OS01G0371500	Intronic	NA	NA	LOC_Os01g27390	glutathione S-transferase, putative, expressed
Chr.1	15292632	15292632	C	C	T	SNP	OS01G0371500	Intronic	NA	NA	LOC_Os01g27390	glutathione S-transferase, putative, expressed
Chr.1	15292870	15292870	A	A	T	SNP	OS01G0371500	Intronic	NA	NA	LOC_Os01g27390	glutathione S-transferase, putative, expressed
Chr.1	15293299	15293299	A	A	T	SNP	OS01G0371500	Intronic	NA	NA	LOC_Os01g27390	glutathione S-transferase, putative, expressed
Chr.1	15293428	15293428	A	A	T	SNP	OS01G0371500	Intronic	NA	NA	LOC_Os01g27390	glutathione S-transferase, putative, expressed
Chr.1	15293484	15293484	C	C	T	SNP	OS01G0371500	Intronic	NA	NA	LOC_Os01g27390	glutathione S-transferase, putative, expressed
Chr.1	20574054	20574054	A	A	T	SNP	OS01G0550000	Exonic	5UTR	NA	LOC_Os01g36920	DEAD-box ATP-dependent RNA helicase, putative, expressed
Chr.1	20573316	20573316	C	C	T	SNP	OS01G0550000	Exonic	CDS	Silent	LOC_Os01g36920	DEAD-box ATP-dependent RNA helicase, putative, expressed
Chr.1	20575859	20575859	G	G	T	SNP	OS01G0550000	Exonic	CDS	Silent	LOC_Os01g36920	DEAD-box ATP-dependent RNA helicase, putative, expressed
Chr.1	20572041	20572041	A	A	T	SNP	OS01G0550000	Intronic	NA	NA	LOC_Os01g36920	DEAD-box ATP-dependent RNA helicase, putative, expressed
Chr.1	20572049	20572049	A	A	T	SNP	OS01G0550000	Intronic	NA	NA	LOC_Os01g36920	DEAD-box ATP-dependent RNA helicase, putative, expressed
Chr.1	20572313	20572313	G	G	T	SNP	OS01G0550000	Intronic	NA	NA	LOC_Os01g36920	DEAD-box ATP-dependent RNA helicase, putative, expressed
Chr.1	20586710	20586710	G	G	T	SNP	OS01G0550100	Intronic	NA	NA	LOC_Os01g36930	ubiquitin carboxyl-terminal hydrolase 6, putative, expressed
Chr.1	20588419	20588419	A	A	T	SNP	OS01G0550100	Intronic	NA	NA	LOC_Os01g36930	ubiquitin carboxyl-terminal hydrolase 6, putative, expressed
Chr.1	20588462	20588462	C	C	T	SNP	OS01G0550100	Intronic	NA	NA	LOC_Os01g36930	ubiquitin carboxyl-terminal hydrolase 6, putative, expressed
Chr.1	20588587	20588587	C	C	T	SNP	OS01G0550100	Intronic	NA	NA	LOC_Os01g36930	ubiquitin carboxyl-terminal hydrolase 6, putative, expressed
Chr.1	20589172	20589172	C	C	T	SNP	OS01G0550100	Intronic	NA	NA	LOC_Os01g36930	ubiquitin carboxyl-terminal hydrolase 6, putative, expressed
Chr.1	20601676	20601676	C	C	T	SNP	OS01G0550300	Exonic	CDS	Silent	LOC_Os01g36950	N-rich protein, putative, expressed
Chr.1	21125640	21125640	C	C	T	SNP	OS01G0558300	Intronic	NA	NA	LOC_Os01g37770	RWD domain containing protein, expressed
Chr.1	21125856	21125856	A	A	T	SNP	OS01G0558300	Intronic	NA	NA	LOC_Os01g37770	RWD domain containing protein, expressed
Chr.1	21126333	21126333	C	C	T	SNP	OS01G0558300	Intronic	NA	NA	LOC_Os01g37770	RWD domain containing protein, expressed
Chr.1	21126635	21126635	C	C	T	SNP	OS01G0558300	Intronic	NA	NA	LOC_Os01g37770	RWD domain containing protein, expressed
Chr.1	21126655	21126655	C	C	T	SNP	OS01G0558300	Intronic	NA	NA	LOC_Os01g37770	RWD domain containing protein, expressed
Chr.1	21127470	21127470	C	C	T	SNP	OS01G0558300	Intronic	NA	NA	LOC_Os01g37770	RWD domain containing protein, expressed
Chr.1	29491740	29491740	C	C	T	SNP	OS01G0709500	Exonic	5UTR	NA	LOC_Os01g51290	protein kinase family protein, putative, expressed
Chr.1	29484636	29484636	C	C	T	SNP	OS01G0709500	Intronic	NA	NA	LOC_Os01g51290	protein kinase family protein, putative, expressed
Chr.1	29489525	29489525	A	A	T	SNP	OS01G0709500	Intronic	NA	NA	LOC_Os01g51290	protein kinase family protein, putative, expressed
Chr.1	29489526	29489526	A	A	T	SNP	OS01G0709500	Intronic	NA	NA	LOC_Os01g51290	protein kinase family protein, putative, expressed
Chr.1	29895652	29895652	C	C	T	SNP	OS01G0717700	Intronic	NA	NA	LOC_Os01g52010	alliin lyase precursor, putative, expressed
Chr.1	31836086	31836086	A	A	T	SNP	OS01G0757900	Exonic	3UTR	NA	LOC_Os01g55310	haloacid dehalogenase-like hydrolase domain-containing protein 1A, putative, expressed
Chr.1	41496947	41496947	G	G	T	SNP	OS01G0944100	Exonic	3UTR	NA	LOC_Os01g71624	expressed protein
Chr.1	41503219	41503219	C	C	T	SNP	OS01G0944100	Exonic	3UTR	NA	LOC_Os01g71624	expressed protein
Chr.1	41503546	41503546	G	G	T	SNP	OS01G0944100	Exonic	3UTR	NA	LOC_Os01g71624	expressed protein
Chr.1	41504692	41504692	C	C	T	SNP	OS01G0944100	Exonic	3UTR	NA	LOC_Os01g71624	expressed protein
Chr.1	41497383	41497383	C	C	T	SNP	OS01G0944100	Intronic	NA	NA	LOC_Os01g71624	expressed protein
Chr.1	41498280	41498280	G	G	T	SNP	OS01G0944100	Intronic	NA	NA	LOC_Os01g71624	expressed protein
Chr.1	41498638	41498638	G	G	T	SNP	OS01G0944100	Intronic	NA	NA	LOC_Os01g71624	expressed protein

Chr.1	41500726	41500726	A	A	T	SNP	OS01G0944100	Intronic	NA	NA	LOC_Os01g71624	expressed protein
Chr.1	21125849	21125855	TATAAGA	TATAAGA	T	INDEL	OS01G0558300	Intronic	NA	NA	LOC_Os01g37770	RWD domain containing protein, expressed
Chr.1	29846698	29846699	TA	TA	T	INDEL	OS01G0717000	Exonic	5UTR	NA	LOC_Os01g51920	phosphotransferase, putative, expressed
Chr.1	29896283	29896284	TA	TA	T	INDEL	OS01G0717700	Intronic	NA	NA	LOC_Os01g52010	alliin lyase precursor, putative, expressed
Chr.1	261979	261979	G	G	T	SNP	OS01G0104800	Intronic	NA	NA	LOC_Os01g01510	sas10/Utp3 family protein, expressed
Chr.1	262363	262363	G	G	T	SNP	OS01G0104800	Intronic	NA	NA	LOC_Os01g01510	sas10/Utp3 family protein, expressed
Chr.1	604335	604335	G	G	T	SNP	OS01G0111500	Exonic	CDS	Silent	LOC_Os01g02110	helix-loop-helix DNA-binding domain containing protein, expressed
Chr.1	604189	604189	G	G	T	SNP	OS01G0111500	Intronic	NA	NA	LOC_Os01g02110	helix-loop-helix DNA-binding domain containing protein, expressed
Chr.1	7029257	7029257	G	G	T	SNP	OS01G0227100	Intronic	NA	NA	LOC_Os01g12710	oxidoreductase, short chain dehydrogenase/reductase family domain containing protein, expressed
Chr.1	7279781	7279781	G	G	T	SNP	OS01G0231700	Intronic	NA	NA	LOC_Os01g13080	60S acidic ribosomal protein, putative, expressed
Chr.1	9116152	9116152	G	G	T	SNP	OS01G0266500	Intronic	NA	NA	LOC_Os01g16146	phenazine biosynthesis protein, putative, expressed
Chr.1	15292699	15292699	G	G	T	SNP	OS01G0371500	Exonic	CDS	Silent	LOC_Os01g27390	glutathione S-transferase, putative, expressed
Chr.1	18127535	18127535	G	G	T	SNP	OS01G0513400	Intronic	NA	NA	LOC_Os01g33000	expressed protein
Chr.1	20740788	20740788	G	G	T	SNP	OS01G0552300	Intronic	NA	NA	LOC_Os01g37130	protein phosphatase 2C, putative, expressed
Chr.1	21637745	21637745	G	G	T	SNP	OS01G0566050	Exonic	CDS	NA	LOC_Os01g38530	ELF3 protein, putative, expressed
Chr.1	24078117	24078117	G	G	T	SNP	OS01G0609300	Exonic	CDS	NA	LOC_Os01g42380	pleiotropic drug resistance protein, putative, expressed
Chr.1	27009040	27009040	G	G	T	SNP	OS01G0661500	Intronic	NA	NA	LOC_Os01g47262	AMSH-like protease, putative, expressed
Chr.1	27083808	27083808	G	G	T	SNP	OS01G0663300	Intronic	NA	NA	LOC_Os01g47400	OsMan01 - Endo-Beta-Mannanase, expressed
Chr.1	27084261	27084261	G	G	T	SNP	OS01G0663300	Intronic	NA	NA	LOC_Os01g47400	OsMan01 - Endo-Beta-Mannanase, expressed
Chr.1	27102987	27102987	G	G	T	SNP	OS01G0663800	Exonic	CDS	Silent	LOC_Os01g47430	protein of unknown function DUF1296 domain containing protein, expressed
Chr.1	27172151	27172151	G	G	T	SNP	OS01G0665200	Intronic	NA	NA	LOC_Os01g47530	CGMC_MAPKCMGC_2.6 - CGMC includes CDA, MAPK, GSK3, and CLKC kinases, expressed
Chr.1	27172845	27172845	G	G	T	SNP	OS01G0665200	Intronic	NA	NA	LOC_Os01g47530	CGMC_MAPKCMGC_2.6 - CGMC includes CDA, MAPK, GSK3, and CLKC kinases, expressed
Chr.1	27178707	27178707	G	G	T	SNP	OS01G0665300	Exonic	3UTR	NA	LOC_Os01g47540	NLI interacting factor-like phosphatase, putative, expressed
Chr.1	29844417	29844417	G	G	T	SNP	OS01G0717000	Intronic	NA	NA	LOC_Os01g51920	phosphotransferase, putative, expressed
Chr.1	29896409	29896409	G	G	T	SNP	OS01G0717700	Intronic	NA	NA	LOC_Os01g52010	alliin lyase precursor, putative, expressed
Chr.1	30158683	30158683	G	G	T	SNP	OS01G0723200	Intronic	NA	NA	LOC_Os01g52490	40S ribosomal protein S24, putative, expressed
Chr.1	30158770	30158770	G	G	T	SNP	OS01G0723200	Intronic	NA	NA	LOC_Os01g52490	40S ribosomal protein S24, putative, expressed
Chr.1	30158772	30158772	G	G	T	SNP	OS01G0723200	Intronic	NA	NA	LOC_Os01g52490	40S ribosomal protein S24, putative, expressed
Chr.1	30159188	30159188	G	G	T	SNP	OS01G0723200	Intronic	NA	NA	LOC_Os01g52490	40S ribosomal protein S24, putative, expressed
Chr.1	31979738	31979738	G	G	T	SNP	OS01G0759900	Exonic	CDS	NA	LOC_Os01g55500	nucleobase-ascorbate transporter, putative, expressed
Chr.1	32003135	32003135	G	G	T	SNP	OS01G0760600	Intronic	NA	NA	LOC_Os01g55540	aminotransferase, classes I and II, domain containing protein, expressed
Chr.1	33075054	33075054	G	G	T	SNP	OS01G0780800	Intronic	NA	NA	LOC_Os01g57240	expressed protein
Chr.1	34550497	34550497	G	G	T	SNP	OS01G0812800	Exonic	3UTR	NA	LOC_Os01g59730	ribosomal protein L7Ae, putative, expressed
Chr.1	34550640	34550640	G	G	T	SNP	OS01G0812800	Exonic	3UTR	NA	LOC_Os01g59730	ribosomal protein L7Ae, putative, expressed
Chr.1	34551159	34551159	G	G	T	SNP	OS01G0812800	Intronic	NA	NA	LOC_Os01g59730	ribosomal protein L7Ae, putative, expressed
Chr.1	35232775	35232775	G	G	T	SNP	OS01G0824600	Intronic	NA	NA	LOC_Os01g60910	CAMK_KIN1/SNF1/Nim1_like.12 - CAMK includes calcium/calmodulin depedent protein kinases, expressed
Chr.1	39565813	39565813	G	G	T	SNP	OS01G0908700	Exonic	3UTR	NA	LOC_Os01g68060	copine, putative, expressed
Chr.1	39691869	39691869	G	G	T	SNP	OS01G0911100	Exonic	CDS	Silent	LOC_Os01g68320	DEAD-box ATP-dependent RNA helicase 30, putative, expressed
Chr.1	264797	264797	C	C	T	SNP	OS01G0104800	Intronic	NA	NA	LOC_Os01g01510	sas10/Utp3 family protein, expressed
Chr.1	266940	266940	C	C	T	SNP	OS01G0104800	Intronic	NA	NA	LOC_Os01g01510	sas10/Utp3 family protein, expressed
Chr.1	266970	266970	C	C	T	SNP	OS01G0104800	Intronic	NA	NA	LOC_Os01g01510	sas10/Utp3 family protein, expressed
Chr.1	603675	603675	C	C	T	SNP	OS01G0111500	Exonic	3UTR	NA	LOC_Os01g02110	helix-loop-helix DNA-binding domain containing protein, expressed
Chr.1	603863	603863	C	C	T	SNP	OS01G0111500	Exonic	3UTR	NA	LOC_Os01g02110	helix-loop-helix DNA-binding domain containing protein, expressed
Chr.1	603925	603925	C	C	T	SNP	OS01G0111500	Exonic	3UTR	NA	LOC_Os01g02110	helix-loop-helix DNA-binding domain containing protein, expressed
Chr.1	605624	605624	C	C	T	SNP	OS01G0111500	Exonic	5UTR	NA	LOC_Os01g02110	helix-loop-helix DNA-binding domain containing protein, expressed
Chr.1	604150	604150	C	C	T	SNP	OS01G0111500	Intronic	NA	NA	LOC_Os01g02110	helix-loop-helix DNA-binding domain containing protein, expressed
Chr.1	604381	604381	C	C	T	SNP	OS01G0111500	Intronic	NA	NA	LOC_Os01g02110	helix-loop-helix DNA-binding domain containing protein, expressed
Chr.1	604479	604479	C	C	T	SNP	OS01G0111500	Intronic	NA	NA	LOC_Os01g02110	helix-loop-helix DNA-binding domain containing protein, expressed
Chr.1	720950	720950	C	C	T	SNP	OS01G0113200	Exonic	3UTR	NA	LOC_Os01g02290	Ser/Thr receptor-like kinase, putative, expressed
Chr.1	2357962	2357962	C	C	T	SNP	OS01G0143300	Exonic	3UTR	NA	LOC_Os01g05010	mitochondrial glycoprotein, putative, expressed
Chr.1	6565572	6565572	C	C	T	SNP	OS01G0220100	Intronic	NA	NA	LOC_Os01g12070	endoglucanase precursor, putative, expressed
Chr.1	6766748	6766748	C	C	T	SNP	OS01G0223600	Exonic	3UTR	NA	LOC_Os01g12390	inactive receptor kinase At2g26730 precursor, putative, expressed
Chr.1	7029477	7029477	C	C	T	SNP	OS01G0227100	Intronic	NA	NA	LOC_Os01g12710	oxidoreductase, short chain dehydrogenase/reductase family domain containing protein, expressed
Chr.1	7550453	7550453	C	C	T	SNP	OS01G0236300	Intronic	NA	NA	LOC_Os01g13520	auxin response factor 1, putative, expressed
Chr.1	8206954	8206954	C	C	T	SNP	OS01G0249200	Exonic	5UTR	NA	LOC_Os01g14670	Cupin domain containing protein, expressed
Chr.1	9234589	9234589	C	C	T	SNP	OS01G0268300	Exonic	CDS	Silent	LOC_Os01g16290	DNA gyrase subunit B, chloroplast/mitochondrial precursor, putative, expressed
Chr.1	9672157	9672157	C	C	T	SNP	OS01G0276200	Intronic	NA	NA	LOC_Os01g16910	outer mitochondrial membrane porin, putative, expressed
Chr.1	9674045	9674045	C	C	T	SNP	OS01G0276200	Intronic	NA	NA	LOC_Os01g16910	outer mitochondrial membrane porin, putative, expressed
Chr.1	13904594	13904594	C	C	T	SNP	OS01G0348900	Exonic	5UTR	NA	LOC_Os01g24710	jacalin-like lectin domain containing protein, expressed
Chr.1	18127820	18127820	C	C	T	SNP	OS01G0513400	Intronic	NA	NA	LOC_Os01g33000	expressed protein
Chr.1	18127907	18127907	C	C	T	SNP	OS01G0513400	Intronic	NA	NA	LOC_Os01g33000	expressed protein
Chr.1	20742426	20742426	C	C	T	SNP	OS01G0552300	Exonic	3UTR	NA	LOC_Os01g37130	protein phosphatase 2C, putative, expressed
Chr.1	20740853	20740853	C	C	T	SNP	OS01G0552300	Intronic	NA	NA	LOC_Os01g37130	protein phosphatase 2C, putative, expressed
Chr.1	20740992	20740992	C	C	T	SNP	OS01G0552300	Intronic	NA	NA	LOC_Os01g37130	protein phosphatase 2C, putative, expressed
Chr.1	20742026	20742026	C	C	T	SNP	OS01G0552300	Intronic	NA	NA	LOC_Os01g37130	protein phosphatase 2C, putative, expressed
Chr.1	21638100	21638100	C	C	T	SNP	OS01G0566050	Exonic	CDS	NA	LOC_Os01g38530	ELF3 protein, putative, expressed
Chr.1	23344512	23344512	C	C	T	SNP	OS01G0595600	Intronic	NA	NA	LOC_Os01g41240	hydrolase, alpha/beta fold family domain containing protein, expressed
Chr.1	23673260	23673260	C	C	T	SNP	OS01G0602400	Exonic	3UTR	NA	LOC_Os01g41810	cytochrome P450 72A1, putative, expressed
Chr.1	23673841	23673841	C	C	T	SNP	OS01G0602400	Exonic	CDS	Silent	LOC_Os01g41810	cytochrome P450 72A1, putative, expressed
Chr.1	23674153	23674153	C	C	T	SNP	OS01G0602400	Exonic	CDS	Silent	LOC_Os01g41810	cytochrome P450 72A1, putative, expressed
Chr.1	23674057	23674057	C	C	T	SNP	OS01G0602400	Exonic	CDS	Missense	LOC_Os01g41810	cytochrome P450 72A1, putative, expressed
Chr.1	23674134	23674134	C	C	T	SNP	OS01G0602400	Exonic	CDS	Missense	LOC_Os01g41810	cytochrome P450 72A1, putative, expressed
Chr.1	24601640	24601640	C	C	T	SNP	OS01G0618400	Exonic	3UTR	NA	LOC_Os01g43120	DEAD/DEAH box helicase, putative, expressed
Chr.1	24784622	24784622	C	C	T	SNP	OS01G0621600	Exonic	3UTR	NA	LOC_Os01g43350	protein kinase family protein, putative, expressed
Chr.1	27083465	27083465	C	C	T	SNP	OS01G0663300	Intronic	NA	NA	LOC_Os01g47400	OsMan01 - Endo-Beta-Mannanase, expressed
Chr.1	27084177	27084177	C	C	T	SNP	OS01G0663300	Intronic	NA	NA	LOC_Os01g47400	OsMan01 - Endo-Beta-Mannanase, expressed
Chr.1	27084201	27084201	C	C	T	SNP	OS01G0663300	Intronic	NA	NA	LOC_Os01g47400	OsMan01 - Endo-Beta-Mannanase, expressed
Chr.1	27102265	27102265	C	C	T	SNP	OS01G0663800	Exonic	3UTR	NA	LOC_Os01g47430	protein of unknown function DUF1296 domain containing protein, expressed
Chr.1	27108786	27108786	C	C	T	SNP	OS01G0663800	Intronic	NA	NA	LOC_Os01g47430	protein of unknown function DUF1296 domain containing protein, expressed
Chr.1	29075117	29075117	C	C	T	SNP	OS01G0702000	Intronic	NA	NA	LOC_Os01g50622	DNA-binding protein DSP1, putative, expressed
Chr.1	29831271	29831271	C	C	T	SNP	OS01G0716800	Intronic	NA	NA	LOC_Os01g51890	endonuclease/exonuclease/phosphatase family domain containing protein, expressed
Chr.1	29832291	29832291	C	C	T	SNP	OS01G0716800	Intronic	NA	NA	LOC_Os01g51890	endonuclease/exonuclease/phosphatase family domain containing protein, expressed
Chr.1	29842922	29842922	C	C	T	SNP	OS01G0717000	Exonic	3UTR	NA	LOC_Os01g51920	phosphotransferase, putative, expressed
Chr.1	29969908	29969908	C	C	T	SNP	OS01G0719100	Exonic	CDS	NA	LOC_Os01g52110	RING finger and CHY zinc finger domain-containing protein 1, putative, expressed
Chr.1	30158049	30158049	C	C	T	SNP	OS01G0723200	Exonic	3UTR	NA	LOC_Os01g52490	40S ribosomal protein S24, putative, expressed

Chr.1	30158593	30158593	C	C	T	SNP	OS01G0723200	Intronic	NA	NA	LOC_Os01g52490	40S ribosomal protein S24, putative, expressed
Chr.1	30183701	30183701	C	C	T	SNP	OS01G0723600	Intronic	NA	NA	LOC_Os01g52530	phosphoribosyl transferase, putative, expressed
Chr.1	30183773	30183773	C	C	T	SNP	OS01G0723600	Intronic	NA	NA	LOC_Os01g52530	phosphoribosyl transferase, putative, expressed
Chr.1	32136530	32136530	C	C	T	SNP	OS01G0763600	Exonic	CDS	Silent	LOC_Os01g55780	glycerophosphoryl diester phosphodiesterase family protein, putative, expressed
Chr.1	34550665	34550665	C	C	T	SNP	OS01G0812800	Exonic	CDS	Silent	LOC_Os01g59730	ribosomal protein L7Ae, putative, expressed
Chr.1	34613258	34613258	C	C	T	SNP	OS01G0813900	Intronic	NA	NA	LOC_Os01g59850	GTPase-activating protein, putative, expressed
Chr.1	34614768	34614768	C	C	T	SNP	OS01G0813900	Intronic	NA	NA	LOC_Os01g59850	GTPase-activating protein, putative, expressed
Chr.1	34617633	34617633	C	C	T	SNP	OS01G0813900	Intronic	NA	NA	LOC_Os01g59850	GTPase-activating protein, putative, expressed
Chr.1	35229707	35229707	C	C	T	SNP	OS01G0824600	Exonic	5UTR	NA	LOC_Os01g60910	CAMK_KIN1/SNF1/Nim1_like.12 - CAMK includes calcium/calmodulin depedent protein kinases, expressed
Chr.1	35232970	35232970	C	C	T	SNP	OS01G0824600	Intronic	NA	NA	LOC_Os01g60910	CAMK_KIN1/SNF1/Nim1_like.12 - CAMK includes calcium/calmodulin depedent protein kinases, expressed
Chr.1	36483628	36483628	C	C	T	SNP	OS01G0848900	Exonic	3UTR	NA	LOC_Os01g62970	expressed protein
Chr.1	36796900	36796900	C	C	T	SNP	OS01G0854000	Exonic	3UTR	NA	LOC_Os01g63480	transferase family protein, putative, expressed
Chr.1	38483533	38483533	C	C	T	SNP	OS01G0885700	Exonic	CDS	Missense	LOC_Os01g66250	S-locus-like receptor protein kinase, putative, expressed
Chr.1	39434177	39434177	C	C	T	SNP	OS01G0905800	Exonic	CDS	Missense	LOC_Os01g67860	fructose-bisphospate aldolase isozyme, putative, expressed
Chr.1	39567071	39567071	C	C	T	SNP	OS01G0908700	Exonic	CDS	Silent	LOC_Os01g68060	copine, putative, expressed
Chr.1	39689925	39689925	C	C	T	SNP	OS01G0911100	Exonic	3UTR	NA	LOC_Os01g68320	DEAD-box ATP-dependent RNA helicase 30, putative, expressed
Chr.1	41005354	41005354	C	C	T	SNP	OS01G0934800	Intronic	NA	NA	LOC_Os01g70850	esterase, putative, expressed
Chr.1	43074818	43074818	C	C	T	SNP	OS01G0974800	Exonic	5UTR	NA	LOC_Os01g74350	soluble inorganic pyrophosphatase, putative, expressed
Chr.1	10655839	10655839	A	A	T	SNP	OS01G0292900	Intronic	NA	NA	LOC_Os01g18850	OsSPL1 - SBP-box gene family member, expressed
Chr.1	605635	605635	A	A	T	SNP	OS01G0111500	Exonic	5UTR	NA	LOC_Os01g02110	helix-loop-helix DNA-binding domain containing protein, expressed
Chr.1	3821780	3821780	A	A	T	SNP	OS01G0174300	Intronic	NA	NA	LOC_Os01g07910	NADH-cytochrome b5 reductase, putative, expressed
Chr.1	7028606	7028606	A	A	T	SNP	OS01G0227100	Intronic	NA	NA	LOC_Os01g12710	oxidoreductase, short chain dehydrogenase/reductase family domain containing protein, expressed
Chr.1	7459170	7459170	A	A	T	SNP	OS01G0235100	Intronic	NA	NA	LOC_Os01g13390	expressed protein
Chr.1	7459223	7459223	A	A	T	SNP	OS01G0235100	Intronic	NA	NA	LOC_Os01g13390	expressed protein
Chr.1	14513946	14513946	A	A	T	SNP	OS01G0358400	Intronic	NA	NA	LOC_Os01g25610	40S ribosomal protein S4, putative, expressed
Chr.1	20739852	20739852	A	A	T	SNP	OS01G0552300	Intronic	NA	NA	LOC_Os01g37130	protein phosphatase 2C, putative, expressed
Chr.1	21632976	21632976	A	A	T	SNP	OS01G0565900	Exonic	3UTR	NA	LOC_Os01g38510	protein transport protein Sec61 subunit beta, putative, expressed
Chr.1	21632977	21632977	A	A	T	SNP	OS01G0565900	Exonic	3UTR	NA	LOC_Os01g38510	protein transport protein Sec61 subunit beta, putative, expressed
Chr.1	21633068	21633068	A	A	T	SNP	OS01G0565900	Exonic	3UTR	NA	LOC_Os01g38510	protein transport protein Sec61 subunit beta, putative, expressed
Chr.1	23675954	23675954	A	A	T	SNP	OS01G0602400	Exonic	5UTR	NA	LOC_Os01g41810	cytochrome P450 72A1, putative, expressed
Chr.1	24075193	24075193	A	A	T	SNP	OS01G0609300	Exonic	3UTR	NA	LOC_Os01g42380	pleiotropic drug resistance protein, putative, expressed
Chr.1	25055942	25055942	A	A	T	SNP	OS01G0627600	Exonic	3UTR	NA	LOC_Os01g43720	cytochrome P450 72A1, putative, expressed
Chr.1	30157993	30157993	A	A	T	SNP	OS01G0723200	Exonic	3UTR	NA	LOC_Os01g52490	40S ribosomal protein S24, putative, expressed
Chr.1	30158496	30158496	A	A	T	SNP	OS01G0723200	Intronic	NA	NA	LOC_Os01g52490	40S ribosomal protein S24, putative, expressed
Chr.1	30158550	30158550	A	A	T	SNP	OS01G0723200	Intronic	NA	NA	LOC_Os01g52490	40S ribosomal protein S24, putative, expressed
Chr.1	30159049	30159049	A	A	T	SNP	OS01G0723200	Intronic	NA	NA	LOC_Os01g52490	40S ribosomal protein S24, putative, expressed
Chr.1	31838746	31838746	A	A	T	SNP	OS01G0757900	Intronic	NA	NA	LOC_Os01g55310	haloacid dehalogenase-like hydrolase domain-containing protein 1A, putative, expressed
Chr.1	33904026	33904026	A	A	T	SNP	OS01G0800500	Intronic	NA	NA	LOC_Os01g58640	nucleotide pyrophosphatase/phosphodiesterase, putative, expressed
Chr.1	34552306	34552306	A	A	T	SNP	OS01G0812800	Intronic	NA	NA	LOC_Os01g59730	ribosomal protein L7Ae, putative, expressed
Chr.1	39779083	39779083	A	A	T	SNP	OS01G0912700	Intronic	NA	NA	LOC_Os01g68450	expressed protein
Chr.1	43075300	43075300	A	A	T	SNP	OS01G0974800	Exonic	5UTR	NA	LOC_Os01g74350	soluble inorganic pyrophosphatase, putative, expressed
Chr.1	43075304	43075304	A	A	T	SNP	OS01G0974800	Exonic	5UTR	NA	LOC_Os01g74350	soluble inorganic pyrophosphatase, putative, expressed
Chr.1	266081	266081	C	C	T	SNP	OS01G0104800	Intronic	NA	NA	LOC_Os01g01510	sas10/Utp3 family protein, expressed
Chr.1	605193	605193	G	G	T	SNP	OS01G0111500	Exonic	CDS	Silent	LOC_Os01g02110	helix-loop-helix DNA-binding domain containing protein, expressed
Chr.1	650906	650906	C	C	T	SNP	OS01G0112500	Intronic	NA	NA	LOC_Os01g02200	armadillo/beta-catenin repeat family protein, putative, expressed
Chr.1	651091	651091	G	G	T	SNP	OS01G0112500	Intronic	NA	NA	LOC_Os01g02200	armadillo/beta-catenin repeat family protein, putative, expressed
Chr.1	651097	651097	A	A	T	SNP	OS01G0112500	Intronic	NA	NA	LOC_Os01g02200	armadillo/beta-catenin repeat family protein, putative, expressed
Chr.1	744503	744503	A	A	T	SNP	OS01G0113700	Exonic	CDS	Missense	LOC_Os01g02350	receptor-like kinase ARK1AS, putative, expressed
Chr.1	744673	744673	C	C	T	SNP	OS01G0113700	Exonic	CDS	Missense	LOC_Os01g02350	receptor-like kinase ARK1AS, putative, expressed
Chr.1	3625050	3625050	C	C	T	SNP	OS01G0170300	Exonic	3UTR	NA	LOC_Os01g07560	receptor-like protein kinase 2 precursor, putative, expressed
Chr.1	3823704	3823704	C	C	T	SNP	OS01G0174300	Intronic	NA	NA	LOC_Os01g07910	NADH-cytochrome b5 reductase, putative, expressed
Chr.1	4216162	4216162	C	C	T	SNP	OS01G0180400	Exonic	CDS	NA	LOC_Os01g08520	DUF581 domain containing protein, expressed
Chr.1	5902323	5902323	C	C	T	SNP	OS01G0208700	Intronic	NA	NA	LOC_Os01g11054	phosphoenolpyruvate carboxylase, putative, expressed
Chr.1	6567633	6567633	G	G	T	SNP	OS01G0220100	Exonic	CDS	Silent	LOC_Os01g12070	endoglucanase precursor, putative, expressed
Chr.1	6566645	6566645	C	C	T	SNP	OS01G0220100	Exonic	CDS	Missense	LOC_Os01g12070	endoglucanase precursor, putative, expressed
Chr.1	6568053	6568053	C	C	T	SNP	OS01G0220100	Exonic	CDS	Missense	LOC_Os01g12070	endoglucanase precursor, putative, expressed
Chr.1	6788184	6788184	C	C	T	SNP	OS01G0223900	Exonic	CDS	Missense	LOC_Os01g12420	receptor-like protein kinase, putative, expressed
Chr.1	7547233	7547233	A	A	T	SNP	OS01G0236300	Exonic	CDS	Missense	LOC_Os01g13520	auxin response factor 1, putative, expressed
Chr.1	7548277	7548277	A	A	T	SNP	OS01G0236300	Intronic	NA	NA	LOC_Os01g13520	auxin response factor 1, putative, expressed
Chr.1	7557768	7557768	C	C	T	SNP	OS01G0236400	Exonic	CDS	Silent	LOC_Os01g13530	ABIL2, putative, expressed
Chr.1	7619781	7619781	C	C	T	SNP	OS01G0237500	Intronic	NA	NA	LOC_Os01g13610	isoflavone reductase homolog IRL, putative, expressed
Chr.1	8204979	8204979	A	A	T	SNP	OS01G0249200	Intronic	NA	NA	LOC_Os01g14670	Cupin domain containing protein, expressed
Chr.1	8789870	8789870	C	C	T	SNP	OS01G0261100	Intronic	NA	NA	LOC_Os01g15630	zinc finger, C3HC4 type domain containing protein, expressed
Chr.1	8949830	8949830	C	C	T	SNP	OS01G0264000	Exonic	CDS	NA	LOC_Os01g15900	expressed protein
Chr.1	9225994	9225994	A	A	T	SNP	OS01G0268300	Intronic	NA	NA	LOC_Os01g16290	DNA gyrase subunit B, chloroplast/mitochondrial precursor, putative, expressed
Chr.1	10011539	10011539	A	A	T	SNP	OS01G0281100	Exonic	5UTR	NA	LOC_Os01g17396	expressed protein
Chr.1	10008646	10008646	C	C	T	SNP	OS01G0281100	Exonic	CDS	Silent	LOC_Os01g17396	expressed protein
Chr.1	10624407	10624407	C	C	T	SNP	OS01G0292200	Intronic	NA	NA	LOC_Os01g18800	CAMK_KIN1/SNF1/Nim1_like.9 - CAMK includes calcium/calmodulin depedent protein kinases, expressed
Chr.1	12365218	12365218	C	C	T	SNP	OS01G0323600	Exonic	CDS	Missense	LOC_Os01g22010	S-adenosylmethionine synthetase, putative, expressed
Chr.1	12365241	12365241	C	C	T	SNP	OS01G0323600	Exonic	CDS	Missense	LOC_Os01g22010	S-adenosylmethionine synthetase, putative, expressed
Chr.1	12365242	12365242	C	C	T	SNP	OS01G0323600	Exonic	CDS	Missense	LOC_Os01g22010	S-adenosylmethionine synthetase, putative, expressed
Chr.1	13904096	13904096	A	A	T	SNP	OS01G0348900	Intronic	NA	NA	LOC_Os01g24710	jacalin-like lectin domain containing protein, expressed
Chr.1	15171624	15171624	C	C	T	SNP	OS01G0369700	Exonic	CDS	Silent	LOC_Os01g27210	glutathione S-transferase, putative, expressed
Chr.1	17993226	17993226	C	C	T	SNP	OS01G0511100	Intronic	NA	NA	LOC_Os01g32780	universal stress protein domain containing protein, putative, expressed
Chr.1	20601142	20601142	C	C	T	SNP	OS01G0550300	Intronic	NA	NA	LOC_Os01g36950	N-rich protein, putative, expressed
Chr.1	20742137	20742137	C	C	T	SNP	OS01G0552300	Exonic	CDS	Missense	LOC_Os01g37130	protein phosphatase 2C, putative, expressed
Chr.1	20740047	20740047	A	A	T	SNP	OS01G0552300	Intronic	NA	NA	LOC_Os01g37130	protein phosphatase 2C, putative, expressed
Chr.1	21128273	21128273	G	G	T	SNP	OS01G0558300	Exonic	5UTR	NA	LOC_Os01g37770	RWD domain containing protein, expressed
Chr.1	21637310	21637310	A	A	T	SNP	OS01G0566050	Exonic	3UTR	NA	LOC_Os01g38530	ELF3 protein, putative, expressed
Chr.1	22139884	22139884	G	G	T	SNP	OS01G0575200	Intronic	NA	NA	LOC_Os01g39330	helix-loop-helix DNA-binding domain containing protein, expressed
Chr.1	22495172	22495172	G	G	T	SNP	OS01G0580800	Exonic	CDS	Missense	LOC_Os01g39890	transposon protein, putative, unclassified, expressed
Chr.1	22499127	22499127	C	C	T	SNP	OS01G0580800	Exonic	CDS	Missense	LOC_Os01g39890	transposon protein, putative, unclassified, expressed
Chr.1	22495813	22495813	C	C	T	SNP	OS01G0580800	Intronic	NA	NA	LOC_Os01g39890	transposon protein, putative, unclassified, expressed
Chr.1	22496045	22496045	C	C	T	SNP	OS01G0580800	Intronic	NA	NA	LOC_Os01g39890	transposon protein, putative, unclassified, expressed
Chr.1	22496062	22496062	C	C	T	SNP	OS01G0580800	Intronic	NA	NA	LOC_Os01g39890	transposon protein, putative, unclassified, expressed

Chr.1	22496515	22496515	C	C	T	SNP	OS01G0580800	Intronic	NA	NA	LOC_Os01g39890	transposon protein, putative, unclassified, expressed
Chr.1	22498587	22498587	C	C	T	SNP	OS01G0580800	Intronic	NA	NA	LOC_Os01g39890	transposon protein, putative, unclassified, expressed
Chr.1	22498591	22498591	C	C	T	SNP	OS01G0580800	Intronic	NA	NA	LOC_Os01g39890	transposon protein, putative, unclassified, expressed
Chr.1	23343318	23343318	C	C	T	SNP	OS01G0595600	Exonic	5UTR	NA	LOC_Os01g41240	hydrolase, alpha/beta fold family domain containing protein, expressed
Chr.1	23343995	23343995	C	C	T	SNP	OS01G0595600	Intronic	NA	NA	LOC_Os01g41240	hydrolase, alpha/beta fold family domain containing protein, expressed
Chr.1	23344016	23344016	G	G	T	SNP	OS01G0595600	Intronic	NA	NA	LOC_Os01g41240	hydrolase, alpha/beta fold family domain containing protein, expressed
Chr.1	23344088	23344088	A	A	T	SNP	OS01G0595600	Intronic	NA	NA	LOC_Os01g41240	hydrolase, alpha/beta fold family domain containing protein, expressed
Chr.1	23675494	23675494	C	C	T	SNP	OS01G0602400	Exonic	CDS	Missense	LOC_Os01g41810	cytochrome P450 72A1, putative, expressed
Chr.1	23675497	23675497	G	G	T	SNP	OS01G0602400	Exonic	CDS	Missense	LOC_Os01g41810	cytochrome P450 72A1, putative, expressed
Chr.1	24115129	24115129	C	C	T	SNP	OS01G0609900	Exonic	CDS	Silent	LOC_Os01g42410	pleiotropic drug resistance protein, putative, expressed
Chr.1	24582010	24582010	A	A	T	SNP	OS01G0618100	Exonic	5UTR	NA	LOC_Os01g43090	oxidoreductase, aldo/keto reductase family protein, putative, expressed
Chr.1	24947172	24947172	G	G	T	SNP	OS01G0624700	Exonic	CDS	Silent	LOC_Os01g43550	WRKY12, expressed
Chr.1	25057973	25057973	G	G	T	SNP	OS01G0627600	Exonic	CDS	NA	LOC_Os01g43720	cytochrome P450 72A1, putative, expressed
Chr.1	25682297	25682297	C	C	T	SNP	OS01G0639600	Exonic	CDS	Silent	LOC_Os01g45250	DUF1645 domain containing protein, putative, expressed
Chr.1	26120230	26120230	C	C	T	SNP	OS01G0648000	Exonic	CDS	Silent	LOC_Os01g45990	potassium channel AKT1, putative, expressed
Chr.1	27084027	27084027	C	C	T	SNP	OS01G0663300	Intronic	NA	NA	LOC_Os01g47400	OsMan01 - Endo-Beta-Mannanase, expressed
Chr.1	27084083	27084083	C	C	T	SNP	OS01G0663300	Intronic	NA	NA	LOC_Os01g47400	OsMan01 - Endo-Beta-Mannanase, expressed
Chr.1	27171383	27171383	A	A	T	SNP	OS01G0665200	Exonic	5UTR	NA	LOC_Os01g47530	CGMC_MAPKCMGC_2.6 - CGMC includes CDA, MAPK, GSK3, and CLKC kinases, expressed
Chr.1	28029587	28029587	C	C	T	SNP	OS01G0680900	Intronic	NA	NA	LOC_Os01g48850	auxin-responsive protein, putative, expressed
Chr.1	28574283	28574283	C	C	T	SNP	OS01G0692300	Exonic	CDS	Silent	LOC_Os01g49740	expressed protein
Chr.1	28849147	28849147	C	C	T	SNP	OS01G0697100	Exonic	CDS	Silent	LOC_Os01g50200	UDP-glucoronosyl and UDP-glucosyl transferase domain containing protein, expressed
Chr.1	29052007	29052007	C	C	T	SNP	OS01G0701700	Exonic	CDS	Silent	LOC_Os01g50610	SAM dependent carboxyl methyltransferase, putative, expressed
Chr.1	29189273	29189273	C	C	T	SNP	OS01G0704100	Exonic	CDS	NA	LOC_Os01g50820	transporter, major facilitator family, putative, expressed
Chr.1	29846641	29846641	A	A	T	SNP	OS01G0717000	Exonic	5UTR	NA	LOC_Os01g51920	phosphotransferase, putative, expressed
Chr.1	29896319	29896319	C	C	T	SNP	OS01G0717700	Intronic	NA	NA	LOC_Os01g52010	alliin lyase precursor, putative, expressed
Chr.1	29896340	29896340	C	C	T	SNP	OS01G0717700	Intronic	NA	NA	LOC_Os01g52010	alliin lyase precursor, putative, expressed
Chr.1	29962754	29962754	C	C	T	SNP	OS01G0719000	Exonic	CDS	Silent	LOC_Os01g52100	DUF581 domain containing protein, expressed
Chr.1	30025991	30025991	G	G	T	SNP	OS01G0720400	Exonic	CDS	Missense	LOC_Os01g52230	phosphoethanolamine/phosphocholine phosphatase, putative, expressed
Chr.1	30159555	30159555	C	C	T	SNP	OS01G0723200	Intronic	NA	NA	LOC_Os01g52490	40S ribosomal protein S24, putative, expressed
Chr.1	30183782	30183782	C	C	T	SNP	OS01G0723600	Intronic	NA	NA	LOC_Os01g52530	phosphoribosyl transferase, putative, expressed
Chr.1	30183821	30183821	G	G	T	SNP	OS01G0723600	Intronic	NA	NA	LOC_Os01g52530	phosphoribosyl transferase, putative, expressed
Chr.1	31839751	31839751	C	C	T	SNP	OS01G0757900	Exonic	5UTR	NA	LOC_Os01g55310	haloacid dehalogenase-like hydrolase domain-containing protein 1A, putative, expressed
Chr.1	31839428	31839428	G	G	T	SNP	OS01G0757900	Exonic	CDS	Missense	LOC_Os01g55310	haloacid dehalogenase-like hydrolase domain-containing protein 1A, putative, expressed
Chr.1	32151953	32151953	C	C	T	SNP	OS01G0764000	Exonic	5UTR	NA	LOC_Os01g55830	glutathione S-transferase, putative, expressed
Chr.1	32259083	32259083	C	C	T	SNP	OS01G0765600	Exonic	3UTR	NA	LOC_Os01g56030	EF hand family protein, putative, expressed
Chr.1	32535536	32535536	G	G	T	SNP	OS01G0771000	Exonic	CDS	Silent	LOC_Os01g56450	DUF567 domain containing protein, putative, expressed
Chr.1	33075089	33075089	C	C	T	SNP	OS01G0780800	Intronic	NA	NA	LOC_Os01g57240	expressed protein
Chr.1	33413325	33413325	C	C	T	SNP	OS01G0787600	Exonic	5UTR	NA	LOC_Os01g57770	OsPOP4 - Putative Prolyl Oligopeptidase homologue, expressed
Chr.1	34552440	34552440	C	C	T	SNP	OS01G0812800	Exonic	CDS	Missense	LOC_Os01g59730	ribosomal protein L7Ae, putative, expressed
Chr.1	35233919	35233919	G	G	T	SNP	OS01G0824600	Exonic	3UTR	NA	LOC_Os01g60910	CAMK_KIN1/SNF1/Nim1_like.12 - CAMK includes calcium/calmodulin depedent protein kinases, expressed
Chr.1	37048201	37048201	A	A	T	SNP	OS01G0857400	Intronic	NA	NA	LOC_Os01g63854	transmembrane amino acid transporter protein, putative, expressed
Chr.1	38399877	38399877	C	C	T	SNP	OS01G0884300	Exonic	CDS	Missense	LOC_Os01g66120	No apical meristem protein, putative, expressed
Chr.1	38482869	38482869	C	C	T	SNP	OS01G0885700	Exonic	CDS	Silent	LOC_Os01g66250	S-locus-like receptor protein kinase, putative, expressed
Chr.1	40502259	40502259	C	C	T	SNP	OS01G0924900	Intronic	NA	NA	LOC_Os01g70020	DEK C terminal domain containing protein, expressed
Chr.1	41729292	41729292	G	G	T	SNP	OS01G0948600	Exonic	CDS	NA	LOC_Os01g72009	expressed protein
Chr.1	42065807	42065807	C	C	T	SNP	OS01G0955000	Intronic	NA	NA	LOC_Os01g72520	phosphoesterase family protein, putative, expressed
Chr.1	42592898	42592898	C	C	T	SNP	OS01G0966000	Exonic	3UTR	NA	LOC_Os01g73514	5-oxoprolinase, putative, expressed
Chr.1	42978329	42978329	G	G	T	SNP	OS01G0973400	Intronic	NA	NA	LOC_Os01g74190	radical SAM enzyme, putative, expressed
Chr.1	43074709	43074709	G	G	T	SNP	OS01G0974800	Exonic	CDS	Missense	LOC_Os01g74350	soluble inorganic pyrophosphatase, putative, expressed
Chr.1	43073044	43073044	C	C	T	SNP	OS01G0974800	Intronic	NA	NA	LOC_Os01g74350	soluble inorganic pyrophosphatase, putative, expressed
Chr.1	43116484	43116484	G	G	T	SNP	OS01G0975900	Exonic	CDS	Silent	LOC_Os01g74450	aquaporin protein, putative, expressed
Chr.1	265475	265478	TGAG	TGAG	T	INDEL	OS01G0104800	Intronic	NA	NA	LOC_Os01g01510	sas10/Utp3 family protein, expressed
Chr.1	605579	605591	TAGCTAGCTAGGG	TAGCTAGCTAGGG	T	INDEL	OS01G0111500	Exonic	5UTR	NA	LOC_Os01g02110	helix-loop-helix DNA-binding domain containing protein, expressed
Chr.1	604713	604731	TAGGAGAAGGAGAAGGAGA	TAGGAGAAGGAGAAGGAGA	T	INDEL	OS01G0111500	Exonic	CDS	InFrame	LOC_Os01g02110	helix-loop-helix DNA-binding domain containing protein, expressed
Chr.1	605084	605087	TGCC	TGCC	T	INDEL	OS01G0111500	Exonic	CDS	InFrame	LOC_Os01g02110	helix-loop-helix DNA-binding domain containing protein, expressed
Chr.1	604454	604455	TA	TA	T	INDEL	OS01G0111500	Intronic	NA	NA	LOC_Os01g02110	helix-loop-helix DNA-binding domain containing protein, expressed
Chr.1	651847	651849	TAG	TAG	T	INDEL	OS01G0112500	Exonic	5UTR	NA	LOC_Os01g02200	armadillo/beta-catenin repeat family protein, putative, expressed
Chr.1	3822802	3822803	TA	TA	T	INDEL	OS01G0174300	Intronic	NA	NA	LOC_Os01g07910	NADH-cytochrome b5 reductase, putative, expressed
Chr.1	4335694	4335708	TAAGCTATAAGCAGC	TAAGCTATAAGCAGC	T	INDEL	OS01G0182600	Intronic	NA	NA	LOC_Os01g08700	GIGANTEA, putative, expressed
Chr.1	9671381	9671382	TG	TG	T	INDEL	OS01G0276200	Intronic	NA	NA	LOC_Os01g16910	outer mitochondrial membrane porin, putative, expressed
Chr.1	15171143	15171146	TCTC	TCTC	T	INDEL	OS01G0369700	Intronic	NA	NA	LOC_Os01g27210	glutathione S-transferase, putative, expressed
Chr.1	15292069	15292070	TA	TA	T	INDEL	OS01G0371500	Intronic	NA	NA	LOC_Os01g27390	glutathione S-transferase, putative, expressed
Chr.1	20584447	20584457	TGTGTGTGTGA	TGTGTGTGTGA	T	INDEL	OS01G0550100	Intronic	NA	NA	LOC_Os01g36930	ubiquitin carboxyl-terminal hydrolase 6, putative, expressed
Chr.1	23345272	23345273	TA	TA	T	INDEL	OS01G0595600	Exonic	3UTR	NA	LOC_Os01g41240	hydrolase, alpha/beta fold family domain containing protein, expressed
Chr.1	23344448	23344453	TTTATC	TTTATC	T	INDEL	OS01G0595600	Intronic	NA	NA	LOC_Os01g41240	hydrolase, alpha/beta fold family domain containing protein, expressed
Chr.1	23344358	23344362	TCTAG	TCTAG	T	INDEL	OS01G0595600	Intronic	NA	NA	LOC_Os01g41240	hydrolase, alpha/beta fold family domain containing protein, expressed
Chr.1	23344484	23344488	TTATA	TTATA	T	INDEL	OS01G0595600	Intronic	NA	NA	LOC_Os01g41240	hydrolase, alpha/beta fold family domain containing protein, expressed
Chr.1	23344028	23344030	TTA	TTA	T	INDEL	OS01G0595600	Intronic	NA	NA	LOC_Os01g41240	hydrolase, alpha/beta fold family domain containing protein, expressed
Chr.1	23344320	23344322	TTA	TTA	T	INDEL	OS01G0595600	Intronic	NA	NA	LOC_Os01g41240	hydrolase, alpha/beta fold family domain containing protein, expressed
Chr.1	23344621	23344623	TAC	TAC	T	INDEL	OS01G0595600	Intronic	NA	NA	LOC_Os01g41240	hydrolase, alpha/beta fold family domain containing protein, expressed
Chr.1	23912314	23912316	TTG	TTG	T	INDEL	OS01G0606900	Intronic	NA	NA	LOC_Os01g42190	heat shock protein DnaJ, putative, expressed
Chr.1	24113960	24113961	TA	TA	T	INDEL	OS01G0609900	Intronic	NA	NA	LOC_Os01g42410	pleiotropic drug resistance protein, putative, expressed
Chr.1	24787882	24787883	TA	TA	T	INDEL	OS01G0621600	Intronic	NA	NA	LOC_Os01g43350	protein kinase family protein, putative, expressed
Chr.1	30183962	30183965	TTTG	TTTG	T	INDEL	OS01G0723600	Intronic	NA	NA	LOC_Os01g52530	phosphoribosyl transferase, putative, expressed
Chr.1	31838859	31838860	TA	TA	T	INDEL	OS01G0757900	Intronic	NA	NA	LOC_Os01g55310	haloacid dehalogenase-like hydrolase domain-containing protein 1A, putative, expressed
Chr.1	32131491	32131493	TTA	TTA	T	INDEL	OS01G0763600	Intronic	NA	NA	LOC_Os01g55780	glycerophosphoryl diester phosphodiesterase family protein, putative, expressed
Chr.1	34551310	34551312	TGA	TGA	T	INDEL	OS01G0812800	Intronic	NA	NA	LOC_Os01g59730	ribosomal protein L7Ae, putative, expressed
Chr.1	36007433	36007442	TCGCCGCCGC	TCGCCGCCGC	T	INDEL	OS01G0839300	Exonic	5UTR	NA	LOC_Os01g62210	50S ribosomal protein L17, putative, expressed
Chr.1	41503180	41503190	TAAACATAGGC	TAAACATAGGC	T	INDEL	OS01G0944100	Exonic	3UTR	NA	LOC_Os01g71624	expressed protein
Chr.1	42064887	42064889	TTA	TTA	T	INDEL	OS01G0955000	Intronic	NA	NA	LOC_Os01g72520	phosphoesterase family protein, putative, expressed
Chr.1	43075419	43075432	TTGTATGCATGTAC	TTGTATGCATGTAC	T	INDEL	OS01G0974800	Exonic	5UTR	NA	LOC_Os01g74350	soluble inorganic pyrophosphatase, putative, expressed
Chr.1	43076252	43076254	TGA	TGA	T	INDEL	OS01G0974800	Intronic	NA	NA	LOC_Os01g74350	soluble inorganic pyrophosphatase, putative, expressed
Chr.1	43115869	43115894	TTAGGTCCTCATTTGGAGGAAGCAAA	TTAGGTCCTCATTTGGAGGAAGCAAA	T	INDEL	OS01G0975900	Exonic	3UTR	NA	LOC_Os01g74450	aquaporin protein, putative, expressed
Chr.4	620598	620598	T	A	TA	INDEL	OS04G0110600	Intronic	NA	NA	LOC_Os04g02000	zinc finger family protein, putative, expressed
Chr.5	23866273	23866275	TAA	T	TA	INDEL	OS05G0485300	Intronic	NA	NA	LOC_Os05g40700	transmembrane protein 56, putative, expressed

Chr.4	32385612	32385612	T	TAA	TA	INDEL	OS04G0636800	Intronic	NA	NA	LOC_Os04g54430	immediate-early protein RSP40, putative, expressed
Chr.4	5846436	5846436	T	T	TA	INDEL	OS04G0186400	Exonic	5UTR	NA	LOC_Os04g10750	inorganic phosphate transporter, putative, expressed
Chr7	27732478	27732478	T	T	TA	INDEL	OS07G0658400	Intronic	NA	NA	LOC_Os07g46460	ferredoxin-dependent glutamate synthase, chloroplast precursor, putative, expressed
Chr7	27737432	27737432	T	T	TA	INDEL	OS07G0658400	Intronic	NA	NA	LOC_Os07g46460	ferredoxin-dependent glutamate synthase, chloroplast precursor, putative, expressed
Chr.9	19783732	19783732	T	T	TA	INDEL	OS09G0509700	Exonic	3UTR	NA	LOC_Os09g33550	CCT/B-box zinc finger protein, putative, expressed
Chr.9	14030797	14030797	T	T	TA	INDEL	OS09G0401000	Exonic	3UTR	NA	LOC_Os09g23620	MYB family transcription factor, putative, expressed
Chr.9	13574327	13574327	T	T	TA	INDEL	OS09G0392400	Exonic	5UTR	NA	LOC_Os09g22500	expressed protein
Chr.8	5366070	5366070	T	T	TA	INDEL	OS08G0191600	Intronic	NA	NA	LOC_Os08g09240	autophagy-related protein, putative, expressed
Chr.8	14416491	14416491	T	T	TA	INDEL	OS08G0327400	Intronic	NA	NA	LOC_Os08g23810	enoyl-acyl-carrier-protein reductase NADH, chloroplast precursor, expressed
Chr.8	2175017	2175017	T	T	TA	INDEL	OS08G0139000	Exonic	3UTR	NA	LOC_Os08g04440	RNA recognition motif containing protein, putative, expressed
Chr.8	3423434	3423434	T	T	TA	INDEL	OS08G0158500	Intronic	NA	NA	LOC_Os08g06200	stomatin-like protein 2, putative, expressed
Chr.6	31145127	31145127	T	T	TA	INDEL	OS06G0730600	Intronic	NA	NA	LOC_Os06g51410	OsPOP15 - Putative Prolyl Oligopeptidase homologue, expressed
Chr.6	16269439	16269439	T	T	TA	INDEL	OS06G0480000	Intronic	NA	NA	LOC_Os06g28590	bg55, putative, expressed
Chr.6	13480517	13480517	T	T	TA	INDEL	OS06G0338200	Intronic	NA	NA	LOC_Os06g23114	copper methylamine oxidase precursor, putative, expressed
Chr.6	8764113	8764113	T	T	TA	INDEL	OS06G0265000	Intronic	NA	NA	LOC_Os06g15420	asparagine synthetase, putative, expressed
Chr.6	8706340	8706340	T	T	TA	INDEL	OS06G0264200	Exonic	CDS	FrameShift	LOC_Os06g15330	CCT/B-box zinc finger protein, putative, expressed
Chr.5	25546285	25546285	T	T	TA	INDEL	OS05G0515200	Exonic	3UTR	NA	LOC_Os05g43910	cytochrome P450, putative, expressed
Chr.5	1995949	1995949	T	T	TA	INDEL	OS05G0134200	Intronic	NA	NA	LOC_Os05g04360	protein phosphatase 2C, putative, expressed
Chr.5	874907	874907	T	T	TA	INDEL	OS05G0116000	Intronic	NA	NA	LOC_Os05g02520	cupin domain containing protein, expressed
Chr.5	339878	339878	T	T	TA	INDEL	OS05G0106200	Intronic	NA	NA	LOC_Os05g01570	auxin-induced protein 5NG4, putative, expressed
Chr.4	31179183	31179183	T	T	TA	INDEL	OS04G0614600	Intronic	NA	NA	LOC_Os04g52450	aminotransferase, putative, expressed
Chr.4	27783848	27783848	T	T	TA	INDEL	OS04G0555300	Exonic	3UTR	NA	LOC_Os04g46880	transporter, major facilitator family, putative, expressed
Chr.4	5322207	5322207	T	T	TA	INDEL	OS04G0178300	Intronic	NA	NA	LOC_Os04g09900	ent-kaurene synthase, chloroplast precursor, putative, expressed
Chr.4	24850085	24850085	T	T	TA	INDEL	OS04G0496800	Intronic	NA	NA	LOC_Os04g41950	calcium-binding mitochondrial protein anon-60Da, putative, expressed
Chr.4	13645039	13645039	T	T	TA	INDEL	OS04G0304200	Intronic	NA	NA	LOC_Os04g23890	AGC_PVPK_like_kin82y.10 - ACG kinases include homologs to PKA, PKG and PKC, expressed
Chr.4	13651690	13651690	T	T	TA	INDEL	OS04G0304200	Intronic	NA	NA	LOC_Os04g23890	AGC_PVPK_like_kin82y.10 - ACG kinases include homologs to PKA, PKG and PKC, expressed
Chr.4	31441454	31441454	T	T	TA	INDEL	OS04G0619000	Intronic	NA	NA	LOC_Os04g52810	no apical meristem protein, putative, expressed
Chr.4	33532084	33532084	T	T	TA	INDEL	OS04G0657500	Intronic	NA	NA	LOC_Os04g56240	lipase, putative, expressed
Chr.4	22624261	22624261	T	T	TA	INDEL	OS04G0453233	Intronic	NA	NA	LOC_Os04g38026	transporter family protein, putative, expressed
Chr.4	22624328	22624328	T	T	TA	INDEL	OS04G0453233	Intronic	NA	NA	LOC_Os04g38026	transporter family protein, putative, expressed
Chr.4	30554466	30554466	T	T	TA	INDEL	OS04G0605300	Intronic	NA	NA	LOC_Os04g51580	leucine rich repeat containing protein, expressed
Chr.3	1615768	1615768	T	T	TA	INDEL	OS03G0128500	Intronic	NA	NA	LOC_Os03g03650	POLD2 - Putative DNA polymerase delta complex subunit, expressed
Chr.3	29145133	29145133	T	T	TA	INDEL	OS03G0719300	Intronic	NA	NA	LOC_Os03g51000	3,4-dihydroxy-2-butanone kinase, putative, expressed
Chr.3	8789628	8789628	T	T	TA	INDEL	OS03G0265900	Intronic	NA	NA	LOC_Os03g15920	expressed protein
Chr.3	11060743	11060743	T	T	TA	INDEL	OS03G0310000	Intronic	NA	NA	LOC_Os03g19670	GDSL-like lipase/acylhydrolase, putative, expressed
Chr.3	16285293	16285293	T	T	TA	INDEL	OS03G0401100	Intronic	NA	NA	LOC_Os03g28300	protein kinase domain containing protein, expressed
Chr.3	26383411	26383411	T	T	TA	INDEL	OS03G0669000	Exonic	3UTR	NA	LOC_Os03g46610	DEAD-box ATP-dependent RNA helicase, putative, expressed
Chr.3	27490513	27490513	T	T	TA	INDEL	OS03G0689100	Intronic	NA	NA	LOC_Os03g48300	histidine acid phosphatase, putative, expressed
Chr.2	8835989	8835989	T	T	TA	INDEL	OS02G0256100	Intronic	NA	NA	LOC_Os02g15690	polygalacturonase, putative, expressed
Chr.2	6835139	6835139	T	T	TA	INDEL	OS02G0222500	Intronic	NA	NA	LOC_Os02g12939	expressed protein
Chr.2	9973659	9973659	T	T	TA	INDEL	OS02G0273700	Intronic	NA	NA	LOC_Os02g17350	VHS and GAT domain containing protein, expressed
Chr.2	9989005	9989005	T	T	TA	INDEL	OS02G0273900	Exonic	3UTR	NA	LOC_Os02g17370	expressed protein
Chr.2	9976743	9976743	T	T	TA	INDEL	OS02G0273700	Intronic	NA	NA	LOC_Os02g17350	VHS and GAT domain containing protein, expressed
Chr.12	25649042	25649042	T	T	TA	INDEL	OS12G0607100	Intronic	NA	NA	LOC_Os12g41400	eukaryotic translation initiation factor 2 subunit gamma, putative, expressed
Chr.12	2249690	2249690	T	T	TA	INDEL	OS12G0145700	Exonic	5UTR	NA	LOC_Os12g05110	pyruvate kinase, putative, expressed
Chr.11	22177566	22177566	T	T	TA	INDEL	OS11G0586300	Intronic	NA	NA	LOC_Os11g37560	expressed protein
Chr.11	2145602	2145602	T	T	TA	INDEL	OS11G0147100	Intronic	NA	NA	LOC_Os11g04990	expressed protein
Chr.11	17353636	17353636	T	T	TA	INDEL	OS11G0490900	Intronic	NA	NA	LOC_Os11g29870	WRKY72, expressed
Chr.11	22273921	22273921	T	T	TA	INDEL	OS11G0587600	Intronic	NA	NA	LOC_Os11g37700	pleiotropic drug resistance protein, putative, expressed
Chr.11	22278546	22278546	T	T	TA	INDEL	OS11G0587600	Intronic	NA	NA	LOC_Os11g37700	pleiotropic drug resistance protein, putative, expressed
Chr.11	22279673	22279673	T	T	TA	INDEL	OS11G0587600	Intronic	NA	NA	LOC_Os11g37700	pleiotropic drug resistance protein, putative, expressed
Chr.11	27488265	27488265	T	T	TA	INDEL	OS11G0679700	Intronic	NA	NA	LOC_Os11g45400	glycerol-3-phosphate acyltransferase, putative, expressed
Chr.1	10655895	10655895	T	T	TA	INDEL	OS01G0292900	Intronic	NA	NA	LOC_Os01g18850	OsSPL1 - SBP-box gene family member, expressed
Chr.1	29490022	29490022	T	T	TA	INDEL	OS01G0709500	Intronic	NA	NA	LOC_Os01g51290	protein kinase family protein, putative, expressed
Chr.1	3941555	3941555	T	T	TA	INDEL	OS01G0176500	Intronic	NA	NA	LOC_Os01g08150	expressed protein
Chr.1	7365218	7365218	T	T	TA	INDEL	OS01G0233000	Intronic	NA	NA	LOC_Os01g13210	salt stress root protein RS1, putative, expressed
Chr.1	26804915	26804915	T	T	TA	INDEL	OS01G0658900	Intronic	NA	NA	LOC_Os01g46970	transcription factor, putative, expressed
Chr.1	41100206	41100206	T	T	TA	INDEL	OS01G0936100	Exonic	3UTR	NA	LOC_Os01g71000	protein kinase APK1B, chloroplast precursor, putative, expressed
Chr.1	21076098	21076098	T	T	TA	INDEL	OS01G0557500	Intronic	NA	NA	LOC_Os01g37690	sodium/calcium exchanger protein, putative, expressed
Chr.4	13645683	13645684	TA	T	TAA	INDEL	OS04G0304200	Intronic	NA	NA	LOC_Os04g23890	AGC_PVPK_like_kin82y.10 - ACG kinases include homologs to PKA, PKG and PKC, expressed
Chr3	21635306	21635307	TA	T	TAA	INDEL	OS03G0586500	Intronic	NA	NA	LOC_Os03g38950	chloroplast post-illumination chlorophyll fluorescence increase protein, putative, expressed
Chr9	22913191	22913192	TA	T	TAA	INDEL	OS09G0572900	Intronic	NA	NA	LOC_Os09g39960	dynamin family protein, putative, expressed
Chr7	20235	20235	T	TA	TAA	INDEL	OS07G0100300	Intronic	NA	NA	LOC_Os07g01030	glycosyl transferase, group 1 domain containing protein, expressed
Chr.6	3447499	3447499	T	TA	TAA	INDEL	OS06G0168500	Intronic	NA	NA	LOC_Os06g07200	syntaxin, putative, expressed
Chr.1	29492240	29492240	T	TA	TAA	INDEL	OS01G0709500	Exonic	5UTR	NA	LOC_Os01g51290	protein kinase family protein, putative, expressed
Chr.6	3447728	3447728	T	TTA	TAA	INDEL	OS06G0168500	Intronic	NA	NA	LOC_Os06g07200	syntaxin, putative, expressed
Chr.4	25134049	25134049	T	T	TAA	INDEL	OS04G0503500	Intronic	NA	NA	LOC_Os04g42470	regulatory subunit, putative, expressed
Chr.3	24817018	24817018	T	T	TAA	INDEL	OS03G0643300	Intronic	NA	NA	LOC_Os03g44150	aminotransferase, putative, expressed
Chr.3	538648	538648	T	T	TAA	INDEL	OS03G0109300	Intronic	NA	NA	LOC_Os03g01880	possible lysine decarboxylase domain containing protein, expressed
Chr.3	16592517	16592517	T	T	TAA	INDEL	OS03G0405500	Intronic	NA	NA	LOC_Os03g29190	PDI, putative, expressed
Chr.1	10342967	10342967	T	T	TAA	INDEL	OS01G0287600	Intronic	NA	NA	LOC_Os01g18400	CHIT10 - Chitinase family protein precursor, expressed
Chr.1	23344059	23344059	T	T	TAA	INDEL	OS01G0595600	Intronic	NA	NA	LOC_Os01g41240	hydrolase, alpha/beta fold family domain containing protein, expressed
Chr.10	12872880	12872880	T	TA	TAAA	INDEL	OS10G0389000	Intronic	NA	NA	LOC_Os10g25010	OsCML8 - Calmodulin-related calcium sensor protein, expressed
Chr.5	2104308	2104308	T	T	TAAA	INDEL	OS05G0135900	Intronic	NA	NA	LOC_Os05g04530	IF, putative, expressed
Chr.4	22183414	22183414	T	T	TAAA	INDEL	OS04G0444800	Intronic	NA	NA	LOC_Os04g36720	ferric-chelate reductase, putative, expressed
Chr.3	26381125	26381125	T	T	TAAA	INDEL	OS03G0669000	Intronic	NA	NA	LOC_Os03g46610	DEAD-box ATP-dependent RNA helicase, putative, expressed
Chr10	15403573	15403574	TA	T	TAAAA	INDEL	OS10G0431900	Intronic	NA	NA	LOC_Os10g29620	tyrosine protein kinase domain containing protein, putative, expressed
Chr2	22469626	22469626	T	TAA	TAAAA	INDEL	OS02G0582600	Exonic	5UTR	NA	LOC_Os02g37160	heavy metal transport/detoxification protein, putative, expressed
Chr.4	5385868	5385868	T	T	TAACGCATCCGTATA	INDEL	OS04G0179200	Exonic	3UTR	NA	LOC_Os04g10010	sex determination protein tasselseed-2, putative, expressed
Chr7	27728456	27728456	T	T	TAATAAGAC	INDEL	OS07G0658400	Intronic	NA	NA	LOC_Os07g46460	ferredoxin-dependent glutamate synthase, chloroplast precursor, putative, expressed
Chr.6	4868303	4868303	T	T	TAATC	INDEL	OS06G0195800	Intronic	NA	NA	LOC_Os06g09560	heat shock protein DnaJ, putative, expressed
Chr.3	28243632	28243632	T	T	TAC	INDEL	OS03G0703000	Intronic	NA	NA	LOC_Os03g49600	Os3bglu7 - beta-glucosidase, exo-beta-glucanse, expressed
Chr.4	333824	333824	T	T	TACAAACACACACACACAC	INDEL	OS04G0105100	Exonic	5UTR	NA	LOC_Os04g01490	zinc finger, C3HC4 type, domain containing protein, expressed
Chr.6	29144950	29144950	T	T	TACACAC	INDEL	OS06G0696600	Exonic	5UTR	NA	LOC_Os06g48180	glycosyl hydrolases family 16, putative, expressed
Chr.10	10391481	10391481	T	TATATATATATACACACACAC	TACACACACAC	INDEL	OS10G0346300	Intronic	NA	NA	LOC_Os10g20610	laccase-15 precursor, putative, expressed

Chr.1	268099	268099	T	T	TAGAG	INDEL	OS01G0104800	Exonic	3UTR	NA	LOC_Os01g01510	sas10/Utp3 family protein, expressed
Chr7	21419256	21419256	T	T	TAGC	INDEL	OS07G0541900	Exonic	5UTR	NA	LOC_Os07g35750	TKL_IRAK_DUF26-ld.3 - DUF26 kinases have homology to DUF26 containing loci, expressed
Chr.6	8848308	8848308	T	T	TAGG	INDEL	OS06G0266800	Exonic	3UTR	NA	LOC_Os06g15620	GASR7 - Gibberellin-regulated GASA/GAST/Snakin family protein precursor, expressed
Chr.5	27959532	27959532	T	T	TATAC	INDEL	OS05G0562000	Intronic	NA	NA	LOC_Os05g48790	expressed protein
Chr.3	2708574	2708574	T	TATTAATTAATTA	TATTAATTA	INDEL	OS03G0148700	Intronic	NA	NA	LOC_Os03g05470	tyrosine protein kinase domain containing protein, putative, expressed
Chr.3	24810323	24810323	T	T	TATTTGCTCGGATGGAGGA	INDEL	OS03G0643300	Exonic	3UTR	NA	LOC_Os03g44150	aminotransferase, putative, expressed
Chr.3	27249404	27249404	T	TCCC	TC	INDEL	OS03G0683700	Intronic	NA	NA	LOC_Os03g47930	expressed protein
Chr7	3610600	3610600	T	T	TC	INDEL	OS07G0167100	Exonic	3UTR	NA	LOC_Os07g07260	glutamine-dependent NAD, putative, expressed
Chr7	22889404	22889404	T	T	TC	INDEL	OS07G0569100	Exonic	3UTR	NA	LOC_Os07g38170	remorin, putative, expressed
Chr7	27554903	27554903	T	T	TC	INDEL	OS07G0655400	Exonic	3UTR	NA	LOC_Os07g46170	OsRhmbd13 - Putative Rhomboid homologue, expressed
Chr.6	9091203	9091203	T	T	TC	INDEL	OS06G0270900	Exonic	5UTR	NA	LOC_Os06g15990	aldehyde dehydrogenase, putative, expressed
Chr.3	1741945	1741945	T	T	TC	INDEL	OS03G0130700	Exonic	5UTR	NA	LOC_Os03g03860	expressed protein
Chr.3	1718195	1718195	T	T	TC	INDEL	OS03G0130400	Intronic	NA	NA	LOC_Os03g03820	adenylate kinase, putative, expressed
Chr.3	33347905	33347905	T	T	TC	INDEL	OS03G0799900	Intronic	NA	NA	LOC_Os03g58570	WD domain, G-beta repeat domain containing protein, expressed
Chr.3	23375359	23375359	T	T	TC	INDEL	OS03G0616700	Intronic	NA	NA	LOC_Os03g42050	exportin-7-A, putative, expressed
Chr.3	1243381	1243381	T	T	TC	INDEL	OS03G0122300	Intronic	NA	NA	LOC_Os03g03034	flavonol synthase/flavanone 3-hydroxylase, putative, expressed
Chr.2	9977785	9977785	T	T	TC	INDEL	OS02G0273700	Exonic	3UTR	NA	LOC_Os02g17350	VHS and GAT domain containing protein, expressed
Chr.2	9973247	9973247	T	T	TC	INDEL	OS02G0273700	Intronic	NA	NA	LOC_Os02g17350	VHS and GAT domain containing protein, expressed
Chr.11	14049405	14049405	T	T	TC	INDEL	OS11G0434000	Intronic	NA	NA	LOC_Os11g24630	magnesium-dependent phosphatase 1, putative, expressed
Chr.1	29846607	29846607	T	T	TC	INDEL	OS01G0717000	Exonic	5UTR	NA	LOC_Os01g51920	phosphotransferase, putative, expressed
Chr.1	28349296	28349296	T	T	TC	INDEL	OS01G0687300	Intronic	NA	NA	LOC_Os01g49310	expressed protein
Chr.1	21126269	21126269	T	T	TCA	INDEL	OS01G0558300	Intronic	NA	NA	LOC_Os01g37770	RWD domain containing protein, expressed
Chr.3	16284835	16284835	T	T	TCAA	INDEL	OS03G0401100	Exonic	CDS	InFrame	LOC_Os03g28300	protein kinase domain containing protein, expressed
Chr.1	21125157	21125157	T	T	TCAAAGTGTC	INDEL	OS01G0558300	Exonic	3UTR	NA	LOC_Os01g37770	RWD domain containing protein, expressed
Chr.9	13568640	13568640	T	T	TCAATCAA	INDEL	OS09G0392400	Intronic	NA	NA	LOC_Os09g22500	expressed protein
Chr7	27174013	27174013	T	T	TCAC	INDEL	OS07G0650100	Exonic	CDS	InFrame	LOC_Os07g45550	expressed protein
Chr2	6146227	6146237	TCTCTCACACA	T	TCACA	INDEL	OS02G0209300	Exonic	3UTR	NA	LOC_Os02g11859	expressed protein
Chr.3	12589358	12589358	T	T	TCACCAC	INDEL	OS03G0338400	Exonic	CDS	InFrame	LOC_Os03g21970	basic helix-loop-helix domain containing protein, expressed
Chr.1	30159420	30159420	T	T	TCACCAC	INDEL	OS01G0723200	Intronic	NA	NA	LOC_Os01g52490	40S ribosomal protein S24, putative, expressed
Chr.11	17352810	17352810	T	T	TCATC	INDEL	OS11G0490900	Intronic	NA	NA	LOC_Os11g29870	WRKY72, expressed
Chr.5	22241344	22241344	T	T	TCC	INDEL	OS05G0453300	Exonic	5UTR	NA	LOC_Os05g37930	expressed protein
Chr.1	34612751	34612751	T	T	TCC	INDEL	OS01G0813900	Exonic	5UTR	NA	LOC_Os01g59850	GTPase-activating protein, putative, expressed
Chr.10	2914033	2914033	T	T	TCGC	INDEL	OS10G0148400	Exonic	CDS	InFrame	LOC_Os10g05780	POT domain containing peptide transporter, putative, expressed
Chr.11	21463891	21463891	T	T	TCGCCGC	INDEL	OS11G0572200	Exonic	CDS	InFrame	LOC_Os11g36400	expressed protein
Chr10	12873261	12873276	TCGCCGCCGCCGCCGC	T	TCGCCGCCGCCGC	INDEL	OS10G0389000	Exonic	CDS	InFrame	LOC_Os10g25010	OsCML8 - Calmodulin-related calcium sensor protein, expressed
Chr.4	31443242	31443242	T	T	TCGCCGGCGCCGCCGC	INDEL	OS04G0619000	Exonic	CDS	InFrame	LOC_Os04g52810	no apical meristem protein, putative, expressed
Chr.2	22687356	22687356	T	T	TCGCCTC	INDEL	OS02G0588500	Exonic	5UTR	NA	LOC_Os02g37590	glycerophosphoryl diester phosphodiesterase family protein, putative, expressed
Chr.1	34552596	34552596	T	T	TCGGTAG	INDEL	OS01G0812800	Exonic	5UTR	NA	LOC_Os01g59730	ribosomal protein L7Ae, putative, expressed
Chr.3	4541438	4541438	T	TCTC	TCTCCTC	INDEL	OS03G0186900	Exonic	CDS	InFrame	LOC_Os03g08790	aspartic proteinase nepenthesin precursor, putative, expressed
Chr.4	619419	619420	AG	A	TG	INDEL	OS04G0110600	Intronic	NA	NA	LOC_Os04g02000	zinc finger family protein, putative, expressed
Chr.6	27143238	27143238	T	T	TG	INDEL	OS06G0659300	Intronic	NA	NA	LOC_Os06g44890	villin protein, putative, expressed
Chr.4	4458948	4458948	T	T	TG	INDEL	OS04G0165700	Intronic	NA	NA	LOC_Os04g08350	cysteine synthase, chloroplast/chromoplast precursor, putative, expressed
Chr.3	26381942	26381942	T	T	TG	INDEL	OS03G0669000	Intronic	NA	NA	LOC_Os03g46610	DEAD-box ATP-dependent RNA helicase, putative, expressed
Chr.3	10042896	10042896	T	T	TG	INDEL	OS03G0289800	Intronic	NA	NA	LOC_Os03g18030	leucoanthocyanidin dioxygenase, putative, expressed
Chr.3	8200720	8200720	T	T	TG	INDEL	OS03G0255100	Intronic	NA	NA	LOC_Os03g15020	beta-galactosidase precursor, putative, expressed
Chr.3	1876198	1876198	T	T	TG	INDEL	OS03G0133300	Intronic	NA	NA	LOC_Os03g04100	expressed protein
Chr.2	9972964	9972964	T	T	TG	INDEL	OS02G0273700	Intronic	NA	NA	LOC_Os02g17350	VHS and GAT domain containing protein, expressed
Chr.2	35098433	35098433	T	T	TG	INDEL	OS02G0817900	Intronic	NA	NA	LOC_Os02g57290	cytochrome P450, putative, expressed
Chr.11	14051091	14051091	T	T	TG	INDEL	OS11G0434000	Intronic	NA	NA	LOC_Os11g24630	magnesium-dependent phosphatase 1, putative, expressed
Chr.11	17353157	17353157	T	T	TG	INDEL	OS11G0490900	Intronic	NA	NA	LOC_Os11g29870	WRKY72, expressed
Chr.10	18539507	18539507	T	T	TG	INDEL	OS10G0488800	Exonic	3UTR	NA	LOC_Os10g34710	myosin-2 heavy chain, non muscle, putative, expressed
Chr.10	3756920	3756920	T	T	TG	INDEL	OS10G0159300	Intronic	NA	NA	LOC_Os10g07160	retrotransposon, putative, centromere-specific, expressed
Chr.1	650984	650984	T	T	TG	INDEL	OS01G0112500	Intronic	NA	NA	LOC_Os01g02200	armadillo/beta-catenin repeat family protein, putative, expressed
Chr.1	7279957	7279957	T	T	TG	INDEL	OS01G0231700	Intronic	NA	NA	LOC_Os01g13080	60S acidic ribosomal protein, putative, expressed
Chr.11	27488399	27488399	T	T	TGA	INDEL	OS11G0679700	Intronic	NA	NA	LOC_Os11g45400	glycerol-3-phosphate acyltransferase, putative, expressed
Chr.5	29490513	29490513	T	T	TGAA	INDEL	OS05G0591900	Exonic	CDS	InFrame	LOC_Os05g51420	hypersensitive-induced response protein, putative, expressed
Chr.4	35297862	35297862	T	T	TGAA	INDEL	OS04G0690100	Exonic	CDS	NA	LOC_Os04g59380	ZOS4-14 - C2H2 zinc finger protein, expressed
Chr.11	7939548	7939548	T	T	TGAG	INDEL	OS11G0247000	Exonic	5UTR	NA	LOC_Os11g14190	ARGOS, putative, expressed
Chr.11	6649216	6649216	T	T	TGC	INDEL	OS11G0227000	Exonic	5UTR	NA	LOC_Os11g11980	expressed protein
Chr.6	10686787	10686787	T	T	TGCACACTCCATATACACCAAA	INDEL	OS06G0291500	Exonic	3UTR	NA	LOC_Os06g18820	serine threonine kinase, putative, expressed
Chr.8	3422695	3422695	T	T	TGCCTTTGATC	INDEL	OS08G0158500	Intronic	NA	NA	LOC_Os08g06200	stomatin-like protein 2, putative, expressed
Chr.3	26385408	26385408	T	T	TGCGGCG	INDEL	OS03G0669000	Exonic	5UTR	NA	LOC_Os03g46610	DEAD-box ATP-dependent RNA helicase, putative, expressed
Chr.9	10253618	10253618	T	T	TGG	INDEL	OS09G0337300	Exonic	CDS	FrameShift	LOC_Os09g16760	ankyrin repeat family protein, putative, expressed
Chr.2	8582716	8582716	T	T	TGTAC	INDEL	OS02G0252200	Exonic	5UTR	NA	LOC_Os02g15340	no apical meristem protein, putative, expressed
Chr.3	1787710	1787710	T	T	TGTGCTTACAATAATGA	INDEL	OS03G0131200	Exonic	3UTR	NA	LOC_Os03g03910	catalase domain containing protein, expressed
Chr7	4504711	4504711	T	T	TTA	INDEL	OS07G0185200	Intronic	NA	NA	LOC_Os07g08760	START domain containing protein, putative, expressed
Chr.4	11240399	11240399	T	T	TTA	INDEL	OS04G0269600	Intronic	NA	NA	LOC_Os04g20164	amine oxidase precursor, putative, expressed
Chr.2	9990102	9990102	T	T	TTA	INDEL	OS02G0273900	Intronic	NA	NA	LOC_Os02g17370	expressed protein
Chr.2	34224068	34224068	T	T	TTA	INDEL	OS02G0802700	Intronic	NA	NA	LOC_Os02g55910	monogalactosyldiacylglycerol synthase, putative, expressed
Chr.11	2759983	2759983	T	T	TTA	INDEL	OS11G0157400	Exonic	3UTR	NA	LOC_Os11g05880	exo70 exocyst complex subunit, putative, expressed
Chr.1	22497422	22497422	T	T	TTA	INDEL	OS01G0580800	Intronic	NA	NA	LOC_Os01g39890	transposon protein, putative, unclassified, expressed
Chr.1	33074042	33074042	T	T	TTA	INDEL	OS01G0780800	Intronic	NA	NA	LOC_Os01g57240	expressed protein
Chr.4	33101698	33101698	T	TA	TTAA	INDEL	OS04G0649500	Exonic	3UTR	NA	LOC_Os04g55600	expressed protein
Chr.3	3564972	3564972	T	T	TTAC	INDEL	OS03G0166000	Intronic	NA	NA	LOC_Os03g06980	nucleic acid binding protein, putative, expressed
Chr.4	28297696	28297696	T	T	TTAGA	INDEL	OS04G0564700	Intronic	NA	NA	LOC_Os04g47700	expressed protein
Chr.3	9014451	9014451	T	TCAGGATG	TTAGGATG	INDEL	OS03G0269900	Intronic	NA	NA	LOC_Os03g16334	fringe-related protein, putative, expressed
Chr.6	19614575	19614575	T	T	TTC	INDEL	OS06G0528600	Intronic	NA	NA	LOC_Os06g33710	spermidine synthase, putative, expressed
Chr.6	30301755	30301755	T	T	TTC	INDEL	OS06G0714200	Exonic	5UTR	NA	LOC_Os06g50030	CAMK_CAMK_like.30 - CAMK includes calcium/calmodulin depedent protein kinases, expressed
Chr.4	27643667	27643667	T	T	TTC	INDEL	OS04G0551800	Exonic	3UTR	NA	LOC_Os04g46620	T-complex protein, putative, expressed
Chr.3	15470988	15470988	T	T	TTC	INDEL	OS03G0387900	Intronic	NA	NA	LOC_Os03g27019	expressed protein
Chr.2	9972771	9972771	T	T	TTC	INDEL	OS02G0273700	Exonic	5UTR	NA	LOC_Os02g17350	VHS and GAT domain containing protein, expressed
Chr.2	33953386	33953386	T	T	TTC	INDEL	OS02G0797600	Exonic	5UTR	NA	LOC_Os02g55430	alginate regulatory protein AlgP, putative, expressed
Chr.1	2275483	2275483	T	T	TTC	INDEL	OS01G0142300	Intronic	NA	NA	LOC_Os01g04920	glycosyl transferase, group 1 domain containing protein, expressed
Chr7	4683996	4683996	T	T	TTCTCTC	INDEL	OS07G0187700	Intronic	NA	NA	LOC_Os07g09000	WD domain, G-beta repeat domain containing protein, expressed
Chr.2	34223263	34223263	T	TTCTCTCTC	TTCTCTCTCTC	INDEL	OS02G0802700	Exonic	CDS	NA	LOC_Os02g55910	monogalactosyldiacylglycerol synthase, putative, expressed

Chr.12	25648108	25648108	T	TTTG	TTG	INDEL	OS12G0607100	Intronic	NA	NA	LOC_Os12g41400	eukaryotic translation initiation factor 2 subunit gamma, putative, expressed
Chr.6	20011878	20011878	T	T	TTG	INDEL	OS06G0534800	Exonic	3UTR	NA	LOC_Os06g34390	zinc finger, C3HC4 type domain containing protein, expressed
Chr.6	23467964	23467964	T	T	TTG	INDEL	OS06G0596300	Intronic	NA	NA	LOC_Os06g39520	myristoyl-acyl carrier protein thioesterase, chloroplast precursor, putative, expressed
Chr.4	5324978	5324978	T	T	TTG	INDEL	OS04G0178300	Intronic	NA	NA	LOC_Os04g09900	ent-kaurene synthase, chloroplast precursor, putative, expressed
Chr.11	6650126	6650126	T	T	TTG	INDEL	OS11G0227000	Exonic	3UTR	NA	LOC_Os11g11980	expressed protein
Chr.4	6479018	6479018	T	T	TTGC	INDEL	OS04G0194500	Intronic	NA	NA	LOC_Os04g11820	white-brown complex homolog protein, putative, expressed
Chr.1	4216145	4216145	T	T	TTGCCGC	INDEL	OS01G0180400	Exonic	CDS	NA	LOC_Os01g08520	DUF581 domain containing protein, expressed
Chr.3	29143340	29143340	T	T	TTGTGTG	INDEL	OS03G0719300	Intronic	NA	NA	LOC_Os03g51000	3,4-dihydroxy-2-butanone kinase, putative, expressed
Chr.3	36005729	36005729	T	T	TTTAA	INDEL	OS03G0854800	Exonic	5UTR	NA	LOC_Os03g63770	RCD1, putative, expressed
Chr.11	17352863	17352863	T	T	TTTAA	INDEL	OS11G0490900	Intronic	NA	NA	LOC_Os11g29870	WRKY72, expressed
Chr.11	17354642	17354642	T	T	TTTAA	INDEL	OS11G0490900	Intronic	NA	NA	LOC_Os11g29870	WRKY72, expressed
Chr.5	2764739	2764739	T	T	TTTCACCACTGATC	INDEL	OS05G0148300	Exonic	3UTR	NA	LOC_Os05g05560	expressed protein
Chr.3	7334922	7334922	T	TTTCTTCTTC	TTTCTTC	INDEL	OS03G0238800	Exonic	5UTR	NA	LOC_Os03g13560	hydroxyproline-rich glycoprotein family protein, putative, expressed
Chr.3	1489128	1489128	T	T	TTTTTAGTG	INDEL	OS03G0126000	Intronic	NA	NA	LOC_Os03g03450	anthranilate phosphoribosyltransferase, chloroplast precursor, putative, expressed
Chr.4	30615636	30615636	T	A	T	SNP	OS04G0606200	Exonic	3UTR	NA	LOC_Os04g51680	expressed protein
Chr2	23995092	23995092	C	A	C	SNP	OS02G0610600	Intronic	NA	NA	LOC_Os02g39720	splicing factor, arginine/serine-rich 7, putative, expressed
Chr6	2952251	2952251	T	A	T	SNP	OS06G0158000	Intronic	NA	NA	LOC_Os06g06350	AMP-binding enzyme, putative, expressed
Chr8	3337836	3337836	T	A	T	SNP	OS08G0157500	Exonic	3UTR	NA	LOC_Os08g06100	O-methyltransferase, putative, expressed
Chr.11	20185904	20185904	T	A	T	SNP	OS11G0547000	Exonic	5UTR	NA	LOC_Os11g34460	OsFBO10 - F-box and other domain containing protein, expressed
Chr.11	21891484	21891484	G	A	G	SNP	OS11G0579800	Exonic	CDS	Silent	LOC_Os11g37080	h/ACA ribonucleoprotein complex subunit 1-like protein 1, putative, expressed
Chr.11	20185260	20185260	G	A	G	SNP	OS11G0547000	Exonic	5UTR	NA	LOC_Os11g34460	OsFBO10 - F-box and other domain containing protein, expressed
Chr.11	22502167	22502167	G	A	G	SNP	OS11G0592000	Exonic	CDS	Silent	LOC_Os11g37950	WIP3 - Wound-induced protein precursor, expressed
Chr.11	6894926	6894926	C	A	C	SNP	OS11G0229500	Exonic	CDS	Silent	LOC_Os11g12340	disease resistance protein RPM1, putative, expressed
Chr.11	27487448	27487448	T	A	T	SNP	OS11G0679700	Exonic	CDS	NA	LOC_Os11g45400	glycerol-3-phosphate acyltransferase, putative, expressed
Chr.11	4971399	4971399	T	A	T	SNP	OS11G0199200	Exonic	3UTR	NA	LOC_Os11g09280	OsPDIL1-1 protein disulfide isomerase PDIL1-1, expressed
Chr.11	4972723	4972723	T	A	T	SNP	OS11G0199200	Intronic	NA	NA	LOC_Os11g09280	OsPDIL1-1 protein disulfide isomerase PDIL1-1, expressed
Chr.11	4972739	4972739	T	A	T	SNP	OS11G0199200	Intronic	NA	NA	LOC_Os11g09280	OsPDIL1-1 protein disulfide isomerase PDIL1-1, expressed
Chr.11	4974263	4974263	T	A	T	SNP	OS11G0199200	Intronic	NA	NA	LOC_Os11g09280	OsPDIL1-1 protein disulfide isomerase PDIL1-1, expressed
Chr.11	4974325	4974325	T	A	T	SNP	OS11G0199200	Intronic	NA	NA	LOC_Os11g09280	OsPDIL1-1 protein disulfide isomerase PDIL1-1, expressed
Chr.11	7125334	7125334	T	A	T	SNP	OS11G0234200	Exonic	CDS	Missense	LOC_Os11g12650	PHD-finger domain containing protein, putative, expressed
Chr.11	7127679	7127679	T	A	T	SNP	OS11G0234200	Intronic	NA	NA	LOC_Os11g12650	PHD-finger domain containing protein, putative, expressed
Chr.11	7128249	7128249	T	A	T	SNP	OS11G0234200	Intronic	NA	NA	LOC_Os11g12650	PHD-finger domain containing protein, putative, expressed
Chr.11	19675827	19675827	T	A	T	SNP	OS11G0539200	Intronic	NA	NA	LOC_Os11g33270	glycosyl hydrolases family 16, putative, expressed
Chr.11	20644436	20644436	T	A	T	SNP	OS11G0555300	Exonic	CDS	Missense	LOC_Os11g35210	NB-ARC domain containing protein, expressed
Chr.11	20645951	20645951	T	A	T	SNP	OS11G0555300	Exonic	CDS	Missense	LOC_Os11g35210	NB-ARC domain containing protein, expressed
Chr.11	20647371	20647371	T	A	T	SNP	OS11G0555300	Intronic	NA	NA	LOC_Os11g35210	NB-ARC domain containing protein, expressed
Chr.11	22175613	22175613	T	A	T	SNP	OS11G0586300	Exonic	3UTR	NA	LOC_Os11g37560	expressed protein
Chr.11	23184873	23184873	T	A	T	SNP	OS11G0602600	Intronic	NA	NA	LOC_Os11g38959	40S ribosomal protein S9-2, putative, expressed
Chr.11	23185302	23185302	T	A	T	SNP	OS11G0602600	Intronic	NA	NA	LOC_Os11g38959	40S ribosomal protein S9-2, putative, expressed
Chr.11	23232287	23232287	T	A	T	SNP	OS11G0603200	Intronic	NA	NA	LOC_Os11g39020	ABC transporter, ATP-binding protein, putative, expressed
Chr.11	26512006	26512006	T	A	T	SNP	OS11G0660500	Intronic	NA	NA	LOC_Os11g43900	translationally-controlled tumor protein, putative, expressed
Chr.11	1226186	1226186	T	A	T	SNP	OS11G0126800	Exonic	CDS	NA	LOC_Os11g03290	nucleoside-triphosphatase, putative, expressed
Chr.11	1225669	1225669	G	A	G	SNP	OS11G0126800	Exonic	CDS	NA	LOC_Os11g03290	nucleoside-triphosphatase, putative, expressed
Chr.11	1226279	1226279	G	A	G	SNP	OS11G0126800	Intronic	NA	NA	LOC_Os11g03290	nucleoside-triphosphatase, putative, expressed
Chr.11	4971494	4971494	G	A	G	SNP	OS11G0199200	Exonic	3UTR	NA	LOC_Os11g09280	OsPDIL1-1 protein disulfide isomerase PDIL1-1, expressed
Chr.11	4972351	4972351	G	A	G	SNP	OS11G0199200	Exonic	CDS	Silent	LOC_Os11g09280	OsPDIL1-1 protein disulfide isomerase PDIL1-1, expressed
Chr.11	4972985	4972985	G	A	G	SNP	OS11G0199200	Intronic	NA	NA	LOC_Os11g09280	OsPDIL1-1 protein disulfide isomerase PDIL1-1, expressed
Chr.11	4973680	4973680	G	A	G	SNP	OS11G0199200	Intronic	NA	NA	LOC_Os11g09280	OsPDIL1-1 protein disulfide isomerase PDIL1-1, expressed
Chr.11	4973699	4973699	G	A	G	SNP	OS11G0199200	Intronic	NA	NA	LOC_Os11g09280	OsPDIL1-1 protein disulfide isomerase PDIL1-1, expressed
Chr.11	4973711	4973711	G	A	G	SNP	OS11G0199200	Intronic	NA	NA	LOC_Os11g09280	OsPDIL1-1 protein disulfide isomerase PDIL1-1, expressed
Chr.11	4973968	4973968	G	A	G	SNP	OS11G0199200	Exonic	CDS	Silent	LOC_Os11g09280	OsPDIL1-1 protein disulfide isomerase PDIL1-1, expressed
Chr.11	4974113	4974113	G	A	G	SNP	OS11G0199200	Intronic	NA	NA	LOC_Os11g09280	OsPDIL1-1 protein disulfide isomerase PDIL1-1, expressed
Chr.11	4974401	4974401	G	A	G	SNP	OS11G0199200	Intronic	NA	NA	LOC_Os11g09280	OsPDIL1-1 protein disulfide isomerase PDIL1-1, expressed
Chr.11	4974544	4974544	G	A	G	SNP	OS11G0199200	Intronic	NA	NA	LOC_Os11g09280	OsPDIL1-1 protein disulfide isomerase PDIL1-1, expressed
Chr.11	4974570	4974570	G	A	G	SNP	OS11G0199200	Intronic	NA	NA	LOC_Os11g09280	OsPDIL1-1 protein disulfide isomerase PDIL1-1, expressed
Chr.11	5565620	5565620	G	A	G	SNP	OS11G0208600	Exonic	3UTR	NA	LOC_Os11g10250	expressed protein
Chr.11	5565662	5565662	G	A	G	SNP	OS11G0208600	Exonic	3UTR	NA	LOC_Os11g10250	expressed protein
Chr.11	5566535	5566535	G	A	G	SNP	OS11G0208600	Exonic	CDS	Nonsense	LOC_Os11g10250	expressed protein
Chr.11	5566998	5566998	G	A	G	SNP	OS11G0208600	Intronic	NA	NA	LOC_Os11g10250	expressed protein
Chr.11	5567482	5567482	G	A	G	SNP	OS11G0208600	Intronic	NA	NA	LOC_Os11g10250	expressed protein
Chr.11	6639428	6639428	G	A	G	SNP	OS11G0226933	Exonic	CDS	NA	LOC_Os11g11960	disease resistance protein RPM1, putative, expressed
Chr.11	6639511	6639511	G	A	G	SNP	OS11G0226933	Exonic	CDS	NA	LOC_Os11g11960	disease resistance protein RPM1, putative, expressed
Chr.11	6639615	6639615	G	A	G	SNP	OS11G0226933	Exonic	CDS	NA	LOC_Os11g11960	disease resistance protein RPM1, putative, expressed
Chr.11	6639812	6639812	G	A	G	SNP	OS11G0226933	Exonic	CDS	NA	LOC_Os11g11960	disease resistance protein RPM1, putative, expressed
Chr.11	6639835	6639835	G	A	G	SNP	OS11G0226933	Exonic	CDS	NA	LOC_Os11g11960	disease resistance protein RPM1, putative, expressed
Chr.11	7125285	7125285	G	A	G	SNP	OS11G0234200	Exonic	CDS	Missense	LOC_Os11g12650	PHD-finger domain containing protein, putative, expressed
Chr.11	7126841	7126841	G	A	G	SNP	OS11G0234200	Intronic	NA	NA	LOC_Os11g12650	PHD-finger domain containing protein, putative, expressed
Chr.11	10865211	10865211	G	A	G	SNP	OS11G0295900	Intronic	NA	NA	LOC_Os11g19060	BABY BOOM 1, putative, expressed
Chr.11	14049690	14049690	G	A	G	SNP	OS11G0434000	Intronic	NA	NA	LOC_Os11g24630	magnesium-dependent phosphatase 1, putative, expressed
Chr.11	15421471	15421471	G	A	G	SNP	OS11G0455500	Intronic	NA	NA	LOC_Os11g26850	erythronate-4-phosphate dehydrogenase, putative, expressed
Chr.11	17050427	17050427	G	A	G	SNP	OS11G0484300	Intronic	NA	NA	LOC_Os11g29380	MCM2 - Putative minichromosome maintenance MCM complex subunit 2, expressed
Chr.11	17509412	17509412	G	A	G	SNP	OS11G0493100	Exonic	CDS	Missense	LOC_Os11g30110	disease resistance RPP13-like protein 1, putative, expressed
Chr.11	18833542	18833542	G	A	G	SNP	OS11G0522900	Exonic	CDS	Silent	LOC_Os11g31980	OsSCP63 - Putative Serine Carboxypeptidase homologue, expressed
Chr.11	19675852	19675852	G	A	G	SNP	OS11G0539200	Intronic	NA	NA	LOC_Os11g33270	glycosyl hydrolases family 16, putative, expressed
Chr.11	19676028	19676028	G	A	G	SNP	OS11G0539200	Intronic	NA	NA	LOC_Os11g33270	glycosyl hydrolases family 16, putative, expressed
Chr.11	20185152	20185152	G	A	G	SNP	OS11G0547000	Exonic	5UTR	NA	LOC_Os11g34460	OsFBO10 - F-box and other domain containing protein, expressed
Chr.11	20518405	20518405	G	A	G	SNP	OS11G0551800	Exonic	3UTR	NA	LOC_Os11g35020	yippee zinc-binding protein, putative, expressed
Chr.11	20643822	20643822	G	A	G	SNP	OS11G0555300	Exonic	CDS	Missense	LOC_Os11g35210	NB-ARC domain containing protein, expressed
Chr.11	20643987	20643987	G	A	G	SNP	OS11G0555300	Exonic	CDS	Missense	LOC_Os11g35210	NB-ARC domain containing protein, expressed
Chr.11	20644154	20644154	G	A	G	SNP	OS11G0555300	Exonic	CDS	Silent	LOC_Os11g35210	NB-ARC domain containing protein, expressed
Chr.11	20644905	20644905	G	A	G	SNP	OS11G0555300	Exonic	CDS	Silent	LOC_Os11g35210	NB-ARC domain containing protein, expressed
Chr.11	20645210	20645210	G	A	G	SNP	OS11G0555300	Exonic	CDS	Missense	LOC_Os11g35210	NB-ARC domain containing protein, expressed
Chr.11	20645402	20645402	G	A	G	SNP	OS11G0555300	Exonic	CDS	Missense	LOC_Os11g35210	NB-ARC domain containing protein, expressed
Chr.11	20645700	20645700	G	A	G	SNP	OS11G0555300	Exonic	CDS	Silent	LOC_Os11g35210	NB-ARC domain containing protein, expressed
Chr.11	20646014	20646014	G	A	G	SNP	OS11G0555300	Exonic	CDS	Missense	LOC_Os11g35210	NB-ARC domain containing protein, expressed

Chr.11	21298309	21298309	G	A	G	SNP	OS11G0570000	Exonic	CDS	Silent	LOC_Os11g36200	receptor-like protein kinase 2 precursor, putative, expressed
Chr.11	21462948	21462948	C	A	C	SNP	OS11G0572200	Exonic	CDS	Missense	LOC_Os11g36400	expressed protein
Chr.11	21992131	21992131	G	A	G	SNP	OS11G0582100	Exonic	CDS	Missense	LOC_Os11g37230	zinc finger, C3HC4 type domain containing protein, expressed
Chr.11	21992229	21992229	G	A	G	SNP	OS11G0582100	Exonic	CDS	Silent	LOC_Os11g37230	zinc finger, C3HC4 type domain containing protein, expressed
Chr.11	22177594	22177594	G	A	G	SNP	OS11G0586300	Intronic	NA	NA	LOC_Os11g37560	expressed protein
Chr.11	22277370	22277370	G	A	G	SNP	OS11G0587600	Exonic	3UTR	NA	LOC_Os11g37700	pleiotropic drug resistance protein, putative, expressed
Chr.11	22279249	22279249	G	A	G	SNP	OS11G0587600	Intronic	NA	NA	LOC_Os11g37700	pleiotropic drug resistance protein, putative, expressed
Chr.11	22279281	22279281	G	A	G	SNP	OS11G0587600	Intronic	NA	NA	LOC_Os11g37700	pleiotropic drug resistance protein, putative, expressed
Chr.11	22511926	22511926	G	A	G	SNP	OS11G0592200	Exonic	CDS	Silent	LOC_Os11g37970	WIP5 - Wound-induced protein precursor, expressed
Chr.11	23185550	23185550	G	A	G	SNP	OS11G0602600	Intronic	NA	NA	LOC_Os11g38959	40S ribosomal protein S9-2, putative, expressed
Chr.11	23232246	23232246	G	A	G	SNP	OS11G0603200	Intronic	NA	NA	LOC_Os11g39020	ABC transporter, ATP-binding protein, putative, expressed
Chr.11	26878378	26878378	G	A	G	SNP	OS11G0666200	Exonic	CDS	Missense	LOC_Os11g44430	protein kinase, putative, expressed
Chr.11	26878445	26878445	G	A	G	SNP	OS11G0666200	Exonic	CDS	Missense	LOC_Os11g44430	protein kinase, putative, expressed
Chr.11	26878543	26878543	G	A	G	SNP	OS11G0666200	Exonic	CDS	Missense	LOC_Os11g44430	protein kinase, putative, expressed
Chr.11	26878644	26878644	G	A	G	SNP	OS11G0666200	Exonic	CDS	Silent	LOC_Os11g44430	protein kinase, putative, expressed
Chr.11	26879488	26879488	G	A	G	SNP	OS11G0666200	Intronic	NA	NA	LOC_Os11g44430	protein kinase, putative, expressed
Chr.11	28121240	28121240	G	A	G	SNP	OS11G0691100	Exonic	CDS	Missense	LOC_Os11g46860	wall-associated receptor kinase-like 4 precursor, putative, expressed
Chr.11	28934249	28934249	G	A	G	SNP	OS11G0707000	Intronic	NA	NA	LOC_Os11g47970	AAA-type ATPase family protein, putative, expressed
Chr.11	1225948	1225948	G	A	G	SNP	OS11G0126800	Intronic	NA	NA	LOC_Os11g03290	nucleoside-triphosphatase, putative, expressed
Chr.11	1226045	1226045	G	A	G	SNP	OS11G0126800	Exonic	CDS	NA	LOC_Os11g03290	nucleoside-triphosphatase, putative, expressed
Chr.11	1226116	1226116	G	A	G	SNP	OS11G0126800	Exonic	CDS	NA	LOC_Os11g03290	nucleoside-triphosphatase, putative, expressed
Chr.11	1226263	1226263	G	A	G	SNP	OS11G0126800	Intronic	NA	NA	LOC_Os11g03290	nucleoside-triphosphatase, putative, expressed
Chr.11	6890514	6890514	G	A	G	SNP	OS11G0229500	Intronic	NA	NA	LOC_Os11g12340	disease resistance protein RPM1, putative, expressed
Chr.11	23231832	23231832	G	A	G	SNP	OS11G0603200	Intronic	NA	NA	LOC_Os11g39020	ABC transporter, ATP-binding protein, putative, expressed
Chr.11	4972143	4972143	C	A	C	SNP	OS11G0199200	Intronic	NA	NA	LOC_Os11g09280	OsPDIL1-1 protein disulfide isomerase PDIL1-1, expressed
Chr.11	20045369	20045369	C	A	C	SNP	OS11G0545000	Exonic	CDS	Missense	LOC_Os11g34230	expressed protein
Chr.11	20046681	20046681	C	A	C	SNP	OS11G0545000	Intronic	NA	NA	LOC_Os11g34230	expressed protein
Chr.11	20645482	20645482	C	A	C	SNP	OS11G0555300	Exonic	CDS	Missense	LOC_Os11g35210	NB-ARC domain containing protein, expressed
Chr.11	20645532	20645532	C	A	C	SNP	OS11G0555300	Exonic	CDS	Silent	LOC_Os11g35210	NB-ARC domain containing protein, expressed
Chr.11	21299085	21299085	C	A	C	SNP	OS11G0570000	Exonic	CDS	Missense	LOC_Os11g36200	receptor-like protein kinase 2 precursor, putative, expressed
Chr.11	21992017	21992017	C	A	C	SNP	OS11G0582100	Exonic	3UTR	NA	LOC_Os11g37230	zinc finger, C3HC4 type domain containing protein, expressed
Chr.11	22176035	22176035	C	A	C	SNP	OS11G0586300	Exonic	CDS	NA	LOC_Os11g37560	expressed protein
Chr.11	22176681	22176681	C	A	C	SNP	OS11G0586300	Intronic	NA	NA	LOC_Os11g37560	expressed protein
Chr.11	22176686	22176686	C	A	C	SNP	OS11G0586300	Intronic	NA	NA	LOC_Os11g37560	expressed protein
Chr.11	26313261	26313261	C	A	C	SNP	OS11G0657300	Intronic	NA	NA	LOC_Os11g43620	LSM domain containing protein, expressed
Chr.11	26315116	26315116	C	A	C	SNP	OS11G0657300	Exonic	3UTR	NA	LOC_Os11g43620	LSM domain containing protein, expressed
Chr.11	26878566	26878566	C	A	C	SNP	OS11G0666200	Exonic	CDS	Silent	LOC_Os11g44430	protein kinase, putative, expressed
Chr.11	1659861	1659861	C	A	C	SNP	OS11G0135900	Exonic	CDS	Missense	LOC_Os11g04104	major facilitator superfamily antiporter, putative, expressed
Chr.11	1665103	1665103	C	A	C	SNP	OS11G0135900	Exonic	5UTR	NA	LOC_Os11g04104	major facilitator superfamily antiporter, putative, expressed
Chr.11	3740185	3740185	T	A	T	SNP	OS11G0175400	Exonic	CDS	Silent	LOC_Os11g07440	plant neutral invertase domain containing protein, expressed
Chr.11	3741109	3741109	G	A	G	SNP	OS11G0175400	Exonic	CDS	Silent	LOC_Os11g07440	plant neutral invertase domain containing protein, expressed
Chr.11	4248165	4248165	G	A	G	SNP	OS11G0183900	Intronic	NA	NA	LOC_Os11g08100	eukaryotic aspartyl protease domain containing protein, expressed
Chr.11	6323313	6323313	C	A	C	SNP	OS11G0220800	Exonic	CDS	Missense	LOC_Os11g11390	ribosomal protein, putative, expressed
Chr.11	6323890	6323890	T	A	T	SNP	OS11G0220800	Exonic	3UTR	NA	LOC_Os11g11390	ribosomal protein, putative, expressed
Chr.11	6890383	6890383	G	A	G	SNP	OS11G0229500	Intronic	NA	NA	LOC_Os11g12340	disease resistance protein RPM1, putative, expressed
Chr.11	6891015	6891015	C	A	C	SNP	OS11G0229500	Intronic	NA	NA	LOC_Os11g12340	disease resistance protein RPM1, putative, expressed
Chr.11	6894921	6894921	G	A	G	SNP	OS11G0229500	Exonic	CDS	Missense	LOC_Os11g12340	disease resistance protein RPM1, putative, expressed
Chr.11	6895595	6895595	G	A	G	SNP	OS11G0229500	Exonic	CDS	Missense	LOC_Os11g12340	disease resistance protein RPM1, putative, expressed
Chr.11	10134634	10134634	G	A	G	SNP	OS11G0283500	Intronic	NA	NA	LOC_Os11g18044	peptide transporter PTR2, putative, expressed
Chr.11	10134748	10134748	G	A	G	SNP	OS11G0283500	Intronic	NA	NA	LOC_Os11g18044	peptide transporter PTR2, putative, expressed
Chr.11	10134965	10134965	G	A	G	SNP	OS11G0283500	Intronic	NA	NA	LOC_Os11g18044	peptide transporter PTR2, putative, expressed
Chr.11	10141007	10141007	G	A	G	SNP	OS11G0283500	Intronic	NA	NA	LOC_Os11g18044	peptide transporter PTR2, putative, expressed
Chr.11	10141287	10141287	G	A	G	SNP	OS11G0283500	Intronic	NA	NA	LOC_Os11g18044	peptide transporter PTR2, putative, expressed
Chr.11	10142169	10142169	T	A	T	SNP	OS11G0283500	Intronic	NA	NA	LOC_Os11g18044	peptide transporter PTR2, putative, expressed
Chr.11	10142675	10142675	G	A	G	SNP	OS11G0283500	Intronic	NA	NA	LOC_Os11g18044	peptide transporter PTR2, putative, expressed
Chr.11	10142756	10142756	G	A	G	SNP	OS11G0283500	Intronic	NA	NA	LOC_Os11g18044	peptide transporter PTR2, putative, expressed
Chr.11	10142870	10142870	C	A	C	SNP	OS11G0283500	Intronic	NA	NA	LOC_Os11g18044	peptide transporter PTR2, putative, expressed
Chr.11	14051504	14051504	G	A	G	SNP	OS11G0434000	Intronic	NA	NA	LOC_Os11g24630	magnesium-dependent phosphatase 1, putative, expressed
Chr.11	14739063	14739063	G	A	G	SNP	OS11G0445300	Exonic	CDS	Silent	LOC_Os11g25860	protein Kinase, putative, expressed
Chr.11	15420458	15420458	G	A	G	SNP	OS11G0455500	Exonic	3UTR	NA	LOC_Os11g26850	erythronate-4-phosphate dehydrogenase, putative, expressed
Chr.11	15421252	15421252	G	A	G	SNP	OS11G0455500	Exonic	CDS	NA	LOC_Os11g26850	erythronate-4-phosphate dehydrogenase, putative, expressed
Chr.11	17235383	17235383	G	A	G	SNP	OS11G0489250	Intronic	NA	NA	LOC_Os11g29720	cytochrome P450, putative, expressed
Chr.11	17236664	17236664	T	A	T	SNP	OS11G0489250	Intronic	NA	NA	LOC_Os11g29720	cytochrome P450, putative, expressed
Chr.11	17353712	17353712	C	A	C	SNP	OS11G0490900	Intronic	NA	NA	LOC_Os11g29870	WRKY72, expressed
Chr.11	17354813	17354813	C	A	C	SNP	OS11G0490900	Intronic	NA	NA	LOC_Os11g29870	WRKY72, expressed
Chr.11	17355117	17355117	T	A	T	SNP	OS11G0490900	Intronic	NA	NA	LOC_Os11g29870	WRKY72, expressed
Chr.11	19181437	19181437	T	A	T	SNP	OS11G0528500	Intronic	NA	NA	LOC_Os11g32500	rubredoxin family protein, putative, expressed
Chr.11	20046287	20046287	G	A	G	SNP	OS11G0545000	Intronic	NA	NA	LOC_Os11g34230	expressed protein
Chr.11	20048393	20048393	C	A	C	SNP	OS11G0545000	Exonic	5UTR	NA	LOC_Os11g34230	expressed protein
Chr.11	20182996	20182996	G	A	G	SNP	OS11G0547000	Exonic	CDS	Silent	LOC_Os11g34460	OsFBO10 - F-box and other domain containing protein, expressed
Chr.11	20183341	20183341	G	A	G	SNP	OS11G0547000	Exonic	CDS	Silent	LOC_Os11g34460	OsFBO10 - F-box and other domain containing protein, expressed
Chr.11	20183548	20183548	G	A	G	SNP	OS11G0547000	Exonic	CDS	Silent	LOC_Os11g34460	OsFBO10 - F-box and other domain containing protein, expressed
Chr.11	20183569	20183569	G	A	G	SNP	OS11G0547000	Exonic	CDS	Silent	LOC_Os11g34460	OsFBO10 - F-box and other domain containing protein, expressed
Chr.11	20184618	20184618	C	A	C	SNP	OS11G0547000	Exonic	5UTR	NA	LOC_Os11g34460	OsFBO10 - F-box and other domain containing protein, expressed
Chr.11	20184802	20184802	G	A	G	SNP	OS11G0547000	Exonic	5UTR	NA	LOC_Os11g34460	OsFBO10 - F-box and other domain containing protein, expressed
Chr.11	20186231	20186231	G	A	G	SNP	OS11G0547000	Exonic	5UTR	NA	LOC_Os11g34460	OsFBO10 - F-box and other domain containing protein, expressed
Chr.11	21892118	21892118	G	A	G	SNP	OS11G0579800	Exonic	CDS	Silent	LOC_Os11g37080	h/ACA ribonucleoprotein complex subunit 1-like protein 1, putative, expressed
Chr.11	21892124	21892124	G	A	G	SNP	OS11G0579800	Exonic	CDS	Silent	LOC_Os11g37080	h/ACA ribonucleoprotein complex subunit 1-like protein 1, putative, expressed
Chr.11	21892153	21892153	C	A	C	SNP	OS11G0579800	Exonic	3UTR	NA	LOC_Os11g37080	h/ACA ribonucleoprotein complex subunit 1-like protein 1, putative, expressed
Chr.11	21892165	21892165	G	A	G	SNP	OS11G0579800	Exonic	3UTR	NA	LOC_Os11g37080	h/ACA ribonucleoprotein complex subunit 1-like protein 1, putative, expressed
Chr.11	21892227	21892227	G	A	G	SNP	OS11G0579800	Exonic	3UTR	NA	LOC_Os11g37080	h/ACA ribonucleoprotein complex subunit 1-like protein 1, putative, expressed
Chr.11	22176507	22176507	C	A	C	SNP	OS11G0586300	Exonic	CDS	NA	LOC_Os11g37560	expressed protein
Chr.11	27488601	27488601	C	A	C	SNP	OS11G0679700	Intronic	NA	NA	LOC_Os11g45400	glycerol-3-phosphate acyltransferase, putative, expressed
Chr.11	27488855	27488855	G	A	G	SNP	OS11G0679700	Intronic	NA	NA	LOC_Os11g45400	glycerol-3-phosphate acyltransferase, putative, expressed
Chr.11	27490042	27490042	C	A	C	SNP	OS11G0679700	Exonic	3UTR	NA	LOC_Os11g45400	glycerol-3-phosphate acyltransferase, putative, expressed

Chr.11	1225717	1225717	G	A	G	SNP	OS11G0126800	Exonic	CDS	NA	LOC_Os11g03290	nucleoside-triphosphatase, putative, expressed
Chr.11	1226164	1226164	T	A	T	SNP	OS11G0126800	Exonic	CDS	NA	LOC_Os11g03290	nucleoside-triphosphatase, putative, expressed
Chr.11	1226183	1226183	G	A	G	SNP	OS11G0126800	Exonic	CDS	NA	LOC_Os11g03290	nucleoside-triphosphatase, putative, expressed
Chr.11	1226313	1226313	T	A	T	SNP	OS11G0126800	Intronic	NA	NA	LOC_Os11g03290	nucleoside-triphosphatase, putative, expressed
Chr.11	1226426	1226426	G	A	G	SNP	OS11G0126800	Exonic	CDS	NA	LOC_Os11g03290	nucleoside-triphosphatase, putative, expressed
Chr.11	1226528	1226528	G	A	G	SNP	OS11G0126800	Exonic	3UTR	NA	LOC_Os11g03290	nucleoside-triphosphatase, putative, expressed
Chr.11	1226531	1226531	T	A	T	SNP	OS11G0126800	Exonic	3UTR	NA	LOC_Os11g03290	nucleoside-triphosphatase, putative, expressed
Chr.11	4972498	4972498	T	A	T	SNP	OS11G0199200	Intronic	NA	NA	LOC_Os11g09280	OsPDIL1-1 protein disulfide isomerase PDIL1-1, expressed
Chr.11	4972611	4972611	C	A	C	SNP	OS11G0199200	Exonic	CDS	Missense	LOC_Os11g09280	OsPDIL1-1 protein disulfide isomerase PDIL1-1, expressed
Chr.11	4973833	4973833	G	A	G	SNP	OS11G0199200	Exonic	CDS	Silent	LOC_Os11g09280	OsPDIL1-1 protein disulfide isomerase PDIL1-1, expressed
Chr.11	4974342	4974342	T	A	T	SNP	OS11G0199200	Intronic	NA	NA	LOC_Os11g09280	OsPDIL1-1 protein disulfide isomerase PDIL1-1, expressed
Chr.11	4974895	4974895	G	A	G	SNP	OS11G0199200	Exonic	CDS	Silent	LOC_Os11g09280	OsPDIL1-1 protein disulfide isomerase PDIL1-1, expressed
Chr.11	5565715	5565715	G	A	G	SNP	OS11G0208600	Exonic	3UTR	NA	LOC_Os11g10250	expressed protein
Chr.11	5587654	5587654	C	A	C	SNP	OS11G0208800	Exonic	CDS	Silent	LOC_Os11g10290	S-domain receptor-like protein kinase, putative, expressed
Chr.11	6639580	6639580	T	A	T	SNP	OS11G0226933	Exonic	CDS	NA	LOC_Os11g11960	disease resistance protein RPM1, putative, expressed
Chr.11	6889546	6889546	C	A	C	SNP	OS11G0229500	Intronic	NA	NA	LOC_Os11g12340	disease resistance protein RPM1, putative, expressed
Chr.11	6890060	6890060	G	A	G	SNP	OS11G0229500	Intronic	NA	NA	LOC_Os11g12340	disease resistance protein RPM1, putative, expressed
Chr.11	7127913	7127913	G	A	G	SNP	OS11G0234200	Intronic	NA	NA	LOC_Os11g12650	PHD-finger domain containing protein, putative, expressed
Chr.11	7128023	7128023	T	A	T	SNP	OS11G0234200	Intronic	NA	NA	LOC_Os11g12650	PHD-finger domain containing protein, putative, expressed
Chr.11	7128084	7128084	G	A	G	SNP	OS11G0234200	Intronic	NA	NA	LOC_Os11g12650	PHD-finger domain containing protein, putative, expressed
Chr.11	7128130	7128130	G	A	G	SNP	OS11G0234200	Intronic	NA	NA	LOC_Os11g12650	PHD-finger domain containing protein, putative, expressed
Chr.11	10863861	10863861	G	A	G	SNP	OS11G0295900	Exonic	CDS	Silent	LOC_Os11g19060	BABY BOOM 1, putative, expressed
Chr.11	10863941	10863941	G	A	G	SNP	OS11G0295900	Exonic	CDS	Missense	LOC_Os11g19060	BABY BOOM 1, putative, expressed
Chr.11	10865197	10865197	G	A	G	SNP	OS11G0295900	Intronic	NA	NA	LOC_Os11g19060	BABY BOOM 1, putative, expressed
Chr.11	11596059	11596059	C	A	C	SNP	OS11G0306400	Exonic	CDS	Missense	LOC_Os11g20090	O-methyltransferase, putative, expressed
Chr.11	14052352	14052352	C	A	C	SNP	OS11G0434000	Exonic	CDS	Missense	LOC_Os11g24630	magnesium-dependent phosphatase 1, putative, expressed
Chr.11	14052435	14052435	C	A	C	SNP	OS11G0434000	Exonic	5UTR	NA	LOC_Os11g24630	magnesium-dependent phosphatase 1, putative, expressed
Chr.11	15422317	15422317	G	A	G	SNP	OS11G0455500	Exonic	CDS	NA	LOC_Os11g26850	erythronate-4-phosphate dehydrogenase, putative, expressed
Chr.11	17235679	17235679	G	A	G	SNP	OS11G0489250	Intronic	NA	NA	LOC_Os11g29720	cytochrome P450, putative, expressed
Chr.11	17284438	17284438	C	A	C	SNP	OS11G0490100	Exonic	CDS	Missense	LOC_Os11g29780	plant-specific domain TIGR01627 family protein, expressed
Chr.11	19675900	19675900	G	A	G	SNP	OS11G0539200	Intronic	NA	NA	LOC_Os11g33270	glycosyl hydrolases family 16, putative, expressed
Chr.11	19676101	19676101	G	A	G	SNP	OS11G0539200	Intronic	NA	NA	LOC_Os11g33270	glycosyl hydrolases family 16, putative, expressed
Chr.11	20182756	20182756	C	A	C	SNP	OS11G0547000	Exonic	CDS	Silent	LOC_Os11g34460	OsFBO10 - F-box and other domain containing protein, expressed
Chr.11	20182774	20182774	G	A	G	SNP	OS11G0547000	Exonic	CDS	Silent	LOC_Os11g34460	OsFBO10 - F-box and other domain containing protein, expressed
Chr.11	20184779	20184779	C	A	C	SNP	OS11G0547000	Exonic	5UTR	NA	LOC_Os11g34460	OsFBO10 - F-box and other domain containing protein, expressed
Chr.11	20186856	20186856	C	A	C	SNP	OS11G0547000	Intronic	NA	NA	LOC_Os11g34460	OsFBO10 - F-box and other domain containing protein, expressed
Chr.11	20187034	20187034	G	A	G	SNP	OS11G0547000	Exonic	CDS	Silent	LOC_Os11g34460	OsFBO10 - F-box and other domain containing protein, expressed
Chr.11	20187064	20187064	C	A	C	SNP	OS11G0547000	Exonic	CDS	Silent	LOC_Os11g34460	OsFBO10 - F-box and other domain containing protein, expressed
Chr.11	20643585	20643585	G	A	G	SNP	OS11G0555300	Exonic	CDS	Missense	LOC_Os11g35210	NB-ARC domain containing protein, expressed
Chr.11	20644172	20644172	G	A	G	SNP	OS11G0555300	Exonic	CDS	Silent	LOC_Os11g35210	NB-ARC domain containing protein, expressed
Chr.11	20702857	20702857	G	A	G	SNP	OS11G0557000	Intronic	NA	NA	LOC_Os11g35310	phytosulfokines precursor, putative, expressed
Chr.11	21891093	21891093	C	A	C	SNP	OS11G0579800	Intronic	NA	NA	LOC_Os11g37080	h/ACA ribonucleoprotein complex subunit 1-like protein 1, putative, expressed
Chr.11	21891103	21891103	T	A	T	SNP	OS11G0579800	Intronic	NA	NA	LOC_Os11g37080	h/ACA ribonucleoprotein complex subunit 1-like protein 1, putative, expressed
Chr.11	21891207	21891207	T	A	T	SNP	OS11G0579800	Intronic	NA	NA	LOC_Os11g37080	h/ACA ribonucleoprotein complex subunit 1-like protein 1, putative, expressed
Chr.11	21891260	21891260	C	A	C	SNP	OS11G0579800	Intronic	NA	NA	LOC_Os11g37080	h/ACA ribonucleoprotein complex subunit 1-like protein 1, putative, expressed
Chr.11	21891914	21891914	G	A	G	SNP	OS11G0579800	Intronic	NA	NA	LOC_Os11g37080	h/ACA ribonucleoprotein complex subunit 1-like protein 1, putative, expressed
Chr.11	21891941	21891941	G	A	G	SNP	OS11G0579800	Intronic	NA	NA	LOC_Os11g37080	h/ACA ribonucleoprotein complex subunit 1-like protein 1, putative, expressed
Chr.11	21891953	21891953	C	A	C	SNP	OS11G0579800	Intronic	NA	NA	LOC_Os11g37080	h/ACA ribonucleoprotein complex subunit 1-like protein 1, putative, expressed
Chr.11	21891955	21891955	C	A	C	SNP	OS11G0579800	Intronic	NA	NA	LOC_Os11g37080	h/ACA ribonucleoprotein complex subunit 1-like protein 1, putative, expressed
Chr.11	21892070	21892070	G	A	G	SNP	OS11G0579800	Exonic	CDS	Silent	LOC_Os11g37080	h/ACA ribonucleoprotein complex subunit 1-like protein 1, putative, expressed
Chr.11	21892079	21892079	C	A	C	SNP	OS11G0579800	Exonic	CDS	Silent	LOC_Os11g37080	h/ACA ribonucleoprotein complex subunit 1-like protein 1, putative, expressed
Chr.11	21892376	21892376	C	A	C	SNP	OS11G0579800	Exonic	3UTR	NA	LOC_Os11g37080	h/ACA ribonucleoprotein complex subunit 1-like protein 1, putative, expressed
Chr.11	22175945	22175945	T	A	T	SNP	OS11G0586300	Exonic	CDS	NA	LOC_Os11g37560	expressed protein
Chr.11	22176242	22176242	C	A	C	SNP	OS11G0586300	Intronic	NA	NA	LOC_Os11g37560	expressed protein
Chr.11	22176719	22176719	C	A	C	SNP	OS11G0586300	Intronic	NA	NA	LOC_Os11g37560	expressed protein
Chr.11	22178401	22178401	C	A	C	SNP	OS11G0586300	Exonic	CDS	NA	LOC_Os11g37560	expressed protein
Chr.11	22178644	22178644	G	A	G	SNP	OS11G0586300	Intronic	NA	NA	LOC_Os11g37560	expressed protein
Chr.11	22178840	22178840	G	A	G	SNP	OS11G0586300	Exonic	CDS	NA	LOC_Os11g37560	expressed protein
Chr.11	22280624	22280624	T	A	T	SNP	OS11G0587600	Intronic	NA	NA	LOC_Os11g37700	pleiotropic drug resistance protein, putative, expressed
Chr.11	22282291	22282291	G	A	G	SNP	OS11G0587600	Exonic	CDS	Missense	LOC_Os11g37700	pleiotropic drug resistance protein, putative, expressed
Chr.11	22502868	22502868	G	A	G	SNP	OS11G0592000	Exonic	3UTR	NA	LOC_Os11g37950	WIP3 - Wound-induced protein precursor, expressed
Chr.11	23231711	23231711	G	A	G	SNP	OS11G0603200	Exonic	CDS	Silent	LOC_Os11g39020	ABC transporter, ATP-binding protein, putative, expressed
Chr.11	23231748	23231748	G	A	G	SNP	OS11G0603200	Exonic	CDS	Missense	LOC_Os11g39020	ABC transporter, ATP-binding protein, putative, expressed
Chr.11	23231752	23231752	G	A	G	SNP	OS11G0603200	Exonic	CDS	Missense	LOC_Os11g39020	ABC transporter, ATP-binding protein, putative, expressed
Chr.11	23232057	23232057	T	A	T	SNP	OS11G0603200	Exonic	CDS	Silent	LOC_Os11g39020	ABC transporter, ATP-binding protein, putative, expressed
Chr.11	23232199	23232199	G	A	G	SNP	OS11G0603200	Intronic	NA	NA	LOC_Os11g39020	ABC transporter, ATP-binding protein, putative, expressed
Chr.11	23459891	23459891	C	A	C	SNP	OS11G0607900	Exonic	3UTR	NA	LOC_Os11g39420	jacalin-like lectin domain containing protein, expressed
Chr.11	26878095	26878095	T	A	T	SNP	OS11G0666200	Exonic	CDS	Silent	LOC_Os11g44430	protein kinase, putative, expressed
Chr.11	26878179	26878179	G	A	G	SNP	OS11G0666200	Exonic	CDS	Silent	LOC_Os11g44430	protein kinase, putative, expressed
Chr.11	26878283	26878283	G	A	G	SNP	OS11G0666200	Exonic	CDS	Silent	LOC_Os11g44430	protein kinase, putative, expressed
Chr.11	26878291	26878291	G	A	G	SNP	OS11G0666200	Exonic	CDS	Missense	LOC_Os11g44430	protein kinase, putative, expressed
Chr.11	26878302	26878302	C	A	C	SNP	OS11G0666200	Exonic	CDS	Missense	LOC_Os11g44430	protein kinase, putative, expressed
Chr.11	27202603	27202603	C	A	C	SNP	OS11G0673100	Exonic	CDS	Missense	LOC_Os11g44940	seed maturation protein PM27, putative, expressed
Chr.11	27487680	27487680	G	A	G	SNP	OS11G0679700	Intronic	NA	NA	LOC_Os11g45400	glycerol-3-phosphate acyltransferase, putative, expressed
Chr.11	27487716	27487716	G	A	G	SNP	OS11G0679700	Intronic	NA	NA	LOC_Os11g45400	glycerol-3-phosphate acyltransferase, putative, expressed
Chr.11	27488773	27488773	C	A	C	SNP	OS11G0679700	Intronic	NA	NA	LOC_Os11g45400	glycerol-3-phosphate acyltransferase, putative, expressed
Chr.11	27488941	27488941	G	A	G	SNP	OS11G0679700	Intronic	NA	NA	LOC_Os11g45400	glycerol-3-phosphate acyltransferase, putative, expressed
Chr.11	27489053	27489053	G	A	G	SNP	OS11G0679700	Intronic	NA	NA	LOC_Os11g45400	glycerol-3-phosphate acyltransferase, putative, expressed
Chr.11	27489069	27489069	T	A	T	SNP	OS11G0679700	Intronic	NA	NA	LOC_Os11g45400	glycerol-3-phosphate acyltransferase, putative, expressed
Chr.11	27489194	27489194	G	A	G	SNP	OS11G0679700	Exonic	CDS	NA	LOC_Os11g45400	glycerol-3-phosphate acyltransferase, putative, expressed
Chr.11	28956671	28956671	C	A	C	SNP	OS11G0707600	Intronic	NA	NA	LOC_Os11g48020	fatty acid hydroxylase, putative, expressed
Chr.4	11272527	11272527	C	A	C	SNP	OS04G0270100	Exonic	5UTR	NA	LOC_Os04g20220	elongation factor Tu, putative, expressed
Chr.4	32168337	32168337	G	A	G	SNP	OS04G0631600	Exonic	CDS	Silent	LOC_Os04g53990	ethylene-responsive protein related, putative, expressed
Chr.4	7309763	7309763	T	A	T	SNP	OS04G0209200	Intronic	NA	NA	LOC_Os04g13210	multidrug resistance-associated protein, putative, expressed
Chr.4	11240755	11240755	T	A	T	SNP	OS04G0269600	Intronic	NA	NA	LOC_Os04g20164	amine oxidase precursor, putative, expressed

Chr.4	18213672	18213672	T	A	T	SNP	OS04G0373400	Intronic	NA	NA	LOC_Os04g30490	MATE efflux family protein, putative, expressed
Chr.4	18215533	18215533	T	A	T	SNP	OS04G0373400	Intronic	NA	NA	LOC_Os04g30490	MATE efflux family protein, putative, expressed
Chr.4	18383273	18383273	T	A	T	SNP	OS04G0376300	Intronic	NA	NA	LOC_Os04g30760	3-oxoacyl-reductase, chloroplast precursor, putative, expressed
Chr.4	18562142	18562142	T	A	T	SNP	OS04G0379900	Intronic	NA	NA	LOC_Os04g31070	acyl-desaturase, chloroplast precursor, putative, expressed
Chr.4	18603752	18603752	T	A	T	SNP	OS04G0380300	Intronic	NA	NA	LOC_Os04g31120	OsFBK14 - F-box domain and kelch repeat containing protein, expressed
Chr.4	18607734	18607734	T	A	T	SNP	OS04G0380300	Intronic	NA	NA	LOC_Os04g31120	OsFBK14 - F-box domain and kelch repeat containing protein, expressed
Chr.4	19600114	19600114	T	A	T	SNP	OS04G0397000	Intronic	NA	NA	LOC_Os04g32550	defender against cell death 1, putative, expressed
Chr.4	22022385	22022385	T	A	T	SNP	OS04G0442200	Intronic	NA	NA	LOC_Os04g36062	expressed protein
Chr.4	25666917	25666917	T	A	T	SNP	OS04G0513400	Exonic	3UTR	NA	LOC_Os04g43390	Os4bglu16 - monolignol beta-glucoside homologue, expressed
Chr.4	32146164	32146164	T	A	T	SNP	OS04G0631200	Intronic	NA	NA	LOC_Os04g53950	glycosyl hydrolases family 16 protein, protein, expressed
Chr.4	32146266	32146266	T	A	T	SNP	OS04G0631200	Intronic	NA	NA	LOC_Os04g53950	glycosyl hydrolases family 16 protein, protein, expressed
Chr.4	32584692	32584692	T	A	T	SNP	OS04G0640600	Exonic	5UTR	NA	LOC_Os04g54800	shikimate kinase, putative, expressed
Chr.4	318179	318179	G	A	G	SNP	OS04G0104900	Intronic	NA	NA	LOC_Os04g01470	O-methyltransferase, putative, expressed
Chr.4	318636	318636	G	A	G	SNP	OS04G0104900	Intronic	NA	NA	LOC_Os04g01470	O-methyltransferase, putative, expressed
Chr.4	318997	318997	G	A	G	SNP	OS04G0104900	Exonic	CDS	Missense	LOC_Os04g01470	O-methyltransferase, putative, expressed
Chr.4	358147	358147	G	A	G	SNP	OS04G0105400	Intronic	NA	NA	LOC_Os04g01520	expressed protein
Chr.4	617907	617907	G	A	G	SNP	OS04G0110600	Exonic	3UTR	NA	LOC_Os04g02000	zinc finger family protein, putative, expressed
Chr.4	4458412	4458412	G	A	G	SNP	OS04G0165700	Intronic	NA	NA	LOC_Os04g08350	cysteine synthase, chloroplast/chromoplast precursor, putative, expressed
Chr.4	5319617	5319617	G	A	G	SNP	OS04G0178300	Intronic	NA	NA	LOC_Os04g09900	ent-kaurene synthase, chloroplast precursor, putative, expressed
Chr.4	5320332	5320332	G	A	G	SNP	OS04G0178300	Exonic	CDS	NA	LOC_Os04g09900	ent-kaurene synthase, chloroplast precursor, putative, expressed
Chr.4	7308000	7308000	G	A	G	SNP	OS04G0209200	Intronic	NA	NA	LOC_Os04g13210	multidrug resistance-associated protein, putative, expressed
Chr.4	7308419	7308419	G	A	G	SNP	OS04G0209200	Intronic	NA	NA	LOC_Os04g13210	multidrug resistance-associated protein, putative, expressed
Chr.4	7308610	7308610	G	A	G	SNP	OS04G0209200	Intronic	NA	NA	LOC_Os04g13210	multidrug resistance-associated protein, putative, expressed
Chr.4	7308698	7308698	G	A	G	SNP	OS04G0209200	Intronic	NA	NA	LOC_Os04g13210	multidrug resistance-associated protein, putative, expressed
Chr.4	7308790	7308790	G	A	G	SNP	OS04G0209200	Intronic	NA	NA	LOC_Os04g13210	multidrug resistance-associated protein, putative, expressed
Chr.4	7309096	7309096	G	A	G	SNP	OS04G0209200	Intronic	NA	NA	LOC_Os04g13210	multidrug resistance-associated protein, putative, expressed
Chr.4	7309471	7309471	G	A	G	SNP	OS04G0209200	Intronic	NA	NA	LOC_Os04g13210	multidrug resistance-associated protein, putative, expressed
Chr.4	7309565	7309565	G	A	G	SNP	OS04G0209200	Intronic	NA	NA	LOC_Os04g13210	multidrug resistance-associated protein, putative, expressed
Chr.4	7309566	7309566	G	A	G	SNP	OS04G0209200	Intronic	NA	NA	LOC_Os04g13210	multidrug resistance-associated protein, putative, expressed
Chr.4	11238672	11238672	G	A	G	SNP	OS04G0269600	Exonic	5UTR	NA	LOC_Os04g20164	amine oxidase precursor, putative, expressed
Chr.4	18214526	18214526	G	A	G	SNP	OS04G0373400	Intronic	NA	NA	LOC_Os04g30490	MATE efflux family protein, putative, expressed
Chr.4	18214591	18214591	G	A	G	SNP	OS04G0373400	Intronic	NA	NA	LOC_Os04g30490	MATE efflux family protein, putative, expressed
Chr.4	18215646	18215646	G	A	G	SNP	OS04G0373400	Intronic	NA	NA	LOC_Os04g30490	MATE efflux family protein, putative, expressed
Chr.4	18560741	18560741	G	A	G	SNP	OS04G0379900	Exonic	CDS	Silent	LOC_Os04g31070	acyl-desaturase, chloroplast precursor, putative, expressed
Chr.4	18560876	18560876	G	A	G	SNP	OS04G0379900	Exonic	CDS	Silent	LOC_Os04g31070	acyl-desaturase, chloroplast precursor, putative, expressed
Chr.4	18561080	18561080	G	A	G	SNP	OS04G0379900	Exonic	CDS	Silent	LOC_Os04g31070	acyl-desaturase, chloroplast precursor, putative, expressed
Chr.4	18562047	18562047	G	A	G	SNP	OS04G0379900	Exonic	CDS	Silent	LOC_Os04g31070	acyl-desaturase, chloroplast precursor, putative, expressed
Chr.4	18562116	18562116	G	A	G	SNP	OS04G0379900	Intronic	NA	NA	LOC_Os04g31070	acyl-desaturase, chloroplast precursor, putative, expressed
Chr.4	18562248	18562248	G	A	G	SNP	OS04G0379900	Intronic	NA	NA	LOC_Os04g31070	acyl-desaturase, chloroplast precursor, putative, expressed
Chr.4	18602898	18602898	G	A	G	SNP	OS04G0380300	Exonic	3UTR	NA	LOC_Os04g31120	OsFBK14 - F-box domain and kelch repeat containing protein, expressed
Chr.4	18602914	18602914	G	A	G	SNP	OS04G0380300	Exonic	3UTR	NA	LOC_Os04g31120	OsFBK14 - F-box domain and kelch repeat containing protein, expressed
Chr.4	18603291	18603291	G	A	G	SNP	OS04G0380300	Exonic	CDS	Silent	LOC_Os04g31120	OsFBK14 - F-box domain and kelch repeat containing protein, expressed
Chr.4	18604400	18604400	G	A	G	SNP	OS04G0380300	Exonic	5UTR	NA	LOC_Os04g31120	OsFBK14 - F-box domain and kelch repeat containing protein, expressed
Chr.4	18605377	18605377	G	A	G	SNP	OS04G0380300	Intronic	NA	NA	LOC_Os04g31120	OsFBK14 - F-box domain and kelch repeat containing protein, expressed
Chr.4	18605664	18605664	G	A	G	SNP	OS04G0380300	Intronic	NA	NA	LOC_Os04g31120	OsFBK14 - F-box domain and kelch repeat containing protein, expressed
Chr.4	18606324	18606324	G	A	G	SNP	OS04G0380300	Exonic	5UTR	NA	LOC_Os04g31120	OsFBK14 - F-box domain and kelch repeat containing protein, expressed
Chr.4	18607686	18607686	G	A	G	SNP	OS04G0380300	Intronic	NA	NA	LOC_Os04g31120	OsFBK14 - F-box domain and kelch repeat containing protein, expressed
Chr.4	18607691	18607691	G	A	G	SNP	OS04G0380300	Intronic	NA	NA	LOC_Os04g31120	OsFBK14 - F-box domain and kelch repeat containing protein, expressed
Chr.4	19599116	19599116	G	A	G	SNP	OS04G0397000	Intronic	NA	NA	LOC_Os04g32550	defender against cell death 1, putative, expressed
Chr.4	21753291	21753291	G	A	G	SNP	OS04G0437600	Exonic	CDS	Silent	LOC_Os04g35700	STE_MEKK_ste11_MAP3K.16 - STE kinases include homologs to sterile 7, sterile 11 and sterile 20 from yeast, expressed
Chr.4	21753539	21753539	G	A	G	SNP	OS04G0437600	Intronic	NA	NA	LOC_Os04g35700	STE_MEKK_ste11_MAP3K.16 - STE kinases include homologs to sterile 7, sterile 11 and sterile 20 from yeast, expressed
Chr.4	21753826	21753826	G	A	G	SNP	OS04G0437600	Intronic	NA	NA	LOC_Os04g35700	STE_MEKK_ste11_MAP3K.16 - STE kinases include homologs to sterile 7, sterile 11 and sterile 20 from yeast, expressed
Chr.4	21756551	21756551	G	A	G	SNP	OS04G0437600	Intronic	NA	NA	LOC_Os04g35700	STE_MEKK_ste11_MAP3K.16 - STE kinases include homologs to sterile 7, sterile 11 and sterile 20 from yeast, expressed
Chr.4	21757708	21757708	G	A	G	SNP	OS04G0437600	Intronic	NA	NA	LOC_Os04g35700	STE_MEKK_ste11_MAP3K.16 - STE kinases include homologs to sterile 7, sterile 11 and sterile 20 from yeast, expressed
Chr.4	22020981	22020981	G	A	G	SNP	OS04G0442200	Intronic	NA	NA	LOC_Os04g36062	expressed protein
Chr.4	22022136	22022136	G	A	G	SNP	OS04G0442200	Intronic	NA	NA	LOC_Os04g36062	expressed protein
Chr.4	22023941	22023941	G	A	G	SNP	OS04G0442200	Exonic	3UTR	NA	LOC_Os04g36062	expressed protein
Chr.4	22450064	22450064	G	A	G	SNP	OS04G0450600	Intronic	NA	NA	LOC_Os04g37760	expressed protein
Chr.4	22916964	22916964	G	A	G	SNP	OS04G0459000	Intronic	NA	NA	LOC_Os04g38570	multidrug resistance protein, putative, expressed
Chr.4	31240671	31240671	G	A	G	SNP	OS04G0615700	Exonic	CDS	Silent	LOC_Os04g52540	retrotransposon protein, putative, unclassified, expressed
Chr.4	31671647	31671647	G	A	G	SNP	OS04G0623300	Intronic	NA	NA	LOC_Os04g53190	CPuORF12 - conserved peptide uORF-containing transcript, expressed
Chr.4	32384876	32384876	G	A	G	SNP	OS04G0636800	Intronic	NA	NA	LOC_Os04g54430	immediate-early protein RSP40, putative, expressed
Chr.4	32386647	32386647	G	A	G	SNP	OS04G0636800	Intronic	NA	NA	LOC_Os04g54430	immediate-early protein RSP40, putative, expressed
Chr.4	32583821	32583821	G	A	G	SNP	OS04G0640600	Intronic	NA	NA	LOC_Os04g54800	shikimate kinase, putative, expressed
Chr.4	32585039	32585039	G	A	G	SNP	OS04G0640600	Intronic	NA	NA	LOC_Os04g54800	shikimate kinase, putative, expressed
Chr.4	34903178	34903178	G	A	G	SNP	OS04G0683600	Exonic	CDS	Silent	LOC_Os04g58700	expressed protein
Chr.4	11278968	11278968	G	A	G	SNP	OS04G0270100	Intronic	NA	NA	LOC_Os04g20220	elongation factor Tu, putative, expressed
Chr.4	5323153	5323153	C	A	C	SNP	OS04G0178300	Exonic	CDS	Silent	LOC_Os04g09900	ent-kaurene synthase, chloroplast precursor, putative, expressed
Chr.4	7308653	7308653	C	A	C	SNP	OS04G0209200	Intronic	NA	NA	LOC_Os04g13210	multidrug resistance-associated protein, putative, expressed
Chr.4	11278931	11278931	C	A	C	SNP	OS04G0270100	Intronic	NA	NA	LOC_Os04g20220	elongation factor Tu, putative, expressed
Chr.4	18214796	18214796	C	A	C	SNP	OS04G0373400	Intronic	NA	NA	LOC_Os04g30490	MATE efflux family protein, putative, expressed
Chr.4	18383924	18383924	C	A	C	SNP	OS04G0376300	Intronic	NA	NA	LOC_Os04g30760	3-oxoacyl-reductase, chloroplast precursor, putative, expressed
Chr.4	18560705	18560705	C	A	C	SNP	OS04G0379900	Exonic	CDS	Silent	LOC_Os04g31070	acyl-desaturase, chloroplast precursor, putative, expressed
Chr.4	18562268	18562268	C	A	C	SNP	OS04G0379900	Intronic	NA	NA	LOC_Os04g31070	acyl-desaturase, chloroplast precursor, putative, expressed
Chr.4	18603934	18603934	C	A	C	SNP	OS04G0380300	Exonic	CDS	Missense	LOC_Os04g31120	OsFBK14 - F-box domain and kelch repeat containing protein, expressed
Chr.4	18604099	18604099	C	A	C	SNP	OS04G0380300	Exonic	CDS	Missense	LOC_Os04g31120	OsFBK14 - F-box domain and kelch repeat containing protein, expressed
Chr.4	18606543	18606543	C	A	C	SNP	OS04G0380300	Intronic	NA	NA	LOC_Os04g31120	OsFBK14 - F-box domain and kelch repeat containing protein, expressed
Chr.4	18608070	18608070	C	A	C	SNP	OS04G0380300	Exonic-ProteinC	NA	NA	LOC_Os04g31120	OsFBK14 - F-box domain and kelch repeat containing protein, expressed
Chr.4	21755024	21755024	C	A	C	SNP	OS04G0437600	Intronic	NA	NA	LOC_Os04g35700	STE_MEKK_ste11_MAP3K.16 - STE kinases include homologs to sterile 7, sterile 11 and sterile 20 from yeast, expressed
Chr.4	22625003	22625003	C	A	C	SNP	OS04G0453233	Intronic	NA	NA	LOC_Os04g38026	transporter family protein, putative, expressed
Chr.4	24301833	24301833	C	A	C	SNP	OS04G0486500	Intronic	NA	NA	LOC_Os04g40940	mitotic spindle checkpoint protein MAD2, putative, expressed
Chr.4	31240217	31240217	C	A	C	SNP	OS04G0615700	Exonic	CDS	Missense	LOC_Os04g52540	retrotransposon protein, putative, unclassified, expressed
Chr.4	32146387	32146387	C	A	C	SNP	OS04G0631200	Intronic	NA	NA	LOC_Os04g53950	glycosyl hydrolases family 16 protein, protein, expressed
Chr.4	32384889	32384889	C	A	C	SNP	OS04G0636800	Intronic	NA	NA	LOC_Os04g54430	immediate-early protein RSP40, putative, expressed
Chr.4	357804	357804	T	A	T	SNP	OS04G0105400	Exonic	3UTR	NA	LOC_Os04g01520	expressed protein

Chr.4	358034	358034	C	A	C	SNP	OS04G0105400	Intronic	NA	NA	LOC_Os04g01520	expressed protein
Chr.4	1126229	1126229	G	A	G	SNP	OS04G0119000	Intronic	NA	NA	LOC_Os04g02880	expressed protein
Chr.4	1126383	1126383	G	A	G	SNP	OS04G0119000	Exonic	CDS	Missense	LOC_Os04g02880	expressed protein
Chr.4	1149663	1149663	G	A	G	SNP	OS04G0119500	Exonic	CDS	Silent	LOC_Os04g02910	membrane related protein CP5, putative, expressed
Chr.4	5521991	5521991	G	A	G	SNP	OS04G0180900	Exonic	5UTR	NA	LOC_Os04g10214	expressed protein
Chr.4	5522479	5522479	G	A	G	SNP	OS04G0180900	Exonic	3UTR	NA	LOC_Os04g10214	expressed protein
Chr.4	5522536	5522536	G	A	G	SNP	OS04G0180900	Exonic	3UTR	NA	LOC_Os04g10214	expressed protein
Chr.4	6226112	6226112	T	A	T	SNP	OS04G0191400	Intronic	NA	NA	LOC_Os04g11390	expressed protein
Chr.4	6232194	6232194	G	A	G	SNP	OS04G0191500	Exonic	CDS	NA	LOC_Os04g11400	expressed protein
Chr.4	6477413	6477413	G	A	G	SNP	OS04G0194500	Exonic	CDS	Missense	LOC_Os04g11820	white-brown complex homolog protein, putative, expressed
Chr.4	6479326	6479326	T	A	T	SNP	OS04G0194500	Intronic	NA	NA	LOC_Os04g11820	white-brown complex homolog protein, putative, expressed
Chr.4	6480025	6480025	T	A	T	SNP	OS04G0194500	Intronic	NA	NA	LOC_Os04g11820	white-brown complex homolog protein, putative, expressed
Chr.4	8587339	8587339	G	A	G	SNP	OS04G0228100	Exonic	CDS	Silent	LOC_Os04g15800	expressed protein
Chr.4	11237144	11237144	C	A	C	SNP	OS04G0269600	Exonic	CDS	Silent	LOC_Os04g20164	amine oxidase precursor, putative, expressed
Chr.4	11239692	11239692	C	A	C	SNP	OS04G0269600	Intronic	NA	NA	LOC_Os04g20164	amine oxidase precursor, putative, expressed
Chr.4	11239746	11239746	G	A	G	SNP	OS04G0269600	Intronic	NA	NA	LOC_Os04g20164	amine oxidase precursor, putative, expressed
Chr.4	11240463	11240463	G	A	G	SNP	OS04G0269600	Intronic	NA	NA	LOC_Os04g20164	amine oxidase precursor, putative, expressed
Chr.4	11241200	11241200	G	A	G	SNP	OS04G0269600	Intronic	NA	NA	LOC_Os04g20164	amine oxidase precursor, putative, expressed
Chr.4	11241264	11241264	G	A	G	SNP	OS04G0269600	Intronic	NA	NA	LOC_Os04g20164	amine oxidase precursor, putative, expressed
Chr.4	11241288	11241288	G	A	G	SNP	OS04G0269600	Intronic	NA	NA	LOC_Os04g20164	amine oxidase precursor, putative, expressed
Chr.4	11241439	11241439	G	A	G	SNP	OS04G0269600	Intronic	NA	NA	LOC_Os04g20164	amine oxidase precursor, putative, expressed
Chr.4	11274335	11274335	C	A	C	SNP	OS04G0270100	Intronic	NA	NA	LOC_Os04g20220	elongation factor Tu, putative, expressed
Chr.4	11275360	11275360	G	A	G	SNP	OS04G0270100	Intronic	NA	NA	LOC_Os04g20220	elongation factor Tu, putative, expressed
Chr.4	11278232	11278232	G	A	G	SNP	OS04G0270100	Intronic	NA	NA	LOC_Os04g20220	elongation factor Tu, putative, expressed
Chr.4	11278387	11278387	G	A	G	SNP	OS04G0270100	Intronic	NA	NA	LOC_Os04g20220	elongation factor Tu, putative, expressed
Chr.4	11280521	11280521	T	A	T	SNP	OS04G0270100	Intronic	NA	NA	LOC_Os04g20220	elongation factor Tu, putative, expressed
Chr.4	11280765	11280765	G	A	G	SNP	OS04G0270100	Intronic	NA	NA	LOC_Os04g20220	elongation factor Tu, putative, expressed
Chr.4	11282353	11282353	G	A	G	SNP	OS04G0270100	Intronic	NA	NA	LOC_Os04g20220	elongation factor Tu, putative, expressed
Chr.4	11282436	11282436	T	A	T	SNP	OS04G0270100	Intronic	NA	NA	LOC_Os04g20220	elongation factor Tu, putative, expressed
Chr.4	11283426	11283426	C	A	C	SNP	OS04G0270100	Intronic	NA	NA	LOC_Os04g20220	elongation factor Tu, putative, expressed
Chr.4	11284665	11284665	C	A	C	SNP	OS04G0270100	Exonic	CDS	NA	LOC_Os04g20220	elongation factor Tu, putative, expressed
Chr.4	11284933	11284933	G	A	G	SNP	OS04G0270100	Intronic	NA	NA	LOC_Os04g20220	elongation factor Tu, putative, expressed
Chr.4	11285762	11285762	G	A	G	SNP	OS04G0270100	Intronic	NA	NA	LOC_Os04g20220	elongation factor Tu, putative, expressed
Chr.4	12425668	12425668	G	A	G	SNP	OS04G0287400	Exonic	CDS	Missense	LOC_Os04g21950	WRKY51, expressed
Chr.4	13493590	13493590	G	A	G	SNP	OS04G0301700	Exonic	3UTR	NA	LOC_Os04g23580	xylosyltransferase, putative, expressed
Chr.4	13493887	13493887	T	A	T	SNP	OS04G0301700	Exonic	3UTR	NA	LOC_Os04g23580	xylosyltransferase, putative, expressed
Chr.4	13496527	13496527	G	A	G	SNP	OS04G0301700	Intronic	NA	NA	LOC_Os04g23580	xylosyltransferase, putative, expressed
Chr.4	13643504	13643504	G	A	G	SNP	OS04G0304200	Intronic	NA	NA	LOC_Os04g23890	AGC_PVPK_like_kin82y.10 - ACG kinases include homologs to PKA, PKG and PKC, expressed
Chr.4	13643725	13643725	G	A	G	SNP	OS04G0304200	Intronic	NA	NA	LOC_Os04g23890	AGC_PVPK_like_kin82y.10 - ACG kinases include homologs to PKA, PKG and PKC, expressed
Chr.4	13647988	13647988	C	A	C	SNP	OS04G0304200	Intronic	NA	NA	LOC_Os04g23890	AGC_PVPK_like_kin82y.10 - ACG kinases include homologs to PKA, PKG and PKC, expressed
Chr.4	13650574	13650574	C	A	C	SNP	OS04G0304200	Intronic	NA	NA	LOC_Os04g23890	AGC_PVPK_like_kin82y.10 - ACG kinases include homologs to PKA, PKG and PKC, expressed
Chr.4	13651161	13651161	G	A	G	SNP	OS04G0304200	Intronic	NA	NA	LOC_Os04g23890	AGC_PVPK_like_kin82y.10 - ACG kinases include homologs to PKA, PKG and PKC, expressed
Chr.4	13651241	13651241	G	A	G	SNP	OS04G0304200	Intronic	NA	NA	LOC_Os04g23890	AGC_PVPK_like_kin82y.10 - ACG kinases include homologs to PKA, PKG and PKC, expressed
Chr.4	13651762	13651762	T	A	T	SNP	OS04G0304200	Intronic	NA	NA	LOC_Os04g23890	AGC_PVPK_like_kin82y.10 - ACG kinases include homologs to PKA, PKG and PKC, expressed
Chr.4	13654626	13654626	T	A	T	SNP	OS04G0304200	Exonic	5UTR	NA	LOC_Os04g23890	AGC_PVPK_like_kin82y.10 - ACG kinases include homologs to PKA, PKG and PKC, expressed
Chr.4	14825255	14825255	T	A	T	SNP	OS04G0321800	Intronic	NA	NA	LOC_Os04g25570	protein phosphatase 2C, putative, expressed
Chr.4	14825559	14825559	G	A	G	SNP	OS04G0321800	Intronic	NA	NA	LOC_Os04g25570	protein phosphatase 2C, putative, expressed
Chr.4	16002108	16002108	G	A	G	SNP	OS04G0339400	Intronic	NA	NA	LOC_Os04g27060	oxidoreductase, aldo/keto reductase family protein, putative, expressed
Chr.4	16002360	16002360	G	A	G	SNP	OS04G0339400	Intronic	NA	NA	LOC_Os04g27060	oxidoreductase, aldo/keto reductase family protein, putative, expressed
Chr.4	16002471	16002471	T	A	T	SNP	OS04G0339400	Intronic	NA	NA	LOC_Os04g27060	oxidoreductase, aldo/keto reductase family protein, putative, expressed
Chr.4	16002660	16002660	T	A	T	SNP	OS04G0339400	Intronic	NA	NA	LOC_Os04g27060	oxidoreductase, aldo/keto reductase family protein, putative, expressed
Chr.4	16003541	16003541	G	A	G	SNP	OS04G0339400	Intronic	NA	NA	LOC_Os04g27060	oxidoreductase, aldo/keto reductase family protein, putative, expressed
Chr.4	16003546	16003546	G	A	G	SNP	OS04G0339400	Intronic	NA	NA	LOC_Os04g27060	oxidoreductase, aldo/keto reductase family protein, putative, expressed
Chr.4	16003784	16003784	G	A	G	SNP	OS04G0339400	Intronic	NA	NA	LOC_Os04g27060	oxidoreductase, aldo/keto reductase family protein, putative, expressed
Chr.4	16004125	16004125	G	A	G	SNP	OS04G0339400	Intronic	NA	NA	LOC_Os04g27060	oxidoreductase, aldo/keto reductase family protein, putative, expressed
Chr.4	16004207	16004207	G	A	G	SNP	OS04G0339400	Intronic	NA	NA	LOC_Os04g27060	oxidoreductase, aldo/keto reductase family protein, putative, expressed
Chr.4	18976428	18976428	C	A	C	SNP	OS04G0386300	Exonic	3UTR	NA	LOC_Os04g31670	expressed protein
Chr.4	19599131	19599131	T	A	T	SNP	OS04G0397000	Intronic	NA	NA	LOC_Os04g32550	defender against cell death 1, putative, expressed
Chr.4	19600295	19600295	G	A	G	SNP	OS04G0397000	Intronic	NA	NA	LOC_Os04g32550	defender against cell death 1, putative, expressed
Chr.4	20359456	20359456	C	A	C	SNP	OS04G0412100	Exonic	3UTR	NA	LOC_Os04g33610	expressed protein
Chr.4	20389896	20389896	G	A	G	SNP	OS04G0412500	Exonic	3UTR	NA	LOC_Os04g33660	bifunctional monodehydroascorbate reductase and carbonic anhydrasenectarin-3 precursor, putative, expressed
Chr.4	20425564	20425564	G	A	G	SNP	OS04G0413500	Intronic	NA	NA	LOC_Os04g33740	glycosyl hydrolases, putative, expressed
Chr.4	20425647	20425647	C	A	C	SNP	OS04G0413500	Intronic	NA	NA	LOC_Os04g33740	glycosyl hydrolases, putative, expressed
Chr.4	20425666	20425666	G	A	G	SNP	OS04G0413500	Intronic	NA	NA	LOC_Os04g33740	glycosyl hydrolases, putative, expressed
Chr.4	20425673	20425673	G	A	G	SNP	OS04G0413500	Intronic	NA	NA	LOC_Os04g33740	glycosyl hydrolases, putative, expressed
Chr.4	20426708	20426708	G	A	G	SNP	OS04G0413500	Exonic	3UTR	NA	LOC_Os04g33740	glycosyl hydrolases, putative, expressed
Chr.4	20520457	20520457	T	A	T	SNP	OS04G0415100	Exonic	3UTR	NA	LOC_Os04g33870	DUF623 domain containing protein, expressed
Chr.4	22448561	22448561	G	A	G	SNP	OS04G0450600	Exonic	CDS	Missense	LOC_Os04g37760	expressed protein
Chr.4	30157179	30157179	T	A	T	SNP	OS04G0597600	Exonic	5UTR	NA	LOC_Os04g50940	peptide transporter PTR2, putative, expressed
Chr.4	30172166	30172166	C	A	C	SNP	OS04G0598000	Exonic	3UTR	NA	LOC_Os04g50970	seed specific protein Bn15D1B, putative, expressed
Chr.4	30172936	30172936	G	A	G	SNP	OS04G0598000	Intronic	NA	NA	LOC_Os04g50970	seed specific protein Bn15D1B, putative, expressed
Chr.4	30287708	30287708	C	A	C	SNP	OS04G0600200	Intronic	NA	NA	LOC_Os04g51150	transposon protein, putative, unclassified, expressed
Chr.4	30288815	30288815	T	A	T	SNP	OS04G0600200	Intronic	NA	NA	LOC_Os04g51150	transposon protein, putative, unclassified, expressed
Chr.4	30289334	30289334	G	A	G	SNP	OS04G0600200	Intronic	NA	NA	LOC_Os04g51150	transposon protein, putative, unclassified, expressed
Chr.4	30556078	30556078	T	A	T	SNP	OS04G0605300	Intronic	NA	NA	LOC_Os04g51580	leucine rich repeat containing protein, expressed
Chr.4	30557563	30557563	G	A	G	SNP	OS04G0605300	Intronic	NA	NA	LOC_Os04g51580	leucine rich repeat containing protein, expressed
Chr.4	30599930	30599930	C	A	C	SNP	OS04G0605900	Exonic	5UTR	NA	LOC_Os04g51630	60S ribosomal protein L7, putative, expressed
Chr.4	30600489	30600489	G	A	G	SNP	OS04G0605900	Intronic	NA	NA	LOC_Os04g51630	60S ribosomal protein L7, putative, expressed
Chr.4	30600692	30600692	T	A	T	SNP	OS04G0605900	Intronic	NA	NA	LOC_Os04g51630	60S ribosomal protein L7, putative, expressed
Chr.4	30600812	30600812	G	A	G	SNP	OS04G0605900	Intronic	NA	NA	LOC_Os04g51630	60S ribosomal protein L7, putative, expressed
Chr.4	30602143	30602143	G	A	G	SNP	OS04G0605900	Exonic	3UTR	NA	LOC_Os04g51630	60S ribosomal protein L7, putative, expressed
Chr.4	30602252	30602252	G	A	G	SNP	OS04G0605900	Exonic	3UTR	NA	LOC_Os04g51630	60S ribosomal protein L7, putative, expressed
Chr.4	30615834	30615834	G	A	G	SNP	OS04G0606200	Exonic	3UTR	NA	LOC_Os04g51680	expressed protein
Chr.4	32168224	32168224	G	A	G	SNP	OS04G0631600	Intronic	NA	NA	LOC_Os04g53990	ethylene-responsive protein related, putative, expressed
Chr.4	32168968	32168968	G	A	G	SNP	OS04G0631600	Intronic	NA	NA	LOC_Os04g53990	ethylene-responsive protein related, putative, expressed

Chr.4	32169134	32169134	T	A	T	SNP	OS04G0631600	Exonic	3UTR	NA	LOC_Os04g53990	ethylene-responsive protein related, putative, expressed
Chr.4	32583877	32583877	G	A	G	SNP	OS04G0640600	Intronic	NA	NA	LOC_Os04g54800	shikimate kinase, putative, expressed
Chr.4	35192649	35192649	G	A	G	SNP	OS04G0688200	Exonic	CDS	Silent	LOC_Os04g59160	peroxidase precursor, putative, expressed
Chr.4	35445464	35445464	T	A	T	SNP	OS04G0692600	Exonic	CDS	NA	LOC_Os04g59610	expressed protein
Chr.4	35449225	35449225	G	A	G	SNP	OS04G0692600	Intronic	NA	NA	LOC_Os04g59610	expressed protein
Chr.4	35449448	35449448	G	A	G	SNP	OS04G0692600	Intronic	NA	NA	LOC_Os04g59610	expressed protein
Chr.4	35449503	35449503	C	A	C	SNP	OS04G0692600	Intronic	NA	NA	LOC_Os04g59610	expressed protein
Chr.4	11239833	11239835	AAT	A	AAT	INDEL	OS04G0269600	Intronic	NA	NA	LOC_Os04g20164	amine oxidase precursor, putative, expressed
Chr.4	13494453	13494464	ACAGAAATAAAC	A	ACAGAAATAAAC	INDEL	OS04G0301700	Intronic	NA	NA	LOC_Os04g23580	xylosyltransferase, putative, expressed
Chr.4	16002449	16002451	AAC	A	AAC	INDEL	OS04G0339400	Intronic	NA	NA	LOC_Os04g27060	oxidoreductase, aldo/keto reductase family protein, putative, expressed
Chr.4	20426378	20426385	ATAGGTAC	A	ATAGGTAC	INDEL	OS04G0413500	Intronic	NA	NA	LOC_Os04g33740	glycosyl hydrolases, putative, expressed
Chr.4	30553274	30553276	ACC	A	ACC	INDEL	OS04G0605300	Exonic	3UTR	NA	LOC_Os04g51580	leucine rich repeat containing protein, expressed
Chr.4	30602184	30602185	AC	A	AC	INDEL	OS04G0605900	Exonic	3UTR	NA	LOC_Os04g51630	60S ribosomal protein L7, putative, expressed
Chr.4	32287124	32287125	AT	A	AT	INDEL	OS04G0634700	Intronic	NA	NA	LOC_Os04g54200	diacylglycerol kinase, putative, expressed
Chr.4	33973028	33973030	ATT	A	ATT	INDEL	OS04G0665600	Exonic	3UTR	NA	LOC_Os04g56990	Myb-like DNA-binding domain containing protein, putative, expressed
Chr.4	35450824	35450825	AG	A	AG	INDEL	OS04G0692600	Intronic	NA	NA	LOC_Os04g59610	expressed protein
Chr.4	619429	619429	G	A	G	SNP	OS04G0110600	Intronic	NA	NA	LOC_Os04g02000	zinc finger family protein, putative, expressed
Chr.4	5319593	5319593	G	A	G	SNP	OS04G0178300	Intronic	NA	NA	LOC_Os04g09900	ent-kaurene synthase, chloroplast precursor, putative, expressed
Chr.4	5321538	5321538	G	A	G	SNP	OS04G0178300	Intronic	NA	NA	LOC_Os04g09900	ent-kaurene synthase, chloroplast precursor, putative, expressed
Chr.4	5322389	5322389	G	A	G	SNP	OS04G0178300	Intronic	NA	NA	LOC_Os04g09900	ent-kaurene synthase, chloroplast precursor, putative, expressed
Chr.4	5322927	5322927	C	A	C	SNP	OS04G0178300	Intronic	NA	NA	LOC_Os04g09900	ent-kaurene synthase, chloroplast precursor, putative, expressed
Chr.4	5323327	5323327	G	A	G	SNP	OS04G0178300	Intronic	NA	NA	LOC_Os04g09900	ent-kaurene synthase, chloroplast precursor, putative, expressed
Chr.4	5323343	5323343	C	A	C	SNP	OS04G0178300	Intronic	NA	NA	LOC_Os04g09900	ent-kaurene synthase, chloroplast precursor, putative, expressed
Chr.4	5323474	5323474	G	A	G	SNP	OS04G0178300	Intronic	NA	NA	LOC_Os04g09900	ent-kaurene synthase, chloroplast precursor, putative, expressed
Chr.4	5323619	5323619	G	A	G	SNP	OS04G0178300	Intronic	NA	NA	LOC_Os04g09900	ent-kaurene synthase, chloroplast precursor, putative, expressed
Chr.4	5386862	5386862	C	A	C	SNP	OS04G0179200	Intronic	NA	NA	LOC_Os04g10010	sex determination protein tasselseed-2, putative, expressed
Chr.4	7308924	7308924	G	A	G	SNP	OS04G0209200	Intronic	NA	NA	LOC_Os04g13210	multidrug resistance-associated protein, putative, expressed
Chr.4	7309340	7309340	G	A	G	SNP	OS04G0209200	Intronic	NA	NA	LOC_Os04g13210	multidrug resistance-associated protein, putative, expressed
Chr.4	7309511	7309511	G	A	G	SNP	OS04G0209200	Intronic	NA	NA	LOC_Os04g13210	multidrug resistance-associated protein, putative, expressed
Chr.4	8949307	8949307	G	A	G	SNP	OS04G0233400	Exonic	CDS	Silent	LOC_Os04g16450	aquaporin protein, putative, expressed
Chr.4	12426024	12426024	G	A	G	SNP	OS04G0287400	Exonic	CDS	Silent	LOC_Os04g21950	WRKY51, expressed
Chr.4	12426156	12426156	G	A	G	SNP	OS04G0287400	Exonic	CDS	Silent	LOC_Os04g21950	WRKY51, expressed
Chr.4	12426265	12426265	G	A	G	SNP	OS04G0287400	Exonic	CDS	Missense	LOC_Os04g21950	WRKY51, expressed
Chr.4	12426711	12426711	T	A	T	SNP	OS04G0287400	Exonic	CDS	Missense	LOC_Os04g21950	WRKY51, expressed
Chr.4	13651057	13651057	C	A	C	SNP	OS04G0304200	Intronic	NA	NA	LOC_Os04g23890	AGC_PVPK_like_kin82y.10 - ACG kinases include homologs to PKA, PKG and PKC, expressed
Chr.4	13651939	13651939	T	A	T	SNP	OS04G0304200	Intronic	NA	NA	LOC_Os04g23890	AGC_PVPK_like_kin82y.10 - ACG kinases include homologs to PKA, PKG and PKC, expressed
Chr.4	14826391	14826391	G	A	G	SNP	OS04G0321800	Exonic	CDS	Silent	LOC_Os04g25570	protein phosphatase 2C, putative, expressed
Chr.4	16002098	16002098	C	A	C	SNP	OS04G0339400	Intronic	NA	NA	LOC_Os04g27060	oxidoreductase, aldo/keto reductase family protein, putative, expressed
Chr.4	16002102	16002102	T	A	T	SNP	OS04G0339400	Intronic	NA	NA	LOC_Os04g27060	oxidoreductase, aldo/keto reductase family protein, putative, expressed
Chr.4	16002199	16002199	G	A	G	SNP	OS04G0339400	Intronic	NA	NA	LOC_Os04g27060	oxidoreductase, aldo/keto reductase family protein, putative, expressed
Chr.4	18382878	18382878	G	A	G	SNP	OS04G0376300	Exonic	3UTR	NA	LOC_Os04g30760	3-oxoacyl-reductase, chloroplast precursor, putative, expressed
Chr.4	18603087	18603087	G	A	G	SNP	OS04G0380300	Exonic	CDS	Silent	LOC_Os04g31120	OsFBK14 - F-box domain and kelch repeat containing protein, expressed
Chr.4	18603205	18603205	G	A	G	SNP	OS04G0380300	Exonic	CDS	Missense	LOC_Os04g31120	OsFBK14 - F-box domain and kelch repeat containing protein, expressed
Chr.4	18605006	18605006	G	A	G	SNP	OS04G0380300	Intronic	NA	NA	LOC_Os04g31120	OsFBK14 - F-box domain and kelch repeat containing protein, expressed
Chr.4	18786626	18786626	G	A	G	SNP	OS04G0382900	Exonic	CDS	Missense	LOC_Os04g31410	RALFL37 - Rapid ALkalinization Factor RALF family protein precursor, expressed
Chr.4	18786635	18786635	G	A	G	SNP	OS04G0382900	Exonic	CDS	Missense	LOC_Os04g31410	RALFL37 - Rapid ALkalinization Factor RALF family protein precursor, expressed
Chr.4	18976221	18976221	G	A	G	SNP	OS04G0386300	Exonic	CDS	NA	LOC_Os04g31670	expressed protein
Chr.4	20424714	20424714	G	A	G	SNP	OS04G0413500	Intronic	NA	NA	LOC_Os04g33740	glycosyl hydrolases, putative, expressed
Chr.4	20708598	20708598	G	A	G	SNP	OS04G0418800	Exonic	CDS	Silent	LOC_Os04g34170	retrotransposon protein, putative, unclassified, expressed
Chr.4	21753795	21753795	G	A	G	SNP	OS04G0437600	Intronic	NA	NA	LOC_Os04g35700	STE_MEKK_ste11_MAP3K.16 - STE kinases include homologs to sterile 7, sterile 11 and sterile 20 from yeast, expressed
Chr.4	22449735	22449735	T	A	T	SNP	OS04G0450600	Intronic	NA	NA	LOC_Os04g37760	expressed protein
Chr.4	22450128	22450128	G	A	G	SNP	OS04G0450600	Intronic	NA	NA	LOC_Os04g37760	expressed protein
Chr.4	22623620	22623620	T	A	T	SNP	OS04G0453233	Intronic	NA	NA	LOC_Os04g38026	transporter family protein, putative, expressed
Chr.4	22624081	22624081	T	A	T	SNP	OS04G0453233	Intronic	NA	NA	LOC_Os04g38026	transporter family protein, putative, expressed
Chr.4	22624268	22624268	G	A	G	SNP	OS04G0453233	Intronic	NA	NA	LOC_Os04g38026	transporter family protein, putative, expressed
Chr.4	25661911	25661911	G	A	G	SNP	OS04G0513400	Exonic	3UTR	NA	LOC_Os04g43390	Os4bglu16 - monolignol beta-glucoside homologue, expressed
Chr.4	25662270	25662270	G	A	G	SNP	OS04G0513400	Exonic	3UTR	NA	LOC_Os04g43390	Os4bglu16 - monolignol beta-glucoside homologue, expressed
Chr.4	25662341	25662341	G	A	G	SNP	OS04G0513400	Exonic	3UTR	NA	LOC_Os04g43390	Os4bglu16 - monolignol beta-glucoside homologue, expressed
Chr.4	28342068	28342068	G	A	G	SNP	OS04G0565500	Exonic	CDS	Silent	LOC_Os04g47780	transmembrane amino acid transporter protein, putative, expressed
Chr.4	30173147	30173147	G	A	G	SNP	OS04G0598000	Intronic	NA	NA	LOC_Os04g50970	seed specific protein Bn15D1B, putative, expressed
Chr.4	30554138	30554138	G	A	G	SNP	OS04G0605300	Intronic	NA	NA	LOC_Os04g51580	leucine rich repeat containing protein, expressed
Chr.4	30554184	30554184	G	A	G	SNP	OS04G0605300	Intronic	NA	NA	LOC_Os04g51580	leucine rich repeat containing protein, expressed
Chr.4	30554207	30554207	G	A	G	SNP	OS04G0605300	Intronic	NA	NA	LOC_Os04g51580	leucine rich repeat containing protein, expressed
Chr.4	30557811	30557811	G	A	G	SNP	OS04G0605300	Intronic	NA	NA	LOC_Os04g51580	leucine rich repeat containing protein, expressed
Chr.4	30557910	30557910	T	A	T	SNP	OS04G0605300	Intronic	NA	NA	LOC_Os04g51580	leucine rich repeat containing protein, expressed
Chr.4	30600129	30600129	G	A	G	SNP	OS04G0605900	Intronic	NA	NA	LOC_Os04g51630	60S ribosomal protein L7, putative, expressed
Chr.4	30600865	30600865	G	A	G	SNP	OS04G0605900	Intronic	NA	NA	LOC_Os04g51630	60S ribosomal protein L7, putative, expressed
Chr.4	31239200	31239200	G	A	G	SNP	OS04G0615700	Exonic	5UTR	NA	LOC_Os04g52540	retrotransposon protein, putative, unclassified, expressed
Chr.4	32145763	32145763	G	A	G	SNP	OS04G0631200	Exonic	CDS	Missense	LOC_Os04g53950	glycosyl hydrolases family 16 protein, protein, expressed
Chr.4	32167683	32167683	G	A	G	SNP	OS04G0631600	Exonic	CDS	Missense	LOC_Os04g53990	ethylene-responsive protein related, putative, expressed
Chr.4	32385000	32385000	G	A	G	SNP	OS04G0636800	Intronic	NA	NA	LOC_Os04g54430	immediate-early protein RSP40, putative, expressed
Chr.4	35444300	35444300	C	A	C	SNP	OS04G0692600	Intronic	NA	NA	LOC_Os04g59610	expressed protein
Chr.4	35444503	35444503	G	A	G	SNP	OS04G0692600	Intronic	NA	NA	LOC_Os04g59610	expressed protein
Chr.4	35451023	35451023	G	A	G	SNP	OS04G0692600	Exonic	3UTR	NA	LOC_Os04g59610	expressed protein
Chr.4	620773	620775	ACT	A	ACT	INDEL	OS04G0110600	Intronic	NA	NA	LOC_Os04g02000	zinc finger family protein, putative, expressed
Chr.4	5321589	5321604	AAAAACGTGCATGCGG	A	AAAAACGTGCATGCGG	INDEL	OS04G0178300	Intronic	NA	NA	LOC_Os04g09900	ent-kaurene synthase, chloroplast precursor, putative, expressed
Chr.4	7308643	7308647	ACCCC	A	ACCCC	INDEL	OS04G0209200	Intronic	NA	NA	LOC_Os04g13210	multidrug resistance-associated protein, putative, expressed
Chr.4	7309120	7309121	AT	A	AT	INDEL	OS04G0209200	Intronic	NA	NA	LOC_Os04g13210	multidrug resistance-associated protein, putative, expressed
Chr.4	11240397	11240399	ATT	A	ATT	INDEL	OS04G0269600	Intronic	NA	NA	LOC_Os04g20164	amine oxidase precursor, putative, expressed
Chr.4	12425564	12425570	ACTTCTT	A	ACTTCTT	INDEL	OS04G0287400	Exonic	5UTR	NA	LOC_Os04g21950	WRKY51, expressed
Chr.4	12426575	12426578	ATCG	A	ATCG	INDEL	OS04G0287400	Intronic	NA	NA	LOC_Os04g21950	WRKY51, expressed
Chr.4	12426783	12426784	AC	A	AC	INDEL	OS04G0287400	Exonic	3UTR	NA	LOC_Os04g21950	WRKY51, expressed
Chr.4	18213798	18213799	AT	A	AT	INDEL	OS04G0373400	Intronic	NA	NA	LOC_Os04g30490	MATE efflux family protein, putative, expressed
Chr.4	18562081	18562082	AT	A	AT	INDEL	OS04G0379900	Intronic	NA	NA	LOC_Os04g31070	acyl-desaturase, chloroplast precursor, putative, expressed
Chr.4	18604915	18604916	AC	A	AC	INDEL	OS04G0380300	Intronic	NA	NA	LOC_Os04g31120	OsFBK14 - F-box domain and kelch repeat containing protein, expressed

Chr.4	18607777	18607797	AAATCTGGGTGATTTTTTATT	A	AAATCTGGGTGATTTTTTATT	INDEL	OS04G0380300	Exonic-ProteinC	NA	NA	LOC_Os04g31120	OsFBK14 - F-box domain and kelch repeat containing protein, expressed
Chr.4	18607973	18607974	AT	A	AT	INDEL	OS04G0380300	Exonic-ProteinC	NA	NA	LOC_Os04g31120	OsFBK14 - F-box domain and kelch repeat containing protein, expressed
Chr.4	22021408	22021411	AGTT	A	AGTT	INDEL	OS04G0442200	Intronic	NA	NA	LOC_Os04g36062	expressed protein
Chr.4	22829486	22829487	AC	A	AC	INDEL	OS04G0456700	Intronic	NA	NA	LOC_Os04g38390	wound/stress protein, putative, expressed
Chr.4	33532356	33532364	AATATATAT	A	AATATATAT	INDEL	OS04G0657500	Intronic	NA	NA	LOC_Os04g56240	lipase, putative, expressed
Chr.5	14295423	14295423	G	A	G	SNP	OS05G0312000	Exonic	CDS	Silent	LOC_Os05g24684	structural constituent of ribosome, putative, expressed
Chr.5	4854247	4854247	G	A	G	SNP	OS05G0180600	Exonic	3UTR	NA	LOC_Os05g08810	phosphatidylinositol 3-kinase, root isoform, putative, expressed
Chr.5	873276	873276	T	A	T	SNP	OS05G0116000	Exonic	3UTR	NA	LOC_Os05g02520	cupin domain containing protein, expressed
Chr.5	874733	874733	T	A	T	SNP	OS05G0116000	Intronic	NA	NA	LOC_Os05g02520	cupin domain containing protein, expressed
Chr.5	6192105	6192105	T	A	T	SNP	OS05G0199500	Intronic	NA	NA	LOC_Os05g11060	galactosyltransferase family protein, putative, expressed
Chr.5	23867737	23867737	T	A	T	SNP	OS05G0485300	Intronic	NA	NA	LOC_Os05g40700	transmembrane protein 56, putative, expressed
Chr.5	836787	836787	G	A	G	SNP	OS05G0115100	Intronic	NA	NA	LOC_Os05g02450	expressed protein
Chr.5	873408	873408	G	A	G	SNP	OS05G0116000	Exonic	CDS	Silent	LOC_Os05g02520	cupin domain containing protein, expressed
Chr.5	874566	874566	G	A	G	SNP	OS05G0116000	Intronic	NA	NA	LOC_Os05g02520	cupin domain containing protein, expressed
Chr.5	875390	875390	G	A	G	SNP	OS05G0116000	Intronic	NA	NA	LOC_Os05g02520	cupin domain containing protein, expressed
Chr.5	875406	875406	G	A	G	SNP	OS05G0116000	Intronic	NA	NA	LOC_Os05g02520	cupin domain containing protein, expressed
Chr.5	875425	875425	G	A	G	SNP	OS05G0116000	Intronic	NA	NA	LOC_Os05g02520	cupin domain containing protein, expressed
Chr.5	2178085	2178085	G	A	G	SNP	OS05G0137400	Intronic	NA	NA	LOC_Os05g04630	retrotransposon protein, putative, SINE subclass, expressed
Chr.5	3720859	3720859	G	A	G	SNP	OS05G0163400	Exonic	5UTR	NA	LOC_Os05g07070	zinc finger, C3HC4 type domain containing protein, expressed
Chr.5	6193214	6193214	G	A	G	SNP	OS05G0199500	Intronic	NA	NA	LOC_Os05g11060	galactosyltransferase family protein, putative, expressed
Chr.5	6194552	6194552	G	A	G	SNP	OS05G0199500	Intronic	NA	NA	LOC_Os05g11060	galactosyltransferase family protein, putative, expressed
Chr.5	7165118	7165118	G	A	G	SNP	OS05G0215600	Intronic	NA	NA	LOC_Os05g12474	expressed protein
Chr.5	7722310	7722310	G	A	G	SNP	OS05G0226900	Exonic	CDS	Silent	LOC_Os05g13900	retrotransposon protein, putative, unclassified, expressed
Chr.5	7722467	7722467	G	A	G	SNP	OS05G0226900	Exonic	CDS	Missense	LOC_Os05g13900	retrotransposon protein, putative, unclassified, expressed
Chr.5	7722618	7722618	G	A	G	SNP	OS05G0226900	Exonic	CDS	Missense	LOC_Os05g13900	retrotransposon protein, putative, unclassified, expressed
Chr.5	9319294	9319294	G	A	G	SNP	OS05G0253200	Intronic	NA	NA	LOC_Os05g16420	SHR5-receptor-like kinase, putative, expressed
Chr.5	9323275	9323275	G	A	G	SNP	OS05G0253200	Exonic	3UTR	NA	LOC_Os05g16420	SHR5-receptor-like kinase, putative, expressed
Chr.5	9323985	9323985	G	A	G	SNP	OS05G0253200	Exonic	3UTR	NA	LOC_Os05g16420	SHR5-receptor-like kinase, putative, expressed
Chr.5	9324978	9324978	G	A	G	SNP	OS05G0253200	Exonic	3UTR	NA	LOC_Os05g16420	SHR5-receptor-like kinase, putative, expressed
Chr.5	9327626	9327626	G	A	G	SNP	OS05G0253200	Exonic	3UTR	NA	LOC_Os05g16420	SHR5-receptor-like kinase, putative, expressed
Chr.5	12455599	12455599	G	A	G	SNP	OS05G0287800	Intronic	NA	NA	LOC_Os05g21180	phosphatidic acid phosphatase-related, putative, expressed
Chr.5	20277725	20277725	G	A	G	SNP	OS05G0414501	Exonic	CDS	Silent	LOC_Os05g34260	transposon protein, putative, Ac/Ds sub-class, expressed
Chr.5	24104958	24104958	G	A	G	SNP	OS05G0490400	Exonic	CDS	Missense	LOC_Os05g41150	expressed protein
Chr.5	24105312	24105312	G	A	G	SNP	OS05G0490400	Exonic	CDS	Missense	LOC_Os05g41150	expressed protein
Chr.5	873188	873188	C	A	C	SNP	OS05G0116000	Exonic	3UTR	NA	LOC_Os05g02520	cupin domain containing protein, expressed
Chr.5	875516	875516	C	A	C	SNP	OS05G0116000	Intronic	NA	NA	LOC_Os05g02520	cupin domain containing protein, expressed
Chr.5	2175524	2175524	C	A	C	SNP	OS05G0137400	Intronic	NA	NA	LOC_Os05g04630	retrotransposon protein, putative, SINE subclass, expressed
Chr.5	6191898	6191898	C	A	C	SNP	OS05G0199500	Intronic	NA	NA	LOC_Os05g11060	galactosyltransferase family protein, putative, expressed
Chr.5	6192742	6192742	C	A	C	SNP	OS05G0199500	Intronic	NA	NA	LOC_Os05g11060	galactosyltransferase family protein, putative, expressed
Chr.5	7163775	7163775	C	A	C	SNP	OS05G0215600	Exonic	3UTR	NA	LOC_Os05g12474	expressed protein
Chr.5	7164455	7164455	C	A	C	SNP	OS05G0215600	Intronic	NA	NA	LOC_Os05g12474	expressed protein
Chr.5	7722630	7722630	G	A	G	SNP	OS05G0226900	Exonic	CDS	Missense	LOC_Os05g13900	retrotransposon protein, putative, unclassified, expressed
Chr.5	19771508	19771508	C	A	C	SNP	OS05G0405900	Intronic	NA	NA	LOC_Os05g33610	expressed protein
Chr.5	24133441	24133441	C	A	C	SNP	OS05G0491100	Intronic	NA	NA	LOC_Os05g41210	OsCam2 - Calmodulin, expressed
Chr.5	21493482	21493482	C	A	C	SNP	OS05G0438500	Exonic	3UTR	NA	LOC_Os05g36260	soluble inorganic pyrophosphatase, putative, expressed
Chr.5	741850	741850	G	A	G	SNP	OS05G0114000	Intronic	NA	NA	LOC_Os05g02310	soluble inorganic pyrophosphatase, putative, expressed
Chr.5	742355	742355	T	A	T	SNP	OS05G0114000	Intronic	NA	NA	LOC_Os05g02310	soluble inorganic pyrophosphatase, putative, expressed
Chr.5	998470	998470	G	A	G	SNP	OS05G0119200	Intronic	NA	NA	LOC_Os05g02770	glycine-rich cell wall structural protein precursor, putative, expressed
Chr.5	1001382	1001382	T	A	T	SNP	OS05G0119300	Exonic	3UTR	NA	LOC_Os05g02780	glycine-rich protein A3, putative, expressed
Chr.5	1002618	1002618	T	A	T	SNP	OS05G0119300	Exonic	5UTR	NA	LOC_Os05g02780	glycine-rich protein A3, putative, expressed
Chr.5	1994385	1994385	T	A	T	SNP	OS05G0134200	Exonic	5UTR	NA	LOC_Os05g04360	protein phosphatase 2C, putative, expressed
Chr.5	1994989	1994989	G	A	G	SNP	OS05G0134200	Intronic	NA	NA	LOC_Os05g04360	protein phosphatase 2C, putative, expressed
Chr.5	2114413	2114413	G	A	G	SNP	OS05G0136200	Exonic	CDS	Missense	LOC_Os05g04550	CAMK_KIN1/SNF1/Nim1_like.3 - CAMK includes calcium/calmodulin depedent protein kinases, expressed
Chr.5	2114826	2114826	C	A	C	SNP	OS05G0136200	Exonic	CDS	Missense	LOC_Os05g04550	CAMK_KIN1/SNF1/Nim1_like.3 - CAMK includes calcium/calmodulin depedent protein kinases, expressed
Chr.5	3720037	3720037	G	A	G	SNP	OS05G0163400	Exonic	CDS	Missense	LOC_Os05g07070	zinc finger, C3HC4 type domain containing protein, expressed
Chr.5	3721912	3721912	C	A	C	SNP	OS05G0163400	Exonic	5UTR	NA	LOC_Os05g07070	zinc finger, C3HC4 type domain containing protein, expressed
Chr.5	3725232	3725232	C	A	C	SNP	OS05G0163400	Exonic	5UTR	NA	LOC_Os05g07070	zinc finger, C3HC4 type domain containing protein, expressed
Chr.5	3733418	3733418	G	A	G	SNP	OS05G0163700	Intronic	NA	NA	LOC_Os05g07090	acyl-coenzyme A dehydrogenase, mitochondrial precursor, putative, expressed
Chr.5	3757444	3757444	G	A	G	SNP	OS05G0163900	Intronic	NA	NA	LOC_Os05g07120	basic helix-loop-helix, putative, expressed
Chr.5	3757740	3757740	G	A	G	SNP	OS05G0163900	Intronic	NA	NA	LOC_Os05g07120	basic helix-loop-helix, putative, expressed
Chr.5	3757747	3757747	C	A	C	SNP	OS05G0163900	Intronic	NA	NA	LOC_Os05g07120	basic helix-loop-helix, putative, expressed
Chr.5	3757832	3757832	G	A	G	SNP	OS05G0163900	Intronic	NA	NA	LOC_Os05g07120	basic helix-loop-helix, putative, expressed
Chr.5	3758390	3758390	G	A	G	SNP	OS05G0163900	Intronic	NA	NA	LOC_Os05g07120	basic helix-loop-helix, putative, expressed
Chr.5	3758622	3758622	G	A	G	SNP	OS05G0163900	Intronic	NA	NA	LOC_Os05g07120	basic helix-loop-helix, putative, expressed
Chr.5	3758793	3758793	G	A	G	SNP	OS05G0163900	Intronic	NA	NA	LOC_Os05g07120	basic helix-loop-helix, putative, expressed
Chr.5	3758806	3758806	G	A	G	SNP	OS05G0163900	Intronic	NA	NA	LOC_Os05g07120	basic helix-loop-helix, putative, expressed
Chr.5	3758840	3758840	G	A	G	SNP	OS05G0163900	Intronic	NA	NA	LOC_Os05g07120	basic helix-loop-helix, putative, expressed
Chr.5	3759480	3759480	G	A	G	SNP	OS05G0163900	Exonic	CDS	Missense	LOC_Os05g07120	basic helix-loop-helix, putative, expressed
Chr.5	3759482	3759482	G	A	G	SNP	OS05G0163900	Exonic	CDS	Silent	LOC_Os05g07120	basic helix-loop-helix, putative, expressed
Chr.5	3759775	3759775	G	A	G	SNP	OS05G0163900	Exonic	3UTR	NA	LOC_Os05g07120	basic helix-loop-helix, putative, expressed
Chr.5	4136491	4136491	G	A	G	SNP	OS05G0169000	Exonic	CDS	Silent	LOC_Os05g07690	thioredoxin, putative, expressed
Chr.5	4136612	4136612	C	A	C	SNP	OS05G0169000	Exonic	3UTR	NA	LOC_Os05g07690	thioredoxin, putative, expressed
Chr.5	4136615	4136615	G	A	G	SNP	OS05G0169000	Exonic	3UTR	NA	LOC_Os05g07690	thioredoxin, putative, expressed
Chr.5	4136741	4136741	G	A	G	SNP	OS05G0169000	Exonic	3UTR	NA	LOC_Os05g07690	thioredoxin, putative, expressed
Chr.5	4136934	4136934	G	A	G	SNP	OS05G0169000	Exonic	3UTR	NA	LOC_Os05g07690	thioredoxin, putative, expressed
Chr.5	4137078	4137078	G	A	G	SNP	OS05G0169000	Exonic	3UTR	NA	LOC_Os05g07690	thioredoxin, putative, expressed
Chr.5	4137102	4137102	C	A	C	SNP	OS05G0169000	Exonic	3UTR	NA	LOC_Os05g07690	thioredoxin, putative, expressed
Chr.5	4137733	4137733	T	A	T	SNP	OS05G0169000	Exonic	3UTR	NA	LOC_Os05g07690	thioredoxin, putative, expressed
Chr.5	4137969	4137969	G	A	G	SNP	OS05G0169000	Exonic	CDS	NA	LOC_Os05g07690	thioredoxin, putative, expressed
Chr.5	4138022	4138022	T	A	T	SNP	OS05G0169000	Exonic	CDS	NA	LOC_Os05g07690	thioredoxin, putative, expressed
Chr.5	4138037	4138037	G	A	G	SNP	OS05G0169000	Exonic	CDS	NA	LOC_Os05g07690	thioredoxin, putative, expressed
Chr.5	4138206	4138206	C	A	C	SNP	OS05G0169000	Exonic	CDS	NA	LOC_Os05g07690	thioredoxin, putative, expressed
Chr.5	4138284	4138284	G	A	G	SNP	OS05G0169000	Exonic	5UTR	NA	LOC_Os05g07690	thioredoxin, putative, expressed
Chr.5	4138289	4138289	G	A	G	SNP	OS05G0169000	Exonic	5UTR	NA	LOC_Os05g07690	thioredoxin, putative, expressed
Chr.5	4138466	4138466	G	A	G	SNP	OS05G0169000	Exonic	5UTR	NA	LOC_Os05g07690	thioredoxin, putative, expressed

Chr.5	4138477	4138477	T	A	T	SNP	OS05G0169000	Exonic	5UTR	NA	LOC_Os05g07690	thioredoxin, putative, expressed
Chr.5	4142341	4142341	T	A	T	SNP	OS05G0169100	Intronic	NA	NA	LOC_Os05g07700	ribosomal protein, putative, expressed
Chr.5	4142386	4142386	G	A	G	SNP	OS05G0169100	Intronic	NA	NA	LOC_Os05g07700	ribosomal protein, putative, expressed
Chr.5	4142456	4142456	G	A	G	SNP	OS05G0169100	Intronic	NA	NA	LOC_Os05g07700	ribosomal protein, putative, expressed
Chr.5	4142494	4142494	G	A	G	SNP	OS05G0169100	Intronic	NA	NA	LOC_Os05g07700	ribosomal protein, putative, expressed
Chr.5	4142564	4142564	T	A	T	SNP	OS05G0169100	Intronic	NA	NA	LOC_Os05g07700	ribosomal protein, putative, expressed
Chr.5	4142594	4142594	G	A	G	SNP	OS05G0169100	Intronic	NA	NA	LOC_Os05g07700	ribosomal protein, putative, expressed
Chr.5	4142595	4142595	C	A	C	SNP	OS05G0169100	Intronic	NA	NA	LOC_Os05g07700	ribosomal protein, putative, expressed
Chr.5	4142797	4142797	G	A	G	SNP	OS05G0169100	Intronic	NA	NA	LOC_Os05g07700	ribosomal protein, putative, expressed
Chr.5	4142855	4142855	G	A	G	SNP	OS05G0169100	Intronic	NA	NA	LOC_Os05g07700	ribosomal protein, putative, expressed
Chr.5	4142959	4142959	C	A	C	SNP	OS05G0169100	Exonic	CDS	Silent	LOC_Os05g07700	ribosomal protein, putative, expressed
Chr.5	4143420	4143420	C	A	C	SNP	OS05G0169100	Intronic	NA	NA	LOC_Os05g07700	ribosomal protein, putative, expressed
Chr.5	4143461	4143461	C	A	C	SNP	OS05G0169100	Intronic	NA	NA	LOC_Os05g07700	ribosomal protein, putative, expressed
Chr.5	4143834	4143834	T	A	T	SNP	OS05G0169100	Exonic	3UTR	NA	LOC_Os05g07700	ribosomal protein, putative, expressed
Chr.5	4143860	4143860	G	A	G	SNP	OS05G0169100	Exonic	3UTR	NA	LOC_Os05g07700	ribosomal protein, putative, expressed
Chr.5	4302000	4302000	C	A	C	SNP	OS05G0171900	Intronic	NA	NA	LOC_Os05g07940	glyoxalase family protein, putative, expressed
Chr.5	4302020	4302020	G	A	G	SNP	OS05G0171900	Intronic	NA	NA	LOC_Os05g07940	glyoxalase family protein, putative, expressed
Chr.5	4302044	4302044	G	A	G	SNP	OS05G0171900	Intronic	NA	NA	LOC_Os05g07940	glyoxalase family protein, putative, expressed
Chr.5	4302068	4302068	G	A	G	SNP	OS05G0171900	Intronic	NA	NA	LOC_Os05g07940	glyoxalase family protein, putative, expressed
Chr.5	4302434	4302434	T	A	T	SNP	OS05G0171900	Exonic	3UTR	NA	LOC_Os05g07940	glyoxalase family protein, putative, expressed
Chr.5	4302657	4302657	G	A	G	SNP	OS05G0171900	Exonic	3UTR	NA	LOC_Os05g07940	glyoxalase family protein, putative, expressed
Chr.5	4302860	4302860	T	A	T	SNP	OS05G0171900	Exonic	3UTR	NA	LOC_Os05g07940	glyoxalase family protein, putative, expressed
Chr.5	4302881	4302881	G	A	G	SNP	OS05G0171900	Exonic	3UTR	NA	LOC_Os05g07940	glyoxalase family protein, putative, expressed
Chr.5	4303746	4303746	G	A	G	SNP	OS05G0171900	Intronic	NA	NA	LOC_Os05g07940	glyoxalase family protein, putative, expressed
Chr.5	4304642	4304642	G	A	G	SNP	OS05G0171900	Intronic	NA	NA	LOC_Os05g07940	glyoxalase family protein, putative, expressed
Chr.5	4305657	4305657	T	A	T	SNP	OS05G0171900	Exonic	3UTR	NA	LOC_Os05g07940	glyoxalase family protein, putative, expressed
Chr.5	4305704	4305704	C	A	C	SNP	OS05G0171900	Exonic	3UTR	NA	LOC_Os05g07940	glyoxalase family protein, putative, expressed
Chr.5	4305705	4305705	G	A	G	SNP	OS05G0171900	Exonic	3UTR	NA	LOC_Os05g07940	glyoxalase family protein, putative, expressed
Chr.5	4854365	4854365	T	A	T	SNP	OS05G0180600	Exonic	3UTR	NA	LOC_Os05g08810	phosphatidylinositol 3-kinase, root isoform, putative, expressed
Chr.5	4854528	4854528	C	A	C	SNP	OS05G0180600	Exonic	3UTR	NA	LOC_Os05g08810	phosphatidylinositol 3-kinase, root isoform, putative, expressed
Chr.5	4856402	4856402	T	A	T	SNP	OS05G0180600	Intronic	NA	NA	LOC_Os05g08810	phosphatidylinositol 3-kinase, root isoform, putative, expressed
Chr.5	4856948	4856948	G	A	G	SNP	OS05G0180600	Intronic	NA	NA	LOC_Os05g08810	phosphatidylinositol 3-kinase, root isoform, putative, expressed
Chr.5	4857006	4857006	T	A	T	SNP	OS05G0180600	Intronic	NA	NA	LOC_Os05g08810	phosphatidylinositol 3-kinase, root isoform, putative, expressed
Chr.5	4857140	4857140	C	A	C	SNP	OS05G0180600	Intronic	NA	NA	LOC_Os05g08810	phosphatidylinositol 3-kinase, root isoform, putative, expressed
Chr.5	4857208	4857208	G	A	G	SNP	OS05G0180600	Intronic	NA	NA	LOC_Os05g08810	phosphatidylinositol 3-kinase, root isoform, putative, expressed
Chr.5	4857418	4857418	G	A	G	SNP	OS05G0180600	Intronic	NA	NA	LOC_Os05g08810	phosphatidylinositol 3-kinase, root isoform, putative, expressed
Chr.5	4858323	4858323	G	A	G	SNP	OS05G0180600	Intronic	NA	NA	LOC_Os05g08810	phosphatidylinositol 3-kinase, root isoform, putative, expressed
Chr.5	4858404	4858404	G	A	G	SNP	OS05G0180600	Intronic	NA	NA	LOC_Os05g08810	phosphatidylinositol 3-kinase, root isoform, putative, expressed
Chr.5	4858798	4858798	C	A	C	SNP	OS05G0180600	Intronic	NA	NA	LOC_Os05g08810	phosphatidylinositol 3-kinase, root isoform, putative, expressed
Chr.5	4859100	4859100	G	A	G	SNP	OS05G0180600	Intronic	NA	NA	LOC_Os05g08810	phosphatidylinositol 3-kinase, root isoform, putative, expressed
Chr.5	4859461	4859461	T	A	T	SNP	OS05G0180600	Intronic	NA	NA	LOC_Os05g08810	phosphatidylinositol 3-kinase, root isoform, putative, expressed
Chr.5	4860645	4860645	T	A	T	SNP	OS05G0180600	Intronic	NA	NA	LOC_Os05g08810	phosphatidylinositol 3-kinase, root isoform, putative, expressed
Chr.5	4860906	4860906	G	A	G	SNP	OS05G0180600	Exonic	CDS	Silent	LOC_Os05g08810	phosphatidylinositol 3-kinase, root isoform, putative, expressed
Chr.5	4861503	4861503	G	A	G	SNP	OS05G0180600	Intronic	NA	NA	LOC_Os05g08810	phosphatidylinositol 3-kinase, root isoform, putative, expressed
Chr.5	4862017	4862017	G	A	G	SNP	OS05G0180600	Exonic	CDS	Silent	LOC_Os05g08810	phosphatidylinositol 3-kinase, root isoform, putative, expressed
Chr.5	4862599	4862599	G	A	G	SNP	OS05G0180600	Intronic	NA	NA	LOC_Os05g08810	phosphatidylinositol 3-kinase, root isoform, putative, expressed
Chr.5	4862904	4862904	G	A	G	SNP	OS05G0180600	Intronic	NA	NA	LOC_Os05g08810	phosphatidylinositol 3-kinase, root isoform, putative, expressed
Chr.5	4862934	4862934	G	A	G	SNP	OS05G0180600	Intronic	NA	NA	LOC_Os05g08810	phosphatidylinositol 3-kinase, root isoform, putative, expressed
Chr.5	4864200	4864200	C	A	C	SNP	OS05G0180600	Intronic	NA	NA	LOC_Os05g08810	phosphatidylinositol 3-kinase, root isoform, putative, expressed
Chr.5	4864250	4864250	G	A	G	SNP	OS05G0180600	Intronic	NA	NA	LOC_Os05g08810	phosphatidylinositol 3-kinase, root isoform, putative, expressed
Chr.5	4864359	4864359	C	A	C	SNP	OS05G0180600	Intronic	NA	NA	LOC_Os05g08810	phosphatidylinositol 3-kinase, root isoform, putative, expressed
Chr.5	4864460	4864460	G	A	G	SNP	OS05G0180600	Intronic	NA	NA	LOC_Os05g08810	phosphatidylinositol 3-kinase, root isoform, putative, expressed
Chr.5	4864705	4864705	C	A	C	SNP	OS05G0180600	Intronic	NA	NA	LOC_Os05g08810	phosphatidylinositol 3-kinase, root isoform, putative, expressed
Chr.5	4864864	4864864	G	A	G	SNP	OS05G0180600	Intronic	NA	NA	LOC_Os05g08810	phosphatidylinositol 3-kinase, root isoform, putative, expressed
Chr.5	5653407	5653407	T	A	T	SNP	OS05G0192100	Intronic	NA	NA	LOC_Os05g10370	acid phosphatase, putative, expressed
Chr.5	5653842	5653842	C	A	C	SNP	OS05G0192100	Intronic	NA	NA	LOC_Os05g10370	acid phosphatase, putative, expressed
Chr.5	5653981	5653981	G	A	G	SNP	OS05G0192100	Intronic	NA	NA	LOC_Os05g10370	acid phosphatase, putative, expressed
Chr.5	6191321	6191321	T	A	T	SNP	OS05G0199500	Exonic	3UTR	NA	LOC_Os05g11060	galactosyltransferase family protein, putative, expressed
Chr.5	6193386	6193386	T	A	T	SNP	OS05G0199500	Intronic	NA	NA	LOC_Os05g11060	galactosyltransferase family protein, putative, expressed
Chr.5	6194380	6194380	T	A	T	SNP	OS05G0199500	Intronic	NA	NA	LOC_Os05g11060	galactosyltransferase family protein, putative, expressed
Chr.5	7163635	7163635	T	A	T	SNP	OS05G0215600	Exonic	3UTR	NA	LOC_Os05g12474	expressed protein
Chr.5	7721744	7721744	G	A	G	SNP	OS05G0226900	Exonic	3UTR	NA	LOC_Os05g13900	retrotransposon protein, putative, unclassified, expressed
Chr.5	7722418	7722418	G	A	G	SNP	OS05G0226900	Exonic	CDS	Silent	LOC_Os05g13900	retrotransposon protein, putative, unclassified, expressed
Chr.5	8002503	8002503	T	A	T	SNP	OS05G0231700	Exonic	CDS	NA	LOC_Os05g14240	aquaporin protein, putative, expressed
Chr.5	9318241	9318241	G	A	G	SNP	OS05G0253200	Intronic	NA	NA	LOC_Os05g16420	SHR5-receptor-like kinase, putative, expressed
Chr.5	9318501	9318501	T	A	T	SNP	OS05G0253200	Exonic	CDS	Missense	LOC_Os05g16420	SHR5-receptor-like kinase, putative, expressed
Chr.5	9322937	9322937	T	A	T	SNP	OS05G0253200	Intronic	NA	NA	LOC_Os05g16420	SHR5-receptor-like kinase, putative, expressed
Chr.5	9326156	9326156	G	A	G	SNP	OS05G0253200	Intronic	NA	NA	LOC_Os05g16420	SHR5-receptor-like kinase, putative, expressed
Chr.5	9328241	9328241	T	A	T	SNP	OS05G0253200	Exonic	3UTR	NA	LOC_Os05g16420	SHR5-receptor-like kinase, putative, expressed
Chr.5	10805362	10805362	G	A	G	SNP	OS05G0269100	Exonic	CDS	Missense	LOC_Os05g18660	OsFBDUF24 - F-box and DUF domain containing protein, expressed
Chr.5	12455294	12455294	C	A	C	SNP	OS05G0287800	Intronic	NA	NA	LOC_Os05g21180	phosphatidic acid phosphatase-related, putative, expressed
Chr.5	12457613	12457613	C	A	C	SNP	OS05G0287800	Exonic	3UTR	NA	LOC_Os05g21180	phosphatidic acid phosphatase-related, putative, expressed
Chr.5	12457632	12457632	T	A	T	SNP	OS05G0287800	Exonic	3UTR	NA	LOC_Os05g21180	phosphatidic acid phosphatase-related, putative, expressed
Chr.5	13309228	13309228	G	A	G	SNP	OS05G0298200	Intronic	NA	NA	LOC_Os05g23320	expressed protein
Chr.5	13312297	13312297	G	A	G	SNP	OS05G0298200	Exonic	3UTR	NA	LOC_Os05g23320	expressed protein
Chr.5	13312364	13312364	G	A	G	SNP	OS05G0298200	Exonic	3UTR	NA	LOC_Os05g23320	expressed protein
Chr.5	13422829	13422829	C	A	C	SNP	OS05G0299700	Exonic	CDS	Silent	LOC_Os05g23470	AN1-like zinc finger domain containing protein, expressed
Chr.5	13529908	13529908	G	A	G	SNP	OS05G0301500	Intronic	NA	NA	LOC_Os05g23600	dolichyl-diphosphooligosaccharide--protein glycosyltransferase 67 kDasubunit precursor, putative, expressed
Chr.5	13545714	13545714	G	A	G	SNP	OS05G0301700	Exonic	CDS	Silent	LOC_Os05g23620	cytochrome c1-1, heme protein, mitochondrial precursor, putative, expressed
Chr.5	13836654	13836654	G	A	G	SNP	OS05G0305900	Exonic	CDS	Missense	LOC_Os05g24010	receptor protein kinase-like, putative, expressed
Chr.5	14298049	14298049	C	A	C	SNP	OS05G0312000	Intronic	NA	NA	LOC_Os05g24684	structural constituent of ribosome, putative, expressed
Chr.5	14300205	14300205	G	A	G	SNP	OS05G0312000	Exonic	CDS	Missense	LOC_Os05g24684	structural constituent of ribosome, putative, expressed
Chr.5	14307061	14307061	T	A	T	SNP	OS05G0312000	Intronic	NA	NA	LOC_Os05g24684	structural constituent of ribosome, putative, expressed
Chr.5	14307755	14307755	G	A	G	SNP	OS05G0312000	Intronic	NA	NA	LOC_Os05g24684	structural constituent of ribosome, putative, expressed
Chr.5	14308008	14308008	G	A	G	SNP	OS05G0312000	Intronic	NA	NA	LOC_Os05g24684	structural constituent of ribosome, putative, expressed

Chr.5	15486927	15486927	G	A	G	SNP	OS05G0331200	Intronic	NA	NA	LOC_Os05g26660	NADH-ubiquinone oxidoreductase, mitochondrial precursor, putative, expressed
Chr.5	17176577	17176577	C	A	C	SNP	OS05G0360400	Exonic	3UTR	NA	LOC_Os05g29710	RING-H2 finger protein, putative, expressed
Chr.5	17932713	17932713	G	A	G	SNP	OS05G0372200	Exonic	CDS	Missense	LOC_Os05g30880	nucleolar matrix protein-related, putative, expressed
Chr.5	18843805	18843805	T	A	T	SNP	OS05G0389400	Intronic	NA	NA	LOC_Os05g32320	integral membrane transporter family protein, putative, expressed
Chr.5	19960727	19960727	G	A	G	SNP	OS05G0408900	Intronic	NA	NA	LOC_Os05g33840	transketolase, putative, expressed
Chr.5	22161161	22161161	G	A	G	SNP	OS05G0451300	Intronic	NA	NA	LOC_Os05g37830	expressed protein
Chr.5	23423654	23423654	G	A	G	SNP	OS05G0476200	Intronic	NA	NA	LOC_Os05g39850	MCM3 - Putative minichromosome maintenance MCM complex subunit 3, expressed
Chr.5	23424468	23424468	C	A	C	SNP	OS05G0476200	Exonic	3UTR	NA	LOC_Os05g39850	MCM3 - Putative minichromosome maintenance MCM complex subunit 3, expressed
Chr.5	24104885	24104885	T	A	T	SNP	OS05G0490400	Exonic	CDS	Missense	LOC_Os05g41150	expressed protein
Chr.5	24105206	24105206	G	A	G	SNP	OS05G0490400	Exonic	CDS	Silent	LOC_Os05g41150	expressed protein
Chr.5	24132957	24132957	G	A	G	SNP	OS05G0491100	Exonic	CDS	Silent	LOC_Os05g41210	OsCam2 - Calmodulin, expressed
Chr.5	24329892	24329892	C	A	C	SNP	OS05G0495300	Exonic	5UTR	NA	LOC_Os05g41550	expressed protein
Chr.5	24671030	24671030	G	A	G	SNP	OS05G0501300	Exonic	CDS	Silent	LOC_Os05g42190	NADPH-dependent FMN reductase domain containing protein, expressed
Chr.5	24779688	24779688	G	A	G	SNP	OS05G0503300	Intronic	NA	NA	LOC_Os05g42350	ferredoxin--nitrite reductase, putative, expressed
Chr.5	24780439	24780439	G	A	G	SNP	OS05G0503300	Exonic	CDS	Missense	LOC_Os05g42350	ferredoxin--nitrite reductase, putative, expressed
Chr.5	25230943	25230943	C	A	C	SNP	OS05G0509500	Exonic	CDS	Missense	LOC_Os05g43390	signal recognition particle 54 kDa protein, putative, expressed
Chr.5	25383759	25383759	T	A	T	SNP	OS05G0512200	Intronic	NA	NA	LOC_Os05g43650	expressed protein
Chr.5	25384914	25384914	G	A	G	SNP	OS05G0512200	Exonic	CDS	Silent	LOC_Os05g43650	expressed protein
Chr.5	27536224	27536224	T	A	T	SNP	OS05G0553800	Intronic	NA	NA	LOC_Os05g48030	anti-silencing protein, ASF1-like domain containing protein, expressed
Chr.5	27632346	27632346	G	A	G	SNP	OS05G0555600	Intronic	NA	NA	LOC_Os05g48200	glutamate synthase, chloroplast precursor, putative, expressed
Chr.5	27701651	27701651	G	A	G	SNP	OS05G0557000	Exonic	3UTR	NA	LOC_Os05g48320	60S ribosomal protein L37a, putative, expressed
Chr.5	28192735	28192735	G	A	G	SNP	OS05G0566400	Exonic	CDS	Missense	LOC_Os05g49140	CGMC_MAPKCMGC_2.8 - CGMC includes CDA, MAPK, GSK3, and CLKC kinases, expressed
Chr.5	338924	338925	AT	A	AT	INDEL	OS05G0106200	Exonic	5UTR	NA	LOC_Os05g01570	auxin-induced protein 5NG4, putative, expressed
Chr.5	3731115	3731116	AG	A	AG	INDEL	OS05G0163700	Intronic	NA	NA	LOC_Os05g07090	acyl-coenzyme A dehydrogenase, mitochondrial precursor, putative, expressed
Chr.5	4137925	4137928	AAAG	A	AAAG	INDEL	OS05G0169000	Exonic	CDS	NA	LOC_Os05g07690	thioredoxin, putative, expressed
Chr.5	4304804	4304805	AT	A	AT	INDEL	OS05G0171900	Intronic	NA	NA	LOC_Os05g07940	glyoxalase family protein, putative, expressed
Chr.5	4858789	4858790	AT	A	AT	INDEL	OS05G0180600	Intronic	NA	NA	LOC_Os05g08810	phosphatidylinositol 3-kinase, root isoform, putative, expressed
Chr.5	4862252	4862254	ATT	A	ATT	INDEL	OS05G0180600	Intronic	NA	NA	LOC_Os05g08810	phosphatidylinositol 3-kinase, root isoform, putative, expressed
Chr.5	7163218	7163219	AT	A	AT	INDEL	OS05G0215600	Exonic	3UTR	NA	LOC_Os05g12474	expressed protein
Chr.5	26825468	26825469	AT	A	AT	INDEL	OS05G0540000	Intronic	NA	NA	LOC_Os05g46260	exostosin, putative, expressed
Chr.5	27701024	27701027	AATC	A	AATC	INDEL	OS05G0557000	Intronic	NA	NA	LOC_Os05g48320	60S ribosomal protein L37a, putative, expressed
Chr.5	27715244	27715245	AT	A	AT	INDEL	OS05G0557200	Intronic	NA	NA	LOC_Os05g48340	proteasome-related, putative, expressed
Chr.5	2029599	2029599	G	A	G	SNP	OS05G0134700	Exonic	CDS	Silent	LOC_Os05g04410	peroxidase precursor, putative, expressed
Chr.5	2177156	2177156	G	A	G	SNP	OS05G0137400	Intronic	NA	NA	LOC_Os05g04630	retrotransposon protein, putative, SINE subclass, expressed
Chr.5	3734953	3734953	G	A	G	SNP	OS05G0163700	Exonic	CDS	Silent	LOC_Os05g07090	acyl-coenzyme A dehydrogenase, mitochondrial precursor, putative, expressed
Chr.5	3758096	3758096	G	A	G	SNP	OS05G0163900	Intronic	NA	NA	LOC_Os05g07120	basic helix-loop-helix, putative, expressed
Chr.5	3758104	3758104	C	A	C	SNP	OS05G0163900	Intronic	NA	NA	LOC_Os05g07120	basic helix-loop-helix, putative, expressed
Chr.5	3759746	3759746	G	A	G	SNP	OS05G0163900	Exonic	3UTR	NA	LOC_Os05g07120	basic helix-loop-helix, putative, expressed
Chr.5	4137377	4137377	G	A	G	SNP	OS05G0169000	Exonic	3UTR	NA	LOC_Os05g07690	thioredoxin, putative, expressed
Chr.5	4137394	4137394	G	A	G	SNP	OS05G0169000	Exonic	3UTR	NA	LOC_Os05g07690	thioredoxin, putative, expressed
Chr.5	4137435	4137435	G	A	G	SNP	OS05G0169000	Exonic	3UTR	NA	LOC_Os05g07690	thioredoxin, putative, expressed
Chr.5	4137535	4137535	G	A	G	SNP	OS05G0169000	Exonic	3UTR	NA	LOC_Os05g07690	thioredoxin, putative, expressed
Chr.5	4137703	4137703	G	A	G	SNP	OS05G0169000	Exonic	3UTR	NA	LOC_Os05g07690	thioredoxin, putative, expressed
Chr.5	4137773	4137773	C	A	C	SNP	OS05G0169000	Exonic	3UTR	NA	LOC_Os05g07690	thioredoxin, putative, expressed
Chr.5	4137819	4137819	T	A	T	SNP	OS05G0169000	Exonic	3UTR	NA	LOC_Os05g07690	thioredoxin, putative, expressed
Chr.5	4138385	4138385	G	A	G	SNP	OS05G0169000	Exonic	5UTR	NA	LOC_Os05g07690	thioredoxin, putative, expressed
Chr.5	4138550	4138550	C	A	C	SNP	OS05G0169000	Exonic	5UTR	NA	LOC_Os05g07690	thioredoxin, putative, expressed
Chr.5	4138691	4138691	G	A	G	SNP	OS05G0169000	Exonic	5UTR	NA	LOC_Os05g07690	thioredoxin, putative, expressed
Chr.5	4138696	4138696	G	A	G	SNP	OS05G0169000	Exonic	5UTR	NA	LOC_Os05g07690	thioredoxin, putative, expressed
Chr.5	4138714	4138714	G	A	G	SNP	OS05G0169000	Exonic	5UTR	NA	LOC_Os05g07690	thioredoxin, putative, expressed
Chr.5	4305493	4305493	G	A	G	SNP	OS05G0171900	Exonic	CDS	Silent	LOC_Os05g07940	glyoxalase family protein, putative, expressed
Chr.5	4862727	4862727	C	A	C	SNP	OS05G0180600	Intronic	NA	NA	LOC_Os05g08810	phosphatidylinositol 3-kinase, root isoform, putative, expressed
Chr.5	4865352	4865352	C	A	C	SNP	OS05G0180600	Exonic	5UTR	NA	LOC_Os05g08810	phosphatidylinositol 3-kinase, root isoform, putative, expressed
Chr.5	4927154	4927154	G	A	G	SNP	OS05G0181700	Exonic	CDS	Silent	LOC_Os05g08900	expressed protein
Chr.5	7721954	7721954	T	A	T	SNP	OS05G0226900	Exonic	3UTR	NA	LOC_Os05g13900	retrotransposon protein, putative, unclassified, expressed
Chr.5	7722102	7722102	G	A	G	SNP	OS05G0226900	Exonic	CDS	Missense	LOC_Os05g13900	retrotransposon protein, putative, unclassified, expressed
Chr.5	7722581	7722581	G	A	G	SNP	OS05G0226900	Exonic	CDS	Missense	LOC_Os05g13900	retrotransposon protein, putative, unclassified, expressed
Chr.5	8902593	8902593	C	A	C	SNP	OS05G0247100	Exonic	3UTR	NA	LOC_Os05g15770	glycosyl hydrolase, putative, expressed
Chr.5	8903025	8903025	G	A	G	SNP	OS05G0247100	Exonic	CDS	Silent	LOC_Os05g15770	glycosyl hydrolase, putative, expressed
Chr.5	8903451	8903451	G	A	G	SNP	OS05G0247100	Exonic	CDS	Silent	LOC_Os05g15770	glycosyl hydrolase, putative, expressed
Chr.5	9321368	9321368	T	A	T	SNP	OS05G0253200	Intronic	NA	NA	LOC_Os05g16420	SHR5-receptor-like kinase, putative, expressed
Chr.5	9321407	9321407	G	A	G	SNP	OS05G0253200	Intronic	NA	NA	LOC_Os05g16420	SHR5-receptor-like kinase, putative, expressed
Chr.5	9326337	9326337	G	A	G	SNP	OS05G0253200	Exonic	3UTR	NA	LOC_Os05g16420	SHR5-receptor-like kinase, putative, expressed
Chr.5	9327446	9327446	G	A	G	SNP	OS05G0253200	Exonic	3UTR	NA	LOC_Os05g16420	SHR5-receptor-like kinase, putative, expressed
Chr.5	11394386	11394386	G	A	G	SNP	OS05G0276500	Exonic	CDS	Silent	LOC_Os05g19570	expansin precursor, putative, expressed
Chr.5	13312401	13312401	G	A	G	SNP	OS05G0298200	Exonic	3UTR	NA	LOC_Os05g23320	expressed protein
Chr.5	13312403	13312403	G	A	G	SNP	OS05G0298200	Exonic	3UTR	NA	LOC_Os05g23320	expressed protein
Chr.5	13422562	13422562	C	A	C	SNP	OS05G0299700	Exonic	3UTR	NA	LOC_Os05g23470	AN1-like zinc finger domain containing protein, expressed
Chr.5	15484400	15484400	C	A	C	SNP	OS05G0331200	Intronic	NA	NA	LOC_Os05g26660	NADH-ubiquinone oxidoreductase, mitochondrial precursor, putative, expressed
Chr.5	15487923	15487923	G	A	G	SNP	OS05G0331200	Intronic	NA	NA	LOC_Os05g26660	NADH-ubiquinone oxidoreductase, mitochondrial precursor, putative, expressed
Chr.5	17583587	17583587	C	A	C	SNP	OS05G0366300	Intronic	NA	NA	LOC_Os05g30340	expressed protein
Chr.5	19958900	19958900	G	A	G	SNP	OS05G0408900	Exonic	CDS	Silent	LOC_Os05g33840	transketolase, putative, expressed
Chr.5	23866248	23866248	G	A	G	SNP	OS05G0485300	Intronic	NA	NA	LOC_Os05g40700	transmembrane protein 56, putative, expressed
Chr.5	23866594	23866594	G	A	G	SNP	OS05G0485300	Intronic	NA	NA	LOC_Os05g40700	transmembrane protein 56, putative, expressed
Chr.5	24106252	24106252	G	A	G	SNP	OS05G0490400	Exonic	5UTR	NA	LOC_Os05g41150	expressed protein
Chr.5	24281519	24281519	G	A	G	SNP	OS05G0494200	Exonic	CDS	Silent	LOC_Os05g41460	cysteine proteinase inhibitor precursor protein, putative, expressed
Chr.5	24329909	24329909	G	A	G	SNP	OS05G0495300	Exonic	5UTR	NA	LOC_Os05g41550	expressed protein
Chr.5	24632420	24632420	G	A	G	SNP	OS05G0500600	Exonic	CDS	Missense	LOC_Os05g42130	MONOCULM 1, putative, expressed
Chr.5	24633525	24633525	G	A	G	SNP	OS05G0500700	Exonic	CDS	NA	LOC_Os05g42140	methyltransferase domain containing protein, expressed
Chr.5	25544856	25544856	G	A	G	SNP	OS05G0515200	Exonic	CDS	Missense	LOC_Os05g43910	cytochrome P450, putative, expressed
Chr.5	26371657	26371657	C	A	C	SNP	OS05G0531200	Exonic	5UTR	NA	LOC_Os05g45460	POEI52 - Pollen Ole e I allergen and extensin family protein precursor, expressed
Chr.5	26371742	26371742	G	A	G	SNP	OS05G0531200	Exonic	CDS	Silent	LOC_Os05g45460	POEI52 - Pollen Ole e I allergen and extensin family protein precursor, expressed
Chr.5	26371841	26371841	C	A	C	SNP	OS05G0531200	Exonic	CDS	Silent	LOC_Os05g45460	POEI52 - Pollen Ole e I allergen and extensin family protein precursor, expressed
Chr.5	26372827	26372827	G	A	G	SNP	OS05G0531200	Exonic	3UTR	NA	LOC_Os05g45460	POEI52 - Pollen Ole e I allergen and extensin family protein precursor, expressed
Chr.5	26903704	26903704	T	A	T	SNP	OS05G0541900	Exonic	3UTR	NA	LOC_Os05g46430	60S ribosomal protein L28-1, putative, expressed

Chr.5	26928618	26928618	G	A	G	SNP	OS05G0542500	Exonic	3UTR	NA	LOC_Os05g46480	late embryogenesis abundant protein, group 3, putative, expressed
Chr.5	27122046	27122046	G	A	G	SNP	OS05G0546400	Exonic	CDS	Silent	LOC_Os05g46840	proline-rich protein, putative, expressed
Chr.5	27461792	27461792	G	A	G	SNP	OS05G0552300	Exonic	5UTR	NA	LOC_Os05g47890	WD domain, G-beta repeat domain containing protein, expressed
Chr.5	27632624	27632624	C	A	C	SNP	OS05G0555600	Intronic	NA	NA	LOC_Os05g48200	glutamate synthase, chloroplast precursor, putative, expressed
Chr.5	27633461	27633461	G	A	G	SNP	OS05G0555600	Intronic	NA	NA	LOC_Os05g48200	glutamate synthase, chloroplast precursor, putative, expressed
Chr.5	27633811	27633811	G	A	G	SNP	OS05G0555600	Intronic	NA	NA	LOC_Os05g48200	glutamate synthase, chloroplast precursor, putative, expressed
Chr.5	27959953	27959953	T	A	T	SNP	OS05G0562000	Intronic	NA	NA	LOC_Os05g48790	expressed protein
Chr.5	27959967	27959967	G	A	G	SNP	OS05G0562000	Intronic	NA	NA	LOC_Os05g48790	expressed protein
Chr.5	4137917	4137919	AAG	A	AAG	INDEL	OS05G0169000	Exonic	CDS	NA	LOC_Os05g07690	thioredoxin, putative, expressed
Chr.5	4864883	4864885	ATT	A	ATT	INDEL	OS05G0180600	Intronic	NA	NA	LOC_Os05g08810	phosphatidylinositol 3-kinase, root isoform, putative, expressed
Chr.5	7167280	7167283	ATTT	A	ATTT	INDEL	OS05G0215600	Intronic	NA	NA	LOC_Os05g12474	expressed protein
Chr.5	9325323	9325326	ACCC	A	ACCC	INDEL	OS05G0253200	Exonic	3UTR	NA	LOC_Os05g16420	SHR5-receptor-like kinase, putative, expressed
Chr.5	9325329	9325331	AAC	A	AAC	INDEL	OS05G0253200	Exonic	3UTR	NA	LOC_Os05g16420	SHR5-receptor-like kinase, putative, expressed
Chr.5	19633370	19633374	AAAAG	A	AAAAG	INDEL	OS05G0402900	Exonic	3UTR	NA	LOC_Os05g33400	basic 7S globulin precursor, putative, expressed
Chr.5	19770655	19770673	ATAGAGGAGTACATGCTTT	A	ATAGAGGAGTACATGCTTT	INDEL	OS05G0405900	Intronic	NA	NA	LOC_Os05g33610	expressed protein
Chr.5	19828969	19828971	AGT	A	AGT	INDEL	OS05G0406800	Exonic	5UTR	NA	LOC_Os05g33690	receptor-like protein kinase precursor, putative, expressed
Chr.5	23752582	23752585	ACAC	A	ACAC	INDEL	OS05G0482700	Intronic	NA	NA	LOC_Os05g40420	2,3-bisphosphoglycerate-independent phosphoglycerate mutase, putative, expressed
Chr.5	24134810	24134813	AGAG	A	AGAG	INDEL	OS05G0491100	Exonic	5UTR	NA	LOC_Os05g41210	OsCam2 - Calmodulin, expressed
Chr.5	27164608	27164611	AGGG	A	AGGG	INDEL	OS05G0547600	Intronic	NA	NA	LOC_Os05g46950	expressed protein
Chr.7	13367218	13367218	G	A	G	SNP	OS07G0418700	Exonic	CDS	NA	LOC_Os07g23660	retrotransposon protein, putative, unclassified, expressed
Chr.7	24397000	24397000	G	A	G	SNP	OS07G0598200	Exonic	CDS	Silent	LOC_Os07g40710	circadian clock coupling factor-related, putative, expressed
Chr.7	15378182	15378182	T	A	T	SNP	OS07G0448400	Exonic	3UTR	NA	LOC_Os07g26660	aquaporin protein, putative, expressed
Chr.7	26053260	26053260	T	A	T	SNP	OS07G0628700	Exonic	CDS	Silent	LOC_Os07g43560	TKL_IRAK_DUF26-lc.24 - DUF26 kinases have homology to DUF26 containing loci, expressed
Chr.7	28274653	28274653	T	A	T	SNP	OS07G0669100	Intronic	NA	NA	LOC_Os07g47290	xylose isomerase, putative, expressed
Chr.7	20649	20649	G	A	G	SNP	OS07G0100300	Exonic	CDS	Missense	LOC_Os07g01030	glycosyl transferase, group 1 domain containing protein, expressed
Chr.7	2644598	2644598	G	A	G	SNP	OS07G0150700	Exonic	CDS	Silent	LOC_Os07g05620	CAMK_KIN1/SNF1/Nim1_like.28 - CAMK includes calcium/calmodulin depedent protein kinases, expressed
Chr.7	13103658	13103658	G	A	G	SNP	OS07G0415200	Exonic	3UTR	NA	LOC_Os07g23244	ribosomal protein L23 family protein, putative, expressed
Chr.7	15282106	15282106	G	A	G	SNP	OS07G0446600	Exonic	3UTR	NA	LOC_Os07g26520	expressed protein
Chr.7	18763623	18763623	G	A	G	SNP	OS07G0499300	Exonic	3UTR	NA	LOC_Os07g31599	expressed protein
Chr.7	21379846	21379846	G	A	G	SNP	OS07G0541400	Exonic	CDS	Missense	LOC_Os07g35690	TKL_IRAK_DUF26-lc.3 - DUF26 kinases have homology to DUF26 containing loci, expressed
Chr.7	21380428	21380428	G	A	G	SNP	OS07G0541400	Intronic	NA	NA	LOC_Os07g35690	TKL_IRAK_DUF26-lc.3 - DUF26 kinases have homology to DUF26 containing loci, expressed
Chr.7	23058980	23058980	G	A	G	SNP	OS07G0571300	Exonic	3UTR	NA	LOC_Os07g38370	expressed protein
Chr.7	23186152	23186152	G	A	G	SNP	OS07G0573600	Intronic	NA	NA	LOC_Os07g38600	REX1 DNA Repair family protein, expressed
Chr.7	23927042	23927042	G	A	G	SNP	OS07G0588000	Intronic	NA	NA	LOC_Os07g39900	interferon-related developmental regulator, putative, expressed
Chr.7	23928900	23928900	G	A	G	SNP	OS07G0588000	Exonic	CDS	Missense	LOC_Os07g39900	interferon-related developmental regulator, putative, expressed
Chr.7	23930414	23930414	G	A	G	SNP	OS07G0588000	Exonic	CDS	Silent	LOC_Os07g39900	interferon-related developmental regulator, putative, expressed
Chr.7	23930800	23930800	G	A	G	SNP	OS07G0588000	Intronic	NA	NA	LOC_Os07g39900	interferon-related developmental regulator, putative, expressed
Chr.7	24396815	24396815	G	A	G	SNP	OS07G0598200	Exonic	5UTR	NA	LOC_Os07g40710	circadian clock coupling factor-related, putative, expressed
Chr.7	24655681	24655681	G	A	G	SNP	OS07G0603100	Exonic	CDS	Missense	LOC_Os07g41180	RNA-binding protein-like, putative, expressed
Chr.7	24658388	24658388	G	A	G	SNP	OS07G0603100	Intronic	NA	NA	LOC_Os07g41180	RNA-binding protein-like, putative, expressed
Chr.7	24658811	24658811	G	A	G	SNP	OS07G0603100	Intronic	NA	NA	LOC_Os07g41180	RNA-binding protein-like, putative, expressed
Chr.7	24966719	24966719	G	A	G	SNP	OS07G0607500	Intronic	NA	NA	LOC_Os07g41660	expressed protein
Chr.7	24967890	24967890	G	A	G	SNP	OS07G0607500	Intronic	NA	NA	LOC_Os07g41660	expressed protein
Chr.7	24990075	24990075	G	A	G	SNP	OS07G0607800	Intronic	NA	NA	LOC_Os07g41694	acidic leucine-rich nuclear phosphoprotein 32-related protein 1, putative, expressed
Chr.7	24990101	24990101	G	A	G	SNP	OS07G0607800	Intronic	NA	NA	LOC_Os07g41694	acidic leucine-rich nuclear phosphoprotein 32-related protein 1, putative, expressed
Chr.7	24990115	24990115	G	A	G	SNP	OS07G0607800	Intronic	NA	NA	LOC_Os07g41694	acidic leucine-rich nuclear phosphoprotein 32-related protein 1, putative, expressed
Chr.7	26776534	26776534	G	A	G	SNP	OS07G0642900	Exonic	CDS	Silent	LOC_Os07g44840	bacterial transferase hexapeptide domain containing protein, expressed
Chr.7	26776580	26776580	G	A	G	SNP	OS07G0642900	Intronic	NA	NA	LOC_Os07g44840	bacterial transferase hexapeptide domain containing protein, expressed
Chr.7	925408	925408	G	A	G	SNP	OS07G0116900	Exonic	3UTR	NA	LOC_Os07g02570	NB-ARC domain containing protein, expressed
Chr.7	13354566	13354566	G	A	G	SNP	OS07G0418600	Exonic	CDS	NA	LOC_Os07g23640	retrotransposon protein, putative, Ty3-gypsy subclass, expressed
Chr.7	18762176	18762176	G	A	G	SNP	OS07G0499300	Exonic	5UTR	NA	LOC_Os07g31599	expressed protein
Chr.7	18763239	18763239	G	A	G	SNP	OS07G0499300	Exonic	CDS	Missense	LOC_Os07g31599	expressed protein
Chr.7	28274570	28274570	G	A	G	SNP	OS07G0669100	Exonic	CDS	Missense	LOC_Os07g47290	xylose isomerase, putative, expressed
Chr.7	28274654	28274654	G	A	G	SNP	OS07G0669100	Intronic	NA	NA	LOC_Os07g47290	xylose isomerase, putative, expressed
Chr.7	926510	926510	C	A	C	SNP	OS07G0116900	Exonic	CDS	Missense	LOC_Os07g02570	NB-ARC domain containing protein, expressed
Chr.7	24991855	24991855	C	A	C	SNP	OS07G0607800	Intronic	NA	NA	LOC_Os07g41694	acidic leucine-rich nuclear phosphoprotein 32-related protein 1, putative, expressed
Chr.7	26777083	26777083	C	A	C	SNP	OS07G0642900	Intronic	NA	NA	LOC_Os07g44840	bacterial transferase hexapeptide domain containing protein, expressed
Chr.7	27553195	27553195	C	A	C	SNP	OS07G0655400	Intronic	NA	NA	LOC_Os07g46170	OsRhmbd13 - Putative Rhomboid homologue, expressed
Chr.7	13366631	13366631	C	A	C	SNP	OS07G0418700	Exonic	CDS	NA	LOC_Os07g23660	retrotransposon protein, putative, unclassified, expressed
Chr.7	3608332	3608332	C	A	C	SNP	OS07G0167100	Intronic	NA	NA	LOC_Os07g07260	glutamine-dependent NAD, putative, expressed
Chr.7	4663556	4663556	C	A	C	SNP	OS07G0187400	Exonic	3UTR	NA	LOC_Os07g08970	uncharacterized protein ycf45, putative, expressed
Chr.7	13367086	13367086	C	A	C	SNP	OS07G0418700	Exonic	CDS	NA	LOC_Os07g23660	retrotransposon protein, putative, unclassified, expressed
Chr.7	18337069	18337069	C	A	C	SNP	OS07G0492000	Exonic	CDS	Silent	LOC_Os07g30970	nucleoside diphosphate kinase, putative, expressed
Chr.7	19235022	19235022	C	A	C	SNP	OS07G0506700	Exonic	CDS	Silent	LOC_Os07g32350	WD domain, G-beta repeat domain containing protein, expressed
Chr.7	21245118	21245118	C	A	C	SNP	OS07G0539400	Intronic	NA	NA	LOC_Os07g35520	glucan endo-1,3-beta-glucosidase precursor, putative, expressed
Chr.7	21245405	21245405	C	A	C	SNP	OS07G0539400	Intronic	NA	NA	LOC_Os07g35520	glucan endo-1,3-beta-glucosidase precursor, putative, expressed
Chr.7	21848476	21848476	C	A	C	SNP	OS07G0550500	Intronic	NA	NA	LOC_Os07g36544	serine/threonine-protein kinase receptor precursor, putative, expressed
Chr.7	22185120	22185120	C	A	C	SNP	OS07G0556200	Intronic	NA	NA	LOC_Os07g37030	cytochrome b6-f complex iron-sulfur subunit, chloroplast precursor, putative, expressed
Chr.7	22889319	22889319	C	A	C	SNP	OS07G0569100	Exonic	3UTR	NA	LOC_Os07g38170	remorin, putative, expressed
Chr.7	24987688	24987688	C	A	C	SNP	OS07G0607800	Intronic	NA	NA	LOC_Os07g41694	acidic leucine-rich nuclear phosphoprotein 32-related protein 1, putative, expressed
Chr.7	26102456	26102456	C	A	C	SNP	OS07G0629500	Exonic	3UTR	NA	LOC_Os07g43604	expressed protein
Chr.7	28780585	28780585	C	A	C	SNP	OS07G0679500	Intronic	NA	NA	LOC_Os07g48180	bZIP transcription factor domain containing protein, expressed
Chr.7	351654	351654	G	A	G	SNP	OS07G0106200	Intronic	NA	NA	LOC_Os07g01560	transporter family protein, putative, expressed
Chr.7	351837	351837	G	A	G	SNP	OS07G0106200	Intronic	NA	NA	LOC_Os07g01560	transporter family protein, putative, expressed
Chr.7	351928	351928	G	A	G	SNP	OS07G0106200	Intronic	NA	NA	LOC_Os07g01560	transporter family protein, putative, expressed
Chr.7	353306	353306	G	A	G	SNP	OS07G0106200	Intronic	NA	NA	LOC_Os07g01560	transporter family protein, putative, expressed
Chr.7	1566271	1566271	G	A	G	SNP	OS07G0129700	Exonic	CDS	Silent	LOC_Os07g03770	Homeobox domain containing protein, expressed
Chr.7	1591738	1591738	G	A	G	SNP	OS07G0130100	Exonic	CDS	Silent	LOC_Os07g03810	lectin-like receptor kinase 7, putative, expressed
Chr.7	1846148	1846148	G	A	G	SNP	OS07G0134500	Intronic	NA	NA	LOC_Os07g04210	Ser/Thr protein phosphatase family protein, putative, expressed
Chr.7	3604252	3604252	G	A	G	SNP	OS07G0167100	Intronic	NA	NA	LOC_Os07g07260	glutamine-dependent NAD, putative, expressed
Chr.7	3605812	3605812	G	A	G	SNP	OS07G0167100	Intronic	NA	NA	LOC_Os07g07260	glutamine-dependent NAD, putative, expressed
Chr.7	3607482	3607482	G	A	G	SNP	OS07G0167100	Intronic	NA	NA	LOC_Os07g07260	glutamine-dependent NAD, putative, expressed
Chr.7	3608145	3608145	G	A	G	SNP	OS07G0167100	Intronic	NA	NA	LOC_Os07g07260	glutamine-dependent NAD, putative, expressed
Chr.7	3727189	3727189	G	A	G	SNP	OS07G0170300	Exonic	CDS	Silent	LOC_Os07g07490	YT521-B, putative, expressed
Chr.7	3729310	3729310	G	A	G	SNP	OS07G0170300	Exonic	CDS	Silent	LOC_Os07g07490	YT521-B, putative, expressed

Chr.7	3729413	3729413	G	A	G	SNP	OS07G0170300	Exonic	CDS	Missense	LOC_Os07g07490	YT521-B, putative, expressed
Chr.7	3729941	3729941	G	A	G	SNP	OS07G0170300	Intronic	NA	NA	LOC_Os07g07490	YT521-B, putative, expressed
Chr.7	5131685	5131685	G	A	G	SNP	OS07G0194800	Intronic	NA	NA	LOC_Os07g09670	galactosyltransferase family protein, putative, expressed
Chr.7	5599899	5599899	G	A	G	SNP	OS07G0204100	Intronic	NA	NA	LOC_Os07g10420	expressed protein
Chr.7	13367166	13367166	G	A	G	SNP	OS07G0418700	Exonic	CDS	NA	LOC_Os07g23660	retrotransposon protein, putative, unclassified, expressed
Chr.7	18336150	18336150	G	A	G	SNP	OS07G0492000	Intronic	NA	NA	LOC_Os07g30970	nucleoside diphosphate kinase, putative, expressed
Chr.7	18336311	18336311	G	A	G	SNP	OS07G0492000	Intronic	NA	NA	LOC_Os07g30970	nucleoside diphosphate kinase, putative, expressed
Chr.7	19231661	19231661	G	A	G	SNP	OS07G0506700	Exonic	3UTR	NA	LOC_Os07g32350	WD domain, G-beta repeat domain containing protein, expressed
Chr.7	19232384	19232384	G	A	G	SNP	OS07G0506700	Exonic	3UTR	NA	LOC_Os07g32350	WD domain, G-beta repeat domain containing protein, expressed
Chr.7	19233174	19233174	G	A	G	SNP	OS07G0506700	Exonic	3UTR	NA	LOC_Os07g32350	WD domain, G-beta repeat domain containing protein, expressed
Chr.7	19233969	19233969	G	A	G	SNP	OS07G0506700	Exonic	3UTR	NA	LOC_Os07g32350	WD domain, G-beta repeat domain containing protein, expressed
Chr.7	19235220	19235220	G	A	G	SNP	OS07G0506700	Intronic	NA	NA	LOC_Os07g32350	WD domain, G-beta repeat domain containing protein, expressed
Chr.7	20218378	20218378	G	A	G	SNP	OS07G0522500	Exonic	CDS	NA	LOC_Os07g33780	pleiotropic drug resistance protein 5, putative, expressed
Chr.7	21245043	21245043	G	A	G	SNP	OS07G0539400	Exonic	CDS	Missense	LOC_Os07g35520	glucan endo-1,3-beta-glucosidase precursor, putative, expressed
Chr.7	21245434	21245434	G	A	G	SNP	OS07G0539400	Exonic	CDS	Silent	LOC_Os07g35520	glucan endo-1,3-beta-glucosidase precursor, putative, expressed
Chr.7	21245720	21245720	G	A	G	SNP	OS07G0539400	Intronic	NA	NA	LOC_Os07g35520	glucan endo-1,3-beta-glucosidase precursor, putative, expressed
Chr.7	21245765	21245765	G	A	G	SNP	OS07G0539400	Intronic	NA	NA	LOC_Os07g35520	glucan endo-1,3-beta-glucosidase precursor, putative, expressed
Chr.7	21246096	21246096	G	A	G	SNP	OS07G0539400	Exonic	CDS	Silent	LOC_Os07g35520	glucan endo-1,3-beta-glucosidase precursor, putative, expressed
Chr.7	21246129	21246129	G	A	G	SNP	OS07G0539400	Exonic	CDS	Silent	LOC_Os07g35520	glucan endo-1,3-beta-glucosidase precursor, putative, expressed
Chr.7	21246163	21246163	G	A	G	SNP	OS07G0539400	Exonic	CDS	Missense	LOC_Os07g35520	glucan endo-1,3-beta-glucosidase precursor, putative, expressed
Chr.7	21281874	21281874	G	A	G	SNP	OS07G0539900	Exonic	CDS	Silent	LOC_Os07g35560	glucan endo-1,3-beta-glucosidase precursor, putative, expressed
Chr.7	21282416	21282416	G	A	G	SNP	OS07G0539900	Exonic	CDS	Nonsense	LOC_Os07g35560	glucan endo-1,3-beta-glucosidase precursor, putative, expressed
Chr.7	21282581	21282581	G	A	G	SNP	OS07G0539900	Exonic	5UTR	NA	LOC_Os07g35560	glucan endo-1,3-beta-glucosidase precursor, putative, expressed
Chr.7	21379072	21379072	G	A	G	SNP	OS07G0541400	Intronic	NA	NA	LOC_Os07g35690	TKL_IRAK_DUF26-lc.3 - DUF26 kinases have homology to DUF26 containing loci, expressed
Chr.7	21848470	21848470	G	A	G	SNP	OS07G0550500	Intronic	NA	NA	LOC_Os07g36544	serine/threonine-protein kinase receptor precursor, putative, expressed
Chr.7	21849323	21849323	G	A	G	SNP	OS07G0550500	Intronic	NA	NA	LOC_Os07g36544	serine/threonine-protein kinase receptor precursor, putative, expressed
Chr.7	21849709	21849709	G	A	G	SNP	OS07G0550500	Intronic	NA	NA	LOC_Os07g36544	serine/threonine-protein kinase receptor precursor, putative, expressed
Chr.7	21849715	21849715	G	A	G	SNP	OS07G0550500	Intronic	NA	NA	LOC_Os07g36544	serine/threonine-protein kinase receptor precursor, putative, expressed
Chr.7	22429168	22429168	G	A	G	SNP	OS07G0561800	Exonic	3UTR	NA	LOC_Os07g37454	urate anion exchanger, putative, expressed
Chr.7	23281026	23281026	G	A	G	SNP	OS07G0575600	Exonic	5UTR	NA	LOC_Os07g38800	lectin-like receptor kinase, putative, expressed
Chr.7	23283666	23283666	G	A	G	SNP	OS07G0575700	Exonic	CDS	Silent	LOC_Os07g38810	lectin receptor-type protein kinase, putative, expressed
Chr.7	24397360	24397360	G	A	G	SNP	OS07G0598200	Exonic	CDS	Silent	LOC_Os07g40710	circadian clock coupling factor-related, putative, expressed
Chr.7	24399063	24399063	G	A	G	SNP	OS07G0598200	Intronic	NA	NA	LOC_Os07g40710	circadian clock coupling factor-related, putative, expressed
Chr.7	24987820	24987820	G	A	G	SNP	OS07G0607800	Exonic	CDS	Missense	LOC_Os07g41694	acidic leucine-rich nuclear phosphoprotein 32-related protein 1, putative, expressed
Chr.7	24988005	24988005	G	A	G	SNP	OS07G0607800	Exonic	CDS	Silent	LOC_Os07g41694	acidic leucine-rich nuclear phosphoprotein 32-related protein 1, putative, expressed
Chr.7	24990002	24990002	G	A	G	SNP	OS07G0607800	Intronic	NA	NA	LOC_Os07g41694	acidic leucine-rich nuclear phosphoprotein 32-related protein 1, putative, expressed
Chr.7	24990161	24990161	G	A	G	SNP	OS07G0607800	Intronic	NA	NA	LOC_Os07g41694	acidic leucine-rich nuclear phosphoprotein 32-related protein 1, putative, expressed
Chr.7	25037423	25037423	G	A	G	SNP	OS07G0608700	Intronic	NA	NA	LOC_Os07g41790	LSM domain containing protein, expressed
Chr.7	25442120	25442120	G	A	G	SNP	OS07G0617000	Intronic	NA	NA	LOC_Os07g42510	AP2 domain containing protein, expressed
Chr.7	26103440	26103440	G	A	G	SNP	OS07G0629500	Exonic	3UTR	NA	LOC_Os07g43604	expressed protein
Chr.7	26103845	26103845	G	A	G	SNP	OS07G0629500	Exonic	3UTR	NA	LOC_Os07g43604	expressed protein
Chr.7	28670715	28670715	G	A	G	SNP	OS07G0677100	Intronic	NA	NA	LOC_Os07g48010	peroxidase precursor, putative, expressed
Chr.7	28712262	28712262	G	A	G	SNP	OS07G0678300	Exonic	3UTR	NA	LOC_Os07g48090	CAMK_KIN1/SNF1/Nim1_like.30 - CAMK includes calcium/calmodulin depedent protein kinases, expressed
Chr.7	18048	18048	T	A	T	SNP	OS07G0100300	Intronic	NA	NA	LOC_Os07g01030	glycosyl transferase, group 1 domain containing protein, expressed
Chr.7	1568787	1568787	T	A	T	SNP	OS07G0129700	Intronic	NA	NA	LOC_Os07g03770	Homeobox domain containing protein, expressed
Chr.7	3606639	3606639	T	A	T	SNP	OS07G0167100	Intronic	NA	NA	LOC_Os07g07260	glutamine-dependent NAD, putative, expressed
Chr.7	3606902	3606902	T	A	T	SNP	OS07G0167100	Intronic	NA	NA	LOC_Os07g07260	glutamine-dependent NAD, putative, expressed
Chr.7	3729731	3729731	T	A	T	SNP	OS07G0170300	Intronic	NA	NA	LOC_Os07g07490	YT521-B, putative, expressed
Chr.7	3761912	3761912	T	A	T	SNP	OS07G0171100	Exonic	3UTR	NA	LOC_Os07g07540	SHOOT1 protein, putative, expressed
Chr.7	13366680	13366680	T	A	T	SNP	OS07G0418700	Exonic	CDS	NA	LOC_Os07g23660	retrotransposon protein, putative, unclassified, expressed
Chr.7	21848522	21848522	T	A	T	SNP	OS07G0550500	Intronic	NA	NA	LOC_Os07g36544	serine/threonine-protein kinase receptor precursor, putative, expressed
Chr.7	21848738	21848738	T	A	T	SNP	OS07G0550500	Intronic	NA	NA	LOC_Os07g36544	serine/threonine-protein kinase receptor precursor, putative, expressed
Chr.7	22184561	22184561	T	A	T	SNP	OS07G0556200	Intronic	NA	NA	LOC_Os07g37030	cytochrome b6-f complex iron-sulfur subunit, chloroplast precursor, putative, expressed
Chr.7	23186196	23186196	T	A	T	SNP	OS07G0573600	Intronic	NA	NA	LOC_Os07g38600	REX1 DNA Repair family protein, expressed
Chr.7	24966241	24966241	T	A	T	SNP	OS07G0607500	Intronic	NA	NA	LOC_Os07g41660	expressed protein
Chr.7	24985989	24985989	T	A	T	SNP	OS07G0607800	Intronic	NA	NA	LOC_Os07g41694	acidic leucine-rich nuclear phosphoprotein 32-related protein 1, putative, expressed
Chr.7	351880	351880	T	A	T	SNP	OS07G0106200	Intronic	NA	NA	LOC_Os07g01560	transporter family protein, putative, expressed
Chr.7	353400	353400	T	A	T	SNP	OS07G0106200	Intronic	NA	NA	LOC_Os07g01560	transporter family protein, putative, expressed
Chr.7	353455	353455	G	A	G	SNP	OS07G0106200	Exonic	CDS	Silent	LOC_Os07g01560	transporter family protein, putative, expressed
Chr.7	353461	353461	G	A	G	SNP	OS07G0106200	Exonic	CDS	Silent	LOC_Os07g01560	transporter family protein, putative, expressed
Chr.7	353578	353578	G	A	G	SNP	OS07G0106200	Exonic	5UTR	NA	LOC_Os07g01560	transporter family protein, putative, expressed
Chr.7	849210	849210	C	A	C	SNP	OS07G0115500	Intronic	NA	NA	LOC_Os07g02460	expressed protein
Chr.7	927853	927853	C	A	C	SNP	OS07G0116900	Intronic	NA	NA	LOC_Os07g02570	NB-ARC domain containing protein, expressed
Chr.7	1512574	1512574	G	A	G	SNP	OS07G0129200	Exonic	CDS	NA	LOC_Os07g03710	SCP-like extracellular protein, expressed
Chr.7	1512577	1512577	G	A	G	SNP	OS07G0129200	Exonic	CDS	NA	LOC_Os07g03710	SCP-like extracellular protein, expressed
Chr.7	1512622	1512622	T	A	T	SNP	OS07G0129200	Exonic	CDS	NA	LOC_Os07g03710	SCP-like extracellular protein, expressed
Chr.7	1566816	1566816	C	A	C	SNP	OS07G0129700	Intronic	NA	NA	LOC_Os07g03770	Homeobox domain containing protein, expressed
Chr.7	1566855	1566855	C	A	C	SNP	OS07G0129700	Intronic	NA	NA	LOC_Os07g03770	Homeobox domain containing protein, expressed
Chr.7	1566894	1566894	G	A	G	SNP	OS07G0129700	Intronic	NA	NA	LOC_Os07g03770	Homeobox domain containing protein, expressed
Chr.7	2193885	2193885	G	A	G	SNP	OS07G0142900	Exonic	CDS	Missense	LOC_Os07g04990	oxidoreductase, aldo/keto reductase family protein, putative, expressed
Chr.7	3608124	3608124	G	A	G	SNP	OS07G0167100	Intronic	NA	NA	LOC_Os07g07260	glutamine-dependent NAD, putative, expressed
Chr.7	3663283	3663283	G	A	G	SNP	OS07G0168300	Exonic	3UTR	NA	LOC_Os07g07320	glutathione S-transferase, putative, expressed
Chr.7	3760043	3760043	G	A	G	SNP	OS07G0171100	Exonic	5UTR	NA	LOC_Os07g07540	SHOOT1 protein, putative, expressed
Chr.7	5132327	5132327	G	A	G	SNP	OS07G0194800	Exonic	CDS	Silent	LOC_Os07g09670	galactosyltransferase family protein, putative, expressed
Chr.7	6968039	6968039	G	A	G	SNP	OS07G0225300	Exonic	5UTR	NA	LOC_Os07g12340	NAC domain-containing protein 67, putative, expressed
Chr.7	13354300	13354300	G	A	G	SNP	OS07G0418600	Exonic	3UTR	NA	LOC_Os07g23640	retrotransposon protein, putative, Ty3-gypsy subclass, expressed
Chr.7	13354938	13354938	G	A	G	SNP	OS07G0418600	Exonic	CDS	NA	LOC_Os07g23640	retrotransposon protein, putative, Ty3-gypsy subclass, expressed
Chr.7	13366653	13366653	G	A	G	SNP	OS07G0418700	Exonic	CDS	NA	LOC_Os07g23660	retrotransposon protein, putative, unclassified, expressed
Chr.7	15409144	15409144	G	A	G	SNP	OS07G0448800	Exonic	CDS	Silent	LOC_Os07g26690	aquaporin protein, putative, expressed
Chr.7	16503373	16503373	G	A	G	SNP	OS07G0466000	Intronic	NA	NA	LOC_Os07g28250	lipase class 3 family protein, putative, expressed
Chr.7	16898086	16898086	C	A	C	SNP	OS07G0471300	Intronic	NA	NA	LOC_Os07g28850	retrotransposon protein, putative, unclassified, expressed
Chr.7	18761639	18761639	G	A	G	SNP	OS07G0499300	Exonic	CDS	NA	LOC_Os07g31599	expressed protein
Chr.7	18762444	18762444	G	A	G	SNP	OS07G0499300	Exonic	3UTR	NA	LOC_Os07g31599	expressed protein
Chr.7	21246275	21246275	G	A	G	SNP	OS07G0539400	Exonic	CDS	Missense	LOC_Os07g35520	glucan endo-1,3-beta-glucosidase precursor, putative, expressed
Chr.7	21246757	21246757	G	A	G	SNP	OS07G0539400	Exonic	CDS	Missense	LOC_Os07g35520	glucan endo-1,3-beta-glucosidase precursor, putative, expressed

Chr.7	21589835	21589835	C	A	C	SNP	OS07G0545300	Exonic	5UTR	NA	LOC_Os07g36130	core histone H2A/H2B/H3/H4, putative, expressed
Chr.7	21848752	21848752	G	A	G	SNP	OS07G0550500	Intronic	NA	NA	LOC_Os07g36544	serine/threonine-protein kinase receptor precursor, putative, expressed
Chr.7	21848830	21848830	T	A	T	SNP	OS07G0550500	Intronic	NA	NA	LOC_Os07g36544	serine/threonine-protein kinase receptor precursor, putative, expressed
Chr.7	21849150	21849150	T	A	T	SNP	OS07G0550500	Intronic	NA	NA	LOC_Os07g36544	serine/threonine-protein kinase receptor precursor, putative, expressed
Chr.7	21849306	21849306	G	A	G	SNP	OS07G0550500	Intronic	NA	NA	LOC_Os07g36544	serine/threonine-protein kinase receptor precursor, putative, expressed
Chr.7	21850629	21850629	C	A	C	SNP	OS07G0550500	Exonic	CDS	Silent	LOC_Os07g36544	serine/threonine-protein kinase receptor precursor, putative, expressed
Chr.7	21850654	21850654	G	A	G	SNP	OS07G0550500	Exonic	CDS	Missense	LOC_Os07g36544	serine/threonine-protein kinase receptor precursor, putative, expressed
Chr.7	21850788	21850788	G	A	G	SNP	OS07G0550500	Exonic	CDS	Silent	LOC_Os07g36544	serine/threonine-protein kinase receptor precursor, putative, expressed
Chr.7	22354786	22354786	C	A	C	SNP	OS07G0559700	Intronic	NA	NA	LOC_Os07g37320	transporter family protein, putative, expressed
Chr.7	22428027	22428027	G	A	G	SNP	OS07G0561800	Exonic	CDS	NA	LOC_Os07g37454	urate anion exchanger, putative, expressed
Chr.7	22428785	22428785	G	A	G	SNP	OS07G0561800	Exonic	3UTR	NA	LOC_Os07g37454	urate anion exchanger, putative, expressed
Chr.7	22874801	22874801	G	A	G	SNP	OS07G0568700	Exonic	CDS	Silent	LOC_Os07g38130	polygalacturonase inhibitor 1 precursor, putative, expressed
Chr.7	23283025	23283025	T	A	T	SNP	OS07G0575700	Exonic	CDS	Missense	LOC_Os07g38810	lectin receptor-type protein kinase, putative, expressed
Chr.7	24398908	24398908	G	A	G	SNP	OS07G0598200	Intronic	NA	NA	LOC_Os07g40710	circadian clock coupling factor-related, putative, expressed
Chr.7	24399210	24399210	C	A	C	SNP	OS07G0598200	Intronic	NA	NA	LOC_Os07g40710	circadian clock coupling factor-related, putative, expressed
Chr.7	24967958	24967958	T	A	T	SNP	OS07G0607500	Intronic	NA	NA	LOC_Os07g41660	expressed protein
Chr.7	24986243	24986243	T	A	T	SNP	OS07G0607800	Intronic	NA	NA	LOC_Os07g41694	acidic leucine-rich nuclear phosphoprotein 32-related protein 1, putative, expressed
Chr.7	24989048	24989048	G	A	G	SNP	OS07G0607800	Intronic	NA	NA	LOC_Os07g41694	acidic leucine-rich nuclear phosphoprotein 32-related protein 1, putative, expressed
Chr.7	24989699	24989699	G	A	G	SNP	OS07G0607800	Intronic	NA	NA	LOC_Os07g41694	acidic leucine-rich nuclear phosphoprotein 32-related protein 1, putative, expressed
Chr.7	24989722	24989722	G	A	G	SNP	OS07G0607800	Intronic	NA	NA	LOC_Os07g41694	acidic leucine-rich nuclear phosphoprotein 32-related protein 1, putative, expressed
Chr.7	25273654	25273654	G	A	G	SNP	OS07G0613800	Exonic	CDS	NA	LOC_Os07g42220	expressed protein
Chr.7	25273658	25273658	G	A	G	SNP	OS07G0613800	Exonic	CDS	NA	LOC_Os07g42220	expressed protein
Chr.7	25441440	25441440	G	A	G	SNP	OS07G0617000	Exonic	CDS	Silent	LOC_Os07g42510	AP2 domain containing protein, expressed
Chr.7	26383882	26383882	G	A	G	SNP	OS07G0635500	Exonic	CDS	Silent	LOC_Os07g44140	cytochrome P450 72A1, putative, expressed
Chr.7	26408752	26408752	G	A	G	SNP	OS07G0636000	Exonic	CDS	Missense	LOC_Os07g44190	h/ACA ribonucleoprotein complex subunit 4, putative, expressed
Chr.7	28274461	28274461	G	A	G	SNP	OS07G0669100	Exonic	CDS	Silent	LOC_Os07g47290	xylose isomerase, putative, expressed
Chr.7	28274693	28274693	G	A	G	SNP	OS07G0669100	Exonic	CDS	Silent	LOC_Os07g47290	xylose isomerase, putative, expressed
Chr.7	28711052	28711052	G	A	G	SNP	OS07G0678300	Exonic	CDS	Silent	LOC_Os07g48090	CAMK_KIN1/SNF1/Nim1_like.30 - CAMK includes calcium/calmodulin depedent protein kinases, expressed
Chr.7	29339443	29339443	G	A	G	SNP	OS07G0689800	Exonic	CDS	Missense	LOC_Os07g49000	DNAJ heat shock N-terminal domain-containing protein, putative, expressed
Chr1	30372524	30372524	G	A	G	SNP	OS01G0728300	Exonic	3UTR	NA	LOC_Os01g52790	cytochrome P450 72A1, putative, expressed
Chr1	3810676	3810676	G	A	G	SNP	OS01G0173900	Exonic	3UTR	NA	LOC_Os01g07870	ABC transporter family protein, putative, expressed
Chr1	1202093	1202093	C	A	C	SNP	OS01G0121300	Exonic	CDS	Missense	LOC_Os01g03110	expressed protein
Chr1	3809316	3809316	T	A	T	SNP	OS01G0173900	Exonic	CDS	Silent	LOC_Os01g07870	ABC transporter family protein, putative, expressed
Chr1	21431893	21431893	C	A	C	SNP	OS01G0563000	Exonic	CDS	Silent	LOC_Os01g38229	peptidyl-prolyl isomerase, putative, expressed
Chr1	8421452	8421452	G	A	G	SNP	OS01G0254100	Exonic	3UTR	NA	LOC_Os01g15020	lissencephaly type-1-like homology motif, putative, expressed
Chr1	31101162	31101162	G	A	G	SNP	OS01G0744000	Exonic	CDS	Silent	LOC_Os01g54080	kinesin motor protein-related, putative, expressed
Chr1	262647	262647	T	A	T	SNP	OS01G0104800	Exonic	CDS	Silent	LOC_Os01g01510	sas10/Utp3 family protein, expressed
Chr1	265304	265304	T	A	T	SNP	OS01G0104800	Exonic	CDS	Silent	LOC_Os01g01510	sas10/Utp3 family protein, expressed
Chr1	14515246	14515246	T	A	T	SNP	OS01G0358400	Exonic	3UTR	NA	LOC_Os01g25610	40S ribosomal protein S4, putative, expressed
Chr1	25237916	25237916	T	A	T	SNP	OS01G0631200	Exonic	3UTR	NA	LOC_Os01g44050	siroheme synthase, putative, expressed
Chr1	26801192	26801192	T	A	T	SNP	OS01G0658900	Intronic	NA	NA	LOC_Os01g46970	transcription factor, putative, expressed
Chr1	27082870	27082870	T	A	T	SNP	OS01G0663300	Intronic	NA	NA	LOC_Os01g47400	OsMan01 - Endo-Beta-Mannanase, expressed
Chr1	27083016	27083016	T	A	T	SNP	OS01G0663300	Intronic	NA	NA	LOC_Os01g47400	OsMan01 - Endo-Beta-Mannanase, expressed
Chr1	27084225	27084225	T	A	T	SNP	OS01G0663300	Intronic	NA	NA	LOC_Os01g47400	OsMan01 - Endo-Beta-Mannanase, expressed
Chr1	27179563	27179563	T	A	T	SNP	OS01G0665300	Exonic	CDS	Missense	LOC_Os01g47540	NLI interacting factor-like phosphatase, putative, expressed
Chr1	27179923	27179923	T	A	T	SNP	OS01G0665300	Exonic	CDS	Missense	LOC_Os01g47540	NLI interacting factor-like phosphatase, putative, expressed
Chr1	29843922	29843922	T	A	T	SNP	OS01G0717000	Intronic	NA	NA	LOC_Os01g51920	phosphotransferase, putative, expressed
Chr1	29970216	29970216	T	A	T	SNP	OS01G0719100	Exonic	5UTR	NA	LOC_Os01g52110	RING finger and CHY zinc finger domain-containing protein 1, putative, expressed
Chr1	30183925	30183925	T	A	T	SNP	OS01G0723600	Intronic	NA	NA	LOC_Os01g52530	phosphoribosyl transferase, putative, expressed
Chr1	31583298	31583298	T	A	T	SNP	OS01G0752700	Exonic	3UTR	NA	LOC_Os01g54910	GTP-binding protein typA/bipA, putative, expressed
Chr1	31730169	31730169	T	A	T	SNP	OS01G0756200	Exonic	3UTR	NA	LOC_Os01g55150	bZIP transcription factor domain containing protein, expressed
Chr1	34145050	34145050	T	A	T	SNP	OS01G0805400	Exonic	3UTR	NA	LOC_Os01g59100	cytokinin-N-glucosyltransferase 1, putative, expressed
Chr1	39225855	39225855	T	A	T	SNP	OS01G0901000	Intronic	NA	NA	LOC_Os01g67500	armadillo/beta-catenin repeat family protein, putative, expressed
Chr1	42741318	42741318	T	A	T	SNP	OS01G0969100	Intronic	NA	NA	LOC_Os01g73790	NAD dependent epimerase/dehydratase family protein, putative, expressed
Chr1	1172803	1172803	T	A	T	SNP	OS01G0120800	Intronic	NA	NA	LOC_Os01g03070	transposon protein, putative, unclassified, expressed
Chr1	34450459	34450459	T	A	T	SNP	OS01G0810800	Exonic	CDS	Missense	LOC_Os01g59570	senescence-induced receptor-like serine/threonine-protein kinase precursor, putative, expressed
Chr1	37557342	37557342	T	A	T	SNP	OS01G0867300	Intronic	NA	NA	LOC_Os01g64730	bZIP transcription factor domain containing protein, expressed
Chr1	26802133	26802133	G	A	G	SNP	OS01G0658900	Exonic	CDS	Missense	LOC_Os01g46970	transcription factor, putative, expressed
Chr1	267628	267628	G	A	G	SNP	OS01G0104800	Exonic	CDS	Missense	LOC_Os01g01510	sas10/Utp3 family protein, expressed
Chr1	268085	268085	G	A	G	SNP	OS01G0104800	Exonic	3UTR	NA	LOC_Os01g01510	sas10/Utp3 family protein, expressed
Chr1	743370	743370	G	A	G	SNP	OS01G0113700	Exonic	CDS	Silent	LOC_Os01g02350	receptor-like kinase ARK1AS, putative, expressed
Chr1	744989	744989	G	A	G	SNP	OS01G0113700	Exonic	CDS	Silent	LOC_Os01g02350	receptor-like kinase ARK1AS, putative, expressed
Chr1	3578271	3578271	G	A	G	SNP	OS01G0169800	Intronic	NA	NA	LOC_Os01g07500	alliin lyase precursor, putative, expressed
Chr1	3820900	3820900	G	A	G	SNP	OS01G0174300	Exonic	CDS	Silent	LOC_Os01g07910	NADH-cytochrome b5 reductase, putative, expressed
Chr1	8546171	8546171	G	A	G	SNP	OS01G0256500	Exonic	3UTR	NA	LOC_Os01g15270	expressed protein
Chr1	9235979	9235979	G	A	G	SNP	OS01G0268300	Intronic	NA	NA	LOC_Os01g16290	DNA gyrase subunit B, chloroplast/mitochondrial precursor, putative, expressed
Chr1	10011062	10011062	G	A	G	SNP	OS01G0281100	Intronic	NA	NA	LOC_Os01g17396	expressed protein
Chr1	10102226	10102226	G	A	G	SNP	OS01G0282800	Exonic	CDS	Silent	LOC_Os01g18050	tubulin/FtsZ domain containing protein, putative, expressed
Chr1	10102890	10102890	G	A	G	SNP	OS01G0282800	Exonic	5UTR	NA	LOC_Os01g18050	tubulin/FtsZ domain containing protein, putative, expressed
Chr1	10342596	10342596	G	A	G	SNP	OS01G0287600	Intronic	NA	NA	LOC_Os01g18400	CHIT10 - Chitinase family protein precursor, expressed
Chr1	10342903	10342903	G	A	G	SNP	OS01G0287600	Intronic	NA	NA	LOC_Os01g18400	CHIT10 - Chitinase family protein precursor, expressed
Chr1	10494266	10494266	G	A	G	SNP	OS01G0290000	Intronic	NA	NA	LOC_Os01g18630	aspartic proteinase oryzasin-1 precursor, putative, expressed
Chr1	22495716	22495716	G	A	G	SNP	OS01G0580800	Intronic	NA	NA	LOC_Os01g39890	transposon protein, putative, unclassified, expressed
Chr1	22496618	22496618	G	A	G	SNP	OS01G0580800	Intronic	NA	NA	LOC_Os01g39890	transposon protein, putative, unclassified, expressed
Chr1	22496680	22496680	G	A	G	SNP	OS01G0580800	Intronic	NA	NA	LOC_Os01g39890	transposon protein, putative, unclassified, expressed
Chr1	22496722	22496722	G	A	G	SNP	OS01G0580800	Intronic	NA	NA	LOC_Os01g39890	transposon protein, putative, unclassified, expressed
Chr1	22497450	22497450	G	A	G	SNP	OS01G0580800	Intronic	NA	NA	LOC_Os01g39890	transposon protein, putative, unclassified, expressed
Chr1	26890435	26890435	G	A	G	SNP	OS01G0660300	Intronic	NA	NA	LOC_Os01g47080	pyruvate kinase, putative, expressed
Chr1	27084176	27084176	G	A	G	SNP	OS01G0663300	Intronic	NA	NA	LOC_Os01g47400	OsMan01 - Endo-Beta-Mannanase, expressed
Chr1	27084260	27084260	G	A	G	SNP	OS01G0663300	Intronic	NA	NA	LOC_Os01g47400	OsMan01 - Endo-Beta-Mannanase, expressed
Chr1	27103339	27103339	G	A	G	SNP	OS01G0663800	Exonic	CDS	Missense	LOC_Os01g47430	protein of unknown function DUF1296 domain containing protein, expressed
Chr1	27179239	27179239	G	A	G	SNP	OS01G0665300	Exonic	3UTR	NA	LOC_Os01g47540	NLI interacting factor-like phosphatase, putative, expressed
Chr1	29484037	29484037	G	A	G	SNP	OS01G0709500	Exonic	CDS	Silent	LOC_Os01g51290	protein kinase family protein, putative, expressed
Chr1	29487413	29487413	G	A	G	SNP	OS01G0709500	Exonic	CDS	Silent	LOC_Os01g51290	protein kinase family protein, putative, expressed
Chr1	29489870	29489870	G	A	G	SNP	OS01G0709500	Intronic	NA	NA	LOC_Os01g51290	protein kinase family protein, putative, expressed

Chr1	29845199	29845199	G	A	G	SNP	OS01G0717000	Exonic	3UTR	NA	LOC_Os01g51920	phosphotransferase, putative, expressed
Chr1	29894794	29894794	G	A	G	SNP	OS01G0717700	Intronic	NA	NA	LOC_Os01g52010	alliin lyase precursor, putative, expressed
Chr1	29896157	29896157	G	A	G	SNP	OS01G0717700	Intronic	NA	NA	LOC_Os01g52010	alliin lyase precursor, putative, expressed
Chr1	29896526	29896526	G	A	G	SNP	OS01G0717700	Intronic	NA	NA	LOC_Os01g52010	alliin lyase precursor, putative, expressed
Chr1	29962142	29962142	G	A	G	SNP	OS01G0719000	Exonic	CDS	Missense	LOC_Os01g52100	DUF581 domain containing protein, expressed
Chr1	30259882	30259882	G	A	G	SNP	OS01G0725800	Intronic	NA	NA	LOC_Os01g52640	suppressor of phythchrome A, putative, expressed
Chr1	31982071	31982071	G	A	G	SNP	OS01G0760000	Exonic	CDS	Silent	LOC_Os01g55510	dynein light chain type 1 domain containing protein, expressed
Chr1	32002401	32002401	G	A	G	SNP	OS01G0760600	Intronic	NA	NA	LOC_Os01g55540	aminotransferase, classes I and II, domain containing protein, expressed
Chr1	32148663	32148663	G	A	G	SNP	OS01G0764000	Exonic	CDS	Silent	LOC_Os01g55830	glutathione S-transferase, putative, expressed
Chr1	34143953	34143953	G	A	G	SNP	OS01G0805400	Intronic	NA	NA	LOC_Os01g59100	cytokinin-N-glucosyltransferase 1, putative, expressed
Chr1	35154763	35154763	G	A	G	SNP	OS01G0823300	Intronic	NA	NA	LOC_Os01g60790	40S ribosomal protein S26, putative, expressed
Chr1	41583701	41583701	G	A	G	SNP	OS01G0946100	Exonic	3UTR	NA	LOC_Os01g71780	WD domain, G-beta repeat domain containing protein, expressed
Chr1	42066086	42066086	G	A	G	SNP	OS01G0955000	Intronic	NA	NA	LOC_Os01g72520	phosphoesterase family protein, putative, expressed
Chr1	42837714	42837714	G	A	G	SNP	OS01G0970900	Exonic	3UTR	NA	LOC_Os01g73950	PPR repeat containing protein, expressed
Chr1	42838910	42838910	G	A	G	SNP	OS01G0970900	Intronic	NA	NA	LOC_Os01g73950	PPR repeat containing protein, expressed
Chr1	42840288	42840288	G	A	G	SNP	OS01G0970900	Intronic	NA	NA	LOC_Os01g73950	PPR repeat containing protein, expressed
Chr1	42840301	42840301	G	A	G	SNP	OS01G0970900	Intronic	NA	NA	LOC_Os01g73950	PPR repeat containing protein, expressed
Chr1	42849161	42849161	G	A	G	SNP	OS01G0971200	Intronic	NA	NA	LOC_Os01g73970	lysine ketoglutarate reductase trans-splicing related 1, putative, expressed
Chr1	42850046	42850046	G	A	G	SNP	OS01G0971200	Exonic	CDS	Silent	LOC_Os01g73970	lysine ketoglutarate reductase trans-splicing related 1, putative, expressed
Chr1	43073745	43073745	G	A	G	SNP	OS01G0974800	Intronic	NA	NA	LOC_Os01g74350	soluble inorganic pyrophosphatase, putative, expressed
Chr1	3822650	3822650	G	A	G	SNP	OS01G0174300	Intronic	NA	NA	LOC_Os01g07910	NADH-cytochrome b5 reductase, putative, expressed
Chr1	3823073	3823073	G	A	G	SNP	OS01G0174300	Intronic	NA	NA	LOC_Os01g07910	NADH-cytochrome b5 reductase, putative, expressed
Chr1	5901966	5901966	G	A	G	SNP	OS01G0208700	Intronic	NA	NA	LOC_Os01g11054	phosphoenolpyruvate carboxylase, putative, expressed
Chr1	31454930	31454930	G	A	G	SNP	OS01G0750500	Intronic	NA	NA	LOC_Os01g54670	coiled-coil domain-containing protein 25, putative, expressed
Chr1	34445869	34445869	G	A	G	SNP	OS01G0810800	Exonic	3UTR	NA	LOC_Os01g59570	senescence-induced receptor-like serine/threonine-protein kinase precursor, putative, expressed
Chr1	34446226	34446226	G	A	G	SNP	OS01G0810800	Exonic	CDS	Silent	LOC_Os01g59570	senescence-induced receptor-like serine/threonine-protein kinase precursor, putative, expressed
Chr1	34446268	34446268	G	A	G	SNP	OS01G0810800	Exonic	CDS	Silent	LOC_Os01g59570	senescence-induced receptor-like serine/threonine-protein kinase precursor, putative, expressed
Chr1	34446301	34446301	G	A	G	SNP	OS01G0810800	Exonic	CDS	Silent	LOC_Os01g59570	senescence-induced receptor-like serine/threonine-protein kinase precursor, putative, expressed
Chr1	34446304	34446304	G	A	G	SNP	OS01G0810800	Exonic	CDS	Silent	LOC_Os01g59570	senescence-induced receptor-like serine/threonine-protein kinase precursor, putative, expressed
Chr1	34446433	34446433	G	A	G	SNP	OS01G0810800	Exonic	CDS	Silent	LOC_Os01g59570	senescence-induced receptor-like serine/threonine-protein kinase precursor, putative, expressed
Chr1	34446700	34446700	G	A	G	SNP	OS01G0810800	Exonic	CDS	Silent	LOC_Os01g59570	senescence-induced receptor-like serine/threonine-protein kinase precursor, putative, expressed
Chr1	8429407	8429407	C	A	C	SNP	OS01G0254100	Intronic	NA	NA	LOC_Os01g15020	lissencephaly type-1-like homology motif, putative, expressed
Chr1	9230261	9230261	C	A	C	SNP	OS01G0268300	Intronic	NA	NA	LOC_Os01g16290	DNA gyrase subunit B, chloroplast/mitochondrial precursor, putative, expressed
Chr1	10200588	10200588	C	A	C	SNP	OS01G0284900	Exonic	3UTR	NA	LOC_Os01g18220	expressed protein
Chr1	22498605	22498605	C	A	C	SNP	OS01G0580800	Intronic	NA	NA	LOC_Os01g39890	transposon protein, putative, unclassified, expressed
Chr1	29461711	29461711	C	A	C	SNP	OS01G0708900	Exonic	CDS	Silent	LOC_Os01g51250	mitochondrial carrier protein, putative, expressed
Chr1	29484560	29484560	C	A	C	SNP	OS01G0709500	Exonic	CDS	Missense	LOC_Os01g51290	protein kinase family protein, putative, expressed
Chr1	29484641	29484641	C	A	C	SNP	OS01G0709500	Intronic	NA	NA	LOC_Os01g51290	protein kinase family protein, putative, expressed
Chr1	29963023	29963023	C	A	C	SNP	OS01G0719000	Exonic	3UTR	NA	LOC_Os01g52100	DUF581 domain containing protein, expressed
Chr1	30183926	30183926	C	A	C	SNP	OS01G0723600	Intronic	NA	NA	LOC_Os01g52530	phosphoribosyl transferase, putative, expressed
Chr1	31580956	31580956	C	A	C	SNP	OS01G0752700	Intronic	NA	NA	LOC_Os01g54910	GTP-binding protein typA/bipA, putative, expressed
Chr1	32148672	32148672	C	A	C	SNP	OS01G0764000	Exonic	CDS	Silent	LOC_Os01g55830	glutathione S-transferase, putative, expressed
Chr1	34143882	34143882	C	A	C	SNP	OS01G0805400	Intronic	NA	NA	LOC_Os01g59100	cytokinin-N-glucosyltransferase 1, putative, expressed
Chr1	42065781	42065781	C	A	C	SNP	OS01G0955000	Intronic	NA	NA	LOC_Os01g72520	phosphoesterase family protein, putative, expressed
Chr1	42741014	42741014	C	A	C	SNP	OS01G0969100	Intronic	NA	NA	LOC_Os01g73790	NAD dependent epimerase/dehydratase family protein, putative, expressed
Chr1	42839979	42839979	C	A	C	SNP	OS01G0970900	Intronic	NA	NA	LOC_Os01g73950	PPR repeat containing protein, expressed
Chr1	42979422	42979422	C	A	C	SNP	OS01G0973400	Intronic	NA	NA	LOC_Os01g74190	radical SAM enzyme, putative, expressed
Chr1	5905712	5905712	C	A	C	SNP	OS01G0208700	Intronic	NA	NA	LOC_Os01g11054	phosphoenolpyruvate carboxylase, putative, expressed
Chr1	11636403	11636403	C	A	C	SNP	OS01G0310100	Intronic	NA	NA	LOC_Os01g20860	phospholipase D. Active site motif family protein, expressed
Chr1	33840923	33840923	C	A	C	SNP	OS01G0799500	Exonic	CDS	Missense	LOC_Os01g58550	methyladenine glycosylase, putative, expressed
Chr1	31455063	31455064	AC	A	AC	INDEL	OS01G0750500	Intronic	NA	NA	LOC_Os01g54670	coiled-coil domain-containing protein 25, putative, expressed
Chr1	1173899	1173900	AT	A	AT	INDEL	OS01G0120800	Intronic	NA	NA	LOC_Os01g03070	transposon protein, putative, unclassified, expressed
Chr1	3808996	3808997	AT	A	AT	INDEL	OS01G0173900	Intronic	NA	NA	LOC_Os01g07870	ABC transporter family protein, putative, expressed
Chr1	3821773	3821777	ATTTT	A	ATTTT	INDEL	OS01G0174300	Intronic	NA	NA	LOC_Os01g07910	NADH-cytochrome b5 reductase, putative, expressed
Chr1	3823705	3823706	AG	A	AG	INDEL	OS01G0174300	Intronic	NA	NA	LOC_Os01g07910	NADH-cytochrome b5 reductase, putative, expressed
Chr1	7029472	7029473	AC	A	AC	INDEL	OS01G0227100	Intronic	NA	NA	LOC_Os01g12710	oxidoreductase, short chain dehydrogenase/reductase family domain containing protein, expressed
Chr1	8206135	8206136	AC	A	AC	INDEL	OS01G0249200	Intronic	NA	NA	LOC_Os01g14670	Cupin domain containing protein, expressed
Chr1	8429675	8429676	AT	A	AT	INDEL	OS01G0254100	Intronic	NA	NA	LOC_Os01g15020	lissencephaly type-1-like homology motif, putative, expressed
Chr1	9674132	9674136	AAAGG	A	AAAGG	INDEL	OS01G0276200	Intronic	NA	NA	LOC_Os01g16910	outer mitochondrial membrane porin, putative, expressed
Chr1	29961535	29961536	AT	A	AT	INDEL	OS01G0719000	Intronic	NA	NA	LOC_Os01g52100	DUF581 domain containing protein, expressed
Chr1	32715982	32715983	AT	A	AT	INDEL	OS01G0773800	Intronic	NA	NA	LOC_Os01g56690	helix-loop-helix DNA-binding domain containing protein, expressed
Chr1	33410662	33410664	AAT	A	AAT	INDEL	OS01G0787600	Intronic	NA	NA	LOC_Os01g57770	OsPOP4 - Putative Prolyl Oligopeptidase homologue, expressed
Chr1	33414931	33414932	AT	A	AT	INDEL	OS01G0787600	Exonic	5UTR	NA	LOC_Os01g57770	OsPOP4 - Putative Prolyl Oligopeptidase homologue, expressed
Chr1	33840927	33840932	ATTTTT	A	ATTTTT	INDEL	OS01G0799500	Exonic	CDS	FrameShift	LOC_Os01g58550	methyladenine glycosylase, putative, expressed
Chr1	33898925	33898926	AT	A	AT	INDEL	OS01G0800500	Intronic	NA	NA	LOC_Os01g58640	nucleotide pyrophosphatase/phosphodiesterase, putative, expressed
Chr1	34151043	34151044	AT	A	AT	INDEL	OS01G0805600	Intronic	NA	NA	LOC_Os01g59120	cyclin, putative, expressed
Chr1	34447582	34447583	AG	A	AG	INDEL	OS01G0810800	Intronic	NA	NA	LOC_Os01g59570	senescence-induced receptor-like serine/threonine-protein kinase precursor, putative, expressed
Chr1	34450453	34450456	ATCT	A	ATCT	INDEL	OS01G0810800	Exonic	CDS	InFrame	LOC_Os01g59570	senescence-induced receptor-like serine/threonine-protein kinase precursor, putative, expressed
Chr1	36296665	36296666	AC	A	AC	INDEL	OS01G0845100	Exonic	3UTR	NA	LOC_Os01g62670	avr9/Cf-9 rapidly elicited protein, putative, expressed
Chr1	36396370	36396376	ATCACAC	A	ATCACAC	INDEL	OS01G0847200	Intronic	NA	NA	LOC_Os01g62840	mannose-1-phosphate guanyltransferase, putative, expressed
Chr1	37557336	37557337	AT	A	AT	INDEL	OS01G0867300	Intronic	NA	NA	LOC_Os01g64730	bZIP transcription factor domain containing protein, expressed
Chr1	37698726	37698727	AT	A	AT	INDEL	OS01G0869800	Intronic	NA	NA	LOC_Os01g64960	chlorophyll A-B binding protein, putative, expressed
Chr1	39922501	39922502	AT	A	AT	INDEL	OS01G0916000	Exonic	3UTR	NA	LOC_Os01g68730	RNA-binding protein FUS, putative, expressed
Chr1	41005269	41005271	AAG	A	AAG	INDEL	OS01G0934800	Intronic	NA	NA	LOC_Os01g70850	esterase, putative, expressed
Chr1	43076269	43076279	AGCCACCGCGC	A	AGCCACCGCGC	INDEL	OS01G0974800	Intronic	NA	NA	LOC_Os01g74350	soluble inorganic pyrophosphatase, putative, expressed
Chr1	43142584	43142587	ACCC	A	ACCC	INDEL	OS01G0976300	Exonic	3UTR	NA	LOC_Os01g74490	heavy metal-associated domain containing protein, expressed
Chr1	1168750	1168750	G	A	G	SNP	OS01G0120700	Exonic	3UTR	NA	LOC_Os01g03060	splicing factor, putative, expressed
Chr1	1169778	1169778	C	A	C	SNP	OS01G0120700	Exonic	5UTR	NA	LOC_Os01g03060	splicing factor, putative, expressed
Chr1	1172040	1172040	G	A	G	SNP	OS01G0120800	Exonic	3UTR	NA	LOC_Os01g03070	transposon protein, putative, unclassified, expressed
Chr1	1172390	1172390	C	A	C	SNP	OS01G0120800	Exonic	CDS	Missense	LOC_Os01g03070	transposon protein, putative, unclassified, expressed
Chr1	1172749	1172749	T	A	T	SNP	OS01G0120800	Intronic	NA	NA	LOC_Os01g03070	transposon protein, putative, unclassified, expressed
Chr1	1172952	1172952	G	A	G	SNP	OS01G0120800	Intronic	NA	NA	LOC_Os01g03070	transposon protein, putative, unclassified, expressed
Chr1	1173014	1173014	T	A	T	SNP	OS01G0120800	Intronic	NA	NA	LOC_Os01g03070	transposon protein, putative, unclassified, expressed
Chr1	1173449	1173449	G	A	G	SNP	OS01G0120800	Intronic	NA	NA	LOC_Os01g03070	transposon protein, putative, unclassified, expressed
Chr1	1173919	1173919	T	A	T	SNP	OS01G0120800	Intronic	NA	NA	LOC_Os01g03070	transposon protein, putative, unclassified, expressed

Chr1	1173938	1173938	G	A	G	SNP	OS01G0120800	Intronic	NA	NA	LOC_Os01g03070	transposon protein, putative, unclassified, expressed
Chr1	1174237	1174237	T	A	T	SNP	OS01G0120800	Intronic	NA	NA	LOC_Os01g03070	transposon protein, putative, unclassified, expressed
Chr1	1174355	1174355	G	A	G	SNP	OS01G0120800	Intronic	NA	NA	LOC_Os01g03070	transposon protein, putative, unclassified, expressed
Chr1	1176621	1176621	G	A	G	SNP	OS01G0120800	Intronic	NA	NA	LOC_Os01g03070	transposon protein, putative, unclassified, expressed
Chr1	1176683	1176683	T	A	T	SNP	OS01G0120800	Intronic	NA	NA	LOC_Os01g03070	transposon protein, putative, unclassified, expressed
Chr1	1177744	1177744	G	A	G	SNP	OS01G0120800	Intronic	NA	NA	LOC_Os01g03070	transposon protein, putative, unclassified, expressed
Chr1	1177858	1177858	G	A	G	SNP	OS01G0120800	Intronic	NA	NA	LOC_Os01g03070	transposon protein, putative, unclassified, expressed
Chr1	1178063	1178063	G	A	G	SNP	OS01G0120800	Intronic	NA	NA	LOC_Os01g03070	transposon protein, putative, unclassified, expressed
Chr1	1178328	1178328	G	A	G	SNP	OS01G0120800	Exonic	CDS	Silent	LOC_Os01g03070	transposon protein, putative, unclassified, expressed
Chr1	1217981	1217981	G	A	G	SNP	OS01G0121500	Exonic	CDS	NA	LOC_Os01g03130	expressed protein
Chr1	1223831	1223831	G	A	G	SNP	OS01G0121600	Exonic	5UTR	NA	LOC_Os01g03144	white-brown complex homolog protein 7, putative, expressed
Chr1	1566845	1566845	T	A	T	SNP	OS01G0128200	Intronic	NA	NA	LOC_Os01g03730	nuclease PA3, putative, expressed
Chr1	1566985	1566985	G	A	G	SNP	OS01G0128200	Intronic	NA	NA	LOC_Os01g03730	nuclease PA3, putative, expressed
Chr1	1567137	1567137	G	A	G	SNP	OS01G0128200	Intronic	NA	NA	LOC_Os01g03730	nuclease PA3, putative, expressed
Chr1	1567151	1567151	G	A	G	SNP	OS01G0128200	Intronic	NA	NA	LOC_Os01g03730	nuclease PA3, putative, expressed
Chr1	1567175	1567175	C	A	C	SNP	OS01G0128200	Intronic	NA	NA	LOC_Os01g03730	nuclease PA3, putative, expressed
Chr1	1567176	1567176	T	A	T	SNP	OS01G0128200	Intronic	NA	NA	LOC_Os01g03730	nuclease PA3, putative, expressed
Chr1	2277054	2277054	G	A	G	SNP	OS01G0142300	Intronic	NA	NA	LOC_Os01g04920	glycosyl transferase, group 1 domain containing protein, expressed
Chr1	2278024	2278024	G	A	G	SNP	OS01G0142300	Intronic	NA	NA	LOC_Os01g04920	glycosyl transferase, group 1 domain containing protein, expressed
Chr1	2383612	2383612	G	A	G	SNP	OS01G0144100	Exonic	3UTR	NA	LOC_Os01g05080	thylakoid lumenal protein, putative, expressed
Chr1	2384892	2384892	C	A	C	SNP	OS01G0144100	Intronic	NA	NA	LOC_Os01g05080	thylakoid lumenal protein, putative, expressed
Chr1	2385489	2385489	C	A	C	SNP	OS01G0144100	Exonic	CDS	Silent	LOC_Os01g05080	thylakoid lumenal protein, putative, expressed
Chr1	3622625	3622625	G	A	G	SNP	OS01G0170300	Exonic	CDS	Missense	LOC_Os01g07560	receptor-like protein kinase 2 precursor, putative, expressed
Chr1	3624481	3624481	G	A	G	SNP	OS01G0170300	Intronic	NA	NA	LOC_Os01g07560	receptor-like protein kinase 2 precursor, putative, expressed
Chr1	3665917	3665917	G	A	G	SNP	OS01G0170900	Exonic	5UTR	NA	LOC_Os01g07620	expressed protein
Chr1	3805990	3805990	C	A	C	SNP	OS01G0173900	Exonic	CDS	Silent	LOC_Os01g07870	ABC transporter family protein, putative, expressed
Chr1	3806260	3806260	G	A	G	SNP	OS01G0173900	Exonic	CDS	Silent	LOC_Os01g07870	ABC transporter family protein, putative, expressed
Chr1	3808347	3808347	G	A	G	SNP	OS01G0173900	Intronic	NA	NA	LOC_Os01g07870	ABC transporter family protein, putative, expressed
Chr1	3808672	3808672	T	A	T	SNP	OS01G0173900	Intronic	NA	NA	LOC_Os01g07870	ABC transporter family protein, putative, expressed
Chr1	3809256	3809256	C	A	C	SNP	OS01G0173900	Exonic	CDS	Silent	LOC_Os01g07870	ABC transporter family protein, putative, expressed
Chr1	3809926	3809926	C	A	C	SNP	OS01G0173900	Exonic	CDS	Silent	LOC_Os01g07870	ABC transporter family protein, putative, expressed
Chr1	3810034	3810034	G	A	G	SNP	OS01G0173900	Intronic	NA	NA	LOC_Os01g07870	ABC transporter family protein, putative, expressed
Chr1	3810122	3810122	C	A	C	SNP	OS01G0173900	Intronic	NA	NA	LOC_Os01g07870	ABC transporter family protein, putative, expressed
Chr1	3810385	3810385	G	A	G	SNP	OS01G0173900	Exonic	3UTR	NA	LOC_Os01g07870	ABC transporter family protein, putative, expressed
Chr1	3810401	3810401	T	A	T	SNP	OS01G0173900	Exonic	3UTR	NA	LOC_Os01g07870	ABC transporter family protein, putative, expressed
Chr1	3810555	3810555	G	A	G	SNP	OS01G0173900	Exonic	3UTR	NA	LOC_Os01g07870	ABC transporter family protein, putative, expressed
Chr1	3810621	3810621	G	A	G	SNP	OS01G0173900	Exonic	3UTR	NA	LOC_Os01g07870	ABC transporter family protein, putative, expressed
Chr1	3820399	3820399	G	A	G	SNP	OS01G0174300	Exonic	3UTR	NA	LOC_Os01g07910	NADH-cytochrome b5 reductase, putative, expressed
Chr1	3820603	3820603	G	A	G	SNP	OS01G0174300	Intronic	NA	NA	LOC_Os01g07910	NADH-cytochrome b5 reductase, putative, expressed
Chr1	3820740	3820740	G	A	G	SNP	OS01G0174300	Intronic	NA	NA	LOC_Os01g07910	NADH-cytochrome b5 reductase, putative, expressed
Chr1	3820823	3820823	G	A	G	SNP	OS01G0174300	Intronic	NA	NA	LOC_Os01g07910	NADH-cytochrome b5 reductase, putative, expressed
Chr1	3821083	3821083	G	A	G	SNP	OS01G0174300	Intronic	NA	NA	LOC_Os01g07910	NADH-cytochrome b5 reductase, putative, expressed
Chr1	3821104	3821104	C	A	C	SNP	OS01G0174300	Intronic	NA	NA	LOC_Os01g07910	NADH-cytochrome b5 reductase, putative, expressed
Chr1	3821256	3821256	C	A	C	SNP	OS01G0174300	Intronic	NA	NA	LOC_Os01g07910	NADH-cytochrome b5 reductase, putative, expressed
Chr1	3821514	3821514	G	A	G	SNP	OS01G0174300	Intronic	NA	NA	LOC_Os01g07910	NADH-cytochrome b5 reductase, putative, expressed
Chr1	3821627	3821627	G	A	G	SNP	OS01G0174300	Intronic	NA	NA	LOC_Os01g07910	NADH-cytochrome b5 reductase, putative, expressed
Chr1	3822011	3822011	T	A	T	SNP	OS01G0174300	Intronic	NA	NA	LOC_Os01g07910	NADH-cytochrome b5 reductase, putative, expressed
Chr1	3822135	3822135	T	A	T	SNP	OS01G0174300	Intronic	NA	NA	LOC_Os01g07910	NADH-cytochrome b5 reductase, putative, expressed
Chr1	3822309	3822309	G	A	G	SNP	OS01G0174300	Intronic	NA	NA	LOC_Os01g07910	NADH-cytochrome b5 reductase, putative, expressed
Chr1	3822334	3822334	G	A	G	SNP	OS01G0174300	Intronic	NA	NA	LOC_Os01g07910	NADH-cytochrome b5 reductase, putative, expressed
Chr1	3822721	3822721	G	A	G	SNP	OS01G0174300	Intronic	NA	NA	LOC_Os01g07910	NADH-cytochrome b5 reductase, putative, expressed
Chr1	3822779	3822779	C	A	C	SNP	OS01G0174300	Intronic	NA	NA	LOC_Os01g07910	NADH-cytochrome b5 reductase, putative, expressed
Chr1	3822798	3822798	T	A	T	SNP	OS01G0174300	Intronic	NA	NA	LOC_Os01g07910	NADH-cytochrome b5 reductase, putative, expressed
Chr1	3822864	3822864	C	A	C	SNP	OS01G0174300	Intronic	NA	NA	LOC_Os01g07910	NADH-cytochrome b5 reductase, putative, expressed
Chr1	3822990	3822990	G	A	G	SNP	OS01G0174300	Intronic	NA	NA	LOC_Os01g07910	NADH-cytochrome b5 reductase, putative, expressed
Chr1	3822997	3822997	G	A	G	SNP	OS01G0174300	Intronic	NA	NA	LOC_Os01g07910	NADH-cytochrome b5 reductase, putative, expressed
Chr1	3823062	3823062	T	A	T	SNP	OS01G0174300	Intronic	NA	NA	LOC_Os01g07910	NADH-cytochrome b5 reductase, putative, expressed
Chr1	3823482	3823482	G	A	G	SNP	OS01G0174300	Intronic	NA	NA	LOC_Os01g07910	NADH-cytochrome b5 reductase, putative, expressed
Chr1	3823537	3823537	G	A	G	SNP	OS01G0174300	Intronic	NA	NA	LOC_Os01g07910	NADH-cytochrome b5 reductase, putative, expressed
Chr1	3823563	3823563	G	A	G	SNP	OS01G0174300	Intronic	NA	NA	LOC_Os01g07910	NADH-cytochrome b5 reductase, putative, expressed
Chr1	3823718	3823718	G	A	G	SNP	OS01G0174300	Intronic	NA	NA	LOC_Os01g07910	NADH-cytochrome b5 reductase, putative, expressed
Chr1	3823740	3823740	G	A	G	SNP	OS01G0174300	Intronic	NA	NA	LOC_Os01g07910	NADH-cytochrome b5 reductase, putative, expressed
Chr1	3824010	3824010	G	A	G	SNP	OS01G0174300	Intronic	NA	NA	LOC_Os01g07910	NADH-cytochrome b5 reductase, putative, expressed
Chr1	3824022	3824022	C	A	C	SNP	OS01G0174300	Intronic	NA	NA	LOC_Os01g07910	NADH-cytochrome b5 reductase, putative, expressed
Chr1	3919340	3919340	G	A	G	SNP	OS01G0176000	Exonic	CDS	Missense	LOC_Os01g08090	flavonol-3-O-glycoside-7-O-glucosyltransferase 1, putative, expressed
Chr1	3919666	3919666	T	A	T	SNP	OS01G0176000	Exonic	CDS	Silent	LOC_Os01g08090	flavonol-3-O-glycoside-7-O-glucosyltransferase 1, putative, expressed
Chr1	5508406	5508406	T	A	T	SNP	OS01G0201100	Intronic	NA	NA	LOC_Os01g10440	xylosyltransferase, putative, expressed
Chr1	5876997	5876997	G	A	G	SNP	OS01G0208400	Exonic	CDS	Silent	LOC_Os01g11010	peptide-N4-asparagine amidase A, putative, expressed
Chr1	5902197	5902197	C	A	C	SNP	OS01G0208700	Intronic	NA	NA	LOC_Os01g11054	phosphoenolpyruvate carboxylase, putative, expressed
Chr1	5902275	5902275	G	A	G	SNP	OS01G0208700	Intronic	NA	NA	LOC_Os01g11054	phosphoenolpyruvate carboxylase, putative, expressed
Chr1	5904271	5904271	G	A	G	SNP	OS01G0208700	Intronic	NA	NA	LOC_Os01g11054	phosphoenolpyruvate carboxylase, putative, expressed
Chr1	5904299	5904299	G	A	G	SNP	OS01G0208700	Intronic	NA	NA	LOC_Os01g11054	phosphoenolpyruvate carboxylase, putative, expressed
Chr1	5904460	5904460	G	A	G	SNP	OS01G0208700	Intronic	NA	NA	LOC_Os01g11054	phosphoenolpyruvate carboxylase, putative, expressed
Chr1	5905774	5905774	G	A	G	SNP	OS01G0208700	Intronic	NA	NA	LOC_Os01g11054	phosphoenolpyruvate carboxylase, putative, expressed
Chr1	5906465	5906465	T	A	T	SNP	OS01G0208700	Intronic	NA	NA	LOC_Os01g11054	phosphoenolpyruvate carboxylase, putative, expressed
Chr1	5906566	5906566	G	A	G	SNP	OS01G0208700	Intronic	NA	NA	LOC_Os01g11054	phosphoenolpyruvate carboxylase, putative, expressed
Chr1	5906708	5906708	G	A	G	SNP	OS01G0208700	Intronic	NA	NA	LOC_Os01g11054	phosphoenolpyruvate carboxylase, putative, expressed
Chr1	5908939	5908939	G	A	G	SNP	OS01G0208700	Intronic	NA	NA	LOC_Os01g11054	phosphoenolpyruvate carboxylase, putative, expressed
Chr1	6612924	6612924	G	A	G	SNP	OS01G0220700	Intronic	NA	NA	LOC_Os01g12130	nodulin MtN3 family protein, putative, expressed
Chr1	6969721	6969721	G	A	G	SNP	OS01G0226300	Exonic	CDS	NA	LOC_Os01g12650	reticulon domain containing protein, putative, expressed
Chr1	7024337	7024337	C	A	C	SNP	OS01G0227100	Exonic	5UTR	NA	LOC_Os01g12710	oxidoreductase, short chain dehydrogenase/reductase family domain containing protein, expressed
Chr1	7030142	7030142	G	A	G	SNP	OS01G0227100	Exonic	3UTR	NA	LOC_Os01g12710	oxidoreductase, short chain dehydrogenase/reductase family domain containing protein, expressed
Chr1	7364714	7364714	C	A	C	SNP	OS01G0233000	Intronic	NA	NA	LOC_Os01g13210	salt stress root protein RS1, putative, expressed
Chr1	7550679	7550679	T	A	T	SNP	OS01G0236300	Intronic	NA	NA	LOC_Os01g13520	auxin response factor 1, putative, expressed
Chr1	7558287	7558287	T	A	T	SNP	OS01G0236400	Intronic	NA	NA	LOC_Os01g13530	ABIL2, putative, expressed

Chr1	7558829	7558829	T	A	T	SNP	OS01G0236400	Intronic	NA	NA	LOC_Os01g13530	ABIL2, putative, expressed
Chr1	7619594	7619594	G	A	G	SNP	OS01G0237500	Intronic	NA	NA	LOC_Os01g13610	isoflavone reductase homolog IRL, putative, expressed
Chr1	7864241	7864241	G	A	G	SNP	OS01G0242600	Intronic	NA	NA	LOC_Os01g14050	C2 domain containing protein, putative, expressed
Chr1	7865788	7865788	G	A	G	SNP	OS01G0242600	Intronic	NA	NA	LOC_Os01g14050	C2 domain containing protein, putative, expressed
Chr1	7866251	7866251	C	A	C	SNP	OS01G0242600	Intronic	NA	NA	LOC_Os01g14050	C2 domain containing protein, putative, expressed
Chr1	8177155	8177155	G	A	G	SNP	OS01G0248500	Intronic	NA	NA	LOC_Os01g14590	pathogen-related protein, putative, expressed
Chr1	8177186	8177186	G	A	G	SNP	OS01G0248500	Intronic	NA	NA	LOC_Os01g14590	pathogen-related protein, putative, expressed
Chr1	8205322	8205322	G	A	G	SNP	OS01G0249200	Intronic	NA	NA	LOC_Os01g14670	Cupin domain containing protein, expressed
Chr1	8205941	8205941	G	A	G	SNP	OS01G0249200	Intronic	NA	NA	LOC_Os01g14670	Cupin domain containing protein, expressed
Chr1	8206024	8206024	C	A	C	SNP	OS01G0249200	Intronic	NA	NA	LOC_Os01g14670	Cupin domain containing protein, expressed
Chr1	8206067	8206067	T	A	T	SNP	OS01G0249200	Intronic	NA	NA	LOC_Os01g14670	Cupin domain containing protein, expressed
Chr1	8406786	8406786	G	A	G	SNP	OS01G0253900	Exonic	CDS	Silent	LOC_Os01g15000	lipase, putative, expressed
Chr1	8421101	8421101	G	A	G	SNP	OS01G0254100	Exonic	3UTR	NA	LOC_Os01g15020	lissencephaly type-1-like homology motif, putative, expressed
Chr1	8422075	8422075	G	A	G	SNP	OS01G0254100	Intronic	NA	NA	LOC_Os01g15020	lissencephaly type-1-like homology motif, putative, expressed
Chr1	8423665	8423665	G	A	G	SNP	OS01G0254100	Intronic	NA	NA	LOC_Os01g15020	lissencephaly type-1-like homology motif, putative, expressed
Chr1	8423965	8423965	G	A	G	SNP	OS01G0254100	Intronic	NA	NA	LOC_Os01g15020	lissencephaly type-1-like homology motif, putative, expressed
Chr1	8424698	8424698	G	A	G	SNP	OS01G0254100	Intronic	NA	NA	LOC_Os01g15020	lissencephaly type-1-like homology motif, putative, expressed
Chr1	8424910	8424910	C	A	C	SNP	OS01G0254100	Intronic	NA	NA	LOC_Os01g15020	lissencephaly type-1-like homology motif, putative, expressed
Chr1	8425661	8425661	G	A	G	SNP	OS01G0254100	Intronic	NA	NA	LOC_Os01g15020	lissencephaly type-1-like homology motif, putative, expressed
Chr1	8426379	8426379	G	A	G	SNP	OS01G0254100	Intronic	NA	NA	LOC_Os01g15020	lissencephaly type-1-like homology motif, putative, expressed
Chr1	8426601	8426601	C	A	C	SNP	OS01G0254100	Intronic	NA	NA	LOC_Os01g15020	lissencephaly type-1-like homology motif, putative, expressed
Chr1	8427287	8427287	C	A	C	SNP	OS01G0254100	Exonic	CDS	Missense	LOC_Os01g15020	lissencephaly type-1-like homology motif, putative, expressed
Chr1	8427732	8427732	G	A	G	SNP	OS01G0254100	Intronic	NA	NA	LOC_Os01g15020	lissencephaly type-1-like homology motif, putative, expressed
Chr1	8428473	8428473	G	A	G	SNP	OS01G0254100	Exonic	CDS	Silent	LOC_Os01g15020	lissencephaly type-1-like homology motif, putative, expressed
Chr1	8428968	8428968	T	A	T	SNP	OS01G0254100	Intronic	NA	NA	LOC_Os01g15020	lissencephaly type-1-like homology motif, putative, expressed
Chr1	8429325	8429325	C	A	C	SNP	OS01G0254100	Intronic	NA	NA	LOC_Os01g15020	lissencephaly type-1-like homology motif, putative, expressed
Chr1	9115208	9115208	C	A	C	SNP	OS01G0266500	Intronic	NA	NA	LOC_Os01g16146	phenazine biosynthesis protein, putative, expressed
Chr1	9658842	9658842	G	A	G	SNP	OS01G0276000	Intronic	NA	NA	LOC_Os01g16890	ribosomal protein L7Ae, putative, expressed
Chr1	9659509	9659509	T	A	T	SNP	OS01G0276000	Intronic	NA	NA	LOC_Os01g16890	ribosomal protein L7Ae, putative, expressed
Chr1	9671362	9671362	G	A	G	SNP	OS01G0276200	Intronic	NA	NA	LOC_Os01g16910	outer mitochondrial membrane porin, putative, expressed
Chr1	9671515	9671515	T	A	T	SNP	OS01G0276200	Intronic	NA	NA	LOC_Os01g16910	outer mitochondrial membrane porin, putative, expressed
Chr1	9675363	9675363	G	A	G	SNP	OS01G0276200	Intronic	NA	NA	LOC_Os01g16910	outer mitochondrial membrane porin, putative, expressed
Chr1	9675942	9675942	G	A	G	SNP	OS01G0276200	Exonic	CDS	NA	LOC_Os01g16910	outer mitochondrial membrane porin, putative, expressed
Chr1	11319134	11319134	T	A	T	SNP	OS01G0305200	Intronic	NA	NA	LOC_Os01g19940	expressed protein
Chr1	21415195	21415195	T	A	T	SNP	OS01G0563000	Intronic	NA	NA	LOC_Os01g38229	peptidyl-prolyl isomerase, putative, expressed
Chr1	21424461	21424461	G	A	G	SNP	OS01G0563000	Intronic	NA	NA	LOC_Os01g38229	peptidyl-prolyl isomerase, putative, expressed
Chr1	21424705	21424705	T	A	T	SNP	OS01G0563000	Exonic	CDS	Silent	LOC_Os01g38229	peptidyl-prolyl isomerase, putative, expressed
Chr1	21426048	21426048	G	A	G	SNP	OS01G0563000	Exonic	CDS	Missense	LOC_Os01g38229	peptidyl-prolyl isomerase, putative, expressed
Chr1	21441147	21441147	G	A	G	SNP	OS01G0563000	Exonic	CDS	Missense	LOC_Os01g38229	peptidyl-prolyl isomerase, putative, expressed
Chr1	21631517	21631517	C	A	C	SNP	OS01G0565900	Exonic	5UTR	NA	LOC_Os01g38510	protein transport protein Sec61 subunit beta, putative, expressed
Chr1	21631710	21631710	T	A	T	SNP	OS01G0565900	Exonic	5UTR	NA	LOC_Os01g38510	protein transport protein Sec61 subunit beta, putative, expressed
Chr1	21631905	21631905	T	A	T	SNP	OS01G0565900	Exonic	5UTR	NA	LOC_Os01g38510	protein transport protein Sec61 subunit beta, putative, expressed
Chr1	22747945	22747945	G	A	G	SNP	OS01G0585200	Exonic	3UTR	NA	LOC_Os01g40290	expressed protein
Chr1	23344102	23344102	G	A	G	SNP	OS01G0595600	Intronic	NA	NA	LOC_Os01g41240	hydrolase, alpha/beta fold family domain containing protein, expressed
Chr1	24075261	24075261	G	A	G	SNP	OS01G0609300	Exonic	3UTR	NA	LOC_Os01g42380	pleiotropic drug resistance protein, putative, expressed
Chr1	24075704	24075704	C	A	C	SNP	OS01G0609300	Intronic	NA	NA	LOC_Os01g42380	pleiotropic drug resistance protein, putative, expressed
Chr1	24079221	24079221	T	A	T	SNP	OS01G0609300	Intronic	NA	NA	LOC_Os01g42380	pleiotropic drug resistance protein, putative, expressed
Chr1	24110159	24110159	G	A	G	SNP	OS01G0609900	Exonic	CDS	NA	LOC_Os01g42410	pleiotropic drug resistance protein, putative, expressed
Chr1	24110177	24110177	C	A	C	SNP	OS01G0609900	Exonic	CDS	NA	LOC_Os01g42410	pleiotropic drug resistance protein, putative, expressed
Chr1	24112099	24112099	G	A	G	SNP	OS01G0609900	Exonic	CDS	NA	LOC_Os01g42410	pleiotropic drug resistance protein, putative, expressed
Chr1	24114494	24114494	G	A	G	SNP	OS01G0609900	Intronic	NA	NA	LOC_Os01g42410	pleiotropic drug resistance protein, putative, expressed
Chr1	24114603	24114603	G	A	G	SNP	OS01G0609900	Intronic	NA	NA	LOC_Os01g42410	pleiotropic drug resistance protein, putative, expressed
Chr1	24393217	24393217	C	A	C	SNP	OS01G0615100	Exonic	CDS	Silent	LOC_Os01g42860	inhibitor I family protein, putative, expressed
Chr1	24393257	24393257	G	A	G	SNP	OS01G0615100	Exonic	CDS	Missense	LOC_Os01g42860	inhibitor I family protein, putative, expressed
Chr1	24579988	24579988	T	A	T	SNP	OS01G0618100	Exonic	CDS	Silent	LOC_Os01g43090	oxidoreductase, aldo/keto reductase family protein, putative, expressed
Chr1	24580143	24580143	G	A	G	SNP	OS01G0618100	Intronic	NA	NA	LOC_Os01g43090	oxidoreductase, aldo/keto reductase family protein, putative, expressed
Chr1	24785374	24785374	G	A	G	SNP	OS01G0621600	Intronic	NA	NA	LOC_Os01g43350	protein kinase family protein, putative, expressed
Chr1	24785376	24785376	G	A	G	SNP	OS01G0621600	Intronic	NA	NA	LOC_Os01g43350	protein kinase family protein, putative, expressed
Chr1	24786734	24786734	T	A	T	SNP	OS01G0621600	Exonic	CDS	Missense	LOC_Os01g43350	protein kinase family protein, putative, expressed
Chr1	24787530	24787530	G	A	G	SNP	OS01G0621600	Intronic	NA	NA	LOC_Os01g43350	protein kinase family protein, putative, expressed
Chr1	25599917	25599917	G	A	G	SNP	OS01G0638000	Exonic	CDS	Silent	LOC_Os01g45110	anthocyanin 3-O-beta-glucosyltransferase, putative, expressed
Chr1	25599980	25599980	G	A	G	SNP	OS01G0638000	Exonic	CDS	Silent	LOC_Os01g45110	anthocyanin 3-O-beta-glucosyltransferase, putative, expressed
Chr1	26223973	26223973	T	A	T	SNP	OS01G0649900	Exonic	CDS	Missense	LOC_Os01g46120	GDSL-like lipase/acylhydrolase, putative, expressed
Chr1	26792025	26792025	G	A	G	SNP	OS01G0658700	Exonic	3UTR	NA	LOC_Os01g46950	aldose 1-epimerase, putative, expressed
Chr1	26801360	26801360	C	A	C	SNP	OS01G0658900	Intronic	NA	NA	LOC_Os01g46970	transcription factor, putative, expressed
Chr1	26805268	26805268	C	A	C	SNP	OS01G0658900	Intronic	NA	NA	LOC_Os01g46970	transcription factor, putative, expressed
Chr1	26889868	26889868	G	A	G	SNP	OS01G0660300	Intronic	NA	NA	LOC_Os01g47080	pyruvate kinase, putative, expressed
Chr1	26894906	26894906	G	A	G	SNP	OS01G0660300	Intronic	NA	NA	LOC_Os01g47080	pyruvate kinase, putative, expressed
Chr1	26894950	26894950	G	A	G	SNP	OS01G0660300	Intronic	NA	NA	LOC_Os01g47080	pyruvate kinase, putative, expressed
Chr1	27102597	27102597	G	A	G	SNP	OS01G0663800	Exonic	CDS	Missense	LOC_Os01g47430	protein of unknown function DUF1296 domain containing protein, expressed
Chr1	27104142	27104142	C	A	C	SNP	OS01G0663800	Intronic	NA	NA	LOC_Os01g47430	protein of unknown function DUF1296 domain containing protein, expressed
Chr1	27107978	27107978	T	A	T	SNP	OS01G0663800	Exonic	CDS	Missense	LOC_Os01g47430	protein of unknown function DUF1296 domain containing protein, expressed
Chr1	27109408	27109408	C	A	C	SNP	OS01G0663800	Intronic	NA	NA	LOC_Os01g47430	protein of unknown function DUF1296 domain containing protein, expressed
Chr1	28031560	28031560	C	A	C	SNP	OS01G0680900	Exonic	5UTR	NA	LOC_Os01g48850	auxin-responsive protein, putative, expressed
Chr1	28573692	28573692	G	A	G	SNP	OS01G0692300	Exonic	CDS	Silent	LOC_Os01g49740	expressed protein
Chr1	29190343	29190343	C	A	C	SNP	OS01G0704100	Exonic	CDS	NA	LOC_Os01g50820	transporter, major facilitator family, putative, expressed
Chr1	30262328	30262328	C	A	C	SNP	OS01G0725800	Intronic	NA	NA	LOC_Os01g52640	suppressor of phythchrome A, putative, expressed
Chr1	30345390	30345390	C	A	C	SNP	OS01G0727800	Exonic	CDS	NA	LOC_Os01g52750	OsSub3 - Putative Subtilisin homologue, expressed
Chr1	30933569	30933569	G	A	G	SNP	OS01G0740600	Exonic	3UTR	NA	LOC_Os01g53810	peptidase, putative, expressed
Chr1	30949522	30949522	G	A	G	SNP	OS01G0741200	Exonic	3UTR	NA	LOC_Os01g53840	protein kinase family protein, putative, expressed
Chr1	31100069	31100069	C	A	C	SNP	OS01G0744000	Intronic	NA	NA	LOC_Os01g54080	kinesin motor protein-related, putative, expressed
Chr1	31105670	31105670	G	A	G	SNP	OS01G0744000	Exonic	CDS	Silent	LOC_Os01g54080	kinesin motor protein-related, putative, expressed
Chr1	31105818	31105818	T	A	T	SNP	OS01G0744000	Intronic	NA	NA	LOC_Os01g54080	kinesin motor protein-related, putative, expressed
Chr1	31273205	31273205	C	A	C	SNP	OS01G0747300	Exonic	3UTR	NA	LOC_Os01g54340	plant-specific domain TIGR01615 family protein, expressed
Chr1	31273206	31273206	G	A	G	SNP	OS01G0747300	Exonic	3UTR	NA	LOC_Os01g54340	plant-specific domain TIGR01615 family protein, expressed

Chr1	31377505	31377505	G	A	G	SNP	OS01G0749400	Exonic	5UTR	NA	LOC_Os01g54560	trehalose synthase, putative, expressed
Chr1	31455050	31455050	G	A	G	SNP	OS01G0750500	Intronic	NA	NA	LOC_Os01g54670	coiled-coil domain-containing protein 25, putative, expressed
Chr1	31524847	31524847	G	A	G	SNP	OS01G0751600	Exonic	CDS	Silent	LOC_Os01g54810	lipase, putative, expressed
Chr1	31526497	31526497	T	A	T	SNP	OS01G0751600	Intronic	NA	NA	LOC_Os01g54810	lipase, putative, expressed
Chr1	31576819	31576819	G	A	G	SNP	OS01G0752700	Exonic	CDS	Silent	LOC_Os01g54910	GTP-binding protein typA/bipA, putative, expressed
Chr1	31577148	31577148	G	A	G	SNP	OS01G0752700	Intronic	NA	NA	LOC_Os01g54910	GTP-binding protein typA/bipA, putative, expressed
Chr1	31577823	31577823	G	A	G	SNP	OS01G0752700	Intronic	NA	NA	LOC_Os01g54910	GTP-binding protein typA/bipA, putative, expressed
Chr1	31577963	31577963	G	A	G	SNP	OS01G0752700	Intronic	NA	NA	LOC_Os01g54910	GTP-binding protein typA/bipA, putative, expressed
Chr1	31578975	31578975	G	A	G	SNP	OS01G0752700	Intronic	NA	NA	LOC_Os01g54910	GTP-binding protein typA/bipA, putative, expressed
Chr1	31579004	31579004	G	A	G	SNP	OS01G0752700	Intronic	NA	NA	LOC_Os01g54910	GTP-binding protein typA/bipA, putative, expressed
Chr1	31580087	31580087	G	A	G	SNP	OS01G0752700	Intronic	NA	NA	LOC_Os01g54910	GTP-binding protein typA/bipA, putative, expressed
Chr1	31580545	31580545	G	A	G	SNP	OS01G0752700	Exonic	CDS	Missense	LOC_Os01g54910	GTP-binding protein typA/bipA, putative, expressed
Chr1	31580897	31580897	G	A	G	SNP	OS01G0752700	Intronic	NA	NA	LOC_Os01g54910	GTP-binding protein typA/bipA, putative, expressed
Chr1	31729965	31729965	G	A	G	SNP	OS01G0756200	Intronic	NA	NA	LOC_Os01g55150	bZIP transcription factor domain containing protein, expressed
Chr1	32132140	32132140	G	A	G	SNP	OS01G0763600	Intronic	NA	NA	LOC_Os01g55780	glycerophosphoryl diester phosphodiesterase family protein, putative, expressed
Chr1	32136270	32136270	G	A	G	SNP	OS01G0763600	Intronic	NA	NA	LOC_Os01g55780	glycerophosphoryl diester phosphodiesterase family protein, putative, expressed
Chr1	32253204	32253204	G	A	G	SNP	OS01G0765500	Exonic	CDS	Silent	LOC_Os01g56020	expressed protein
Chr1	32254018	32254018	G	A	G	SNP	OS01G0765500	Exonic	CDS	Missense	LOC_Os01g56020	expressed protein
Chr1	32715305	32715305	G	A	G	SNP	OS01G0773800	Intronic	NA	NA	LOC_Os01g56690	helix-loop-helix DNA-binding domain containing protein, expressed
Chr1	32759263	32759263	T	A	T	SNP	OS01G0775100	Exonic	3UTR	NA	LOC_Os01g56780	plus-3 domain containing protein, expressed
Chr1	32760027	32760027	T	A	T	SNP	OS01G0775100	Intronic	NA	NA	LOC_Os01g56780	plus-3 domain containing protein, expressed
Chr1	32760703	32760703	G	A	G	SNP	OS01G0775100	Intronic	NA	NA	LOC_Os01g56780	plus-3 domain containing protein, expressed
Chr1	32760859	32760859	G	A	G	SNP	OS01G0775100	Exonic	CDS	NA	LOC_Os01g56780	plus-3 domain containing protein, expressed
Chr1	32762262	32762262	T	A	T	SNP	OS01G0775100	Exonic	CDS	NA	LOC_Os01g56780	plus-3 domain containing protein, expressed
Chr1	32771486	32771486	G	A	G	SNP	OS01G0775200	Exonic	3UTR	NA	LOC_Os01g56790	expressed protein
Chr1	32848891	32848891	G	A	G	SNP	OS01G0776600	Intronic	NA	NA	LOC_Os01g56880	purple acid phosphatase precursor, putative, expressed
Chr1	32849273	32849273	G	A	G	SNP	OS01G0776600	Intronic	NA	NA	LOC_Os01g56880	purple acid phosphatase precursor, putative, expressed
Chr1	32849787	32849787	T	A	T	SNP	OS01G0776600	Intronic	NA	NA	LOC_Os01g56880	purple acid phosphatase precursor, putative, expressed
Chr1	32850415	32850415	C	A	C	SNP	OS01G0776600	Intronic	NA	NA	LOC_Os01g56880	purple acid phosphatase precursor, putative, expressed
Chr1	33073854	33073854	C	A	C	SNP	OS01G0780800	Exonic	CDS	Missense	LOC_Os01g57240	expressed protein
Chr1	33074712	33074712	G	A	G	SNP	OS01G0780800	Intronic	NA	NA	LOC_Os01g57240	expressed protein
Chr1	33074731	33074731	G	A	G	SNP	OS01G0780800	Intronic	NA	NA	LOC_Os01g57240	expressed protein
Chr1	33185655	33185655	T	A	T	SNP	OS01G0783200	Exonic	3UTR	NA	LOC_Os01g57420	diacylglycerol kinase, putative, expressed
Chr1	33187417	33187417	T	A	T	SNP	OS01G0783200	Intronic	NA	NA	LOC_Os01g57420	diacylglycerol kinase, putative, expressed
Chr1	33187831	33187831	G	A	G	SNP	OS01G0783200	Intronic	NA	NA	LOC_Os01g57420	diacylglycerol kinase, putative, expressed
Chr1	33189188	33189188	C	A	C	SNP	OS01G0783200	Intronic	NA	NA	LOC_Os01g57420	diacylglycerol kinase, putative, expressed
Chr1	33190325	33190325	C	A	C	SNP	OS01G0783200	Intronic	NA	NA	LOC_Os01g57420	diacylglycerol kinase, putative, expressed
Chr1	33190581	33190581	T	A	T	SNP	OS01G0783200	Exonic	CDS	Silent	LOC_Os01g57420	diacylglycerol kinase, putative, expressed
Chr1	33191279	33191279	G	A	G	SNP	OS01G0783200	Intronic	NA	NA	LOC_Os01g57420	diacylglycerol kinase, putative, expressed
Chr1	33191788	33191788	G	A	G	SNP	OS01G0783200	Intronic	NA	NA	LOC_Os01g57420	diacylglycerol kinase, putative, expressed
Chr1	33191985	33191985	C	A	C	SNP	OS01G0783200	Intronic	NA	NA	LOC_Os01g57420	diacylglycerol kinase, putative, expressed
Chr1	33192411	33192411	T	A	T	SNP	OS01G0783200	Intronic	NA	NA	LOC_Os01g57420	diacylglycerol kinase, putative, expressed
Chr1	33192646	33192646	T	A	T	SNP	OS01G0783200	Intronic	NA	NA	LOC_Os01g57420	diacylglycerol kinase, putative, expressed
Chr1	33199251	33199251	C	A	C	SNP	OS01G0783500	Intronic	NA	NA	LOC_Os01g57450	universal stress protein domain containing protein, putative, expressed
Chr1	33199686	33199686	G	A	G	SNP	OS01G0783500	Intronic	NA	NA	LOC_Os01g57450	universal stress protein domain containing protein, putative, expressed
Chr1	33199715	33199715	G	A	G	SNP	OS01G0783500	Intronic	NA	NA	LOC_Os01g57450	universal stress protein domain containing protein, putative, expressed
Chr1	33199909	33199909	G	A	G	SNP	OS01G0783500	Intronic	NA	NA	LOC_Os01g57450	universal stress protein domain containing protein, putative, expressed
Chr1	33410165	33410165	G	A	G	SNP	OS01G0787600	Intronic	NA	NA	LOC_Os01g57770	OsPOP4 - Putative Prolyl Oligopeptidase homologue, expressed
Chr1	33410524	33410524	G	A	G	SNP	OS01G0787600	Intronic	NA	NA	LOC_Os01g57770	OsPOP4 - Putative Prolyl Oligopeptidase homologue, expressed
Chr1	33410611	33410611	G	A	G	SNP	OS01G0787600	Intronic	NA	NA	LOC_Os01g57770	OsPOP4 - Putative Prolyl Oligopeptidase homologue, expressed
Chr1	33411430	33411430	C	A	C	SNP	OS01G0787600	Intronic	NA	NA	LOC_Os01g57770	OsPOP4 - Putative Prolyl Oligopeptidase homologue, expressed
Chr1	33411576	33411576	G	A	G	SNP	OS01G0787600	Intronic	NA	NA	LOC_Os01g57770	OsPOP4 - Putative Prolyl Oligopeptidase homologue, expressed
Chr1	33411661	33411661	G	A	G	SNP	OS01G0787600	Intronic	NA	NA	LOC_Os01g57770	OsPOP4 - Putative Prolyl Oligopeptidase homologue, expressed
Chr1	33411663	33411663	T	A	T	SNP	OS01G0787600	Intronic	NA	NA	LOC_Os01g57770	OsPOP4 - Putative Prolyl Oligopeptidase homologue, expressed
Chr1	33411682	33411682	C	A	C	SNP	OS01G0787600	Intronic	NA	NA	LOC_Os01g57770	OsPOP4 - Putative Prolyl Oligopeptidase homologue, expressed
Chr1	33411760	33411760	G	A	G	SNP	OS01G0787600	Intronic	NA	NA	LOC_Os01g57770	OsPOP4 - Putative Prolyl Oligopeptidase homologue, expressed
Chr1	33411786	33411786	G	A	G	SNP	OS01G0787600	Intronic	NA	NA	LOC_Os01g57770	OsPOP4 - Putative Prolyl Oligopeptidase homologue, expressed
Chr1	33411792	33411792	C	A	C	SNP	OS01G0787600	Intronic	NA	NA	LOC_Os01g57770	OsPOP4 - Putative Prolyl Oligopeptidase homologue, expressed
Chr1	33611428	33611428	C	A	C	SNP	OS01G0793500	Exonic	CDS	Silent	LOC_Os01g58114	expressed protein
Chr1	33767565	33767565	T	A	T	SNP	OS01G0797600	Exonic	CDS	Silent	LOC_Os01g58420	AP2 domain containing protein, expressed
Chr1	33838809	33838809	T	A	T	SNP	OS01G0799500	Intronic	NA	NA	LOC_Os01g58550	methyladenine glycosylase, putative, expressed
Chr1	33839397	33839397	G	A	G	SNP	OS01G0799500	Intronic	NA	NA	LOC_Os01g58550	methyladenine glycosylase, putative, expressed
Chr1	33839421	33839421	G	A	G	SNP	OS01G0799500	Intronic	NA	NA	LOC_Os01g58550	methyladenine glycosylase, putative, expressed
Chr1	33839507	33839507	G	A	G	SNP	OS01G0799500	Intronic	NA	NA	LOC_Os01g58550	methyladenine glycosylase, putative, expressed
Chr1	33840933	33840933	T	A	T	SNP	OS01G0799500	Exonic	CDS	Missense	LOC_Os01g58550	methyladenine glycosylase, putative, expressed
Chr1	33840934	33840934	T	A	T	SNP	OS01G0799500	Exonic	CDS	Missense	LOC_Os01g58550	methyladenine glycosylase, putative, expressed
Chr1	33897827	33897827	C	A	C	SNP	OS01G0800500	Intronic	NA	NA	LOC_Os01g58640	nucleotide pyrophosphatase/phosphodiesterase, putative, expressed
Chr1	33898099	33898099	C	A	C	SNP	OS01G0800500	Intronic	NA	NA	LOC_Os01g58640	nucleotide pyrophosphatase/phosphodiesterase, putative, expressed
Chr1	33898832	33898832	G	A	G	SNP	OS01G0800500	Intronic	NA	NA	LOC_Os01g58640	nucleotide pyrophosphatase/phosphodiesterase, putative, expressed
Chr1	33898882	33898882	T	A	T	SNP	OS01G0800500	Intronic	NA	NA	LOC_Os01g58640	nucleotide pyrophosphatase/phosphodiesterase, putative, expressed
Chr1	33899305	33899305	G	A	G	SNP	OS01G0800500	Intronic	NA	NA	LOC_Os01g58640	nucleotide pyrophosphatase/phosphodiesterase, putative, expressed
Chr1	33899367	33899367	G	A	G	SNP	OS01G0800500	Intronic	NA	NA	LOC_Os01g58640	nucleotide pyrophosphatase/phosphodiesterase, putative, expressed
Chr1	33900344	33900344	G	A	G	SNP	OS01G0800500	Intronic	NA	NA	LOC_Os01g58640	nucleotide pyrophosphatase/phosphodiesterase, putative, expressed
Chr1	33900723	33900723	G	A	G	SNP	OS01G0800500	Intronic	NA	NA	LOC_Os01g58640	nucleotide pyrophosphatase/phosphodiesterase, putative, expressed
Chr1	33900838	33900838	C	A	C	SNP	OS01G0800500	Intronic	NA	NA	LOC_Os01g58640	nucleotide pyrophosphatase/phosphodiesterase, putative, expressed
Chr1	33900868	33900868	C	A	C	SNP	OS01G0800500	Intronic	NA	NA	LOC_Os01g58640	nucleotide pyrophosphatase/phosphodiesterase, putative, expressed
Chr1	33903514	33903514	G	A	G	SNP	OS01G0800500	Intronic	NA	NA	LOC_Os01g58640	nucleotide pyrophosphatase/phosphodiesterase, putative, expressed
Chr1	33903546	33903546	G	A	G	SNP	OS01G0800500	Intronic	NA	NA	LOC_Os01g58640	nucleotide pyrophosphatase/phosphodiesterase, putative, expressed
Chr1	33903809	33903809	G	A	G	SNP	OS01G0800500	Intronic	NA	NA	LOC_Os01g58640	nucleotide pyrophosphatase/phosphodiesterase, putative, expressed
Chr1	33904091	33904091	C	A	C	SNP	OS01G0800500	Intronic	NA	NA	LOC_Os01g58640	nucleotide pyrophosphatase/phosphodiesterase, putative, expressed
Chr1	34034864	34034864	G	A	G	SNP	OS01G0803200	Exonic	3UTR	NA	LOC_Os01g58890	cysteine proteinase inhibitor precursor protein, putative, expressed
Chr1	34142798	34142798	G	A	G	SNP	OS01G0805400	Intronic	NA	NA	LOC_Os01g59100	cytokinin-N-glucosyltransferase 1, putative, expressed
Chr1	34142887	34142887	C	A	C	SNP	OS01G0805400	Intronic	NA	NA	LOC_Os01g59100	cytokinin-N-glucosyltransferase 1, putative, expressed
Chr1	34143043	34143043	G	A	G	SNP	OS01G0805400	Intronic	NA	NA	LOC_Os01g59100	cytokinin-N-glucosyltransferase 1, putative, expressed
Chr1	34445814	34445814	T	A	T	SNP	OS01G0810800	Exonic	3UTR	NA	LOC_Os01g59570	senescence-induced receptor-like serine/threonine-protein kinase precursor, putative, expressed

Chr1	34445932	34445932	G	A	G	SNP	OS01G0810800	Exonic	3UTR	NA	LOC_Os01g59570	senescence-induced receptor-like serine/threonine-protein kinase precursor, putative, expressed
Chr1	34446817	34446817	T	A	T	SNP	OS01G0810800	Intronic	NA	NA	LOC_Os01g59570	senescence-induced receptor-like serine/threonine-protein kinase precursor, putative, expressed
Chr1	34446904	34446904	G	A	G	SNP	OS01G0810800	Intronic	NA	NA	LOC_Os01g59570	senescence-induced receptor-like serine/threonine-protein kinase precursor, putative, expressed
Chr1	34446976	34446976	G	A	G	SNP	OS01G0810800	Intronic	NA	NA	LOC_Os01g59570	senescence-induced receptor-like serine/threonine-protein kinase precursor, putative, expressed
Chr1	34446989	34446989	G	A	G	SNP	OS01G0810800	Intronic	NA	NA	LOC_Os01g59570	senescence-induced receptor-like serine/threonine-protein kinase precursor, putative, expressed
Chr1	34447036	34447036	G	A	G	SNP	OS01G0810800	Intronic	NA	NA	LOC_Os01g59570	senescence-induced receptor-like serine/threonine-protein kinase precursor, putative, expressed
Chr1	34447164	34447164	G	A	G	SNP	OS01G0810800	Intronic	NA	NA	LOC_Os01g59570	senescence-induced receptor-like serine/threonine-protein kinase precursor, putative, expressed
Chr1	34448527	34448527	G	A	G	SNP	OS01G0810800	Intronic	NA	NA	LOC_Os01g59570	senescence-induced receptor-like serine/threonine-protein kinase precursor, putative, expressed
Chr1	34448573	34448573	T	A	T	SNP	OS01G0810800	Intronic	NA	NA	LOC_Os01g59570	senescence-induced receptor-like serine/threonine-protein kinase precursor, putative, expressed
Chr1	34449061	34449061	T	A	T	SNP	OS01G0810800	Intronic	NA	NA	LOC_Os01g59570	senescence-induced receptor-like serine/threonine-protein kinase precursor, putative, expressed
Chr1	34449410	34449410	T	A	T	SNP	OS01G0810800	Intronic	NA	NA	LOC_Os01g59570	senescence-induced receptor-like serine/threonine-protein kinase precursor, putative, expressed
Chr1	34449479	34449479	G	A	G	SNP	OS01G0810800	Intronic	NA	NA	LOC_Os01g59570	senescence-induced receptor-like serine/threonine-protein kinase precursor, putative, expressed
Chr1	34450769	34450769	G	A	G	SNP	OS01G0810800	Intronic	NA	NA	LOC_Os01g59570	senescence-induced receptor-like serine/threonine-protein kinase precursor, putative, expressed
Chr1	34450851	34450851	G	A	G	SNP	OS01G0810800	Intronic	NA	NA	LOC_Os01g59570	senescence-induced receptor-like serine/threonine-protein kinase precursor, putative, expressed
Chr1	34450864	34450864	G	A	G	SNP	OS01G0810800	Intronic	NA	NA	LOC_Os01g59570	senescence-induced receptor-like serine/threonine-protein kinase precursor, putative, expressed
Chr1	34451268	34451268	C	A	C	SNP	OS01G0810800	Intronic	NA	NA	LOC_Os01g59570	senescence-induced receptor-like serine/threonine-protein kinase precursor, putative, expressed
Chr1	34551544	34551544	T	A	T	SNP	OS01G0812800	Intronic	NA	NA	LOC_Os01g59730	ribosomal protein L7Ae, putative, expressed
Chr1	34552559	34552559	C	A	C	SNP	OS01G0812800	Exonic	CDS	Missense	LOC_Os01g59730	ribosomal protein L7Ae, putative, expressed
Chr1	34617235	34617235	G	A	G	SNP	OS01G0813900	Intronic	NA	NA	LOC_Os01g59850	GTPase-activating protein, putative, expressed
Chr1	34617306	34617306	T	A	T	SNP	OS01G0813900	Intronic	NA	NA	LOC_Os01g59850	GTPase-activating protein, putative, expressed
Chr1	34618292	34618292	G	A	G	SNP	OS01G0813900	Exonic	CDS	Silent	LOC_Os01g59850	GTPase-activating protein, putative, expressed
Chr1	34770480	34770480	G	A	G	SNP	OS01G0817000	Intronic	NA	NA	LOC_Os01g60110	E2F-related protein, putative, expressed
Chr1	34771269	34771269	T	A	T	SNP	OS01G0817000	Intronic	NA	NA	LOC_Os01g60110	E2F-related protein, putative, expressed
Chr1	34836761	34836761	G	A	G	SNP	OS01G0818000	Intronic	NA	NA	LOC_Os01g60230	auxin efflux carrier component, putative, expressed
Chr1	34837659	34837659	C	A	C	SNP	OS01G0818000	Intronic	NA	NA	LOC_Os01g60230	auxin efflux carrier component, putative, expressed
Chr1	35231018	35231018	G	A	G	SNP	OS01G0824600	Exonic	CDS	Missense	LOC_Os01g60910	CAMK_KIN1/SNF1/Nim1_like.12 - CAMK includes calcium/calmodulin depedent protein kinases, expressed
Chr1	35231129	35231129	G	A	G	SNP	OS01G0824600	Exonic	CDS	Missense	LOC_Os01g60910	CAMK_KIN1/SNF1/Nim1_like.12 - CAMK includes calcium/calmodulin depedent protein kinases, expressed
Chr1	35231373	35231373	C	A	C	SNP	OS01G0824600	Exonic	CDS	Missense	LOC_Os01g60910	CAMK_KIN1/SNF1/Nim1_like.12 - CAMK includes calcium/calmodulin depedent protein kinases, expressed
Chr1	35234366	35234366	G	A	G	SNP	OS01G0824600	Exonic	3UTR	NA	LOC_Os01g60910	CAMK_KIN1/SNF1/Nim1_like.12 - CAMK includes calcium/calmodulin depedent protein kinases, expressed
Chr1	35332759	35332759	G	A	G	SNP	OS01G0826000	Exonic	CDS	Missense	LOC_Os01g61070	heavy metal-associated domain containing protein, expressed
Chr1	35333372	35333372	G	A	G	SNP	OS01G0826000	Exonic	3UTR	NA	LOC_Os01g61070	heavy metal-associated domain containing protein, expressed
Chr1	35482162	35482162	G	A	G	SNP	OS01G0829100	Exonic	5UTR	NA	LOC_Os01g61330	ankyrin homolog precursor, putative, expressed
Chr1	36083354	36083354	G	A	G	SNP	OS01G0840700	Intronic	NA	NA	LOC_Os01g62350	60S ribosomal protein L36-2, putative, expressed
Chr1	36083830	36083830	G	A	G	SNP	OS01G0840700	Intronic	NA	NA	LOC_Os01g62350	60S ribosomal protein L36-2, putative, expressed
Chr1	36412693	36412693	G	A	G	SNP	OS01G0847700	Intronic	NA	NA	LOC_Os01g62870	oxidoreductase, aldo/keto reductase family protein, putative, expressed
Chr1	36481894	36481894	T	A	T	SNP	OS01G0848900	Intronic	NA	NA	LOC_Os01g62970	expressed protein
Chr1	36482860	36482860	G	A	G	SNP	OS01G0848900	Intronic	NA	NA	LOC_Os01g62970	expressed protein
Chr1	36634606	36634606	T	A	T	SNP	OS01G0850800	Intronic	NA	NA	LOC_Os01g63200	laccase precursor protein, putative, expressed
Chr1	36796866	36796866	G	A	G	SNP	OS01G0854000	Exonic	3UTR	NA	LOC_Os01g63480	transferase family protein, putative, expressed
Chr1	36796877	36796877	G	A	G	SNP	OS01G0854000	Exonic	3UTR	NA	LOC_Os01g63480	transferase family protein, putative, expressed
Chr1	37022923	37022923	G	A	G	SNP	OS01G0856900	Intronic	NA	NA	LOC_Os01g63810	starch binding domain containing protein, putative, expressed
Chr1	37022932	37022932	G	A	G	SNP	OS01G0856900	Intronic	NA	NA	LOC_Os01g63810	starch binding domain containing protein, putative, expressed
Chr1	37023196	37023196	C	A	C	SNP	OS01G0856900	Exonic	CDS	Missense	LOC_Os01g63810	starch binding domain containing protein, putative, expressed
Chr1	37023268	37023268	G	A	G	SNP	OS01G0856900	Exonic	3UTR	NA	LOC_Os01g63810	starch binding domain containing protein, putative, expressed
Chr1	37024318	37024318	G	A	G	SNP	OS01G0857000	Intronic	NA	NA	LOC_Os01g63820	double-stranded RNA binding motif containing protein, expressed
Chr1	37025391	37025391	G	A	G	SNP	OS01G0857000	Intronic	NA	NA	LOC_Os01g63820	double-stranded RNA binding motif containing protein, expressed
Chr1	37027012	37027012	T	A	T	SNP	OS01G0857000	Intronic	NA	NA	LOC_Os01g63820	double-stranded RNA binding motif containing protein, expressed
Chr1	37028482	37028482	G	A	G	SNP	OS01G0857000	Intronic	NA	NA	LOC_Os01g63820	double-stranded RNA binding motif containing protein, expressed
Chr1	37045249	37045249	G	A	G	SNP	OS01G0857400	Intronic	NA	NA	LOC_Os01g63854	transmembrane amino acid transporter protein, putative, expressed
Chr1	37045263	37045263	C	A	C	SNP	OS01G0857400	Intronic	NA	NA	LOC_Os01g63854	transmembrane amino acid transporter protein, putative, expressed
Chr1	37045347	37045347	G	A	G	SNP	OS01G0857400	Intronic	NA	NA	LOC_Os01g63854	transmembrane amino acid transporter protein, putative, expressed
Chr1	37046225	37046225	G	A	G	SNP	OS01G0857400	Intronic	NA	NA	LOC_Os01g63854	transmembrane amino acid transporter protein, putative, expressed
Chr1	37046786	37046786	G	A	G	SNP	OS01G0857400	Intronic	NA	NA	LOC_Os01g63854	transmembrane amino acid transporter protein, putative, expressed
Chr1	37046813	37046813	G	A	G	SNP	OS01G0857400	Intronic	NA	NA	LOC_Os01g63854	transmembrane amino acid transporter protein, putative, expressed
Chr1	37046883	37046883	T	A	T	SNP	OS01G0857400	Intronic	NA	NA	LOC_Os01g63854	transmembrane amino acid transporter protein, putative, expressed
Chr1	37047598	37047598	T	A	T	SNP	OS01G0857400	Intronic	NA	NA	LOC_Os01g63854	transmembrane amino acid transporter protein, putative, expressed
Chr1	37047932	37047932	G	A	G	SNP	OS01G0857400	Intronic	NA	NA	LOC_Os01g63854	transmembrane amino acid transporter protein, putative, expressed
Chr1	37048352	37048352	G	A	G	SNP	OS01G0857400	Intronic	NA	NA	LOC_Os01g63854	transmembrane amino acid transporter protein, putative, expressed
Chr1	37048643	37048643	G	A	G	SNP	OS01G0857400	Intronic	NA	NA	LOC_Os01g63854	transmembrane amino acid transporter protein, putative, expressed
Chr1	37048657	37048657	G	A	G	SNP	OS01G0857400	Intronic	NA	NA	LOC_Os01g63854	transmembrane amino acid transporter protein, putative, expressed
Chr1	37049762	37049762	G	A	G	SNP	OS01G0857400	Exonic	5UTR	NA	LOC_Os01g63854	transmembrane amino acid transporter protein, putative, expressed
Chr1	37440516	37440516	G	A	G	SNP	OS01G0865100	Exonic	3UTR	NA	LOC_Os01g64520	uricase, putative, expressed
Chr1	37440666	37440666	G	A	G	SNP	OS01G0865100	Intronic	NA	NA	LOC_Os01g64520	uricase, putative, expressed
Chr1	37440715	37440715	T	A	T	SNP	OS01G0865100	Exonic	3UTR	NA	LOC_Os01g64520	uricase, putative, expressed
Chr1	37442377	37442377	G	A	G	SNP	OS01G0865100	Exonic	3UTR	NA	LOC_Os01g64520	uricase, putative, expressed
Chr1	37442575	37442575	G	A	G	SNP	OS01G0865100	Exonic	3UTR	NA	LOC_Os01g64520	uricase, putative, expressed
Chr1	37443831	37443831	C	A	C	SNP	OS01G0865100	Intronic	NA	NA	LOC_Os01g64520	uricase, putative, expressed
Chr1	37520118	37520118	G	A	G	SNP	OS01G0866400	Exonic	CDS	Silent	LOC_Os01g64660	fructose-1,6-bisphosphatase, putative, expressed
Chr1	37521835	37521835	C	A	C	SNP	OS01G0866400	Intronic	NA	NA	LOC_Os01g64660	fructose-1,6-bisphosphatase, putative, expressed
Chr1	37522010	37522010	G	A	G	SNP	OS01G0866400	Intronic	NA	NA	LOC_Os01g64660	fructose-1,6-bisphosphatase, putative, expressed
Chr1	37523105	37523105	G	A	G	SNP	OS01G0866500	Intronic	NA	NA	LOC_Os01g64670	soluble inorganic pyrophosphatase, putative, expressed
Chr1	37523244	37523244	G	A	G	SNP	OS01G0866500	Intronic	NA	NA	LOC_Os01g64670	soluble inorganic pyrophosphatase, putative, expressed
Chr1	37523534	37523534	C	A	C	SNP	OS01G0866500	Exonic	CDS	Missense	LOC_Os01g64670	soluble inorganic pyrophosphatase, putative, expressed
Chr1	37557051	37557051	C	A	C	SNP	OS01G0867300	Exonic	3UTR	NA	LOC_Os01g64730	bZIP transcription factor domain containing protein, expressed
Chr1	37665312	37665312	G	A	G	SNP	OS01G0869200	Intronic	NA	NA	LOC_Os01g64890	CorA-like magnesium transporter protein, putative, expressed
Chr1	37697906	37697906	G	A	G	SNP	OS01G0869800	Intronic	NA	NA	LOC_Os01g64960	chlorophyll A-B binding protein, putative, expressed
Chr1	37698531	37698531	C	A	C	SNP	OS01G0869800	Intronic	NA	NA	LOC_Os01g64960	chlorophyll A-B binding protein, putative, expressed
Chr1	37698978	37698978	G	A	G	SNP	OS01G0869800	Exonic	CDS	Silent	LOC_Os01g64960	chlorophyll A-B binding protein, putative, expressed
Chr1	38264668	38264668	G	A	G	SNP	OS01G0881500	Intronic	NA	NA	LOC_Os01g65900	chitin-inducible gibberellin-responsive protein, putative, expressed
Chr1	38264806	38264806	G	A	G	SNP	OS01G0881500	Intronic	NA	NA	LOC_Os01g65900	chitin-inducible gibberellin-responsive protein, putative, expressed
Chr1	38400779	38400779	C	A	C	SNP	OS01G0884300	Intronic	NA	NA	LOC_Os01g66120	No apical meristem protein, putative, expressed
Chr1	39550086	39550086	G	A	G	SNP	OS01G0908400	Intronic	NA	NA	LOC_Os01g68030	expressed protein
Chr1	39567761	39567761	G	A	G	SNP	OS01G0908700	Intronic	NA	NA	LOC_Os01g68060	copine, putative, expressed
Chr1	39568046	39568046	T	A	T	SNP	OS01G0908700	Intronic	NA	NA	LOC_Os01g68060	copine, putative, expressed
Chr1	39688819	39688819	C	A	C	SNP	OS01G0911100	Intronic	NA	NA	LOC_Os01g68320	DEAD-box ATP-dependent RNA helicase 30, putative, expressed

Chr1	39691263	39691263	G	A	G	SNP	OS01G0911100	Intronic	NA	NA	LOC_Os01g68320	DEAD-box ATP-dependent RNA helicase 30, putative, expressed
Chr1	39779311	39779311	C	A	C	SNP	OS01G0912700	Exonic	CDS	Missense	LOC_Os01g68450	expressed protein
Chr1	39954679	39954679	C	A	C	SNP	OS01G0916600	Intronic	NA	NA	LOC_Os01g68790	RNA recognition motif containing protein, putative, expressed
Chr1	40994602	40994602	C	A	C	SNP	OS01G0934350	Exonic	CDS	NA	LOC_Os01g70820	lumenal PsbP, putative, expressed
Chr1	41079497	41079497	C	A	C	SNP	OS01G0935800	Exonic	3UTR	NA	LOC_Os01g70970	protein kinase, putative, expressed
Chr1	41097375	41097375	G	A	G	SNP	OS01G0936100	Exonic	CDS	Silent	LOC_Os01g71000	protein kinase APK1B, chloroplast precursor, putative, expressed
Chr1	41098899	41098899	C	A	C	SNP	OS01G0936100	Intronic	NA	NA	LOC_Os01g71000	protein kinase APK1B, chloroplast precursor, putative, expressed
Chr1	41188404	41188404	G	A	G	SNP	OS01G0938100	Exonic	CDS	Missense	LOC_Os01g71190	photosystem II reaction center PSB28 protein, chloroplast precursor, putative, expressed
Chr1	42066223	42066223	T	A	T	SNP	OS01G0955000	Intronic	NA	NA	LOC_Os01g72520	phosphoesterase family protein, putative, expressed
Chr1	262573	262573	T	A	T	SNP	OS01G0104800	Intronic	NA	NA	LOC_Os01g01510	sas10/Utp3 family protein, expressed
Chr1	744368	744368	T	A	T	SNP	OS01G0113700	Intronic	NA	NA	LOC_Os01g02350	receptor-like kinase ARK1AS, putative, expressed
Chr1	1179285	1179285	G	A	G	SNP	OS01G0120800	Exonic	5UTR	NA	LOC_Os01g03070	transposon protein, putative, unclassified, expressed
Chr1	1203343	1203343	G	A	G	SNP	OS01G0121300	Exonic	CDS	Missense	LOC_Os01g03110	expressed protein
Chr1	1217645	1217645	C	A	C	SNP	OS01G0121500	Exonic	CDS	NA	LOC_Os01g03130	expressed protein
Chr1	1903530	1903530	G	A	G	SNP	OS01G0134700	Exonic	CDS	Missense	LOC_Os01g04280	calmodulin binding protein, putative, expressed
Chr1	3804678	3804678	G	A	G	SNP	OS01G0173900	Exonic	5UTR	NA	LOC_Os01g07870	ABC transporter family protein, putative, expressed
Chr1	3805144	3805144	G	A	G	SNP	OS01G0173900	Exonic	CDS	Silent	LOC_Os01g07870	ABC transporter family protein, putative, expressed
Chr1	3805309	3805309	C	A	C	SNP	OS01G0173900	Exonic	CDS	Silent	LOC_Os01g07870	ABC transporter family protein, putative, expressed
Chr1	3820261	3820261	T	A	T	SNP	OS01G0174300	Exonic	3UTR	NA	LOC_Os01g07910	NADH-cytochrome b5 reductase, putative, expressed
Chr1	3823568	3823568	G	A	G	SNP	OS01G0174300	Intronic	NA	NA	LOC_Os01g07910	NADH-cytochrome b5 reductase, putative, expressed
Chr1	3824234	3824234	G	A	G	SNP	OS01G0174300	Exonic	CDS	Missense	LOC_Os01g07910	NADH-cytochrome b5 reductase, putative, expressed
Chr1	3824472	3824472	G	A	G	SNP	OS01G0174300	Exonic	5UTR	NA	LOC_Os01g07910	NADH-cytochrome b5 reductase, putative, expressed
Chr1	3918287	3918287	G	A	G	SNP	OS01G0176000	Exonic	CDS	Missense	LOC_Os01g08090	flavonol-3-O-glycoside-7-O-glucosyltransferase 1, putative, expressed
Chr1	3941956	3941956	G	A	G	SNP	OS01G0176500	Exonic	CDS	Missense	LOC_Os01g08150	expressed protein
Chr1	3941964	3941964	G	A	G	SNP	OS01G0176500	Exonic	CDS	Missense	LOC_Os01g08150	expressed protein
Chr1	5508367	5508367	G	A	G	SNP	OS01G0201100	Intronic	NA	NA	LOC_Os01g10440	xylosyltransferase, putative, expressed
Chr1	5876399	5876399	C	A	C	SNP	OS01G0208400	Exonic	CDS	Missense	LOC_Os01g11010	peptide-N4-asparagine amidase A, putative, expressed
Chr1	5876595	5876595	G	A	G	SNP	OS01G0208400	Exonic	CDS	Silent	LOC_Os01g11010	peptide-N4-asparagine amidase A, putative, expressed
Chr1	5900149	5900149	G	A	G	SNP	OS01G0208700	Exonic	CDS	Missense	LOC_Os01g11054	phosphoenolpyruvate carboxylase, putative, expressed
Chr1	5901772	5901772	G	A	G	SNP	OS01G0208700	Intronic	NA	NA	LOC_Os01g11054	phosphoenolpyruvate carboxylase, putative, expressed
Chr1	5902567	5902567	G	A	G	SNP	OS01G0208700	Intronic	NA	NA	LOC_Os01g11054	phosphoenolpyruvate carboxylase, putative, expressed
Chr1	5902874	5902874	G	A	G	SNP	OS01G0208700	Intronic	NA	NA	LOC_Os01g11054	phosphoenolpyruvate carboxylase, putative, expressed
Chr1	5902991	5902991	C	A	C	SNP	OS01G0208700	Intronic	NA	NA	LOC_Os01g11054	phosphoenolpyruvate carboxylase, putative, expressed
Chr1	5903083	5903083	G	A	G	SNP	OS01G0208700	Intronic	NA	NA	LOC_Os01g11054	phosphoenolpyruvate carboxylase, putative, expressed
Chr1	5903122	5903122	G	A	G	SNP	OS01G0208700	Intronic	NA	NA	LOC_Os01g11054	phosphoenolpyruvate carboxylase, putative, expressed
Chr1	5903544	5903544	G	A	G	SNP	OS01G0208700	Intronic	NA	NA	LOC_Os01g11054	phosphoenolpyruvate carboxylase, putative, expressed
Chr1	5903681	5903681	G	A	G	SNP	OS01G0208700	Intronic	NA	NA	LOC_Os01g11054	phosphoenolpyruvate carboxylase, putative, expressed
Chr1	5904959	5904959	G	A	G	SNP	OS01G0208700	Intronic	NA	NA	LOC_Os01g11054	phosphoenolpyruvate carboxylase, putative, expressed
Chr1	5905170	5905170	G	A	G	SNP	OS01G0208700	Intronic	NA	NA	LOC_Os01g11054	phosphoenolpyruvate carboxylase, putative, expressed
Chr1	5905962	5905962	G	A	G	SNP	OS01G0208700	Intronic	NA	NA	LOC_Os01g11054	phosphoenolpyruvate carboxylase, putative, expressed
Chr1	5906173	5906173	G	A	G	SNP	OS01G0208700	Intronic	NA	NA	LOC_Os01g11054	phosphoenolpyruvate carboxylase, putative, expressed
Chr1	5983898	5983898	G	A	G	SNP	OS01G0209800	Exonic	CDS	Silent	LOC_Os01g11160	amino acid permease family protein, putative, expressed
Chr1	6769399	6769399	C	A	C	SNP	OS01G0223600	Exonic	CDS	NA	LOC_Os01g12390	inactive receptor kinase At2g26730 precursor, putative, expressed
Chr1	7364385	7364385	G	A	G	SNP	OS01G0233000	Exonic	CDS	Missense	LOC_Os01g13210	salt stress root protein RS1, putative, expressed
Chr1	7364450	7364450	G	A	G	SNP	OS01G0233000	Exonic	CDS	Silent	LOC_Os01g13210	salt stress root protein RS1, putative, expressed
Chr1	7547231	7547231	G	A	G	SNP	OS01G0236300	Exonic	CDS	Silent	LOC_Os01g13520	auxin response factor 1, putative, expressed
Chr1	7557754	7557754	G	A	G	SNP	OS01G0236400	Exonic	CDS	Missense	LOC_Os01g13530	ABIL2, putative, expressed
Chr1	7865105	7865105	T	A	T	SNP	OS01G0242600	Intronic	NA	NA	LOC_Os01g14050	C2 domain containing protein, putative, expressed
Chr1	8206032	8206032	G	A	G	SNP	OS01G0249200	Intronic	NA	NA	LOC_Os01g14670	Cupin domain containing protein, expressed
Chr1	8206036	8206036	T	A	T	SNP	OS01G0249200	Intronic	NA	NA	LOC_Os01g14670	Cupin domain containing protein, expressed
Chr1	8404682	8404682	G	A	G	SNP	OS01G0253900	Exonic	CDS	Silent	LOC_Os01g15000	lipase, putative, expressed
Chr1	8404748	8404748	C	A	C	SNP	OS01G0253900	Exonic	CDS	Silent	LOC_Os01g15000	lipase, putative, expressed
Chr1	8546147	8546147	T	A	T	SNP	OS01G0256500	Exonic	3UTR	NA	LOC_Os01g15270	expressed protein
Chr1	8546154	8546154	C	A	C	SNP	OS01G0256500	Exonic	3UTR	NA	LOC_Os01g15270	expressed protein
Chr1	8546650	8546650	C	A	C	SNP	OS01G0256500	Exonic	CDS	Silent	LOC_Os01g15270	expressed protein
Chr1	9860292	9860292	C	A	C	SNP	OS01G0278900	Exonic	CDS	Missense	LOC_Os01g17150	plastid-specific 50S ribosomal protein 5, chloroplast precursor, putative, expressed
Chr1	10009031	10009031	G	A	G	SNP	OS01G0281100	Intronic	NA	NA	LOC_Os01g17396	expressed protein
Chr1	10009827	10009827	G	A	G	SNP	OS01G0281100	Intronic	NA	NA	LOC_Os01g17396	expressed protein
Chr1	10201369	10201369	T	A	T	SNP	OS01G0284900	Intronic	NA	NA	LOC_Os01g18220	expressed protein
Chr1	10342531	10342531	G	A	G	SNP	OS01G0287600	Exonic	CDS	Silent	LOC_Os01g18400	CHIT10 - Chitinase family protein precursor, expressed
Chr1	10342630	10342630	G	A	G	SNP	OS01G0287600	Intronic	NA	NA	LOC_Os01g18400	CHIT10 - Chitinase family protein precursor, expressed
Chr1	10342804	10342804	C	A	C	SNP	OS01G0287600	Intronic	NA	NA	LOC_Os01g18400	CHIT10 - Chitinase family protein precursor, expressed
Chr1	10343174	10343174	C	A	C	SNP	OS01G0287600	Exonic	CDS	Silent	LOC_Os01g18400	CHIT10 - Chitinase family protein precursor, expressed
Chr1	10343535	10343535	G	A	G	SNP	OS01G0287600	Exonic	CDS	Missense	LOC_Os01g18400	CHIT10 - Chitinase family protein precursor, expressed
Chr1	10494961	10494961	C	A	C	SNP	OS01G0290000	Exonic	CDS	Missense	LOC_Os01g18630	aspartic proteinase oryzasin-1 precursor, putative, expressed
Chr1	10654586	10654586	G	A	G	SNP	OS01G0292900	Exonic	CDS	Missense	LOC_Os01g18850	OsSPL1 - SBP-box gene family member, expressed
Chr1	15569474	15569474	G	A	G	SNP	OS01G0376700	Intronic	NA	NA	LOC_Os01g27880	sucrose-phosphatase, putative, expressed
Chr1	21424072	21424072	T	A	T	SNP	OS01G0563000	Intronic	NA	NA	LOC_Os01g38229	peptidyl-prolyl isomerase, putative, expressed
Chr1	21424118	21424118	G	A	G	SNP	OS01G0563000	Intronic	NA	NA	LOC_Os01g38229	peptidyl-prolyl isomerase, putative, expressed
Chr1	21425696	21425696	G	A	G	SNP	OS01G0563000	Exonic	CDS	Silent	LOC_Os01g38229	peptidyl-prolyl isomerase, putative, expressed
Chr1	21426088	21426088	T	A	T	SNP	OS01G0563000	Exonic	CDS	Missense	LOC_Os01g38229	peptidyl-prolyl isomerase, putative, expressed
Chr1	21426191	21426191	G	A	G	SNP	OS01G0563000	Exonic	CDS	Silent	LOC_Os01g38229	peptidyl-prolyl isomerase, putative, expressed
Chr1	21426218	21426218	C	A	C	SNP	OS01G0563000	Intronic	NA	NA	LOC_Os01g38229	peptidyl-prolyl isomerase, putative, expressed
Chr1	21426263	21426263	G	A	G	SNP	OS01G0563000	Intronic	NA	NA	LOC_Os01g38229	peptidyl-prolyl isomerase, putative, expressed
Chr1	21428414	21428414	T	A	T	SNP	OS01G0563000	Intronic	NA	NA	LOC_Os01g38229	peptidyl-prolyl isomerase, putative, expressed
Chr1	21431665	21431665	G	A	G	SNP	OS01G0563000	Exonic	CDS	Missense	LOC_Os01g38229	peptidyl-prolyl isomerase, putative, expressed
Chr1	21431677	21431677	G	A	G	SNP	OS01G0563000	Exonic	CDS	Missense	LOC_Os01g38229	peptidyl-prolyl isomerase, putative, expressed
Chr1	21433297	21433297	T	A	T	SNP	OS01G0563000	Exonic	CDS	Missense	LOC_Os01g38229	peptidyl-prolyl isomerase, putative, expressed
Chr1	21441336	21441336	T	A	T	SNP	OS01G0563000	Exonic	3UTR	NA	LOC_Os01g38229	peptidyl-prolyl isomerase, putative, expressed
Chr1	21441361	21441361	G	A	G	SNP	OS01G0563000	Exonic	3UTR	NA	LOC_Os01g38229	peptidyl-prolyl isomerase, putative, expressed
Chr1	21631321	21631321	G	A	G	SNP	OS01G0565900	Exonic	5UTR	NA	LOC_Os01g38510	protein transport protein Sec61 subunit beta, putative, expressed
Chr1	22495060	22495060	G	A	G	SNP	OS01G0580800	Exonic	CDS	Silent	LOC_Os01g39890	transposon protein, putative, unclassified, expressed
Chr1	22496113	22496113	G	A	G	SNP	OS01G0580800	Intronic	NA	NA	LOC_Os01g39890	transposon protein, putative, unclassified, expressed
Chr1	22496823	22496823	T	A	T	SNP	OS01G0580800	Intronic	NA	NA	LOC_Os01g39890	transposon protein, putative, unclassified, expressed
Chr1	22497648	22497648	G	A	G	SNP	OS01G0580800	Intronic	NA	NA	LOC_Os01g39890	transposon protein, putative, unclassified, expressed

Chr1	22499072	22499072	G	A	G	SNP	OS01G0580800	Exonic	CDS	Missense	LOC_Os01g39890	transposon protein, putative, unclassified, expressed
Chr1	22754722	22754722	G	A	G	SNP	OS01G0585300	Exonic	CDS	Silent	LOC_Os01g40310	expressed protein
Chr1	22754740	22754740	G	A	G	SNP	OS01G0585300	Exonic	CDS	Silent	LOC_Os01g40310	expressed protein
Chr1	23675778	23675778	G	A	G	SNP	OS01G0602400	Exonic	CDS	Missense	LOC_Os01g41810	cytochrome P450 72A1, putative, expressed
Chr1	23912461	23912461	C	A	C	SNP	OS01G0606900	Intronic	NA	NA	LOC_Os01g42190	heat shock protein DnaJ, putative, expressed
Chr1	24113500	24113500	G	A	G	SNP	OS01G0609900	Intronic	NA	NA	LOC_Os01g42410	pleiotropic drug resistance protein, putative, expressed
Chr1	24114399	24114399	G	A	G	SNP	OS01G0609900	Intronic	NA	NA	LOC_Os01g42410	pleiotropic drug resistance protein, putative, expressed
Chr1	24114550	24114550	G	A	G	SNP	OS01G0609900	Intronic	NA	NA	LOC_Os01g42410	pleiotropic drug resistance protein, putative, expressed
Chr1	24581680	24581680	G	A	G	SNP	OS01G0618100	Exonic	CDS	Silent	LOC_Os01g43090	oxidoreductase, aldo/keto reductase family protein, putative, expressed
Chr1	25234782	25234782	C	A	C	SNP	OS01G0631200	Exonic	CDS	NA	LOC_Os01g44050	siroheme synthase, putative, expressed
Chr1	25682256	25682256	G	A	G	SNP	OS01G0639600	Exonic	CDS	Missense	LOC_Os01g45250	DUF1645 domain containing protein, putative, expressed
Chr1	26795775	26795775	G	A	G	SNP	OS01G0658700	Exonic	5UTR	NA	LOC_Os01g46950	aldose 1-epimerase, putative, expressed
Chr1	26803625	26803625	G	A	G	SNP	OS01G0658900	Intronic	NA	NA	LOC_Os01g46970	transcription factor, putative, expressed
Chr1	26805213	26805213	G	A	G	SNP	OS01G0658900	Intronic	NA	NA	LOC_Os01g46970	transcription factor, putative, expressed
Chr1	26805355	26805355	G	A	G	SNP	OS01G0658900	Intronic	NA	NA	LOC_Os01g46970	transcription factor, putative, expressed
Chr1	26805356	26805356	C	A	C	SNP	OS01G0658900	Intronic	NA	NA	LOC_Os01g46970	transcription factor, putative, expressed
Chr1	26895505	26895505	G	A	G	SNP	OS01G0660300	Intronic	NA	NA	LOC_Os01g47080	pyruvate kinase, putative, expressed
Chr1	26895684	26895684	G	A	G	SNP	OS01G0660300	Intronic	NA	NA	LOC_Os01g47080	pyruvate kinase, putative, expressed
Chr1	27171847	27171847	G	A	G	SNP	OS01G0665200	Exonic	5UTR	NA	LOC_Os01g47530	CGMC_MAPKCMGC_2.6 - CGMC includes CDA, MAPK, GSK3, and CLKC kinases, expressed
Chr1	27172330	27172330	G	A	G	SNP	OS01G0665200	Intronic	NA	NA	LOC_Os01g47530	CGMC_MAPKCMGC_2.6 - CGMC includes CDA, MAPK, GSK3, and CLKC kinases, expressed
Chr1	27183083	27183083	G	A	G	SNP	OS01G0665300	Intronic	NA	NA	LOC_Os01g47540	NLI interacting factor-like phosphatase, putative, expressed
Chr1	27341193	27341193	G	A	G	SNP	OS01G0668100	Exonic	CDS	Silent	LOC_Os01g47780	fasciclin domain containing protein, expressed
Chr1	27341449	27341449	T	A	T	SNP	OS01G0668100	Exonic	CDS	Missense	LOC_Os01g47780	fasciclin domain containing protein, expressed
Chr1	27341565	27341565	G	A	G	SNP	OS01G0668100	Exonic	CDS	Silent	LOC_Os01g47780	fasciclin domain containing protein, expressed
Chr1	28573807	28573807	C	A	C	SNP	OS01G0692300	Exonic	CDS	Missense	LOC_Os01g49740	expressed protein
Chr1	28848791	28848791	G	A	G	SNP	OS01G0697100	Exonic	CDS	Missense	LOC_Os01g50200	UDP-glucoronosyl and UDP-glucosyl transferase domain containing protein, expressed
Chr1	28849195	28849195	C	A	C	SNP	OS01G0697100	Exonic	CDS	Silent	LOC_Os01g50200	UDP-glucoronosyl and UDP-glucosyl transferase domain containing protein, expressed
Chr1	28849352	28849352	G	A	G	SNP	OS01G0697100	Exonic	CDS	Missense	LOC_Os01g50200	UDP-glucoronosyl and UDP-glucosyl transferase domain containing protein, expressed
Chr1	29488546	29488546	G	A	G	SNP	OS01G0709500	Exonic	CDS	Missense	LOC_Os01g51290	protein kinase family protein, putative, expressed
Chr1	29491515	29491515	G	A	G	SNP	OS01G0709500	Exonic	5UTR	NA	LOC_Os01g51290	protein kinase family protein, putative, expressed
Chr1	29846614	29846614	T	A	T	SNP	OS01G0717000	Exonic	5UTR	NA	LOC_Os01g51920	phosphotransferase, putative, expressed
Chr1	29895677	29895677	G	A	G	SNP	OS01G0717700	Intronic	NA	NA	LOC_Os01g52010	alliin lyase precursor, putative, expressed
Chr1	29896314	29896314	G	A	G	SNP	OS01G0717700	Intronic	NA	NA	LOC_Os01g52010	alliin lyase precursor, putative, expressed
Chr1	29961325	29961325	G	A	G	SNP	OS01G0719000	Intronic	NA	NA	LOC_Os01g52100	DUF581 domain containing protein, expressed
Chr1	30026201	30026201	C	A	C	SNP	OS01G0720400	Exonic	CDS	Missense	LOC_Os01g52230	phosphoethanolamine/phosphocholine phosphatase, putative, expressed
Chr1	31099678	31099678	G	A	G	SNP	OS01G0744000	Intronic	NA	NA	LOC_Os01g54080	kinesin motor protein-related, putative, expressed
Chr1	31102752	31102752	G	A	G	SNP	OS01G0744000	Exonic	CDS	Missense	LOC_Os01g54080	kinesin motor protein-related, putative, expressed
Chr1	31272955	31272955	G	A	G	SNP	OS01G0747300	Exonic	CDS	NA	LOC_Os01g54340	plant-specific domain TIGR01615 family protein, expressed
Chr1	31766547	31766547	C	A	C	SNP	OS01G0756900	Exonic	CDS	NA	LOC_Os01g55220	expressed protein
Chr1	32002683	32002683	C	A	C	SNP	OS01G0760600	Intronic	NA	NA	LOC_Os01g55540	aminotransferase, classes I and II, domain containing protein, expressed
Chr1	33189020	33189020	G	A	G	SNP	OS01G0783200	Intronic	NA	NA	LOC_Os01g57420	diacylglycerol kinase, putative, expressed
Chr1	33410227	33410227	T	A	T	SNP	OS01G0787600	Intronic	NA	NA	LOC_Os01g57770	OsPOP4 - Putative Prolyl Oligopeptidase homologue, expressed
Chr1	33410229	33410229	T	A	T	SNP	OS01G0787600	Intronic	NA	NA	LOC_Os01g57770	OsPOP4 - Putative Prolyl Oligopeptidase homologue, expressed
Chr1	33410241	33410241	T	A	T	SNP	OS01G0787600	Intronic	NA	NA	LOC_Os01g57770	OsPOP4 - Putative Prolyl Oligopeptidase homologue, expressed
Chr1	33410280	33410280	C	A	C	SNP	OS01G0787600	Intronic	NA	NA	LOC_Os01g57770	OsPOP4 - Putative Prolyl Oligopeptidase homologue, expressed
Chr1	33410370	33410370	C	A	C	SNP	OS01G0787600	Intronic	NA	NA	LOC_Os01g57770	OsPOP4 - Putative Prolyl Oligopeptidase homologue, expressed
Chr1	33410583	33410583	G	A	G	SNP	OS01G0787600	Intronic	NA	NA	LOC_Os01g57770	OsPOP4 - Putative Prolyl Oligopeptidase homologue, expressed
Chr1	33410709	33410709	T	A	T	SNP	OS01G0787600	Intronic	NA	NA	LOC_Os01g57770	OsPOP4 - Putative Prolyl Oligopeptidase homologue, expressed
Chr1	33412259	33412259	G	A	G	SNP	OS01G0787600	Exonic	5UTR	NA	LOC_Os01g57770	OsPOP4 - Putative Prolyl Oligopeptidase homologue, expressed
Chr1	33412313	33412313	G	A	G	SNP	OS01G0787600	Exonic	5UTR	NA	LOC_Os01g57770	OsPOP4 - Putative Prolyl Oligopeptidase homologue, expressed
Chr1	33414685	33414685	G	A	G	SNP	OS01G0787600	Exonic	5UTR	NA	LOC_Os01g57770	OsPOP4 - Putative Prolyl Oligopeptidase homologue, expressed
Chr1	33414801	33414801	G	A	G	SNP	OS01G0787600	Exonic	5UTR	NA	LOC_Os01g57770	OsPOP4 - Putative Prolyl Oligopeptidase homologue, expressed
Chr1	33837482	33837482	T	A	T	SNP	OS01G0799500	Exonic	5UTR	NA	LOC_Os01g58550	methyladenine glycosylase, putative, expressed
Chr1	33837688	33837688	T	A	T	SNP	OS01G0799500	Exonic	CDS	Missense	LOC_Os01g58550	methyladenine glycosylase, putative, expressed
Chr1	33839333	33839333	C	A	C	SNP	OS01G0799500	Intronic	NA	NA	LOC_Os01g58550	methyladenine glycosylase, putative, expressed
Chr1	33899491	33899491	T	A	T	SNP	OS01G0800500	Intronic	NA	NA	LOC_Os01g58640	nucleotide pyrophosphatase/phosphodiesterase, putative, expressed
Chr1	34132726	34132726	G	A	G	SNP	OS01G0804900	Exonic	CDS	Missense	LOC_Os01g59050	cytochrome P450, putative, expressed
Chr1	34143931	34143931	G	A	G	SNP	OS01G0805400	Intronic	NA	NA	LOC_Os01g59100	cytokinin-N-glucosyltransferase 1, putative, expressed
Chr1	34445752	34445752	C	A	C	SNP	OS01G0810800	Exonic	3UTR	NA	LOC_Os01g59570	senescence-induced receptor-like serine/threonine-protein kinase precursor, putative, expressed
Chr1	34446382	34446382	G	A	G	SNP	OS01G0810800	Exonic	CDS	Silent	LOC_Os01g59570	senescence-induced receptor-like serine/threonine-protein kinase precursor, putative, expressed
Chr1	34446809	34446809	C	A	C	SNP	OS01G0810800	Intronic	NA	NA	LOC_Os01g59570	senescence-induced receptor-like serine/threonine-protein kinase precursor, putative, expressed
Chr1	34446818	34446818	G	A	G	SNP	OS01G0810800	Intronic	NA	NA	LOC_Os01g59570	senescence-induced receptor-like serine/threonine-protein kinase precursor, putative, expressed
Chr1	34447629	34447629	C	A	C	SNP	OS01G0810800	Intronic	NA	NA	LOC_Os01g59570	senescence-induced receptor-like serine/threonine-protein kinase precursor, putative, expressed
Chr1	34447661	34447661	T	A	T	SNP	OS01G0810800	Intronic	NA	NA	LOC_Os01g59570	senescence-induced receptor-like serine/threonine-protein kinase precursor, putative, expressed
Chr1	34448287	34448287	T	A	T	SNP	OS01G0810800	Intronic	NA	NA	LOC_Os01g59570	senescence-induced receptor-like serine/threonine-protein kinase precursor, putative, expressed
Chr1	34448326	34448326	C	A	C	SNP	OS01G0810800	Intronic	NA	NA	LOC_Os01g59570	senescence-induced receptor-like serine/threonine-protein kinase precursor, putative, expressed
Chr1	34452089	34452089	G	A	G	SNP	OS01G0810800	Exonic	CDS	Silent	LOC_Os01g59570	senescence-induced receptor-like serine/threonine-protein kinase precursor, putative, expressed
Chr1	34453577	34453577	G	A	G	SNP	OS01G0810800	Exonic	CDS	Missense	LOC_Os01g59570	senescence-induced receptor-like serine/threonine-protein kinase precursor, putative, expressed
Chr1	34454193	34454193	G	A	G	SNP	OS01G0810800	Intronic	NA	NA	LOC_Os01g59570	senescence-induced receptor-like serine/threonine-protein kinase precursor, putative, expressed
Chr1	34454242	34454242	G	A	G	SNP	OS01G0810800	Intronic	NA	NA	LOC_Os01g59570	senescence-induced receptor-like serine/threonine-protein kinase precursor, putative, expressed
Chr1	35084680	35084680	G	A	G	SNP	OS01G0821900	Exonic	CDS	NA	LOC_Os01g60670	receptor-like protein kinase precursor, putative, expressed
Chr1	35481720	35481720	G	A	G	SNP	OS01G0829100	Exonic	CDS	Silent	LOC_Os01g61330	ankyrin homolog precursor, putative, expressed
Chr1	37022833	37022833	G	A	G	SNP	OS01G0856900	Intronic	NA	NA	LOC_Os01g63810	starch binding domain containing protein, putative, expressed
Chr1	37043956	37043956	G	A	G	SNP	OS01G0857400	Exonic	CDS	Silent	LOC_Os01g63854	transmembrane amino acid transporter protein, putative, expressed
Chr1	37044139	37044139	C	A	C	SNP	OS01G0857400	Exonic	CDS	Silent	LOC_Os01g63854	transmembrane amino acid transporter protein, putative, expressed
Chr1	37045305	37045305	G	A	G	SNP	OS01G0857400	Intronic	NA	NA	LOC_Os01g63854	transmembrane amino acid transporter protein, putative, expressed
Chr1	37045312	37045312	G	A	G	SNP	OS01G0857400	Intronic	NA	NA	LOC_Os01g63854	transmembrane amino acid transporter protein, putative, expressed
Chr1	37048153	37048153	G	A	G	SNP	OS01G0857400	Intronic	NA	NA	LOC_Os01g63854	transmembrane amino acid transporter protein, putative, expressed
Chr1	37048342	37048342	G	A	G	SNP	OS01G0857400	Intronic	NA	NA	LOC_Os01g63854	transmembrane amino acid transporter protein, putative, expressed
Chr1	37358640	37358640	G	A	G	SNP	OS01G0863300	Intronic	NA	NA	LOC_Os01g64360	MYB family transcription factor, putative, expressed
Chr1	37557341	37557341	T	A	T	SNP	OS01G0867300	Intronic	NA	NA	LOC_Os01g64730	bZIP transcription factor domain containing protein, expressed
Chr1	37825692	37825692	C	A	C	SNP	OS01G0872100	Exonic	CDS	Silent	LOC_Os01g65169	proton-dependent oligopeptide transport, putative, expressed
Chr1	37826127	37826127	C	A	C	SNP	OS01G0872100	Exonic	CDS	Missense	LOC_Os01g65169	proton-dependent oligopeptide transport, putative, expressed
Chr1	37826781	37826781	T	A	T	SNP	OS01G0872100	Exonic	3UTR	NA	LOC_Os01g65169	proton-dependent oligopeptide transport, putative, expressed
Chr1	37826783	37826783	T	A	T	SNP	OS01G0872100	Exonic	3UTR	NA	LOC_Os01g65169	proton-dependent oligopeptide transport, putative, expressed

Chr1	37826785	37826785	T	A	T	SNP	OS01G0872100	Exonic	3UTR	NA	LOC_Os01g65169	proton-dependent oligopeptide transport, putative, expressed
Chr1	38483419	38483419	G	A	G	SNP	OS01G0885700	Exonic	CDS	Missense	LOC_Os01g66250	S-locus-like receptor protein kinase, putative, expressed
Chr1	39226048	39226048	T	A	T	SNP	OS01G0901000	Intronic	NA	NA	LOC_Os01g67500	armadillo/beta-catenin repeat family protein, putative, expressed
Chr1	39867365	39867365	G	A	G	SNP	OS01G0915000	Exonic	CDS	Silent	LOC_Os01g68650	plant-specific domain TIGR01615 family protein, expressed
Chr1	39926527	39926527	G	A	G	SNP	OS01G0916100	Exonic	CDS	NA	LOC_Os01g68740	keratin, type I cytoskeletal 9, putative, expressed
Chr1	41582435	41582435	G	A	G	SNP	OS01G0946100	Exonic	CDS	Missense	LOC_Os01g71780	WD domain, G-beta repeat domain containing protein, expressed
Chr1	41582701	41582701	G	A	G	SNP	OS01G0946100	Exonic	CDS	Silent	LOC_Os01g71780	WD domain, G-beta repeat domain containing protein, expressed
Chr1	41583302	41583302	G	A	G	SNP	OS01G0946100	Exonic	CDS	Missense	LOC_Os01g71780	WD domain, G-beta repeat domain containing protein, expressed
Chr1	42064340	42064340	G	A	G	SNP	OS01G0955000	Exonic	CDS	Silent	LOC_Os01g72520	phosphoesterase family protein, putative, expressed
Chr1	42065892	42065892	C	A	C	SNP	OS01G0955000	Intronic	NA	NA	LOC_Os01g72520	phosphoesterase family protein, putative, expressed
Chr1	42592829	42592829	G	A	G	SNP	OS01G0966000	Exonic	3UTR	NA	LOC_Os01g73514	5-oxoprolinase, putative, expressed
Chr1	42979643	42979643	G	A	G	SNP	OS01G0973400	Exonic	CDS	Silent	LOC_Os01g74190	radical SAM enzyme, putative, expressed
Chr1	43117102	43117102	G	A	G	SNP	OS01G0975900	Exonic	CDS	Silent	LOC_Os01g74450	aquaporin protein, putative, expressed
Chr1	43117172	43117172	T	A	T	SNP	OS01G0975900	Exonic	5UTR	NA	LOC_Os01g74450	aquaporin protein, putative, expressed
Chr1	262560	262561	AT	A	AT	INDEL	OS01G0104800	Intronic	NA	NA	LOC_Os01g01510	sas10/Utp3 family protein, expressed
Chr1	1567217	1567219	AGC	A	AGC	INDEL	OS01G0128200	Intronic	NA	NA	LOC_Os01g03730	nuclease PA3, putative, expressed
Chr1	8206719	8206723	ATATG	A	ATATG	INDEL	OS01G0249200	Intronic	NA	NA	LOC_Os01g14670	Cupin domain containing protein, expressed
Chr1	8426637	8426661	ATATATGTCTAGATTCATTTACATC	A	ATATATGTCTAGATTCATTTACATC	INDEL	OS01G0254100	Intronic	NA	NA	LOC_Os01g15020	lissencephaly type-1-like homology motif, putative, expressed
Chr1	10203135	10203138	ATCC	A	ATCC	INDEL	OS01G0284900	Exonic	CDS	InFrame	LOC_Os01g18220	expressed protein
Chr1	10342956	10342957	AC	A	AC	INDEL	OS01G0287600	Intronic	NA	NA	LOC_Os01g18400	CHIT10 - Chitinase family protein precursor, expressed
Chr1	21415636	21415643	AGAGTAAT	A	AGAGTAAT	INDEL	OS01G0563000	Intronic	NA	NA	LOC_Os01g38229	peptidyl-prolyl isomerase, putative, expressed
Chr1	21631202	21631203	AT	A	AT	INDEL	OS01G0565900	Exonic	5UTR	NA	LOC_Os01g38510	protein transport protein Sec61 subunit beta, putative, expressed
Chr1	22496616	22496617	AT	A	AT	INDEL	OS01G0580800	Intronic	NA	NA	LOC_Os01g39890	transposon protein, putative, unclassified, expressed
Chr1	22496965	22496966	AT	A	AT	INDEL	OS01G0580800	Intronic	NA	NA	LOC_Os01g39890	transposon protein, putative, unclassified, expressed
Chr1	26802570	26802575	AAAAAT	A	AAAAAT	INDEL	OS01G0658900	Intronic	NA	NA	LOC_Os01g46970	transcription factor, putative, expressed
Chr1	27326301	27326304	AAGG	A	AAGG	INDEL	OS01G0667900	Exonic	3UTR	NA	LOC_Os01g47760	OsGrx_I1 - glutaredoxin subgroup III, expressed
Chr1	29846699	29846701	AAT	A	AAT	INDEL	OS01G0717000	Exonic	5UTR	NA	LOC_Os01g51920	phosphotransferase, putative, expressed
Chr1	29896284	29896285	AT	A	AT	INDEL	OS01G0717700	Intronic	NA	NA	LOC_Os01g52010	alliin lyase precursor, putative, expressed
Chr1	30180665	30180672	ACCCCCCC	A	ACCCCCCC	INDEL	OS01G0723600	Exonic	5UTR	NA	LOC_Os01g52530	phosphoribosyl transferase, putative, expressed
Chr1	31273474	31273498	ACCCGTCACTGTCTCTATTATTTCT	A	ACCCGTCACTGTCTCTATTATTTCT	INDEL	OS01G0747300	Exonic	3UTR	NA	LOC_Os01g54340	plant-specific domain TIGR01615 family protein, expressed
Chr1	31575732	31575735	ACCG	A	ACCG	INDEL	OS01G0752700	Exonic	5UTR	NA	LOC_Os01g54910	GTP-binding protein typA/bipA, putative, expressed
Chr1	32848830	32848832	ATT	A	ATT	INDEL	OS01G0776600	Intronic	NA	NA	LOC_Os01g56880	purple acid phosphatase precursor, putative, expressed
Chr1	33411377	33411379	AAG	A	AAG	INDEL	OS01G0787600	Intronic	NA	NA	LOC_Os01g57770	OsPOP4 - Putative Prolyl Oligopeptidase homologue, expressed
Chr1	33903759	33903775	ATTCCAACTGGTAAAAT	A	ATTCCAACTGGTAAAAT	INDEL	OS01G0800500	Intronic	NA	NA	LOC_Os01g58640	nucleotide pyrophosphatase/phosphodiesterase, putative, expressed
Chr1	34144903	34144912	AGGACGGACC	A	AGGACGGACC	INDEL	OS01G0805400	Exonic	CDS	InFrame	LOC_Os01g59100	cytokinin-N-glucosyltransferase 1, putative, expressed
Chr1	35480227	35480230	AAAC	A	AAAC	INDEL	OS01G0829100	Exonic	3UTR	NA	LOC_Os01g61330	ankyrin homolog precursor, putative, expressed
Chr1	35482180	35482181	AG	A	AG	INDEL	OS01G0829100	Exonic	5UTR	NA	LOC_Os01g61330	ankyrin homolog precursor, putative, expressed
Chr1	35499206	35499218	AGCCGCCGCCGCC	A	AGCCGCCGCCGCC	INDEL	OS01G0829800	Exonic	5UTR	NA	LOC_Os01g61380	lactate/malate dehydrogenase, putative, expressed
Chr1	41583785	41583787	ATC	A	ATC	INDEL	OS01G0946100	Exonic	3UTR	NA	LOC_Os01g71780	WD domain, G-beta repeat domain containing protein, expressed
Chr10	13016787	13016787	G	A	G	SNP	OS10G0391200	Exonic	CDS	Silent	LOC_Os10g25210	OsFBX384 - F-box domain containing protein, expressed
Chr10	21207566	21207566	G	A	G	SNP	OS10G0542900	Exonic	3UTR	NA	LOC_Os10g39680	CHIT14 - Chitinase family protein precursor, expressed
Chr10	19173053	19173053	G	A	G	SNP	OS10G0502600	Exonic	3UTR	NA	LOC_Os10g35870	cytochrome b5-like Heme/Steroid binding domain containing protein, expressed
Chr10	21563165	21563165	C	A	C	SNP	OS10G0550000	Exonic	3UTR	NA	LOC_Os10g40250	expressed protein
Chr10	7686081	7686081	T	A	T	SNP	OS10G0208500	Intronic	NA	NA	LOC_Os10g14180	expressed protein
Chr10	7686132	7686132	T	A	T	SNP	OS10G0208500	Intronic	NA	NA	LOC_Os10g14180	expressed protein
Chr10	20844244	20844244	G	A	G	SNP	OS10G0535800	Intronic	NA	NA	LOC_Os10g39100	uncharacterized Cys-rich domain containing protein, putative, expressed
Chr10	20844278	20844278	G	A	G	SNP	OS10G0535800	Intronic	NA	NA	LOC_Os10g39100	uncharacterized Cys-rich domain containing protein, putative, expressed
Chr10	20844300	20844300	G	A	G	SNP	OS10G0535800	Intronic	NA	NA	LOC_Os10g39100	uncharacterized Cys-rich domain containing protein, putative, expressed
Chr10	18538302	18538302	G	A	G	SNP	OS10G0488800	Exonic	CDS	NA	LOC_Os10g34710	myosin-2 heavy chain, non muscle, putative, expressed
Chr10	15899769	15899769	G	A	G	SNP	OS10G0442400	Intronic	NA	NA	LOC_Os10g30560	UDP-glucoronosyl and UDP-glucosyl transferase domain containing protein, expressed
Chr10	10232736	10232736	G	A	G	SNP	OS10G0344000	Exonic	3UTR	NA	LOC_Os10g20350	MATE efflux family protein, putative, expressed
Chr10	7561996	7561996	G	A	G	SNP	OS10G0206500	Intronic	NA	NA	LOC_Os10g13914	POEI27 - Pollen Ole e I allergen and extensin family protein precursor, expressed
Chr10	10294469	10294469	G	A	G	SNP	OS10G0344900	Exonic	CDS	Silent	LOC_Os10g20450	MATE efflux family protein, putative, expressed
Chr10	21206902	21206902	G	A	G	SNP	OS10G0542900	Intronic	NA	NA	LOC_Os10g39680	CHIT14 - Chitinase family protein precursor, expressed
Chr10	21206903	21206903	G	A	G	SNP	OS10G0542900	Intronic	NA	NA	LOC_Os10g39680	CHIT14 - Chitinase family protein precursor, expressed
Chr10	21206932	21206932	G	A	G	SNP	OS10G0542900	Intronic	NA	NA	LOC_Os10g39680	CHIT14 - Chitinase family protein precursor, expressed
Chr10	13153467	13153467	G	A	G	SNP	OS10G0394100	Intronic	NA	NA	LOC_Os10g25430	Ser/Thr protein phosphatase family protein, putative, expressed
Chr10	6299814	6299814	G	A	G	SNP	OS10G0190900	Intronic	NA	NA	LOC_Os10g11354	MATE efflux family protein, putative, expressed
Chr10	10295085	10295085	C	A	C	SNP	OS10G0344900	Intronic	NA	NA	LOC_Os10g20450	MATE efflux family protein, putative, expressed
Chr10	6298973	6298973	C	A	C	SNP	OS10G0190900	Intronic	NA	NA	LOC_Os10g11354	MATE efflux family protein, putative, expressed
Chr10	21207335	21207335	C	A	C	SNP	OS10G0542900	Exonic	CDS	Silent	LOC_Os10g39680	CHIT14 - Chitinase family protein precursor, expressed
Chr10	21560455	21560455	G	A	G	SNP	OS10G0550000	Exonic	CDS	Missense	LOC_Os10g40250	expressed protein
Chr10	21560893	21560893	G	A	G	SNP	OS10G0550000	Exonic	CDS	Missense	LOC_Os10g40250	expressed protein
Chr10	21561058	21561058	G	A	G	SNP	OS10G0550000	Exonic	CDS	Missense	LOC_Os10g40250	expressed protein
Chr10	13016599	13016599	G	A	G	SNP	OS10G0391200	Exonic	CDS	Missense	LOC_Os10g25210	OsFBX384 - F-box domain containing protein, expressed
Chr10	2363290	2363290	C	A	C	SNP	OS10G0138100	Exonic	CDS	Missense	LOC_Os10g04860	aldehyde oxidase, putative, expressed
Chr10	23090589	23090589	G	A	G	SNP	OS10G0579100	Intronic	NA	NA	LOC_Os10g42820	early-responsive to dehydration protein-related, putative, expressed
Chr10	23093381	23093381	C	A	C	SNP	OS10G0579100	Exonic	5UTR	NA	LOC_Os10g42820	early-responsive to dehydration protein-related, putative, expressed
Chr10	23080248	23080248	G	A	G	SNP	OS10G0578900	Intronic	NA	NA	LOC_Os10g42790	cytidylyltransferase domain containing protein, expressed
Chr10	21759275	21759275	G	A	G	SNP	OS10G0554200	Intronic	NA	NA	LOC_Os10g40600	peptide transporter PTR2, putative, expressed
Chr10	21759870	21759870	T	A	T	SNP	OS10G0554200	Intronic	NA	NA	LOC_Os10g40600	peptide transporter PTR2, putative, expressed
Chr10	21760994	21760994	G	A	G	SNP	OS10G0554200	Intronic	NA	NA	LOC_Os10g40600	peptide transporter PTR2, putative, expressed
Chr10	21697257	21697257	G	A	G	SNP	OS10G0552800	Exonic	3UTR	NA	LOC_Os10g40530	LTPL146 - Protease inhibitor/seed storage/LTP family protein precursor, expressed
Chr10	21620228	21620228	C	A	C	SNP	OS10G0550900	Intronic	NA	NA	LOC_Os10g40360	proline oxidase, mitochondrial precursor, putative, expressed
Chr10	21558741	21558741	T	A	T	SNP	OS10G0550000	Intronic	NA	NA	LOC_Os10g40250	expressed protein
Chr10	21561710	21561710	G	A	G	SNP	OS10G0550000	Intronic	NA	NA	LOC_Os10g40250	expressed protein
Chr10	21360897	21360897	G	A	G	SNP	OS10G0546400	Exonic	5UTR	NA	LOC_Os10g39920	expressed protein
Chr10	21206037	21206037	G	A	G	SNP	OS10G0542900	Intronic	NA	NA	LOC_Os10g39680	CHIT14 - Chitinase family protein precursor, expressed
Chr10	21206503	21206503	G	A	G	SNP	OS10G0542900	Intronic	NA	NA	LOC_Os10g39680	CHIT14 - Chitinase family protein precursor, expressed
Chr10	21206720	21206720	G	A	G	SNP	OS10G0542900	Intronic	NA	NA	LOC_Os10g39680	CHIT14 - Chitinase family protein precursor, expressed
Chr10	21206816	21206816	G	A	G	SNP	OS10G0542900	Intronic	NA	NA	LOC_Os10g39680	CHIT14 - Chitinase family protein precursor, expressed
Chr10	20214276	20214276	C	A	C	SNP	OS10G0522000	Exonic	5UTR	NA	LOC_Os10g37770	dehydration response related protein, putative, expressed
Chr10	19362977	19362977	G	A	G	SNP	OS10G0506200	Intronic	NA	NA	LOC_Os10g36210	valyl-tRNA synthetase, putative, expressed
Chr10	19173043	19173043	G	A	G	SNP	OS10G0502600	Exonic	3UTR	NA	LOC_Os10g35870	cytochrome b5-like Heme/Steroid binding domain containing protein, expressed
Chr10	19095589	19095589	T	A	T	SNP	OS10G0500600	Exonic	3UTR	NA	LOC_Os10g35710	expressed protein

Chr10	15639474	15639474	G	A	G	SNP	OS10G0437200	Intronic	NA	NA	LOC_Os10g30100	3-5 exonuclease family protein, putative, expressed
Chr10	15640042	15640042	G	A	G	SNP	OS10G0437200	Exonic	3UTR	NA	LOC_Os10g30100	3-5 exonuclease family protein, putative, expressed
Chr10	15641905	15641905	G	A	G	SNP	OS10G0437200	Intronic	NA	NA	LOC_Os10g30100	3-5 exonuclease family protein, putative, expressed
Chr10	15643425	15643425	T	A	T	SNP	OS10G0437200	Intronic	NA	NA	LOC_Os10g30100	3-5 exonuclease family protein, putative, expressed
Chr10	15404319	15404319	G	A	G	SNP	OS10G0431900	Intronic	NA	NA	LOC_Os10g29620	tyrosine protein kinase domain containing protein, putative, expressed
Chr10	15404359	15404359	G	A	G	SNP	OS10G0431900	Intronic	NA	NA	LOC_Os10g29620	tyrosine protein kinase domain containing protein, putative, expressed
Chr10	15404419	15404419	G	A	G	SNP	OS10G0431900	Intronic	NA	NA	LOC_Os10g29620	tyrosine protein kinase domain containing protein, putative, expressed
Chr10	15405394	15405394	G	A	G	SNP	OS10G0431900	Intronic	NA	NA	LOC_Os10g29620	tyrosine protein kinase domain containing protein, putative, expressed
Chr10	15307875	15307875	G	A	G	SNP	OS10G0430200	Intronic	NA	NA	LOC_Os10g29470	dehydrogenase, putative, expressed
Chr10	15308106	15308106	G	A	G	SNP	OS10G0430200	Intronic	NA	NA	LOC_Os10g29470	dehydrogenase, putative, expressed
Chr10	15308150	15308150	G	A	G	SNP	OS10G0430200	Intronic	NA	NA	LOC_Os10g29470	dehydrogenase, putative, expressed
Chr10	14747880	14747880	G	A	G	SNP	OS10G0419300	Intronic	NA	NA	LOC_Os10g28340	heat stress transcription factor, putative, expressed
Chr10	14748717	14748717	G	A	G	SNP	OS10G0419300	Intronic	NA	NA	LOC_Os10g28340	heat stress transcription factor, putative, expressed
Chr10	13149521	13149521	C	A	C	SNP	OS10G0394100	Intronic	NA	NA	LOC_Os10g25430	Ser/Thr protein phosphatase family protein, putative, expressed
Chr10	13150165	13150165	G	A	G	SNP	OS10G0394100	Intronic	NA	NA	LOC_Os10g25430	Ser/Thr protein phosphatase family protein, putative, expressed
Chr10	13150684	13150684	T	A	T	SNP	OS10G0394100	Intronic	NA	NA	LOC_Os10g25430	Ser/Thr protein phosphatase family protein, putative, expressed
Chr10	13151400	13151400	G	A	G	SNP	OS10G0394100	Intronic	NA	NA	LOC_Os10g25430	Ser/Thr protein phosphatase family protein, putative, expressed
Chr10	13152977	13152977	C	A	C	SNP	OS10G0394100	Intronic	NA	NA	LOC_Os10g25430	Ser/Thr protein phosphatase family protein, putative, expressed
Chr10	13133288	13133288	G	A	G	SNP	OS10G0393800	Exonic	3UTR	NA	LOC_Os10g25400	GDSL-like lipase/acylhydrolase, putative, expressed
Chr10	13133310	13133310	G	A	G	SNP	OS10G0393800	Exonic	3UTR	NA	LOC_Os10g25400	GDSL-like lipase/acylhydrolase, putative, expressed
Chr10	12970122	12970122	G	A	G	SNP	OS10G0390500	Intronic	NA	NA	LOC_Os10g25130	aminotransferase, classes I and II, domain containing protein, expressed
Chr10	12971210	12971210	C	A	C	SNP	OS10G0390500	Intronic	NA	NA	LOC_Os10g25130	aminotransferase, classes I and II, domain containing protein, expressed
Chr10	12973469	12973469	G	A	G	SNP	OS10G0390500	Intronic	NA	NA	LOC_Os10g25130	aminotransferase, classes I and II, domain containing protein, expressed
Chr10	12023322	12023322	C	A	C	SNP	OS10G0377000	Intronic	NA	NA	LOC_Os10g23060	expressed protein
Chr10	12023849	12023849	C	A	C	SNP	OS10G0377000	Exonic	3UTR	NA	LOC_Os10g23060	expressed protein
Chr10	7559599	7559599	G	A	G	SNP	OS10G0206500	Intronic	NA	NA	LOC_Os10g13914	POEI27 - Pollen Ole e I allergen and extensin family protein precursor, expressed
Chr10	6295883	6295883	G	A	G	SNP	OS10G0190900	Exonic	CDS	NA	LOC_Os10g11354	MATE efflux family protein, putative, expressed
Chr10	3753512	3753512	G	A	G	SNP	OS10G0159300	Exonic	3UTR	NA	LOC_Os10g07160	retrotransposon, putative, centromere-specific, expressed
Chr10	3753542	3753542	C	A	C	SNP	OS10G0159300	Exonic	3UTR	NA	LOC_Os10g07160	retrotransposon, putative, centromere-specific, expressed
Chr10	3754319	3754319	G	A	G	SNP	OS10G0159300	Exonic	3UTR	NA	LOC_Os10g07160	retrotransposon, putative, centromere-specific, expressed
Chr10	3754573	3754573	G	A	G	SNP	OS10G0159300	Exonic	3UTR	NA	LOC_Os10g07160	retrotransposon, putative, centromere-specific, expressed
Chr10	3754918	3754918	T	A	T	SNP	OS10G0159300	Exonic	3UTR	NA	LOC_Os10g07160	retrotransposon, putative, centromere-specific, expressed
Chr10	3754933	3754933	C	A	C	SNP	OS10G0159300	Exonic	3UTR	NA	LOC_Os10g07160	retrotransposon, putative, centromere-specific, expressed
Chr10	3756329	3756329	G	A	G	SNP	OS10G0159300	Exonic	5UTR	NA	LOC_Os10g07160	retrotransposon, putative, centromere-specific, expressed
Chr10	3439162	3439162	T	A	T	SNP	OS10G0154700	Exonic	3UTR	NA	LOC_Os10g06630	peptidyl-prolyl cis-trans isomerase, putative, expressed
Chr10	3439309	3439309	G	A	G	SNP	OS10G0154700	Exonic	3UTR	NA	LOC_Os10g06630	peptidyl-prolyl cis-trans isomerase, putative, expressed
Chr10	2359408	2359408	G	A	G	SNP	OS10G0138100	Intronic	NA	NA	LOC_Os10g04860	aldehyde oxidase, putative, expressed
Chr10	2360199	2360199	G	A	G	SNP	OS10G0138100	Intronic	NA	NA	LOC_Os10g04860	aldehyde oxidase, putative, expressed
Chr10	2360660	2360660	G	A	G	SNP	OS10G0138100	Intronic	NA	NA	LOC_Os10g04860	aldehyde oxidase, putative, expressed
Chr10	2360718	2360718	G	A	G	SNP	OS10G0138100	Intronic	NA	NA	LOC_Os10g04860	aldehyde oxidase, putative, expressed
Chr10	2360726	2360726	G	A	G	SNP	OS10G0138100	Intronic	NA	NA	LOC_Os10g04860	aldehyde oxidase, putative, expressed
Chr10	2361061	2361061	G	A	G	SNP	OS10G0138100	Intronic	NA	NA	LOC_Os10g04860	aldehyde oxidase, putative, expressed
Chr10	2361235	2361235	C	A	C	SNP	OS10G0138100	Intronic	NA	NA	LOC_Os10g04860	aldehyde oxidase, putative, expressed
Chr10	2361619	2361619	G	A	G	SNP	OS10G0138100	Intronic	NA	NA	LOC_Os10g04860	aldehyde oxidase, putative, expressed
Chr10	2366064	2366064	C	A	C	SNP	OS10G0138100	Intronic	NA	NA	LOC_Os10g04860	aldehyde oxidase, putative, expressed
Chr10	2367163	2367163	C	A	C	SNP	OS10G0138100	Intronic	NA	NA	LOC_Os10g04860	aldehyde oxidase, putative, expressed
Chr10	2367377	2367377	G	A	G	SNP	OS10G0138100	Intronic	NA	NA	LOC_Os10g04860	aldehyde oxidase, putative, expressed
Chr10	378275	378275	G	A	G	SNP	OS10G0104900	Intronic	NA	NA	LOC_Os10g01570	C-5 cytosine-specific DNA methylase, putative, expressed
Chr10	379038	379038	G	A	G	SNP	OS10G0104900	Intronic	NA	NA	LOC_Os10g01570	C-5 cytosine-specific DNA methylase, putative, expressed
Chr10	380644	380644	G	A	G	SNP	OS10G0104900	Intronic	NA	NA	LOC_Os10g01570	C-5 cytosine-specific DNA methylase, putative, expressed
Chr10	69221	69221	G	A	G	SNP	OS10G0100500	Intronic	NA	NA	LOC_Os10g01060	protein kinase family protein, putative, expressed
Chr10	13883200	13883200	G	A	G	SNP	OS10G0406300	Exonic	CDS	Nonsense	LOC_Os10g26620	dof zinc finger domain containing protein, putative, expressed
Chr10	23088876	23088876	T	A	T	SNP	OS10G0579100	Exonic	CDS	Silent	LOC_Os10g42820	early-responsive to dehydration protein-related, putative, expressed
Chr10	21561393	21561393	G	A	G	SNP	OS10G0550000	Exonic	CDS	Silent	LOC_Os10g40250	expressed protein
Chr10	15305040	15305040	C	A	C	SNP	OS10G0430200	Exonic	CDS	Silent	LOC_Os10g29470	dehydrogenase, putative, expressed
Chr10	14603200	14603200	G	A	G	SNP	OS10G0416800	Exonic	CDS	Silent	LOC_Os10g28120	glycosyl hydrolase, putative, expressed
Chr10	13133121	13133121	T	A	T	SNP	OS10G0393800	Exonic	CDS	Silent	LOC_Os10g25400	GDSL-like lipase/acylhydrolase, putative, expressed
Chr10	13016835	13016835	T	A	T	SNP	OS10G0391200	Exonic	CDS	Silent	LOC_Os10g25210	OsFBX384 - F-box domain containing protein, expressed
Chr10	13016937	13016937	G	A	G	SNP	OS10G0391200	Exonic	CDS	Silent	LOC_Os10g25210	OsFBX384 - F-box domain containing protein, expressed
Chr10	2362373	2362373	G	A	G	SNP	OS10G0138100	Exonic	CDS	Silent	LOC_Os10g04860	aldehyde oxidase, putative, expressed
Chr10	23088261	23088263	AAC	A	AAC	INDEL	OS10G0579100	Exonic	3UTR	NA	LOC_Os10g42820	early-responsive to dehydration protein-related, putative, expressed
Chr10	23092327	23092328	AT	A	AT	INDEL	OS10G0579100	Intronic	NA	NA	LOC_Os10g42820	early-responsive to dehydration protein-related, putative, expressed
Chr10	21759298	21759299	AT	A	AT	INDEL	OS10G0554200	Intronic	NA	NA	LOC_Os10g40600	peptide transporter PTR2, putative, expressed
Chr10	21761033	21761040	AATGATGC	A	AATGATGC	INDEL	OS10G0554200	Intronic	NA	NA	LOC_Os10g40600	peptide transporter PTR2, putative, expressed
Chr10	15307397	15307398	AG	A	AG	INDEL	OS10G0430200	Intronic	NA	NA	LOC_Os10g29470	dehydrogenase, putative, expressed
Chr10	378417	378418	AT	A	AT	INDEL	OS10G0104900	Intronic	NA	NA	LOC_Os10g01570	C-5 cytosine-specific DNA methylase, putative, expressed
Chr10	21761740	21761740	G	A	G	SNP	OS10G0554200	Exonic	CDS	Missense	LOC_Os10g40600	peptide transporter PTR2, putative, expressed
Chr10	21207297	21207297	G	A	G	SNP	OS10G0542900	Exonic	CDS	Missense	LOC_Os10g39680	CHIT14 - Chitinase family protein precursor, expressed
Chr10	13133087	13133087	C	A	C	SNP	OS10G0393800	Exonic	CDS	Missense	LOC_Os10g25400	GDSL-like lipase/acylhydrolase, putative, expressed
Chr10	3755228	3755228	G	A	G	SNP	OS10G0159300	Exonic	CDS	Missense	LOC_Os10g07160	retrotransposon, putative, centromere-specific, expressed
Chr10	3755241	3755241	G	A	G	SNP	OS10G0159300	Exonic	CDS	Missense	LOC_Os10g07160	retrotransposon, putative, centromere-specific, expressed
Chr10	3438746	3438746	G	A	G	SNP	OS10G0154700	Exonic	CDS	Missense	LOC_Os10g06630	peptidyl-prolyl cis-trans isomerase, putative, expressed
Chr10	3438768	3438768	G	A	G	SNP	OS10G0154700	Exonic	CDS	Missense	LOC_Os10g06630	peptidyl-prolyl cis-trans isomerase, putative, expressed
Chr10	2913343	2913343	G	A	G	SNP	OS10G0148400	Exonic	CDS	Missense	LOC_Os10g05780	POT domain containing peptide transporter, putative, expressed
Chr10	2913805	2913805	G	A	G	SNP	OS10G0148400	Exonic	CDS	Missense	LOC_Os10g05780	POT domain containing peptide transporter, putative, expressed
Chr10	2914169	2914169	G	A	G	SNP	OS10G0148400	Exonic	CDS	Missense	LOC_Os10g05780	POT domain containing peptide transporter, putative, expressed
Chr10	2914210	2914210	G	A	G	SNP	OS10G0148400	Exonic	CDS	Missense	LOC_Os10g05780	POT domain containing peptide transporter, putative, expressed
Chr10	2507375	2507375	T	A	T	SNP	OS10G0140700	Exonic	CDS	Missense	LOC_Os10g05130	expressed protein
Chr10	67342	67342	G	A	G	SNP	OS10G0100500	Exonic	CDS	Missense	LOC_Os10g01060	protein kinase family protein, putative, expressed
Chr10	21760788	21760788	G	A	G	SNP	OS10G0554200	Intronic	NA	NA	LOC_Os10g40600	peptide transporter PTR2, putative, expressed
Chr10	21760805	21760805	G	A	G	SNP	OS10G0554200	Intronic	NA	NA	LOC_Os10g40600	peptide transporter PTR2, putative, expressed
Chr10	20894561	20894561	G	A	G	SNP	OS10G0536500	Intronic	NA	NA	LOC_Os10g39150	expressed protein
Chr10	20212079	20212079	C	A	C	SNP	OS10G0522000	Intronic	NA	NA	LOC_Os10g37770	dehydration response related protein, putative, expressed
Chr10	20214185	20214185	G	A	G	SNP	OS10G0522000	Exonic	CDS	NA	LOC_Os10g37770	dehydration response related protein, putative, expressed
Chr10	18637601	18637601	G	A	G	SNP	OS10G0490900	Exonic	3UTR	NA	LOC_Os10g34920	secretory protein, putative, expressed

Chr10	18637605	18637605	G	A	G	SNP	OS10G0490900	Intronic	NA	NA	LOC_Os10g34920	secretory protein, putative, expressed
Chr10	15308656	15308656	G	A	G	SNP	OS10G0430200	Intronic	NA	NA	LOC_Os10g29470	dehydrogenase, putative, expressed
Chr10	15308682	15308682	G	A	G	SNP	OS10G0430200	Intronic	NA	NA	LOC_Os10g29470	dehydrogenase, putative, expressed
Chr10	13154009	13154009	C	A	C	SNP	OS10G0394100	Exonic	5UTR	NA	LOC_Os10g25430	Ser/Thr protein phosphatase family protein, putative, expressed
Chr10	12052865	12052865	G	A	G	SNP	OS10G0377400	Exonic	5UTR	NA	LOC_Os10g23100	ras-related protein, putative, expressed
Chr10	10396385	10396385	C	A	C	SNP	OS10G0346300	Intronic	NA	NA	LOC_Os10g20610	laccase-15 precursor, putative, expressed
Chr10	10396390	10396390	T	A	T	SNP	OS10G0346300	Intronic	NA	NA	LOC_Os10g20610	laccase-15 precursor, putative, expressed
Chr10	7685429	7685429	G	A	G	SNP	OS10G0208500	Exonic	5UTR	NA	LOC_Os10g14180	expressed protein
Chr10	7685449	7685449	G	A	G	SNP	OS10G0208500	Exonic	5UTR	NA	LOC_Os10g14180	expressed protein
Chr10	7685766	7685766	G	A	G	SNP	OS10G0208500	Intronic	NA	NA	LOC_Os10g14180	expressed protein
Chr10	7685923	7685923	C	A	C	SNP	OS10G0208500	Intronic	NA	NA	LOC_Os10g14180	expressed protein
Chr10	3754156	3754156	G	A	G	SNP	OS10G0159300	Exonic	3UTR	NA	LOC_Os10g07160	retrotransposon, putative, centromere-specific, expressed
Chr10	3756693	3756693	C	A	C	SNP	OS10G0159300	Intronic	NA	NA	LOC_Os10g07160	retrotransposon, putative, centromere-specific, expressed
Chr10	3438546	3438546	T	A	T	SNP	OS10G0154700	Exonic	5UTR	NA	LOC_Os10g06630	peptidyl-prolyl cis-trans isomerase, putative, expressed
Chr10	3438578	3438578	C	A	C	SNP	OS10G0154700	Exonic	5UTR	NA	LOC_Os10g06630	peptidyl-prolyl cis-trans isomerase, putative, expressed
Chr10	2508049	2508049	G	A	G	SNP	OS10G0140700	Exonic	3UTR	NA	LOC_Os10g05130	expressed protein
Chr10	21761997	21761997	C	A	C	SNP	OS10G0554200	Exonic	CDS	Silent	LOC_Os10g40600	peptide transporter PTR2, putative, expressed
Chr10	21360732	21360732	T	A	T	SNP	OS10G0546400	Exonic	CDS	Silent	LOC_Os10g39920	expressed protein
Chr10	21019692	21019692	G	A	G	SNP	OS10G0539500	Exonic	CDS	Silent	LOC_Os10g39410	Core histone H2A/H2B/H3/H4 domain containing protein, putative, expressed
Chr10	14749559	14749559	G	A	G	SNP	OS10G0419300	Exonic	CDS	Silent	LOC_Os10g28340	heat stress transcription factor, putative, expressed
Chr10	14658736	14658736	G	A	G	SNP	OS10G0417800	Exonic	CDS	Silent	LOC_Os10g28210	plant-specific domain TIGR01615 family protein, expressed
Chr10	14590763	14590763	C	A	C	SNP	OS10G0416500	Exonic	CDS	Silent	LOC_Os10g28080	glycosyl hydrolase, putative, expressed
Chr10	13132983	13132983	G	A	G	SNP	OS10G0393800	Exonic	CDS	Silent	LOC_Os10g25400	GDSL-like lipase/acylhydrolase, putative, expressed
Chr10	12869399	12869399	G	A	G	SNP	OS10G0388900	Exonic	CDS	Silent	LOC_Os10g25000	expressed protein
Chr10	12869870	12869870	G	A	G	SNP	OS10G0388900	Exonic	CDS	Silent	LOC_Os10g25000	expressed protein
Chr10	10393370	10393370	G	A	G	SNP	OS10G0346300	Exonic	CDS	Silent	LOC_Os10g20610	laccase-15 precursor, putative, expressed
Chr10	10293467	10293467	G	A	G	SNP	OS10G0344900	Exonic	CDS	Silent	LOC_Os10g20450	MATE efflux family protein, putative, expressed
Chr10	2913309	2913309	G	A	G	SNP	OS10G0148400	Exonic	CDS	Silent	LOC_Os10g05780	POT domain containing peptide transporter, putative, expressed
Chr10	2913585	2913585	C	A	C	SNP	OS10G0148400	Exonic	CDS	Silent	LOC_Os10g05780	POT domain containing peptide transporter, putative, expressed
Chr10	2914029	2914029	G	A	G	SNP	OS10G0148400	Exonic	CDS	Silent	LOC_Os10g05780	POT domain containing peptide transporter, putative, expressed
Chr10	2914038	2914038	G	A	G	SNP	OS10G0148400	Exonic	CDS	Silent	LOC_Os10g05780	POT domain containing peptide transporter, putative, expressed
Chr10	2363198	2363198	G	A	G	SNP	OS10G0138100	Exonic	CDS	Silent	LOC_Os10g04860	aldehyde oxidase, putative, expressed
Chr10	2365745	2365745	G	A	G	SNP	OS10G0138100	Exonic	CDS	Silent	LOC_Os10g04860	aldehyde oxidase, putative, expressed
Chr10	2365913	2365913	G	A	G	SNP	OS10G0138100	Exonic	CDS	Silent	LOC_Os10g04860	aldehyde oxidase, putative, expressed
Chr10	23157201	23157209	AGATCGATC	A	AGATCGATC	INDEL	OS10G0580400	Intronic	NA	NA	LOC_Os10g42960	urea active transporter, putative, expressed
Chr10	21204435	21204453	ATTAAAAAGGAAAACCCAT	A	ATTAAAAAGGAAAACCCAT	INDEL	OS10G0542800	Exonic	3UTR	NA	LOC_Os10g39670	protein kinase family protein, putative, expressed
Chr10	20894621	20894622	AT	A	AT	INDEL	OS10G0536500	Intronic	NA	NA	LOC_Os10g39150	expressed protein
Chr10	15896401	15896418	AGAAGTTGAATTTTAACC	A	AGAAGTTGAATTTTAACC	INDEL	OS10G0442400	Intronic	NA	NA	LOC_Os10g30560	UDP-glucoronosyl and UDP-glucosyl transferase domain containing protein, expressed
Chr10	15404447	15404453	AACACGT	A	AACACGT	INDEL	OS10G0431900	Intronic	NA	NA	LOC_Os10g29620	tyrosine protein kinase domain containing protein, putative, expressed
Chr10	14749797	14749798	AC	A	AC	INDEL	OS10G0419300	Exonic	5UTR	NA	LOC_Os10g28340	heat stress transcription factor, putative, expressed
Chr10	3439223	3439228	ATACAT	A	ATACAT	INDEL	OS10G0154700	Exonic	3UTR	NA	LOC_Os10g06630	peptidyl-prolyl cis-trans isomerase, putative, expressed
Chr11	20185352	20185353	AT	A	AT	INDEL	OS11G0547000	Exonic	5UTR	NA	LOC_Os11g34460	OsFBO10 - F-box and other domain containing protein, expressed
Chr11	759750	759753	AAAG	A	AAAG	INDEL	OS11G0116900	Exonic	3UTR	NA	LOC_Os11g02480	WRKY46, expressed
Chr11	1225454	1225455	AT	A	AT	INDEL	OS11G0126800	Intronic	NA	NA	LOC_Os11g03290	nucleoside-triphosphatase, putative, expressed
Chr11	4971479	4971481	ACT	A	ACT	INDEL	OS11G0199200	Exonic	3UTR	NA	LOC_Os11g09280	OsPDIL1-1 protein disulfide isomerase PDIL1-1, expressed
Chr11	4972704	4972706	AAT	A	AAT	INDEL	OS11G0199200	Intronic	NA	NA	LOC_Os11g09280	OsPDIL1-1 protein disulfide isomerase PDIL1-1, expressed
Chr11	4973438	4973439	AT	A	AT	INDEL	OS11G0199200	Intronic	NA	NA	LOC_Os11g09280	OsPDIL1-1 protein disulfide isomerase PDIL1-1, expressed
Chr11	4974209	4974210	AT	A	AT	INDEL	OS11G0199200	Intronic	NA	NA	LOC_Os11g09280	OsPDIL1-1 protein disulfide isomerase PDIL1-1, expressed
Chr11	4974458	4974459	AG	A	AG	INDEL	OS11G0199200	Intronic	NA	NA	LOC_Os11g09280	OsPDIL1-1 protein disulfide isomerase PDIL1-1, expressed
Chr11	5565811	5565814	ATCT	A	ATCT	INDEL	OS11G0208600	Exonic	3UTR	NA	LOC_Os11g10250	expressed protein
Chr11	10863950	10863953	AGGC	A	AGGC	INDEL	OS11G0295900	Exonic	CDS	InFrame	LOC_Os11g19060	BABY BOOM 1, putative, expressed
Chr11	10864486	10864487	AT	A	AT	INDEL	OS11G0295900	Intronic	NA	NA	LOC_Os11g19060	BABY BOOM 1, putative, expressed
Chr11	19677039	19677053	ATTTGATCAAGTTTG	A	ATTTGATCAAGTTTG	INDEL	OS11G0539200	Intronic	NA	NA	LOC_Os11g33270	glycosyl hydrolases family 16, putative, expressed
Chr11	21892088	21892091	ACCT	A	ACCT	INDEL	OS11G0579800	Exonic	CDS	InFrame	LOC_Os11g37080	h/ACA ribonucleoprotein complex subunit 1-like protein 1, putative, expressed
Chr11	22176287	22176289	AGC	A	AGC	INDEL	OS11G0586300	Intronic	NA	NA	LOC_Os11g37560	expressed protein
Chr11	22176562	22176565	AATG	A	AATG	INDEL	OS11G0586300	Intronic	NA	NA	LOC_Os11g37560	expressed protein
Chr11	23493965	23493967	AAC	A	AAC	INDEL	OS11G0608300	Intronic	NA	NA	LOC_Os11g39450	cysteine-rich receptor-like protein kinase 7 precursor, putative, expressed
Chr11	26879377	26879389	ATTTAAAATTTTT	A	ATTTAAAATTTTT	INDEL	OS11G0666200	Intronic	NA	NA	LOC_Os11g44430	protein kinase, putative, expressed
Chr11	28933457	28933458	AT	A	AT	INDEL	OS11G0707000	Intronic	NA	NA	LOC_Os11g47970	AAA-type ATPase family protein, putative, expressed
Chr11	28935181	28935191	AACAAGCAAGT	A	AACAAGCAAGT	INDEL	OS11G0707000	Intronic	NA	NA	LOC_Os11g47970	AAA-type ATPase family protein, putative, expressed
Chr12	3755582	3755582	C	A	C	SNP	OS12G0174100	Exonic	CDS	Silent	LOC_Os12g07540	growth regulator related protein, putative, expressed
Chr12	3129099	3129099	G	A	G	SNP	OS12G0162100	Exonic	CDS	Silent	LOC_Os12g06490	STE_PAK_Ste20_Slob_Wnk.6 - STE kinases include homologs to sterile 7, sterile 11 and sterile 20 from yeast, expressed
Chr12	5016823	5016823	G	A	G	SNP	OS12G0197100	Exonic	CDS	Silent	LOC_Os12g09540	phosphoribosylamine--glycine ligase, putative, expressed
Chr12	4535837	4535837	T	A	T	SNP	OS12G0190000	Intronic	NA	NA	LOC_Os12g08810	VTC2, putative, expressed
Chr12	9918960	9918960	T	A	T	SNP	OS12G0271700	Intronic	NA	NA	LOC_Os12g17320	polyprenyl synthetase, putative, expressed
Chr12	9920398	9920398	T	A	T	SNP	OS12G0271700	Intronic	NA	NA	LOC_Os12g17320	polyprenyl synthetase, putative, expressed
Chr12	17574896	17574896	T	A	T	SNP	OS12G0479400	Intronic	NA	NA	LOC_Os12g29520	auxin response factor, putative, expressed
Chr12	19181534	19181534	T	A	T	SNP	OS12G0503000	Intronic	NA	NA	LOC_Os12g31860	ureide permease, putative, expressed
Chr12	22136169	22136169	T	A	T	SNP	OS12G0547600	Intronic	NA	NA	LOC_Os12g36110	calmodulin binding protein, putative, expressed
Chr12	22136186	22136186	T	A	T	SNP	OS12G0547600	Intronic	NA	NA	LOC_Os12g36110	calmodulin binding protein, putative, expressed
Chr12	22632913	22632913	T	A	T	SNP	OS12G0556200	Exonic	3UTR	NA	LOC_Os12g36910	calmodulin binding protein, putative, expressed
Chr12	23032615	23032615	T	A	T	SNP	OS12G0562100	Exonic	CDS	Missense	LOC_Os12g37530	expressed protein
Chr12	23034978	23034978	T	A	T	SNP	OS12G0562100	Intronic	NA	NA	LOC_Os12g37530	expressed protein
Chr12	23035120	23035120	T	A	T	SNP	OS12G0562100	Intronic	NA	NA	LOC_Os12g37530	expressed protein
Chr12	23501735	23501735	T	A	T	SNP	OS12G0570700	Intronic	NA	NA	LOC_Os12g38270	metallothionein, putative, expressed
Chr12	23502083	23502083	T	A	T	SNP	OS12G0570700	Exonic	3UTR	NA	LOC_Os12g38270	metallothionein, putative, expressed
Chr12	23807646	23807646	T	A	T	SNP	OS12G0576600	Intronic	NA	NA	LOC_Os12g38750	nucleotide pyrophosphatase/phosphodiesterase, putative, expressed
Chr12	4507429	4507429	G	A	G	SNP	OS12G0189400	Intronic	NA	NA	LOC_Os12g08770	photosystem I reaction center subunit N, chloroplast precursor, putative, expressed
Chr12	4533193	4533193	G	A	G	SNP	OS12G0190000	Exonic	3UTR	NA	LOC_Os12g08810	VTC2, putative, expressed
Chr12	4533529	4533529	G	A	G	SNP	OS12G0190000	Exonic	CDS	Silent	LOC_Os12g08810	VTC2, putative, expressed
Chr12	4533939	4533939	G	A	G	SNP	OS12G0190000	Intronic	NA	NA	LOC_Os12g08810	VTC2, putative, expressed
Chr12	4534345	4534345	G	A	G	SNP	OS12G0190000	Intronic	NA	NA	LOC_Os12g08810	VTC2, putative, expressed
Chr12	4534401	4534401	G	A	G	SNP	OS12G0190000	Intronic	NA	NA	LOC_Os12g08810	VTC2, putative, expressed
Chr12	4535533	4535533	G	A	G	SNP	OS12G0190000	Intronic	NA	NA	LOC_Os12g08810	VTC2, putative, expressed
Chr12	9917705	9917705	G	A	G	SNP	OS12G0271700	Intronic	NA	NA	LOC_Os12g17320	polyprenyl synthetase, putative, expressed

Chr12	9918033	9918033	G	A	G	SNP	OS12G0271700	Intronic	NA	NA	LOC_Os12g17320	polyprenyl synthetase, putative, expressed
Chr12	9918893	9918893	G	A	G	SNP	OS12G0271700	Intronic	NA	NA	LOC_Os12g17320	polyprenyl synthetase, putative, expressed
Chr12	9920391	9920391	G	A	G	SNP	OS12G0271700	Intronic	NA	NA	LOC_Os12g17320	polyprenyl synthetase, putative, expressed
Chr12	17572391	17572391	G	A	G	SNP	OS12G0479400	Exonic	3UTR	NA	LOC_Os12g29520	auxin response factor, putative, expressed
Chr12	17572879	17572879	G	A	G	SNP	OS12G0479400	Intronic	NA	NA	LOC_Os12g29520	auxin response factor, putative, expressed
Chr12	18339783	18339783	G	A	G	SNP	OS12G0489100	Intronic	NA	NA	LOC_Os12g30540	ubiquitin carboxyl-terminal hydrolase, putative, expressed
Chr12	19181620	19181620	G	A	G	SNP	OS12G0503000	Intronic	NA	NA	LOC_Os12g31860	ureide permease, putative, expressed
Chr12	19181944	19181944	G	A	G	SNP	OS12G0503000	Intronic	NA	NA	LOC_Os12g31860	ureide permease, putative, expressed
Chr12	19182884	19182884	G	A	G	SNP	OS12G0503000	Exonic	3UTR	NA	LOC_Os12g31860	ureide permease, putative, expressed
Chr12	19686249	19686249	G	A	G	SNP	OS12G0511000	Intronic	NA	NA	LOC_Os12g32630	OsFBX448 - F-box domain containing protein, expressed
Chr12	21489719	21489719	G	A	G	SNP	OS12G0538800	Exonic	CDS	Missense	LOC_Os12g35350	kelch repeat protein, putative, expressed
Chr12	22136021	22136021	G	A	G	SNP	OS12G0547600	Exonic	CDS	Silent	LOC_Os12g36110	calmodulin binding protein, putative, expressed
Chr12	22136071	22136071	G	A	G	SNP	OS12G0547600	Exonic	CDS	Missense	LOC_Os12g36110	calmodulin binding protein, putative, expressed
Chr12	22533833	22533833	G	A	G	SNP	OS12G0554500	Intronic	NA	NA	LOC_Os12g36770	lipase class 3 family protein, putative, expressed
Chr12	22533867	22533867	G	A	G	SNP	OS12G0554500	Intronic	NA	NA	LOC_Os12g36770	lipase class 3 family protein, putative, expressed
Chr12	22591996	22591996	G	A	G	SNP	OS12G0555200	Intronic	NA	NA	LOC_Os12g36850	pathogenesis-related Bet v I family protein, putative, expressed
Chr12	22593278	22593278	G	A	G	SNP	OS12G0555200	Exonic	3UTR	NA	LOC_Os12g36850	pathogenesis-related Bet v I family protein, putative, expressed
Chr12	22630204	22630204	G	A	G	SNP	OS12G0556200	Intronic	NA	NA	LOC_Os12g36910	calmodulin binding protein, putative, expressed
Chr12	22630290	22630290	G	A	G	SNP	OS12G0556200	Intronic	NA	NA	LOC_Os12g36910	calmodulin binding protein, putative, expressed
Chr12	22632634	22632634	G	A	G	SNP	OS12G0556200	Exonic	CDS	Missense	LOC_Os12g36910	calmodulin binding protein, putative, expressed
Chr12	23031170	23031170	G	A	G	SNP	OS12G0562100	Exonic	3UTR	NA	LOC_Os12g37530	expressed protein
Chr12	23031749	23031749	G	A	G	SNP	OS12G0562100	Exonic	CDS	Silent	LOC_Os12g37530	expressed protein
Chr12	23033435	23033435	G	A	G	SNP	OS12G0562100	Exonic	CDS	Silent	LOC_Os12g37530	expressed protein
Chr12	23501719	23501719	G	A	G	SNP	OS12G0570700	Intronic	NA	NA	LOC_Os12g38270	metallothionein, putative, expressed
Chr12	23808056	23808056	G	A	G	SNP	OS12G0576600	Intronic	NA	NA	LOC_Os12g38750	nucleotide pyrophosphatase/phosphodiesterase, putative, expressed
Chr12	1514488	1514488	G	A	G	SNP	OS12G0131400	Exonic	3UTR	NA	LOC_Os12g03740	OsFBX438 - F-box domain containing protein, expressed
Chr12	1514506	1514506	G	A	G	SNP	OS12G0131400	Exonic	3UTR	NA	LOC_Os12g03740	OsFBX438 - F-box domain containing protein, expressed
Chr12	1514508	1514508	G	A	G	SNP	OS12G0131400	Exonic	3UTR	NA	LOC_Os12g03740	OsFBX438 - F-box domain containing protein, expressed
Chr12	1514711	1514711	G	A	G	SNP	OS12G0131400	Exonic	3UTR	NA	LOC_Os12g03740	OsFBX438 - F-box domain containing protein, expressed
Chr12	4533187	4533187	C	A	C	SNP	OS12G0190000	Exonic	3UTR	NA	LOC_Os12g08810	VTC2, putative, expressed
Chr12	19695052	19695052	C	A	C	SNP	OS12G0511200	Exonic	5UTR	NA	LOC_Os12g32640	haemolysin-III, putative, expressed
Chr12	22429610	22429610	C	A	C	SNP	OS12G0552500	Exonic	3UTR	NA	LOC_Os12g36640	universal stress protein domain containing protein, putative, expressed
Chr12	274253	274253	G	A	G	SNP	OS12G0105300	Intronic	NA	NA	LOC_Os12g01449	chloroplast unusual positioning protein, putative, expressed
Chr12	274456	274456	T	A	T	SNP	OS12G0105300	Intronic	NA	NA	LOC_Os12g01449	chloroplast unusual positioning protein, putative, expressed
Chr12	275459	275459	T	A	T	SNP	OS12G0105300	Intronic	NA	NA	LOC_Os12g01449	chloroplast unusual positioning protein, putative, expressed
Chr12	276167	276167	G	A	G	SNP	OS12G0105300	Exonic	CDS	Silent	LOC_Os12g01449	chloroplast unusual positioning protein, putative, expressed
Chr12	291079	291079	G	A	G	SNP	OS12G0105500	Exonic	CDS	Silent	LOC_Os12g01480	phosphatidate cytidylyltransferase, putative, expressed
Chr12	681284	681284	G	A	G	SNP	OS12G0113500	Intronic	NA	NA	LOC_Os12g02200	CAMK_KIN1/SNF1/Nim1_like.6 - CAMK includes calcium/calmodulin depedent protein kinases, expressed
Chr12	1514980	1514980	G	A	G	SNP	OS12G0131400	Exonic	CDS	Silent	LOC_Os12g03740	OsFBX438 - F-box domain containing protein, expressed
Chr12	1517006	1517006	G	A	G	SNP	OS12G0131400	Intronic	NA	NA	LOC_Os12g03740	OsFBX438 - F-box domain containing protein, expressed
Chr12	1517060	1517060	G	A	G	SNP	OS12G0131400	Intronic	NA	NA	LOC_Os12g03740	OsFBX438 - F-box domain containing protein, expressed
Chr12	1517278	1517278	G	A	G	SNP	OS12G0131400	Intronic	NA	NA	LOC_Os12g03740	OsFBX438 - F-box domain containing protein, expressed
Chr12	1518139	1518139	G	A	G	SNP	OS12G0131400	Intronic	NA	NA	LOC_Os12g03740	OsFBX438 - F-box domain containing protein, expressed
Chr12	1518161	1518161	T	A	T	SNP	OS12G0131400	Intronic	NA	NA	LOC_Os12g03740	OsFBX438 - F-box domain containing protein, expressed
Chr12	1518163	1518163	T	A	T	SNP	OS12G0131400	Intronic	NA	NA	LOC_Os12g03740	OsFBX438 - F-box domain containing protein, expressed
Chr12	1518705	1518705	C	A	C	SNP	OS12G0131400	Intronic	NA	NA	LOC_Os12g03740	OsFBX438 - F-box domain containing protein, expressed
Chr12	1518786	1518786	G	A	G	SNP	OS12G0131400	Intronic	NA	NA	LOC_Os12g03740	OsFBX438 - F-box domain containing protein, expressed
Chr12	2247517	2247517	T	A	T	SNP	OS12G0145700	Intronic	NA	NA	LOC_Os12g05110	pyruvate kinase, putative, expressed
Chr12	2249319	2249319	G	A	G	SNP	OS12G0145700	Intronic	NA	NA	LOC_Os12g05110	pyruvate kinase, putative, expressed
Chr12	2950782	2950782	T	A	T	SNP	OS12G0159000	Exonic	3UTR	NA	LOC_Os12g06220	harpin-induced protein 1 domain containing protein, expressed
Chr12	3232058	3232058	T	A	T	SNP	OS12G0163700	Intronic	NA	NA	LOC_Os12g06660	actin, putative, expressed
Chr12	3233334	3233334	G	A	G	SNP	OS12G0163700	Intronic	NA	NA	LOC_Os12g06660	actin, putative, expressed
Chr12	3233390	3233390	T	A	T	SNP	OS12G0163700	Intronic	NA	NA	LOC_Os12g06660	actin, putative, expressed
Chr12	3487110	3487110	G	A	G	SNP	OS12G0168700	Intronic	NA	NA	LOC_Os12g07110	acyl-CoA synthetase protein, putative, expressed
Chr12	3488062	3488062	T	A	T	SNP	OS12G0168700	Intronic	NA	NA	LOC_Os12g07110	acyl-CoA synthetase protein, putative, expressed
Chr12	3754353	3754353	T	A	T	SNP	OS12G0174100	Intronic	NA	NA	LOC_Os12g07540	growth regulator related protein, putative, expressed
Chr12	3755024	3755024	G	A	G	SNP	OS12G0174100	Intronic	NA	NA	LOC_Os12g07540	growth regulator related protein, putative, expressed
Chr12	3755318	3755318	C	A	C	SNP	OS12G0174100	Intronic	NA	NA	LOC_Os12g07540	growth regulator related protein, putative, expressed
Chr12	3755319	3755319	G	A	G	SNP	OS12G0174100	Intronic	NA	NA	LOC_Os12g07540	growth regulator related protein, putative, expressed
Chr12	3756516	3756516	C	A	C	SNP	OS12G0174100	Exonic	CDS	Silent	LOC_Os12g07540	growth regulator related protein, putative, expressed
Chr12	3756840	3756840	C	A	C	SNP	OS12G0174100	Intronic	NA	NA	LOC_Os12g07540	growth regulator related protein, putative, expressed
Chr12	3756854	3756854	G	A	G	SNP	OS12G0174100	Intronic	NA	NA	LOC_Os12g07540	growth regulator related protein, putative, expressed
Chr12	3757170	3757170	G	A	G	SNP	OS12G0174100	Exonic	CDS	Silent	LOC_Os12g07540	growth regulator related protein, putative, expressed
Chr12	3757562	3757562	G	A	G	SNP	OS12G0174100	Exonic	CDS	Silent	LOC_Os12g07540	growth regulator related protein, putative, expressed
Chr12	3757642	3757642	G	A	G	SNP	OS12G0174100	Exonic	CDS	Missense	LOC_Os12g07540	growth regulator related protein, putative, expressed
Chr12	3757982	3757982	C	A	C	SNP	OS12G0174100	Intronic	NA	NA	LOC_Os12g07540	growth regulator related protein, putative, expressed
Chr12	3758038	3758038	T	A	T	SNP	OS12G0174100	Intronic	NA	NA	LOC_Os12g07540	growth regulator related protein, putative, expressed
Chr12	3758265	3758265	G	A	G	SNP	OS12G0174100	Exonic	3UTR	NA	LOC_Os12g07540	growth regulator related protein, putative, expressed
Chr12	4172894	4172894	T	A	T	SNP	OS12G0182200	Intronic	NA	NA	LOC_Os12g08170	2-oxo acid dehydrogenases acyltransferase domain containing protein, expressed
Chr12	4173025	4173025	C	A	C	SNP	OS12G0182200	Intronic	NA	NA	LOC_Os12g08170	2-oxo acid dehydrogenases acyltransferase domain containing protein, expressed
Chr12	4173608	4173608	T	A	T	SNP	OS12G0182200	Intronic	NA	NA	LOC_Os12g08170	2-oxo acid dehydrogenases acyltransferase domain containing protein, expressed
Chr12	4174424	4174424	T	A	T	SNP	OS12G0182200	Intronic	NA	NA	LOC_Os12g08170	2-oxo acid dehydrogenases acyltransferase domain containing protein, expressed
Chr12	4174643	4174643	C	A	C	SNP	OS12G0182200	Intronic	NA	NA	LOC_Os12g08170	2-oxo acid dehydrogenases acyltransferase domain containing protein, expressed
Chr12	4174931	4174931	T	A	T	SNP	OS12G0182200	Intronic	NA	NA	LOC_Os12g08170	2-oxo acid dehydrogenases acyltransferase domain containing protein, expressed
Chr12	5015478	5015478	G	A	G	SNP	OS12G0197100	Intronic	NA	NA	LOC_Os12g09540	phosphoribosylamine--glycine ligase, putative, expressed
Chr12	5015647	5015647	T	A	T	SNP	OS12G0197100	Intronic	NA	NA	LOC_Os12g09540	phosphoribosylamine--glycine ligase, putative, expressed
Chr12	5015760	5015760	G	A	G	SNP	OS12G0197100	Exonic	CDS	Silent	LOC_Os12g09540	phosphoribosylamine--glycine ligase, putative, expressed
Chr12	5070841	5070841	C	A	C	SNP	OS12G0198000	Exonic	3UTR	NA	LOC_Os12g09620	expressed protein
Chr12	5674519	5674519	T	A	T	SNP	OS12G0208900	Exonic	5UTR	NA	LOC_Os12g10630	ZF-HD protein dimerisation region containing protein, expressed
Chr12	5674851	5674851	T	A	T	SNP	OS12G0208900	Intronic	NA	NA	LOC_Os12g10630	ZF-HD protein dimerisation region containing protein, expressed
Chr12	5674992	5674992	C	A	C	SNP	OS12G0208900	Intronic	NA	NA	LOC_Os12g10630	ZF-HD protein dimerisation region containing protein, expressed
Chr12	5675187	5675187	C	A	C	SNP	OS12G0208900	Intronic	NA	NA	LOC_Os12g10630	ZF-HD protein dimerisation region containing protein, expressed
Chr12	19180711	19180711	T	A	T	SNP	OS12G0503000	Exonic	3UTR	NA	LOC_Os12g31860	ureide permease, putative, expressed
Chr12	19181705	19181705	G	A	G	SNP	OS12G0503000	Intronic	NA	NA	LOC_Os12g31860	ureide permease, putative, expressed
Chr12	19181719	19181719	T	A	T	SNP	OS12G0503000	Intronic	NA	NA	LOC_Os12g31860	ureide permease, putative, expressed

Chr12	19181723	19181723	G	A	G	SNP	OS12G0503000	Intronic	NA	NA	LOC_Os12g31860	ureide permease, putative, expressed
Chr12	19181726	19181726	C	A	C	SNP	OS12G0503000	Intronic	NA	NA	LOC_Os12g31860	ureide permease, putative, expressed
Chr12	19181754	19181754	T	A	T	SNP	OS12G0503000	Intronic	NA	NA	LOC_Os12g31860	ureide permease, putative, expressed
Chr12	19181813	19181813	G	A	G	SNP	OS12G0503000	Intronic	NA	NA	LOC_Os12g31860	ureide permease, putative, expressed
Chr12	19181851	19181851	G	A	G	SNP	OS12G0503000	Intronic	NA	NA	LOC_Os12g31860	ureide permease, putative, expressed
Chr12	19182115	19182115	G	A	G	SNP	OS12G0503000	Intronic	NA	NA	LOC_Os12g31860	ureide permease, putative, expressed
Chr12	19183654	19183654	G	A	G	SNP	OS12G0503000	Exonic	CDS	Silent	LOC_Os12g31860	ureide permease, putative, expressed
Chr12	19183738	19183738	G	A	G	SNP	OS12G0503000	Intronic	NA	NA	LOC_Os12g31860	ureide permease, putative, expressed
Chr12	19183895	19183895	G	A	G	SNP	OS12G0503000	Exonic	CDS	Silent	LOC_Os12g31860	ureide permease, putative, expressed
Chr12	19183907	19183907	G	A	G	SNP	OS12G0503000	Exonic	CDS	Silent	LOC_Os12g31860	ureide permease, putative, expressed
Chr12	19184287	19184287	T	A	T	SNP	OS12G0503000	Intronic	NA	NA	LOC_Os12g31860	ureide permease, putative, expressed
Chr12	19185478	19185478	G	A	G	SNP	OS12G0503000	Exonic	5UTR	NA	LOC_Os12g31860	ureide permease, putative, expressed
Chr12	19185589	19185589	T	A	T	SNP	OS12G0503000	Exonic	5UTR	NA	LOC_Os12g31860	ureide permease, putative, expressed
Chr12	19185607	19185607	C	A	C	SNP	OS12G0503000	Exonic	5UTR	NA	LOC_Os12g31860	ureide permease, putative, expressed
Chr12	21490899	21490899	G	A	G	SNP	OS12G0538800	Intronic	NA	NA	LOC_Os12g35350	kelch repeat protein, putative, expressed
Chr12	21491116	21491116	T	A	T	SNP	OS12G0538800	Intronic	NA	NA	LOC_Os12g35350	kelch repeat protein, putative, expressed
Chr12	22133969	22133969	G	A	G	SNP	OS12G0547600	Intronic	NA	NA	LOC_Os12g36110	calmodulin binding protein, putative, expressed
Chr12	22134580	22134580	T	A	T	SNP	OS12G0547600	Exonic	CDS	NA	LOC_Os12g36110	calmodulin binding protein, putative, expressed
Chr12	22134970	22134970	C	A	C	SNP	OS12G0547600	Exonic	CDS	NA	LOC_Os12g36110	calmodulin binding protein, putative, expressed
Chr12	22135872	22135872	G	A	G	SNP	OS12G0547600	Exonic	CDS	Silent	LOC_Os12g36110	calmodulin binding protein, putative, expressed
Chr12	25518536	25518536	G	A	G	SNP	OS12G0604700	Exonic	3UTR	NA	LOC_Os12g41180	LSTK-1-like kinase, putative, expressed
Chr12	25520460	25520460	C	A	C	SNP	OS12G0604700	Intronic	NA	NA	LOC_Os12g41180	LSTK-1-like kinase, putative, expressed
Chr12	26780947	26780947	C	A	C	SNP	OS12G0626400	Intronic	NA	NA	LOC_Os12g43130	phytoene synthase, chloroplast precursor, putative, expressed
Chr12	1517183	1517184	AG	A	AG	INDEL	OS12G0131400	Intronic	NA	NA	LOC_Os12g03740	OsFBX438 - F-box domain containing protein, expressed
Chr12	1518968	1518969	AC	A	AC	INDEL	OS12G0131400	Intronic	NA	NA	LOC_Os12g03740	OsFBX438 - F-box domain containing protein, expressed
Chr12	2216467	2216468	AT	A	AT	INDEL	OS12G0145100	Intronic	NA	NA	LOC_Os12g05050	stem-specific protein TSJT1, putative, expressed
Chr12	2249685	2249686	AT	A	AT	INDEL	OS12G0145700	Exonic	5UTR	NA	LOC_Os12g05110	pyruvate kinase, putative, expressed
Chr12	3754859	3754865	ATCGGTT	A	ATCGGTT	INDEL	OS12G0174100	Intronic	NA	NA	LOC_Os12g07540	growth regulator related protein, putative, expressed
Chr12	3758271	3758274	ACGT	A	ACGT	INDEL	OS12G0174100	Exonic	3UTR	NA	LOC_Os12g07540	growth regulator related protein, putative, expressed
Chr12	5674900	5674905	AGGAGG	A	AGGAGG	INDEL	OS12G0208900	Intronic	NA	NA	LOC_Os12g10630	ZF-HD protein dimerisation region containing protein, expressed
Chr12	19184978	19184981	AAAG	A	AAAG	INDEL	OS12G0503000	Exonic	5UTR	NA	LOC_Os12g31860	ureide permease, putative, expressed
Chr12	77633	77633	G	A	G	SNP	OS12G0101800	Intronic	NA	NA	LOC_Os12g01140	AGC_PVPK_like_kin82y.2 - ACG kinases include homologs to PKA, PKG and PKC, expressed
Chr12	77710	77710	G	A	G	SNP	OS12G0101800	Intronic	NA	NA	LOC_Os12g01140	AGC_PVPK_like_kin82y.2 - ACG kinases include homologs to PKA, PKG and PKC, expressed
Chr12	77731	77731	G	A	G	SNP	OS12G0101800	Intronic	NA	NA	LOC_Os12g01140	AGC_PVPK_like_kin82y.2 - ACG kinases include homologs to PKA, PKG and PKC, expressed
Chr12	77865	77865	G	A	G	SNP	OS12G0101800	Intronic	NA	NA	LOC_Os12g01140	AGC_PVPK_like_kin82y.2 - ACG kinases include homologs to PKA, PKG and PKC, expressed
Chr12	85261	85261	T	A	T	SNP	OS12G0101800	Exonic	CDS	Silent	LOC_Os12g01140	AGC_PVPK_like_kin82y.2 - ACG kinases include homologs to PKA, PKG and PKC, expressed
Chr12	89279	89279	G	A	G	SNP	OS12G0101800	Exonic	CDS	NA	LOC_Os12g01140	AGC_PVPK_like_kin82y.2 - ACG kinases include homologs to PKA, PKG and PKC, expressed
Chr12	274891	274891	G	A	G	SNP	OS12G0105300	Intronic	NA	NA	LOC_Os12g01449	chloroplast unusual positioning protein, putative, expressed
Chr12	276255	276255	G	A	G	SNP	OS12G0105300	Exonic	CDS	Missense	LOC_Os12g01449	chloroplast unusual positioning protein, putative, expressed
Chr12	276455	276455	G	A	G	SNP	OS12G0105300	Exonic	CDS	Silent	LOC_Os12g01449	chloroplast unusual positioning protein, putative, expressed
Chr12	276481	276481	G	A	G	SNP	OS12G0105300	Exonic	CDS	Missense	LOC_Os12g01449	chloroplast unusual positioning protein, putative, expressed
Chr12	1516512	1516512	G	A	G	SNP	OS12G0131400	Intronic	NA	NA	LOC_Os12g03740	OsFBX438 - F-box domain containing protein, expressed
Chr12	1517094	1517094	G	A	G	SNP	OS12G0131400	Intronic	NA	NA	LOC_Os12g03740	OsFBX438 - F-box domain containing protein, expressed
Chr12	1517215	1517215	G	A	G	SNP	OS12G0131400	Intronic	NA	NA	LOC_Os12g03740	OsFBX438 - F-box domain containing protein, expressed
Chr12	1517223	1517223	G	A	G	SNP	OS12G0131400	Intronic	NA	NA	LOC_Os12g03740	OsFBX438 - F-box domain containing protein, expressed
Chr12	1519260	1519260	G	A	G	SNP	OS12G0131400	Exonic	CDS	Missense	LOC_Os12g03740	OsFBX438 - F-box domain containing protein, expressed
Chr12	2836573	2836573	G	A	G	SNP	OS12G0157000	Exonic	CDS	NA	LOC_Os12g06080	expressed protein
Chr12	3483997	3483997	G	A	G	SNP	OS12G0168700	Exonic	5UTR	NA	LOC_Os12g07110	acyl-CoA synthetase protein, putative, expressed
Chr12	3753883	3753883	C	A	C	SNP	OS12G0174100	Exonic	CDS	Silent	LOC_Os12g07540	growth regulator related protein, putative, expressed
Chr12	3757716	3757716	C	A	C	SNP	OS12G0174100	Intronic	NA	NA	LOC_Os12g07540	growth regulator related protein, putative, expressed
Chr12	3758359	3758359	G	A	G	SNP	OS12G0174100	Exonic	3UTR	NA	LOC_Os12g07540	growth regulator related protein, putative, expressed
Chr12	4176544	4176544	G	A	G	SNP	OS12G0182200	Exonic	CDS	Silent	LOC_Os12g08170	2-oxo acid dehydrogenases acyltransferase domain containing protein, expressed
Chr12	4189306	4189306	G	A	G	SNP	OS12G0182500	Exonic	CDS	Missense	LOC_Os12g08200	expressed protein
Chr12	4507397	4507397	G	A	G	SNP	OS12G0189400	Intronic	NA	NA	LOC_Os12g08770	photosystem I reaction center subunit N, chloroplast precursor, putative, expressed
Chr12	4533478	4533478	G	A	G	SNP	OS12G0190000	Exonic	CDS	Silent	LOC_Os12g08810	VTC2, putative, expressed
Chr12	4533595	4533595	G	A	G	SNP	OS12G0190000	Exonic	CDS	Silent	LOC_Os12g08810	VTC2, putative, expressed
Chr12	4534290	4534290	G	A	G	SNP	OS12G0190000	Intronic	NA	NA	LOC_Os12g08810	VTC2, putative, expressed
Chr12	4535096	4535096	G	A	G	SNP	OS12G0190000	Exonic	CDS	Silent	LOC_Os12g08810	VTC2, putative, expressed
Chr12	4536268	4536268	T	A	T	SNP	OS12G0190000	Intronic	NA	NA	LOC_Os12g08810	VTC2, putative, expressed
Chr12	4536731	4536731	C	A	C	SNP	OS12G0190000	Exonic	CDS	Missense	LOC_Os12g08810	VTC2, putative, expressed
Chr12	5071250	5071250	C	A	C	SNP	OS12G0198000	Exonic	CDS	Silent	LOC_Os12g09620	expressed protein
Chr12	5675944	5675944	G	A	G	SNP	OS12G0208900	Exonic	CDS	Silent	LOC_Os12g10630	ZF-HD protein dimerisation region containing protein, expressed
Chr12	5676344	5676344	G	A	G	SNP	OS12G0208900	Exonic	CDS	Missense	LOC_Os12g10630	ZF-HD protein dimerisation region containing protein, expressed
Chr12	9351028	9351028	C	A	C	SNP	OS12G0264500	Exonic	5UTR	NA	LOC_Os12g16350	enoyl-CoA hydratase/isomerase family protein, putative, expressed
Chr12	9353126	9353126	T	A	T	SNP	OS12G0264500	Intronic	NA	NA	LOC_Os12g16350	enoyl-CoA hydratase/isomerase family protein, putative, expressed
Chr12	9917517	9917517	G	A	G	SNP	OS12G0271700	Intronic	NA	NA	LOC_Os12g17320	polyprenyl synthetase, putative, expressed
Chr12	9917560	9917560	T	A	T	SNP	OS12G0271700	Intronic	NA	NA	LOC_Os12g17320	polyprenyl synthetase, putative, expressed
Chr12	9918267	9918267	T	A	T	SNP	OS12G0271700	Intronic	NA	NA	LOC_Os12g17320	polyprenyl synthetase, putative, expressed
Chr12	9918713	9918713	G	A	G	SNP	OS12G0271700	Intronic	NA	NA	LOC_Os12g17320	polyprenyl synthetase, putative, expressed
Chr12	9918786	9918786	T	A	T	SNP	OS12G0271700	Intronic	NA	NA	LOC_Os12g17320	polyprenyl synthetase, putative, expressed
Chr12	9918791	9918791	T	A	T	SNP	OS12G0271700	Intronic	NA	NA	LOC_Os12g17320	polyprenyl synthetase, putative, expressed
Chr12	9918800	9918800	T	A	T	SNP	OS12G0271700	Intronic	NA	NA	LOC_Os12g17320	polyprenyl synthetase, putative, expressed
Chr12	9919486	9919486	T	A	T	SNP	OS12G0271700	Intronic	NA	NA	LOC_Os12g17320	polyprenyl synthetase, putative, expressed
Chr12	9919816	9919816	T	A	T	SNP	OS12G0271700	Intronic	NA	NA	LOC_Os12g17320	polyprenyl synthetase, putative, expressed
Chr12	9920349	9920349	G	A	G	SNP	OS12G0271700	Intronic	NA	NA	LOC_Os12g17320	polyprenyl synthetase, putative, expressed
Chr12	9920486	9920486	T	A	T	SNP	OS12G0271700	Intronic	NA	NA	LOC_Os12g17320	polyprenyl synthetase, putative, expressed
Chr12	9920509	9920509	G	A	G	SNP	OS12G0271700	Intronic	NA	NA	LOC_Os12g17320	polyprenyl synthetase, putative, expressed
Chr12	11204515	11204515	G	A	G	SNP	OS12G0290150	Exonic	CDS	Missense	LOC_Os12g19304	Fe-S metabolism associated domain containing protein, expressed
Chr12	19180890	19180890	C	A	C	SNP	OS12G0503000	Exonic	3UTR	NA	LOC_Os12g31860	ureide permease, putative, expressed
Chr12	19183979	19183979	G	A	G	SNP	OS12G0503000	Exonic	CDS	Silent	LOC_Os12g31860	ureide permease, putative, expressed
Chr12	19184088	19184088	T	A	T	SNP	OS12G0503000	Intronic	NA	NA	LOC_Os12g31860	ureide permease, putative, expressed
Chr12	19184266	19184266	G	A	G	SNP	OS12G0503000	Intronic	NA	NA	LOC_Os12g31860	ureide permease, putative, expressed
Chr12	19185006	19185006	G	A	G	SNP	OS12G0503000	Exonic	5UTR	NA	LOC_Os12g31860	ureide permease, putative, expressed
Chr12	19185123	19185123	G	A	G	SNP	OS12G0503000	Exonic	5UTR	NA	LOC_Os12g31860	ureide permease, putative, expressed
Chr12	19185217	19185217	G	A	G	SNP	OS12G0503000	Exonic	5UTR	NA	LOC_Os12g31860	ureide permease, putative, expressed

Chr12	19185528	19185528	C	A	C	SNP	OS12G0503000	Exonic	5UTR	NA	LOC_Os12g31860	ureide permease, putative, expressed
Chr12	19185810	19185810	G	A	G	SNP	OS12G0503000	Exonic	5UTR	NA	LOC_Os12g31860	ureide permease, putative, expressed
Chr12	19185822	19185822	T	A	T	SNP	OS12G0503000	Exonic	5UTR	NA	LOC_Os12g31860	ureide permease, putative, expressed
Chr12	19687434	19687434	C	A	C	SNP	OS12G0511000	Exonic	CDS	Missense	LOC_Os12g32630	OsFBX448 - F-box domain containing protein, expressed
Chr12	19696308	19696308	C	A	C	SNP	OS12G0511200	Intronic	NA	NA	LOC_Os12g32640	haemolysin-III, putative, expressed
Chr12	19696370	19696370	G	A	G	SNP	OS12G0511200	Intronic	NA	NA	LOC_Os12g32640	haemolysin-III, putative, expressed
Chr12	19696589	19696589	G	A	G	SNP	OS12G0511200	Intronic	NA	NA	LOC_Os12g32640	haemolysin-III, putative, expressed
Chr12	19696998	19696998	G	A	G	SNP	OS12G0511200	Intronic	NA	NA	LOC_Os12g32640	haemolysin-III, putative, expressed
Chr12	19696999	19696999	G	A	G	SNP	OS12G0511200	Intronic	NA	NA	LOC_Os12g32640	haemolysin-III, putative, expressed
Chr12	19697029	19697029	G	A	G	SNP	OS12G0511200	Intronic	NA	NA	LOC_Os12g32640	haemolysin-III, putative, expressed
Chr12	19697285	19697285	G	A	G	SNP	OS12G0511200	Intronic	NA	NA	LOC_Os12g32640	haemolysin-III, putative, expressed
Chr12	19697429	19697429	C	A	C	SNP	OS12G0511200	Exonic	CDS	Missense	LOC_Os12g32640	haemolysin-III, putative, expressed
Chr12	22135776	22135776	G	A	G	SNP	OS12G0547600	Exonic	CDS	Silent	LOC_Os12g36110	calmodulin binding protein, putative, expressed
Chr12	22136663	22136663	G	A	G	SNP	OS12G0547600	Exonic	CDS	Silent	LOC_Os12g36110	calmodulin binding protein, putative, expressed
Chr12	22533201	22533201	C	A	C	SNP	OS12G0554500	Exonic	CDS	Missense	LOC_Os12g36770	lipase class 3 family protein, putative, expressed
Chr12	22534145	22534145	T	A	T	SNP	OS12G0554500	Intronic	NA	NA	LOC_Os12g36770	lipase class 3 family protein, putative, expressed
Chr12	22629336	22629336	T	A	T	SNP	OS12G0556200	Intronic	NA	NA	LOC_Os12g36910	calmodulin binding protein, putative, expressed
Chr12	22629337	22629337	G	A	G	SNP	OS12G0556200	Intronic	NA	NA	LOC_Os12g36910	calmodulin binding protein, putative, expressed
Chr12	22629584	22629584	T	A	T	SNP	OS12G0556200	Intronic	NA	NA	LOC_Os12g36910	calmodulin binding protein, putative, expressed
Chr12	23035501	23035501	T	A	T	SNP	OS12G0562100	Exonic	5UTR	NA	LOC_Os12g37530	expressed protein
Chr12	23808608	23808608	G	A	G	SNP	OS12G0576600	Exonic	CDS	NA	LOC_Os12g38750	nucleotide pyrophosphatase/phosphodiesterase, putative, expressed
Chr12	25325902	25325902	G	A	G	SNP	OS12G0601400	Intronic	NA	NA	LOC_Os12g40900	OsIAA31 - Auxin-responsive Aux/IAA gene family member, expressed
Chr12	26783556	26783556	C	A	C	SNP	OS12G0626400	Exonic	CDS	NA	LOC_Os12g43130	phytoene synthase, chloroplast precursor, putative, expressed
Chr12	27060307	27060307	T	A	T	SNP	OS12G0632000	Exonic	5UTR	NA	LOC_Os12g43600	RNA recognition motif containing protein, expressed
Chr12	88594	88595	AT	A	AT	INDEL	OS12G0101800	Intronic	NA	NA	LOC_Os12g01140	AGC_PVPK_like_kin82y.2 - ACG kinases include homologs to PKA, PKG and PKC, expressed
Chr12	4536193	4536194	AT	A	AT	INDEL	OS12G0190000	Intronic	NA	NA	LOC_Os12g08810	VTC2, putative, expressed
Chr12	5675038	5675039	AT	A	AT	INDEL	OS12G0208900	Intronic	NA	NA	LOC_Os12g10630	ZF-HD protein dimerisation region containing protein, expressed
Chr12	5675071	5675072	AT	A	AT	INDEL	OS12G0208900	Intronic	NA	NA	LOC_Os12g10630	ZF-HD protein dimerisation region containing protein, expressed
Chr12	5675273	5675276	ATAG	A	ATAG	INDEL	OS12G0208900	Intronic	NA	NA	LOC_Os12g10630	ZF-HD protein dimerisation region containing protein, expressed
Chr12	19181548	19181550	AAT	A	AAT	INDEL	OS12G0503000	Intronic	NA	NA	LOC_Os12g31860	ureide permease, putative, expressed
Chr12	19184789	19184790	AT	A	AT	INDEL	OS12G0503000	Exonic	5UTR	NA	LOC_Os12g31860	ureide permease, putative, expressed
Chr12	19185795	19185801	AGAGTGT	A	AGAGTGT	INDEL	OS12G0503000	Exonic	5UTR	NA	LOC_Os12g31860	ureide permease, putative, expressed
Chr12	19185909	19185914	AACCTT	A	AACCTT	INDEL	OS12G0503000	Exonic	5UTR	NA	LOC_Os12g31860	ureide permease, putative, expressed
Chr12	22429572	22429573	AC	A	AC	INDEL	OS12G0552500	Exonic	3UTR	NA	LOC_Os12g36640	universal stress protein domain containing protein, putative, expressed
Chr12	22531512	22531513	AT	A	AT	INDEL	OS12G0554500	Intronic	NA	NA	LOC_Os12g36770	lipase class 3 family protein, putative, expressed
Chr12	22591986	22591995	ATTTTAAGGG	A	ATTTTAAGGG	INDEL	OS12G0555200	Intronic	NA	NA	LOC_Os12g36850	pathogenesis-related Bet v I family protein, putative, expressed
Chr12	22630158	22630159	AT	A	AT	INDEL	OS12G0556200	Intronic	NA	NA	LOC_Os12g36910	calmodulin binding protein, putative, expressed
Chr12	22631938	22631940	ACT	A	ACT	INDEL	OS12G0556200	Intronic	NA	NA	LOC_Os12g36910	calmodulin binding protein, putative, expressed
Chr2	22468467	22468467	C	A	C	SNP	OS02G0582600	Exonic	3UTR	NA	LOC_Os02g37160	heavy metal transport/detoxification protein, putative, expressed
Chr2	27105006	27105006	G	A	G	SNP	OS02G0667500	Exonic	CDS	NA	LOC_Os02g44730	tetracycline transporter protein, putative, expressed
Chr2	27436164	27436164	G	A	G	SNP	OS02G0673700	Exonic	3UTR	NA	LOC_Os02g45200	dof zinc finger domain containing protein, putative, expressed
Chr2	30520558	30520558	G	A	G	SNP	OS02G0732200	Exonic	CDS	Missense	LOC_Os02g49950	spotted leaf 11, putative, expressed
Chr2	30974275	30974275	G	A	G	SNP	OS02G0740600	Exonic	3UTR	NA	LOC_Os02g50710	expressed protein
Chr2	33257071	33257071	G	A	G	SNP	OS02G0783625	Exonic	CDS	Silent	LOC_Os02g54254	saccharopine dehydrogenase, putative, expressed
Chr2	6831263	6831263	T	A	T	SNP	OS02G0222500	Exonic	3UTR	NA	LOC_Os02g12939	expressed protein
Chr2	35349778	35349778	G	A	G	SNP	OS02G0823100	Exonic	5UTR	NA	LOC_Os02g57720	aquaporin protein, putative, expressed
Chr2	919808	919808	T	A	T	SNP	OS02G0117500	Intronic	NA	NA	LOC_Os02g02540	glutamate receptor, putative, expressed
Chr2	1080841	1080841	T	A	T	SNP	OS02G0120800	Exonic	3UTR	NA	LOC_Os02g02840	ras-related protein, putative, expressed
Chr2	1308534	1308534	T	A	T	SNP	OS02G0125100	Intronic	NA	NA	LOC_Os02g03260	3-isopropylmalate dehydratase large subunit 2, putative, expressed
Chr2	5156872	5156872	T	A	T	SNP	OS02G0192500	Intronic	NA	NA	LOC_Os02g09930	CSLA1 - cellulose synthase-like family A; mannan synthase, expressed
Chr2	8836223	8836223	T	A	T	SNP	OS02G0256100	Intronic	NA	NA	LOC_Os02g15690	polygalacturonase, putative, expressed
Chr2	8836565	8836565	T	A	T	SNP	OS02G0256100	Intronic	NA	NA	LOC_Os02g15690	polygalacturonase, putative, expressed
Chr2	18828442	18828442	T	A	T	SNP	OS02G0518100	Intronic	NA	NA	LOC_Os02g31874	DUF803 domain containing, putative, expressed
Chr2	22510390	22510390	T	A	T	SNP	OS02G0583700	Intronic	NA	NA	LOC_Os02g37250	hypothetical protein
Chr2	25778807	25778807	T	A	T	SNP	OS02G0641900	Intronic	NA	NA	LOC_Os02g42870	MYB family transcription factor, putative, expressed
Chr2	28701916	28701916	T	A	T	SNP	OS02G0698000	Exonic	5UTR	NA	LOC_Os02g47020	phosphoribulokinase/Uridine kinase family protein, expressed
Chr2	31806640	31806640	T	A	T	SNP	OS02G0755900	Intronic	NA	NA	LOC_Os02g51930	cytokinin-O-glucosyltransferase 2, putative, expressed
Chr2	32529818	32529818	T	A	T	SNP	OS02G0771100	Intronic	NA	NA	LOC_Os02g53140	COP1, putative, expressed
Chr2	32530680	32530680	T	A	T	SNP	OS02G0771100	Intronic	NA	NA	LOC_Os02g53140	COP1, putative, expressed
Chr2	32531452	32531452	T	A	T	SNP	OS02G0771100	Exonic	CDS	Silent	LOC_Os02g53140	COP1, putative, expressed
Chr2	35082940	35082940	T	A	T	SNP	OS02G0817700	Exonic	CDS	Silent	LOC_Os02g57260	3-ketoacyl-CoA thiolase, peroxisomal precursor, putative, expressed
Chr2	6813719	6813719	T	A	T	SNP	OS02G0222100	Intronic	NA	NA	LOC_Os02g12900	cysteine synthase, putative, expressed
Chr2	14943198	14943198	T	A	T	SNP	OS02G0452500	Intronic	NA	NA	LOC_Os02g25580	Sec1 family transport protein, putative, expressed
Chr2	27046750	27046750	T	A	T	SNP	OS02G0666200	Intronic	NA	NA	LOC_Os02g44630	aquaporin protein, putative, expressed
Chr2	769884	769884	G	A	G	SNP	OS02G0114200	Exonic	CDS	Silent	LOC_Os02g02320	OsSCP6 - Putative Serine Carboxypeptidase homologue, expressed
Chr2	1081493	1081493	G	A	G	SNP	OS02G0120800	Intronic	NA	NA	LOC_Os02g02840	ras-related protein, putative, expressed
Chr2	1081507	1081507	G	A	G	SNP	OS02G0120800	Intronic	NA	NA	LOC_Os02g02840	ras-related protein, putative, expressed
Chr2	1084225	1084225	G	A	G	SNP	OS02G0120800	Exonic	5UTR	NA	LOC_Os02g02840	ras-related protein, putative, expressed
Chr2	1305307	1305307	G	A	G	SNP	OS02G0125100	Intronic	NA	NA	LOC_Os02g03260	3-isopropylmalate dehydratase large subunit 2, putative, expressed
Chr2	1307457	1307457	G	A	G	SNP	OS02G0125100	Intronic	NA	NA	LOC_Os02g03260	3-isopropylmalate dehydratase large subunit 2, putative, expressed
Chr2	1309146	1309146	G	A	G	SNP	OS02G0125100	Exonic	CDS	Silent	LOC_Os02g03260	3-isopropylmalate dehydratase large subunit 2, putative, expressed
Chr2	1449147	1449147	G	A	G	SNP	OS02G0127900	Exonic	3UTR	NA	LOC_Os02g03560	expressed protein
Chr2	3038696	3038696	G	A	G	SNP	OS02G0155400	Exonic	CDS	Silent	LOC_Os02g06090	phytosulfokine receptor precursor, putative, expressed
Chr2	3039066	3039066	G	A	G	SNP	OS02G0155400	Exonic	CDS	Missense	LOC_Os02g06090	phytosulfokine receptor precursor, putative, expressed
Chr2	4390510	4390510	G	A	G	SNP	OS02G0179200	Exonic	CDS	Missense	LOC_Os02g08270	class I glutamine amidotransferase, putative, expressed
Chr2	4888538	4888538	G	A	G	SNP	OS02G0188000	Exonic	3UTR	NA	LOC_Os02g09510	limonoid UDP-glucosyltransferase, putative, expressed
Chr2	6833041	6833041	G	A	G	SNP	OS02G0222500	Exonic	3UTR	NA	LOC_Os02g12939	expressed protein
Chr2	6833086	6833086	G	A	G	SNP	OS02G0222500	Exonic	3UTR	NA	LOC_Os02g12939	expressed protein
Chr2	6833474	6833474	G	A	G	SNP	OS02G0222500	Exonic	3UTR	NA	LOC_Os02g12939	expressed protein
Chr2	6833490	6833490	G	A	G	SNP	OS02G0222500	Exonic	3UTR	NA	LOC_Os02g12939	expressed protein
Chr2	6833493	6833493	G	A	G	SNP	OS02G0222500	Exonic	3UTR	NA	LOC_Os02g12939	expressed protein
Chr2	8835949	8835949	G	A	G	SNP	OS02G0256100	Intronic	NA	NA	LOC_Os02g15690	polygalacturonase, putative, expressed
Chr2	8836007	8836007	G	A	G	SNP	OS02G0256100	Exonic	CDS	Silent	LOC_Os02g15690	polygalacturonase, putative, expressed
Chr2	8836232	8836232	G	A	G	SNP	OS02G0256100	Intronic	NA	NA	LOC_Os02g15690	polygalacturonase, putative, expressed
Chr2	8837490	8837490	G	A	G	SNP	OS02G0256100	Exonic	3UTR	NA	LOC_Os02g15690	polygalacturonase, putative, expressed

Chr2	8979563	8979563	G	A	G	SNP	OS02G0258900	Exonic	CDS	Missense	LOC_Os02g15870	molybdenum cofactor biosynthesis protein 1, putative, expressed
Chr2	16117194	16117194	G	A	G	SNP	OS02G0473200	Intronic	NA	NA	LOC_Os02g27360	aspartic proteinase-like protein 2 precursor, putative, expressed
Chr2	17538656	17538656	G	A	G	SNP	OS02G0498300	Intronic	NA	NA	LOC_Os02g29510	DUF803 domain containing, putative, expressed
Chr2	18345030	18345030	G	A	G	SNP	OS02G0511800	Exonic	CDS	NA	LOC_Os02g30790	SAR DNA-binding protein-like, putative, expressed
Chr2	18345814	18345814	G	A	G	SNP	OS02G0511800	Intronic	NA	NA	LOC_Os02g30790	SAR DNA-binding protein-like, putative, expressed
Chr2	18575482	18575482	G	A	G	SNP	OS02G0515200	Exonic	3UTR	NA	LOC_Os02g31080	hydroxyproline-rich glycoprotein family protein, putative, expressed
Chr2	21932452	21932452	G	A	G	SNP	OS02G0572400	Exonic	CDS	Silent	LOC_Os02g36340	riboflavin biosynthesis protein ribAB, chloroplast precursor, putative, expressed
Chr2	21933699	21933699	G	A	G	SNP	OS02G0572400	Intronic	NA	NA	LOC_Os02g36340	riboflavin biosynthesis protein ribAB, chloroplast precursor, putative, expressed
Chr2	22478607	22478607	G	A	G	SNP	OS02G0582900	Intronic	NA	NA	LOC_Os02g37190	expressed protein
Chr2	22510283	22510283	G	A	G	SNP	OS02G0583700	Intronic	NA	NA	LOC_Os02g37250	hypothetical protein
Chr2	22510300	22510300	G	A	G	SNP	OS02G0583700	Intronic	NA	NA	LOC_Os02g37250	hypothetical protein
Chr2	25779433	25779433	G	A	G	SNP	OS02G0641900	Intronic	NA	NA	LOC_Os02g42870	MYB family transcription factor, putative, expressed
Chr2	26422347	26422347	G	A	G	SNP	OS02G0654700	Exonic	3UTR	NA	LOC_Os02g43790	ethylene-responsive transcription factor, putative, expressed
Chr2	27437770	27437770	G	A	G	SNP	OS02G0673700	Intronic	NA	NA	LOC_Os02g45200	dof zinc finger domain containing protein, putative, expressed
Chr2	28777835	28777835	G	A	G	SNP	OS02G0699700	Exonic	CDS	NA	LOC_Os02g47150	DNA topoisomerase 2, putative, expressed
Chr2	30461154	30461154	G	A	G	SNP	OS02G0731200	Intronic	NA	NA	LOC_Os02g49840	OsMADS57 - MADS-box family gene with MIKCc type-box, expressed
Chr2	30520170	30520170	G	A	G	SNP	OS02G0732200	Intronic	NA	NA	LOC_Os02g49950	spotted leaf 11, putative, expressed
Chr2	31624716	31624716	G	A	G	SNP	OS02G0752300	Intronic	NA	NA	LOC_Os02g51640	expressed protein
Chr2	31807739	31807739	G	A	G	SNP	OS02G0755900	Exonic	3UTR	NA	LOC_Os02g51930	cytokinin-O-glucosyltransferase 2, putative, expressed
Chr2	31959362	31959362	G	A	G	SNP	OS02G0759000	Intronic	NA	NA	LOC_Os02g52190	helix-loop-helix DNA-binding protein, putative, expressed
Chr2	32530728	32530728	G	A	G	SNP	OS02G0771100	Exonic	CDS	Silent	LOC_Os02g53140	COP1, putative, expressed
Chr2	32532069	32532069	G	A	G	SNP	OS02G0771100	Intronic	NA	NA	LOC_Os02g53140	COP1, putative, expressed
Chr2	34044934	34044934	G	A	G	SNP	OS02G0799300	Intronic	NA	NA	LOC_Os02g55590	expressed protein
Chr2	35082301	35082301	G	A	G	SNP	OS02G0817700	Intronic	NA	NA	LOC_Os02g57260	3-ketoacyl-CoA thiolase, peroxisomal precursor, putative, expressed
Chr2	35082657	35082657	G	A	G	SNP	OS02G0817700	Intronic	NA	NA	LOC_Os02g57260	3-ketoacyl-CoA thiolase, peroxisomal precursor, putative, expressed
Chr2	35083006	35083006	G	A	G	SNP	OS02G0817700	Exonic	CDS	Silent	LOC_Os02g57260	3-ketoacyl-CoA thiolase, peroxisomal precursor, putative, expressed
Chr2	35095725	35095725	G	A	G	SNP	OS02G0817900	Intronic	NA	NA	LOC_Os02g57290	cytochrome P450, putative, expressed
Chr2	6834119	6834119	G	A	G	SNP	OS02G0222500	Exonic	3UTR	NA	LOC_Os02g12939	expressed protein
Chr2	6834403	6834403	G	A	G	SNP	OS02G0222500	Exonic	3UTR	NA	LOC_Os02g12939	expressed protein
Chr2	27046778	27046778	G	A	G	SNP	OS02G0666200	Intronic	NA	NA	LOC_Os02g44630	aquaporin protein, putative, expressed
Chr2	30927088	30927088	G	A	G	SNP	OS02G0739700	Intronic	NA	NA	LOC_Os02g50630	E2F family transcription factor protein, putative, expressed
Chr2	30927205	30927205	G	A	G	SNP	OS02G0739700	Intronic	NA	NA	LOC_Os02g50630	E2F family transcription factor protein, putative, expressed
Chr2	1081494	1081494	C	A	C	SNP	OS02G0120800	Intronic	NA	NA	LOC_Os02g02840	ras-related protein, putative, expressed
Chr2	1081495	1081495	C	A	C	SNP	OS02G0120800	Intronic	NA	NA	LOC_Os02g02840	ras-related protein, putative, expressed
Chr2	1308579	1308579	C	A	C	SNP	OS02G0125100	Intronic	NA	NA	LOC_Os02g03260	3-isopropylmalate dehydratase large subunit 2, putative, expressed
Chr2	1309188	1309188	C	A	C	SNP	OS02G0125100	Exonic	CDS	Missense	LOC_Os02g03260	3-isopropylmalate dehydratase large subunit 2, putative, expressed
Chr2	1449095	1449095	C	A	C	SNP	OS02G0127900	Exonic	CDS	NA	LOC_Os02g03560	expressed protein
Chr2	6833060	6833060	C	A	C	SNP	OS02G0222500	Exonic	3UTR	NA	LOC_Os02g12939	expressed protein
Chr2	6833472	6833472	C	A	C	SNP	OS02G0222500	Exonic	3UTR	NA	LOC_Os02g12939	expressed protein
Chr2	18345846	18345846	C	A	C	SNP	OS02G0511800	Intronic	NA	NA	LOC_Os02g30790	SAR DNA-binding protein-like, putative, expressed
Chr2	24737053	24737053	C	A	C	SNP	OS02G0621600	Exonic	3UTR	NA	LOC_Os02g40820	expressed protein
Chr2	28749717	28749717	C	A	C	SNP	OS02G0699000	Exonic	5UTR	NA	LOC_Os02g47090	peptide transporter PTR2, putative, expressed
Chr2	28750129	28750129	C	A	C	SNP	OS02G0699000	Intronic	NA	NA	LOC_Os02g47090	peptide transporter PTR2, putative, expressed
Chr2	28805559	28805559	C	A	C	SNP	OS02G0700100	Exonic	3UTR	NA	LOC_Os02g47180	WD repeat-containing protein, putative, expressed
Chr2	30461951	30461951	C	A	C	SNP	OS02G0731200	Intronic	NA	NA	LOC_Os02g49840	OsMADS57 - MADS-box family gene with MIKCc type-box, expressed
Chr2	31925528	31925528	C	A	C	SNP	OS02G0758100	Intronic	NA	NA	LOC_Os02g52160	LMBR1 integral membrane protein, putative, expressed
Chr2	35098408	35098408	C	A	C	SNP	OS02G0817900	Intronic	NA	NA	LOC_Os02g57290	cytochrome P450, putative, expressed
Chr2	6833256	6833256	C	A	C	SNP	OS02G0222500	Exonic	3UTR	NA	LOC_Os02g12939	expressed protein
Chr2	15641988	15641990	AAT	A	AAT	INDEL	OS02G0465400	Intronic	NA	NA	LOC_Os02g26650	delta14-sterol reductase, putative, expressed
Chr2	5157483	5157484	AC	A	AC	INDEL	OS02G0192500	Intronic	NA	NA	LOC_Os02g09930	CSLA1 - cellulose synthase-like family A; mannan synthase, expressed
Chr2	20749886	20749887	AC	A	AC	INDEL	OS02G0551100	Intronic	NA	NA	LOC_Os02g34600	CAMK_CAMK_like.13 - CAMK includes calcium/calmodulin depedent protein kinases, expressed
Chr2	27045250	27045255	ACTGCT	A	ACTGCT	INDEL	OS02G0666200	Intronic	NA	NA	LOC_Os02g44630	aquaporin protein, putative, expressed
Chr2	4122160	4122161	AG	A	AG	INDEL	OS02G0175400	Intronic	NA	NA	LOC_Os02g07870	ATP synthase, putative, expressed
Chr2	31807735	31807736	AG	A	AG	INDEL	OS02G0755900	Exonic	3UTR	NA	LOC_Os02g51930	cytokinin-O-glucosyltransferase 2, putative, expressed
Chr2	6812199	6812200	AT	A	AT	INDEL	OS02G0222100	Intronic	NA	NA	LOC_Os02g12900	cysteine synthase, putative, expressed
Chr2	6831218	6831219	AT	A	AT	INDEL	OS02G0222500	Exonic	3UTR	NA	LOC_Os02g12939	expressed protein
Chr2	8729584	8729585	AT	A	AT	INDEL	OS02G0254700	Intronic	NA	NA	LOC_Os02g15550	spermidine synthase, putative, expressed
Chr2	11486755	11486756	AT	A	AT	INDEL	OS02G0299300	Intronic	NA	NA	LOC_Os02g19650	hydrolase, alpha/beta fold family domain containing protein, expressed
Chr2	13174206	13174207	AT	A	AT	INDEL	OS02G0327000	Intronic	NA	NA	LOC_Os02g22130	expressed protein
Chr2	22300513	22300514	AT	A	AT	INDEL	OS02G0579600	Intronic	NA	NA	LOC_Os02g36924	OsMADS27 - MADS-box family gene with MIKCc type-box, expressed
Chr2	24567122	24567123	AT	A	AT	INDEL	OS02G0618100	Intronic	NA	NA	LOC_Os02g40500	OsGrx_C2.1 - glutaredoxin subgroup I, expressed
Chr2	26930988	26930989	AT	A	AT	INDEL	OS02G0664000	Intronic	NA	NA	LOC_Os02g44500	glutathione peroxidase, putative, expressed
Chr2	27105237	27105238	AT	A	AT	INDEL	OS02G0667500	Intronic	NA	NA	LOC_Os02g44730	tetracycline transporter protein, putative, expressed
Chr2	29788268	29788269	AT	A	AT	INDEL	OS02G0717600	Intronic	NA	NA	LOC_Os02g48640	expressed protein
Chr2	30457444	30457445	AT	A	AT	INDEL	OS02G0731200	Intronic	NA	NA	LOC_Os02g49840	OsMADS57 - MADS-box family gene with MIKCc type-box, expressed
Chr2	30460781	30460782	AT	A	AT	INDEL	OS02G0731200	Intronic	NA	NA	LOC_Os02g49840	OsMADS57 - MADS-box family gene with MIKCc type-box, expressed
Chr2	29521736	29521739	ATAC	A	ATAC	INDEL	OS02G0712700	Intronic	NA	NA	LOC_Os02g48210	lectin-like protein kinase, putative, expressed
Chr2	79908	79914	ATATGTG	A	ATATGTG	INDEL	OS02G0101500	Exonic	3UTR	NA	LOC_Os02g01150	erythronate-4-phosphate dehydrogenase domain containing protein, expressed
Chr2	29948609	29948611	ATC	A	ATC	INDEL	OS02G0721700	Intronic	NA	NA	LOC_Os02g48980	GPI-anchored protein, putative, expressed
Chr2	13174266	13174270	ATCTC	A	ATCTC	INDEL	OS02G0327000	Intronic	NA	NA	LOC_Os02g22130	expressed protein
Chr2	921786	921786	G	A	G	SNP	OS02G0117500	Exonic	CDS	Missense	LOC_Os02g02540	glutamate receptor, putative, expressed
Chr2	922816	922816	G	A	G	SNP	OS02G0117500	Exonic	CDS	Missense	LOC_Os02g02540	glutamate receptor, putative, expressed
Chr2	923610	923610	G	A	G	SNP	OS02G0117500	Exonic	CDS	Missense	LOC_Os02g02540	glutamate receptor, putative, expressed
Chr2	1396378	1396378	T	A	T	SNP	OS02G0126500	Exonic	3UTR	NA	LOC_Os02g03420	pentatricopeptide repeat-containing protein, putative, expressed
Chr2	3506087	3506087	G	A	G	SNP	OS02G0165100	Exonic	CDS	Silent	LOC_Os02g06930	protein kinase, putative, expressed
Chr2	3506223	3506223	G	A	G	SNP	OS02G0165100	Intronic	NA	NA	LOC_Os02g06930	protein kinase, putative, expressed
Chr2	3507737	3507737	G	A	G	SNP	OS02G0165100	Intronic	NA	NA	LOC_Os02g06930	protein kinase, putative, expressed
Chr2	5155355	5155355	G	A	G	SNP	OS02G0192500	Intronic	NA	NA	LOC_Os02g09930	CSLA1 - cellulose synthase-like family A; mannan synthase, expressed
Chr2	5157152	5157152	C	A	C	SNP	OS02G0192500	Intronic	NA	NA	LOC_Os02g09930	CSLA1 - cellulose synthase-like family A; mannan synthase, expressed
Chr2	5157770	5157770	T	A	T	SNP	OS02G0192500	Intronic	NA	NA	LOC_Os02g09930	CSLA1 - cellulose synthase-like family A; mannan synthase, expressed
Chr2	5194560	5194560	C	A	C	SNP	OS02G0193300	Exonic	3UTR	NA	LOC_Os02g09990	expressed protein
Chr2	5253670	5253670	T	A	T	SNP	OS02G0194200	Exonic	3UTR	NA	LOC_Os02g10080	zinc finger C-x8-C-x5-C-x3-H type family protein, expressed
Chr2	5707716	5707716	G	A	G	SNP	OS02G0202200	Intronic	NA	NA	LOC_Os02g10780	SPX domain containing protein, putative, expressed
Chr2	5707848	5707848	G	A	G	SNP	OS02G0202200	Intronic	NA	NA	LOC_Os02g10780	SPX domain containing protein, putative, expressed
Chr2	5707938	5707938	G	A	G	SNP	OS02G0202200	Intronic	NA	NA	LOC_Os02g10780	SPX domain containing protein, putative, expressed

Chr2	5708294	5708294	C	A	C	SNP	OS02G0202200	Intronic	NA	NA	LOC_Os02g10780	SPX domain containing protein, putative, expressed
Chr2	5760124	5760124	G	A	G	SNP	OS02G0202950	Exonic	CDS	NA	LOC_Os02g10860	bZIP transcription factor domain containing protein, expressed
Chr2	5775839	5775839	C	A	C	SNP	OS02G0203300	Exonic	CDS	Silent	LOC_Os02g10880	UDP-glucoronosyl and UDP-glucosyl transferase domain containing protein, expressed
Chr2	6717779	6717779	T	A	T	SNP	OS02G0220500	Intronic	NA	NA	LOC_Os02g12794	elongation factor 1-gamma, putative, expressed
Chr2	6811359	6811359	C	A	C	SNP	OS02G0222100	Exonic	5UTR	NA	LOC_Os02g12900	cysteine synthase, putative, expressed
Chr2	6811674	6811674	C	A	C	SNP	OS02G0222100	Exonic	5UTR	NA	LOC_Os02g12900	cysteine synthase, putative, expressed
Chr2	6811733	6811733	G	A	G	SNP	OS02G0222100	Exonic	CDS	NA	LOC_Os02g12900	cysteine synthase, putative, expressed
Chr2	6812252	6812252	T	A	T	SNP	OS02G0222100	Intronic	NA	NA	LOC_Os02g12900	cysteine synthase, putative, expressed
Chr2	6812402	6812402	G	A	G	SNP	OS02G0222100	Intronic	NA	NA	LOC_Os02g12900	cysteine synthase, putative, expressed
Chr2	6812453	6812453	G	A	G	SNP	OS02G0222100	Intronic	NA	NA	LOC_Os02g12900	cysteine synthase, putative, expressed
Chr2	6812555	6812555	G	A	G	SNP	OS02G0222100	Exonic	CDS	NA	LOC_Os02g12900	cysteine synthase, putative, expressed
Chr2	6812657	6812657	T	A	T	SNP	OS02G0222100	Intronic	NA	NA	LOC_Os02g12900	cysteine synthase, putative, expressed
Chr2	6813090	6813090	G	A	G	SNP	OS02G0222100	Intronic	NA	NA	LOC_Os02g12900	cysteine synthase, putative, expressed
Chr2	6813150	6813150	T	A	T	SNP	OS02G0222100	Intronic	NA	NA	LOC_Os02g12900	cysteine synthase, putative, expressed
Chr2	6813450	6813450	C	A	C	SNP	OS02G0222100	Intronic	NA	NA	LOC_Os02g12900	cysteine synthase, putative, expressed
Chr2	6813682	6813682	C	A	C	SNP	OS02G0222100	Exonic	3UTR	NA	LOC_Os02g12900	cysteine synthase, putative, expressed
Chr2	6813767	6813767	T	A	T	SNP	OS02G0222100	Intronic	NA	NA	LOC_Os02g12900	cysteine synthase, putative, expressed
Chr2	6813792	6813792	G	A	G	SNP	OS02G0222100	Intronic	NA	NA	LOC_Os02g12900	cysteine synthase, putative, expressed
Chr2	6813930	6813930	G	A	G	SNP	OS02G0222100	Exonic	3UTR	NA	LOC_Os02g12900	cysteine synthase, putative, expressed
Chr2	6831231	6831231	C	A	C	SNP	OS02G0222500	Exonic	3UTR	NA	LOC_Os02g12939	expressed protein
Chr2	6832543	6832543	G	A	G	SNP	OS02G0222500	Exonic	3UTR	NA	LOC_Os02g12939	expressed protein
Chr2	7084278	7084278	C	A	C	SNP	OS02G0226300	Intronic	NA	NA	LOC_Os02g13300	mitochondrial carrier protein, putative, expressed
Chr2	7087283	7087283	C	A	C	SNP	OS02G0226300	Intronic	NA	NA	LOC_Os02g13300	mitochondrial carrier protein, putative, expressed
Chr2	7087348	7087348	T	A	T	SNP	OS02G0226300	Intronic	NA	NA	LOC_Os02g13300	mitochondrial carrier protein, putative, expressed
Chr2	7088341	7088341	T	A	T	SNP	OS02G0226300	Exonic	CDS	Missense	LOC_Os02g13300	mitochondrial carrier protein, putative, expressed
Chr2	7220665	7220665	G	A	G	SNP	OS02G0228300	Exonic	3UTR	NA	LOC_Os02g13510	receptor-like protein kinase 5 precursor, putative, expressed
Chr2	7221975	7221975	G	A	G	SNP	OS02G0228300	Exonic	CDS	Silent	LOC_Os02g13510	receptor-like protein kinase 5 precursor, putative, expressed
Chr2	7224124	7224124	C	A	C	SNP	OS02G0228300	Exonic	CDS	Missense	LOC_Os02g13510	receptor-like protein kinase 5 precursor, putative, expressed
Chr2	7224194	7224194	G	A	G	SNP	OS02G0228300	Exonic	CDS	Missense	LOC_Os02g13510	receptor-like protein kinase 5 precursor, putative, expressed
Chr2	7437814	7437814	G	A	G	SNP	OS02G0231700	Exonic	CDS	Missense	LOC_Os02g13780	receptor-like protein kinase 2 precursor, putative, expressed
Chr2	8728784	8728784	C	A	C	SNP	OS02G0254700	Intronic	NA	NA	LOC_Os02g15550	spermidine synthase, putative, expressed
Chr2	9925682	9925682	G	A	G	SNP	OS02G0272900	Intronic	NA	NA	LOC_Os02g17304	resistance protein, putative, expressed
Chr2	10741618	10741618	G	A	G	SNP	OS02G0285800	Exonic	CDS	Missense	LOC_Os02g18450	GTP-binding protein typA/bipA, putative, expressed
Chr2	10743016	10743016	C	A	C	SNP	OS02G0285800	Intronic	NA	NA	LOC_Os02g18450	GTP-binding protein typA/bipA, putative, expressed
Chr2	10746217	10746217	C	A	C	SNP	OS02G0285800	Intronic	NA	NA	LOC_Os02g18450	GTP-binding protein typA/bipA, putative, expressed
Chr2	11485942	11485942	T	A	T	SNP	OS02G0299300	Intronic	NA	NA	LOC_Os02g19650	hydrolase, alpha/beta fold family domain containing protein, expressed
Chr2	11485944	11485944	C	A	C	SNP	OS02G0299300	Intronic	NA	NA	LOC_Os02g19650	hydrolase, alpha/beta fold family domain containing protein, expressed
Chr2	12870658	12870658	T	A	T	SNP	OS02G0321900	Exonic	CDS	Missense	LOC_Os02g21660	L1P family of ribosomal proteins domain containing protein, expressed
Chr2	12872233	12872233	T	A	T	SNP	OS02G0321900	Exonic	3UTR	NA	LOC_Os02g21660	L1P family of ribosomal proteins domain containing protein, expressed
Chr2	13175398	13175398	G	A	G	SNP	OS02G0327000	Intronic	NA	NA	LOC_Os02g22130	expressed protein
Chr2	14942872	14942872	T	A	T	SNP	OS02G0452500	Exonic	3UTR	NA	LOC_Os02g25580	Sec1 family transport protein, putative, expressed
Chr2	14945743	14945743	T	A	T	SNP	OS02G0452500	Intronic	NA	NA	LOC_Os02g25580	Sec1 family transport protein, putative, expressed
Chr2	14946101	14946101	G	A	G	SNP	OS02G0452500	Intronic	NA	NA	LOC_Os02g25580	Sec1 family transport protein, putative, expressed
Chr2	14947092	14947092	G	A	G	SNP	OS02G0452500	Intronic	NA	NA	LOC_Os02g25580	Sec1 family transport protein, putative, expressed
Chr2	14948836	14948836	G	A	G	SNP	OS02G0452500	Intronic	NA	NA	LOC_Os02g25580	Sec1 family transport protein, putative, expressed
Chr2	14949332	14949332	T	A	T	SNP	OS02G0452500	Intronic	NA	NA	LOC_Os02g25580	Sec1 family transport protein, putative, expressed
Chr2	14954578	14954578	G	A	G	SNP	OS02G0452500	Intronic	NA	NA	LOC_Os02g25580	Sec1 family transport protein, putative, expressed
Chr2	14955870	14955870	G	A	G	SNP	OS02G0452500	Exonic	3UTR	NA	LOC_Os02g25580	Sec1 family transport protein, putative, expressed
Chr2	14957704	14957704	G	A	G	SNP	OS02G0452500	Intronic	NA	NA	LOC_Os02g25580	Sec1 family transport protein, putative, expressed
Chr2	14958324	14958324	G	A	G	SNP	OS02G0452500	Intronic	NA	NA	LOC_Os02g25580	Sec1 family transport protein, putative, expressed
Chr2	14958527	14958527	G	A	G	SNP	OS02G0452500	Intronic	NA	NA	LOC_Os02g25580	Sec1 family transport protein, putative, expressed
Chr2	14958573	14958573	C	A	C	SNP	OS02G0452500	Intronic	NA	NA	LOC_Os02g25580	Sec1 family transport protein, putative, expressed
Chr2	14959355	14959355	G	A	G	SNP	OS02G0452500	Intronic	NA	NA	LOC_Os02g25580	Sec1 family transport protein, putative, expressed
Chr2	14959673	14959673	G	A	G	SNP	OS02G0452500	Intronic	NA	NA	LOC_Os02g25580	Sec1 family transport protein, putative, expressed
Chr2	16011251	16011251	G	A	G	SNP	OS02G0471500	Intronic	NA	NA	LOC_Os02g27220	protein phosphatase 2C, putative, expressed
Chr2	16012297	16012297	G	A	G	SNP	OS02G0471500	Exonic	CDS	Missense	LOC_Os02g27220	protein phosphatase 2C, putative, expressed
Chr2	16119066	16119066	G	A	G	SNP	OS02G0473200	Exonic	CDS	Missense	LOC_Os02g27360	aspartic proteinase-like protein 2 precursor, putative, expressed
Chr2	17534883	17534883	G	A	G	SNP	OS02G0498300	Intronic	NA	NA	LOC_Os02g29510	DUF803 domain containing, putative, expressed
Chr2	17536150	17536150	C	A	C	SNP	OS02G0498300	Intronic	NA	NA	LOC_Os02g29510	DUF803 domain containing, putative, expressed
Chr2	20153450	20153450	C	A	C	SNP	OS02G0542500	Exonic	CDS	Missense	LOC_Os02g33780	serine-aspartate repeat-containing protein I precursor, putative, expressed
Chr2	20153759	20153759	G	A	G	SNP	OS02G0542500	Exonic	CDS	Silent	LOC_Os02g33780	serine-aspartate repeat-containing protein I precursor, putative, expressed
Chr2	20746402	20746402	G	A	G	SNP	OS02G0551100	Exonic	3UTR	NA	LOC_Os02g34600	CAMK_CAMK_like.13 - CAMK includes calcium/calmodulin depedent protein kinases, expressed
Chr2	20747588	20747588	G	A	G	SNP	OS02G0551100	Intronic	NA	NA	LOC_Os02g34600	CAMK_CAMK_like.13 - CAMK includes calcium/calmodulin depedent protein kinases, expressed
Chr2	20748991	20748991	G	A	G	SNP	OS02G0551100	Intronic	NA	NA	LOC_Os02g34600	CAMK_CAMK_like.13 - CAMK includes calcium/calmodulin depedent protein kinases, expressed
Chr2	20812122	20812122	G	A	G	SNP	OS02G0552000	Exonic	CDS	Missense	LOC_Os02g34690	transmembrane 9 superfamily member, putative, expressed
Chr2	20814592	20814592	C	A	C	SNP	OS02G0552000	Exonic	CDS	Missense	LOC_Os02g34690	transmembrane 9 superfamily member, putative, expressed
Chr2	21064302	21064302	G	A	G	SNP	OS02G0556900	Exonic	3UTR	NA	LOC_Os02g35100	DDT domain containing protein, putative, expressed
Chr2	21065208	21065208	T	A	T	SNP	OS02G0556900	Intronic	NA	NA	LOC_Os02g35100	DDT domain containing protein, putative, expressed
Chr2	21065753	21065753	C	A	C	SNP	OS02G0556900	Exonic	CDS	Missense	LOC_Os02g35100	DDT domain containing protein, putative, expressed
Chr2	21067128	21067128	T	A	T	SNP	OS02G0556900	Intronic	NA	NA	LOC_Os02g35100	DDT domain containing protein, putative, expressed
Chr2	21068231	21068231	T	A	T	SNP	OS02G0556900	Intronic	NA	NA	LOC_Os02g35100	DDT domain containing protein, putative, expressed
Chr2	21489567	21489567	C	A	C	SNP	OS02G0565600	Intronic	NA	NA	LOC_Os02g35770	homeobox associated leucine zipper, putative, expressed
Chr2	21810817	21810817	G	A	G	SNP	OS02G0570700	Exonic	CDS	Silent	LOC_Os02g36190	cytochrome P450, putative, expressed
Chr2	22296993	22296993	G	A	G	SNP	OS02G0579600	Intronic	NA	NA	LOC_Os02g36924	OsMADS27 - MADS-box family gene with MIKCc type-box, expressed
Chr2	22298217	22298217	C	A	C	SNP	OS02G0579600	Intronic	NA	NA	LOC_Os02g36924	OsMADS27 - MADS-box family gene with MIKCc type-box, expressed
Chr2	22299324	22299324	G	A	G	SNP	OS02G0579600	Intronic	NA	NA	LOC_Os02g36924	OsMADS27 - MADS-box family gene with MIKCc type-box, expressed
Chr2	22301430	22301430	T	A	T	SNP	OS02G0579600	Intronic	NA	NA	LOC_Os02g36924	OsMADS27 - MADS-box family gene with MIKCc type-box, expressed
Chr2	22301738	22301738	C	A	C	SNP	OS02G0579600	Exonic	3UTR	NA	LOC_Os02g36924	OsMADS27 - MADS-box family gene with MIKCc type-box, expressed
Chr2	23528109	23528109	G	A	G	SNP	OS02G0601300	Intronic	NA	NA	LOC_Os02g38920	glyceraldehyde-3-phosphate dehydrogenase, putative, expressed
Chr2	24570191	24570191	C	A	C	SNP	OS02G0618200	Intronic	NA	NA	LOC_Os02g40510	response regulator receiver domain containing protein, expressed
Chr2	24571316	24571316	G	A	G	SNP	OS02G0618200	Exonic	CDS	Missense	LOC_Os02g40510	response regulator receiver domain containing protein, expressed
Chr2	24691050	24691050	G	A	G	SNP	OS02G0620600	Exonic	CDS	Silent	LOC_Os02g40730	ammonium transporter protein, putative, expressed
Chr2	25779065	25779065	G	A	G	SNP	OS02G0641900	Intronic	NA	NA	LOC_Os02g42870	MYB family transcription factor, putative, expressed
Chr2	25879856	25879856	G	A	G	SNP	OS02G0643800	Exonic	3UTR	NA	LOC_Os02g42990	OsSAUR11 - Auxin-responsive SAUR gene family member, expressed
Chr2	26609031	26609031	G	A	G	SNP	OS02G0658100	Intronic	NA	NA	LOC_Os02g44080	aquaporin protein, putative, expressed

Chr2	26930372	26930372	T	A	T	SNP	OS02G0664000	Exonic	CDS	Missense	LOC_Os02g44500	glutathione peroxidase, putative, expressed
Chr2	26931020	26931020	G	A	G	SNP	OS02G0664000	Intronic	NA	NA	LOC_Os02g44500	glutathione peroxidase, putative, expressed
Chr2	26931679	26931679	T	A	T	SNP	OS02G0664000	Intronic	NA	NA	LOC_Os02g44500	glutathione peroxidase, putative, expressed
Chr2	27046654	27046654	G	A	G	SNP	OS02G0666200	Intronic	NA	NA	LOC_Os02g44630	aquaporin protein, putative, expressed
Chr2	27046724	27046724	T	A	T	SNP	OS02G0666200	Intronic	NA	NA	LOC_Os02g44630	aquaporin protein, putative, expressed
Chr2	27047169	27047169	G	A	G	SNP	OS02G0666200	Exonic	3UTR	NA	LOC_Os02g44630	aquaporin protein, putative, expressed
Chr2	27105523	27105523	T	A	T	SNP	OS02G0667500	Exonic	CDS	NA	LOC_Os02g44730	tetracycline transporter protein, putative, expressed
Chr2	27435534	27435534	C	A	C	SNP	OS02G0673700	Exonic	3UTR	NA	LOC_Os02g45200	dof zinc finger domain containing protein, putative, expressed
Chr2	27436093	27436093	G	A	G	SNP	OS02G0673700	Exonic	3UTR	NA	LOC_Os02g45200	dof zinc finger domain containing protein, putative, expressed
Chr2	29422722	29422722	G	A	G	SNP	OS02G0710300	Exonic	3UTR	NA	LOC_Os02g48060	helix-loop-helix DNA-binding domain containing protein, expressed
Chr2	29520931	29520931	G	A	G	SNP	OS02G0712700	Intronic	NA	NA	LOC_Os02g48210	lectin-like protein kinase, putative, expressed
Chr2	29521045	29521045	G	A	G	SNP	OS02G0712700	Intronic	NA	NA	LOC_Os02g48210	lectin-like protein kinase, putative, expressed
Chr2	29521099	29521099	C	A	C	SNP	OS02G0712700	Intronic	NA	NA	LOC_Os02g48210	lectin-like protein kinase, putative, expressed
Chr2	29521973	29521973	G	A	G	SNP	OS02G0712700	Exonic	CDS	Silent	LOC_Os02g48210	lectin-like protein kinase, putative, expressed
Chr2	29522374	29522374	G	A	G	SNP	OS02G0712700	Exonic	CDS	Missense	LOC_Os02g48210	lectin-like protein kinase, putative, expressed
Chr2	29523235	29523235	G	A	G	SNP	OS02G0712700	Exonic	5UTR	NA	LOC_Os02g48210	lectin-like protein kinase, putative, expressed
Chr2	29604588	29604588	C	A	C	SNP	OS02G0714200	Exonic	3UTR	NA	LOC_Os02g48360	pyrophosphate--fructose 6-phosphate 1-phosphotransferase subunit alpha, putative, expressed
Chr2	29604812	29604812	C	A	C	SNP	OS02G0714200	Intronic	NA	NA	LOC_Os02g48360	pyrophosphate--fructose 6-phosphate 1-phosphotransferase subunit alpha, putative, expressed
Chr2	29605142	29605142	G	A	G	SNP	OS02G0714200	Intronic	NA	NA	LOC_Os02g48360	pyrophosphate--fructose 6-phosphate 1-phosphotransferase subunit alpha, putative, expressed
Chr2	29608055	29608055	G	A	G	SNP	OS02G0714200	Exonic	CDS	Silent	LOC_Os02g48360	pyrophosphate--fructose 6-phosphate 1-phosphotransferase subunit alpha, putative, expressed
Chr2	29608345	29608345	T	A	T	SNP	OS02G0714200	Intronic	NA	NA	LOC_Os02g48360	pyrophosphate--fructose 6-phosphate 1-phosphotransferase subunit alpha, putative, expressed
Chr2	29609533	29609533	G	A	G	SNP	OS02G0714200	Intronic	NA	NA	LOC_Os02g48360	pyrophosphate--fructose 6-phosphate 1-phosphotransferase subunit alpha, putative, expressed
Chr2	29787608	29787608	G	A	G	SNP	OS02G0717600	Exonic	CDS	Silent	LOC_Os02g48640	expressed protein
Chr2	29787939	29787939	T	A	T	SNP	OS02G0717600	Intronic	NA	NA	LOC_Os02g48640	expressed protein
Chr2	29948715	29948715	G	A	G	SNP	OS02G0721700	Intronic	NA	NA	LOC_Os02g48980	GPI-anchored protein, putative, expressed
Chr2	30457457	30457457	C	A	C	SNP	OS02G0731200	Intronic	NA	NA	LOC_Os02g49840	OsMADS57 - MADS-box family gene with MIKCc type-box, expressed
Chr2	30458854	30458854	G	A	G	SNP	OS02G0731200	Intronic	NA	NA	LOC_Os02g49840	OsMADS57 - MADS-box family gene with MIKCc type-box, expressed
Chr2	30459274	30459274	G	A	G	SNP	OS02G0731200	Intronic	NA	NA	LOC_Os02g49840	OsMADS57 - MADS-box family gene with MIKCc type-box, expressed
Chr2	30461268	30461268	T	A	T	SNP	OS02G0731200	Intronic	NA	NA	LOC_Os02g49840	OsMADS57 - MADS-box family gene with MIKCc type-box, expressed
Chr2	30519843	30519843	G	A	G	SNP	OS02G0732200	Intronic	NA	NA	LOC_Os02g49950	spotted leaf 11, putative, expressed
Chr2	30520035	30520035	G	A	G	SNP	OS02G0732200	Intronic	NA	NA	LOC_Os02g49950	spotted leaf 11, putative, expressed
Chr2	30899595	30899595	T	A	T	SNP	OS02G0739400	Exonic	3UTR	NA	LOC_Os02g50600	glycosyl transferase 8 domain containing protein, putative, expressed
Chr2	30930328	30930328	T	A	T	SNP	OS02G0739900	Exonic	3UTR	NA	LOC_Os02g50640	HEAT repeat family protein, putative, expressed
Chr2	30930592	30930592	G	A	G	SNP	OS02G0739900	Exonic	3UTR	NA	LOC_Os02g50640	HEAT repeat family protein, putative, expressed
Chr2	30930813	30930813	G	A	G	SNP	OS02G0739900	Intronic	NA	NA	LOC_Os02g50640	HEAT repeat family protein, putative, expressed
Chr2	30930839	30930839	G	A	G	SNP	OS02G0739900	Intronic	NA	NA	LOC_Os02g50640	HEAT repeat family protein, putative, expressed
Chr2	30931835	30931835	T	A	T	SNP	OS02G0739900	Intronic	NA	NA	LOC_Os02g50640	HEAT repeat family protein, putative, expressed
Chr2	30974232	30974232	G	A	G	SNP	OS02G0740600	Exonic	3UTR	NA	LOC_Os02g50710	expressed protein
Chr2	30974238	30974238	C	A	C	SNP	OS02G0740600	Exonic	3UTR	NA	LOC_Os02g50710	expressed protein
Chr2	31161715	31161715	G	A	G	SNP	OS02G0743400	Exonic	CDS	Silent	LOC_Os02g50960	auxin efflux carrier component, putative, expressed
Chr2	31407321	31407321	G	A	G	SNP	OS02G0747500	Exonic	5UTR	NA	LOC_Os02g51290	HVA22, putative, expressed
Chr2	31407421	31407421	T	A	T	SNP	OS02G0747500	Intronic	NA	NA	LOC_Os02g51290	HVA22, putative, expressed
Chr2	31408974	31408974	G	A	G	SNP	OS02G0747500	Exonic	CDS	Missense	LOC_Os02g51290	HVA22, putative, expressed
Chr2	31409448	31409448	T	A	T	SNP	OS02G0747500	Exonic	3UTR	NA	LOC_Os02g51290	HVA22, putative, expressed
Chr2	31435335	31435335	C	A	C	SNP	OS02G0748100	Intronic	NA	NA	LOC_Os02g51330	MAG2, putative, expressed
Chr2	31435391	31435391	G	A	G	SNP	OS02G0748100	Intronic	NA	NA	LOC_Os02g51330	MAG2, putative, expressed
Chr2	31444774	31444774	G	A	G	SNP	OS02G0748300	Exonic	3UTR	NA	LOC_Os02g51350	OsFBK10 - F-box domain and kelch repeat containing protein, expressed
Chr2	31446989	31446989	G	A	G	SNP	OS02G0748300	Intronic	NA	NA	LOC_Os02g51350	OsFBK10 - F-box domain and kelch repeat containing protein, expressed
Chr2	31626574	31626574	G	A	G	SNP	OS02G0752300	Exonic	CDS	Missense	LOC_Os02g51640	expressed protein
Chr2	31664853	31664853	G	A	G	SNP	OS02G0753000	Exonic	CDS	Missense	LOC_Os02g51680	uncharacterized glycosyl hydrolase Rv2006/MT2062, putative, expressed
Chr2	31926004	31926004	T	A	T	SNP	OS02G0758100	Intronic	NA	NA	LOC_Os02g52160	LMBR1 integral membrane protein, putative, expressed
Chr2	31927171	31927171	G	A	G	SNP	OS02G0758100	Intronic	NA	NA	LOC_Os02g52160	LMBR1 integral membrane protein, putative, expressed
Chr2	31929387	31929387	G	A	G	SNP	OS02G0758100	Exonic	CDS	NA	LOC_Os02g52160	LMBR1 integral membrane protein, putative, expressed
Chr2	32052217	32052217	G	A	G	SNP	OS02G0761100	Exonic	3UTR	NA	LOC_Os02g52360	peptidyl-prolyl cis-trans isomerase CYP40, putative, expressed
Chr2	32052599	32052599	G	A	G	SNP	OS02G0761200	Exonic	3UTR	NA	LOC_Os02g52370	armadillo/beta-catenin repeat family protein, putative, expressed
Chr2	32055293	32055293	G	A	G	SNP	OS02G0761200	Exonic	CDS	Missense	LOC_Os02g52370	armadillo/beta-catenin repeat family protein, putative, expressed
Chr2	32095946	32095946	G	A	G	SNP	OS02G0762200	Exonic	CDS	Missense	LOC_Os02g52460	zinc knuckle domain containing protein, expressed
Chr2	32096126	32096126	G	A	G	SNP	OS02G0762200	Exonic	CDS	Silent	LOC_Os02g52460	zinc knuckle domain containing protein, expressed
Chr2	32096911	32096911	G	A	G	SNP	OS02G0762200	Intronic	NA	NA	LOC_Os02g52460	zinc knuckle domain containing protein, expressed
Chr2	32097105	32097105	G	A	G	SNP	OS02G0762200	Intronic	NA	NA	LOC_Os02g52460	zinc knuckle domain containing protein, expressed
Chr2	32097926	32097926	G	A	G	SNP	OS02G0762200	Intronic	NA	NA	LOC_Os02g52460	zinc knuckle domain containing protein, expressed
Chr2	32098029	32098029	C	A	C	SNP	OS02G0762200	Intronic	NA	NA	LOC_Os02g52460	zinc knuckle domain containing protein, expressed
Chr2	32098289	32098289	G	A	G	SNP	OS02G0762200	Exonic	CDS	Silent	LOC_Os02g52460	zinc knuckle domain containing protein, expressed
Chr2	32098674	32098674	G	A	G	SNP	OS02G0762200	Exonic	5UTR	NA	LOC_Os02g52460	zinc knuckle domain containing protein, expressed
Chr2	32119164	32119164	G	A	G	SNP	OS02G0762600	Exonic	3UTR	NA	LOC_Os02g52490	expressed protein
Chr2	33197544	33197544	T	A	T	SNP	OS02G0782600	Intronic	NA	NA	LOC_Os02g54150	SAC domain containing protein, putative, expressed
Chr2	33253980	33253980	C	A	C	SNP	OS02G0783625	Intronic	NA	NA	LOC_Os02g54254	saccharopine dehydrogenase, putative, expressed
Chr2	33776344	33776344	G	A	G	SNP	OS02G0794600	Exonic	CDS	Silent	LOC_Os02g55134	cytochrome c oxidase copper chaperone, putative, expressed
Chr2	34635981	34635981	T	A	T	SNP	OS02G0810200	Intronic	NA	NA	LOC_Os02g56540	kinesin motor domain containing protein, putative, expressed
Chr2	34834955	34834955	G	A	G	SNP	OS02G0813350	Intronic	NA	NA	LOC_Os02g56840	OsFBX70 - F-box domain containing protein, expressed
Chr2	35094096	35094096	C	A	C	SNP	OS02G0817900	Intronic	NA	NA	LOC_Os02g57290	cytochrome P450, putative, expressed
Chr2	839096	839096	G	A	G	SNP	OS02G0115900	Exonic	CDS	Silent	LOC_Os02g02410	DnaK family protein, putative, expressed
Chr2	1083954	1083954	C	A	C	SNP	OS02G0120800	Exonic	5UTR	NA	LOC_Os02g02840	ras-related protein, putative, expressed
Chr2	1309468	1309468	G	A	G	SNP	OS02G0125100	Exonic	CDS	Silent	LOC_Os02g03260	3-isopropylmalate dehydratase large subunit 2, putative, expressed
Chr2	1447302	1447302	G	A	G	SNP	OS02G0127900	Exonic	5UTR	NA	LOC_Os02g03560	expressed protein
Chr2	2076824	2076824	G	A	G	SNP	OS02G0139000	Exonic	CDS	Missense	LOC_Os02g04640	Myb-like DNA-binding domain containing protein, putative, expressed
Chr2	4890082	4890082	G	A	G	SNP	OS02G0188000	Exonic	CDS	Silent	LOC_Os02g09510	limonoid UDP-glucosyltransferase, putative, expressed
Chr2	5158497	5158497	G	A	G	SNP	OS02G0192500	Exonic	CDS	Silent	LOC_Os02g09930	CSLA1 - cellulose synthase-like family A; mannan synthase, expressed
Chr2	5250144	5250144	G	A	G	SNP	OS02G0194200	Exonic	5UTR	NA	LOC_Os02g10080	zinc finger C-x8-C-x5-C-x3-H type family protein, expressed
Chr2	5250179	5250179	C	A	C	SNP	OS02G0194200	Exonic	5UTR	NA	LOC_Os02g10080	zinc finger C-x8-C-x5-C-x3-H type family protein, expressed
Chr2	5250667	5250667	G	A	G	SNP	OS02G0194200	Exonic	5UTR	NA	LOC_Os02g10080	zinc finger C-x8-C-x5-C-x3-H type family protein, expressed
Chr2	5252034	5252034	C	A	C	SNP	OS02G0194200	Intronic	NA	NA	LOC_Os02g10080	zinc finger C-x8-C-x5-C-x3-H type family protein, expressed
Chr2	5776183	5776183	G	A	G	SNP	OS02G0203300	Exonic	CDS	Missense	LOC_Os02g10880	UDP-glucoronosyl and UDP-glucosyl transferase domain containing protein, expressed
Chr2	6812755	6812755	C	A	C	SNP	OS02G0222100	Exonic	3UTR	NA	LOC_Os02g12900	cysteine synthase, putative, expressed
Chr2	6812841	6812841	G	A	G	SNP	OS02G0222100	Exonic	3UTR	NA	LOC_Os02g12900	cysteine synthase, putative, expressed

Chr2	6832806	6832806	G	A	G	SNP	OS02G0222500	Exonic	3UTR	NA	LOC_Os02g12939	expressed protein
Chr2	6834080	6834080	G	A	G	SNP	OS02G0222500	Exonic	3UTR	NA	LOC_Os02g12939	expressed protein
Chr2	6834263	6834263	C	A	C	SNP	OS02G0222500	Exonic	3UTR	NA	LOC_Os02g12939	expressed protein
Chr2	6834383	6834383	C	A	C	SNP	OS02G0222500	Exonic	3UTR	NA	LOC_Os02g12939	expressed protein
Chr2	7220840	7220840	C	A	C	SNP	OS02G0228300	Exonic	3UTR	NA	LOC_Os02g13510	receptor-like protein kinase 5 precursor, putative, expressed
Chr2	7220877	7220877	G	A	G	SNP	OS02G0228300	Exonic	3UTR	NA	LOC_Os02g13510	receptor-like protein kinase 5 precursor, putative, expressed
Chr2	7221382	7221382	G	A	G	SNP	OS02G0228300	Exonic	CDS	Silent	LOC_Os02g13510	receptor-like protein kinase 5 precursor, putative, expressed
Chr2	7222398	7222398	G	A	G	SNP	OS02G0228300	Exonic	CDS	Silent	LOC_Os02g13510	receptor-like protein kinase 5 precursor, putative, expressed
Chr2	7222401	7222401	G	A	G	SNP	OS02G0228300	Exonic	CDS	Silent	LOC_Os02g13510	receptor-like protein kinase 5 precursor, putative, expressed
Chr2	7222419	7222419	G	A	G	SNP	OS02G0228300	Exonic	CDS	Silent	LOC_Os02g13510	receptor-like protein kinase 5 precursor, putative, expressed
Chr2	7222452	7222452	G	A	G	SNP	OS02G0228300	Exonic	CDS	Silent	LOC_Os02g13510	receptor-like protein kinase 5 precursor, putative, expressed
Chr2	7222514	7222514	C	A	C	SNP	OS02G0228300	Exonic	CDS	Missense	LOC_Os02g13510	receptor-like protein kinase 5 precursor, putative, expressed
Chr2	7224036	7224036	G	A	G	SNP	OS02G0228300	Exonic	CDS	Silent	LOC_Os02g13510	receptor-like protein kinase 5 precursor, putative, expressed
Chr2	7465209	7465209	G	A	G	SNP	OS02G0232000	Exonic	3UTR	NA	LOC_Os02g13800	HSF-type DNA-binding domain containing protein, expressed
Chr2	8834554	8834554	G	A	G	SNP	OS02G0256100	Exonic	5UTR	NA	LOC_Os02g15690	polygalacturonase, putative, expressed
Chr2	8834689	8834689	G	A	G	SNP	OS02G0256100	Exonic	CDS	Missense	LOC_Os02g15690	polygalacturonase, putative, expressed
Chr2	8835140	8835140	G	A	G	SNP	OS02G0256100	Exonic	CDS	Missense	LOC_Os02g15690	polygalacturonase, putative, expressed
Chr2	8836201	8836201	G	A	G	SNP	OS02G0256100	Intronic	NA	NA	LOC_Os02g15690	polygalacturonase, putative, expressed
Chr2	8837190	8837190	T	A	T	SNP	OS02G0256100	Exonic	CDS	Silent	LOC_Os02g15690	polygalacturonase, putative, expressed
Chr2	12466204	12466204	G	A	G	SNP	OS02G0314600	Exonic	3UTR	NA	LOC_Os02g21040	aspartic proteinase nepenthesin precursor, putative, expressed
Chr2	13097764	13097764	G	A	G	SNP	OS02G0325600	Exonic	CDS	Silent	LOC_Os02g22020	MYB family transcription factor, putative, expressed
Chr2	14962010	14962010	G	A	G	SNP	OS02G0452500	Exonic	5UTR	NA	LOC_Os02g25580	Sec1 family transport protein, putative, expressed
Chr2	15735108	15735108	G	A	G	SNP	OS02G0467600	Exonic	CDS	Silent	LOC_Os02g26810	cytochrome P450, putative, expressed
Chr2	16010604	16010604	G	A	G	SNP	OS02G0471500	Exonic	CDS	Missense	LOC_Os02g27220	protein phosphatase 2C, putative, expressed
Chr2	16119952	16119952	C	A	C	SNP	OS02G0473200	Intronic	NA	NA	LOC_Os02g27360	aspartic proteinase-like protein 2 precursor, putative, expressed
Chr2	17159604	17159604	G	A	G	SNP	OS02G0491400	Intronic	NA	NA	LOC_Os02g28980	peptidyl-prolyl isomerase, putative, expressed
Chr2	18576799	18576799	G	A	G	SNP	OS02G0515200	Exonic	CDS	Silent	LOC_Os02g31080	hydroxyproline-rich glycoprotein family protein, putative, expressed
Chr2	18831501	18831501	G	A	G	SNP	OS02G0518100	Intronic	NA	NA	LOC_Os02g31874	DUF803 domain containing, putative, expressed
Chr2	20152908	20152908	G	A	G	SNP	OS02G0542500	Exonic	CDS	Missense	LOC_Os02g33780	serine-aspartate repeat-containing protein I precursor, putative, expressed
Chr2	20153726	20153726	C	A	C	SNP	OS02G0542500	Exonic	CDS	Missense	LOC_Os02g33780	serine-aspartate repeat-containing protein I precursor, putative, expressed
Chr2	20154119	20154119	T	A	T	SNP	OS02G0542500	Exonic	3UTR	NA	LOC_Os02g33780	serine-aspartate repeat-containing protein I precursor, putative, expressed
Chr2	20747128	20747128	T	A	T	SNP	OS02G0551100	Intronic	NA	NA	LOC_Os02g34600	CAMK_CAMK_like.13 - CAMK includes calcium/calmodulin depedent protein kinases, expressed
Chr2	21740847	21740847	G	A	G	SNP	OS02G0569900	Exonic	CDS	Missense	LOC_Os02g36110	cytochrome P450, putative, expressed
Chr2	23993847	23993847	G	A	G	SNP	OS02G0610600	Exonic	CDS	Missense	LOC_Os02g39720	splicing factor, arginine/serine-rich 7, putative, expressed
Chr2	23996297	23996297	G	A	G	SNP	OS02G0610600	Exonic	5UTR	NA	LOC_Os02g39720	splicing factor, arginine/serine-rich 7, putative, expressed
Chr2	24350847	24350847	G	A	G	SNP	OS02G0615800	Exonic	5UTR	NA	LOC_Os02g40240	receptor kinase, putative, expressed
Chr2	24351167	24351167	C	A	C	SNP	OS02G0615800	Exonic	CDS	Missense	LOC_Os02g40240	receptor kinase, putative, expressed
Chr2	24351234	24351234	C	A	C	SNP	OS02G0615800	Exonic	CDS	Missense	LOC_Os02g40240	receptor kinase, putative, expressed
Chr2	24351386	24351386	G	A	G	SNP	OS02G0615800	Exonic	CDS	Missense	LOC_Os02g40240	receptor kinase, putative, expressed
Chr2	24352834	24352834	G	A	G	SNP	OS02G0615800	Exonic	CDS	Silent	LOC_Os02g40240	receptor kinase, putative, expressed
Chr2	24353005	24353005	G	A	G	SNP	OS02G0615800	Exonic	CDS	Missense	LOC_Os02g40240	receptor kinase, putative, expressed
Chr2	24353015	24353015	G	A	G	SNP	OS02G0615800	Exonic	CDS	Missense	LOC_Os02g40240	receptor kinase, putative, expressed
Chr2	24353149	24353149	T	A	T	SNP	OS02G0615800	Exonic	CDS	Silent	LOC_Os02g40240	receptor kinase, putative, expressed
Chr2	24353167	24353167	G	A	G	SNP	OS02G0615800	Exonic	CDS	Silent	LOC_Os02g40240	receptor kinase, putative, expressed
Chr2	24353269	24353269	T	A	T	SNP	OS02G0615800	Exonic	CDS	Silent	LOC_Os02g40240	receptor kinase, putative, expressed
Chr2	24353338	24353338	G	A	G	SNP	OS02G0615800	Exonic	CDS	Silent	LOC_Os02g40240	receptor kinase, putative, expressed
Chr2	24353868	24353868	G	A	G	SNP	OS02G0615800	Exonic	CDS	Missense	LOC_Os02g40240	receptor kinase, putative, expressed
Chr2	24353908	24353908	G	A	G	SNP	OS02G0615800	Exonic	CDS	Silent	LOC_Os02g40240	receptor kinase, putative, expressed
Chr2	24571733	24571733	G	A	G	SNP	OS02G0618200	Exonic	CDS	Missense	LOC_Os02g40510	response regulator receiver domain containing protein, expressed
Chr2	24879664	24879664	G	A	G	SNP	OS02G0624300	Exonic	CDS	Silent	LOC_Os02g41510	MYB family transcription factor, putative, expressed
Chr2	24904687	24904687	G	A	G	SNP	OS02G0624400	Intronic	NA	NA	LOC_Os02g41520	glycosyl transferase 8 domain containing protein, putative, expressed
Chr2	25446704	25446704	T	A	T	SNP	OS02G0634700	Intronic	NA	NA	LOC_Os02g42310	OsSCP8 - Putative Serine Carboxypeptidase homologue, expressed
Chr2	25780090	25780090	C	A	C	SNP	OS02G0641900	Exonic	CDS	Missense	LOC_Os02g42870	MYB family transcription factor, putative, expressed
Chr2	25879181	25879181	C	A	C	SNP	OS02G0643800	Exonic	CDS	Missense	LOC_Os02g42990	OsSAUR11 - Auxin-responsive SAUR gene family member, expressed
Chr2	26410608	26410608	C	A	C	SNP	OS02G0654600	Exonic	CDS	Silent	LOC_Os02g43770	DUF630/DUF632 domains containing protein, putative, expressed
Chr2	26411762	26411762	C	A	C	SNP	OS02G0654600	Exonic	CDS	Missense	LOC_Os02g43770	DUF630/DUF632 domains containing protein, putative, expressed
Chr2	26422728	26422728	C	A	C	SNP	OS02G0654700	Exonic	CDS	Missense	LOC_Os02g43790	ethylene-responsive transcription factor, putative, expressed
Chr2	27046848	27046848	T	A	T	SNP	OS02G0666200	Intronic	NA	NA	LOC_Os02g44630	aquaporin protein, putative, expressed
Chr2	27249337	27249337	C	A	C	SNP	OS02G0671100	Exonic	CDS	Missense	LOC_Os02g44990	OsFBDUF13 - F-box and DUF domain containing protein, expressed
Chr2	27249425	27249425	G	A	G	SNP	OS02G0671100	Exonic	CDS	Silent	LOC_Os02g44990	OsFBDUF13 - F-box and DUF domain containing protein, expressed
Chr2	27436796	27436796	G	A	G	SNP	OS02G0673700	Exonic	CDS	Missense	LOC_Os02g45200	dof zinc finger domain containing protein, putative, expressed
Chr2	27436797	27436797	C	A	C	SNP	OS02G0673700	Exonic	CDS	Silent	LOC_Os02g45200	dof zinc finger domain containing protein, putative, expressed
Chr2	28750749	28750749	C	A	C	SNP	OS02G0699000	Intronic	NA	NA	LOC_Os02g47090	peptide transporter PTR2, putative, expressed
Chr2	28751006	28751006	G	A	G	SNP	OS02G0699000	Exonic	CDS	Missense	LOC_Os02g47090	peptide transporter PTR2, putative, expressed
Chr2	28751738	28751738	G	A	G	SNP	OS02G0699000	Exonic	CDS	Missense	LOC_Os02g47090	peptide transporter PTR2, putative, expressed
Chr2	29421718	29421718	G	A	G	SNP	OS02G0710300	Exonic	CDS	Silent	LOC_Os02g48060	helix-loop-helix DNA-binding domain containing protein, expressed
Chr2	29522081	29522081	G	A	G	SNP	OS02G0712700	Exonic	CDS	Silent	LOC_Os02g48210	lectin-like protein kinase, putative, expressed
Chr2	29522224	29522224	C	A	C	SNP	OS02G0712700	Exonic	CDS	Missense	LOC_Os02g48210	lectin-like protein kinase, putative, expressed
Chr2	29522391	29522391	G	A	G	SNP	OS02G0712700	Exonic	CDS	Missense	LOC_Os02g48210	lectin-like protein kinase, putative, expressed
Chr2	29523018	29523018	G	A	G	SNP	OS02G0712700	Exonic	CDS	Missense	LOC_Os02g48210	lectin-like protein kinase, putative, expressed
Chr2	29523189	29523189	G	A	G	SNP	OS02G0712700	Exonic	5UTR	NA	LOC_Os02g48210	lectin-like protein kinase, putative, expressed
Chr2	29523194	29523194	G	A	G	SNP	OS02G0712700	Exonic	5UTR	NA	LOC_Os02g48210	lectin-like protein kinase, putative, expressed
Chr2	30519777	30519777	G	A	G	SNP	OS02G0732200	Intronic	NA	NA	LOC_Os02g49950	spotted leaf 11, putative, expressed
Chr2	30926915	30926915	G	A	G	SNP	OS02G0739700	Intronic	NA	NA	LOC_Os02g50630	E2F family transcription factor protein, putative, expressed
Chr2	31626404	31626404	G	A	G	SNP	OS02G0752300	Exonic	CDS	Missense	LOC_Os02g51640	expressed protein
Chr2	31807162	31807162	G	A	G	SNP	OS02G0755900	Exonic	CDS	NA	LOC_Os02g51930	cytokinin-O-glucosyltransferase 2, putative, expressed
Chr2	31927428	31927428	C	A	C	SNP	OS02G0758100	Intronic	NA	NA	LOC_Os02g52160	LMBR1 integral membrane protein, putative, expressed
Chr2	31968483	31968483	G	A	G	SNP	OS02G0759400	Exonic	CDS	Missense	LOC_Os02g52210	zinc finger, C3HC4 type domain containing protein, expressed
Chr2	31969492	31969492	G	A	G	SNP	OS02G0759400	Exonic	3UTR	NA	LOC_Os02g52210	zinc finger, C3HC4 type domain containing protein, expressed
Chr2	32098582	32098582	G	A	G	SNP	OS02G0762200	Exonic	CDS	Missense	LOC_Os02g52460	zinc knuckle domain containing protein, expressed
Chr2	32528672	32528672	G	A	G	SNP	OS02G0771100	Intronic	NA	NA	LOC_Os02g53140	COP1, putative, expressed
Chr2	32532968	32532968	G	A	G	SNP	OS02G0771100	Intronic	NA	NA	LOC_Os02g53140	COP1, putative, expressed
Chr2	33954118	33954118	C	A	C	SNP	OS02G0797600	Exonic	3UTR	NA	LOC_Os02g55430	alginate regulatory protein AlgP, putative, expressed
Chr2	34042294	34042294	G	A	G	SNP	OS02G0799300	Intronic	NA	NA	LOC_Os02g55590	expressed protein
Chr2	34227259	34227259	T	A	T	SNP	OS02G0802700	Exonic	3UTR	NA	LOC_Os02g55910	monogalactosyldiacylglycerol synthase, putative, expressed

Chr2	34349137	34349137	C	A	C	SNP	OS02G0805100	Intronic	NA	NA	LOC_Os02g56120	OsIAA9 - Auxin-responsive Aux/IAA gene family member, expressed
Chr2	34673890	34673890	G	A	G	SNP	OS02G0810900	Intronic	NA	NA	LOC_Os02g56600	no apical meristem protein, putative, expressed
Chr2	34675019	34675019	G	A	G	SNP	OS02G0810900	Exonic	CDS	Missense	LOC_Os02g56600	no apical meristem protein, putative, expressed
Chr2	34835086	34835086	G	A	G	SNP	OS02G0813350	Intronic	NA	NA	LOC_Os02g56840	OsFBX70 - F-box domain containing protein, expressed
Chr2	35083255	35083255	T	A	T	SNP	OS02G0817700	Intronic	NA	NA	LOC_Os02g57260	3-ketoacyl-CoA thiolase, peroxisomal precursor, putative, expressed
Chr2	35083938	35083938	G	A	G	SNP	OS02G0817700	Exonic	CDS	Silent	LOC_Os02g57260	3-ketoacyl-CoA thiolase, peroxisomal precursor, putative, expressed
Chr2	35094044	35094044	G	A	G	SNP	OS02G0817900	Intronic	NA	NA	LOC_Os02g57290	cytochrome P450, putative, expressed
Chr2	35350145	35350145	C	A	C	SNP	OS02G0823100	Exonic	CDS	Silent	LOC_Os02g57720	aquaporin protein, putative, expressed
Chr2	826407	826423	AGGAGTACTGCGGCTTC	A	AGGAGTACTGCGGCTTC	INDEL	OS02G0115700	Exonic	5UTR	NA	LOC_Os02g02400	catalase isozyme A, putative, expressed
Chr2	1081034	1081035	AG	A	AG	INDEL	OS02G0120800	Intronic	NA	NA	LOC_Os02g02840	ras-related protein, putative, expressed
Chr2	1083649	1083650	AT	A	AT	INDEL	OS02G0120800	Intronic	NA	NA	LOC_Os02g02840	ras-related protein, putative, expressed
Chr2	1447176	1447179	ACCT	A	ACCT	INDEL	OS02G0127900	Exonic	5UTR	NA	LOC_Os02g03560	expressed protein
Chr2	4009890	4009891	AT	A	AT	INDEL	OS02G0173200	Exonic	3UTR	NA	LOC_Os02g07690	VQ domain containing protein, putative, expressed
Chr2	4297185	4297201	AATATATATATATATAT	A	AATATATATATATATAT	INDEL	OS02G0177800	Intronic	NA	NA	LOC_Os02g08120	calmodulin binding protein, putative, expressed
Chr2	6447086	6447090	ATCGC	A	ATCGC	INDEL	OS02G0215200	Exonic	5UTR	NA	LOC_Os02g12380	histone deacetylase, putative, expressed
Chr2	6811612	6811619	ATAGGCAG	A	ATAGGCAG	INDEL	OS02G0222100	Exonic	5UTR	NA	LOC_Os02g12900	cysteine synthase, putative, expressed
Chr2	7517734	7517762	ACTGCACCAACCGCAGCACACTCAAAGCC	A	ACTGCACCAACCGCAGCACACTCAAAGCC	INDEL	OS02G0232900	Intronic	NA	NA	LOC_Os02g13870	aquaporin protein, putative, expressed
Chr2	7519135	7519141	ATCTCTC	A	ATCTCTC	INDEL	OS02G0232900	Exonic	CDS	InFrame	LOC_Os02g13870	aquaporin protein, putative, expressed
Chr2	7978638	7978641	AGGG	A	AGGG	INDEL	OS02G0241100	Exonic	5UTR	NA	LOC_Os02g14480	receptor-like kinase, putative, expressed
Chr2	8582780	8582811	ATTTGCATGCATATACACTTTATCCTGTCCAG	A	ATTTGCATGCATATACACTTTATCCTGTCCAG	INDEL	OS02G0252200	Intronic	NA	NA	LOC_Os02g15340	no apical meristem protein, putative, expressed
Chr2	8836575	8836579	ACCAT	A	ACCAT	INDEL	OS02G0256100	Intronic	NA	NA	LOC_Os02g15690	polygalacturonase, putative, expressed
Chr2	12466108	12466120	AGCCGGCGCCGGC	A	AGCCGGCGCCGGC	INDEL	OS02G0314600	Exonic	CDS	InFrame	LOC_Os02g21040	aspartic proteinase nepenthesin precursor, putative, expressed
Chr2	14948127	14948133	ACCCCCC	A	ACCCCCC	INDEL	OS02G0452500	Intronic	NA	NA	LOC_Os02g25580	Sec1 family transport protein, putative, expressed
Chr2	17535879	17535890	AGATATATATGT	A	AGATATATATGT	INDEL	OS02G0498300	Intronic	NA	NA	LOC_Os02g29510	DUF803 domain containing, putative, expressed
Chr2	18831240	18831241	AT	A	AT	INDEL	OS02G0518100	Intronic	NA	NA	LOC_Os02g31874	DUF803 domain containing, putative, expressed
Chr2	20152544	20152550	AAAGGAG	A	AAAGGAG	INDEL	OS02G0542500	Exonic	5UTR	NA	LOC_Os02g33780	serine-aspartate repeat-containing protein I precursor, putative, expressed
Chr2	20152638	20152649	ATCTCGTCCTCG	A	ATCTCGTCCTCG	INDEL	OS02G0542500	Exonic	5UTR	NA	LOC_Os02g33780	serine-aspartate repeat-containing protein I precursor, putative, expressed
Chr2	20153548	20153563	AAGAAGCTGAAGCAGC	A	AAGAAGCTGAAGCAGC	INDEL	OS02G0542500	Exonic	CDS	InFrame	LOC_Os02g33780	serine-aspartate repeat-containing protein I precursor, putative, expressed
Chr2	20746525	20746531	ACAGGAT	A	ACAGGAT	INDEL	OS02G0551100	Exonic	3UTR	NA	LOC_Os02g34600	CAMK_CAMK_like.13 - CAMK includes calcium/calmodulin depedent protein kinases, expressed
Chr2	26422285	26422287	ATC	A	ATC	INDEL	OS02G0654700	Exonic	3UTR	NA	LOC_Os02g43790	ethylene-responsive transcription factor, putative, expressed
Chr2	28750805	28750807	AAC	A	AAC	INDEL	OS02G0699000	Intronic	NA	NA	LOC_Os02g47090	peptide transporter PTR2, putative, expressed
Chr2	30459213	30459214	AG	A	AG	INDEL	OS02G0731200	Intronic	NA	NA	LOC_Os02g49840	OsMADS57 - MADS-box family gene with MIKCc type-box, expressed
Chr2	31852579	31852581	AAT	A	AAT	INDEL	OS02G0757100	Exonic	3UTR	NA	LOC_Os02g52040	phosphate-induced protein 1 conserved region domain containing protein, expressed
Chr3	22150525	22150525	G	A	G	SNP	OS03G0595300	Exonic	3UTR	NA	LOC_Os03g39830	expressed protein
Chr3	33034657	33034657	T	A	T	SNP	OS03G0794200	Exonic	CDS	NA	LOC_Os03g58010	acetyltransferase, GNAT family, putative, expressed
Chr3	15473805	15473805	T	A	T	SNP	OS03G0387900	Exonic	3UTR	NA	LOC_Os03g27019	expressed protein
Chr3	8703241	8703241	G	A	G	SNP	OS03G0264400	Exonic	CDS	Silent	LOC_Os03g15780	anthranilate synthase component I-1, chloroplast precursor, putative, expressed
Chr3	27503691	27503691	G	A	G	SNP	OS03G0689100	Exonic	CDS	Silent	LOC_Os03g48300	histidine acid phosphatase, putative, expressed
Chr3	12633113	12633113	C	A	C	SNP	OS03G0339900	Exonic	CDS	Missense	LOC_Os03g22050	CAMK_KIN1/SNF1/Nim1_like.16 - CAMK includes calcium/calmodulin depedent protein kinases, expressed
Chr3	32567909	32567909	G	A	G	SNP	OS03G0784700	Exonic	CDS	Missense	LOC_Os03g57120	ferredoxin--NADP reductase, chloroplast precursor, putative, expressed
Chr3	10906064	10906064	C	A	C	SNP	OS03G0306700	Exonic	5UTR	NA	LOC_Os03g19370	CPuORF4 - conserved peptide uORF-containing transcript, expressed
Chr3	23375700	23375700	G	A	G	SNP	OS03G0616700	Exonic	CDS	NA	LOC_Os03g42050	exportin-7-A, putative, expressed
Chr3	16593673	16593673	G	A	G	SNP	OS03G0405500	Exonic	CDS	Missense	LOC_Os03g29190	PDI, putative, expressed
Chr3	9449642	9449642	G	A	G	SNP	OS03G0278300	Exonic	3UTR	NA	LOC_Os03g17010	RNA recognition motif containing protein, putative, expressed
Chr3	35870974	35870974	T	A	T	SNP	OS03G0851900	Intronic	NA	NA	LOC_Os03g63500	ATPase, AFG1 family domain containing protein, expressed
Chr3	12587749	12587749	T	A	T	SNP	OS03G0338400	Exonic	5UTR	NA	LOC_Os03g21970	basic helix-loop-helix domain containing protein, expressed
Chr3	12587990	12587990	T	A	T	SNP	OS03G0338400	Intronic	NA	NA	LOC_Os03g21970	basic helix-loop-helix domain containing protein, expressed
Chr3	10693869	10693869	T	A	T	SNP	OS03G0303100	Exonic	3UTR	NA	LOC_Os03g19090	expressed protein
Chr3	10572523	10572523	T	A	T	SNP	OS03G0300600	Intronic	NA	NA	LOC_Os03g18870	heat shock protein DnaJ, putative, expressed
Chr3	909493	909493	T	A	T	SNP	OS03G0116000	Exonic	CDS	Silent	LOC_Os03g02480	inner membrane protein, putative, expressed
Chr3	32670044	32670044	T	A	T	SNP	OS03G0786700	Intronic	NA	NA	LOC_Os03g57280	N-dimethylguanosine tRNA methyltransferase, putative, expressed
Chr3	36288617	36288617	T	A	T	SNP	OS03G0859800	Intronic	NA	NA	LOC_Os03g64219	OTU-like cysteine protease family protein, putative, expressed
Chr3	26631000	26631000	T	A	T	SNP	OS03G0673800	Intronic	NA	NA	LOC_Os03g47070	RXW8, putative, expressed
Chr3	10994142	10994142	T	A	T	SNP	OS03G0308800	Intronic	NA	NA	LOC_Os03g19560	tetratricopeptide repeat domain containing protein, expressed
Chr3	30075162	30075162	T	A	T	SNP	OS03G0734100	Intronic	NA	NA	LOC_Os03g52370	PIII4 - Proteinase inhibitor II family protein precursor, expressed
Chr3	10394457	10394457	G	A	G	SNP	OS03G0297100	Intronic	NA	NA	LOC_Os03g18570	40S ribosomal protein S7, putative, expressed
Chr3	35792353	35792353	G	A	G	SNP	OS03G0850400	Intronic	NA	NA	LOC_Os03g63330	aspartokinase, chloroplast precursor, putative, expressed
Chr3	35793117	35793117	G	A	G	SNP	OS03G0850400	Intronic	NA	NA	LOC_Os03g63330	aspartokinase, chloroplast precursor, putative, expressed
Chr3	35869217	35869217	G	A	G	SNP	OS03G0851900	Intronic	NA	NA	LOC_Os03g63500	ATPase, AFG1 family domain containing protein, expressed
Chr3	35869363	35869363	G	A	G	SNP	OS03G0851900	Intronic	NA	NA	LOC_Os03g63500	ATPase, AFG1 family domain containing protein, expressed
Chr3	35872509	35872509	G	A	G	SNP	OS03G0851900	Intronic	NA	NA	LOC_Os03g63500	ATPase, AFG1 family domain containing protein, expressed
Chr3	31347031	31347031	G	A	G	SNP	OS03G0758300	Intronic	NA	NA	LOC_Os03g55100	cyclic nucleotide-gated ion channel 2, putative, expressed
Chr3	31347039	31347039	G	A	G	SNP	OS03G0758300	Intronic	NA	NA	LOC_Os03g55100	cyclic nucleotide-gated ion channel 2, putative, expressed
Chr3	4417856	4417856	G	A	G	SNP	OS03G0184100	Exonic	CDS	Silent	LOC_Os03g08580	expressed protein
Chr3	4546930	4546930	G	A	G	SNP	OS03G0187000	Intronic	NA	NA	LOC_Os03g08810	expressed protein
Chr3	11008414	11008414	G	A	G	SNP	OS03G0309000	Intronic	NA	NA	LOC_Os03g19580	expressed protein
Chr3	34908634	34908634	G	A	G	SNP	OS03G0831100	Exonic	CDS	Silent	LOC_Os03g61560	expressed protein
Chr3	908823	908823	G	A	G	SNP	OS03G0116000	Intronic	NA	NA	LOC_Os03g02480	inner membrane protein, putative, expressed
Chr3	11327318	11327318	G	A	G	SNP	OS03G0315400	Exonic	CDS	Silent	LOC_Os03g20090	MYB family transcription factor, putative, expressed
Chr3	32668274	32668274	G	A	G	SNP	OS03G0786700	Intronic	NA	NA	LOC_Os03g57280	N-dimethylguanosine tRNA methyltransferase, putative, expressed
Chr3	32669607	32669607	G	A	G	SNP	OS03G0786700	Intronic	NA	NA	LOC_Os03g57280	N-dimethylguanosine tRNA methyltransferase, putative, expressed
Chr3	4003281	4003281	G	A	G	SNP	OS03G0174900	Intronic	NA	NA	LOC_Os03g07880	nuclear transcription factor Y subunit, putative, expressed
Chr3	7129081	7129081	G	A	G	SNP	OS03G0235000	Intronic	NA	NA	LOC_Os03g13210	peroxidase precursor, putative, expressed
Chr3	1613407	1613407	G	A	G	SNP	OS03G0128500	Intronic	NA	NA	LOC_Os03g03650	POLD2 - Putative DNA polymerase delta complex subunit, expressed
Chr3	7349731	7349731	G	A	G	SNP	OS03G0239200	Intronic	NA	NA	LOC_Os03g13590	potyvirus VPg interacting protein, putative, expressed
Chr3	36003761	36003761	G	A	G	SNP	OS03G0854800	Exonic	CDS	Silent	LOC_Os03g63770	RCD1, putative, expressed
Chr3	36005574	36005574	G	A	G	SNP	OS03G0854800	Exonic	5UTR	NA	LOC_Os03g63770	RCD1, putative, expressed
Chr3	26629137	26629137	G	A	G	SNP	OS03G0673800	Exonic	3UTR	NA	LOC_Os03g47070	RXW8, putative, expressed
Chr3	26631496	26631496	G	A	G	SNP	OS03G0673800	Intronic	NA	NA	LOC_Os03g47070	RXW8, putative, expressed
Chr3	35042963	35042963	G	A	G	SNP	OS03G0833900	Exonic	3UTR	NA	LOC_Os03g61810	tRNA-specific adenosine deaminase, putative, expressed
Chr3	24814155	24814155	G	A	G	SNP	OS03G0643300	Intronic	NA	NA	LOC_Os03g44150	aminotransferase, putative, expressed
Chr3	12631396	12631396	G	A	G	SNP	OS03G0339900	Exonic	3UTR	NA	LOC_Os03g22050	CAMK_KIN1/SNF1/Nim1_like.16 - CAMK includes calcium/calmodulin depedent protein kinases, expressed
Chr3	10393945	10393945	C	A	C	SNP	OS03G0297100	Exonic	CDS	Silent	LOC_Os03g18570	40S ribosomal protein S7, putative, expressed
Chr3	35872548	35872548	C	A	C	SNP	OS03G0851900	Intronic	NA	NA	LOC_Os03g63500	ATPase, AFG1 family domain containing protein, expressed
Chr3	4123278	4123278	C	A	C	SNP	OS03G0178100	Intronic	NA	NA	LOC_Os03g08070	copper-transporting ATPase PAA1, putative, expressed

Chr3	31346871	31346871	C	A	C	SNP	OS03G0758300	Intronic	NA	NA	LOC_Os03g55100	cyclic nucleotide-gated ion channel 2, putative, expressed
Chr3	33215113	33215113	C	A	C	SNP	OS03G0797400	Exonic	CDS	Missense	LOC_Os03g58300	indole-3-glycerol phosphate lyase, chloroplast precursor, putative, expressed
Chr3	15670404	15670404	C	A	C	SNP	OS03G0391100	Intronic	NA	NA	LOC_Os03g27360	RING-H2 finger protein ATL5H precursor, putative, expressed
Chr3	35663150	35663150	C	A	C	SNP	OS03G0848100	Exonic	3UTR	NA	LOC_Os03g63074	Ser/Thr protein phosphatase family protein, expressed
Chr3	32042549	32042549	G	A	G	SNP	OS03G0773150	Intronic	NA	NA	LOC_Os03g56241	40S ribosomal protein S29, putative, expressed
Chr3	35127760	35127760	C	A	C	SNP	OS03G0836000	Intronic	NA	NA	LOC_Os03g61970	actin, putative, expressed
Chr3	35128879	35128879	G	A	G	SNP	OS03G0836000	Intronic	NA	NA	LOC_Os03g61970	actin, putative, expressed
Chr3	24811738	24811738	G	A	G	SNP	OS03G0643300	Intronic	NA	NA	LOC_Os03g44150	aminotransferase, putative, expressed
Chr3	24815315	24815315	G	A	G	SNP	OS03G0643300	Intronic	NA	NA	LOC_Os03g44150	aminotransferase, putative, expressed
Chr3	24816797	24816797	G	A	G	SNP	OS03G0643300	Intronic	NA	NA	LOC_Os03g44150	aminotransferase, putative, expressed
Chr3	24817071	24817071	G	A	G	SNP	OS03G0643300	Intronic	NA	NA	LOC_Os03g44150	aminotransferase, putative, expressed
Chr3	8704673	8704673	G	A	G	SNP	OS03G0264400	Exonic	CDS	Missense	LOC_Os03g15780	anthranilate synthase component I-1, chloroplast precursor, putative, expressed
Chr3	6898232	6898232	C	A	C	SNP	OS03G0230300	Exonic	3UTR	NA	LOC_Os03g12820	ATP8, putative, expressed
Chr3	6897310	6897310	G	A	G	SNP	OS03G0230300	Intronic	NA	NA	LOC_Os03g12820	ATP8, putative, expressed
Chr3	16840153	16840153	C	A	C	SNP	OS03G0409100	Exonic	5UTR	NA	LOC_Os03g29540	ATP-dependent protease, putative, expressed
Chr3	16838056	16838056	G	A	G	SNP	OS03G0409100	Exonic	5UTR	NA	LOC_Os03g29540	ATP-dependent protease, putative, expressed
Chr3	16839137	16839137	G	A	G	SNP	OS03G0409100	Exonic	5UTR	NA	LOC_Os03g29540	ATP-dependent protease, putative, expressed
Chr3	16839524	16839524	G	A	G	SNP	OS03G0409100	Exonic	5UTR	NA	LOC_Os03g29540	ATP-dependent protease, putative, expressed
Chr3	16839998	16839998	G	A	G	SNP	OS03G0409100	Exonic	5UTR	NA	LOC_Os03g29540	ATP-dependent protease, putative, expressed
Chr3	16840205	16840205	G	A	G	SNP	OS03G0409100	Exonic	5UTR	NA	LOC_Os03g29540	ATP-dependent protease, putative, expressed
Chr3	16838839	16838839	T	A	T	SNP	OS03G0409100	Exonic	5UTR	NA	LOC_Os03g29540	ATP-dependent protease, putative, expressed
Chr3	16839160	16839160	T	A	T	SNP	OS03G0409100	Exonic	5UTR	NA	LOC_Os03g29540	ATP-dependent protease, putative, expressed
Chr3	16839855	16839855	T	A	T	SNP	OS03G0409100	Exonic	5UTR	NA	LOC_Os03g29540	ATP-dependent protease, putative, expressed
Chr3	16839856	16839856	T	A	T	SNP	OS03G0409100	Exonic	5UTR	NA	LOC_Os03g29540	ATP-dependent protease, putative, expressed
Chr3	8200889	8200889	G	A	G	SNP	OS03G0255100	Intronic	NA	NA	LOC_Os03g15020	beta-galactosidase precursor, putative, expressed
Chr3	3527803	3527803	G	A	G	SNP	OS03G0165400	Exonic	CDS	Silent	LOC_Os03g06940	beta-galactosidase, putative, expressed
Chr3	12795332	12795332	G	A	G	SNP	OS03G0343400	Exonic	CDS	Silent	LOC_Os03g22330	blue-light photoreceptor PHR2, putative, expressed
Chr3	12795434	12795434	G	A	G	SNP	OS03G0343400	Exonic	CDS	Silent	LOC_Os03g22330	blue-light photoreceptor PHR2, putative, expressed
Chr3	12797804	12797804	G	A	G	SNP	OS03G0343400	Exonic	3UTR	NA	LOC_Os03g22330	blue-light photoreceptor PHR2, putative, expressed
Chr3	18637153	18637153	G	A	G	SNP	OS03G0440900	Intronic	NA	NA	LOC_Os03g32580	BRASSINOSTEROID INSENSITIVE 1-associated receptor kinase 1 precursor, putative, expressed
Chr3	18637877	18637877	G	A	G	SNP	OS03G0440900	Intronic	NA	NA	LOC_Os03g32580	BRASSINOSTEROID INSENSITIVE 1-associated receptor kinase 1 precursor, putative, expressed
Chr3	12631329	12631329	G	A	G	SNP	OS03G0339900	Exonic	3UTR	NA	LOC_Os03g22050	CAMK_KIN1/SNF1/Nim1_like.16 - CAMK includes calcium/calmodulin depedent protein kinases, expressed
Chr3	12631407	12631407	G	A	G	SNP	OS03G0339900	Exonic	3UTR	NA	LOC_Os03g22050	CAMK_KIN1/SNF1/Nim1_like.16 - CAMK includes calcium/calmodulin depedent protein kinases, expressed
Chr3	12631472	12631472	G	A	G	SNP	OS03G0339900	Exonic	3UTR	NA	LOC_Os03g22050	CAMK_KIN1/SNF1/Nim1_like.16 - CAMK includes calcium/calmodulin depedent protein kinases, expressed
Chr3	12631962	12631962	G	A	G	SNP	OS03G0339900	Exonic	3UTR	NA	LOC_Os03g22050	CAMK_KIN1/SNF1/Nim1_like.16 - CAMK includes calcium/calmodulin depedent protein kinases, expressed
Chr3	6641479	6641479	G	A	G	SNP	OS03G0226400	Exonic	3UTR	NA	LOC_Os03g12530	cation efflux family protein, putative, expressed
Chr3	27335922	27335922	T	A	T	SNP	OS03G0685300	Intronic	NA	NA	LOC_Os03g48060	class I glutamine amidotransferase, putative, expressed
Chr3	27291416	27291416	T	A	T	SNP	OS03G0684400	Intronic	NA	NA	LOC_Os03g48000	CorA-like magnesium transporter protein, putative, expressed
Chr3	9499337	9499337	C	A	C	SNP	OS03G0279200	Exonic	CDS	Silent	LOC_Os03g17100	Core histone H2A/H2B/H3/H4 domain containing protein, putative, expressed
Chr3	31346993	31346993	G	A	G	SNP	OS03G0758300	Intronic	NA	NA	LOC_Os03g55100	cyclic nucleotide-gated ion channel 2, putative, expressed
Chr3	31347780	31347780	G	A	G	SNP	OS03G0758300	Intronic	NA	NA	LOC_Os03g55100	cyclic nucleotide-gated ion channel 2, putative, expressed
Chr3	31348235	31348235	G	A	G	SNP	OS03G0758300	Exonic	CDS	Missense	LOC_Os03g55100	cyclic nucleotide-gated ion channel 2, putative, expressed
Chr3	31347656	31347656	T	A	T	SNP	OS03G0758300	Intronic	NA	NA	LOC_Os03g55100	cyclic nucleotide-gated ion channel 2, putative, expressed
Chr3	9421857	9421857	G	A	G	SNP	OS03G0277600	Exonic	3UTR	NA	LOC_Os03g16950	cysteine-rich repeat secretory protein 55 precursor, putative, expressed
Chr3	9421990	9421990	G	A	G	SNP	OS03G0277600	Exonic	CDS	Silent	LOC_Os03g16950	cysteine-rich repeat secretory protein 55 precursor, putative, expressed
Chr3	9422056	9422056	G	A	G	SNP	OS03G0277600	Exonic	CDS	Silent	LOC_Os03g16950	cysteine-rich repeat secretory protein 55 precursor, putative, expressed
Chr3	9425762	9425762	G	A	G	SNP	OS03G0277700	Exonic	3UTR	NA	LOC_Os03g16960	cysteine-rich repeat secretory protein 55 precursor, putative, expressed
Chr3	9426310	9426310	G	A	G	SNP	OS03G0277700	Exonic	CDS	Silent	LOC_Os03g16960	cysteine-rich repeat secretory protein 55 precursor, putative, expressed
Chr3	9421851	9421851	T	A	T	SNP	OS03G0277600	Exonic	3UTR	NA	LOC_Os03g16950	cysteine-rich repeat secretory protein 55 precursor, putative, expressed
Chr3	31437173	31437173	C	A	C	SNP	OS03G0760000	Exonic	CDS	Missense	LOC_Os03g55230	cytochrome P450, putative, expressed
Chr3	22085798	22085798	G	A	G	SNP	OS03G0594100	Exonic	CDS	Missense	LOC_Os03g39690	cytochrome P450, putative, expressed
Chr3	31437562	31437562	G	A	G	SNP	OS03G0760000	Exonic	3UTR	NA	LOC_Os03g55230	cytochrome P450, putative, expressed
Chr3	30064603	30064603	T	A	T	SNP	OS03G0733800	Exonic	CDS	Missense	LOC_Os03g52340	endoplasmic oxidoreductin-1 precursor, putative, expressed
Chr3	23371614	23371614	G	A	G	SNP	OS03G0616700	Intronic	NA	NA	LOC_Os03g42050	exportin-7-A, putative, expressed
Chr3	23371799	23371799	G	A	G	SNP	OS03G0616700	Intronic	NA	NA	LOC_Os03g42050	exportin-7-A, putative, expressed
Chr3	23371816	23371816	T	A	T	SNP	OS03G0616700	Intronic	NA	NA	LOC_Os03g42050	exportin-7-A, putative, expressed
Chr3	23372304	23372304	T	A	T	SNP	OS03G0616700	Intronic	NA	NA	LOC_Os03g42050	exportin-7-A, putative, expressed
Chr3	23373197	23373197	T	A	T	SNP	OS03G0616700	Intronic	NA	NA	LOC_Os03g42050	exportin-7-A, putative, expressed
Chr3	27250252	27250252	C	A	C	SNP	OS03G0683700	Intronic	NA	NA	LOC_Os03g47930	expressed protein
Chr3	27462699	27462699	C	A	C	SNP	OS03G0688200	Intronic	NA	NA	LOC_Os03g48260	expressed protein
Chr3	31555786	31555786	C	A	C	SNP	OS03G0762900	Intronic	NA	NA	LOC_Os03g55470	expressed protein
Chr3	12869567	12869567	G	A	G	SNP	OS03G0345200	Intronic	NA	NA	LOC_Os03g22460	expressed protein
Chr3	15469854	15469854	G	A	G	SNP	OS03G0387900	Intronic	NA	NA	LOC_Os03g27019	expressed protein
Chr3	15472062	15472062	G	A	G	SNP	OS03G0387900	Exonic	CDS	Missense	LOC_Os03g27019	expressed protein
Chr3	27459728	27459728	G	A	G	SNP	OS03G0688200	Intronic	NA	NA	LOC_Os03g48260	expressed protein
Chr3	27460280	27460280	G	A	G	SNP	OS03G0688200	Intronic	NA	NA	LOC_Os03g48260	expressed protein
Chr3	28265285	28265285	G	A	G	SNP	OS03G0703300	Intronic	NA	NA	LOC_Os03g49630	expressed protein
Chr3	28265289	28265289	G	A	G	SNP	OS03G0703300	Intronic	NA	NA	LOC_Os03g49630	expressed protein
Chr3	29387124	29387124	G	A	G	SNP	OS03G0723400	Intronic	NA	NA	LOC_Os03g51350	expressed protein
Chr3	31557971	31557971	G	A	G	SNP	OS03G0762900	Exonic	3UTR	NA	LOC_Os03g55470	expressed protein
Chr3	33697610	33697610	G	A	G	SNP	OS03G0806600	Exonic	CDS	Missense	LOC_Os03g59180	expressed protein
Chr3	15468388	15468388	T	A	T	SNP	OS03G0387900	Intronic	NA	NA	LOC_Os03g27019	expressed protein
Chr3	29452437	29452437	T	A	T	SNP	OS03G0724600	Intronic	NA	NA	LOC_Os03g51470	expressed protein
Chr3	32377191	32377191	C	A	C	SNP	OS03G0780800	Exonic	CDS	Silent	LOC_Os03g56820	fatty acid hydroxylase, putative, expressed
Chr3	32378013	32378013	G	A	G	SNP	OS03G0780800	Intronic	NA	NA	LOC_Os03g56820	fatty acid hydroxylase, putative, expressed
Chr3	32568491	32568491	G	A	G	SNP	OS03G0784700	Exonic	CDS	Silent	LOC_Os03g57120	ferredoxin--NADP reductase, chloroplast precursor, putative, expressed
Chr3	9013821	9013821	C	A	C	SNP	OS03G0269900	Intronic	NA	NA	LOC_Os03g16334	fringe-related protein, putative, expressed
Chr3	9014300	9014300	G	A	G	SNP	OS03G0269900	Intronic	NA	NA	LOC_Os03g16334	fringe-related protein, putative, expressed
Chr3	9014023	9014023	T	A	T	SNP	OS03G0269900	Intronic	NA	NA	LOC_Os03g16334	fringe-related protein, putative, expressed
Chr3	33546875	33546875	G	A	G	SNP	OS03G0803900	Exonic	3UTR	NA	LOC_Os03g58920	galactosyltransferase family protein, putative, expressed
Chr3	8353543	8353543	G	A	G	SNP	OS03G0258200	Exonic	3UTR	NA	LOC_Os03g15270	gibberellin receptor GID1L2, putative, expressed
Chr3	6370876	6370876	G	A	G	SNP	OS03G0221500	Intronic	NA	NA	LOC_Os03g12140	glucan endo-1,3-beta-glucosidase precursor, putative, expressed
Chr3	35027635	35027635	G	A	G	SNP	OS03G0833600	Intronic	NA	NA	LOC_Os03g61780	glucan endo-1,3-beta-glucosidase-related, putative, expressed
Chr3	6460432	6460432	G	A	G	SNP	OS03G0223400	Intronic	NA	NA	LOC_Os03g12290	glutamine synthetase, catalytic domain containing protein, expressed
Chr3	6460470	6460470	G	A	G	SNP	OS03G0223400	Intronic	NA	NA	LOC_Os03g12290	glutamine synthetase, catalytic domain containing protein, expressed

Chr3	25063807	25063807	T	A	T	SNP	OS03G0647500	Exonic	CDS	NA	LOC_Os03g44530	GTPase of unknown function domain containing protein, putative, expressed
Chr3	27487750	27487750	C	A	C	SNP	OS03G0689100	Intronic	NA	NA	LOC_Os03g48300	histidine acid phosphatase, putative, expressed
Chr3	27494819	27494819	C	A	C	SNP	OS03G0689100	Exonic	CDS	Silent	LOC_Os03g48300	histidine acid phosphatase, putative, expressed
Chr3	27498073	27498073	C	A	C	SNP	OS03G0689100	Intronic	NA	NA	LOC_Os03g48300	histidine acid phosphatase, putative, expressed
Chr3	27489428	27489428	G	A	G	SNP	OS03G0689100	Intronic	NA	NA	LOC_Os03g48300	histidine acid phosphatase, putative, expressed
Chr3	27494597	27494597	G	A	G	SNP	OS03G0689100	Intronic	NA	NA	LOC_Os03g48300	histidine acid phosphatase, putative, expressed
Chr3	27496450	27496450	G	A	G	SNP	OS03G0689100	Intronic	NA	NA	LOC_Os03g48300	histidine acid phosphatase, putative, expressed
Chr3	27490965	27490965	T	A	T	SNP	OS03G0689100	Intronic	NA	NA	LOC_Os03g48300	histidine acid phosphatase, putative, expressed
Chr3	27494780	27494780	T	A	T	SNP	OS03G0689100	Intronic	NA	NA	LOC_Os03g48300	histidine acid phosphatase, putative, expressed
Chr3	27497351	27497351	T	A	T	SNP	OS03G0689100	Intronic	NA	NA	LOC_Os03g48300	histidine acid phosphatase, putative, expressed
Chr3	27499444	27499444	T	A	T	SNP	OS03G0689100	Intronic	NA	NA	LOC_Os03g48300	histidine acid phosphatase, putative, expressed
Chr3	33215646	33215646	G	A	G	SNP	OS03G0797400	Intronic	NA	NA	LOC_Os03g58300	indole-3-glycerol phosphate lyase, chloroplast precursor, putative, expressed
Chr3	33215936	33215936	G	A	G	SNP	OS03G0797400	Intronic	NA	NA	LOC_Os03g58300	indole-3-glycerol phosphate lyase, chloroplast precursor, putative, expressed
Chr3	33216016	33216016	G	A	G	SNP	OS03G0797400	Intronic	NA	NA	LOC_Os03g58300	indole-3-glycerol phosphate lyase, chloroplast precursor, putative, expressed
Chr3	32010490	32010490	G	A	G	SNP	OS03G0772600	Exonic	3UTR	NA	LOC_Os03g56160	lectin-like receptor kinase 7, putative, expressed
Chr3	10040210	10040210	G	A	G	SNP	OS03G0289800	Intronic	NA	NA	LOC_Os03g18030	leucoanthocyanidin dioxygenase, putative, expressed
Chr3	10041470	10041470	T	A	T	SNP	OS03G0289800	Intronic	NA	NA	LOC_Os03g18030	leucoanthocyanidin dioxygenase, putative, expressed
Chr3	28052048	28052048	C	A	C	SNP	OS03G0699700	Exonic	CDS	Silent	LOC_Os03g49260	lipoxygenase, putative, expressed
Chr3	28049968	28049968	G	A	G	SNP	OS03G0699700	Intronic	NA	NA	LOC_Os03g49260	lipoxygenase, putative, expressed
Chr3	30318802	30318802	G	A	G	SNP	OS03G0738600	Exonic	3UTR	NA	LOC_Os03g52860	lipoxygenase, putative, expressed
Chr3	3627247	3627247	C	A	C	SNP	OS03G0167000	Intronic	NA	NA	LOC_Os03g07100	LTPL82 - Protease inhibitor/seed storage/LTP family protein precursor, expressed
Chr3	29455689	29455689	G	A	G	SNP	OS03G0724700	Intronic	NA	NA	LOC_Os03g51479	maf, putative, expressed
Chr3	20793666	20793666	T	A	T	SNP	OS03G0571900	Intronic	NA	NA	LOC_Os03g37490	MATE efflux family protein, putative, expressed
Chr3	29560629	29560629	C	A	C	SNP	OS03G0726500	Exonic	3UTR	NA	LOC_Os03g51650	membrane protein, putative, expressed
Chr3	29564693	29564693	C	A	C	SNP	OS03G0726500	Intronic	NA	NA	LOC_Os03g51650	membrane protein, putative, expressed
Chr3	29560668	29560668	G	A	G	SNP	OS03G0726500	Exonic	3UTR	NA	LOC_Os03g51650	membrane protein, putative, expressed
Chr3	29562137	29562137	T	A	T	SNP	OS03G0726500	Intronic	NA	NA	LOC_Os03g51650	membrane protein, putative, expressed
Chr3	9445695	9445695	G	A	G	SNP	OS03G0278200	Intronic	NA	NA	LOC_Os03g17000	NAD dependent epimerase/dehydratase family domain containing protein, expressed
Chr3	9447365	9447365	G	A	G	SNP	OS03G0278200	Exonic	CDS	Silent	LOC_Os03g17000	NAD dependent epimerase/dehydratase family domain containing protein, expressed
Chr3	9446044	9446044	T	A	T	SNP	OS03G0278200	Intronic	NA	NA	LOC_Os03g17000	NAD dependent epimerase/dehydratase family domain containing protein, expressed
Chr3	32236300	32236300	G	A	G	SNP	OS03G0777000	Exonic	3UTR	NA	LOC_Os03g56580	no apical meristem protein, putative, expressed
Chr3	2750667	2750667	C	A	C	SNP	OS03G0149300	Intronic	NA	NA	LOC_Os03g05530	nodulin, putative, expressed
Chr3	30810768	30810768	C	A	C	SNP	OS03G0748700	Intronic	NA	NA	LOC_Os03g53750	nuclear prelamin A recognition factor, putative, expressed
Chr3	30811724	30811724	G	A	G	SNP	OS03G0748700	Exonic	CDS	Silent	LOC_Os03g53750	nuclear prelamin A recognition factor, putative, expressed
Chr3	4002117	4002117	G	A	G	SNP	OS03G0174900	Exonic	3UTR	NA	LOC_Os03g07880	nuclear transcription factor Y subunit, putative, expressed
Chr3	4003543	4003543	G	A	G	SNP	OS03G0174900	Exonic	CDS	Missense	LOC_Os03g07880	nuclear transcription factor Y subunit, putative, expressed
Chr3	28244398	28244398	G	A	G	SNP	OS03G0703000	Intronic	NA	NA	LOC_Os03g49600	Os3bglu7 - beta-glucosidase, exo-beta-glucanse, expressed
Chr3	28252107	28252107	T	A	T	SNP	OS03G0703100	Exonic	CDS	Missense	LOC_Os03g49610	Os3bglu8 - beta-glucosidase, exo-beta-glucansase, high similarity to Os3bglu7, expressed
Chr3	9846107	9846107	T	A	T	SNP	OS03G0285700	Exonic	CDS	Silent	LOC_Os03g17690	OsAPx1 - Cytosolic Ascorbate Peroxidase encoding gene 1-8, expressed
Chr3	16590767	16590767	C	A	C	SNP	OS03G0405500	Intronic	NA	NA	LOC_Os03g29190	PDI, putative, expressed
Chr3	16590702	16590702	G	A	G	SNP	OS03G0405500	Intronic	NA	NA	LOC_Os03g29190	PDI, putative, expressed
Chr3	16593999	16593999	G	A	G	SNP	OS03G0405500	Intronic	NA	NA	LOC_Os03g29190	PDI, putative, expressed
Chr3	30853541	30853541	G	A	G	SNP	OS03G0749300	Exonic	5UTR	NA	LOC_Os03g53800	periplasmic beta-glucosidase precursor, putative, expressed
Chr3	28144334	28144334	G	A	G	SNP	OS03G0701200	Intronic	NA	NA	LOC_Os03g49440	phosphatase, putative, expressed
Chr3	8220988	8220988	C	A	C	SNP	OS03G0255500	Exonic	CDS	Missense	LOC_Os03g15050	phosphoenolpyruvate carboxykinase, putative, expressed
Chr3	8219228	8219228	G	A	G	SNP	OS03G0255500	Exonic	CDS	Silent	LOC_Os03g15050	phosphoenolpyruvate carboxykinase, putative, expressed
Chr3	8224039	8224039	G	A	G	SNP	OS03G0255500	Intronic	NA	NA	LOC_Os03g15050	phosphoenolpyruvate carboxykinase, putative, expressed
Chr3	8221519	8221519	T	A	T	SNP	OS03G0255500	Intronic	NA	NA	LOC_Os03g15050	phosphoenolpyruvate carboxykinase, putative, expressed
Chr3	30075116	30075116	G	A	G	SNP	OS03G0734100	Intronic	NA	NA	LOC_Os03g52370	PIII4 - Proteinase inhibitor II family protein precursor, expressed
Chr3	30075118	30075118	G	A	G	SNP	OS03G0734100	Intronic	NA	NA	LOC_Os03g52370	PIII4 - Proteinase inhibitor II family protein precursor, expressed
Chr3	30075140	30075140	G	A	G	SNP	OS03G0734100	Intronic	NA	NA	LOC_Os03g52370	PIII4 - Proteinase inhibitor II family protein precursor, expressed
Chr3	30075323	30075323	G	A	G	SNP	OS03G0734100	Exonic	CDS	Missense	LOC_Os03g52370	PIII4 - Proteinase inhibitor II family protein precursor, expressed
Chr3	9575820	9575820	G	A	G	SNP	OS03G0280400	Intronic	NA	NA	LOC_Os03g17200	plant-specific domain TIGR01589 family protein, expressed
Chr3	9576038	9576038	G	A	G	SNP	OS03G0280400	Exonic	CDS	Silent	LOC_Os03g17200	plant-specific domain TIGR01589 family protein, expressed
Chr3	9575671	9575671	T	A	T	SNP	OS03G0280400	Intronic	NA	NA	LOC_Os03g17200	plant-specific domain TIGR01589 family protein, expressed
Chr3	31472281	31472281	C	A	C	SNP	OS03G0761100	Exonic	3UTR	NA	LOC_Os03g55320	protein phosphatase 2C, putative, expressed
Chr3	31472527	31472527	G	A	G	SNP	OS03G0761100	Exonic	CDS	Silent	LOC_Os03g55320	protein phosphatase 2C, putative, expressed
Chr3	31473739	31473739	G	A	G	SNP	OS03G0761100	Intronic	NA	NA	LOC_Os03g55320	protein phosphatase 2C, putative, expressed
Chr3	31473734	31473734	T	A	T	SNP	OS03G0761100	Intronic	NA	NA	LOC_Os03g55320	protein phosphatase 2C, putative, expressed
Chr3	29017529	29017529	C	A	C	SNP	OS03G0717000	Intronic	NA	NA	LOC_Os03g50810	receptor protein kinase TMK1 precursor, putative, expressed
Chr3	29016997	29016997	G	A	G	SNP	OS03G0717000	Intronic	NA	NA	LOC_Os03g50810	receptor protein kinase TMK1 precursor, putative, expressed
Chr3	24468257	24468257	C	A	C	SNP	OS03G0638400	Intronic	NA	NA	LOC_Os03g43750	retrotransposon, putative, centromere-specific, expressed
Chr3	24470637	24470637	G	A	G	SNP	OS03G0638400	Intronic	NA	NA	LOC_Os03g43750	retrotransposon, putative, centromere-specific, expressed
Chr3	24471093	24471093	G	A	G	SNP	OS03G0638400	Intronic	NA	NA	LOC_Os03g43750	retrotransposon, putative, centromere-specific, expressed
Chr3	24471590	24471590	G	A	G	SNP	OS03G0638400	Exonic	5UTR	NA	LOC_Os03g43750	retrotransposon, putative, centromere-specific, expressed
Chr3	24468052	24468052	T	A	T	SNP	OS03G0638400	Intronic	NA	NA	LOC_Os03g43750	retrotransposon, putative, centromere-specific, expressed
Chr3	24471830	24471830	T	A	T	SNP	OS03G0638400	Exonic	5UTR	NA	LOC_Os03g43750	retrotransposon, putative, centromere-specific, expressed
Chr3	15670120	15670120	G	A	G	SNP	OS03G0391100	Intronic	NA	NA	LOC_Os03g27360	RING-H2 finger protein ATL5H precursor, putative, expressed
Chr3	15670188	15670188	G	A	G	SNP	OS03G0391100	Intronic	NA	NA	LOC_Os03g27360	RING-H2 finger protein ATL5H precursor, putative, expressed
Chr3	15670429	15670429	G	A	G	SNP	OS03G0391100	Intronic	NA	NA	LOC_Os03g27360	RING-H2 finger protein ATL5H precursor, putative, expressed
Chr3	15671064	15671064	G	A	G	SNP	OS03G0391100	Exonic	CDS	Silent	LOC_Os03g27360	RING-H2 finger protein ATL5H precursor, putative, expressed
Chr3	24597849	24597849	C	A	C	SNP	OS03G0639800	Intronic	NA	NA	LOC_Os03g43860	SNF7 domain containing protein, putative, expressed
Chr3	24597574	24597574	G	A	G	SNP	OS03G0639800	Exonic	CDS	Silent	LOC_Os03g43860	SNF7 domain containing protein, putative, expressed
Chr3	24598119	24598119	G	A	G	SNP	OS03G0639800	Intronic	NA	NA	LOC_Os03g43860	SNF7 domain containing protein, putative, expressed
Chr3	24599047	24599047	T	A	T	SNP	OS03G0639800	Intronic	NA	NA	LOC_Os03g43860	SNF7 domain containing protein, putative, expressed
Chr3	4989510	4989510	G	A	G	SNP	OS03G0195800	Exonic	CDS	Missense	LOC_Os03g09970	sulfate transporter, putative, expressed
Chr3	23490196	23490196	C	A	C	SNP	OS03G0619400	Intronic	NA	NA	LOC_Os03g42220	T-complex protein, putative, expressed
Chr3	23486475	23486475	G	A	G	SNP	OS03G0619400	Intronic	NA	NA	LOC_Os03g42220	T-complex protein, putative, expressed
Chr3	26952810	26952810	C	A	C	SNP	OS03G0679700	Intronic	NA	NA	LOC_Os03g47610	thiamine biosynthesis protein thiC, putative, expressed
Chr3	26955728	26955728	C	A	C	SNP	OS03G0679700	Intronic	NA	NA	LOC_Os03g47610	thiamine biosynthesis protein thiC, putative, expressed
Chr3	26957102	26957102	G	A	G	SNP	OS03G0679700	Intronic	NA	NA	LOC_Os03g47610	thiamine biosynthesis protein thiC, putative, expressed
Chr3	25002084	25002084	G	A	G	SNP	OS03G0646200	Exonic	CDS	Silent	LOC_Os03g44430	ubiquitin carboxyl-terminal hydrolase, family 1, putative, expressed
Chr3	32934378	32934378	G	A	G	SNP	OS03G0791800	Intronic	NA	NA	LOC_Os03g57790	ubiquitin-conjugating enzyme, putative, expressed
Chr3	31296047	31296047	G	A	G	SNP	OS03G0757200	Intronic	NA	NA	LOC_Os03g55030	UDP-glucoronosyl and UDP-glucosyl transferase domain containing protein, expressed
Chr3	31296231	31296231	G	A	G	SNP	OS03G0757200	Intronic	NA	NA	LOC_Os03g55030	UDP-glucoronosyl and UDP-glucosyl transferase domain containing protein, expressed

Chr3	31296372	31296372	G	A	G	SNP	OS03G0757200	Intronic	NA	NA	LOC_Os03g55030	UDP-glucoronosyl and UDP-glucosyl transferase domain containing protein, expressed
Chr3	31296374	31296374	G	A	G	SNP	OS03G0757200	Intronic	NA	NA	LOC_Os03g55030	UDP-glucoronosyl and UDP-glucosyl transferase domain containing protein, expressed
Chr3	31296311	31296311	T	A	T	SNP	OS03G0757200	Intronic	NA	NA	LOC_Os03g55030	UDP-glucoronosyl and UDP-glucosyl transferase domain containing protein, expressed
Chr3	12792919	12792919	C	A	C	SNP	OS03G0343300	Exonic	3UTR	NA	LOC_Os03g22320	utp14 protein, putative, expressed
Chr3	12792575	12792575	G	A	G	SNP	OS03G0343300	Intronic	NA	NA	LOC_Os03g22320	utp14 protein, putative, expressed
Chr3	33347499	33347499	G	A	G	SNP	OS03G0799900	Intronic	NA	NA	LOC_Os03g58570	WD domain, G-beta repeat domain containing protein, expressed
Chr3	16287549	16287564	AAAAGATATTATGTAC	A	AAAAGATATTATGTAC	INDEL	OS03G0401100	Intronic	NA	NA	LOC_Os03g28300	protein kinase domain containing protein, expressed
Chr3	35662980	35662988	ACCGATATG	A	ACCGATATG	INDEL	OS03G0848100	Exonic	3UTR	NA	LOC_Os03g63074	Ser/Thr protein phosphatase family protein, expressed
Chr3	9446040	9446042	ACT	A	ACT	INDEL	OS03G0278200	Intronic	NA	NA	LOC_Os03g17000	NAD dependent epimerase/dehydratase family domain containing protein, expressed
Chr3	31605242	31605244	ACT	A	ACT	INDEL	OS03G0764100	Exonic	3UTR	NA	LOC_Os03g55540	ZOS3-18 - C2H2 zinc finger protein, expressed
Chr3	24599159	24599160	AG	A	AG	INDEL	OS03G0639800	Intronic	NA	NA	LOC_Os03g43860	SNF7 domain containing protein, putative, expressed
Chr3	27244224	27244225	AG	A	AG	INDEL	OS03G0683700	Intronic	NA	NA	LOC_Os03g47930	expressed protein
Chr3	33216030	33216032	AGT	A	AGT	INDEL	OS03G0797400	Intronic	NA	NA	LOC_Os03g58300	indole-3-glycerol phosphate lyase, chloroplast precursor, putative, expressed
Chr3	9014388	9014389	AT	A	AT	INDEL	OS03G0269900	Intronic	NA	NA	LOC_Os03g16334	fringe-related protein, putative, expressed
Chr3	15473714	15473715	AT	A	AT	INDEL	OS03G0387900	Exonic	3UTR	NA	LOC_Os03g27019	expressed protein
Chr3	29387027	29387028	AT	A	AT	INDEL	OS03G0723400	Intronic	NA	NA	LOC_Os03g51350	expressed protein
Chr3	31316505	31316506	AT	A	AT	INDEL	OS03G0757900	Intronic	NA	NA	LOC_Os03g55070	UDP-glucose 6-dehydrogenase, putative, expressed
Chr3	15471187	15471202	ATATTAAGTTCTAACT	A	ATATTAAGTTCTAACT	INDEL	OS03G0387900	Intronic	NA	NA	LOC_Os03g27019	expressed protein
Chr3	16754140	16754149	ATCTTCTTCT	A	ATCTTCTTCT	INDEL	OS03G0407900	Exonic	5UTR	NA	LOC_Os03g29410	tyrosine protein kinase domain containing protein, putative, expressed
Chr3	35869414	35869414	G	A	G	SNP	OS03G0851900	Intronic	NA	NA	LOC_Os03g63500	ATPase, AFG1 family domain containing protein, expressed
Chr3	16839726	16839726	G	A	G	SNP	OS03G0409100	Exonic	5UTR	NA	LOC_Os03g29540	ATP-dependent protease, putative, expressed
Chr3	16840227	16840227	G	A	G	SNP	OS03G0409100	Exonic	5UTR	NA	LOC_Os03g29540	ATP-dependent protease, putative, expressed
Chr3	16840240	16840240	G	A	G	SNP	OS03G0409100	Exonic	5UTR	NA	LOC_Os03g29540	ATP-dependent protease, putative, expressed
Chr3	16840244	16840244	G	A	G	SNP	OS03G0409100	Exonic	5UTR	NA	LOC_Os03g29540	ATP-dependent protease, putative, expressed
Chr3	8200930	8200930	G	A	G	SNP	OS03G0255100	Intronic	NA	NA	LOC_Os03g15020	beta-galactosidase precursor, putative, expressed
Chr3	12631425	12631425	T	A	T	SNP	OS03G0339900	Exonic	3UTR	NA	LOC_Os03g22050	CAMK_KIN1/SNF1/Nim1_like.16 - CAMK includes calcium/calmodulin depedent protein kinases, expressed
Chr3	11731364	11731364	G	A	G	SNP	OS03G0323600	Intronic	NA	NA	LOC_Os03g20730	chaperone protein dnaJ, putative, expressed
Chr3	32711203	32711203	T	A	T	SNP	OS03G0787300	Exonic	3UTR	NA	LOC_Os03g57340	chaperone protein dnaJ, putative, expressed
Chr3	2077118	2077118	G	A	G	SNP	OS03G0137700	Exonic	CDS	Silent	LOC_Os03g04480	CorA-like magnesium transporter protein, putative, expressed
Chr3	10906348	10906348	C	A	C	SNP	OS03G0306700	Exonic	CDS	NA	LOC_Os03g19370	CPuORF4 - conserved peptide uORF-containing transcript, expressed
Chr3	31345159	31345159	C	A	C	SNP	OS03G0758300	Exonic	CDS	Silent	LOC_Os03g55100	cyclic nucleotide-gated ion channel 2, putative, expressed
Chr3	9425930	9425930	G	A	G	SNP	OS03G0277700	Exonic	CDS	Missense	LOC_Os03g16960	cysteine-rich repeat secretory protein 55 precursor, putative, expressed
Chr3	22084246	22084246	G	A	G	SNP	OS03G0594100	Exonic	CDS	Silent	LOC_Os03g39690	cytochrome P450, putative, expressed
Chr3	31436671	31436671	G	A	G	SNP	OS03G0760000	Exonic	CDS	Missense	LOC_Os03g55230	cytochrome P450, putative, expressed
Chr3	33834867	33834867	C	A	C	SNP	OS03G0809000	Exonic	3UTR	NA	LOC_Os03g59440	dirigent, putative, expressed
Chr3	7852348	7852348	C	A	C	SNP	OS03G0248600	Intronic	NA	NA	LOC_Os03g14450	enolase, putative, expressed
Chr3	7852392	7852392	C	A	C	SNP	OS03G0248600	Intronic	NA	NA	LOC_Os03g14450	enolase, putative, expressed
Chr3	1496311	1496311	G	A	G	SNP	OS03G0126100	Exonic	CDS	NA	LOC_Os03g03460	erythronate-4-phosphate dehydrogenase, putative, expressed
Chr3	4545652	4545652	G	A	G	SNP	OS03G0187000	Exonic	CDS	Missense	LOC_Os03g08810	expressed protein
Chr3	10692371	10692371	C	A	C	SNP	OS03G0303100	Exonic	5UTR	NA	LOC_Os03g19090	expressed protein
Chr3	12870882	12870882	T	A	T	SNP	OS03G0345200	Intronic	NA	NA	LOC_Os03g22460	expressed protein
Chr3	1240304	1240304	G	A	G	SNP	OS03G0122300	Intronic	NA	NA	LOC_Os03g03034	flavonol synthase/flavanone 3-hydroxylase, putative, expressed
Chr3	33550345	33550345	T	A	T	SNP	OS03G0803900	Exonic	CDS	Missense	LOC_Os03g58920	galactosyltransferase family protein, putative, expressed
Chr3	35026625	35026625	G	A	G	SNP	OS03G0833600	Exonic	CDS	Missense	LOC_Os03g61780	glucan endo-1,3-beta-glucosidase-related, putative, expressed
Chr3	35027475	35027475	C	A	C	SNP	OS03G0833600	Exonic	CDS	Missense	LOC_Os03g61780	glucan endo-1,3-beta-glucosidase-related, putative, expressed
Chr3	35027666	35027666	G	A	G	SNP	OS03G0833600	Intronic	NA	NA	LOC_Os03g61780	glucan endo-1,3-beta-glucosidase-related, putative, expressed
Chr3	35027697	35027697	G	A	G	SNP	OS03G0833600	Intronic	NA	NA	LOC_Os03g61780	glucan endo-1,3-beta-glucosidase-related, putative, expressed
Chr3	35028042	35028042	G	A	G	SNP	OS03G0833600	Intronic	NA	NA	LOC_Os03g61780	glucan endo-1,3-beta-glucosidase-related, putative, expressed
Chr3	10572916	10572916	G	A	G	SNP	OS03G0300600	Intronic	NA	NA	LOC_Os03g18870	heat shock protein DnaJ, putative, expressed
Chr3	35161752	35161752	G	A	G	SNP	OS03G0836800	Intronic	NA	NA	LOC_Os03g62060	hydrolase, putative, expressed
Chr3	35161758	35161758	G	A	G	SNP	OS03G0836800	Intronic	NA	NA	LOC_Os03g62060	hydrolase, putative, expressed
Chr3	35161943	35161943	G	A	G	SNP	OS03G0836800	Intronic	NA	NA	LOC_Os03g62060	hydrolase, putative, expressed
Chr3	35161955	35161955	G	A	G	SNP	OS03G0836800	Intronic	NA	NA	LOC_Os03g62060	hydrolase, putative, expressed
Chr3	35162376	35162376	G	A	G	SNP	OS03G0836800	Intronic	NA	NA	LOC_Os03g62060	hydrolase, putative, expressed
Chr3	35162673	35162673	G	A	G	SNP	OS03G0836800	Exonic	CDS	NA	LOC_Os03g62060	hydrolase, putative, expressed
Chr3	7440933	7440933	G	A	G	SNP	OS03G0240600	Exonic	CDS	Missense	LOC_Os03g13740	immediate-early fungal elicitor protein CMPG1, putative, expressed
Chr3	33215916	33215916	G	A	G	SNP	OS03G0797400	Intronic	NA	NA	LOC_Os03g58300	indole-3-glycerol phosphate lyase, chloroplast precursor, putative, expressed
Chr3	33215981	33215981	G	A	G	SNP	OS03G0797400	Intronic	NA	NA	LOC_Os03g58300	indole-3-glycerol phosphate lyase, chloroplast precursor, putative, expressed
Chr3	33217297	33217297	G	A	G	SNP	OS03G0797400	Exonic	5UTR	NA	LOC_Os03g58300	indole-3-glycerol phosphate lyase, chloroplast precursor, putative, expressed
Chr3	33905995	33905995	G	A	G	SNP	OS03G0810100	Exonic	CDS	Silent	LOC_Os03g59570	IPP transferase, putative, expressed
Chr3	29564494	29564494	T	A	T	SNP	OS03G0726500	Intronic	NA	NA	LOC_Os03g51650	membrane protein, putative, expressed
Chr3	29564513	29564513	G	A	G	SNP	OS03G0726500	Intronic	NA	NA	LOC_Os03g51650	membrane protein, putative, expressed
Chr3	29564522	29564522	T	A	T	SNP	OS03G0726500	Intronic	NA	NA	LOC_Os03g51650	membrane protein, putative, expressed
Chr3	29565587	29565587	G	A	G	SNP	OS03G0726500	Exonic	CDS	Silent	LOC_Os03g51650	membrane protein, putative, expressed
Chr3	9445629	9445629	G	A	G	SNP	OS03G0278200	Intronic	NA	NA	LOC_Os03g17000	NAD dependent epimerase/dehydratase family domain containing protein, expressed
Chr3	2751714	2751714	C	A	C	SNP	OS03G0149300	Intronic	NA	NA	LOC_Os03g05530	nodulin, putative, expressed
Chr3	2751888	2751888	G	A	G	SNP	OS03G0149300	Intronic	NA	NA	LOC_Os03g05530	nodulin, putative, expressed
Chr3	28243874	28243874	G	A	G	SNP	OS03G0703000	Intronic	NA	NA	LOC_Os03g49600	Os3bglu7 - beta-glucosidase, exo-beta-glucanse, expressed
Chr3	28243893	28243893	G	A	G	SNP	OS03G0703000	Intronic	NA	NA	LOC_Os03g49600	Os3bglu7 - beta-glucosidase, exo-beta-glucanse, expressed
Chr3	28244445	28244445	G	A	G	SNP	OS03G0703000	Intronic	NA	NA	LOC_Os03g49600	Os3bglu7 - beta-glucosidase, exo-beta-glucanse, expressed
Chr3	28244558	28244558	C	A	C	SNP	OS03G0703000	Intronic	NA	NA	LOC_Os03g49600	Os3bglu7 - beta-glucosidase, exo-beta-glucanse, expressed
Chr3	30482861	30482861	G	A	G	SNP	OS03G0742900	Intronic	NA	NA	LOC_Os03g53150	OsIAA13 - Auxin-responsive Aux/IAA gene family member, expressed
Chr3	30483019	30483019	C	A	C	SNP	OS03G0742900	Exonic	CDS	Missense	LOC_Os03g53150	OsIAA13 - Auxin-responsive Aux/IAA gene family member, expressed
Chr3	7560064	7560064	G	A	G	SNP	OS03G0242900	Exonic	CDS	Silent	LOC_Os03g13930	OsSub28 - Putative Subtilisin homologue, expressed
Chr3	31489039	31489039	G	A	G	SNP	OS03G0761500	Exonic	CDS	Silent	LOC_Os03g55350	OsSub31 - Putative Subtilisin homologue, expressed
Chr3	8223602	8223602	G	A	G	SNP	OS03G0255500	Intronic	NA	NA	LOC_Os03g15050	phosphoenolpyruvate carboxykinase, putative, expressed
Chr3	7347620	7347620	G	A	G	SNP	OS03G0239200	Exonic	5UTR	NA	LOC_Os03g13590	potyvirus VPg interacting protein, putative, expressed
Chr3	10163593	10163593	G	A	G	SNP	OS03G0292100	Exonic	CDS	Silent	LOC_Os03g18150	protein phosphatase 2C, putative, expressed
Chr3	31474399	31474399	T	A	T	SNP	OS03G0761100	Exonic	CDS	Missense	LOC_Os03g55320	protein phosphatase 2C, putative, expressed
Chr3	10219156	10219156	G	A	G	SNP	OS03G0293500	Exonic	CDS	Silent	LOC_Os03g18220	pyruvate decarboxylase isozyme 2, putative, expressed
Chr3	36006766	36006766	G	A	G	SNP	OS03G0854800	Exonic	5UTR	NA	LOC_Os03g63770	RCD1, putative, expressed
Chr3	24469581	24469581	G	A	G	SNP	OS03G0638400	Intronic	NA	NA	LOC_Os03g43750	retrotransposon, putative, centromere-specific, expressed
Chr3	24469741	24469741	G	A	G	SNP	OS03G0638400	Intronic	NA	NA	LOC_Os03g43750	retrotransposon, putative, centromere-specific, expressed
Chr3	21088467	21088467	G	A	G	SNP	OS03G0576700	Intronic	NA	NA	LOC_Os03g37970	ribosomal protein L13, putative, expressed
Chr3	9451018	9451018	G	A	G	SNP	OS03G0278300	Exonic	CDS	Missense	LOC_Os03g17010	RNA recognition motif containing protein, putative, expressed

Chr3	26629367	26629367	G	A	G	SNP	OS03G0673800	Exonic	3UTR	NA	LOC_Os03g47070	RXW8, putative, expressed
Chr3	26632113	26632113	G	A	G	SNP	OS03G0673800	Exonic	5UTR	NA	LOC_Os03g47070	RXW8, putative, expressed
Chr3	35663657	35663657	C	A	C	SNP	OS03G0848100	Exonic	CDS	NA	LOC_Os03g63074	Ser/Thr protein phosphatase family protein, expressed
Chr3	35663787	35663787	G	A	G	SNP	OS03G0848100	Exonic	CDS	NA	LOC_Os03g63074	Ser/Thr protein phosphatase family protein, expressed
Chr3	25003344	25003344	C	A	C	SNP	OS03G0646200	Exonic	CDS	Silent	LOC_Os03g44430	ubiquitin carboxyl-terminal hydrolase, family 1, putative, expressed
Chr3	32933328	32933328	G	A	G	SNP	OS03G0791800	Intronic	NA	NA	LOC_Os03g57790	ubiquitin-conjugating enzyme, putative, expressed
Chr3	31296641	31296641	G	A	G	SNP	OS03G0757200	Exonic	CDS	Missense	LOC_Os03g55030	UDP-glucoronosyl and UDP-glucosyl transferase domain containing protein, expressed
Chr3	31296867	31296867	G	A	G	SNP	OS03G0757200	Exonic	CDS	Silent	LOC_Os03g55030	UDP-glucoronosyl and UDP-glucosyl transferase domain containing protein, expressed
Chr3	31296876	31296876	C	A	C	SNP	OS03G0757200	Exonic	CDS	Silent	LOC_Os03g55030	UDP-glucoronosyl and UDP-glucosyl transferase domain containing protein, expressed
Chr3	33832361	33832361	C	A	C	SNP	OS03G0808900	Exonic	CDS	Silent	LOC_Os03g59430	uncharacterized glycosyltransferase, putative, expressed
Chr3	35332544	35332544	G	A	G	SNP	OS03G0840200	Exonic	CDS	Missense	LOC_Os03g62370	WD40-like Beta Propeller Repeat family protein, expressed
Chr3	190052	190060	ATATTCATT	A	ATATTCATT	INDEL	OS03G0102700	Intronic	NA	NA	LOC_Os03g01270	expansin precursor, putative, expressed
Chr3	3371693	3371694	AT	A	AT	INDEL	OS03G0162200	Intronic	NA	NA	LOC_Os03g06670	Core histone H2A/H2B/H3/H4 domain containing protein, putative, expressed
Chr3	3381623	3381624	AC	A	AC	INDEL	OS03G0162500	Exonic	3UTR	NA	LOC_Os03g06680	plant-specific domain TIGR01615 family protein, expressed
Chr3	4004794	4004813	ATTCCAATCATCCAATACCG	A	ATTCCAATCATCCAATACCG	INDEL	OS03G0174900	Intronic	NA	NA	LOC_Os03g07880	nuclear transcription factor Y subunit, putative, expressed
Chr3	4829446	4829452	AGCGGCG	A	AGCGGCG	INDEL	OS03G0192700	Exonic	5UTR	NA	LOC_Os03g09250	inositol-3-phosphate synthase, putative, expressed
Chr3	7849682	7849683	AT	A	AT	INDEL	OS03G0248600	Intronic	NA	NA	LOC_Os03g14450	enolase, putative, expressed
Chr3	12224736	12224737	AT	A	AT	INDEL	OS03G0331700	Exonic	3UTR	NA	LOC_Os03g21380	OsCML27 - Calmodulin-related calcium sensor protein, expressed
Chr3	15668252	15668264	ATCCTCCTCCTCC	A	ATCCTCCTCCTCC	INDEL	OS03G0391100	Exonic	CDS	InFrame	LOC_Os03g27360	RING-H2 finger protein ATL5H precursor, putative, expressed
Chr3	16592522	16592525	AAAC	A	AAAC	INDEL	OS03G0405500	Intronic	NA	NA	LOC_Os03g29190	PDI, putative, expressed
Chr3	21635692	21635699	AGCAACTT	A	AGCAACTT	INDEL	OS03G0586500	Intronic	NA	NA	LOC_Os03g38950	chloroplast post-illumination chlorophyll fluorescence increase protein, putative, expressed
Chr3	24471769	24471803	AACTCTCGTGGAATTTGTTAGAAATAAATCCTACC	A	AACTCTCGTGGAATTTGTTAGAAATAAATCCTACC	INDEL	OS03G0638400	Exonic	5UTR	NA	LOC_Os03g43750	retrotransposon, putative, centromere-specific, expressed
Chr3	31347210	31347211	AG	A	AG	INDEL	OS03G0758300	Intronic	NA	NA	LOC_Os03g55100	cyclic nucleotide-gated ion channel 2, putative, expressed
Chr3	34828872	34828883	ATGCTCTCTAGC	A	ATGCTCTCTAGC	INDEL	OS03G0829200	Exonic	CDS	FrameShift	LOC_Os03g61360	hydrolase, alpha/beta fold family domain containing protein, expressed
Chr3	35129009	35129014	ACATGG	A	ACATGG	INDEL	OS03G0836000	Intronic	NA	NA	LOC_Os03g61970	actin, putative, expressed
Chr3	35161678	35161695	ATAAGGGACCATAACCCT	A	ATAAGGGACCATAACCCT	INDEL	OS03G0836800	Intronic	NA	NA	LOC_Os03g62060	hydrolase, putative, expressed
Chr3	35380205	35380209	ATAAG	A	ATAAG	INDEL	OS03G0841600	Exonic	3UTR	NA	LOC_Os03g62480	anthocyanidin 5,3-O-glucosyltransferase, putative, expressed
Chr6	301582	301582	T	A	T	SNP	OS06G0104300	Exonic	CDS	Silent	LOC_Os06g01490	monocopper oxidase, putative, expressed
Chr6	9326252	9326252	G	A	G	SNP	OS06G0274800	Exonic	3UTR	NA	LOC_Os06g16350	peroxidase precursor, putative, expressed
Chr6	20832720	20832720	G	A	G	SNP	OS06G0549900	Exonic	3UTR	NA	LOC_Os06g35700	reticuline oxidase-like protein precursor, putative, expressed
Chr6	11699695	11699695	G	A	G	SNP	OS06G0308300	Exonic	CDS	Silent	LOC_Os06g20370	microtubule associated protein, putative, expressed
Chr6	678983	678983	C	A	C	SNP	OS06G0112100	Exonic	3UTR	NA	LOC_Os06g02210	bark storage protein A precursor, putative, expressed
Chr6	2321491	2321491	T	A	T	SNP	OS06G0143900	Intronic	NA	NA	LOC_Os06g05180	coatomer subunit beta, putative, expressed
Chr6	13480445	13480445	T	A	T	SNP	OS06G0338200	Intronic	NA	NA	LOC_Os06g23114	copper methylamine oxidase precursor, putative, expressed
Chr6	31128428	31128428	T	A	T	SNP	OS06G0730300	Intronic	NA	NA	LOC_Os06g51390	expressed protein
Chr6	3450821	3450821	T	A	T	SNP	OS06G0168600	Exonic	CDS	Missense	LOC_Os06g07210	ribonucleoside-diphosphate reductase large subunit, putative, expressed
Chr6	5281986	5281986	T	A	T	SNP	OS06G0204400	Intronic	NA	NA	LOC_Os06g10280	CDP-alcohol phosphatidyltransferase, putative, expressed
Chr6	4324014	4324014	T	A	T	SNP	OS06G0185900	Intronic	NA	NA	LOC_Os06g08670	glutathione peroxidase, putative, expressed
Chr6	3361468	3361468	G	A	G	SNP	OS06G0166900	Intronic	NA	NA	LOC_Os06g07070	BRASSINOSTEROID INSENSITIVE 1-associated receptor kinase 1 precursor, putative, expressed
Chr6	2321645	2321645	G	A	G	SNP	OS06G0143900	Intronic	NA	NA	LOC_Os06g05180	coatomer subunit beta, putative, expressed
Chr6	2325446	2325446	G	A	G	SNP	OS06G0143900	Intronic	NA	NA	LOC_Os06g05180	coatomer subunit beta, putative, expressed
Chr6	2325551	2325551	G	A	G	SNP	OS06G0143900	Intronic	NA	NA	LOC_Os06g05180	coatomer subunit beta, putative, expressed
Chr6	2327077	2327077	G	A	G	SNP	OS06G0143900	Intronic	NA	NA	LOC_Os06g05180	coatomer subunit beta, putative, expressed
Chr6	2328355	2328355	G	A	G	SNP	OS06G0143900	Intronic	NA	NA	LOC_Os06g05180	coatomer subunit beta, putative, expressed
Chr6	2329126	2329126	G	A	G	SNP	OS06G0143900	Exonic	CDS	Silent	LOC_Os06g05180	coatomer subunit beta, putative, expressed
Chr6	13477923	13477923	G	A	G	SNP	OS06G0338200	Intronic	NA	NA	LOC_Os06g23114	copper methylamine oxidase precursor, putative, expressed
Chr6	13478276	13478276	G	A	G	SNP	OS06G0338200	Intronic	NA	NA	LOC_Os06g23114	copper methylamine oxidase precursor, putative, expressed
Chr6	13480191	13480191	G	A	G	SNP	OS06G0338200	Intronic	NA	NA	LOC_Os06g23114	copper methylamine oxidase precursor, putative, expressed
Chr6	13481933	13481933	G	A	G	SNP	OS06G0338200	Intronic	NA	NA	LOC_Os06g23114	copper methylamine oxidase precursor, putative, expressed
Chr6	308058	308058	G	A	G	SNP	OS06G0104400	Exonic	CDS	Silent	LOC_Os06g01500	IQ calmodulin-binding motif family protein, putative, expressed
Chr6	26650293	26650293	G	A	G	SNP	OS06G0651000	Exonic	3UTR	NA	LOC_Os06g44170	NAD dependent epimerase/dehydratase family protein, putative, expressed
Chr6	31138309	31138309	G	A	G	SNP	OS06G0730600	Intronic	NA	NA	LOC_Os06g51410	OsPOP15 - Putative Prolyl Oligopeptidase homologue, expressed
Chr6	31139633	31139633	G	A	G	SNP	OS06G0730600	Intronic	NA	NA	LOC_Os06g51410	OsPOP15 - Putative Prolyl Oligopeptidase homologue, expressed
Chr6	31142024	31142024	G	A	G	SNP	OS06G0730600	Intronic	NA	NA	LOC_Os06g51410	OsPOP15 - Putative Prolyl Oligopeptidase homologue, expressed
Chr6	26677554	26677554	G	A	G	SNP	OS06G0651900	Exonic	3UTR	NA	LOC_Os06g44220	OsRCI2-9 - Putative low temperature and salt responsive protein, expressed
Chr6	3642391	3642391	G	A	G	SNP	OS06G0172000	Exonic	CDS	Missense	LOC_Os06g07550	PPR repeat domain containing protein, putative, expressed
Chr6	17067715	17067715	G	A	G	SNP	OS06G0493100	Intronic	NA	NA	LOC_Os06g29730	RALFL28 - Rapid ALkalinization Factor RALF family protein precursor, expressed
Chr6	31127882	31127882	C	A	C	SNP	OS06G0730300	Intronic	NA	NA	LOC_Os06g51390	expressed protein
Chr6	25834236	25834236	C	A	C	SNP	OS06G0636100	Intronic	NA	NA	LOC_Os06g43000	nitrate-induced NOI protein, expressed
Chr6	17067705	17067705	C	A	C	SNP	OS06G0493100	Intronic	NA	NA	LOC_Os06g29730	RALFL28 - Rapid ALkalinization Factor RALF family protein precursor, expressed
Chr6	3445221	3445221	C	A	C	SNP	OS06G0168500	Exonic	3UTR	NA	LOC_Os06g07200	syntaxin, putative, expressed
Chr6	5282103	5282103	C	A	C	SNP	OS06G0204400	Intronic	NA	NA	LOC_Os06g10280	CDP-alcohol phosphatidyltransferase, putative, expressed
Chr6	643930	643930	G	A	G	SNP	OS06G0111500	Intronic	NA	NA	LOC_Os06g02144	6-phosphogluconate dehydrogenase, decarboxylating, putative, expressed
Chr6	23107922	23107922	T	A	T	SNP	OS06G0589300	Intronic	NA	NA	LOC_Os06g38950	ABC transporter, ATP-binding protein, putative, expressed
Chr6	23108338	23108338	G	A	G	SNP	OS06G0589300	Intronic	NA	NA	LOC_Os06g38950	ABC transporter, ATP-binding protein, putative, expressed
Chr6	23108362	23108362	C	A	C	SNP	OS06G0589300	Intronic	NA	NA	LOC_Os06g38950	ABC transporter, ATP-binding protein, putative, expressed
Chr6	23108421	23108421	T	A	T	SNP	OS06G0589300	Intronic	NA	NA	LOC_Os06g38950	ABC transporter, ATP-binding protein, putative, expressed
Chr6	23109570	23109570	G	A	G	SNP	OS06G0589300	Intronic	NA	NA	LOC_Os06g38950	ABC transporter, ATP-binding protein, putative, expressed
Chr6	23110154	23110154	C	A	C	SNP	OS06G0589300	Intronic	NA	NA	LOC_Os06g38950	ABC transporter, ATP-binding protein, putative, expressed
Chr6	23111861	23111861	T	A	T	SNP	OS06G0589300	Intronic	NA	NA	LOC_Os06g38950	ABC transporter, ATP-binding protein, putative, expressed
Chr6	23111977	23111977	G	A	G	SNP	OS06G0589300	Exonic	CDS	Silent	LOC_Os06g38950	ABC transporter, ATP-binding protein, putative, expressed
Chr6	23112058	23112058	G	A	G	SNP	OS06G0589300	Exonic	CDS	Silent	LOC_Os06g38950	ABC transporter, ATP-binding protein, putative, expressed
Chr6	23112384	23112384	C	A	C	SNP	OS06G0589300	Intronic	NA	NA	LOC_Os06g38950	ABC transporter, ATP-binding protein, putative, expressed
Chr6	23112578	23112578	G	A	G	SNP	OS06G0589300	Intronic	NA	NA	LOC_Os06g38950	ABC transporter, ATP-binding protein, putative, expressed
Chr6	2951446	2951446	G	A	G	SNP	OS06G0158000	Intronic	NA	NA	LOC_Os06g06350	AMP-binding enzyme, putative, expressed
Chr6	2952384	2952384	G	A	G	SNP	OS06G0158000	Intronic	NA	NA	LOC_Os06g06350	AMP-binding enzyme, putative, expressed
Chr6	2952603	2952603	C	A	C	SNP	OS06G0158000	Intronic	NA	NA	LOC_Os06g06350	AMP-binding enzyme, putative, expressed
Chr6	681465	681465	G	A	G	SNP	OS06G0112100	Intronic	NA	NA	LOC_Os06g02210	bark storage protein A precursor, putative, expressed
Chr6	16264302	16264302	G	A	G	SNP	OS06G0480000	Exonic	3UTR	NA	LOC_Os06g28590	bg55, putative, expressed
Chr6	16265025	16265025	G	A	G	SNP	OS06G0480000	Exonic	CDS	Silent	LOC_Os06g28590	bg55, putative, expressed
Chr6	30306663	30306663	C	A	C	SNP	OS06G0714200	Intronic	NA	NA	LOC_Os06g50030	CAMK_CAMK_like.30 - CAMK includes calcium/calmodulin depedent protein kinases, expressed
Chr6	30306730	30306730	G	A	G	SNP	OS06G0714200	Intronic	NA	NA	LOC_Os06g50030	CAMK_CAMK_like.30 - CAMK includes calcium/calmodulin depedent protein kinases, expressed
Chr6	9564398	9564398	G	A	G	SNP	OS06G0278000	Exonic	CDS	Silent	LOC_Os06g16640	carboxyl-terminal peptidase, putative, expressed
Chr6	9564539	9564539	G	A	G	SNP	OS06G0278000	Exonic	CDS	Silent	LOC_Os06g16640	carboxyl-terminal peptidase, putative, expressed
Chr6	9564542	9564542	G	A	G	SNP	OS06G0278000	Exonic	CDS	Silent	LOC_Os06g16640	carboxyl-terminal peptidase, putative, expressed

Chr6	9564759	9564759	G	A	G	SNP	OS06G0278000	Intronic	NA	NA	LOC_Os06g16640	carboxyl-terminal peptidase, putative, expressed
Chr6	9565009	9565009	G	A	G	SNP	OS06G0278000	Exonic	CDS	Silent	LOC_Os06g16640	carboxyl-terminal peptidase, putative, expressed
Chr6	9565501	9565501	G	A	G	SNP	OS06G0278000	Intronic	NA	NA	LOC_Os06g16640	carboxyl-terminal peptidase, putative, expressed
Chr6	9565731	9565731	G	A	G	SNP	OS06G0278000	Intronic	NA	NA	LOC_Os06g16640	carboxyl-terminal peptidase, putative, expressed
Chr6	9565967	9565967	C	A	C	SNP	OS06G0278000	Exonic	CDS	Missense	LOC_Os06g16640	carboxyl-terminal peptidase, putative, expressed
Chr6	9566025	9566025	G	A	G	SNP	OS06G0278000	Exonic	CDS	Silent	LOC_Os06g16640	carboxyl-terminal peptidase, putative, expressed
Chr6	9566319	9566319	G	A	G	SNP	OS06G0278000	Intronic	NA	NA	LOC_Os06g16640	carboxyl-terminal peptidase, putative, expressed
Chr6	9566403	9566403	G	A	G	SNP	OS06G0278000	Intronic	NA	NA	LOC_Os06g16640	carboxyl-terminal peptidase, putative, expressed
Chr6	9566500	9566500	C	A	C	SNP	OS06G0278000	Intronic	NA	NA	LOC_Os06g16640	carboxyl-terminal peptidase, putative, expressed
Chr6	9566540	9566540	T	A	T	SNP	OS06G0278000	Intronic	NA	NA	LOC_Os06g16640	carboxyl-terminal peptidase, putative, expressed
Chr6	9566638	9566638	G	A	G	SNP	OS06G0278000	Exonic	CDS	Silent	LOC_Os06g16640	carboxyl-terminal peptidase, putative, expressed
Chr6	24144623	24144623	G	A	G	SNP	OS06G0607200	Intronic	NA	NA	LOC_Os06g40510	CRAL/TRIO domain containing protein, expressed
Chr6	24147088	24147088	C	A	C	SNP	OS06G0607200	Intronic	NA	NA	LOC_Os06g40510	CRAL/TRIO domain containing protein, expressed
Chr6	414272	414272	G	A	G	SNP	OS06G0106100	Intronic	NA	NA	LOC_Os06g01700	CWC15 homolog A, putative, expressed
Chr6	414351	414351	G	A	G	SNP	OS06G0106100	Intronic	NA	NA	LOC_Os06g01700	CWC15 homolog A, putative, expressed
Chr6	414354	414354	G	A	G	SNP	OS06G0106100	Intronic	NA	NA	LOC_Os06g01700	CWC15 homolog A, putative, expressed
Chr6	415424	415424	G	A	G	SNP	OS06G0106100	Intronic	NA	NA	LOC_Os06g01700	CWC15 homolog A, putative, expressed
Chr6	8179160	8179160	T	A	T	SNP	OS06G0256900	Exonic	3UTR	NA	LOC_Os06g14540	endoglucanase, putative, expressed
Chr6	21363574	21363574	G	A	G	SNP	OS06G0558900	Exonic	3UTR	NA	LOC_Os06g36380	expressed protein
Chr6	23134905	23134905	G	A	G	SNP	OS06G0589600	Intronic	NA	NA	LOC_Os06g38970	expressed protein
Chr6	23134924	23134924	G	A	G	SNP	OS06G0589600	Intronic	NA	NA	LOC_Os06g38970	expressed protein
Chr6	23135054	23135054	G	A	G	SNP	OS06G0589600	Exonic	CDS	NA	LOC_Os06g38970	expressed protein
Chr6	23135509	23135509	G	A	G	SNP	OS06G0589600	Intronic	NA	NA	LOC_Os06g38970	expressed protein
Chr6	23135779	23135779	G	A	G	SNP	OS06G0589600	Exonic	CDS	NA	LOC_Os06g38970	expressed protein
Chr6	23227370	23227370	G	A	G	SNP	OS06G0591200	Intronic	NA	NA	LOC_Os06g39110	expressed protein
Chr6	23227384	23227384	G	A	G	SNP	OS06G0591200	Intronic	NA	NA	LOC_Os06g39110	expressed protein
Chr6	23227543	23227543	G	A	G	SNP	OS06G0591200	Intronic	NA	NA	LOC_Os06g39110	expressed protein
Chr6	23227587	23227587	G	A	G	SNP	OS06G0591200	Intronic	NA	NA	LOC_Os06g39110	expressed protein
Chr6	23228090	23228090	G	A	G	SNP	OS06G0591200	Exonic	3UTR	NA	LOC_Os06g39110	expressed protein
Chr6	23228140	23228140	G	A	G	SNP	OS06G0591200	Exonic	3UTR	NA	LOC_Os06g39110	expressed protein
Chr6	23228168	23228168	G	A	G	SNP	OS06G0591200	Exonic	3UTR	NA	LOC_Os06g39110	expressed protein
Chr6	23232869	23232869	T	A	T	SNP	OS06G0591400	Intronic	NA	NA	LOC_Os06g39120	expressed protein
Chr6	25933783	25933783	G	A	G	SNP	OS06G0638200	Intronic	NA	NA	LOC_Os06g43140	GA16396-PA, putative, expressed
Chr6	25934702	25934702	T	A	T	SNP	OS06G0638200	Intronic	NA	NA	LOC_Os06g43140	GA16396-PA, putative, expressed
Chr6	8848363	8848363	G	A	G	SNP	OS06G0266800	Exonic	3UTR	NA	LOC_Os06g15620	GASR7 - Gibberellin-regulated GASA/GAST/Snakin family protein precursor, expressed
Chr6	4322182	4322182	G	A	G	SNP	OS06G0185900	Intronic	NA	NA	LOC_Os06g08670	glutathione peroxidase, putative, expressed
Chr6	4322216	4322216	C	A	C	SNP	OS06G0185900	Intronic	NA	NA	LOC_Os06g08670	glutathione peroxidase, putative, expressed
Chr6	4322235	4322235	C	A	C	SNP	OS06G0185900	Intronic	NA	NA	LOC_Os06g08670	glutathione peroxidase, putative, expressed
Chr6	4323551	4323551	G	A	G	SNP	OS06G0185900	Exonic	CDS	Silent	LOC_Os06g08670	glutathione peroxidase, putative, expressed
Chr6	4323637	4323637	G	A	G	SNP	OS06G0185900	Intronic	NA	NA	LOC_Os06g08670	glutathione peroxidase, putative, expressed
Chr6	4323807	4323807	G	A	G	SNP	OS06G0185900	Intronic	NA	NA	LOC_Os06g08670	glutathione peroxidase, putative, expressed
Chr6	4323854	4323854	C	A	C	SNP	OS06G0185900	Intronic	NA	NA	LOC_Os06g08670	glutathione peroxidase, putative, expressed
Chr6	4323901	4323901	G	A	G	SNP	OS06G0185900	Intronic	NA	NA	LOC_Os06g08670	glutathione peroxidase, putative, expressed
Chr6	4323930	4323930	G	A	G	SNP	OS06G0185900	Intronic	NA	NA	LOC_Os06g08670	glutathione peroxidase, putative, expressed
Chr6	4324186	4324186	G	A	G	SNP	OS06G0185900	Intronic	NA	NA	LOC_Os06g08670	glutathione peroxidase, putative, expressed
Chr6	4324318	4324318	T	A	T	SNP	OS06G0185900	Intronic	NA	NA	LOC_Os06g08670	glutathione peroxidase, putative, expressed
Chr6	4324329	4324329	G	A	G	SNP	OS06G0185900	Intronic	NA	NA	LOC_Os06g08670	glutathione peroxidase, putative, expressed
Chr6	4324374	4324374	G	A	G	SNP	OS06G0185900	Intronic	NA	NA	LOC_Os06g08670	glutathione peroxidase, putative, expressed
Chr6	4324770	4324770	G	A	G	SNP	OS06G0185900	Intronic	NA	NA	LOC_Os06g08670	glutathione peroxidase, putative, expressed
Chr6	4324946	4324946	G	A	G	SNP	OS06G0185900	Exonic	3UTR	NA	LOC_Os06g08670	glutathione peroxidase, putative, expressed
Chr6	4324953	4324953	T	A	T	SNP	OS06G0185900	Exonic	3UTR	NA	LOC_Os06g08670	glutathione peroxidase, putative, expressed
Chr6	24402355	24402355	C	A	C	SNP	OS06G0611900	Intronic	NA	NA	LOC_Os06g40940	glycine dehydrogenase, putative, expressed
Chr6	24402755	24402755	G	A	G	SNP	OS06G0611900	Intronic	NA	NA	LOC_Os06g40940	glycine dehydrogenase, putative, expressed
Chr6	24402758	24402758	G	A	G	SNP	OS06G0611900	Intronic	NA	NA	LOC_Os06g40940	glycine dehydrogenase, putative, expressed
Chr6	14648047	14648047	T	A	T	SNP	OS06G0356800	Exonic	5UTR	NA	LOC_Os06g25010	glycosyl hydrolase, putative, expressed
Chr6	4869425	4869425	G	A	G	SNP	OS06G0195800	Intronic	NA	NA	LOC_Os06g09560	heat shock protein DnaJ, putative, expressed
Chr6	4870073	4870073	C	A	C	SNP	OS06G0195800	Exonic	3UTR	NA	LOC_Os06g09560	heat shock protein DnaJ, putative, expressed
Chr6	23854228	23854228	G	A	G	SNP	OS06G0603000	Exonic	CDS	Silent	LOC_Os06g40080	heme oxygenase 1, putative, expressed
Chr6	3040410	3040410	G	A	G	SNP	OS06G0159450	Exonic	3UTR	NA	LOC_Os06g06460	histone H3, putative, expressed
Chr6	3040451	3040451	G	A	G	SNP	OS06G0159450	Exonic	3UTR	NA	LOC_Os06g06460	histone H3, putative, expressed
Chr6	4653657	4653657	G	A	G	SNP	OS06G0192400	Exonic	CDS	Silent	LOC_Os06g09270	hypro1, putative, expressed
Chr6	306028	306028	T	A	T	SNP	OS06G0104400	Exonic	3UTR	NA	LOC_Os06g01500	IQ calmodulin-binding motif family protein, putative, expressed
Chr6	308584	308584	T	A	T	SNP	OS06G0104400	Exonic	CDS	Missense	LOC_Os06g01500	IQ calmodulin-binding motif family protein, putative, expressed
Chr6	21893927	21893927	G	A	G	SNP	OS06G0567200	Exonic	CDS	Silent	LOC_Os06g37080	L-ascorbate oxidase precursor, putative, expressed
Chr6	21894979	21894979	G	A	G	SNP	OS06G0567200	Intronic	NA	NA	LOC_Os06g37080	L-ascorbate oxidase precursor, putative, expressed
Chr6	21896350	21896350	G	A	G	SNP	OS06G0567200	Intronic	NA	NA	LOC_Os06g37080	L-ascorbate oxidase precursor, putative, expressed
Chr6	6103596	6103596	G	A	G	SNP	OS06G0218900	Intronic	NA	NA	LOC_Os06g11520	LMBR1 integral membrane protein, putative, expressed
Chr6	26377194	26377194	G	A	G	SNP	OS06G0646000	Intronic	NA	NA	LOC_Os06g43800	methyltransferase domain containing protein, putative, expressed
Chr6	11697222	11697222	G	A	G	SNP	OS06G0308300	Exonic	CDS	Silent	LOC_Os06g20370	microtubule associated protein, putative, expressed
Chr6	11699455	11699455	G	A	G	SNP	OS06G0308300	Intronic	NA	NA	LOC_Os06g20370	microtubule associated protein, putative, expressed
Chr6	11699671	11699671	G	A	G	SNP	OS06G0308300	Exonic	CDS	Silent	LOC_Os06g20370	microtubule associated protein, putative, expressed
Chr6	11699888	11699888	G	A	G	SNP	OS06G0308300	Intronic	NA	NA	LOC_Os06g20370	microtubule associated protein, putative, expressed
Chr6	11699966	11699966	T	A	T	SNP	OS06G0308300	Intronic	NA	NA	LOC_Os06g20370	microtubule associated protein, putative, expressed
Chr6	11700196	11700196	T	A	T	SNP	OS06G0308300	Intronic	NA	NA	LOC_Os06g20370	microtubule associated protein, putative, expressed
Chr6	11700259	11700259	G	A	G	SNP	OS06G0308300	Intronic	NA	NA	LOC_Os06g20370	microtubule associated protein, putative, expressed
Chr6	11700653	11700653	C	A	C	SNP	OS06G0308300	Intronic	NA	NA	LOC_Os06g20370	microtubule associated protein, putative, expressed
Chr6	299550	299550	G	A	G	SNP	OS06G0104300	Intronic	NA	NA	LOC_Os06g01490	monocopper oxidase, putative, expressed
Chr6	23468936	23468936	G	A	G	SNP	OS06G0596300	Intronic	NA	NA	LOC_Os06g39520	myristoyl-acyl carrier protein thioesterase, chloroplast precursor, putative, expressed
Chr6	23469490	23469490	C	A	C	SNP	OS06G0596300	Intronic	NA	NA	LOC_Os06g39520	myristoyl-acyl carrier protein thioesterase, chloroplast precursor, putative, expressed
Chr6	23470871	23470871	G	A	G	SNP	OS06G0596300	Exonic	3UTR	NA	LOC_Os06g39520	myristoyl-acyl carrier protein thioesterase, chloroplast precursor, putative, expressed
Chr6	2987313	2987313	T	A	T	SNP	OS06G0158500	Exonic	3UTR	NA	LOC_Os06g06400	NBS-LRR type disease resistance protein, putative, expressed
Chr6	2987468	2987468	G	A	G	SNP	OS06G0158500	Exonic	3UTR	NA	LOC_Os06g06400	NBS-LRR type disease resistance protein, putative, expressed
Chr6	2990173	2990173	C	A	C	SNP	OS06G0158500	Exonic	3UTR	NA	LOC_Os06g06400	NBS-LRR type disease resistance protein, putative, expressed
Chr6	25833993	25833993	C	A	C	SNP	OS06G0636100	Intronic	NA	NA	LOC_Os06g43000	nitrate-induced NOI protein, expressed
Chr6	25835983	25835983	G	A	G	SNP	OS06G0636100	Intronic	NA	NA	LOC_Os06g43000	nitrate-induced NOI protein, expressed

Chr6	25836011	25836011	G	A	G	SNP	OS06G0636100	Intronic	NA	NA	LOC_Os06g43000	nitrate-induced NOI protein, expressed
Chr6	25836058	25836058	G	A	G	SNP	OS06G0636100	Intronic	NA	NA	LOC_Os06g43000	nitrate-induced NOI protein, expressed
Chr6	25836129	25836129	G	A	G	SNP	OS06G0636100	Intronic	NA	NA	LOC_Os06g43000	nitrate-induced NOI protein, expressed
Chr6	25836219	25836219	C	A	C	SNP	OS06G0636100	Intronic	NA	NA	LOC_Os06g43000	nitrate-induced NOI protein, expressed
Chr6	9326965	9326965	G	A	G	SNP	OS06G0274800	Intronic	NA	NA	LOC_Os06g16350	peroxidase precursor, putative, expressed
Chr6	9327338	9327338	G	A	G	SNP	OS06G0274800	Intronic	NA	NA	LOC_Os06g16350	peroxidase precursor, putative, expressed
Chr6	20896602	20896602	G	A	G	SNP	OS06G0551400	Exonic	CDS	Missense	LOC_Os06g35814	ras-related protein, putative, expressed
Chr6	20897058	20897058	G	A	G	SNP	OS06G0551400	Intronic	NA	NA	LOC_Os06g35814	ras-related protein, putative, expressed
Chr6	20897123	20897123	G	A	G	SNP	OS06G0551400	Intronic	NA	NA	LOC_Os06g35814	ras-related protein, putative, expressed
Chr6	20897691	20897691	C	A	C	SNP	OS06G0551400	Intronic	NA	NA	LOC_Os06g35814	ras-related protein, putative, expressed
Chr6	20897995	20897995	G	A	G	SNP	OS06G0551400	Intronic	NA	NA	LOC_Os06g35814	ras-related protein, putative, expressed
Chr6	20899899	20899899	G	A	G	SNP	OS06G0551400	Intronic	NA	NA	LOC_Os06g35814	ras-related protein, putative, expressed
Chr6	20901101	20901101	C	A	C	SNP	OS06G0551400	Intronic	NA	NA	LOC_Os06g35814	ras-related protein, putative, expressed
Chr6	22864300	22864300	C	A	C	SNP	OS06G0585950	Exonic	CDS	Missense	LOC_Os06g38590	receptor-like protein kinase precursor, putative, expressed
Chr6	20832698	20832698	T	A	T	SNP	OS06G0549900	Exonic	3UTR	NA	LOC_Os06g35700	reticuline oxidase-like protein precursor, putative, expressed
Chr6	26715365	26715365	G	A	G	SNP	OS06G0652600	Exonic	CDS	Missense	LOC_Os06g44280	retrotransposon protein, putative, Ty3-gypsy subclass, expressed
Chr6	26716083	26716083	G	A	G	SNP	OS06G0652600	Intronic	NA	NA	LOC_Os06g44280	retrotransposon protein, putative, Ty3-gypsy subclass, expressed
Chr6	1471635	1471635	G	A	G	SNP	OS06G0127900	Exonic	CDS	Missense	LOC_Os06g03720	ribonucleoside-diphosphate reductase small chain, putative, expressed
Chr6	9283694	9283694	C	A	C	SNP	OS06G0274200	Intronic	NA	NA	LOC_Os06g16290	ribosomal protein L7Ae, putative, expressed
Chr6	9283908	9283908	G	A	G	SNP	OS06G0274200	Intronic	NA	NA	LOC_Os06g16290	ribosomal protein L7Ae, putative, expressed
Chr6	9284278	9284278	G	A	G	SNP	OS06G0274200	Intronic	NA	NA	LOC_Os06g16290	ribosomal protein L7Ae, putative, expressed
Chr6	9284575	9284575	C	A	C	SNP	OS06G0274200	Intronic	NA	NA	LOC_Os06g16290	ribosomal protein L7Ae, putative, expressed
Chr6	9284781	9284781	G	A	G	SNP	OS06G0274200	Intronic	NA	NA	LOC_Os06g16290	ribosomal protein L7Ae, putative, expressed
Chr6	9285103	9285103	G	A	G	SNP	OS06G0274200	Exonic	3UTR	NA	LOC_Os06g16290	ribosomal protein L7Ae, putative, expressed
Chr6	13042305	13042305	T	A	T	SNP	OS06G0329900	Intronic	NA	NA	LOC_Os06g22440	SAM dependent carboxyl methyltransferase, putative, expressed
Chr6	10686850	10686850	G	A	G	SNP	OS06G0291500	Exonic	3UTR	NA	LOC_Os06g18820	serine threonine kinase, putative, expressed
Chr6	10687590	10687590	T	A	T	SNP	OS06G0291500	Exonic	3UTR	NA	LOC_Os06g18820	serine threonine kinase, putative, expressed
Chr6	22353297	22353297	T	A	T	SNP	OS06G0575400	Exonic	CDS	Nonsense	LOC_Os06g37750	S-locus-like receptor protein kinase, putative, expressed
Chr6	23305258	23305258	T	A	T	SNP	OS06G0593100	Intronic	NA	NA	LOC_Os06g39260	solute carrier family 35 member B1, putative, expressed
Chr6	23305445	23305445	C	A	C	SNP	OS06G0593100	Intronic	NA	NA	LOC_Os06g39260	solute carrier family 35 member B1, putative, expressed
Chr6	19615919	19615919	T	A	T	SNP	OS06G0528600	Intronic	NA	NA	LOC_Os06g33710	spermidine synthase, putative, expressed
Chr6	19616074	19616074	G	A	G	SNP	OS06G0528600	Intronic	NA	NA	LOC_Os06g33710	spermidine synthase, putative, expressed
Chr6	19617498	19617498	T	A	T	SNP	OS06G0528600	Intronic	NA	NA	LOC_Os06g33710	spermidine synthase, putative, expressed
Chr6	19618112	19618112	G	A	G	SNP	OS06G0528600	Intronic	NA	NA	LOC_Os06g33710	spermidine synthase, putative, expressed
Chr6	19618155	19618155	G	A	G	SNP	OS06G0528600	Intronic	NA	NA	LOC_Os06g33710	spermidine synthase, putative, expressed
Chr6	19619375	19619375	G	A	G	SNP	OS06G0528600	Exonic	3UTR	NA	LOC_Os06g33710	spermidine synthase, putative, expressed
Chr6	21598338	21598338	T	A	T	SNP	OS06G0562600	Exonic	3UTR	NA	LOC_Os06g36700	T-complex protein, putative, expressed
Chr6	21598932	21598932	G	A	G	SNP	OS06G0562600	Intronic	NA	NA	LOC_Os06g36700	T-complex protein, putative, expressed
Chr6	21600364	21600364	G	A	G	SNP	OS06G0562600	Exonic	CDS	Silent	LOC_Os06g36700	T-complex protein, putative, expressed
Chr6	21601896	21601896	G	A	G	SNP	OS06G0562600	Intronic	NA	NA	LOC_Os06g36700	T-complex protein, putative, expressed
Chr6	1555055	1555055	G	A	G	SNP	OS06G0129400	Exonic	5UTR	NA	LOC_Os06g03860	uncharacterized membrane protein, putative, expressed
Chr6	1556368	1556368	G	A	G	SNP	OS06G0129400	Intronic	NA	NA	LOC_Os06g03860	uncharacterized membrane protein, putative, expressed
Chr6	1557099	1557099	G	A	G	SNP	OS06G0129400	Intronic	NA	NA	LOC_Os06g03860	uncharacterized membrane protein, putative, expressed
Chr6	26305984	26305984	G	A	G	SNP	OS06G0644600	Exonic	CDS	Missense	LOC_Os06g43690	WD repeat-containing protein, putative, expressed
Chr6	26306094	26306094	T	A	T	SNP	OS06G0644600	Intronic	NA	NA	LOC_Os06g43690	WD repeat-containing protein, putative, expressed
Chr6	26308919	26308919	C	A	C	SNP	OS06G0644600	Intronic	NA	NA	LOC_Os06g43690	WD repeat-containing protein, putative, expressed
Chr6	26308995	26308995	T	A	T	SNP	OS06G0644600	Intronic	NA	NA	LOC_Os06g43690	WD repeat-containing protein, putative, expressed
Chr6	26312357	26312357	T	A	T	SNP	OS06G0644600	Exonic	3UTR	NA	LOC_Os06g43690	WD repeat-containing protein, putative, expressed
Chr6	26510031	26510031	C	A	C	SNP	OS06G0649000	Exonic	3UTR	NA	LOC_Os06g44010	WRKY28, expressed
Chr6	25919933	25919933	G	A	G	SNP	OS06G0638000	Exonic	CDS	Missense	LOC_Os06g43120	zinc finger C-x8-C-x5-C-x3-H type family protein, expressed
Chr6	25920087	25920087	T	A	T	SNP	OS06G0638000	Exonic	CDS	Missense	LOC_Os06g43120	zinc finger C-x8-C-x5-C-x3-H type family protein, expressed
Chr6	25920336	25920336	T	A	T	SNP	OS06G0638000	Exonic	CDS	Missense	LOC_Os06g43120	zinc finger C-x8-C-x5-C-x3-H type family protein, expressed
Chr6	25921222	25921222	G	A	G	SNP	OS06G0638000	Exonic	CDS	Silent	LOC_Os06g43120	zinc finger C-x8-C-x5-C-x3-H type family protein, expressed
Chr6	25922019	25922019	G	A	G	SNP	OS06G0638000	Exonic	CDS	Missense	LOC_Os06g43120	zinc finger C-x8-C-x5-C-x3-H type family protein, expressed
Chr6	25922497	25922497	T	A	T	SNP	OS06G0638000	Exonic	CDS	Silent	LOC_Os06g43120	zinc finger C-x8-C-x5-C-x3-H type family protein, expressed
Chr6	25923360	25923360	G	A	G	SNP	OS06G0638000	Intronic	NA	NA	LOC_Os06g43120	zinc finger C-x8-C-x5-C-x3-H type family protein, expressed
Chr6	25927020	25927020	G	A	G	SNP	OS06G0638000	Intronic	NA	NA	LOC_Os06g43120	zinc finger C-x8-C-x5-C-x3-H type family protein, expressed
Chr6	1385337	1385337	T	A	T	SNP	OS06G0125800	Intronic	NA	NA	LOC_Os06g03580	zinc RING finger protein, putative, expressed
Chr6	27679649	27679650	AG	A	AG	INDEL	OS06G0668200	Intronic	NA	NA	LOC_Os06g45710	phosphoglycerate kinase protein, putative, expressed
Chr6	25926295	25926296	AT	A	AT	INDEL	OS06G0638000	Intronic	NA	NA	LOC_Os06g43120	zinc finger C-x8-C-x5-C-x3-H type family protein, expressed
Chr6	25834710	25834712	AAG	A	AAG	INDEL	OS06G0636100	Intronic	NA	NA	LOC_Os06g43000	nitrate-induced NOI protein, expressed
Chr6	24145195	24145197	AAT	A	AAT	INDEL	OS06G0607200	Intronic	NA	NA	LOC_Os06g40510	CRAL/TRIO domain containing protein, expressed
Chr6	23227549	23227550	AT	A	AT	INDEL	OS06G0591200	Intronic	NA	NA	LOC_Os06g39110	expressed protein
Chr6	23111846	23111847	AT	A	AT	INDEL	OS06G0589300	Intronic	NA	NA	LOC_Os06g38950	ABC transporter, ATP-binding protein, putative, expressed
Chr6	11700503	11700506	ATAG	A	ATAG	INDEL	OS06G0308300	Intronic	NA	NA	LOC_Os06g20370	microtubule associated protein, putative, expressed
Chr6	4324012	4324013	AT	A	AT	INDEL	OS06G0185900	Intronic	NA	NA	LOC_Os06g08670	glutathione peroxidase, putative, expressed
Chr6	2950399	2950400	AT	A	AT	INDEL	OS06G0158000	Intronic	NA	NA	LOC_Os06g06350	AMP-binding enzyme, putative, expressed
Chr6	2951218	2951220	AAG	A	AAG	INDEL	OS06G0158000	Intronic	NA	NA	LOC_Os06g06350	AMP-binding enzyme, putative, expressed
Chr6	1361748	1361749	AT	A	AT	INDEL	OS06G0125200	Intronic	NA	NA	LOC_Os06g03520	DUF581 domain containing protein, expressed
Chr6	2953048	2953048	T	A	T	SNP	OS06G0158000	Intronic	NA	NA	LOC_Os06g06350	AMP-binding enzyme, putative, expressed
Chr6	13407631	13407631	C	A	C	SNP	OS06G0336200	Exonic	CDS	Silent	LOC_Os06g22960	aquaporin protein, putative, expressed
Chr6	1415752	1415752	G	A	G	SNP	OS06G0126500	Exonic	CDS	Silent	LOC_Os06g03640	BAG domain containing protein, expressed
Chr6	5680571	5680571	G	A	G	SNP	OS06G0211200	Intronic	NA	NA	LOC_Os06g10880	bZIP transcription factor, putative, expressed
Chr6	5681176	5681176	G	A	G	SNP	OS06G0211200	Intronic	NA	NA	LOC_Os06g10880	bZIP transcription factor, putative, expressed
Chr6	30302453	30302453	G	A	G	SNP	OS06G0714200	Exonic	CDS	Silent	LOC_Os06g50030	CAMK_CAMK_like.30 - CAMK includes calcium/calmodulin depedent protein kinases, expressed
Chr6	9566729	9566729	G	A	G	SNP	OS06G0278000	Intronic	NA	NA	LOC_Os06g16640	carboxyl-terminal peptidase, putative, expressed
Chr6	9566878	9566878	G	A	G	SNP	OS06G0278000	Exonic	CDS	Missense	LOC_Os06g16640	carboxyl-terminal peptidase, putative, expressed
Chr6	2329548	2329548	C	A	C	SNP	OS06G0143900	Exonic	5UTR	NA	LOC_Os06g05180	coatomer subunit beta, putative, expressed
Chr6	13478216	13478216	G	A	G	SNP	OS06G0338200	Intronic	NA	NA	LOC_Os06g23114	copper methylamine oxidase precursor, putative, expressed
Chr6	13479694	13479694	T	A	T	SNP	OS06G0338200	Intronic	NA	NA	LOC_Os06g23114	copper methylamine oxidase precursor, putative, expressed
Chr6	13480818	13480818	C	A	C	SNP	OS06G0338200	Intronic	NA	NA	LOC_Os06g23114	copper methylamine oxidase precursor, putative, expressed
Chr6	13480986	13480986	G	A	G	SNP	OS06G0338200	Intronic	NA	NA	LOC_Os06g23114	copper methylamine oxidase precursor, putative, expressed
Chr6	13481329	13481329	G	A	G	SNP	OS06G0338200	Intronic	NA	NA	LOC_Os06g23114	copper methylamine oxidase precursor, putative, expressed
Chr6	13482382	13482382	G	A	G	SNP	OS06G0338200	Intronic	NA	NA	LOC_Os06g23114	copper methylamine oxidase precursor, putative, expressed
Chr6	13482398	13482398	G	A	G	SNP	OS06G0338200	Intronic	NA	NA	LOC_Os06g23114	copper methylamine oxidase precursor, putative, expressed

Chr6	30917774	30917774	G	A	G	SNP	OS06G0726800	Exonic	CDS	Silent	LOC_Os06g51110	cyclin, putative, expressed
Chr6	1361483	1361483	C	A	C	SNP	OS06G0125200	Exonic	5UTR	NA	LOC_Os06g03520	DUF581 domain containing protein, expressed
Chr6	8179711	8179711	G	A	G	SNP	OS06G0256900	Exonic	CDS	Silent	LOC_Os06g14540	endoglucanase, putative, expressed
Chr6	2473044	2473044	G	A	G	SNP	OS06G0147300	Exonic	CDS	Missense	LOC_Os06g05470	expressed protein
Chr6	2473056	2473056	T	A	T	SNP	OS06G0147300	Exonic	CDS	Missense	LOC_Os06g05470	expressed protein
Chr6	21362907	21362907	G	A	G	SNP	OS06G0558900	Exonic	5UTR	NA	LOC_Os06g36380	expressed protein
Chr6	23135915	23135915	T	A	T	SNP	OS06G0589600	Exonic	CDS	NA	LOC_Os06g38970	expressed protein
Chr6	23135940	23135940	C	A	C	SNP	OS06G0589600	Exonic	CDS	NA	LOC_Os06g38970	expressed protein
Chr6	23136000	23136000	G	A	G	SNP	OS06G0589600	Exonic	3UTR	NA	LOC_Os06g38970	expressed protein
Chr6	23228068	23228068	G	A	G	SNP	OS06G0591200	Exonic	3UTR	NA	LOC_Os06g39110	expressed protein
Chr6	24373657	24373657	G	A	G	SNP	OS06G0611300	Exonic	CDS	Missense	LOC_Os06g40870	expressed protein
Chr6	26385411	26385411	T	A	T	SNP	OS06G0646100	Exonic	3UTR	NA	LOC_Os06g43810	expressed protein
Chr6	4322326	4322326	T	A	T	SNP	OS06G0185900	Intronic	NA	NA	LOC_Os06g08670	glutathione peroxidase, putative, expressed
Chr6	4322663	4322663	G	A	G	SNP	OS06G0185900	Intronic	NA	NA	LOC_Os06g08670	glutathione peroxidase, putative, expressed
Chr6	14647368	14647368	C	A	C	SNP	OS06G0356800	Exonic	CDS	Missense	LOC_Os06g25010	glycosyl hydrolase, putative, expressed
Chr6	14647452	14647452	C	A	C	SNP	OS06G0356800	Exonic	CDS	Missense	LOC_Os06g25010	glycosyl hydrolase, putative, expressed
Chr6	14647970	14647970	G	A	G	SNP	OS06G0356800	Exonic	CDS	Missense	LOC_Os06g25010	glycosyl hydrolase, putative, expressed
Chr6	26647726	26647726	G	A	G	SNP	OS06G0650900	Exonic	5UTR	NA	LOC_Os06g44160	heat shock protein DnaJ, putative, expressed
Chr6	20404832	20404832	G	A	G	SNP	OS06G0542300	Exonic	CDS	Missense	LOC_Os06g35060	heavy metal-associated domain containing protein, expressed
Chr6	20404859	20404859	G	A	G	SNP	OS06G0542300	Exonic	CDS	Missense	LOC_Os06g35060	heavy metal-associated domain containing protein, expressed
Chr6	23857643	23857643	C	A	C	SNP	OS06G0603000	Exonic	CDS	Missense	LOC_Os06g40080	heme oxygenase 1, putative, expressed
Chr6	3040829	3040829	G	A	G	SNP	OS06G0159450	Exonic	CDS	NA	LOC_Os06g06460	histone H3, putative, expressed
Chr6	3040950	3040950	G	A	G	SNP	OS06G0159450	Exonic	CDS	NA	LOC_Os06g06460	histone H3, putative, expressed
Chr6	3041117	3041117	G	A	G	SNP	OS06G0159450	Exonic	CDS	NA	LOC_Os06g06460	histone H3, putative, expressed
Chr6	3041191	3041191	C	A	C	SNP	OS06G0159450	Exonic	CDS	NA	LOC_Os06g06460	histone H3, putative, expressed
Chr6	30993383	30993383	C	A	C	SNP	OS06G0728000	Exonic	5UTR	NA	LOC_Os06g51220	HMG1/2, putative, expressed
Chr6	4655042	4655042	C	A	C	SNP	OS06G0192400	Exonic	CDS	Missense	LOC_Os06g09270	hypro1, putative, expressed
Chr6	4332743	4332743	C	A	C	SNP	OS06G0186100	Exonic	CDS	Silent	LOC_Os06g08690	leucine-rich repeat receptor protein kinase EXS precursor, putative, expressed
Chr6	4333075	4333075	C	A	C	SNP	OS06G0186100	Exonic	CDS	Missense	LOC_Os06g08690	leucine-rich repeat receptor protein kinase EXS precursor, putative, expressed
Chr6	26227588	26227588	G	A	G	SNP	OS06G0643500	Exonic	CDS	Silent	LOC_Os06g43600	LTPL129 - Protease inhibitor/seed storage/LTP family protein precursor, expressed
Chr6	23471311	23471311	C	A	C	SNP	OS06G0596300	Exonic	3UTR	NA	LOC_Os06g39520	myristoyl-acyl carrier protein thioesterase, chloroplast precursor, putative, expressed
Chr6	25835752	25835752	G	A	G	SNP	OS06G0636100	Intronic	NA	NA	LOC_Os06g43000	nitrate-induced NOI protein, expressed
Chr6	25835758	25835758	G	A	G	SNP	OS06G0636100	Intronic	NA	NA	LOC_Os06g43000	nitrate-induced NOI protein, expressed
Chr6	31143868	31143868	G	A	G	SNP	OS06G0730600	Intronic	NA	NA	LOC_Os06g51410	OsPOP15 - Putative Prolyl Oligopeptidase homologue, expressed
Chr6	26676703	26676703	T	A	T	SNP	OS06G0651900	Exonic	5UTR	NA	LOC_Os06g44220	OsRCI2-9 - Putative low temperature and salt responsive protein, expressed
Chr6	20676821	20676821	G	A	G	SNP	OS06G0546500	Exonic	CDS	Missense	LOC_Os06g35480	peroxidase precursor, putative, expressed
Chr6	25214623	25214623	G	A	G	SNP	OS06G0625500	Exonic	CDS	Missense	LOC_Os06g42000	peroxiredoxin, putative, expressed
Chr6	17067552	17067552	G	A	G	SNP	OS06G0493100	Intronic	NA	NA	LOC_Os06g29730	RALFL28 - Rapid ALkalinization Factor RALF family protein precursor, expressed
Chr6	22865360	22865360	G	A	G	SNP	OS06G0585950	Exonic	CDS	Silent	LOC_Os06g38590	receptor-like protein kinase precursor, putative, expressed
Chr6	9283464	9283464	C	A	C	SNP	OS06G0274200	Intronic	NA	NA	LOC_Os06g16290	ribosomal protein L7Ae, putative, expressed
Chr6	9283653	9283653	T	A	T	SNP	OS06G0274200	Intronic	NA	NA	LOC_Os06g16290	ribosomal protein L7Ae, putative, expressed
Chr6	9284069	9284069	C	A	C	SNP	OS06G0274200	Intronic	NA	NA	LOC_Os06g16290	ribosomal protein L7Ae, putative, expressed
Chr6	9284146	9284146	G	A	G	SNP	OS06G0274200	Intronic	NA	NA	LOC_Os06g16290	ribosomal protein L7Ae, putative, expressed
Chr6	9284158	9284158	T	A	T	SNP	OS06G0274200	Intronic	NA	NA	LOC_Os06g16290	ribosomal protein L7Ae, putative, expressed
Chr6	9284252	9284252	G	A	G	SNP	OS06G0274200	Intronic	NA	NA	LOC_Os06g16290	ribosomal protein L7Ae, putative, expressed
Chr6	9284489	9284489	T	A	T	SNP	OS06G0274200	Intronic	NA	NA	LOC_Os06g16290	ribosomal protein L7Ae, putative, expressed
Chr6	9284490	9284490	G	A	G	SNP	OS06G0274200	Intronic	NA	NA	LOC_Os06g16290	ribosomal protein L7Ae, putative, expressed
Chr6	9284587	9284587	G	A	G	SNP	OS06G0274200	Intronic	NA	NA	LOC_Os06g16290	ribosomal protein L7Ae, putative, expressed
Chr6	9284727	9284727	T	A	T	SNP	OS06G0274200	Intronic	NA	NA	LOC_Os06g16290	ribosomal protein L7Ae, putative, expressed
Chr6	9284806	9284806	G	A	G	SNP	OS06G0274200	Intronic	NA	NA	LOC_Os06g16290	ribosomal protein L7Ae, putative, expressed
Chr6	27384583	27384583	C	A	C	SNP	OS06G0663400	Intronic	NA	NA	LOC_Os06g45300	serine/threonine-protein kinase HT1, putative, expressed
Chr6	19618474	19618474	G	A	G	SNP	OS06G0528600	Intronic	NA	NA	LOC_Os06g33710	spermidine synthase, putative, expressed
Chr6	20012010	20012010	G	A	G	SNP	OS06G0534800	Exonic	3UTR	NA	LOC_Os06g34390	zinc finger, C3HC4 type domain containing protein, expressed
Chr6	31130374	31130377	AAGG	A	AAGG	INDEL	OS06G0730300	Exonic	CDS	InFrame	LOC_Os06g51390	expressed protein
Chr6	30301827	30301828	AC	A	AC	INDEL	OS06G0714200	Exonic	5UTR	NA	LOC_Os06g50030	CAMK_CAMK_like.30 - CAMK includes calcium/calmodulin depedent protein kinases, expressed
Chr6	26646954	26646955	AT	A	AT	INDEL	OS06G0650900	Exonic	5UTR	NA	LOC_Os06g44160	heat shock protein DnaJ, putative, expressed
Chr6	26228716	26228718	AAT	A	AAT	INDEL	OS06G0643500	Exonic	3UTR	NA	LOC_Os06g43600	LTPL129 - Protease inhibitor/seed storage/LTP family protein precursor, expressed
Chr6	25835932	25835933	AT	A	AT	INDEL	OS06G0636100	Intronic	NA	NA	LOC_Os06g43000	nitrate-induced NOI protein, expressed
Chr6	24036530	24036546	AGTGTGTGTGTGTGTGT	A	AGTGTGTGTGTGTGTGT	INDEL	OS06G0605900	Exonic	3UTR	NA	LOC_Os06g40360	OsFBL30 - F-box domain and LRR containing protein, expressed
Chr6	23135970	23135971	AC	A	AC	INDEL	OS06G0589600	Exonic	3UTR	NA	LOC_Os06g38970	expressed protein
Chr6	23135979	23135981	AGG	A	AGG	INDEL	OS06G0589600	Exonic	3UTR	NA	LOC_Os06g38970	expressed protein
Chr6	23108589	23108598	ATATAAGAAC	A	ATATAAGAAC	INDEL	OS06G0589300	Intronic	NA	NA	LOC_Os06g38950	ABC transporter, ATP-binding protein, putative, expressed
Chr6	17067667	17067668	AT	A	AT	INDEL	OS06G0493100	Intronic	NA	NA	LOC_Os06g29730	RALFL28 - Rapid ALkalinization Factor RALF family protein precursor, expressed
Chr6	13478172	13478173	AC	A	AC	INDEL	OS06G0338200	Intronic	NA	NA	LOC_Os06g23114	copper methylamine oxidase precursor, putative, expressed
Chr6	9284809	9284813	AACTT	A	AACTT	INDEL	OS06G0274200	Intronic	NA	NA	LOC_Os06g16290	ribosomal protein L7Ae, putative, expressed
Chr6	8765193	8765197	ACCCC	A	ACCCC	INDEL	OS06G0265000	Intronic	NA	NA	LOC_Os06g15420	asparagine synthetase, putative, expressed
Chr6	3447979	3447982	AAAT	A	AAAT	INDEL	OS06G0168500	Intronic	NA	NA	LOC_Os06g07200	syntaxin, putative, expressed
Chr6	3040957	3040960	AGAG	A	AGAG	INDEL	OS06G0159450	Exonic	CDS	NA	LOC_Os06g06460	histone H3, putative, expressed
Chr6	2328519	2328521	AAT	A	AAT	INDEL	OS06G0143900	Intronic	NA	NA	LOC_Os06g05180	coatomer subunit beta, putative, expressed
Chr7	21590746	21590748	AAG	A	AAG	INDEL	OS07G0545300	Exonic	3UTR	NA	LOC_Os07g36130	core histone H2A/H2B/H3/H4, putative, expressed
Chr7	21590180	21590182	ATT	A	ATT	INDEL	OS07G0545300	Intronic	NA	NA	LOC_Os07g36130	core histone H2A/H2B/H3/H4, putative, expressed
Chr7	21245224	21245252	ATGGCATTTAGTTTCAAAACAAAACATAC	A	ATGGCATTTAGTTTCAAAACAAAACATAC	INDEL	OS07G0539400	Intronic	NA	NA	LOC_Os07g35520	glucan endo-1,3-beta-glucosidase precursor, putative, expressed
Chr7	19656258	19656261	AGAG	A	AGAG	INDEL	OS07G0513000	Exonic	3UTR	NA	LOC_Os07g32880	ATP synthase gamma chain, putative, expressed
Chr7	19656092	19656097	ATACTC	A	ATACTC	INDEL	OS07G0513000	Exonic	3UTR	NA	LOC_Os07g32880	ATP synthase gamma chain, putative, expressed
Chr7	3607198	3607201	ATGC	A	ATGC	INDEL	OS07G0167100	Intronic	NA	NA	LOC_Os07g07260	glutamine-dependent NAD, putative, expressed
Chr7	559614	559624	ACAACAAGTCC	A	ACAACAAGTCC	INDEL	OS07G0110200	Exonic	3UTR	NA	LOC_Os07g01920	nucleolar GTP-binding protein 1, putative, expressed
Chr7	21850285	21850288	ACTC	A	ACTC	INDEL	OS07G0550500	Exonic	CDS	InFrame	LOC_Os07g36544	serine/threonine-protein kinase receptor precursor, putative, expressed
Chr7	27903986	27903987	AC	A	AC	INDEL	OS07G0661500	Exonic	3UTR	NA	LOC_Os07g46690	PHD-finger family protein, expressed
Chr7	26484750	26484752	ACC	A	ACC	INDEL	OS07G0637300	Intronic	NA	NA	LOC_Os07g44330	kinase, putative, expressed
Chr7	24989954	24989965	ATATAGTATTTT	A	ATATAGTATTTT	INDEL	OS07G0607800	Intronic	NA	NA	LOC_Os07g41694	acidic leucine-rich nuclear phosphoprotein 32-related protein 1, putative, expressed
Chr7	24658223	24658234	AACTACCACGGT	A	AACTACCACGGT	INDEL	OS07G0603100	Intronic	NA	NA	LOC_Os07g41180	RNA-binding protein-like, putative, expressed
Chr7	22370294	22370295	AT	A	AT	INDEL	OS07G0560300	Exonic	3UTR	NA	LOC_Os07g37350	armadillo/beta-catenin repeat family protein, putative, expressed
Chr7	22185299	22185326	AAACACCAATTGTTCAATGTTCAGTCTT	A	AAACACCAATTGTTCAATGTTCAGTCTT	INDEL	OS07G0556200	Intronic	NA	NA	LOC_Os07g37030	cytochrome b6-f complex iron-sulfur subunit, chloroplast precursor, putative, expressed
Chr7	21846748	21846751	ACCC	A	ACCC	INDEL	OS07G0550500	Exonic	3UTR	NA	LOC_Os07g36544	serine/threonine-protein kinase receptor precursor, putative, expressed
Chr7	16896898	16896899	AC	A	AC	INDEL	OS07G0471300	Intronic	NA	NA	LOC_Os07g28850	retrotransposon protein, putative, unclassified, expressed

Chr7	15602608	15602622	AGATATTACTAATAG	A	AGATATTACTAATAG	INDEL	OS07G0452500	Intronic	NA	NA	LOC_Os07g26940	ORM1, putative, expressed
Chr7	15602994	15602995	AC	A	AC	INDEL	OS07G0452500	Intronic	NA	NA	LOC_Os07g26940	ORM1, putative, expressed
Chr7	1566636	1566643	ATACTCCG	A	ATACTCCG	INDEL	OS07G0129700	Intronic	NA	NA	LOC_Os07g03770	Homeobox domain containing protein, expressed
Chr7	351187	351188	AT	A	AT	INDEL	OS07G0106200	Intronic	NA	NA	LOC_Os07g01560	transporter family protein, putative, expressed
Chr7	351592	351597	ATTGAT	A	ATTGAT	INDEL	OS07G0106200	Intronic	NA	NA	LOC_Os07g01560	transporter family protein, putative, expressed
Chr7	352897	352899	AGT	A	AGT	INDEL	OS07G0106200	Intronic	NA	NA	LOC_Os07g01560	transporter family protein, putative, expressed
Chr7	353312	353319	ATTTTGTT	A	ATTTTGTT	INDEL	OS07G0106200	Intronic	NA	NA	LOC_Os07g01560	transporter family protein, putative, expressed
Chr8	20462090	20462090	G	A	G	SNP	OS08G0425800	Exonic	CDS	NA	LOC_Os08g32980	expressed protein
Chr8	21054844	21054844	G	A	G	SNP	OS08G0434300	Exonic	3UTR	NA	LOC_Os08g33720	lactate/malate dehydrogenase, putative, expressed
Chr8	25421571	25421571	C	A	C	SNP	OS08G0512600	Exonic	3UTR	NA	LOC_Os08g40170	cyclin-dependent kinase B2-1, putative, expressed
Chr8	21013073	21013073	G	A	G	SNP	OS08G0433200	Exonic	CDS	Missense	LOC_Os08g33640	expressed protein
Chr8	21055696	21055696	G	A	G	SNP	OS08G0434300	Exonic	CDS	NA	LOC_Os08g33720	lactate/malate dehydrogenase, putative, expressed
Chr8	20249410	20249410	G	A	G	SNP	OS08G0422600	Exonic	CDS	NA	LOC_Os08g32690	expressed protein
Chr8	19206760	19206763	ATCG	A	ATCG	INDEL	OS08G0402500	Exonic	5UTR	NA	LOC_Os08g31090	expressed protein
Chr8	5234142	5234142	T	A	T	SNP	OS08G0189400	Intronic	NA	NA	LOC_Os08g08990	Cupin domain containing protein, expressed
Chr8	5983288	5983288	T	A	T	SNP	OS08G0203100	Intronic	NA	NA	LOC_Os08g10290	SHR5-receptor-like kinase, putative, expressed
Chr8	5984381	5984381	T	A	T	SNP	OS08G0203100	Exonic	3UTR	NA	LOC_Os08g10290	SHR5-receptor-like kinase, putative, expressed
Chr8	5984423	5984423	T	A	T	SNP	OS08G0203100	Exonic	3UTR	NA	LOC_Os08g10290	SHR5-receptor-like kinase, putative, expressed
Chr8	5985860	5985860	T	A	T	SNP	OS08G0203100	Exonic	3UTR	NA	LOC_Os08g10290	SHR5-receptor-like kinase, putative, expressed
Chr8	5985951	5985951	T	A	T	SNP	OS08G0203100	Exonic	3UTR	NA	LOC_Os08g10290	SHR5-receptor-like kinase, putative, expressed
Chr8	5985957	5985957	T	A	T	SNP	OS08G0203100	Exonic	3UTR	NA	LOC_Os08g10290	SHR5-receptor-like kinase, putative, expressed
Chr8	6207519	6207519	T	A	T	SNP	OS08G0206400	Intronic	NA	NA	LOC_Os08g10550	potassium transporter, putative, expressed
Chr8	6207605	6207605	T	A	T	SNP	OS08G0206400	Intronic	NA	NA	LOC_Os08g10550	potassium transporter, putative, expressed
Chr8	6208231	6208231	T	A	T	SNP	OS08G0206400	Intronic	NA	NA	LOC_Os08g10550	potassium transporter, putative, expressed
Chr8	7942897	7942897	T	A	T	SNP	OS08G0230200	Intronic	NA	NA	LOC_Os08g13350	expressed protein
Chr8	14416492	14416492	T	A	T	SNP	OS08G0327400	Intronic	NA	NA	LOC_Os08g23810	enoyl-acyl-carrier-protein reductase NADH, chloroplast precursor, expressed
Chr8	17573829	17573829	T	A	T	SNP	OS08G0374800	Intronic	NA	NA	LOC_Os08g28730	NAD dependent epimerase/dehydratase family protein, putative, expressed
Chr8	3294132	3294132	T	A	T	SNP	OS08G0156800	Intronic	NA	NA	LOC_Os08g06040	60S ribosomal protein L34, putative, expressed
Chr8	686991	686991	G	A	G	SNP	OS08G0112900	Intronic	NA	NA	LOC_Os08g02094	GDSL-like lipase/acylhydrolase, putative, expressed
Chr8	687060	687060	G	A	G	SNP	OS08G0112900	Intronic	NA	NA	LOC_Os08g02094	GDSL-like lipase/acylhydrolase, putative, expressed
Chr8	687074	687074	G	A	G	SNP	OS08G0112900	Intronic	NA	NA	LOC_Os08g02094	GDSL-like lipase/acylhydrolase, putative, expressed
Chr8	687164	687164	G	A	G	SNP	OS08G0112900	Intronic	NA	NA	LOC_Os08g02094	GDSL-like lipase/acylhydrolase, putative, expressed
Chr8	688216	688216	G	A	G	SNP	OS08G0112900	Intronic	NA	NA	LOC_Os08g02094	GDSL-like lipase/acylhydrolase, putative, expressed
Chr8	5141316	5141316	G	A	G	SNP	OS08G0187800	Exonic	CDS	Silent	LOC_Os08g08840	phosphate/phosphate translocator, putative, expressed
Chr8	5982922	5982922	G	A	G	SNP	OS08G0203100	Intronic	NA	NA	LOC_Os08g10290	SHR5-receptor-like kinase, putative, expressed
Chr8	5983348	5983348	G	A	G	SNP	OS08G0203100	Exonic	CDS	NA	LOC_Os08g10290	SHR5-receptor-like kinase, putative, expressed
Chr8	5983379	5983379	G	A	G	SNP	OS08G0203100	Intronic	NA	NA	LOC_Os08g10290	SHR5-receptor-like kinase, putative, expressed
Chr8	5983415	5983415	G	A	G	SNP	OS08G0203100	Intronic	NA	NA	LOC_Os08g10290	SHR5-receptor-like kinase, putative, expressed
Chr8	5983663	5983663	G	A	G	SNP	OS08G0203100	Intronic	NA	NA	LOC_Os08g10290	SHR5-receptor-like kinase, putative, expressed
Chr8	5984740	5984740	G	A	G	SNP	OS08G0203100	Exonic	3UTR	NA	LOC_Os08g10290	SHR5-receptor-like kinase, putative, expressed
Chr8	5985097	5985097	G	A	G	SNP	OS08G0203100	Exonic	3UTR	NA	LOC_Os08g10290	SHR5-receptor-like kinase, putative, expressed
Chr8	5985693	5985693	G	A	G	SNP	OS08G0203100	Intronic	NA	NA	LOC_Os08g10290	SHR5-receptor-like kinase, putative, expressed
Chr8	5985984	5985984	G	A	G	SNP	OS08G0203100	Exonic	3UTR	NA	LOC_Os08g10290	SHR5-receptor-like kinase, putative, expressed
Chr8	6206839	6206839	G	A	G	SNP	OS08G0206400	Intronic	NA	NA	LOC_Os08g10550	potassium transporter, putative, expressed
Chr8	6207190	6207190	G	A	G	SNP	OS08G0206400	Intronic	NA	NA	LOC_Os08g10550	potassium transporter, putative, expressed
Chr8	6207617	6207617	G	A	G	SNP	OS08G0206400	Intronic	NA	NA	LOC_Os08g10550	potassium transporter, putative, expressed
Chr8	6207650	6207650	G	A	G	SNP	OS08G0206400	Intronic	NA	NA	LOC_Os08g10550	potassium transporter, putative, expressed
Chr8	7365786	7365786	G	A	G	SNP	OS08G0221300	Intronic	NA	NA	LOC_Os08g12449	expressed protein
Chr8	8761034	8761034	G	A	G	SNP	OS08G0243500	Intronic	NA	NA	LOC_Os08g14570	NADPH reductase, putative, expressed
Chr8	9507285	9507285	G	A	G	SNP	OS08G0256000	Exonic	3UTR	NA	LOC_Os08g15650	expressed protein
Chr8	9507372	9507372	G	A	G	SNP	OS08G0256000	Exonic	3UTR	NA	LOC_Os08g15650	expressed protein
Chr8	11608771	11608771	G	A	G	SNP	OS08G0290700	Exonic	3UTR	NA	LOC_Os08g19420	O-methyltransferase, putative, expressed
Chr8	12268248	12268248	G	A	G	SNP	OS08G0299400	Intronic	NA	NA	LOC_Os08g20420	MGD2, putative, expressed
Chr8	13956808	13956808	G	A	G	SNP	OS08G0320400	Exonic	3UTR	NA	LOC_Os08g23150	indole-3-glycerol phosphate synthase, chloroplast precursor, putative, expressed
Chr8	17511143	17511143	G	A	G	SNP	OS08G0374000	Exonic	CDS	Silent	LOC_Os08g28670	pathogenesis-related Bet v I family protein, putative, expressed
Chr8	17511197	17511197	G	A	G	SNP	OS08G0374000	Exonic	CDS	Silent	LOC_Os08g28670	pathogenesis-related Bet v I family protein, putative, expressed
Chr8	17511476	17511476	G	A	G	SNP	OS08G0374000	Exonic	CDS	Missense	LOC_Os08g28670	pathogenesis-related Bet v I family protein, putative, expressed
Chr8	20424349	20424349	G	A	G	SNP	OS08G0425200	Intronic	NA	NA	LOC_Os08g32930	expressed protein
Chr8	7364473	7364473	G	A	G	SNP	OS08G0221300	Intronic	NA	NA	LOC_Os08g12449	expressed protein
Chr8	7365419	7365419	G	A	G	SNP	OS08G0221300	Exonic	3UTR	NA	LOC_Os08g12449	expressed protein
Chr8	20252512	20252512	G	A	G	SNP	OS08G0422600	Intronic	NA	NA	LOC_Os08g32690	expressed protein
Chr8	26728519	26728519	G	A	G	SNP	OS08G0535200	Intronic	NA	NA	LOC_Os08g42350	nodulin MtN3 family protein, putative, expressed
Chr8	581740	581740	C	A	C	SNP	OS08G0111200	Intronic	NA	NA	LOC_Os08g01940	non-lysosomal glucosylceramidase, putative, expressed
Chr8	5365702	5365702	C	A	C	SNP	OS08G0191600	Intronic	NA	NA	LOC_Os08g09240	autophagy-related protein, putative, expressed
Chr8	5983367	5983367	C	A	C	SNP	OS08G0203100	Exonic	CDS	NA	LOC_Os08g10290	SHR5-receptor-like kinase, putative, expressed
Chr8	5983500	5983500	C	A	C	SNP	OS08G0203100	Exonic	CDS	NA	LOC_Os08g10290	SHR5-receptor-like kinase, putative, expressed
Chr8	5984407	5984407	C	A	C	SNP	OS08G0203100	Exonic	3UTR	NA	LOC_Os08g10290	SHR5-receptor-like kinase, putative, expressed
Chr8	6206064	6206064	C	A	C	SNP	OS08G0206400	Exonic	5UTR	NA	LOC_Os08g10550	potassium transporter, putative, expressed
Chr8	8761793	8761793	C	A	C	SNP	OS08G0243500	Intronic	NA	NA	LOC_Os08g14570	NADPH reductase, putative, expressed
Chr8	10812286	10812286	C	A	C	SNP	OS08G0278900	Intronic	NA	NA	LOC_Os08g17680	stromal cell-derived factor 2-like protein precursor, putative, expressed
Chr8	16065388	16065388	C	A	C	SNP	OS08G0352100	Intronic	NA	NA	LOC_Os08g26350	expressed protein
Chr8	17511290	17511290	C	A	C	SNP	OS08G0374000	Intronic	NA	NA	LOC_Os08g28670	pathogenesis-related Bet v I family protein, putative, expressed
Chr8	20427314	20427314	C	A	C	SNP	OS08G0425200	Intronic	NA	NA	LOC_Os08g32930	expressed protein
Chr8	2979250	2979250	T	A	T	SNP	OS08G0151800	Exonic	CDS	Silent	LOC_Os08g05570	monodehydroascorbate reductase, putative, expressed
Chr8	3420573	3420573	T	A	T	SNP	OS08G0158500	Exonic	5UTR	NA	LOC_Os08g06200	stomatin-like protein 2, putative, expressed
Chr8	9384801	9384801	T	A	T	SNP	OS08G0253800	Intronic	NA	NA	LOC_Os08g15420	CSLC3 - cellulose synthase-like family C, expressed
Chr8	17570911	17570911	T	A	T	SNP	OS08G0374800	Intronic	NA	NA	LOC_Os08g28730	NAD dependent epimerase/dehydratase family protein, putative, expressed
Chr8	19031845	19031845	T	A	T	SNP	OS08G0398800	Exonic	CDS	Missense	LOC_Os08g30820	cleavage and polyadenylation specificity factor subunit 5, putative, expressed
Chr8	20425783	20425783	T	A	T	SNP	OS08G0425200	Intronic	NA	NA	LOC_Os08g32930	expressed protein
Chr8	20426844	20426844	T	A	T	SNP	OS08G0425200	Intronic	NA	NA	LOC_Os08g32930	expressed protein
Chr8	21011327	21011327	T	A	T	SNP	OS08G0433200	Intronic	NA	NA	LOC_Os08g33640	expressed protein
Chr8	24275019	24275019	T	A	T	SNP	OS08G0491200	Exonic	CDS	Missense	LOC_Os08g38320	AGC_PVPK_like_kin82y.14 - ACG kinases include homologs to PKA, PKG and PKC, expressed
Chr8	24854352	24854352	T	A	T	SNP	OS08G0503000	Exonic	5UTR	NA	LOC_Os08g39330	skin secretory protein xP2 precursor, putative, expressed
Chr8	25245297	25245297	T	A	T	SNP	OS08G0509100	Intronic	NA	NA	LOC_Os08g39850	lipoxygenase, chloroplast precursor, putative, expressed
Chr8	25247592	25247592	T	A	T	SNP	OS08G0509100	Intronic	NA	NA	LOC_Os08g39850	lipoxygenase, chloroplast precursor, putative, expressed

Chr8	688324	688324	G	A	G	SNP	OS08G0112900	Intronic	NA	NA	LOC_Os08g02094	GDSL-like lipase/acylhydrolase, putative, expressed
Chr8	1566957	1566957	G	A	G	SNP	OS08G0127100	Exonic	3UTR	NA	LOC_Os08g03350	amino acid transporter, putative, expressed
Chr8	1836346	1836346	G	A	G	SNP	OS08G0132600	Exonic	3UTR	NA	LOC_Os08g03840	expressed protein
Chr8	2174805	2174805	G	A	G	SNP	OS08G0139000	Exonic	3UTR	NA	LOC_Os08g04440	RNA recognition motif containing protein, putative, expressed
Chr8	2175883	2175883	G	A	G	SNP	OS08G0139000	Exonic	CDS	Missense	LOC_Os08g04440	RNA recognition motif containing protein, putative, expressed
Chr8	2513335	2513335	G	A	G	SNP	OS08G0144400	Exonic	3UTR	NA	LOC_Os08g04920	OsDegp11 - Putative Deg protease homologue, expressed
Chr8	2979678	2979678	G	A	G	SNP	OS08G0151800	Exonic	CDS	Missense	LOC_Os08g05570	monodehydroascorbate reductase, putative, expressed
Chr8	2980775	2980775	G	A	G	SNP	OS08G0151800	Intronic	NA	NA	LOC_Os08g05570	monodehydroascorbate reductase, putative, expressed
Chr8	2981737	2981737	G	A	G	SNP	OS08G0151800	Intronic	NA	NA	LOC_Os08g05570	monodehydroascorbate reductase, putative, expressed
Chr8	3281916	3281916	G	A	G	SNP	OS08G0156600	Intronic	NA	NA	LOC_Os08g06010	transporter, major facilitator family, putative, expressed
Chr8	3293800	3293800	G	A	G	SNP	OS08G0156800	Intronic	NA	NA	LOC_Os08g06040	60S ribosomal protein L34, putative, expressed
Chr8	3294787	3294787	G	A	G	SNP	OS08G0156800	Intronic	NA	NA	LOC_Os08g06040	60S ribosomal protein L34, putative, expressed
Chr8	3337862	3337862	G	A	G	SNP	OS08G0157500	Exonic	3UTR	NA	LOC_Os08g06100	O-methyltransferase, putative, expressed
Chr8	3340027	3340027	G	A	G	SNP	OS08G0157500	Intronic	NA	NA	LOC_Os08g06100	O-methyltransferase, putative, expressed
Chr8	3366874	3366874	G	A	G	SNP	OS08G0157600	Intronic	NA	NA	LOC_Os08g06110	MYB family transcription factor, putative, expressed
Chr8	3369645	3369645	G	A	G	SNP	OS08G0157600	Intronic	NA	NA	LOC_Os08g06110	MYB family transcription factor, putative, expressed
Chr8	3369786	3369786	G	A	G	SNP	OS08G0157600	Intronic	NA	NA	LOC_Os08g06110	MYB family transcription factor, putative, expressed
Chr8	3390090	3390090	G	A	G	SNP	OS08G0157900	Intronic	NA	NA	LOC_Os08g06140	no apical meristem protein, putative, expressed
Chr8	3391010	3391010	G	A	G	SNP	OS08G0157900	Intronic	NA	NA	LOC_Os08g06140	no apical meristem protein, putative, expressed
Chr8	3461415	3461415	G	A	G	SNP	OS08G0159500	Exonic	3UTR	NA	LOC_Os08g06280	LSD1 zinc finger domain containing protein, expressed
Chr8	3548796	3548796	G	A	G	SNP	OS08G0160500	Intronic	NA	NA	LOC_Os08g06380	CSLF6 - cellulose synthase-like family F; beta1,3;1,4 glucan synthase, expressed
Chr8	4948294	4948294	G	A	G	SNP	OS08G0184300	Intronic	NA	NA	LOC_Os08g08570	expressed protein
Chr8	5142526	5142526	G	A	G	SNP	OS08G0187800	Intronic	NA	NA	LOC_Os08g08840	phosphate/phosphate translocator, putative, expressed
Chr8	5242851	5242851	G	A	G	SNP	OS08G0189600	Intronic	NA	NA	LOC_Os08g09010	Cupin domain containing protein, expressed
Chr8	7365619	7365619	G	A	G	SNP	OS08G0221300	Intronic	NA	NA	LOC_Os08g12449	expressed protein
Chr8	7365924	7365924	G	A	G	SNP	OS08G0221300	Intronic	NA	NA	LOC_Os08g12449	expressed protein
Chr8	9384267	9384267	G	A	G	SNP	OS08G0253800	Intronic	NA	NA	LOC_Os08g15420	CSLC3 - cellulose synthase-like family C, expressed
Chr8	9384775	9384775	G	A	G	SNP	OS08G0253800	Intronic	NA	NA	LOC_Os08g15420	CSLC3 - cellulose synthase-like family C, expressed
Chr8	17572908	17572908	G	A	G	SNP	OS08G0374800	Intronic	NA	NA	LOC_Os08g28730	NAD dependent epimerase/dehydratase family protein, putative, expressed
Chr8	17575784	17575784	G	A	G	SNP	OS08G0374800	Intronic	NA	NA	LOC_Os08g28730	NAD dependent epimerase/dehydratase family protein, putative, expressed
Chr8	20425381	20425381	G	A	G	SNP	OS08G0425200	Intronic	NA	NA	LOC_Os08g32930	expressed protein
Chr8	20426978	20426978	G	A	G	SNP	OS08G0425200	Intronic	NA	NA	LOC_Os08g32930	expressed protein
Chr8	20462469	20462469	G	A	G	SNP	OS08G0425800	Exonic	3UTR	NA	LOC_Os08g32980	expressed protein
Chr8	20463441	20463441	G	A	G	SNP	OS08G0425800	Exonic	3UTR	NA	LOC_Os08g32980	expressed protein
Chr8	20463638	20463638	G	A	G	SNP	OS08G0425800	Exonic	3UTR	NA	LOC_Os08g32980	expressed protein
Chr8	21052666	21052666	G	A	G	SNP	OS08G0434100	Exonic	3UTR	NA	LOC_Os08g33710	ribonuclease T2 family domain containing protein, expressed
Chr8	21054967	21054967	G	A	G	SNP	OS08G0434300	Exonic	CDS	NA	LOC_Os08g33720	lactate/malate dehydrogenase, putative, expressed
Chr8	21055395	21055395	G	A	G	SNP	OS08G0434300	Exonic	CDS	NA	LOC_Os08g33720	lactate/malate dehydrogenase, putative, expressed
Chr8	24261434	24261434	G	A	G	SNP	OS08G0490900	Exonic	CDS	Silent	LOC_Os08g38300	Core histone H2A/H2B/H3/H4 domain containing protein, putative, expressed
Chr8	24274775	24274775	G	A	G	SNP	OS08G0491200	Intronic	NA	NA	LOC_Os08g38320	AGC_PVPK_like_kin82y.14 - ACG kinases include homologs to PKA, PKG and PKC, expressed
Chr8	24854349	24854349	G	A	G	SNP	OS08G0503000	Exonic	5UTR	NA	LOC_Os08g39330	skin secretory protein xP2 precursor, putative, expressed
Chr8	25242162	25242162	G	A	G	SNP	OS08G0509100	Intronic	NA	NA	LOC_Os08g39850	lipoxygenase, chloroplast precursor, putative, expressed
Chr8	25243380	25243380	G	A	G	SNP	OS08G0509100	Intronic	NA	NA	LOC_Os08g39850	lipoxygenase, chloroplast precursor, putative, expressed
Chr8	25244036	25244036	G	A	G	SNP	OS08G0509100	Intronic	NA	NA	LOC_Os08g39850	lipoxygenase, chloroplast precursor, putative, expressed
Chr8	25244243	25244243	G	A	G	SNP	OS08G0509100	Intronic	NA	NA	LOC_Os08g39850	lipoxygenase, chloroplast precursor, putative, expressed
Chr8	25245186	25245186	G	A	G	SNP	OS08G0509100	Intronic	NA	NA	LOC_Os08g39850	lipoxygenase, chloroplast precursor, putative, expressed
Chr8	25245202	25245202	G	A	G	SNP	OS08G0509100	Intronic	NA	NA	LOC_Os08g39850	lipoxygenase, chloroplast precursor, putative, expressed
Chr8	25245287	25245287	G	A	G	SNP	OS08G0509100	Intronic	NA	NA	LOC_Os08g39850	lipoxygenase, chloroplast precursor, putative, expressed
Chr8	25245318	25245318	G	A	G	SNP	OS08G0509100	Intronic	NA	NA	LOC_Os08g39850	lipoxygenase, chloroplast precursor, putative, expressed
Chr8	25246249	25246249	G	A	G	SNP	OS08G0509100	Intronic	NA	NA	LOC_Os08g39850	lipoxygenase, chloroplast precursor, putative, expressed
Chr8	25246515	25246515	G	A	G	SNP	OS08G0509100	Intronic	NA	NA	LOC_Os08g39850	lipoxygenase, chloroplast precursor, putative, expressed
Chr8	25249948	25249948	G	A	G	SNP	OS08G0509100	Exonic	3UTR	NA	LOC_Os08g39850	lipoxygenase, chloroplast precursor, putative, expressed
Chr8	25416900	25416900	G	A	G	SNP	OS08G0512500	Intronic	NA	NA	LOC_Os08g40160	thylakoid lumen protein, chloroplast precursor, putative, expressed
Chr8	25769889	25769889	G	A	G	SNP	OS08G0519100	Exonic	3UTR	NA	LOC_Os08g40720	FAD-binding and arabino-lactone oxidase domains containing protein, putative, expressed
Chr8	27128415	27128415	G	A	G	SNP	OS08G0542100	Intronic	NA	NA	LOC_Os08g42920	60S ribosomal protein L7, putative, expressed
Chr8	27480322	27480322	G	A	G	SNP	OS08G0547900	Exonic	CDS	Silent	LOC_Os08g43440	cytochrome P450, putative, expressed
Chr8	2174854	2174854	C	A	C	SNP	OS08G0139000	Exonic	3UTR	NA	LOC_Os08g04440	RNA recognition motif containing protein, putative, expressed
Chr8	2978709	2978709	C	A	C	SNP	OS08G0151800	Exonic	CDS	Silent	LOC_Os08g05570	monodehydroascorbate reductase, putative, expressed
Chr8	3360905	3360905	C	A	C	SNP	OS08G0157600	Exonic	5UTR	NA	LOC_Os08g06110	MYB family transcription factor, putative, expressed
Chr8	3363607	3363607	C	A	C	SNP	OS08G0157600	Intronic	NA	NA	LOC_Os08g06110	MYB family transcription factor, putative, expressed
Chr8	3389509	3389509	C	A	C	SNP	OS08G0157900	Exonic	3UTR	NA	LOC_Os08g06140	no apical meristem protein, putative, expressed
Chr8	3547767	3547767	C	A	C	SNP	OS08G0160500	Exonic	CDS	Silent	LOC_Os08g06380	CSLF6 - cellulose synthase-like family F; beta1,3;1,4 glucan synthase, expressed
Chr8	3549210	3549210	C	A	C	SNP	OS08G0160500	Intronic	NA	NA	LOC_Os08g06380	CSLF6 - cellulose synthase-like family F; beta1,3;1,4 glucan synthase, expressed
Chr8	5242742	5242742	C	A	C	SNP	OS08G0189600	Intronic	NA	NA	LOC_Os08g09010	Cupin domain containing protein, expressed
Chr8	7367468	7367468	C	A	C	SNP	OS08G0221300	Intronic	NA	NA	LOC_Os08g12449	expressed protein
Chr8	19031623	19031623	C	A	C	SNP	OS08G0398800	Exonic	3UTR	NA	LOC_Os08g30820	cleavage and polyadenylation specificity factor subunit 5, putative, expressed
Chr8	20424214	20424214	C	A	C	SNP	OS08G0425200	Intronic	NA	NA	LOC_Os08g32930	expressed protein
Chr8	20464108	20464108	C	A	C	SNP	OS08G0425800	Exonic	3UTR	NA	LOC_Os08g32980	expressed protein
Chr8	24854440	24854440	C	A	C	SNP	OS08G0503000	Intronic	NA	NA	LOC_Os08g39330	skin secretory protein xP2 precursor, putative, expressed
Chr8	25247553	25247553	C	A	C	SNP	OS08G0509100	Intronic	NA	NA	LOC_Os08g39850	lipoxygenase, chloroplast precursor, putative, expressed
Chr8	26842799	26842799	C	A	C	SNP	OS08G0536800	Intronic	NA	NA	LOC_Os08g42470	BEE 1, putative, expressed
Chr8	25244240	25244241	AT	A	AT	INDEL	OS08G0509100	Intronic	NA	NA	LOC_Os08g39850	lipoxygenase, chloroplast precursor, putative, expressed
Chr8	2977938	2977946	AGTGGGTGG	A	AGTGGGTGG	INDEL	OS08G0151800	Intronic	NA	NA	LOC_Os08g05570	monodehydroascorbate reductase, putative, expressed
Chr8	2507721	2507723	AAG	A	AAG	INDEL	OS08G0144400	Intronic	NA	NA	LOC_Os08g04920	OsDegp11 - Putative Deg protease homologue, expressed
Chr8	687243	687243	T	A	T	SNP	OS08G0112900	Intronic	NA	NA	LOC_Os08g02094	GDSL-like lipase/acylhydrolase, putative, expressed
Chr8	688660	688660	T	A	T	SNP	OS08G0112900	Intronic	NA	NA	LOC_Os08g02094	GDSL-like lipase/acylhydrolase, putative, expressed
Chr8	688804	688804	T	A	T	SNP	OS08G0112900	Intronic	NA	NA	LOC_Os08g02094	GDSL-like lipase/acylhydrolase, putative, expressed
Chr8	688875	688875	G	A	G	SNP	OS08G0112900	Intronic	NA	NA	LOC_Os08g02094	GDSL-like lipase/acylhydrolase, putative, expressed
Chr8	689028	689028	G	A	G	SNP	OS08G0112900	Intronic	NA	NA	LOC_Os08g02094	GDSL-like lipase/acylhydrolase, putative, expressed
Chr8	689119	689119	G	A	G	SNP	OS08G0112900	Intronic	NA	NA	LOC_Os08g02094	GDSL-like lipase/acylhydrolase, putative, expressed
Chr8	689125	689125	G	A	G	SNP	OS08G0112900	Intronic	NA	NA	LOC_Os08g02094	GDSL-like lipase/acylhydrolase, putative, expressed
Chr8	689140	689140	G	A	G	SNP	OS08G0112900	Intronic	NA	NA	LOC_Os08g02094	GDSL-like lipase/acylhydrolase, putative, expressed
Chr8	690789	690789	G	A	G	SNP	OS08G0112900	Intronic	NA	NA	LOC_Os08g02094	GDSL-like lipase/acylhydrolase, putative, expressed
Chr8	691616	691616	G	A	G	SNP	OS08G0112900	Intronic	NA	NA	LOC_Os08g02094	GDSL-like lipase/acylhydrolase, putative, expressed
Chr8	691653	691653	T	A	T	SNP	OS08G0112900	Intronic	NA	NA	LOC_Os08g02094	GDSL-like lipase/acylhydrolase, putative, expressed

Chr8	2920147	2920147	G	A	G	SNP	OS08G0150700	Intronic	NA	NA	LOC_Os08g05480	OsFBX261 - F-box domain containing protein, expressed
Chr8	3726021	3726021	G	A	G	SNP	OS08G0163100	Exonic	CDS	Missense	LOC_Os08g06600	nuf2 family protein, expressed
Chr8	3726087	3726087	C	A	C	SNP	OS08G0163100	Exonic	CDS	Missense	LOC_Os08g06600	nuf2 family protein, expressed
Chr8	5140022	5140022	G	A	G	SNP	OS08G0187800	Intronic	NA	NA	LOC_Os08g08840	phosphate/phosphate translocator, putative, expressed
Chr8	5234059	5234059	C	A	C	SNP	OS08G0189400	Intronic	NA	NA	LOC_Os08g08990	Cupin domain containing protein, expressed
Chr8	5982639	5982639	G	A	G	SNP	OS08G0203100	Exonic	CDS	NA	LOC_Os08g10290	SHR5-receptor-like kinase, putative, expressed
Chr8	5985765	5985765	C	A	C	SNP	OS08G0203100	Exonic	3UTR	NA	LOC_Os08g10290	SHR5-receptor-like kinase, putative, expressed
Chr8	6206278	6206278	T	A	T	SNP	OS08G0206400	Exonic	5UTR	NA	LOC_Os08g10550	potassium transporter, putative, expressed
Chr8	6207698	6207698	T	A	T	SNP	OS08G0206400	Intronic	NA	NA	LOC_Os08g10550	potassium transporter, putative, expressed
Chr8	7364073	7364073	G	A	G	SNP	OS08G0221300	Intronic	NA	NA	LOC_Os08g12449	expressed protein
Chr8	7364394	7364394	G	A	G	SNP	OS08G0221300	Intronic	NA	NA	LOC_Os08g12449	expressed protein
Chr8	7364526	7364526	G	A	G	SNP	OS08G0221300	Intronic	NA	NA	LOC_Os08g12449	expressed protein
Chr8	7364685	7364685	C	A	C	SNP	OS08G0221300	Intronic	NA	NA	LOC_Os08g12449	expressed protein
Chr8	7364909	7364909	G	A	G	SNP	OS08G0221300	Intronic	NA	NA	LOC_Os08g12449	expressed protein
Chr8	9508510	9508510	G	A	G	SNP	OS08G0256000	Exonic	5UTR	NA	LOC_Os08g15650	expressed protein
Chr8	17576347	17576347	C	A	C	SNP	OS08G0374800	Exonic	5UTR	NA	LOC_Os08g28730	NAD dependent epimerase/dehydratase family protein, putative, expressed
Chr8	19207043	19207043	G	A	G	SNP	OS08G0402500	Intronic	NA	NA	LOC_Os08g31090	expressed protein
Chr8	19207138	19207138	G	A	G	SNP	OS08G0402500	Intronic	NA	NA	LOC_Os08g31090	expressed protein
Chr8	19207270	19207270	G	A	G	SNP	OS08G0402500	Exonic	CDS	Silent	LOC_Os08g31090	expressed protein
Chr8	20423930	20423930	T	A	T	SNP	OS08G0425200	Exonic	CDS	Silent	LOC_Os08g32930	expressed protein
Chr8	20426445	20426445	G	A	G	SNP	OS08G0425200	Intronic	NA	NA	LOC_Os08g32930	expressed protein
Chr8	20427483	20427483	G	A	G	SNP	OS08G0425200	Exonic	3UTR	NA	LOC_Os08g32930	expressed protein
Chr8	20464757	20464757	G	A	G	SNP	OS08G0425800	Exonic	CDS	Missense	LOC_Os08g32980	expressed protein
Chr8	20464767	20464767	G	A	G	SNP	OS08G0425800	Exonic	CDS	Missense	LOC_Os08g32980	expressed protein
Chr8	22645711	22645711	C	A	C	SNP	OS08G0461000	Exonic	CDS	Missense	LOC_Os08g35900	OsFBL49 - F-box domain and LRR containing protein, expressed
Chr8	23119018	23119018	G	A	G	SNP	OS08G0470200	Exonic	CDS	Silent	LOC_Os08g36630	bifunctional monodehydroascorbate reductase and carbonic anhydrasenectarin-3 precursor, putative, expressed
Chr8	24854840	24854840	G	A	G	SNP	OS08G0503000	Intronic	NA	NA	LOC_Os08g39330	skin secretory protein xP2 precursor, putative, expressed
Chr8	24855474	24855474	G	A	G	SNP	OS08G0503000	Exonic	3UTR	NA	LOC_Os08g39330	skin secretory protein xP2 precursor, putative, expressed
Chr8	25242197	25242197	G	A	G	SNP	OS08G0509100	Intronic	NA	NA	LOC_Os08g39850	lipoxygenase, chloroplast precursor, putative, expressed
Chr8	25242289	25242289	G	A	G	SNP	OS08G0509100	Intronic	NA	NA	LOC_Os08g39850	lipoxygenase, chloroplast precursor, putative, expressed
Chr8	25242313	25242313	G	A	G	SNP	OS08G0509100	Intronic	NA	NA	LOC_Os08g39850	lipoxygenase, chloroplast precursor, putative, expressed
Chr8	25246433	25246433	G	A	G	SNP	OS08G0509100	Intronic	NA	NA	LOC_Os08g39850	lipoxygenase, chloroplast precursor, putative, expressed
Chr8	25246521	25246521	G	A	G	SNP	OS08G0509100	Intronic	NA	NA	LOC_Os08g39850	lipoxygenase, chloroplast precursor, putative, expressed
Chr8	25246570	25246570	C	A	C	SNP	OS08G0509100	Intronic	NA	NA	LOC_Os08g39850	lipoxygenase, chloroplast precursor, putative, expressed
Chr8	25249224	25249224	C	A	C	SNP	OS08G0509100	Exonic	CDS	Missense	LOC_Os08g39850	lipoxygenase, chloroplast precursor, putative, expressed
Chr8	26728516	26728516	G	A	G	SNP	OS08G0535200	Intronic	NA	NA	LOC_Os08g42350	nodulin MtN3 family protein, putative, expressed
Chr8	27480490	27480490	C	A	C	SNP	OS08G0547900	Exonic	CDS	Missense	LOC_Os08g43440	cytochrome P450, putative, expressed
Chr8	9099368	9099377	AGCGGCGCCG	A	AGCGGCGCCG	INDEL	OS08G0249000	Exonic	CDS	InFrame	LOC_Os08g15050	CCT/B-box zinc finger protein, putative, expressed
Chr8	28009662	28009670	ACCGTCGCG	A	ACCGTCGCG	INDEL	OS08G0559700	Exonic	5UTR	NA	LOC_Os08g44540	expressed protein
Chr8	25244253	25244262	ACTAGTAGAG	A	ACTAGTAGAG	INDEL	OS08G0509100	Intronic	NA	NA	LOC_Os08g39850	lipoxygenase, chloroplast precursor, putative, expressed
Chr8	5984975	5984978	ACTT	A	ACTT	INDEL	OS08G0203100	Exonic	3UTR	NA	LOC_Os08g10290	SHR5-receptor-like kinase, putative, expressed
Chr8	17511603	17511606	AGCT	A	AGCT	INDEL	OS08G0374000	Exonic	5UTR	NA	LOC_Os08g28670	pathogenesis-related Bet v I family protein, putative, expressed
Chr8	19746803	19746805	AGG	A	AGG	INDEL	OS08G0412700	Exonic	3UTR	NA	LOC_Os08g31850	expressed protein
Chr8	5983702	5983703	AT	A	AT	INDEL	OS08G0203100	Intronic	NA	NA	LOC_Os08g10290	SHR5-receptor-like kinase, putative, expressed
Chr8	2977419	2977422	ATCT	A	ATCT	INDEL	OS08G0151800	Exonic	5UTR	NA	LOC_Os08g05570	monodehydroascorbate reductase, putative, expressed
Chr8	9507331	9507334	ATTG	A	ATTG	INDEL	OS08G0256000	Exonic	3UTR	NA	LOC_Os08g15650	expressed protein
Chr9	21956491	21956491	G	A	G	SNP	OS09G0554200	Exonic	3UTR	NA	LOC_Os09g38110	RING-H2 finger protein, putative, expressed
Chr9	11807848	11807848	C	A	C	SNP	OS09G0361400	Exonic	3UTR	NA	LOC_Os09g19734	isochorismate synthase 1, chloroplast precursor, putative, expressed
Chr9	20370730	20370730	T	A	T	SNP	OS09G0521400	Exonic	CDS	Missense	LOC_Os09g34960	hydroxymethylglutaryl-CoA synthase, putative, expressed
Chr9	13570500	13570500	G	A	G	SNP	OS09G0392400	Exonic	CDS	NA	LOC_Os09g22500	expressed protein
Chr9	11807748	11807748	G	A	G	SNP	OS09G0361400	Exonic	CDS	Silent	LOC_Os09g19734	isochorismate synthase 1, chloroplast precursor, putative, expressed
Chr9	13572136	13572136	G	A	G	SNP	OS09G0392400	Exonic	5UTR	NA	LOC_Os09g22500	expressed protein
Chr9	22770286	22770286	G	A	G	SNP	OS09G0570400	Exonic	CDS	Silent	LOC_Os09g39680	transporter, major facilitator family, putative, expressed
Chr9	7200075	7200075	T	A	T	SNP	OS09G0297000	Intronic	NA	NA	LOC_Os09g12560	ferrochelatase-2, chloroplast precursor, putative, expressed
Chr9	7280508	7280508	T	A	T	SNP	OS09G0298500	Intronic	NA	NA	LOC_Os09g12720	zinc finger, C3HC4 type domain containing protein, expressed
Chr9	9359905	9359905	T	A	T	SNP	OS09G0321900	Intronic	NA	NA	LOC_Os09g15320	ubiquitin-conjugating enzyme, putative, expressed
Chr9	16189704	16189704	T	A	T	SNP	OS09G0438000	Intronic	NA	NA	LOC_Os09g26660	ferric reductase, putative, expressed
Chr9	18320697	18320697	T	A	T	SNP	OS09G0478300	Intronic	NA	NA	LOC_Os09g30130	CSLE6 - cellulose synthase-like family E, expressed
Chr9	18709134	18709134	T	A	T	SNP	OS09G0484800	Intronic	NA	NA	LOC_Os09g31120	pirin, putative, expressed
Chr9	18718654	18718654	T	A	T	SNP	OS09G0484900	Exonic	3UTR	NA	LOC_Os09g31130	citrate transporter, putative, expressed
Chr9	18761475	18761475	T	A	T	SNP	OS09G0486500	Exonic	3UTR	NA	LOC_Os09g31200	AN1-like zinc finger domain containing protein, expressed
Chr9	18850719	18850719	T	A	T	SNP	OS09G0488700	Intronic	NA	NA	LOC_Os09g31360	expressed protein
Chr9	7471074	7471074	G	A	G	SNP	OS09G0300800	Intronic	NA	NA	LOC_Os09g12970	plant protein of unknown function domain containing protein, expressed
Chr9	7475818	7475818	G	A	G	SNP	OS09G0300800	Intronic	NA	NA	LOC_Os09g12970	plant protein of unknown function domain containing protein, expressed
Chr9	7476504	7476504	G	A	G	SNP	OS09G0300800	Intronic	NA	NA	LOC_Os09g12970	plant protein of unknown function domain containing protein, expressed
Chr9	7476565	7476565	G	A	G	SNP	OS09G0300800	Intronic	NA	NA	LOC_Os09g12970	plant protein of unknown function domain containing protein, expressed
Chr9	7482402	7482402	G	A	G	SNP	OS09G0300800	Exonic	CDS	Missense	LOC_Os09g12970	plant protein of unknown function domain containing protein, expressed
Chr9	8694561	8694561	G	A	G	SNP	OS09G0315700	Exonic	CDS	Silent	LOC_Os09g14670	phosphoenolpyruvate carboxylase, putative, expressed
Chr9	8694576	8694576	G	A	G	SNP	OS09G0315700	Exonic	CDS	Silent	LOC_Os09g14670	phosphoenolpyruvate carboxylase, putative, expressed
Chr9	8694738	8694738	G	A	G	SNP	OS09G0315700	Exonic	CDS	Silent	LOC_Os09g14670	phosphoenolpyruvate carboxylase, putative, expressed
Chr9	8695024	8695024	G	A	G	SNP	OS09G0315700	Exonic	CDS	Silent	LOC_Os09g14670	phosphoenolpyruvate carboxylase, putative, expressed
Chr9	8695461	8695461	G	A	G	SNP	OS09G0315700	Exonic	CDS	Silent	LOC_Os09g14670	phosphoenolpyruvate carboxylase, putative, expressed
Chr9	9358871	9358871	G	A	G	SNP	OS09G0321900	Intronic	NA	NA	LOC_Os09g15320	ubiquitin-conjugating enzyme, putative, expressed
Chr9	9360303	9360303	G	A	G	SNP	OS09G0321900	Intronic	NA	NA	LOC_Os09g15320	ubiquitin-conjugating enzyme, putative, expressed
Chr9	9368696	9368696	G	A	G	SNP	OS09G0322000	Intronic	NA	NA	LOC_Os09g15330	transporter family protein, putative, expressed
Chr9	9568799	9568799	G	A	G	SNP	OS09G0325700	Exonic	3UTR	NA	LOC_Os09g15670	protein phosphatase 2C, putative, expressed
Chr9	11806674	11806674	C	A	C	SNP	OS09G0361400	Exonic	5UTR	NA	LOC_Os09g19734	isochorismate synthase 1, chloroplast precursor, putative, expressed
Chr9	11807243	11807243	G	A	G	SNP	OS09G0361400	Exonic	5UTR	NA	LOC_Os09g19734	isochorismate synthase 1, chloroplast precursor, putative, expressed
Chr9	11844910	11844910	G	A	G	SNP	OS09G0362500	Exonic	CDS	NA	LOC_Os09g19790	puromycin-sensitive aminopeptidase, putative, expressed
Chr9	11850322	11850322	G	A	G	SNP	OS09G0362500	Intronic	NA	NA	LOC_Os09g19790	puromycin-sensitive aminopeptidase, putative, expressed
Chr9	16154168	16154168	G	A	G	SNP	OS09G0437500	Intronic	NA	NA	LOC_Os09g26620	auxin-repressed protein, putative, expressed
Chr9	16154229	16154229	G	A	G	SNP	OS09G0437500	Intronic	NA	NA	LOC_Os09g26620	auxin-repressed protein, putative, expressed
Chr9	16154603	16154603	G	A	G	SNP	OS09G0437500	Exonic	3UTR	NA	LOC_Os09g26620	auxin-repressed protein, putative, expressed
Chr9	16303254	16303254	G	A	G	SNP	OS09G0439700	Intronic	NA	NA	LOC_Os09g26830	lung seven transmembrane domain containing protein, putative, expressed
Chr9	17903188	17903188	G	A	G	SNP	OS09G0470500	Exonic	5UTR	NA	LOC_Os09g29460	homeobox associated leucine zipper, putative, expressed

Chr9	17903194	17903194	G	A	G	SNP	OS09G0470500	Exonic	5UTR	NA	LOC_Os09g29460	homeobox associated leucine zipper, putative, expressed
Chr9	18318697	18318697	G	A	G	SNP	OS09G0478300	Exonic	CDS	Silent	LOC_Os09g30130	CSLE6 - cellulose synthase-like family E, expressed
Chr9	18319043	18319043	G	A	G	SNP	OS09G0478300	Intronic	NA	NA	LOC_Os09g30130	CSLE6 - cellulose synthase-like family E, expressed
Chr9	18319924	18319924	G	A	G	SNP	OS09G0478300	Intronic	NA	NA	LOC_Os09g30130	CSLE6 - cellulose synthase-like family E, expressed
Chr9	18320003	18320003	G	A	G	SNP	OS09G0478300	Intronic	NA	NA	LOC_Os09g30130	CSLE6 - cellulose synthase-like family E, expressed
Chr9	18320072	18320072	G	A	G	SNP	OS09G0478300	Intronic	NA	NA	LOC_Os09g30130	CSLE6 - cellulose synthase-like family E, expressed
Chr9	18320086	18320086	G	A	G	SNP	OS09G0478300	Intronic	NA	NA	LOC_Os09g30130	CSLE6 - cellulose synthase-like family E, expressed
Chr9	18320132	18320132	G	A	G	SNP	OS09G0478300	Intronic	NA	NA	LOC_Os09g30130	CSLE6 - cellulose synthase-like family E, expressed
Chr9	18321654	18321654	G	A	G	SNP	OS09G0478300	Exonic	5UTR	NA	LOC_Os09g30130	CSLE6 - cellulose synthase-like family E, expressed
Chr9	18321783	18321783	G	A	G	SNP	OS09G0478300	Exonic	5UTR	NA	LOC_Os09g30130	CSLE6 - cellulose synthase-like family E, expressed
Chr9	18670075	18670075	G	A	G	SNP	OS09G0483400	Intronic	NA	NA	LOC_Os09g31031	ubiquitin family protein, putative, expressed
Chr9	18670369	18670369	G	A	G	SNP	OS09G0483400	Intronic	NA	NA	LOC_Os09g31031	ubiquitin family protein, putative, expressed
Chr9	18709096	18709096	G	A	G	SNP	OS09G0484800	Intronic	NA	NA	LOC_Os09g31120	pirin, putative, expressed
Chr9	18709122	18709122	G	A	G	SNP	OS09G0484800	Intronic	NA	NA	LOC_Os09g31120	pirin, putative, expressed
Chr9	18761375	18761375	G	A	G	SNP	OS09G0486500	Exonic	3UTR	NA	LOC_Os09g31200	AN1-like zinc finger domain containing protein, expressed
Chr9	20869811	20869811	G	A	G	SNP	OS09G0532000	Intronic	NA	NA	LOC_Os09g36200	senescence-inducible chloroplast stay-green protein 1, putative, expressed
Chr9	21226486	21226486	G	A	G	SNP	OS09G0539100	Intronic	NA	NA	LOC_Os09g36800	3-dehydroquinate synthase, putative, expressed
Chr9	21226733	21226733	G	A	G	SNP	OS09G0539100	Intronic	NA	NA	LOC_Os09g36800	3-dehydroquinate synthase, putative, expressed
Chr9	21228658	21228658	G	A	G	SNP	OS09G0539100	Intronic	NA	NA	LOC_Os09g36800	3-dehydroquinate synthase, putative, expressed
Chr9	21280040	21280040	G	A	G	SNP	OS09G0540600	Intronic	NA	NA	LOC_Os09g36900	WD domain, G-beta repeat domain containing protein, expressed
Chr9	22884315	22884315	G	A	G	SNP	OS09G0572400	Exonic	CDS	Silent	LOC_Os09g39910	ABC transporter, ATP-binding protein, putative, expressed
Chr9	22905712	22905712	G	A	G	SNP	OS09G0572700	Exonic	5UTR	NA	LOC_Os09g39940	plastocyanin-like domain containing protein, putative, expressed
Chr9	22905738	22905738	G	A	G	SNP	OS09G0572700	Exonic	5UTR	NA	LOC_Os09g39940	plastocyanin-like domain containing protein, putative, expressed
Chr9	22905874	22905874	G	A	G	SNP	OS09G0572700	Exonic	5UTR	NA	LOC_Os09g39940	plastocyanin-like domain containing protein, putative, expressed
Chr9	22905929	22905929	G	A	G	SNP	OS09G0572700	Exonic	5UTR	NA	LOC_Os09g39940	plastocyanin-like domain containing protein, putative, expressed
Chr9	22906054	22906054	G	A	G	SNP	OS09G0572700	Exonic	5UTR	NA	LOC_Os09g39940	plastocyanin-like domain containing protein, putative, expressed
Chr9	22906078	22906078	G	A	G	SNP	OS09G0572700	Exonic	5UTR	NA	LOC_Os09g39940	plastocyanin-like domain containing protein, putative, expressed
Chr9	22906106	22906106	G	A	G	SNP	OS09G0572700	Exonic	5UTR	NA	LOC_Os09g39940	plastocyanin-like domain containing protein, putative, expressed
Chr9	22906484	22906484	G	A	G	SNP	OS09G0572700	Exonic	5UTR	NA	LOC_Os09g39940	plastocyanin-like domain containing protein, putative, expressed
Chr9	22906493	22906493	G	A	G	SNP	OS09G0572700	Exonic	5UTR	NA	LOC_Os09g39940	plastocyanin-like domain containing protein, putative, expressed
Chr9	22906498	22906498	G	A	G	SNP	OS09G0572700	Exonic	5UTR	NA	LOC_Os09g39940	plastocyanin-like domain containing protein, putative, expressed
Chr9	22906516	22906516	G	A	G	SNP	OS09G0572700	Exonic	5UTR	NA	LOC_Os09g39940	plastocyanin-like domain containing protein, putative, expressed
Chr9	22906519	22906519	G	A	G	SNP	OS09G0572700	Exonic	5UTR	NA	LOC_Os09g39940	plastocyanin-like domain containing protein, putative, expressed
Chr9	22906951	22906951	G	A	G	SNP	OS09G0572700	Exonic	5UTR	NA	LOC_Os09g39940	plastocyanin-like domain containing protein, putative, expressed
Chr9	22911644	22911644	G	A	G	SNP	OS09G0572900	Exonic	3UTR	NA	LOC_Os09g39960	dynamin family protein, putative, expressed
Chr9	22913358	22913358	G	A	G	SNP	OS09G0572900	Intronic	NA	NA	LOC_Os09g39960	dynamin family protein, putative, expressed
Chr9	7001109	7001109	G	A	G	SNP	OS09G0294000	Intronic	NA	NA	LOC_Os09g12290	bifunctional aspartokinase/homoserine dehydrogenase, chloroplast precursor, putative, expressed
Chr9	7001134	7001134	G	A	G	SNP	OS09G0294000	Intronic	NA	NA	LOC_Os09g12290	bifunctional aspartokinase/homoserine dehydrogenase, chloroplast precursor, putative, expressed
Chr9	11806359	11806359	G	A	G	SNP	OS09G0361400	Exonic	5UTR	NA	LOC_Os09g19734	isochorismate synthase 1, chloroplast precursor, putative, expressed
Chr9	7199926	7199926	C	A	C	SNP	OS09G0297000	Intronic	NA	NA	LOC_Os09g12560	ferrochelatase-2, chloroplast precursor, putative, expressed
Chr9	7280156	7280156	C	A	C	SNP	OS09G0298500	Intronic	NA	NA	LOC_Os09g12720	zinc finger, C3HC4 type domain containing protein, expressed
Chr9	7470985	7470985	C	A	C	SNP	OS09G0300800	Intronic	NA	NA	LOC_Os09g12970	plant protein of unknown function domain containing protein, expressed
Chr9	7472060	7472060	C	A	C	SNP	OS09G0300800	Intronic	NA	NA	LOC_Os09g12970	plant protein of unknown function domain containing protein, expressed
Chr9	7473044	7473044	C	A	C	SNP	OS09G0300800	Intronic	NA	NA	LOC_Os09g12970	plant protein of unknown function domain containing protein, expressed
Chr9	7480624	7480624	C	A	C	SNP	OS09G0300800	Intronic	NA	NA	LOC_Os09g12970	plant protein of unknown function domain containing protein, expressed
Chr9	7481224	7481224	C	A	C	SNP	OS09G0300800	Exonic	CDS	Missense	LOC_Os09g12970	plant protein of unknown function domain containing protein, expressed
Chr9	9654132	9654132	C	A	C	SNP	OS09G0327300	Exonic	CDS	Missense	LOC_Os09g15810	bifunctional protein folD, putative, expressed
Chr9	11845477	11845477	C	A	C	SNP	OS09G0362500	Exonic	CDS	NA	LOC_Os09g19790	puromycin-sensitive aminopeptidase, putative, expressed
Chr9	18670304	18670304	C	A	C	SNP	OS09G0483400	Intronic	NA	NA	LOC_Os09g31031	ubiquitin family protein, putative, expressed
Chr9	18852915	18852915	C	A	C	SNP	OS09G0488700	Exonic	3UTR	NA	LOC_Os09g31360	expressed protein
Chr9	22907765	22907765	C	A	C	SNP	OS09G0572700	Exonic	5UTR	NA	LOC_Os09g39940	plastocyanin-like domain containing protein, putative, expressed
Chr9	22911645	22911645	C	A	C	SNP	OS09G0572900	Exonic	3UTR	NA	LOC_Os09g39960	dynamin family protein, putative, expressed
Chr9	22915508	22915508	C	A	C	SNP	OS09G0572900	Intronic	NA	NA	LOC_Os09g39960	dynamin family protein, putative, expressed
Chr9	2128208	2128208	G	A	G	SNP	OS09G0127800	Exonic	CDS	Missense	LOC_Os09g04110	coatomer alpha subunit, putative, expressed
Chr9	3049065	3049065	G	A	G	SNP	OS09G0240200	Intronic	NA	NA	LOC_Os09g06464	CCT/B-box zinc finger protein, putative, expressed
Chr9	3051515	3051515	G	A	G	SNP	OS09G0240200	Intronic	NA	NA	LOC_Os09g06464	CCT/B-box zinc finger protein, putative, expressed
Chr9	3056587	3056587	G	A	G	SNP	OS09G0240200	Intronic	NA	NA	LOC_Os09g06464	CCT/B-box zinc finger protein, putative, expressed
Chr9	3057664	3057664	G	A	G	SNP	OS09G0240200	Intronic	NA	NA	LOC_Os09g06464	CCT/B-box zinc finger protein, putative, expressed
Chr9	3058944	3058944	G	A	G	SNP	OS09G0240200	Intronic	NA	NA	LOC_Os09g06464	CCT/B-box zinc finger protein, putative, expressed
Chr9	3585424	3585424	T	A	T	SNP	OS09G0247600	Intronic	NA	NA	LOC_Os09g07290	GDSL-like lipase/acylhydrolase, putative, expressed
Chr9	6388141	6388141	T	A	T	SNP	OS09G0286600	Exonic	3UTR	NA	LOC_Os09g11460	AP2 domain containing protein, expressed
Chr9	6388143	6388143	G	A	G	SNP	OS09G0286600	Exonic	3UTR	NA	LOC_Os09g11460	AP2 domain containing protein, expressed
Chr9	6388157	6388157	G	A	G	SNP	OS09G0286600	Exonic	3UTR	NA	LOC_Os09g11460	AP2 domain containing protein, expressed
Chr9	6388669	6388669	C	A	C	SNP	OS09G0286600	Exonic	3UTR	NA	LOC_Os09g11460	AP2 domain containing protein, expressed
Chr9	6388805	6388805	G	A	G	SNP	OS09G0286600	Exonic	3UTR	NA	LOC_Os09g11460	AP2 domain containing protein, expressed
Chr9	6388862	6388862	G	A	G	SNP	OS09G0286600	Exonic	3UTR	NA	LOC_Os09g11460	AP2 domain containing protein, expressed
Chr9	6388910	6388910	C	A	C	SNP	OS09G0286600	Exonic	3UTR	NA	LOC_Os09g11460	AP2 domain containing protein, expressed
Chr9	6405074	6405074	G	A	G	SNP	OS09G0287000	Intronic	NA	NA	LOC_Os09g11480	AP2 domain containing protein, expressed
Chr9	6996028	6996028	G	A	G	SNP	OS09G0294000	Exonic	CDS	Silent	LOC_Os09g12290	bifunctional aspartokinase/homoserine dehydrogenase, chloroplast precursor, putative, expressed
Chr9	6998281	6998281	G	A	G	SNP	OS09G0294000	Intronic	NA	NA	LOC_Os09g12290	bifunctional aspartokinase/homoserine dehydrogenase, chloroplast precursor, putative, expressed
Chr9	6998406	6998406	G	A	G	SNP	OS09G0294000	Intronic	NA	NA	LOC_Os09g12290	bifunctional aspartokinase/homoserine dehydrogenase, chloroplast precursor, putative, expressed
Chr9	6999965	6999965	C	A	C	SNP	OS09G0294000	Intronic	NA	NA	LOC_Os09g12290	bifunctional aspartokinase/homoserine dehydrogenase, chloroplast precursor, putative, expressed
Chr9	7002421	7002421	C	A	C	SNP	OS09G0294000	Intronic	NA	NA	LOC_Os09g12290	bifunctional aspartokinase/homoserine dehydrogenase, chloroplast precursor, putative, expressed
Chr9	7006787	7006787	G	A	G	SNP	OS09G0294000	Intronic	NA	NA	LOC_Os09g12290	bifunctional aspartokinase/homoserine dehydrogenase, chloroplast precursor, putative, expressed
Chr9	9652997	9652997	G	A	G	SNP	OS09G0327300	Intronic	NA	NA	LOC_Os09g15810	bifunctional protein folD, putative, expressed
Chr9	9653078	9653078	T	A	T	SNP	OS09G0327300	Intronic	NA	NA	LOC_Os09g15810	bifunctional protein folD, putative, expressed
Chr9	9653511	9653511	G	A	G	SNP	OS09G0327300	Intronic	NA	NA	LOC_Os09g15810	bifunctional protein folD, putative, expressed
Chr9	9653902	9653902	G	A	G	SNP	OS09G0327300	Exonic	CDS	Silent	LOC_Os09g15810	bifunctional protein folD, putative, expressed
Chr9	9654251	9654251	G	A	G	SNP	OS09G0327300	Exonic	CDS	Silent	LOC_Os09g15810	bifunctional protein folD, putative, expressed
Chr9	9654325	9654325	G	A	G	SNP	OS09G0327300	Intronic	NA	NA	LOC_Os09g15810	bifunctional protein folD, putative, expressed
Chr9	9654330	9654330	G	A	G	SNP	OS09G0327300	Intronic	NA	NA	LOC_Os09g15810	bifunctional protein folD, putative, expressed
Chr9	9654392	9654392	G	A	G	SNP	OS09G0327300	Intronic	NA	NA	LOC_Os09g15810	bifunctional protein folD, putative, expressed
Chr9	9654807	9654807	C	A	C	SNP	OS09G0327300	Exonic	CDS	Missense	LOC_Os09g15810	bifunctional protein folD, putative, expressed
Chr9	10252822	10252822	C	A	C	SNP	OS09G0337300	Exonic	5UTR	NA	LOC_Os09g16760	ankyrin repeat family protein, putative, expressed
Chr9	10253043	10253043	C	A	C	SNP	OS09G0337300	Exonic	5UTR	NA	LOC_Os09g16760	ankyrin repeat family protein, putative, expressed

Chr9	10339288	10339288	C	A	C	SNP	OS09G0338500	Exonic	CDS	Silent	LOC_Os09g16920	desaturase/cytochrome b5 protein, putative, expressed
Chr9	10340282	10340282	G	A	G	SNP	OS09G0338500	Exonic	3UTR	NA	LOC_Os09g16920	desaturase/cytochrome b5 protein, putative, expressed
Chr9	11806268	11806268	G	A	G	SNP	OS09G0361400	Exonic	5UTR	NA	LOC_Os09g19734	isochorismate synthase 1, chloroplast precursor, putative, expressed
Chr9	11806441	11806441	G	A	G	SNP	OS09G0361400	Exonic	5UTR	NA	LOC_Os09g19734	isochorismate synthase 1, chloroplast precursor, putative, expressed
Chr9	11806638	11806638	G	A	G	SNP	OS09G0361400	Exonic	5UTR	NA	LOC_Os09g19734	isochorismate synthase 1, chloroplast precursor, putative, expressed
Chr9	11806731	11806731	G	A	G	SNP	OS09G0361400	Exonic	5UTR	NA	LOC_Os09g19734	isochorismate synthase 1, chloroplast precursor, putative, expressed
Chr9	11806844	11806844	G	A	G	SNP	OS09G0361400	Exonic	5UTR	NA	LOC_Os09g19734	isochorismate synthase 1, chloroplast precursor, putative, expressed
Chr9	11807290	11807290	T	A	T	SNP	OS09G0361400	Exonic	5UTR	NA	LOC_Os09g19734	isochorismate synthase 1, chloroplast precursor, putative, expressed
Chr9	11807364	11807364	T	A	T	SNP	OS09G0361400	Exonic	CDS	Silent	LOC_Os09g19734	isochorismate synthase 1, chloroplast precursor, putative, expressed
Chr9	11807898	11807898	T	A	T	SNP	OS09G0361400	Exonic	3UTR	NA	LOC_Os09g19734	isochorismate synthase 1, chloroplast precursor, putative, expressed
Chr9	11808106	11808106	T	A	T	SNP	OS09G0361400	Exonic	3UTR	NA	LOC_Os09g19734	isochorismate synthase 1, chloroplast precursor, putative, expressed
Chr9	11808171	11808171	C	A	C	SNP	OS09G0361400	Exonic	3UTR	NA	LOC_Os09g19734	isochorismate synthase 1, chloroplast precursor, putative, expressed
Chr9	11808280	11808280	T	A	T	SNP	OS09G0361400	Exonic	3UTR	NA	LOC_Os09g19734	isochorismate synthase 1, chloroplast precursor, putative, expressed
Chr9	11808340	11808340	G	A	G	SNP	OS09G0361400	Exonic	3UTR	NA	LOC_Os09g19734	isochorismate synthase 1, chloroplast precursor, putative, expressed
Chr9	11808384	11808384	G	A	G	SNP	OS09G0361400	Exonic	3UTR	NA	LOC_Os09g19734	isochorismate synthase 1, chloroplast precursor, putative, expressed
Chr9	11808485	11808485	C	A	C	SNP	OS09G0361400	Exonic	CDS	Silent	LOC_Os09g19734	isochorismate synthase 1, chloroplast precursor, putative, expressed
Chr9	12803651	12803651	T	A	T	SNP	OS09G0379900	Exonic	CDS	Silent	LOC_Os09g21210	beta-glucan-binding protein 4, putative, expressed
Chr9	13155126	13155126	G	A	G	SNP	OS09G0385700	Exonic	3UTR	NA	LOC_Os09g21710	AN1-like zinc finger domain containing protein, expressed
Chr9	13555732	13555732	C	A	C	SNP	OS09G0392000	Exonic	CDS	Silent	LOC_Os09g22440	expressed protein
Chr9	13557878	13557878	G	A	G	SNP	OS09G0392000	Exonic	3UTR	NA	LOC_Os09g22440	expressed protein
Chr9	13567545	13567545	G	A	G	SNP	OS09G0392400	Exonic	3UTR	NA	LOC_Os09g22500	expressed protein
Chr9	13567560	13567560	T	A	T	SNP	OS09G0392400	Exonic	3UTR	NA	LOC_Os09g22500	expressed protein
Chr9	13567811	13567811	G	A	G	SNP	OS09G0392400	Intronic	NA	NA	LOC_Os09g22500	expressed protein
Chr9	13567833	13567833	G	A	G	SNP	OS09G0392400	Intronic	NA	NA	LOC_Os09g22500	expressed protein
Chr9	13567842	13567842	G	A	G	SNP	OS09G0392400	Intronic	NA	NA	LOC_Os09g22500	expressed protein
Chr9	13569971	13569971	G	A	G	SNP	OS09G0392400	Exonic	3UTR	NA	LOC_Os09g22500	expressed protein
Chr9	13570117	13570117	T	A	T	SNP	OS09G0392400	Exonic	3UTR	NA	LOC_Os09g22500	expressed protein
Chr9	13570294	13570294	G	A	G	SNP	OS09G0392400	Exonic	3UTR	NA	LOC_Os09g22500	expressed protein
Chr9	13572220	13572220	T	A	T	SNP	OS09G0392400	Exonic	5UTR	NA	LOC_Os09g22500	expressed protein
Chr9	13572602	13572602	G	A	G	SNP	OS09G0392400	Exonic	5UTR	NA	LOC_Os09g22500	expressed protein
Chr9	13572647	13572647	T	A	T	SNP	OS09G0392400	Exonic	5UTR	NA	LOC_Os09g22500	expressed protein
Chr9	13572680	13572680	T	A	T	SNP	OS09G0392400	Exonic	5UTR	NA	LOC_Os09g22500	expressed protein
Chr9	13572999	13572999	C	A	C	SNP	OS09G0392400	Exonic	5UTR	NA	LOC_Os09g22500	expressed protein
Chr9	13573134	13573134	C	A	C	SNP	OS09G0392400	Exonic	5UTR	NA	LOC_Os09g22500	expressed protein
Chr9	13573582	13573582	G	A	G	SNP	OS09G0392400	Exonic	5UTR	NA	LOC_Os09g22500	expressed protein
Chr9	13573610	13573610	C	A	C	SNP	OS09G0392400	Exonic	5UTR	NA	LOC_Os09g22500	expressed protein
Chr9	13573620	13573620	T	A	T	SNP	OS09G0392400	Exonic	5UTR	NA	LOC_Os09g22500	expressed protein
Chr9	13697906	13697906	G	A	G	SNP	OS09G0394700	Intronic	NA	NA	LOC_Os09g23150	monoglyceride lipase, putative, expressed
Chr9	13698542	13698542	G	A	G	SNP	OS09G0394700	Intronic	NA	NA	LOC_Os09g23150	monoglyceride lipase, putative, expressed
Chr9	14532090	14532090	G	A	G	SNP	OS09G0409950	Intronic	NA	NA	LOC_Os09g24440	expressed protein
Chr9	14532897	14532897	T	A	T	SNP	OS09G0409950	Intronic	NA	NA	LOC_Os09g24440	expressed protein
Chr9	14533332	14533332	C	A	C	SNP	OS09G0409950	Intronic	NA	NA	LOC_Os09g24440	expressed protein
Chr9	14635413	14635413	G	A	G	SNP	OS09G0412300	Exonic	3UTR	NA	LOC_Os09g24580	EF hand family protein, putative, expressed
Chr9	14870745	14870745	T	A	T	SNP	OS09G0415700	Exonic	3UTR	NA	LOC_Os09g24900	methyltransferase, putative, expressed
Chr9	15927957	15927957	C	A	C	SNP	OS09G0433800	Exonic	3UTR	NA	LOC_Os09g26370	DUF581 domain containing protein, expressed
Chr9	16189298	16189298	G	A	G	SNP	OS09G0438000	Intronic	NA	NA	LOC_Os09g26660	ferric reductase, putative, expressed
Chr9	16189304	16189304	G	A	G	SNP	OS09G0438000	Intronic	NA	NA	LOC_Os09g26660	ferric reductase, putative, expressed
Chr9	16191069	16191069	G	A	G	SNP	OS09G0438000	Intronic	NA	NA	LOC_Os09g26660	ferric reductase, putative, expressed
Chr9	16191170	16191170	G	A	G	SNP	OS09G0438000	Exonic	CDS	Missense	LOC_Os09g26660	ferric reductase, putative, expressed
Chr9	16191546	16191546	G	A	G	SNP	OS09G0438000	Intronic	NA	NA	LOC_Os09g26660	ferric reductase, putative, expressed
Chr9	16192828	16192828	T	A	T	SNP	OS09G0438000	Intronic	NA	NA	LOC_Os09g26660	ferric reductase, putative, expressed
Chr9	16195152	16195152	G	A	G	SNP	OS09G0438000	Intronic	NA	NA	LOC_Os09g26660	ferric reductase, putative, expressed
Chr9	16195939	16195939	T	A	T	SNP	OS09G0438000	Intronic	NA	NA	LOC_Os09g26660	ferric reductase, putative, expressed
Chr9	16196638	16196638	G	A	G	SNP	OS09G0438000	Intronic	NA	NA	LOC_Os09g26660	ferric reductase, putative, expressed
Chr9	16196855	16196855	T	A	T	SNP	OS09G0438000	Exonic	3UTR	NA	LOC_Os09g26660	ferric reductase, putative, expressed
Chr9	16197096	16197096	T	A	T	SNP	OS09G0438000	Exonic	3UTR	NA	LOC_Os09g26660	ferric reductase, putative, expressed
Chr9	16225705	16225705	G	A	G	SNP	OS09G0438400	Intronic	NA	NA	LOC_Os09g26700	choline/ethanolamine kinase, putative, expressed
Chr9	16228674	16228674	T	A	T	SNP	OS09G0438400	Exonic	CDS	NA	LOC_Os09g26700	choline/ethanolamine kinase, putative, expressed
Chr9	16388891	16388891	C	A	C	SNP	OS09G0441400	Intronic	NA	NA	LOC_Os09g26960	cytochrome P450, putative, expressed
Chr9	16390388	16390388	G	A	G	SNP	OS09G0441400	Exonic	3UTR	NA	LOC_Os09g26960	cytochrome P450, putative, expressed
Chr9	16441628	16441628	G	A	G	SNP	OS09G0442300	Intronic	NA	NA	LOC_Os09g27030	oryzain gamma chain precursor, putative, expressed
Chr9	16441818	16441818	G	A	G	SNP	OS09G0442300	Intronic	NA	NA	LOC_Os09g27030	oryzain gamma chain precursor, putative, expressed
Chr9	16442092	16442092	T	A	T	SNP	OS09G0442300	Intronic	NA	NA	LOC_Os09g27030	oryzain gamma chain precursor, putative, expressed
Chr9	16445029	16445029	G	A	G	SNP	OS09G0442400	Exonic	5UTR	NA	LOC_Os09g27040	GEX1, putative, expressed
Chr9	16446691	16446691	T	A	T	SNP	OS09G0442400	Exonic	CDS	Silent	LOC_Os09g27040	GEX1, putative, expressed
Chr9	16931005	16931005	G	A	G	SNP	OS09G0451500	Exonic	5UTR	NA	LOC_Os09g27830	OsPDIL2-3 protein disulfide isomerase PDIL2-3, expressed
Chr9	16932007	16932007	T	A	T	SNP	OS09G0451500	Intronic	NA	NA	LOC_Os09g27830	OsPDIL2-3 protein disulfide isomerase PDIL2-3, expressed
Chr9	16934536	16934536	G	A	G	SNP	OS09G0451500	Exonic	CDS	Missense	LOC_Os09g27830	OsPDIL2-3 protein disulfide isomerase PDIL2-3, expressed
Chr9	16935087	16935087	G	A	G	SNP	OS09G0451500	Intronic	NA	NA	LOC_Os09g27830	OsPDIL2-3 protein disulfide isomerase PDIL2-3, expressed
Chr9	16935876	16935876	C	A	C	SNP	OS09G0451500	Exonic	3UTR	NA	LOC_Os09g27830	OsPDIL2-3 protein disulfide isomerase PDIL2-3, expressed
Chr9	16959583	16959583	T	A	T	SNP	OS09G0452200	Intronic	NA	NA	LOC_Os09g27890	lysM domain-containing GPI-anchored protein precursor, putative, expressed
Chr9	16959703	16959703	G	A	G	SNP	OS09G0452200	Intronic	NA	NA	LOC_Os09g27890	lysM domain-containing GPI-anchored protein precursor, putative, expressed
Chr9	16960025	16960025	G	A	G	SNP	OS09G0452200	Intronic	NA	NA	LOC_Os09g27890	lysM domain-containing GPI-anchored protein precursor, putative, expressed
Chr9	16960559	16960559	G	A	G	SNP	OS09G0452200	Intronic	NA	NA	LOC_Os09g27890	lysM domain-containing GPI-anchored protein precursor, putative, expressed
Chr9	16961985	16961985	C	A	C	SNP	OS09G0452200	Exonic	3UTR	NA	LOC_Os09g27890	lysM domain-containing GPI-anchored protein precursor, putative, expressed
Chr9	16962123	16962123	C	A	C	SNP	OS09G0452200	Exonic	3UTR	NA	LOC_Os09g27890	lysM domain-containing GPI-anchored protein precursor, putative, expressed
Chr9	17081564	17081564	C	A	C	SNP	OS09G0454600	Intronic	NA	NA	LOC_Os09g28160	phosphate carrier protein, mitochondrial precursor, putative, expressed
Chr9	18978732	18978732	G	A	G	SNP	OS09G0491740	Exonic	3UTR	NA	LOC_Os09g31478	auxin efflux carrier component, putative, expressed
Chr9	18978769	18978769	G	A	G	SNP	OS09G0491740	Exonic	3UTR	NA	LOC_Os09g31478	auxin efflux carrier component, putative, expressed
Chr9	18979001	18979001	G	A	G	SNP	OS09G0491740	Exonic	3UTR	NA	LOC_Os09g31478	auxin efflux carrier component, putative, expressed
Chr9	18979162	18979162	T	A	T	SNP	OS09G0491740	Exonic	CDS	Silent	LOC_Os09g31478	auxin efflux carrier component, putative, expressed
Chr9	18979308	18979308	G	A	G	SNP	OS09G0491740	Intronic	NA	NA	LOC_Os09g31478	auxin efflux carrier component, putative, expressed
Chr9	18980217	18980217	C	A	C	SNP	OS09G0491740	Intronic	NA	NA	LOC_Os09g31478	auxin efflux carrier component, putative, expressed
Chr9	18980941	18980941	C	A	C	SNP	OS09G0491740	Intronic	NA	NA	LOC_Os09g31478	auxin efflux carrier component, putative, expressed
Chr9	18982366	18982366	G	A	G	SNP	OS09G0491740	Intronic	NA	NA	LOC_Os09g31478	auxin efflux carrier component, putative, expressed

Chr9	18982372	18982372	C	A	C	SNP	OS09G0491740	Intronic	NA	NA	LOC_Os09g31478	auxin efflux carrier component, putative, expressed
Chr9	18990113	18990113	G	A	G	SNP	OS09G0491772	Exonic	CDS	Silent	LOC_Os09g31486	DnaK family protein, putative, expressed
Chr9	19584544	19584544	C	A	C	SNP	OS09G0506000	Intronic	NA	NA	LOC_Os09g32840	nucleotide pyrophosphatase/phosphodiesterase, putative, expressed
Chr9	19868354	19868354	G	A	G	SNP	OS09G0511200	Exonic	CDS	Missense	LOC_Os09g33640	expressed protein
Chr9	19967630	19967630	G	A	G	SNP	OS09G0513100	Intronic	NA	NA	LOC_Os09g33820	lecithine cholesterol acyltransferase, putative, expressed
Chr9	19967778	19967778	G	A	G	SNP	OS09G0513100	Exonic	CDS	NA	LOC_Os09g33820	lecithine cholesterol acyltransferase, putative, expressed
Chr9	19968028	19968028	G	A	G	SNP	OS09G0513100	Intronic	NA	NA	LOC_Os09g33820	lecithine cholesterol acyltransferase, putative, expressed
Chr9	20344925	20344925	G	A	G	SNP	OS09G0520700	Intronic	NA	NA	LOC_Os09g34910	DEAD-box ATP-dependent RNA helicase 7, putative, expressed
Chr9	20368226	20368226	T	A	T	SNP	OS09G0521400	Intronic	NA	NA	LOC_Os09g34960	hydroxymethylglutaryl-CoA synthase, putative, expressed
Chr9	20496544	20496544	G	A	G	SNP	OS09G0524800	Exonic	5UTR	NA	LOC_Os09g35630	BAG domain-containing protein, putative, expressed
Chr9	21189721	21189721	G	A	G	SNP	OS09G0538400	Exonic	3UTR	NA	LOC_Os09g36730	MYB family transcription factor, putative, expressed
Chr9	21278389	21278389	T	A	T	SNP	OS09G0540600	Intronic	NA	NA	LOC_Os09g36900	WD domain, G-beta repeat domain containing protein, expressed
Chr9	21278689	21278689	G	A	G	SNP	OS09G0540600	Intronic	NA	NA	LOC_Os09g36900	WD domain, G-beta repeat domain containing protein, expressed
Chr9	21279341	21279341	G	A	G	SNP	OS09G0540600	Intronic	NA	NA	LOC_Os09g36900	WD domain, G-beta repeat domain containing protein, expressed
Chr9	21344074	21344074	G	A	G	SNP	OS09G0542000	Exonic	CDS	Missense	LOC_Os09g37006	protein of unknown function domain containing protein, expressed
Chr9	21345639	21345639	G	A	G	SNP	OS09G0542000	Intronic	NA	NA	LOC_Os09g37006	protein of unknown function domain containing protein, expressed
Chr9	21679322	21679322	G	A	G	SNP	OS09G0548200	Intronic	NA	NA	LOC_Os09g37600	lysM domain-containing GPI-anchored protein precursor, putative, expressed
Chr9	21857009	21857009	G	A	G	SNP	OS09G0551600	Intronic	NA	NA	LOC_Os09g37910	HMG1/2, putative, expressed
Chr9	21857516	21857516	C	A	C	SNP	OS09G0551600	Intronic	NA	NA	LOC_Os09g37910	HMG1/2, putative, expressed
Chr9	21857699	21857699	G	A	G	SNP	OS09G0551600	Intronic	NA	NA	LOC_Os09g37910	HMG1/2, putative, expressed
Chr9	21857773	21857773	C	A	C	SNP	OS09G0551600	Intronic	NA	NA	LOC_Os09g37910	HMG1/2, putative, expressed
Chr9	21858083	21858083	G	A	G	SNP	OS09G0551600	Intronic	NA	NA	LOC_Os09g37910	HMG1/2, putative, expressed
Chr9	21858253	21858253	G	A	G	SNP	OS09G0551600	Exonic	CDS	Silent	LOC_Os09g37910	HMG1/2, putative, expressed
Chr9	21858274	21858274	T	A	T	SNP	OS09G0551600	Exonic	CDS	Silent	LOC_Os09g37910	HMG1/2, putative, expressed
Chr9	21858715	21858715	C	A	C	SNP	OS09G0551600	Exonic	3UTR	NA	LOC_Os09g37910	HMG1/2, putative, expressed
Chr9	21895706	21895706	G	A	G	SNP	OS09G0552600	Exonic	3UTR	NA	LOC_Os09g37976	cupin domain containing protein, expressed
Chr9	21917982	21917982	C	A	C	SNP	OS09G0553200	Exonic	CDS	Silent	LOC_Os09g38030	UTP--glucose-1-phosphate uridylyltransferase, putative, expressed
Chr9	22427804	22427804	G	A	G	SNP	OS09G0564200	Exonic	CDS	Missense	LOC_Os09g39070	thiol protease SEN102 precursor, putative, expressed
Chr9	22428144	22428144	G	A	G	SNP	OS09G0564200	Intronic	NA	NA	LOC_Os09g39070	thiol protease SEN102 precursor, putative, expressed
Chr9	22432420	22432420	G	A	G	SNP	OS09G0564200	Intronic	NA	NA	LOC_Os09g39070	thiol protease SEN102 precursor, putative, expressed
Chr9	22432439	22432439	G	A	G	SNP	OS09G0564200	Intronic	NA	NA	LOC_Os09g39070	thiol protease SEN102 precursor, putative, expressed
Chr9	22694545	22694545	C	A	C	SNP	OS09G0568400	Intronic	NA	NA	LOC_Os09g39500	ubiquitin fusion protein, putative, expressed
Chr9	22769228	22769228	G	A	G	SNP	OS09G0570400	Intronic	NA	NA	LOC_Os09g39680	transporter, major facilitator family, putative, expressed
Chr9	22770444	22770444	T	A	T	SNP	OS09G0570400	Intronic	NA	NA	LOC_Os09g39680	transporter, major facilitator family, putative, expressed
Chr9	22431552	22431553	AT	A	AT	INDEL	OS09G0564200	Intronic	NA	NA	LOC_Os09g39070	thiol protease SEN102 precursor, putative, expressed
Chr9	20368012	20368016	AGGAT	A	AGGAT	INDEL	OS09G0521400	Intronic	NA	NA	LOC_Os09g34960	hydroxymethylglutaryl-CoA synthase, putative, expressed
Chr9	16932427	16932428	AT	A	AT	INDEL	OS09G0451500	Intronic	NA	NA	LOC_Os09g27830	OsPDIL2-3 protein disulfide isomerase PDIL2-3, expressed
Chr9	16441757	16441758	AT	A	AT	INDEL	OS09G0442300	Intronic	NA	NA	LOC_Os09g27030	oryzain gamma chain precursor, putative, expressed
Chr9	16190173	16190174	AT	A	AT	INDEL	OS09G0438000	Intronic	NA	NA	LOC_Os09g26660	ferric reductase, putative, expressed
Chr9	13573048	13573049	AT	A	AT	INDEL	OS09G0392400	Exonic	5UTR	NA	LOC_Os09g22500	expressed protein
Chr9	13573303	13573312	AAAATAACTC	A	AAAATAACTC	INDEL	OS09G0392400	Exonic	5UTR	NA	LOC_Os09g22500	expressed protein
Chr9	11806277	11806278	AT	A	AT	INDEL	OS09G0361400	Exonic	5UTR	NA	LOC_Os09g19734	isochorismate synthase 1, chloroplast precursor, putative, expressed
Chr9	9654106	9654114	ATTTTTGAT	A	ATTTTTGAT	INDEL	OS09G0327300	Intronic	NA	NA	LOC_Os09g15810	bifunctional protein folD, putative, expressed
Chr9	3049228	3049229	AC	A	AC	INDEL	OS09G0240200	Intronic	NA	NA	LOC_Os09g06464	CCT/B-box zinc finger protein, putative, expressed
Chr9	907562	907562	C	A	C	SNP	OS09G0110100	Exonic	CDS	Missense	LOC_Os09g02250	transposon protein, putative, unclassified, expressed
Chr9	1535821	1535821	G	A	G	SNP	OS09G0120033	Exonic	3UTR	NA	LOC_Os09g03190	expressed protein
Chr9	1537177	1537177	T	A	T	SNP	OS09G0120033	Exonic	5UTR	NA	LOC_Os09g03190	expressed protein
Chr9	3057637	3057637	G	A	G	SNP	OS09G0240200	Intronic	NA	NA	LOC_Os09g06464	CCT/B-box zinc finger protein, putative, expressed
Chr9	3059011	3059011	G	A	G	SNP	OS09G0240200	Intronic	NA	NA	LOC_Os09g06464	CCT/B-box zinc finger protein, putative, expressed
Chr9	3062148	3062148	G	A	G	SNP	OS09G0240200	Intronic	NA	NA	LOC_Os09g06464	CCT/B-box zinc finger protein, putative, expressed
Chr9	3583585	3583585	T	A	T	SNP	OS09G0247600	Intronic	NA	NA	LOC_Os09g07290	GDSL-like lipase/acylhydrolase, putative, expressed
Chr9	6388069	6388069	T	A	T	SNP	OS09G0286600	Exonic	3UTR	NA	LOC_Os09g11460	AP2 domain containing protein, expressed
Chr9	6388072	6388072	G	A	G	SNP	OS09G0286600	Exonic	3UTR	NA	LOC_Os09g11460	AP2 domain containing protein, expressed
Chr9	6388465	6388465	G	A	G	SNP	OS09G0286600	Exonic	3UTR	NA	LOC_Os09g11460	AP2 domain containing protein, expressed
Chr9	6388731	6388731	T	A	T	SNP	OS09G0286600	Exonic	3UTR	NA	LOC_Os09g11460	AP2 domain containing protein, expressed
Chr9	6388762	6388762	G	A	G	SNP	OS09G0286600	Exonic	3UTR	NA	LOC_Os09g11460	AP2 domain containing protein, expressed
Chr9	6388775	6388775	C	A	C	SNP	OS09G0286600	Exonic	3UTR	NA	LOC_Os09g11460	AP2 domain containing protein, expressed
Chr9	6389562	6389562	G	A	G	SNP	OS09G0286600	Exonic	CDS	Missense	LOC_Os09g11460	AP2 domain containing protein, expressed
Chr9	6997600	6997600	G	A	G	SNP	OS09G0294000	Intronic	NA	NA	LOC_Os09g12290	bifunctional aspartokinase/homoserine dehydrogenase, chloroplast precursor, putative, expressed
Chr9	6998040	6998040	G	A	G	SNP	OS09G0294000	Intronic	NA	NA	LOC_Os09g12290	bifunctional aspartokinase/homoserine dehydrogenase, chloroplast precursor, putative, expressed
Chr9	6998080	6998080	G	A	G	SNP	OS09G0294000	Intronic	NA	NA	LOC_Os09g12290	bifunctional aspartokinase/homoserine dehydrogenase, chloroplast precursor, putative, expressed
Chr9	6998180	6998180	G	A	G	SNP	OS09G0294000	Intronic	NA	NA	LOC_Os09g12290	bifunctional aspartokinase/homoserine dehydrogenase, chloroplast precursor, putative, expressed
Chr9	6998485	6998485	G	A	G	SNP	OS09G0294000	Intronic	NA	NA	LOC_Os09g12290	bifunctional aspartokinase/homoserine dehydrogenase, chloroplast precursor, putative, expressed
Chr9	6998578	6998578	G	A	G	SNP	OS09G0294000	Intronic	NA	NA	LOC_Os09g12290	bifunctional aspartokinase/homoserine dehydrogenase, chloroplast precursor, putative, expressed
Chr9	6999142	6999142	G	A	G	SNP	OS09G0294000	Intronic	NA	NA	LOC_Os09g12290	bifunctional aspartokinase/homoserine dehydrogenase, chloroplast precursor, putative, expressed
Chr9	6999211	6999211	G	A	G	SNP	OS09G0294000	Intronic	NA	NA	LOC_Os09g12290	bifunctional aspartokinase/homoserine dehydrogenase, chloroplast precursor, putative, expressed
Chr9	6999246	6999246	G	A	G	SNP	OS09G0294000	Intronic	NA	NA	LOC_Os09g12290	bifunctional aspartokinase/homoserine dehydrogenase, chloroplast precursor, putative, expressed
Chr9	6999519	6999519	G	A	G	SNP	OS09G0294000	Intronic	NA	NA	LOC_Os09g12290	bifunctional aspartokinase/homoserine dehydrogenase, chloroplast precursor, putative, expressed
Chr9	7000188	7000188	G	A	G	SNP	OS09G0294000	Intronic	NA	NA	LOC_Os09g12290	bifunctional aspartokinase/homoserine dehydrogenase, chloroplast precursor, putative, expressed
Chr9	7000466	7000466	G	A	G	SNP	OS09G0294000	Intronic	NA	NA	LOC_Os09g12290	bifunctional aspartokinase/homoserine dehydrogenase, chloroplast precursor, putative, expressed
Chr9	7000727	7000727	G	A	G	SNP	OS09G0294000	Intronic	NA	NA	LOC_Os09g12290	bifunctional aspartokinase/homoserine dehydrogenase, chloroplast precursor, putative, expressed
Chr9	7009397	7009397	G	A	G	SNP	OS09G0294000	Exonic	5UTR	NA	LOC_Os09g12290	bifunctional aspartokinase/homoserine dehydrogenase, chloroplast precursor, putative, expressed
Chr9	7198981	7198981	G	A	G	SNP	OS09G0297000	Exonic	CDS	NA	LOC_Os09g12560	ferrochelatase-2, chloroplast precursor, putative, expressed
Chr9	7471322	7471322	C	A	C	SNP	OS09G0300800	Intronic	NA	NA	LOC_Os09g12970	plant protein of unknown function domain containing protein, expressed
Chr9	7473840	7473840	G	A	G	SNP	OS09G0300800	Intronic	NA	NA	LOC_Os09g12970	plant protein of unknown function domain containing protein, expressed
Chr9	7474818	7474818	C	A	C	SNP	OS09G0300800	Intronic	NA	NA	LOC_Os09g12970	plant protein of unknown function domain containing protein, expressed
Chr9	7475026	7475026	G	A	G	SNP	OS09G0300800	Intronic	NA	NA	LOC_Os09g12970	plant protein of unknown function domain containing protein, expressed
Chr9	7476654	7476654	G	A	G	SNP	OS09G0300800	Intronic	NA	NA	LOC_Os09g12970	plant protein of unknown function domain containing protein, expressed
Chr9	7477628	7477628	G	A	G	SNP	OS09G0300800	Intronic	NA	NA	LOC_Os09g12970	plant protein of unknown function domain containing protein, expressed
Chr9	7479328	7479328	G	A	G	SNP	OS09G0300800	Intronic	NA	NA	LOC_Os09g12970	plant protein of unknown function domain containing protein, expressed
Chr9	7479385	7479385	G	A	G	SNP	OS09G0300800	Intronic	NA	NA	LOC_Os09g12970	plant protein of unknown function domain containing protein, expressed
Chr9	7479508	7479508	G	A	G	SNP	OS09G0300800	Intronic	NA	NA	LOC_Os09g12970	plant protein of unknown function domain containing protein, expressed
Chr9	9568278	9568278	C	A	C	SNP	OS09G0325700	Exonic	CDS	Missense	LOC_Os09g15670	protein phosphatase 2C, putative, expressed
Chr9	9568313	9568313	C	A	C	SNP	OS09G0325700	Exonic	CDS	Missense	LOC_Os09g15670	protein phosphatase 2C, putative, expressed
Chr9	9652443	9652443	G	A	G	SNP	OS09G0327300	Exonic	CDS	Silent	LOC_Os09g15810	bifunctional protein folD, putative, expressed

Chr9	9652470	9652470	C	A	C	SNP	OS09G0327300	Exonic	CDS	Silent	LOC_Os09g15810	bifunctional protein folD, putative, expressed
Chr9	9652752	9652752	G	A	G	SNP	OS09G0327300	Intronic	NA	NA	LOC_Os09g15810	bifunctional protein folD, putative, expressed
Chr9	9652844	9652844	T	A	T	SNP	OS09G0327300	Intronic	NA	NA	LOC_Os09g15810	bifunctional protein folD, putative, expressed
Chr9	9652887	9652887	T	A	T	SNP	OS09G0327300	Intronic	NA	NA	LOC_Os09g15810	bifunctional protein folD, putative, expressed
Chr9	9652923	9652923	C	A	C	SNP	OS09G0327300	Intronic	NA	NA	LOC_Os09g15810	bifunctional protein folD, putative, expressed
Chr9	9652929	9652929	T	A	T	SNP	OS09G0327300	Intronic	NA	NA	LOC_Os09g15810	bifunctional protein folD, putative, expressed
Chr9	9654458	9654458	T	A	T	SNP	OS09G0327300	Intronic	NA	NA	LOC_Os09g15810	bifunctional protein folD, putative, expressed
Chr9	9654487	9654487	G	A	G	SNP	OS09G0327300	Intronic	NA	NA	LOC_Os09g15810	bifunctional protein folD, putative, expressed
Chr9	10130306	10130306	C	A	C	SNP	OS09G0334500	Exonic	CDS	Missense	LOC_Os09g16510	WRKY74, expressed
Chr9	10252432	10252432	G	A	G	SNP	OS09G0337300	Exonic	CDS	NA	LOC_Os09g16760	ankyrin repeat family protein, putative, expressed
Chr9	10252451	10252451	G	A	G	SNP	OS09G0337300	Exonic	CDS	NA	LOC_Os09g16760	ankyrin repeat family protein, putative, expressed
Chr9	11806538	11806538	C	A	C	SNP	OS09G0361400	Exonic	5UTR	NA	LOC_Os09g19734	isochorismate synthase 1, chloroplast precursor, putative, expressed
Chr9	11806540	11806540	G	A	G	SNP	OS09G0361400	Exonic	5UTR	NA	LOC_Os09g19734	isochorismate synthase 1, chloroplast precursor, putative, expressed
Chr9	11806838	11806838	G	A	G	SNP	OS09G0361400	Exonic	5UTR	NA	LOC_Os09g19734	isochorismate synthase 1, chloroplast precursor, putative, expressed
Chr9	11956135	11956135	G	A	G	SNP	OS09G0364400	Exonic	CDS	Silent	LOC_Os09g19970	expressed protein
Chr9	11956688	11956688	C	A	C	SNP	OS09G0364400	Exonic	CDS	Missense	LOC_Os09g19970	expressed protein
Chr9	12782995	12782995	C	A	C	SNP	OS09G0379600	Exonic	CDS	Missense	LOC_Os09g21180	homeobox associated leucine zipper, putative, expressed
Chr9	12805033	12805033	C	A	C	SNP	OS09G0379900	Exonic	CDS	Missense	LOC_Os09g21210	beta-glucan-binding protein 4, putative, expressed
Chr9	12805040	12805040	C	A	C	SNP	OS09G0379900	Exonic	CDS	Silent	LOC_Os09g21210	beta-glucan-binding protein 4, putative, expressed
Chr9	12805111	12805111	G	A	G	SNP	OS09G0379900	Exonic	CDS	Missense	LOC_Os09g21210	beta-glucan-binding protein 4, putative, expressed
Chr9	12805220	12805220	G	A	G	SNP	OS09G0379900	Exonic	5UTR	NA	LOC_Os09g21210	beta-glucan-binding protein 4, putative, expressed
Chr9	13569553	13569553	G	A	G	SNP	OS09G0392400	Exonic	3UTR	NA	LOC_Os09g22500	expressed protein
Chr9	13569556	13569556	G	A	G	SNP	OS09G0392400	Exonic	3UTR	NA	LOC_Os09g22500	expressed protein
Chr9	13574130	13574130	T	A	T	SNP	OS09G0392400	Exonic	5UTR	NA	LOC_Os09g22500	expressed protein
Chr9	13575084	13575084	G	A	G	SNP	OS09G0392400	Exonic	CDS	NA	LOC_Os09g22500	expressed protein
Chr9	13781373	13781373	G	A	G	SNP	OS09G0395600	Exonic	3UTR	NA	LOC_Os09g23220	glycosyl hydrolases family 16, putative, expressed
Chr9	15927416	15927416	G	A	G	SNP	OS09G0433800	Exonic	CDS	Silent	LOC_Os09g26370	DUF581 domain containing protein, expressed
Chr9	15959801	15959801	T	A	T	SNP	OS09G0434500	Exonic	CDS	Missense	LOC_Os09g26420	AP2 domain containing protein, expressed
Chr9	15959818	15959818	G	A	G	SNP	OS09G0434500	Exonic	CDS	Missense	LOC_Os09g26420	AP2 domain containing protein, expressed
Chr9	16188652	16188652	G	A	G	SNP	OS09G0438000	Intronic	NA	NA	LOC_Os09g26660	ferric reductase, putative, expressed
Chr9	16188661	16188661	T	A	T	SNP	OS09G0438000	Intronic	NA	NA	LOC_Os09g26660	ferric reductase, putative, expressed
Chr9	16188988	16188988	G	A	G	SNP	OS09G0438000	Intronic	NA	NA	LOC_Os09g26660	ferric reductase, putative, expressed
Chr9	16189069	16189069	G	A	G	SNP	OS09G0438000	Intronic	NA	NA	LOC_Os09g26660	ferric reductase, putative, expressed
Chr9	16189633	16189633	G	A	G	SNP	OS09G0438000	Intronic	NA	NA	LOC_Os09g26660	ferric reductase, putative, expressed
Chr9	16189833	16189833	C	A	C	SNP	OS09G0438000	Intronic	NA	NA	LOC_Os09g26660	ferric reductase, putative, expressed
Chr9	16389645	16389645	C	A	C	SNP	OS09G0441400	Exonic	CDS	Missense	LOC_Os09g26960	cytochrome P450, putative, expressed
Chr9	16439192	16439192	T	A	T	SNP	OS09G0442300	Exonic	CDS	NA	LOC_Os09g27030	oryzain gamma chain precursor, putative, expressed
Chr9	16439514	16439514	T	A	T	SNP	OS09G0442300	Intronic	NA	NA	LOC_Os09g27030	oryzain gamma chain precursor, putative, expressed
Chr9	16445544	16445544	G	A	G	SNP	OS09G0442400	Exonic	CDS	Silent	LOC_Os09g27040	GEX1, putative, expressed
Chr9	16447376	16447376	G	A	G	SNP	OS09G0442400	Exonic	CDS	Missense	LOC_Os09g27040	GEX1, putative, expressed
Chr9	16447732	16447732	G	A	G	SNP	OS09G0442400	Exonic	CDS	Silent	LOC_Os09g27040	GEX1, putative, expressed
Chr9	16959063	16959063	G	A	G	SNP	OS09G0452200	Exonic	CDS	Missense	LOC_Os09g27890	lysM domain-containing GPI-anchored protein precursor, putative, expressed
Chr9	17082209	17082209	G	A	G	SNP	OS09G0454600	Exonic	CDS	Silent	LOC_Os09g28160	phosphate carrier protein, mitochondrial precursor, putative, expressed
Chr9	17449545	17449545	G	A	G	SNP	OS09G0461500	Exonic	5UTR	NA	LOC_Os09g28690	gibberellin receptor GID1L2, putative, expressed
Chr9	17498890	17498890	G	A	G	SNP	OS09G0462700	Exonic	CDS	NA	LOC_Os09g28810	RNA recognition motif containing protein, putative, expressed
Chr9	18034168	18034168	G	A	G	SNP	OS09G0472100	Exonic	5UTR	NA	LOC_Os09g29660	white-brown complex homolog protein 11, putative, expressed
Chr9	18039009	18039009	G	A	G	SNP	OS09G0472100	Exonic	CDS	Missense	LOC_Os09g29660	white-brown complex homolog protein 11, putative, expressed
Chr9	18319767	18319767	G	A	G	SNP	OS09G0478300	Intronic	NA	NA	LOC_Os09g30130	CSLE6 - cellulose synthase-like family E, expressed
Chr9	18320108	18320108	G	A	G	SNP	OS09G0478300	Intronic	NA	NA	LOC_Os09g30130	CSLE6 - cellulose synthase-like family E, expressed
Chr9	18320350	18320350	C	A	C	SNP	OS09G0478300	Intronic	NA	NA	LOC_Os09g30130	CSLE6 - cellulose synthase-like family E, expressed
Chr9	18320470	18320470	G	A	G	SNP	OS09G0478300	Intronic	NA	NA	LOC_Os09g30130	CSLE6 - cellulose synthase-like family E, expressed
Chr9	18321870	18321870	G	A	G	SNP	OS09G0478300	Exonic	5UTR	NA	LOC_Os09g30130	CSLE6 - cellulose synthase-like family E, expressed
Chr9	18321895	18321895	G	A	G	SNP	OS09G0478300	Exonic	5UTR	NA	LOC_Os09g30130	CSLE6 - cellulose synthase-like family E, expressed
Chr9	18322005	18322005	G	A	G	SNP	OS09G0478300	Exonic	5UTR	NA	LOC_Os09g30130	CSLE6 - cellulose synthase-like family E, expressed
Chr9	18322214	18322214	C	A	C	SNP	OS09G0478300	Exonic	5UTR	NA	LOC_Os09g30130	CSLE6 - cellulose synthase-like family E, expressed
Chr9	18322334	18322334	G	A	G	SNP	OS09G0478300	Exonic	5UTR	NA	LOC_Os09g30130	CSLE6 - cellulose synthase-like family E, expressed
Chr9	18352043	18352043	G	A	G	SNP	OS09G0478600	Exonic	CDS	NA	LOC_Os09g30160	zinc finger, C3HC4 type domain containing protein, expressed
Chr9	18441111	18441111	C	A	C	SNP	OS09G0480400	Exonic	5UTR	NA	LOC_Os09g30280	glycosyl transferase 8 domain containing protein, putative, expressed
Chr9	18669745	18669745	G	A	G	SNP	OS09G0483400	Intronic	NA	NA	LOC_Os09g31031	ubiquitin family protein, putative, expressed
Chr9	18669995	18669995	G	A	G	SNP	OS09G0483400	Intronic	NA	NA	LOC_Os09g31031	ubiquitin family protein, putative, expressed
Chr9	18708304	18708304	G	A	G	SNP	OS09G0484800	Exonic	CDS	Missense	LOC_Os09g31120	pirin, putative, expressed
Chr9	18708852	18708852	C	A	C	SNP	OS09G0484800	Intronic	NA	NA	LOC_Os09g31120	pirin, putative, expressed
Chr9	18709759	18709759	G	A	G	SNP	OS09G0484800	Exonic	CDS	Silent	LOC_Os09g31120	pirin, putative, expressed
Chr9	18716214	18716214	C	A	C	SNP	OS09G0484900	Exonic	CDS	Silent	LOC_Os09g31130	citrate transporter, putative, expressed
Chr9	18945015	18945015	G	A	G	SNP	OS09G0491596	Exonic	CDS	Silent	LOC_Os09g31446	expressed protein
Chr9	18991150	18991150	G	A	G	SNP	OS09G0491772	Exonic	5UTR	NA	LOC_Os09g31486	DnaK family protein, putative, expressed
Chr9	19012243	19012243	C	A	C	SNP	OS09G0491820	Intronic	NA	NA	LOC_Os09g31502	dehydrogenase, putative, expressed
Chr9	19585880	19585880	G	A	G	SNP	OS09G0506000	Exonic	CDS	Missense	LOC_Os09g32840	nucleotide pyrophosphatase/phosphodiesterase, putative, expressed
Chr9	20156876	20156876	G	A	G	SNP	OS09G0517000	Exonic	CDS	Silent	LOC_Os09g34140	expressed protein
Chr9	20494946	20494946	G	A	G	SNP	OS09G0524800	Exonic	3UTR	NA	LOC_Os09g35630	BAG domain-containing protein, putative, expressed
Chr9	20495943	20495943	C	A	C	SNP	OS09G0524800	Intronic	NA	NA	LOC_Os09g35630	BAG domain-containing protein, putative, expressed
Chr9	20495944	20495944	C	A	C	SNP	OS09G0524800	Intronic	NA	NA	LOC_Os09g35630	BAG domain-containing protein, putative, expressed
Chr9	20496599	20496599	G	A	G	SNP	OS09G0524800	Exonic	5UTR	NA	LOC_Os09g35630	BAG domain-containing protein, putative, expressed
Chr9	20496646	20496646	G	A	G	SNP	OS09G0524800	Exonic	5UTR	NA	LOC_Os09g35630	BAG domain-containing protein, putative, expressed
Chr9	20869261	20869261	G	A	G	SNP	OS09G0532000	Exonic	CDS	Silent	LOC_Os09g36200	senescence-inducible chloroplast stay-green protein 1, putative, expressed
Chr9	20869289	20869289	G	A	G	SNP	OS09G0532000	Exonic	CDS	Missense	LOC_Os09g36200	senescence-inducible chloroplast stay-green protein 1, putative, expressed
Chr9	20869739	20869739	G	A	G	SNP	OS09G0532000	Intronic	NA	NA	LOC_Os09g36200	senescence-inducible chloroplast stay-green protein 1, putative, expressed
Chr9	22432277	22432277	G	A	G	SNP	OS09G0564200	Intronic	NA	NA	LOC_Os09g39070	thiol protease SEN102 precursor, putative, expressed
Chr9	22432453	22432453	G	A	G	SNP	OS09G0564200	Intronic	NA	NA	LOC_Os09g39070	thiol protease SEN102 precursor, putative, expressed
Chr9	22883401	22883401	G	A	G	SNP	OS09G0572400	Exonic	3UTR	NA	LOC_Os09g39910	ABC transporter, ATP-binding protein, putative, expressed
Chr9	22906869	22906869	G	A	G	SNP	OS09G0572700	Exonic	5UTR	NA	LOC_Os09g39940	plastocyanin-like domain containing protein, putative, expressed
Chr9	22907208	22907208	G	A	G	SNP	OS09G0572700	Exonic	5UTR	NA	LOC_Os09g39940	plastocyanin-like domain containing protein, putative, expressed
Chr9	22907313	22907313	G	A	G	SNP	OS09G0572700	Exonic	5UTR	NA	LOC_Os09g39940	plastocyanin-like domain containing protein, putative, expressed
Chr9	22907344	22907344	C	A	C	SNP	OS09G0572700	Exonic	5UTR	NA	LOC_Os09g39940	plastocyanin-like domain containing protein, putative, expressed
Chr9	22907495	22907495	G	A	G	SNP	OS09G0572700	Exonic	5UTR	NA	LOC_Os09g39940	plastocyanin-like domain containing protein, putative, expressed

Chr9	22916704	22916704	C	A	C	SNP	OS09G0572900	Exonic	5UTR	NA	LOC_Os09g39960	dynamin family protein, putative, expressed
Chr9	22810855	22810873	ACTAGAGTATATTTTCTTC	A	ACTAGAGTATATTTTCTTC	INDEL	OS09G0571400	Exonic	3UTR	NA	LOC_Os09g39780	peptidyl-prolyl cis-trans isomerase, putative, expressed
Chr9	21858679	21858682	ATGT	A	ATGT	INDEL	OS09G0551600	Exonic	3UTR	NA	LOC_Os09g37910	HMG1/2, putative, expressed
Chr9	21226134	21226136	AAG	A	AAG	INDEL	OS09G0539100	Exonic	3UTR	NA	LOC_Os09g36800	3-dehydroquinate synthase, putative, expressed
Chr9	20367727	20367728	AT	A	AT	INDEL	OS09G0521400	Intronic	NA	NA	LOC_Os09g34960	hydroxymethylglutaryl-CoA synthase, putative, expressed
Chr9	18039185	18039186	AC	A	AC	INDEL	OS09G0472100	Exonic	3UTR	NA	LOC_Os09g29660	white-brown complex homolog protein 11, putative, expressed
Chr9	17392578	17392579	AG	A	AG	INDEL	OS09G0459900	Exonic	5UTR	NA	LOC_Os09g28580	expressed protein
Chr9	16439450	16439465	ACCGCCGCTGCCGCCG	A	ACCGCCGCTGCCGCCG	INDEL	OS09G0442300	Intronic	NA	NA	LOC_Os09g27030	oryzain gamma chain precursor, putative, expressed
Chr9	13574682	13574683	AC	A	AC	INDEL	OS09G0392400	Intronic	NA	NA	LOC_Os09g22500	expressed protein
Chr9	11806396	11806397	AG	A	AG	INDEL	OS09G0361400	Exonic	5UTR	NA	LOC_Os09g19734	isochorismate synthase 1, chloroplast precursor, putative, expressed
Chr9	11806508	11806511	AATT	A	AATT	INDEL	OS09G0361400	Exonic	5UTR	NA	LOC_Os09g19734	isochorismate synthase 1, chloroplast precursor, putative, expressed
Chr9	10338604	10338607	ACTC	A	ACTC	INDEL	OS09G0338500	Exonic	5UTR	NA	LOC_Os09g16920	desaturase/cytochrome b5 protein, putative, expressed
Chr9	10129723	10129724	AT	A	AT	INDEL	OS09G0334500	Intronic	NA	NA	LOC_Os09g16510	WRKY74, expressed
Chr9	9654356	9654364	ATTACTGGC	A	ATTACTGGC	INDEL	OS09G0327300	Intronic	NA	NA	LOC_Os09g15810	bifunctional protein folD, putative, expressed
Chr9	7476422	7476423	AC	A	AC	INDEL	OS09G0300800	Intronic	NA	NA	LOC_Os09g12970	plant protein of unknown function domain containing protein, expressed
Chr9	7281023	7281027	ACCCC	A	ACCCC	INDEL	OS09G0298500	Intronic	NA	NA	LOC_Os09g12720	zinc finger, C3HC4 type domain containing protein, expressed
Chr9	7281578	7281596	AATGTGGGAGGTGAAAAGG	A	AATGTGGGAGGTGAAAAGG	INDEL	OS09G0298500	Exonic	3UTR	NA	LOC_Os09g12720	zinc finger, C3HC4 type domain containing protein, expressed
Chr9	7199455	7199456	AT	A	AT	INDEL	OS09G0297000	Intronic	NA	NA	LOC_Os09g12560	ferrochelatase-2, chloroplast precursor, putative, expressed
Chr9	7204558	7204570	AGGTAGCTTAAGT	A	AGGTAGCTTAAGT	INDEL	OS09G0297000	Exonic	3UTR	NA	LOC_Os09g12560	ferrochelatase-2, chloroplast precursor, putative, expressed
Chr9	6388165	6388168	AAAG	A	AAAG	INDEL	OS09G0286600	Exonic	3UTR	NA	LOC_Os09g11460	AP2 domain containing protein, expressed
Chr.4	32385721	32385742	ACTAGGACACAATATATTAAAA	A,ACCTAGGACACAATATATTAAAA	ACTAGGACACAATATATTAAAA	INDEL	OS04G0636800	Intronic	NA	NA	LOC_Os04g54430	immediate-early protein RSP40, putative, expressed
Chr2	6812958	6812958	C	A,G	C	SNP	OS02G0222100	Intronic	NA	NA	LOC_Os02g12900	cysteine synthase, putative, expressed
Chr6	3041006	3041006	T	A,G	T	INDEL	OS06G0159450	Exonic	CDS	NA	LOC_Os06g06460	histone H3, putative, expressed
Chr1	32848834	32848834	G	A,T	G	INDEL	OS01G0776600	Intronic	NA	NA	LOC_Os01g56880	purple acid phosphatase precursor, putative, expressed
Chr11	27490040	27490040	C	A,T	C	SNP	OS11G0679700	Exonic	3UTR	NA	LOC_Os11g45400	glycerol-3-phosphate acyltransferase, putative, expressed
Chr3	35127865	35127865	C	A,T	C	SNP	OS03G0836000	Intronic	NA	NA	LOC_Os03g61970	actin, putative, expressed
Chr1	21922042	21922042	A	AAAAAT	A	INDEL	OS01G0571100	Exonic	3UTR	NA	LOC_Os01g39000	ZmGR2c, putative, expressed
Chr8	21171556	21171556	A	AAAAG	A	INDEL	OS08G0435900	Exonic	3UTR	NA	LOC_Os08g33820	chlorophyll A-B binding protein, putative, expressed
Chr9	7473934	7473934	A	AAAAGT	A	INDEL	OS09G0300800	Intronic	NA	NA	LOC_Os09g12970	plant protein of unknown function domain containing protein, expressed
Chr11	4108842	4108842	A	AAAATCAAACACCAGT	A	INDEL	OS11G0181900	Exonic	CDS	InFrame	LOC_Os11g07940	expressed protein
Chr.4	18213702	18213702	A	AAAATTAGT	A	INDEL	OS04G0373400	Intronic	NA	NA	LOC_Os04g30490	MATE efflux family protein, putative, expressed
Chr3	30853443	30853443	A	AAAC	A	INDEL	OS03G0749300	Exonic	5UTR	NA	LOC_Os03g53800	periplasmic beta-glucosidase precursor, putative, expressed
Chr1	39569875	39569875	A	AAACC	A	INDEL	OS01G0908700	Exonic	5UTR	NA	LOC_Os01g68060	copine, putative, expressed
Chr12	1514526	1514526	A	AAAG	A	INDEL	OS12G0131400	Exonic	3UTR	NA	LOC_Os12g03740	OsFBX438 - F-box domain containing protein, expressed
Chr2	1083689	1083689	A	AAAG	A	INDEL	OS02G0120800	Intronic	NA	NA	LOC_Os02g02840	ras-related protein, putative, expressed
Chr2	18575320	18575320	A	AAAT	A	INDEL	OS02G0515200	Exonic	3UTR	NA	LOC_Os02g31080	hydroxyproline-rich glycoprotein family protein, putative, expressed
Chr.4	31281265	31281265	A	AAATT	A	INDEL	OS04G0616300	Intronic	NA	NA	LOC_Os04g52600	SHR5-receptor-like kinase, putative, expressed
Chr1	21424139	21424139	A	AAATT	A	INDEL	OS01G0563000	Intronic	NA	NA	LOC_Os01g38229	peptidyl-prolyl isomerase, putative, expressed
Chr12	5676354	5676354	A	AAATT	A	INDEL	OS12G0208900	Exonic	CDS	FrameShift	LOC_Os12g10630	ZF-HD protein dimerisation region containing protein, expressed
Chr1	22496642	22496642	A	AAC	A	INDEL	OS01G0580800	Intronic	NA	NA	LOC_Os01g39890	transposon protein, putative, unclassified, expressed
Chr11	20182626	20182626	A	AAC	A	INDEL	OS11G0547000	Exonic	3UTR	NA	LOC_Os11g34460	OsFBO10 - F-box and other domain containing protein, expressed
Chr12	22593500	22593500	A	AAC	A	INDEL	OS12G0555200	Exonic	3UTR	NA	LOC_Os12g36850	pathogenesis-related Bet v I family protein, putative, expressed
Chr2	23993823	23993823	A	AAC	A	INDEL	OS02G0610600	Exonic	CDS	FrameShift	LOC_Os02g39720	splicing factor, arginine/serine-rich 7, putative, expressed
Chr9	21677485	21677485	A	AAC	A	INDEL	OS09G0548200	Exonic	5UTR	NA	LOC_Os09g37600	lysM domain-containing GPI-anchored protein precursor, putative, expressed
Chr3	190550	190550	A	AACCACTAT	A	INDEL	OS03G0102700	Intronic	NA	NA	LOC_Os03g01270	expansin precursor, putative, expressed
Chr.4	32386139	32386139	A	AACGAGC	A	INDEL	OS04G0636800	Intronic	NA	NA	LOC_Os04g54430	immediate-early protein RSP40, putative, expressed
Chr.4	5385869	5385869	A	AACGCATCC	A	INDEL	OS04G0179200	Exonic	3UTR	NA	LOC_Os04g10010	sex determination protein tasselseed-2, putative, expressed
Chr1	22497022	22497022	A	AACT	A	INDEL	OS01G0580800	Intronic	NA	NA	LOC_Os01g39890	transposon protein, putative, unclassified, expressed
Chr7	25037103	25037103	A	AACTGGGAGTGGAGTTGGTG	A	INDEL	OS07G0608700	Intronic	NA	NA	LOC_Os07g41790	LSM domain containing protein, expressed
Chr1	9671789	9671789	A	AAG	A	INDEL	OS01G0276200	Intronic	NA	NA	LOC_Os01g16910	outer mitochondrial membrane porin, putative, expressed
Chr1	1171112	1171112	A	AAG	A	INDEL	OS01G0120700	Exonic	5UTR	NA	LOC_Os01g03060	splicing factor, putative, expressed
Chr12	19184838	19184838	A	AAG	A	INDEL	OS12G0503000	Exonic	5UTR	NA	LOC_Os12g31860	ureide permease, putative, expressed
Chr12	22534452	22534452	A	AAG	A	INDEL	OS12G0554500	Intronic	NA	NA	LOC_Os12g36770	lipase class 3 family protein, putative, expressed
Chr2	31624431	31624431	A	AAG	A	INDEL	OS02G0752300	Intronic	NA	NA	LOC_Os02g51640	expressed protein
Chr7	18762868	18762868	A	AAG	A	INDEL	OS07G0499300	Intronic	NA	NA	LOC_Os07g31599	expressed protein
Chr8	18324410	18324410	A	AAG	A	INDEL	OS08G0387700	Intronic	NA	NA	LOC_Os08g29809	resistance protein LR10, putative, expressed
Chr.4	32167113	32167113	A	AAGC	A	INDEL	OS04G0631600	Exonic	CDS	InFrame	LOC_Os04g53990	ethylene-responsive protein related, putative, expressed
Chr.4	32167611	32167611	A	AAGC	A	INDEL	OS04G0631600	Exonic	CDS	InFrame	LOC_Os04g53990	ethylene-responsive protein related, putative, expressed
Chr1	41583629	41583629	A	AAGCAGC	A	INDEL	OS01G0946100	Exonic	CDS	InFrame	LOC_Os01g71780	WD domain, G-beta repeat domain containing protein, expressed
Chr1	43142106	43142106	A	AAGCAGC	A	INDEL	OS01G0976300	Exonic	CDS	InFrame	LOC_Os01g74490	heavy metal-associated domain containing protein, expressed
Chr.5	26171233	26171233	A	AAT	A	INDEL	OS05G0525900	Exonic	3UTR	NA	LOC_Os05g45020	zinc finger/CCCH transcription factor, putative, expressed
Chr1	15572400	15572400	A	AAT	A	INDEL	OS01G0376700	Intronic	NA	NA	LOC_Os01g27880	sucrose-phosphatase, putative, expressed
Chr1	42840380	42840380	A	AAT	A	INDEL	OS01G0970900	Intronic	NA	NA	LOC_Os01g73950	PPR repeat containing protein, expressed
Chr11	20519146	20519146	A	AAT	A	INDEL	OS11G0551800	Intronic	NA	NA	LOC_Os11g35020	yippee zinc-binding protein, putative, expressed
Chr11	28933381	28933381	A	AAT	A	INDEL	OS11G0707000	Intronic	NA	NA	LOC_Os11g47970	AAA-type ATPase family protein, putative, expressed
Chr12	19183027	19183027	A	AAT	A	INDEL	OS12G0503000	Exonic	3UTR	NA	LOC_Os12g31860	ureide permease, putative, expressed
Chr2	8728147	8728147	A	AAT	A	INDEL	OS02G0254700	Intronic	NA	NA	LOC_Os02g15550	spermidine synthase, putative, expressed
Chr2	35094064	35094064	A	AAT	A	INDEL	OS02G0817900	Intronic	NA	NA	LOC_Os02g57290	cytochrome P450, putative, expressed
Chr3	35044029	35044029	A	AAT	A	INDEL	OS03G0833900	Intronic	NA	NA	LOC_Os03g61810	tRNA-specific adenosine deaminase, putative, expressed
Chr6	26306542	26306542	A	AAT	A	INDEL	OS06G0644600	Intronic	NA	NA	LOC_Os06g43690	WD repeat-containing protein, putative, expressed
Chr8	5243505	5243505	A	AAT	A	INDEL	OS08G0189600	Exonic	3UTR	NA	LOC_Os08g09010	Cupin domain containing protein, expressed
Chr9	20367992	20367992	A	AAT	A	INDEL	OS09G0521400	Intronic	NA	NA	LOC_Os09g34960	hydroxymethylglutaryl-CoA synthase, putative, expressed
Chr8	692445	692445	A	AATAG	A	INDEL	OS08G0112900	Intronic	NA	NA	LOC_Os08g02094	GDSL-like lipase/acylhydrolase, putative, expressed
Chr9	7872580	7872580	A	AATATAT	A	INDEL	OS09G0306400	Intronic	NA	NA	LOC_Os09g13570	CPuORF2 - conserved peptide uORF-containing transcript, expressed
Chr9	12783231	12783231	A	AATATAT	A	INDEL	OS09G0379600	Exonic	5UTR	NA	LOC_Os09g21180	homeobox associated leucine zipper, putative, expressed
Chr.4	621876	621876	A	AATCATCCATATCAAAACTC	A	INDEL	OS04G0110600	Exonic	3UTR	NA	LOC_Os04g02000	zinc finger family protein, putative, expressed
Chr12	22133980	22133980	A	AATT	A	INDEL	OS12G0547600	Intronic	NA	NA	LOC_Os12g36110	calmodulin binding protein, putative, expressed
Chr3	35027585	35027585	A	AATT	A	INDEL	OS03G0833600	Intronic	NA	NA	LOC_Os03g61780	glucan endo-1,3-beta-glucosidase-related, putative, expressed
Chr7	351228	351228	A	AATTTCCT	A	INDEL	OS07G0106200	Intronic	NA	NA	LOC_Os07g01560	transporter family protein, putative, expressed
Chr2	10741958	10741958	A	AATTTT	A	INDEL	OS02G0285800	Intronic	NA	NA	LOC_Os02g18450	GTP-binding protein typA/bipA, putative, expressed
Chr.4	18213671	18213671	A	AATTTTGGT	A	INDEL	OS04G0373400	Intronic	NA	NA	LOC_Os04g30490	MATE efflux family protein, putative, expressed
Chr.5	13423560	13423560	A	AC	A	INDEL	OS05G0299700	Exonic	5UTR	NA	LOC_Os05g23470	AN1-like zinc finger domain containing protein, expressed
Chr1	33618591	33618591	A	AC	A	INDEL	OS01G0793800	Exonic	3UTR	NA	LOC_Os01g58130	expressed protein
Chr11	10140212	10140212	A	AC	A	INDEL	OS11G0283500	Intronic	NA	NA	LOC_Os11g18044	peptide transporter PTR2, putative, expressed
Chr11	4974355	4974355	A	AC	A	INDEL	OS11G0199200	Intronic	NA	NA	LOC_Os11g09280	OsPDIL1-1 protein disulfide isomerase PDIL1-1, expressed

Chr12	19184657	19184657	A	AC	A	INDEL	OS12G0503000	Exonic	5UTR	NA	LOC_Os12g31860	ureide permease, putative, expressed
Chr2	14953390	14953390	A	AC	A	INDEL	OS02G0452500	Intronic	NA	NA	LOC_Os02g25580	Sec1 family transport protein, putative, expressed
Chr2	19104641	19104641	A	AC	A	INDEL	OS02G0523500	Intronic	NA	NA	LOC_Os02g32350	TUDOR protein with multiple SNc domains, putative, expressed
Chr3	28243633	28243633	A	AC	A	INDEL	OS03G0703000	Intronic	NA	NA	LOC_Os03g49600	Os3bglu7 - beta-glucosidase, exo-beta-glucanse, expressed
Chr3	29564867	29564867	A	AC	A	INDEL	OS03G0726500	Intronic	NA	NA	LOC_Os03g51650	membrane protein, putative, expressed
Chr3	32626988	32626988	A	AC	A	INDEL	OS03G0786000	Intronic	NA	NA	LOC_Os03g57210	glycine-rich protein, putative, expressed
Chr6	8848663	8848663	A	AC	A	INDEL	OS06G0266800	Exonic	5UTR	NA	LOC_Os06g15620	GASR7 - Gibberellin-regulated GASA/GAST/Snakin family protein precursor, expressed
Chr8	5983722	5983722	A	AC	A	INDEL	OS08G0203100	Intronic	NA	NA	LOC_Os08g10290	SHR5-receptor-like kinase, putative, expressed
Chr9	13574079	13574079	A	AC	A	INDEL	OS09G0392400	Exonic	5UTR	NA	LOC_Os09g22500	expressed protein
Chr7	3663178	3663178	A	ACAAAG	A	INDEL	OS07G0168300	Exonic	3UTR	NA	LOC_Os07g07320	glutathione S-transferase, putative, expressed
Chr.5	1002436	1002436	A	ACAAAT	A	INDEL	OS05G0119300	Exonic	5UTR	NA	LOC_Os05g02780	glycine-rich protein A3, putative, expressed
Chr7	13354472	13354472	A	ACAACC	A	INDEL	OS07G0418600	Exonic	3UTR	NA	LOC_Os07g23640	retrotransposon protein, putative, Ty3-gypsy subclass, expressed
Chr9	14030641	14030641	A	ACAAGAGAT	A	INDEL	OS09G0401000	Exonic	3UTR	NA	LOC_Os09g23620	MYB family transcription factor, putative, expressed
Chr.5	2028963	2028963	A	ACAAT	A	INDEL	OS05G0134700	Intronic	NA	NA	LOC_Os05g04410	peroxidase precursor, putative, expressed
Chr7	24985441	24985441	A	ACAC	A	INDEL	OS07G0607800	Exonic	CDS	InFrame	LOC_Os07g41694	acidic leucine-rich nuclear phosphoprotein 32-related protein 1, putative, expressed
Chr2	842534	842534	A	ACACCAC	A	INDEL	OS02G0115900	Exonic	5UTR	NA	LOC_Os02g02410	DnaK family protein, putative, expressed
Chr12	26007788	26007788	A	ACACCCC	A	INDEL	OS12G0613700	Exonic	5UTR	NA	LOC_Os12g41950	auxin response factor, putative, expressed
Chr7	24988608	24988608	A	ACC	A	INDEL	OS07G0607800	Intronic	NA	NA	LOC_Os07g41694	acidic leucine-rich nuclear phosphoprotein 32-related protein 1, putative, expressed
Chr.4	27372682	27372682	A	ACCGCCG	A	INDEL	OS04G0546800	Exonic	CDS	InFrame	LOC_Os04g46220	ethylene-responsive transcription factor, putative, expressed
Chr12	19695831	19695831	A	ACCT	A	INDEL	OS12G0511200	Exonic	CDS	InFrame	LOC_Os12g32640	haemolysin-III, putative, expressed
Chr1	5875874	5875874	A	ACG	A	INDEL	OS01G0208400	Exonic	3UTR	NA	LOC_Os01g11010	peptide-N4-asparagine amidase A, putative, expressed
Chr3	11327505	11327505	A	ACG	A	INDEL	OS03G0315400	Intronic	NA	NA	LOC_Os03g20090	MYB family transcription factor, putative, expressed
Chr9	21229309	21229309	A	ACGACGG	A	INDEL	OS09G0539100	Exonic	5UTR	NA	LOC_Os09g36800	3-dehydroquinate synthase, putative, expressed
Chr3	32852520	32852520	A	ACGC	A	INDEL	OS03G0790500	Exonic	CDS	InFrame	LOC_Os03g57640	gibberellin receptor GID1L2, putative, expressed
Chr7	21589844	21589844	A	ACGC	A	INDEL	OS07G0545300	Exonic	5UTR	NA	LOC_Os07g36130	core histone H2A/H2B/H3/H4, putative, expressed
Chr7	19235795	19235795	A	ACGC	A	INDEL	OS07G0506700	Intronic	NA	NA	LOC_Os07g32350	WD domain, G-beta repeat domain containing protein, expressed
Chr9	21276543	21276543	A	ACGCCGGCGAGCCCACCAC	A	INDEL	OS09G0540600	Exonic	CDS	InFrame	LOC_Os09g36900	WD domain, G-beta repeat domain containing protein, expressed
Chr1	3918143	3918143	A	ACGG	A	INDEL	OS01G0176000	Exonic	CDS	InFrame	LOC_Os01g08090	flavonol-3-O-glycoside-7-O-glucosyltransferase 1, putative, expressed
Chr2	26143568	26143568	A	ACGG	A	INDEL	OS02G0649300	Exonic	CDS	InFrame	LOC_Os02g43330	homeobox associated leucine zipper, putative, expressed
Chr9	13781853	13781853	A	ACGG	A	INDEL	OS09G0395600	Intronic	NA	NA	LOC_Os09g23220	glycosyl hydrolases family 16, putative, expressed
Chr2	29604540	29604540	A	ACT	A	INDEL	OS02G0714200	Exonic	3UTR	NA	LOC_Os02g48360	pyrophosphate--fructose 6-phosphate 1-phosphotransferase subunit alpha, putative, expressed
Chr3	12793227	12793227	A	ACT	A	INDEL	OS03G0343300	Exonic	3UTR	NA	LOC_Os03g22320	utp14 protein, putative, expressed
Chr1	31454849	31454849	A	ACTACC	A	INDEL	OS01G0750500	Intronic	NA	NA	LOC_Os01g54670	coiled-coil domain-containing protein 25, putative, expressed
Chr2	34635949	34635949	A	ACTAGTACTC	A	INDEL	OS02G0810200	Intronic	NA	NA	LOC_Os02g56540	kinesin motor domain containing protein, putative, expressed
Chr11	28935864	28935864	A	ACTATTACATATCC	A	INDEL	OS11G0707000	Intronic	NA	NA	LOC_Os11g47970	AAA-type ATPase family protein, putative, expressed
Chr11	27488176	27488176	A	ACTGAT	A	INDEL	OS11G0679700	Intronic	NA	NA	LOC_Os11g45400	glycerol-3-phosphate acyltransferase, putative, expressed
Chr.4	12426921	12426921	A	AG	A	INDEL	OS04G0287400	Exonic	3UTR	NA	LOC_Os04g21950	WRKY51, expressed
Chr.4	30615705	30615705	A	AG	A	INDEL	OS04G0606200	Exonic	3UTR	NA	LOC_Os04g51680	expressed protein
Chr.5	21492829	21492829	A	AG	A	INDEL	OS05G0438500	Intronic	NA	NA	LOC_Os05g36260	soluble inorganic pyrophosphatase, putative, expressed
Chr1	33840907	33840907	A	AG	A	INDEL	OS01G0799500	Exonic	CDS	FrameShift	LOC_Os01g58550	methyladenine glycosylase, putative, expressed
Chr1	33840939	33840939	A	AG	A	INDEL	OS01G0799500	Exonic	CDS	FrameShift	LOC_Os01g58550	methyladenine glycosylase, putative, expressed
Chr1	5901663	5901663	A	AG	A	INDEL	OS01G0208700	Intronic	NA	NA	LOC_Os01g11054	phosphoenolpyruvate carboxylase, putative, expressed
Chr11	22281548	22281548	A	AG	A	INDEL	OS11G0587600	Intronic	NA	NA	LOC_Os11g37700	pleiotropic drug resistance protein, putative, expressed
Chr2	23996156	23996156	A	AG	A	INDEL	OS02G0610600	Intronic	NA	NA	LOC_Os02g39720	splicing factor, arginine/serine-rich 7, putative, expressed
Chr3	24470806	24470806	A	AG	A	INDEL	OS03G0638400	Intronic	NA	NA	LOC_Os03g43750	retrotransposon, putative, centromere-specific, expressed
Chr3	29564707	29564707	A	AG	A	INDEL	OS03G0726500	Intronic	NA	NA	LOC_Os03g51650	membrane protein, putative, expressed
Chr3	12224679	12224679	A	AG	A	SNP	OS03G0331700	Exonic	3UTR	NA	LOC_Os03g21380	OsCML27 - Calmodulin-related calcium sensor protein, expressed
Chr6	25836041	25836041	A	AG	A	INDEL	OS06G0636100	Intronic	NA	NA	LOC_Os06g43000	nitrate-induced NOI protein, expressed
Chr6	4323990	4323990	A	AG	A	INDEL	OS06G0185900	Intronic	NA	NA	LOC_Os06g08670	glutathione peroxidase, putative, expressed
Chr6	307095	307095	A	AG	A	INDEL	OS06G0104400	Intronic	NA	NA	LOC_Os06g01500	IQ calmodulin-binding motif family protein, putative, expressed
Chr6	9284094	9284094	A	AG	A	INDEL	OS06G0274200	Intronic	NA	NA	LOC_Os06g16290	ribosomal protein L7Ae, putative, expressed
Chr7	21380393	21380393	A	AG	A	INDEL	OS07G0541400	Intronic	NA	NA	LOC_Os07g35690	TKL_IRAK_DUF26-lc.3 - DUF26 kinases have homology to DUF26 containing loci, expressed
Chr8	17511289	17511289	A	AG	A	INDEL	OS08G0374000	Intronic	NA	NA	LOC_Os08g28670	pathogenesis-related Bet v I family protein, putative, expressed
Chr9	19967282	19967282	A	AG	A	INDEL	OS09G0513100	Intronic	NA	NA	LOC_Os09g33820	lecithine cholesterol acyltransferase, putative, expressed
Chr9	9652517	9652517	A	AG	A	INDEL	OS09G0327300	Intronic	NA	NA	LOC_Os09g15810	bifunctional protein folD, putative, expressed
Chr7	26776150	26776150	A	AGAAAAACTACATTCAAG	A	INDEL	OS07G0642900	Intronic	NA	NA	LOC_Os07g44840	bacterial transferase hexapeptide domain containing protein, expressed
Chr6	26647710	26647710	A	AGAC	A	INDEL	OS06G0650900	Exonic	5UTR	NA	LOC_Os06g44160	heat shock protein DnaJ, putative, expressed
Chr11	20182667	20182667	A	AGAG	A	INDEL	OS11G0547000	Exonic	3UTR	NA	LOC_Os11g34460	OsFBO10 - F-box and other domain containing protein, expressed
Chr6	23737213	23737213	A	AGAG	A	INDEL	OS06G0601100	Exonic	CDS	InFrame	LOC_Os06g39912	expressed protein
Chr9	14531161	14531161	A	AGAG	A	INDEL	OS09G0409950	Exonic	5UTR	NA	LOC_Os09g24440	expressed protein
Chr10	22831345	22831345	A	AGAGGAG	A	INDEL	OS10G0574700	Exonic	5UTR	NA	LOC_Os10g42410	zinc-binding protein, putative, expressed
Chr9	18707936	18707936	A	AGCAGCAGCG	A	INDEL	OS09G0484800	Exonic	5UTR	NA	LOC_Os09g31120	pirin, putative, expressed
Chr1	29960842	29960842	A	AGCC	A	INDEL	OS01G0719000	Exonic	5UTR	NA	LOC_Os01g52100	DUF581 domain containing protein, expressed
Chr11	4974910	4974910	A	AGCC	A	INDEL	OS11G0199200	Exonic	CDS	InFrame	LOC_Os11g09280	OsPDIL1-1 protein disulfide isomerase PDIL1-1, expressed
Chr12	5070977	5070977	A	AGCG	A	INDEL	OS12G0198000	Exonic	CDS	InFrame	LOC_Os12g09620	expressed protein
Chr1	34835035	34835035	A	AGCT	A	INDEL	OS01G0818000	Intronic	NA	NA	LOC_Os01g60230	auxin efflux carrier component, putative, expressed
Chr10	21759841	21759841	A	AGCTACC	A	INDEL	OS10G0554200	Intronic	NA	NA	LOC_Os10g40600	peptide transporter PTR2, putative, expressed
Chr9	18710059	18710059	A	AGG	A	INDEL	OS09G0484800	Exonic	3UTR	NA	LOC_Os09g31120	pirin, putative, expressed
Chr2	34673720	34673720	A	AGGAGCCGCCGCCGCC	A	INDEL	OS02G0810900	Exonic	CDS	InFrame	LOC_Os02g56600	no apical meristem protein, putative, expressed
Chr3	35044567	35044567	A	AGGAGCGGAGC	A	INDEL	OS03G0833900	Exonic	5UTR	NA	LOC_Os03g61810	tRNA-specific adenosine deaminase, putative, expressed
Chr8	24855047	24855047	A	AGGC	A	INDEL	OS08G0503000	Exonic	CDS	InFrame	LOC_Os08g39330	skin secretory protein xP2 precursor, putative, expressed
Chr9	21367031	21367031	A	AGGGAGC	A	INDEL	OS09G0542500	Exonic	CDS	InFrame	LOC_Os09g37040	DUF630/DUF632 domains containing protein, putative, expressed
Chr1	23911733	23911733	A	AGGGGAG	A	INDEL	OS01G0606900	Exonic	CDS	InFrame	LOC_Os01g42190	heat shock protein DnaJ, putative, expressed
Chr2	20811858	20811858	A	AGT	A	INDEL	OS02G0552000	Intronic	NA	NA	LOC_Os02g34690	transmembrane 9 superfamily member, putative, expressed
Chr6	23228339	23228339	A	AGT	A	INDEL	OS06G0591200	Exonic	3UTR	NA	LOC_Os06g39110	expressed protein
Chr1	29896475	29896475	A	AGTATTCTCCTG	A	INDEL	OS01G0717700	Intronic	NA	NA	LOC_Os01g52010	alliin lyase precursor, putative, expressed
Chr2	5157937	5157937	A	AGTGTGTTGGT	A	INDEL	OS02G0192500	Intronic	NA	NA	LOC_Os02g09930	CSLA1 - cellulose synthase-like family A; mannan synthase, expressed
Chr9	21190709	21190709	A	AGTT	A	INDEL	OS09G0538400	Exonic	5UTR	NA	LOC_Os09g36730	MYB family transcription factor, putative, expressed
Chr.4	13650540	13650540	A	AT	A	INDEL	OS04G0304200	Intronic	NA	NA	LOC_Os04g23890	AGC_PVPK_like_kin82y.10 - ACG kinases include homologs to PKA, PKG and PKC, expressed
Chr.4	12426806	12426806	A	AT	A	INDEL	OS04G0287400	Exonic	3UTR	NA	LOC_Os04g21950	WRKY51, expressed
Chr.4	12426843	12426843	A	AT	A	INDEL	OS04G0287400	Exonic	3UTR	NA	LOC_Os04g21950	WRKY51, expressed
Chr.5	4137836	4137836	A	AT	A	INDEL	OS05G0169000	Exonic	3UTR	NA	LOC_Os05g07690	thioredoxin, putative, expressed
Chr.5	4142468	4142468	A	AT	A	INDEL	OS05G0169100	Intronic	NA	NA	LOC_Os05g07700	ribosomal protein, putative, expressed
Chr.5	24781408	24781408	A	AT	A	INDEL	OS05G0503300	Intronic	NA	NA	LOC_Os05g42350	ferredoxin--nitrite reductase, putative, expressed
Chr.5	836742	836742	A	AT	A	INDEL	OS05G0115100	Intronic	NA	NA	LOC_Os05g02450	expressed protein

Chr.5	27166221	27166221	A	AT	A	INDEL	OS05G0547600	Intronic	NA	NA	LOC_Os05g46950	expressed protein
Chr1	2275726	2275726	A	AT	A	INDEL	OS01G0142300	Intronic	NA	NA	LOC_Os01g04920	glycosyl transferase, group 1 domain containing protein, expressed
Chr1	8295257	8295257	A	AT	A	INDEL	OS01G0251100	Intronic	NA	NA	LOC_Os01g14830	ribosomal protein L3, putative, expressed
Chr1	8428954	8428954	A	AT	A	INDEL	OS01G0254100	Intronic	NA	NA	LOC_Os01g15020	lissencephaly type-1-like homology motif, putative, expressed
Chr1	21631497	21631497	A	AT	A	INDEL	OS01G0565900	Exonic	5UTR	NA	LOC_Os01g38510	protein transport protein Sec61 subunit beta, putative, expressed
Chr1	26895100	26895100	A	AT	A	INDEL	OS01G0660300	Intronic	NA	NA	LOC_Os01g47080	pyruvate kinase, putative, expressed
Chr1	34448201	34448201	A	AT	A	INDEL	OS01G0810800	Intronic	NA	NA	LOC_Os01g59570	senescence-induced receptor-like serine/threonine-protein kinase precursor, putative, expressed
Chr1	37048283	37048283	A	AT	A	INDEL	OS01G0857400	Intronic	NA	NA	LOC_Os01g63854	transmembrane amino acid transporter protein, putative, expressed
Chr1	37521197	37521197	A	AT	A	INDEL	OS01G0866400	Intronic	NA	NA	LOC_Os01g64660	fructose-1,6-bisphosphatase, putative, expressed
Chr1	42063418	42063418	A	AT	A	INDEL	OS01G0955000	Exonic	3UTR	NA	LOC_Os01g72520	phosphoesterase family protein, putative, expressed
Chr1	10200734	10200734	A	AT	A	INDEL	OS01G0284900	Exonic	3UTR	NA	LOC_Os01g18220	expressed protein
Chr1	29895937	29895937	A	AT	A	INDEL	OS01G0717700	Intronic	NA	NA	LOC_Os01g52010	alliin lyase precursor, putative, expressed
Chr1	29835057	29835057	A	AT	A	INDEL	OS01G0716800	Intronic	NA	NA	LOC_Os01g51890	endonuclease/exonuclease/phosphatase family domain containing protein, expressed
Chr1	33412298	33412298	A	AT	A	INDEL	OS01G0787600	Exonic	5UTR	NA	LOC_Os01g57770	OsPOP4 - Putative Prolyl Oligopeptidase homologue, expressed
Chr1	33899495	33899495	A	AT	A	INDEL	OS01G0800500	Intronic	NA	NA	LOC_Os01g58640	nucleotide pyrophosphatase/phosphodiesterase, putative, expressed
Chr1	42064889	42064889	A	AT	A	INDEL	OS01G0955000	Intronic	NA	NA	LOC_Os01g72520	phosphoesterase family protein, putative, expressed
Chr10	21206670	21206670	A	AT	A	INDEL	OS10G0542900	Intronic	NA	NA	LOC_Os10g39680	CHIT14 - Chitinase family protein precursor, expressed
Chr10	19439213	19439213	A	AT	A	INDEL	OS10G0507800	Intronic	NA	NA	LOC_Os10g36370	heat shock protein DnaJ, putative, expressed
Chr10	14749400	14749400	A	AT	A	INDEL	OS10G0419300	Intronic	NA	NA	LOC_Os10g28340	heat stress transcription factor, putative, expressed
Chr10	2359109	2359109	A	AT	A	INDEL	OS10G0138100	Intronic	NA	NA	LOC_Os10g04860	aldehyde oxidase, putative, expressed
Chr10	18535975	18535975	A	AT	A	INDEL	OS10G0488800	Intronic	NA	NA	LOC_Os10g34710	myosin-2 heavy chain, non muscle, putative, expressed
Chr11	4972720	4972720	A	AT	A	INDEL	OS11G0199200	Intronic	NA	NA	LOC_Os11g09280	OsPDIL1-1 protein disulfide isomerase PDIL1-1, expressed
Chr11	4972490	4972490	A	AT	A	INDEL	OS11G0199200	Intronic	NA	NA	LOC_Os11g09280	OsPDIL1-1 protein disulfide isomerase PDIL1-1, expressed
Chr11	19677803	19677803	A	AT	A	INDEL	OS11G0539200	Exonic	3UTR	NA	LOC_Os11g33270	glycosyl hydrolases family 16, putative, expressed
Chr12	3755209	3755209	A	AT	A	INDEL	OS12G0174100	Intronic	NA	NA	LOC_Os12g07540	growth regulator related protein, putative, expressed
Chr12	19185191	19185191	A	AT	A	INDEL	OS12G0503000	Exonic	5UTR	NA	LOC_Os12g31860	ureide permease, putative, expressed
Chr12	4507583	4507583	A	AT	A	INDEL	OS12G0189400	Exonic	3UTR	NA	LOC_Os12g08770	photosystem I reaction center subunit N, chloroplast precursor, putative, expressed
Chr12	22629596	22629596	A	AT	A	INDEL	OS12G0556200	Intronic	NA	NA	LOC_Os12g36910	calmodulin binding protein, putative, expressed
Chr12	24185174	24185174	A	AT	A	INDEL	OS12G0582700	Intronic	NA	NA	LOC_Os12g39310	cytochrome P450, putative, expressed
Chr12	9920218	9920218	A	AT	A	INDEL	OS12G0271700	Intronic	NA	NA	LOC_Os12g17320	polyprenyl synthetase, putative, expressed
Chr12	19182797	19182797	A	AT	A	INDEL	OS12G0503000	Exonic	3UTR	NA	LOC_Os12g31860	ureide permease, putative, expressed
Chr2	4451369	4451369	A	AT	A	INDEL	OS02G0180000	Intronic	NA	NA	LOC_Os02g08364	protein phosphatase 2C, putative, expressed
Chr2	6717743	6717743	A	AT	A	INDEL	OS02G0220500	Intronic	NA	NA	LOC_Os02g12794	elongation factor 1-gamma, putative, expressed
Chr2	9974981	9974981	A	AT	A	INDEL	OS02G0273700	Intronic	NA	NA	LOC_Os02g17350	VHS and GAT domain containing protein, expressed
Chr2	9977783	9977783	A	AT	A	INDEL	OS02G0273700	Exonic	3UTR	NA	LOC_Os02g17350	VHS and GAT domain containing protein, expressed
Chr2	14951446	14951446	A	AT	A	INDEL	OS02G0452500	Intronic	NA	NA	LOC_Os02g25580	Sec1 family transport protein, putative, expressed
Chr2	31665712	31665712	A	AT	A	INDEL	OS02G0753000	Exonic	3UTR	NA	LOC_Os02g51680	uncharacterized glycosyl hydrolase Rv2006/MT2062, putative, expressed
Chr2	28750095	28750095	A	AT	A	INDEL	OS02G0699000	Intronic	NA	NA	LOC_Os02g47090	peptide transporter PTR2, putative, expressed
Chr2	5158385	5158385	A	AT	A	INDEL	OS02G0192500	Intronic	NA	NA	LOC_Os02g09930	CSLA1 - cellulose synthase-like family A; mannan synthase, expressed
Chr3	3105049	3105049	A	AT	A	INDEL	OS03G0157800	Intronic	NA	NA	LOC_Os03g06190	3-5 exonuclease family protein, putative, expressed
Chr3	22948371	22948371	A	AT	A	INDEL	OS03G0609000	Exonic	5UTR	NA	LOC_Os03g41280	expressed protein
Chr3	27462229	27462229	A	AT	A	INDEL	OS03G0688200	Intronic	NA	NA	LOC_Os03g48260	expressed protein
Chr3	35042704	35042704	A	AT	A	INDEL	OS03G0833900	Exonic	3UTR	NA	LOC_Os03g61810	tRNA-specific adenosine deaminase, putative, expressed
Chr3	10772576	10772576	A	AT	A	INDEL	OS03G0304500	Exonic	3UTR	NA	LOC_Os03g19200	DNAJ heat shock N-terminal domain-containing protein, putative, expressed
Chr3	10906123	10906123	A	AT	A	INDEL	OS03G0306700	Exonic	5UTR	NA	LOC_Os03g19370	CPuORF4 - conserved peptide uORF-containing transcript, expressed
Chr3	9499785	9499785	A	AT	A	INDEL	OS03G0279200	Exonic	5UTR	NA	LOC_Os03g17100	Core histone H2A/H2B/H3/H4 domain containing protein, putative, expressed
Chr6	25925749	25925749	A	AT	A	INDEL	OS06G0638000	Intronic	NA	NA	LOC_Os06g43120	zinc finger C-x8-C-x5-C-x3-H type family protein, expressed
Chr6	9284049	9284049	A	AT	A	INDEL	OS06G0274200	Intronic	NA	NA	LOC_Os06g16290	ribosomal protein L7Ae, putative, expressed
Chr6	9284496	9284496	A	AT	A	INDEL	OS06G0274200	Intronic	NA	NA	LOC_Os06g16290	ribosomal protein L7Ae, putative, expressed
Chr6	2952249	2952249	A	AT	A	INDEL	OS06G0158000	Intronic	NA	NA	LOC_Os06g06350	AMP-binding enzyme, putative, expressed
Chr7	27554913	27554913	A	AT	A	INDEL	OS07G0655400	Exonic	3UTR	NA	LOC_Os07g46170	OsRhmbd13 - Putative Rhomboid homologue, expressed
Chr7	25038077	25038077	A	AT	A	INDEL	OS07G0608700	Intronic	NA	NA	LOC_Os07g41790	LSM domain containing protein, expressed
Chr7	2195058	2195058	A	AT	A	INDEL	OS07G0142900	Intronic	NA	NA	LOC_Os07g04990	oxidoreductase, aldo/keto reductase family protein, putative, expressed
Chr7	559379	559379	A	AT	A	INDEL	OS07G0110200	Exonic	3UTR	NA	LOC_Os07g01920	nucleolar GTP-binding protein 1, putative, expressed
Chr7	24986118	24986118	A	AT	A	INDEL	OS07G0607800	Intronic	NA	NA	LOC_Os07g41694	acidic leucine-rich nuclear phosphoprotein 32-related protein 1, putative, expressed
Chr7	24986372	24986372	A	AT	A	INDEL	OS07G0607800	Intronic	NA	NA	LOC_Os07g41694	acidic leucine-rich nuclear phosphoprotein 32-related protein 1, putative, expressed
Chr8	3281332	3281332	A	AT	A	INDEL	OS08G0156600	Intronic	NA	NA	LOC_Os08g06010	transporter, major facilitator family, putative, expressed
Chr8	14417870	14417870	A	AT	A	INDEL	OS08G0327400	Intronic	NA	NA	LOC_Os08g23810	enoyl-acyl-carrier-protein reductase NADH, chloroplast precursor, expressed
Chr8	5983448	5983448	A	AT	A	INDEL	OS08G0203100	Intronic	NA	NA	LOC_Os08g10290	SHR5-receptor-like kinase, putative, expressed
Chr8	5985856	5985856	A	AT	A	INDEL	OS08G0203100	Exonic	3UTR	NA	LOC_Os08g10290	SHR5-receptor-like kinase, putative, expressed
Chr9	21858332	21858332	A	AT	A	INDEL	OS09G0551600	Intronic	NA	NA	LOC_Os09g37910	HMG1/2, putative, expressed
Chr9	17391522	17391522	A	AT	A	INDEL	OS09G0459900	Intronic	NA	NA	LOC_Os09g28580	expressed protein
Chr9	16440875	16440875	A	AT	A	INDEL	OS09G0442300	Intronic	NA	NA	LOC_Os09g27030	oryzain gamma chain precursor, putative, expressed
Chr9	16225964	16225964	A	AT	A	INDEL	OS09G0438400	Intronic	NA	NA	LOC_Os09g26700	choline/ethanolamine kinase, putative, expressed
Chr9	13696291	13696291	A	AT	A	INDEL	OS09G0394700	Intronic	NA	NA	LOC_Os09g23150	monoglyceride lipase, putative, expressed
Chr9	13696506	13696506	A	AT	A	INDEL	OS09G0394700	Intronic	NA	NA	LOC_Os09g23150	monoglyceride lipase, putative, expressed
Chr9	3655810	3655810	A	AT	A	INDEL	OS09G0248100	Intronic	NA	NA	LOC_Os09g07350	fasciclin-like arabinogalactan protein 8 precursor, putative, expressed
[truncated: 2,711,726 more chars]
